# Small RNA and degradome profiling involved in seed development and oil synthesis of *Brassica napus*

**DOI:** 10.1371/journal.pone.0204998

**Published:** 2018-10-17

**Authors:** Wenhui Wei, Gan Li, Xiaoling Jiang, Yuquan Wang, Zhihui Ma, Zhipeng Niu, Zhiwei Wang, Xinxin Geng

**Affiliations:** 1 College of Life Science and Technology, Henan Institute of Science and Technology / Collaborative Innovation Center of Modern Biological Breeding, Henan Province, Xinxiang, China; 2 Applied Biotechnology Center, Wuhan Institute of Bioengineering, Wuhan, China; Dokuz Eylul Universitesi, TURKEY

## Abstract

MicroRNAs (miRNAs) play a prominent role in post-transcriptional gene expression regulation and have been involved in various biological and metabolic processes to regulate gene expression. For *Brassica napus*, improving seed-weight and oil-content is the main breeding goal. In order to better understand the regulation mechanism of miRNAs during seed-weight formation and oil-content accumulation in *B*. *napus*, in this study, a high-throughput sequencing technology was used to profile miRNAs expression of *Brassica napus* immature seeds from one to six weeks after flowering. A total of 1,276 miRNAs, including 1,248 novel and 28 known miRNAs, were obtained from both the high-seed-weight with low-oil-content RNA pool (S03) and the low-seed-weight with high-oil-content RNA pool (S04). Analysis of their expression profiles disclosed that 300 novel and two known miRNAs were differentially expressed between S03 and S04. For degradome analysis, 57 genes with 64 degradation sites were predicted to be targeted for degradation by these miRNAs. Further bioinformatics analysis indicated that these differentially expressed miRNAs might participate in regulation of myriad cellular and molecular processes, during seed development and oil synthesis. Finally, 6 target genes with potential roles in regulation of seed development and 9 other targets in seed oil synthesis, were further confirmed as candidate genes from small RNA and degradome sequencing.

## Introduction

As one of the major oil crops in the world, *Brassica napus* plays a critical role in supply of vegetable oil [1]. Improvement of seed oil-production yield is the ultimate goal of *B*. *napus* breeding. Oil-production yield consists of two components, i.e. seed yield and seed oil-content. Seed yield mainly depends on silique number per unit area, seed number per silique, and seed-weight. In these factors, seed-weight is a key and stable component for evaluating seed yield [2]. Recent research has found that improving seed-weight is the most important approach to enhance the seed yield of *B*. *napus* [3]. Seed-weight and seed oil-content have great variation among *B*. *napus* cultivars or lines with different genetic backgrounds.

Gene expression is regulated at both transcriptional and post-transcriptional levels to ensure correct responses to myriad stresses, as well as regular growth and development [3–4]. Endogenous small RNAs (sRNAs) with a length of 21–24 nucleotides (nt) are known as important regulators of gene expression in most aspects of plant biology [5–8]. Currently, with high-throughput sequencing technologies several kinds of endogenous sRNAs have been widely recognized as essential and effective regulators in diverse biological processes of many eukaryotic organisms [8, 9]. Among these, two major classes of endogenous sRNAs, short-interfering RNAs (siRNAs) and microRNAs (miRNAs), have crucial functions in regulating the processes of plant growth and development, such as seed germination and development [5, 10, 11], organ formation [12, 13], auxin signaling [14, 15] and stress responses [16, 17], through translational repression and endonucleolytic cleavage at post-transcriptional level [12, 18–20]. In plants, mature miRNAs are generated from precursor miRNA (pre-miRNAs), which are transported from nucleus to cytoplasm with the help of Exportin-5 before processed to mature miRNAs [21].

Compared with *Arabidopsis* and other model plants, only a few miRNAs and their targets have been identified in seed development and oil synthesis of *B*. *napus* [22]. For examples, Zhou *et al*. found 84 miRNAs including 19 novel miRNA members between Cd-treated and non-treated *B*. *napus* [23]. Shen *et al*. [24] reported a total of 645 miRNAs including 280 conserved and 365 novel miRNAs from two *B*. *napus* cultivars (Ningyou7 and Tapidor). From the early siliques of two *B*. *napus* cultivars with different types of oil-content, 59 miRNAs including 50 conserved miRNAs and 9 new miRNAs were screened [25]. Xu *et al*. [22] identified 62 *Brassica*-specific and 41 conserved miRNAs from pooled *B*. *napus* tissues. Huang *et al*. [5] used *B*. *rapa* reference genome to detect about 10 novel and 500 conserved miRNAs from the whole seeds. Wang *et al*. [26] identified 826 miRNA sequences, including 523 conserved and 303 novel miRNAs in different stages of *B*. *napus* seeds. Wang *et al*. [27] found that a total of 85 known miRNAs from 30 families and 1,160 novel miRNAs were identified, of which 24, including 5 known and 19 novel miRNAs, were involved in fatty acid biosynthesis. However, there were a few reports on small RNA, miRNA and their function analysis during seed-weight formation and oil-content accumulation in *B*. *napus* so far. In order to better understand the regulation mechanism of miRNAs during seed-weight formation and oil-content accumulation in *B*. *napus*, it is necessary to profile miRNAs and define their temporal and spatial expression patterns during seed development.

Degradome sequencing, as an effective experimental approach developed to identify miRNA targets, combines the advantages of high-throughput RNA sequencing technologies with bioinformatic analysis, and has been successfully applied to miRNA target genes screening in *Arabidopsis* [28], rice [29,30], *B*. *napus* [22], and other plants [15, 31–33]. With degradome sequencing, many miRNA targets have been identified and the accurate pairing information between degradation fragments and miRNA in plants has been determined [28, 30].

In this study, we first identified a pool of differentially expressed (DE) miRNAs in seed development between two *B*. *napus* lines with extremely different seed-weight and seed oil-content, using high-throughput Illumina sequencing technology to monitor sRNA libraries of one to six weeks’ seeds after flowering of these two lines. We further predicted hundreds of targets of these miRNAs, with functional annotation from GO [34], Cluster of Orthologous Groups of proteins (COG) [35], Kyoto Encyclopedia of Genes and Genomes (KEGG) [36], Swiss-Prot [37], and Non-redundant protein (NR) [38] databases. Moreover, using degradome sequencing, we validated some miRNA targets and their degradation sites. These data provided a foundation for evaluating the important regulatory roles of miRNAs in seed development and oil synthesis.

## Results

### Seed-weights and oil-contents of two extreme *B*. *napus* lines

**Fig 1:**
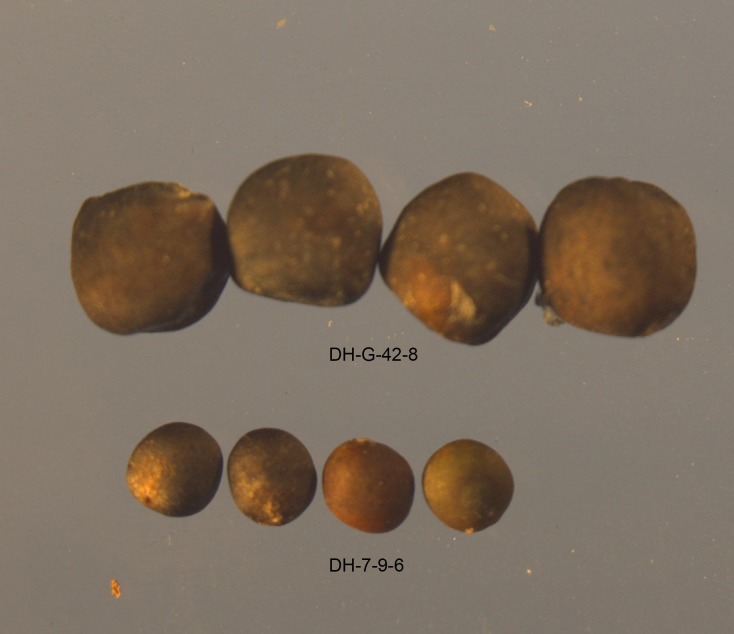
Seed phenotype of two extreme lines (large-seed line DH-G-42-8 and small-seed line DH-7-9-6) in *Brassica napus*.

The 1,000-seed-weight and seed oil-content traits of DH-G-42 and DH-7-9 performed differently. The seed size and weight of the large-seed line DH-G-42-8 were significantly greater than those of the small-seed line DH-7-9-6 (Table 1 and Fig 1). However, the seed oil-content of DH-G-42-8 was much lower than that of DH-7-9-6 (Table 1). These two extreme lines (DH-G-42-8 and DH-7-9-6) were selected for small RNA and degradome profiling to screen for DE miRNAs and their targets.

**Table 1:** Performances of the 1,000-seed-weight and oil-content traits in lines DH-G-42 and DH-7-9. Seed-weight and oil-content of two extreme *B*. *napus* lines DH-G-42-8 and DH-7-9-6 were selected (in bold).

Number	1000-seed -weight (g)	Oil-content (%)	Number	1000-seed -weight (g)	Oil-content (%)
DH-G-42-1	6.07 ±0.063	26.88	DH-7-9-1	2.74 ±0.018	39.20
DH-G-42-2	5.82 ±0.080	24.72	DH-7-9-2	2.54 ±0.011	39.88
DH-G-42-3	5.91 ±0.059	25.65	DH-7-9-3	2.66 ±0.013	41.76
DH-G-42-4	6.01 ±0.054	20.67	DH-7-9-4	2.48 ±0.012	38.22
DH-G-42-5	6.13 ±0.071	27.90	DH-7-9-5	2.60 ±0.023	39.21
DH-G-42-6	6.11 ±0.092	21.66	**DH-7-9-6**	**2.42 ±0.015**	**40.88**
DH-G-42-7	5.99 ±0.056	23.54	DH-7-9-7	2.51 ±0.025	39.52
**DH-G-42-8**	**6.24 ±0.050**	**24.10**	DH-7-9-8	2.59 ±0.015	37.60
DH-G-42-9	6.03 ±0.087	26.71	DH-7-9-9	2.79 ±0.029	38.41
DH-G-42-10	6.02 ±0.064	24.47	DH-7-9-10	2.63 ±0.02	39.53

### Global analysis of developing seed sRNAs from the two extreme lines

For two extreme lines, small RNAs from developing seeds at week 1, 2, 3, 4, 5 and 6 after fertilization were collected respectively, and then pooled together as two separate libraries for high-throughput Illumina deep-sequencing to determine their DE miRNAs. These two libraries yielded a total of 46 million raw reads and 40.34 Mb clean reads after filtering. In total, 18,277,880 (69.4%) and 20,079,520 (76.25%) sequences were obtained from these two libraries derived from DH-G-42-8 and DH-7-9-6, respectively, after eliminating the low quality tags and adaptors. These reads equal to 6,618,080 (51.52%) and 7,877,398 (61.32%) unique sRNA sequences in the large-seed library (S03) and small-seed library (S04), respectively (S1 Table). All the unique sRNA sequences were further used to align with the reference *B*. *napus* genome [39] using SOAP [40]. These sRNA sequences were found to match *B*. *napus* genome perfectly (S1 Table). In addition, almost all types of sRNA (rRNA, tRNA, degradation exons or introns tags, miRNA, siRNA, snRNA, snoRNA and repeat sRNA) could be discovered from deep-sequencing data (Table 2). In both datasets, only 45.65% of the total sRNAs, representing 12.84% of the unique sRNAs, shared common sequences, and the total unique sequences in S03 were much less than those in S04 (S1 Table). The results suggest that a broader regulation pattern of gene expression mediated by sRNA exists in the large-seed line (DH-G-42-8) versus the small-seed line (DH-7-9-6).

**Table 2:** Distribution of small RNAs among different categories.

Category	S03		S04	
	Number	Percentage	Number	Percentage
**Genome**	11,496,619	60.41	12,335,822	57.90
**rRNA**	647,944	3.40	841,539	3.95
**scRNA**	0	0.00	0	0.00
**snRNA**	806	0.00	988	0.00
**snoRNA**	393	0.00	500	0.00
**tRNA**	82,285	0.43	101,629	0.48
**Repbase**	22,174	0.12	33,733	0.16
**Other**	6,781,261	35.63	7,991,005	37.51
**clean-reads**	19,031,482	100.00	21,305,216	100.00

It has been reported that sRNA length distribution frequently reflects the specificity of a particular species or tissue [25, 41]. In our two libraries, 92.80% and 93.56%, respectively, of the total sRNAs’ length was distributed between 20–24 nt (S2 Table). Previous studies have shown that 24-nt long sRNAs were more abundant than 21-nt sRNAs in plants [5, 22, 42]. Our results also show that the most abundant population of small RNAs length was 24 nt and the second largest was 21 nt in the two libraries, similar to the previous results in other plants [25]. Thus, the percentages of sRNA sequences with 21 nt or 24 nt (33.25% and 34.98% in S03, 28.05% and 25.63% in S04, respectively) were significantly higher than others. Unexpectedly, 21-nt long miRNAs are more abundant than 24-nt long miRNAs in two libraries and their distribution was different from that of total sRNAs.

It has been well recognized that in a few plants most miRNA sequences show a strong bias for a uridine (U) at position 1, whereas the majority of 24-nt long siRNAs have an apparent preference for 5’ adenosine (A) [5, 43–45]. The same patterns were also observed in S03 and S04 derived from *B*. *napus* (S1 Fig). The cause of such nucleotide composition bias might be attributed by the cutting site specificity of cytoplasmic Dicer enzymes [5]. Deep sequencing of miRNA was analyzed with the miRDeep2 software v2.0.5 [46], and the precursor miRNA sequences with hairpin-structure and the fold RNA structures of every candidate miRNA were listed in S3 Table and S2 Fig.

### Identification of known, conserved and novel miRNAs

**Fig 2:**
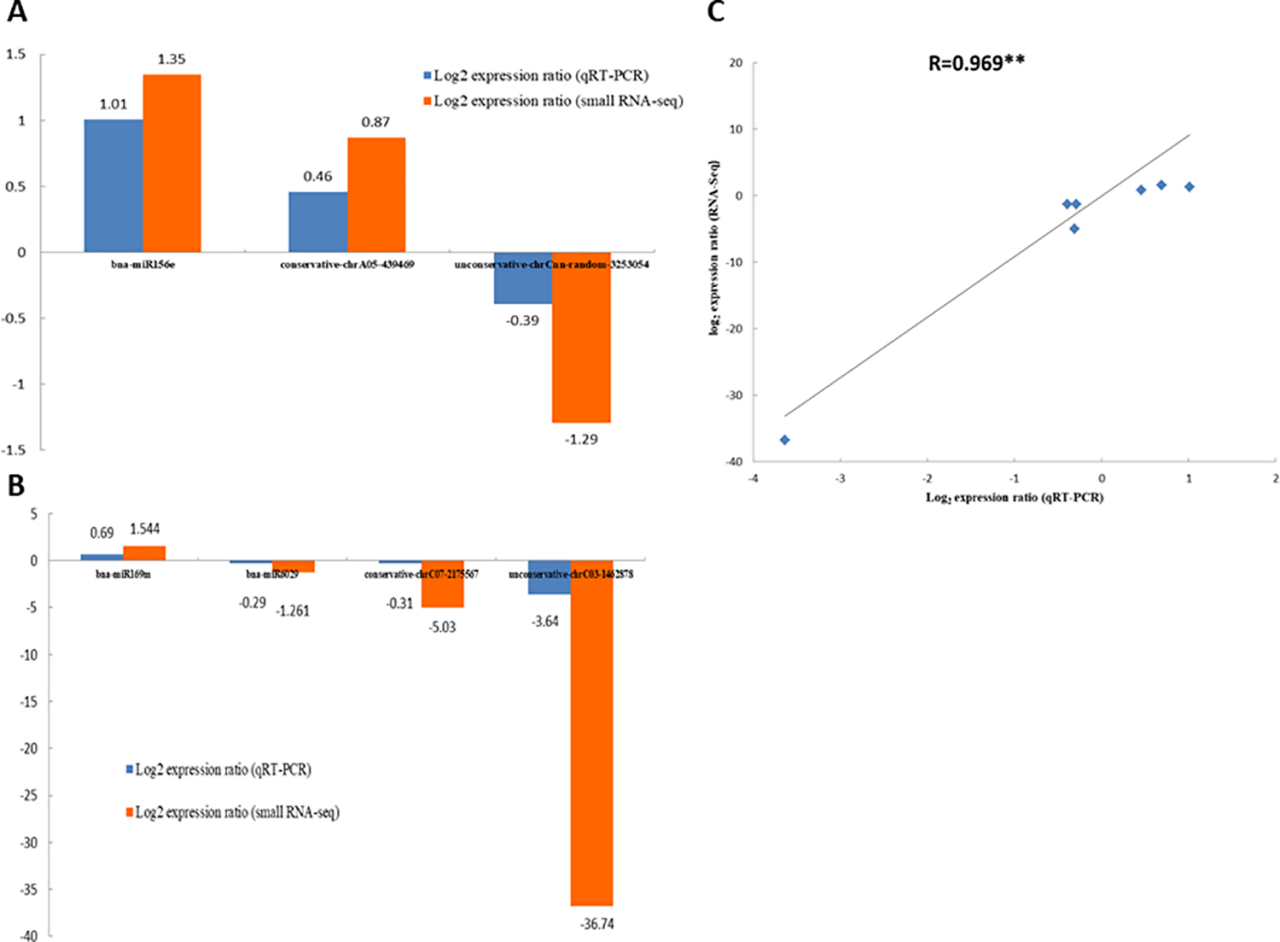
QRT-PCR validation of 7 miRNAs. **(A)** The small RNA-Seq log_2_ value (expression ratios of S03-RPKM/S04-RPKM) and the qRT-PCR log_2_ value (expression ratios of S03/S04) of three important miRNAs (bna-miR156e, conservative-chrA05-439469 and unconservative-chrCnn-random-3253054) which the expression abundance was extremely high in two libraries (S03 and S04). **(B)** The small RNA-Seq log_2_ value (expression ratios of S03-RPKM/S04-RPKM) and the qRT-PCR log_2_ value (expression ratios of S03/S04) of two known miRNAs (bna-miR169m and bna-miR6029), one conservative miRNA (conservative-chrC07-2175567) and one novel miRNA (unconservative-chrC03-1462878) highly differentially expressed between S03 and S04. **(C)** Correlation analysis of the miRNA expression ratios obtained from the qRT-PCR and small RNA-seq data of 7 miRNAs (*p*-value <0.01).

High-throughput sequencing is deemed as a useful tool for miRNA expression profiling because of its good reproducibility [8]. A total of 1,158 novel, 90 conservative and 28 known miRNAs were obtained by Illumina deep-sequencing method from S03 and S04 (Table 3 and S4 Table). Among 28 known miRNAs, Bna-miR166d was the most abundant in S03 and S04 datsets, accounting for 30.40% and 25.05% of sequence reads. Among 90 conservative miRNAs, conservative-chrA08-686185 was the most highly expressed miRNA in the data sets. However, in the novel miRNAs group, unconservative-chrC04-1716601 was the most abundant non-conserved miRNA in S03 and accounted for only 2.74%. In S04, unconservative-chrC08-2447451 was the most abundant non-conserved miRNA and accounted for 3.14%. It was also noted that the expression levels of several known miRNA sequences were much higher than those of novel miRNAs in the data sets, such as bna-miR166a, bna-miR166d and bna-miR167d (S4 Table). The sequencing results of three miRNAs (bna-miR156e, conservative-chrA05-439469 and unconservative-chrCnn-random-3253054) whose expression abundance was extremely high in both libraries, were further verified by quantitative RT-PCR (qRT-PCR) analyses (Fig 2A).

**Table 3:** Summary of miRNA and target gene number from S03 and S04.

Type	MiRNA number	Target MiRNA number	Target number
**Know**	28	9	76
**Conservative**	90	15	397
**Unconservative novel**	1,158	157	70
**Total**	1,276	181	503

### Differentially expressed miRNAs between two extreme lines

Interestingly, differential expression analysis results show that 300 novel and 2 known miRNAs among all identified miRNAs were extremely differentially expressed between these two extreme lines. Totally, 182 were up-regulated and 120 were down-regulated in S03 (S5 Table), with the expression level of miRNA in S04 as the reference. For all DE miRNAs, it is notable from sequencing results that two known miRNAs (bna-miR169m and bna-miR6029), one conservative miRNA (conservative-chrC07-2175567), and one novel miRNA (unconservative-chrC03-1462878) were highly differentially expressed between S03 and S04, as confirmed by qRT-PCR (Fig 2B). It suggests that these DE miRNAs most likely play very important roles in seed development and oil synthesis in *B*. *napus*. Moreover, a correlation analysis (Fig 2C) of expression levels of 7 selected miRNAs (bna-miR156e, conservative-chrA05-439469, unconservative-chrCnn-random-3253054, bna-miR169m, bna-miR6029, conservative-chrC07-2175567 and unconservative-chrC03-1462878) shows a significant correlation (correlation coefficient R = 0.969, *p*-value <0.01) between two methods, qRT-PCR and small RNA sequencing (small RNA-Seq), suggesting that the data generated from small RNA-Seq assay of this study are sufficient for investigating the differential expression of miRNAs between S03 and S04.

### Identification of putative miRNA targets in two extreme lines

Plant miRNAs with their target genes have a nearly perfect pairing, and function through cleaving targets directly or, in some cases, by translational repression. Therefore, identifying miRNA target genes is the key to understand their functions. Potential targets of miRNAs were computationally predicted using TargetFinder software v1.6 script [47]. Starting with 1,276 total miRNA sequences, a set of 503 potential targets for 157 novel miRNAs, 15 conserved miRNAs and 9 known miRNAs were predicted (Table 3 and S6 and S7 Tables). Among 302 DE miRNAs, 119 potential targets for 45 miRNAs were identified (S8 Table). The detailed sequence information of newly identified targets for all miRNAs and DE miRNAs are listed in S6–S8 Tables.

**Fig 3:**
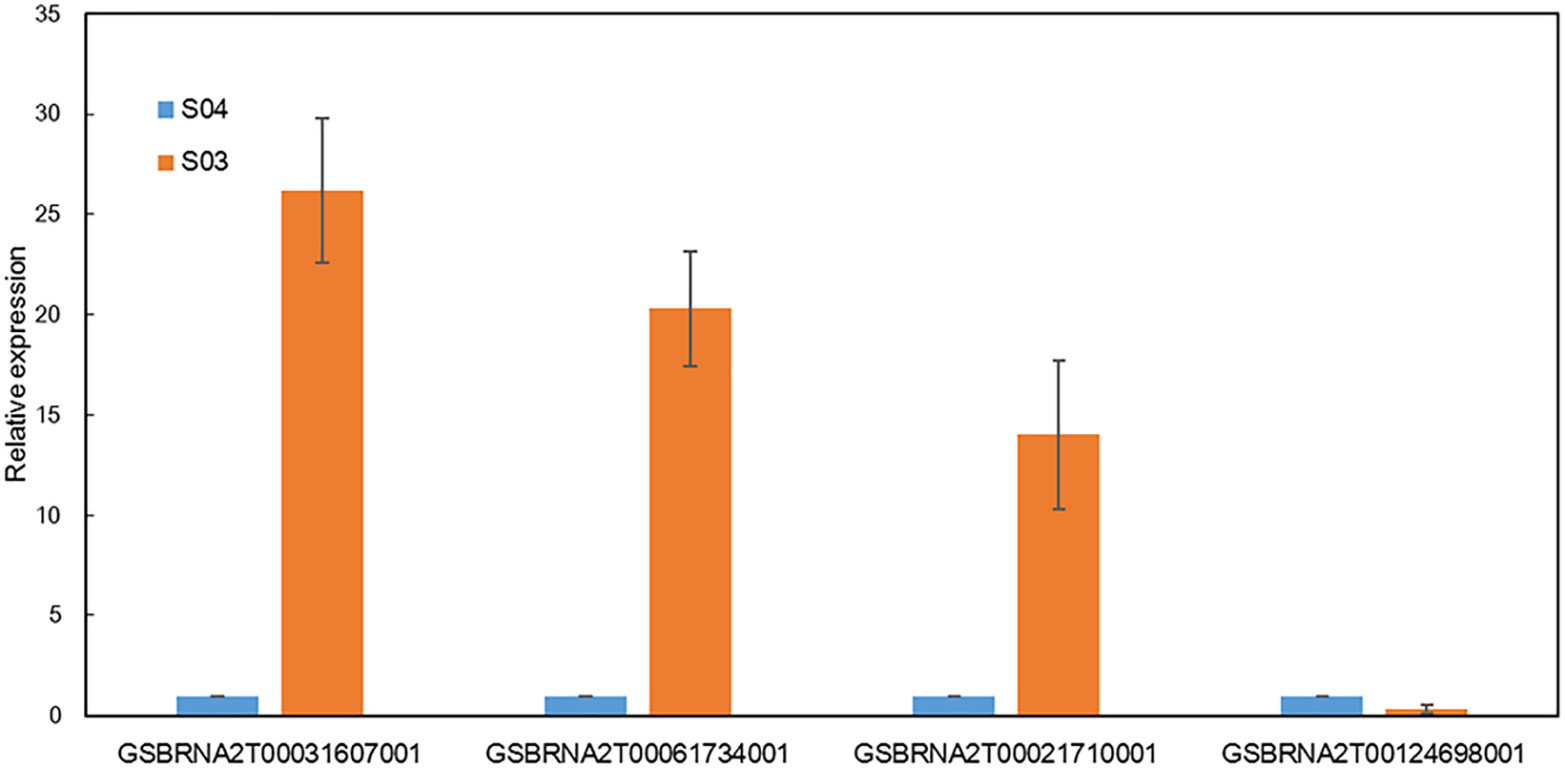
Quantitative real-time RT-PCR analysis on the relative expression level of four vital miRNA targets in S03 and S04: *GSBRNA2T00031607001* (the target of unconservative-chrC09-2659344), *GSBRNA2T00061734001* and *GSBRNA2T00021710001* (the targets of unconservative-chrC08-2341054), *GSBRNA2T00124698001* (the target of bna-miRNA6029).

**Fig 4:**
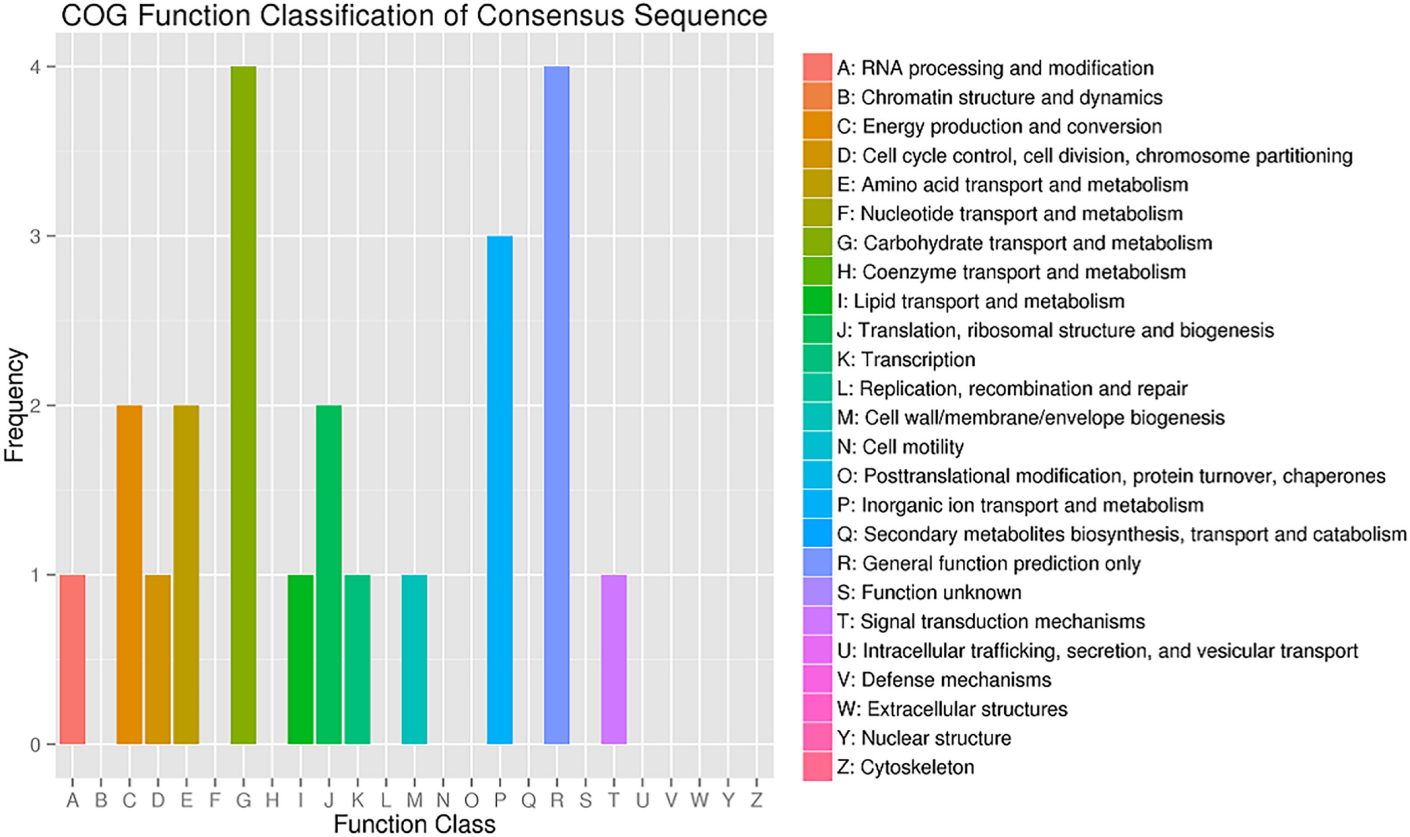
COG function classification of differentially expressed miRNA targets between S03 and S04.

**Fig 5:**
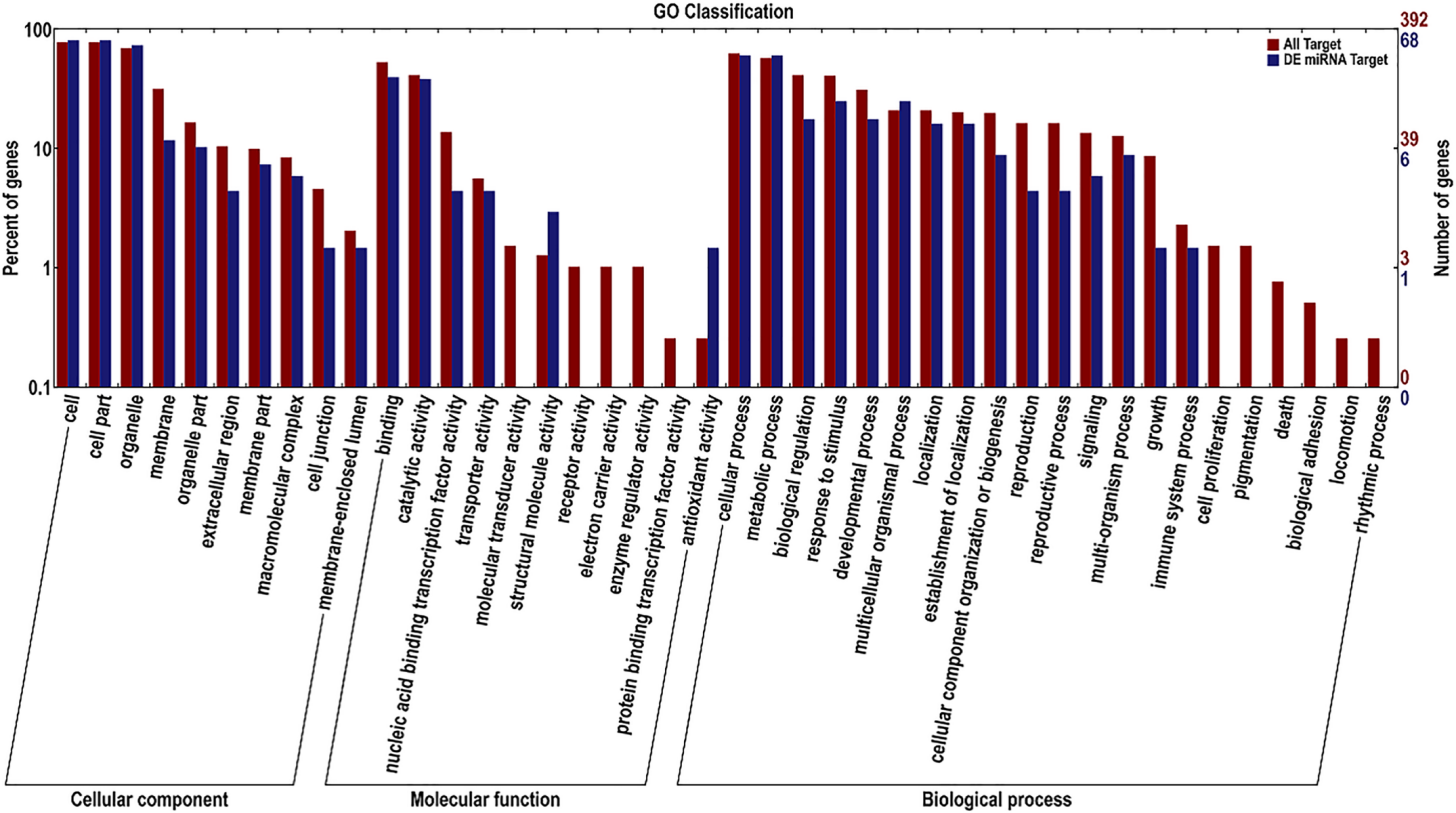
GO function classification according to cellular component, molecular function and biological process of all and differentially expressed miRNA targets between S03 and S04.

We also used qRT-PCR to examine the abundance of some vital miRNA targets. The expression level of *GSBRNA2T00031607001*, a target of the novel miRNA unconservative-chrC09-2659344, in S03 was higher than that in S04. *GSBRNA2T00061734001* and *GSBRNA2T00021710001* are two targets of the novel miRNA unconservative-chrC08-2341054, and both expressed at higher levels in S03 than S04. However, a putative target (*GSBRNA2T00124698001*) of the known miRNA bna-miRNA6029, had a lower expression level in S03 than S04. QRT-PCR results were well consistent with our small RNA-Seq analysis results (Fig 3). Since plant miRNAs usually have a strong favor for their target genes with important functions [44], the predicted target sequences of all miRNAs and DE miRNAs were subjected to blast search with GO, COG, KEGG, Swiss-Prot and NR databases, 465 targets from total miRNAs and 98 targets from DE miRNAs could successfully obtain annotated functional information (Table 4 and S9–S11 Tables). Among all 465 miRNA targets, 116 (24.94%) could be annotated into COG database, 392 (84.30%) into GO database, and 69 (14.84%) into KEGG database. However, for a total of 98 DE miRNA targets, only 23 obtained COG functional annotation. These targets were found to be involved in carbohydrate transport and metabolism process (17.39%), general function prediction only (17.39%), inorganic ion transport and metabolism process (13.04%), energy production and conversion process (8.69%), amino acid transport and metabolism process (8.69%), translation, ribosomal structure and biogenesis process (8.69%), RNA processing and modification process (4.35%), transcription (4.35%), cell cycle control, cell division and chromosome partitioning process (4.35%), lipid transport and metabolism process (4.35%), cell wall/membrane/envelope biogenesis process (4.35%), and signal transduction mechanisms process (4.35%) (Fig 4). In GO biological process enrichment analysis, 68 DE miRNA targets were classified into cellular component (43.25%), molecular function (14.19%), and biological process (42.56%) (Fig 5). In KEGG database, for 14 DE miRNA targets, each could be involved into different pathways including RNA transport, phosphatidylinositol signaling system, glycolysis/gluconeogenesis, butanoate metabolism, phenylalanine metabolism, citrate cycle (TCA cycle), pyruvate metabolism, valine, phenylpropanoid biosynthesis, leucine and isoleucine biosynthesis, aminoacyl-tRNA biosynthesis, inositol phosphate metabolism, ribosome, alpha-linolenic acid metabolism and endocytosis (S12 Table and S3 Fig). The detailed annotation of each miRNA target is shown in S10 and S11 Tables. From annotation information above, it could indicate that all those targets and their corresponding miRNAs may play a vital role during seed-weight accumulation and oil synthesis in *B*. *napus*.

**Table 4:** Summary of annotated all and differentially expressed miRNA targets.

Annotated databases	All miRNA targets	Differentially expressed miRNA targets
**COG**	116	14
**GO**	392	68
**KEGG**	69	14
**Swiss-Prot**	340	57
**NR**	465	98
**All**	465	98

### Putative target genes involved in seed development and oil synthesis

Analysis of *B*. *napus* small RNA libraries facilitated us to identify 3 putative target genes potentially involved in seed development (1 gene) and oil synthesis (2 genes) (Table 5). *GSBRNA2T00056839001* participates in lipid transport and metabolism process, *GSBRNA2T00044758001* plays very important roles in cell division, and *GSBRNA2T00015018001* takes part in energy production and conversion process. Homologous analyses and functional prediction of these three candidate genes show that, *GSBRNA2T00044758001* has high identity with *Arabidopsis AT4G30080* (85%), which encodes auxin responsive factors that are involved in regulating auxin signaling and therefore affects cell division and proliferation [48]. Therefore, the gene *GSBRNA2T00044758001* is likely involved in regulating seed development in *B*. *napus*. *GSBRNA2T00015018001* has high identity with *Arabidopsis AT1G59900* (97%), which encodes the pyruvate dehydrogenase complex (PDC) that catalyzes pyruvate to generate aceyl CoA, the direct substrate of fatty acid synthesis [49]. Therefore, the gene *GSBRNA2T00015018001* might play a key role in lipid synthesis pathway and have a direct effect on seed oil-content.

**Table 5:** Three candidate miRNA target genes involved in seed-weight and oil synthesis of *B*. *napus*.

Candidate genes	Annotated information	Databases
***GSBRNA2T00056839001***	Lipid transport and metabolism	COG
***GSBRNA2T00044758001***	Cell division	GO
	ARF 16 (*Arabidopsis thaliana*)	Swissprot
	ARF 16–2 (*Brassica napus*)	Nr
***GSBRNA2T00015018001***	Ubiquitin ligase E1	KEGG
	Pyruvate dehydrogenase complex E1 (*Arabidopsis thaliana*)	Swissprot
	Pyruvate dehydrogenase complex E1(*Brassica napus*)	Nr

### Degradome analysis of miRNA-guided cleavage of target genes

Through degradome sequencing approach, we obtained totals of 41.42M raw reads and 30.89M clean reads after filtering. To verify miRNA-guided target cleavage, degradation sites and cleavage products were predicted by Cleaveland software 4.0 [50]. Degraded targets were categorized into 5 classes in accordance with their relative abundance of reads at cleavage sites (categories 0, 1, 2, 3 and 4) as reported in *Arabidopsis* [16], grapevine [31], *B*. *napus* [22] and maize [9]. Categories 0, 1, 2 and 3 all own more than one raw read, except category 4 that has only one raw read at the cleavage position.

**Fig 6:**
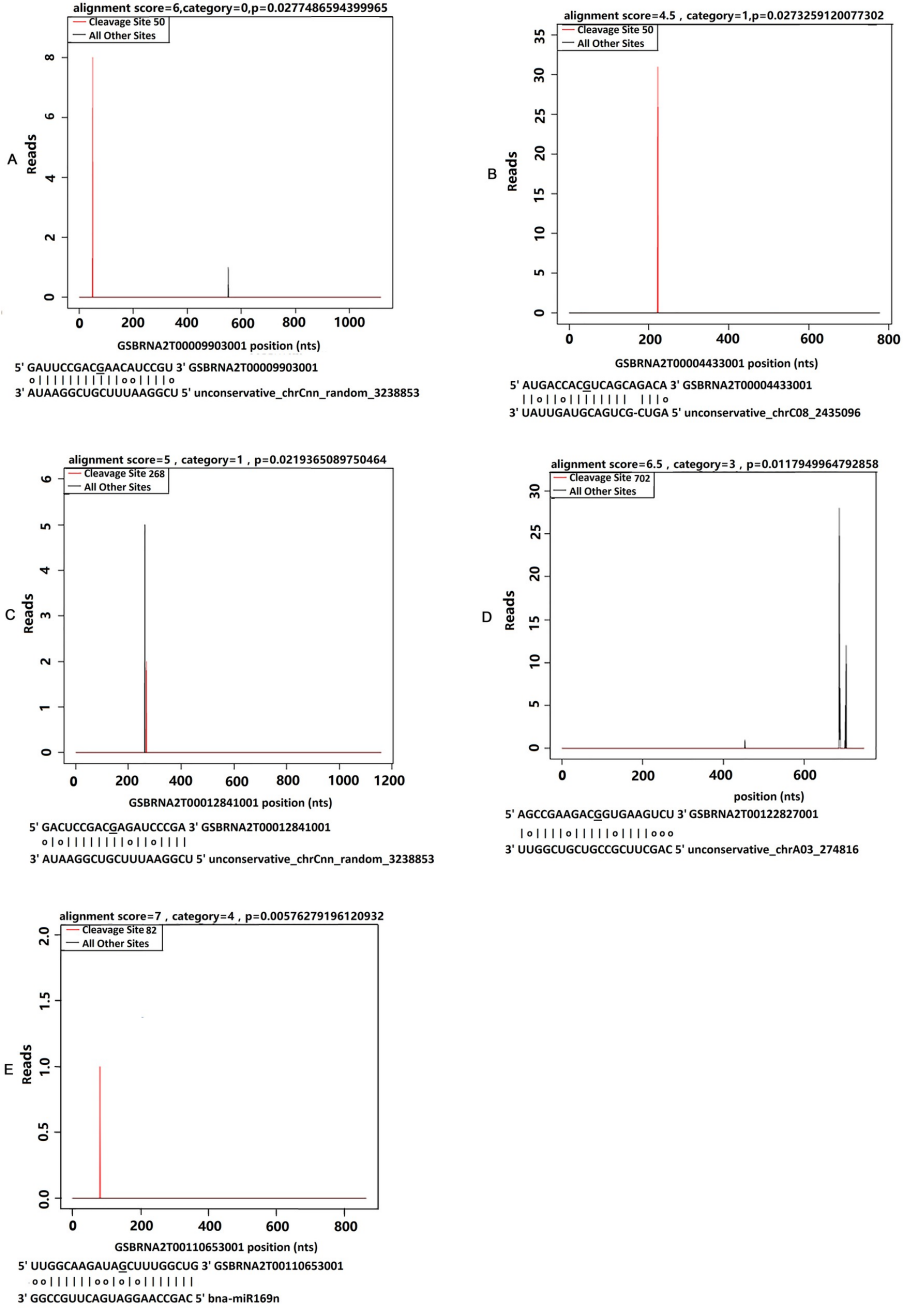
MiRNA targets using degradome sequencing are presented in the form of target plots (t-plots). The normalized numbers in plotting the cleavages on target mRNAs were used to refer to as ‘target plots’ (t-plots) by German *et al*. [28]. Representative t-plots for category 0 **(A)**, category 1 **(B)**, category 2 **(C)**, category 3 **(D)** and category 4 **(E)** are shown (Category 0–4 were based on Addo-Quaye *et al*. (2008), Xu *et al*. (2012) and Liu *et al*. (2014a)). The cleavage sites are shown in red, and all the other sites are in black. The underlined nucleotide on the target transcript indicates the cleavage site detected in the degradome.

With the threshold *p*-value <0.05, a total of 57 target genes were predicted to be degraded with 64 degradation sites (S13 Table, S4 Fig). Sixteen targets from these 57 target genes were classified into category 0, whose abundance of degradome sequences at the cleavage was expected to be higher than the maximum on the transcript (Fig 6A). The most abundant population contains 22 targets, and were classified into category 1, in which the maximum on the transcript overlaps with the abundance of degradome sequences (Fig 6B). Five targets were classified into Category 2 (Fig 6C), in which the abundance is located between the median and maximum for the transcript. Two targets fell into Category 3, in which the abundance equals to or less than the median for transcript (Fig 6D). Twelve of 57 targets were classed into category 4 (Fig 6E).

Previous studies reported that mRNA fragments targeted by the 5’ ends of a miRNA would incline to the complementary nucleotide of miRNA’s 10th nucleotide [17, 27]. Consistent with this, our results show that almost all 142 miRNAs guided 64 target cleavages at the 10th nucleotide (S13 Table). It means that all 64 predicted targets have specific cleavage sites on the complementary sequences of each miRNA. Moreover, results demonstrate that different miRNAs could regulate the same target gene (S13 Table). For example, 20 different miRNAs target the same gene *GSBRNA2T00085933001*. Another 22 different miRNAs target the same gene *GSBRNA2T00104433001*. Meanwhile, one miRNA could target different genes. For example, the miRNA unconservative-chrCnn-random-3238853 target 10 different genes (S13 Table). Both novel miRNAs, unconservative-chrAnn-random-3000993 and unconservative-chrAnn-random-3000995, target the same genes *GSBRNA2T00114316001* and *GSBRNA2T00111014001* (S13 Table).

From annotated information of these 57 degraded target genes in S14 Table, it was interestingly found that 12 miRNA targets are probably involved in some important processes during seed development (5 genes) and oil synthesis (7 genes) (Table 6). Genes *GSBRNA2T00122970001* (the target of unconservative-chrCnn-random-3238853) and *GSBRNA2T00151623001* (the target of unconservative-chrA02-195125 and unconservative-chrA06-523926) participate in cell division and development. Gene *GSBRNA2T00030795001*, the target of unconservative-chrC08-2356729, positively regulates cell size. The homologous *Arabidopsis* gene *AT5G13530* of *GSBRNA2T00140338001* encodes an E3 ubiquitin-protein ligase, which has been reported to affect seed development and maturation. *GSBRNA2T00021710001*, the target gene of unconservative-chrAnn-random-2962078, is involved in the process of seed development. In summary, these five genes above might be involved in the process of seed development and thereby affect seed-weight of *B*. *napus*.

**Table 6:** Twelve candidate degraded target genes involved in seed-weight and oil synthesis of *B*. *napus*.

Candidate genes	Annotated information	Databases
***GSBRNA2T00122970001***	Cell division	GO
	WPP protein	Swissprot
	WPP protein	Nr
***GSBRNA2T00151623001***	Cell division	GO
	Frataxin, mitochondrial	Swissprot
	Frataxin	Nr
***GSBRNA2T00140338001***	Cell development	GO
	E3 ubiquitin-protein ligase	Swissprot
	E3 ubiquitin-protein ligase	Nr
***GSBRNA2T00021710001***	Seed development	GO
	ZFWD4	Swissprot
	Transducin/WD40 domain-containing protein	Nr
***GSBRNA2T00030795001***	Regulation of cell size	GO
	Regulation of seed growth	GO
	Phosphate transporter PHO1 homolog 5	Swisspro
***GSBRNA2T00122827001***	Response to lipid	GO
***GSBRNA2T00071654001***	Energy production and conversion	COG
	Succinyl-CoA ligase; succinyl-CoA synthetase	KEGG
	Succinyl-CoA ligase subunit alpha-1	Swissprot
***GSBRNA2T00080762001***	Energy production and conversion	COG
	Succinyl-CoA ligase; succinyl-CoA synthetase	KEGG
	Succinyl-CoA ligase subunit alpha-1	Swissprot
***GSBRNA2T00022584001***	Phosphoglycerate dehydrogenase activity	GO
	D-3-phosphoglycerate dehydrogenase	Swissprot
***GSBRNA2T00141442001***	Fatty acid catabolic process	GO
***GSBRNA2T00136074001***	Very long-chain fatty acid metabolic process	GO
	Fatty acid alpha-hydroxylase activit	GO
***GSBRNA2T00104433001***	Very long-chain fatty acid metabolic process	GO
	Fatty acid alpha-hydroxylase activit	GO

The target gene *GSBRNA2T00141442001* of the novel miRNA unconservative-chrCnn-random-3264385, participates in fatty acid catabolic process. *GSBRNA2T00071654001* (the target of unconservative-chrC05-1900490) and *GSBRNA2T00080762001* (the target of unconservative-chrC05-1900490) are involved in lipid transport and metabolism pathway. Two other genes *GSBRNA2T00136074001* (the target of unconservative-chrA03-274816, unconservative-chrA05-480212, unconservative-chrA07-648408 and unconservative-chrA07-677856) and *GSBRNA2T00104433001* (the target of 22 unconservative miRNAs) participate in very long-chain fatty acid metabolic process and fatty acid alpha-hydroxylase activity. Gene *GSBRNA2T00122827001* (the target of unconservative-chrA03-274816, unconservative-chrA05-480212, unconservative-chrA07-648408 and unconservative-chrA07-677856) are responsive to lipid. Gene *GSBRNA2T00022584001* (the target of unconservative-chrA05-464962, conservative-chrA09-840088 and conservative-chrC04-1604978) encodes glyceraldehyde-3-phosphate dehydrogenase (GPDH), which increases the production of 3-phosphoglycerate to enter the oil synthesis pathway and finally raises the lipid production [51]. In summary, these seven target genes above might play very important roles in the process of seed oil synthesis in *B*. *napus*.

## Discussion

High-throughput sequencing analysis of plant tissues at different developmental stages not only is effective in identifying a large number of sRNAs including novel miRNAs [16], but also provides an alternative way to estimate the expression profiles of miRNA genes [52]. Recently, several studies have reported large numbers of sRNAs in rice seeds [53, 54]. However, limited sRNAs (including miRNAs) for seed development have been identified from *B*. *napus*. In this study, with the reference of *B*. *napus* genome, high-seed-weight with low-oil-content RNA pool (S03) and low-seed-weight with high-oil-content RNA pool (S04) were used to profile miRNAs. Totally 1,248 novel and 28 known miRNAs were discovered in our study. It expands the number of miRNAs and their targets, which help better understand the regulation mechanism of seed-weight and oil-content during seed development.

We established small RNA libraries from different stages of seeds between two *B*. *napus* lines (DH-G-42-8 and DH-7-9-6) with extremely different seed-weight and seed oil-content to screen DE miRNAs and target genes. However, similar studies used only one *B*. *napus* cultivar’s different stage seeds to screen DE miRNAs and target genes [26–27]. Meanwhile, it is known that seed-weight and seed oil-content both play very significant roles during seed formation in oil crops. Most previous similar studies focused on either seed-weight or seed oil-content. Our study not only identified the miRNAs and their targets on fatty acid biosynthesis, but also obtained certain important miRNAs and their targets on seed-weight in *B*. *napus*. For all 28 known miRNAs sequencing data, we found interestingly that the reads number of 26 known miRNAs (92.85%) were high in S04 (S4 Table), which demonstrated the known miRNAs expression level had a negative correlation with the seed development rate. A total of 300 novel and 2 known DE miRNAs were obtained from two extreme lines. About 60.26% of 302 DE miRNAs were up-regulated and 39.74% down-regulated in S03, with S04 as the reference. Two known miRNAs, bna-miR169m and bna-miR6029, are up-regulated and down-regulated, respectively, in the low-oil-content line. In previous study, bna-miR169 was also shown to have a higher expression level in the low-oil-content cultivar than in the high-oil-content cultivar [25]. However, in contrast to our results, bna-miR6029 was found to have higher expression in the low-oil-content line than in the high-oil-content line. Of two known miRNAs, only bna-miR6029 owned target gene (*GSBRNA2T00124698001*). *GSBRNA2T00124698001* was much more highly expressed in S04 than in S03 and encodes a WEB family protein in *Arabidopsis thaliana*. Zhao *et al*. [25] identified bna-miR6029 and its target gene *TC101312* that encodes VirE2-interacting protein 1 (VIP1), a member of basic-Leu zipper transcription factors [55]. Several novel identified miRNAs were much more highly expressed in S03 than in S04, and target a pool of the same predicted genes (S7 Table). From COG annotation, they were involved in lipid transport and metabolism, energy production and conversion. In GO annotation, they participate in cellular component (nucleus, mitochondrial matrix, cytosol, cytoplasm), biological process (sterol biosynthetic process, pentacyclic triterpenoid biosynthetic process, telomere maintenance, glycolysis, DNA metabolic process) and molecular function (lupeol synthase activity, beta-amyrin synthase activity, DNA helicase activity, DNA repair, DNA duplex unwinding, pyruvate dehydrogenase (acetyl-transferring) activity). From KEGG database, gene *GSBRNA2T00015018001* could be annotated in five KEGG pathways: ko00010, ko00020, ko00290, ko00620 and ko00650 (S12 Table). Interestingly, some novel identified miRNAs like unconservative-chrCnn-random-3264385, unconservative-chrC05-1900490, unconservative-chrCnn-random-3238853, unconservative-chrAnn-random-2962078, unconservative-chrC08-2356729, etc. regulate a slice of important processes highly related to seed development and oil formation including fatty acid catabolic process, lipid transport and metabolism, cell division and development, seed development and cell size regulation. From annotated analysis, we can conclude that all those targets above and their derived miRNAs may play vital roles during seed-weight accumulation and oil synthesis in *B*. *napus*.

Most of the miRNAs in plants were completely or almost completely complementary with their target genes, and the cutting action often occurred at the tenth or eleventh nucleotides of complementary sites to regulate the expression of target genes. As to the results of our degradome analysis, most of 80 miRNAs guided their 64 target cleavages at the tenth nucleotide (S13 Table). It is quite consistent with that published by Xu *et al*. [22], whose studies found all target cleavages guided by five bna-miRNAs happened at the tenth or eleventh nucleotide. It seems that all predicted targets were found to have specific cleavage sites corresponding to the complementary sequences of a miRNA. Li *et al*. [29] confirmed two different miRNAs could act on the same target gene using degradation sequencing, e.g. miR156 and miR529b owned a certain homologous sequences (16 nucleotide overlap in the middle part of the sequence, 14 of which are exactly the same), acted on gene *Os11g30370* in rice. In present study, degradome analysis, i.e. miRNA-guided cleavage of target gene, showed that different miRNAs could regulate the same target gene and one miRNA could detect different target genes simultaneously. In conclusion, the presence of a large set of DE miRNAs involved in diverse developmental and metabolic processes between S03 and S04 suggests the crucial functions of miRNAs in developmental seeds with different seed-weights and oil-content. To enhance seed-weight and oil-content by improving the seed development rate, further research on the regulatory interactions between miRNAs and their target genes will provide valuable insights into the complex roles of miRNAs in regulating seed development in *B*. *napus*. The data obtained through degradome sequencing is beneficial to confirm the upstream miRNA sheared sites and to understand the regulation mechanisms of downstream target genes. In addition, we can also combine the biological information of several annotated analysis to analyze the regulation of miRNAs and their targets and explore miRNA specific regulation, ta-siRNA cutting function, processing of miRNA precursor, and the evolutionary relationship between miRNAs and their target mRNAs using degradome sequencing data.

## Conclusions

The small RNA expression and degradome analyses here provide informative values on developing seeds of *B*. *napus*. It expands the pool of novel and known miRNAs and enriches the function information of miRNAs and their targets in *B*. *napus*. The comparison between two *B*. *napus* lines with extremely different seed-weights and oil-contents revealed that some miRNAs may be involved in the regulation of *B*. *napus* seed development and oil synthesis. The detailed expression analysis of some miRNAs and their targets provided clues for their various roles in regulating the development of *B*. *napus* seed, which may be useful for further analysis and provide new perspectives on the regulation of gene expression networks of plant miRNAs and seed development, particularly in dicot plants.

## Materials and methods

### Plant materials and growth conditions

Two *B*. *napus* lines with extremely different seed-weights and oil-contents from sister lines DH-G-42 and DH-7-9 [56] respectively, a high-seed-weight with low-oil-content line (DH-G-42-8) and a low-seed-weight with high-oil-content line (DH-7-9-6) were planted and grown under non-stressed conditions from September 2015 to May 2016 in the field at the experimental farm of the Oil Crops Research Institute of the Chinese Academy of Agricultural Sciences, Wuhan, China. The Oil Crops Research Institute of the Chinese Academy of Agricultural Sciences issued the permission for each location in our experiment. There is no specific permissions were required for these locations. We have confirmed that the location is not privately-owned or protected in any way and the field studies did not involve endangered or protected species.

The seeds of 10 plants from two lines were selected to measure the dry seed-weight and oil-content at yielding time. Seed samples were oven-dried at 80 °C to a constant weight, then weighed. The seed oil-content was measured by near infrared spectroscope. The flowering time of DH-G-42-8 and DH-7-9-6 began from 7th March 2016 and 29th February 2016, respectively. Unmature seeds at each time point of one to six weeks after flowering were obtained from DH-G-42-8 and DH-7-9-6 lines, respectively, frozen in liquid nitrogen immediately, and stored at -80 °C.

### Small RNA isolation, library construction, and sequencing

Total RNAs were isolated from seeds at each time point of one to six weeks after flowering, respectively, using TRIzol reagent (Invitrogen, Carlsbad, CA, USA). The extracted RNA was qualified and quantified using a NanoDrop 2000 UV-Vis spectrophotometer (NanoDrop, Wilmington, DE, USA) and the samples showed a 260/280 nm ratio between 1.8 and 2.2 and an OD260/230 >1.0. For DH-G-42-8 line, equal RNAs at each time point were mixed as high-seed-weight with low-oil-content RNA pool (S03), and the low-seed-weight with high-oil-content RNA pool (S04) was also obtained from DH-7-9-6 line. RNase-free DNase I (Takara, Japan) was used to remove the residual DNA for 30 min at 37 °C. After testing the quality of RNAs, Illumina Truseq small RNA Sample Pre Kit was used to construct small RNA library. Because of the special structure of small RNA (5’ end of phosphate group and 3’ end of hydroxyl group), total RNAs were used as the starting sample and then reverse transcription was performed with 3’ end and 5’ end of small RNAs mixed with adaptors, followed by PCR amplification and PAGE gel separation of the target DNA fragment. cDNA library was obtained after gel extraction. Three replicates of each sample were used for small RNA sequencing.

After library is constructed, Qubit 2.0 Fluorometer (Invitrogen, Carlsbad, CA, USA) was then used for preliminary quantitative analysis of the concentration of a small RNA. The library was further diluted to 2 ng·μl^-1^, and the Agilent 2100 Bioanalyzer (Agilent, Palo Alto, California, USA) was used to detect the insert size of library. In the samples where the insert sizes were in line with expectations, qRT-PCR method was used to accurately quantify the effective concentration of the library (>2 nM), to ensure the quality of the library. Different libraries were mixed according to the effective concentrations and the demand of target data volume and finally sequenced by Illumina HiSeq 2500 sequencing system (Leiden, The Netherlands).

### Identification of novel miRNAs and prediction of miRNA targets

Clean reads were finally obtained after filtering low quality reads, adaptors, long and short sequences. To identify novel miRNAs and predict miRNA targets, following analyses were performed as described by Zhang *et al*. [57]. Clean reads were searched against GenBank Rfam database [58] using Basic Local-Alignment Search Tool (BLAST) and the reference genome of *B*. *napus* [39], and finally the sRNA annotation information was obtained. MiRDeep2 software v2.0.5 was used to analyze the clean reads, identify the miRNA, and determine the expression level [50]. Based on the above results, target genes were predicted and their differential expression was analyzed. The identified miRNAs which were not available in the miRBase database (http://microrna.sanger.ac.uk/sequences/, release 13.0) were assigned as novel miRNAs.

### Functional enrichment analysis of miRNA targets

GO, a gene functionality descriptions database, is widely used in functional annotation and enrichment analysis [34]. COG database is established based on the phylogenetic relationship of algae, bacteria, and eukaryotes. The gene products could be classified as orthologous relationship by using the COG database [35]. In the organism, different genes coordinate to execute the biological function. Pathway analysis is helpful to further interpret gene function. KEGG is the main public database on Pathway [36]. Here, potential miRNA targets were blast against the GO, COG, KEGG pathway, Swiss-Prot [37] and NR [38] databases to get their functional enrichment analysis with BLAST software v2.2.26 [59].

### Differential expression analysis

The expression patterns of all miRNAs were compared between S03 and S04 to determine DE miRNAs [60]. During DE miRNA screening, we took the False Discovery Rate (FDR) <0.01 and the Fold Change ≥2 as the standard. Here, FDR is obtained through the *p*-value correction and used as the key index of differential gene expression analysis.

Fold-change formula:

$$\mathrm{Fold}\;\mathrm{change}=\log_{2}(\mathrm{normalized}\;\mathrm{expression}\;\mathrm{level}\;\mathrm{of}\;\mathrm{miRNA}\;\mathrm{in}\;S04/S03)$$

Calculate the Fold Change and FDR from their normalized expression. If the miRNA’s Fold Change ≥2, FDR <0.01 indicates that the miRNA in S03 was significantly different from that in S04.

### Quantitative real-time RT-PCR

Seven important miRNAs (bna-miR156e, conservative-chrA05-439469, unconservative-chrCnn-random-3253054, bna-miR169m, bna-miR6029, conservative-chrC07-2175567 and unconservative-chrC03-1462878) were analyzed using qRT-PCR between S03 and S04. Mature miRNA was reverse transcribed into cDNAs using a miRNA specific stem-loop reverse transcription primer and a reverse transcriptase enzyme (Promega, Madison, WI, USA). Expression levels of these miRNAs were analyzed by qRT-PCR using miRNA-specific primers (S15 Table) with an ABI PRISM 7500 Sequence Detection System (Applied Biosystems, Foster City, CA, USA). For each reaction, 5 μl of 1:20 diluted template cDNA was mixed with 10 μl SYBR green reaction mix (Toyobo, Osaka, Japan), and 0.5 μl each of the forward and reverse primers and 4 μl of diethylpyrocarbonate (DEPC)-treated ddH_2_O were added to a final volume of 20 μl. The amplification program was as follows: 95°C for 5 min, followed by 15 s at 95°C, 15 s at 65°C, and 32 s at 72°C for 40 cycles.

The transcription levels of several target genes (*GSBRNA2T00056839001*, *GSBRNA2T00031607001*, *GSBRNA2T00044758001* and *GSBRNA2T00021710001*) of DE miRNAs were also assayed by qRT-PCR, with the following cycle conditions: denaturation at 95°C for 15 s, annealing at 60°C for 15 s, and extension at 72°C for 32 s. 18S rRNA was used as the internal standard because it is uniformly expressed in *B*. *napus* tissues [61]. All reactions of each sample were performed with three biological replicates. The comparative Ct method was used for data analysis in this study.

### Degradome analysis

Total RNA was extracted with RNA extraction kit (Invitrogen, Carlsbad, CA, USA). Three replicates of each sample were used for small RNA sequencing. The mRNA was first enriched with Oligo (dT) magnetic beads, then followed by connecting 5' joint, reverse transcription after PCR amplification, restriction enzyme *Mme*I, connecting 3' joint, PCR amplification, gel extraction target fragment, and finally analyzed by using Illumina HiSeq 2500 sequencing system. After jointing and filtering of the original tags, the clean tags and cluster tags (clustering data of clean tags) were finally obtained. Cluster tags were blast to the reference genome of *B*. *napus*, and got the distribution of tags in the genome of *B*. *napus*. Cluster tags and Rfam database were blast, and non-coding RNA was annotated. Not annotated sequences would be used for degradation site analysis. The degradation sites and cleavage product of miRNA were detected by Cleaveland software 4.0 [50]. The condition was set as *p*-value <0.05.

## Supporting information

S1 Fig **Most of miRNA sequences showed a strong bias for a uridine at position 1 and the majority of 24 nucleotide-long siRNAs have an apparent preference for 5’ adenosine in S03 (A) and S04 (B).** A represents adenosine; U represents uridine; C represents cytidine; G represents guanosine.
(TIF)

S2 Fig The foldRNA structure of every novel miRNA. (PDF)

S3 Fig KEGG pathway maps of differentially expressed miRNA targets. (PDF)

S4 Fig The T-plots and cleavage sites of 57 degraded target genes. (PDF)

S1 Table Unique and total sRNA sequences between S03 and S04. (XLSX)

S2 Table Summary of the length distribution of clean reads and miRNA by sequencing. (XLSX)

S3 Table MiRNA expression list. (XLSX)

S4 Table Totals of 1,248 novel and 28 known miRNAs obtained from S03 and S04 by sequencing. (XLSX)

S5 Table A total of 300 novel and 2 known differentially expressed miRNAs between S03 and S04. (XLSX)

S6 Table All targets of miRNAs and their sequences. (XLSX)

S7 Table Totals of 503 potential targets from 181 miRNAs. (XLSX)

S8 Table S04 vs S03 differential expressed target gene list. (XLSX)

S9 Table Summary of GO function classification of differentially expressed miRNA targets. (XLSX)

S10 Table Annotated information of 98 targets from differentially expressed miRNAs. (XLSX)

S11 Table Annotated information of 465 targets from total miRNAs. (XLSX)

S12 Table Summary of KEEG pathway of differentially expressed miRNA targets. (XLSX)

S13 Table Sixty-four cleavage sites of miRNAs from degradome sequencing. (XLSX)

S14 Table Annotated information of 57 degraded target genes. (XLSX)

S15 Table The primers and probes used in this study. (XLSX)
