# Supplementary figures and images for: Small RNA and degradome profiling involved in seed development and oil synthesis of Brassica napus
Source: PLoS One. 2018 Oct 17;13(10):e0204998. doi: 10.1371/journal.pone.0204998 (PMC6192625; doi:10.1371/journal.pone.0204998)

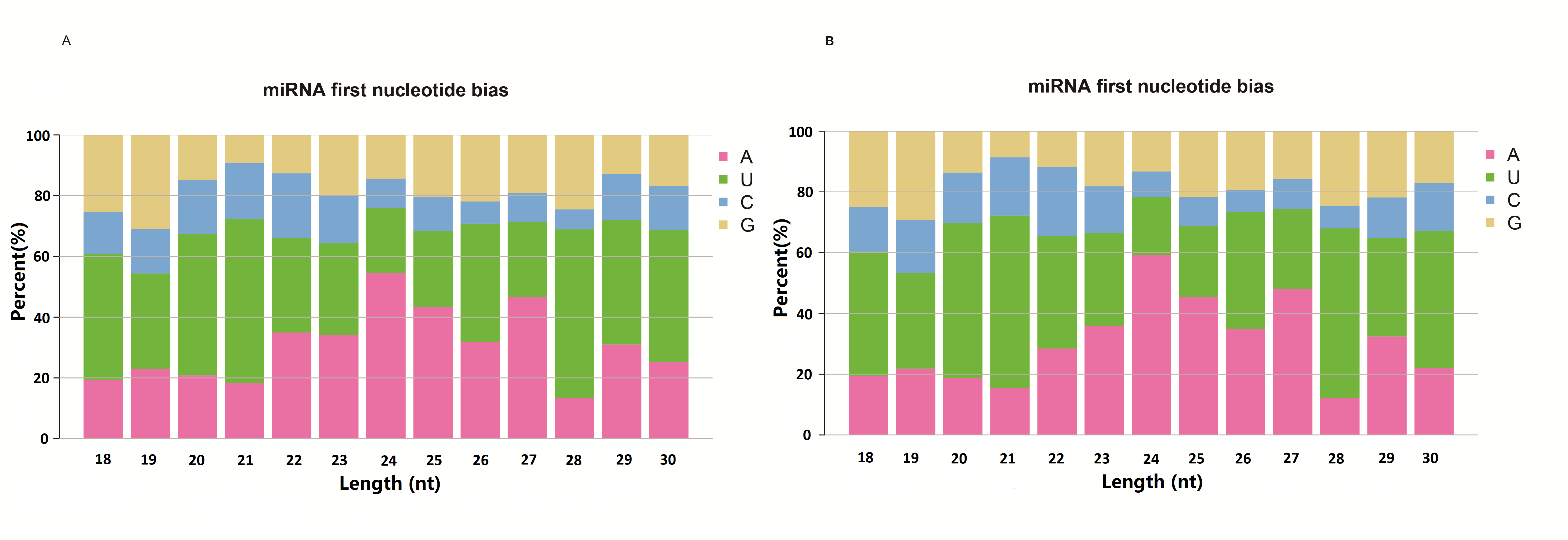

Supplement: S1 Fig — Most of miRNA sequences showed a strong bias for a uridine at position 1 and the majority of 24 nucleotide-long siRNAs have an apparent preference for 5’ adenosine in S03 (A) and S04 (B). A represents adenosine; U represents uridine; C represents cytidine; G represents guanosine. (TIF) [file pone.0204998.s001.tif]
