# Supplementary material for: Small RNA and degradome profiling involved in seed development and oil synthesis of Brassica napus
Source: PLoS One. 2018 Oct 17;13(10):e0204998. doi: 10.1371/journal.pone.0204998 (PMC6192625; doi:10.1371/journal.pone.0204998)
Supplement: S3 Fig — (PDF) [file pone.0204998.s003.pdf]

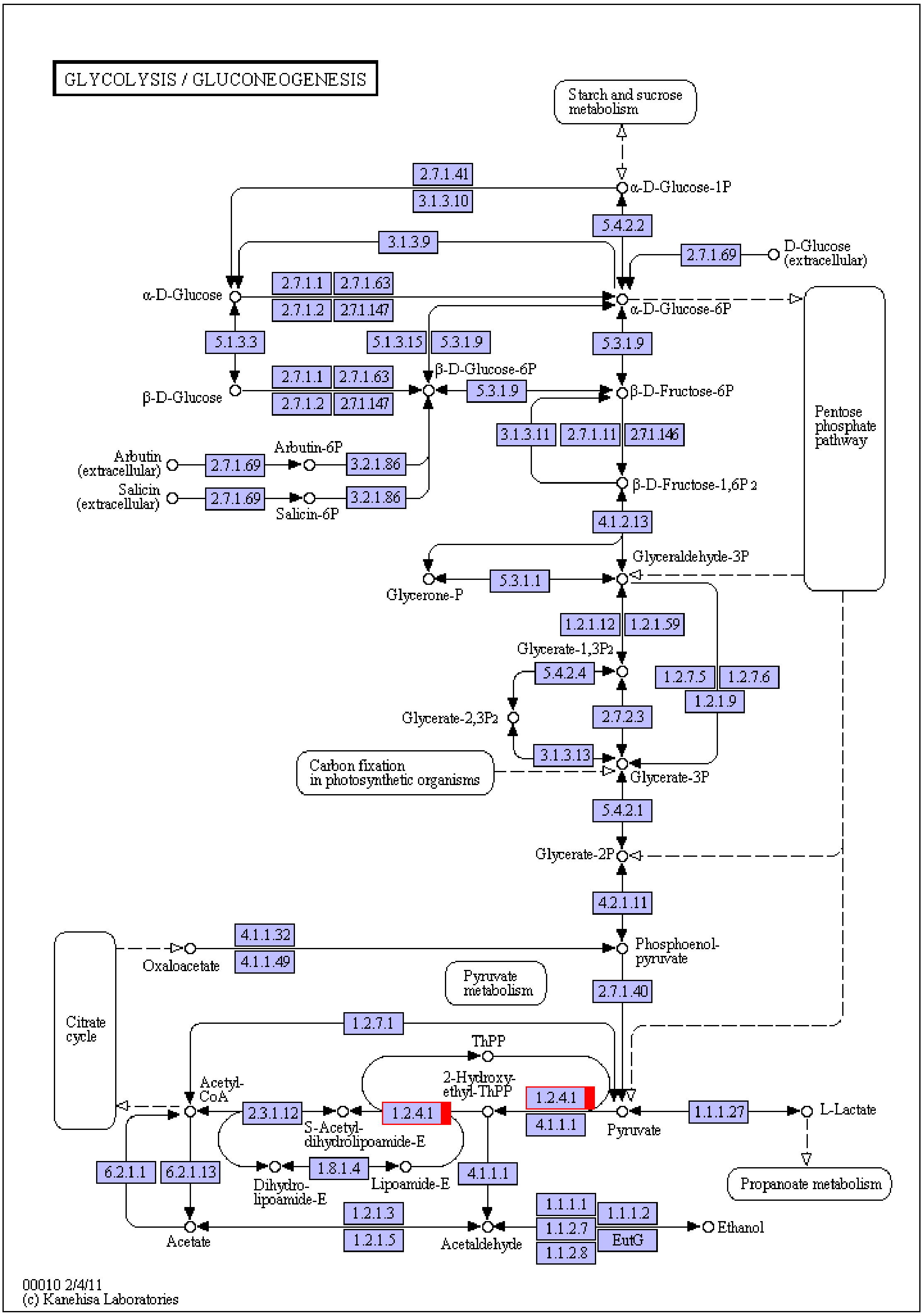
GLYCOLYSIS / GLUCONEOGENESIS
Starch and sucrose metabolism
2.7.1.41
3.1.3.10
α-D-Glucose-1P
5.4.2.2
3.1.3.9
2.7.1.69
D-Glucose (extracellular)
α-D-Glucose
2.7.1.1
2.7.1.63
2.7.1.2
2.7.1.147
α-D-Glucose-6P
5.1.3.3
5.1.3.15
5.3.1.9
β-D-Glucose
β-D-Glucose-6P
β-D-Fructose-6P
Pentose phosphate pathway
3.1.3.11
2.7.1.11
2.7.1.146
Arbutin (extracellular)
Arbutin-6P
3.2.1.86
Salicin (extracellular)
Salicin-6P
β-D-Fructose-1,6P2
4.1.2.13
Glyceraldehyde-3P
5.3.1.1
Glycerone-P
1.2.1.12
1.2.1.59
Glycerate-1,3P2
5.4.2.4
1.2.7.5
1.2.7.6
1.2.1.9
Glycerate-2,3P2
2.7.2.3
3.1.3.13
Carbon fixation in photosynthetic organisms
Glycerate-3P
5.4.2.1
Glycerate-2P
4.2.1.11
4.1.1.32
4.1.1.49
Oxaloacetate
Phosphoenol-pyruvate
Citrate cycle
Pyruvate metabolism
2.7.1.40
1.2.7.1
ThPP
2-Hydroxy-ethyl-ThPP
1.2.4.1
4.1.1.1
Acetyl-CoA
2.3.1.12
S-Acetyl-dihydrolipoamide-E
Pyruvate
1.1.1.27
L-Lactate
1.8.1.4
Dihydro-lipoamide-E
Lipoamide-E
6.2.1.1
6.2.1.13
Propanoate metabolism
1.2.1.3
1.2.1.5
1.1.1.1
1.1.1.2
1.1.2.7
EutG
1.1.2.8
Acetate
Acetaldehyde
Ethanol
00010 2/4/11
(c) Kanehisa Laboratories

Ko00010

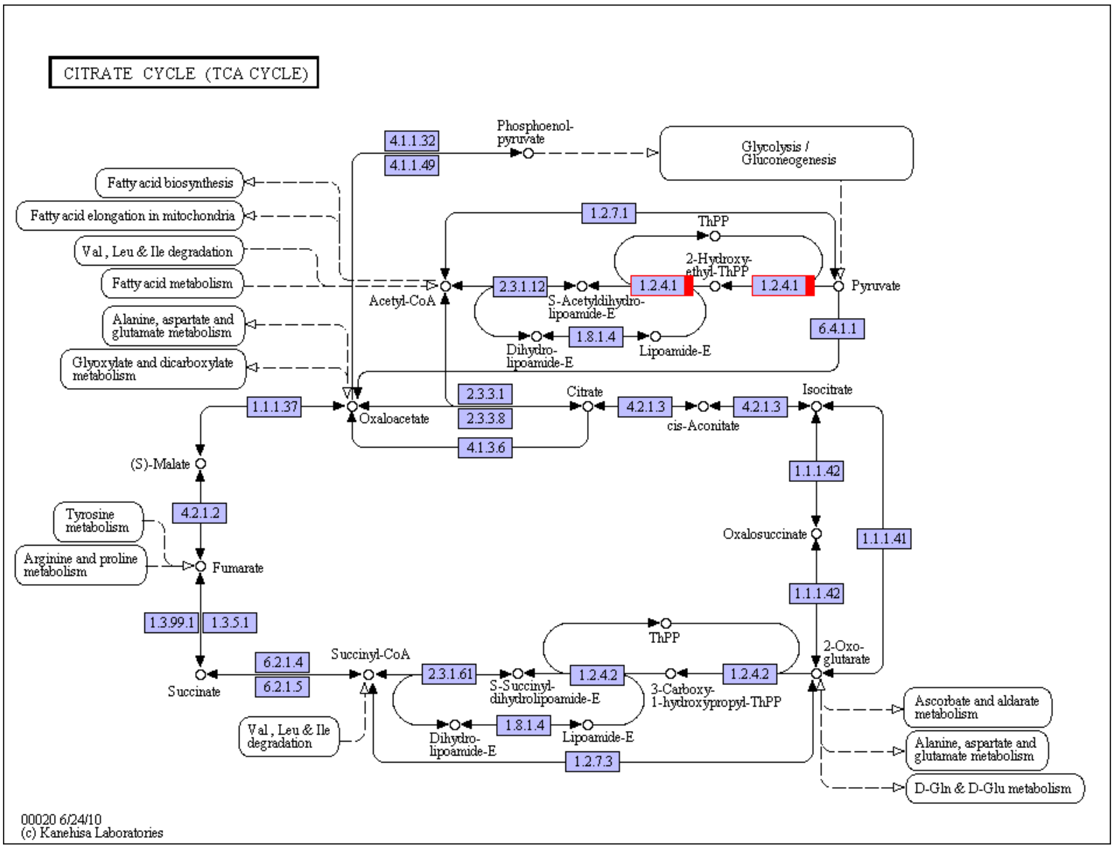
CITRATE CYCLE (TCA CYCLE)
Phosphoenol-pyruvate
Glycolysis / Gluconeogenesis
4.1.1.32
4.1.1.49
Fatty acid biosynthesis
Fatty acid elongation in mitochondria
Val, Leu & Ile degradation
Fatty acid metabolism
Alanine, aspartate and glutamate metabolism
Glyoxylate and dicarboxylate metabolism
1.2.7.1
ThPP
2-Hydroxy-ethyl-ThPP
Acetyl-CoA
2.3.1.12
S-Acetyldihydro-lipoamide-E
1.2.4.1
1.2.4.1
Pyruvate
Dihydro-lipoamide-E
1.8.1.4
Lipoamide-E
6.4.1.1
2.3.3.1
2.3.3.8
4.1.3.6
Citrate
4.2.1.3
cis-Aconitate
4.2.1.3
Isocitrate
1.1.1.37
Oxaloacetate
(S)-Malate
1.1.1.42
Oxalosuccinate
1.1.1.41
4.2.1.2
Tyrosine metabolism
Arginine and proline metabolism
Fumarate
1.1.1.42
1.3.99.1
1.3.5.1
ThPP
Succinyl-CoA
6.2.1.4
6.2.1.5
Succinate
2.3.1.61
S-Succinyl-dihydrolipoamide-E
1.2.4.2
3-Carboxy-1-hydroxypropyl-ThPP
1.2.4.2
2-Oxo-glutarate
Val, Leu & Ile degradation
Dihydro-lipoamide-E
1.8.1.4
Lipoamide-E
1.2.7.3
Ascorbate and aldarate metabolism
Alanine, aspartate and glutamate metabolism
D-Gln & D-Glu metabolism
00020 6/24/10
(c) Kanehisa Laboratories

Ko00020

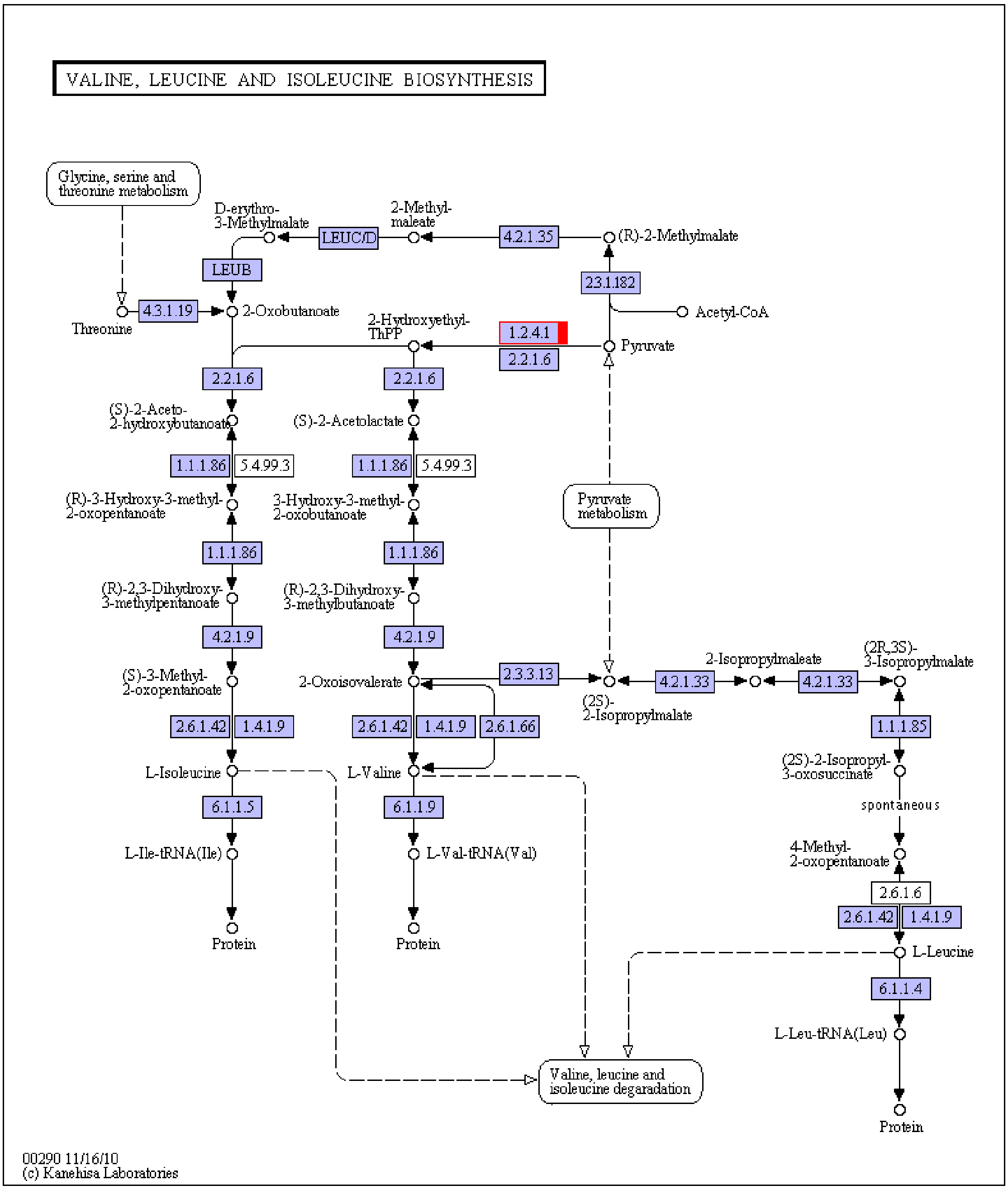
VALINE, LEUCINE AND ISOLEUCINE BIOSYNTHESIS
Glycine, serine and threonine metabolism
D-erythro-3-Methylmalate
LEUC/D
2-Methyl-maleate
4.2.1.35
(R)-2-Methylmalate
LEUB
2.3.1.182
Acetyl-CoA
Threonine
4.3.1.19
2-Oxobutanoate
2-Hydroxyethyl-ThPP
1.2.4.1
2.2.1.6
Pyruvate
2.2.1.6
2.2.1.6
(S)-2-Aceto-2-hydroxybutanoate
(S)-2-Acetolactate
1.1.1.86
5.4.99.3
1.1.1.86
5.4.99.3
(R)-3-Hydroxy-3-methyl-2-oxopentanoate
3-Hydroxy-3-methyl-2-oxobutanoate
Pyruvate metabolism
1.1.1.86
1.1.1.86
(R)-2,3-Dihydroxy-3-methylpentanoate
(R)-2,3-Dihydroxy-3-methylbutanoate
4.2.1.9
4.2.1.9
(S)-3-Methyl-2-oxopentanoate
2-Oxoisovalerate
2.3.3.13
(2S)-2-Isopropylmalate
4.2.1.33
2-Isopropylmaleate
4.2.1.33
(2R,3S)-3-Isopropylmalate
2.6.1.42
1.4.1.9
2.6.1.42
1.4.1.9
2.6.1.66
1.1.1.85
L-Isoleucine
L-Valine
(2S)-2-Isopropyl-3-oxosuccinate
6.1.1.5
6.1.1.9
spontaneous
L-Ile-tRNA(Ile)
L-Val-tRNA(Val)
4-Methyl-2-oxopentanoate
2.6.1.6
2.6.1.42
1.4.1.9
Protein
Protein
L-Leucine
6.1.1.4
L-Leu-tRNA(Leu)
Valine, leucine and isoleucine degaradation
Protein
00290 11/16/10
(c) Kanehisa Laboratories

Ko00290

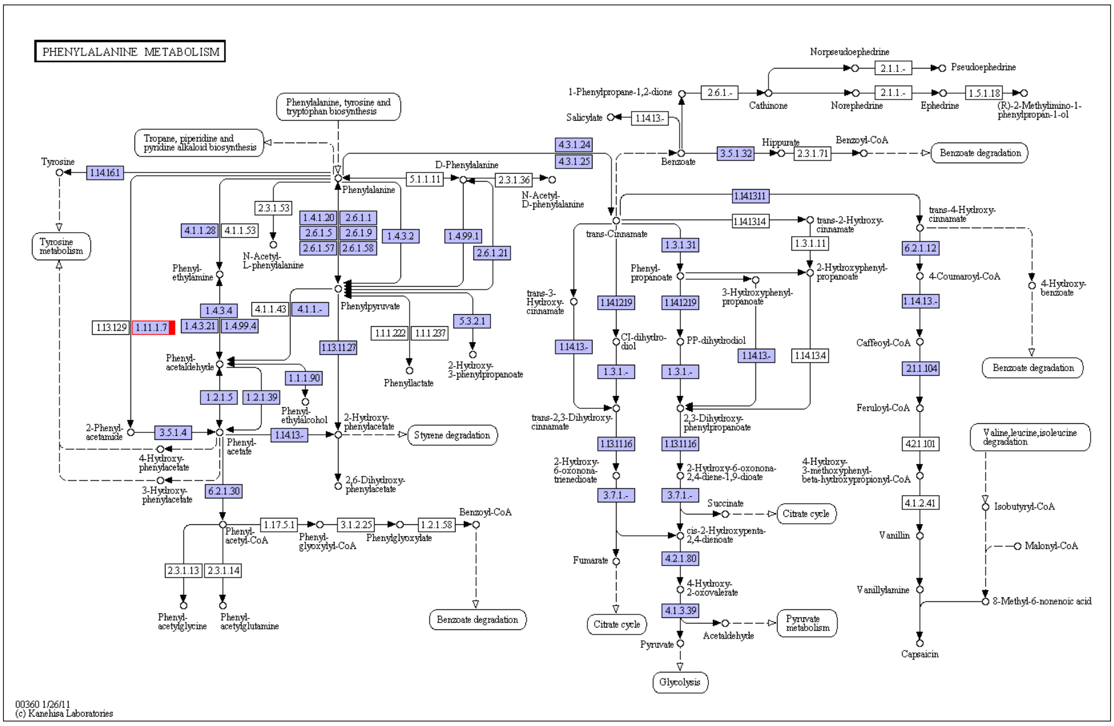


Ko000360

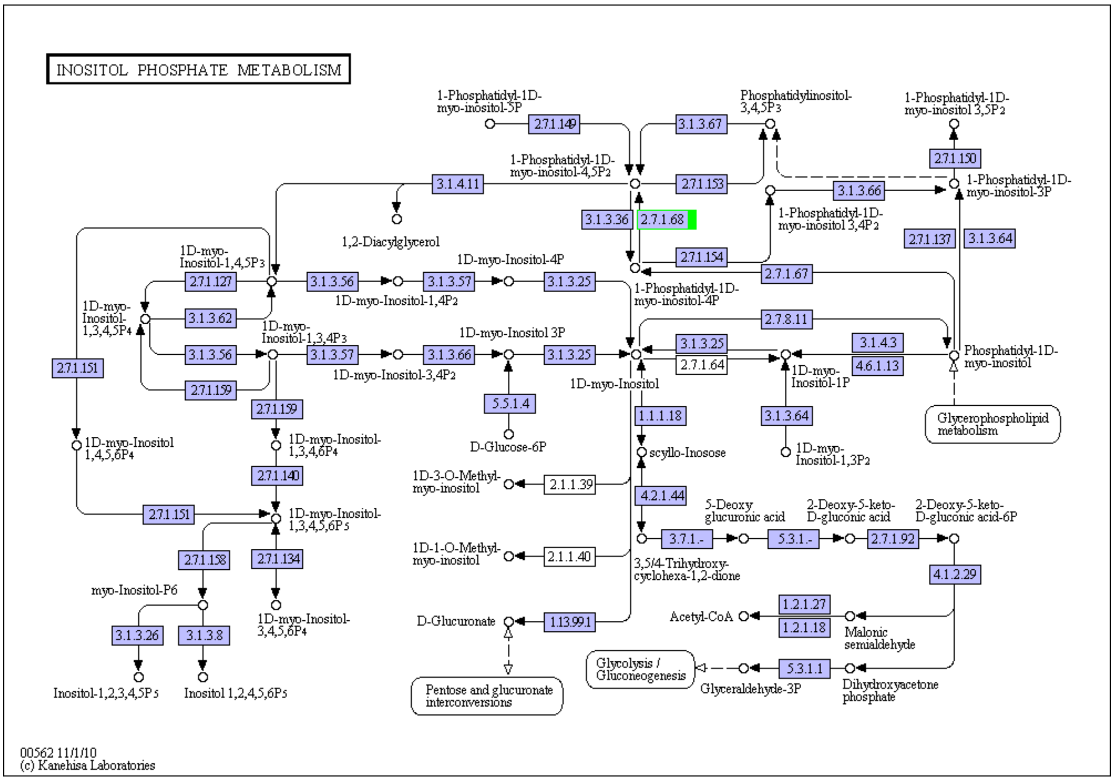


Ko00562

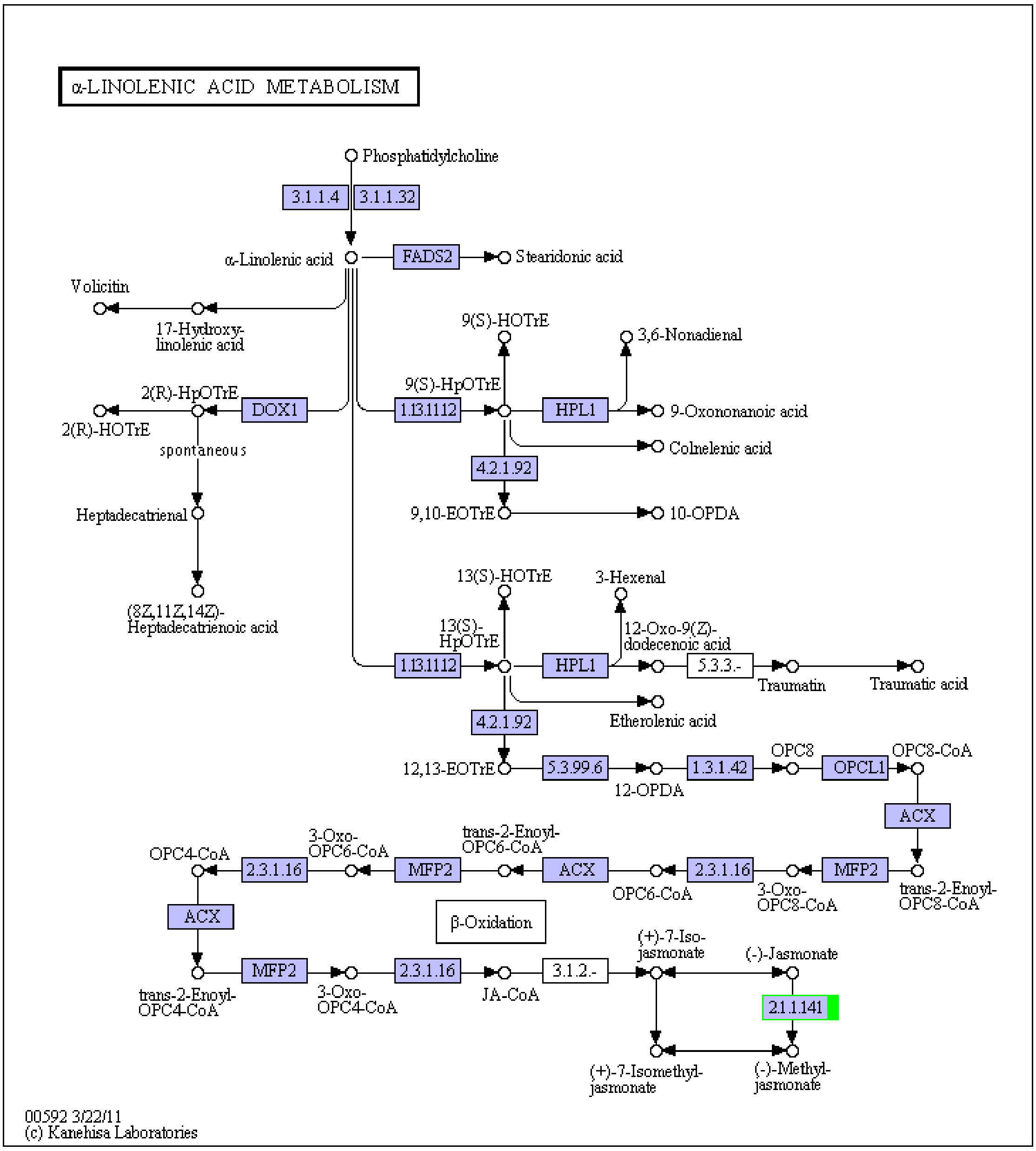
α-LINOLENIC ACID METABOLISM
Phosphatidylcholine
3.1.1.4
3.1.1.32
α-Linolenic acid
FADS2
Stearidonic acid
Volicitin
17-Hydroxy-linolenic acid
9(S)-HOTrE
3,6-Nonadienal
2(R)-HpOTrE
DOX1
2(R)-HOTrE
9(S)-HpOTrE
1.13.11.12
HPL1
9-Oxononanoic acid
Colnelenic acid
spontaneous
4.2.1.92
Heptadecatrienal
9,10-EOTrE
10-OPDA
13(S)-HOTrE
3-Hexenal
(8Z,11Z,14Z)-Heptadecatrienoic acid
13(S)-HpOTrE
12-Oxo-9(Z)-dodecenoic acid
1.13.11.12
HPL1
5.3.3.-
Traumatin
Traumatic acid
Etherolenic acid
4.2.1.92
OPC8
OPC8-CoA
12,13-EOTrE
5.3.99.6
1.3.1.42
OPCL1
12-OPDA
ACX
3-Oxo-OPC6-CoA
trans-2-Enoyl-OPC6-CoA
OPC4-CoA
2.3.1.16
MFP2
ACX
2.3.1.16
MFP2
OPC6-CoA
3-Oxo-OPC8-CoA
trans-2-Enoyl-OPC8-CoA
ACX
β-Oxidation
(+)-7-Iso-jasmonate
(-)-Jasmonate
MFP2
2.3.1.16
3.1.2.-
trans-2-Enoyl-OPC4-CoA
3-Oxo-OPC4-CoA
JA-CoA
2.1.1.141
(+)-7-Isomethyl-jasmonate
(-)-Methyl-jasmonate
00592 3/22/11
(c) Kanehisa Laboratories

Ko00592

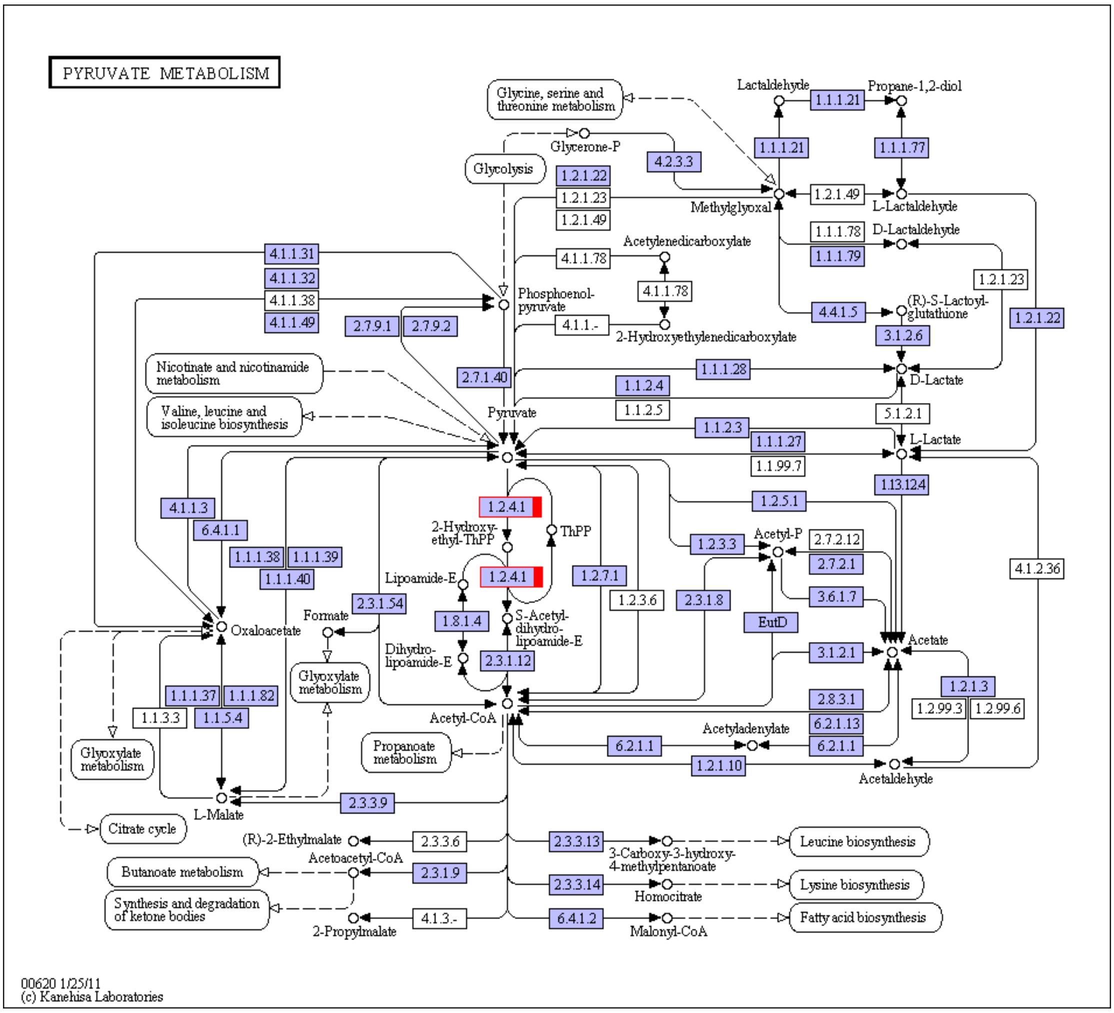
PYRUVATE METABOLISM
Glycine, serine and threonine metabolism
Lactaldehyde
Propane-1,2-diol
1.1.1.21
1.1.1.21
1.1.1.77
Glycerone-P
4.2.3.3
Glycolysis
1.2.1.22
1.2.1.23
1.2.1.49
Methylglyoxal
1.2.1.49
L-Lactaldehyde
1.1.1.78
D-Lactaldehyde
1.1.1.79
Acetylenedicarboxylate
4.1.1.78
4.1.1.31
4.1.1.32
4.1.1.38
4.1.1.49
Phosphoenol-pyruvate
4.1.1.78
1.2.1.23
4.4.1.5
(R)-S-Lactoyl-glutathione
1.2.1.22
2.7.9.1
2.7.9.2
4.1.1.-
2-Hydroxyethylenedicarboxylate
3.1.2.6
Nicotinate and nicotinamide metabolism
1.1.1.28
D-Lactate
2.7.1.40
1.1.2.4
Valine, leucine and isoleucine biosynthesis
1.1.2.5
5.1.2.1
Pyruvate
1.1.2.3
1.1.1.27
L-Lactate
1.1.99.7
1.13.12.4
4.1.1.3
1.2.4.1
1.2.5.1
6.4.1.1
2-Hydroxy-ethyl-ThPP
ThPP
1.2.3.3
Acetyl-P
2.7.2.12
1.1.1.38
1.1.1.39
2.7.2.1
4.1.2.36
1.1.1.40
Lipoamide-E
1.2.4.1
1.2.7.1
3.6.1.7
2.3.1.54
1.2.3.6
2.3.1.8
Formate
1.8.1.4
S-Acetyl-dihydro-lipoamide-E
EutD
Oxaloacetate
Acetate
Dihydro-lipoamide-E
2.3.1.12
3.1.2.1
Glyoxylate metabolism
1.2.1.3
1.1.1.37
1.1.1.82
2.8.3.1
1.2.99.3
1.2.99.6
1.1.3.3
1.1.5.4
Acetyl-CoA
6.2.1.13
Glyoxylate metabolism
Acetyladenylate
Propanoate metabolism
6.2.1.1
6.2.1.1
1.2.1.10
Acetaldehyde
2.3.3.9
L-Malate
Citrate cycle
(R)-2-Ethylmalate
2.3.3.6
2.3.3.13
3-Carboxy-3-hydroxy-4-methylpentanoate
Leucine biosynthesis
Acetoacetyl-CoA
Butanoate metabolism
2.3.1.9
2.3.3.14
Homocitrate
Lysine biosynthesis
Synthesis and degradation of ketone bodies
4.1.3.-
6.4.1.2
2-Propylmalate
Malonyl-CoA
Fatty acid biosynthesis
00620 1/25/11
(c) Kanehisa Laboratories

Ko00620

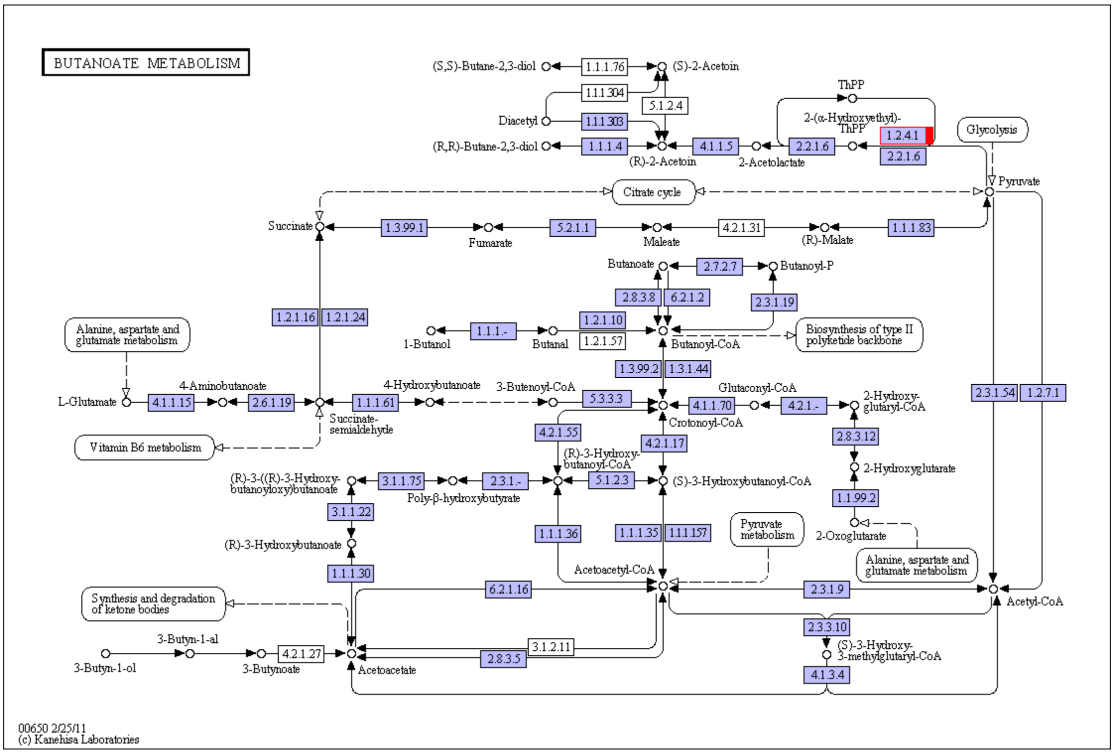


Ko00650

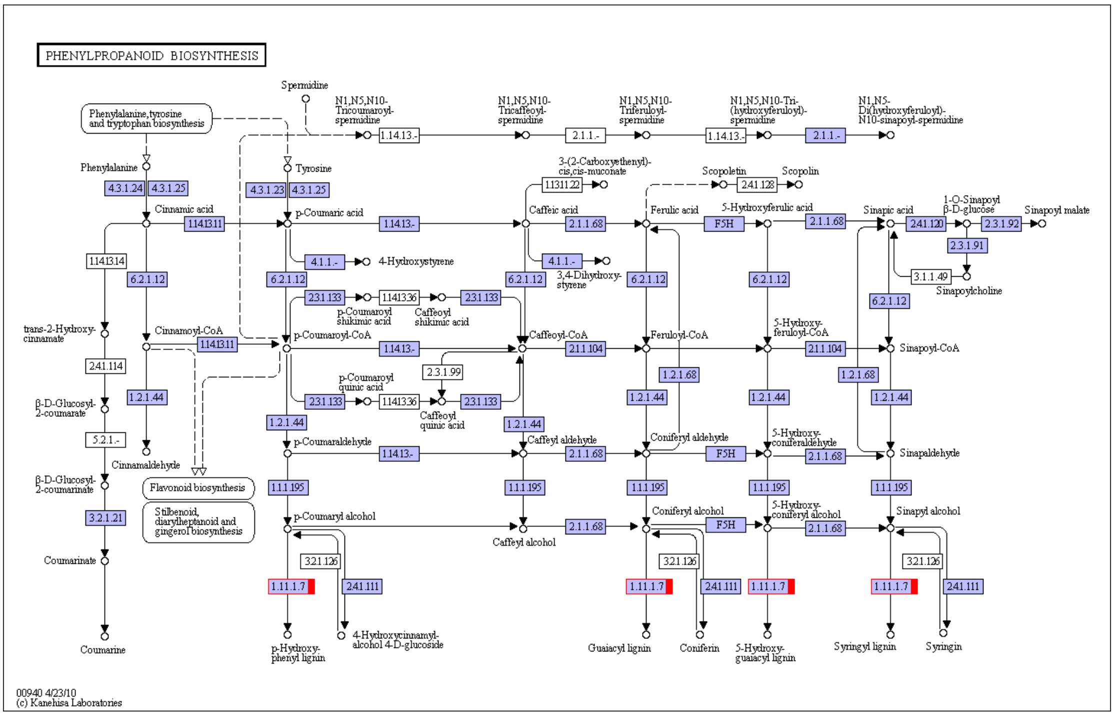


Ko00940

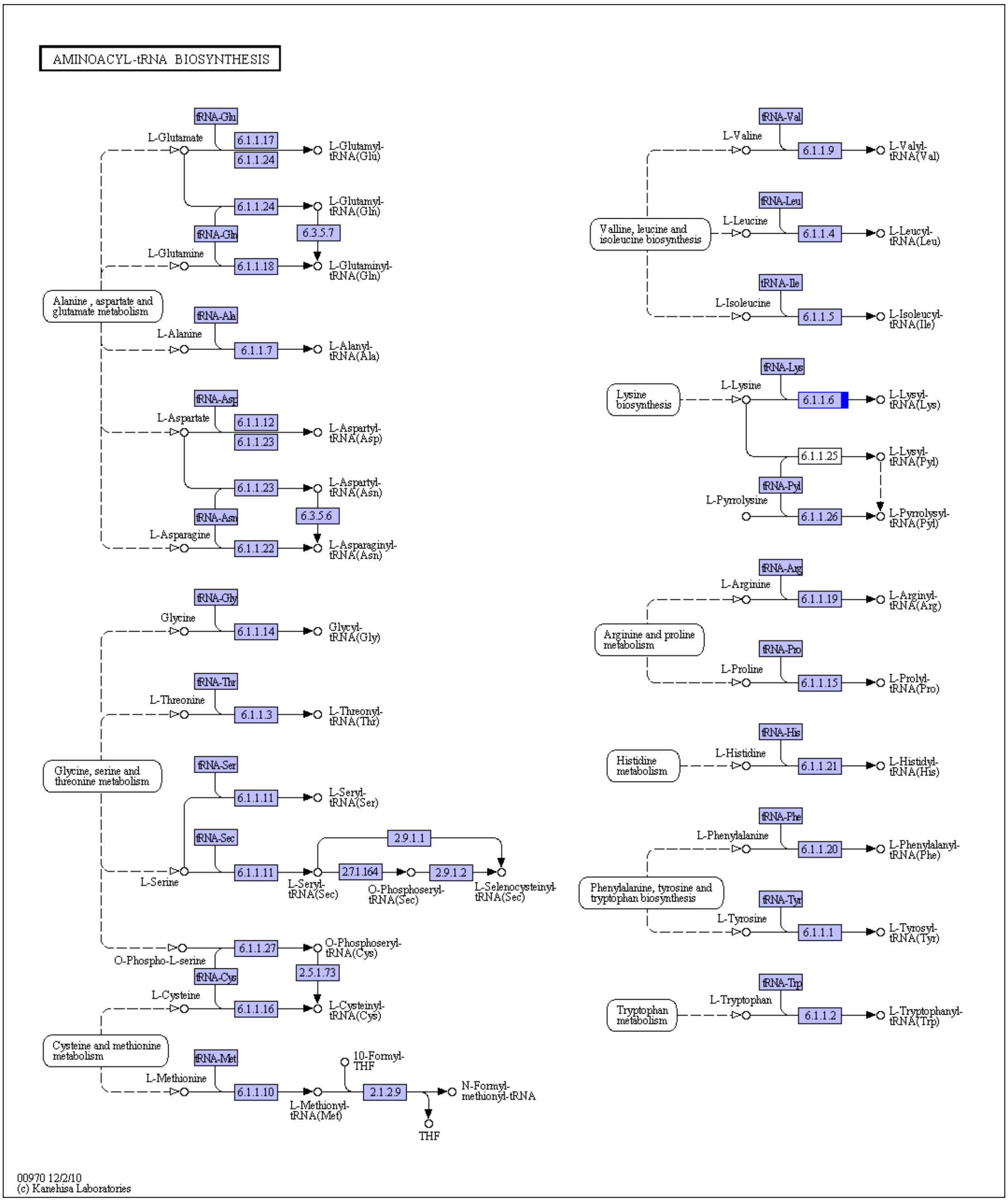
AMINOACYL-tRNA BIOSYNTHESIS
tRNA-Glu
L-Glutamate
6.1.1.17
6.1.1.24
L-Glutamyl-tRNA(Glu)
L-Glutamyl-tRNA(Gln)
tRNA-Gln
6.3.5.7
L-Glutamine
6.1.1.18
L-Glutaminyl-tRNA(Gln)
Alanine , aspartate and glutamate metabolism
tRNA-Ala
L-Alanine
6.1.1.7
L-Alanyl-tRNA(Ala)
tRNA-Asp
L-Aspartate
6.1.1.12
6.1.1.23
L-Aspartyl-tRNA(Asp)
L-Aspartyl-tRNA(Asn)
tRNA-Asn
6.3.5.6
L-Asparagine
6.1.1.22
L-Asparaginyl-tRNA(Asn)
tRNA-Gly
Glycine
6.1.1.14
Glycyl-tRNA(Gly)
tRNA-Thr
L-Threonine
6.1.1.3
L-Threonyl-tRNA(Thr)
Glycine, serine and threonine metabolism
tRNA-Ser
6.1.1.11
L-Seryl-tRNA(Ser)
tRNA-Sec
2.9.1.1
L-Serine
L-Seryl-tRNA(Sec)
2.7.1.164
O-Phosphoseryl-tRNA(Sec)
2.9.1.2
L-Selenocysteinyl-tRNA(Sec)
O-Phospho-L-serine
6.1.1.27
O-Phosphoseryl-tRNA(Cys)
tRNA-Cys
2.5.1.73
L-Cysteine
6.1.1.16
L-Cysteinyl-tRNA(Cys)
Cysteine and methionine metabolism
tRNA-Met
L-Methionine
6.1.1.10
L-Methionyl-tRNA(Met)
10-Formyl-THF
2.1.2.9
N-Formyl-methionyl-tRNA
THF
tRNA-Val
L-Valine
6.1.1.9
L-Valyl-tRNA(Val)
Valline, leucine and isoleucine biosynthesis
tRNA-Leu
L-Leucine
6.1.1.4
L-Leucyl-tRNA(Leu)
tRNA-Ile
L-Isoleucine
6.1.1.5
L-Isoleucyl-tRNA(Ile)
tRNA-Lys
Lysine biosynthesis
L-Lysine
6.1.1.6
L-Lysyl-tRNA(Lys)
6.1.1.25
L-Lysyl-tRNA(Pyl)
tRNA-Pyl
L-Pyrrolysine
6.1.1.26
L-Pyrrolysyl-tRNA(Pyl)
tRNA-Arg
L-Arginine
6.1.1.19
L-Arginyl-tRNA(Arg)
Arginine and proline metabolism
tRNA-Pro
L-Proline
6.1.1.15
L-Prolyl-tRNA(Pro)
tRNA-His
Histidine metabolism
L-Histidine
6.1.1.21
L-Histidyl-tRNA(His)
tRNA-Phe
L-Phenylalanine
6.1.1.20
L-Phenylalanyl-tRNA(Phe)
Phenylalanine, tyrosine and tryptophan biosynthesis
tRNA-Tyr
L-Tyrosine
6.1.1.1
L-Tyrosyl-tRNA(Tyr)
tRNA-Trp
Tryptophan metabolism
L-Tryptophan
6.1.1.2
L-Tryptophanyl-tRNA(Trp)
00970 12/2/10
(c) Kanehisa Laboratories

Ko00970

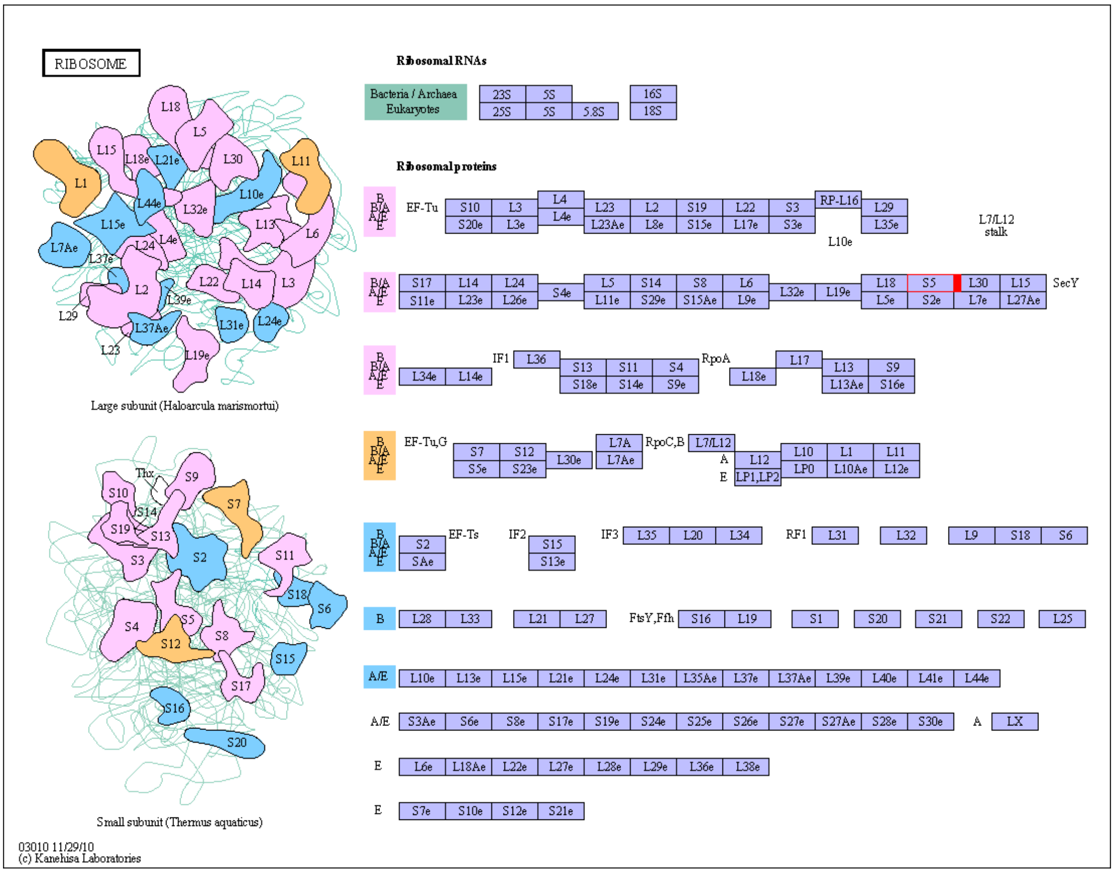
RIBOSOME
Ribosomal RNAs
Bacteria / Archaea
Eukaryotes
23S
5S
16S
25S
5S
5.8S
18S
Ribosomal proteins
Large subunit (Haloarcula marismortui)
Small subunit (Thermus aquaticus)
EF-Tu
EF-Tu,G
EF-Ts
IF1
IF2
IF3
RpoA
RpoC,B
RF1
FtsY,Ffh
SecY
L7/L12 stalk
L10e
03010 11/29/10
(c) Kanehisa Laboratories

Ko03010

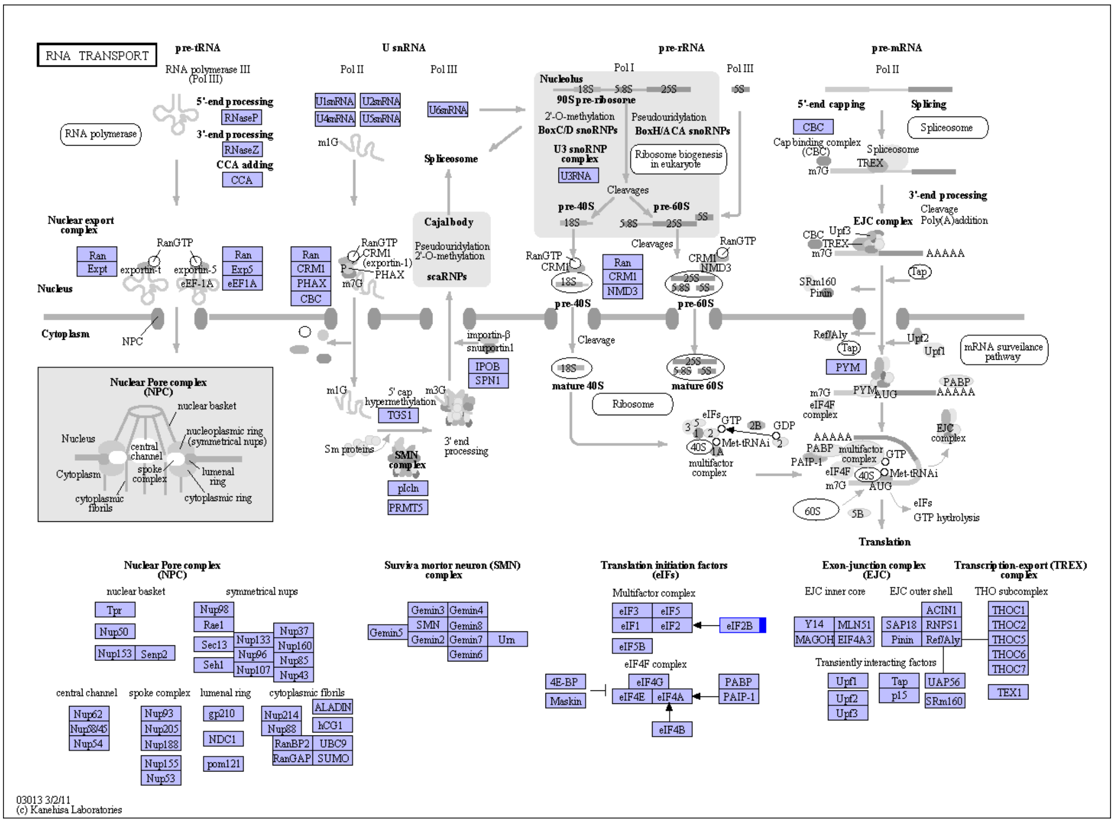

RNA TRANSPORT
pre-tRNA
RNA polymerase III (Pol III)
5'-end processing
RNaseP
3'-end processing
RNaseZ
CCA adding
CCA
RNA polymerase
U snRNA
Pol II
Pol III
U1snRNA
U2snRNA
U4snRNA
U5snRNA
U6snRNA
m1G
Spliceosome
pre-rRNA
Pol I
Pol III
Nucleolus
18S
5.8S
25S
5S
90S pre-ribosome
2'-O-methylation
BoxC/D snoRNPs
Pseudouridylation
BoxH/ACA snoRNPs
U3 snoRNP complex
U3RNA
Ribosome biogenesis in eukaryote
Cleavages
pre-40S
pre-60S
pre-mRNA
Pol II
5'-end capping
Splicing
CBC
Spliceosome
Cap binding complex (CBC)
m7G
TREX
3'-end processing
Cleavage
Poly(A)addition
EJC complex
Upf3
AAAAA
Tap
SRm160
Pinin
Nuclear export complex
RanGTP
Ran
Expt
exportin-t
exportin-5
eEF-1A
Exp5
eEF1A
Nucleus
Ran
CRM1
PHAX
CBC
RanGTP
CRM1 (exportin-1)
PHAX
m7G
Cajal body
Pseudouridylation
2'-O-methylation
scaRNPs
RanGTP
CRM1
Cleavages
Ran
CRM1
NMD3
Cytoplasm
NPC
importin-β
snurportin1
IPOB
SPN1
Cleavage
mature 40S
mature 60S
Ribosome
Ref/Aly
Upf2
Upf1
mRNA surveillance pathway
PYM
PABP
Nuclear Pore complex (NPC)
nuclear basket
nucleoplasmic ring (symmetrical nups)
central channel
spoke complex
lumenal ring
cytoplasmic fibrils
cytoplasmic ring
m1G
5' cap hypermethylation
m3G
TGS1
Sm proteins
SMN complex
3' end processing
pICln
PRMT5
eIFs
GTP
GDP
2B
Met-tRNAi
40S
multifactor complex
eIF4F complex
PAIP-1
AUG
EJC complex
60S
5B
GTP hydrolysis
Translation
Nuclear Pore complex (NPC)
nuclear basket
symmetrical nups
Tpr
Nup50
Nup153
Senp2
Nup98
Rae1
Sec13
Seh1
Nup133
Nup96
Nup107
Nup37
Nup160
Nup85
Nup43
central channel
spoke complex
lumenal ring
cytoplasmic fibrils
Nup62
Nup58/45
Nup54
Nup93
Nup205
Nup188
Nup155
Nup53
gp210
NDC1
pom121
Nup214
Nup88
RanBP2
RanGAP
ALADIN
hCG1
UBC9
SUMO
Surviva mortor neuron (SMN) complex
Gemin3
Gemin4
SMN
Gemin8
Gemin5
Gemin2
Gemin7
Unr
Gemin6
Translation initiation factors (eIFs)
Multifactor complex
eIF3
eIF5
eIF1
eIF2
eIF2B
eIF5B
eIF4F complex
4E-BP
Maskin
eIF4G
eIF4E
eIF4A
PABP
PAIP-1
eIF4B
Exon-junction complex (EJC)
EJC inner core
EJC outer shell
Y14
MLN51
MAGOH
EIF4A3
SAP18
ACIN1
RNPS1
Pinin
Ref/Aly
Transiently interacting factors
Upf1
Upf2
Upf3
Tap
p15
UAP56
SRm160
Transcription-export (TREX) complex
THO subcomplex
THOC1
THOC2
THOC5
THOC6
THOC7
TEX1
03013 3/2/11
(c) Kanehisa Laboratories


Ko03013

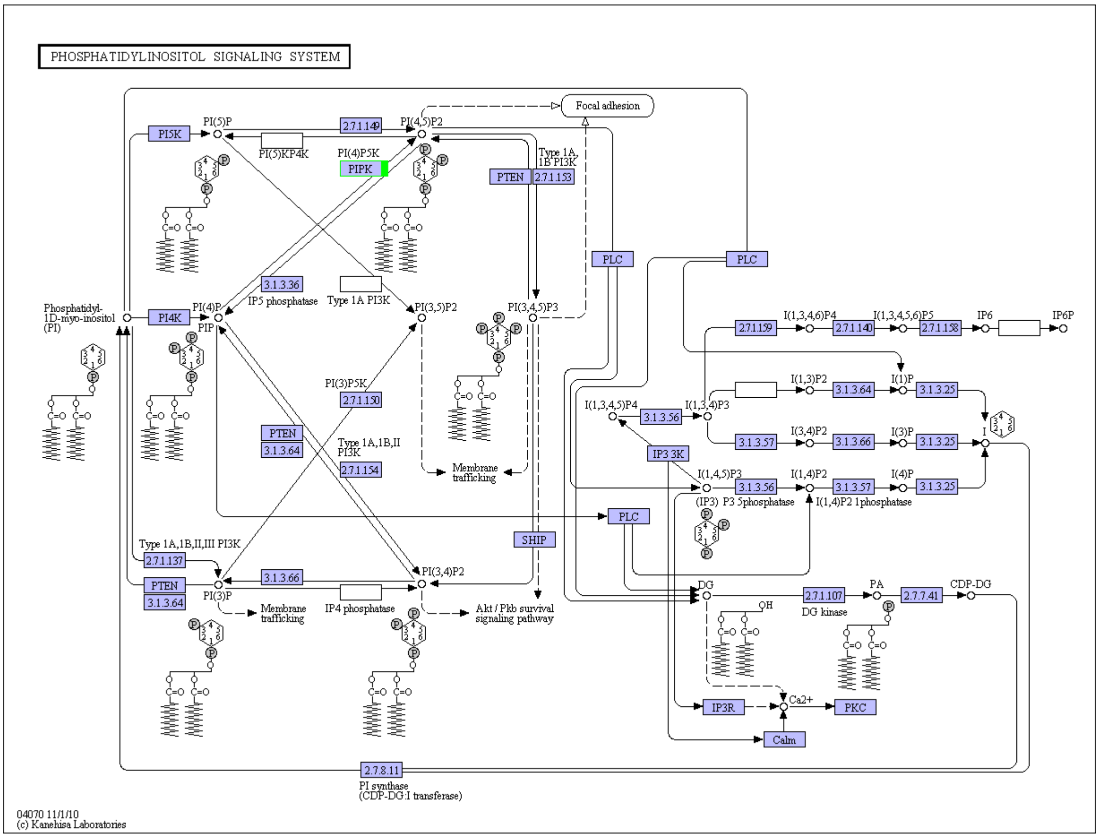
PHOSPHATIDYLINOSITOL SIGNALING SYSTEM
Focal adhesion
PI5K
PI(5)P
2.7.1.149
PI(4,5)P2
PI(5)P4K
PI(4)P5K
PIPK
Type 1A,
1B PI3K
PTEN
2.7.1.153
PLC
3.1.3.36
IP5 phosphatase
Type 1A PI3K
PI(3,5)P2
PI(3,4,5)P3
Phosphatidyl-
1D-myo-inositol
(PI)
PI4K
PI(4)P
PIP
PI(3)P5K
2.7.1.150
PTEN
3.1.3.64
Type 1A,1B,II
PI3K
2.7.1.154
Membrane
trafficking
SHIP
Type 1A,1B,II,III PI3K
2.7.1.137
3.1.3.66
PI(3,4)P2
PI(3)P
IP4 phosphatase
Akt / Pkb survival
signaling pathway
2.7.1.159
I(1,3,4,6)P4
2.7.1.140
I(1,3,4,5,6)P5
2.7.1.158
IP6
IP6P
I(1,3)P2
3.1.3.64
I(1)P
3.1.3.25
I(1,3,4,5)P4
3.1.3.56
I(1,3,4)P3
3.1.3.57
I(3,4)P2
I(3)P
I
IP3 3K
I(1,4,5)P3
(IP3) P3 5phosphatase
I(1,4)P2
I(1,4)P2 1phosphatase
I(4)P
DG
2.7.1.107
DG kinase
PA
2.7.7.41
CDP-DG
OH
IP3R
Ca2+
PKC
Calm
2.7.8.11
PI synthase
(CDP-DG:I transferase)
04070 11/1/10
(c) Kanehisa Laboratories

Ko04070

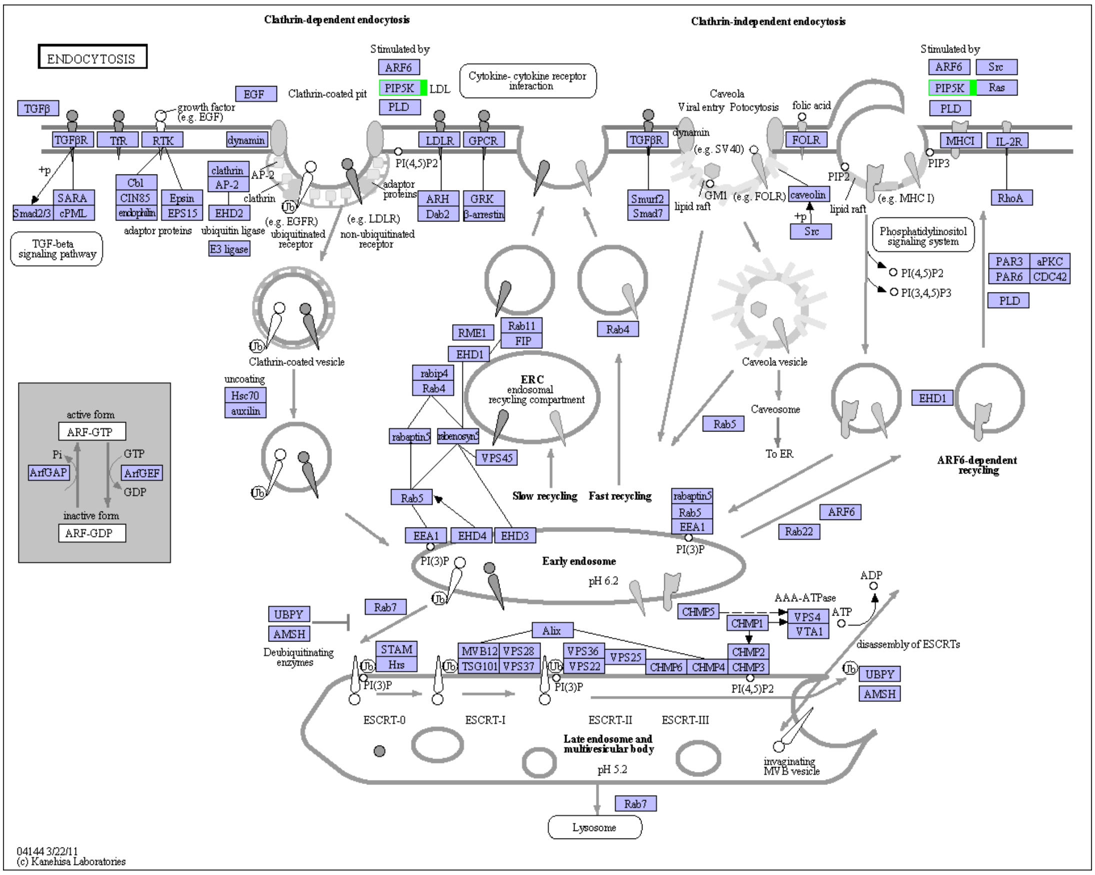
Clathrin-dependent endocytosis
Clathrin-independent endocytosis
ENDOCYTOSIS
Stimulated by
ARF6
PIP5K
PLD
LDL
EGF
Clathrin-coated pit
Cytokine- cytokine receptor interaction
TGFβ
growth factor (e.g. EGF)
TGFβR
TfR
RTK
dynamin
LDLR
GPCR
PI(4,5)P2
AP-2
clathrin
adaptor proteins
SARA
Smad2/3
cPML
Cbl
CIN85
endophilin
Epsin
EPS15
AP-2
EHD2
E3 ligase
ARH
Dab2
GRK
β-arrestin
adaptor proteins
ubiquitin ligase
(e.g. EGFR) ubiquitinated receptor
(e.g. LDLR) non-ubiquitinated receptor
TGF-beta signaling pathway
Clathrin-coated vesicle
uncoating
Hsc70
auxilin
active form
ARF-GTP
ArfGAP
ArfGEF
Pi
GTP
GDP
inactive form
ARF-GDP
RME1
Rab11
FIP
EHD1
rabip4
Rab4
ERC endosomal recycling compartment
rabaptin5
rabenosyn5
VPS45
Rab5
Rab4
Slow recycling
Fast recycling
EEA1
EHD4
EHD3
PI(3)P
Early endosome
pH 6.2
rabaptin5
Rab5
EEA1
Caveola
Viral entry
Potocytosis
folic acid
TGFβR
Smurf2
Smad7
dynamin
(e.g. SV40)
GM1
lipid raft
(e.g. FOLR)
FOLR
caveolin
Src
PIP2
PIP3
lipid raft
(e.g. MHC I)
Stimulated by
ARF6
Src
PIP5K
Ras
PLD
MHCI
IL-2R
RhoA
Phosphatidylinositol signaling system
PI(4,5)P2
PI(3,4,5)P3
PAR3
aPKC
PAR6
CDC42
PLD
Caveola vesicle
Caveosome
Rab5
To ER
EHD1
ARF6-dependent recycling
ARF6
Rab22
UBPY
AMSH
Deubiquitinating enzymes
Rab7
STAM
Hrs
Alix
MVB12
VPS28
TSG101
VPS37
VPS36
VPS22
VPS25
CHMP6
CHMP4
CHMP2
CHMP3
CHMP5
CHMP1
VPS4
VTA1
AAA-ATPase
ADP
ATP
disassembly of ESCRTs
UBPY
AMSH
PI(3)P
PI(4,5)P2
ESCRT-0
ESCRT-I
ESCRT-II
ESCRT-III
Late endosome and multivesicular body
pH 5.2
invaginating MVB vesicle
Rab7
Lysosome
04144 3/22/11
(c) Kanehisa Laboratories

Ko04144
