# Supplementary material for: Small RNA and degradome profiling involved in seed development and oil synthesis of Brassica napus
Source: PLoS One. 2018 Oct 17;13(10):e0204998. doi: 10.1371/journal.pone.0204998 (PMC6192625; doi:10.1371/journal.pone.0204998)
Supplement: S4 Fig — (PDF) [file pone.0204998.s004.pdf]

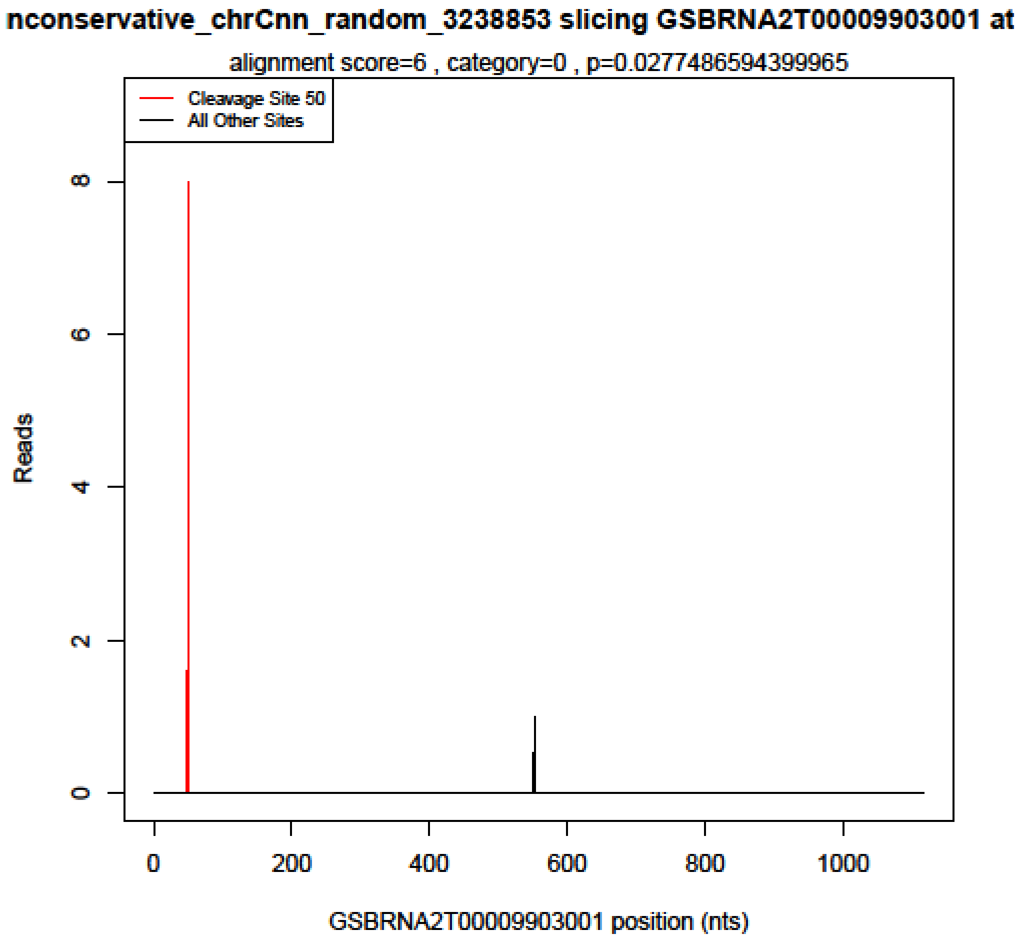

nconservative_chrCnn_random_3238853 slicing GSBRNA2T00009903001 at
alignment score=6 , category=0 , p=0.0277486594399965
Cleavage Site 50
All Other Sites
Reads
0
2
4
6
8
0
200
400
600
800
1000
GSBRNA2T00009903001 position (nts)

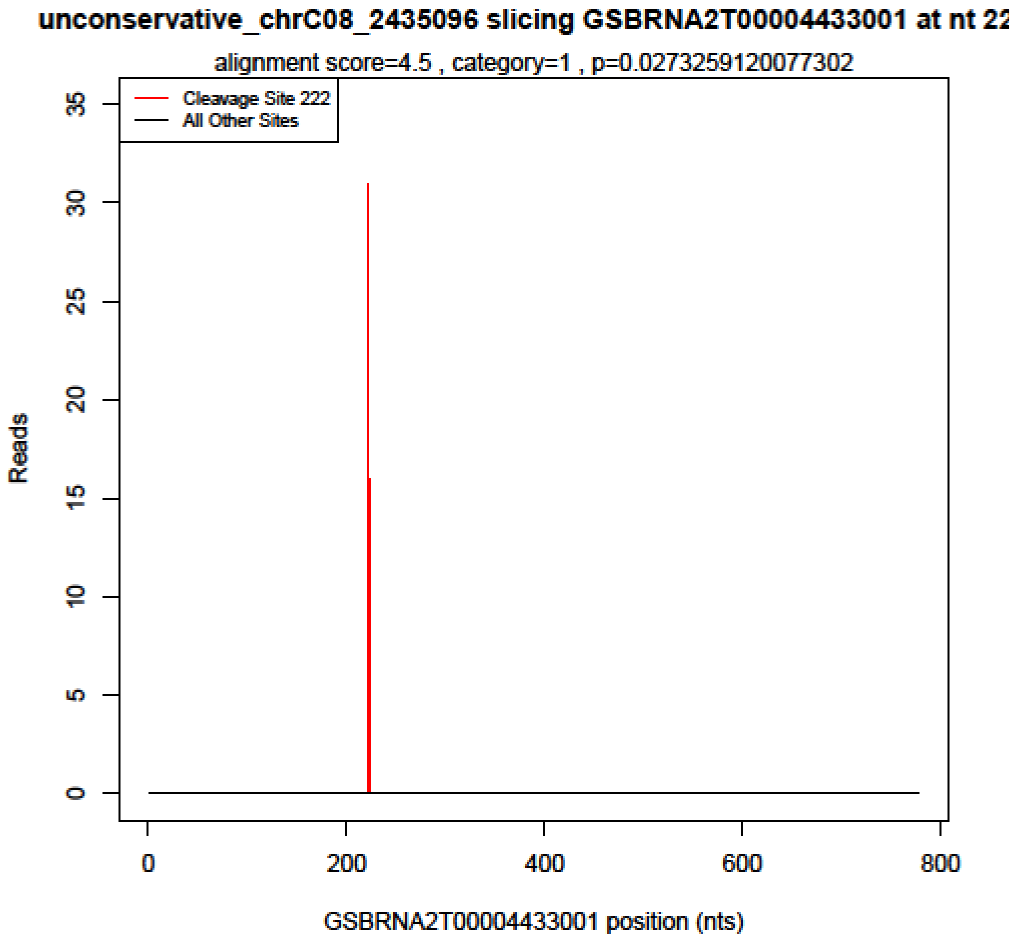
unconservative_chrC08_2435096 slicing GSBRNA2T00004433001 at nt 22
alignment score=4.5 , category=1 , p=0.0273259120077302
Cleavage Site 222
All Other Sites
Reads
0
5
10
15
20
25
30
35
0
200
400
600
800
GSBRNA2T00004433001 position (nts)

ıconservative_chrCnn_random_3238853 slicing GSBRNA2T00012841001 at

alignment score=5 , category=2 , p=0.0219365089750464

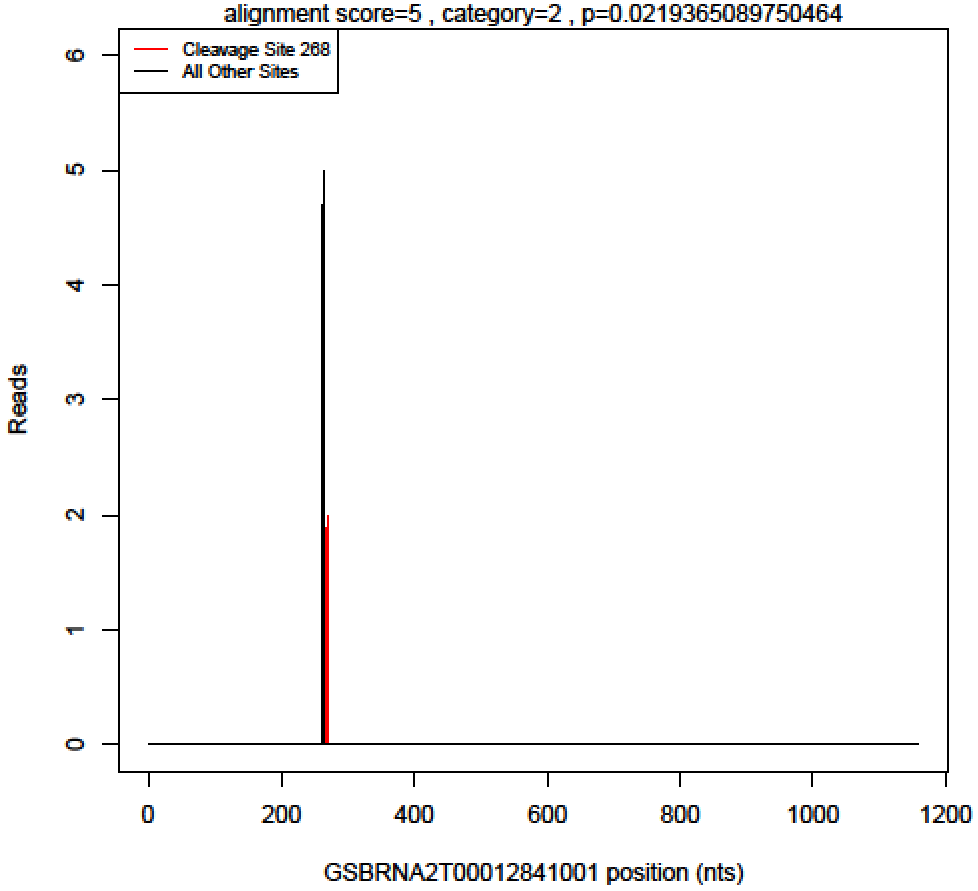

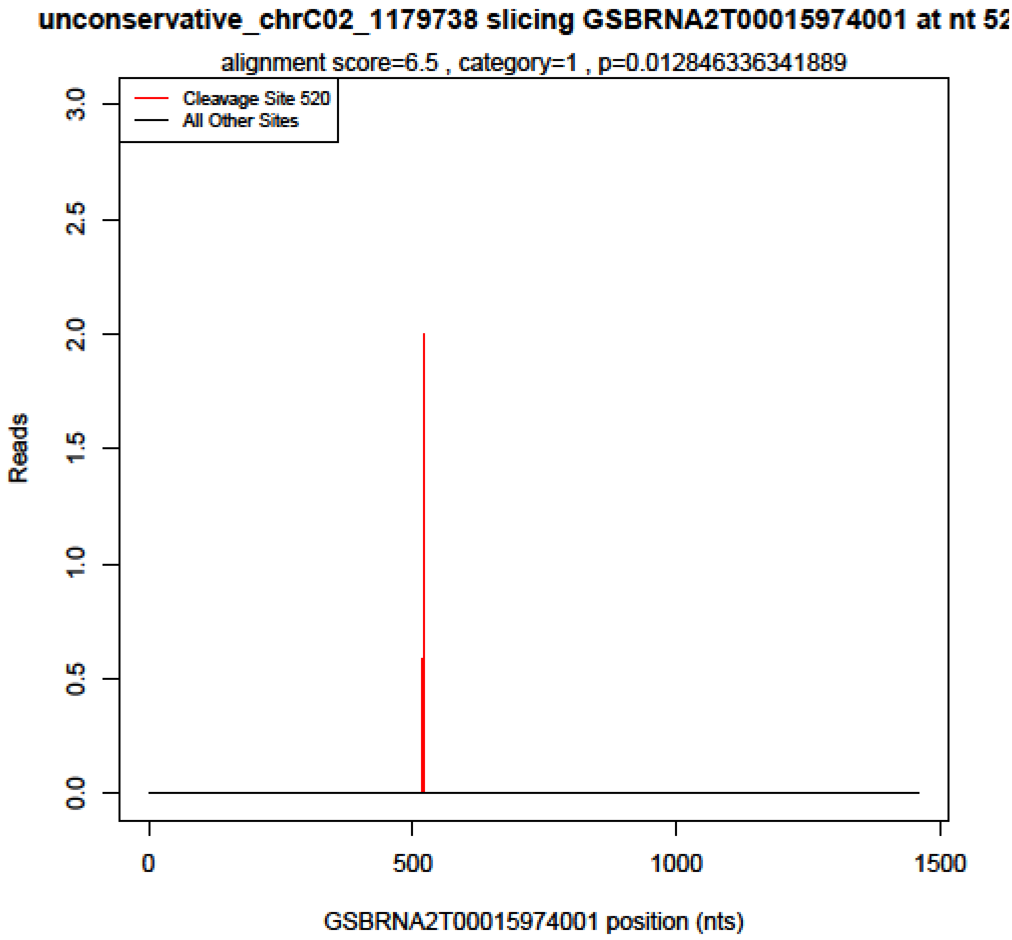

unconservative_chrC02_1179738 slicing GSBRNA2T00015974001 at nt 52
alignment score=6.5 , category=1 , p=0.012846336341889
Cleavage Site 520
All Other Sites
Reads
0.0
0.5
1.0
1.5
2.0
2.5
3.0
0
500
1000
1500
GSBRNA2T00015974001 position (nts)

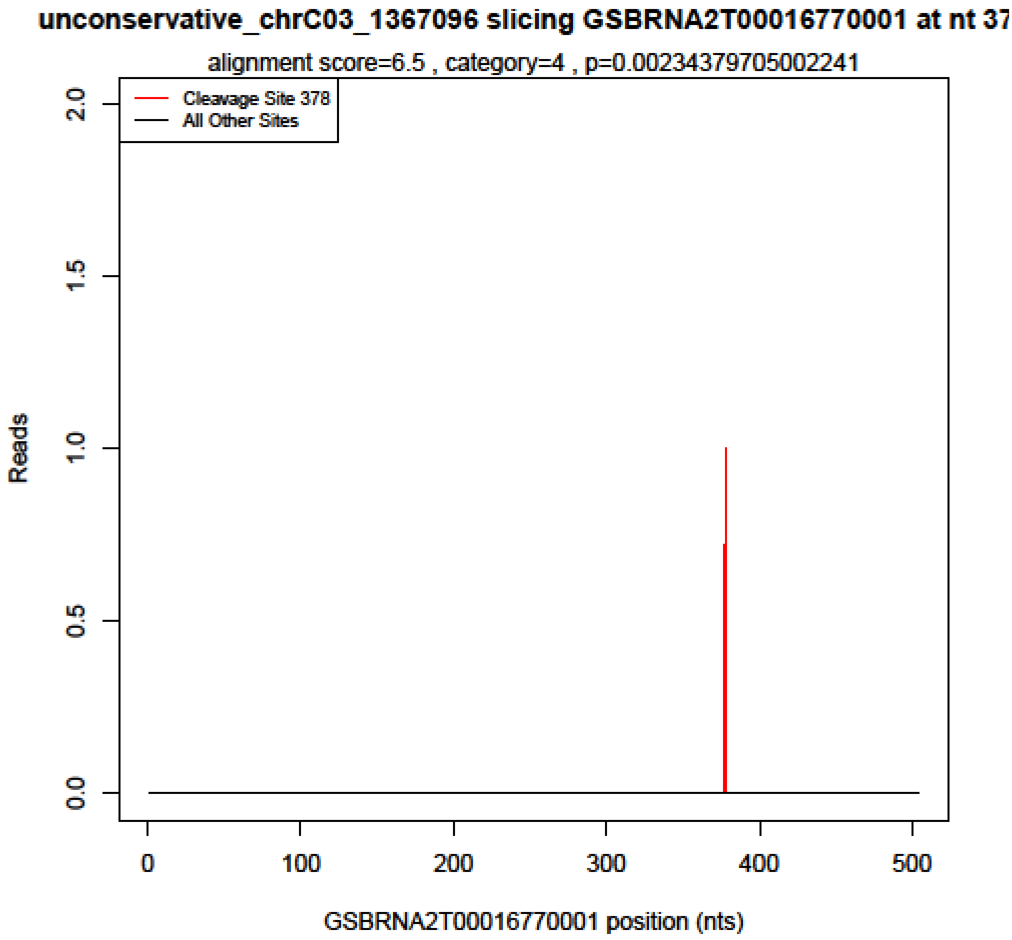

unconservative_chrC03_1367096 slicing GSBRNA2T00016770001 at nt 37
alignment score=6.5 , category=4 , p=0.00234379705002241
Cleavage Site 378
All Other Sites
Reads
0.0
0.5
1.0
1.5
2.0
0
100
200
300
400
500
GSBRNA2T00016770001 position (nts)

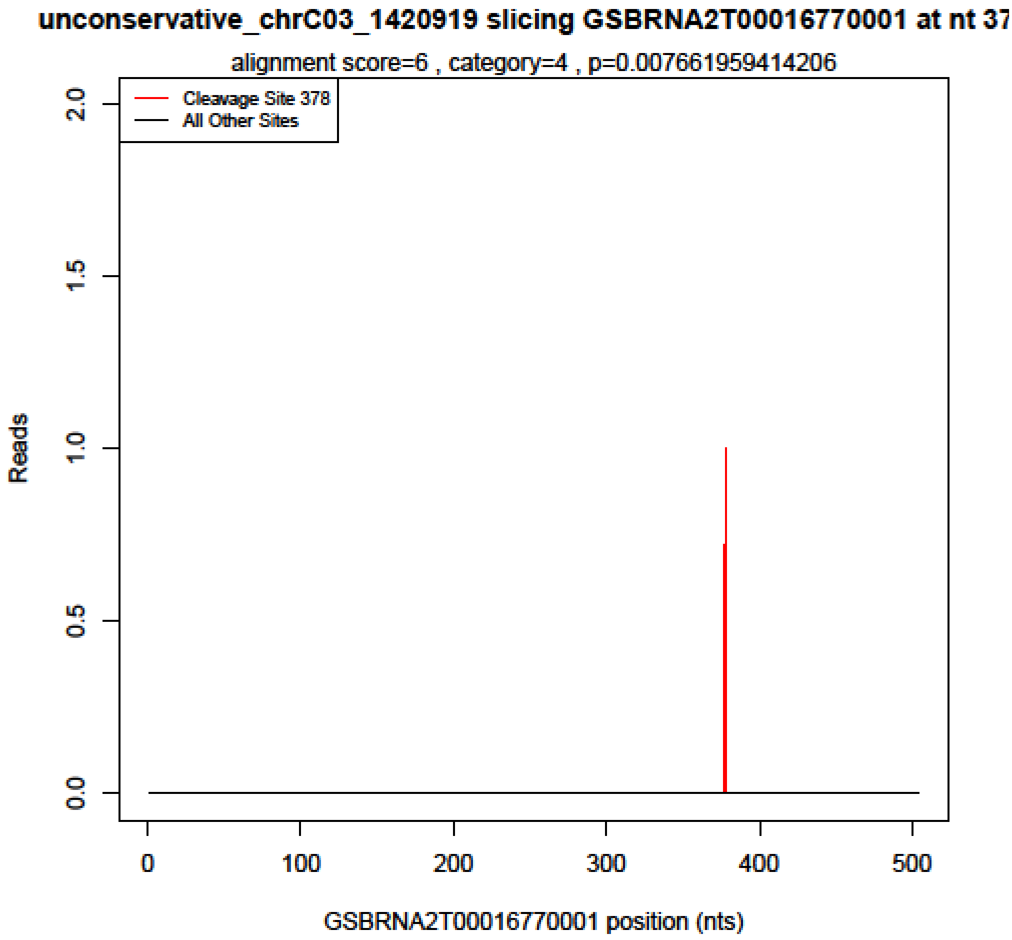
unconservative_chrC03_1420919 slicing GSBRNA2T00016770001 at nt 37
alignment score=6 , category=4 , p=0.007661959414206
Cleavage Site 378
All Other Sites
Reads
0.0
0.5
1.0
1.5
2.0
0
100
200
300
400
500
GSBRNA2T00016770001 position (nts)

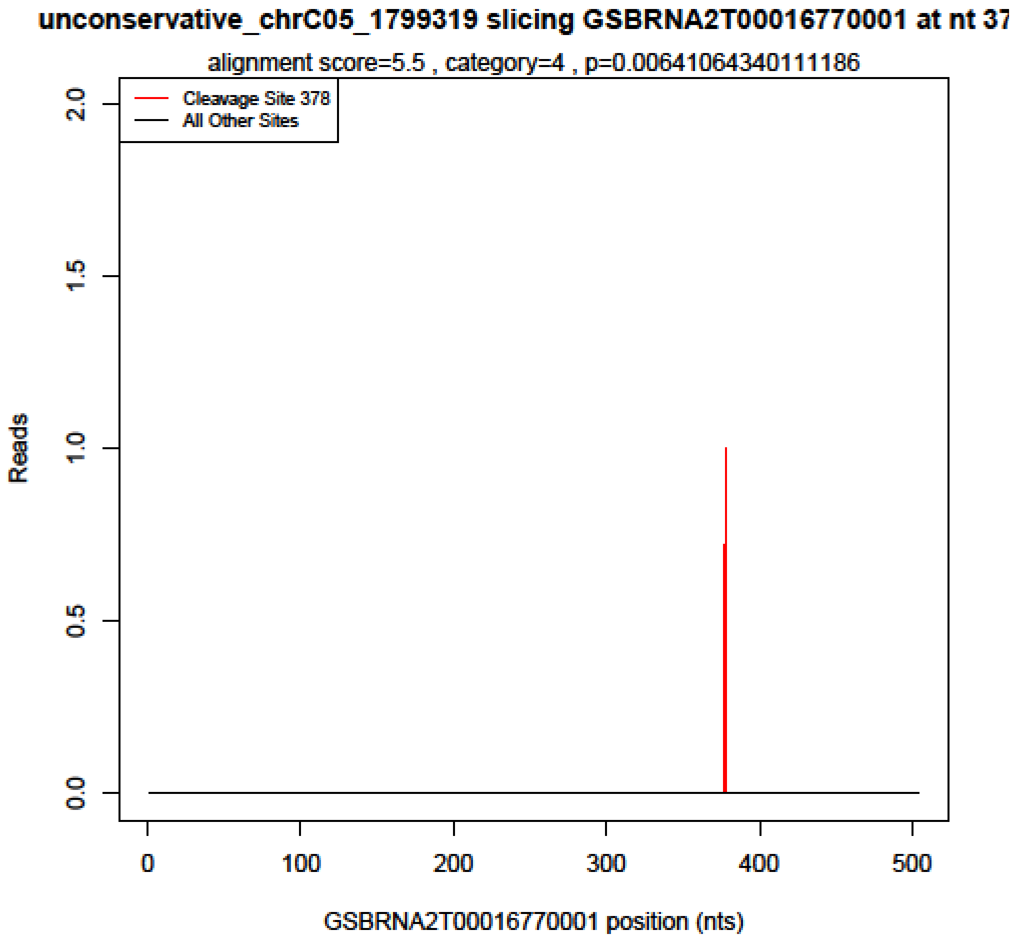

unconservative_chrC05_1799319 slicing GSBRNA2T00016770001 at nt 37
alignment score=5.5 , category=4 , p=0.00641064340111186
Cleavage Site 378
All Other Sites
Reads
0.0
0.5
1.0
1.5
2.0
0
100
200
300
400
500
GSBRNA2T00016770001 position (nts)

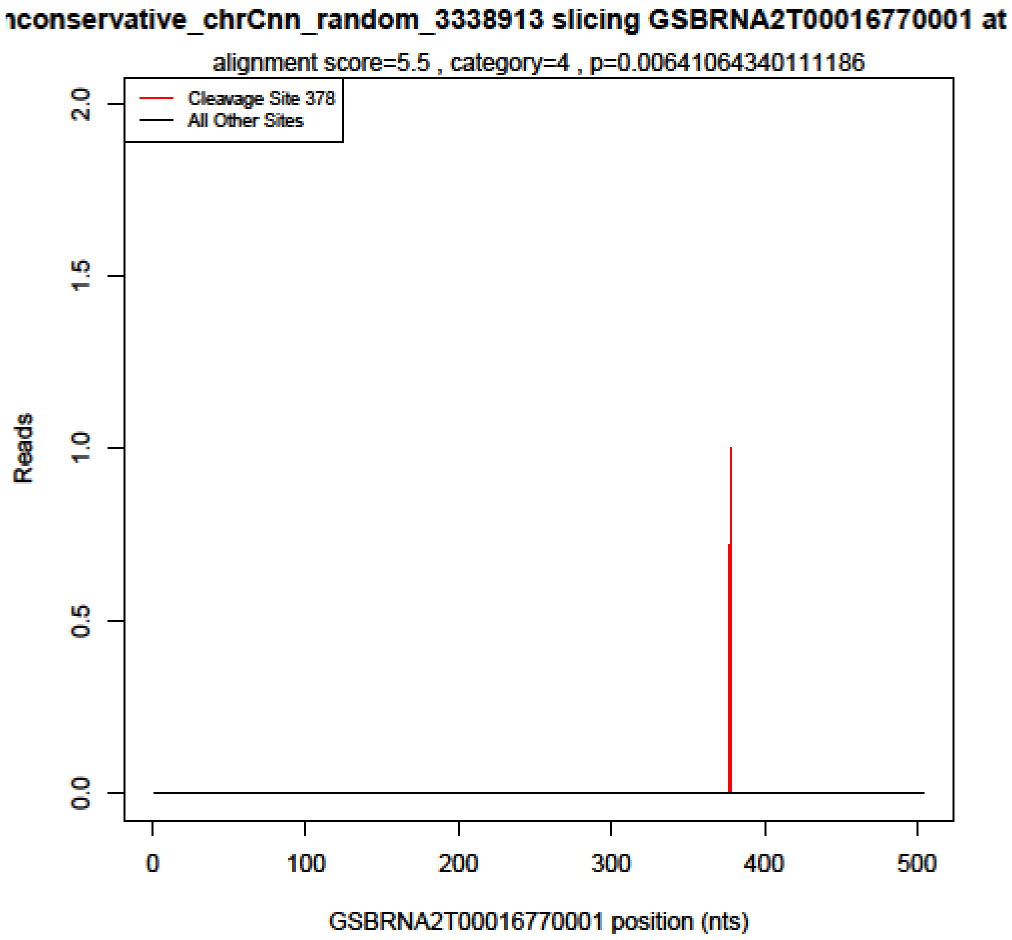

nconservative_chrCnn_random_3338913 slicing GSBRNA2T00016770001 at
alignment score=5.5 , category=4 , p=0.00641064340111186
Cleavage Site 378
All Other Sites
Reads
0.0
0.5
1.0
1.5
2.0
0
100
200
300
400
500
GSBRNA2T00016770001 position (nts)

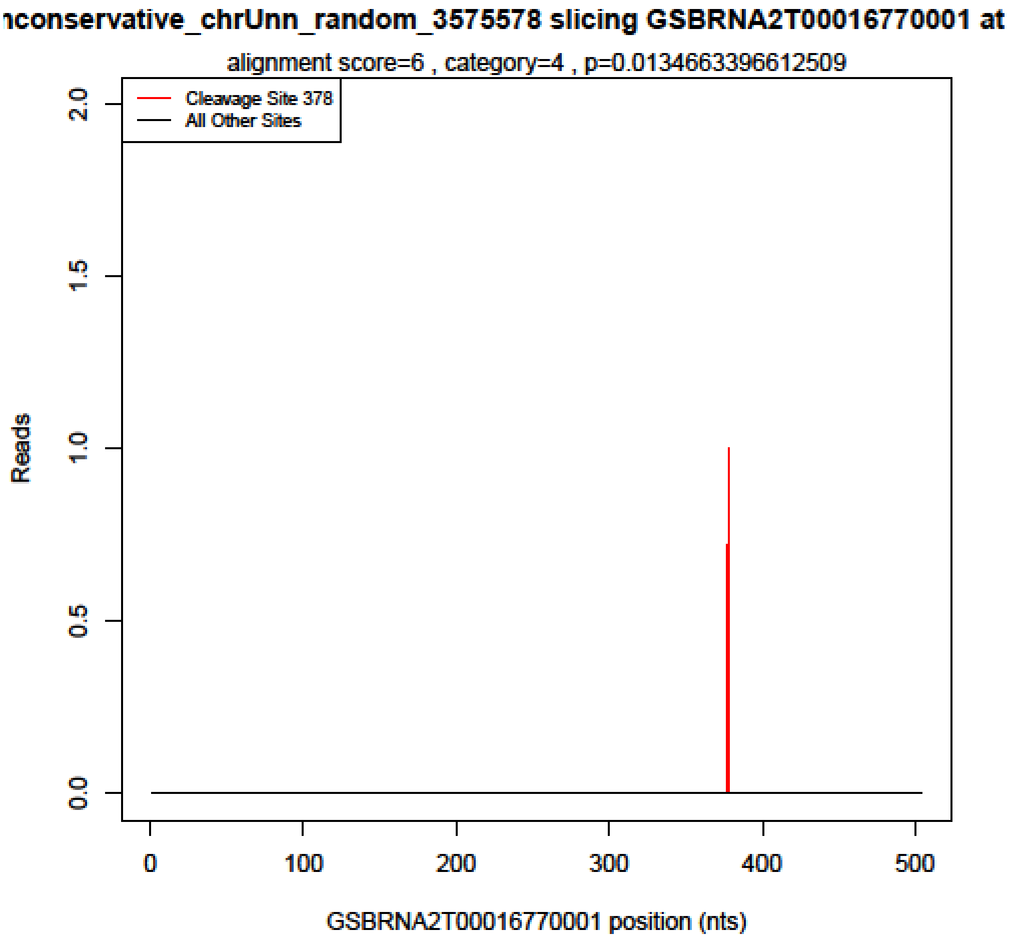

nconservative_chrUnn_random_3575578 slicing GSBRNA2T00016770001 at
alignment score=6 , category=4 , p=0.0134663396612509
Cleavage Site 378
All Other Sites
Reads
0.0
0.5
1.0
1.5
2.0
0
100
200
300
400
500
GSBRNA2T00016770001 position (nts)

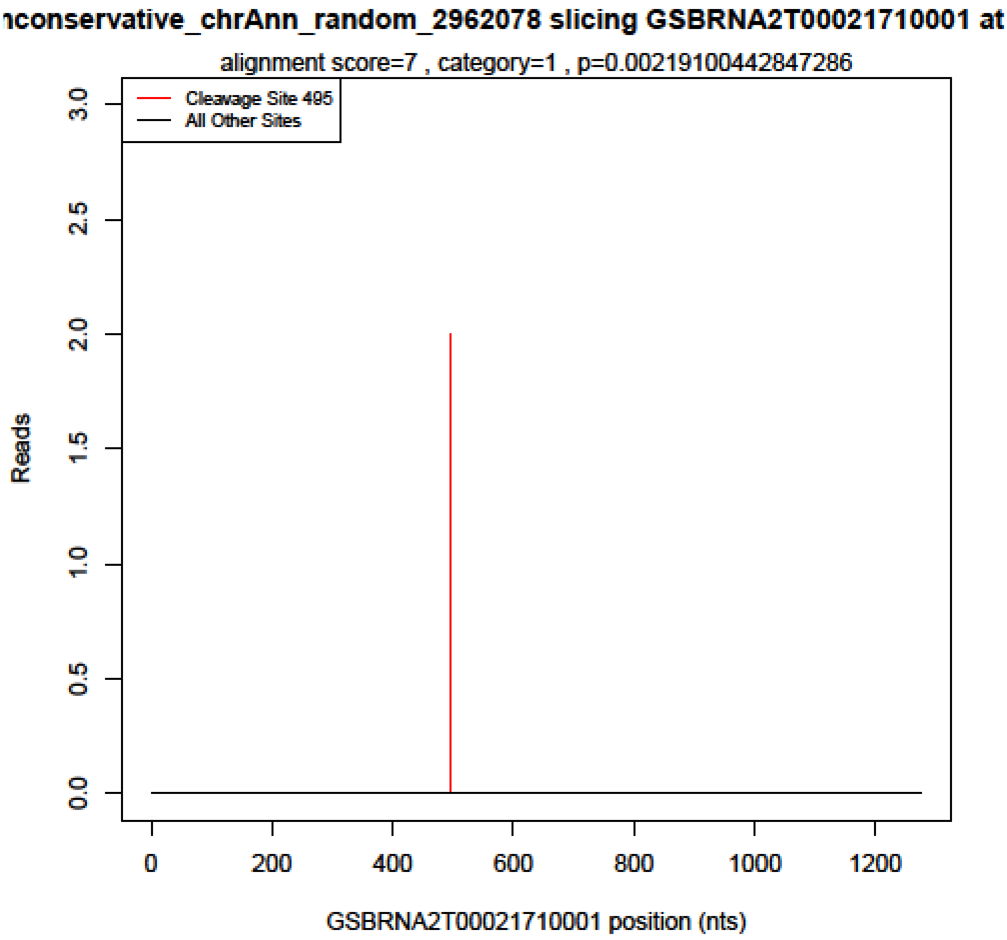
nconservative_chrAnn_random_2962078 slicing GSBRNA2T00021710001 at
alignment score=7 , category=1 , p=0.00219100442847286
Cleavage Site 495
All Other Sites
Reads
0.0
0.5
1.0
1.5
2.0
2.5
3.0
0
200
400
600
800
1000
1200
GSBRNA2T00021710001 position (nts)

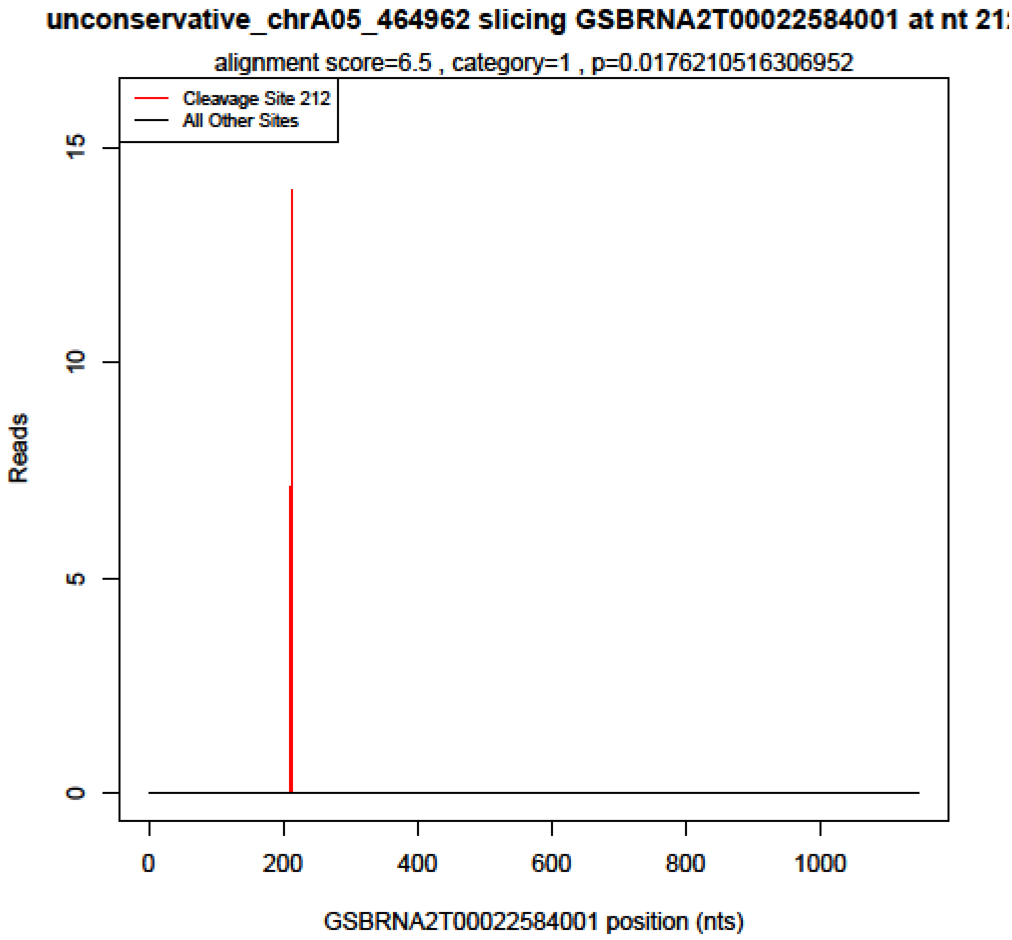

unconservative_chrA05_464962 slicing GSBRNA2T00022584001 at nt 21
alignment score=6.5 , category=1 , p=0.0176210516306952
Cleavage Site 212
All Other Sites
Reads
0
5
10
15
0
200
400
600
800
1000
GSBRNA2T00022584001 position (nts)

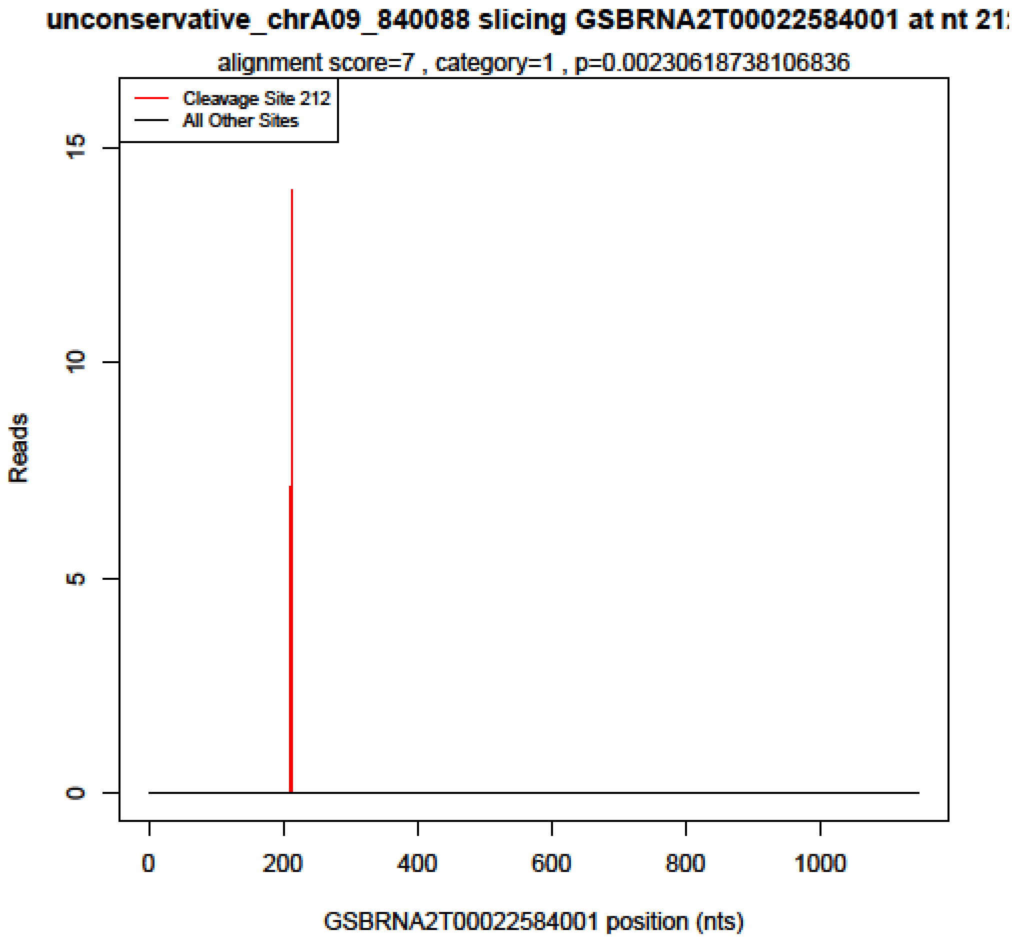

unconservative_chrA09_840088 slicing GSBRNA2T00022584001 at nt 21
alignment score=7 , category=1 , p=0.00230618738106836
Cleavage Site 212
All Other Sites
Reads
0
5
10
15
0
200
400
600
800
1000
GSBRNA2T00022584001 position (nts)

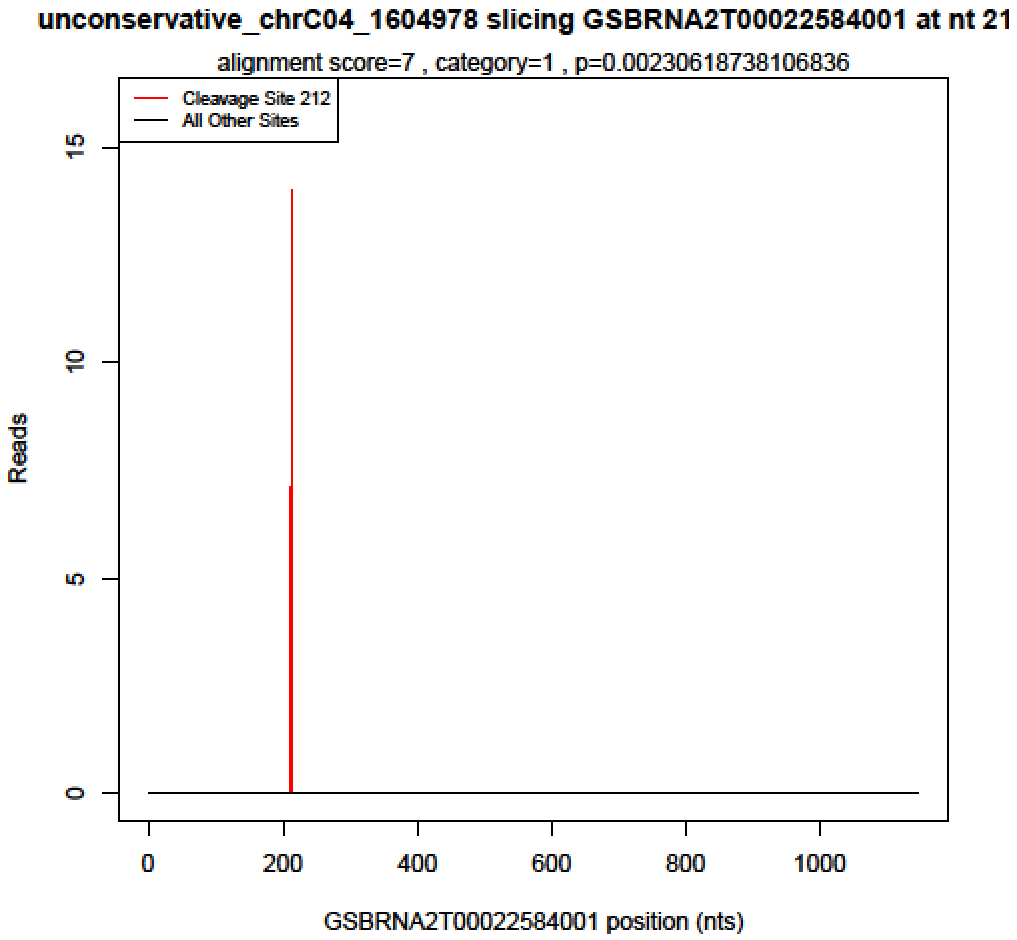

unconservative_chrC04_1604978 slicing GSBRNA2T00022584001 at nt 21
alignment score=7 , category=1 , p=0.00230618738106836
Cleavage Site 212
All Other Sites
Reads
0
5
10
15
0
200
400
600
800
1000
GSBRNA2T00022584001 position (nts)

unconservative_chrA06_547631 slicing GSBRNA2T00024561001 at nt 58

alignment score=4 , category=1 , p=0.0286724346814085

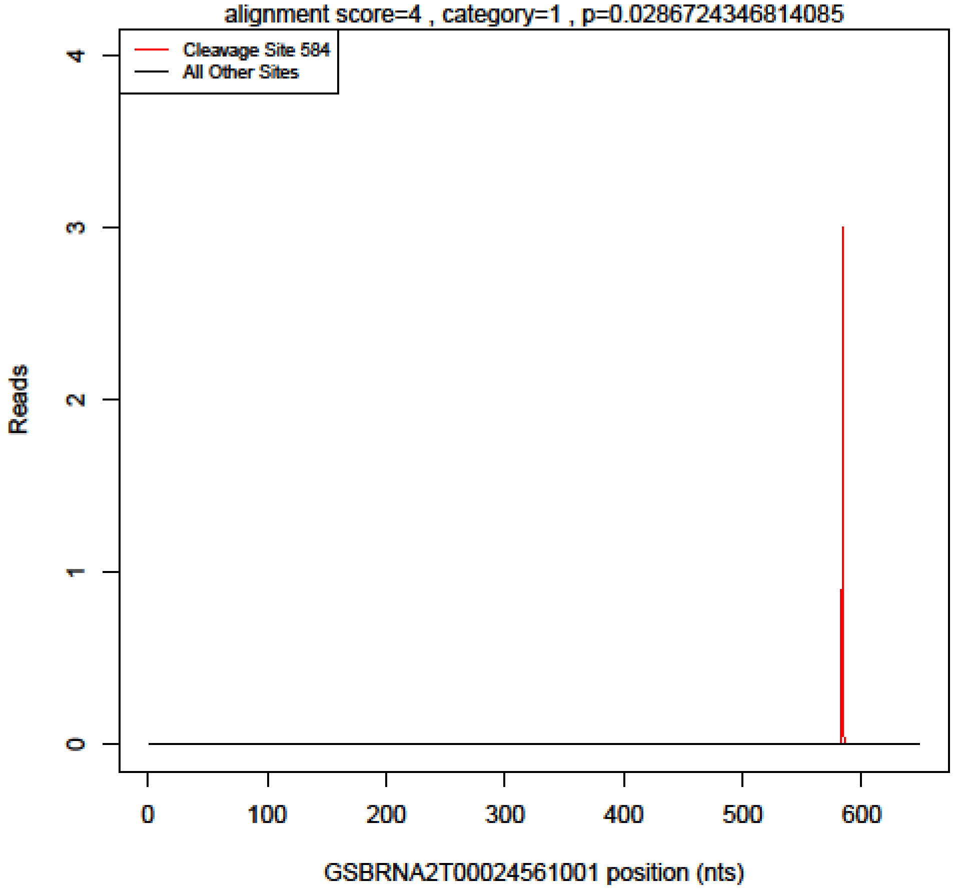

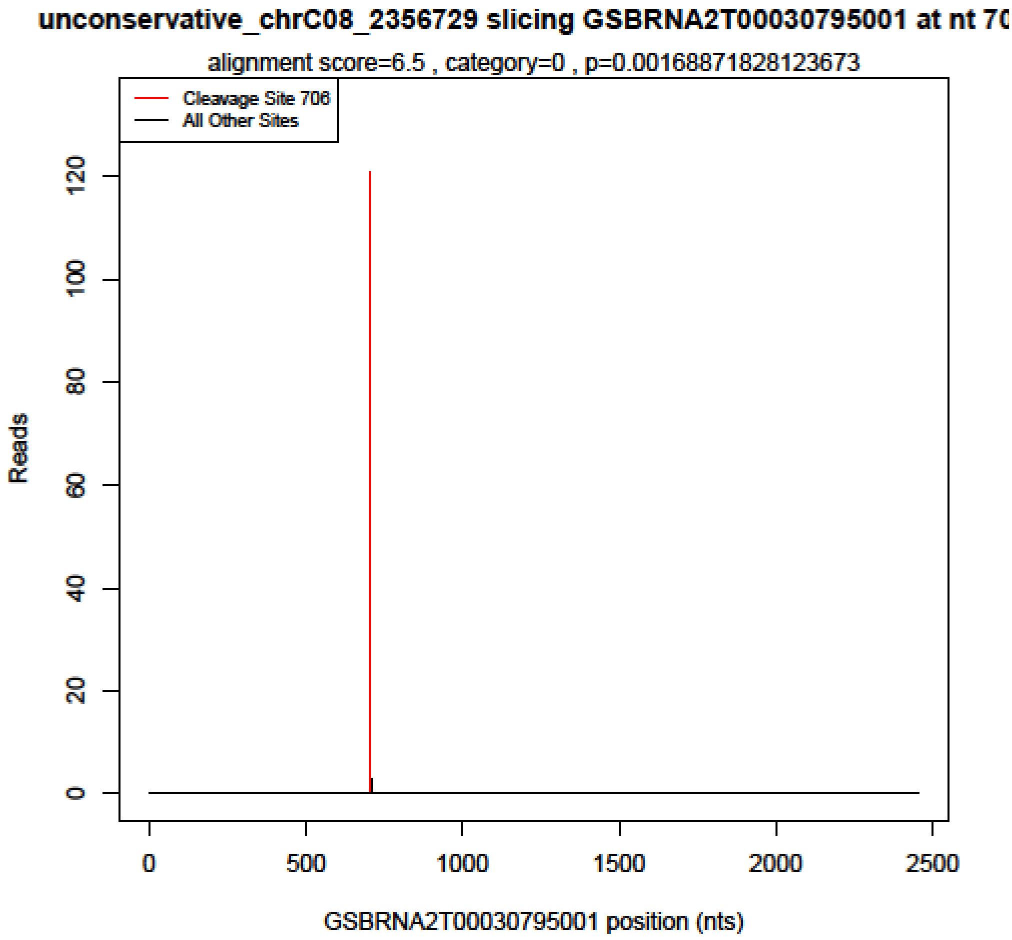

unconservative_chrC08_2356729 slicing GSBRNA2T00030795001 at nt 70
alignment score=6.5 , category=0 , p=0.00168871828123673
Cleavage Site 706
All Other Sites
Reads
0
20
40
60
80
100
120
0
500
1000
1500
2000
2500
GSBRNA2T00030795001 position (nts)

nconservative_chrCnn_random_3238853 slicing GSBRNA2T00024574001 at

alignment score=7 , category=1 , p=0.0497456572986954

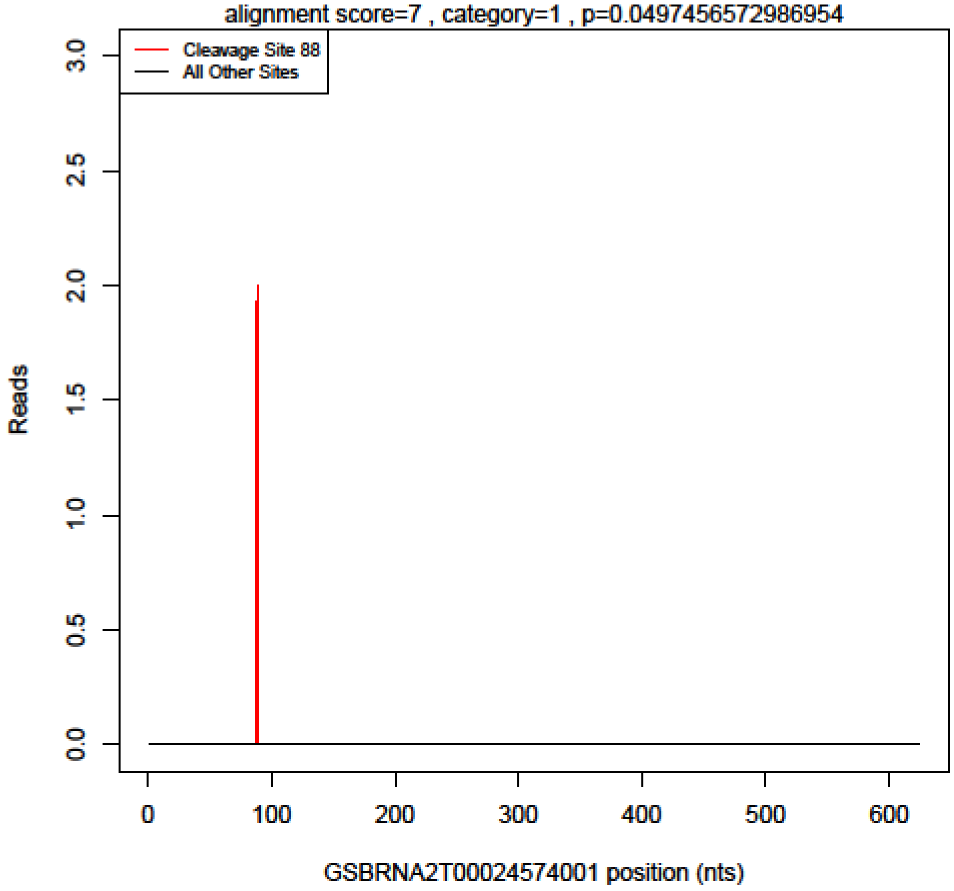

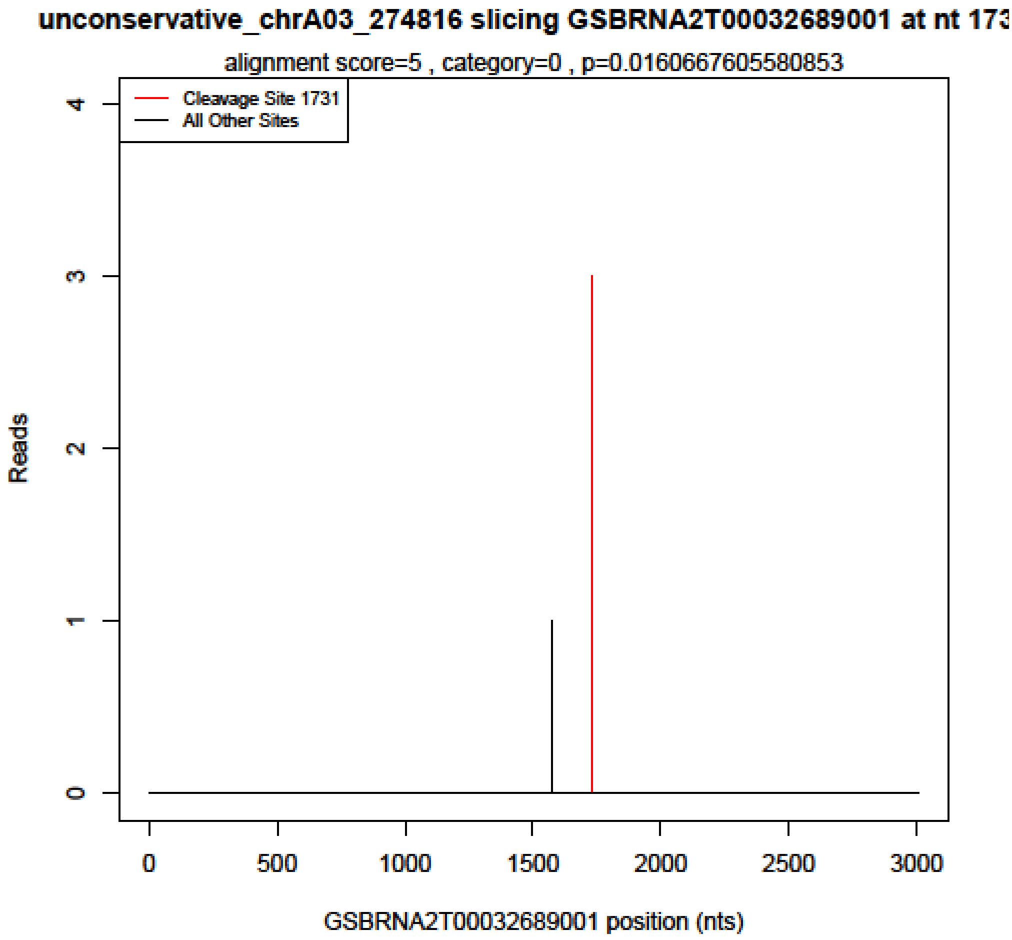
unconservative_chrA03_274816 slicing GSBRNA2T00032689001 at nt 173
alignment score=5 , category=0 , p=0.0160667605580853
Cleavage Site 1731
All Other Sites
Reads
0
1
2
3
4
0
500
1000
1500
2000
2500
3000
GSBRNA2T00032689001 position (nts)

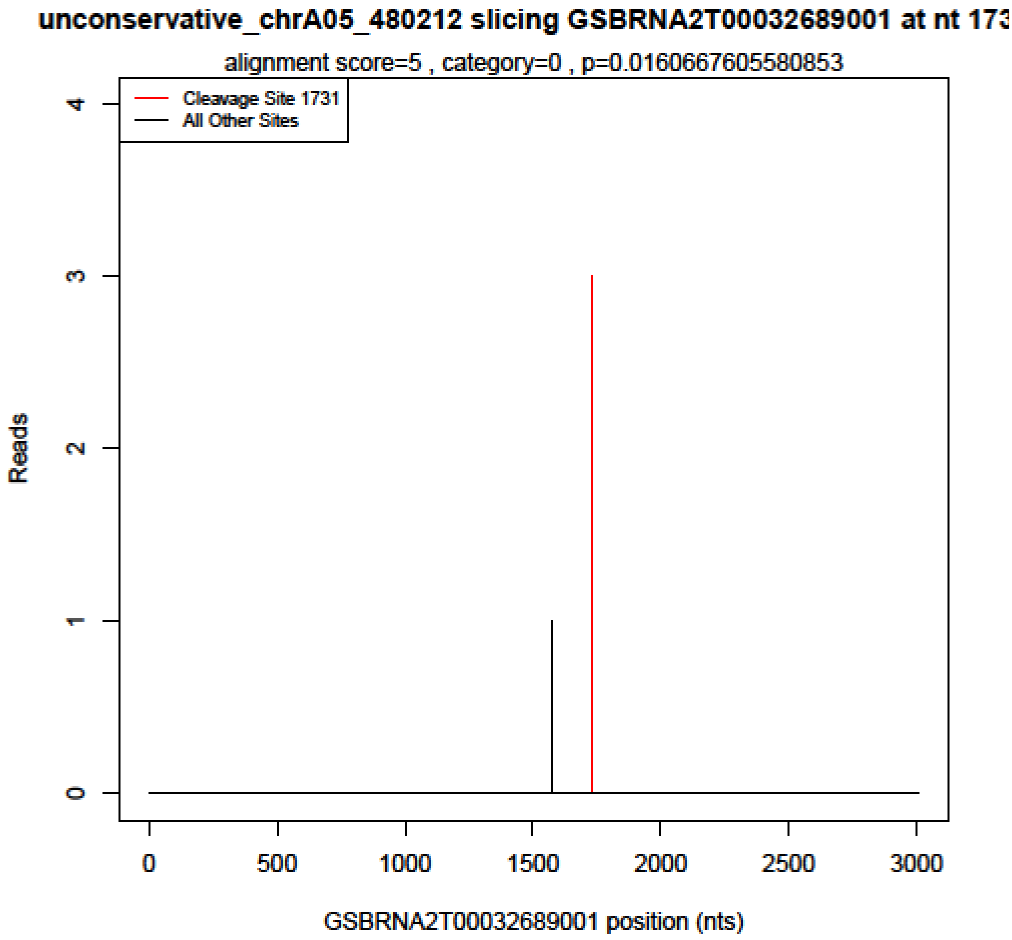

unconservative_chrA05_480212 slicing GSBRNA2T00032689001 at nt 173
alignment score=5 , category=0 , p=0.0160667605580853
Cleavage Site 1731
All Other Sites
Reads
0
1
2
3
4
0
500
1000
1500
2000
2500
3000
GSBRNA2T00032689001 position (nts)

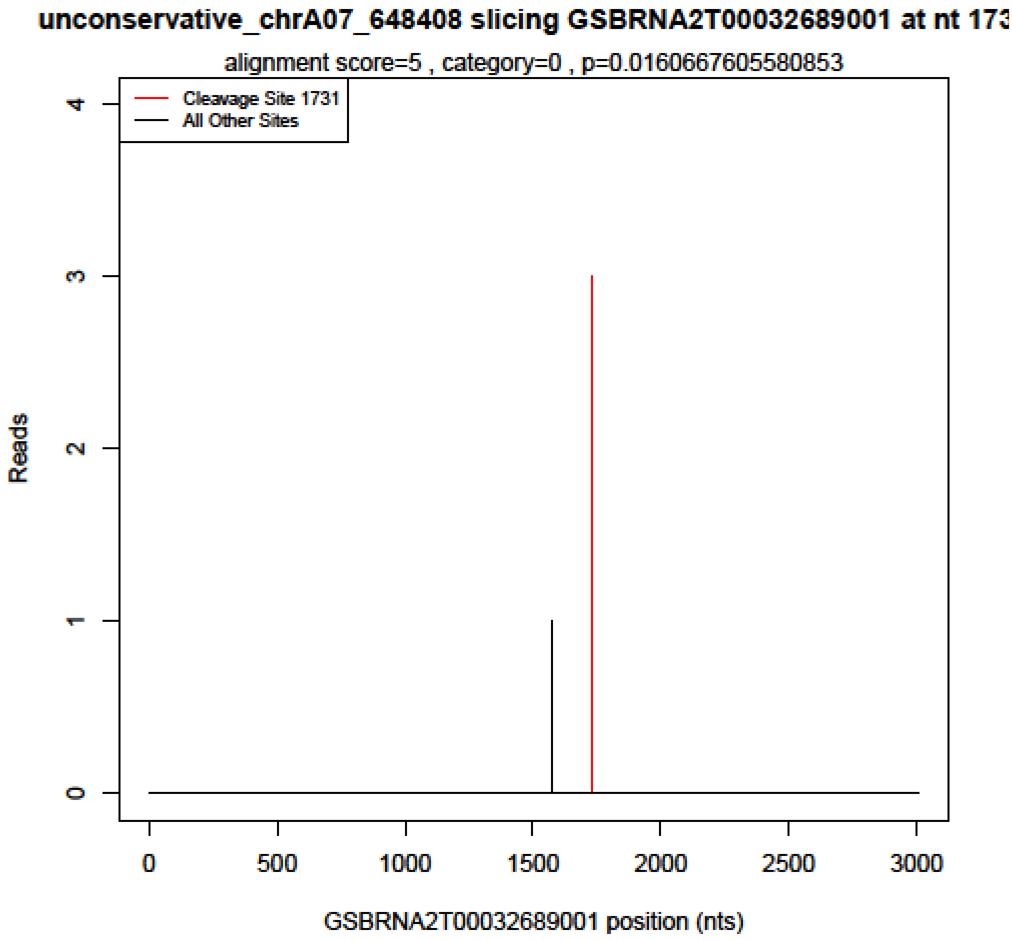

unconservative_chrA07_648408 slicing GSBRNA2T00032689001 at nt 173
alignment score=5 , category=0 , p=0.0160667605580853
Cleavage Site 1731
All Other Sites
Reads
0
1
2
3
4
0
500
1000
1500
2000
2500
3000
GSBRNA2T00032689001 position (nts)

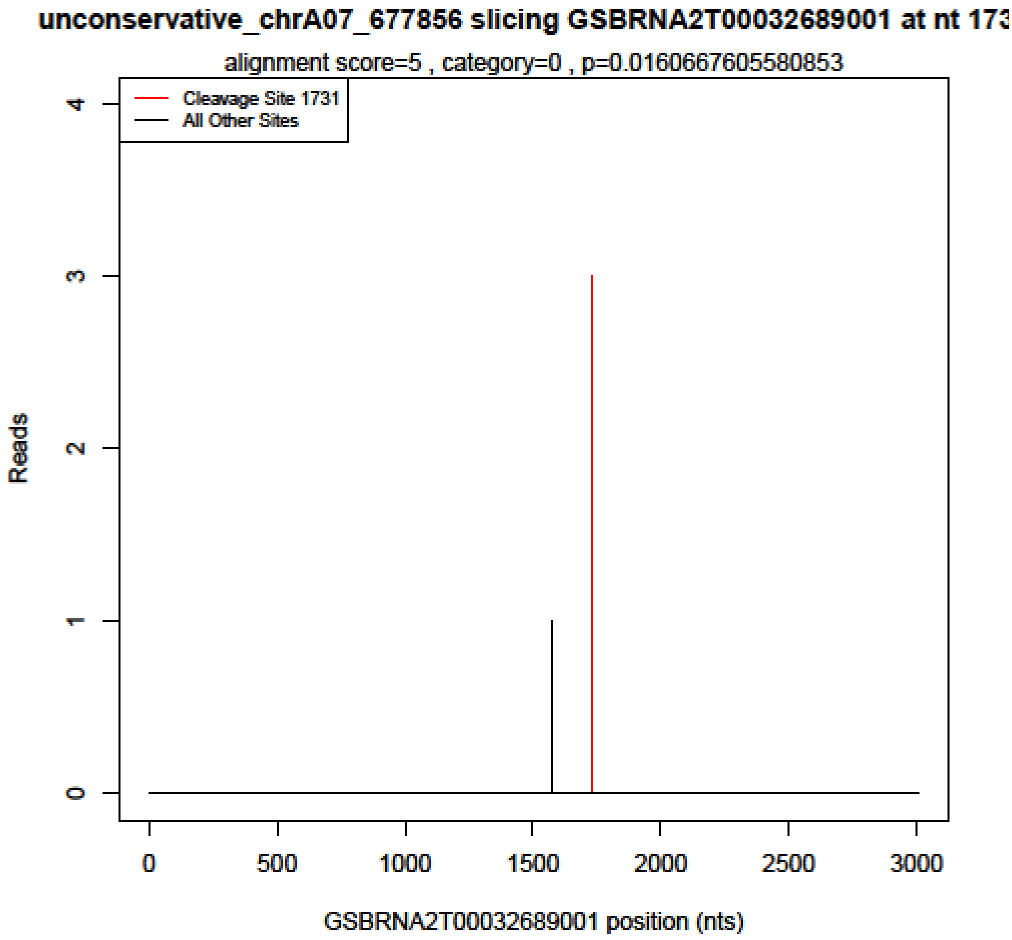

unconservative_chrA07_677856 slicing GSBRNA2T00032689001 at nt 173
alignment score=5 , category=0 , p=0.0160667605580853
Cleavage Site 1731
All Other Sites
Reads
0
1
2
3
4
0
500
1000
1500
2000
2500
3000
GSBRNA2T00032689001 position (nts)

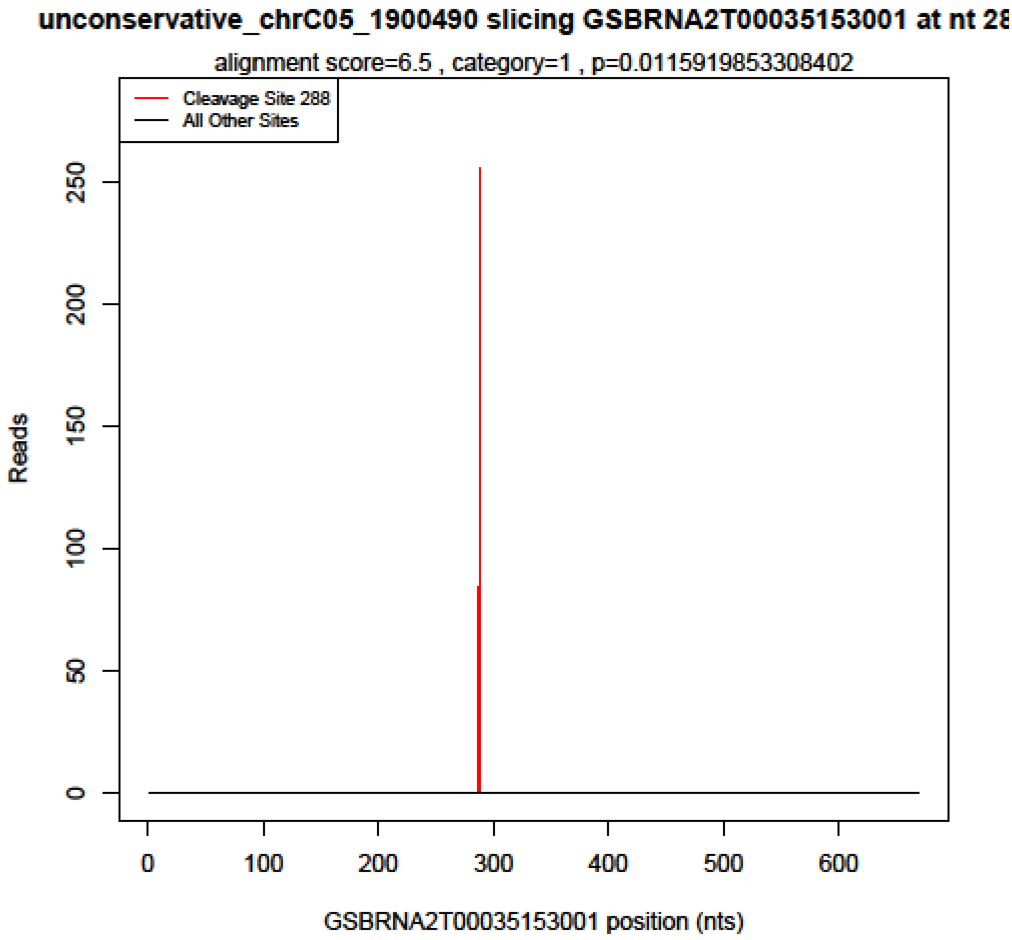

unconservative_chrC05_1900490 slicing GSBRNA2T00035153001 at nt 28
alignment score=6.5 , category=1 , p=0.0115919853308402
Cleavage Site 288
All Other Sites
Reads
0
50
100
150
200
250
0
100
200
300
400
500
600
GSBRNA2T00035153001 position (nts)

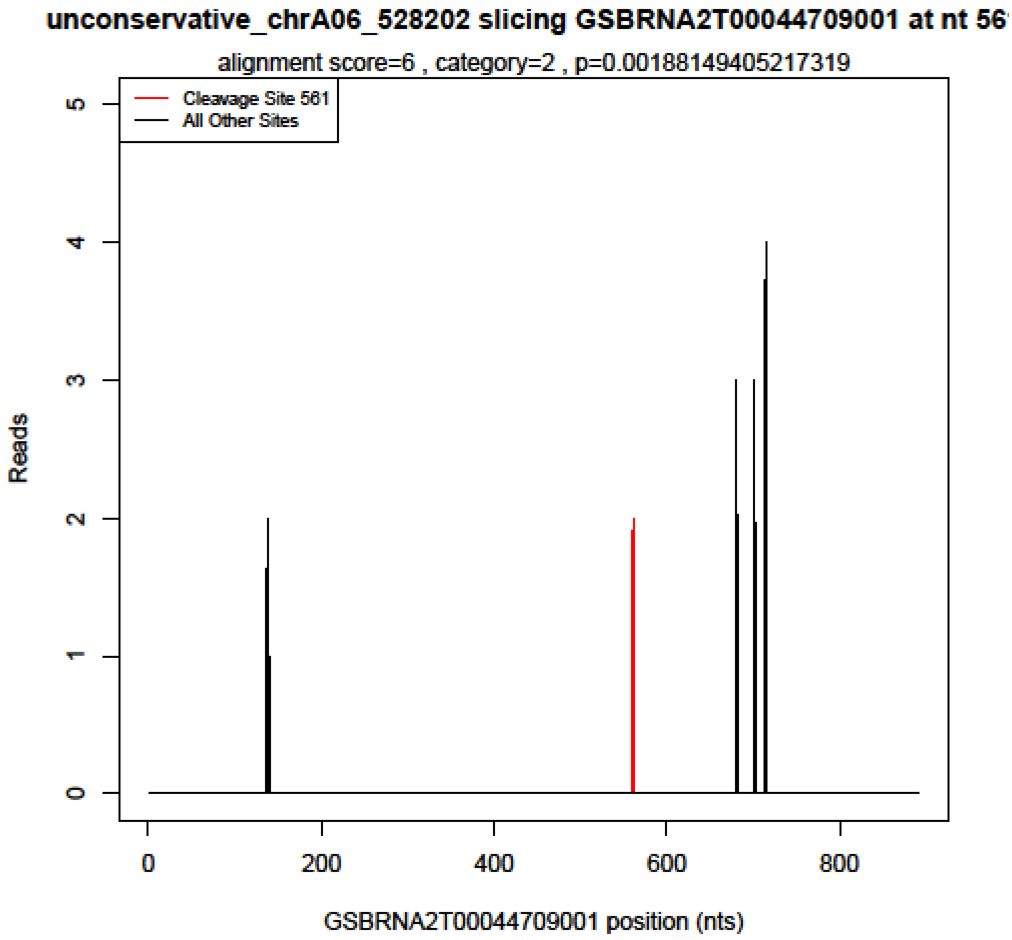

unconservative_chrA06_528202 slicing GSBRNA2T00044709001 at nt 56
alignment score=6 , category=2 , p=0.00188149405217319
Cleavage Site 561
All Other Sites
Reads
0
1
2
3
4
5
0
200
400
600
800
GSBRNA2T00044709001 position (nts)

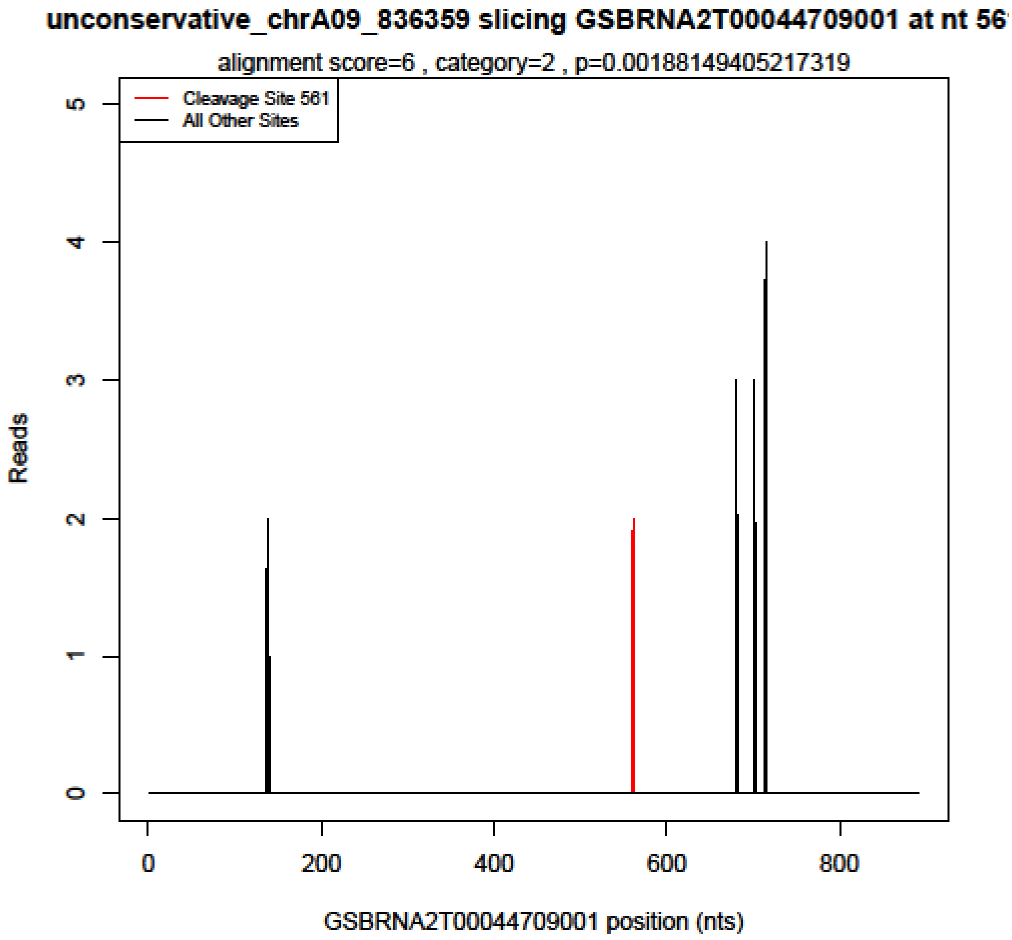
unconservative_chrA09_836359 slicing GSBRNA2T00044709001 at nt 56
alignment score=6 , category=2 , p=0.00188149405217319
Cleavage Site 561
All Other Sites
Reads
0
1
2
3
4
5
0
200
400
600
800
GSBRNA2T00044709001 position (nts)

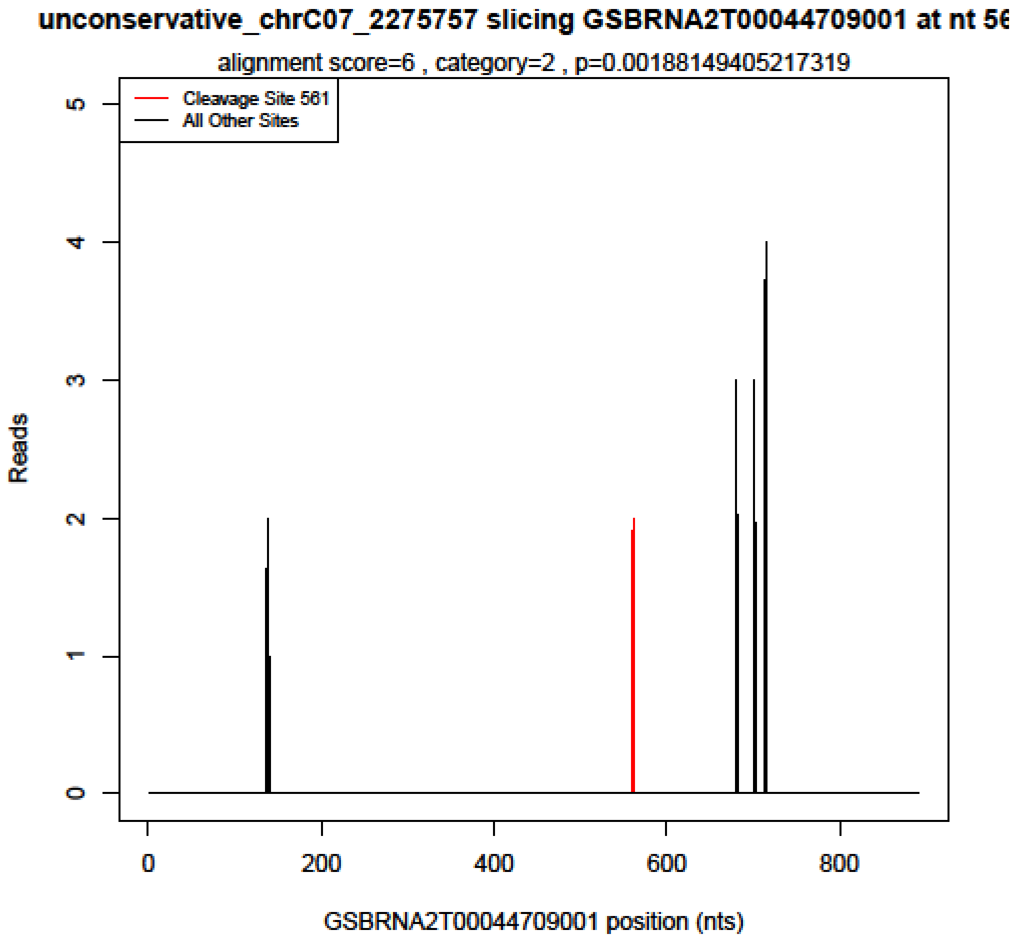
unconservative_chrC07_2275757 slicing GSBRNA2T00044709001 at nt 56
alignment score=6 , category=2 , p=0.00188149405217319
Cleavage Site 561
All Other Sites
Reads
0
1
2
3
4
5
0
200
400
600
800
GSBRNA2T00044709001 position (nts)

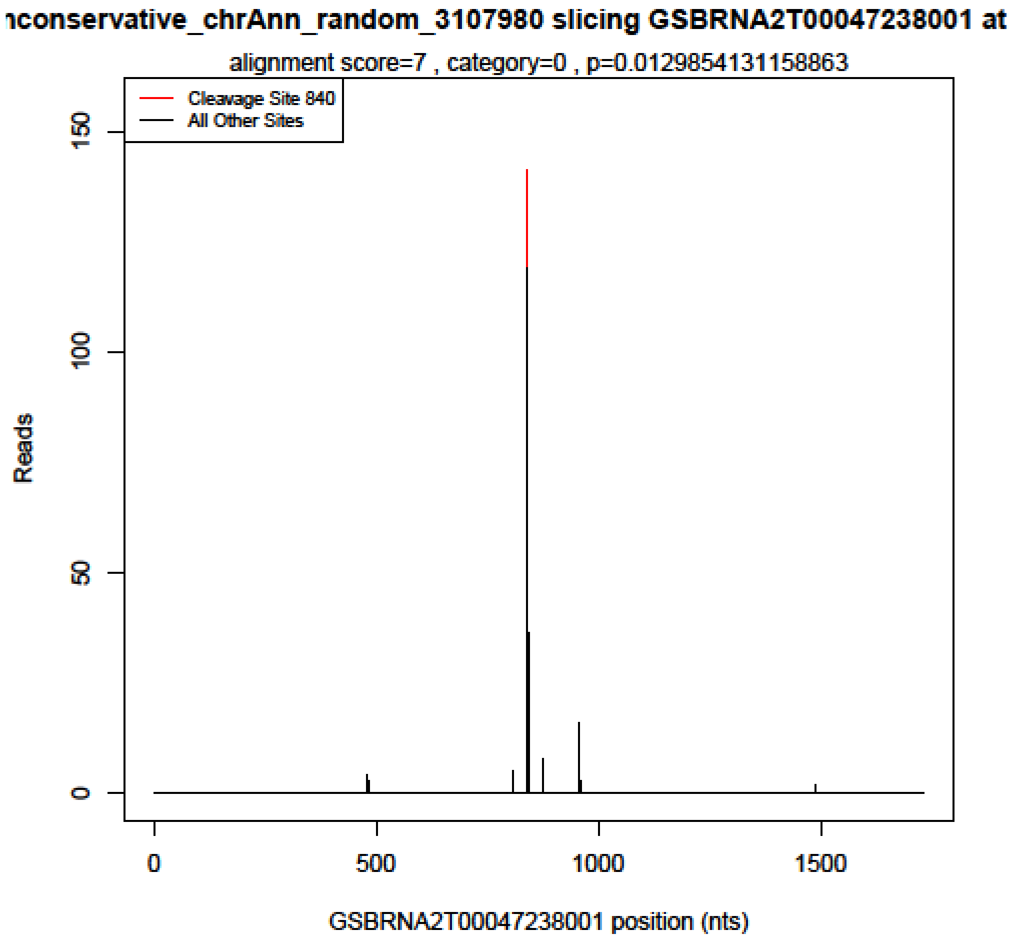

nconservative_chrAnn_random_3107980 slicing GSBRNA2T00047238001 at
alignment score=7 , category=0 , p=0.0129854131158863
Cleavage Site 840
All Other Sites
Reads
0
50
100
150
0
500
1000
1500
GSBRNA2T00047238001 position (nts)

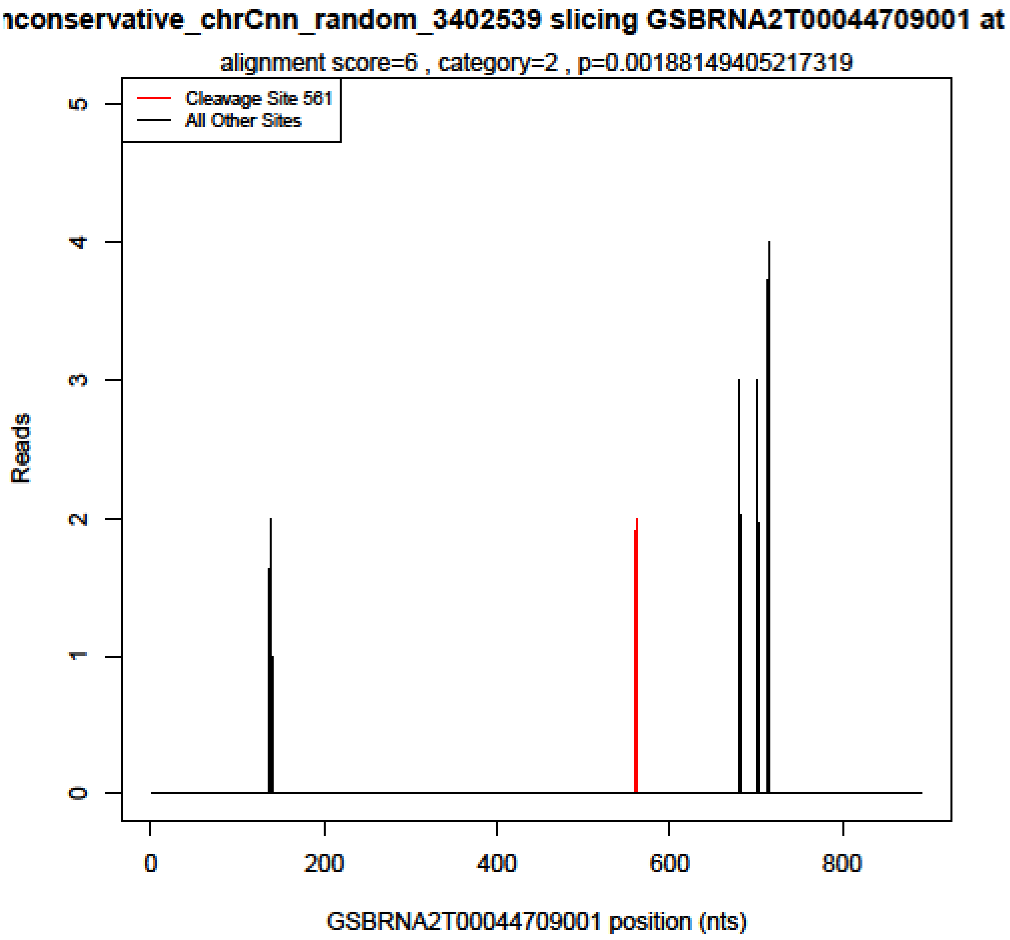

nconservative_chrCnn_random_3402539 slicing GSBRNA2T00044709001 at
alignment score=6 , category=2 , p=0.00188149405217319
Cleavage Site 561
All Other Sites
Reads
0
1
2
3
4
5
0
200
400
600
800
GSBRNA2T00044709001 position (nts)

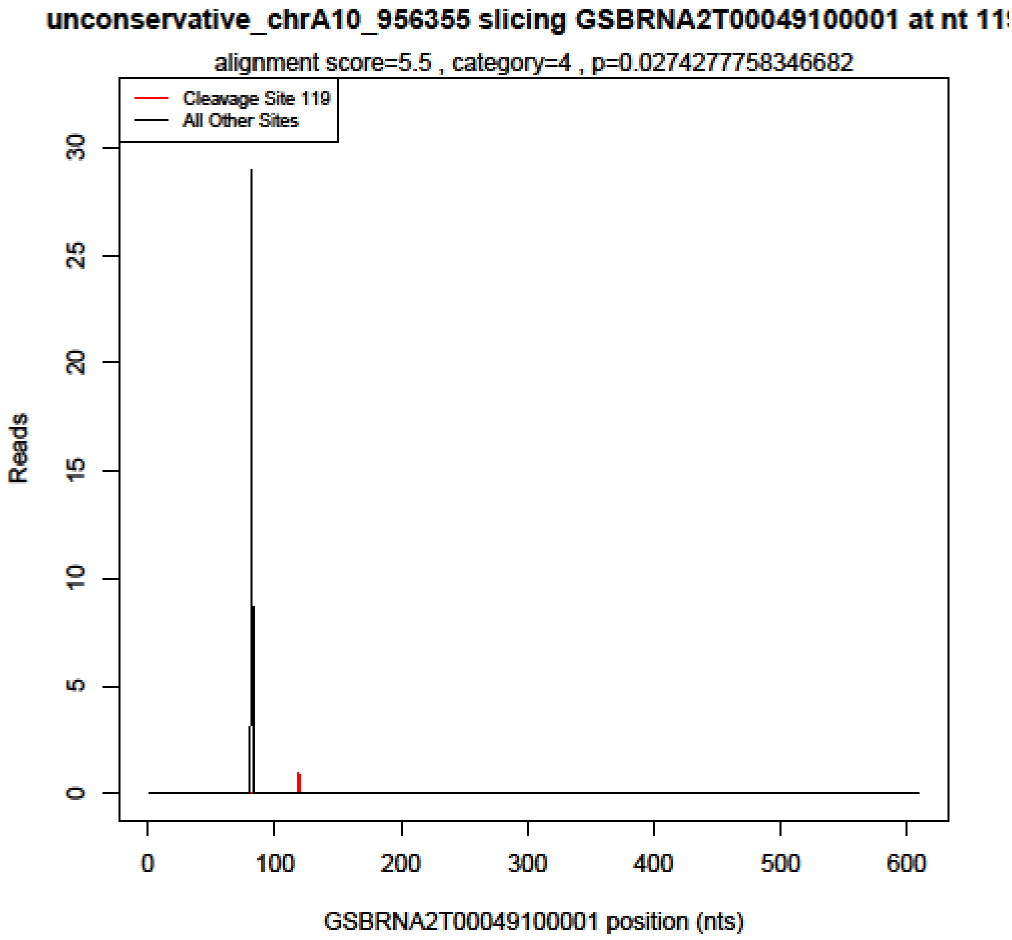

unconservative_chrA10_956355 slicing GSBRNA2T00049100001 at nt 11
alignment score=5.5 , category=4 , p=0.0274277758346682
Cleavage Site 119
All Other Sites
Reads
0
5
10
15
20
25
30
0
100
200
300
400
500
600
GSBRNA2T00049100001 position (nts)

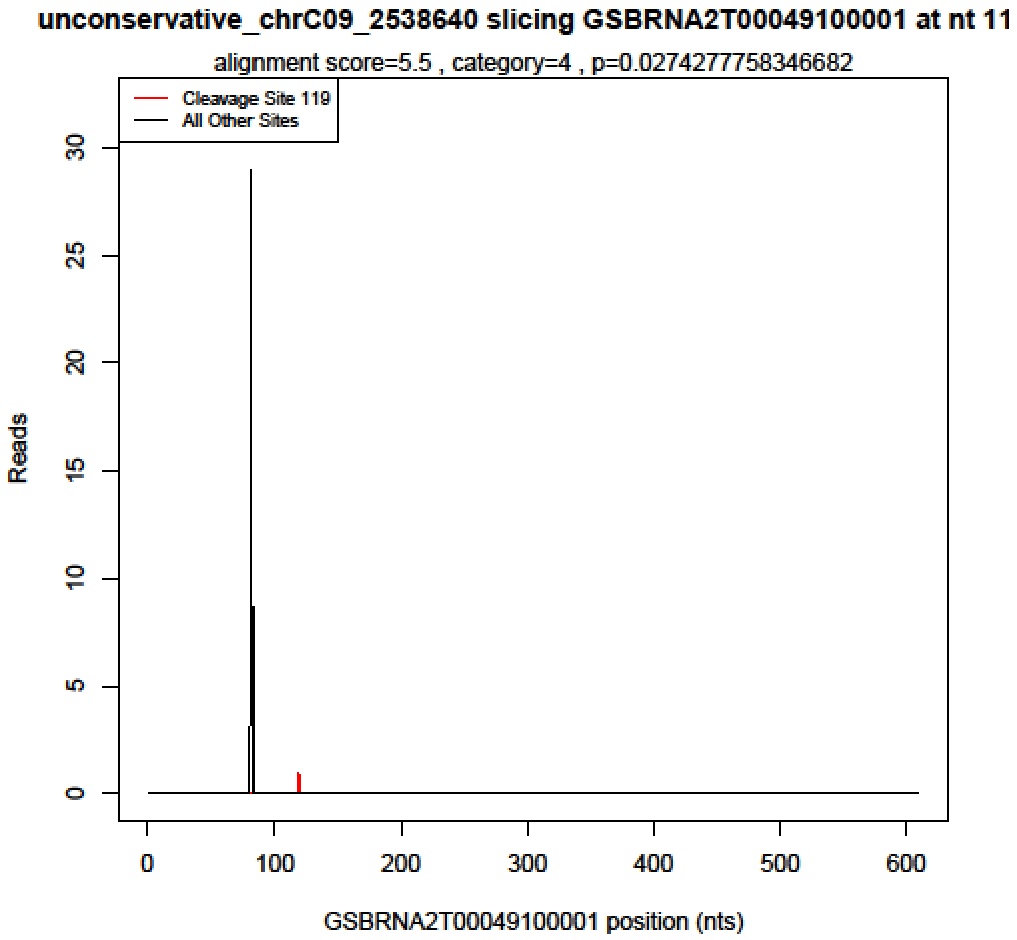

unconservative_chrC09_2538640 slicing GSBRNA2T00049100001 at nt 11
alignment score=5.5 , category=4 , p=0.0274277758346682
Cleavage Site 119
All Other Sites
Reads
0
5
10
15
20
25
30
0
100
200
300
400
500
600
GSBRNA2T00049100001 position (nts)

nconservative_chrAnn_random_3061750 slicing GSBRNA2T00049791001 at

alignment score=6.5 , category=0 , p=0.00584204054073001

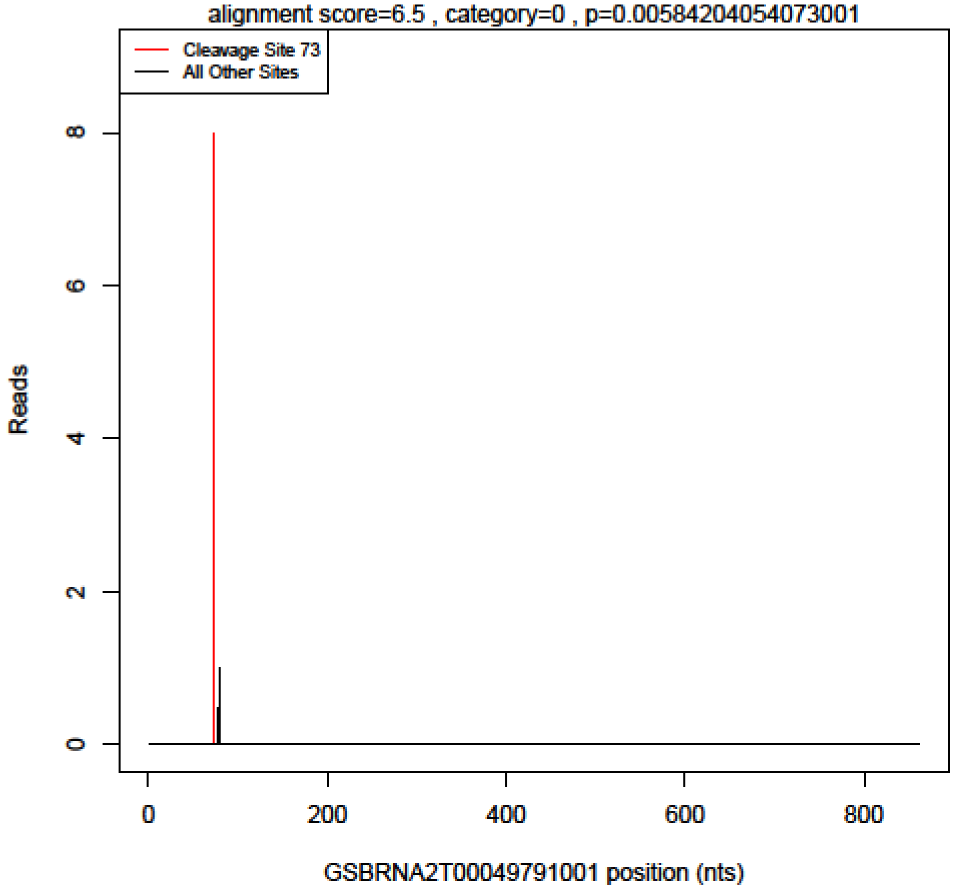

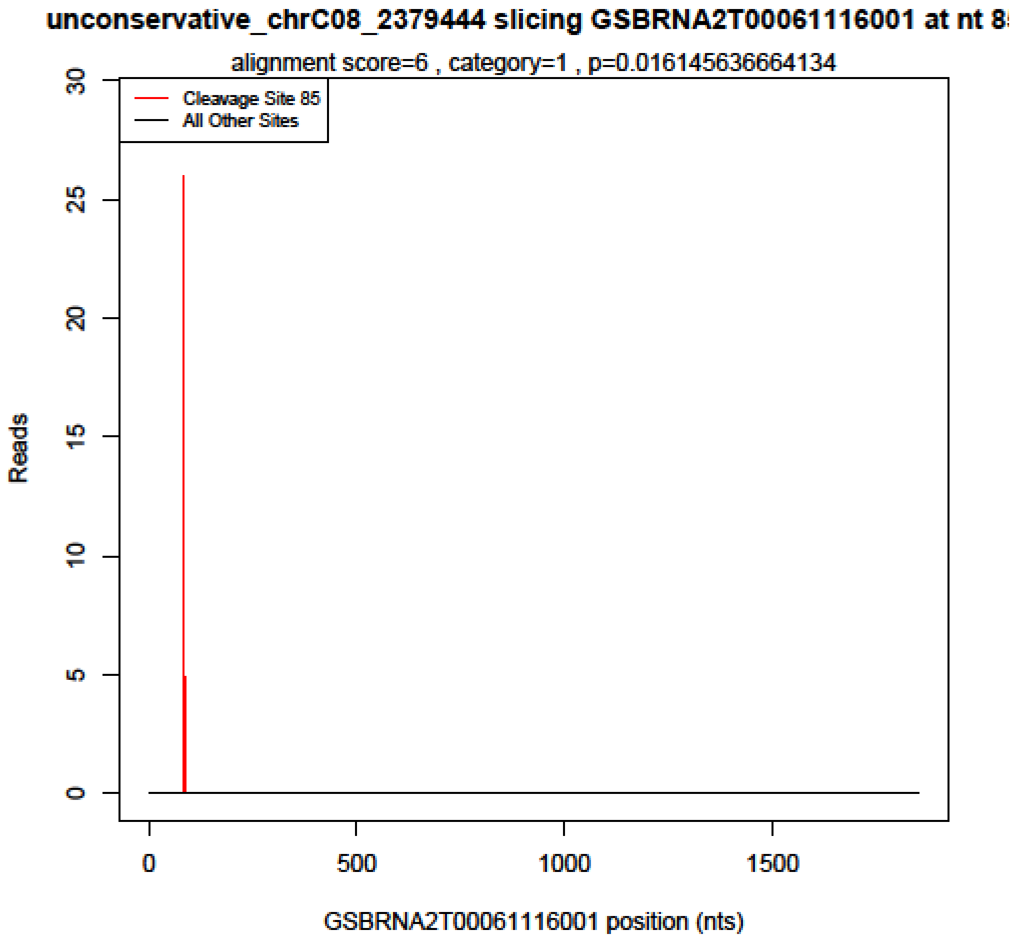

unconservative_chrC08_2379444 slicing GSBRNA2T00061116001 at nt 8
alignment score=6 , category=1 , p=0.016145636664134
Cleavage Site 85
All Other Sites
Reads
0
5
10
15
20
25
30
0
500
1000
1500
GSBRNA2T00061116001 position (nts)

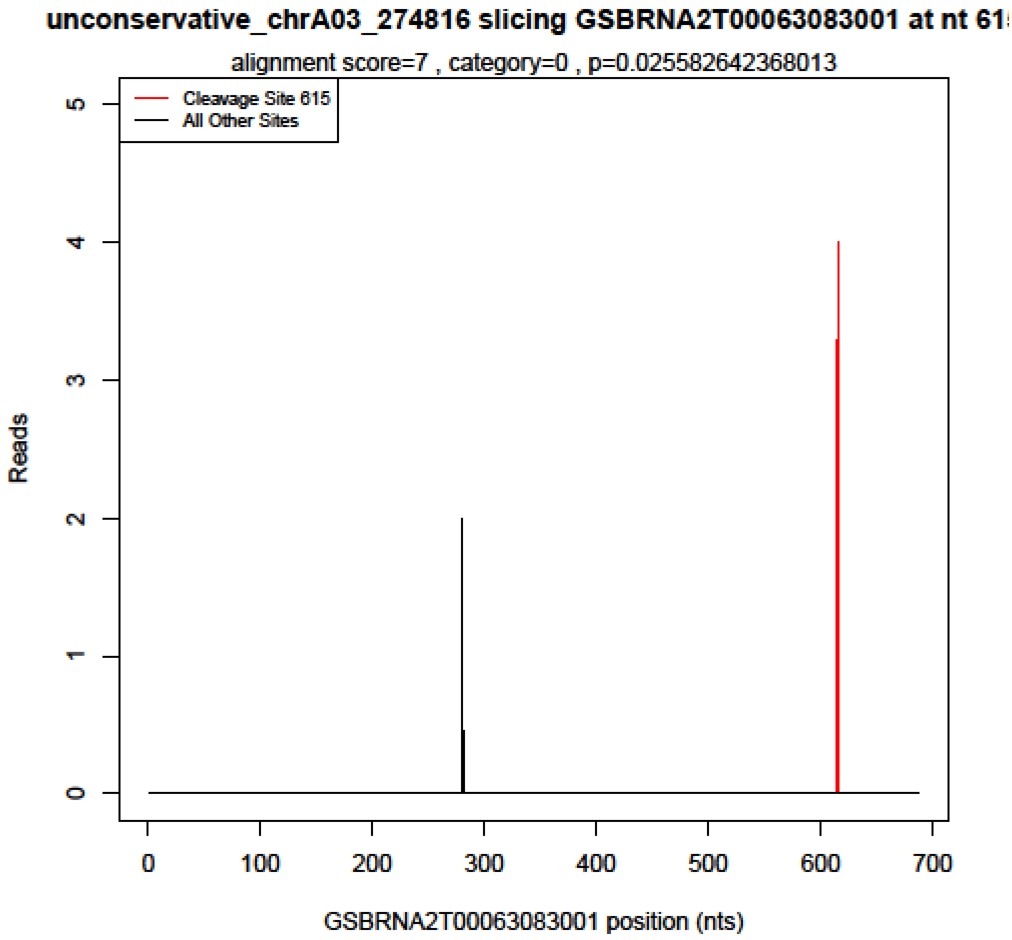

unconservative_chrA03_274816 slicing GSBRNA2T00063083001 at nt 61
alignment score=7 , category=0 , p=0.025582642368013
Cleavage Site 615
All Other Sites
Reads
0
1
2
3
4
5
0
100
200
300
400
500
600
700
GSBRNA2T00063083001 position (nts)

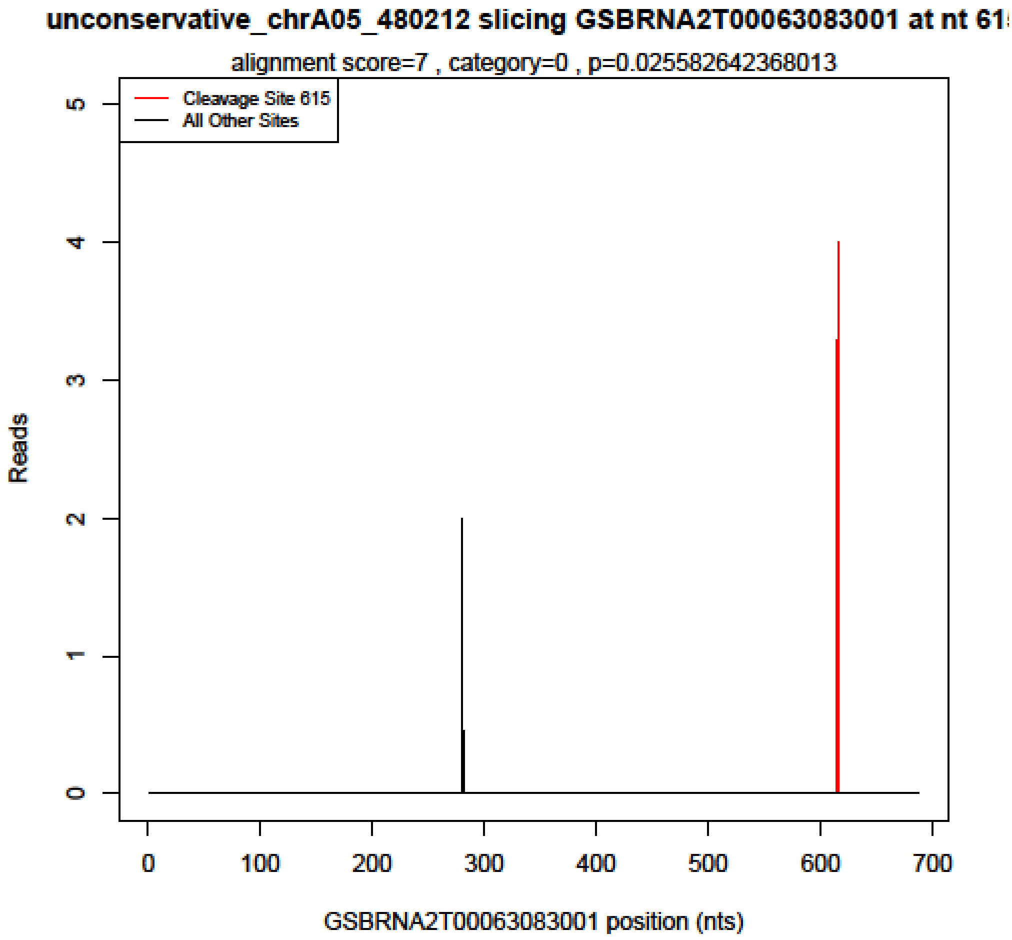

unconservative_chrA05_480212 slicing GSBRNA2T00063083001 at nt 61
alignment score=7 , category=0 , p=0.025582642368013
Cleavage Site 615
All Other Sites
Reads
0
1
2
3
4
5
0
100
200
300
400
500
600
700
GSBRNA2T00063083001 position (nts)

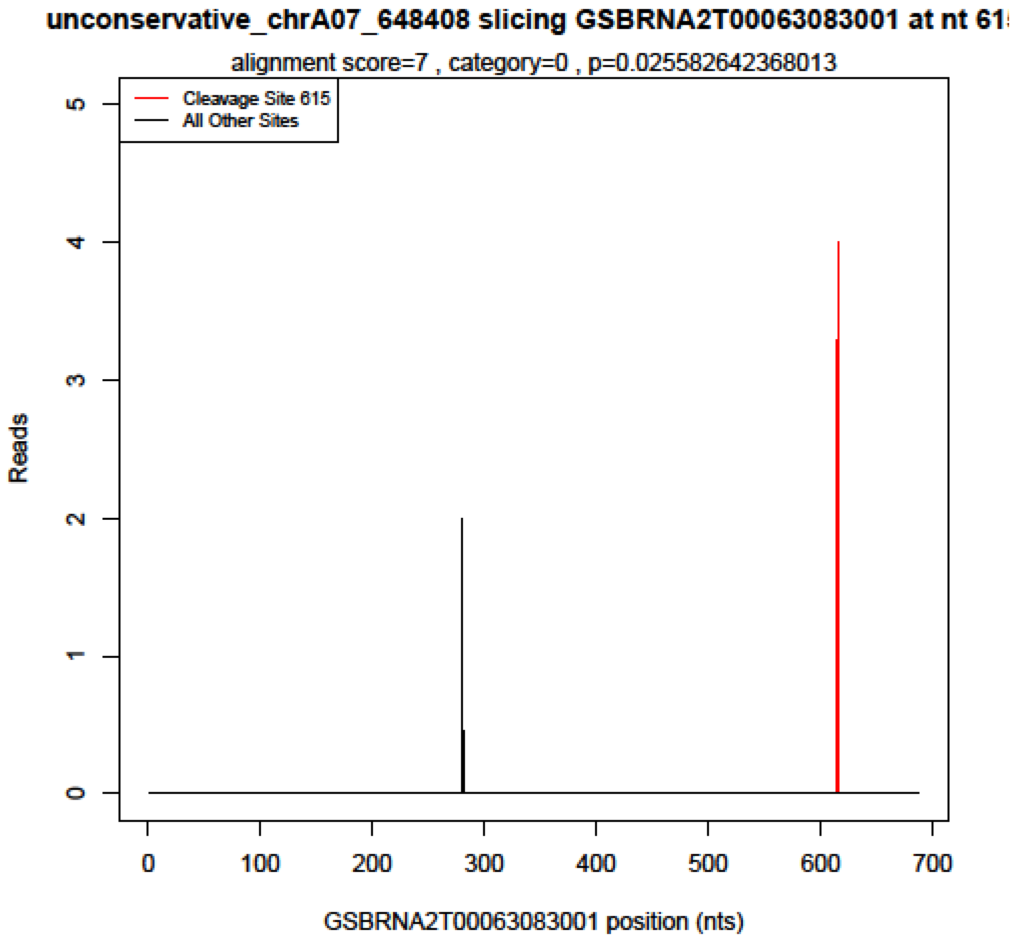
unconservative_chrA07_648408 slicing GSBRNA2T00063083001 at nt 61
alignment score=7 , category=0 , p=0.025582642368013
Cleavage Site 615
All Other Sites
Reads
0
1
2
3
4
5
0
100
200
300
400
500
600
700
GSBRNA2T00063083001 position (nts)

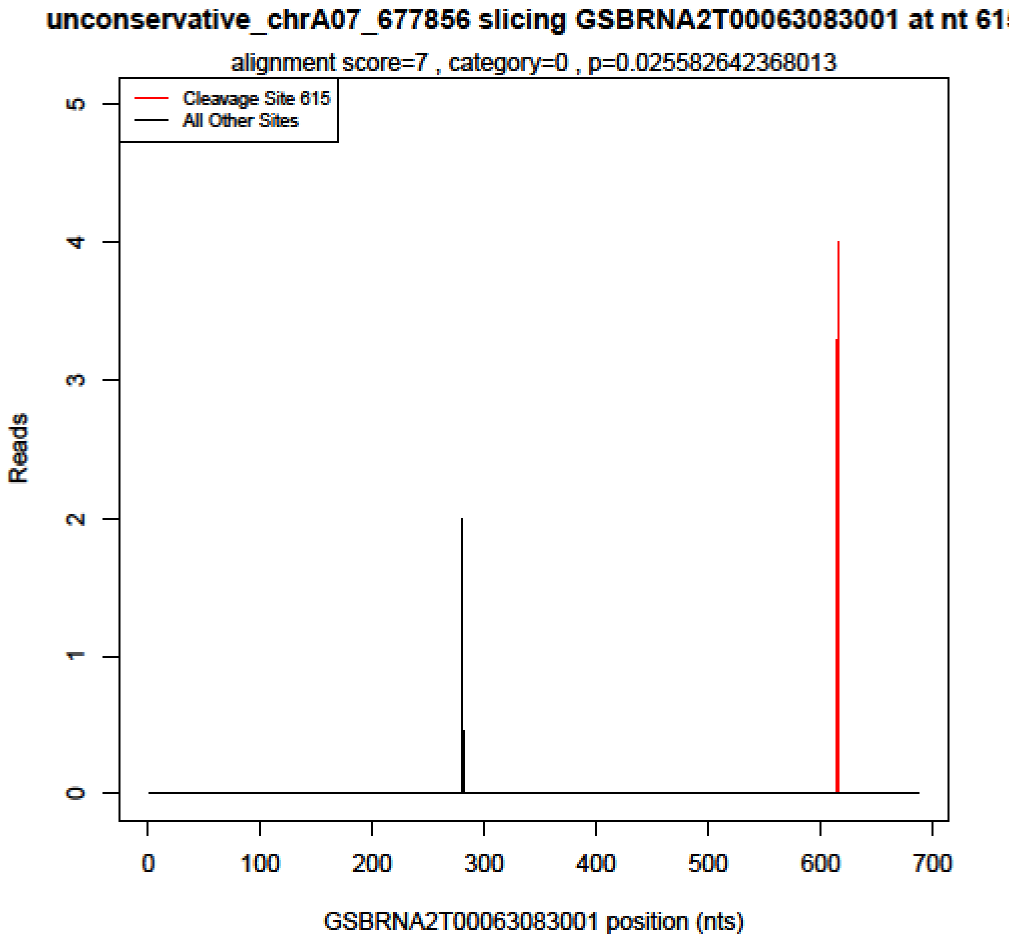

unconservative_chrA07_677856 slicing GSBRNA2T00063083001 at nt 61
alignment score=7 , category=0 , p=0.025582642368013
Cleavage Site 615
All Other Sites
Reads
0
1
2
3
4
5
0
100
200
300
400
500
600
700
GSBRNA2T00063083001 position (nts)

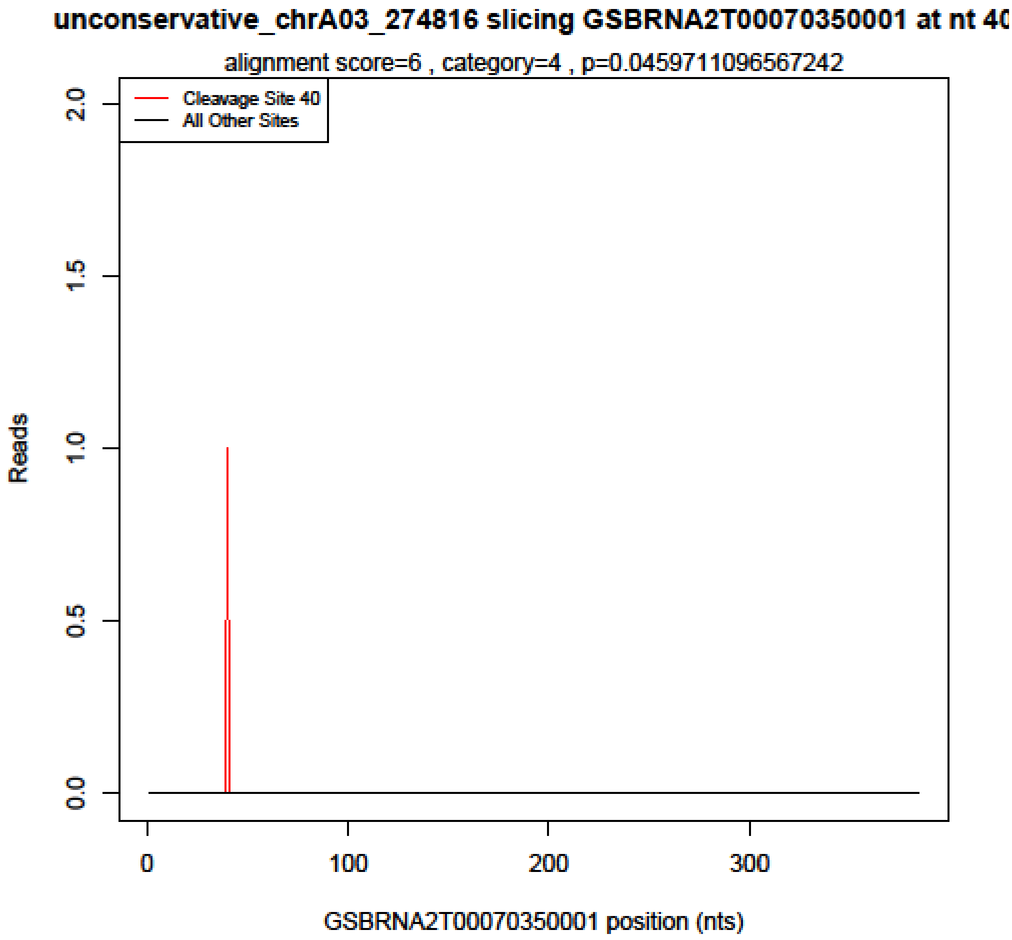

unconservative_chrA03_274816 slicing GSBRNA2T00070350001 at nt 40
alignment score=6 , category=4 , p=0.0459711096567242
Cleavage Site 40
All Other Sites
Reads
0.0
0.5
1.0
1.5
2.0
0
100
200
300
GSBRNA2T00070350001 position (nts)

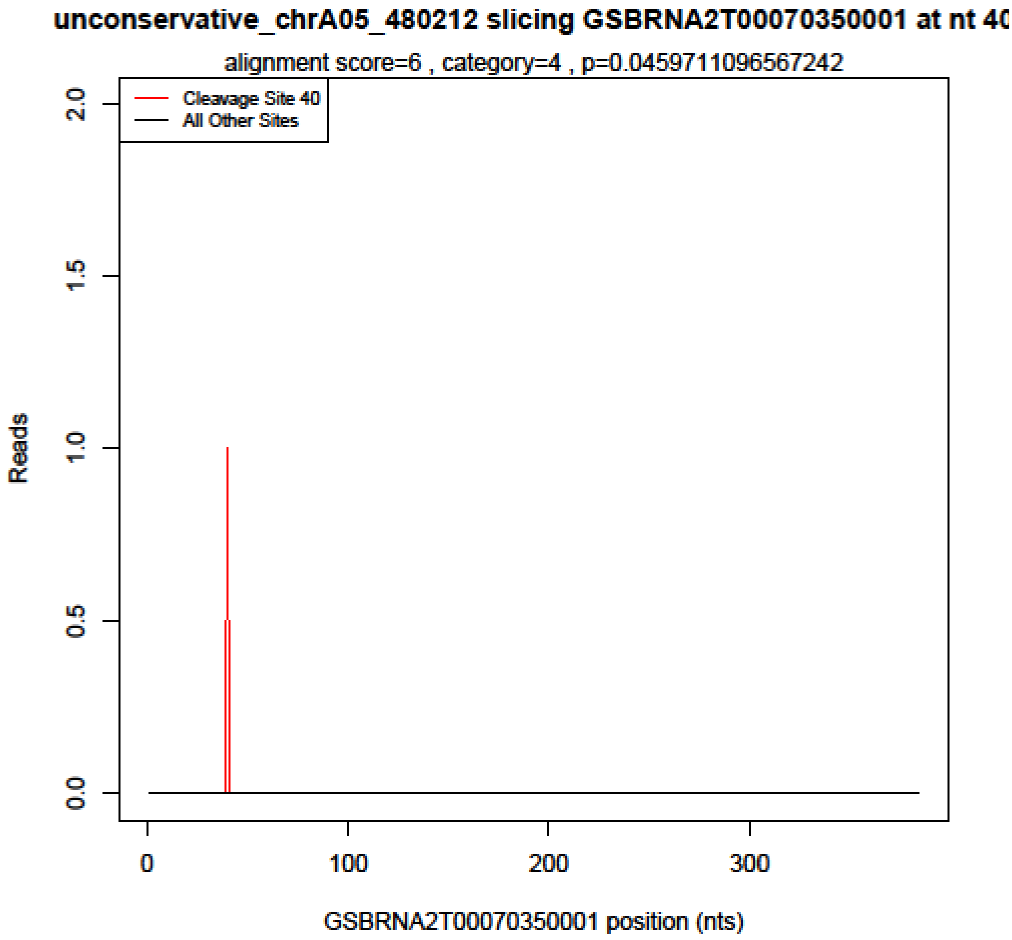
unconservative_chrA05_480212 slicing GSBRNA2T00070350001 at nt 40
alignment score=6 , category=4 , p=0.0459711096567242
Cleavage Site 40
All Other Sites
Reads
0.0
0.5
1.0
1.5
2.0
0
100
200
300
GSBRNA2T00070350001 position (nts)

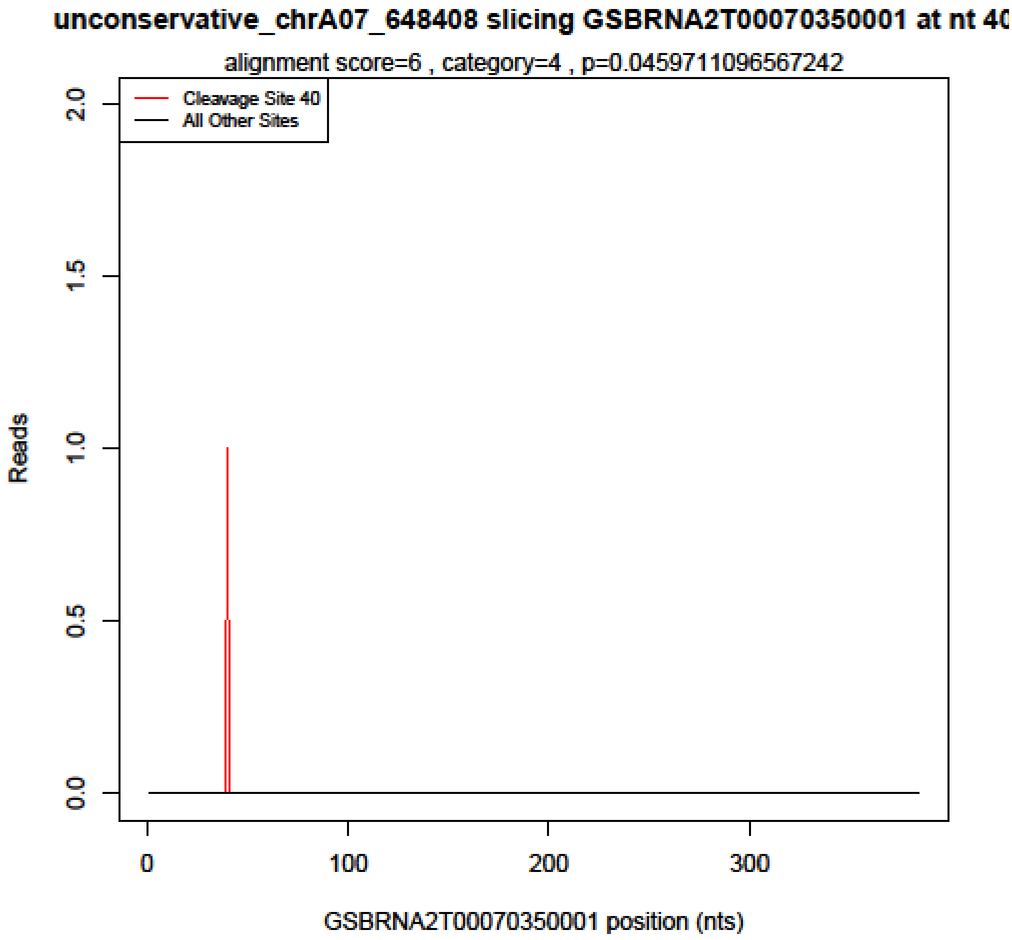
unconservative_chrA07_648408 slicing GSBRNA2T00070350001 at nt 40
alignment score=6 , category=4 , p=0.0459711096567242
Cleavage Site 40
All Other Sites
Reads
0.0
0.5
1.0
1.5
2.0
0
100
200
300
GSBRNA2T00070350001 position (nts)

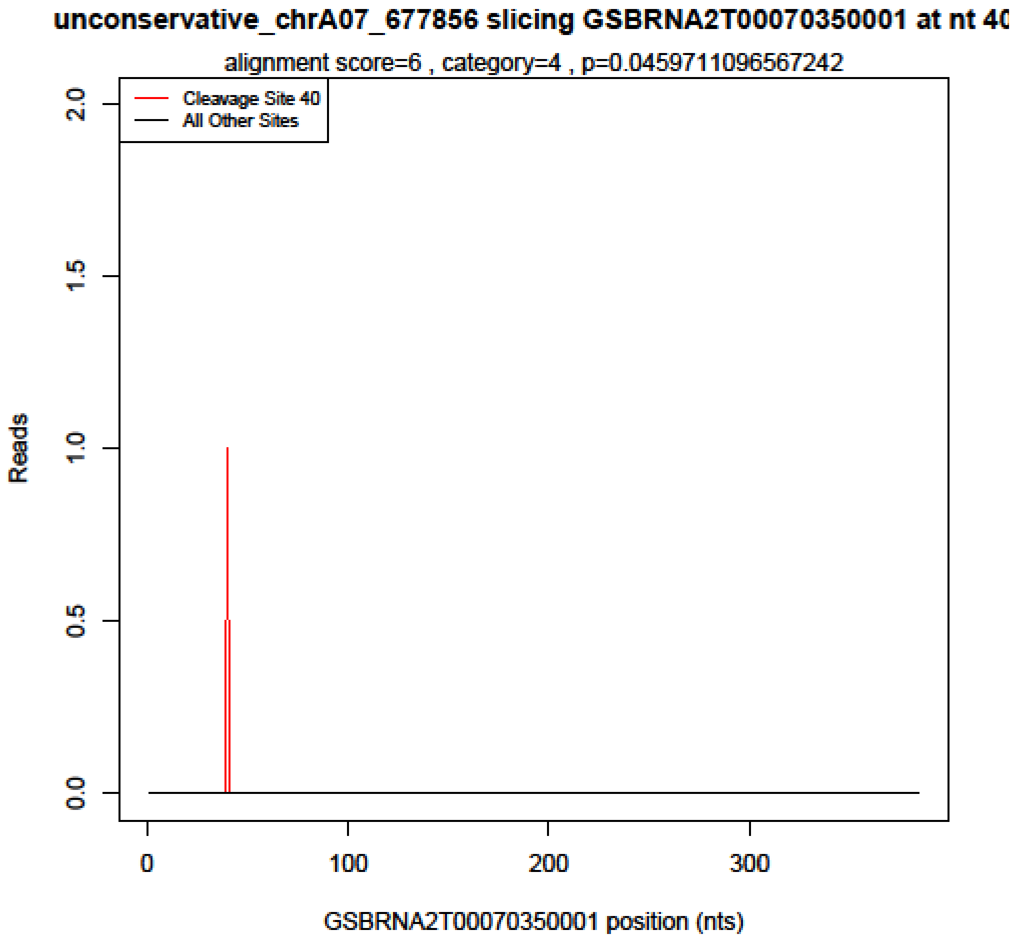
unconservative_chrA07_677856 slicing GSBRNA2T00070350001 at nt 40
alignment score=6 , category=4 , p=0.0459711096567242
Cleavage Site 40
All Other Sites
Reads
0.0
0.5
1.0
1.5
2.0
0
100
200
300
GSBRNA2T00070350001 position (nts)

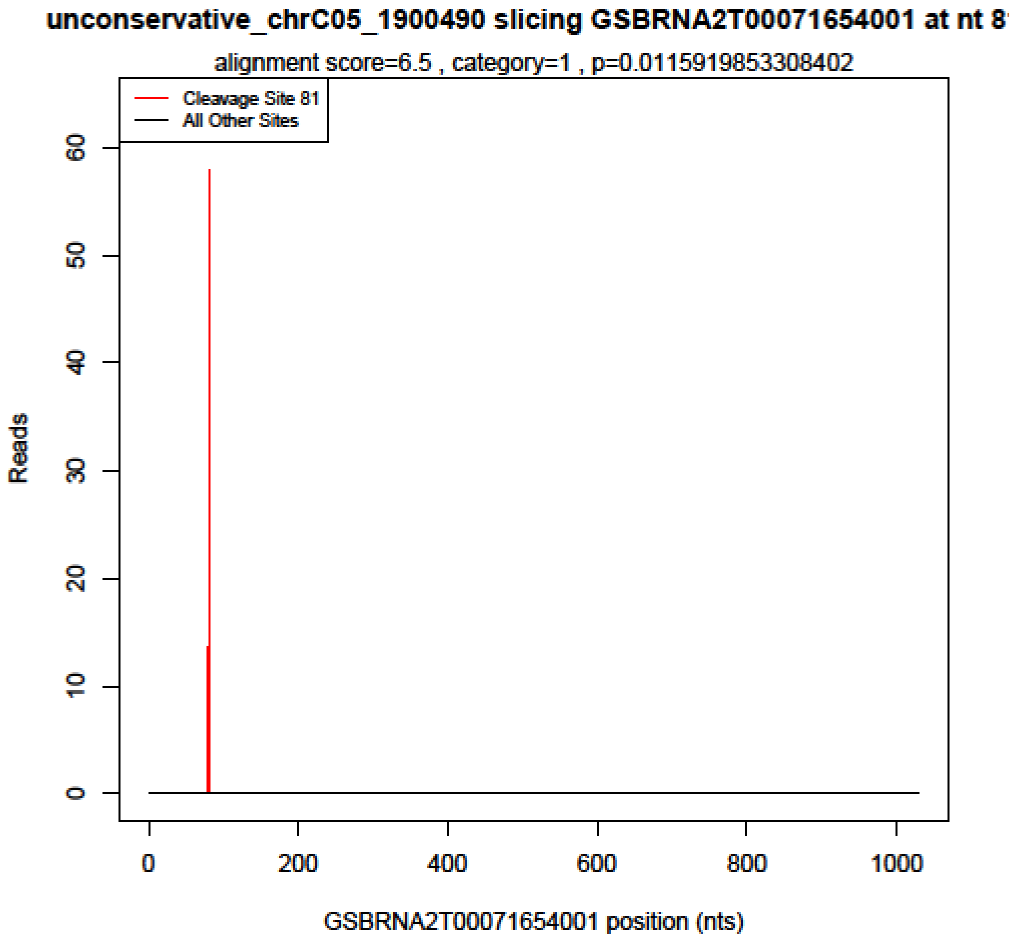
unconservative_chrC05_1900490 slicing GSBRNA2T00071654001 at nt 8
alignment score=6.5 , category=1 , p=0.0115919853308402
Cleavage Site 81
All Other Sites
Reads
0
10
20
30
40
50
60
0
200
400
600
800
1000
GSBRNA2T00071654001 position (nts)

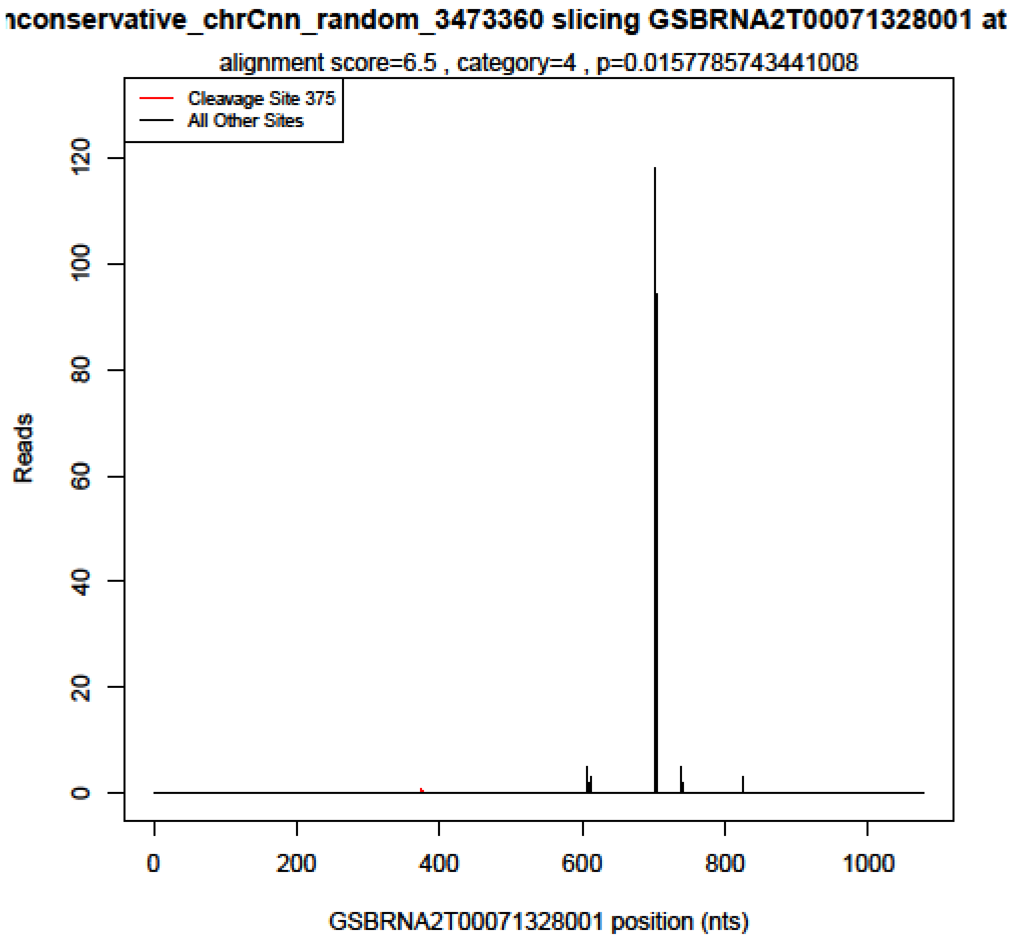

nconservative_chrCnn_random_3473360 slicing GSBRNA2T00071328001 at
alignment score=6.5 , category=4 , p=0.0157785743441008
Cleavage Site 375
All Other Sites
Reads
0
20
40
60
80
100
120
0
200
400
600
800
1000
GSBRNA2T00071328001 position (nts)

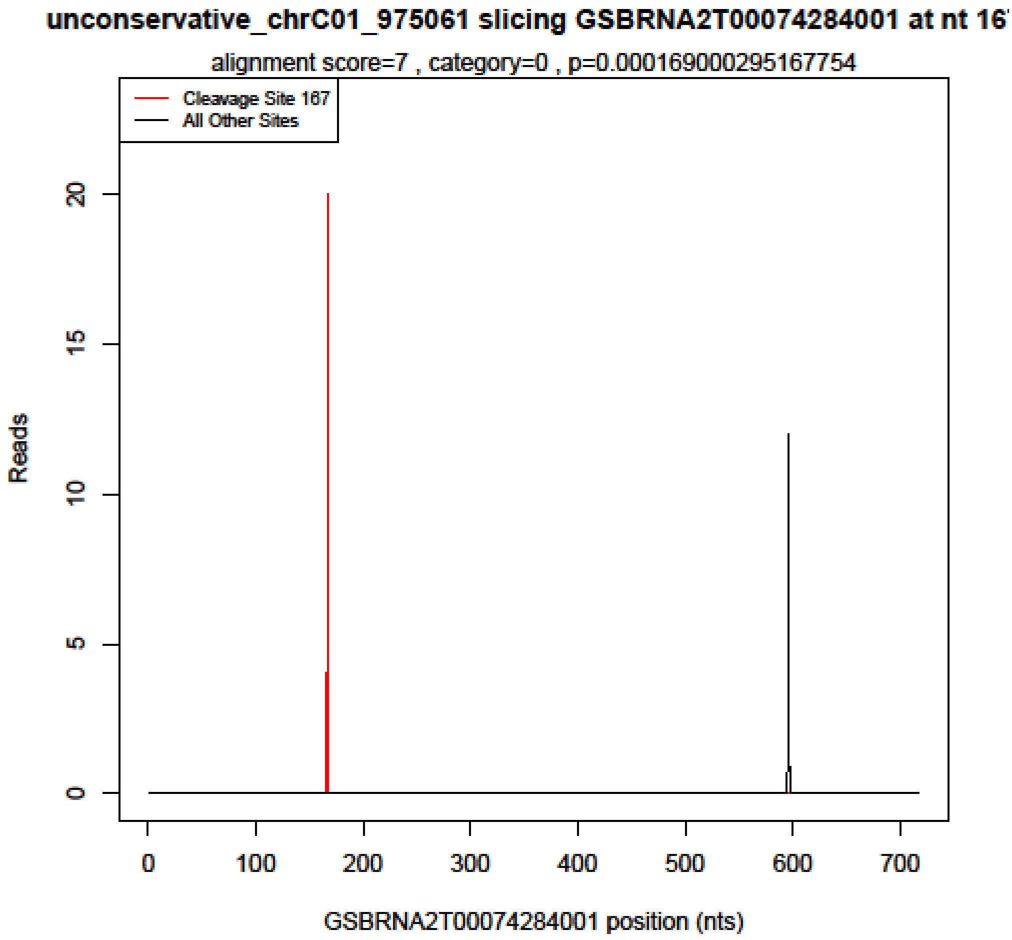

unconservative_chrC01_975061 slicing GSBRNA2T00074284001 at nt 16
alignment score=7 , category=0 , p=0.000169000295167754
Cleavage Site 167
All Other Sites
Reads
0
5
10
15
20
0
100
200
300
400
500
600
700
GSBRNA2T00074284001 position (nts)

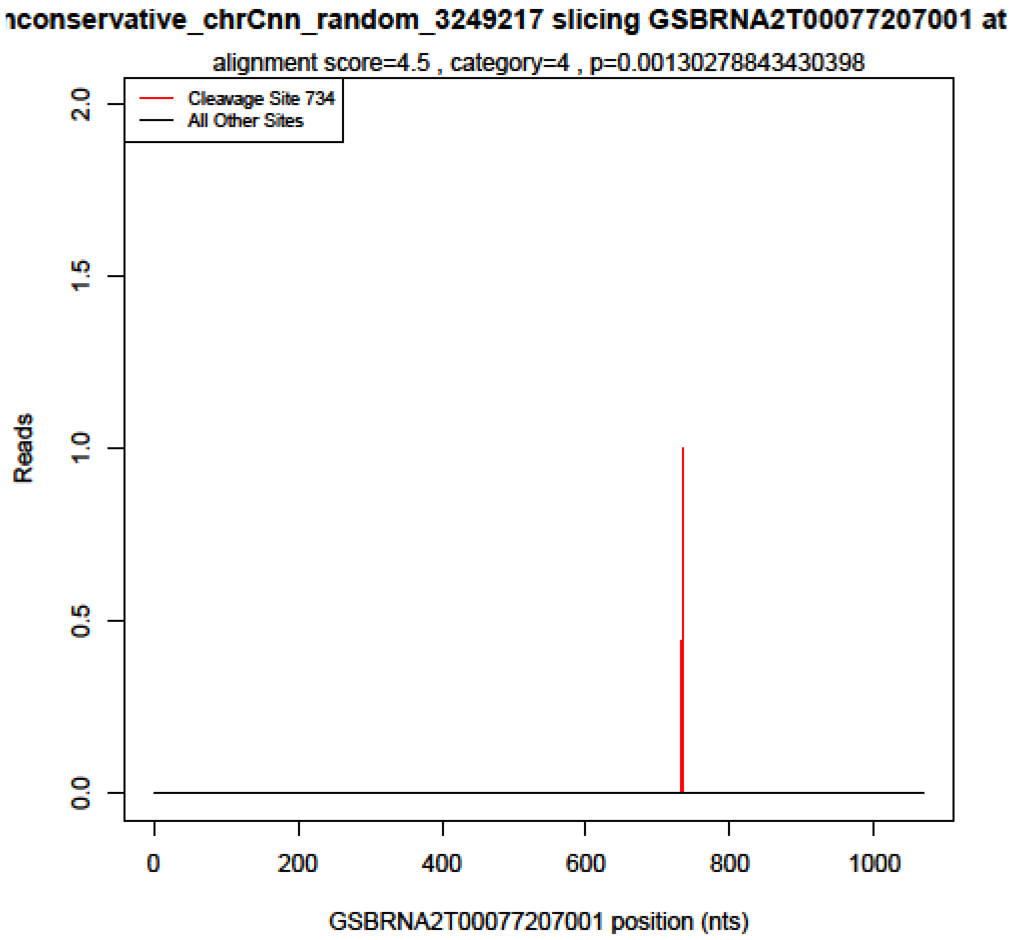

nconservative_chrCnn_random_3249217 slicing GSBRNA2T00077207001 at
alignment score=4.5 , category=4 , p=0.00130278843430398
Cleavage Site 734
All Other Sites
Reads
2.0
1.5
1.0
0.5
0.0
0
200
400
600
800
1000
GSBRNA2T00077207001 position (nts)

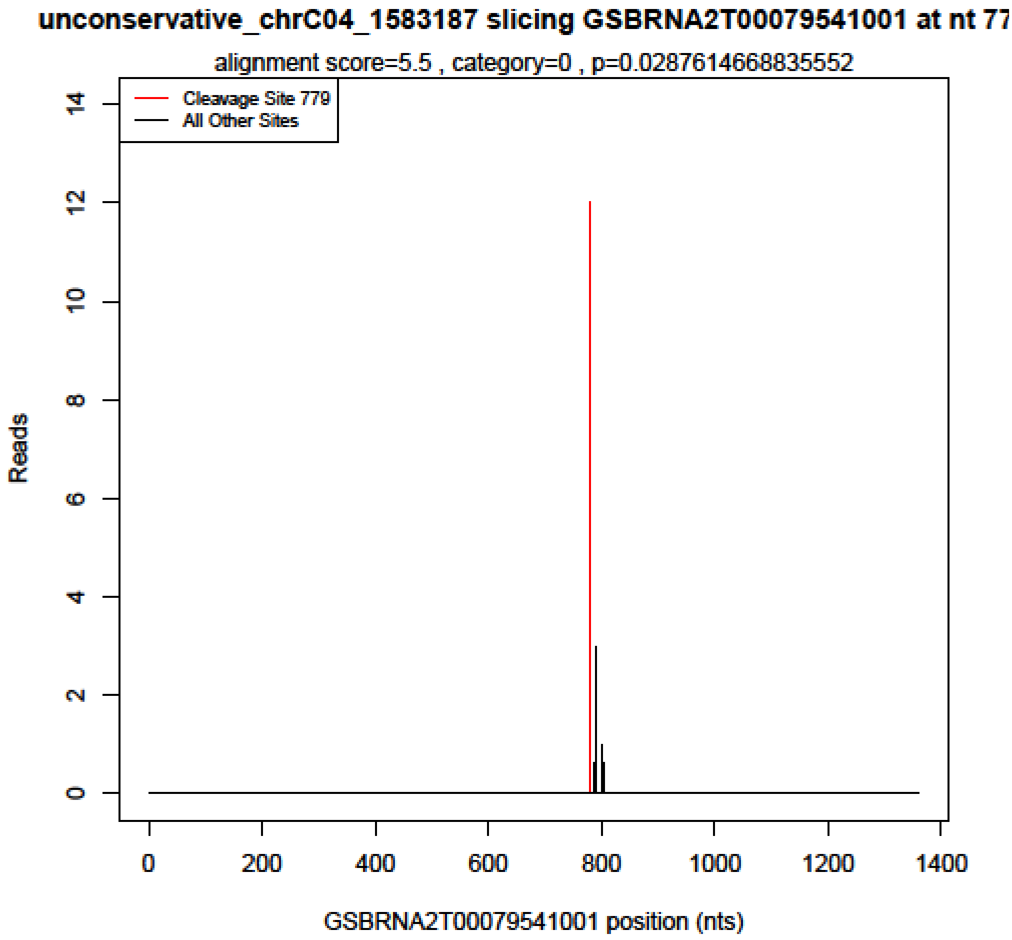

unconservative_chrC04_1583187 slicing GSBRNA2T00079541001 at nt 77
alignment score=5.5 , category=0 , p=0.0287614668835552
Cleavage Site 779
All Other Sites
Reads
0
2
4
6
8
10
12
14
0
200
400
600
800
1000
1200
1400
GSBRNA2T00079541001 position (nts)

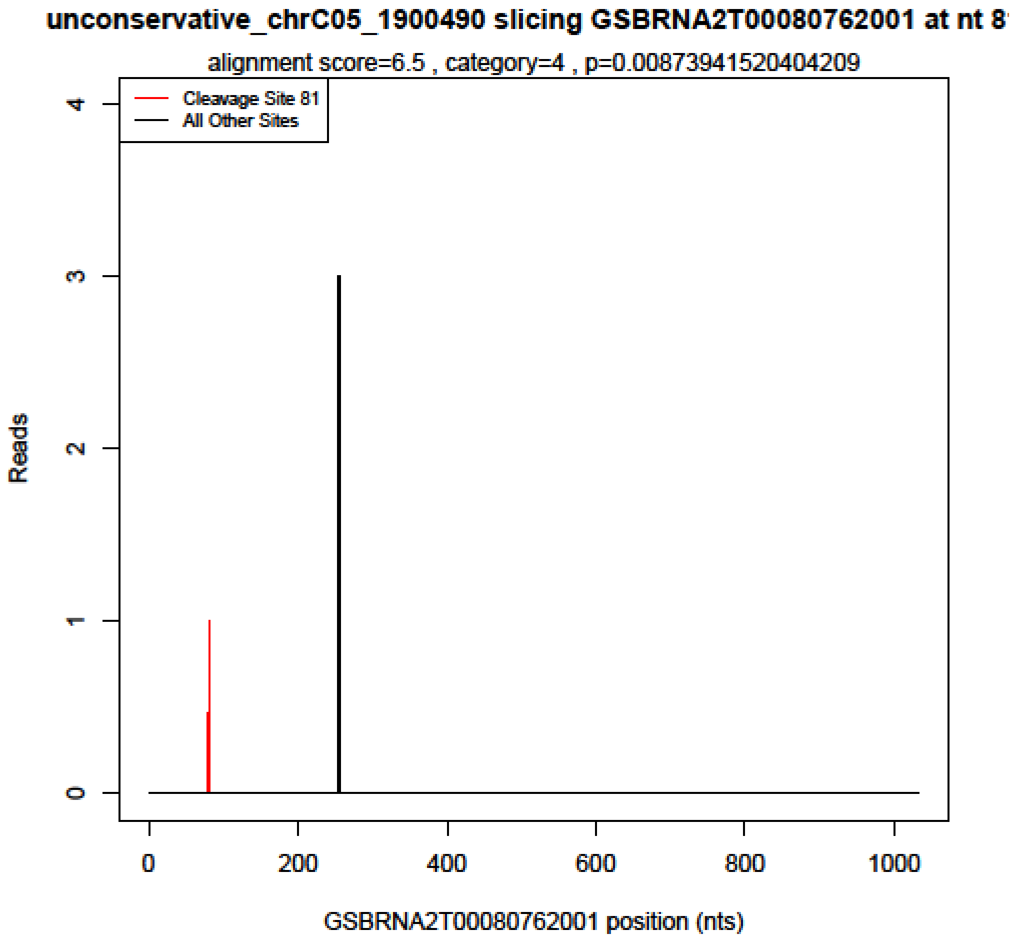

unconservative_chrC05_1900490 slicing GSBRNA2T00080762001 at nt 8
alignment score=6.5 , category=4 , p=0.00873941520404209
Cleavage Site 81
All Other Sites
Reads
0
1
2
3
4
0
200
400
600
800
1000
GSBRNA2T00080762001 position (nts)

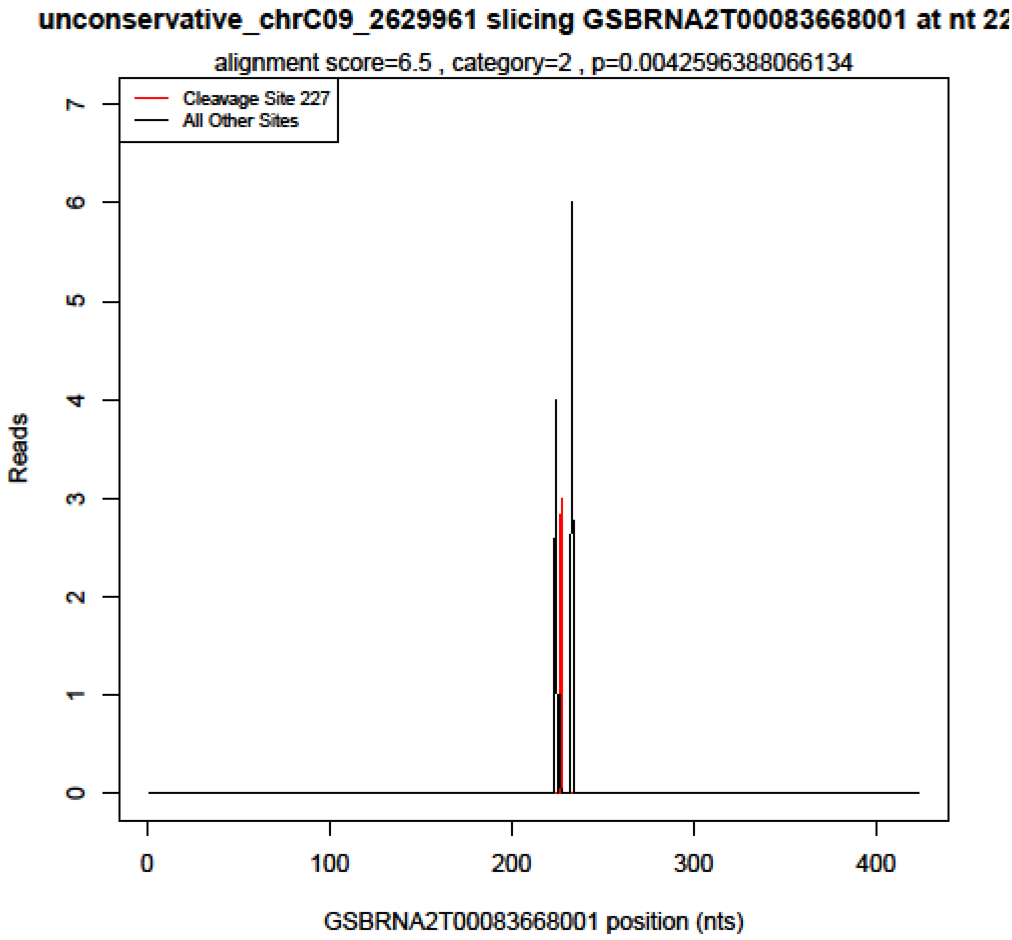

unconservative_chrC09_2629961 slicing GSBRNA2T00083668001 at nt 22
alignment score=6.5 , category=2 , p=0.0042596388066134
Cleavage Site 227
All Other Sites
Reads
0
1
2
3
4
5
6
7
0
100
200
300
400
GSBRNA2T00083668001 position (nts)

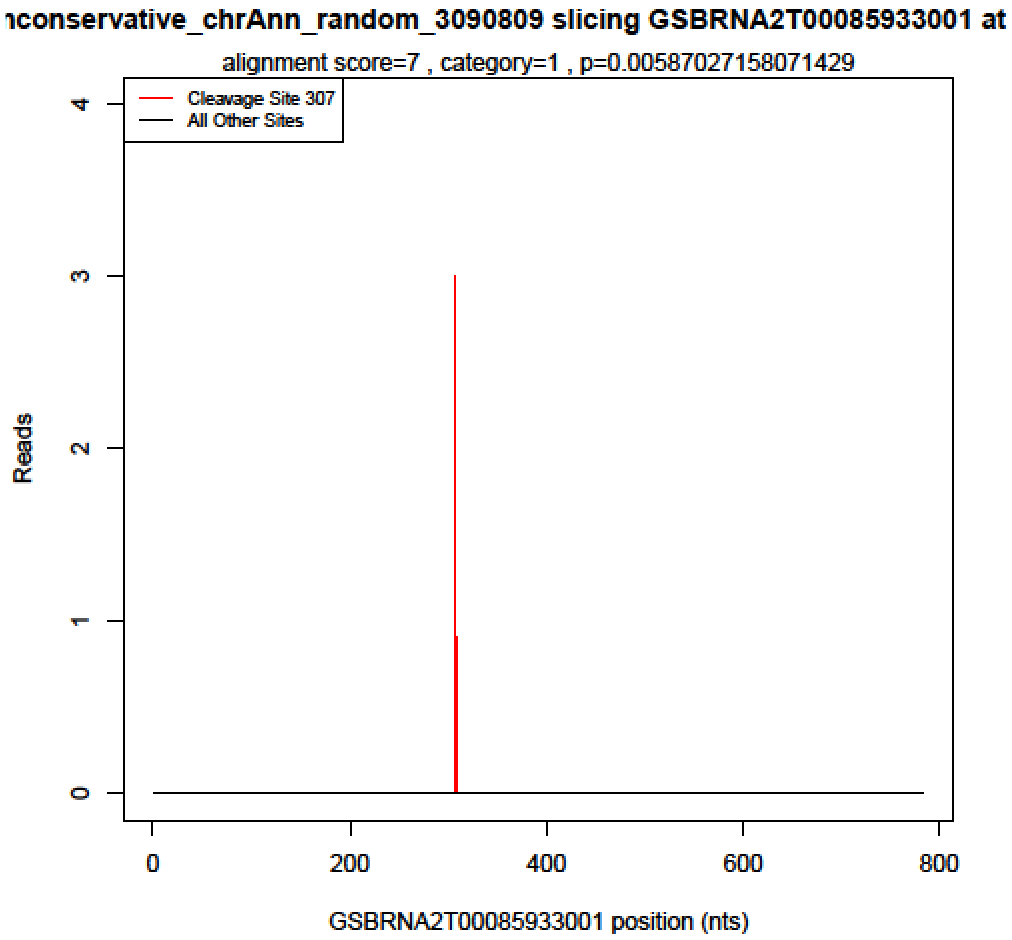

nconservative_chrAnn_random_3090809 slicing GSBRNA2T00085933001 at
alignment score=7 , category=1 , p=0.00587027158071429
Cleavage Site 307
All Other Sites
Reads
0
1
2
3
4
0
200
400
600
800
GSBRNA2T00085933001 position (nts)

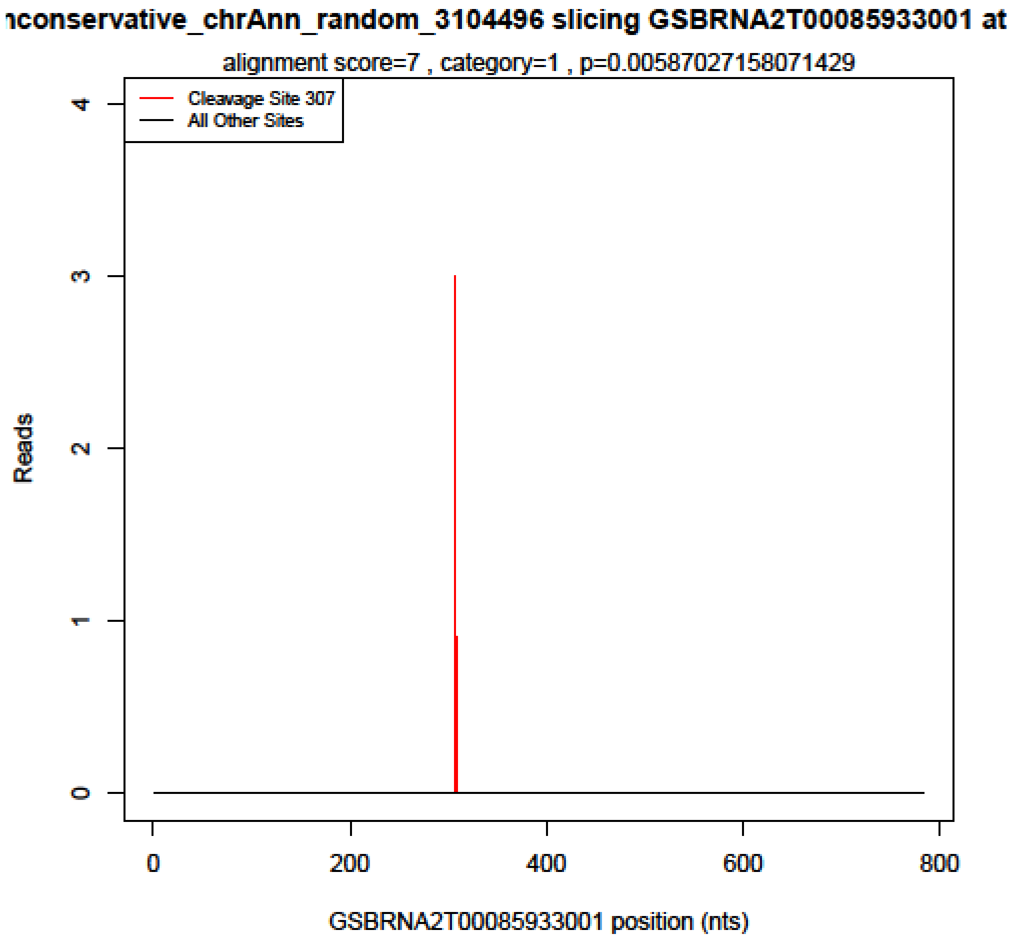

nconservative_chrAnn_random_3104496 slicing GSBRNA2T00085933001 at
alignment score=7 , category=1 , p=0.00587027158071429
Cleavage Site 307
All Other Sites
Reads
0
1
2
3
4
0
200
400
600
800
GSBRNA2T00085933001 position (nts)

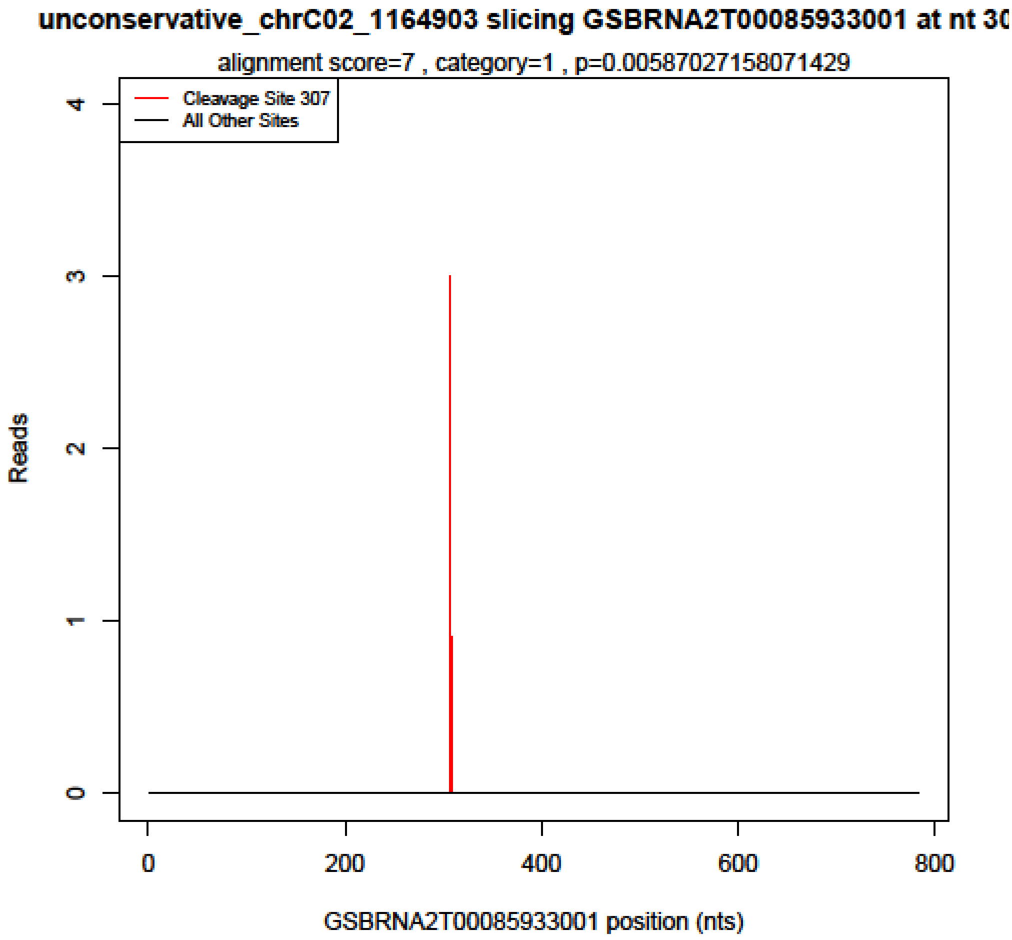

unconservative_chrC02_1164903 slicing GSBRNA2T00085933001 at nt 30
alignment score=7 , category=1 , p=0.00587027158071429
Cleavage Site 307
All Other Sites
Reads
0
1
2
3
4
0
200
400
600
800
GSBRNA2T00085933001 position (nts)

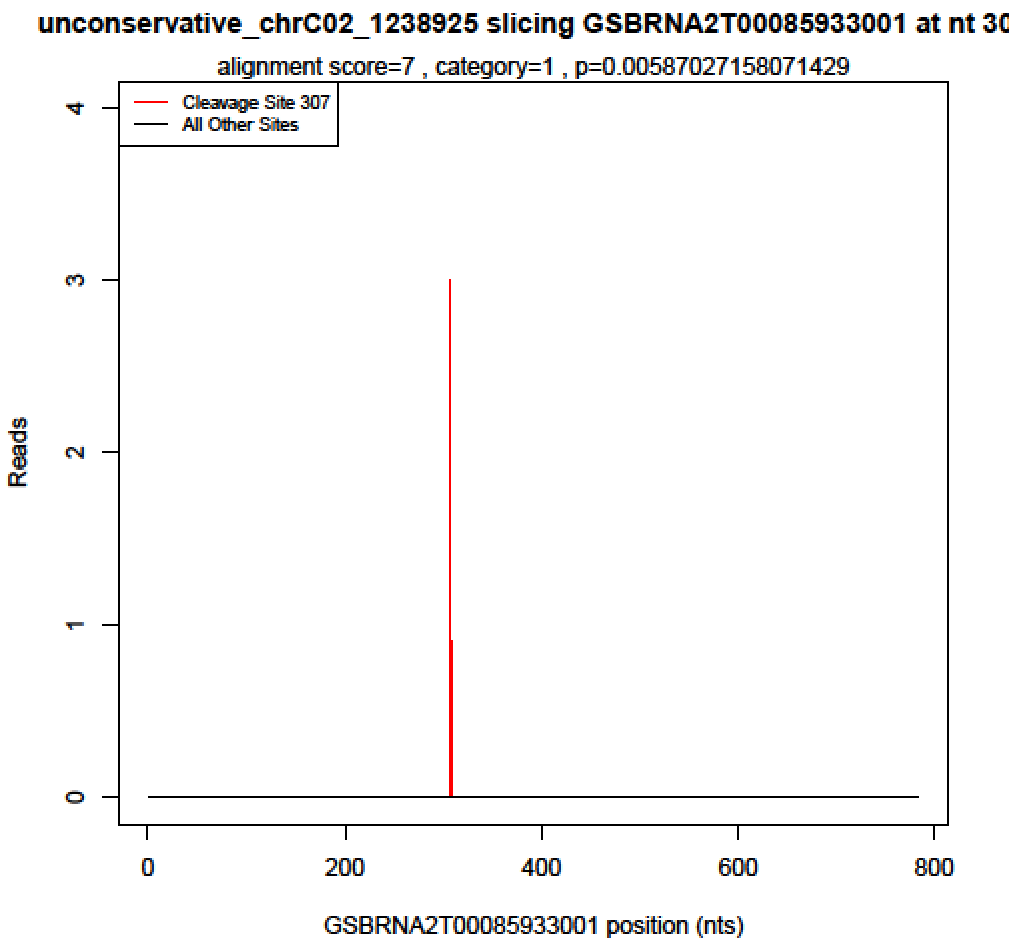
unconservative_chrC02_1238925 slicing GSBRNA2T00085933001 at nt 30
alignment score=7 , category=1 , p=0.00587027158071429
Cleavage Site 307
All Other Sites
Reads
0
1
2
3
4
0
200
400
600
800
GSBRNA2T00085933001 position (nts)

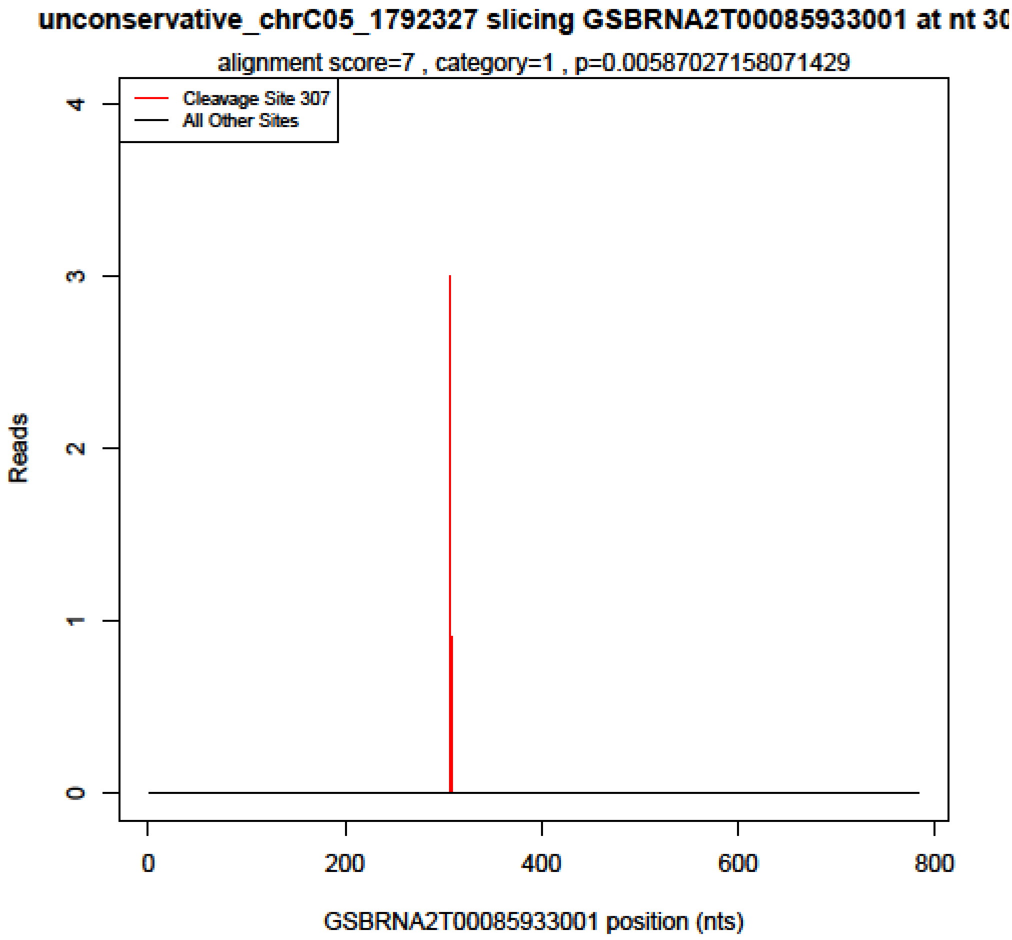

unconservative_chrC05_1792327 slicing GSBRNA2T00085933001 at nt 30
alignment score=7 , category=1 , p=0.00587027158071429
Cleavage Site 307
All Other Sites
Reads
0
1
2
3
4
0
200
400
600
800
GSBRNA2T00085933001 position (nts)

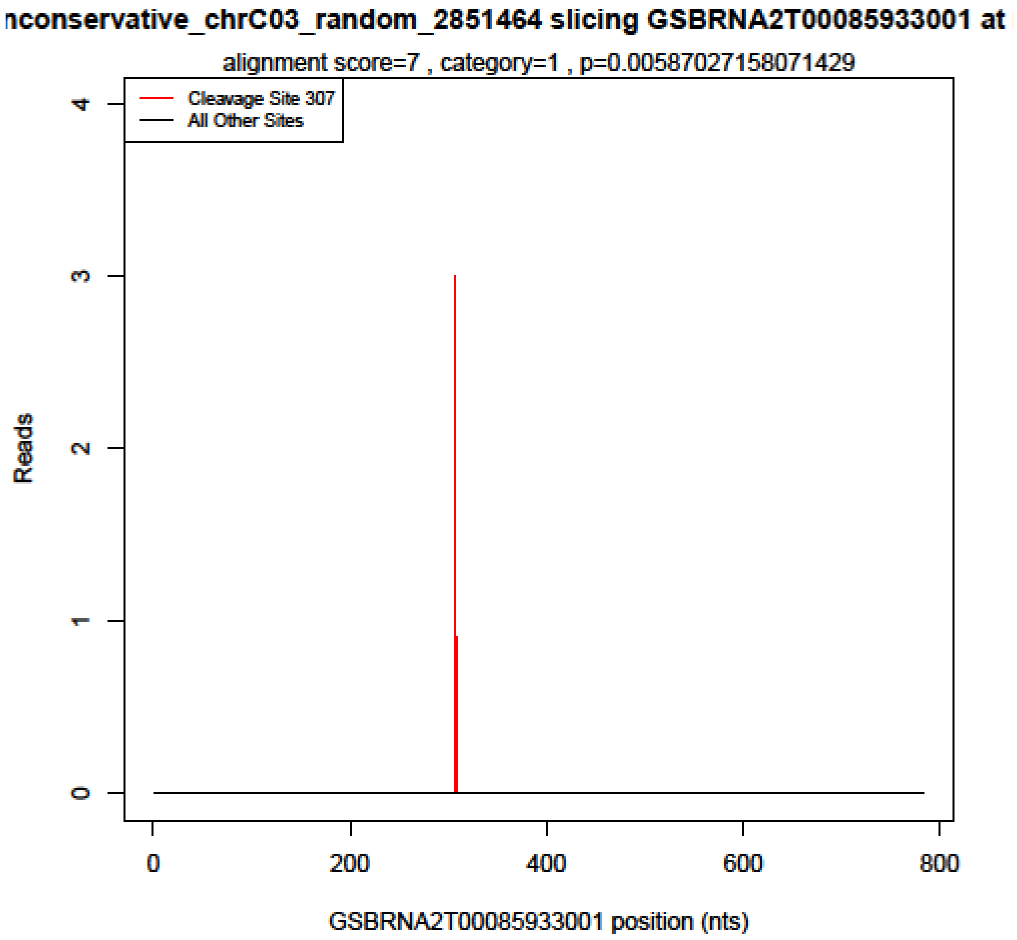
nconservative_chrC03_random_2851464 slicing GSBRNA2T00085933001 at
alignment score=7 , category=1 , p=0.00587027158071429
Cleavage Site 307
All Other Sites
Reads
0
1
2
3
4
0
200
400
600
800
GSBRNA2T00085933001 position (nts)

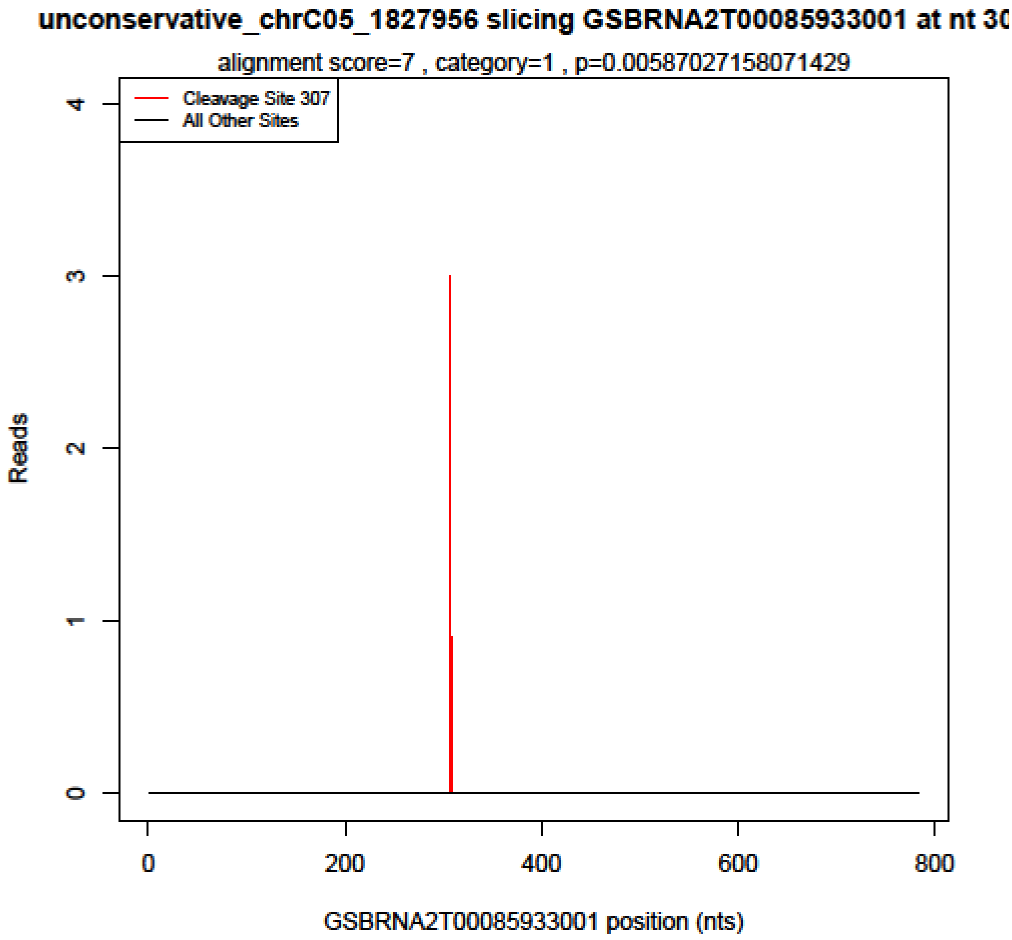

unconservative_chrC05_1827956 slicing GSBRNA2T00085933001 at nt 30
alignment score=7 , category=1 , p=0.00587027158071429
Cleavage Site 307
All Other Sites
Reads
0
1
2
3
4
0
200
400
600
800
GSBRNA2T00085933001 position (nts)

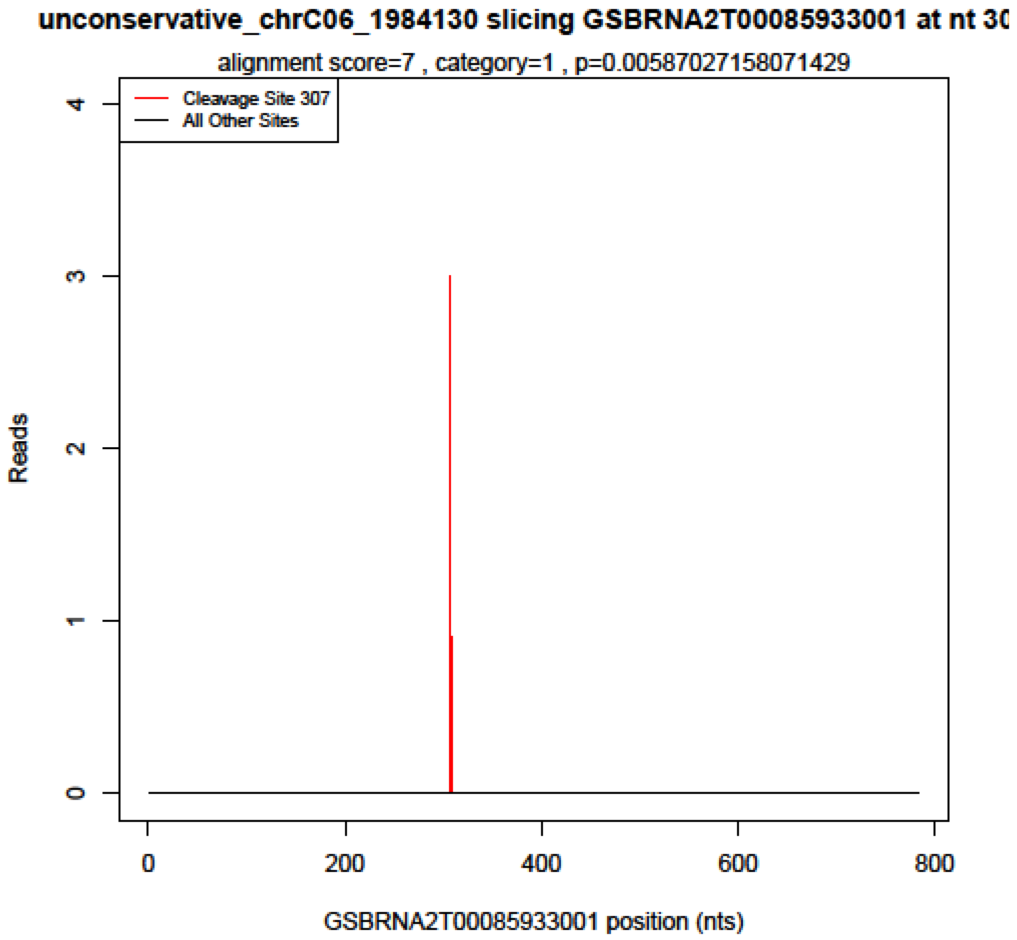

unconservative_chrC06_1984130 slicing GSBRNA2T00085933001 at nt 30
alignment score=7 , category=1 , p=0.00587027158071429
Cleavage Site 307
All Other Sites
Reads
0
1
2
3
4
0
200
400
600
800
GSBRNA2T00085933001 position (nts)

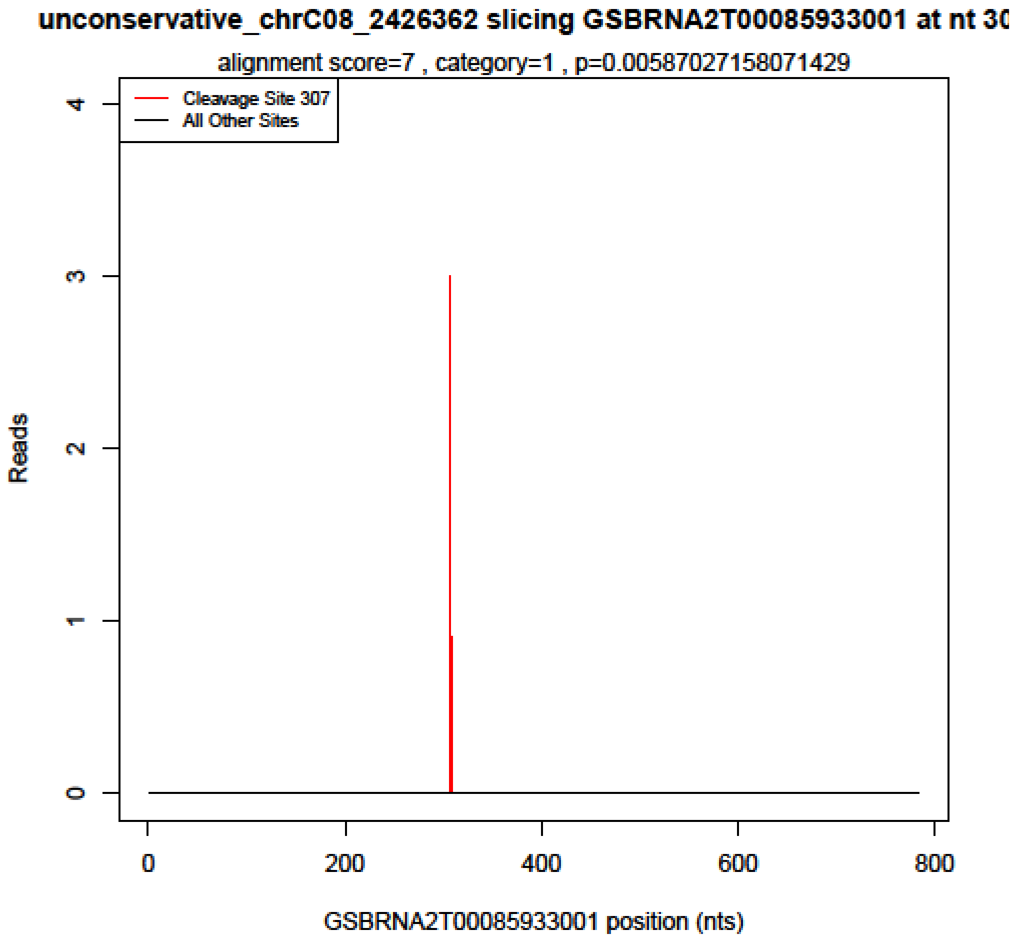
unconservative_chrC08_2426362 slicing GSBRNA2T00085933001 at nt 30
alignment score=7 , category=1 , p=0.00587027158071429
Cleavage Site 307
All Other Sites
Reads
0
1
2
3
4
0
200
400
600
800
GSBRNA2T00085933001 position (nts)

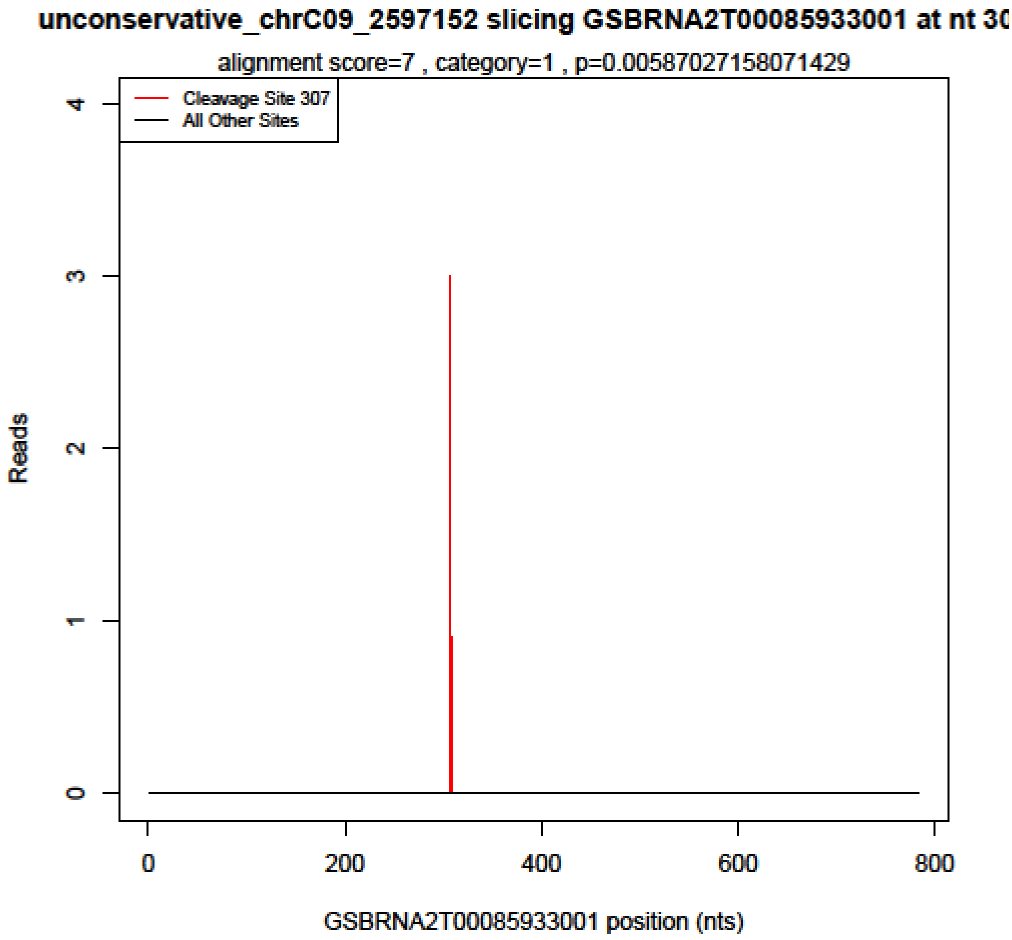
unconservative_chrC09_2597152 slicing GSBRNA2T00085933001 at nt 30
alignment score=7 , category=1 , p=0.00587027158071429
Cleavage Site 307
All Other Sites
Reads
0
1
2
3
4
0
200
400
600
800
GSBRNA2T00085933001 position (nts)

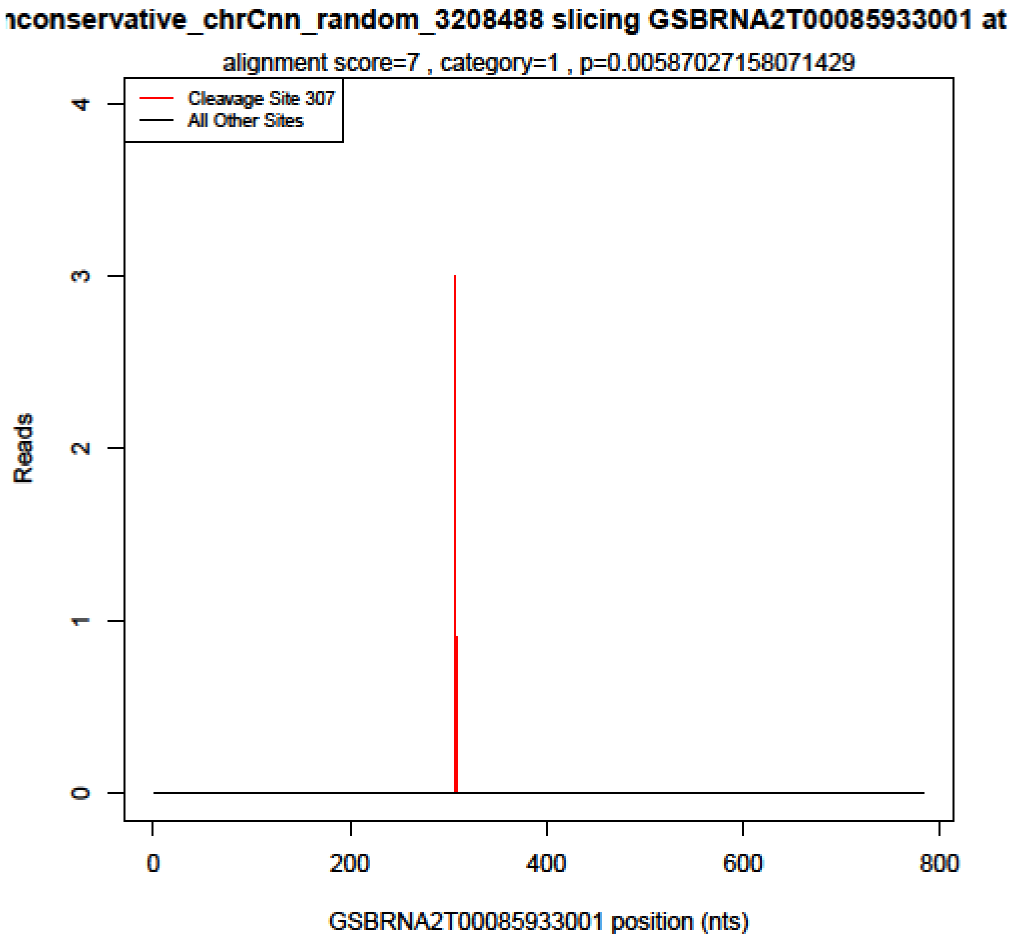

nconservative_chrCnn_random_3208488 slicing GSBRNA2T00085933001 at
alignment score=7 , category=1 , p=0.00587027158071429
Cleavage Site 307
All Other Sites
Reads
0
1
2
3
4
0
200
400
600
800
GSBRNA2T00085933001 position (nts)

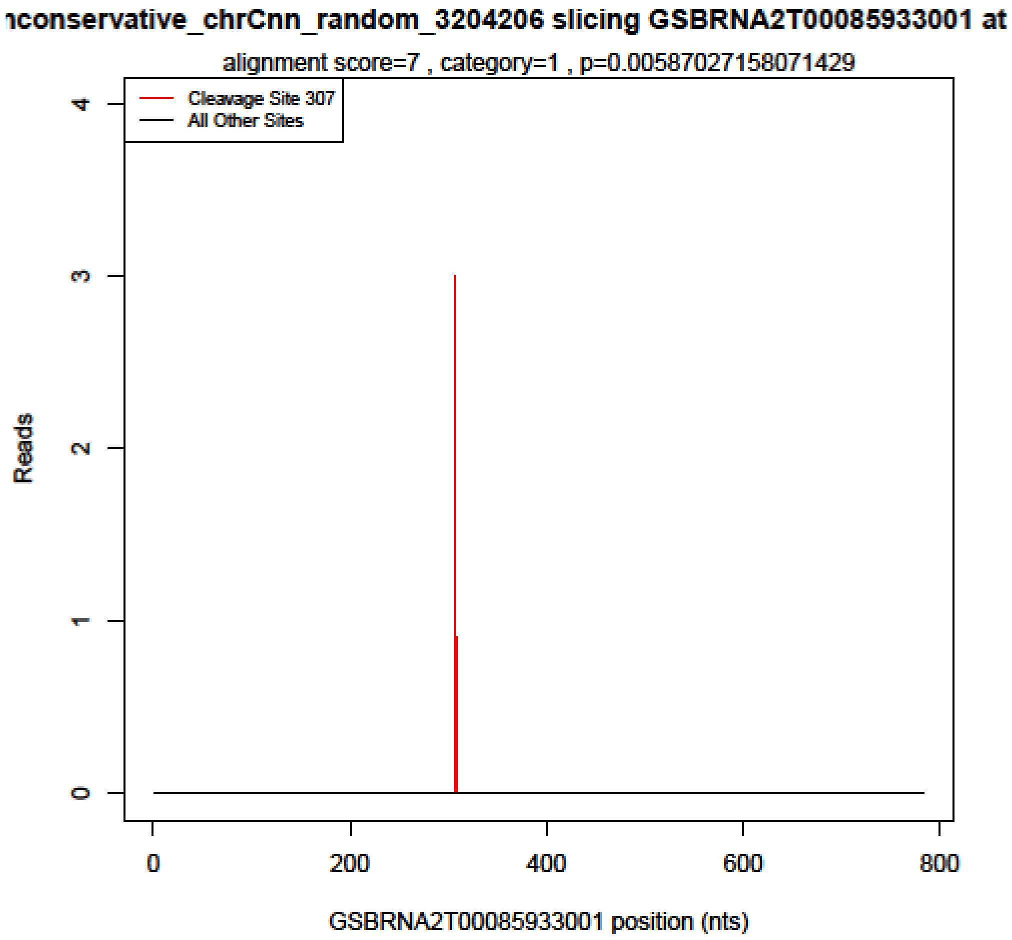

nconservative_chrCnn_random_3204206 slicing GSBRNA2T00085933001 at
alignment score=7 , category=1 , p=0.00587027158071429
Cleavage Site 307
All Other Sites
Reads
0
1
2
3
4
0
200
400
600
800
GSBRNA2T00085933001 position (nts)

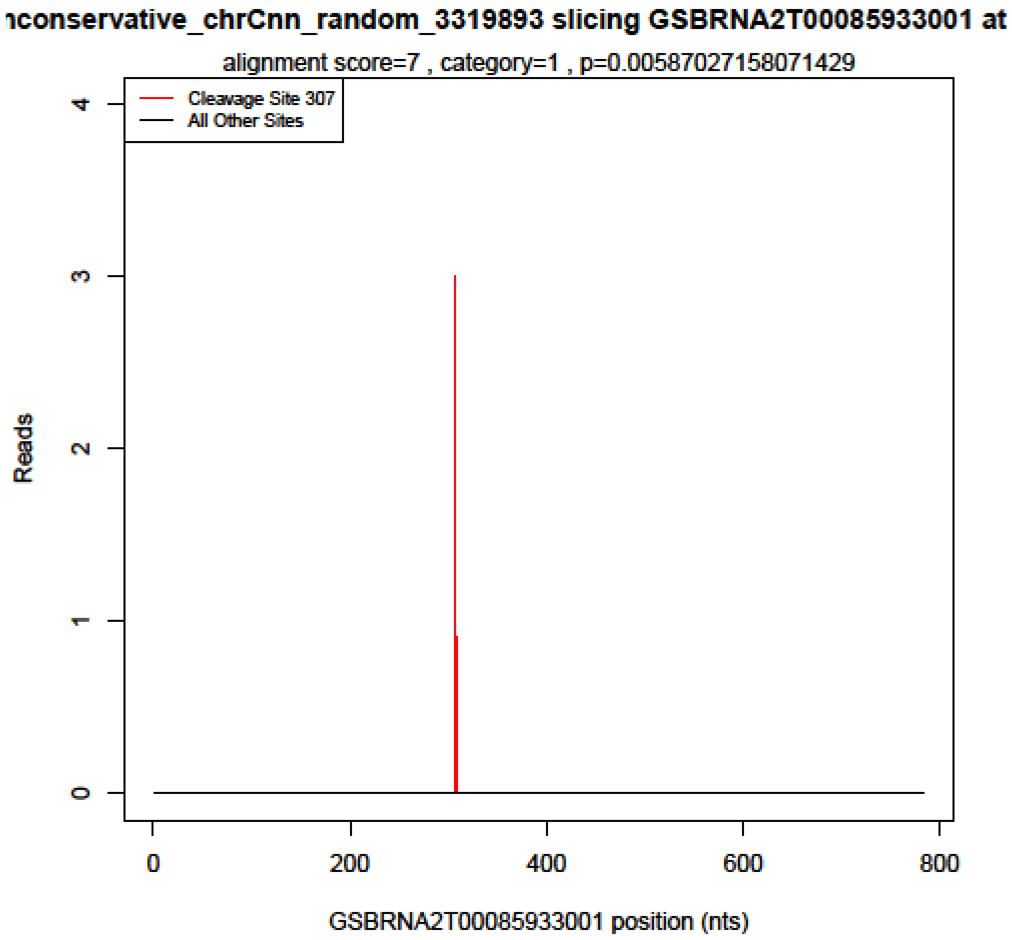
nconservative_chrCnn_random_3319893 slicing GSBRNA2T00085933001 at
alignment score=7 , category=1 , p=0.00587027158071429
Cleavage Site 307
All Other Sites
Reads
0
1
2
3
4
0
200
400
600
800
GSBRNA2T00085933001 position (nts)

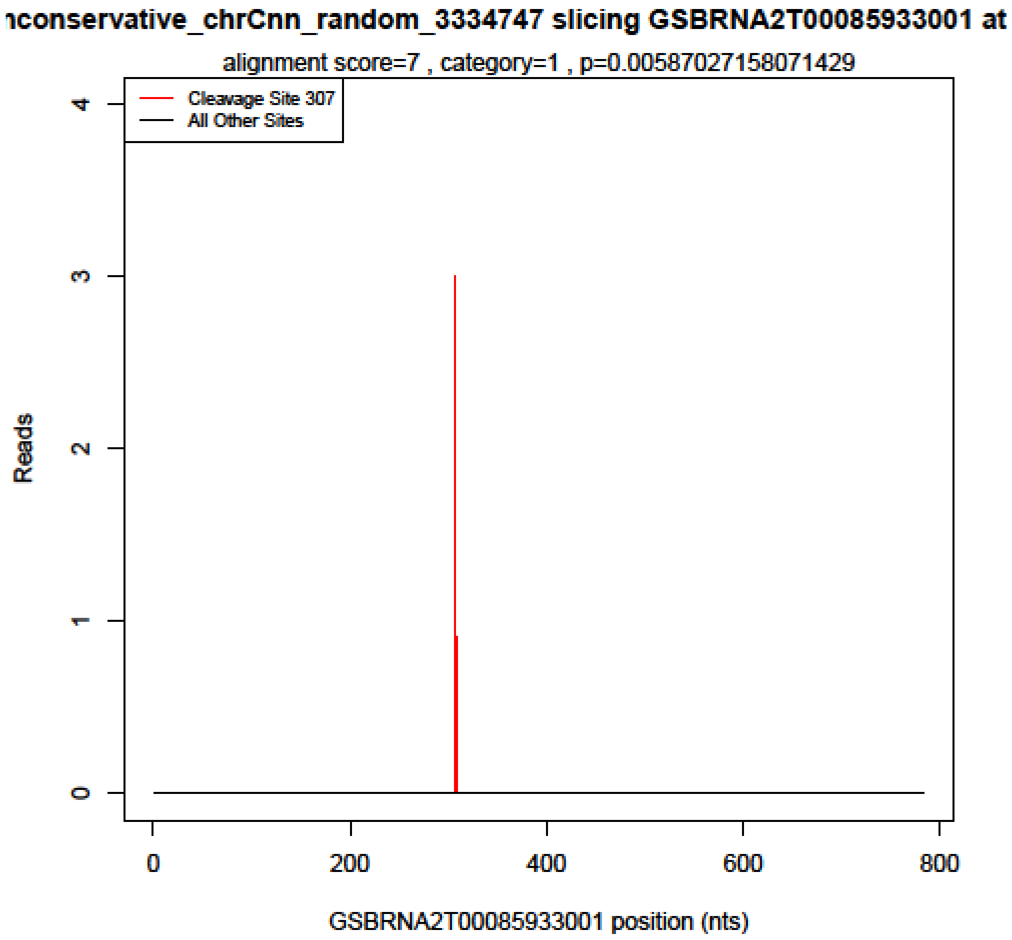

nconservative_chrCnn_random_3334747 slicing GSBRNA2T00085933001 at
alignment score=7 , category=1 , p=0.00587027158071429
Cleavage Site 307
All Other Sites
Reads
0
1
2
3
4
0
200
400
600
800
GSBRNA2T00085933001 position (nts)

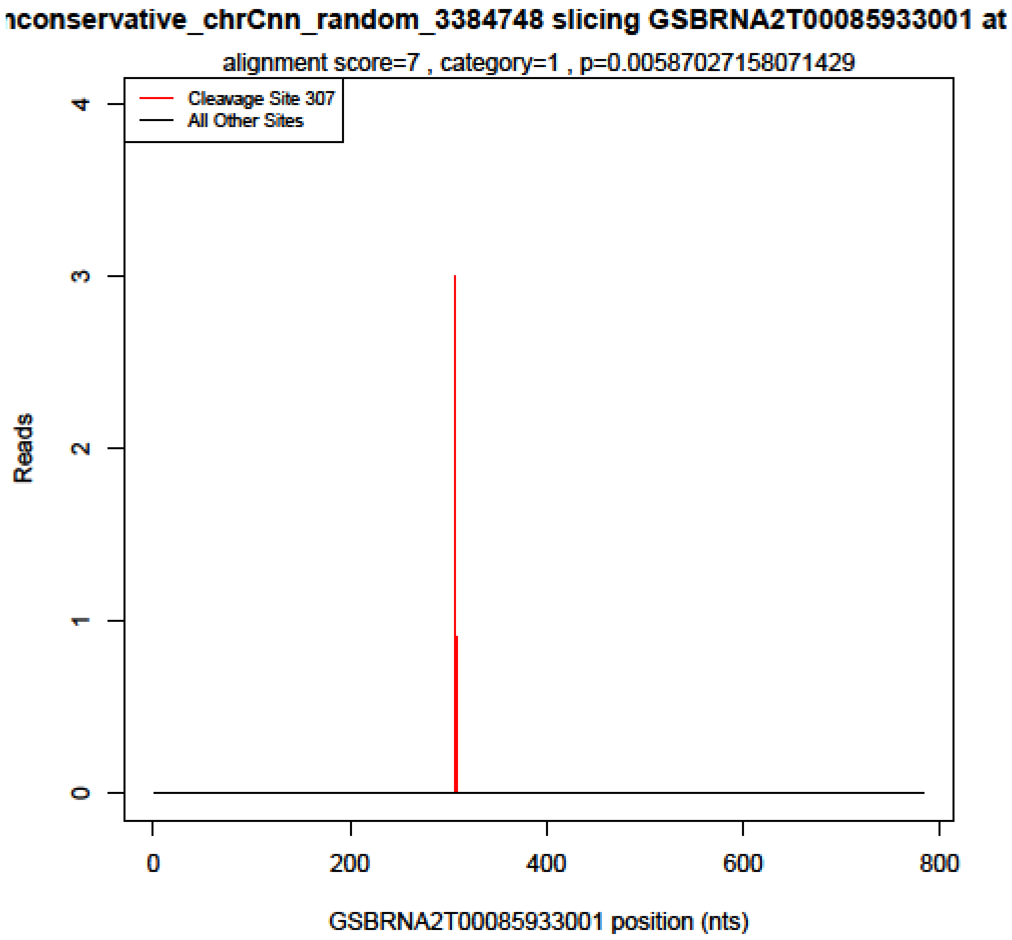

nconservative_chrCnn_random_3384748 slicing GSBRNA2T00085933001 at
alignment score=7 , category=1 , p=0.00587027158071429
Cleavage Site 307
All Other Sites
Reads
0
1
2
3
4
0
200
400
600
800
GSBRNA2T00085933001 position (nts)

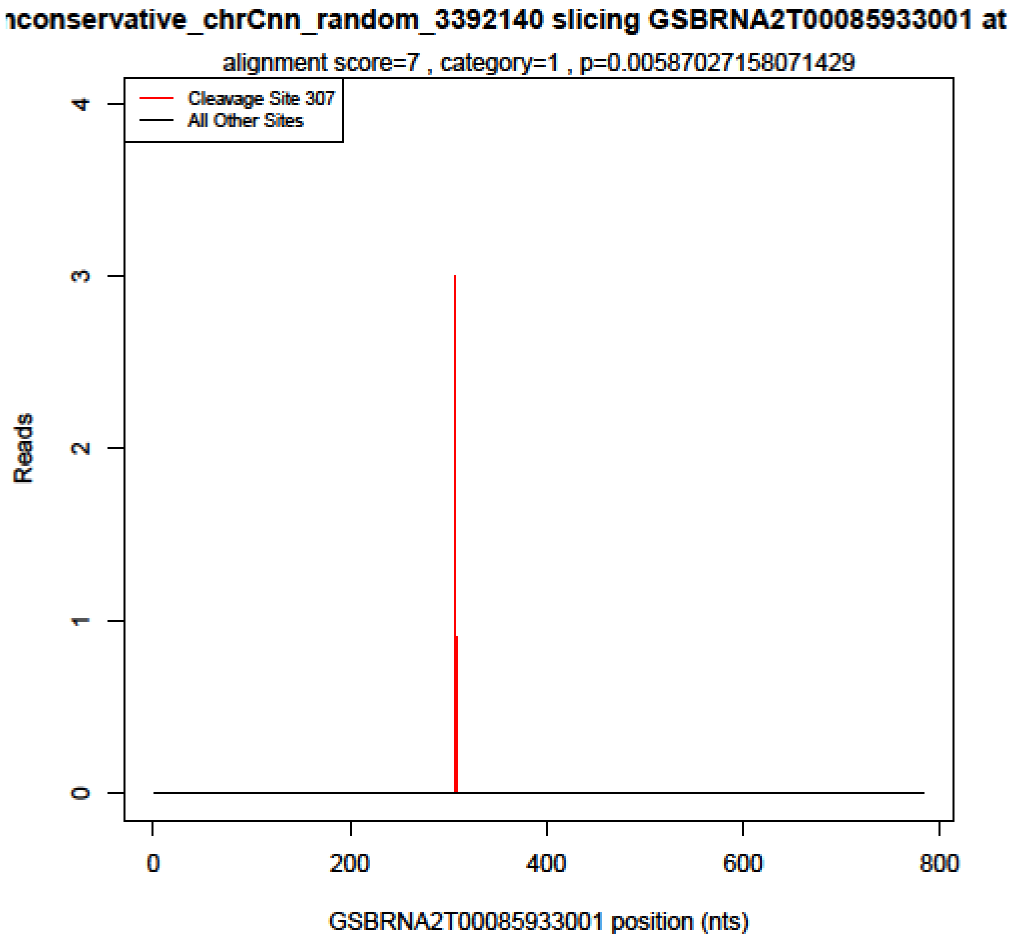
nconservative_chrCnn_random_3392140 slicing GSBRNA2T00085933001 at
alignment score=7 , category=1 , p=0.00587027158071429
Cleavage Site 307
All Other Sites
Reads
0
1
2
3
4
0
200
400
600
800
GSBRNA2T00085933001 position (nts)

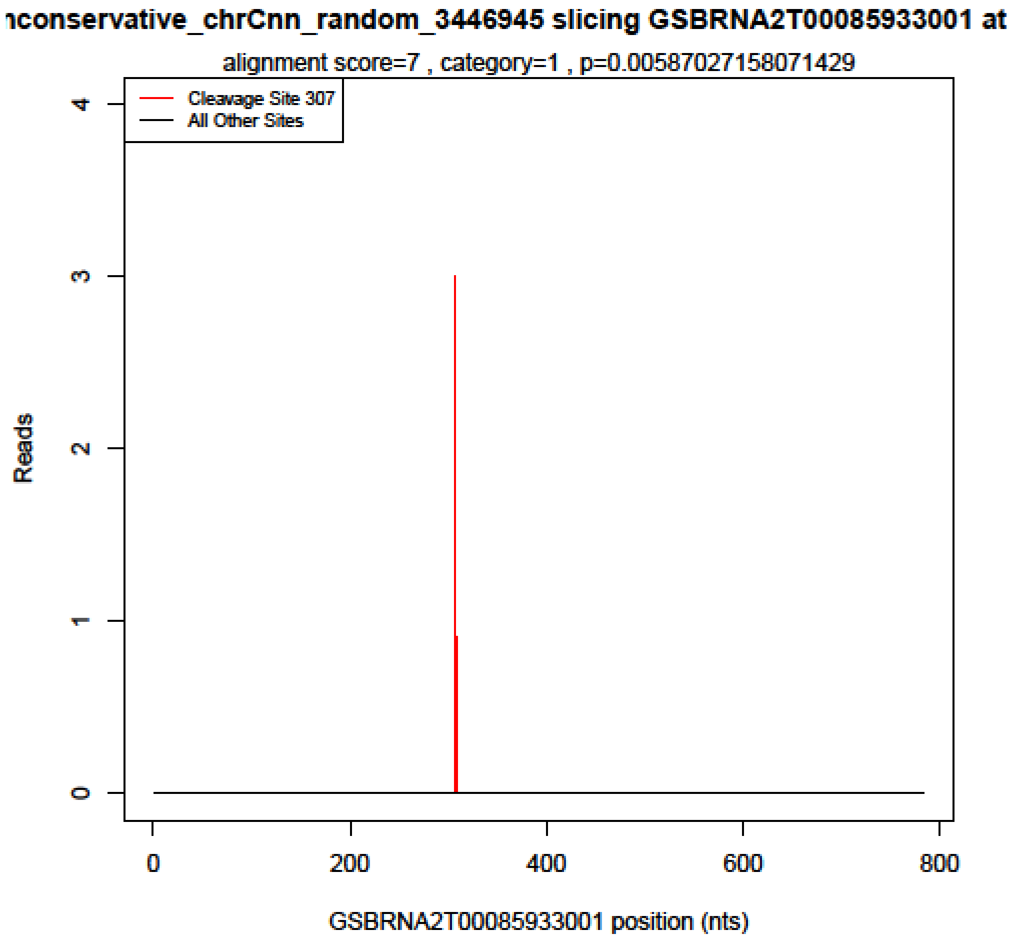

nconservative_chrCnn_random_3446945 slicing GSBRNA2T00085933001 at
alignment score=7 , category=1 , p=0.00587027158071429
Cleavage Site 307
All Other Sites
Reads
0
1
2
3
4
0
200
400
600
800
GSBRNA2T00085933001 position (nts)

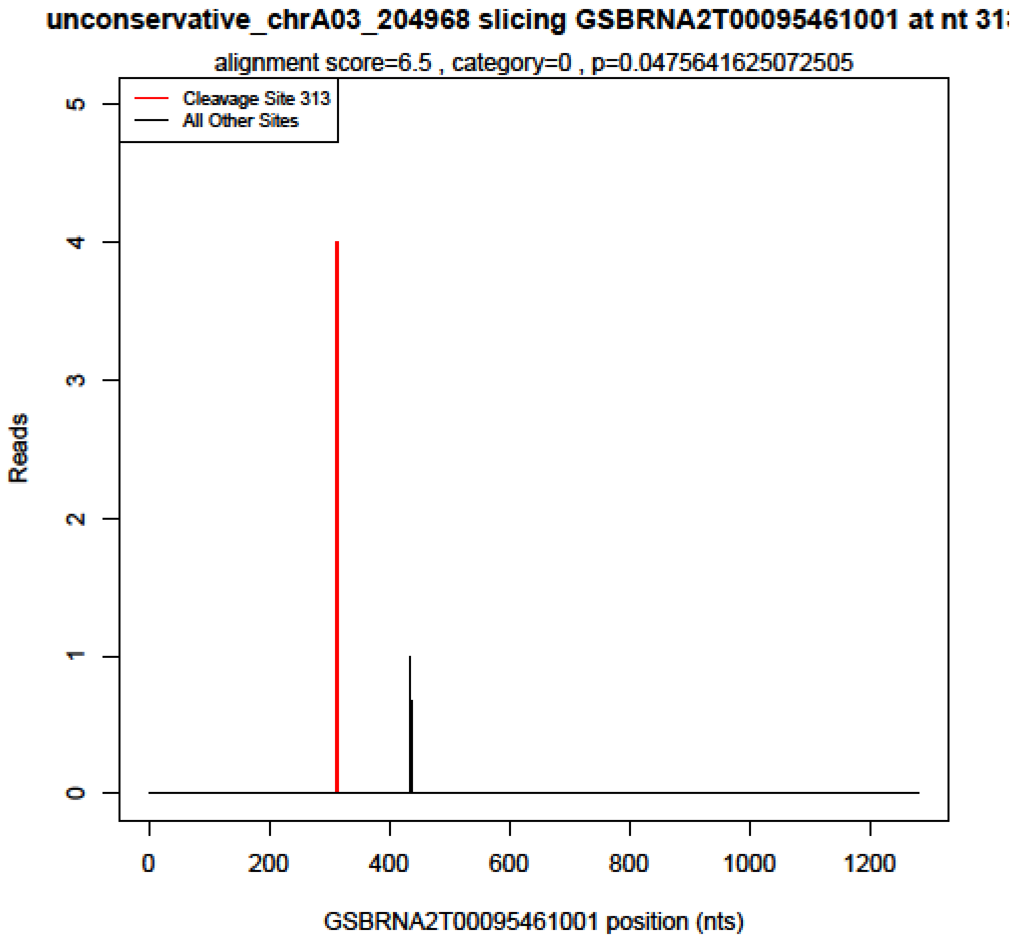

unconservative_chrA03_204968 slicing GSBRNA2T00095461001 at nt 31
alignment score=6.5 , category=0 , p=0.0475641625072505
Cleavage Site 313
All Other Sites
Reads
0
1
2
3
4
5
0
200
400
600
800
1000
1200
GSBRNA2T00095461001 position (nts)

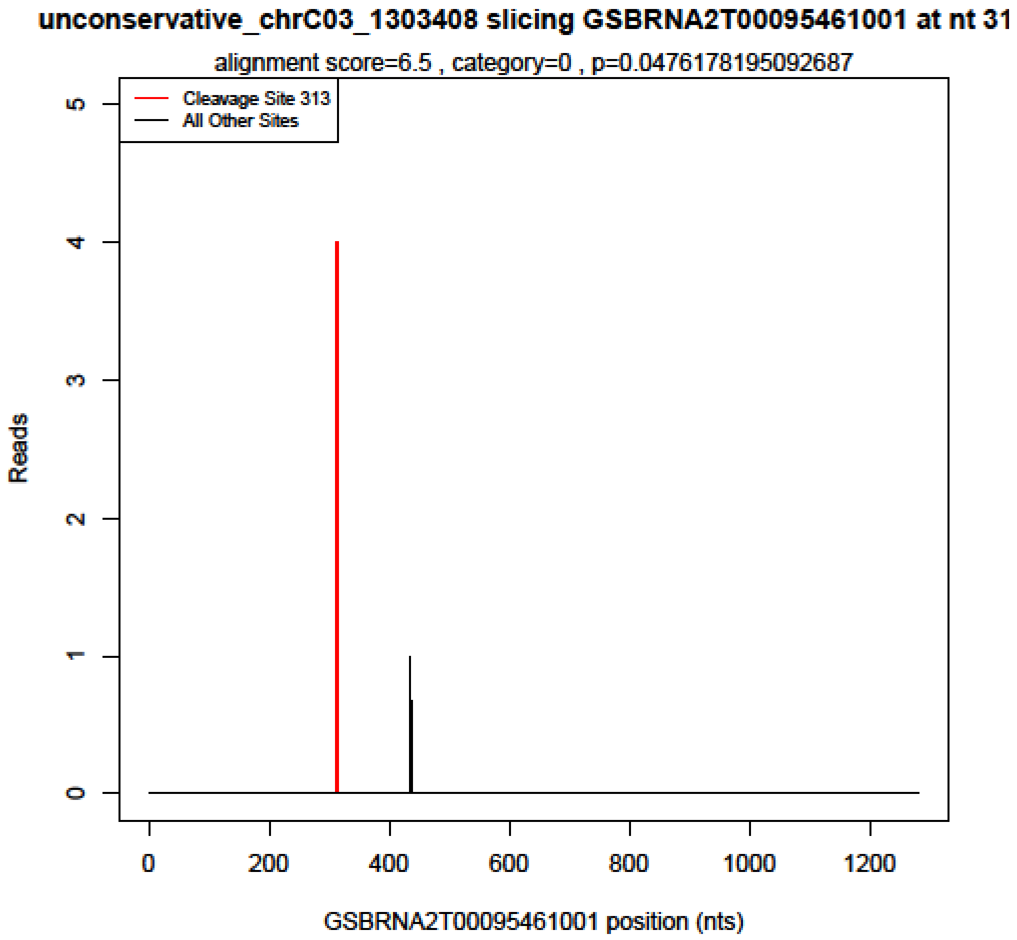
unconservative_chrC03_1303408 slicing GSBRNA2T00095461001 at nt 31
alignment score=6.5 , category=0 , p=0.0476178195092687
Cleavage Site 313
All Other Sites
Reads
0
1
2
3
4
5
0
200
400
600
800
1000
1200
GSBRNA2T00095461001 position (nts)

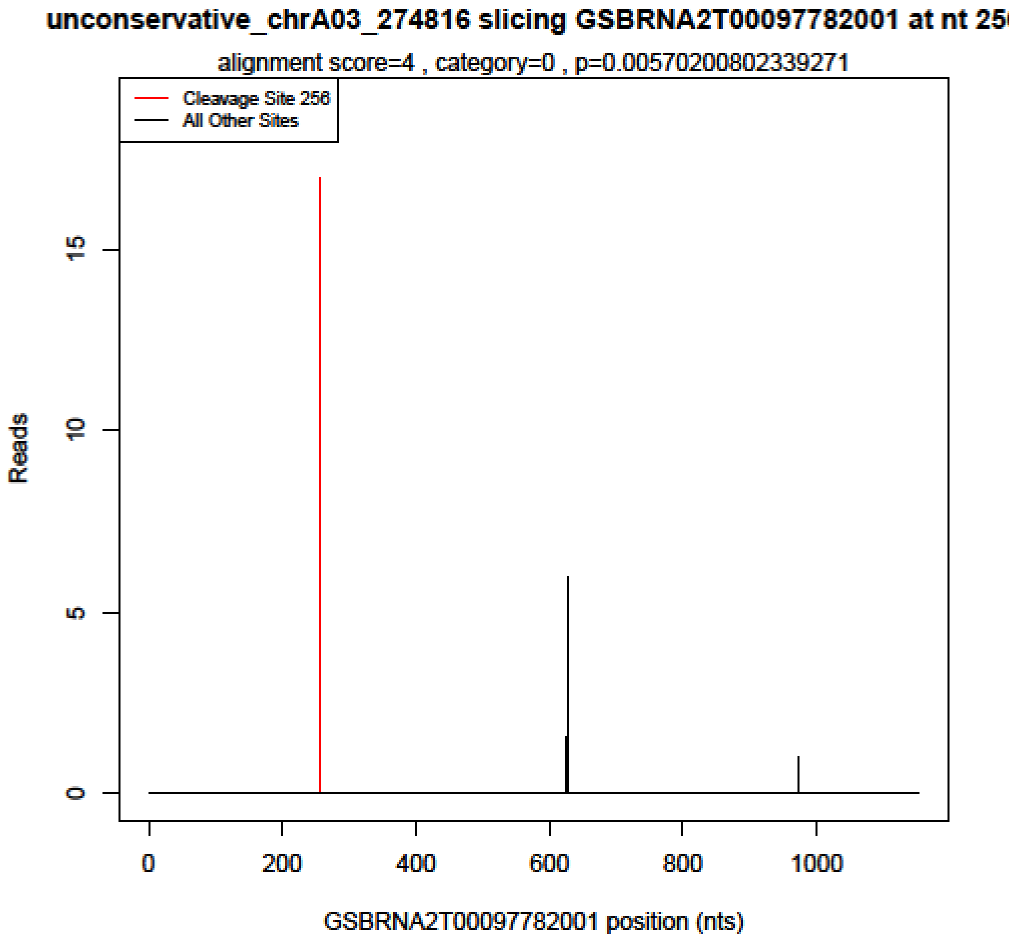

unconservative_chrA03_274816 slicing GSBRNA2T00097782001 at nt 25
alignment score=4 , category=0 , p=0.00570200802339271
Cleavage Site 256
All Other Sites
Reads
0
5
10
15
0
200
400
600
800
1000
GSBRNA2T00097782001 position (nts)

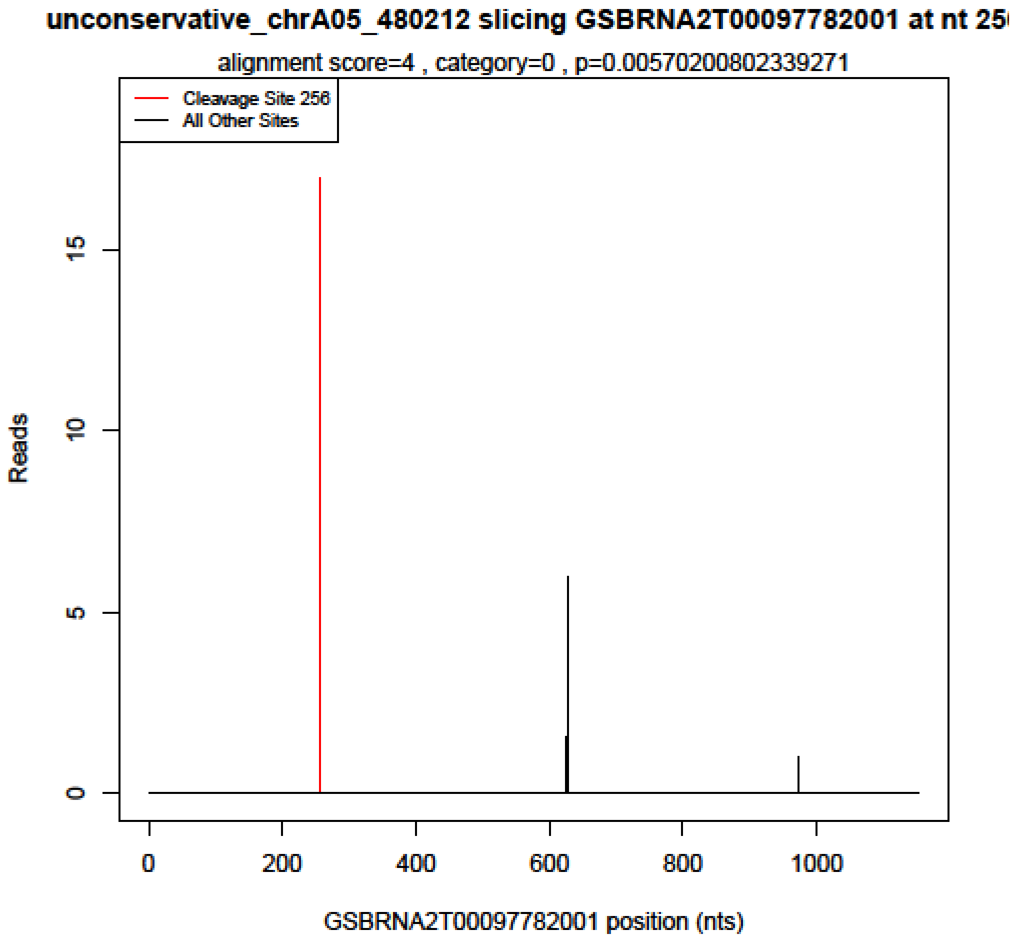

unconservative_chrA05_480212 slicing GSBRNA2T00097782001 at nt 25
alignment score=4 , category=0 , p=0.00570200802339271
Cleavage Site 256
All Other Sites
Reads
0
5
10
15
0
200
400
600
800
1000
GSBRNA2T00097782001 position (nts)

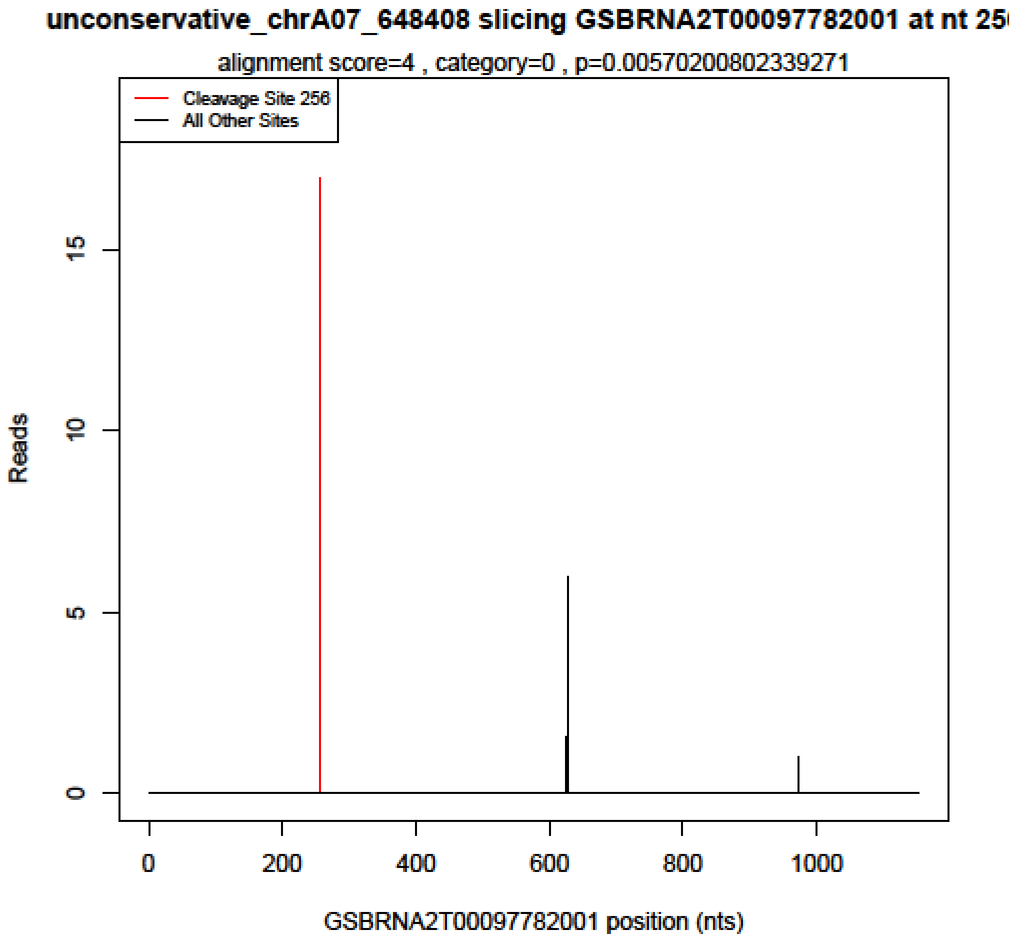
unconservative_chrA07_648408 slicing GSBRNA2T00097782001 at nt 25
alignment score=4 , category=0 , p=0.00570200802339271
Cleavage Site 256
All Other Sites
Reads
0
5
10
15
0
200
400
600
800
1000
GSBRNA2T00097782001 position (nts)

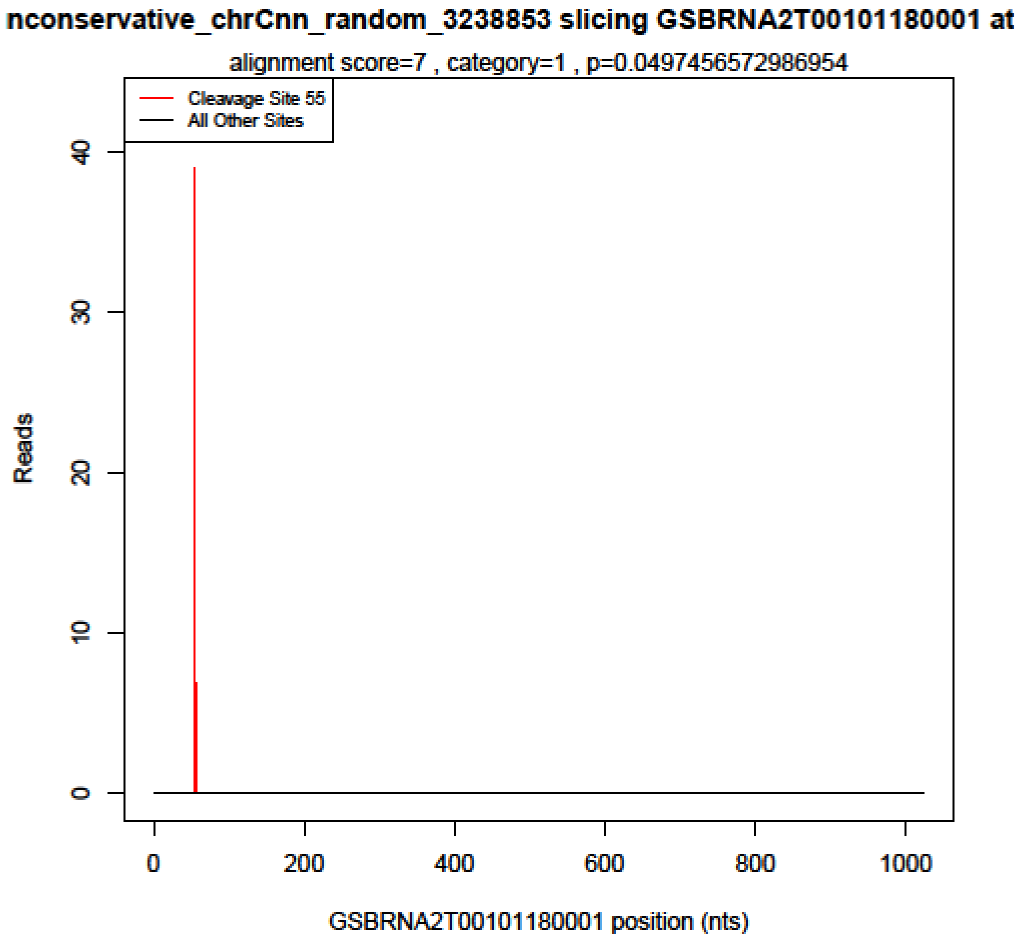

nconservative_chrCnn_random_3238853 slicing GSBRNA2T00101180001 at
alignment score=7 , category=1 , p=0.0497456572986954
Cleavage Site 55
All Other Sites
Reads
0
10
20
30
40
0
200
400
600
800
1000
GSBRNA2T00101180001 position (nts)

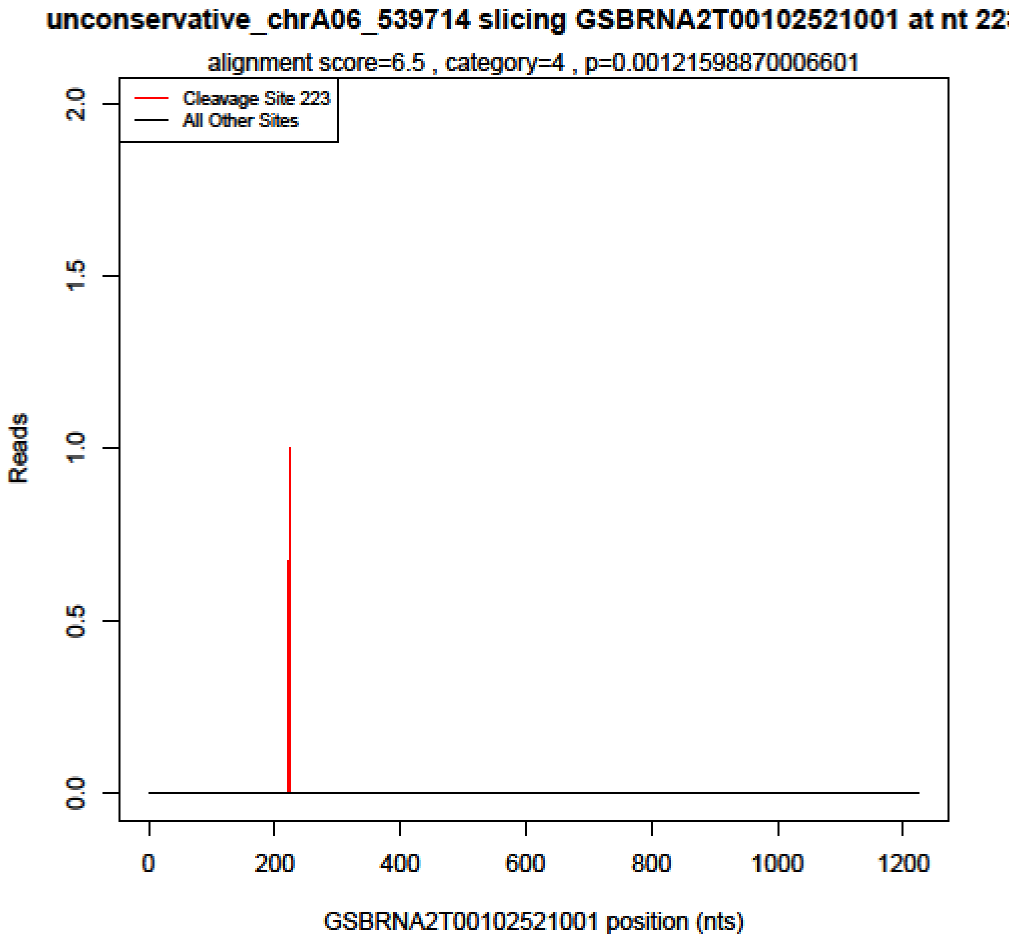

unconservative_chrA06_539714 slicing GSBRNA2T00102521001 at nt 22
alignment score=6.5 , category=4 , p=0.00121598870006601
Cleavage Site 223
All Other Sites
Reads
0.0
0.5
1.0
1.5
2.0
0
200
400
600
800
1000
1200
GSBRNA2T00102521001 position (nts)

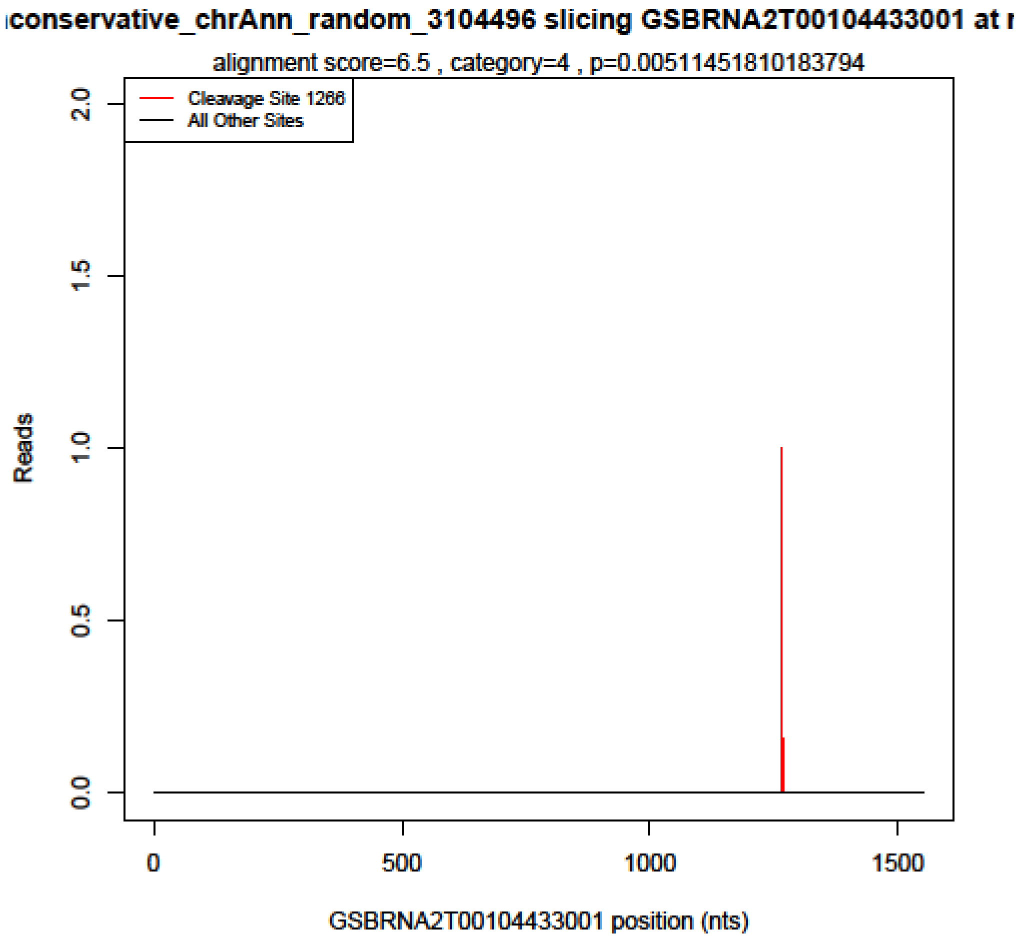

conservative_chrAnn_random_3104496 slicing GSBRNA2T00104433001 at
alignment score=6.5 , category=4 , p=0.00511451810183794
Cleavage Site 1266
All Other Sites
Reads
0.0
0.5
1.0
1.5
2.0
0
500
1000
1500
GSBRNA2T00104433001 position (nts)

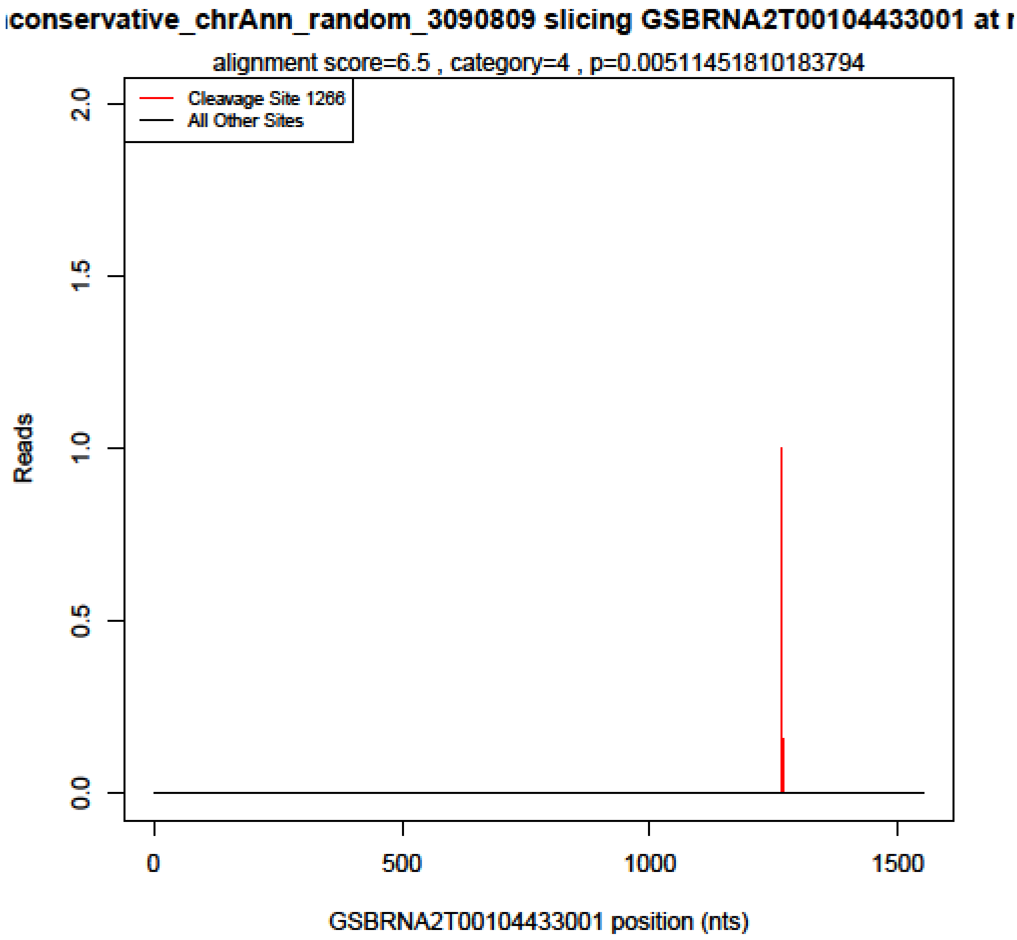

conservative_chrAnn_random_3090809 slicing GSBRNA2T00104433001 at r
alignment score=6.5 , category=4 , p=0.00511451810183794
Cleavage Site 1266
All Other Sites
Reads
0.0
0.5
1.0
1.5
2.0
0
500
1000
1500
GSBRNA2T00104433001 position (nts)

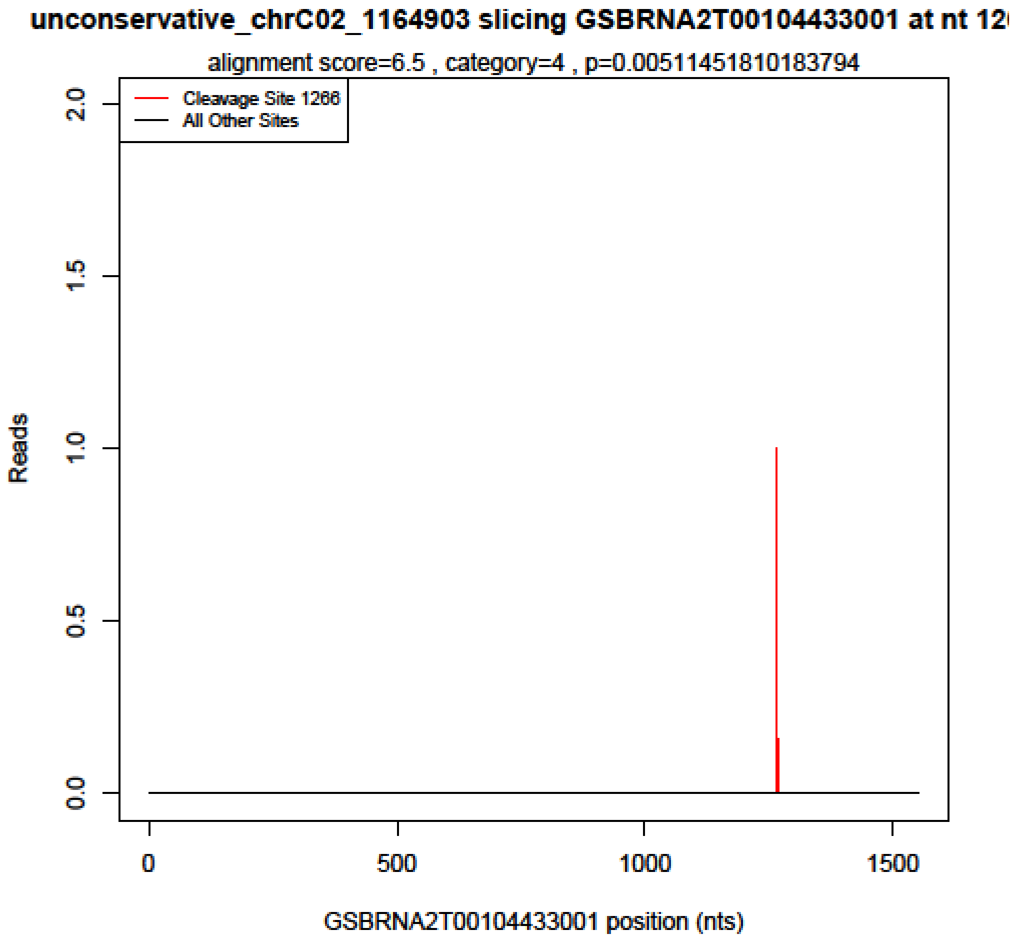
unconservative_chrC02_1164903 slicing GSBRNA2T00104433001 at nt 12
alignment score=6.5 , category=4 , p=0.00511451810183794
Cleavage Site 1266
All Other Sites
Reads
0.0
0.5
1.0
1.5
2.0
0
500
1000
1500
GSBRNA2T00104433001 position (nts)

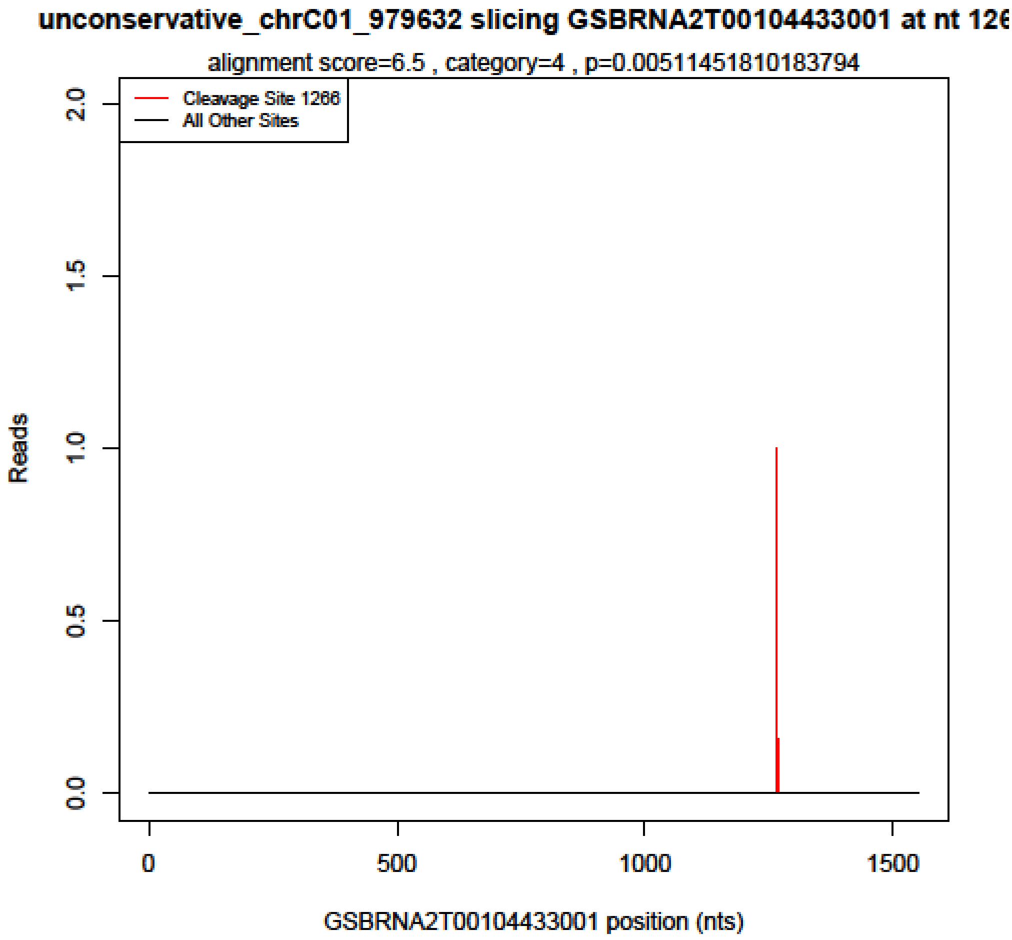

unconservative_chrC01_979632 slicing GSBRNA2T00104433001 at nt 126
alignment score=6.5 , category=4 , p=0.00511451810183794
Cleavage Site 1266
All Other Sites
Reads
0.0
0.5
1.0
1.5
2.0
0
500
1000
1500
GSBRNA2T00104433001 position (nts)

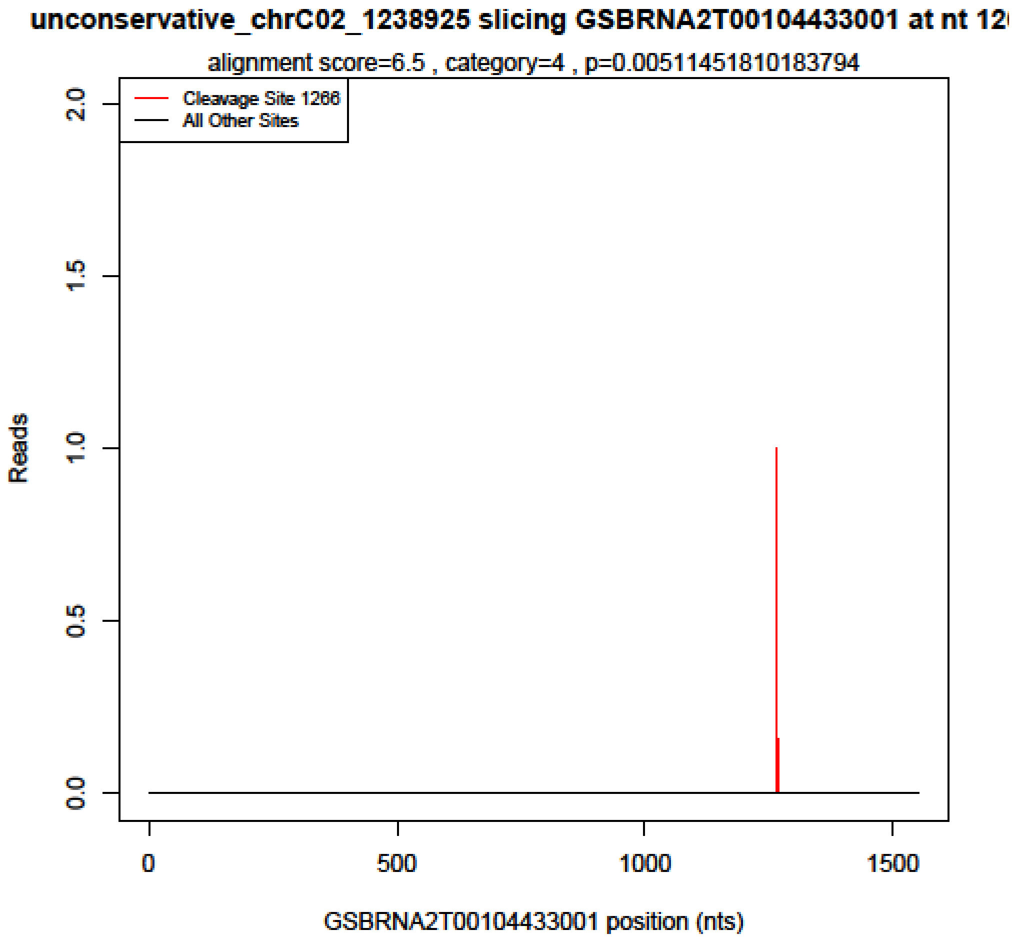

unconservative_chrC02_1238925 slicing GSBRNA2T00104433001 at nt 12
alignment score=6.5 , category=4 , p=0.00511451810183794
Cleavage Site 1266
All Other Sites
Reads
2.0
1.5
1.0
0.5
0.0
0
500
1000
1500
GSBRNA2T00104433001 position (nts)

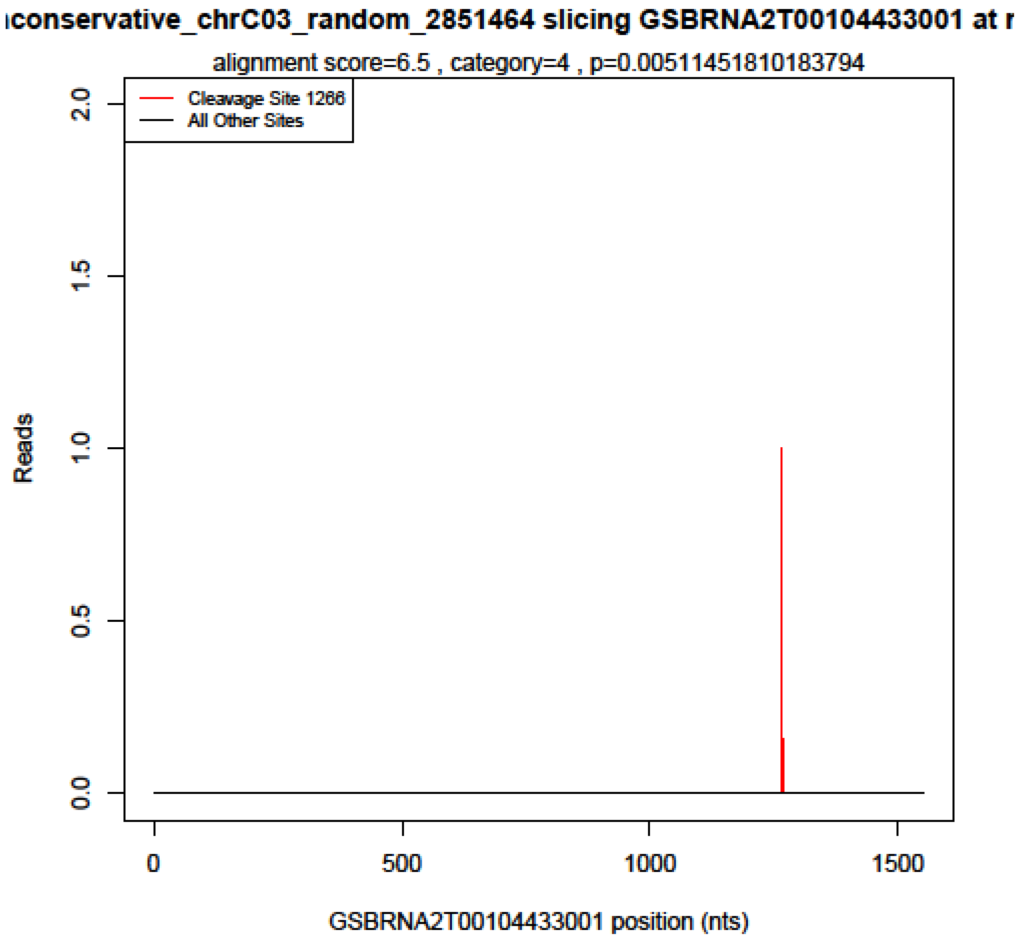
iconservative_chrC03_random_2851464 slicing GSBRNA2T00104433001 at r
alignment score=6.5 , category=4 , p=0.00511451810183794
Cleavage Site 1266
All Other Sites
Reads
0.0
0.5
1.0
1.5
2.0
0
500
1000
1500
GSBRNA2T00104433001 position (nts)

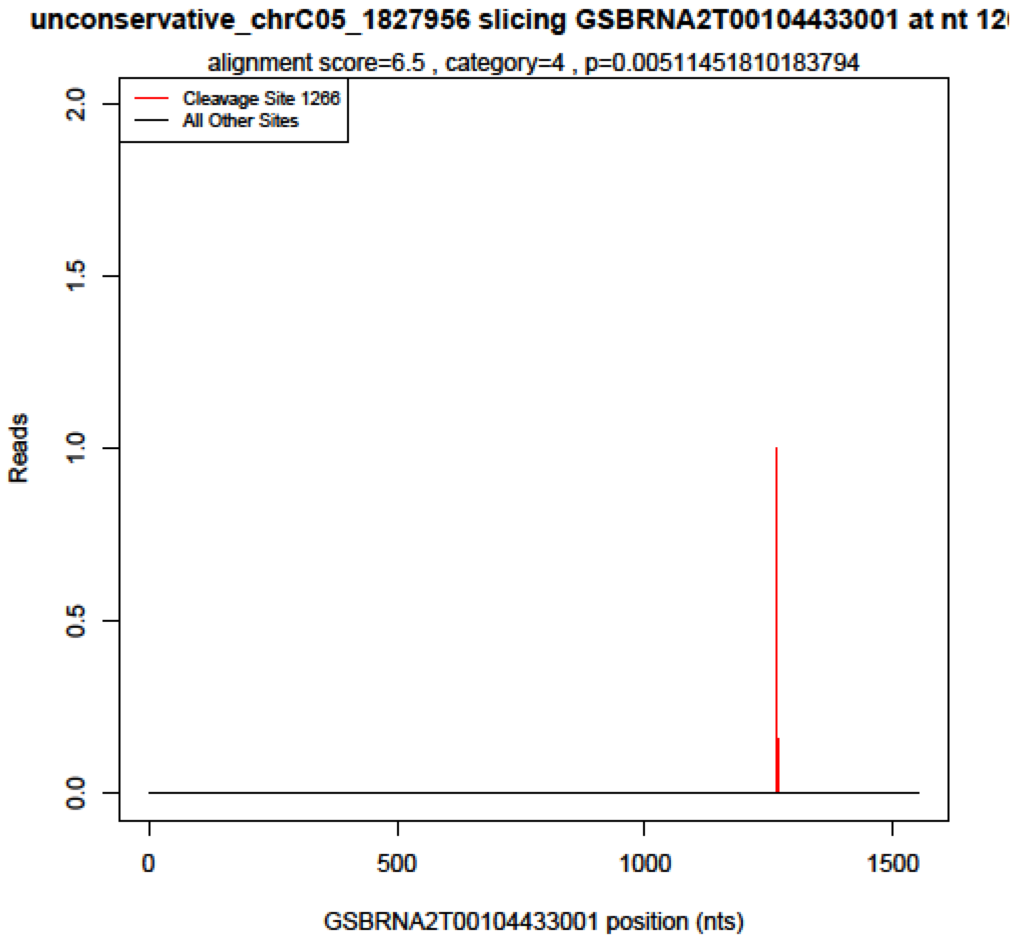
unconservative_chrC05_1827956 slicing GSBRNA2T00104433001 at nt 12
alignment score=6.5 , category=4 , p=0.00511451810183794
Cleavage Site 1266
All Other Sites
Reads
0.0
0.5
1.0
1.5
2.0
0
500
1000
1500
GSBRNA2T00104433001 position (nts)

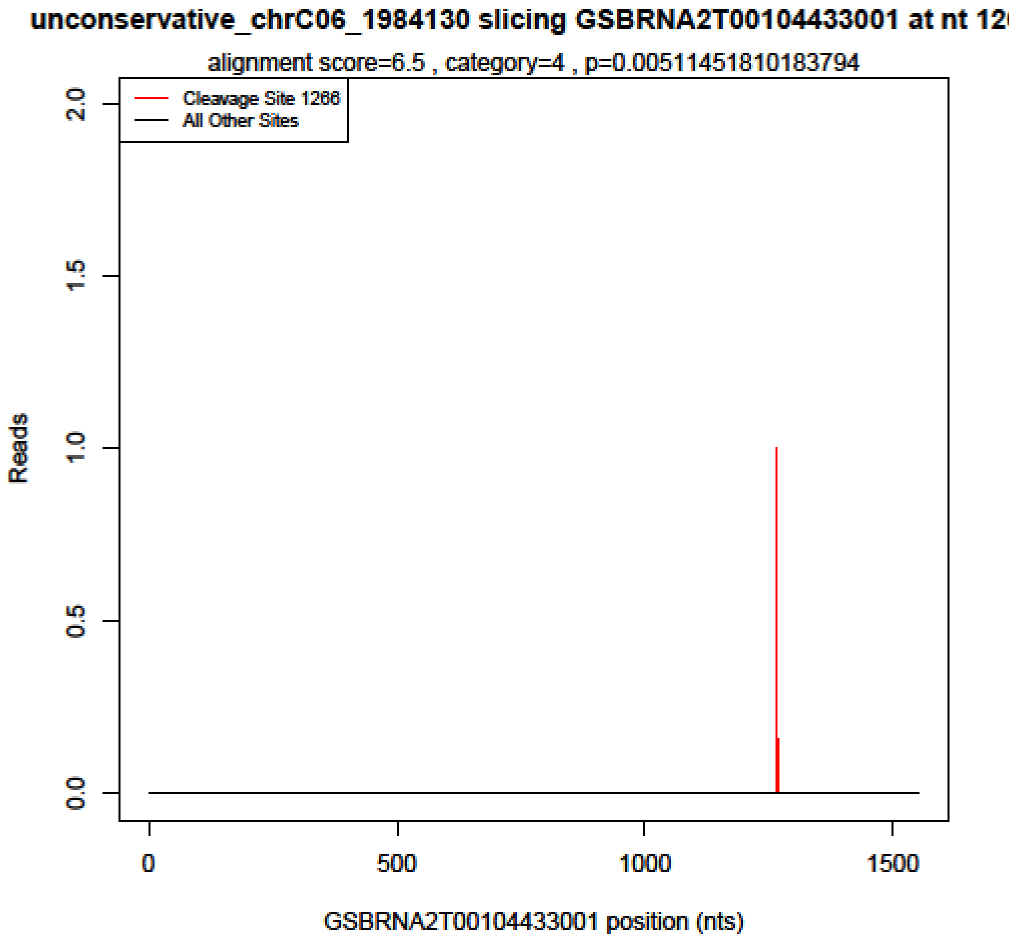

unconservative_chrC06_1984130 slicing GSBRNA2T00104433001 at nt 12
alignment score=6.5 , category=4 , p=0.00511451810183794
Cleavage Site 1266
All Other Sites
Reads
0.0
0.5
1.0
1.5
2.0
0
500
1000
1500
GSBRNA2T00104433001 position (nts)

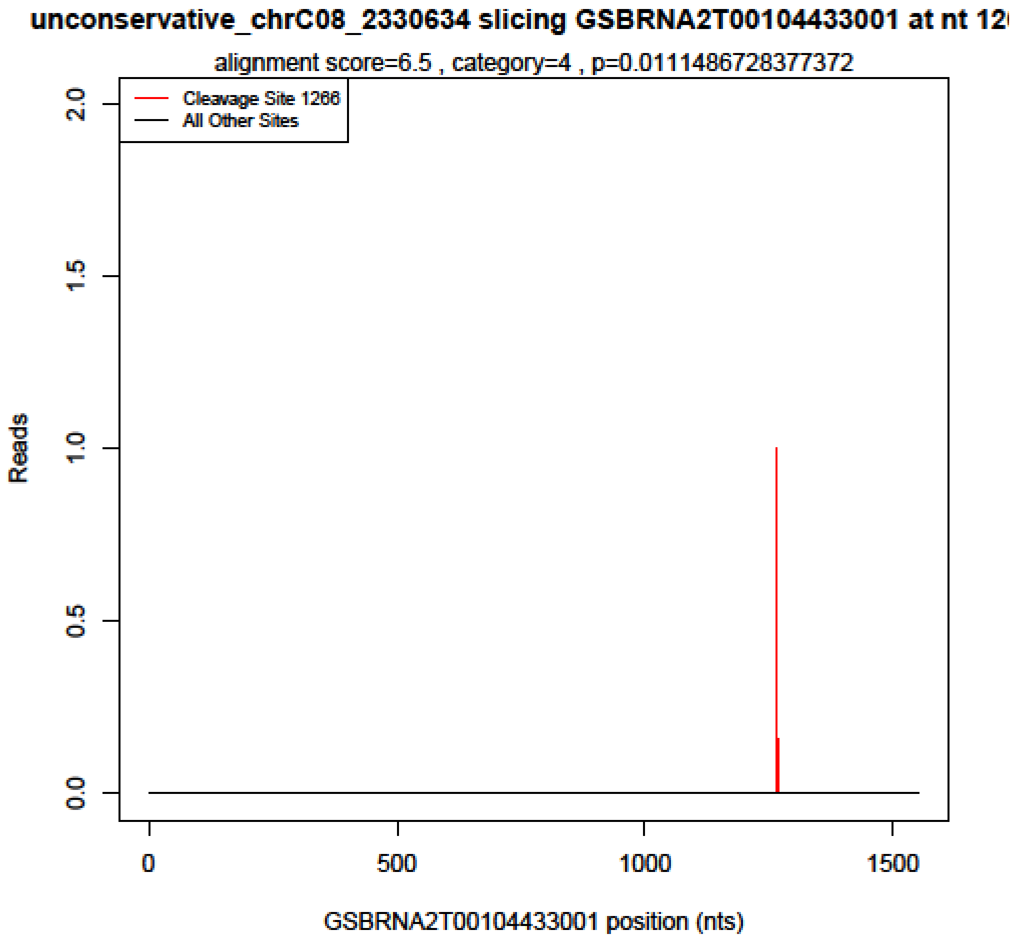
unconservative_chrC08_2330634 slicing GSBRNA2T00104433001 at nt 12
alignment score=6.5 , category=4 , p=0.0111486728377372
Cleavage Site 1266
All Other Sites
Reads
0.0
0.5
1.0
1.5
2.0
0
500
1000
1500
GSBRNA2T00104433001 position (nts)

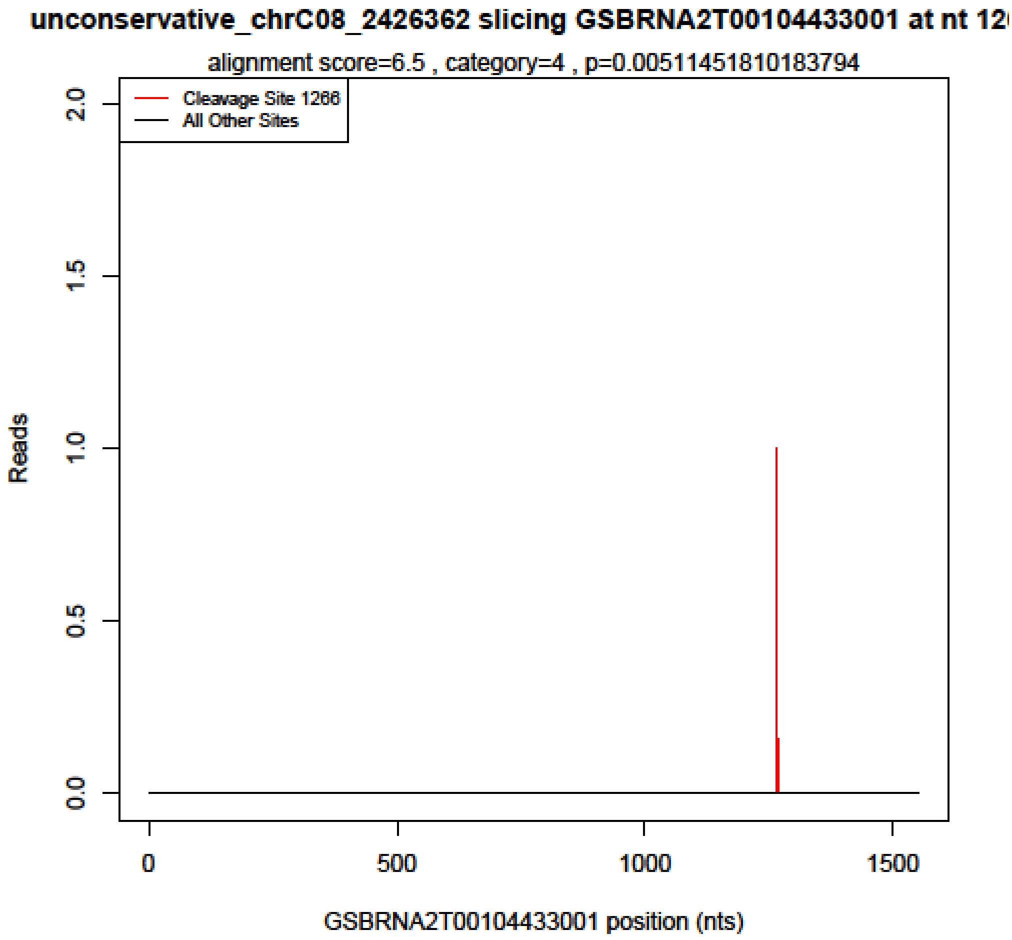
unconservative_chrC08_2426362 slicing GSBRNA2T00104433001 at nt 12
alignment score=6.5 , category=4 , p=0.00511451810183794
Cleavage Site 1266
All Other Sites
Reads
0.0
0.5
1.0
1.5
2.0
0
500
1000
1500
GSBRNA2T00104433001 position (nts)

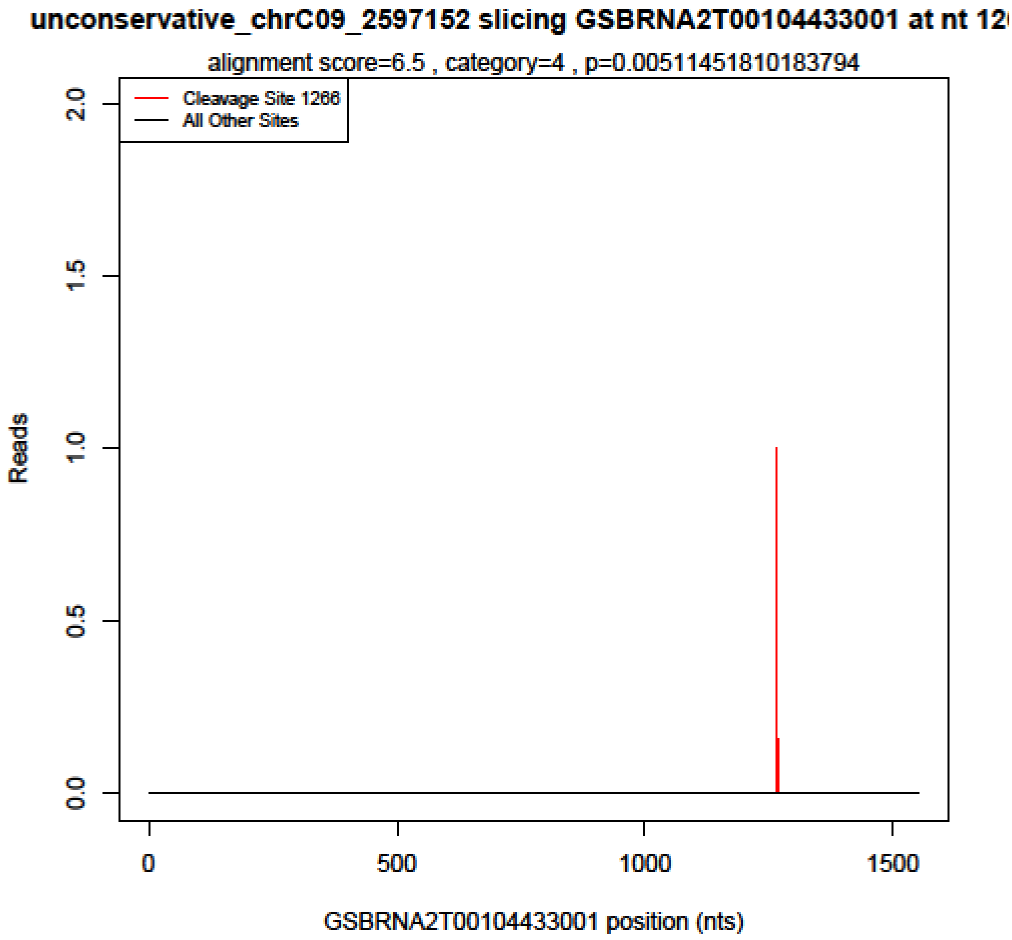

unconservative_chrC09_2597152 slicing GSBRNA2T00104433001 at nt 12
alignment score=6.5 , category=4 , p=0.00511451810183794
Cleavage Site 1266
All Other Sites
Reads
2.0
1.5
1.0
0.5
0.0
0
500
1000
1500
GSBRNA2T00104433001 position (nts)

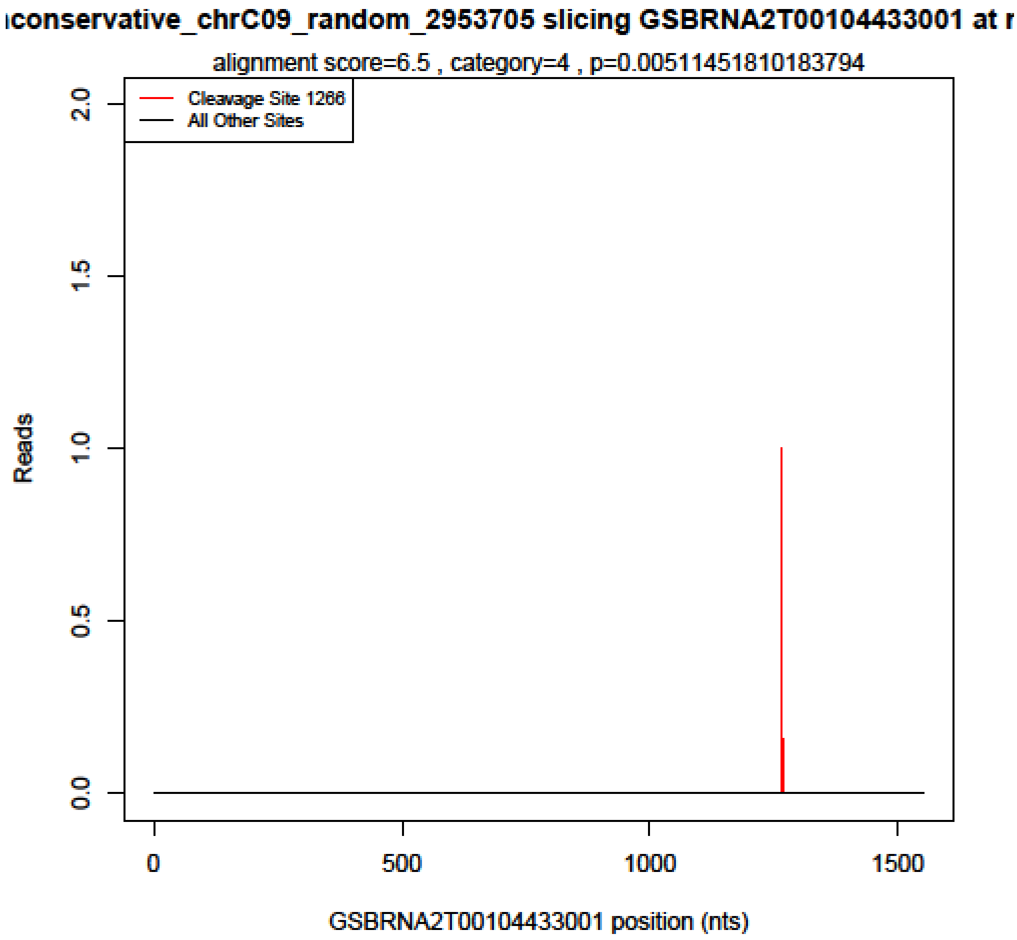

iconservative_chrC09_random_2953705 slicing GSBRNA2T00104433001 at r
alignment score=6.5 , category=4 , p=0.00511451810183794
Cleavage Site 1266
All Other Sites
Reads
0.0
0.5
1.0
1.5
2.0
0
500
1000
1500
GSBRNA2T00104433001 position (nts)

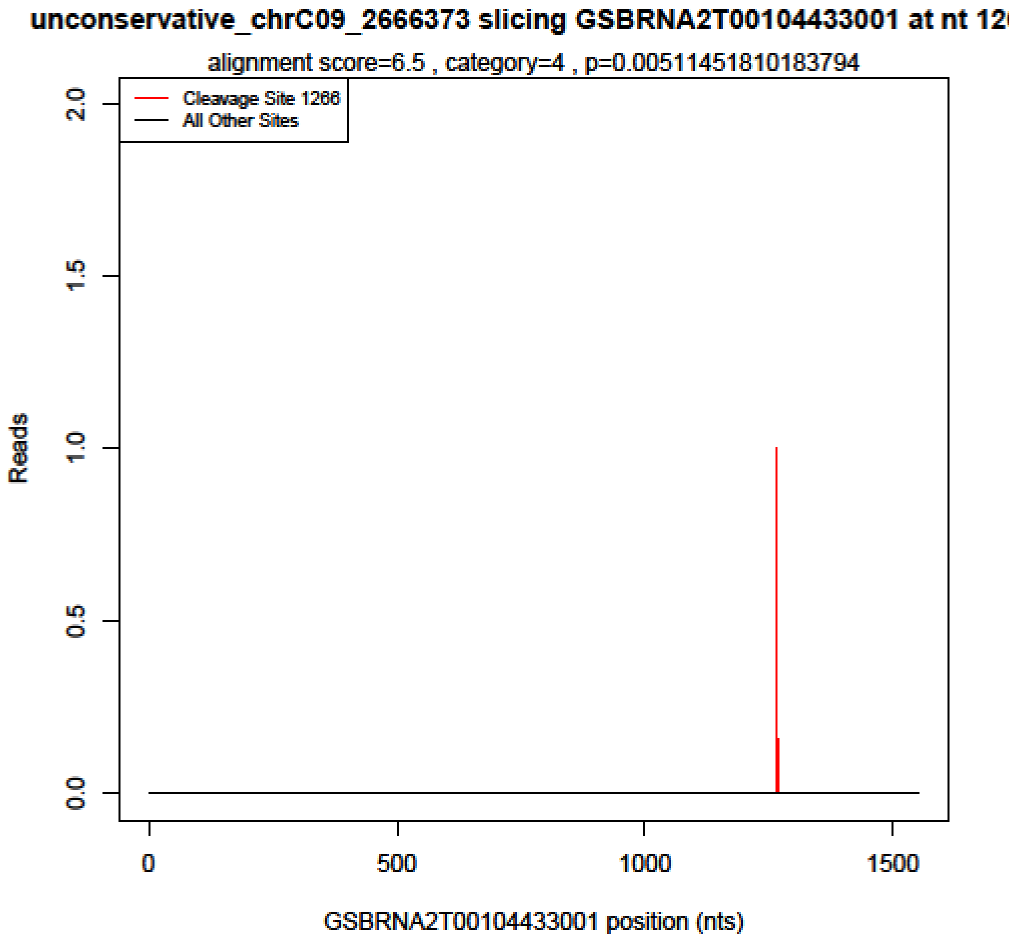

unconservative_chrC09_2666373 slicing GSBRNA2T00104433001 at nt 12
alignment score=6.5 , category=4 , p=0.00511451810183794
Cleavage Site 1266
All Other Sites
Reads
0.0
0.5
1.0
1.5
2.0
0
500
1000
1500
GSBRNA2T00104433001 position (nts)

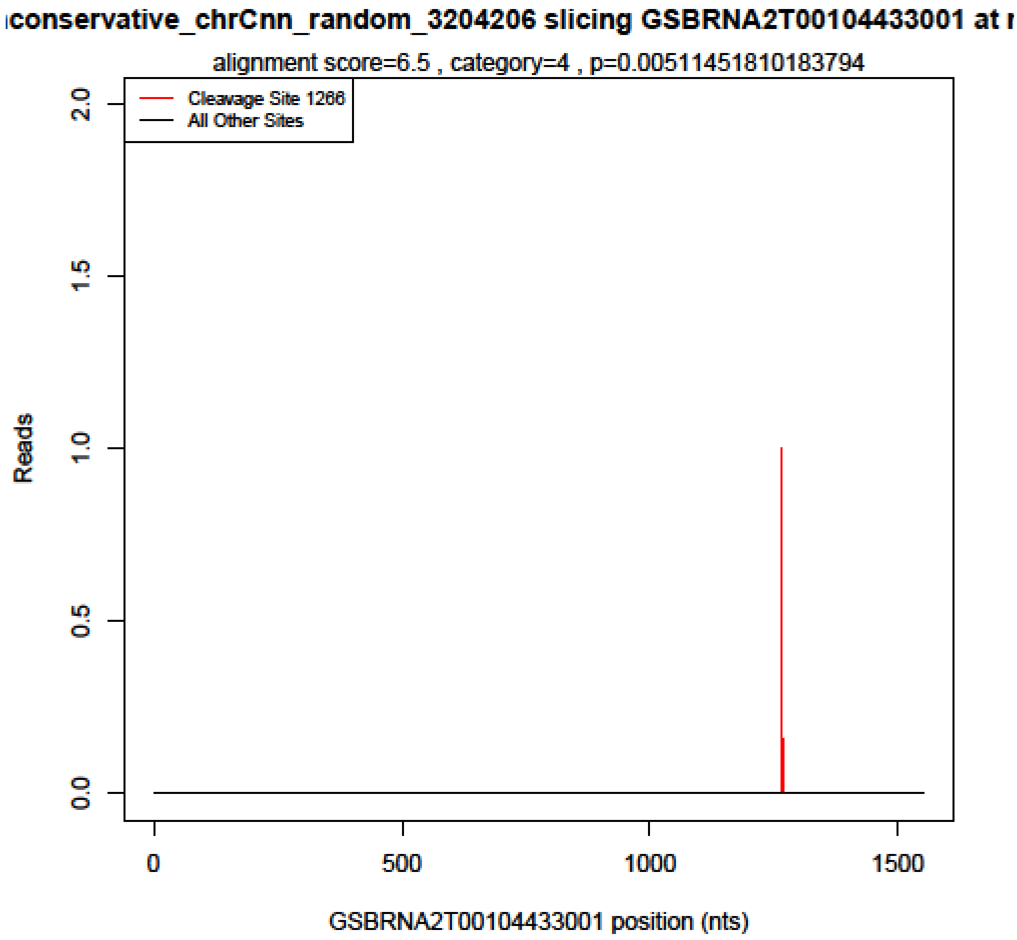

conservative_chrCnn_random_3204206 slicing GSBRNA2T00104433001 at
alignment score=6.5 , category=4 , p=0.00511451810183794
Cleavage Site 1266
All Other Sites
Reads
0.0
0.5
1.0
1.5
2.0
0
500
1000
1500
GSBRNA2T00104433001 position (nts)

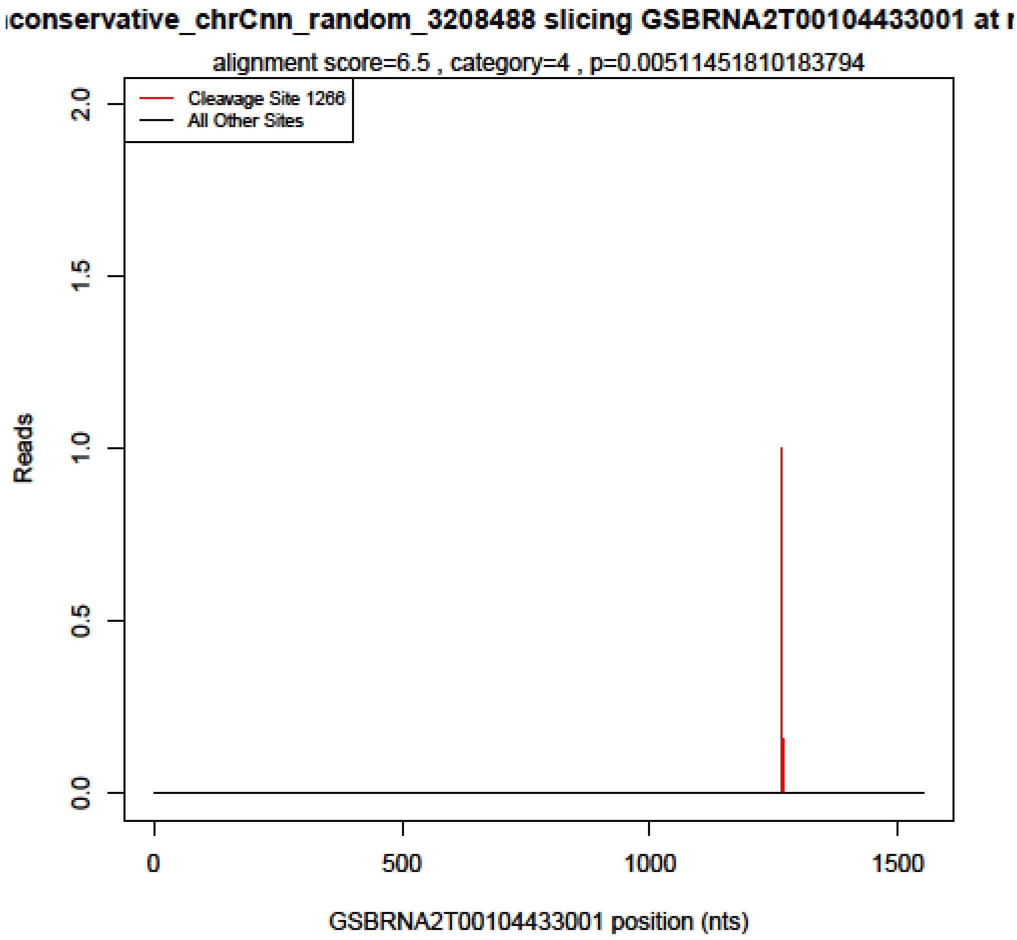

conservative_chrCnn_random_3208488 slicing GSBRNA2T00104433001 at
alignment score=6.5 , category=4 , p=0.00511451810183794
Cleavage Site 1266
All Other Sites
Reads
0.0
0.5
1.0
1.5
2.0
0
500
1000
1500
GSBRNA2T00104433001 position (nts)

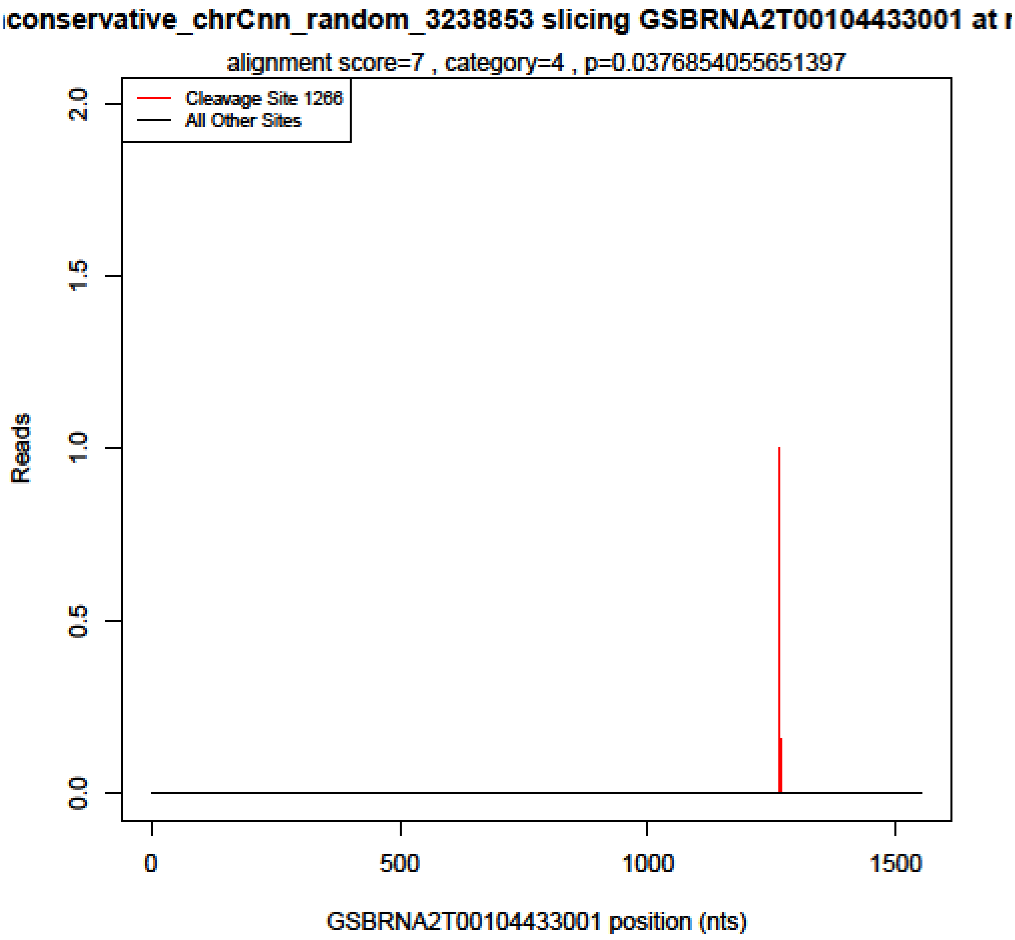

conservative_chrCnn_random_3238853 slicing GSBRNA2T00104433001 at r
alignment score=7 , category=4 , p=0.0376854055651397
Cleavage Site 1266
All Other Sites
Reads
0.0
0.5
1.0
1.5
2.0
0
500
1000
1500
GSBRNA2T00104433001 position (nts)

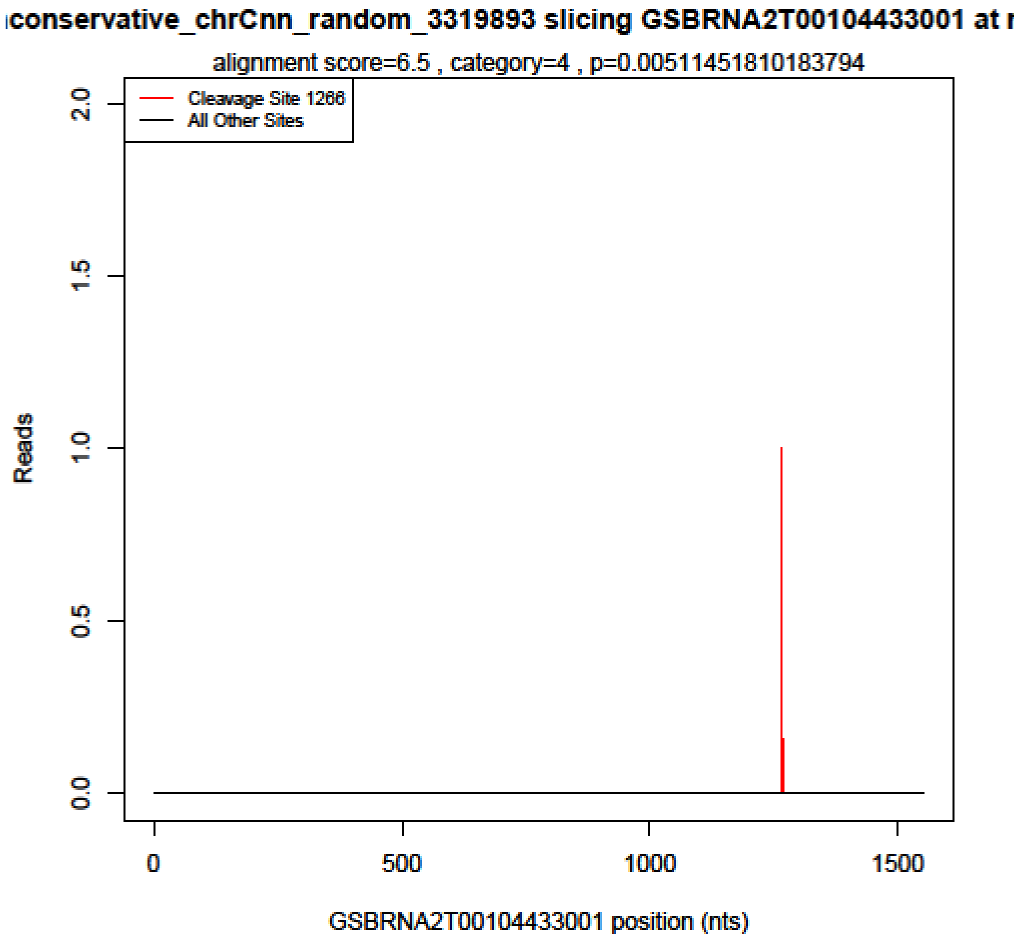

conservative_chrCnn_random_3319893 slicing GSBRNA2T00104433001 at
alignment score=6.5 , category=4 , p=0.00511451810183794
Cleavage Site 1266
All Other Sites
Reads
0.0
0.5
1.0
1.5
2.0
0
500
1000
1500
GSBRNA2T00104433001 position (nts)

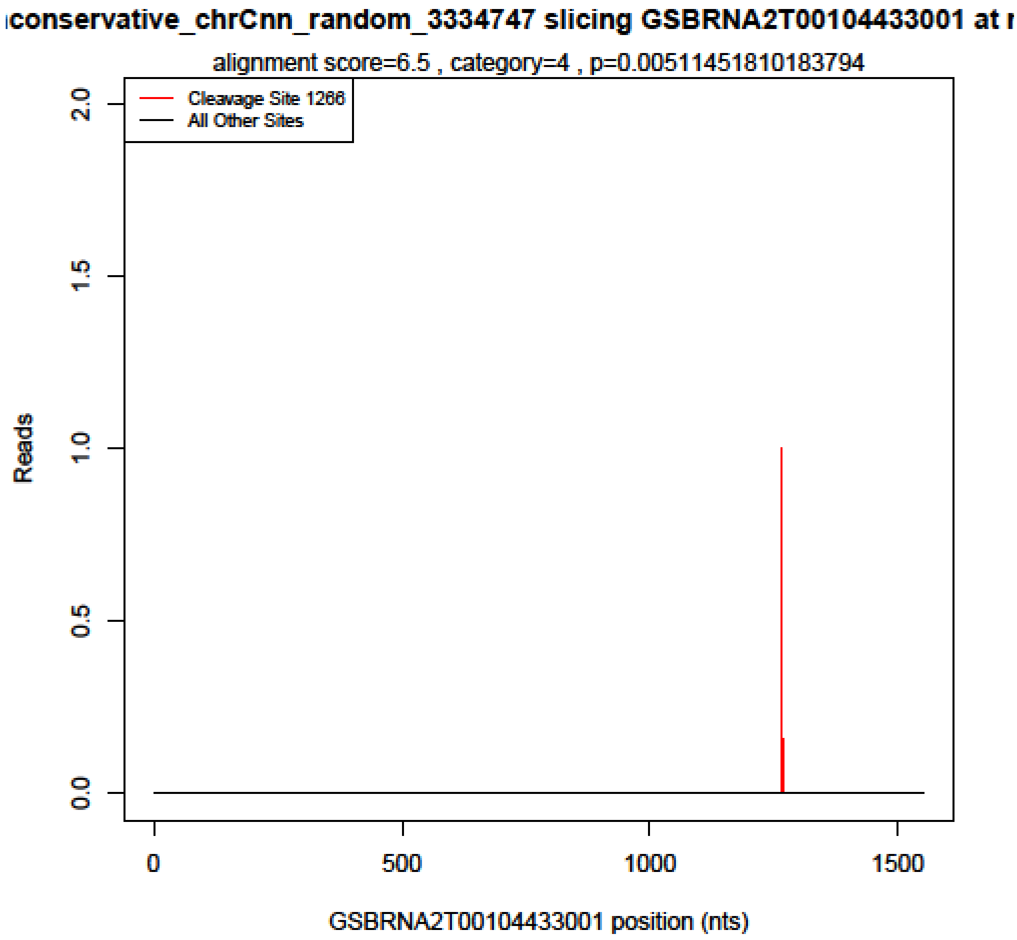

conservative_chrCnn_random_3334747 slicing GSBRNA2T00104433001 at r
alignment score=6.5 , category=4 , p=0.00511451810183794
Cleavage Site 1266
All Other Sites
Reads
0.0
0.5
1.0
1.5
2.0
0
500
1000
1500
GSBRNA2T00104433001 position (nts)

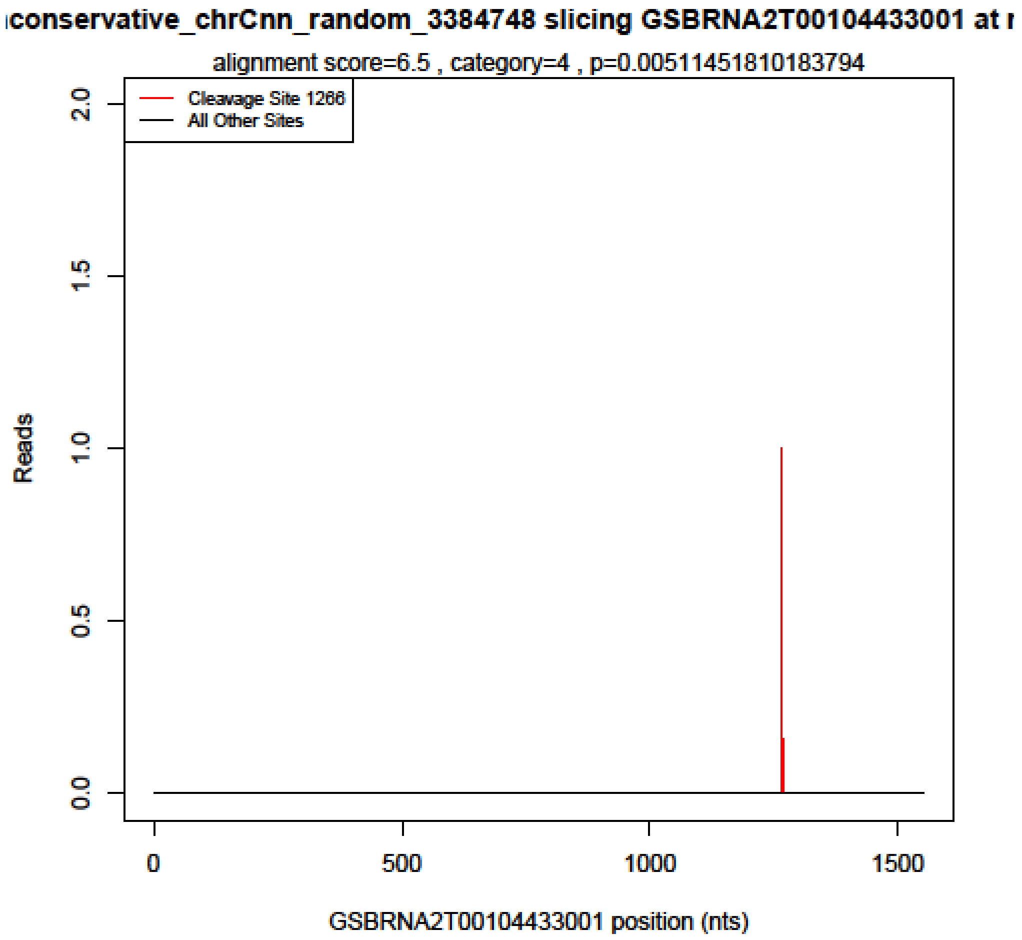
conservative_chrCnn_random_3384748 slicing GSBRNA2T00104433001 at
alignment score=6.5 , category=4 , p=0.00511451810183794
Cleavage Site 1266
All Other Sites
Reads
0.0
0.5
1.0
1.5
2.0
0
500
1000
1500
GSBRNA2T00104433001 position (nts)

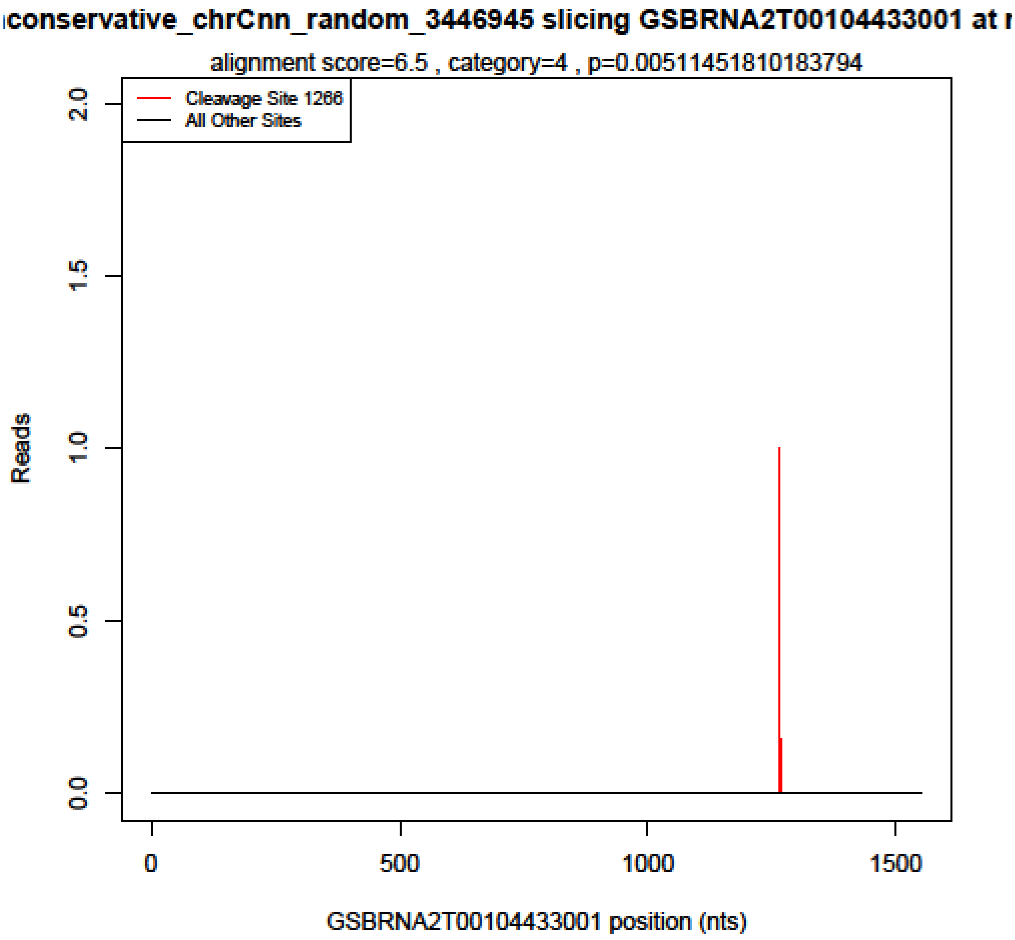

conservative_chrCnn_random_3446945 slicing GSBRNA2T00104433001 at r
alignment score=6.5 , category=4 , p=0.00511451810183794
Cleavage Site 1266
All Other Sites
Reads
0.0
0.5
1.0
1.5
2.0
0
500
1000
1500
GSBRNA2T00104433001 position (nts)

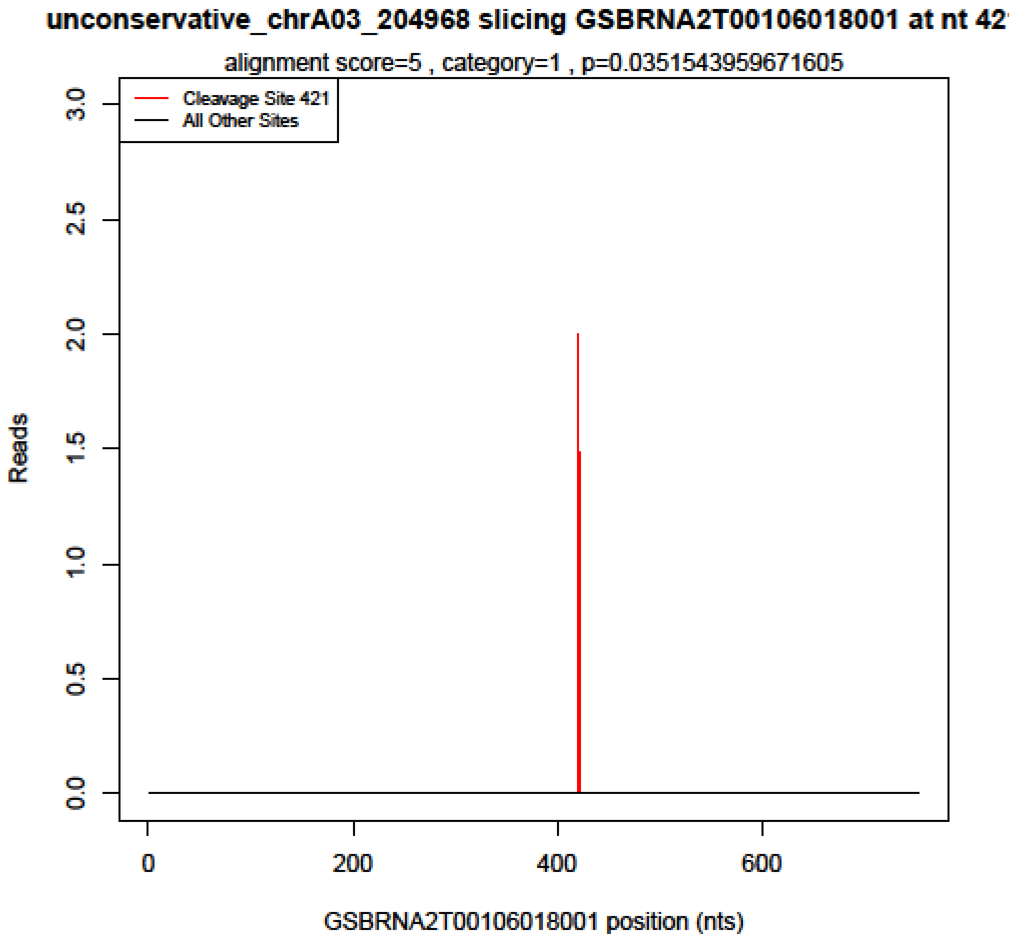

unconservative_chrA03_204968 slicing GSBRNA2T00106018001 at nt 42
alignment score=5 , category=1 , p=0.0351543959671605
Cleavage Site 421
All Other Sites
Reads
0.0
0.5
1.0
1.5
2.0
2.5
3.0
0
200
400
600
GSBRNA2T00106018001 position (nts)

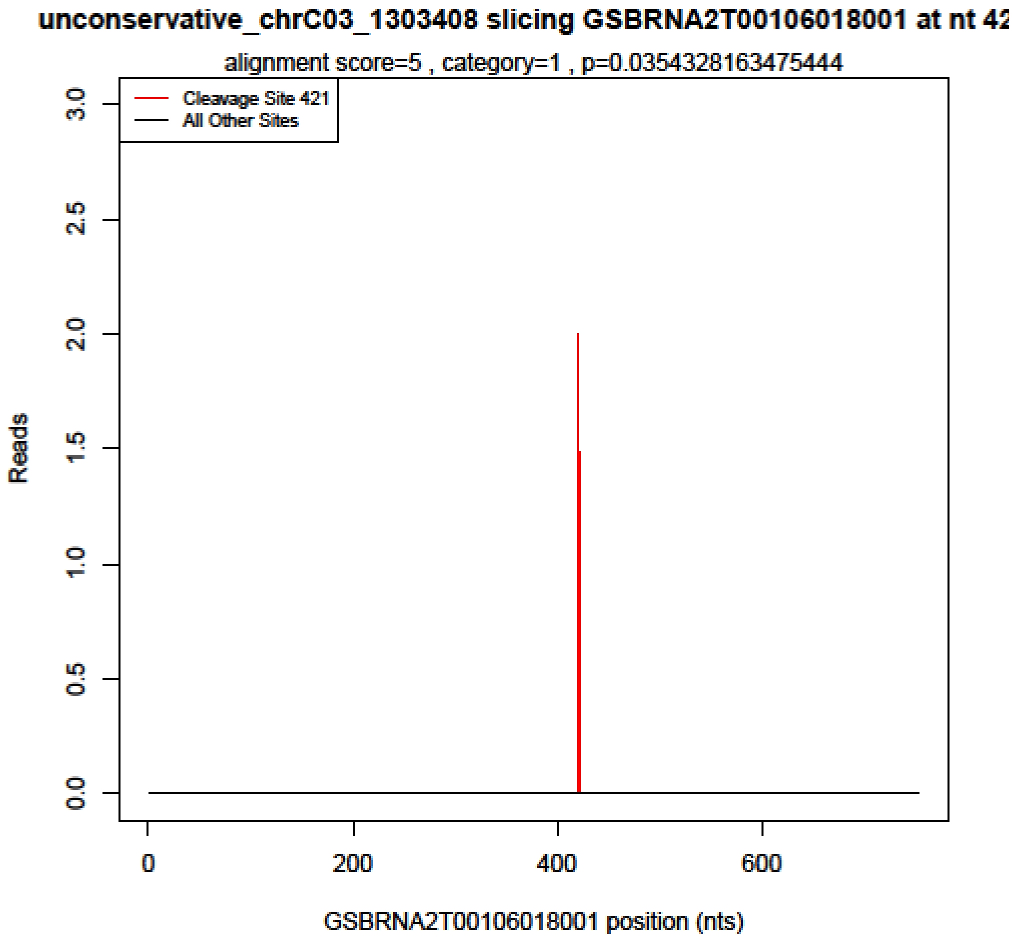

unconservative_chrC03_1303408 slicing GSBRNA2T00106018001 at nt 42
alignment score=5 , category=1 , p=0.0354328163475444
Cleavage Site 421
All Other Sites
Reads
0.0
0.5
1.0
1.5
2.0
2.5
3.0
0
200
400
600
GSBRNA2T00106018001 position (nts)

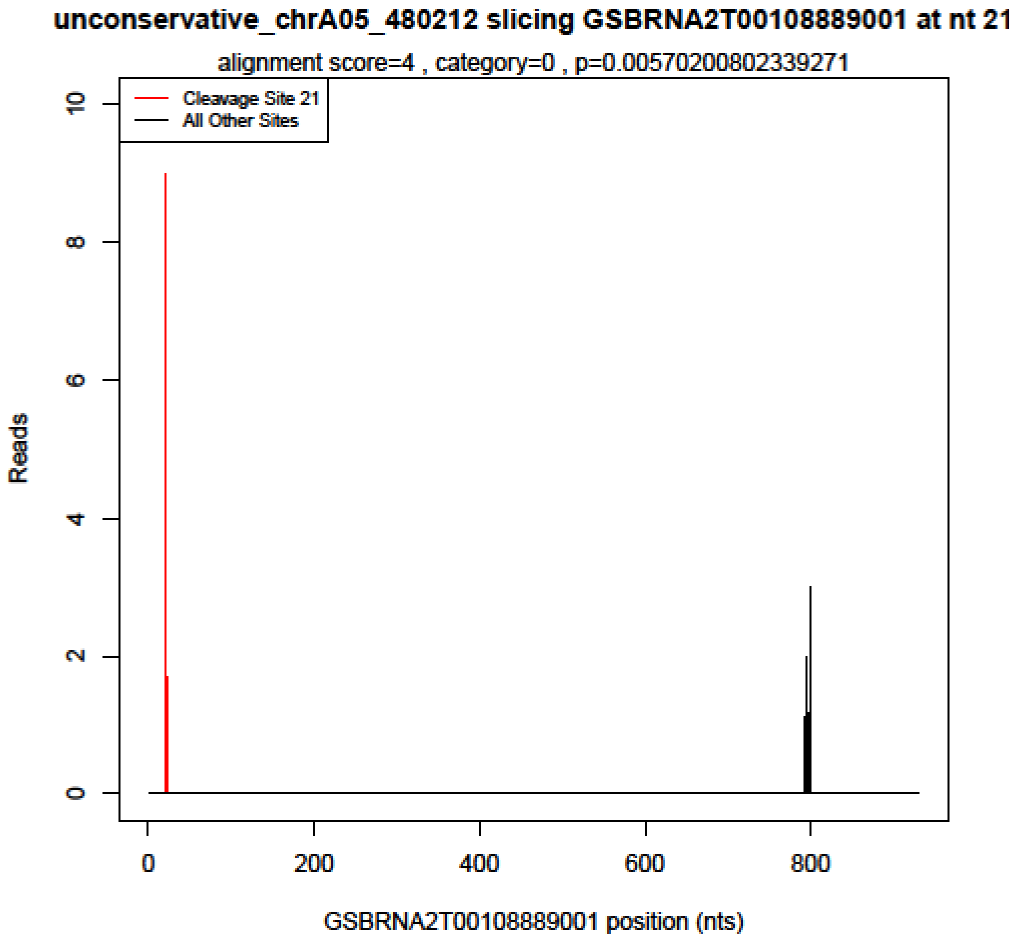

unconservative_chrA05_480212 slicing GSBRNA2T00108889001 at nt 21
alignment score=4 , category=0 , p=0.00570200802339271
Cleavage Site 21
All Other Sites
Reads
0
2
4
6
8
10
0
200
400
600
800
GSBRNA2T00108889001 position (nts)

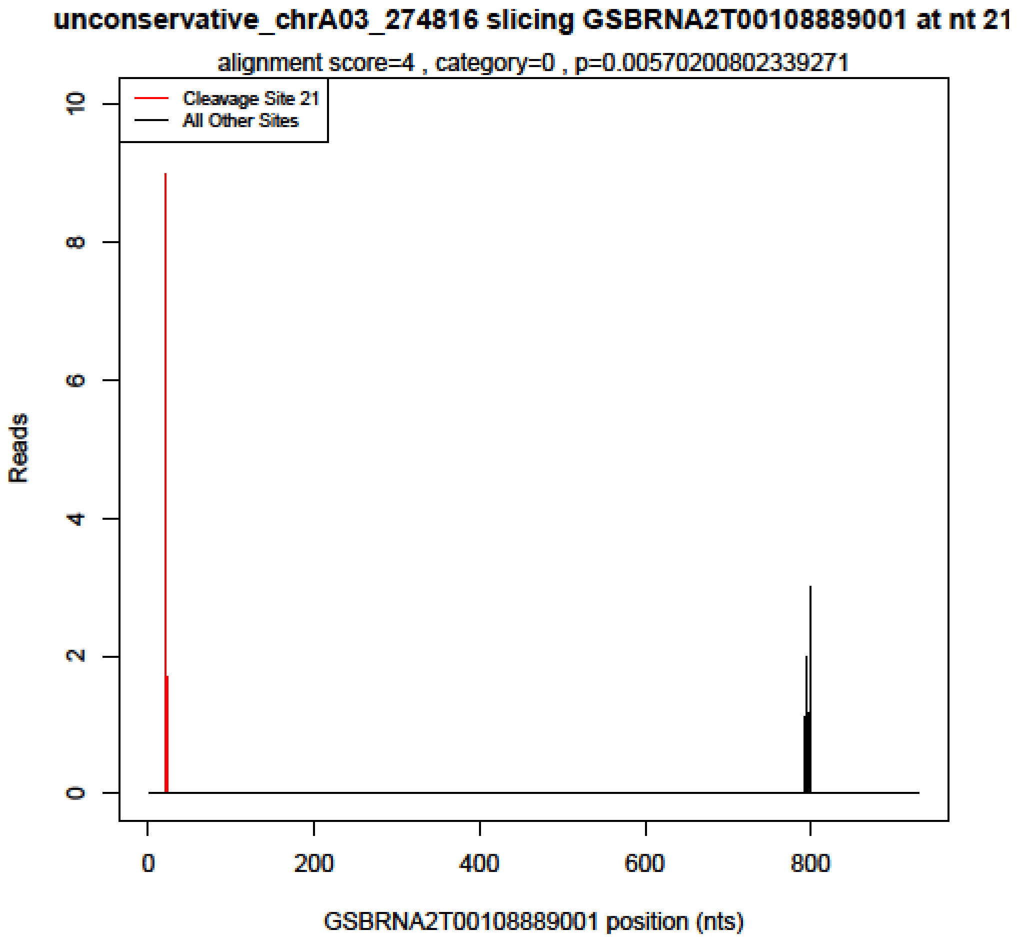

unconservative_chrA03_274816 slicing GSBRNA2T00108889001 at nt 21
alignment score=4 , category=0 , p=0.00570200802339271
Cleavage Site 21
All Other Sites
Reads
0
2
4
6
8
10
0
200
400
600
800
GSBRNA2T00108889001 position (nts)

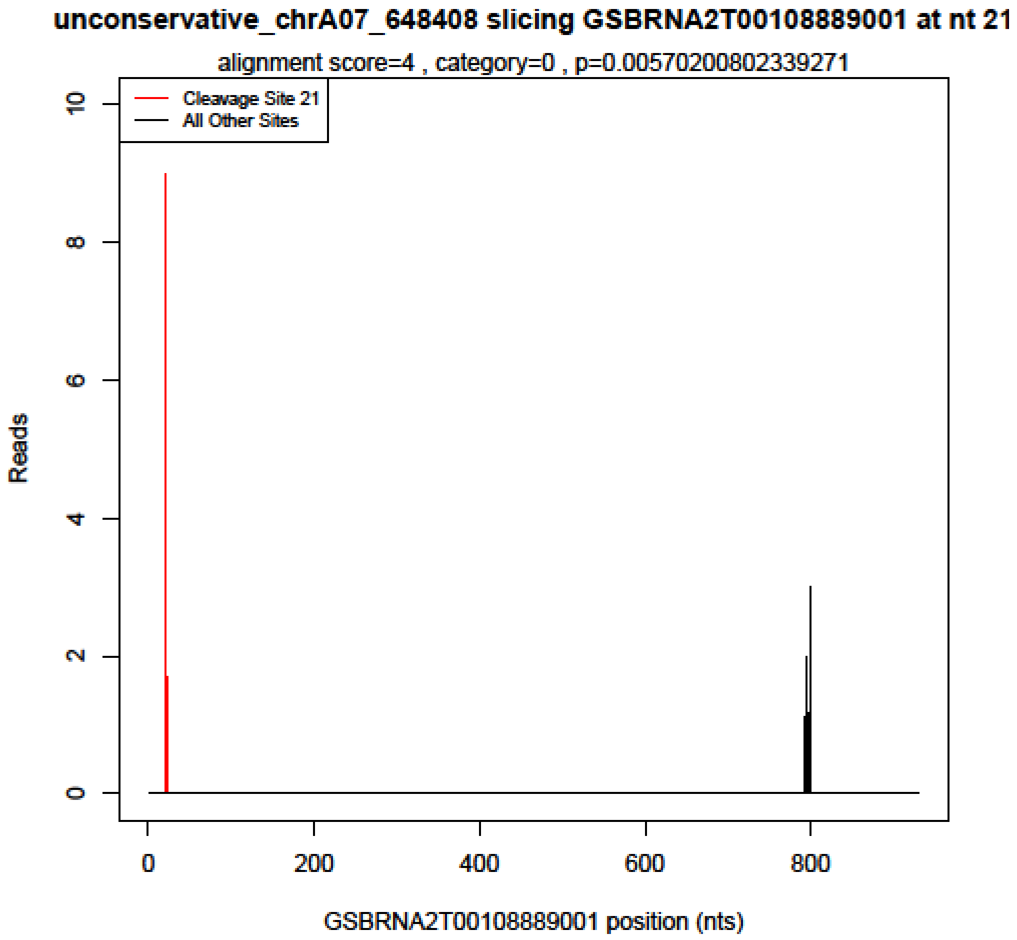

unconservative_chrA07_648408 slicing GSBRNA2T00108889001 at nt 21
alignment score=4 , category=0 , p=0.00570200802339271
Cleavage Site 21
All Other Sites
Reads
0
2
4
6
8
10
0
200
400
600
800
GSBRNA2T00108889001 position (nts)

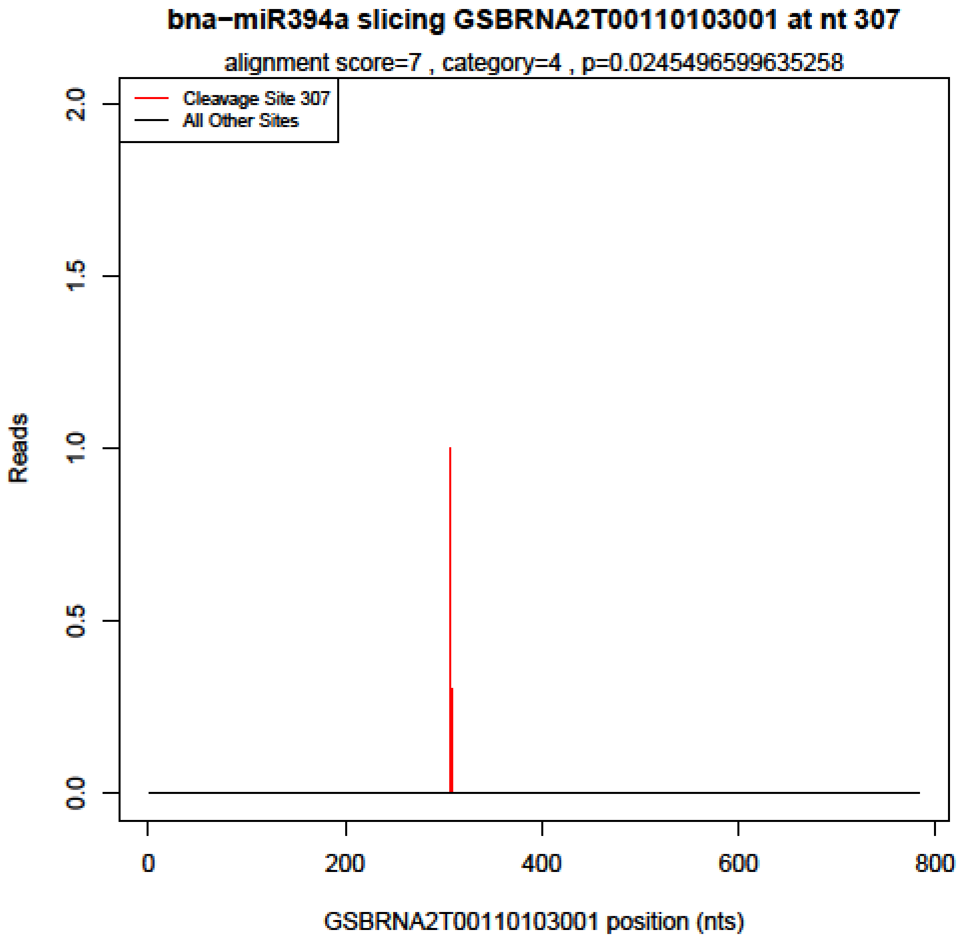

bna−miR394a slicing GSBRNA2T00110103001 at nt 307
alignment score=7 , category=4 , p=0.0245496599635258
Cleavage Site 307
All Other Sites
Reads
0.0
0.5
1.0
1.5
2.0
0
200
400
600
800
GSBRNA2T00110103001 position (nts)

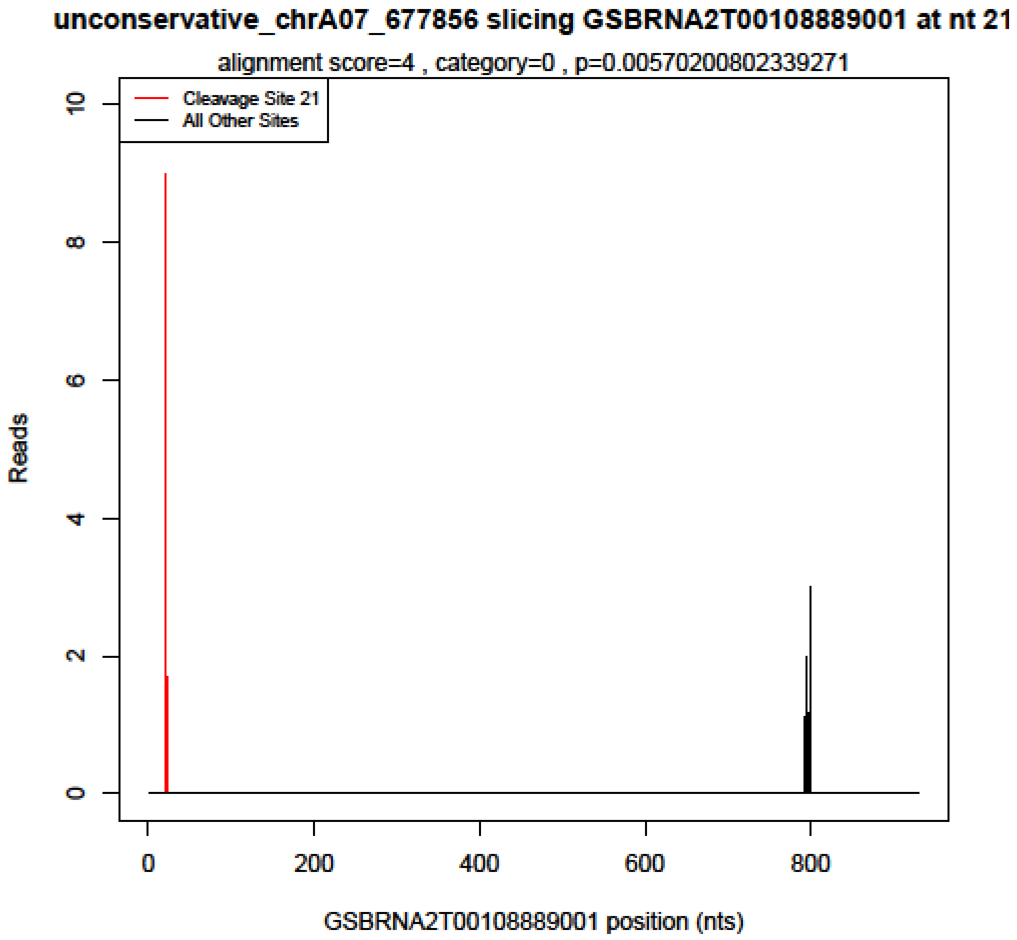
unconservative_chrA07_677856 slicing GSBRNA2T00108889001 at nt 21
alignment score=4 , category=0 , p=0.00570200802339271
Cleavage Site 21
All Other Sites
Reads
0
2
4
6
8
10
0
200
400
600
800
GSBRNA2T00108889001 position (nts)

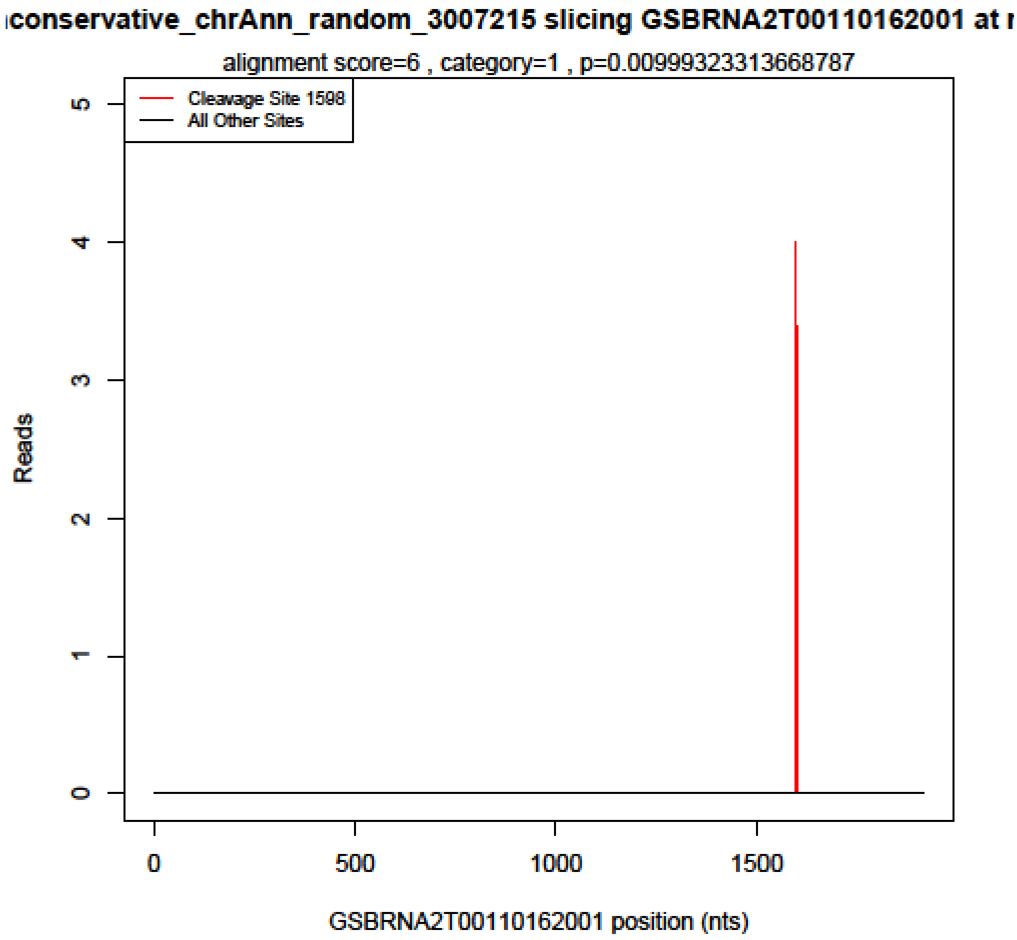
conservative_chrAnn_random_3007215 slicing GSBRNA2T00110162001 at r
alignment score=6 , category=1 , p=0.00999323313668787
Cleavage Site 1598
All Other Sites
Reads
0
1
2
3
4
5
0
500
1000
1500
GSBRNA2T00110162001 position (nts)

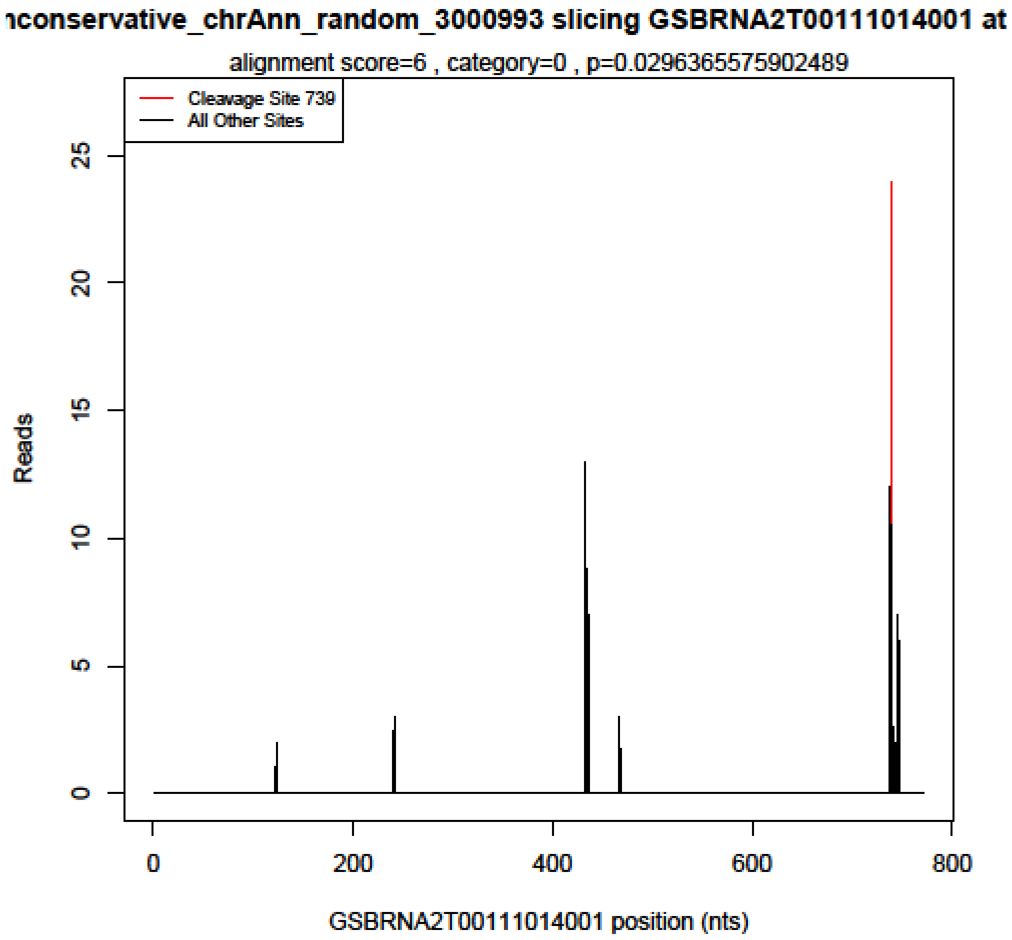
nconservative_chrAnn_random_3000993 slicing GSBRNA2T00111014001 at
alignment score=6 , category=0 , p=0.0296365575902489
Cleavage Site 739
All Other Sites
Reads
0
5
10
15
20
25
0
200
400
600
800
GSBRNA2T00111014001 position (nts)

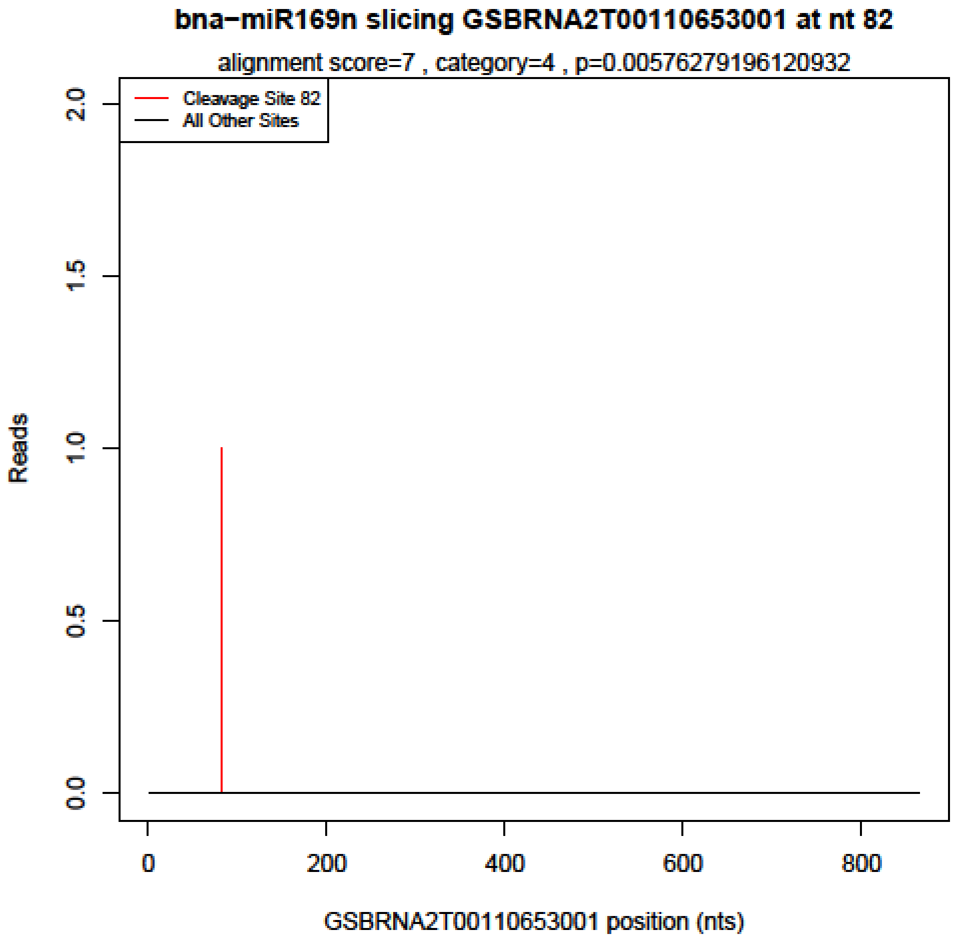

bna−miR169n slicing GSBRNA2T00110653001 at nt 82
alignment score=7 , category=4 , p=0.00576279196120932
Cleavage Site 82
All Other Sites
Reads
0.0
0.5
1.0
1.5
2.0
0
200
400
600
800
GSBRNA2T00110653001 position (nts)

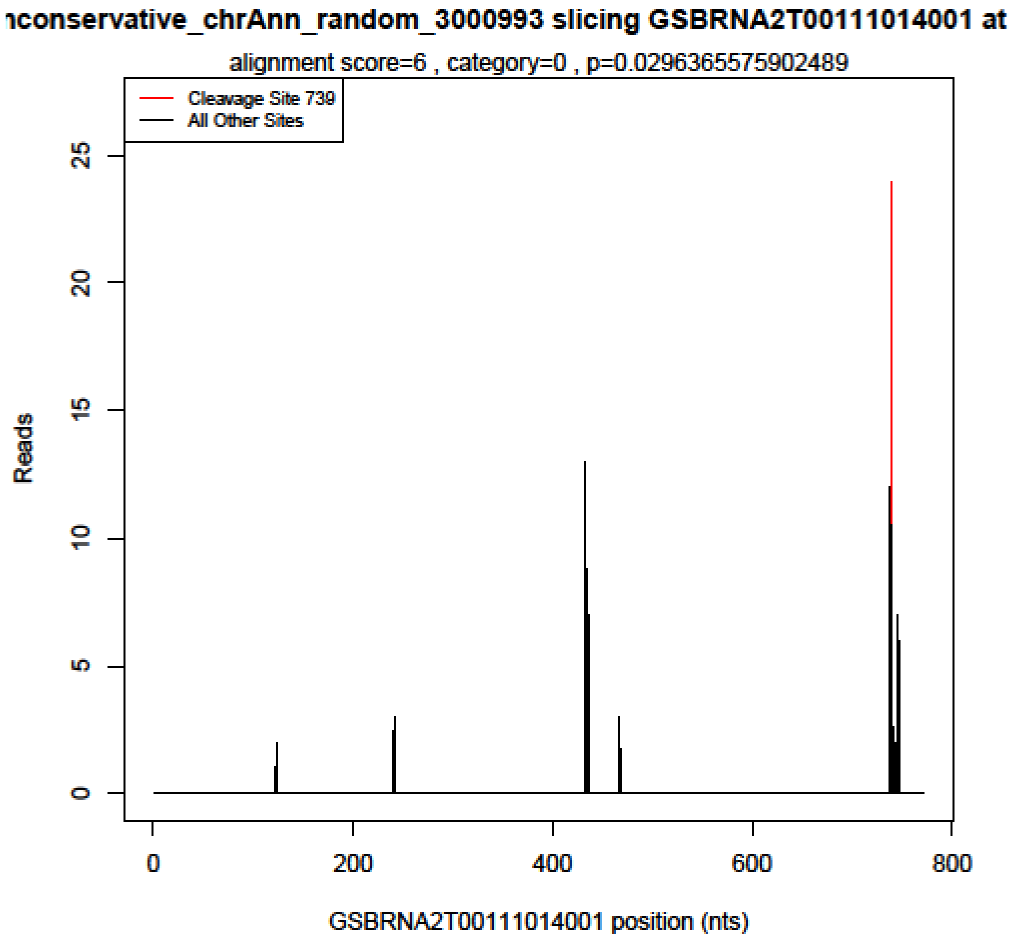

nconservative_chrAnn_random_3000993 slicing GSBRNA2T00111014001 at
alignment score=6 , category=0 , p=0.0296365575902489
Cleavage Site 739
All Other Sites
Reads
0
5
10
15
20
25
0
200
400
600
800
GSBRNA2T00111014001 position (nts)

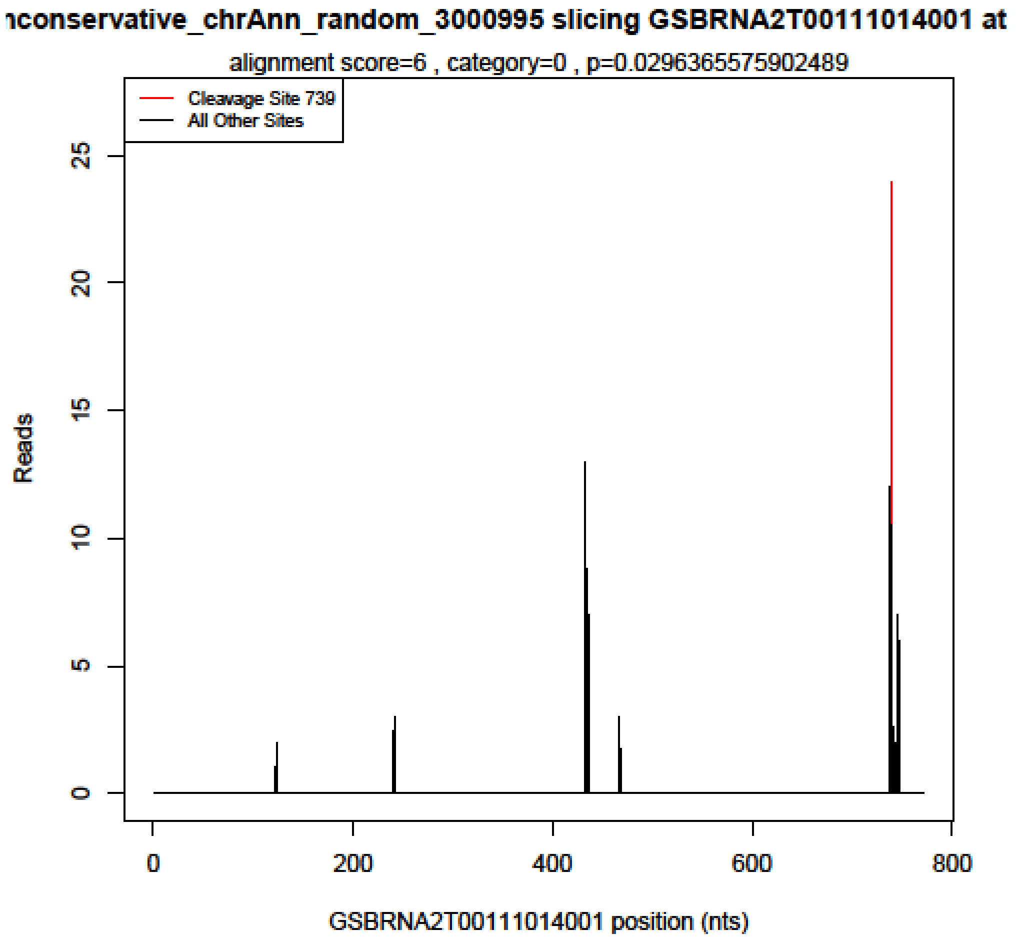
nconservative_chrAnn_random_3000995 slicing GSBRNA2T00111014001 at
alignment score=6 , category=0 , p=0.0296365575902489
Cleavage Site 739
All Other Sites
Reads
0
5
10
15
20
25
0
200
400
600
800
GSBRNA2T00111014001 position (nts)

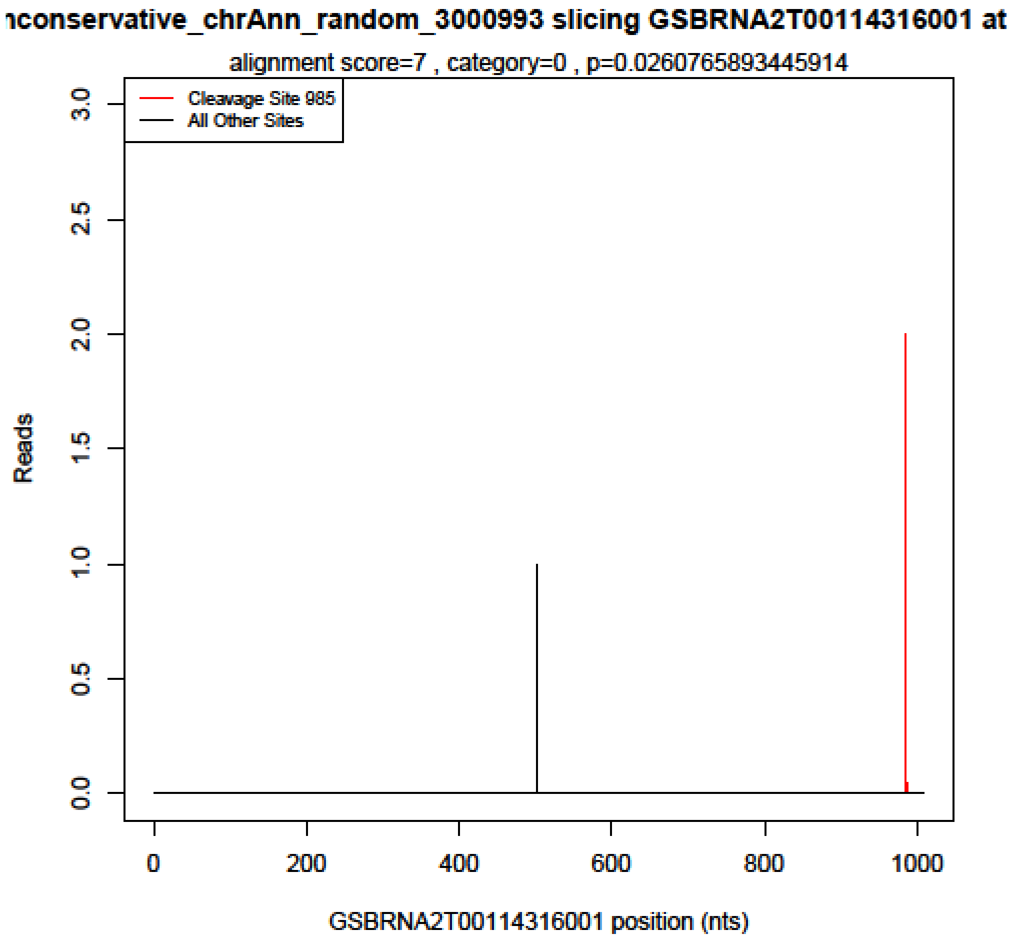

nconservative_chrAnn_random_3000993 slicing GSBRNA2T00114316001 at
alignment score=7 , category=0 , p=0.0260765893445914
Cleavage Site 985
All Other Sites
Reads
0.0
0.5
1.0
1.5
2.0
2.5
3.0
0
200
400
600
800
1000
GSBRNA2T00114316001 position (nts)

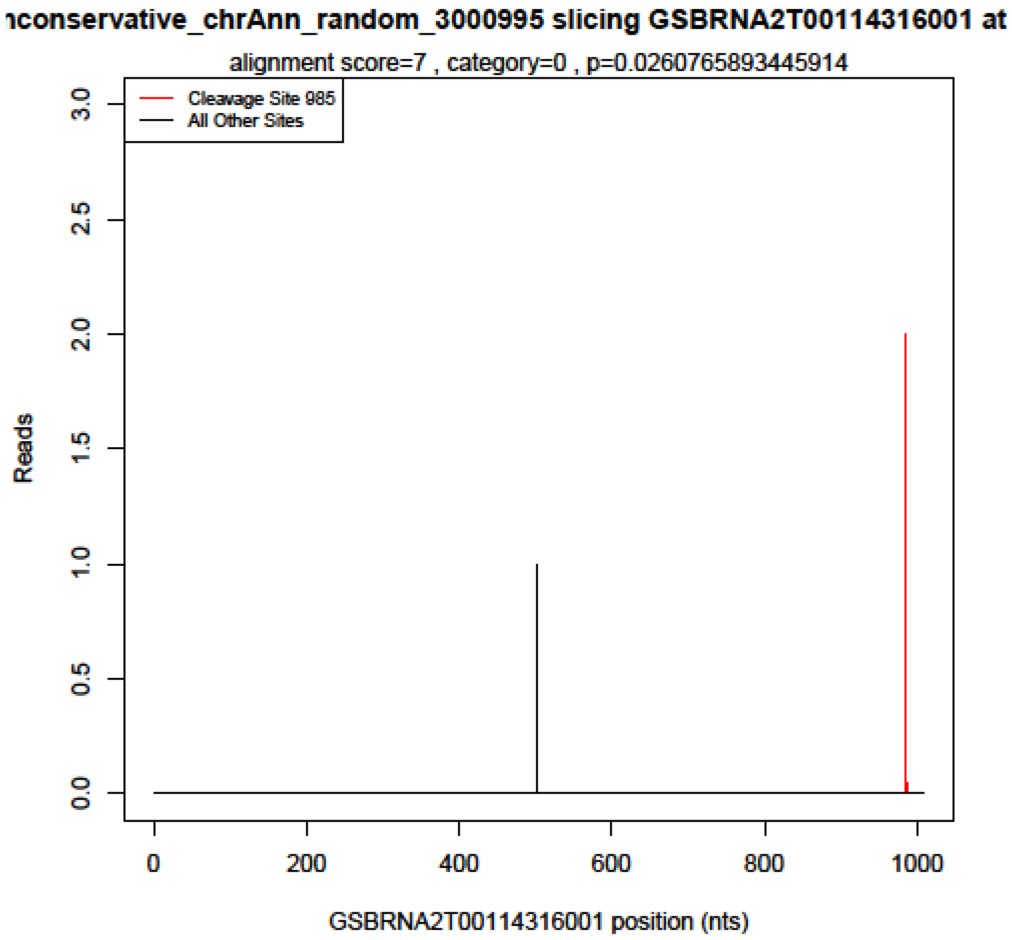
nconservative_chrAnn_random_3000995 slicing GSBRNA2T00114316001 at
alignment score=7 , category=0 , p=0.0260765893445914
Cleavage Site 985
All Other Sites
Reads
0.0
0.5
1.0
1.5
2.0
2.5
3.0
0
200
400
600
800
1000
GSBRNA2T00114316001 position (nts)

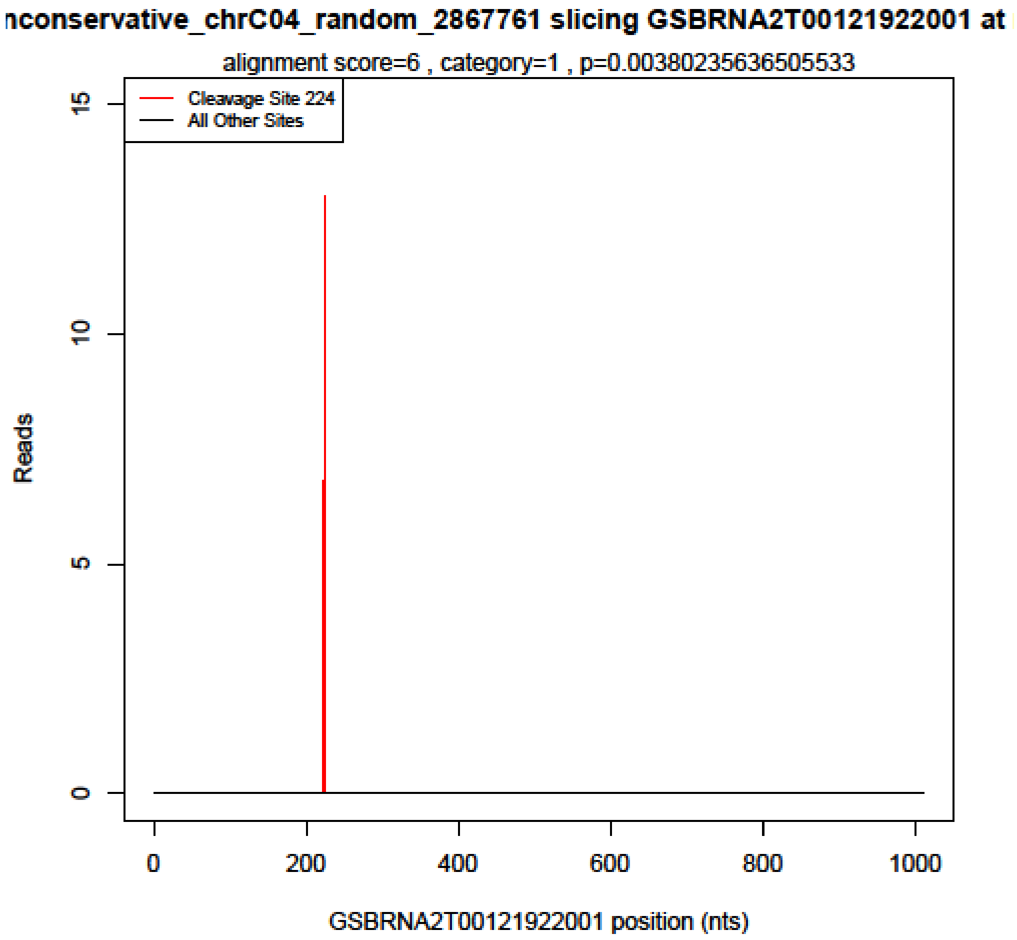

nconservative_chrC04_random_2867761 slicing GSBRNA2T00121922001 at
alignment score=6 , category=1 , p=0.00380235636505533
Cleavage Site 224
All Other Sites
Reads
0
5
10
15
0
200
400
600
800
1000
GSBRNA2T00121922001 position (nts)

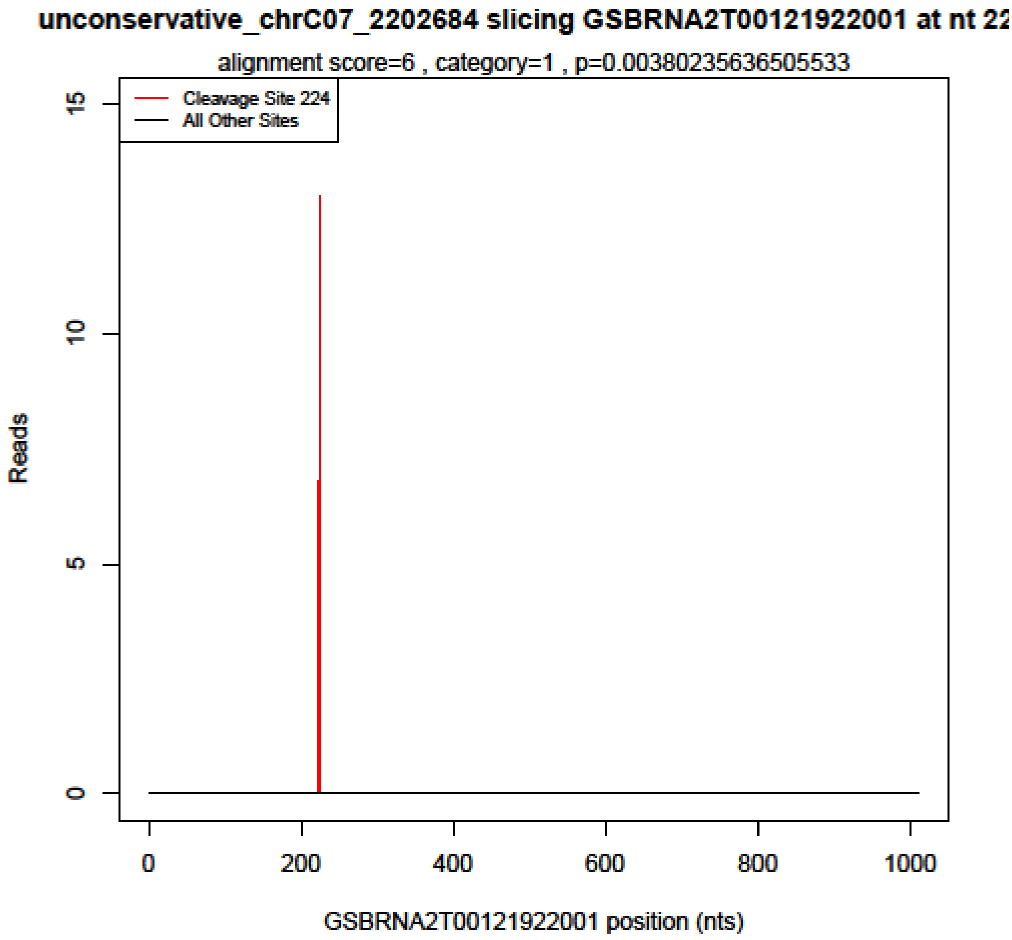
unconservative_chrC07_2202684 slicing GSBRNA2T00121922001 at nt 22
alignment score=6 , category=1 , p=0.00380235636505533
Cleavage Site 224
All Other Sites
Reads
0
5
10
15
0
200
400
600
800
1000
GSBRNA2T00121922001 position (nts)

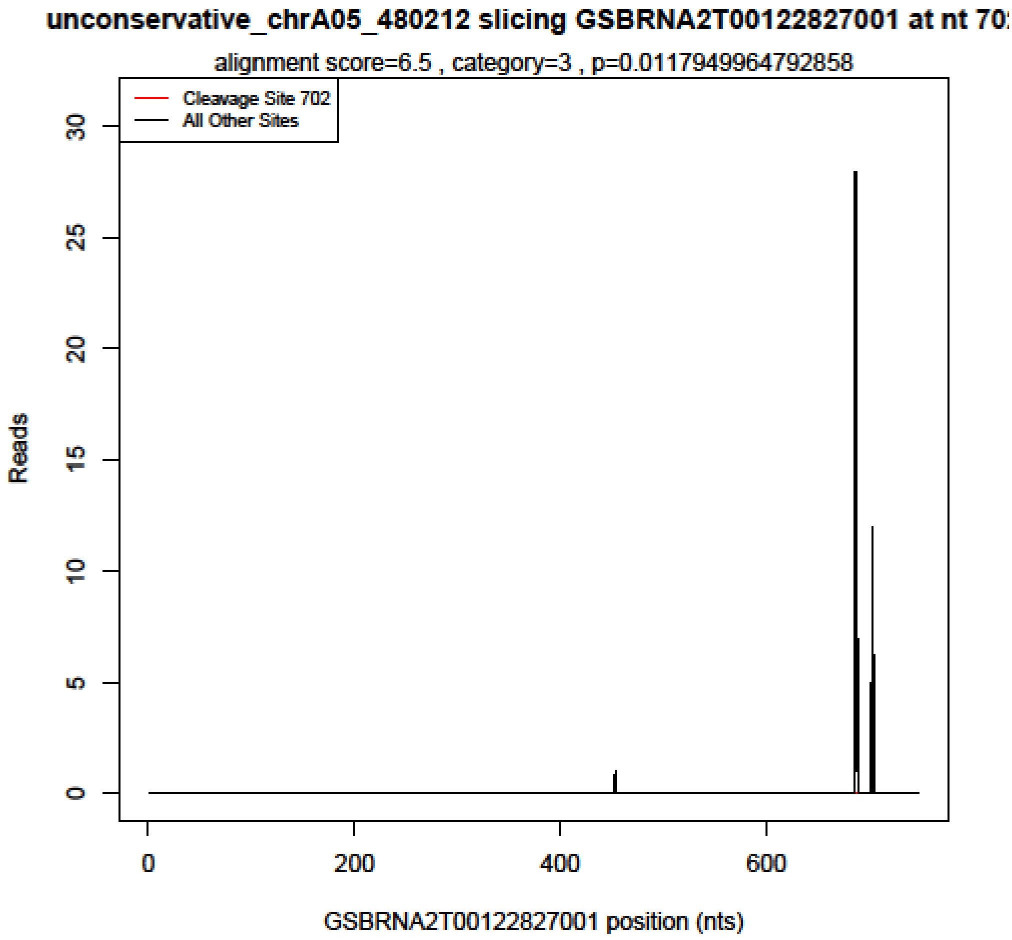
unconservative_chrA05_480212 slicing GSBRNA2T00122827001 at nt 70
alignment score=6.5 , category=3 , p=0.0117949964792858
Cleavage Site 702
All Other Sites
Reads
0
5
10
15
20
25
30
0
200
400
600
GSBRNA2T00122827001 position (nts)

unconservative_chrA07_648408 slicing GSBRNA2T00122827001 at nt 70[illegible]

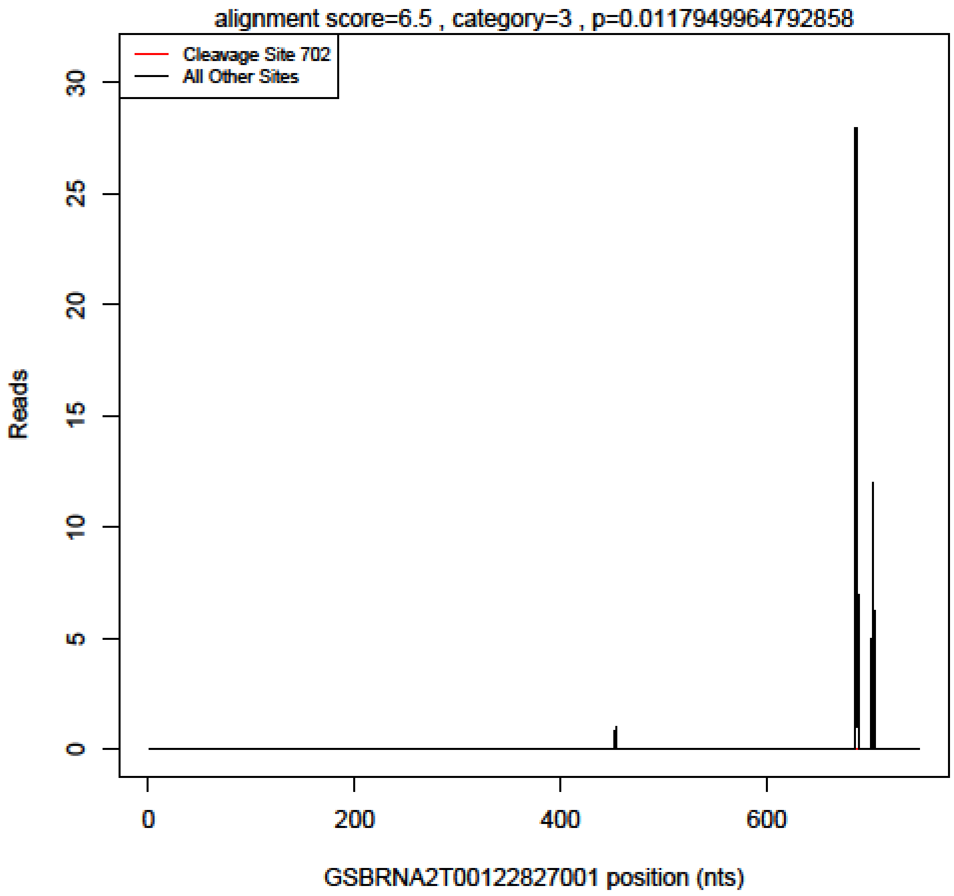

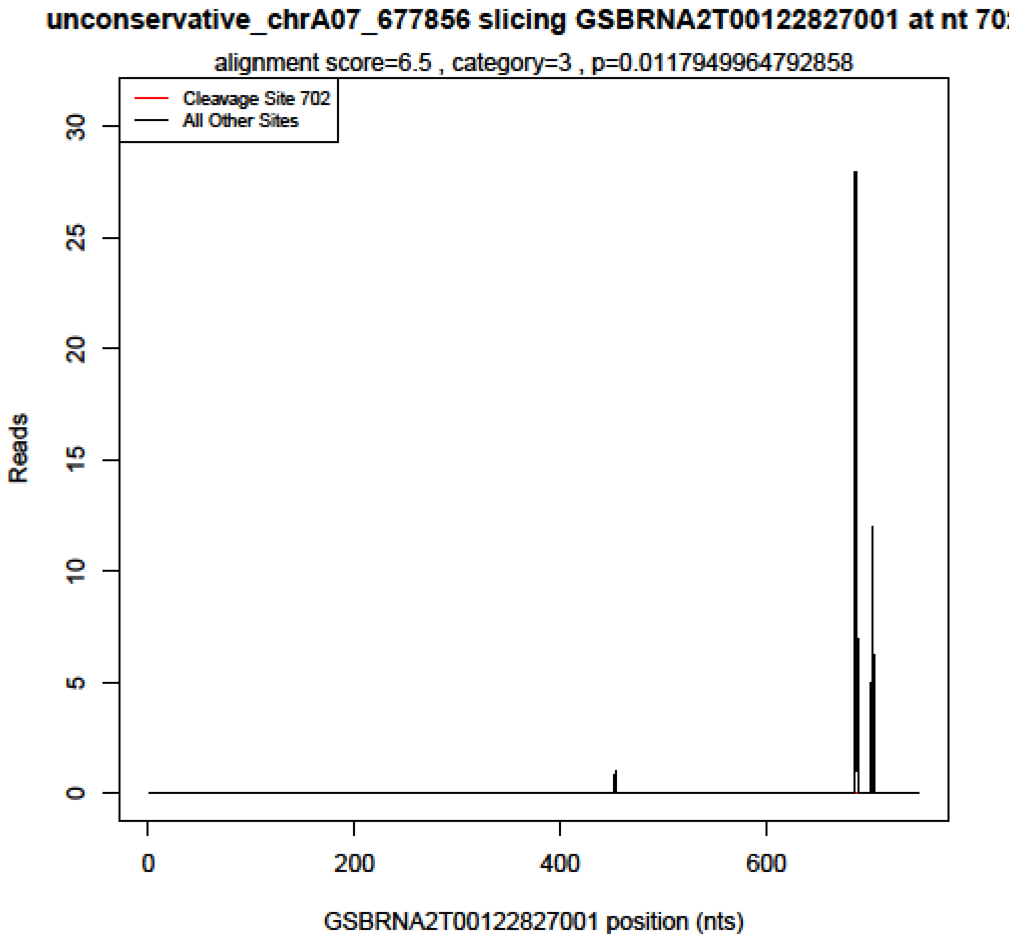
unconservative_chrA07_677856 slicing GSBRNA2T00122827001 at nt 70
alignment score=6.5 , category=3 , p=0.0117949964792858
Cleavage Site 702
All Other Sites
Reads
0
5
10
15
20
25
30
0
200
400
600
GSBRNA2T00122827001 position (nts)

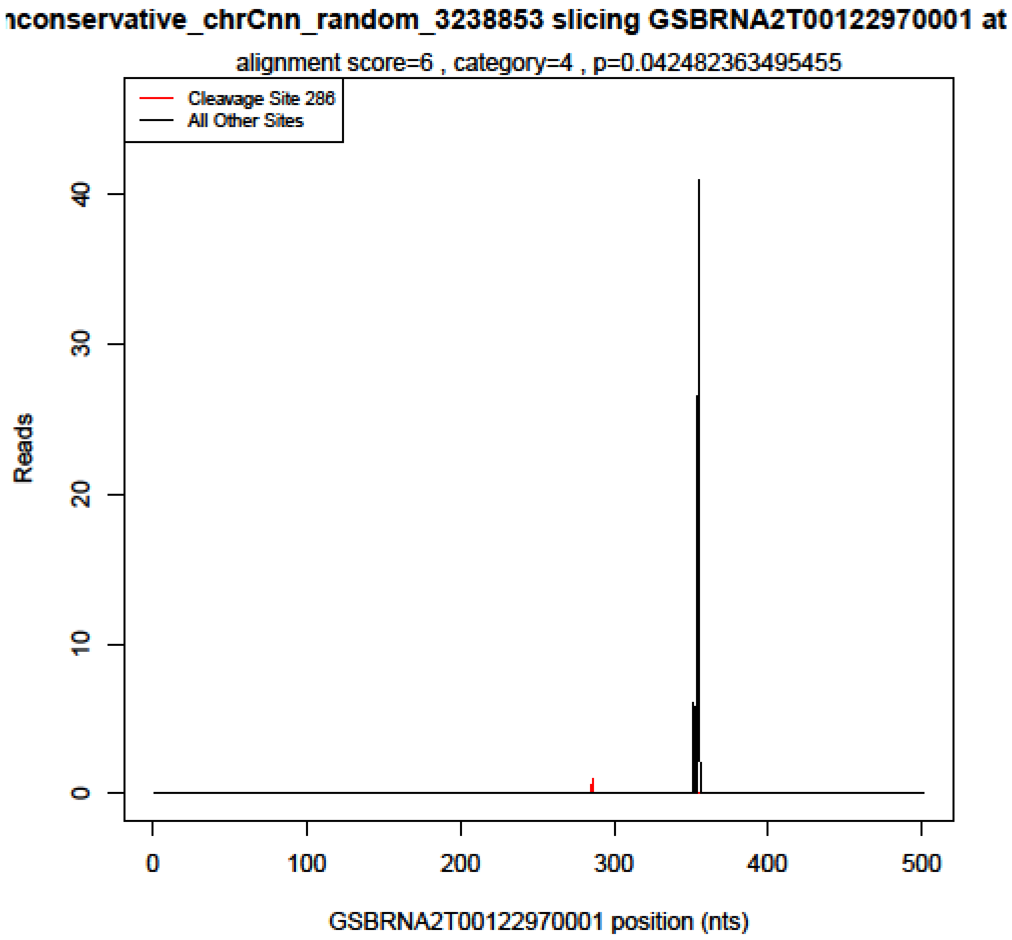
nconservative_chrCnn_random_3238853 slicing GSBRNA2T00122970001 at
alignment score=6 , category=4 , p=0.042482363495455
Cleavage Site 286
All Other Sites
Reads
0
10
20
30
40
0
100
200
300
400
500
GSBRNA2T00122970001 position (nts)

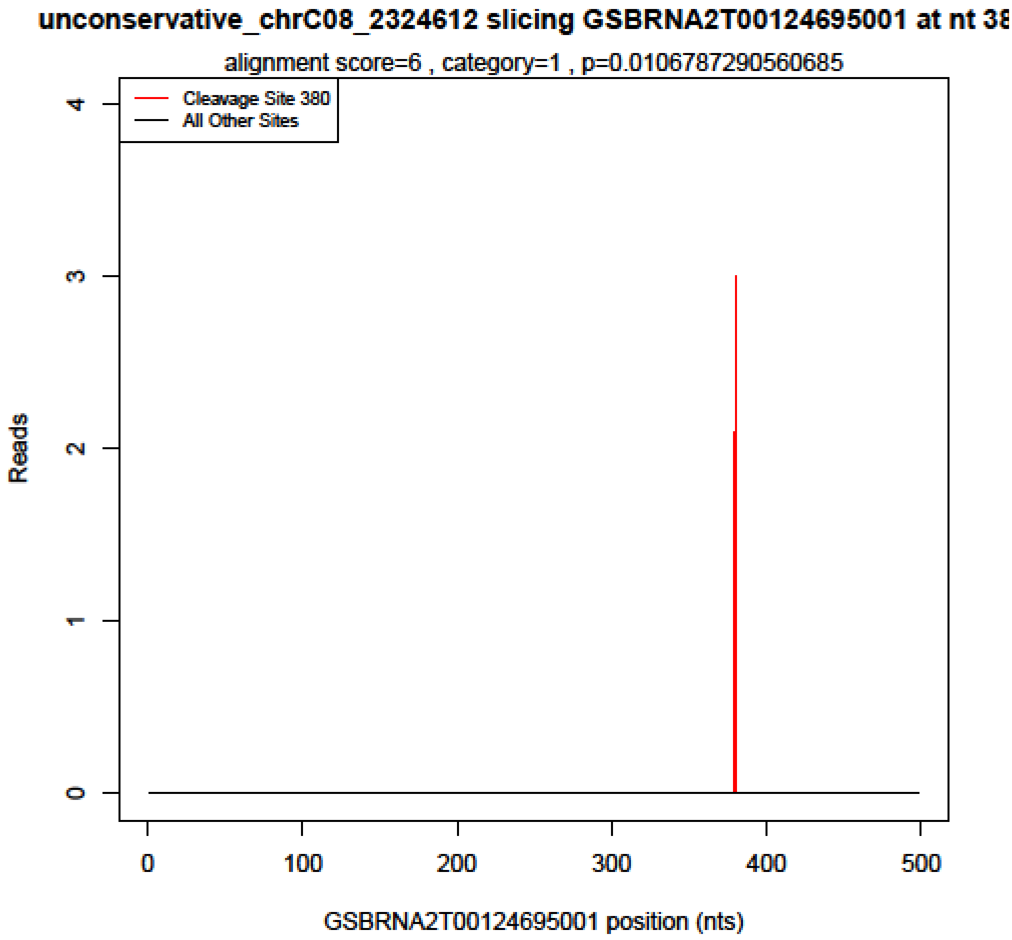

unconservative_chrC08_2324612 slicing GSBRNA2T00124695001 at nt 38
alignment score=6 , category=1 , p=0.0106787290560685
Cleavage Site 380
All Other Sites
Reads
0
1
2
3
4
0
100
200
300
400
500
GSBRNA2T00124695001 position (nts)

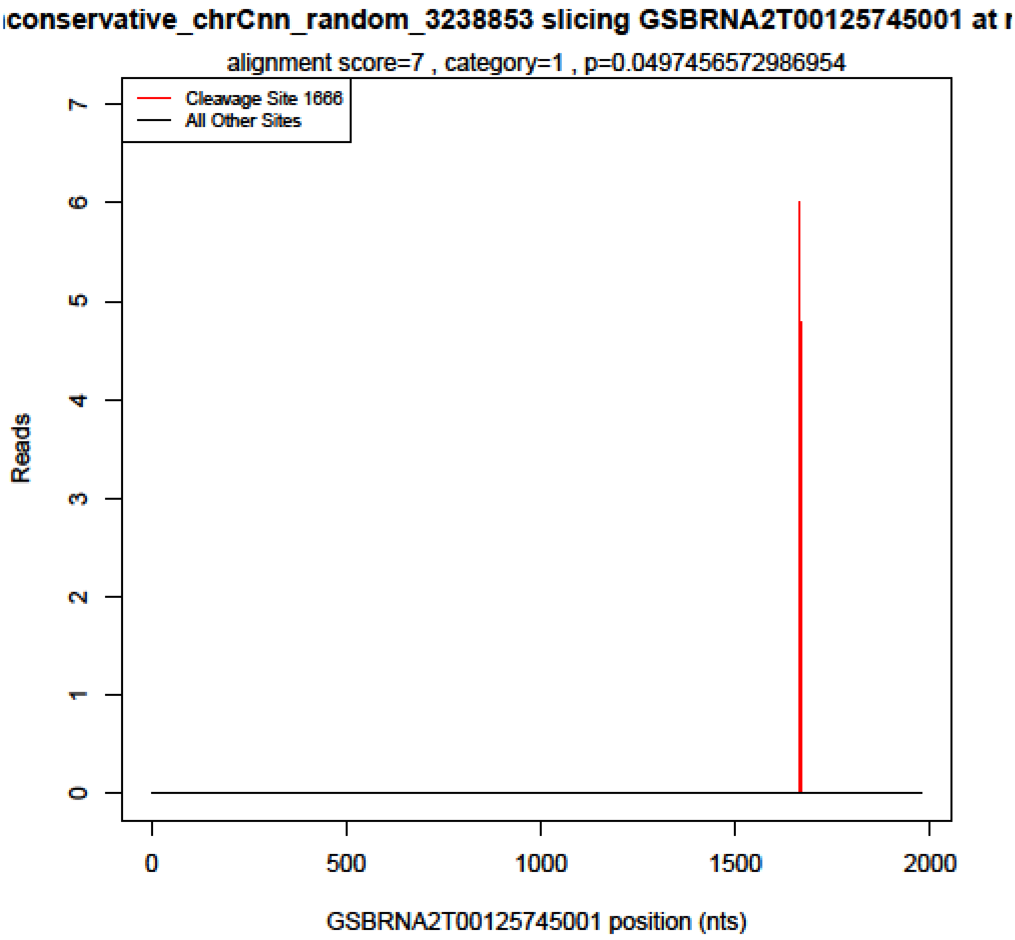

conservative_chrCnn_random_3238853 slicing GSBRNA2T00125745001 at r
alignment score=7 , category=1 , p=0.0497456572986954
Cleavage Site 1666
All Other Sites
Reads
0
1
2
3
4
5
6
7
0
500
1000
1500
2000
GSBRNA2T00125745001 position (nts)

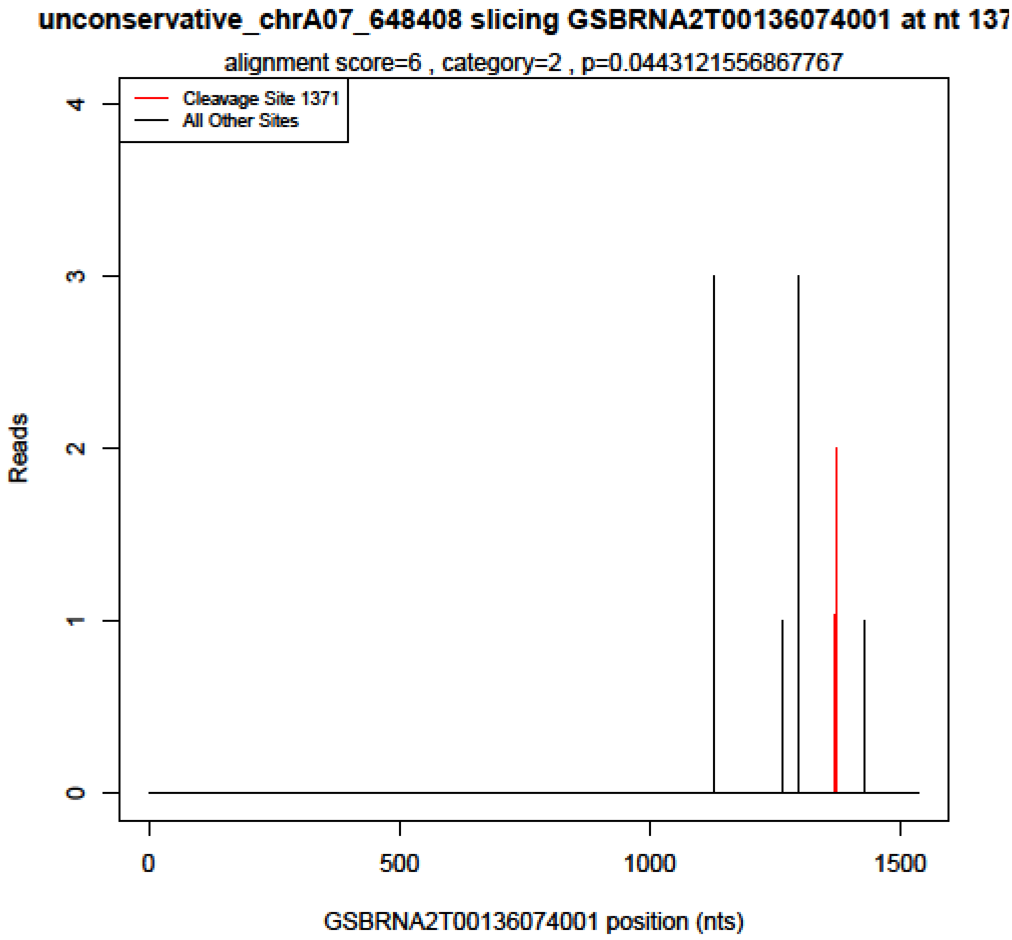
unconservative_chrA07_648408 slicing GSBRNA2T00136074001 at nt 137
alignment score=6 , category=2 , p=0.0443121556867767
Cleavage Site 1371
All Other Sites
Reads
0
1
2
3
4
0
500
1000
1500
GSBRNA2T00136074001 position (nts)

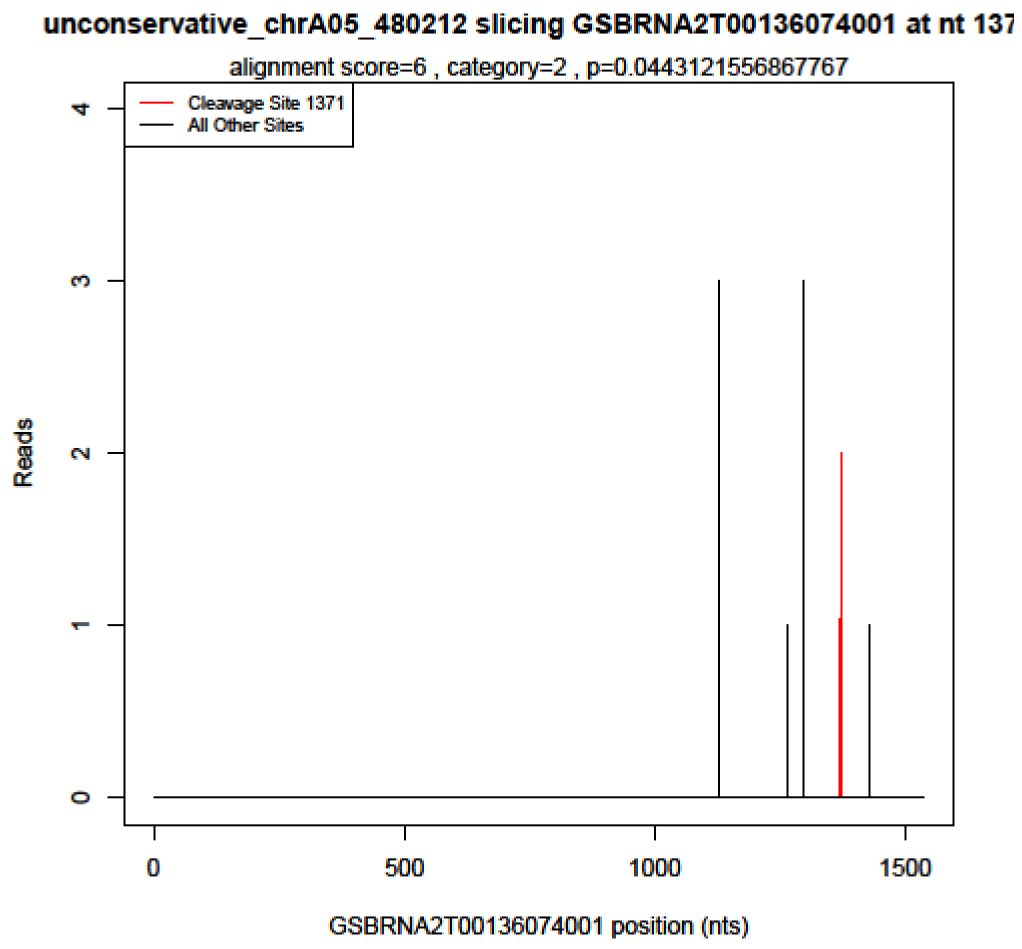
unconservative_chrA05_480212 slicing GSBRNA2T00136074001 at nt 137
alignment score=6 , category=2 , p=0.0443121556867767
Cleavage Site 1371
All Other Sites
Reads
0
1
2
3
4
0
500
1000
1500
GSBRNA2T00136074001 position (nts)

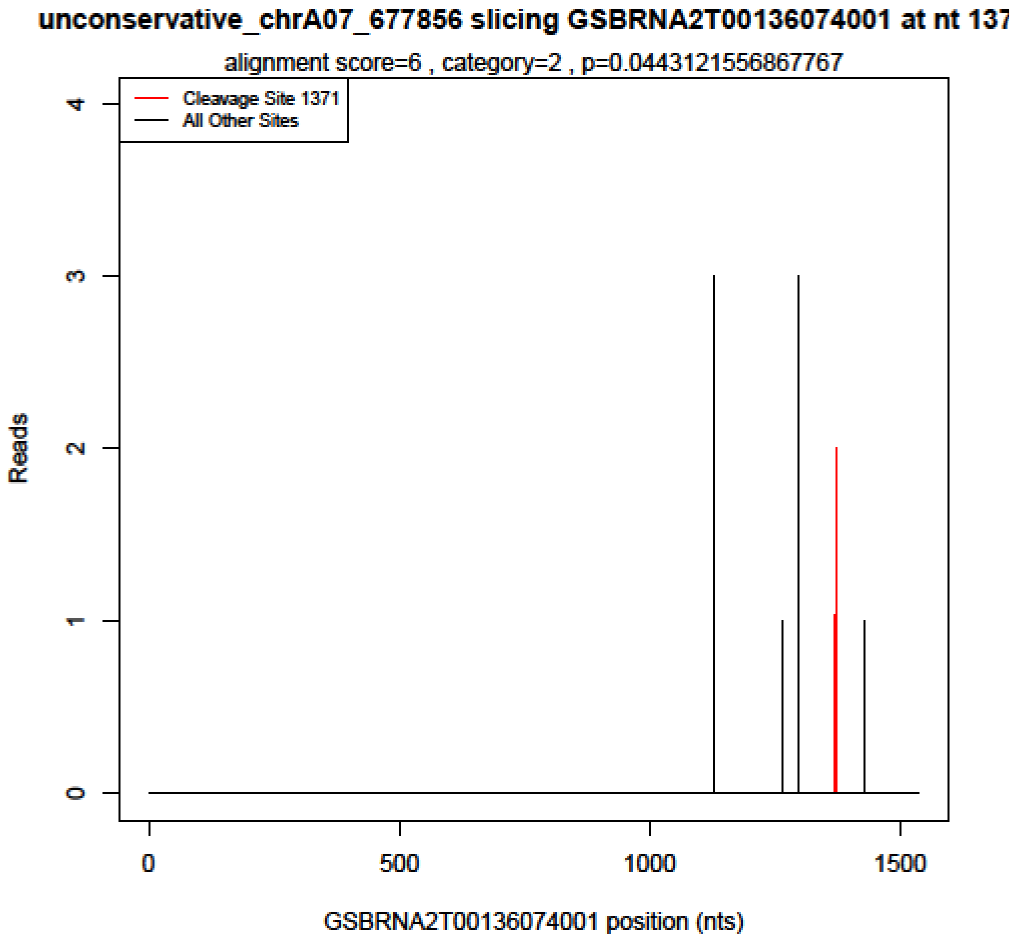

unconservative_chrA07_677856 slicing GSBRNA2T00136074001 at nt 137
alignment score=6 , category=2 , p=0.0443121556867767
Cleavage Site 1371
All Other Sites
Reads
0
1
2
3
4
0
500
1000
1500
GSBRNA2T00136074001 position (nts)

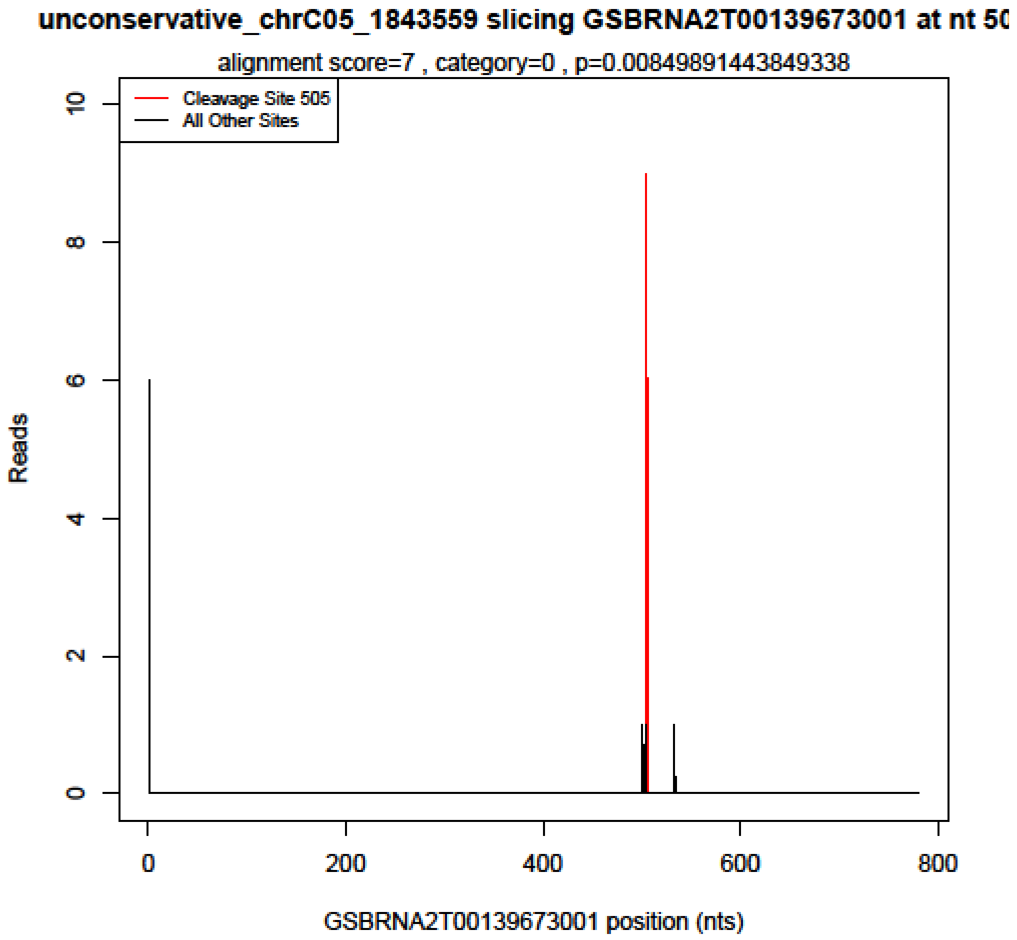

unconservative_chrC05_1843559 slicing GSBRNA2T00139673001 at nt 50
alignment score=7 , category=0 , p=0.00849891443849338
Cleavage Site 505
All Other Sites
Reads
0
2
4
6
8
10
0
200
400
600
800
GSBRNA2T00139673001 position (nts)

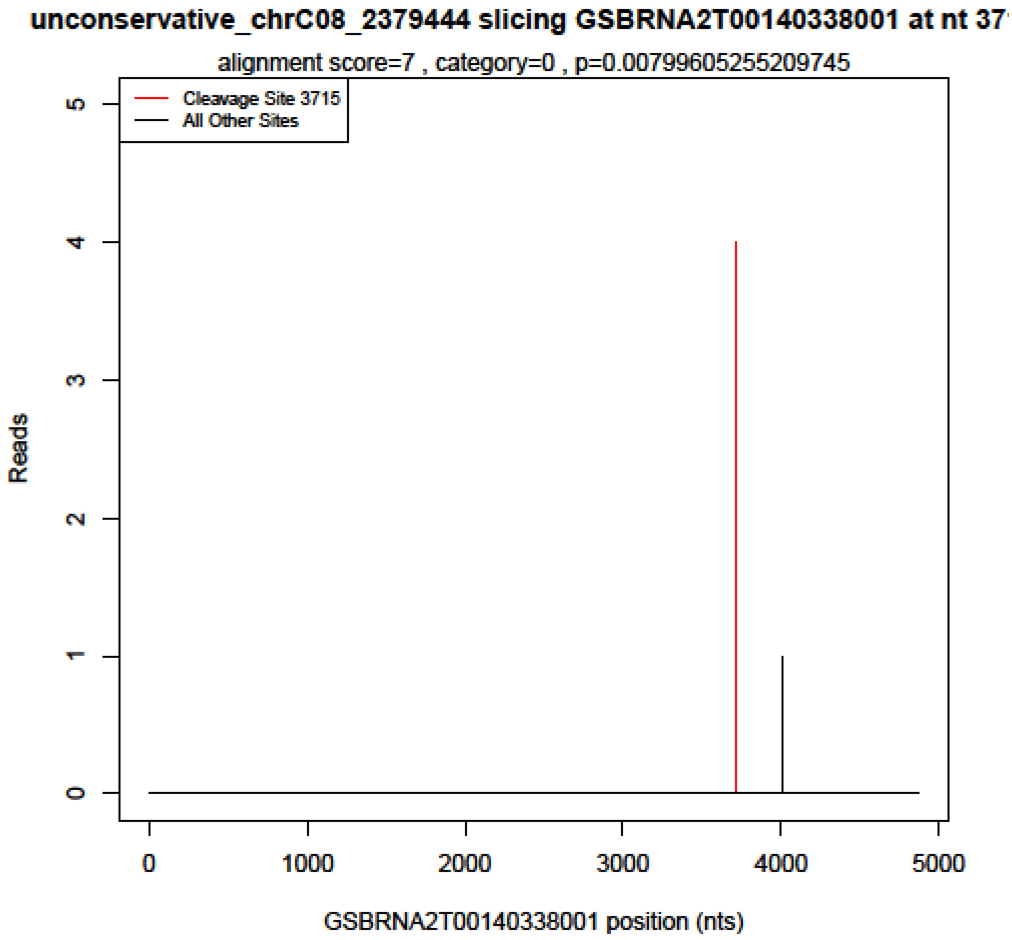

unconservative_chrC08_2379444 slicing GSBRNA2T00140338001 at nt 37
alignment score=7 , category=0 , p=0.00799605255209745
Cleavage Site 3715
All Other Sites
Reads
0
1
2
3
4
5
0
1000
2000
3000
4000
5000
GSBRNA2T00140338001 position (nts)

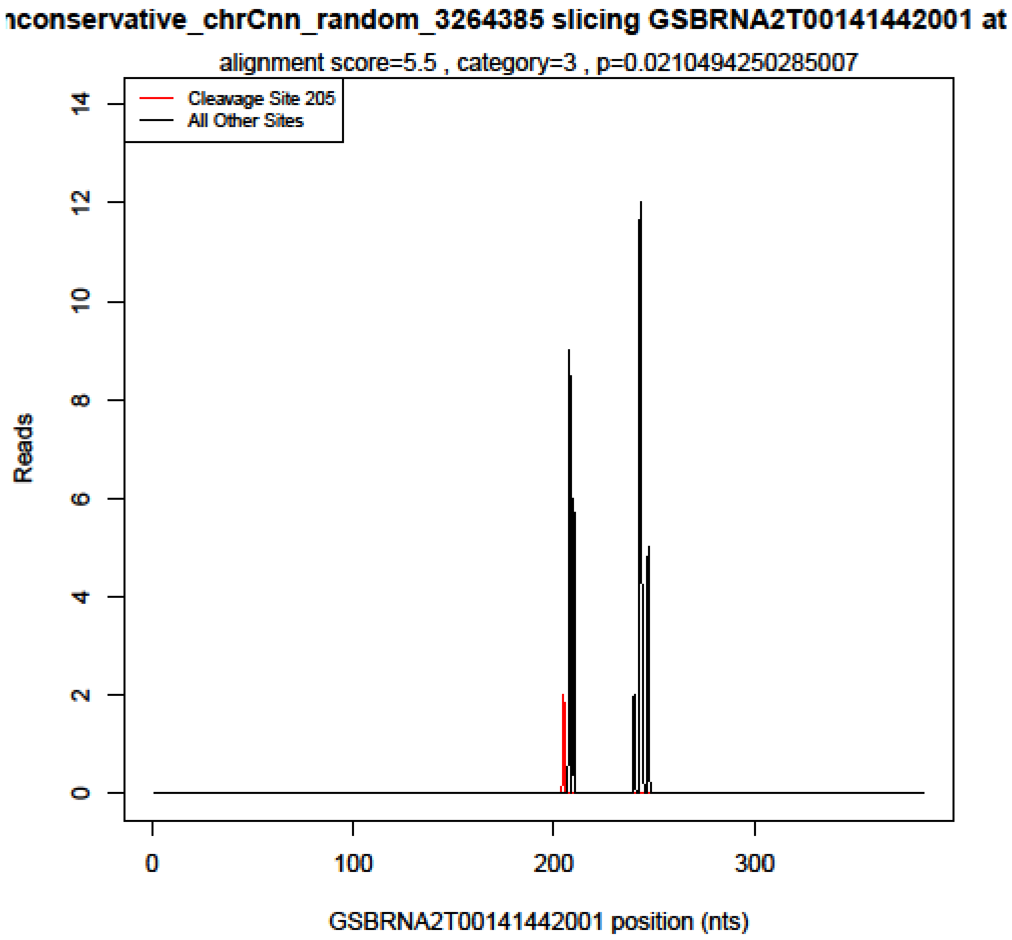

nconservative_chrCnn_random_3264385 slicing GSBRNA2T00141442001 at
alignment score=5.5 , category=3 , p=0.0210494250285007
Cleavage Site 205
All Other Sites
Reads
0
2
4
6
8
10
12
14
0
100
200
300
GSBRNA2T00141442001 position (nts)

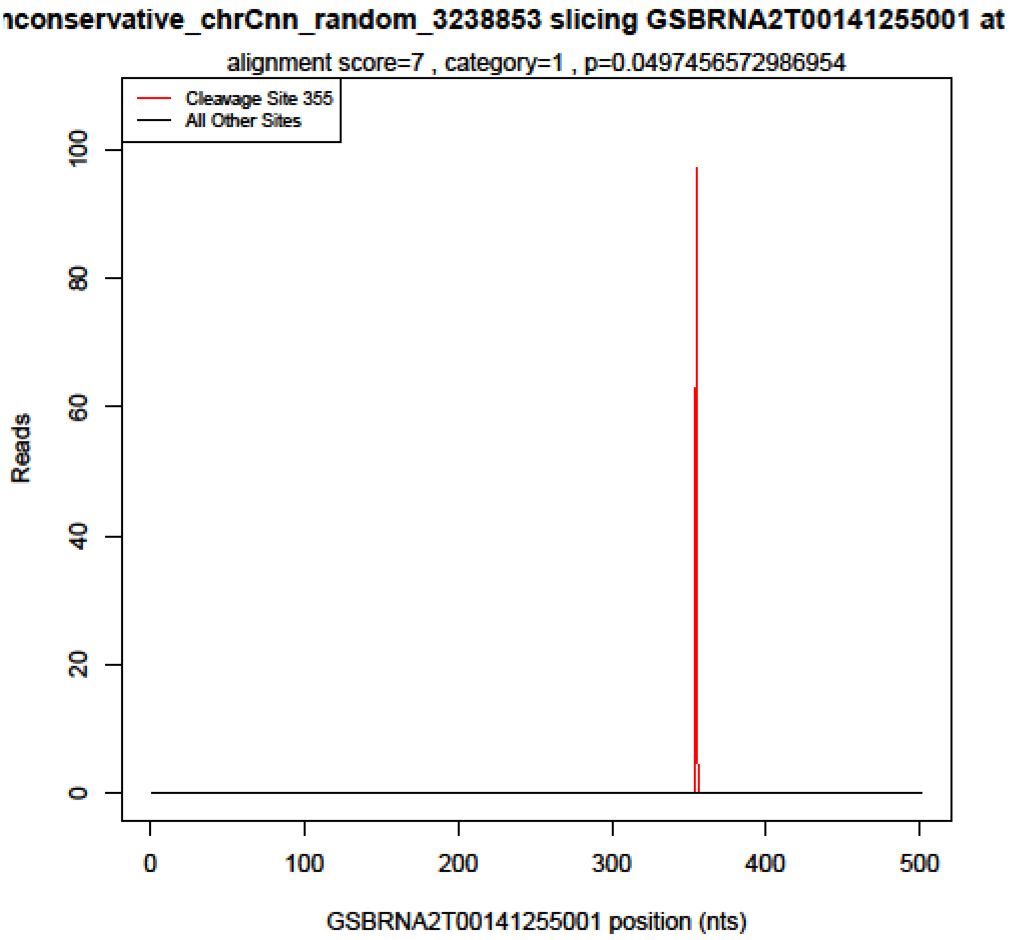
nconservative_chrCnn_random_3238853 slicing GSBRNA2T00141255001 at
alignment score=7 , category=1 , p=0.0497456572986954
Cleavage Site 355
All Other Sites
Reads
0
20
40
60
80
100
0
100
200
300
400
500
GSBRNA2T00141255001 position (nts)

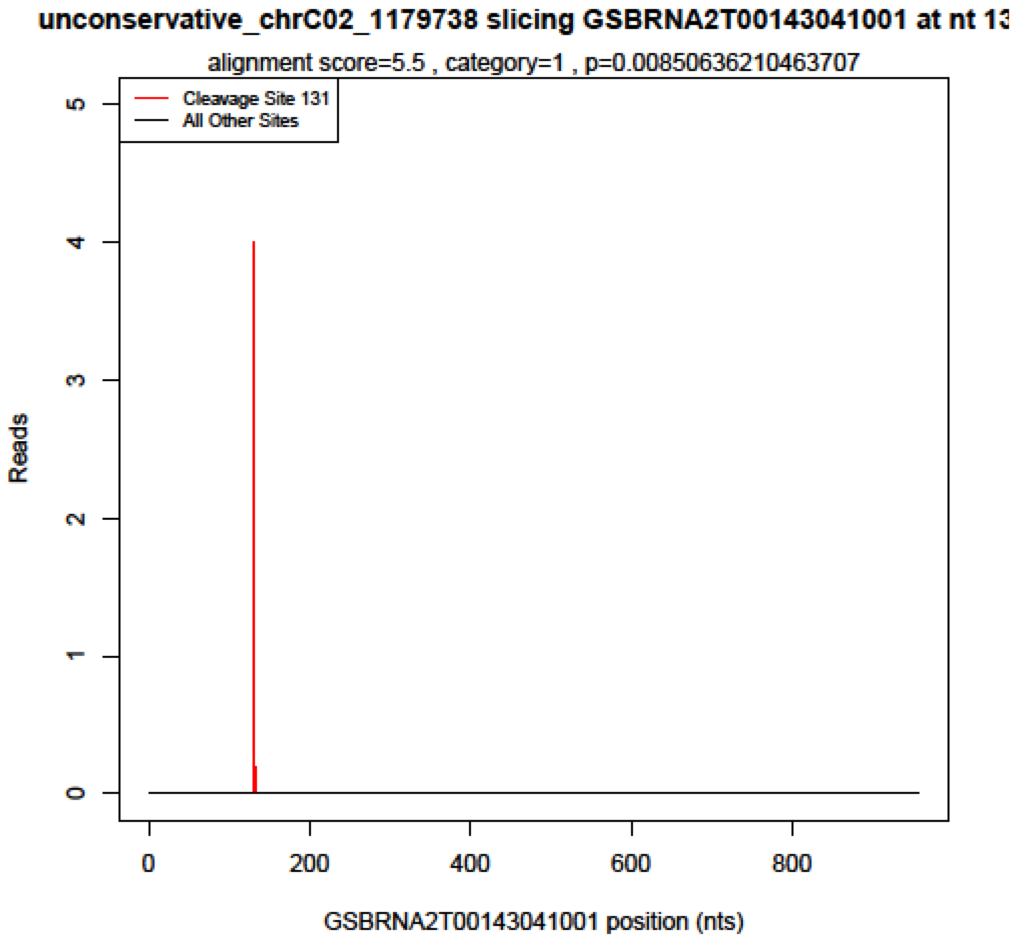

unconservative_chrC02_1179738 slicing GSBRNA2T00143041001 at nt 13
alignment score=5.5 , category=1 , p=0.00850636210463707
Cleavage Site 131
All Other Sites
Reads
0
1
2
3
4
5
0
200
400
600
800
GSBRNA2T00143041001 position (nts)

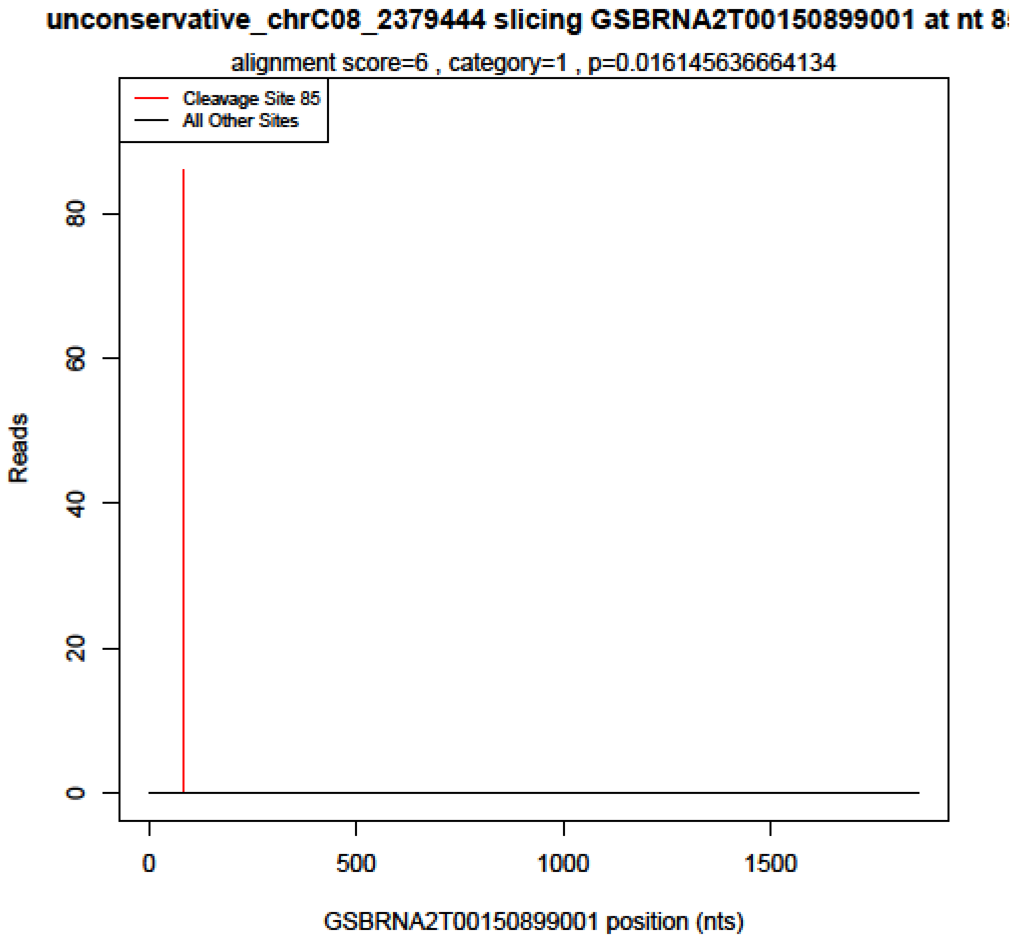
unconservative_chrC08_2379444 slicing GSBRNA2T00150899001 at nt 8
alignment score=6 , category=1 , p=0.016145636664134
Cleavage Site 85
All Other Sites
Reads
0
20
40
60
80
0
500
1000
1500
GSBRNA2T00150899001 position (nts)

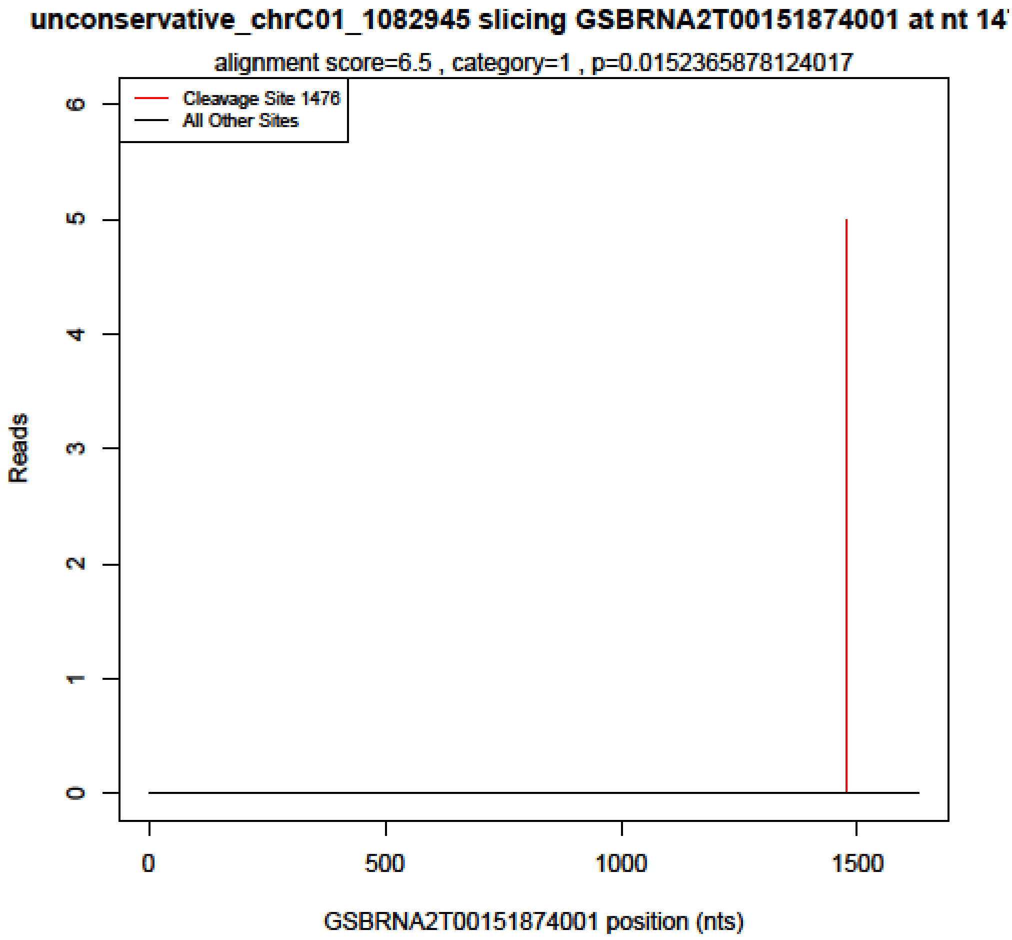
unconservative_chrC01_1082945 slicing GSBRNA2T00151874001 at nt 14
alignment score=6.5 , category=1 , p=0.0152365878124017
Cleavage Site 1476
All Other Sites
Reads
0
1
2
3
4
5
6
0
500
1000
1500
GSBRNA2T00151874001 position (nts)

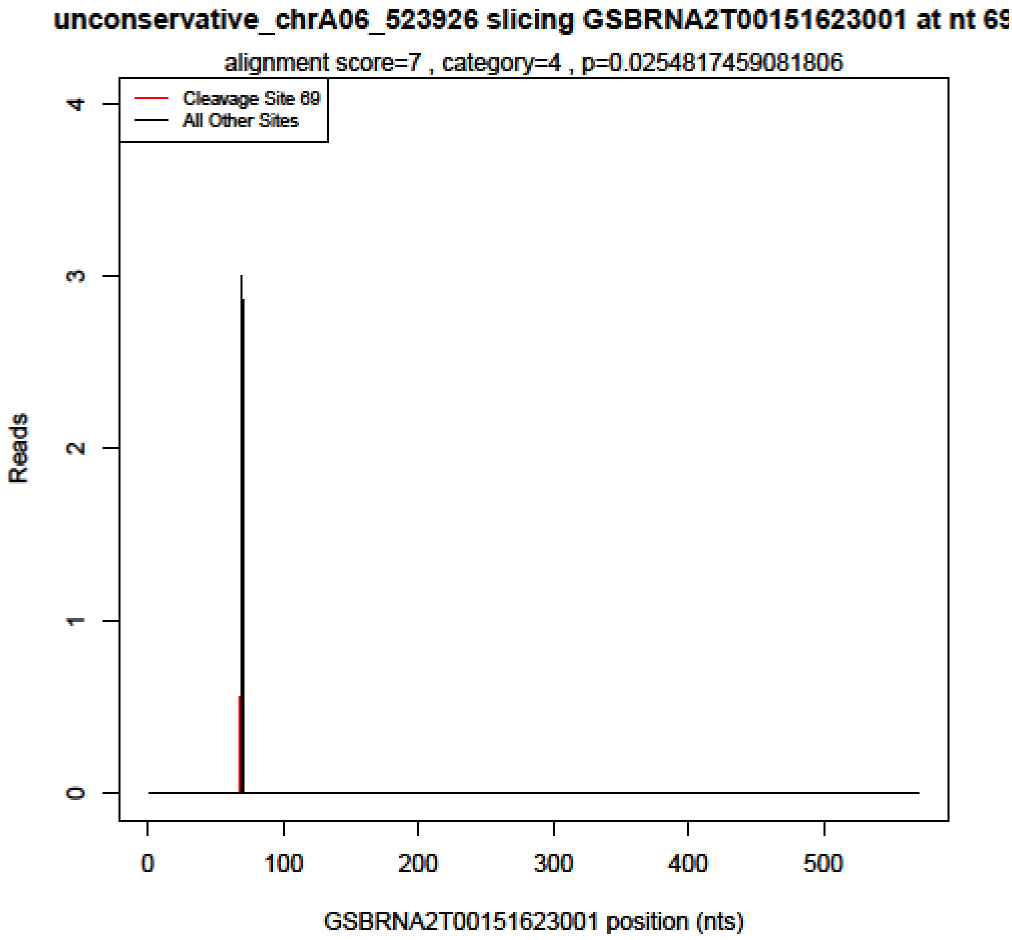

unconservative_chrA06_523926 slicing GSBRNA2T00151623001 at nt 69
alignment score=7 , category=4 , p=0.0254817459081806
Cleavage Site 69
All Other Sites
Reads
0
1
2
3
4
0
100
200
300
400
500
GSBRNA2T00151623001 position (nts)

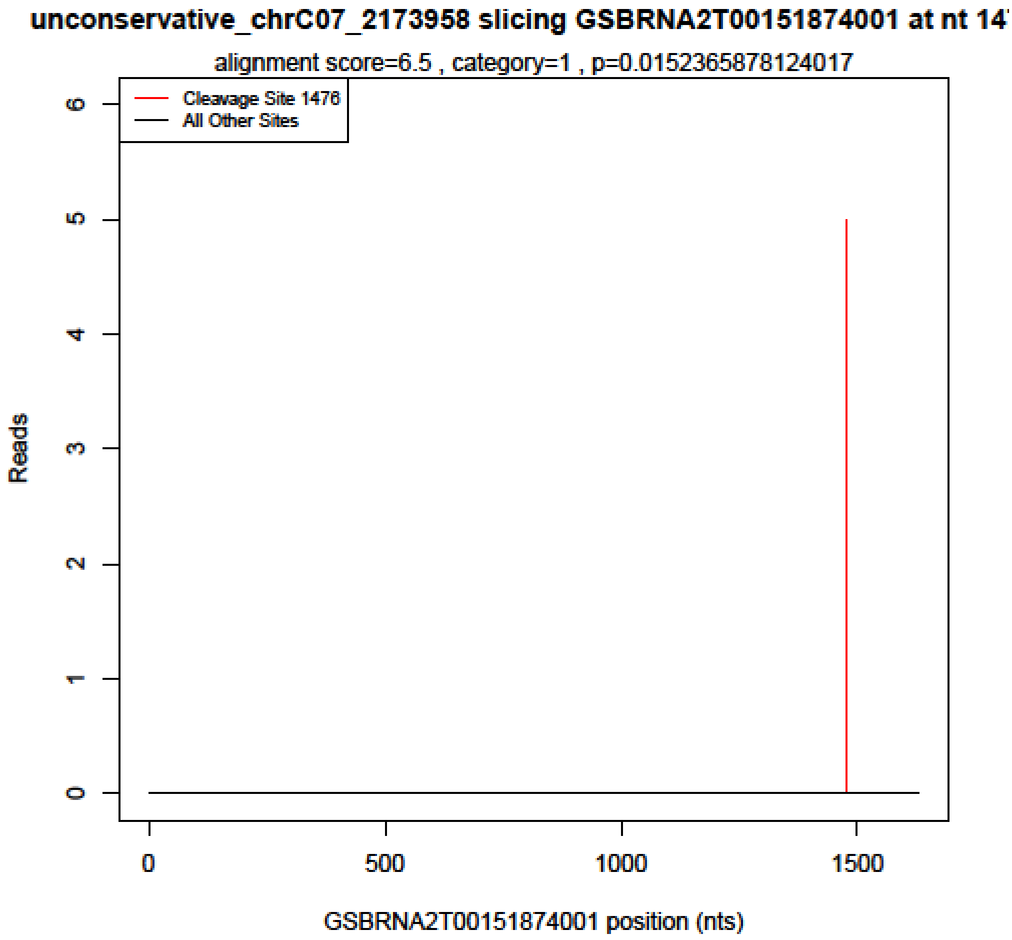

unconservative_chrC07_2173958 slicing GSBRNA2T00151874001 at nt 14
alignment score=6.5 , category=1 , p=0.0152365878124017
Cleavage Site 1476
All Other Sites
Reads
0
1
2
3
4
5
6
0
500
1000
1500
GSBRNA2T00151874001 position (nts)

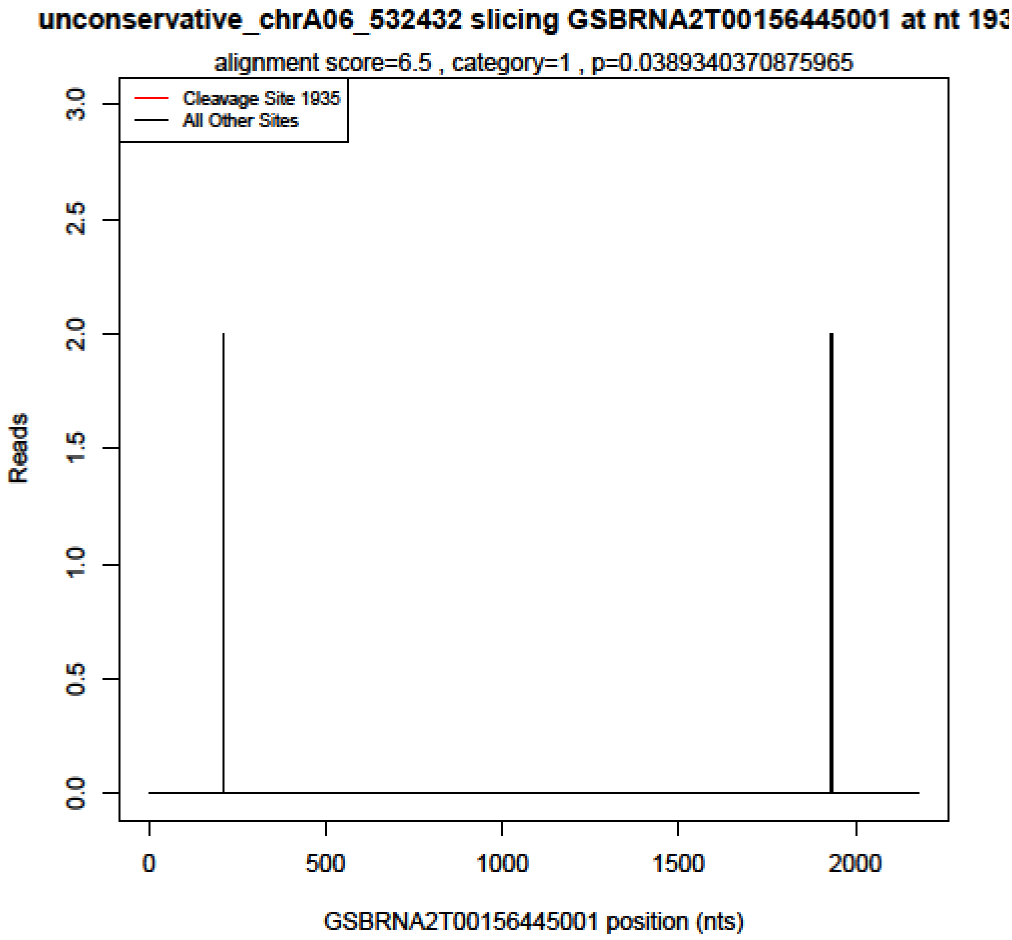
unconservative_chrA06_532432 slicing GSBRNA2T00156445001 at nt 193
alignment score=6.5 , category=1 , p=0.0389340370875965
Cleavage Site 1935
All Other Sites
Reads
0.0
0.5
1.0
1.5
2.0
2.5
3.0
0
500
1000
1500
2000
GSBRNA2T00156445001 position (nts)

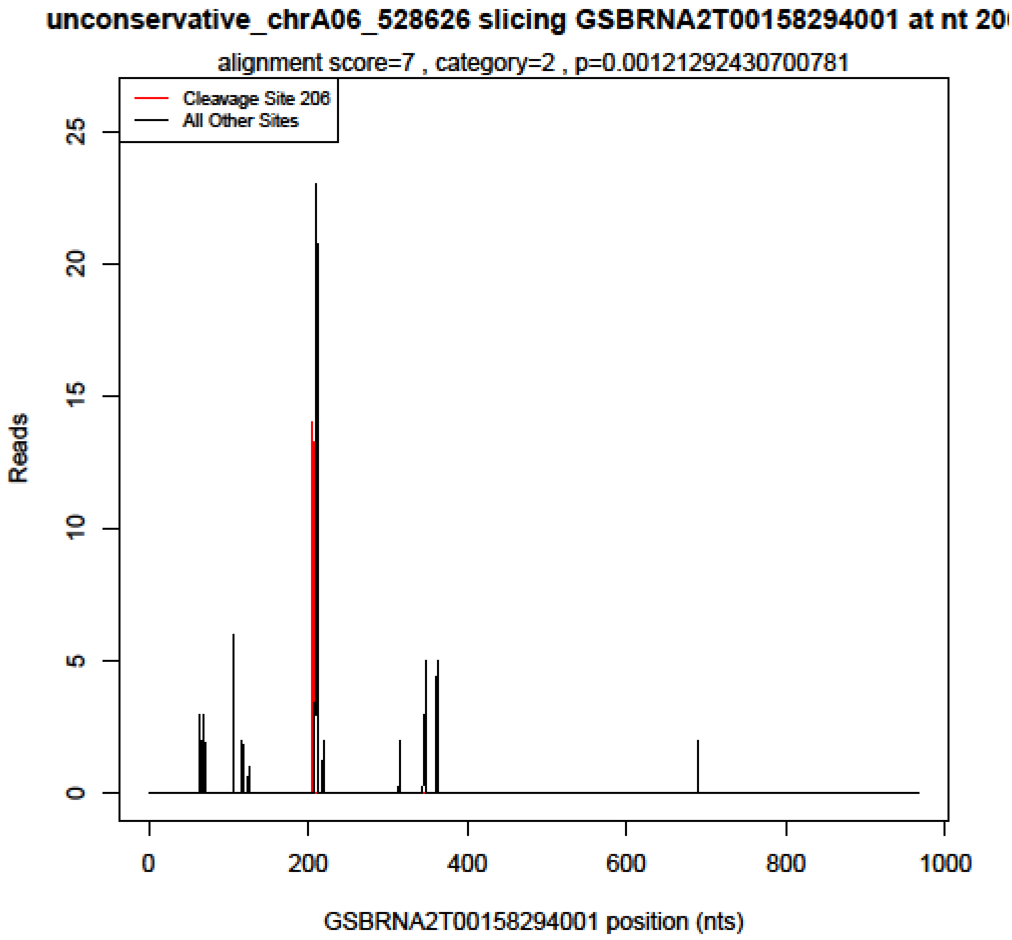

unconservative_chrA06_528626 slicing GSBRNA2T00158294001 at nt 20
alignment score=7 , category=2 , p=0.00121292430700781
Cleavage Site 206
All Other Sites
Reads
0
5
10
15
20
25
0
200
400
600
800
1000
GSBRNA2T00158294001 position (nts)

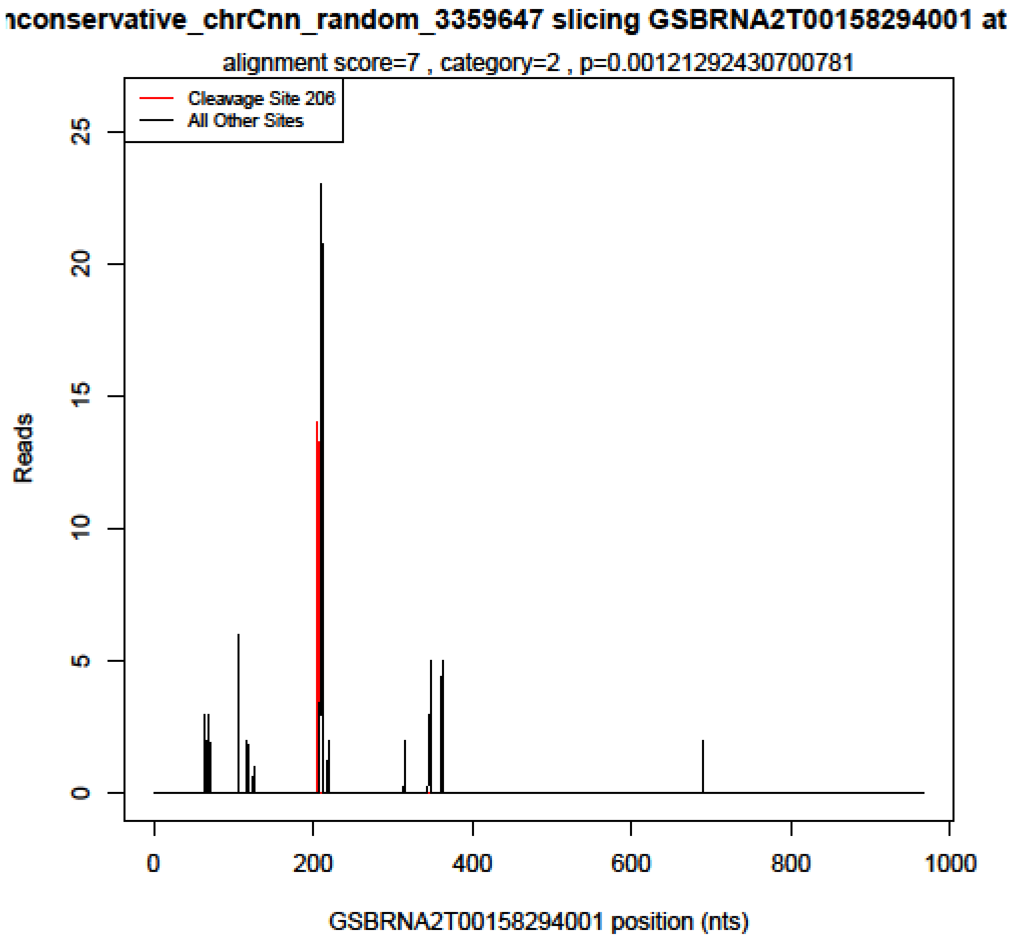

nconservative_chrCnn_random_3359647 slicing GSBRNA2T00158294001 at
alignment score=7 , category=2 , p=0.00121292430700781
Cleavage Site 206
All Other Sites
Reads
0
5
10
15
20
25
0
200
400
600
800
1000
GSBRNA2T00158294001 position (nts)
