# Supplementary material for: Small RNA and degradome profiling involved in seed development and oil synthesis of Brassica napus
Source: PLoS One. 2018 Oct 17;13(10):e0204998. doi: 10.1371/journal.pone.0204998 (PMC6192625; doi:10.1371/journal.pone.0204998)
Supplement: S2 Fig — (PDF) [file pone.0204998.s002.pdf]

Provisional ID	:	chrA01_9951
Score total	:	1.3
Score for star read(s)	:	3.9
Score for read counts	:	-2.8
Score for mfe	:	0.3
Score for randfold	:	0
Score for cons. seed	:	
Total read count	:	6
Mature read count	:	5
Loop read count	:	0
Star read count	:	1

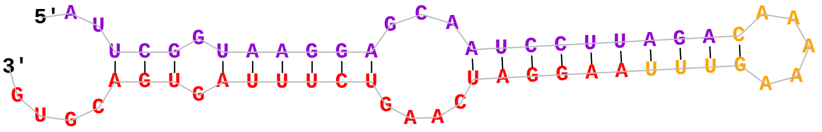

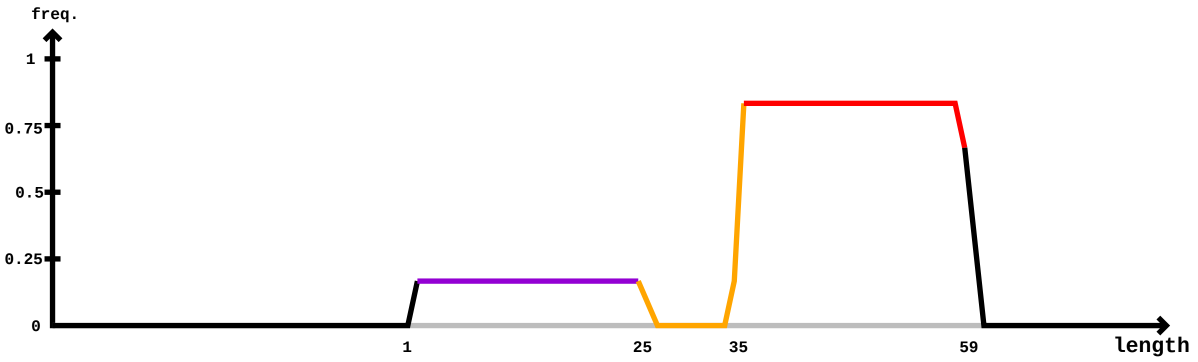

Star Mature

```
5'- aggcgaauccagauaaaauaauauuaucaaaauucaauucgguaaggagcaauccuuagacaaaaaguuuaaggaucaagucuuuagugacguggccacgucucauuggaugga -3'   obs
    aggcgaauccagauaaaauaauauuaucaaaauucaauucgguaaggagcaauccuuagacaaaaaguuuaaggaucaagucuuuagugacguggccacgucucauuggaugga        exp
    .(((.......(((((.......)))))..........(((.((((((...((((((((((.....))))))))))....)))))).)))....))).(((((....)))))..   reads   mm   sample
    ....................................auucgguaaAgagcaauccuuaga......................................................   1       1    S04
    .....................................................................uaaggaucaagucuuuagugacgu.....................   1       0    S04
    ......................................................................aaggaucaagucuuuagugacgug....................   3       0    S04
    ......................................................................aaggaucaagucuuuagugacguU....................   1       1    S04
```

Provisional ID : chrA01_14644
Score total : 1.1
Score for star read(s) : -1.3
Score for read counts : 0
Score for mfe : 2.4
Score for randfold : 0
Score for cons. seed :
Total read count : 12
Mature read count : 12
Loop read count : 0
Star read count : 0

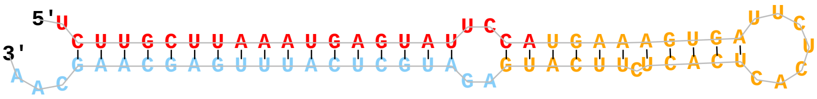

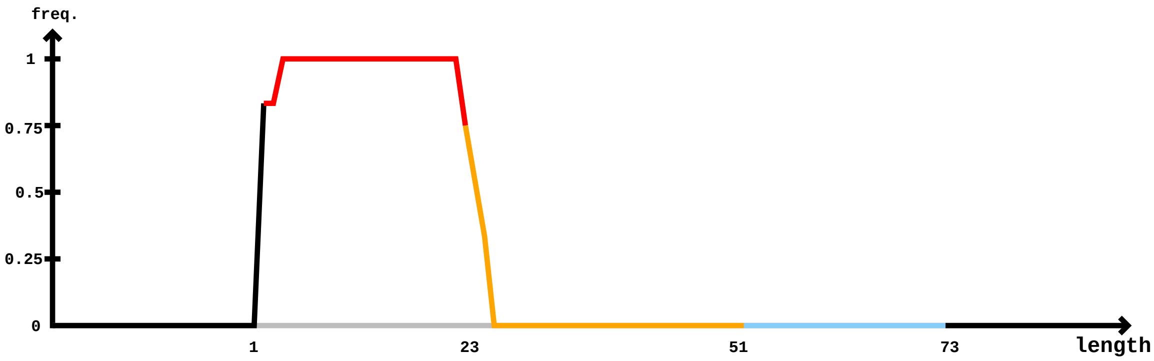

Mature Star

```
5'- ccuuccgcuuucccaauguuucuugcuuaaaugaguauuccaugaaagugauucucacucacucuucaugagaugcucauuugagcaagcaacguuuggaaguggaacua   -3'   exp
    ..((((((((.((.((((((.(((((((((((((((((..(((((((((((.......))))).))))))..))))))))))))))))).)))))).))))))))))...   reads   mm   sample
    ....................ucuugcuuaaaugaguauucca....................................................................   4       0    S03

    ....................ucuugcuuaaaugaguauucc.....................................................................   1       0    S04
    ....................ucuugcuuaaaugaguauucA.....................................................................   1       1    S04
    ....................ucuugcuuaaaugaguauucU.....................................................................   1       1    S04
    ....................ucuugcuuaaaugaguauucca....................................................................   1       0    S04
    ....................ucuugcuuaaaugaguauuccaug..................................................................   2       0    S04
    ......................uugcuuaaaugaguauuccaug..................................................................   2       0    S04
```

Provisional ID : chrA01_32285
Score total : 0
Score for star read(s) : -1.3
Score for read counts : 0
Score for mfe : 1.3
Score for randfold : 0
Score for cons. seed :
Total read count : 41
Mature read count : 41
Loop read count : 0
Star read count : 0

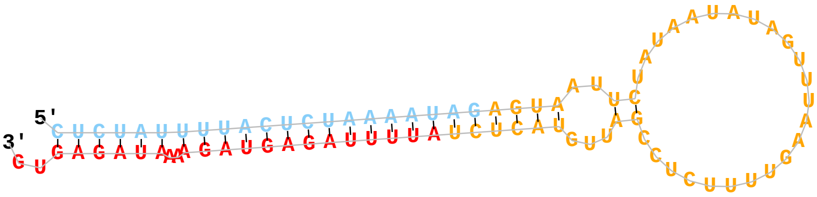

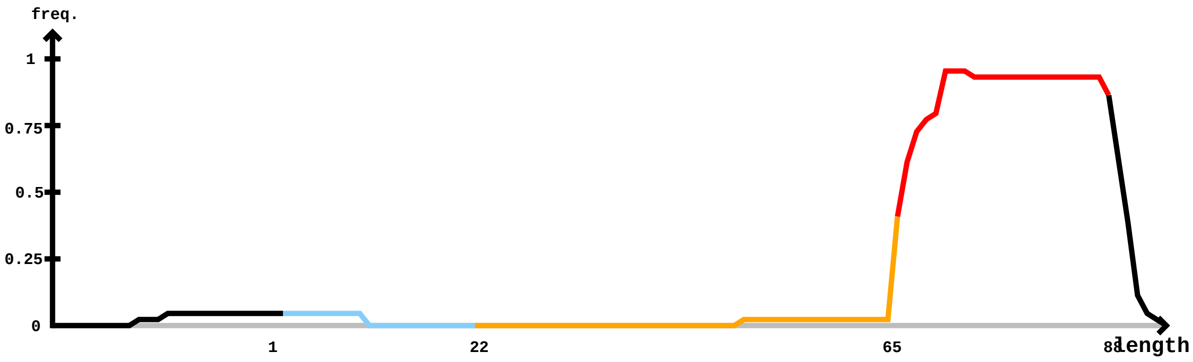

Star Mature

```
5'- uaagaccaucuccaauggcuuacucuauuuuacucuaaaauagaguaauucuauaauauaguuuaaguuuucuccgauuguacucuauuuuagaguagaaaauagagugaugaa -3' exp
    .....((((.....)))).((((((((((((((((((((((((((((..((........................))...)))))))))))))))))))..)))))))))....  reads  mm  sample
    .......aucuccaauggcuuacucuauuuu...................................................................................  1  0  S03
    ..........uccaauggcuuacucuauuuu...................................................................................  1  0  S03
    ......................................................................................auuuuagaguagaaaauagagug.....  7  0  S03
    ......................................................................................auuuuagaguagaaaauagaguga....  1  0  S03
    .......................................................................................uuuuagaguagaaaauagagug.....  1  0  S03
    .......................................................................................uuuuagaguagaaaauagagugau...  1  0  S03
    ........................................................................................uuuagaguagaaaauagagugau...  1  0  S03

    ......................................................................ucuccgauuguacucuauuuuaga....................  1  0  S04
    ......................................................................................auuuuagaguagaaaauagagu......  1  0  S04
    ......................................................................................auuuuagaguagaaaauagagG......  1  1  S04
    ......................................................................................auuuuagaguagaaaauagagug.....  6  0  S04
    ......................................................................................auuuuagaguagaaaauagagugU....  1  1  S04
    .......................................................................................uuuuagaguagaaaauagagu......  1  0  S04
    .......................................................................................uuuuagaguagaaaauagagug.....  1  0  S04
    .......................................................................................uuuuagaguagaaaauagagugau...  3  0  S04
    .......................................................................................uuuuagaguagaGaauagagugau...  1  1  S04
    .......................................................................................uuuuagaguagaaaauagagugaC...  1  1  S04
    ........................................................................................uuuagaguagaaaauagagug.....  1  0  S04
    ........................................................................................uuuagaguagaaaauGgagug.....  1  1  S04
    ........................................................................................uuuagaguagaaaauagagugau...  1  0  S04
    ........................................................................................uuuagaguagaaaauagagugaug..  1  0  S04
    .........................................................................................uuagaguagaaaauagagugau...  1  0  S04
    .........................................................................................uuagaguagaaaauagagugauga.  1  0  S04
    ..........................................................................................uagaguagaaaauagagugaugaa  1  0  S04
    ...........................................................................................agaguagaaaauagagug.....  2  0  S04
    ...........................................................................................agaguagaaaauagagugau...  3  0  S04
    ...........................................................................................agaguagaaaauagagugauA..  1  1  S04
    ...........................................................................................agaguagaaaauagagugaug..  1  0  S04
```

Provisional ID	:	chrA01_34930
Score total	:	0.2
Score for star read(s)	:	-1.3
Score for read counts	:	0
Score for mfe	:	1.5
Score for randfold	:	0
Score for cons. seed	:	
Total read count	:	13
Mature read count	:	13
Loop read count	:	0
Star read count	:	0

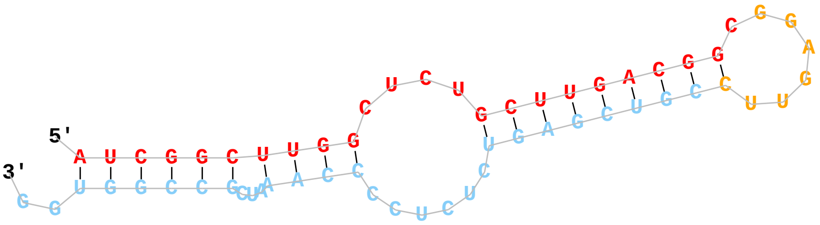

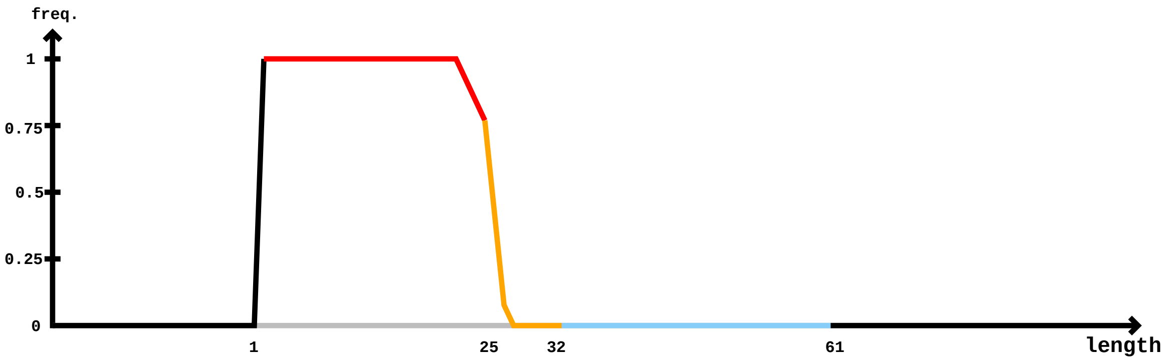

Mature **Star**

```
5'- uccuucgugaacuguucuucaucggcuuggcucugcuugacggcggaguuccgucgagucucuccccaaaucgccgguggaaggugguc ugcucucuccuugacgcguguc -3'    exp
    ......((..((..(..(((((((((((((....(((((((((.......)))))))))......))))...)))))))))..)..))..)).........(((....)))   reads   mm   sample
    ....................aucggcuuggcucugcuugac......................................................................   1       0    S03
    ....................aucggcuuggcucugcuugacg.....................................................................   1       0    S03
    ....................aucggcuuggcucugcuugacgg....................................................................   1       0    S03
    ....................Gucggcuuggcucugcuugacggc...................................................................   1       1    S03
    ....................aucggcuuggcucugcuugacggc...................................................................   2       0    S03
    ....................aucggcuuggcucugcuugacggcgU.................................................................   1       1    S03

    ....................aucggcuuggcucugcuugacggc...................................................................   3       0    S04
    ....................aucggcuuggcucugcuugGcggc...................................................................   1       1    S04
    ....................aucggcuuggcucugcuugacggU...................................................................   1       1    S04
    ....................aucggcAuggcucugcuugacggc...................................................................   1       1    S04
```

Provisional ID	:	chrA01_46199
Score total	:	33.5
Score for star read(s)	:	3.9
Score for read counts	:	28.2
Score for mfe	:	1.5
Score for randfold	:	0
Score for cons. seed	:	
Total read count	:	67
Mature read count	:	59
Loop read count	:	0
Star read count	:	8

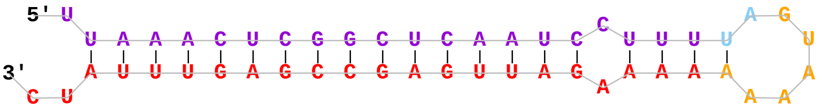

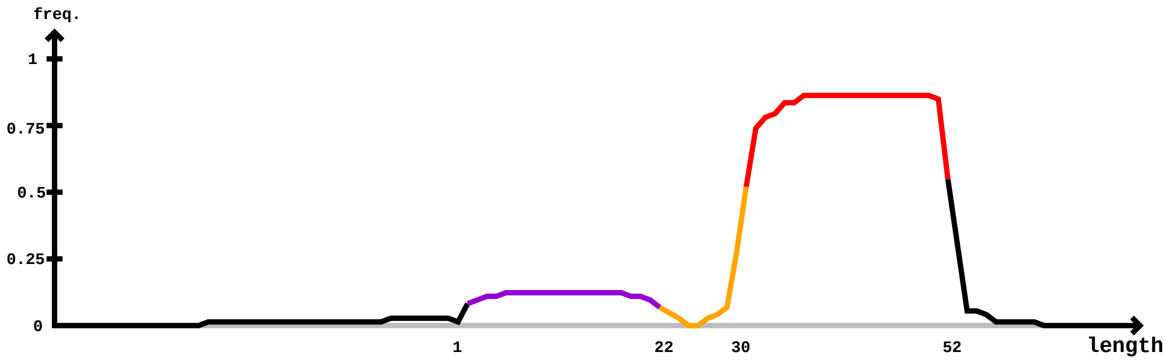

Star Mature

```
5'- uagauuaguaauucacguccguuuuauuaguuuaauugcaauuaaacucggcucaauccuuuuaguaaaaaaaagauugagccgaguuuaucuaguguauauacuguuuuu  -3'  obs
    uagauuaguaauucacguccguuuuauuaguuuaauugcaauuaaacucggcucaauccuuuuaguaaaaaaaagauugagccgaguuuaucuaguguauauacuguuuuu       exp
    (((((((((((..(......)..)))))))))))..((((..((((((((((((((((.((((......)))).))))))))))))))))).....))))............   reads   mm   sample
    .................................aauCgcaauuaaacucggcucaauc.....................................................   1       1    S03
    .........................................uuaaacucggcucaauccuuu.................................................   3       0    S03
    .........................................uuaaacucggcucaauccuuuua...............................................   1       0    S03
    ...........................................aaacucggcucaauccuu..................................................   1       0    S03
    .............................................acucggcucaauccuuuua...............................................   1       0    S03
    ..................................................................aaaaaaaagauugagccgaguuuaC....................   1       1    S03
    ..................................................................aaaaaaaagauugagccgaguuuauc...................   1       0    S03
    ...................................................................aaaaaaagauugagccgaguuuaA....................   1       1    S03
    ....................................................................aaaaaagauugagccgaguuuauA...................   1       1    S03
    .....................................................................aaaaagauugagccgaguuuau....................   1       0    S03
    .....................................................................aaaaagauugagccgaguuuaA....................   2       1    S03
    .....................................................................aaaaagauugagccgaguuuauA...................   2       1    S03
    ......................................................................aaaagauugagccgaguuuau....................   3       0    S03
    ......................................................................aaaagauugagccgaguuuauc...................   1       0    S03
    ......................................................................aaaagauugagccgaguuuauA...................   1       1    S03
    ......................................................................aaaagauugagccgaguuuaucuagu...............   1       0    S03
    .......................................................................aaagauugagccgaguuuauA...................   4       1    S03
    .........................................................................agauugagccgaguuuauA...................   1       1    S03
    ............................................................................uugagccgaguuuaucuagu...............   1       0    S03

    ..............acguccguuuuauuaguuuaauugca.......................................................................   1       0    S04
    .........................................uuaaacucggcucaaucUu...................................................   1       1    S04
    ..........................................uaaacucggcucaauccuu..................................................   1       0    S04
    ...................................................................aaaaaaagauugagccgaguuua.....................   1       0    S04
    .....................................................................aaaaGgauugagccgaguuuau....................   6       1    S04
    .....................................................................aaaaGgauugagccgaguuuauc...................   4       1    S04
    ......................................................................aaaGgauugagccgaguuuau....................   4       1    S04
    ......................................................................aaaGgauugagccgaguuuauc...................   7       1    S04
    ......................................................................aaaGgauugagccgaguuuaucu..................   1       1    S04
    .......................................................................aaGgauugagccgaguuuau....................   2       1    S04
    .......................................................................aaGgauugagccgaguuuauc...................   6       1    S04
    .......................................................................aaGgauugagccgaguuuaucu..................   3       1    S04
    .......................................................................aaGgauugagccgaguuuaucuag................   1       1    S04
```

Star **Mature**

uagauuaguaauucacguccguuuuauuaguuuaauugcaauuaaaacucggcucaauccuuuuaguaaaaaaaagauugagccgaguuuaucuaguguauauacuguuuuu			
....aGgauugagccgaguuuau....................	2	1	S04
....aGgauugagccgaguuuauc...................	1	1	S04
....gauugagccgaguuuauA...................	3	1	S04
....uugagccgaguuuaucuaguguaua..........	1	0	S04

Provisional ID	:	chrA01_60332
Score total	:	1.5
Score for star read(s)	:	3.9
Score for read counts	:	-4.9
Score for mfe	:	2.6
Score for randfold	:	0
Score for cons. seed	:	
Total read count	:	2
Mature read count	:	1
Loop read count	:	0
Star read count	:	1

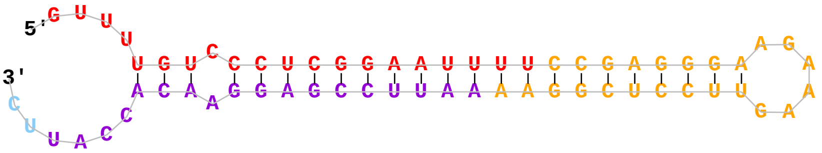

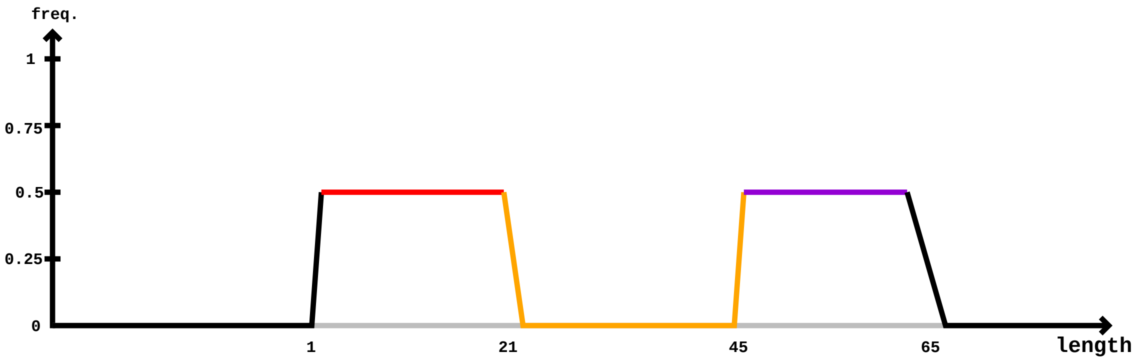

Mature Star

```
5'- aaaauuccgaggaaaauuccgagggaguuuugucccucggaauuuuccgagggaagaaaguuccucggaaaauuccgaggaacaccauucccucggaauuucgaaaaa   -3'   obs
    aaaauuccgaggaaaauuccgagggaguuuugucccucggaauuuuccgagggaagaaaguuccucggaaaauuccgaggaacaccauucccucggaauuucgaaaaa         exp
    .......(((....((((((((((((((..(((.((((((((((((((((((((......)))))))))))))))))))).)))..))))))))))))))))).....    reads   mm   sample
    ..........................guuuuguccGucggaauuuu..............................................................    1       1    S03

    ......................................................................aauuccgaggaacaccau....................    1       0    S04
```

Provisional ID	:	chrA01_61766
Score total	:	0.4
Score for star read(s)	:	3.9
Score for read counts	:	-4.9
Score for mfe	:	1.5
Score for randfold	:	0
Score for cons. seed	:	
Total read count	:	2
Mature read count	:	1
Loop read count	:	0
Star read count	:	1

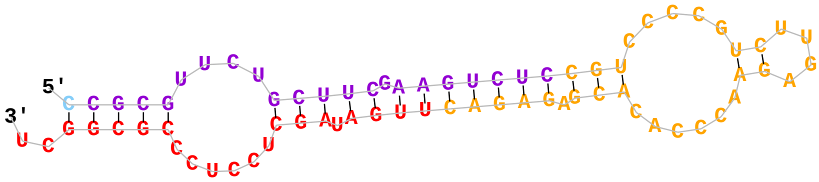

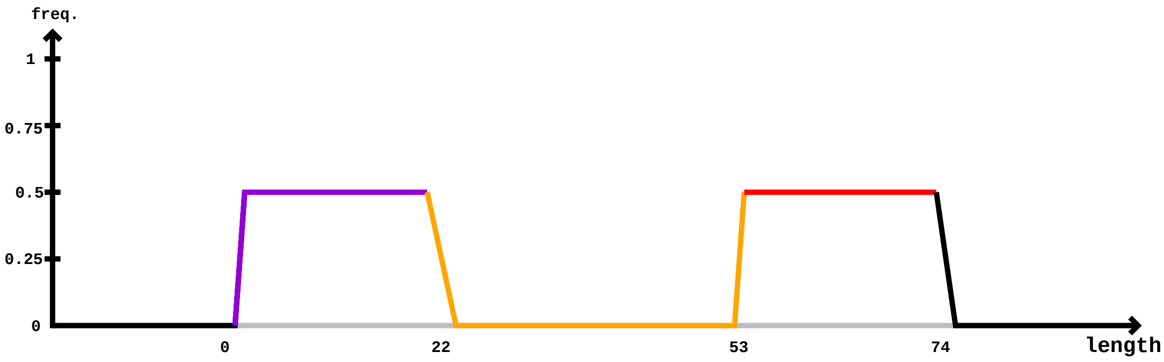

Star Mature

```
5'- gcugagcaacgcgcacgccgcguucugcuucgaagucuccguccccgucuugagaacccacacgagagacuugauagcuccucccgcggcuaucacagcuuuguucugguc   -3'   obs
    gcugagcaacgcgcacgccgcguucugcuucgaagucuccguccccgucuugagaacccacacgagagacuugauagcuccucccgcggcuaucacagcuuuguucugguc         exp
    ((((((((.((....(((((....(((((.((((((((((.....((....))......))).))))))))).)))......)))))).......)).)))))).))).   reads   mm   sample
    ..................cgcguucugcuucgaagucuc........................................................................   1       0    S03

    ......................................................................uugauagcuccucccgcggcu....................   1       0    S04
```

Provisional ID	:	chrA01_64399
Score total	:	1
Score for star read(s)	:	-1.3
Score for read counts	:	0
Score for mfe	:	2.3
Score for randfold	:	0
Score for cons. seed	:	
Total read count	:	473
Mature read count	:	461
Loop read count	:	12
Star read count	:	0

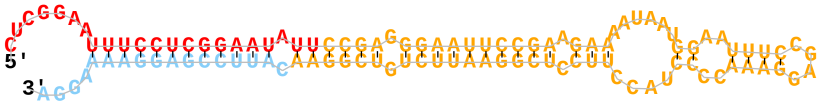

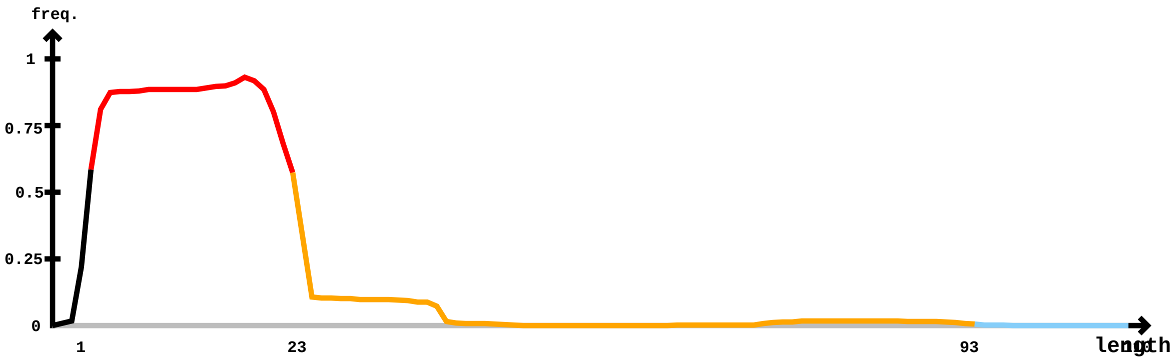

Mature **Star**

```
5'- uccucggaauuuccucggaauauuccgagggaauuccgaagaaaauaauggaauuuccgaggaaaccccuaccuuccucggaauucugucggaacauuccgaggaaaaggac  -3'   exp
    ((((.....((((((((((((.((((((.((((((((((.(((......((..((((....))))..))....))).)))))))))).)))))).))))))))))))))))).  reads   mm   sample
    uccucggaauuuccucggaa............................................................................................  1       0    S03
    uccucggaauuuccucggaaua..........................................................................................  1       0    S03
    uccucggaauuuccucggaauauC........................................................................................  1       1    S03
    .ccucggaauuuccucgga.............................................................................................  7       0    S03
    .ccucggaauuuccucggaa............................................................................................  1       0    S03
    .ccucggaauuuccucggaau...........................................................................................  4       0    S03
    .ccucggaauuuccucggaaua..........................................................................................  4       0    S03
    .Acucggaauuuccucggaauau.........................................................................................  1       1    S03
    .ccucggaauuuccucggaauau.........................................................................................  9       0    S03
    .ccucggaauuuccucggaauaA.........................................................................................  1       1    S03
    .ccucggaauuuccucggaauauu........................................................................................  9       0    S03
    .ccucggaauuuccucggaauauuc.......................................................................................  12      0    S03
    ..cucggaauuuccucggaa............................................................................................  7       0    S03
    ..cucggaauuuccucggaau...........................................................................................  8       0    S03
    ..cucggaauuuccucggaaC...........................................................................................  1       1    S03
    ..cucggaauuuccucggaaua..........................................................................................  13      0    S03
    ..cucggaauuuccucggaauau.........................................................................................  8       0    S03
    ..cucggaauuuccucggaauauu........................................................................................  39      0    S03
    ..cucggaauuuccucggaauauC........................................................................................  4       1    S03
    ..cucggaauuuccucggaauauuc.......................................................................................  5       0    S03
    ...ucggaauuuccucggaau...........................................................................................  11      0    S03
    ...ucggaauuuccucggaaua..........................................................................................  4       0    S03
    ...ucggaauuuccucggaauau.........................................................................................  4       0    S03
    ...ucggaauuuccucggaauauu........................................................................................  34      0    S03
    ...ucggaauuuccucggaauauC........................................................................................  3       1    S03
    ...ucggaauuuccCcggaauauuc.......................................................................................  1       1    S03
    ....cggaauuuccucggaaua..........................................................................................  4       0    S03
    ....cggaauuuccucggaauau.........................................................................................  2       0    S03
    ....cggaauuuccucggaauauu........................................................................................  12      0    S03
    ....cggaauuuccucggaauauC........................................................................................  2       1    S03
    ....cggaauuuccCcggaauauu........................................................................................  1       1    S03
    ....cggaauuuccucggaauauuc.......................................................................................  1       0    S03
    .....ggaauuuccucggaauau.........................................................................................  2       0    S03
    ......aauuuccucggaauauuccga.....................................................................................  1       0    S03
```

Mature **Star**

```
uccucggaauuuccucggaauauuccgagggaauuccgaagaaaauaauggaauuuccgaggaaaccccuaccuuccucggaauucugucggaacauuccgaggaaaaggac
........auuuccucggaauauucc......................................................................................  1  0  S03
........auuuccucggaauauuccgagg..................................................................................  1  0  S03
..............ucCgaauauuccgagggaauu.............................................................................  1  1  S03
..............ucCgaauauuccgagggaauuc............................................................................  1  1  S03
..............ucggaauauuccgagggaauuc............................................................................  1  0  S03
...............cggaauauuccgagggaauuc............................................................................  1  0  S03
...............cCgaauauuccgagggaauuccga.........................................................................  2  1  S03
................ggaauauuccgagggaau..............................................................................  1  0  S03
.................gaauauuccgagggaauuccga.........................................................................  4  0  S03
..................aauauuccgGgggaauuccg..........................................................................  1  1  S03
..................aauauuccgagggaauuccg..........................................................................  1  0  S03
..................aauauuccgagggaauuccga.........................................................................  5  0  S03
..................aauauuccgagggaauuccgC.........................................................................  1  1  S03
..................Nauauuccgagggaauuccgaa........................................................................  1  1  S03
..................aauauuccgagggaauuccgaU........................................................................  1  1  S03
...................auauuccgagggaauuccg..........................................................................  5  0  S03
...................auauuccgagggaauuccgC.........................................................................  1  1  S03
...................auauuccgagggaauuccgU.........................................................................  1  1  S03
...................auauuccgagggaauuccga.........................................................................  3  0  S03
...................auauuccgagggaauuccgG.........................................................................  1  1  S03
...................auauuccgagggaauuccgaUg.......................................................................  1  1  S03
....................uauuccgagggaauuccU..........................................................................  1  1  S03
....................uauuccgagggaauuccga.........................................................................  3  0  S03
.....................auuccgagggaauuccga.........................................................................  1  0  S03
.....................auuccgagggaauuccgG.........................................................................  1  1  S03
.....................auuccgagggaauuccgaa........................................................................  1  0  S03
.....................auuccgaCggaauuccgaagaaaa...................................................................  1  1  S03
......................uuccgaCggaauuccgaagaaaau..................................................................  1  1  S03
........................ccgaCggaauuccgaagaaaaua.................................................................  1  1  S03
.........................cgagggaauuccgaUgaaa....................................................................  1  1  S03
...............................................................aacccUuaccuuccucggaauucu.........................  1  1  S03
........................................................................cuuccucggaauucugucA.....................  1  1  S03
........................................................................cuuccucggaauucugucggaG..................  1  1  S03
.........................................................................uuccucggaauucugucgg....................  1  0  S03
.........................................................................uuccucggaauucugucggaaG.................  1  1  S03
............................................................................cucggaauucCgucggaacauu..............  1  1  S03

uGcucggaauuuccucggaauauu........................................................................................  1  1  S04
uccucggaauuuGcucggaauauu........................................................................................  2  1  S04
ucAucggaauuuccucggaauauu........................................................................................  2  1  S04
uAcucggaauuuccucggaauauu........................................................................................  1  1  S04
.ccGcggaauuuccucgga.............................................................................................  1  1  S04
.ccucggaauuuccucgga.............................................................................................  13  0  S04
.ccucggaauuuccucggaa............................................................................................  6  0  S04
.ccucggaauuuccucggaaC...........................................................................................  1  1  S04
.ccucggaauuuccucggaau...........................................................................................  3  0  S04
.ccucggaauuuccucggaauG..........................................................................................  1  1  S04
.ccucggaauuuccucggaaua..........................................................................................  4  0  S04
.ccucggaauuuccucggaauau.........................................................................................  8  0  S04
.ccucggaauuuccucggaauauu........................................................................................  5  0  S04
.ccucggaauuuccucggaauauC........................................................................................  1  1  S04
.ccucggaauuuccucggaauauCc.......................................................................................  1  1  S04
.ccucggaauuuccucggaauauuc.......................................................................................  14  0  S04
..cucggaauuuccucggaa............................................................................................  8  0  S04
..cucggaauuuccucggaau...........................................................................................  9  0  S04
..cucggaauuuccucggaaua..........................................................................................  22  0  S04
..cucggaauuuccucggaauG..........................................................................................  1  1  S04
..cucggaauuuccucggaauau.........................................................................................  16  0  S04
..cucggaauuuccucggaauauu........................................................................................  40  0  S04
..Nucggaauuuccucggaauauu........................................................................................  1  1  S04
..cucggaauuuccucggaauauC........................................................................................  4  1  S04
..cucggaauuuccucggaauauuc.......................................................................................  3  0  S04
..cucggaauuuccucggaaCauuc.......................................................................................  1  1  S04
..cucggaauuuccucggaauauucc......................................................................................  1  0  S04
...ucggaauuuccucggaau...........................................................................................  11  0  S04
...ucggaauuuccucggaaua..........................................................................................  7  0  S04
...ucggaauuuccucggaauau.........................................................................................  4  0  S04
...ucggaauuuccucggaauauC........................................................................................  1  1  S04
...Ncggaauuuccucggaauauu........................................................................................  1  1  S04
...ucggaaCuuccucggaauauu........................................................................................  1  1  S04
```

Mature **Star**

uccucggaauuuccucggaauauuccgagggaauuccgaagaaaauaauggaauuuccgaggaaaccccuaccuuccucggaauucugucggaacauuccgaggaaaaggac			
...ucggaauuuccucggaauauu..	32	0	S04
...ucggaauCuccucggaauauu..	1	1	S04
...ucggaauuuccucggaauauuc...	3	0	S04
....cggaauuuccucggaaua..	2	0	S04
....cggaauuuccucggaauau...	2	0	S04
....cggaauuuccucggaauauu..	6	0	S04
....cggaauuuccucggaauauuc...	1	0	S04
........auuuccucggaauauuccgagU..	1	1	S04
.................gaauauuccgagggaauuccga...	2	0	S04
..................aauaAuccgagggaauuccga...	1	1	S04
...................auauuccgagggaauuccga...	2	0	S04
....................uauuccgagggaauuccga...	2	0	S04
....cuuccucggaauucugucgga...................	1	0	S04
....uccucggaauucugucgga...................	1	0	S04
....cucggaauucugucggaac.................	1	0	S04

Provisional ID	:	chrA01_64877
Score total	:	1.6
Score for star read(s)	:	3.9
Score for read counts	:	-2.8
Score for mfe	:	0.7
Score for randfold	:	0
Score for cons. seed	:	
Total read count	:	6
Mature read count	:	5
Loop read count	:	0
Star read count	:	1

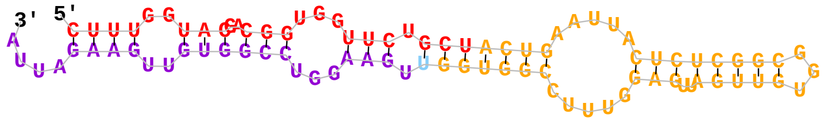

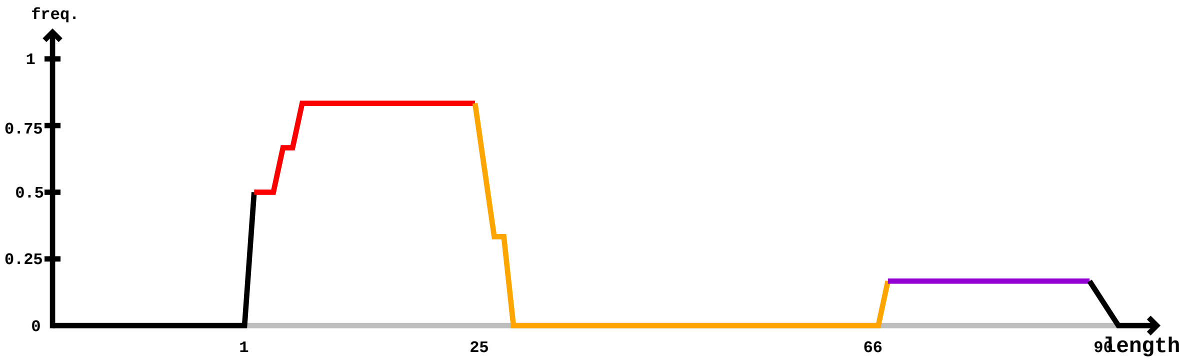


```
                       Mature                                          Star
5'- agagaccguuuugcuuagucuuugguacgacggugguucugcuacugaauuacucucggcgguguugauugagguuuccgguggugaagguccgguguugaagauuaauuaa   -3'    obs
    agagaccguuuugcuuagucuuugguacgacggugguucugcuacugaauuacucucggcgguguugauugagguuuccgguggcuugaagguccgguguugaagauuaauuaa          exp
    ..............(((((((((..(((..(((...(((.(((((((.....((((((((...)))))..))).....))))))).)))...))))))..)))))))))....   reads      mm         sample
    ......................ugguacgacggugguucugcuacG...................................................................   1          1          S03
    .....................................................................................ugaagguccgguguugaagauuC.....   1          1          S03

    ...................cuuugguacgacggugguucugcC......................................................................   2          1          S04
    ...................cuuugguacgacggugguucugcu......................................................................   1          0          S04
    ........................guacgacggugguucugcuacu...................................................................   1          0          S04
```

Provisional ID	:	chrA01_84314
Score total	:	1
Score for star read(s)	:	-1.3
Score for read counts	:	0
Score for mfe	:	2.3
Score for randfold	:	0
Score for cons. seed	:	
Total read count	:	19
Mature read count	:	19
Loop read count	:	0
Star read count	:	0

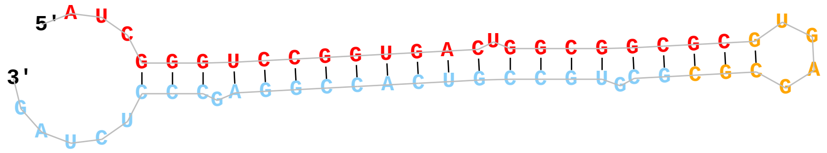

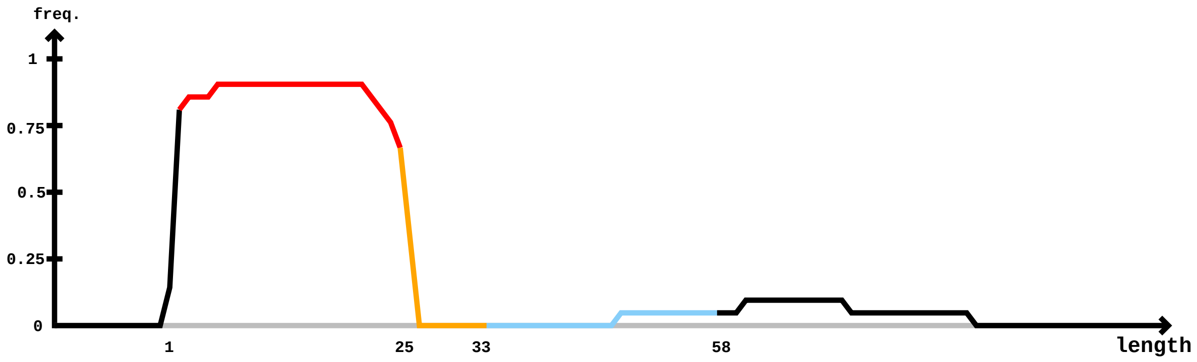

Mature Star

```
5'- ggggaucuguaaucggguccggugacuggcggcgcgugagcgcgcgugccgucaccggagcccucuagccaccagcaaagcuguugauuucugcuucggucuugucuccuuucc   -3'    exp
    ((((((........((((((((((((.(((((((((....))))).))))))))))))).)))......((.((((...)))).))......((....))...)))))).....    reads      mm       sample
    ..........aaucggguccggugacuggcgA..................................................................................    1          1        S03
    ...........aucggguccggugacuggcggcgc...............................................................................    1          0        S03
    ...........aucggguccggugacuggcggcgU...............................................................................    1          1        S03
    .........................................................gagcccucuagccaccagUaaagc.................................    1          1        S03

    ..........aaucggguccggugacuggcggcA................................................................................    1          1        S04
    ..........aaucggguccggugacuggcggcg................................................................................    1          0        S04
    ...........aucggguccggugacuggcggcgU...............................................................................    3          1        S04
    ...........aucggguccggugacuggAggcgc...............................................................................    1          1        S04
    ...........aucggguccggugacGggcggcgc...............................................................................    1          1        S04
    ...........aucUgguccggugacuggcggcgc...............................................................................    1          1        S04
    ...........aucggguccggugacuggcggcgc...............................................................................    6          0        S04
    ............ucAgguccggugacuggcg...................................................................................    1          1        S04
    ...............gguUcggugacuggcggc.................................................................................    1          1        S04
    ......................................................................accagcaaagcuguugauuucugc....................    1          0        S04
```

Provisional ID	:	chrA01_89350
Score total	:	0.3
Score for star read(s)	:	-1.3
Score for read counts	:	0
Score for mfe	:	1.6
Score for randfold	:	0
Score for cons. seed	:	
Total read count	:	13
Mature read count	:	13
Loop read count	:	0
Star read count	:	0

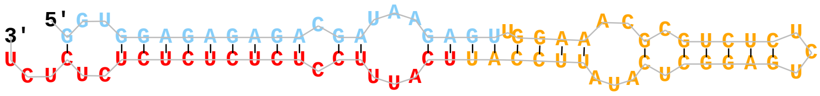

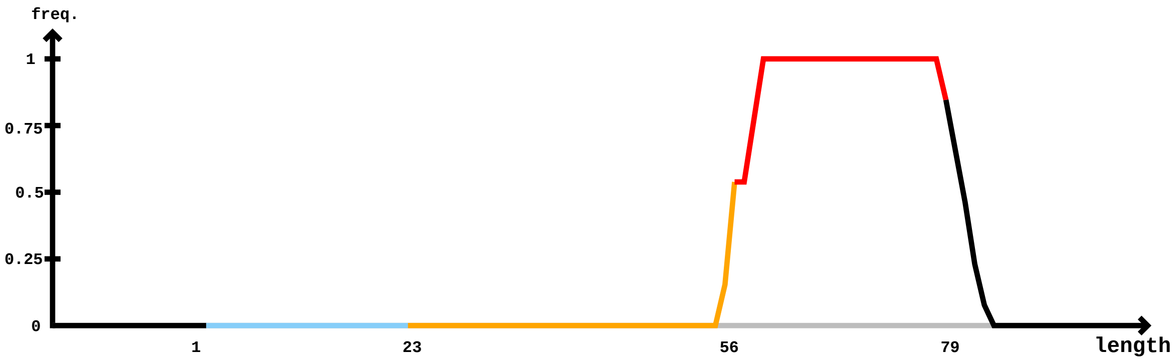


```
                    Star                                            Mature
5'- ggaaagugggggugguggagagagacgauaagaguuggaaacgcgucucucugaggcucauauuccauucauuuccucucucucucucucucucucucucucucucucucu   -3'    exp
    (((.((.(((((..((.(((((((((.((...((((.((((..(.(((((...))))).)...))))))))...)).)))))))))..))..))))).)).)))........    reads    mm    sample
    ...................................................................Cucauuuccucucucucucucucu....................    1        1     S03
    ....................................................................ucauuuccucucucucucucuc.....................    1        0     S03
    ....................................................................ucauuuccucucucucucucucu....................    1        0     S03
    ....................................................................ucauuuccucucucucucucucucu..................    1        0     S03
    ......................................................................auuuccucucucucucucucucu..................    1        0     S03
    ......................................................................auuuccCcucucucucucucucu..................    1        1     S03
    .......................................................................uuuccucucucucucucucucucuG.................    1        1     S03

    ...................................................................Cucauuuccucucucucucucuc.....................    1        1     S04
    ....................................................................ucauuuccucucucucucucucG....................    2        1     S04
    ......................................................................auuuccucucucucucucucA....................    1        1     S04
    .......................................................................uuuccucucucucucucucucucuG.................    1        1     S04
    .......................................................................uuuccucucucucucucucucucuAu................    1        1     S04
```

Provisional ID	:	chrA01_90044
Score total	:	0
Score for star read(s)	:	3.9
Score for read counts	:	-4.4
Score for mfe	:	0.5
Score for randfold	:	0
Score for cons. seed	:	
Total read count	:	3
Mature read count	:	2
Loop read count	:	0
Star read count	:	1

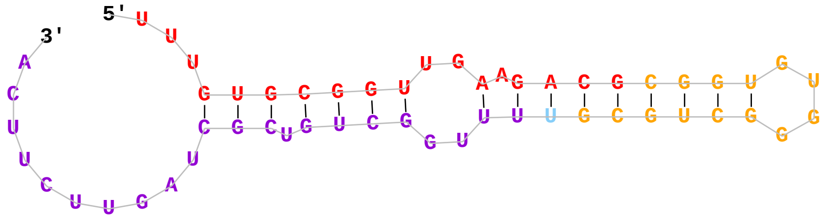

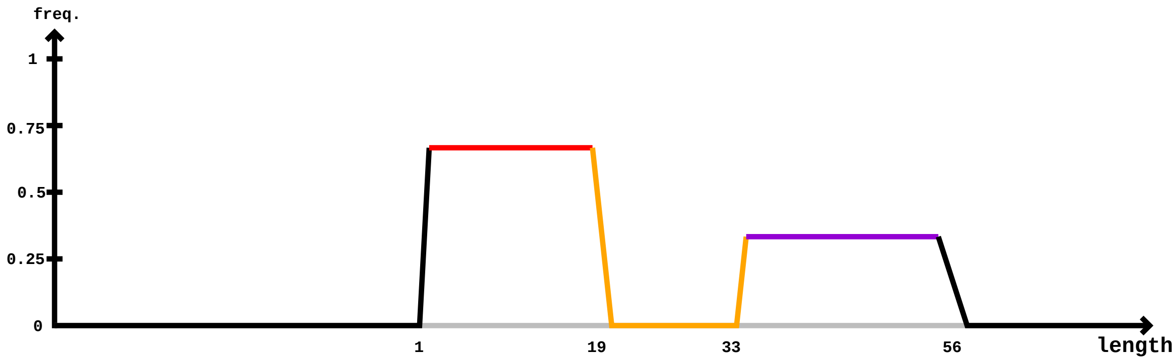

Mature Star

```
5'- ugucggugugugguugguuugucguauaugugaggauuuugugcgguugaagacgcggugugggcugcguuuuggcugucgcuaguucuucauuuguuuuuucuuuuuagau  -3'   obs
    ugucggugugugguugguuugucguauaugugaggauuuugugcgguugaagacgcggugugggcugcguuuuggcugucgcuaguucuucauuuguuuuuucuuuuuagau         exp
    .......((.(((..((........(((.(((((((....(((((((..(.((((((((....)))))))))..)))).)))....))))))).))).....))..))).))   reads   mm   sample
    ....................................uuugAgcgguugaagacg.........................................................   1       1    S03
    .....................................................................uuuggcugucgcuaguucuuca....................   1       0    S03

    ....................................uuugAgcgguugaagacg.........................................................   1       1    S04
```

Provisional ID	:	chrA01_random_2678411
Score total	:	0.2
Score for star read(s)	:	-1.3
Score for read counts	:	0
Score for mfe	:	1.5
Score for randfold	:	0
Score for cons. seed	:	
Total read count	:	54
Mature read count	:	54
Loop read count	:	0
Star read count	:	0

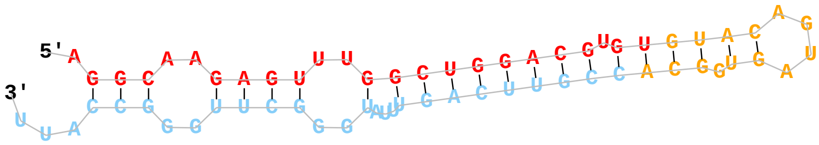

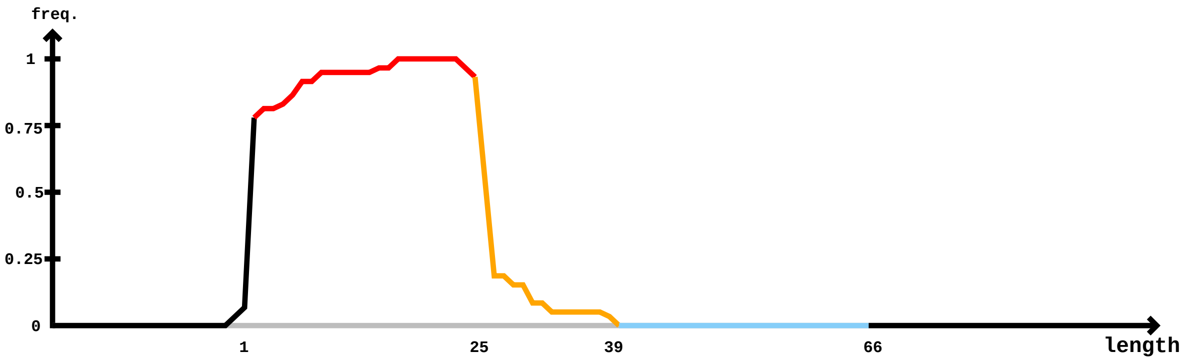

Mature Star

```
5'- agggccucaacacgguggauaggcaagaguuuggcuggacgugugguacaguaguggcaccguucaguuuaugggcuugggccauuggguuuguuucgguuugccuuugaugggc   -3'    exp
    .(((((..((((..(.(((.(((..((((..(((((((((.((((((....)).))))))))))))...)..))))..))))))).)...))))..)))))((((.....))))    reads   mm   sample
    ..................Guaggcaagaguuuggcuggacgu........................................................................    1       1    S03
    ....................aggcaagaguuuggcuggacgugu......................................................................    16      0    S03
    ...........................aguuuggcuggacguguguacagu...............................................................    2       0    S03
    ...................................uggacguguguacaguaguggca........................................................    2       0    S03

    ..................Guaggcaagaguuuggcuggacgu........................................................................    1       1    S04
    ...................uaggcaagaguuuggcuggacgug.......................................................................    2       0    S04
    ....................aggcaagaguuuggcuggacgugC......................................................................    1       1    S04
    ....................aggcaagaguuuggcuggacgugu......................................................................    22      0    S04
    ....................aggcaagaguuuggcuggacgugG......................................................................    1       1    S04
    ....................aggcaagaguuuggcuggacgugA......................................................................    2       1    S04
    .....................ggcaagaguuuggcuggacgugu......................................................................    1       0    S04
    .....................ggcaagaguuuggcuggacgugug.....................................................................    1       0    S04
    .......................caagaguuuggcuggacguguguG...................................................................    1       1    S04
    ........................aagaguuuggcuggacgugugCa...................................................................    1       1    S04
    ........................aagaguuuggcuggacguguguaca.................................................................    1       0    S04
    .........................agaguuuggcuggacguguguacU.................................................................    1       1    S04
    .........................agaguuuggcuggacguguguaca.................................................................    2       0    S04
    .................................gcuggacgugugua Uaguaguggc.........................................................    1       1    S04
```

Provisional ID	:	chrA02_97002
Score total	:	0.4
Score for star read(s)	:	3.9
Score for read counts	:	-4.4
Score for mfe	:	0.9
Score for randfold	:	0
Score for cons. seed	:	
Total read count	:	3
Mature read count	:	1
Loop read count	:	0
Star read count	:	2

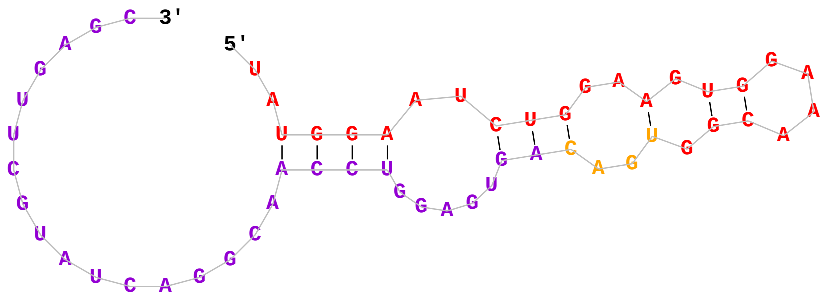

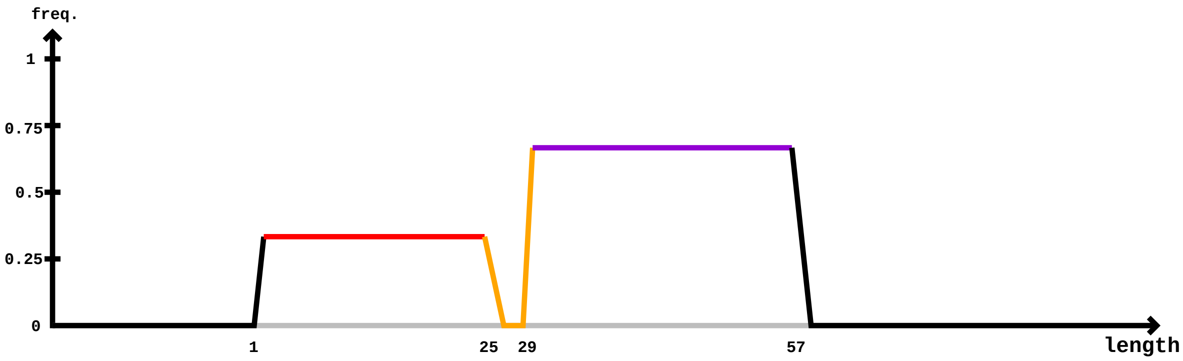

Mature Star

```
5'- gacaguauugggucugcucguauggaaucuggaaguggaaacggugacagugagguccaacggacuaugcuugagcuucucaaucaacuugauggauucgaagcgucaaacaa   -3'   obs
    gacaguauugggucugcucguauggaaucuggaaguggaaacggugacagugagguccaacggacuaugcuugagcuucucaaucaacuugauggauucgaagcgucaaacaa         exp
    (((.((.((((((((((((((((((..((((....((((.(((....).))....)))).))))))))))..))))...((((.....)))).)))))))).))))).....    reads    mm    sample
    ....................uauggaaucuggaaguggaaacgA.....................................................................    1        1     S03

    ................................................agugagguccaacggacuaugcuugagc.....................................    1        0     S04
    ................................................agugagguccaacggacuaugcuugagU.....................................    1        1     S04
```

Provisional ID	:	chrA02_99971
Score total	:	0.4
Score for star read(s)	:	3.9
Score for read counts	:	-4.9
Score for mfe	:	1.5
Score for randfold	:	0
Score for cons. seed	:	
Total read count	:	2
Mature read count	:	1
Loop read count	:	0
Star read count	:	1

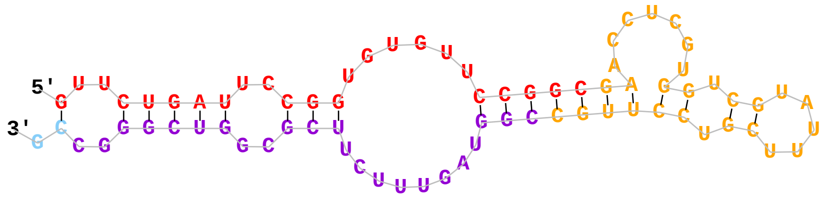

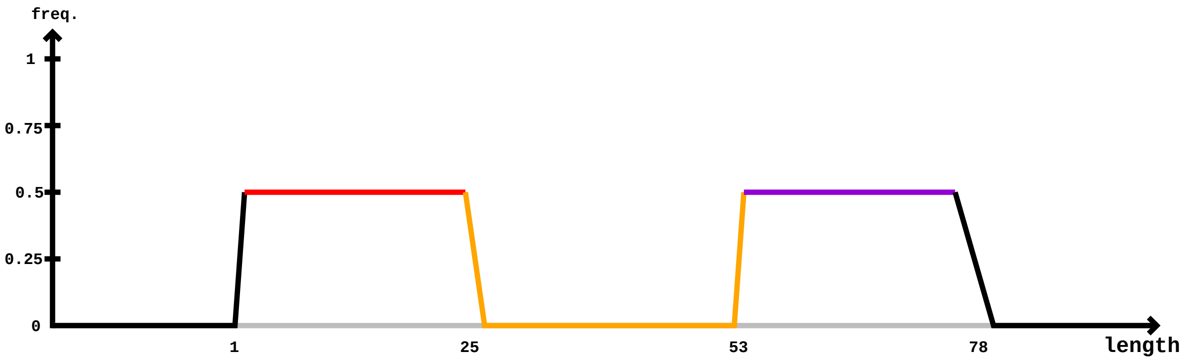

Mature Star

```
5'- guuguaucugguggugugguucugauuccgguguguuccggcgaaccucguggucguauuucguccuugccgguaguuucuucgcggucgggccgcgaucguuggaggugugu   -3'    obs
    guuguaucugguggugugguucugauuccgguguguuccggcgaaccucguggucguauuucguccuugccgguaguuucuucgcggucgggccgcgaucguuggaggugugu           exp
    ......(((..(((((((((.(((((..(((......(((((((.......((.((.....)).)))))))))........)))..)))))))))).))))..)))....... reads    mm    sample
    ..................guucugauuUcgguguguuccggc....................................................................... 1        1     S03

    ......................................................................cgguaguuucuucgcggucgggc.................... 1        0     S04
```

Provisional ID	:	chrA02_101706
Score total	:	2076.3
Score for star read(s)	:	3.9
Score for read counts	:	2070.5
Score for mfe	:	1.9
Score for randfold	:	0
Score for cons. seed	:	
Total read count	:	4073
Mature read count	:	3952
Loop read count	:	4
Star read count	:	117

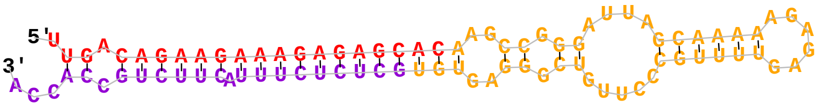

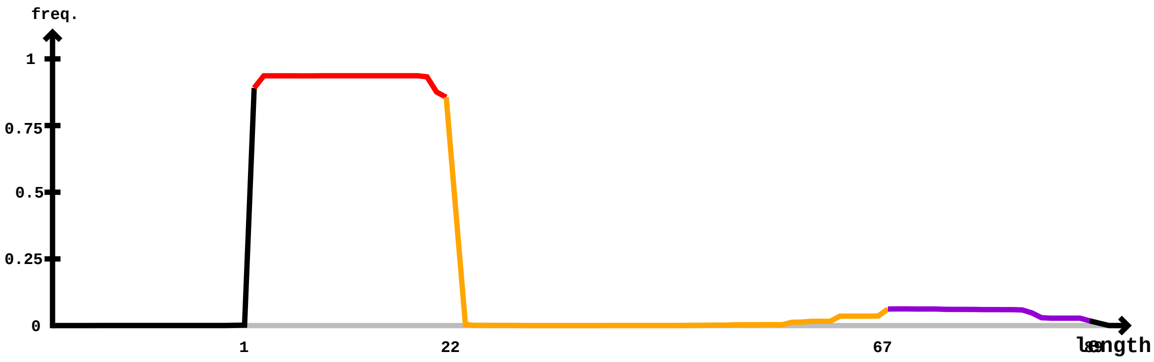

Mature **Star**

```
5'- agaagacgaugagaaaauguugacagaagaaagagagcacaagccgggauuagcaaaaagagaguuuugcccuugucgggagugugcucucuuuacuucugccaccauca -3'    obs
    agaagacgaugagaaaauguugacagaagaaagagagcacaagccgggauuagcaaaaagagaguuuugcccuugucgggagugugcucucuuuacuucugccaccauca        exp
    .......((((.(.......((.((((((((((((((((((..((.((....((((((......)))))).....)).))..))))))))))))).))))))).))))))).   reads   mm   sample
    ...agacgaugagaaaauguugac......................................................................................   1   0   S03
    .................uguugacagaagaaagagagU........................................................................   1   1   S03
    .................uguugacagaagaaagagagc........................................................................   1   0   S03
    ..................guugacagaagaaagagagcac......................................................................   1   0   S03
    ...................uugacagaagaaagagaU.........................................................................   3   1   S03
    ...................uugacagaagaaagagag.........................................................................   1   0   S03
    ...................uugacagaagaaagagagU........................................................................   14   1   S03
    ...................uugacagaagaaagagagc........................................................................   75   0   S03
    ...................uugacagaagaaagagagA........................................................................   3   1   S03
    ...................uugacagaagaUagagagc........................................................................   2   1   S03
    ...................uugacagaagaaagagagG........................................................................   1   1   S03
    ...................uugacagGagaaagagagc........................................................................   1   1   S03
    ...................uugacagaagaaagagaUc........................................................................   2   1   S03
    ...................uugacagaagaaagagagcG.......................................................................   1   1   S03
    ...................uugacagaagaaagagagca.......................................................................   22   0   S03
    ...................uugacagaagaUagagagca.......................................................................   5   1   S03
    ...................uugacagaagCaagagagca.......................................................................   1   1   S03
    ...................uugacagaagaaagagUgcac......................................................................   1   1   S03
    ...................uuCacagaagaaagagagcac......................................................................   1   1   S03
    ...................uugaUagaagaaagagagcac......................................................................   1   1   S03
    ...................uugacagaagaaagagagcGc......................................................................   4   1   S03
    ...................uugCcagaagaaagagagcac......................................................................   1   1   S03
    ...................uugacagaGgaaagagagcac......................................................................   3   1   S03
    ...................uugacagaagaUagagagcac......................................................................   10   1   S03
    ...................uugacagaagaaagagaAcac......................................................................   4   1   S03
    ...................Nugacagaagaaagagagcac......................................................................   2   1   S03
    ...................uugacagaagaaagGgagcac......................................................................   4   1   S03
    ...................uugUcagaagaaagagagcac......................................................................   2   1   S03
    ...................uugaAagaagaaagagagcac......................................................................   1   1   S03
    ...................uugGcagaagaaagagagcac......................................................................   5   1   S03
    ...................uCgacagaagaaagagagcac......................................................................   10   1   S03
    ...................uugacagaagaaaAagagcac......................................................................   2   1   S03
    ...................uugacagaagaaagagagGac......................................................................   1   1   S03
```

Mature **Star**

agaagacgaugagaaaauguugacagaagaaagagagcacaagccgggauuagcaaaaagagaguuuugcccuugucgggagugugcucucuuuacuucugccaccauca			
...................uugacCgaagaaagagagcac..	3	1	S03
...................uugacGgaagaaagagagcac..	2	1	S03
...................uuAacagaagaaagagagcac..	1	1	S03
...................uugacagaagaaGgagagcac..	5	1	S03
...................uugacagCagaaagagagcac..	1	1	S03
...................uugacagaagaaagagGgcac..	4	1	S03
...................uugacagaagaaagUgagcac..	2	1	S03
...................uugacagGagaaagagagcac..	2	1	S03
...................Cugacagaagaaagagagcac..	5	1	S03
...................uugacUgaagaaagagagcac..	2	1	S03
...................uugacagaagaaagagagcaU..	179	1	S03
...................uugacagaagUaagagagcac..	1	1	S03
...................uugacagaagaaagagagcaG..	2	1	S03
...................uugacagaagaaagagCgcac..	1	1	S03
...................uugacagaagGaagagagcac..	9	1	S03
...................uugacagaagaaagagagcaA..	25	1	S03
...................uuUacagaagaaagagagcac..	3	1	S03
...................uugacagaaAaaagagagcac..	1	1	S03
...................uGgacagaagaaagagagcac..	2	1	S03
...................Gugacagaagaaagagagcac..	4	1	S03
...................uugacagaagaaagagagcUc..	3	1	S03
...................uugacagaagaaagagagcac..	1143	0	S03
...................uugacagaagaaagagagUac..	4	1	S03
...................uugacagaagUaagagagcaca...	1	1	S03
...................uugacagaagaaagagagcaca...	20	0	S03
...................uugacagaagaaagagagcacU...	39	1	S03
...................uugacagaagaaagagagcacG...	5	1	S03
...................uugacagaagaaagagagcacC...	6	1	S03
...................uugacagaagaaagagagcacUa..	3	1	S03
....................ugacagaagaaagagagc..	3	0	S03
....................ugacagaagaaagagagU..	2	1	S03
....................ugacagaagaaagagagca...	1	0	S03
....................Agacagaagaaagagagca...	1	1	S03
....................ugacagaagaaagagagcG...	1	1	S03
....................ugacagGagaaagagagca...	1	1	S03
....................ugacagaagGaagagagcac..	2	1	S03
....................ugacagaagaaagagagcaU..	9	1	S03
....................ugacagaagaaagagagcaA..	1	1	S03
....................ugacagaagaaagagagcac..	51	0	S03
....................ugCcagaagaaagagagcaca...	1	1	S03
....................ugacagaagaaagagagcaca...	11	0	S03
....................ugacagaagaaagagagcacU...	4	1	S03
..........................aagaaagagagcacaagccgA...	2	1	S03
....aaagagaguuuugcccuugucgggagug..........................	1	0	S03
....uuuugcccuugucgggagugugcu......................	1	0	S03
....uuugcccuugucgggagugugU.......................	1	1	S03
....uugcccuugucgggagugugcu......................	1	0	S03
....ugcccuugucgggagugugcucuU...................	1	1	S03
....ugcccuugucgggagugAgcucucu..................	1	1	S03
....cccuugucgggagugugcucuc...................	3	0	S03
....uugucgggagugugcucucuuuacG.............	1	1	S03
....ucgggagugugcucucuuuacuucu..........	9	0	S03
....ucgggaCugugcucucuuuacuucu..........	1	1	S03
....ucgggagugugcucucuuuacuucC..........	3	1	S03
....ucgggagugugcucucuuuacuucA..........	2	1	S03
....cgggagugugcucucCuuacuucug.........	1	1	S03
....gggagugugcucucuuuacuuc...........	2	0	S03
....gggagugugcucucuuuacuucu..........	3	0	S03
....gggagugugcucucuuuacuucC..........	2	1	S03
....agugugcucucuuuacuucCg.........	1	1	S03
....agugugcucucuuuacuucuU.........	1	1	S03
....agugugcucucuuuacuucuA.........	3	1	S03
....agCgugcucucuuuacuucug.........	1	1	S03
....agugugcucucuuuacuucug.........	36	0	S03
....agugugcucucuuuacuucugA........	2	1	S03
....ugcucucuuuacuucugccacU....	1	1	S03
....ugcucucuuuacuucugccacA....	1	1	S03
....gcucucuuuacuucugccacA....	1	1	S03
....gcucucuuuacuucugccacc....	7	0	S03
....gcucucuuuacuucugccacU....	10	1	S03

```
                   Mature                                                             Star
agaagacgaugagaaaauguugacagaagaaagagagcacaagccgggauuagcaaaaagagaguuuugcccuugucgggagugugcucucuuuacuucugccaccauca
.....................................................................................gcucucuuuacuucugccaccG...   1      1    S03
.....................................................................................gcucucuuuacuucugccaccU...   3      1    S03
.....................................................................................gcucucuuuacuucugccacca...   23     0    S03
.....................................................................................gcucucuuuacuucugccaccC...   1      1    S03
.....................................................................................gcucucuuuacuucugccaccau..   1      0    S03
......................................................................................cucucuuuacuucugccacca...   1      0    S03

.................uguugacCgaagaaagagagcacaagc..................................................................   1      1    S04
..................guugacagaagaaagagagcac......................................................................   1      0    S04
...................uugacagaagaaagagaU.........................................................................   7      1    S04
...................uugacagaagaaagagag.........................................................................   6      0    S04
...................uuUacagaagaaagagagc........................................................................   1      1    S04
...................uugacagaagUaagagagc........................................................................   1      1    S04
...................uGgacagaagaaagagagc........................................................................   1      1    S04
...................uugacagaagaaagagaCc........................................................................   1      1    S04
...................uugacagaagaUagagagc........................................................................   5      1    S04
...................uugacagaagaaagagagU........................................................................   16     1    S04
...................uugacagaagaaagagagc........................................................................   87     0    S04
...................uugacagUagaaagagagc........................................................................   1      1    S04
...................uugacagaagaaagGgagc........................................................................   1      1    S04
...................uugacagaagaaagagaUc........................................................................   3      1    S04
...................uugacagaagaaagagagA........................................................................   5      1    S04
...................uugacaAaagaaagagagc........................................................................   1      1    S04
...................uugacagaagaaGgagagc........................................................................   1      1    S04
...................uugacagaagaaagagagcG.......................................................................   3      1    S04
...................uugacagaGgaaagagagca.......................................................................   1      1    S04
...................uugacagaagaaGgagagca.......................................................................   1      1    S04
...................uugacagaagaUagagagca.......................................................................   7      1    S04
...................uugacagaagaaagagagca.......................................................................   30     0    S04
...................Nugacagaagaaagagagcac......................................................................   3      1    S04
...................uugacCgaagaaagagagcac......................................................................   1      1    S04
...................Augacagaagaaagagagcac......................................................................   2      1    S04
...................uugacagaagUaagagagcac......................................................................   3      1    S04
...................uugacagaagaaagagaUcac......................................................................   1      1    S04
...................uugaUagaagaaagagagcac......................................................................   1      1    S04
...................uAgacagaagaaagagagcac......................................................................   1      1    S04
...................uCgacagaagaaagagagcac......................................................................   5      1    S04
...................uugacagaagaaagagagUac......................................................................   2      1    S04
...................uugacagaagGaagagagcac......................................................................   8      1    S04
...................uugacagaagaaagagagcaU......................................................................   231    1    S04
...................uugacagaagaaaUagagcac......................................................................   1      1    S04
...................uugacagaagaaagagaAcac......................................................................   2      1    S04
...................uugacagaagaaagaAagcac......................................................................   1      1    S04
...................Cugacagaagaaagagagcac......................................................................   2      1    S04
...................uugacagaagaaGgagagcac......................................................................   7      1    S04
...................uugacagaagaaagagagcac......................................................................   1410   0    S04
...................uugGcagaagaaagagagcac......................................................................   7      1    S04
...................uugacagaagCaagagagcac......................................................................   1      1    S04
...................uugacagaagaaagGgagcac......................................................................   5      1    S04
...................uugacagaagaaagagagAac......................................................................   2      1    S04
...................uugacagaagaaaAagagcac......................................................................   1      1    S04
...................uugacagaagaaagagUgcac......................................................................   1      1    S04
...................uugacagGagaaagagagcac......................................................................   5      1    S04
...................uugacagaagaUagagagcac......................................................................   34     1    S04
...................uugacagaagaaagagagcGc......................................................................   5      1    S04
...................Gugacagaagaaagagagcac......................................................................   6      1    S04
...................uugacagaGgaaagagagcac......................................................................   5      1    S04
...................uugacagaagaaagagaCcac......................................................................   1      1    S04
...................uugacagaaAaaagagagcac......................................................................   1      1    S04
...................uugacUgaagaaagagagcac......................................................................   4      1    S04
...................uugacGgaagaaagagagcac......................................................................   3      1    S04
...................uugacagaagaaagagGgcac......................................................................   4      1    S04
...................uugacagaagaaaNagagcac......................................................................   1      1    S04
...................uugacagaagaaagagagcCc......................................................................   2      1    S04
...................uuUacagaagaaagagagcac......................................................................   2      1    S04
...................uugacagaagaaagagagcaA......................................................................   35     1    S04
...................uugacagaagaaagagCgcac......................................................................   1      1    S04
...................uugacagaagaaagagagcUc......................................................................   2      1    S04
...................uugacaUaagaaagagagcac......................................................................   1      1    S04
...................Gugacagaagaaagagagcaca.....................................................................   1      1    S04
```

Mature **Star**

agaagacgaugagaaaauguugacagaagaaagagagcacaagccgggauuagcaaaaagagaguuuugcccuugucgggagugugcucucuuuacuucugccaccauca			
...................uugacagaagaaagagagcacG...	6	1	S04
...................uugacagaagaaagagagcacC...	5	1	S04
...................uugacagaagaaagagagcaca...	23	0	S04
...................uugacagaagaaagagagcacU...	61	1	S04
...................uugacagaagaaagagagcacCa..	2	1	S04
...................uugacagaagaaagagagcacUa..	2	1	S04
...................uugacagaagaaagagagcacaU..	3	1	S04
...................uugacagaagaaagagagcacaa..	1	0	S04
....................ugacagGagaaagagagc..	1	1	S04
....................ugacagaagaaagagagU..	2	1	S04
....................ugacagaagaaagagagc..	9	0	S04
....................ugacagaagaaagagagca...	7	0	S04
....................uAacagaagaaagagagcac..	1	1	S04
....................ugacagaagaaagagagcaU..	7	1	S04
....................ugacagaagaaagUgagcac..	1	1	S04
....................ugacagaagaaagagagcaA..	1	1	S04
....................Ggacagaagaaagagagcac..	2	1	S04
....................ugacagaagaaagagagcac..	52	0	S04
....................ugacagaagaaagGgagcac..	1	1	S04
....................ugCcagaagaaagagagcac..	1	1	S04
....................uUacagaagaaagagagcac..	1	1	S04
....................ugacagaagaaagaUagcaca...	1	1	S04
....................ugacagaagaaagagagcacU...	10	1	S04
....................ugacagaagaaagagagcaca...	5	0	S04
....................ugacagaagaaagagagcacC...	1	1	S04
....................ugacagaagaaagagagcacaU..	2	1	S04
....uugcccuugucgggagugugcucuc...................	1	0	S04
....cccuugucgggagugugcucucuuu................	1	0	S04
....cccuugucgggagugugcucucuuua...............	1	0	S04
....ccuugucgggagugugcucucuuua...............	1	0	S04
....ucgggagugugcucucuuuacuuc...........	1	0	S04
....ucgggagugugcucucuuuacuucC..........	3	1	S04
....ucgggagugugcucucuuuacuucu..........	15	0	S04
....ucgggagugugcucucuuuacuucA..........	3	1	S04
....ucgggagugugcucuCuuacuucu..........	1	1	S04
....cgggagugugcucucuuuacuucu..........	1	0	S04
....cgggagugugcucucuuuacuucug.........	1	0	S04
....gggagugugcucucuuuacuuc...........	1	0	S04
....gggagugugcucucuuuacuucu..........	4	0	S04
....agugugcucucuuuacuuc...........	1	0	S04
....agGgugcucucuuuacuucug.........	1	1	S04
....agugugcucucuuuacuucuA.........	6	1	S04
....agugugcucucuuuacuucug.........	22	0	S04
....agugugcucucuuuacuucuU.........	1	1	S04
....agugugcucCcuuuacuucug.........	2	1	S04
....agugugcucucuuuacuucugA........	5	1	S04
....ugcucucuuuacuucugccacc....	1	0	S04
....gcucucuuuacuucugccacc....	12	0	S04
....gcucucuuuacuucugccacA....	1	1	S04
....gcucucuuuacuucugccacG....	1	1	S04
....gcucucuuuacuCcugccacc....	1	1	S04
....gcucucuuuacuucugccacU....	6	1	S04
....gcucucuuuacuucugccaccG...	1	1	S04
....gcucucuuuacuucugccaccU...	1	1	S04
....gcucucuuuacuucugccacUa...	1	1	S04
....gcGcucuuuacuucugccacca...	1	1	S04
....gcucucuuuacuucugccacca...	37	0	S04
....gcucucuuuacuucugccGcca...	1	1	S04
....gcucucuuuacuucugccaccau..	1	0	S04
....cucucuuuacuucugccaccau..	1	0	S04
....ucucuuuacuucugccaccauc.	1	0	S04

Provisional ID	:	chrA02_112866
Score total	:	4.3
Score for star read(s)	:	3.9
Score for read counts	:	3.2
Score for mfe	:	-2.7
Score for randfold	:	0
Score for cons. seed	:	
Total read count	:	18
Mature read count	:	16
Loop read count	:	0
Star read count	:	2

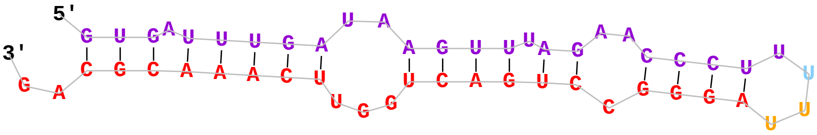

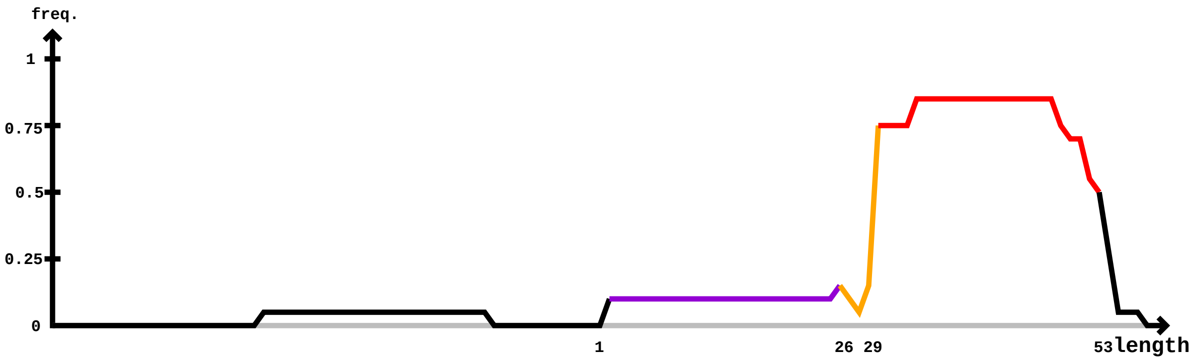

Star Mature

```
5'- aauauguggaguauauaucuauguggauucuaaugaauaaaauuaaccuuuaauuuugugauuugauaaguuuagaacccuuuuuagggccugacugguucaaacgcagcgguug  -3'   obs
    aauauguggaguauauaucuauguggauucuaaugaauaaaauuaaccuuuaauuuugugauuugauaaguuuagaacccuuuuuagggccugacugguucaaacgcagcgguug        exp
    ..((((((((.......)))))))).((((....))))....(((((.......(((((.(((((..((((.((..((((....)))).))))))...)))))))))).)))))   reads     mm     sample
    .....................................................................................agggccugacugAuucaaacgc........   1         1      S03
    .....................................................................................agggAcugacugguucaaacgca.......   1         1      S03
    .....................................................................................agggccugacugguucaaacgcag......   2         0      S03
    .....................................................................................agggccugacugguucaaacgcagc.....   1         0      S03
    .........................................................................................ccugacugguucaaacgcaC......   1         1      S03

    ....................auguggauucuaaugaauaaaauua......................................................................   1         0      S04
    .........................................................gugauuugauaaguuuagaacccuC.................................   1         1      S04
    .........................................................gugauuugauaaguuuagaacccuu.................................   1         0      S04
    .................................................................................uuuuagggccugacuAguucaaac..........   1         1      S04
    ....................................................................................uagggccugacugguucaaa...........   2         0      S04
    .....................................................................................agggccugacugguucaaacgc........   2         0      S04
    .....................................................................................agggccugacugguucaaacgcag......   4         0      S04
    .....................................................................................agggccugacugguucaaacgcagc.....   1         0      S04
    .........................................................................................ccugacugguucGaacgcagcggu..   1         1      S04
```

Provisional ID	:	chrA02_115096
Score total	:	0.2
Score for star read(s)	:	-1.3
Score for read counts	:	0
Score for mfe	:	1.5
Score for randfold	:	0
Score for cons. seed	:	
Total read count	:	135
Mature read count	:	134
Loop read count	:	1
Star read count	:	0

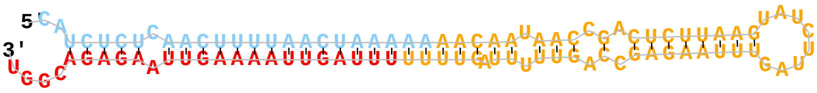

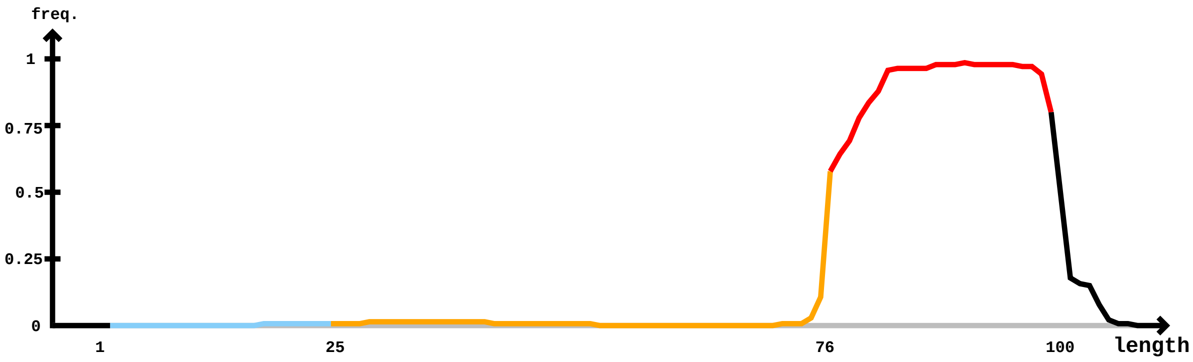


```
                     Star                                                                   Mature
5'- aaaacaucucucaacuuuuaacuaaaaaaacaauaaccgacucuuaaguaucuuaguuuaagagccaguuuuuaguuuuuuuaguuaaaaguuaagagacgguuucuuauauuc   -3'    exp
    .((((.(((((.((((((((((((((((((((((.(((.(.((((((((.......)))))))).).))).)).))))))))))))))))))))).))))).)))).........   reads   mm   sample
    ...............................aauaaccgacuUuuaaguaucuua...........................................................   1       1    S03
    .............................................................................uuuuuaguuaaaaguuaagagacggu...........   1       0    S03
    ..............................................................................uuuuaguuaaaaguuaagagacgg............   5       0    S03
    ...............................................................................uuuaguuaaaaguuaagagacggC...........   3       1    S03
    ...............................................................................uuGaguuaaaaguuaagagacggu...........   1       1    S03
    ...............................................................................uuuaguuaaaaguuaagagacggu...........   19      0    S03
    ...............................................................................uuuaUuuaaaaguuaagagacggu...........   1       1    S03
    ...............................................................................uuuaguuaaCaguuaagagacggu...........   1       1    S03
    ...............................................................................uuuagCuaaaaguuaagagacggu...........   1       1    S03
    ................................................................................uuaguuaaaaguuaagagacg.............   2       0    S03
    ................................................................................uuaguuaaaaguuaagagacggu...........   2       0    S03
    .................................................................................uaguuaaaaguuaagagacggu...........   2       0    S03
    .................................................................................uaguuaaaaguuaagagacgguu..........   2       0    S03
    .................................................................................uaguuaaaaguuaagagacgguuu.........   2       0    S03
    ..................................................................................aguuaCaaguuaagagacggu...........   1       1    S03
    ..................................................................................aguuaaaaguuaagagacggu...........   3       0    S03
    ..................................................................................aguuaaaaguuaagagacgguuG.........   1       1    S03
    ...................................................................................guuaaaaguuaagagacggu...........   1       0    S03
    ...................................................................................guuaaaaguuCagagacggu...........   1       1    S03
    ...................................................................................guuaaaaguuaagagacgguuucu.......   2       0    S03
    ....................................................................................uuaaaaguuaagagacggC...........   3       1    S03
    ....................................................................................uuaaaaguuaagagacgguuucG.......   1       1    S03
    .....................................................................................uaaaaguuaagagacgguuuc........   1       0    S03
    .....................................................................................uaaaaguuaagagacgguuucu.......   2       0    S03
    .....................................................................................uaaaaguuaagagacgguuucuG......   5       1    S03
    ......................................................................................aaaaguuaagagacgguuucuua.....   1       0    S03
    .............................................................................................aagagacgguuucuuaua...   1       0    S03

    ....................acuaaaaaaacaauaaccgacucu......................................................................   1       0    S04
    ..........................................................................guuuuuuuaguuaaaaguua....................   1       0    S04
    .............................................................................uuuuuaguuaaaaguuUagaga...............   1       1    S04
    .............................................................................uuuuuaguuaaaaguuaagagacg.............   1       0    S04
    ..............................................................................uuuuaguuaaaagGuaagagacgg............   1       1    S04
    ..............................................................................uuuuaguuaaaaguuaagagacgg............   5       0    S04
```

Star Mature

aaaacaucucucaacuuuuaacuaaaaaaacaauaaccgacucuuaaguaucuuaguuuaagagccaguuuuuaguuuuuuuaguuaaaaguuaagagacgguuucuuauauuc			
....uuuaguuaaaaguuaagagacg.............	1	0	S04
....uuuaguuaaaaguuaagagacgg............	5	0	S04
....uuuaguuaaaaguuaagagacggC...........	1	1	S04
....uuuaguuaaaagGuaagagacggu...........	1	1	S04
....uuuaguuaaaaguuaagagacggu...........	30	0	S04
....uuuaguuaaaaguuaagGgacggu...........	2	1	S04
....uuaguuaaaaguuaagagacgg............	2	0	S04
....uuaguuaaaaguuaagagacggu...........	3	0	S04
....uaguuaaaaguuCagagacggu...........	1	1	S04
....aguuaaaaguuaagagacgg............	2	0	S04
....aguuaaaaguuaagagacggu...........	3	0	S04
....aguuaaaaguuaagagacggC...........	2	1	S04
....guuaaaaguuaagagacggu...........	1	0	S04
....guuaaaaguuaagagacgguuucu.......	3	0	S04
....uuaaaaguuaagagacggu...........	1	0	S04
....uuaaaaguuaagagacgguuucuu......	1	0	S04
....uaaaaguuaagagacgguuucu.......	2	0	S04
....uaaaaguuaagagacgguuucuG......	1	1	S04
....guuaagagacgguuucuG......	1	1	S04
....guuaagagacgguuucuGa.....	1	1	S04

Provisional ID	:	chrA02_128683
Score total	:	1
Score for star read(s)	:	-1.3
Score for read counts	:	0
Score for mfe	:	2.3
Score for randfold	:	0
Score for cons. seed	:	
Total read count	:	18
Mature read count	:	18
Loop read count	:	0
Star read count	:	0

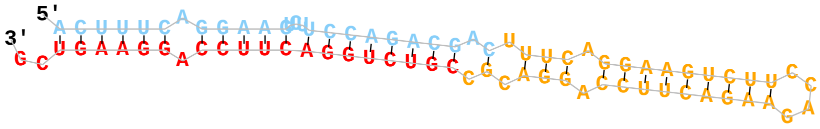

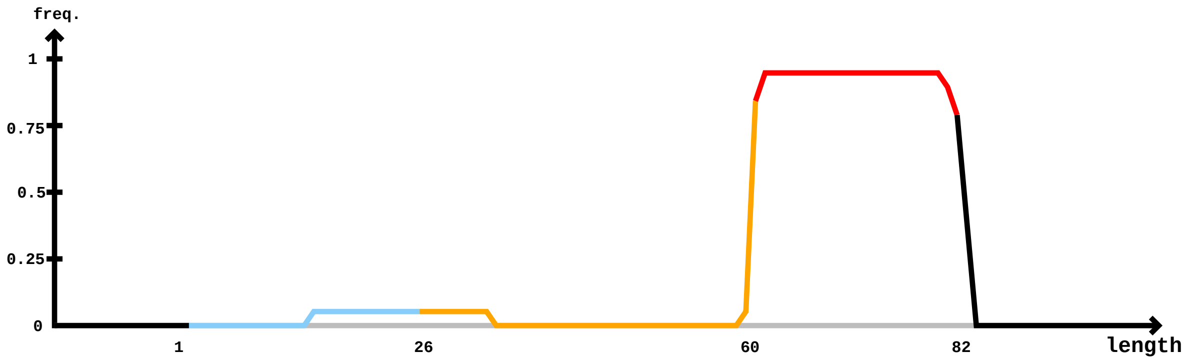

Star Mature

```
5'- augcgcuagacgacuuucaggaagucuuccagacgacuuucaggaagucuuccagaagacuuccaggacgccgucuggacuuccaggaagucgucagucgucugacaaagucu   -3'    exp
    ..(((((.((((((((((.(((((...((((((((.(.(((.(((((((((....))))))))).))).).))))))))))))).))))))))))))).)))..(((...))).    reads      mm        sample
    .........................cuuccagacgacuuAcagg.....................................................................    1          1         S03
    ......................................................................ccgucuggacuuccaggaagucg....................    1          0         S03
    .......................................................................cgucuggacuuccaggaaguc.....................    1          0         S03
    .......................................................................cgucuggacuuccaggaagucg....................    3          0         S03
    ........................................................................gucuggacuuccaggaagucg....................    2          0         S03

    .......................................................................cgucuggacuuccaggGagu......................    1          1         S04
    .......................................................................cgucuggacuuccaggaaguA.....................    1          1         S04
    .......................................................................cgucuggacuuccaggaaguUg....................    1          1         S04
    .......................................................................cgucuggacuuccaggaagucg....................    7          0         S04
    .......................................................................cgucuggacuuccCggaagucg....................    1          1         S04
```

Provisional ID	:	chrA02_135723
Score total	:	0.8
Score for star read(s)	:	3.9
Score for read counts	:	-4.9
Score for mfe	:	1.9
Score for randfold	:	0
Score for cons. seed	:	
Total read count	:	2
Mature read count	:	1
Loop read count	:	0
Star read count	:	1

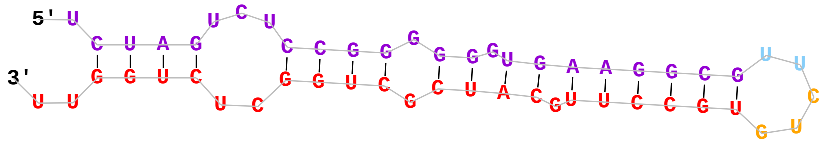

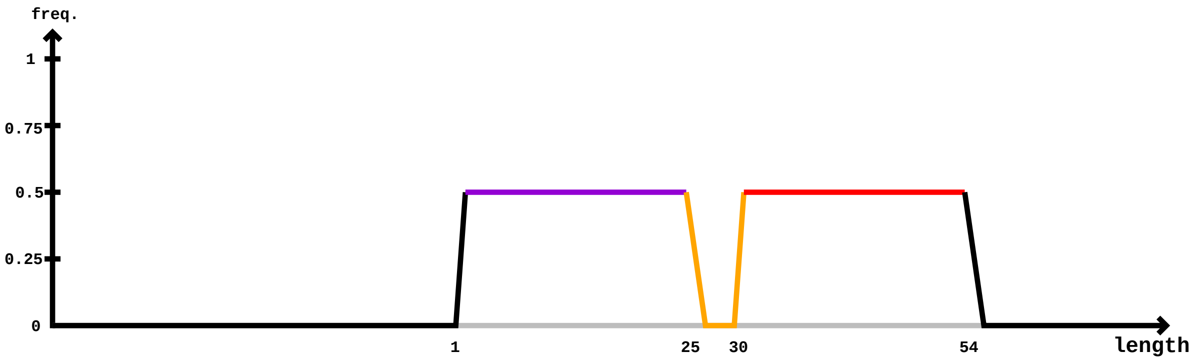

Star Mature

```
5'- uuccgggaggcggaugcuucuuaagcucugcuaucgccggcucuagucuccggggggugaaggcguucugugccuugcaucgcuggcucugguuucccuaguucuguuuuaggg   -3'    obs
    uuccgggaggcggaugcuucuuaagcucugcuaucgccggcucuagucuccggggggugaaggcguucugugccuugcaucgcuggcucugguuucccuaguucuguuuuaggg          exp
    ..((((((((((((.(((.....))))))))).)).))))..((((...((((.((.((((((((.....)))))).)))).))))..))))...((((((.......))))))   reads    mm    sample
    .........................................ucuagucuccggggggugaaggcA.................................................   1        1     S04
    ......................................................................ugccuugcaucgcuggcucugguu....................   1        0     S04
```

Provisional ID : chrA02_157250
Score total : 1075.1
Score for star read(s) : 3.9
Score for read counts : 1082.5
Score for mfe : -11.2
Score for randfold : 0
Score for cons. seed :
Total read count : 2135
Mature read count : 2134
Loop read count : 0
Star read count : 1

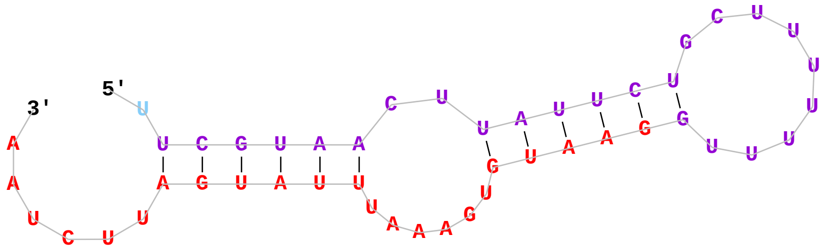

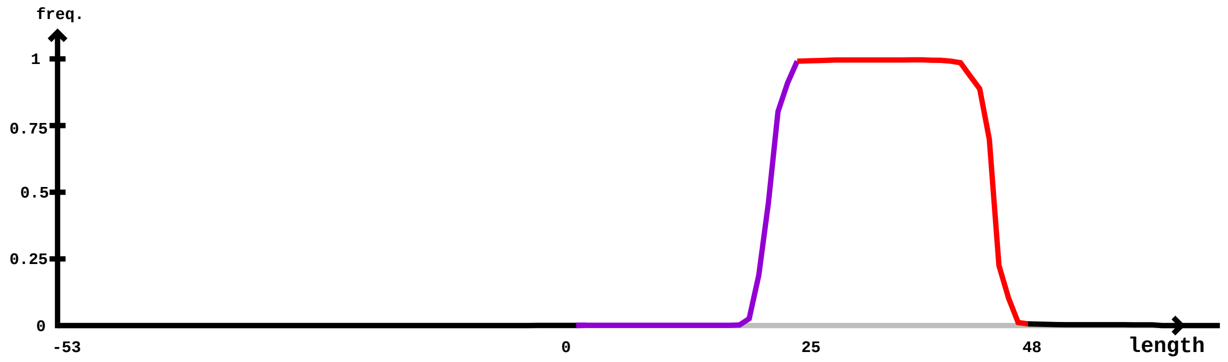

Star Mature

```
5'- auuugauggguaauauguaaauaaaaaucauuguuauuuguauuuaucagaguucguaacuuauucugcuuuuuuuggaaugugaaauuuaugauucuaauaaauauauaaauaa  -3'  obs
    auuugauggguaauauguaaauaaaaaucauuguuauuuguauuuaucagaguucguaacuuauucugcuuuuuuuggaaugugaaauuuaugauucuaauaaauauauaaauaa        exp
    ....(((((....(((....)))....)))))......(((((((((.(((((.(((((..((((((.........))))))......)))))))))).))))))))).......  reads  mm  sample
    ................................................agaguucguaacuuauucugcuu............................................  1  0  S03
    .....................................................ucguaacuuaCucugcuuuuuuug......................................  1  1  S03
    .....................................................................uuuuuuuggaaugugaaauuuaugaC....................  1  1  S03
    .....................................................................uuuuuuuggaaugugaGauuuaugau....................  1  1  S03
    ......................................................................uuuuuuggaaugugaaauuuGu.......................  1  1  S03
    ......................................................................uuuuuuggaaugugaCauuuaug......................  1  1  S03
    ......................................................................uuuuuuggaaugugaaauuuauA......................  2  1  S03
    ......................................................................uuuuuuggaaugugaaauuuaug......................  8  0  S03
    ......................................................................uuuuuuggaaugugaaauuuauga.....................  3  0  S03
    ......................................................................uuuuuuggaaugugaaauuuaugau....................  2  0  S03
    .......................................................................uuuuuggaaugugaaauu..........................  3  0  S03
    .......................................................................uuuuuggaaugugaaauuuau.......................  2  0  S03
    .......................................................................uuuuuggaaugugaaauuuaug......................  13  0  S03
    .......................................................................uuuuuggaaugugaaGuuuaug......................  1  1  S03
    .......................................................................uuuuuggaauguUaaauuuaug......................  2  1  S03
    .......................................................................uuuuuggaaugugUaauuuaug......................  1  1  S03
    .......................................................................uuuuuggaaugugaaauuuauga.....................  16  0  S03
    .......................................................................uuuuuggaaugugaaauuuaugG.....................  1  1  S03
    .......................................................................uuuuuggaaugugaaauuuauUau....................  1  1  S03
    .......................................................................uuuuuggaaugugaaauuuaugaG....................  1  1  S03
    .......................................................................uuuuuggaauguUaaauuuaugau....................  5  1  S03
    .......................................................................uuuuuggaaugugaaauuuaugGu....................  1  1  S03
    .......................................................................uuuuuggaGugugaaauuuaugau....................  1  1  S03
    .......................................................................uuuuuggaaugugaGauuuaugau....................  1  1  S03
    .......................................................................uuuuuggaaugugaaauuuaugau....................  95  0  S03
    .......................................................................uuuuuggaaugCgaaauuuaugau....................  1  1  S03
    .......................................................................uuuuuggaaugugaaauuuaugaA....................  2  1  S03
    .......................................................................uuuuuggaaugugaaauuuaugaC....................  11  1  S03
    .......................................................................uuuuuggaaGgugaaauuuaugau....................  1  1  S03
    .......................................................................uuuuuUgaaugugaaauuuaugau....................  1  1  S03
    .......................................................................uuuuuggaaugugaaauuuaugauu...................  5  0  S03
    .......................................................................uuuuuggaaugugaaauuuaugauG...................  1  1  S03
    .......................................................................uuuuuggaaugugaaauuuaugauc..................  1  0  S03
```

```
                                                          Star                   Mature
auuugauggguaauauguaaauaaaaaucauuguuauuuguauuuaucagaguucguaacuuauucugcuuuuuuuggaaugugaaauuuaugauucuaauaaauauauaaaauaa
........................................................................Cuuuggaaugugaaauuu..........................   1   1   S03
........................................................................uuuuggaaugugaaauuu..........................   2   0   S03
........................................................................uuuuggaaugugaaauuua.........................   1   0   S03
........................................................................uuuuggaaugugaaauuuau........................   1   0   S03
........................................................................uuuuggaaugugaaauuuaug.......................   12  0   S03
........................................................................uuuuggaaugugaaauuuaugG......................   1   1   S03
........................................................................uuuuggaaugugaaauuuauga......................   3   0   S03
........................................................................Cuuuggaaugugaaauuuaugau.....................   1   1   S03
........................................................................uuuuggaaugugaaauuuaugaA.....................   1   1   S03
........................................................................uuuuggaaugugUaauuuaugau.....................   1   1   S03
........................................................................uuuuggaaugugaaauuuaugau.....................   29  0   S03
........................................................................uuuuggaaugugaGauuuaugauu....................   2   1   S03
........................................................................uuuuggaaugugaaauuuaugauC....................   19  1   S03
........................................................................uuuuggaaugugaaauuuaugauu....................   170 0   S03
........................................................................uuuuggaaugAgaaauuuaugauu....................   1   1   S03
........................................................................uuuuggaaugugaaauuuaugaCu....................   2   1   S03
........................................................................uuuuggaaugugaaauuuaugauG....................   3   1   S03
........................................................................uuuuggaauguUaaauuuaugauu....................   9   1   S03
........................................................................uuuuggaaugGgaaauuuaugauu....................   2   1   S03
........................................................................Cuuuggaaugugaaauuuaugauu....................   1   1   S03
........................................................................Auuuggaaugugaaauuuaugauu....................   1   1   S03
........................................................................uuuuggaaugugaaauuuaugauA....................   1   1   S03
........................................................................uuuuggaaugugUaauuuaugauu....................   1   1   S03
.........................................................................uuuggaaugugaaauuuaug.......................   5   0   S03
.........................................................................uuuggaaugugaaauuuauga......................   2   0   S03
.........................................................................uuuggaaugugaaauuuaugUu.....................   1   1   S03
.........................................................................uuuggaaugugaaauuuaugaC.....................   1   1   S03
.........................................................................uuuggaaugugaaauuuaugau.....................   13  0   S03
.........................................................................uuuggaaugugaaauuuaugauC....................   19  1   S03
.........................................................................uuAggaaugugaaauuuaugauu....................   1   1   S03
.........................................................................uuuUgaaugugaaauuuaugauu....................   1   1   S03
.........................................................................uuuggaaugugaaauuuaugUuu....................   1   1   S03
.........................................................................uuuggaaugCgaaauuuaugauu....................   1   1   S03
.........................................................................Guuggaaugugaaauuuaugauu....................   3   1   S03
.........................................................................uuuggaaugugaaauuuaugauG....................   10  1   S03
.........................................................................uuuggaaugugaaauuuaugauA....................   5   1   S03
.........................................................................uuuggaaugugaaauuuaugauu....................   143 0   S03
.........................................................................uuuggaaugugaaauuuaugaCu....................   2   1   S03
.........................................................................uuuggaauguUaaauuuaugauu....................   5   1   S03
.........................................................................uuuggaaugugaaauuuaugGuu....................   1   1   S03
.........................................................................uuuggaaugugaaauuuaugauuc...................   80  0   S03
.........................................................................uuuggaaugCgaaauuuaugauuc...................   1   1   S03
.........................................................................uuuggaauguUaaauuuaugauuc...................   3   1   S03
.........................................................................uuugUaaugugaaauuuaugauuc...................   1   1   S03
.........................................................................uuuggaaugugaaauuuaugauuG...................   2   1   S03
.........................................................................uuuggaaugugaaauuuaugauucA..................   2   1   S03
..........................................................................uuggaaugugaaauuuaug.......................   1   0   S03
..........................................................................uuggaaugGgaaauuuauga......................   1   1   S03
..........................................................................uuggaaugugaaauuuaugUu.....................   1   1   S03
..........................................................................uuggaaugugaaauuuaugau.....................   4   0   S03
..........................................................................uuggaaugugaaauuuaugauC....................   3   1   S03
..........................................................................uuggaauguUaaauuuaugauu....................   1   1   S03
..........................................................................uuggaaugugaaauuuaugauu....................   25  0   S03
..........................................................................uuggaauguUaaauuuaugauuc...................   1   1   S03
..........................................................................uuggaaugugaaauuuaugaCuc...................   1   1   S03
..........................................................................uuggaaugugaaauuuaugauuc...................   14  0   S03
..........................................................................uuggaaugGgaaauuuaugauucu..................   1   1   S03
..........................................................................uuggaaugugaaauuuaugauucu..................   37  0   S03
..........................................................................Nuggaaugugaaauuuaugauucu..................   1   1   S03
..........................................................................uuggaaugugaaauuuaugauucA..................   4   1   S03
..........................................................................uuggaaugugaaauuuaugauucC..................   9   1   S03
..........................................................................uuggaaugugaaauuuaugauucuU.................   1   1   S03
...........................................................................uggaaugugaaauuuaug.......................   3   0   S03
...........................................................................uggaauguUaaauuuauga......................   1   1   S03
...........................................................................uggaaugugaaauuuaugau.....................   3   0   S03
...........................................................................uggaaugugaaauuuaugaC.....................   1   1   S03
...........................................................................uggaaugugaaauuuaugauu....................   11  0   S03
...........................................................................uggaauguUaaauuuaugauu....................   1   1   S03
...........................................................................uggaaugugaaauuuaugauC....................   1   1   S03
...........................................................................ugUaaugugaaauuuaugauuc...................   1   1   S03
```

```
                                                                            Star              Mature
auuugauggguaauauguaaauaaaaaucauuguuauuuguauuuaucagaguucguaacuuauucugcuuuuuuuggaaugugaaauuuaugauucuaauaaauauauaaauaa
...........................................................................uggaaugugaaauuuaugGuuc..................   1     1     S03
...........................................................................uggaaugugaaauuuaugauuc..................   9     0     S03
...........................................................................uggaaugugaaauuuaugauucA.................   36    1     S03
...........................................................................uggaaugugaaauuuaugauucu.................   4     0     S03
...........................................................................uggaaugugaaauuuaugauucuU................   1     1     S03
...........................................................................uggaaugugaaauuuaugauucAa................   1     1     S03
............................................................................ggaaugugaaauuuaugauu...................   1     0     S03
............................................................................gUaaugugaaauuuaugauucua................   1     1     S03
.............................................................................Uaaugugaaauuuaugauucuaau..............   1     1     S03
.............................................................................gaaugugaaauuuaugauucuaau..............   1     0     S03
..............................................................................aaugugaaauuuaugauucuG................   1     1     S03
..............................................................................aaugugaaauuuaugauucuaau..............   1     0     S03
...............................................................................augugaaauuuaugauucuaaua.............   1     0     S03
...............................................................................augugaaauuuaugauucuaauaa............   1     0     S03
.........................................................................................uaugauucuaauaaauauauaaau..   1     0     S03
..........................................................................................augauucuaauaaauauauaaau..   1     0     S03

.....................................................................uuuuuuuggaaugugaaauuua........................   1     0     S04
......................................................................uuuuuuggaaugugaaauuuU........................   1     1     S04
......................................................................Nuuuuuggaaugugaaauuuau.......................   1     1     S04
......................................................................uuuuuuggaaugugaaauuuauA......................   3     1     S04
......................................................................uuuuuuggaaugugaaauuuaug......................   9     0     S04
......................................................................uuuuuuggaaugugaaauuuauga.....................   13    0     S04
......................................................................uuuuuuggaauguUaaauuuauga.....................   1     1     S04
......................................................................uuuuuuggaaugugaaauuuaugaG....................   1     1     S04
......................................................................uuuuuuggaaugAgaaauuuaugau....................   1     1     S04
......................................................................uuuuuuggaaugugaaauuuaugau....................   5     0     S04
.......................................................................uuuuuggaaugugaaauu..........................   1     0     S04
.......................................................................uuuuuggaaugugaaauuu.........................   2     0     S04
.......................................................................uuuuuggaaugugaaauuua........................   1     0     S04
.......................................................................uuuuuggaaugugaaauuuau.......................   1     0     S04
.......................................................................uuuuuggaaugugaaauuuauC......................   1     1     S04
.......................................................................uuuuuggaaugugaaauuuauU......................   3     1     S04
.......................................................................uuuuuggaaugugaaauuuaug......................   25    0     S04
.......................................................................uuuuuggaaugugaaauuuauga.....................   19    0     S04
.......................................................................uuuuuggaaugugaaauuuaugau....................   116   0     S04
.......................................................................uuuuuggaaugugaaauuuaugGu....................   2     1     S04
.......................................................................uuuuuggaaugugaaauuuauUau....................   2     1     S04
.......................................................................uuuuuggaaugugaaauuuaugaC....................   6     1     S04
.......................................................................uuuuuggaauguUaaauuuaugau....................   5     1     S04
.......................................................................uuuuuggaaugugaaauuuaugaG....................   3     1     S04
.......................................................................uuuuuggaaugugaaauuuaugauu...................   4     0     S04
.......................................................................uuuuuggaaugugaGauuuaugauu...................   1     1     S04
.......................................................................uuuuuggUaugugaaauuuaugauu...................   1     1     S04
........................................................................uuuuggaaugugaaauuu.........................   1     0     S04
........................................................................uuuuggaaugugaaauuua........................   1     0     S04
........................................................................uuuuggaaugugaaauuuau.......................   3     0     S04
........................................................................uuuuggaauguUaaauuuau.......................   1     1     S04
........................................................................uuuuggaaugugaaauuuaug......................   6     0     S04
........................................................................uuuuggaaugugaaauuuauga.....................   28    0     S04
........................................................................uuuuggaaugugaaauuuaugG.....................   2     1     S04
........................................................................uuuuggaaugugaaauuuaugC.....................   1     1     S04
........................................................................Cuuuggaaugugaaauuuaugau....................   1     1     S04
........................................................................uuuuggaaugugaaauuuaugau....................   47    0     S04
........................................................................uuuuggaauguUaaauuuaugau....................   2     1     S04
........................................................................uuuuggaaugugaaauuuaugaG....................   1     1     S04
........................................................................uuuuggaaugAgaaauuuaugau....................   1     1     S04
........................................................................uGuuggaaugugaaauuuaugau....................   1     1     S04
........................................................................uuuuggaaugugaaauuuaugaC....................   5     1     S04
........................................................................Guuuggaaugugaaauuuaugauu...................   1     1     S04
........................................................................uuuGggaaugugaaauuuaugauu...................   1     1     S04
........................................................................uuuuUgaaugugaaauuuaugauu...................   1     1     S04
........................................................................uuuuggaaugCgaaauuuaugauu...................   1     1     S04
........................................................................uuuuggaaugugaaauuuaugauA...................   1     1     S04
........................................................................uuuuggaaugugUaauuuaugauu...................   1     1     S04
........................................................................uuuuggaaGgugaaauuuaugauu...................   1     1     S04
........................................................................uuuuggaaugugaaauuuaugUuu...................   1     1     S04
........................................................................uuuuggaaugugaaauuuaugauC...................   19    1     S04
........................................................................uuuuggaaugugaaauuuaugaCu...................   2     1     S04
........................................................................uuuuggaaugugaaauuuaugauu...................   186   0     S04
```

```
                                                     Star                    Mature
auuugauggguaauauguaaauaaaaaucauuguuauuuguauuuaucagaguucguaacuuauucugcuuuuuuuggaaugugaaauuuaugauucuaauaaauauauaaauaa
........................................................................Cuuuggaaugugaaauuuaugauu...................   2     1     S04
........................................................................uuuuggaaugugaGauuuaugauu...................   2     1     S04
........................................................................uuuuggaauguUaaauuuaugauu...................   9     1     S04
........................................................................uuuuggaaugugaaauuuaugauG...................   2     1     S04
.........................................................................uuuggaaugugaaauuua........................   2     0     S04
.........................................................................uAuggaaugugaaauuuau.......................   1     1     S04
.........................................................................uuuggaaugugaaauuuau.......................   1     0     S04
.........................................................................uuuggaaugugaaauuuaug......................   10    0     S04
.........................................................................uuuggaauguUaaauuuauga.....................   1     1     S04
.........................................................................uuuggaaugugaaauuuaugG.....................   1     1     S04
.........................................................................uuuggaaugugaaauuuauga.....................   5     0     S04
.........................................................................uuuggaaugugaaUuuuaugau....................   1     1     S04
.........................................................................uuuggaGugugaaauuuaugau....................   2     1     S04
.........................................................................uuuggaaugugaaauuuaugau....................   17    0     S04
.........................................................................uuuggaaugAgaaauuuaugau....................   1     1     S04
.........................................................................uuuggaaugugaaauuuaugaA....................   1     1     S04
.........................................................................uuuggaaugugaaauuuaugaC....................   2     1     S04
.........................................................................uuuggaauguUaaauuuaugau....................   1     1     S04
.........................................................................uuuggaaugugaaUuuuaugauu...................   1     1     S04
.........................................................................uuuggaaugugUaauuuaugauu...................   1     1     S04
.........................................................................uuuggaaugCgaaauuuaugauu...................   1     1     S04
.........................................................................uuuggaaugugaaauuuaugauA...................   6     1     S04
.........................................................................uuuggaaugugaCauuuaugauu...................   1     1     S04
.........................................................................uuuggaaugugaaauuuaugauG...................   9     1     S04
.........................................................................uuuggaaugAgaaauuuaugauu...................   2     1     S04
.........................................................................uuuggaaugugaaauuuaugauu...................   240   0     S04
.........................................................................uuuggaauguUaaauuuaugauu...................   17    1     S04
.........................................................................uuuUgaaugugaaauuuaugauu...................   1     1     S04
.........................................................................uuuggaaugugaaauuuaugauC...................   24    1     S04
.........................................................................uuuggaaugugaaauuuaugaCu...................   1     1     S04
.........................................................................uuuggaauguCaaauuuaugauu...................   1     1     S04
.........................................................................uuuUgaaugugaaauuuaugauuc..................   1     1     S04
.........................................................................uuuggaauguUaaauuuaugauuc..................   1     1     S04
.........................................................................uuuggaGugugaaauuuaugauuc..................   2     1     S04
.........................................................................uuuggaaugugaaauuuaugauGc..................   1     1     S04
.........................................................................uuuggaaugugaaauuuaugauuG..................   1     1     S04
.........................................................................uuuggaaugugaaauuuaugauuc..................   93    0     S04
.........................................................................uuuggaaugugaaauuuaugauucA.................   1     1     S04
..........................................................................uuggaaugugaaauuuau.......................   1     0     S04
..........................................................................uuggaaugugaaauuuaug......................   2     0     S04
..........................................................................uAggaaugugaaauuuauga.....................   1     1     S04
..........................................................................uuggaaugugaaauuuauga.....................   3     0     S04
..........................................................................uuggaaugugaaauuuaugau....................   5     0     S04
..........................................................................uuggaaugugaaauuuaugUuu...................   1     1     S04
..........................................................................uuggaaugugaaauuuaugauC...................   3     1     S04
..........................................................................uuggaaugugaaauuuaugauG...................   1     1     S04
..........................................................................uuggaaugugaaauuuaugauu...................   26    0     S04
..........................................................................uAggaaugugaaauuuaugauuc..................   1     1     S04
..........................................................................uuggaaugugaaauuuaugGuuc..................   1     1     S04
..........................................................................uuggaaugugaaauuuaugaCuc..................   1     1     S04
..........................................................................uuggaaugugaaauuuaugauuG..................   2     1     S04
..........................................................................uuggaaugugaaauuuaugauuc..................   26    0     S04
..........................................................................uuggaaugugaaauuuaugauucu.................   41    0     S04
..........................................................................uuggaaugugaaauuuaugauucA.................   7     1     S04
..........................................................................Cuggaaugugaaauuuaugauucu.................   1     1     S04
..........................................................................uuggaaugugaaauuuaugauCcu.................   1     1     S04
..........................................................................uuggaaugugaaauuuaugauucC.................   6     1     S04
..........................................................................uuggaaugugaaauuuaugauucuU................   1     1     S04
..........................................................................uuggaaugugaaauuuaugauucuC................   1     1     S04
...........................................................................uggaaugugaaauuuaug......................   1     0     S04
...........................................................................uggaaugCgaaauuuauga.....................   1     1     S04
...........................................................................uggaaugugaaauuuauga.....................   2     0     S04
...........................................................................uggaaugugaaauuuaugau....................   7     0     S04
...........................................................................uggaaugugaaauuuaugaCu...................   1     1     S04
...........................................................................uggaaugugaaauuuaugauu...................   13    0     S04
...........................................................................uggaaugugaaauuuaugauC...................   1     1     S04
...........................................................................uggaauguUaaauuuaugauu...................   1     1     S04
...........................................................................uggaaugugaaauuuaugauuc..................   24    0     S04
...........................................................................uggaaugugaaauuuaugauucu.................   4     0     S04
...........................................................................uggaaugugaaauuuaugauucA.................   47    1     S04
```

```
                                                     Star                   Mature
auuugaugggguaauauguaaauaaaaaucauuguuauuuguauuuaucagaguucguaacuuauucugcuuuuuuuggaaugugaaauuuaugauucuaauaaauauauaaauaa
...........................................................................uggaaugugaaauuuaugauucua................	3	0	S04
..............................................................................aaugugaaauuuaugauucuG................	1	1	S04
..............................................................................aaugugaaauuuaugauucuGa...............	1	1	S04
...............................................................................augugaaauuuaugauucuaaua.............	1	0	S04
.......................................................................................uuuaugauucuaauaaauauaua.....	1	0	S04
..........................................................................................augauucuaauaaUuauauaaau..	1	1	S04
..........................................................................................augauucuaauaaauauauaaau..	3	0	S04
```

Provisional ID	:	chrA02_170296
Score total	:	120.8
Score for star read(s)	:	3.9
Score for read counts	:	116.9
Score for mfe	:	0.1
Score for randfold	:	0
Score for cons. seed	:	
Total read count	:	241
Mature read count	:	240
Loop read count	:	0
Star read count	:	1

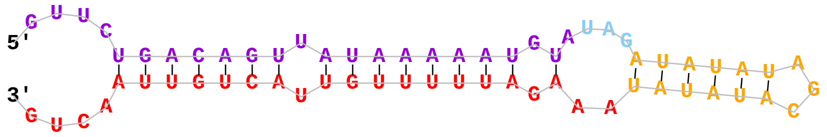

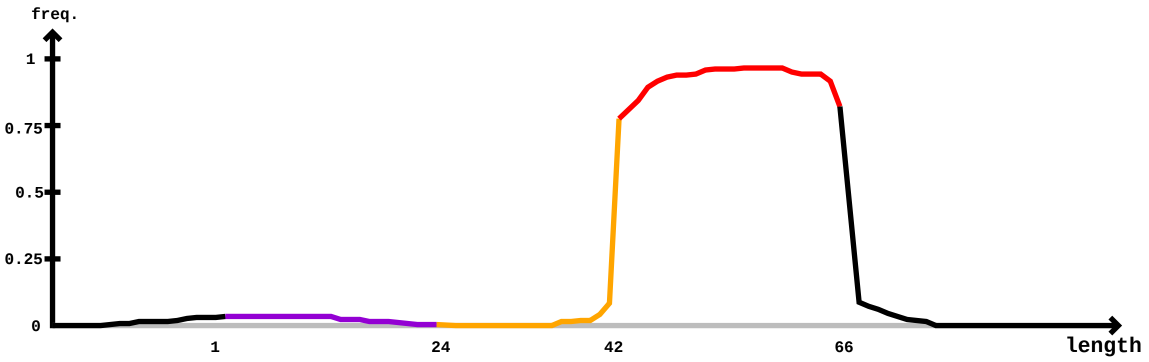


```
                    Star                                         Mature
5'- guucaacugcagugcgguucugacaguuauaaaaauguauagauauauagcauauauaaagauuuuuguuacuguuaacuguggggcggaacgggacaaugguuaucau   -3'    obs
    guucaacugcagugcgguucugacaguuauaaaaauguauagauauauagcauauauaaagauuuuuguuacuguuaacuguggggcggaacgggacaaugguuaucau          exp
    ((((..((((..((((((..(((((((.((((((((.(....((((((...))))))..).)))))))).)))))))))))))..))))....))))............   reads   mm   sample
    ....aacugcUgugcgguucugacaguu.................................................................................   1       1    S03
    .....acugcUgugcgguucugacaguu.................................................................................   1       1    S03
    .......ugcagugcgguucugacaguu.................................................................................   1       0    S03
    .............gcgguucugaUaguuauaaaaau.........................................................................   1       1    S03
    ...................................................auauauaaagauuuuuguuacugu..................................   1       0    S03
    .....................................................auauaaagauuuuuguuacuguu.................................   1       0    S03
    .......................................................auaaagauuuuuguuacuguuaac..............................   2       0    S03
    ........................................................Aaaagauuuuuguuacuguuaacu.............................   1       1    S03
    ........................................................Aaaagauuuuuguuacuguuaacug............................   1       1    S03
    .........................................................aaagauuuuuguuacuguuGacu.............................   1       1    S03
    .........................................................aaagauuuuuguuacuguuaaUu.............................   1       1    S03
    .........................................................aaagauuuuuguuacuguuaacuU............................   3       1    S03
    .........................................................aaagauuCuuguuacuguuaacug............................   1       1    S03
    .........................................................aaagauuuuuguuacuguuaacug............................   40      0    S03
    .........................................................aaagCuuuuuguuacuguuaacug............................   1       1    S03
    .........................................................aaagauuuCuguuacuguuaacug............................   1       1    S03
    .........................................................aaagauuuuuguuacuguuGacug............................   1       1    S03
    .........................................................Gaagauuuuuguuacuguuaacug............................   1       1    S03
    ..........................................................aagauuuuuguuacuguuaacu.............................   1       0    S03
    ..........................................................aagauuuuuguuacuguuaacug............................   1       0    S03
    ..........................................................aagauuuuuguuacuguuGacugu...........................   1       1    S03
    ...........................................................agauuuuuguuacuguuaacuC............................   1       1    S03
    ...........................................................agauGuuuguuacuguuaacug............................   1       1    S03
    ...........................................................agauuuuuguuacuguuaacugug..........................   2       0    S03
    ............................................................gauuuuuguuacuguuaacug............................   2       0    S03
    ............................................................gauuuuuguuacuguuaacugu...........................   1       0    S03
    ............................................................gauuuuuguuacuguuaacugugg.........................   1       0    S03
    .............................................................auuuuuguuacuguuaacuguggA........................   1       1    S03
    .............................................................auuuuuguuacuguuaacugugg.........................   2       0    S03
    ..............................................................uuuuuguuacuguuaacuguAggg.......................   1       1    S03
    ...................................................................guuacuguuaacuguggggc......................   1       0    S03
    ......................................................................acuguuaacuguggggcgg....................   1       0    S03

    .......ugcagugcgguucugacaguuaua..............................................................................   1       0    S04
```

Star   Mature

```
guucaacugcagugcgguucugacaguuauaaaaauguauagauauauagcauauauaaagauuuuuguuacuguuaacuguggggcggaacgggacaaugguuaucau
...........gugcgguucugacaguuauaaaa...........................................................................    1      0     S04
............ugcgguucugacaguuaua..............................................................................    1      0     S04
............ugcgAuucugacaguuauaaaaa..........................................................................    1      1     S04
................guucugacaguuauaaaaauguG......................................................................    1      1     S04
...................................................auauauaaagauuuuuguuacugC..................................    1      1     S04
...................................................auauauaaagauuuuuguuacugu..................................    2      0     S04
.......................................................auaaagauuuuuguuacuguu.................................    1      0     S04
.......................................................auaaagauuuuuguuacuguuaac..............................    3      0     S04
........................................................uaaagauuuuuguuacuguuaacG.............................    1      1     S04
........................................................uaaagauuuuuguuacuguuaacu.............................    1      0     S04
........................................................Aaaagauuuuuguuacuguuaacu.............................    6      1     S04
........................................................Aaaagauuuuuguuacuguuaacug............................    1      1     S04
.........................................................aaagauuuuuguuacuguuaac..............................    1      0     S04
.........................................................aaagauuuuugAuacuguuaac..............................    1      1     S04
.........................................................aaagauuuuuguuacuguuaacu.............................    5      0     S04
.........................................................aaagauuuuuguuacuguuaacA.............................    1      1     S04
.........................................................aaagauuuuuguuacuguuaaUu.............................    1      1     S04
.........................................................aaagauuuuuguuacugAuaacug............................    1      1     S04
.........................................................aaagauuuCuguuacuguuaacug............................    1      1     S04
.........................................................aaGgauuuuuguuacuguuaacug............................    1      1     S04
.........................................................aaagauuuuuguuacuguuGacug............................    1      1     S04
.........................................................aaagauuuAuguuacuguuaacug............................    1      1     S04
.........................................................aaagauuuuuguuacuguuaacCg............................    1      1     S04
.........................................................aaagauuuuuguuacuguuaacuC............................    1      1     S04
.........................................................aaagauuuuuguuacuguuaacuU............................    2      1     S04
.........................................................aaagauuuuuguuUcuguuaacug............................    1      1     S04
.........................................................Gaagauuuuuguuacuguuaacug............................    2      1     S04
.........................................................aaagCuuuuuguuacuguuaacug............................    1      1     S04
.........................................................aaagauuuuuguuacuguuaaUug............................    2      1     S04
.........................................................aaagauuuuuguuacuguuaacug............................    108    0     S04
..........................................................aagauuuuuguuacuguuaacu.............................    4      0     S04
..........................................................aaCauuuuuguuacuguuaacug............................    1      1     S04
..........................................................aagauuuuuguuacuguuaacug............................    1      0     S04
...........................................................agauuuuuguuacuguuaacu.............................    1      0     S04
...........................................................agauuuuuguuacuguuaacug............................    2      0     S04
...........................................................agauuuuuguuacuguuaacugu...........................    1      0     S04
...........................................................agauuuuuguuacuguuaacugug..........................    1      0     S04
............................................................gauuuuuguuacuguuaacug............................    6      0     S04
............................................................gauuuuuguuacuguuaacuguU..........................    1      1     S04
............................................................Aauuuuuguuacuguuaacugugg.........................    1      1     S04
............................................................gauuuuuguuacuguuaacugugg.........................    1      0     S04
.............................................................auuuuuguuacuguuaacug............................    2      0     S04
.............................................................auuuuuguuacuguuaacuguggA........................    1      1     S04
..............................................................uuuuuguuacuguuaacu.............................    1      0     S04
..............................................................uuuuuguuacuguuaacugugggg.......................    2      0     S04
...............................................................uuuuguuacuguuaacuguggggc......................    2      0     S04
..................................................................uguuacuguuaacuguggggcg.....................    1      0     S04
..................................................................uguuacuguuaacugugggAcgga...................    1      1     S04
..................................................................uguuacuguuaacuguggggcAga...................    1      1     S04
..................................................................uguuacuguuaacuguggUgcgga...................    1      1     S04
..................................................................uguuacuguuaacuguggggcgga...................    1      0     S04
```

Provisional ID	:	chrA02_186355
Score total	:	0.1
Score for star read(s)	:	-1.3
Score for read counts	:	0
Score for mfe	:	1.4
Score for randfold	:	0
Score for cons. seed	:	
Total read count	:	108
Mature read count	:	106
Loop read count	:	1
Star read count	:	1

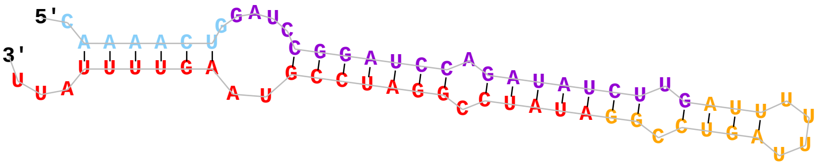

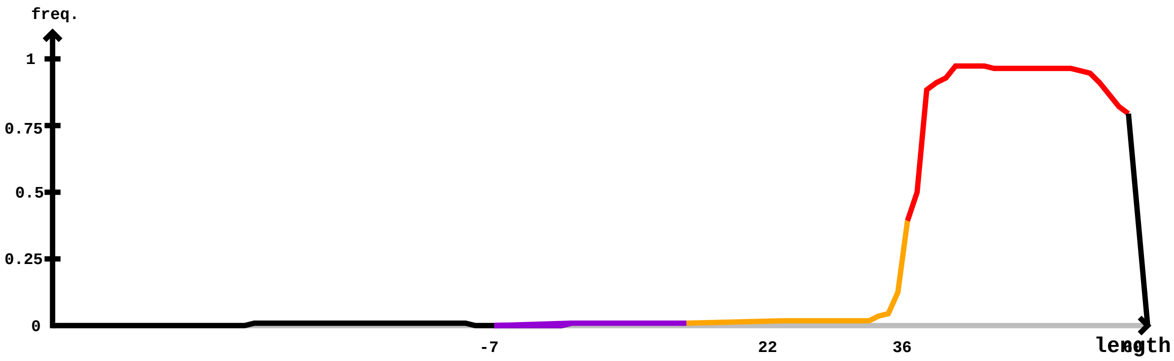


```
                                                                    Star                         Mature
5'- cagaaggaccgaauccggaucaggauaguaaaauauuggaccgucaaaacuggauccggauccagauaucuugauuuuuuaguccggauauccggauccguaaguuuuauua  -3'   obs
    cagaaggaccgaauccggaucaggauaguaaaauauuggaccgucaaaacuggauccggauccagauaucuugauuuuuuaguccggauauccggauccguaaguuuuauua        exp
    .....((.((((((((......)))).........)))).))...(((((.....(((((((.(((((((.((((....)))).))))))).)))))))..))))))....   reads     mm        sample
    ....................................................gauccggauccagauaCcuug.......................................   1         1         S03
    ........................................................................gauuuuuuaguccggauauccAga................   1         1         S03
    ..........................................................................uuuuuuaguccggauauc....................   1         0         S03
    .....................................................................................ggauauccggauccguaaguuuu....   1         0         S03
    ......................................................................................gauauccggauccguaaguu......   1         0         S03
    ......................................................................................gauauccggauccguaaguuuuau..   1         0         S03
    ......................................................................................gauauccggauccguaaguuuuauu.   3         0         S03
    .......................................................................................auauccggauccguaagu.......   1         0         S03
    .......................................................................................auauccggauccguaaguuuuauu.   7         0         S03
    .......................................................................................auauccggauccguaaguuuuauC.   1         1         S03
    .......................................................................................auaCccggauccguaaguuuuauu.   1         1         S03
    ........................................................................................uauccggauccguaaguuuuauC.   1         1         S03
    ........................................................................................uauccggauccguaaguuuuauu.   2         0         S03
    .........................................................................................auccggauccguaaguuuuauC.   1         1         S03
    .........................................................................................Nuccggauccguaaguuuuauu.   1         1         S03
    .........................................................................................auccggauccguaaguuuuauu.   3         0         S03
    .........................................................................................auccggauccguaaguuuuauuG   5         1         S03
    .........................................................................................auccggauccguaaguuuuauua   7         0         S03
    ............................................................................................cggauccguaaguuuuauuU   1         1         S03
    ............................................................................................cggauccguaaguuuuauuG   2         1         S03

    ...................ucaggauaguaaaauauuggacU......................................................................   1         1         S04
    ....................................................................................cggauauccggauccguaaguuu.....   2         0         S04
    ......................................................................................gauauccggauccguaaguuu.....   1         0         S04
    ......................................................................................gauauccggauccguaaguuuu....   1         0         S04
    ......................................................................................gauauccggauccguaaguuuua...   1         0         S04
    ......................................................................................gauGuccggauccguaaguuuuauu.   1         1         S04
    .......................................................................................auauccggauccguaaguuuu....   1         0         S04
    .......................................................................................auauccggauccguaaguuuua...   1         0         S04
    .......................................................................................auauccggauccguaaguuuuC...   1         1         S04
    .......................................................................................auauccggauccguaaguuuuau..   1         0         S04
    .......................................................................................auauccggauccguaaguuuuauu.   14        0         S04
    .......................................................................................auauccggauccguaaguuuuauC.   2         1         S04
```

Star Mature

```
cagaaggaccgaauccggaucaggauaguaaaauauuggaccgucaaaacuggauccggauccagauaucuugauuuuuuaguccggauauccggauccguaaguuuuauua
........................................................................................uauccggauccguaaguuuua...   1   0   S04
........................................................................................uauccggauccguaaguuuuau..   1   0   S04
........................................................................................uauccggauccguaaguuuuauu.   6   0   S04
........................................................................................uauccggauccguaaguuuuauua   1   0   S04
.........................................................................................auccggauccguaaguuu.....   1   0   S04
.........................................................................................auccggauccguaaguuuu....   2   0   S04
.........................................................................................auccggauccguaaguuuua...   1   0   S04
.........................................................................................auccggauccguaaguuuuauu.   3   0   S04
.........................................................................................auccggauccguaaguuuuauua  12   0   S04
.........................................................................................auccggauccguaaguuuuauuC   1   1   S04
.........................................................................................auccggauccguaaguuuuauuG   6   1   S04
..........................................................................................uccggauccguaaguuuuauC.   1   1   S04
..........................................................................................uccggauccguaaguuuuauua   1   0   S04
..........................................................................................uccggauccguaaguuuuauuG   1   1   S04
...........................................................................................ccggauccguaaguuuuauu.   1   0   S04
...........................................................................................ccggauccguaaguuuuauuG   1   1   S04
............................................................................................cggauccguaaguuuuauu.   1   0   S04
............................................................................................cggauccguaaguuuuauuG   1   1   S04
............................................................................................cggauccguaaguuuuauua   1   0   S04
```

Provisional ID	:	chrA02_199685
Score total	:	0
Score for star read(s)	:	-1.3
Score for read counts	:	0
Score for mfe	:	1.2
Score for randfold	:	0
Score for cons. seed	:	
Total read count	:	36
Mature read count	:	36
Loop read count	:	0
Star read count	:	0

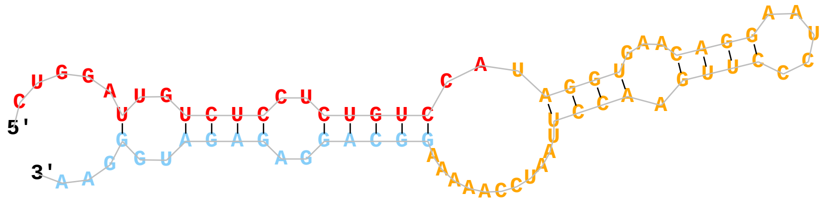

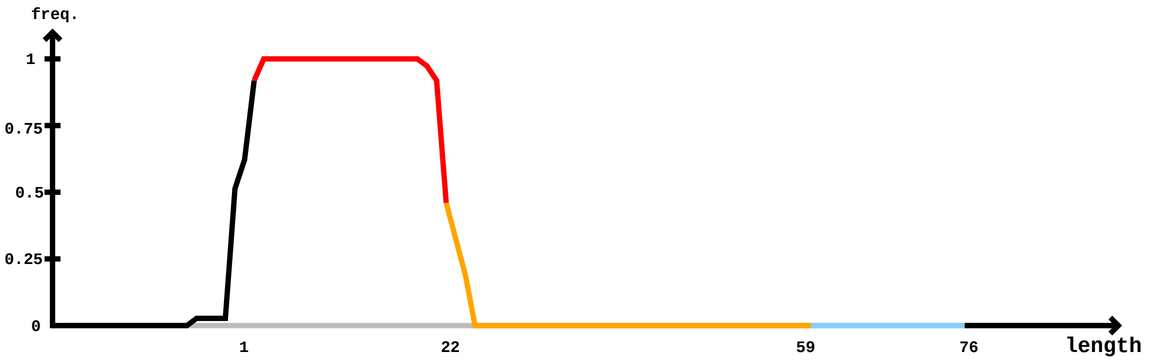

Mature   Star

```
5'- ccaggucuuccccaagcuucuggauugucuccucuguccauaggugaacaggaaucccuugaaccuuaauccaaaaaggcaggagagaugggaaguuuuuggagacugu   -3'    exp
    ...((((((...((((((((....((((((..(((((...((((...((((.....)))).))))............)))))..))))))))))))))..))))))..    reads    mm    sample
    .............aagcuucuggauugucuccucugu........................................................................    1    0    S03
    .................uucuggauugucuccucuguc.......................................................................    1    0    S03
    .................uucuggauugucuccucugucc......................................................................    1    0    S03
    .................uucuggauugucuccucuguccUu....................................................................    1    1    S03
    .................uucuggauugucuccucuguccauU...................................................................    1    1    S03
    ..................ucuggauugucuccucuguccaua...................................................................    1    0    S03
    ....................uggauugucuccucugucc......................................................................    1    0    S03

    .................uucuggauugucuccucuguG.......................................................................    1    1    S04
    .................uucuggauugucuccucugucc......................................................................    5    0    S04
    .................uucuggauugucuccucugucA......................................................................    1    1    S04
    .................uucuggauugucuccucugucU......................................................................    3    1    S04
    .................uucuggauugucuccucuguccC.....................................................................    1    1    S04
    .................uucuggauugucuccucuguccaAa...................................................................    1    1    S04
    .................uucuggauugucuccucuguccauU...................................................................    2    1    S04
    ..................ucuggauugucuccucugucc......................................................................    2    0    S04
    ..................ucuggauugucuccucuguccaua...................................................................    1    0    S04
    ...................cuggauugucuccucugucc......................................................................    3    0    S04
    ...................cuggauugucuccucugucca.....................................................................    5    0    S04
    ...................cuggauugucuccCcugucca.....................................................................    1    1    S04
    ...................cuggauugucuccucuguccG.....................................................................    1    1    S04
    ...................cuggauugucuccucuguccaua...................................................................    1    0    S04
    ....................uggaCugucuccucugucc......................................................................    1    1    S04
    ....................uggauugucuccucugucca.....................................................................    1    0    S04
```

Provisional ID	:	chrA02_random_2682742
Score total	:	78.2
Score for star read(s)	:	3.9
Score for read counts	:	72.5
Score for mfe	:	1.8
Score for randfold	:	0
Score for cons. seed	:	
Total read count	:	154
Mature read count	:	153
Loop read count	:	0
Star read count	:	1

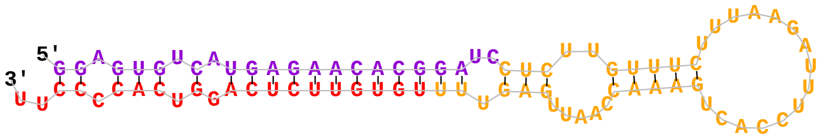

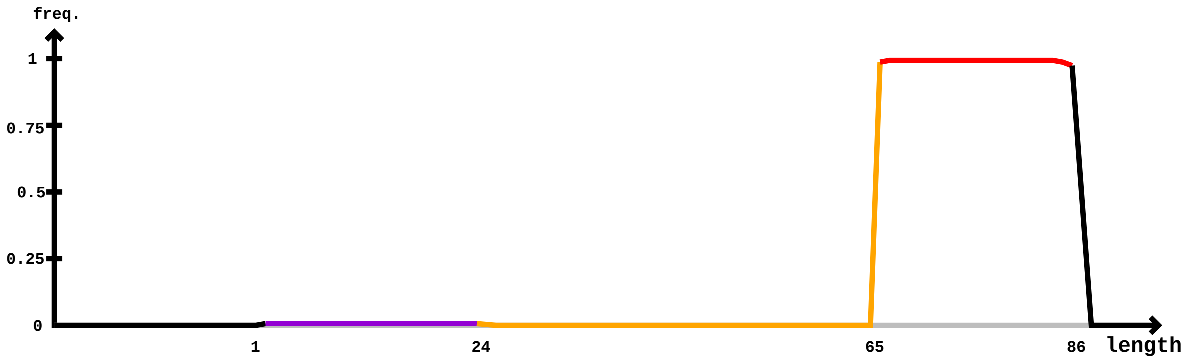

Star Mature

```
5'- acaacaggugagaucucaaaggagugucaugagaacacggaucucuuguuucuuuaagauuuccacugaaaccaauugaguuuuguguucucaggucaccccuuugaaucuc  -3'  obs
    acaacaggugagaucucaaaggagugucaugagaacacggauccucuuguuucuuuaagauuuccacugaaaccaauugaguuuuguguucucaggucaccccuuugaaucuc        exp
    .........(((((.(((((((.(((.(.((((((((((((..(((..(((((..............))))).....))).))))))))))))).).))).)))))))))))  reads  mm  sample
    ....................................................................................uguguucucaggucacccA..........  1  1  S03
    ....................................................................................uguguucucaggucaccccuC........  2  1  S03
    ....................................................................................uguguucucaggucaccccuu........  3  0  S03
    ....................................................................................uguguucucaggucaccccuG........  62  1  S03
    ....................................................................................uguguucucaggucaccccuA........  5  1  S03

    ....................ggagugucaugagaacacggauc......................................................................  1  0  S04
    ....................................................................................uguguucucaggucaccccu.........  1  0  S04
    ....................................................................................uguguucucaggucaccccuC........  2  1  S04
    ....................................................................................uguguucucaggucaccccCu........  1  1  S04
    ....................................................................................uguguucucaggucaccccuA........  4  1  S04
    ....................................................................................uguguucucaggucaccccuu........  7  0  S04
    ....................................................................................uguguucucaggucaccccuG........  63  1  S04
    ....................................................................................uguguucucaggucaccccuuu.......  1  0  S04
    .....................................................................................guguucucaggucaccccu.........  1  0  S04
```

Provisional ID	:	chrA03_213863
Score total	:	0
Score for star read(s)	:	-1.3
Score for read counts	:	0
Score for mfe	:	1.3
Score for randfold	:	0
Score for cons. seed	:	
Total read count	:	42
Mature read count	:	41
Loop read count	:	0
Star read count	:	1

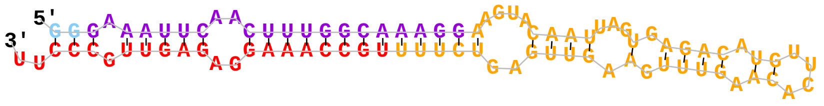

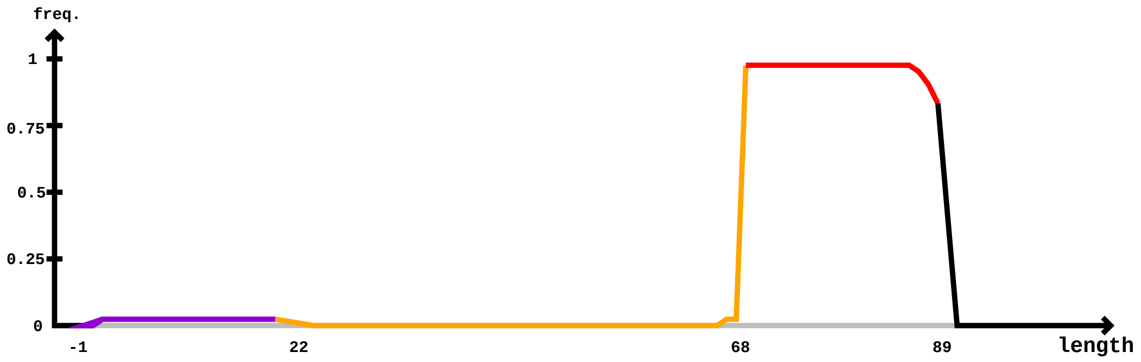

Star **Mature**

```
5'- agggaaauucaacuuuggcaaaggaaguacaauuagugagacauguucacaaguuugaaguugagucuuuugccaaaggagaguugcccuuuacuguucuaucuuuug  -3'  obs
    agggaaauucaacuuuggcaaaggaaguacaauuagugagacauguucacaaguuugaaguugagucuuuugccaaaggagaguugcccuuuacuguucuaucuuuug        exp
    ((((.(((((..(((((((((((((....((((...(.((((.((....)).)))).).))))..)))))))))))))..))))).)))).................. reads  mm  sample
    ......................................................................ugccaaaggagaguugcccu.................. 3      0   S03
    ......................................................................ugccaaaggagaguugcccuA................. 8      1   S03

    ...Uaaauucaacuuuggcaaagg.................................................................................... 1      1   S04
    ...................................................................uuugccaaGggagaguugccc.................... 1      1   S04
    ......................................................................ugccaaaggagaguugcc.................... 1      0   S04
    ......................................................................ugccaaaggagaguugccc................... 1      0   S04
    ......................................................................ugccaaaggagaguugcccCu................. 1      1   S04
    ......................................................................ugccaaaggagaguugcccuA................. 24     1   S04
    ......................................................................ugccaaaggagaguugcccuu................. 2      0   S04
```

Provisional ID	:	chrA03_236012
Score total	:	1.1
Score for star read(s)	:	3.9
Score for read counts	:	-3.3
Score for mfe	:	0.6
Score for randfold	:	0
Score for cons. seed	:	
Total read count	:	5
Mature read count	:	4
Loop read count	:	0
Star read count	:	1

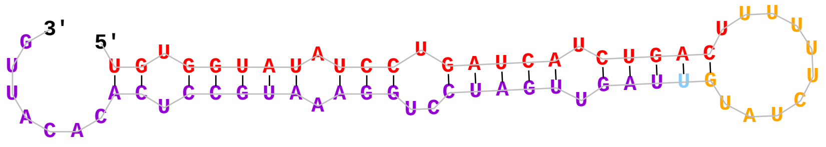

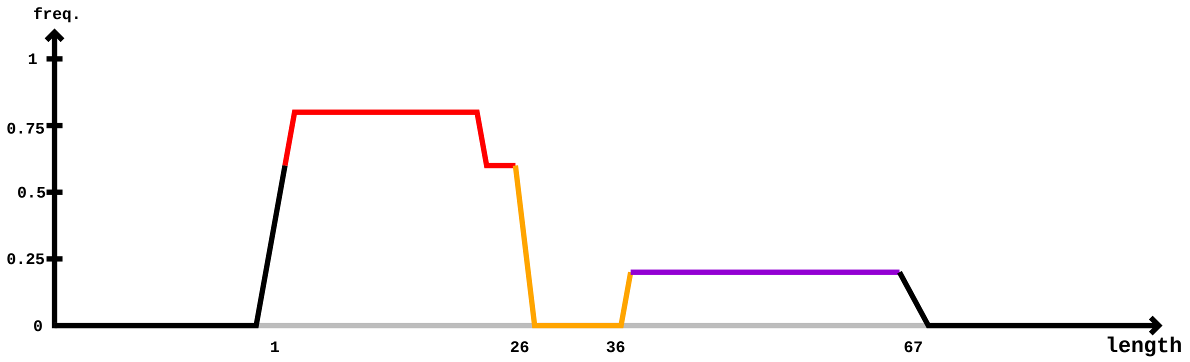


```
                          Mature                     Star
5'- acugacgcaauauugauuagguugugguauauccugaucaucugacuuuuuucuauguuaguugauccuggaaaugccucacacauugauucauaaacuuucaguucuguguu   -3'    obs
    acugacgcaauauugauuagguugugguauauccugaucaucugacuuuuuucuauguuaguugauccuggaaaugccucacacauugauucauaaacuuucaguucuguguu          exp
    ...((((((..(((((..((((((.(((((.(((.(((((.(((((..........))))).))))).))).))))).))...............)))))))))..))))))   reads     mm      sample
    ....................guugugguauauccugaucaucu......................................................................   1         0       S03

    ......................uugugguauauccugaucaucugacu..................................................................   1         0       S04
    .......................ugugguauauccugaucaucugacu.................................................................   1         0       S04
    ........................gugguauauccugaucaucugacu.................................................................   1         0       S04
    ..........................................................uaguugauccuggaaaugccucacacauug.........................   1         0       S04
```

Provisional ID	:	chrA03_243775
Score total	:	0.4
Score for star read(s)	:	-1.3
Score for read counts	:	0
Score for mfe	:	1.7
Score for randfold	:	0
Score for cons. seed	:	
Total read count	:	16
Mature read count	:	4
Loop read count	:	12
Star read count	:	0

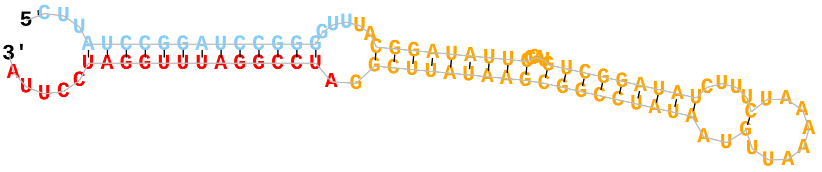

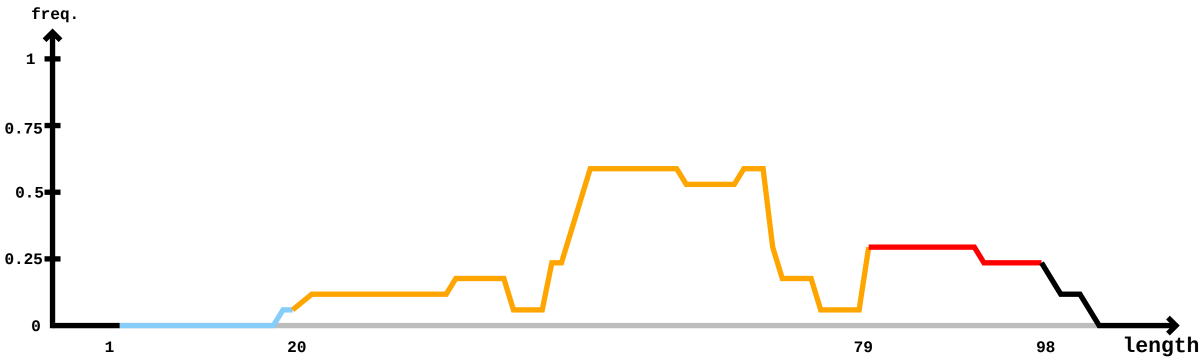

Star Mature

```
5'- cuauucuuauccggauccggggguuuacggauauucggaugucggauaucuuucuaaaaauuguaauauccggcgaauauucggauccggauuuggauccuuaaaauaaauaaaaa   -3'    exp
    ........((((((((((((.....(((((((((....(((((((((....(........)..))))))))))))))))))..)))))))))))).................   reads      mm         sample
    .......................................................................ggcgaauauucggauccggauuugg....................   1          0          S03
    ....................................................................................auccggauuuggauccuuaaaau.........   1          0          S03

    .......................uuuacggauauucggaugucggau.....................................................................   1          0          S04
    .........................uacggauauucggaugucggau.....................................................................   1          0          S04
    .........................................ucggauaucuuucuaaaaauuguG...................................................   1          1          S04
    ...................................................uucuaaaaGuuguaauauccggc..........................................   1          1          S04
    ...................................................uucuaaaaauuguaauauccggcU.........................................   1          1          S04
    ...................................................uucuaaaaauuguaauauccggcg.........................................   1          0          S04
    .....................................................cuaaaaauuguaauauccggc..........................................   2          0          S04
    ......................................................uaaaaauuguaauauccggc..........................................   2          0          S04
    .......................................................aaaaauuguaauaucUggcgaaua.....................................   2          1          S04
    ....................................................................................auccggauuuggauccuua.............   2          0          S04
    ....................................................................................auccggauuuggauccuuaaaaua........   1          0          S04
```

Provisional ID	:	chrA03_259226
Score total	:	0.8
Score for star read(s)	:	-1.3
Score for read counts	:	0
Score for mfe	:	2.1
Score for randfold	:	0
Score for cons. seed	:	
Total read count	:	167
Mature read count	:	162
Loop read count	:	0
Star read count	:	5

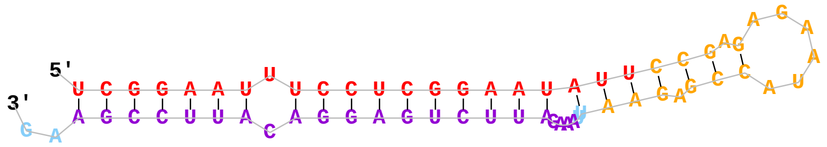

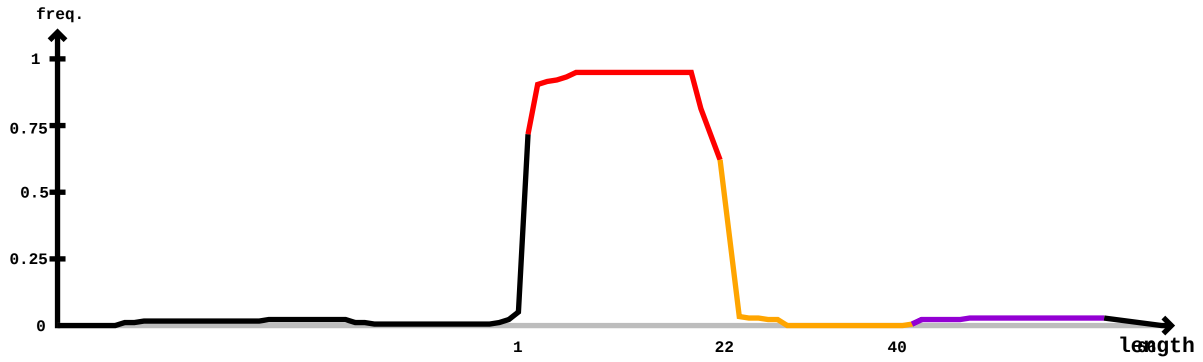

Mature Star

```
5'- cgacgagaauggucgucagaaauuucucgucggaaaauaaaaauuucuucggaauuuccucggaauauuccgagagaauaccgagaauuaaagauucugaggacauuccgaagaa  -3'  obs
    cgacgagaauggucgucagaaauuucucgucggaaaauaaaaauuucuucggaauuuccucggaauauuccgagagaauaccgagaauuaaagauucugaggacauuccgaagaa        exp
    (((((((((...((....))...)))))))))...........((((((((((.((((((((((((((((.(......))).)))).....)))))))))).))))))))))   reads   mm   sample
    ....................aauuucucgucggaaaauaaaaauu......................................................................   1   0   S03
    ..............................................cuucggCauuuccucggaau.................................................   1   1   S03
    ...............................................uucggaauuuccucggaau.................................................   1   0   S03
    ................................................ucggaauuuccucggaau.................................................   11  0   S03
    ................................................ucggaauuuccucggaaua................................................   4   0   S03
    ................................................ucggaauuuccucggaauau...............................................   4   0   S03
    ................................................ucggaauuuccucggaauauu..............................................   34  0   S03
    ................................................ucggaauuuccucggaauauC.............................................   3   1   S03
    ................................................ucggaauuuccCcggaauauuc.............................................   1   1   S03
    .................................................cggaauuuccucggaaua................................................   4   0   S03
    .................................................cggaauuuccucggaauau...............................................   2   0   S03
    .................................................cggaauuuccCcggaauauu..............................................   1   1   S03
    .................................................cggaauuuccucggaauauC.............................................   2   1   S03
    .................................................cggaauuuccucggaauauu..............................................   12  0   S03
    .................................................cggaauuuccucggaauauuc.............................................   1   0   S03
    ..................................................ggaauuuccucggaauau...............................................   2   0   S03
    ...................................................gaauuucGucggaauauuccgaga........................................   1   1   S03
    ....................................................aauuuccucggaauauuccga..........................................   1   0   S03
    ....................................................aauuucGucggaauauuccgaga........................................   1   1   S03
    .....................................................auuuccucggaauauucc............................................   1   0   S03
    .........................................................................................aaagauucugaggacauuccga....   3   0   S03

    .....agaauggucgucagaaauuucucU......................................................................................   1   1   S04
    .....agaauggucgucagaaauuucucg......................................................................................   1   0   S04
    .......aaCggucgucagaaauuucucguc....................................................................................   1   1   S04
    .............................................ucAucggaauuuccucggaauauu..............................................   2   1   S04
    ..............................................cuucggaauuuccucggaauau...............................................   1   0   S04
    ...............................................uucggaauuuccucggaauau...............................................   1   0   S04
    ...............................................uucggaauuuccucggaaAau...............................................   1   1   S04
    ...............................................Nucggaauuuccucggaauauu..............................................   1   1   S04
    ...............................................uucggaauuuccucggaauauC.............................................   1   1   S04
    ................................................ucggaauuuccucggaau.................................................   11  0   S04
    ................................................ucggaauuuccucggaaua................................................   7   0   S04
```

```
                                               Mature                                   Star
cgacgagaauggucgucagaaauuucucgucggaaaauaaaauuucuucggaauuuccucggaauauuccgagagaauaccgagaauuaaagauucugaggacauuccgaagaa
...............................................ucggaauuuccucggaauau...............................................	4	0	S04
...............................................ucggaauCuccucggaauauu..............................................	1	1	S04
...............................................ucggaaCuuccucggaauauu..............................................	1	1	S04
...............................................ucggaauuuccucggaauauu..............................................	32	0	S04
...............................................Ncggaauuuccucggaauauu..............................................	1	1	S04
...............................................ucggaauuuccucggaauauC..............................................	1	1	S04
...............................................ucggaauuuccucggaauauuc.............................................	3	0	S04
................................................cggaauuuccucggaaua................................................	2	0	S04
................................................cggaauuuccucggaauau...............................................	2	0	S04
................................................cggaauuuccucggaauauu..............................................	6	0	S04
................................................cggaauuuccucggaauauuc.............................................	1	0	S04
....................................................auuuccucggaauauuccgagU........................................	1	1	S04
....................................................auuucGucggaauauuccgaga........................................	1	1	S04
.......................................................................................uaaagauucugaggacauuccgaG...	1	1	S04
.............................................................................................uuAugaggacauuccgaa...	1	1	S04
```

Provisional ID	:	chrA03_260104
Score total	:	0.3
Score for star read(s)	:	3.9
Score for read counts	:	-4.9
Score for mfe	:	1.4
Score for randfold	:	0
Score for cons. seed	:	
Total read count	:	2
Mature read count	:	1
Loop read count	:	0
Star read count	:	1

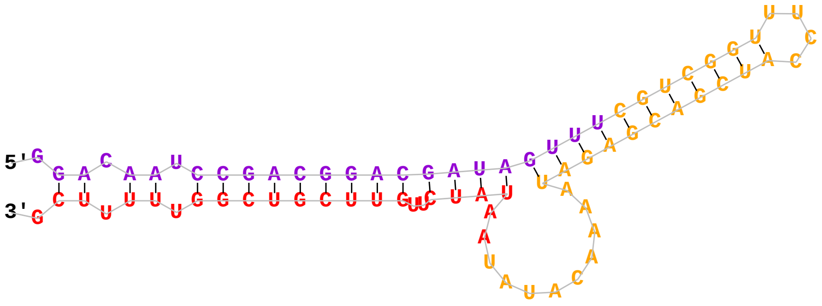

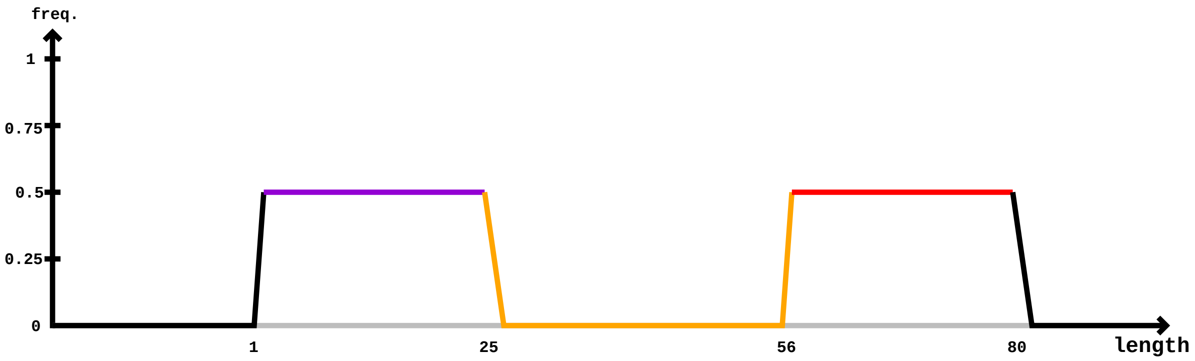


```
                    Star                                                   Mature
5'- aaaacaugcuacauuccgauggacaauccgacggacgauaguuucgucgguuuccaucgacgagauaaaacauauaauaucuuguucgucgguuuuucgucagaaauuucugau   -3'    obs
    aaaacaugcuacauuccgauggacaauccgacggacgauaguuucgucgguuuccaucgacgagauaaaacauauaauaucuuguucgucgguuuuucgucagaaauuucugau          exp
    .............(((.((((((.((.((((((((((((((((((((((((....)))))))))))...........))))..))))))))))).)))))).))).........   reads   mm   sample
    ....................ggacaauccgacggacgauaguuu......................................................................   1       0    S04
    ...........................................................................aauaucuuguucgucgguuuuucg...............   1       0    S04
```

Provisional ID : chrA03_266631
Score total : 0.9
Score for star read(s) : -1.3
Score for read counts : 0
Score for mfe : 2.2
Score for randfold : 0
Score for cons. seed :
Total read count : 106
Mature read count : 106
Loop read count : 0
Star read count : 0

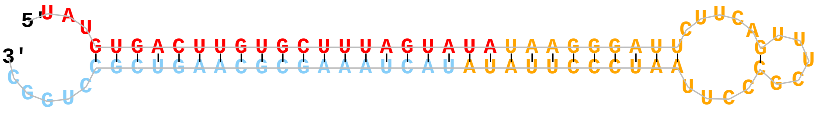

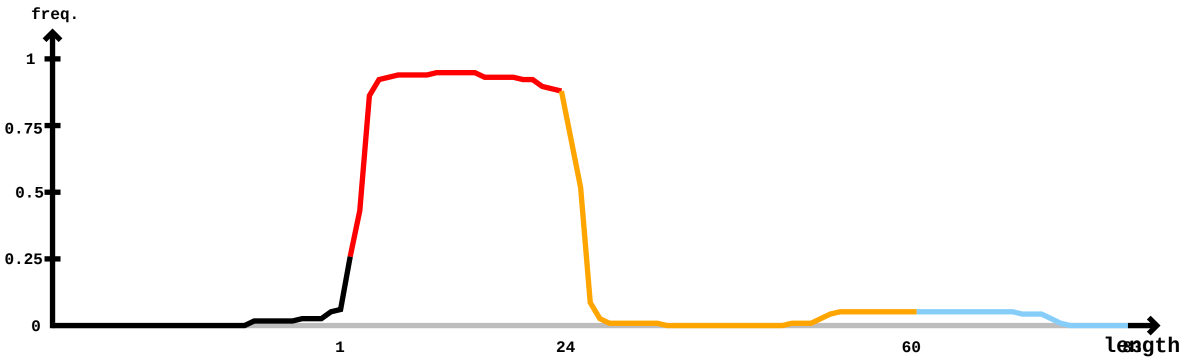

Mature Star

```
5'- uaaaauccaaauggcaaauuuugauuuguuaugugacuugugcuuuaguauauaagggauucuucaguuucgcccuuaaucccuuauauacuaaagcgcaagucgccuggcga   -3'    exp
    ..(((((.((((.....)))).)))))((((.(((((((((((((((((((((((((((((.....(.....)....))))))))))))))))))))))))))))).))))..    reads    mm    sample
    ...................uuugauuuguuaugugacuugugc......................................................................    1    0    S03
    ...................uuugauuuguuaugugacuugugU......................................................................    1    1    S03
    ...........................guuaugugacuugugcuuuaguU...............................................................    1    1    S03
    ............................uuauguAacuugugcuuuaguaua.............................................................    1    1    S03
    .............................uaugugacuugugcuuuaguaua.............................................................    3    0    S03
    .............................uaugugacuugugcuuCaguaua.............................................................    1    1    S03
    .............................uaugugacuugugcuuuaguaCa.............................................................    2    1    S03
    .............................uaugugacuugugcuuuaguauau............................................................    1    0    S03
    ..............................augugacuugugcuuuaguaua.............................................................    1    0    S03
    ..............................augugacuugugcuuuaguauau............................................................    2    0    S03
    ..............................augugacuugugcuuuaguauaua...........................................................    2    0    S03
    ..............................augugacuuguAcuuuaguauaua...........................................................    1    1    S03
    ...............................ugugacuugugcuuuagu................................................................    1    0    S03
    ...............................ugugacuuguAcuuuaguaua.............................................................    1    1    S03
    ...............................ugugacuugugcuuuaguaua.............................................................    3    0    S03
    ...............................ugugacuugugcuuuaguauau............................................................    1    0    S03
    ...............................ugugacuugugcuuuGguauaua...........................................................    1    1    S03
    ...............................ugugacuuguAcuuuaguauaua...........................................................    1    1    S03
    ...............................ugugacuugugcuuuaguauauG...........................................................    1    1    S03
    ...............................ugugacuugugcuuuaguauauU...........................................................    1    1    S03
    ...............................ugugacuugugcuuuaguauaua...........................................................    13   0    S03
    ................................gugacuugugcuuuaguauau............................................................    1    0    S03
    ................................gugacuugugcuuuaguauaua...........................................................    2    0    S03
    ................................gugacuugugcuuuaguauauaag.........................................................    1    0    S03
    .................................ugacuugugcuuuaguauaua...........................................................    1    0    S03
    ...........................................................................uuaaucccuuauauacuaaagcgc..............    1    0    S03
    ..............................................................................aucccuuauauacuaaagcgcaaA...........    1    1    S03
    ..............................................................................aucccuuauauacuaaagcgcaag...........    1    0    S03
    ...............................................................................ucccuuauauacuaaagcgcaagu..........    1    0    S03
    ...............................................................................ucAcuuauauacuaaagcgcaagu..........    1    1    S03

    ........................uuGguuaugugacuugugcuuua..................................................................    1    1    S04
    ...........................guuaugugacuugugcuuuagu................................................................    1    0    S04
    ...........................guuaugugacuugugcuuuaguauau............................................................    1    0    S04
```

Mature / Star			
uaaaauccaaauggcaaauuuugauuuguuaugugacuugugcuuuaguauauaagggauucuucaguuucgcccuuaaucccuuauauacuaaagcgcaagucgccuggcga			
.............................uaugugacuugugcuuuaguauG...	1	1	S04
.............................uaugugacuugugcuuuaguaua...	13	0	S04
.............................uaugugacuugugcuuuaguauau..	1	0	S04
.............................uaugugacuugugcuuuaguauauaaA...	1	1	S04
..............................augugacuugugcuuuagu..	1	0	S04
..............................augugacuugugcuuuaguau..	1	0	S04
..............................augugacuugugcuuuaguauau..	1	0	S04
..............................augugacuugugcuuuaguauaua...	9	0	S04
..............................augugacuugugcuuuaguauauG...	1	1	S04
..............................augugacuugugcuuuaguauauaa..	1	0	S04
...............................ugugacuugugcuuuaguaua...	1	0	S04
...............................ugugacuugugcuuuaguauau..	5	0	S04
...............................ugugacuugugcuuuaguauaG..	1	1	S04
...............................ugugacuugugcuuuaguaAaua...	1	1	S04
...............................ugugacuugugcuuuaguauaua...	12	0	S04
...............................ugugacuugugcuuuaguauaCa...	1	1	S04
...............................ugugacuugugcuuuaguauauG...	1	1	S04
...............................ugugacuugugcuuuaguauauU...	1	1	S04
...............................ugugacuugugcuuuaguauauaa..	4	0	S04
................................gugacuugugcuuuaguauaua...	1	0	S04
................................gugacuugugcuuuaguauauaa..	2	0	S04
..................................gacuugugcuuuaguaua...	1	0	S04
......................................ugugcuuuaguauauaagggauuc...	1	0	S04
....cccuuauauacuaaagcgcaaguc.........	1	0	S04

Provisional ID	:	chrA03_266922
Score total	:	11.2
Score for star read(s)	:	3.9
Score for read counts	:	5.2
Score for mfe	:	2
Score for randfold	:	0
Score for cons. seed	:	
Total read count	:	22
Mature read count	:	16
Loop read count	:	0
Star read count	:	6

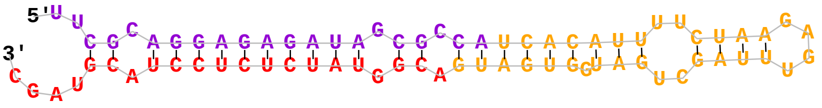

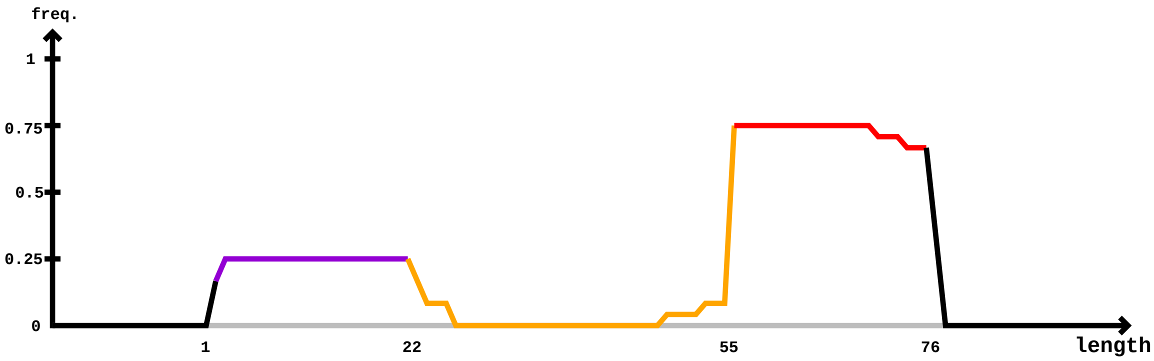


```
                 Star                                              Mature
5'- cacauauagauuugcuucgcaggagagauagcgccaucacauuuucuaagaguuuagcugauggugaugacgguaucucuccuacguagcuauccuuauaugugcaucua  -3'   obs
    cacauauagauuugcuucgcaggagagauagcgccaucacauuuucuaagaguuuagcugauggugaugacgguaucucuccuacguagcuauccuuauaugugcaucua        exp
    ((((((((((..(((.((.((((((((((.((.(((((((((..((((....))))..))).)))))).)).)))))))))).)).))).)))..))))))))......   reads      mm        sample
    ...............uucgcaggagagauagcgcca..........................................................................   1          0         S03
    ................ucgcaggagagauagcgccaucac......................................................................   1          0         S03
    ...................................................................augacgguaucucuccuacguagA...................   1          1         S03
    .....................................................................acgguaucucuccuacgu.......................   1          0         S03
    .....................................................................acgguaucucuccuacguagU....................   1          1         S03
    .....................................................................Gcgguaucucuccuacguagc....................   1          1         S03
    .....................................................................acgguaucucuccuacguagc....................   5          0         S03

    ...............uucgcaggagagauagcgccG..........................................................................   1          1         S04
    ...............uucgcaggagagauagcgccU..........................................................................   1          1         S04
    ...............uucgcaggagagauagcgcca..........................................................................   1          0         S04
    ................ucgcaggagagauagcgccaucaA......................................................................   1          1         S04
    ..............................................................gCugaugacgguaucucuccua..........................   1          1         S04
    .....................................................................acgguaucucuccuacguagU....................   1          1         S04
    .....................................................................acgguaucucuccuacguagc....................   6          0         S04
    .....................................................................acgguaucucuccuacguagA....................   1          1         S04
```

Provisional ID : chrA03_275572
Score total : 44.5
Score for star read(s) : 3.9
Score for read counts : 53.7
Score for mfe : -13
Score for randfold : 0
Score for cons. seed :
Total read count : 117
Mature read count : 116
Loop read count : 0
Star read count : 1

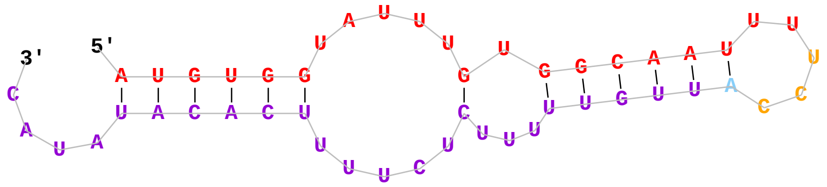

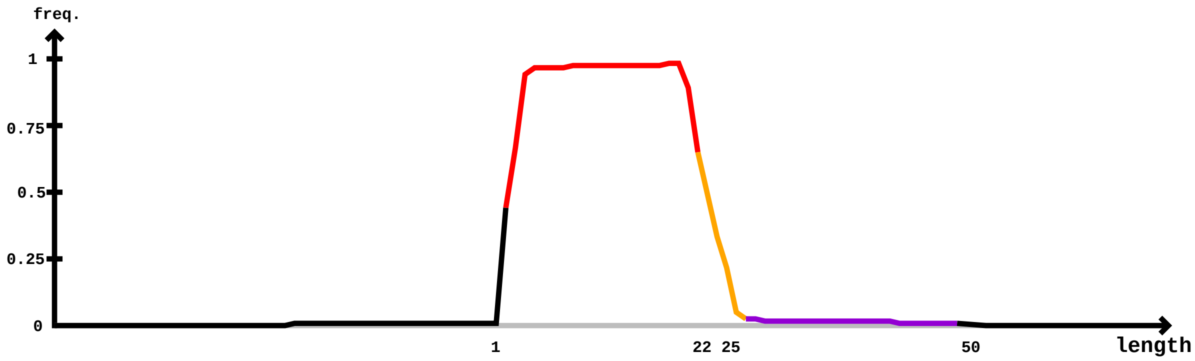

Mature Star

```
5'- uuauccuuuguuaaaagugaugguaaaaguuauuaguguaaacauaugugguauuuguggcaauuuuccauuguuuuucucuuuucacauauacgaaauuuuuuccuuuauauu   -3'    obs
    uuauccuuuguuaaaagugaugguaaaaguuauuaguguaaacauaugugguauuuguggcaauuuuccauuguuuuucucuuuucacauauacgaaauuuuuuccuuuauauu          exp
    ((((((((....)))).)))))..((((..(..(((......((((((((.....(.((((((.....))))))...).....))))))))).....)))..)..)))).....   reads   mm   sample
    .......................uaaaaguuauuaguguaGacaua....................................................................   1       1    S03
    .............................................augugguauuuguggcaau..................................................   3       0    S03
    .............................................augugguauuuguggcaauu.................................................   5       0    S03
    .............................................augugguaAuuguggcaauu.................................................   1       1    S03
    .............................................augugguauuuguggcaauuu................................................   9       0    S03
    .............................................augugguauuuguggcaauuuu...............................................   3       0    S03
    .............................................augugguauuuguggcaauuuuc..............................................   2       0    S03
    .............................................augugguauuuguggcaauuuucc.............................................   5       0    S03
    ..............................................ugugguauuuguggcaau..................................................   4       0    S03
    ..............................................ugugguauuuguggcaauu.................................................   4       0    S03
    ..............................................ugugguauuuguggcaauuu................................................   3       0    S03
    ..............................................ugugguauuuguggcaauuuuc..............................................   1       0    S03
    ..............................................ugugguauuuguggcaauuuuA..............................................   1       1    S03
    ..............................................ugugguauuuguggcaauuuucc.............................................   5       0    S03
    ..............................................ugugguauuuguggcaauuuuccG............................................   1       1    S03
    ...............................................gugguauuuguggcaauu.................................................   7       0    S03
    ...............................................gugguauuuguggcaauuu................................................   4       0    S03
    ...............................................gugguauuuguggcaauuuu...............................................   1       0    S03
    ...............................................gugguauuuguggcaauuuGc..............................................   3       1    S03
    ...............................................gugguauuuguggcaauuuuc..............................................   1       0    S03
    ...............................................gugguauuuguggcaauuuucca............................................   2       0    S03
    ................................................ugguauuuguggcaauuu................................................   1       0    S03
    ..............................................................auuuuccauuguuuuucucuuuuc............................   1       0    S03

    .............................................augugguauuuguggcaau..................................................   2       0    S04
    .............................................augugguauuuguggcaauu.................................................   2       0    S04
    .............................................augugguauuuguggcaauuu................................................   7       0    S04
    .............................................augugguauuuguggcaauuuu...............................................   4       0    S04
    .............................................augugguauuuguAgcaauuuuc..............................................   1       1    S04
    .............................................augugguauuuguggcaauuuuA..............................................   1       1    S04
    .............................................augugguauuuguggcaauuuuc..............................................   3       0    S04
    .............................................augugguauuuguggcaauuuucc.............................................   4       0    S04
    ..............................................ugugguauuuguggcaau..................................................   2       0    S04
```

```
                                             Mature                  Star
uuauccuuuguuaaaagugaugguaaaaguuauuaguguaaacauaugugguauuuguggcaauuuuccauuguuuuucucuuuucacauauacgaaauuuuuuccuuuauauu
..............................................ugugguauuuguggcaauu.................................................   3   0   S04
..............................................ugugguauuuguggcaauuu................................................   1   0   S04
..............................................ugugguauuuguggcaauuuuc..............................................   1   0   S04
..............................................ugugguauuuguggcaauuuucc.............................................   2   0   S04
...............................................gugguauuuguggcaauu.................................................   7   0   S04
...............................................gugguauuuguggcaauuu................................................   3   0   S04
...............................................gugguauuuguggcaauuuucc.............................................   3   0   S04
...............................................guggAauuuguggcaauuuucc.............................................   1   1   S04
...............................................gugguauuuguggcaauuuucca............................................   1   0   S04
................................................ugguauuuguggcaauuu................................................   2   0   S04
....................................................auuuguggcaauuuuccauu..........................................   1   0   S04
......................................................................uuguuuuucucuuuucacauauac....................   1   0   S04
```

Provisional ID	:	chrA03_276196
Score total	:	0.8
Score for star read(s)	:	3.9
Score for read counts	:	4.2
Score for mfe	:	-7.2
Score for randfold	:	0
Score for cons. seed	:	
Total read count	:	20
Mature read count	:	18
Loop read count	:	0
Star read count	:	2

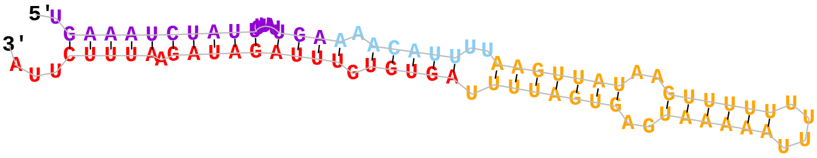

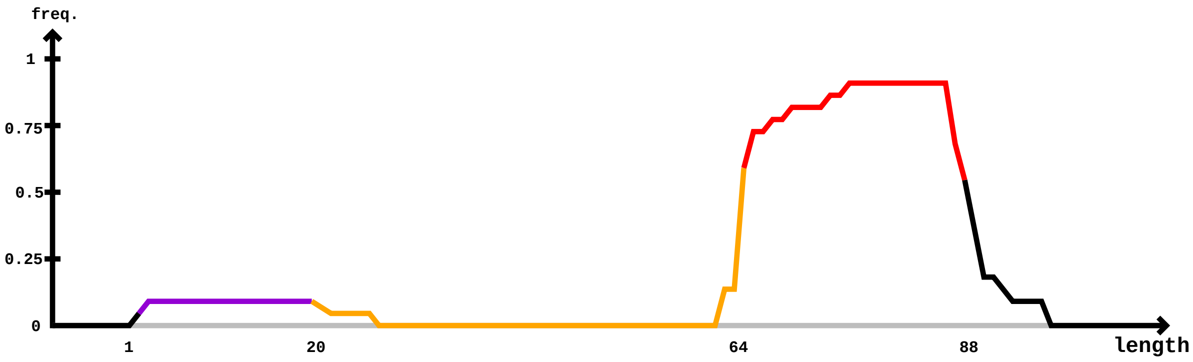

Star Mature

```
5'- aauacuuugaaaucuauuguuauugaaaacauuuuaaguuauaaguuuuuuuuuaaaaaugagugauuuuaguguguuuagauagaauuucuuaguuaaaaauuuuaaaauuua -3'   obs
    aauacuuugaaaucuauuguuauugaaaacauuuuaaguuauaaguuuuuuuuuaaaaaugagugauuuuaguguguuuagauagaauuucuuaguuaaaaauuuuaaaauuua        exp
    ...(((..((((((((((.....((((.(((((..(((((((..((((((....))))))..))))))).))))).))))))))).)))))..)))..................   reads   mm   sample
    ....................................................................uuaguguguuuagauagaauuucu......................   1       0    S03
    ......................................................................aguguguuuagauagaauuucuu.....................   2       0    S03
    ......................................................................aguguguuuagauagaauuucuua....................   3       0    S03
    .......................................................................guguguuuagauagaauuucu......................   2       0    S03
    ...........................................................................guuuagauagaauuucuuaguuG................   1       1    S03
    ...............................................................................agauagaauuucuuaguuaaaaC............   1       1    S03

    .......ugaaaucuauuguuaCuga........................................................................................   1       1    S04
    ........gaaaucuauugGuauugaaaacau..................................................................................   1       1    S04
    ....................................................................uuaguguguuuagauagaauuucu......................   2       0    S04
    ......................................................................aguguguuuagauagaauuucuu.....................   1       0    S04
    ......................................................................aguguguuuagauagaauuucuua....................   1       0    S04
    ......................................................................aguguguuuagauagaauuucuuC....................   1       1    S04
    ......................................................................aguguguuuagauagaauuucuuG....................   1       1    S04
    ......................................................................aguUuguuuagauagaauuucuua....................   1       1    S04
    .......................................................................guguguuuagauagaauuucuuag...................   1       0    S04
    .........................................................................guguuuagauagaauuucuuaguu.................   1       0    S04
    .................................................................................auagaauuucuuaguuaaaaC............   1       1    S04
```

Provisional ID	:	chrA03_281032
Score total	:	21.3
Score for star read(s)	:	3.9
Score for read counts	:	15.9
Score for mfe	:	1.5
Score for randfold	:	0
Score for cons. seed	:	
Total read count	:	43
Mature read count	:	41
Loop read count	:	0
Star read count	:	2

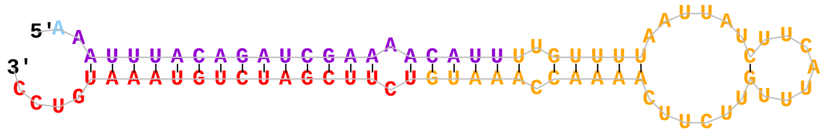

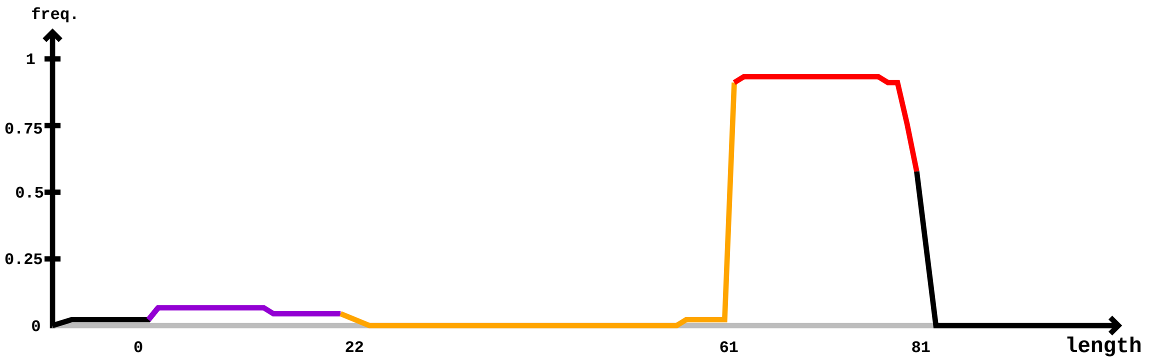

Star **Mature**

```
5'- uuccucggaaauuuacagaucgaaaacauuuuguuuuaauuaucuucauuuguucuucaaaaccaaaugucuucgaucuguaaaugucc aaggaaagggaaauagaagg -3'   obs
    uuccucggaaauuuacagaucgaaaacauuuuguuuuaauuaucuucauuuguucuucaaaaccaaaugucuucgaucuguaaaugucc aaggaaagggaaauagaagg        exp
```

```
5'- uuccucggaaauuuacagaucgaaaacauuuuguuuuaauuaucuucauuuguucuucaaaaccaaaugucuucgaucuguaaaauguccaaggaaagggaaauagaagg -3'   obs
```

```
5'- uuccucggaaauuuacagaucgaaaacauuuuguuuuaauuaucuucauuuguucuucaaaaccaaaugucuucgaucuguaaaugucc aaggaaagggaaauagaagg
```

```
5'- uuccucggaaauuuacagaucgaaaacauuuuguuuuaauuaucuucauuuguucuucaaaaccaaaugucuucgaucuguaaaugucc
```

```
5'- uuccucggaaauuuacagaucgaaaacauuuuguuuuaauuaucuucauuuguucuucaaaaccaaaugucuucgaucuguaaauguccaaggaaagggaaauagaagg -3'   obs
    uuccucggaaauuuacagaucgaaaacauuuuguuuuaauuaucuucauuuguucuucaaaaccaaaugucuucgaucuguaaauguccaaggaaagggaaauagaagg        exp
    (((((.(((.((((((((((((((.((((((.(((((......(.......)......))))).)))))).)))))))))))))).))).))))).............. reads  mm  sample
    uuccucggaaauuuacagauc........................................................................................ 1      0   S03
    .....................................................................ucuucgaucuguaaaugu...................... 3      0   S03
    .....................................................................ucuucgaucuguaaauUu...................... 1      1   S03
    .....................................................................ucuucgaucuguaaauguA..................... 1      1   S03
    .....................................................................ucuucgaucuguaaauguc..................... 3      0   S03
    .....................................................................ucuucgaucuguaaaugucA.................... 4      1   S03
    .....................................................................ucuucgaucuguaaaugucU.................... 4      1   S03
    .....................................................................ucuucgaucuguaaaugucc.................... 5      0   S03

    .........Cauuuacagaucgaaaacauu............................................................................... 2      1   S04
    ................................................................aaaugucuucgaucuguaaau........................ 1      0   S04
    .....................................................................ucuucgaucuguaaaugu...................... 1      0   S04
    .....................................................................ucuucgaucuguaaauUu...................... 1      1   S04
    .....................................................................ucuucgaucuguaaaugC...................... 1      1   S04
    .....................................................................ucuucgaucuguaaauguU..................... 1      1   S04
    .....................................................................ucuucgaucuguaaauguc..................... 2      0   S04
    .....................................................................ucuucgaucuguGaauguc..................... 1      1   S04
    .....................................................................ucuucgaucuguaaaugucU.................... 4      1   S04
    .....................................................................ucuucgaucuguaaaugucc.................... 5      0   S04
    .....................................................................ucuucgaucCguaaaugucc.................... 1      1   S04
    .....................................................................ucuucgaucuguaaaugucA.................... 1      1   S04
    .....................................................................ucuucgaucuguaaauguccU................... 1      1   S04
    ......................................................................cuucgaucuguaaaugucc.................... 1      0   S04
```

Provisional ID	:	chrA03_284953
Score total	:	0.3
Score for star read(s)	:	-1.3
Score for read counts	:	0
Score for mfe	:	1.6
Score for randfold	:	0
Score for cons. seed	:	
Total read count	:	163
Mature read count	:	156
Loop read count	:	0
Star read count	:	7

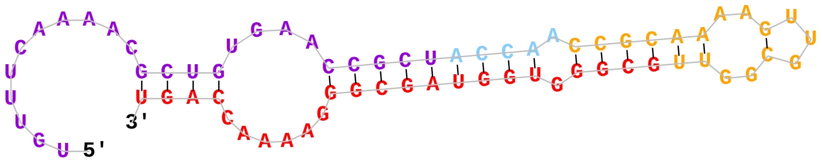

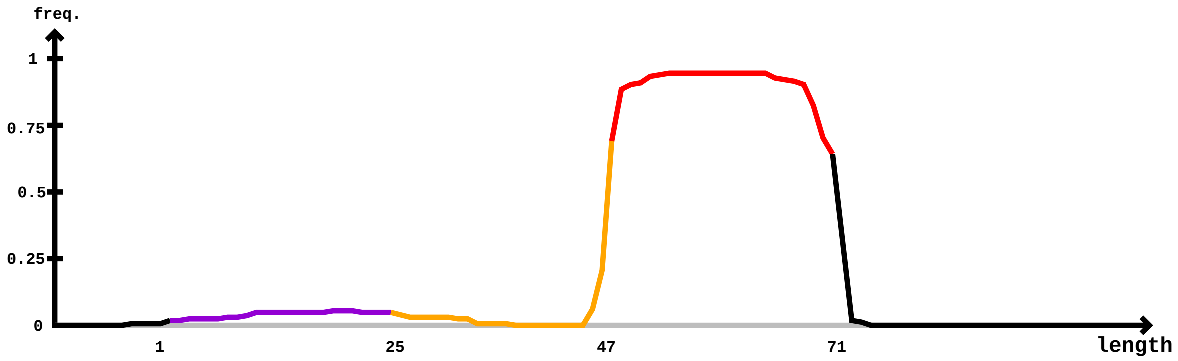

Star **Mature**

```
5'- uuugugugauuguuucaaaacgcugugaaccgcuaccaaccgcaaaaguugcgguugcgggugguagcgggaaaaccagucaugcccuaaacugauauuuacaugaaguaaa   -3'    obs
    uuugugugauuguuucaaaacgcugugaaccgcuaccaaccgcaaaaguugcgguugcgggugguagcgggaaaaccagucaugcccuaaacugauauuuacaugaaguaaa          exp
    ...(((((((((((((....((((....((((((..((((((((.....))))))))..)))))))))).)))).)))))))))............(((((.....)))))   reads     mm     sample
    ..................aaUgcugugaaccgcuaccaaccg......................................................................   1         1      S03
    ...........................aaccgcuaccaaccgcaaa..................................................................   1         0      S03
    ......................................................uugcgggugguagcgggaa.......................................   1         0      S03
    ......................................................uugcgggugguagcgggaaa......................................   1         0      S03
    ......................................................uugcgggugguagcgggaaaac....................................   1         0      S03
    ......................................................uugcgggugguagcgggaaaacc...................................   3         0      S03
    .......................................................ugcgggugguagcgggaa.......................................   1         0      S03
    .......................................................ugcgggugguagcgggaaaU.....................................   1         1      S03
    .......................................................ugcgggugguagcgggaaaacc...................................   2         0      S03
    .......................................................ugcgggugguagcgggaaaaccU..................................   1         1      S03
    .......................................................ugcgggugguagcgggaaaGcca..................................   1         1      S03
    .......................................................ugcgggugguagcgggaaaaccag.................................   2         0      S03
    .......................................................Ngcgggugguagcgggaaaaccag.................................   1         1      S03
    .......................................................uCcgggugguagcgggaaaaccaguc...............................   1         1      S03
    ........................................................Ucgggugguagcgggaaaacc...................................   1         1      S03
    ........................................................gcgggugguagcgggaaaacc...................................   3         0      S03
    ........................................................gcgggugguagcgggaaaacca..................................   4         0      S03
    ........................................................gcgggGgguagcgggaaaaccagu................................   1         1      S03
    ........................................................gcgggugguagcgggaaaaccagC................................   4         1      S03
    ........................................................gcgggugguagcgggaaaaccagu................................   22        0      S03
    ........................................................gcgggugguagcgggaaaaccagG................................   2         1      S03
    ........................................................gcgggugguagcgggaaaaccagA................................   1         1      S03
    ........................................................gcgggugguagcgggaaaaccaguU...............................   1         1      S03
    .........................................................cgggugguagcgggaaaacca..................................   3         0      S03
    .........................................................cgggugguagcgggaaaaccagC................................   1         1      S03
    .........................................................cgggugguagcgggaaaaccagu................................   3         0      S03
    .........................................................cgggugguagcgggaaaaccaguU...............................   2         1      S03
    .........................................................cgggugguagcgggaaaaccaguc...............................   8         0      S03
    ..........................................................gggugguagcgggaaaaccagu................................   2         0      S03
    ............................................................gugguagcgggaaaaccagu................................   1         0      S03
    ............................................................gugguagcgggaaaaccaguc...............................   1         0      S03
    ............................................................gugguagcgggaaaaccaguca..............................   1         0      S03
    .............................................................ugguagcgggaaaaccagucau.............................   1         0      S03
```

Star Mature

```
uuugugugauuguuucaaaacgcugugaaccgcuaccaaccgcaaaaguugcgguugcgggugguagcgggaaaaccagucaugcccuaaacugauauuuacaugaaguaaa
..............................................................gguagcgggaaaaccagu................................	1	0	S03

......ugauuguuucaaaaUgcugugaac..................................................................................	1	1	S04
..........uguuucaaaacgcugugaaccgcA..............................................................................	1	1	S04
..........uguuucaaaacgcugugaaccgcu..............................................................................	1	0	S04
............uuucaaaacgcugugaaccgcu..............................................................................	1	0	S04
................aaaacgcugugaaccgcuaccaac........................................................................	1	0	S04
...................acgcugugaaccgcuaccaaccg......................................................................	1	0	S04
...................acgcugugaaccgcuaccaaccA......................................................................	1	1	S04
......................................................uugcgggugguagcgggaa.......................................	1	0	S04
......................................................uugcgggugguagcgggaaaacc...................................	1	0	S04
......................................................uugcgggugguagcgggaGaacca..................................	1	1	S04
......................................................uugcgggugguagcgggaaaacca..................................	1	0	S04
.......................................................ugcgggugguagcgggaaaac....................................	1	0	S04
.......................................................ugcgggugguagcgggaaaacc...................................	1	0	S04
.......................................................ugcgggugguagcgggaaaacca..................................	6	0	S04
.......................................................ugcgggugguagcgggaaGaccag.................................	1	1	S04
.......................................................ugcgggugguagcgggaaaaccag.................................	5	0	S04
........................................................gcgggugguagcgggaaaGcc...................................	1	1	S04
........................................................gcgggugguagcgggaaaacca..................................	1	0	S04
........................................................gcgggCgguagcgggaaaacca..................................	1	1	S04
........................................................gcgggugguagcgggaaaaccag.................................	1	0	S04
........................................................gcgggugguagcgggaaaaccagC................................	4	1	S04
........................................................gcgggGgguagcgggaaaaccagu................................	1	1	S04
........................................................gcgggugguagcgggaaaaccagu................................	32	0	S04
.........................................................cgggugguagcgggaaaacc...................................	1	0	S04
.........................................................cgggugguagcgggaaaacca..................................	1	0	S04
.........................................................cgggugguagcgggaaaaccagu................................	1	0	S04
.........................................................cgggugguagcgggaaaaccaguU...............................	2	1	S04
.........................................................cgggugguagcgggaaaaccaguc...............................	10	0	S04
..........................................................gggugguagcgggaaaaccaguc...............................	1	0	S04
...........................................................ggugguagcgggaaaaccagucau.............................	1	0	S04
............................................................gugguagcgggaaaaccaguc...............................	1	0	S04
```

Provisional ID	:	chrA03_288587
Score total	:	0.7
Score for star read(s)	:	-1.3
Score for read counts	:	0
Score for mfe	:	2
Score for randfold	:	0
Score for cons. seed	:	
Total read count	:	54
Mature read count	:	14
Loop read count	:	1
Star read count	:	39

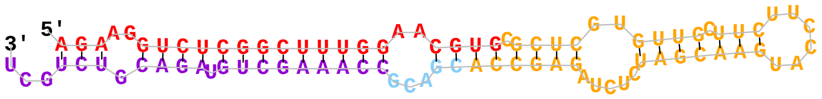

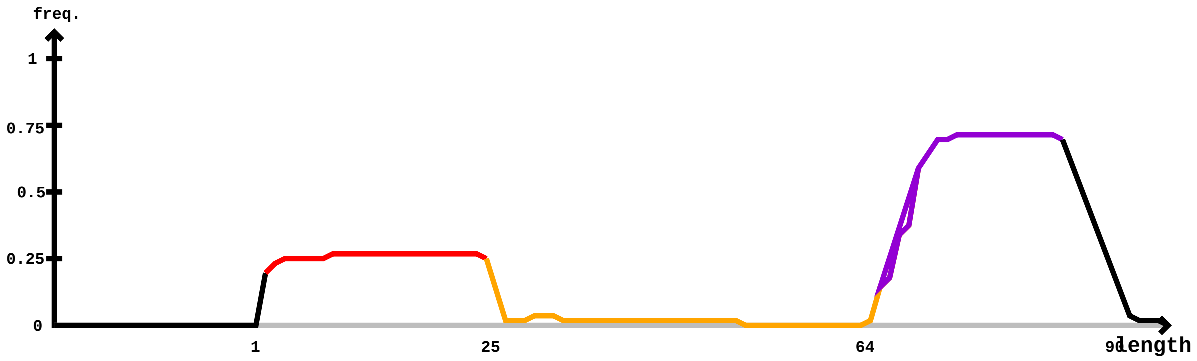


```
                                      Mature                                                                   Star
5'- auuuacccagucaagaagguagaaggucucggcuuuggaacgugcgcucguguugcuucuuccaugaacgaucucuagagccacgacgccaaagcuguagacgucugcuuuauc   -3'   obs
    auuuacccagucaagaagguagaaggucucggcuuuggaacgugcgcucguguugcuucuuccaugaacgaucucuagagccacgacgccaaagcuguagacgucugcuuuauc         exp
    ...............((((((((..(((((((((((((..((((.((((..((((.(((......))))))).....))))))))...))))))))).)))).))))))))...   reads   mm   sample
    ................................................cguguugcuucuuccaugaacU............................................   1   1   S03
    ...................................................................................cgacgccaaagcuguagacguU.........   1   1   S03
    ....................................................................................gacgccaaagcuguagacA...........   1   1   S03
    ....................................................................................gacUccaaagcuguagacgucugc......   3   1   S03
    ....................................................................................gacgccaaagcuguagacAucugc......   1   1   S03
    .....................................................................................acgccaaagcuguagacgucu........   1   0   S03
    ......................................................................................cgccaaagcuguagacAuc.........   1   1   S03
    ......................................................................................cgccaaagcuguagacgucugc......   1   0   S03
    ......................................................................................cUccaaagcuguagacgucugc......   4   1   S03
    .......................................................................................gccaaagcuguagacAucugc......   1   1   S03
    ........................................................................................ccaaagcuguagacguUu........   1   1   S03
    ........................................................................................ccaaagcuguagacAucugcu.....   3   1   S03
    ........................................................................................ccaaagcuguagacAucugcuuu...   1   1   S03
    .........................................................................................caaagcuguagacAucugcu.....   1   1   S03
    ..........................................................................................aaagcuguagacAucugcu.....   3   1   S03
    ............................................................................................agcuguagacgucugcuuuauc   1   0   S03

    ....................agaaggucucggcuuuggaacgu.......................................................................   1   0   S04
    ....................agaaggucucggcuuuggaacgug......................................................................   8   0   S04
    ....................Ngaaggucucggcuuuggaacgug......................................................................   1   1   S04
    ....................agaGggucucggcuuuggaacgug......................................................................   1   1   S04
    .....................gaaggucucggcuuuggaacgugc.....................................................................   2   0   S04
    ......................aaggucucggcuuuggaacgugU.....................................................................   1   1   S04
    ...........................cucggcuuuggaacgugcgcucgu...............................................................   1   0   S04
    ....................................................................................gacUccaaagcuguagacgucugc......   2   1   S04
    .....................................................................................acgccaaagcuguagacgucugcu.....   1   0   S04
    ......................................................................................cUccaaagcuguagacgucu........   1   1   S04
    ......................................................................................cgccaaagcuguagacAucugc......   2   1   S04
    .......................................................................................gccaaagcuguagacAucugc......   1   1   S04
    ........................................................................................ccaaagcuguagacCucugc......   1   1   S04
    ........................................................................................ccaaagcuguagacAucugcu.....   6   1   S04
    .........................................................................................caaagcuguagacAucugcu.....   2   1   S04
```

Provisional ID	:	chrA03_308665
Score total	:	0.6
Score for star read(s)	:	3.9
Score for read counts	:	-4.4
Score for mfe	:	1.1
Score for randfold	:	0
Score for cons. seed	:	
Total read count	:	3
Mature read count	:	1
Loop read count	:	0
Star read count	:	2

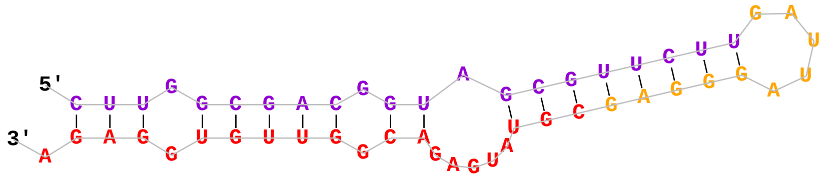

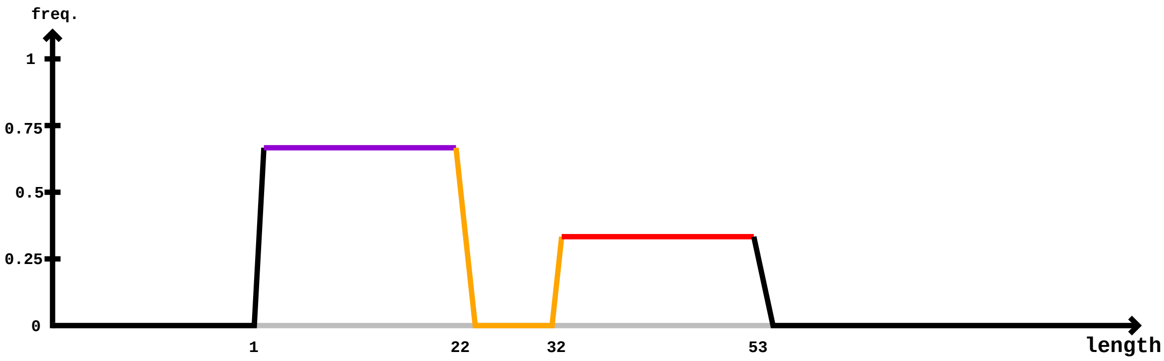

Star Mature

```
5'- acguaccucgaaggaggcgacuuggcgacgguagcguucuugauuagggagcguaugagacgguuguggagagagagguguugccugccgaugguuuugagacagagcuug   -3'   obs
    acguaccucgaaggaggcgacuuggcgacgguagcguucuugauuagggagcguaugagacgguuguggagagagagguguugccugccgaugguuuugagacagagcuug         exp
    .....((((....))))...(((.(((((.((.((((((((.....)))))))).....)).))))).)))....(((.....)))......(((((((...)))))))..   reads   mm   sample
    ....................cuuggcgacgguagcguucuu......................................................................   1       0    S03

    ....................cuuggcgacgguagcguucuu......................................................................   1       0    S04
    ...................................................cguaugagacgguuguggaga.......................................   1       0    S04
```

Provisional ID : chrA03_314063
Score total : 94.3
Score for star read(s) : 3.9
Score for read counts : 102.1
Score for mfe : -11.6
Score for randfold : 0
Score for cons. seed :
Total read count : 212
Mature read count : 211
Loop read count : 0
Star read count : 1

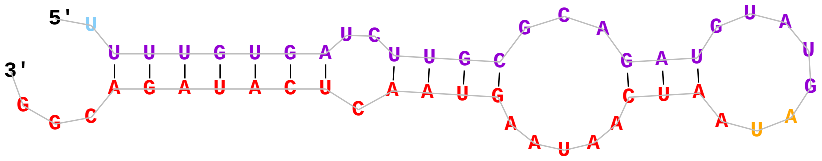

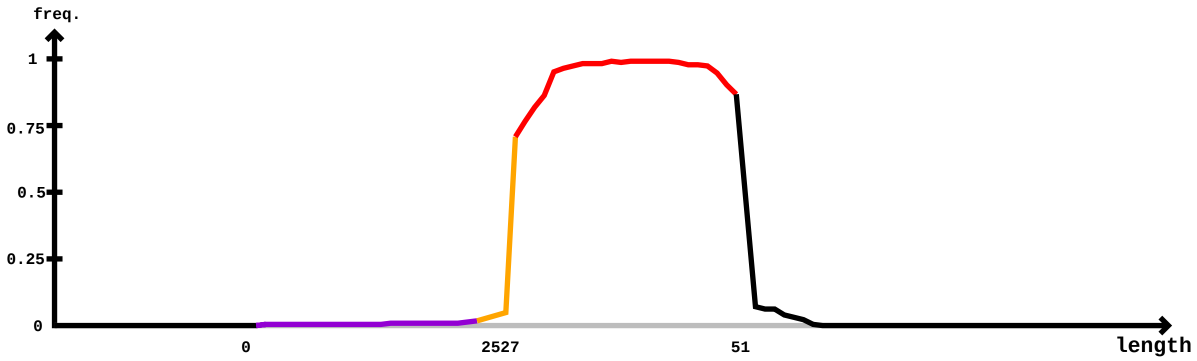

Star Mature

```
5'- guuauaauuuaaauucuaauuuugugaucuugcgcagauguaugauaaucaauaaguaacucauagacgggaacacuauuagugguaauuuuauggaggauucuucuucugu   -3'   obs
    guuauaauuuaaauucuaauuuugugaucuugcgcagauguaugauaaucaauaaguaacucauagacgggaacacuauuagugguaauuuuauggaggauucuucuucugu         exp
    ............((((...(((((((..((((...(((........))).....)))).)))))))..))))(((.....)))........((((((((......))))))))   reads   mm   sample
    .........................................augauaaucaauaaguaacuca.................................................   1       0    S03
    ..........................................ugauaaucaauaaguaacucauaga.............................................   1       0    S03
    ...........................................gauaaucaauaaguaacucauaga.............................................   2       0    S03
    ...........................................gauaaucaauaaguaacucauagac............................................   1       0    S03
    .............................................uaaucaauaaguaacucauagacg...........................................   1       0    S03
    ..............................................aaucaauaaguaacucauaga.............................................   1       0    S03
    ..............................................aaucaauaaguaacucauagac............................................   3       0    S03
    ..............................................aaucaauaaguaacucauagacg...........................................   1       0    S03
    ..............................................aaucaauGaguaacucauagacg...........................................   1       1    S03
    ..............................................aaCcaauaaguaacucauagacgg..........................................   1       1    S03
    ..............................................aaucaauaaguaacCcauagacgg..........................................   1       1    S03
    ..............................................aaucaauaaguaacucauaAacgg..........................................   2       1    S03
    ..............................................aaucGauaaguaacucauagacgg..........................................   2       1    S03
    ..............................................aaucaauaaguaacucauagacgg..........................................   59      0    S03
    ..............................................aaucaauaaguaacucauagacgC..........................................   1       1    S03
    ..............................................Gaucaauaaguaacucauagacgg..........................................   1       1    S03
    ..............................................aaucaauaaguaGcucauagacgg..........................................   1       1    S03
    ...............................................aucaauaaguaacucauagacg...........................................   2       0    S03
    ...............................................aucaauaaguaacucauagacgg..........................................   3       0    S03
    ...............................................aucaauaaguaGcucauagacgg..........................................   1       1    S03
    ...............................................aucaauaaguaacucauagacggg.........................................   3       0    S03
    ................................................ucaauaaguaacucauGgac............................................   1       1    S03
    ................................................ucaauaaguaacucauagacgg..........................................   6       0    S03
    .................................................caaCaaguaacucauagac............................................   1       1    S03
    .................................................caauaaguaacucauagac............................................   1       0    S03
    .................................................caauaaguaacucauagacg...........................................   1       0    S03
    .................................................caauaaguaacucauagacgg..........................................   2       0    S03
    .................................................caauaaguaacucauagacggga........................................   1       0    S03
    ..................................................aauaaguaacucauagac............................................   1       0    S03
    ..................................................aauaaguaGcucauagacgg..........................................   1       1    S03
    ..................................................aauaaguaacucauagacgg..........................................   10      0    S03
    ..................................................aauaaguaacucauagacgggaac......................................   1       0    S03
    ..................................................aauaaguaacucauagacgggaaA......................................   1       1    S03
```

```
                    Star                   Mature
guuauaauuuaaauucuaauuuugugaucuugcgcagauguaugauaaucaauaaguaacucauagacgggaacacuauuagugguaauuuuauggaggauucuucuucucugu
..................................................aauaaguaacCcauagacgggaac........................................   1   1   S03
..................................................aauaaguaacucauagacCggaac........................................   1   1   S03
...................................................auaaguaacucauagacgg............................................   1   0   S03
...................................................auaaguaacucauagacgggaaca.......................................   1   0   S03
....................................................uaaguaacucauagacgggaacac......................................   2   0   S03
.....................................................aaguaacucauagacgggaacacu.....................................   2   0   S03
..........................................................acucauagUcgggaacacua....................................   1   1   S03

....................uuugugaucuugcgcagauguaug......................................................................   1   0   S04
.................................gGagauguaugauaaucaauaagu.........................................................   1   1   S04
...........................................gauaaucaauaaguaacucauaga...............................................   1   0   S04
............................................auaaucaauaaguaacucauagac..............................................   1   0   S04
.............................................uaaucaauaaguaacucau..................................................   2   0   S04
..............................................aaucaauaaguaacucauag................................................   1   0   S04
..............................................aaucaGuaaguaacucauaga...............................................   1   1   S04
..............................................aaucaauaaguaacucauagac..............................................   1   0   S04
..............................................aaucaauaaguaacucauagacg.............................................   1   0   S04
..............................................aaucaauaaguaacucauagacgC............................................   1   1   S04
..............................................aaCcaauaaguaacucauagacgg............................................   1   1   S04
..............................................Naucaauaaguaacucauagacgg............................................   1   1   S04
..............................................aaucaauaaguaacucauagacCg............................................   1   1   S04
..............................................aaucaauaaguaacucGuagacgg............................................   1   1   S04
..............................................aaucaauaaguaacucauagacgg............................................   60  0   S04
..............................................aaucGauaaguaacucauagacgg............................................   1   1   S04
..............................................aaGcaauaaguaacucauagacgg............................................   1   1   S04
..............................................aaucaGuaaguaacucauagacgg............................................   3   1   S04
..............................................aaucaauaaguaacucauagacUg............................................   1   1   S04
...............................................aucaauaaguaacucauagacg.............................................   1   0   S04
...............................................aucaauaaguaacucauagacgg............................................   2   0   S04
...............................................aucaauaaguaacucauagacggg...........................................   1   0   S04
................................................ucaauaaguaacucauagacgg............................................   4   0   S04
................................................ucaauaaguaacucauagacggga..........................................   1   0   S04
.................................................caauaaguaacucauagacgg............................................   3   0   S04
.................................................caauaaguaacucauagacggU...........................................   1   1   S04
..................................................aauaaguaacucauagacgC............................................   1   1   S04
..................................................aauaaguaacucauagacgg............................................   2   0   S04
..................................................aauaaguaacucauagacgggaac........................................   1   0   S04
...................................................auaaguaacuUauagacgggaaca.......................................   1   1   S04
........................................................uaacucauagacgggaacacu.....................................   2   0   S04
```

Provisional ID	:	chrA03_random_2691257
Score total	:	91.3
Score for star read(s)	:	3.9
Score for read counts	:	85.8
Score for mfe	:	1.6
Score for randfold	:	0
Score for cons. seed	:	
Total read count	:	180
Mature read count	:	168
Loop read count	:	0
Star read count	:	12

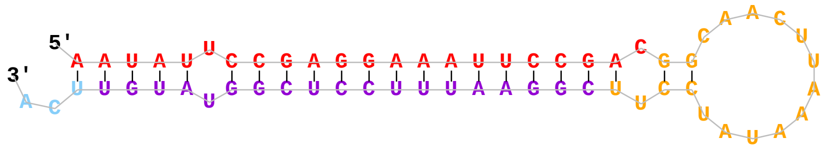

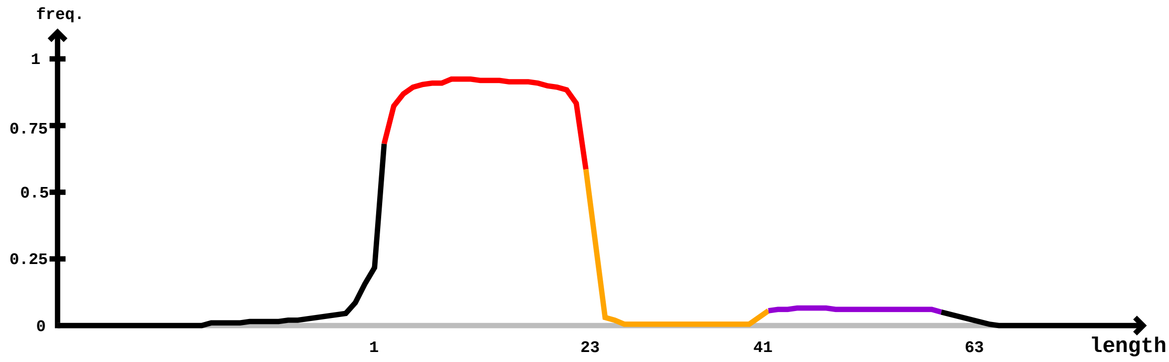

Mature **Star**

```
5'- uauuuggcggcugguuugcccgguuaaaugaaaauauuccgaggaaauuccgacggcaacuuaaauauccuucggaauuuccucgguauguucauuaauucgaggaaaaaa  -3'  obs
    uauuuggcggcugguuugcccgguuaaaugaaaauauuccgaggaaauuccgacggcaacuuaaauauccuucggaauuuccucgguauguucauuaauucgaggaaaaaa        exp
    ((((((((((......)))..)))))))..(((((.((((((((((((((((.((............)).)))))))))))))))).)))))...................   reads   mm   sample
    ..................cccgguuaaaugaaaauauuccUa.....................................................................   1       1    S03
    .........................aaaugaaaauauuccgaggaaauu..............................................................   1       0    S03
    .............................gaaaauauuccgaggaaauuccga..........................................................   1       0    S03
    .............................gaaaauauCccgaggaaauuccga..........................................................   1       1    S03
    ..............................aaaauauuccgaggaaauuccga..........................................................   2       0    S03
    ..............................aaaauauuccgaggaaauuccgac.........................................................   4       0    S03
    ...............................aaauauuccgaggaaauuccg...........................................................   1       0    S03
    ...............................aaauauuccgaggaaauuccga..........................................................   2       0    S03
    ...............................aaauauuccgaggaaauuccgac.........................................................   3       0    S03
    ................................aauauuccgaggaaauuc.............................................................   1       0    S03
    ................................aauauuccgaggaaauuccg...........................................................   3       0    S03
    ................................aauauuccgaggaaauuccgU..........................................................   1       1    S03
    ................................aauauuccgaggaaauuccga..........................................................   14      0    S03
    ................................aauauuccgaggaaauuccgac.........................................................   21      0    S03
    ................................aauauuccgCggaaauuccgac.........................................................   1       1    S03
    ................................aauauuccgGggaaauuccgac.........................................................   1       1    S03
    ................................aauauuccgaggaaauuccAac.........................................................   2       1    S03
    ................................aauauuccgaggaaauuccgacg........................................................   1       0    S03
    ................................aauauuccgaggaaauuccgacC........................................................   1       1    S03
    .................................auauuccgaggaaauuccg...........................................................   2       0    S03
    .................................auauuccgaggaaauuccga..........................................................   2       0    S03
    .................................auauuccgaggaaauuccgac.........................................................   7       0    S03
    .................................auauuccgaggaaauuccAac.........................................................   1       1    S03
    .................................auauuccgaggaaauuccgacC........................................................   1       1    S03
    .................................auauuccgaggaaauuccgacg........................................................   1       0    S03
    ..................................uauuccgaggaaauuccg...........................................................   1       0    S03
    ..................................uauuccgaggaaauuccgac.........................................................   3       0    S03
    ..................................uauuccgaggaaauuccgacg........................................................   1       0    S03
    ...................................auuccgaggaaauuccga..........................................................   2       0    S03
    ...................................auuccgaggaaauuccgac.........................................................   2       0    S03
    .......................................................gcCacuuaaauauccuucggaauu................................   1       1    S03
    .......................................................................ucggaauuuccucgguaugu....................   2       0    S03
    .......................................................................ucggaauuuccucgguauguu...................   1       0    S03
```

Mature Star

```
uauuuggcggcugguuugcccgguuaaaugaaaauauuccgaggaaauuccgacggcaacuuaaaauauccuucggaauuuccucgguauguucauuaaauucgaggaaaaaa
.........................................................................cggaauuuccucgguaugC.....................    1    1    S03
.........................................................................cggaauuuccucgguaugG.....................    1    1    S03
.........................................................................cggaauuuccucgguaugu.....................    1    0    S03

..............uuugcccgguuaaaugaaaaC..............................................................................    1    1    S04
..............uuugcccgguuaaaugaaaau..............................................................................    1    0    S04
......................guuGaaugaaaauauuccgagga....................................................................    1    1    S04
........................uaaaugaaaauauuccgaggaaau.................................................................    1    0    S04
..........................aaugaaaauauuccgaggaaCuu................................................................    1    1    S04
...........................augaaaauauuccgaggaaauucc..............................................................    1    0    S04
............................ugaaaauauuccgaggaaauuccg.............................................................    1    0    S04
.............................gaaaauauuccgaggaaauuccga............................................................    5    0    S04
.............................gaaaauauuccgaggaaauuccCa............................................................    1    1    S04
..............................aaaauauuccgGggaaauuccgac...........................................................    1    1    S04
..............................aaaauauuccgaggaaauuccgac...........................................................    7    0    S04
...............................aaauauuccgaggaaauuccga............................................................    3    0    S04
...............................aaauauuccgaggaaauuccgac...........................................................    3    0    S04
................................aauauuccgaggaaauucc..............................................................    1    0    S04
................................aauauuccgaggaaauuccga............................................................    7    0    S04
................................aauauuccgUggaaauuccga............................................................    1    1    S04
................................aauauuccgaggaaauuccgU............................................................    1    1    S04
................................aauauuccgaggaaauuccgac...........................................................    24   0    S04
................................aauauuccgaggaaauuccAac...........................................................    7    1    S04
................................aauauuccgaggaaauuccAacg..........................................................    1    1    S04
................................aauauuccgaggaaauuccgacg..........................................................    3    0    S04
................................aauauuccgaggaaauuccAacgg.........................................................    1    1    S04
.................................auauuccgaggaaauuccg.............................................................    2    0    S04
.................................auauuccgaggaaauuccga............................................................    5    0    S04
.................................auauuccgaggaaauuccgac...........................................................    5    0    S04
.................................auauuccgaggaaauuccgacg..........................................................    2    0    S04
..................................uauuccgaggaaauuccga............................................................    1    0    S04
..................................uauuccgaggaaauuccgac...........................................................    1    0    S04
..................................uauuccgaggaaauuccgacC..........................................................    1    1    S04
..................................uauuccgaggaaauuccgacgU.........................................................    1    1    S04
...................................auuccgaggaaauuccgac...........................................................    1    0    S04
...................................auuccgaggaaauuccgacg..........................................................    2    0    S04
....................................uuccgaggaaauuccgac...........................................................    1    0    S04
....................................uuccgaggaaauuccgacg..........................................................    1    0    S04
.....................................uccgaggaaauuccgacg..........................................................    1    0    S04
.......................................cgaggaaauuccgaAggc........................................................    1    1    S04
.......................................cgaggaaauuUcgacggc........................................................    1    1    S04
.......................................cgaggaaauuccgacggc........................................................    1    0    S04
........................................................................ucggaaAuuccucgguaug......................    1    1    S04
........................................................................ucggaauuuccucgguauguu....................    1    0    S04
.........................................................................cggaauuuccucgguaug......................    1    0    S04
.........................................................................cggaauuuccucgguaugu.....................    1    0    S04
..........................................................................ggaauuuccucgguaugu.....................    1    0    S04
............................................................................aauuuccucgguauguucauu.................    1    0    S04
```

Provisional ID	:	chrA03_random_2692801
Score total	:	0
Score for star read(s)	:	-1.3
Score for read counts	:	-0.3
Score for mfe	:	1.6
Score for randfold	:	0
Score for cons. seed	:	
Total read count	:	11
Mature read count	:	11
Loop read count	:	0
Star read count	:	0

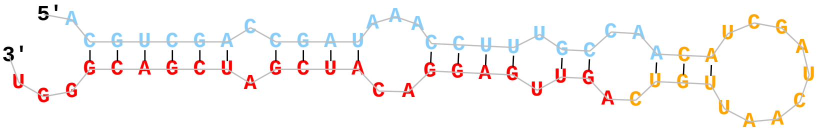

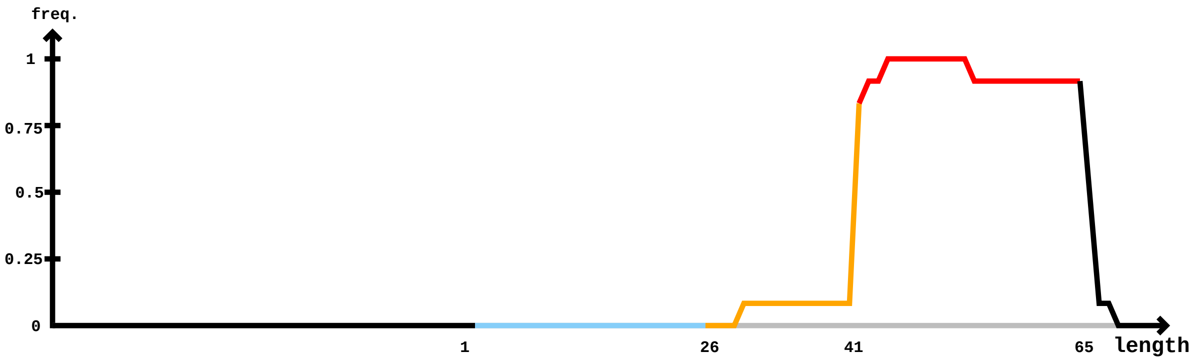

Star Mature

```
5'- guaugcagacaacgagagagugucgaucgaugggcuaguaagacgucgaccgauaaaccuuugccaacaucgaucaauugucaguugaggacaucgaucgacggguucuagcca   -3'    exp
    .......((((.(....)..))))........((((((.((..((((((.((((...((((.((..(((.........)))..)).)))).))))..)))).))))))..)))))))).    reads    mm    sample
    ......................................................................cgaucaauugucaguugaggacau....................    1    0    S03
    ..................................................................................aguugaggacaucgaucgacgggC........    3    1    S03
    ..................................................................................aguugaggacaucgaucgacgggG........    1    1    S03
    .....................................................................................ugaggacaucgaucgacggguucA.....    1    1    S03

    ..................................................................................aguugaggacaucgaucgacgggA........    1    1    S04
    ..................................................................................aguugaggacaucgaucgacgUgu........    2    1    S04
    ..................................................................................aguugaggacaucgaucgacgAgu........    2    1    S04
    ...................................................................................guugaggacaucgaucgacggguC.......    1    1    S04
```

Provisional ID	:	chrA03_random_2694764
Score total	:	0.8
Score for star read(s)	:	3.9
Score for read counts	:	-3.9
Score for mfe	:	0.8
Score for randfold	:	0
Score for cons. seed	:	
Total read count	:	4
Mature read count	:	3
Loop read count	:	0
Star read count	:	1

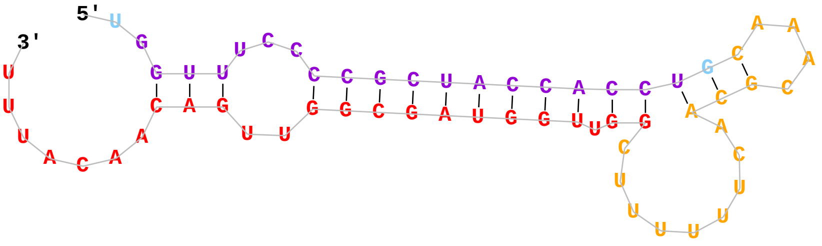

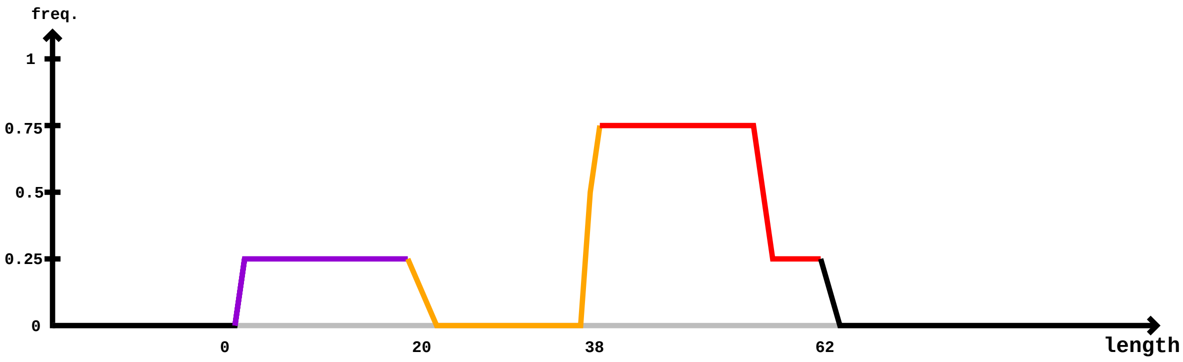

Star Mature

```
5'- acuaauaguggucugacugguuuccccgcuaccaccugcaaacgcaacuuuuuucgguugguagcgguugacaacauuucaaaucacuauaaaucgcuucaaaccaaucugaa   -3'   obs
    acuaauaguggucugacugguuuccccgcuaccaccugcaaacgcaacuuuuuucgguugguagcgguugacaacauuucaaaucacuauaaaucgcuucaaaccaaucugaa         exp
    ....((((((((.(((...(((((.((((((((((((((....))).........)).)))))))))..)).)))...))).))))))))......................  reads   mm   sample
    ..................gguuuccccgcuaccacUu............................................................................  1       1    S03
    ......................................................cgguugguagcgguugac.........................................  1       0    S03
    ......................................................cgguugguagcgguugaca........................................  1       0    S03
    .......................................................gguugguagcgguugacaacauuC..................................  1       1    S03
```

Provisional ID	:	chrA03_random_2696397
Score total	:	0.5
Score for star read(s)	:	-1.3
Score for read counts	:	0
Score for mfe	:	1.8
Score for randfold	:	0
Score for cons. seed	:	
Total read count	:	33
Mature read count	:	33
Loop read count	:	0
Star read count	:	0

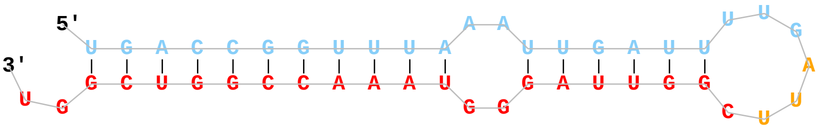

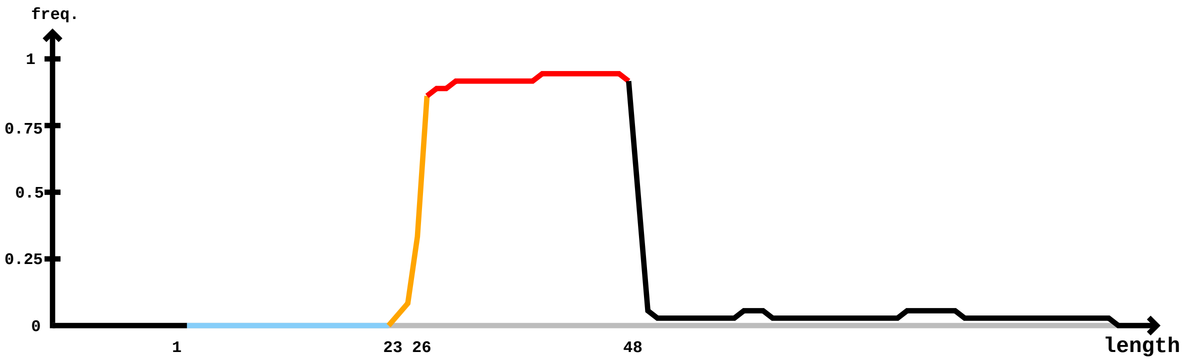

Star Mature

```
5'- aaccgguuggucugaccgguuuaaauugauuuugauucgguuaggguaaaccggucgguuaagucaaauugauuucugaucaaggguugaccagauugaucuuugaccagcga   -3'   exp
    .....(((((((.((((((((((..((((((.......))))))..))))))))))....(((((.....)))))..((((((.((....))...))))))...)))))))..   reads   mm   sample
    ...................................uucgguuaggguaaaccggucggu......................................................   2       0    S03
    ..................................Aucgguuaggguaaaccggucggu......................................................   1       1    S03
    ....................................ucgguuaggguaaaccggucgguu.....................................................   7       0    S03
    .....................................cgguuaggguaaaccggucggu......................................................   13      0    S03
    .....................................cgguuaggguaaaccggucgguu.....................................................   1       0    S03
    ......................................gguuaggguaaaccggucgguu.....................................................   1       0    S03
    ........................................uuaggguaaaccggucgguu.....................................................   1       0    S03
    .................................................accggucgguuaagucaaauugGu........................................   1       1    S03

    ....................................ucgguuaggguaaaccggucgguu.....................................................   2       0    S04
    .....................................cgguuaggguaaaccggucgg.......................................................   1       0    S04
    .....................................cgguuaggguaaaccggucggu......................................................   3       0    S04
    .....................................cgguuaggguaaaccggucgguua....................................................   1       0    S04
    ......................................................................gauuucugaucaaggguugacca....................   1       0    S04
    .......................................................................................ugaccagauugaucuuugacca....   1       0    S04
```

Provisional ID	:	chrA03_random_2696441
Score total	:	0.5
Score for star read(s)	:	-1.3
Score for read counts	:	0
Score for mfe	:	1.8
Score for randfold	:	0
Score for cons. seed	:	
Total read count	:	33
Mature read count	:	33
Loop read count	:	0
Star read count	:	0

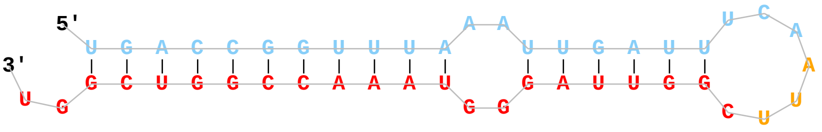

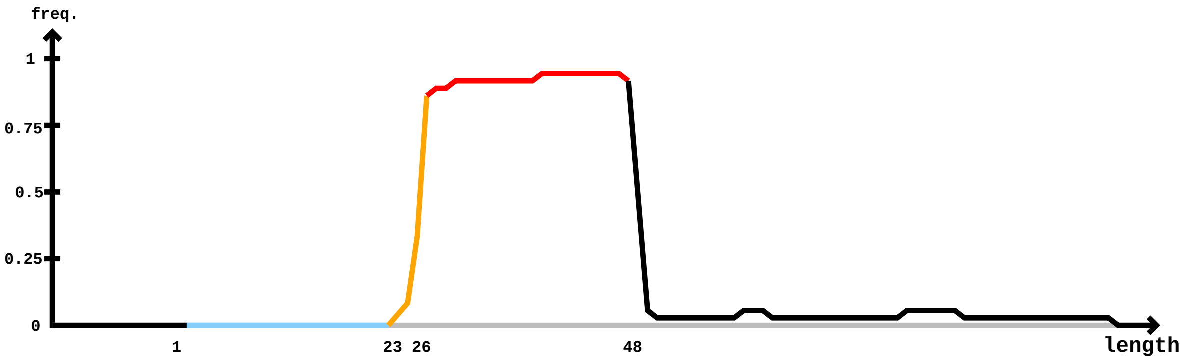

Star Mature

```
5'- aaccaguuggucugaccgguuuaaauugauuucaauucgguuaggguaaaccggucgguuaagucaaauugauuucugaucaaggguugaccagauugaucuuugaccagcga   -3'   exp
    .....(((((((.((((((((((..((((((.......))))))..))))))))))....(((((.....)))))..((((((.((....))...))))))...)))))))..     reads   mm   sample
    ...................................uucgguuaggguaaaccggucggu......................................................     2       0    S03
    ...................................Aucgguuaggguaaaccggucggu......................................................     1       1    S03
    ....................................ucgguuaggguaaaccggucgguu.....................................................     7       0    S03
    .....................................cgguuaggguaaaccggucggu......................................................     13      0    S03
    .....................................cgguuaggguaaaccggucgguu.....................................................     1       0    S03
    ......................................gguuaggguaaaccggucgguu.....................................................     1       0    S03
    ........................................uuaggguaaaccggucgguu.....................................................     1       0    S03
    .................................................accggucgguuaagucaaauugGu........................................     1       1    S03

    ....................................ucgguuaggguaaaccggucgguu.....................................................     2       0    S04
    .....................................cgguuaggguaaaccggucgg.......................................................     1       0    S04
    .....................................cgguuaggguaaaccggucggu......................................................     3       0    S04
    .....................................cgguuaggguaaaccggucgguua....................................................     1       0    S04
    ......................................................................gauuucugaucaaggguugacca....................     1       0    S04
    .......................................................................................ugaccagauugaucuuugacca....     1       0    S04
```

Provisional ID	:	chrA03_random_2698515
Score total	:	1.9
Score for star read(s)	:	3.9
Score for read counts	:	-3.9
Score for mfe	:	2
Score for randfold	:	0
Score for cons. seed	:	
Total read count	:	4
Mature read count	:	3
Loop read count	:	0
Star read count	:	1

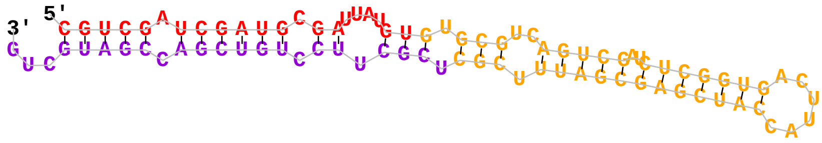

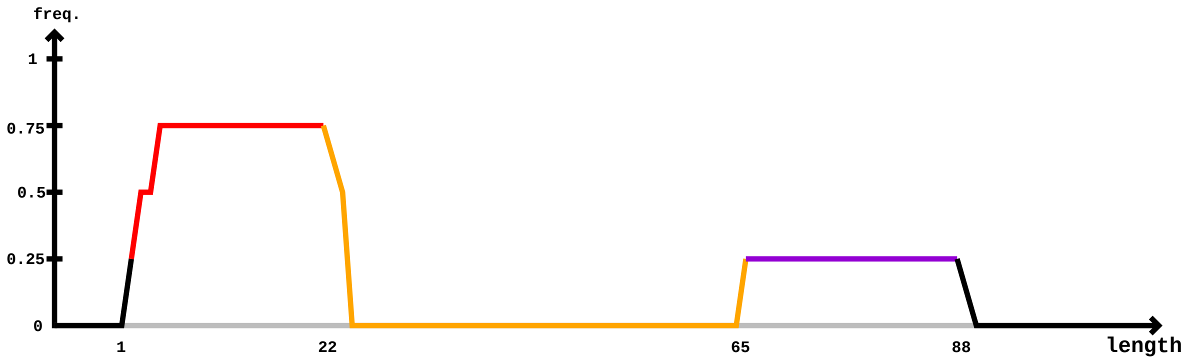

Mature   Star

```
5'- acucggcgucgaucgaugcgauuaugugugcgucagucgaucucggugacuuaccaucgagcgauuucgcucgcuuccugucgaccgaugcugauaucuggugcugagcugcg   -3'    obs
    acucggcgucgaucgaugcgauuaugugugcgucagucgaucucggugacuuaccaucgagcgauuucgcucgcuuccugucgaccgaugcugauaucuggugcugagcugcg          exp
    ..((((((((.((((((.((....(((.(((..(((((..(((((((......)))))))))))).))).))).)).)))))).)))))))))........((......)).   reads      mm        sample
    ......cgucgaucgaugcgauuaugu......................................................................................   1          0         S03
    .......gucgaucgaugcgauuaugugu....................................................................................   1          0         S03
    .........cgaucgaugcgauuaugugu....................................................................................   1          0         S03

    ......................................................................ucgcuuccugucgaccgaugcug....................   1          0         S04
```

Provisional ID	:	chrA03_random_2706162
Score total	:	0
Score for star read(s)	:	-1.3
Score for read counts	:	0
Score for mfe	:	1.2
Score for randfold	:	0
Score for cons. seed	:	
Total read count	:	20
Mature read count	:	20
Loop read count	:	0
Star read count	:	0

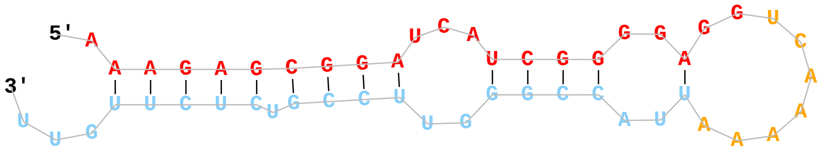

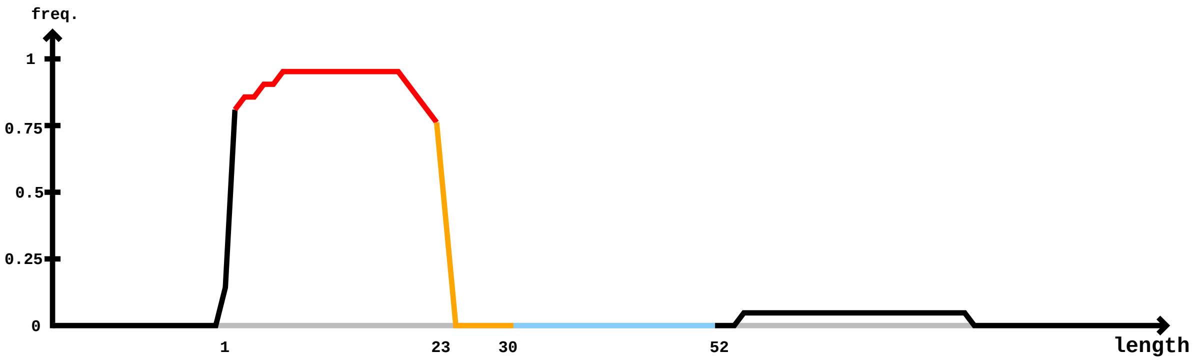

Mature Star

```
5'- uccucgagccaaaucuuaaagagcggaucaucggggaggucaaaaauuaccggguuccgucucuuguuauaacuguggcucggcuuuugcggcaagugguaagugcagauuggc   -3'    exp
    .((.(((((((.......(((((((((...((((..(.........)..))))..)))).)))))..........)))))))((.(((((.(....).))))).)).....)).    reads       mm          sample
    ................Aaaagagcggaucaucggggaggu..........................................................................    1           1           S03
    ................uaaagagcggaucaucggggaggu..........................................................................    1           0           S03
    .................aaagagcggaucaucggg...............................................................................    1           0           S03
    .................aaagagcggaucaucgggg..............................................................................    1           0           S03
    .................aaagagcggaucaucgggga.............................................................................    1           0           S03
    .................aaagagcggaucaCcggggag............................................................................    1           1           S03
    .................aaagagcggaucaucggggagg...........................................................................    7           0           S03
    .................aaagagcggaucaucggggagU...........................................................................    2           1           S03
    ..................aagagcggaucaucggggaggu..........................................................................    1           0           S03
    ....................gagcggaucaucggggaggA..........................................................................    1           1           S03

    ................uaaagagcggaucaucggggaggA..........................................................................    1           1           S04
    .................aaagagcggaucaucggggagg...........................................................................    1           0           S04
    ......................gcggaucaucggggaggu..........................................................................    1           0           S04
    ......................................................................aacuguggcucggcuuuugcggca....................    1           0           S04
```

Provisional ID	:	chrA03_random_2706548
Score total	:	0.2
Score for star read(s)	:	3.9
Score for read counts	:	-2.8
Score for mfe	:	-0.7
Score for randfold	:	0
Score for cons. seed	:	
Total read count	:	6
Mature read count	:	5
Loop read count	:	0
Star read count	:	1

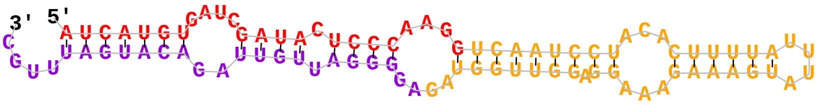

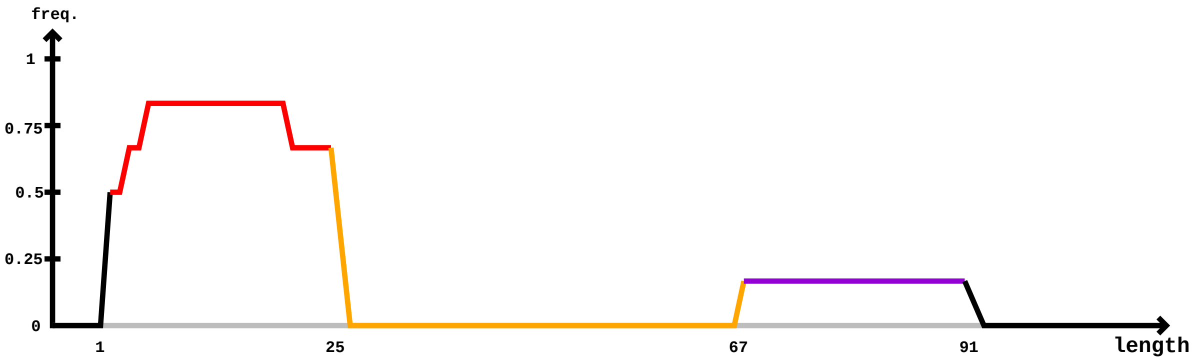

Mature Star

```
5'- uugcaucaugugaucgauacucccaaggucaauccuacacuuuuauuuaugaaagaaaggagguugguagaggggauuguuagacaugauuugcaaaguuguugacuaggugaa   -3'    obs
    uugcaucaugugaucgauacucccaaggucaauccuacacuuuuauuuaugaaagaaaggagguugguagaggggauuguuagacaugauuugcaaaguuguugacuaggugaa          exp
    ((((((((((....((((.((((...((((((((...((((((....))))))...)).)))))))....)))).))))..))))))..)))))....................   reads   mm   sample
    ....aucaugugaucgauacucc...........................................................................................   1       0    S03
    ....aucaugugaucgauacucccaagg......................................................................................   1       0    S03
    ......caugugaucgauacucccaaggu.....................................................................................   1       0    S03
    ........ugugaucgaCacucccaaggu.....................................................................................   1       1    S03

    ....aucaugugaucgauacucccaagg......................................................................................   1       0    S04
    ......................................................................aggggauuguuagacaugauuugc....................   1       0    S04
```

Provisional ID	:	chrA04_355424
Score total	:	8.4
Score for star read(s)	:	3.9
Score for read counts	:	3.2
Score for mfe	:	1.3
Score for randfold	:	0
Score for cons. seed	:	
Total read count	:	18
Mature read count	:	10
Loop read count	:	0
Star read count	:	8

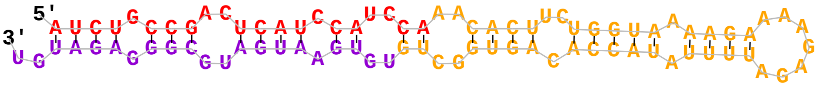

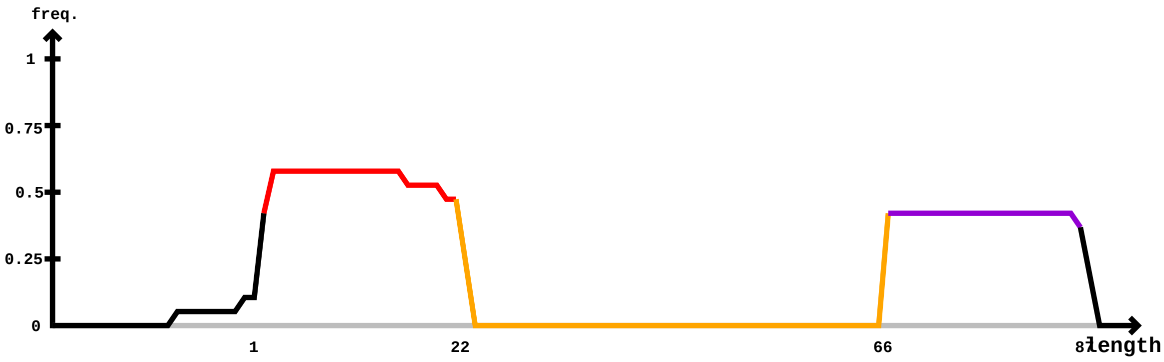

Mature Star

```
5'- aacaagaagaggguagaaacaucugccgacucauccauccaaacacuucugguaaaagaaaagagauuuuauaccacaguggcugugugaaugaugcgggagauguuuuca   -3'    obs
    aacaagaagaggguagaaacaucugccgacucauccauccaaacacuucugguaaaagaaaagagauuuuauaccacaguggcugugugaaugaugcgggagauguuuuca          exp
    ...............((((((((.(((..((((.((..((..((((..(((((.((((.......)))).))))).))))..))..)).))))..))).))))))))..    reads     mm     sample
    ..................acaucugccgacucauccauc........................................................................    1         0      S03
    ....................aucugccgacucauccauccG......................................................................    1         1      S03
    ....................aucugccgacucauccaucca......................................................................    1         0      S03
    ....................aucugccgacucauccauccC......................................................................    1         1      S03
    .....................................................................................ugugaaugaugcgggagaugu.....    2         0      S03

    ...........gguagaaacaucugcAgacucauc............................................................................    1         1      S04
    ....................aucugccgacucauccaucca......................................................................    3         0      S04
    .....................ucugccgacucauccaucca......................................................................    1         0      S04
    .....................ucugccgacucauccauccaC.....................................................................    1         1      S04
    .....................ucugccgacucauccauccaU.....................................................................    1         1      S04
    .....................................................................................ugugaaugaugcgggagauA......    1         1      S04
    .....................................................................................ugugaaugaugcgggagaugC.....    1         1      S04
    .....................................................................................ugugaaugaugcgggagaugu.....    2         0      S04
    .....................................................................................ugCgaaugaugcgggagaugu.....    1         1      S04
    .....................................................................................ugugaaugaugcgggagauguu....    1         0      S04
```

Provisional ID	:	chrA04_358292
Score total	:	0.6
Score for star read(s)	:	3.9
Score for read counts	:	-4.9
Score for mfe	:	1.7
Score for randfold	:	0
Score for cons. seed	:	
Total read count	:	2
Mature read count	:	1
Loop read count	:	0
Star read count	:	1

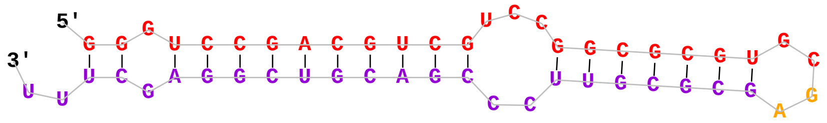

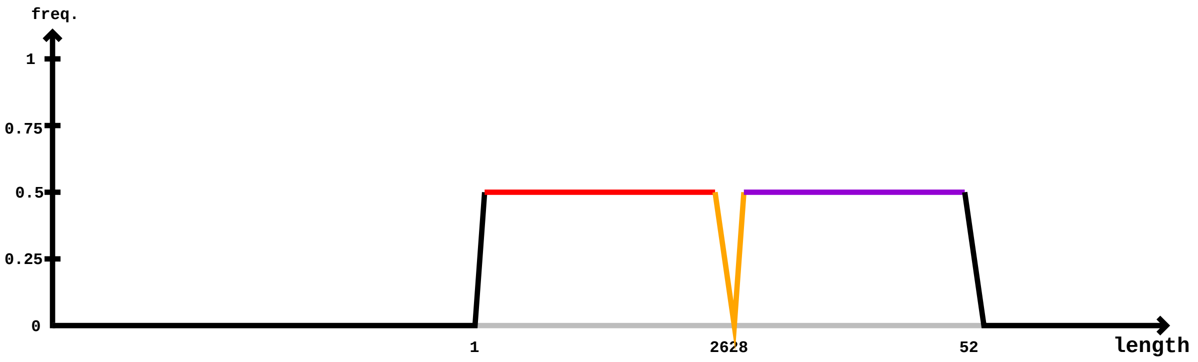

Mature **Star**

```
5'- caacggaccuuucucucucggccgcaaacaaagaucuaguuuuggguccgacgucguccggcgcgugcgagcgcguucccgacgucggagcuuucaucuuccucuuuuagucuu  -3'   obs
    caacggaccuuucucucucggccgcaaacaaagaucuaguuuuggguccgacgucguccggcgcgugcgagcgcguucccgacgucggagcuuucaucuuccucuuuuagucuu         exp
    ...(((.((..........))))).....(((((...((....((.((((((((((...(((((((....)))))))..)))))))))).)).....))...))))).......   reads   mm   sample
    ...........................................ggguccgacgucguccggcgcgugG.............................................   1       1    S04
    ......................................................................gcgcguucccgacgucggagcuuu....................   1       0    S04
```

Provisional ID	:	chrA04_366478
Score total	:	1.1
Score for star read(s)	:	-1.3
Score for read counts	:	0
Score for mfe	:	2.4
Score for randfold	:	0
Score for cons. seed	:	
Total read count	:	213
Mature read count	:	209
Loop read count	:	0
Star read count	:	4

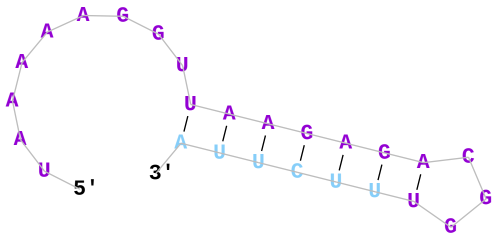

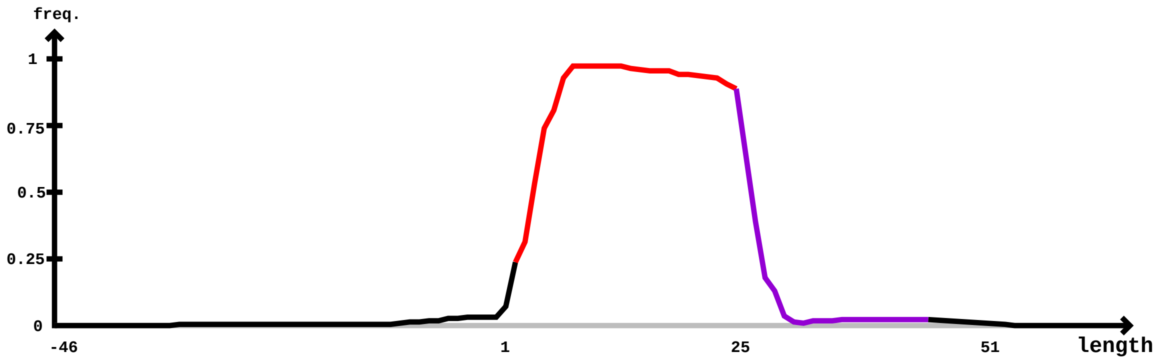

Mature Star

```
5'- acucucagaacaugaccggugaggguggaguuauuaacggaauauaagaaaccgucucuuaacuuuuaacuaaaaagguuaagagacgguuucuuauauuccguuaaaaa  -3'   obs
    acucucagaacaugaccggugaggguggaguuauuaacggaauauaagaaaccgucucuuaacuuuuaacuaaaaagguuaagagacgguuucuuauauuccguuaaaaa        exp
    (((((((...(......).))))))).......((((((((((((((((((((((((((((((((((......))))))))))))))))))))))))))))))))))...   reads     mm     sample
    .....................................cggaauauaagaaaccgucucuu..................................................   1         0      S03
    .........................................auauaagaaaccgucucuGaacuu.............................................   1         1      S03
    .............................................aagaaaccgucucuuaacuuu............................................   1         0      S03
    .............................................aagaaaccgucucuuaacuuuua..........................................   2         0      S03
    ..............................................agaaaccgucucuuaacuuuua..........................................   2         0      S03
    ..............................................agaaGccgucucuuaacuuuuaac........................................   1         1      S03
    ..............................................agaaaccgucucuuaacuuuuaac........................................   14        0      S03
    ..............................................agaaaccgucucuuaacuuuuaacu.......................................   1         0      S03
    ...............................................gaaaccgucucuGaacuuuuaacu.......................................   1         1      S03
    ...............................................gaaaccgucucuuaacuuuuaacu.......................................   4         0      S03
    ...............................................gaaaccgucucuuaacuuuuaacG.......................................   1         1      S03
    ................................................aaaccgucucuuaacuuuu...........................................   1         0      S03
    ................................................aaaccgucucuuaacuuuuaac........................................   3         0      S03
    ................................................aaaccgucucuuaacuuuuaacu.......................................   13        0      S03
    ................................................aaaccgucucuGaacuuuuaacu.......................................   1         1      S03
    ................................................aaaccgucucuuaacuuuuaacua......................................   9         0      S03
    .................................................aaccgucucuuaacuuuuaac........................................   6         0      S03
    .................................................aaccgucucuuaacuuuuaacu.......................................   3         0      S03
    .................................................aaccgucucuuaacuuuuaacua......................................   8         0      S03
    .................................................aaccgucucuuaacuuuuaacuG......................................   1         1      S03
    .................................................aaccgucucuuaacuuuuaGcua......................................   1         1      S03
    .................................................aaccgucucuAaacuuuuaacua......................................   1         1      S03
    .................................................aaccgucucuuUacuuuuaacuaa.....................................   1         1      S03
    .................................................aaccgucucuuaacuuuuaacuaa.....................................   1         0      S03
    .................................................aaccgucucuAaacuuuuaacuaa.....................................   1         1      S03
    ..................................................accgucucuuaacuuuuaacuaaa....................................   4         0      S03
    ...................................................ccgucucuuaacuuuuaac........................................   2         0      S03
    ...................................................ccgucucuuaacuuuuaacu.......................................   4         0      S03
    ...................................................ccgucucuuaacuuuuaacua......................................   1         0      S03
    ...................................................ccgucucuuaacuuuuaacuaaa....................................   5         0      S03
    ...................................................ccgucucuuaacuuuuaacuaaaa...................................   3         0      S03
    ...................................................ccgucucuAaacuuuuaacuaaaa...................................   1         1      S03
    ....................................................cgucucuuaacuuuuaacua......................................   1         0      S03
```

```
                                                  Mature          Star
acucucagaacaugaccggugaggguggaguuauuaacggaauauaagaaaccgucucuuaacuuuuaacuaaaaagguuaagagacgguuucuuauauuccguuaaaaa
....................................................cgucucuuaacuuuuaacuaa.....................................    1    0    S03
....................................................cgucGcuuaacuuuuaacuaa.....................................    1    1    S03
....................................................cgucucuuaacuuuuaacuaaa....................................    2    0    S03
....................................................cgucucuuaacuuuuaacuaaaaa..................................    1    0    S03
......................................................................uaaaaagguuaagagacggu....................    1    0    S03
................................................................................aagagacgguuucuuaua............    1    0    S03

...........augaccggugaggguggagGuauu...........................................................................    1    1    S04
..................................uaacggaauauaagaaaccAucuc....................................................    1    1    S04
...................................aacggaauauaagaaaccAucuc....................................................    1    1    S04
...................................aacggaauauaagaaaccAucucu...................................................    1    1    S04
.......................................gaauGuaagaaaccgucucuuaac...............................................    2    1    S04
.............................................aagaaaccgucucuuaac...............................................    1    0    S04
.............................................aagaaaccgucucuuaacuuuua..........................................    2    0    S04
.............................................aagaaaccgucucuGaacuuuuaa.........................................    1    1    S04
.............................................aagaaaccgucucuuaacuuuuaa.........................................    1    0    S04
.............................................aagaaaccgucucuuaacuuuuaG.........................................    1    1    S04
..............................................agaaaccgucucuuaacuuuuaGc........................................    2    1    S04
..............................................agaGaccgucucuuaacuuuuaac........................................    1    1    S04
..............................................agaaaccgucucuuaacuuuuaac........................................    13   0    S04
..............................................agaaGccgucucuuaacuuuuaac........................................    1    1    S04
..............................................agaaaccgucucuuaacuuuAaac........................................    1    1    S04
..............................................agaaaccgucucuuaacuuuuaacu.......................................    1    0    S04
...............................................gaaaccgucucuuaacuuuuaac........................................    1    0    S04
...............................................gaaaccgucucuuaacuuuuaacG.......................................    2    1    S04
...............................................gaaaccgucucuuaacuuuuaacu.......................................    8    0    S04
................................................aaaccgucucuuaacuuuuaacu.......................................    7    0    S04
................................................aaaccgucucuGaacuuuuaacua......................................    1    1    S04
................................................aaaccgucucuuaacuuuuaacua......................................    12   0    S04
................................................aaaccgucucuuaacuuuuaacuG......................................    2    1    S04
.................................................aaccgucucuAaacuuuuaa.........................................    1    1    S04
.................................................aaccgucucuuaacuuuuaac........................................    8    0    S04
.................................................aaccgucucuuaacuuuuaacu.......................................    5    0    S04
.................................................aaccgucucuAaacuuuuaacu.......................................    1    1    S04
.................................................aaccgucucuGaacuuuuaacu.......................................    1    1    S04
.................................................aaccgucucuuaacuuuuaacuG......................................    3    1    S04
.................................................aaccgucucuuaacuuuuaacua......................................    1    0    S04
.................................................aaccgucucuuaacuuuuaacuaa.....................................    3    0    S04
..................................................accgucucuuaacuuuuaacu.......................................    2    0    S04
..................................................accgucucuGaacuuuuaacu.......................................    1    1    S04
..................................................accgucucuuaacuuuuaacua......................................    2    0    S04
..................................................accgucucuuaacuuuuaacuaa.....................................    1    0    S04
..................................................accgucucuuaacuuuuaacuaaa....................................    3    0    S04
..................................................accgGcucuuaacuuuuaacuaaa....................................    1    1    S04
..................................................Nccgucucuuaacuuuuaacuaaa....................................    1    1    S04
...................................................ccgucucuuaacuuuuaacu.......................................    3    0    S04
...................................................ccgucucuuaacuuuuaacua......................................    4    0    S04
...................................................ccgucucuuaacuuuuaacuaaa....................................    3    0    S04
...................................................ccgucucuuaacuuuuaacuaaaa...................................    1    0    S04
....................................................cgucucuuaacuuuuaacuaa.....................................    2    0    S04
....................................................cgucucuuaacuuuuaacuaaa....................................    2    0    S04
....................................................................acuaaaaaAguuaagagacggu....................    1    1    S04
.............................................................................guuaagagacgguuucuG...............    1    1    S04
.............................................................................guuaagagacgguuucuGa..............    1    1    S04
```

Provisional ID	:	chrA04_372393
Score total	:	0.2
Score for star read(s)	:	-1.3
Score for read counts	:	-1.3
Score for mfe	:	2.9
Score for randfold	:	0
Score for cons. seed	:	
Total read count	:	9
Mature read count	:	6
Loop read count	:	3
Star read count	:	0

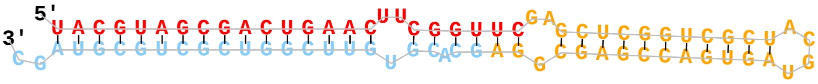

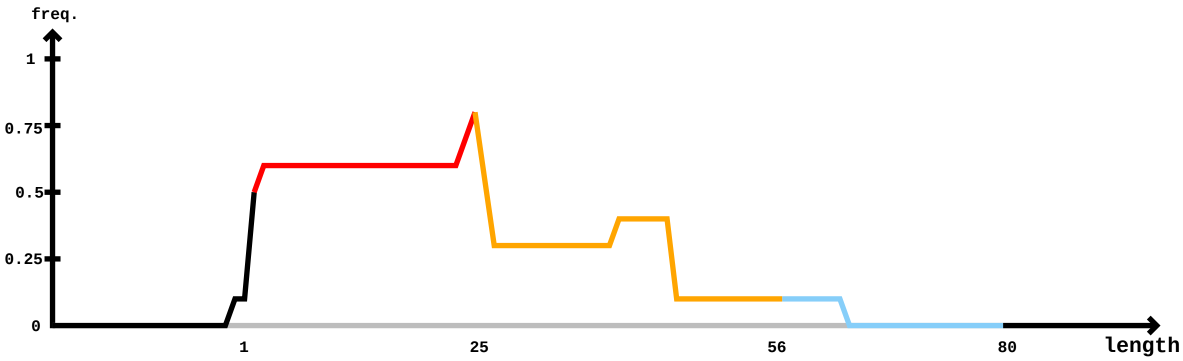

Mature   Star

```
5'- cgaccgagcgcucgaucgcuacguagcgacugaacuucgguucgagcucggucgcuacguagugaccgagcggagcacguguucggucgcugcguagcgauccuuuucgagcu   -3'    exp
    .........((((((((((((((((((((((((((..((((((..(((((((((((....))))))))))).)))).)).))))))))))))))))))))......)))))).    reads    mm    sample
    ...................uacguagcgacugaacuucgguuc......................................................................    1        0     S03
    ....................acguagcgacugaacuucgguuc......................................................................    1        0     S03
    .........................................ucgagcucggucgcuacguagu..................................................    1        0     S03
    .........................................uUgagcucggucgcuacguagu..................................................    1        1     S03
    ..........................................Ugagcucggucgcuacguagu..................................................    1        1     S03

    .................gcuacguagcgacugaacuucggC........................................................................    1        1     S04
    ...................uacguagcgacugaacuuUgguuc......................................................................    1        1     S04
    ...................uacguagcgacugaacuucgguuc......................................................................    2        0     S04
    .........................................................Aguagugaccgagcggagcacgug................................    1        1     S04
```

Provisional ID	:	chrA05_400527
Score total	:	0
Score for star read(s)	:	-1.3
Score for read counts	:	0
Score for mfe	:	1.2
Score for randfold	:	0
Score for cons. seed	:	
Total read count	:	110
Mature read count	:	110
Loop read count	:	0
Star read count	:	0

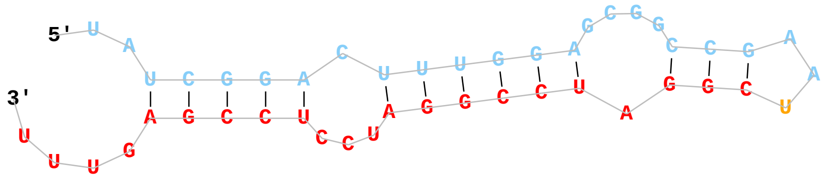

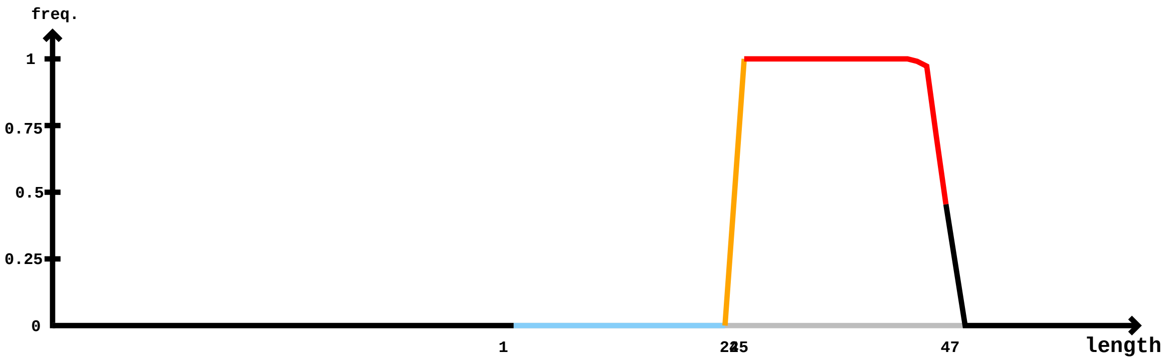

Star Mature

```
5'- ggccucgaacucgagcucccaccuucuucaccggcaaacaacggguuaucggacuuuggagcggccgaaucggauccggauccuccgaguuucucgauguccauguaggug  -3'    exp
    ((.((((....))))...))........((((.(((.(((.((((...(((((.((((((....(((...))).))))))...)))))....)))).))).)))...))).))))  reads      mm         sample
    .....................................................................ucggauccggauccuUcgag......................     1          1          S03
    .....................................................................ucggauccggauccuUcgagu.....................     9          1          S03
    .....................................................................ucggauccggauccuUcgaguu....................     4          1          S03
    .....................................................................ucggauccggauccuUcgaguuu...................     6          1          S03
    ......................................................................cggauccggauccuUcgagu.....................     13         1          S03
    ......................................................................cggauccggauccuUcgaguu....................     10         1          S03
    ......................................................................cggauccggauccuUcgaguuu...................     20         1          S03
    ......................................................................cggauccggauccuUcgaguuuc..................     2          1          S03

    .....................................................................ucggauccggauccuUcgagu.....................     5          1          S04
    .....................................................................ucggauccggauccuUcgaguu....................     7          1          S04
    .....................................................................ucggauccggauccuUcgaguuu...................     7          1          S04
    ......................................................................cggauccggauccuUcga........................     1          1          S04
    ......................................................................cggauccggauccuUcgag.......................     1          1          S04
    ......................................................................cggauccggauccuUcgagu......................     2          1          S04
    ......................................................................cggauccggauccuUcgaguu.....................     6          1          S04
    ......................................................................cggauccggauccuccgaguu.....................     1          0          S04
    ......................................................................cggauccggauccuUcgaguuu....................     15         1          S04
```

Provisional ID	:	chrA05_400558
Score total	:	4.8
Score for star read(s)	:	3.9
Score for read counts	:	-0.3
Score for mfe	:	1.3
Score for randfold	:	0
Score for cons. seed	:	
Total read count	:	11
Mature read count	:	8
Loop read count	:	0
Star read count	:	3

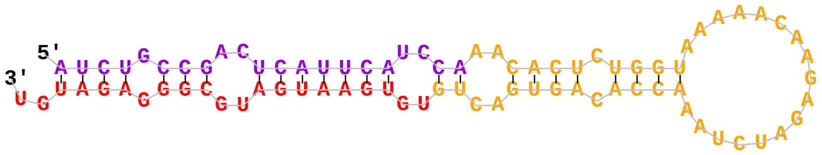

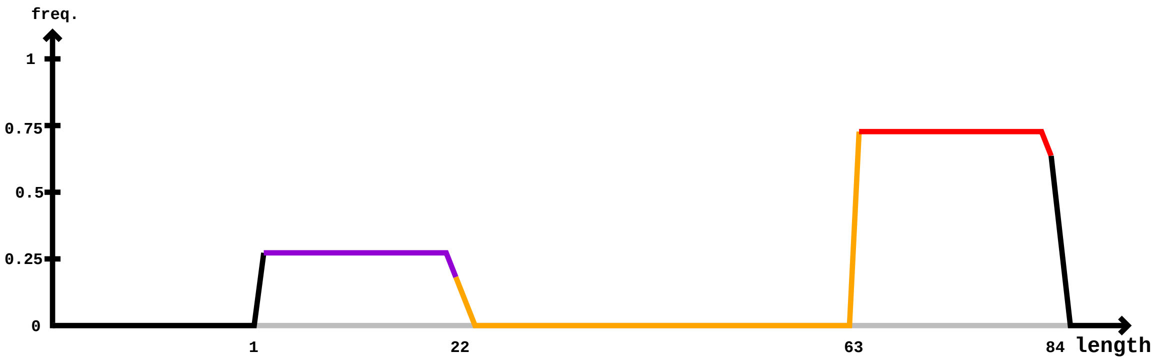

Star **Mature**

```
5'- uagaacaagaggguagaaauaucugccgacucauucauccaaacacucugguaaaaacaagagaucuaaaccacagugacugugugaaugaugcgggagauguuuuucgu  -3'   obs
    uagaacaagaggguagaaauaucugccgacucauucauccaaacacucugguaaaaacaagagaucuaaaccacagugacugugugaaugaugcgggagauguuuuucgu         exp
    ...........(.((((((((((.(((..(((((((..((..((((.((((................)))).))))..))..)))))))..))).))))))))))).  reads   mm   sample
    ....................aucugccgacucauucaucca.....................................................................  1       0    S03
    ..................................................................................ugugaaugaugcgggagaugu.......  2       0    S03

    ....................aucugccgacucauucaucc......................................................................  1       0    S04
    ....................aucugccgacucauucauccC.....................................................................  1       1    S04
    ..................................................................................ugugaaugaugcgggagauA........  1       1    S04
    ..................................................................................ugugaaugaugcgggagaugC.......  1       1    S04
    ..................................................................................ugugaaugaugcgggagaugu.......  2       0    S04
    ..................................................................................ugCgaaugaugcgggagaugu.......  1       1    S04
    ..................................................................................ugugaaugaugcgggagauguu......  1       0    S04
```

Provisional ID	:	chrA05_412035
Score total	:	0.4
Score for star read(s)	:	-1.3
Score for read counts	:	0
Score for mfe	:	1.7
Score for randfold	:	0
Score for cons. seed	:	
Total read count	:	1116
Mature read count	:	1116
Loop read count	:	0
Star read count	:	0

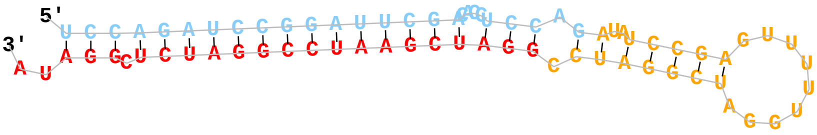

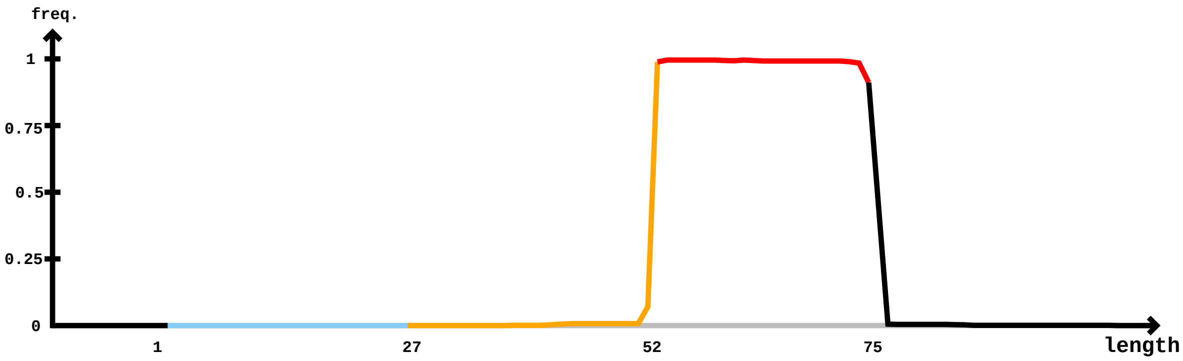

Star Mature

```
5'- cauuuuacuauccagauccggauucgacaccuccagauauccgaguuuuuggaucggauccggaucgaauccggaucucggauaaaauucccaagccuaauauccuccauuag  -3'  exp
    ........(((((((((((((((((((....(((.((..(((((.........))))))).)))))))))))))))))).))))).............(((((.......)))))   reads  mm  sample
    ..................................................ggaucggauccggaucga.............................................   1  0  S03
    ..................................................ggaucggauccggaucgaauc..........................................   1  0  S03
    ...................................................gaucggauccggaucgaaucc.........................................   1  0  S03
    ....................................................aucggauccggaucgaau...........................................   1  0  S03
    ....................................................aucggauccggaucgaaucG.........................................   1  1  S03
    ............................................................cggaucgaauccggaucucgg................................   1  0  S03
    ............................................................cggaucgaauccggaucucgga...............................   1  0  S03
    ............................................................cggaucgaauccggaucucggau..............................   11  0  S03
    ............................................................cggaucgaauccggaucucggauG.............................   2  1  S03
    ............................................................cggaucgaauccggaucucggaua.............................   21  0  S03
    ............................................................cggaucgaauccggUucucggaua.............................   1  1  S03
    .............................................................ggaucgaauccggaucucggau..............................   23  0  S03
    .............................................................ggaucgaauccggaucucggaA..............................   1  1  S03
    .............................................................ggaucgaauccggaucucggCua.............................   1  1  S03
    .............................................................ggaCcgaauccggaucucggaua.............................   1  1  S03
    .............................................................ggaucgaauccggaucucggaGa.............................   3  1  S03
    .............................................................ggGucgaauccggaucucggaua.............................   2  1  S03
    .............................................................ggaucgaauccggaucucggaua.............................   324  0  S03
    .............................................................Ngaucgaauccggaucucggaua.............................   2  1  S03
    .............................................................ggaucgaauccggUucucggaua.............................   1  1  S03
    .............................................................ggaucgaauccggaucucggauU.............................   3  1  S03
    .............................................................ggaucgaauccggaucucggauG.............................   119  1  S03
    .............................................................ggaucgaauccggaucucggauUa............................   1  1  S03
    .............................................................ggaucgaauccggaucucggauaa............................   4  0  S03
    ..............................................................gGucgaauccggaucucggaua.............................   1  1  S03
    ......................................................................ccCgaucucggauaaaauuccc.....................   1  1  S03

    ..............................................uuuuggaAcggauccggaucga.............................................   1  1  S04
    ..................................................ggaucggauccggaucgaauc..........................................   1  0  S04
    ...................................................gaucggauccggaucgaa............................................   1  0  S04
    ............................................................cggaucgaauccggaucucggau..............................   6  0  S04
    ............................................................cggaucgaauccggGucucggau..............................   1  1  S04
    ............................................................cggaucgaauccggaucucggauG.............................   1  1  S04
    ............................................................cggaucgaauccggaucucggauU.............................   2  1  S04
```

Star **Mature**

```
cauuuuacuauccagauccggauucgacaccuccagauauccgaguuuuuggaucggauccggaucgaauccggaucucggauaaaauucccaagccuaauauccuccauuag
............................................................cggaucgaauccggaucucggaua.............................   25    0    S04
.............................................................ggaucgaauccggaucucgg................................   2     0    S04
.............................................................ggaucgaauccggaucucgga...............................   4     0    S04
.............................................................ggaucgaauccggaucucggau..............................   39    0    S04
.............................................................ggaucgaauccggGucucggau..............................   1     1    S04
.............................................................ggaCcgaauccggaucucggaua.............................   2     1    S04
.............................................................ggaucgaauccggaucucggaAa.............................   1     1    S04
.............................................................ggaucgaauccggaucucggaua.............................   365   0    S04
.............................................................ggGucgaauccggaucucggaua.............................   1     1    S04
.............................................................gCaucgaauccggaucucggaua.............................   1     1    S04
.............................................................ggaucgaauccgCaucucggaua.............................   1     1    S04
.............................................................ggaucgaauccggGucucggaua.............................   2     1    S04
.............................................................ggaucgaauccggaucucggaGa.............................   4     1    S04
.............................................................ggaucgaauccggaucucggauG.............................   108   1    S04
.............................................................ggaucgaauccggaGcucggaua.............................   1     1    S04
.............................................................ggaucgaauccggaAcucggaua.............................   1     1    S04
.............................................................ggaucgaauccggaucucggauU.............................   4     1    S04
.............................................................ggaucgaauccggaucucggauaG............................   1     1    S04
.............................................................ggaucgaauccggaucucggauaU............................   1     1    S04
.............................................................ggaucgaauccggaucucggauaa............................   11    0    S04
.............................................................ggaucgaauccggaucucggauaaa...........................   1     0    S04
..............................................................gaucgaauccggaucucgga...............................   1     0    S04
..............................................................gaucgaauccggaucucggaua.............................   4     0    S04
..............................................................gaucgaauccggaucucggauG.............................   2     1    S04
......................................................................ccggaucucggauaaaauuccca....................   1     0    S04
......................................................................ccggaucucggauaaaauucccaG...................   2     1    S04
.....................................................................................Gauucccaagccuaauauccucca....   1     1    S04
```

Provisional ID	:	chrA05_414144
Score total	:	1.4
Score for star read(s)	:	-1.3
Score for read counts	:	0
Score for mfe	:	2.7
Score for randfold	:	0
Score for cons. seed	:	
Total read count	:	12
Mature read count	:	12
Loop read count	:	0
Star read count	:	0

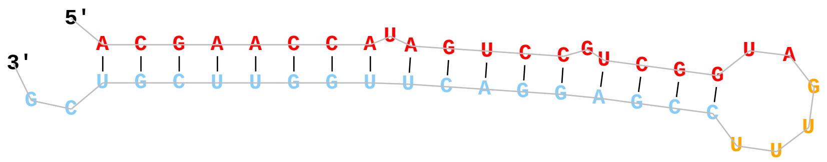

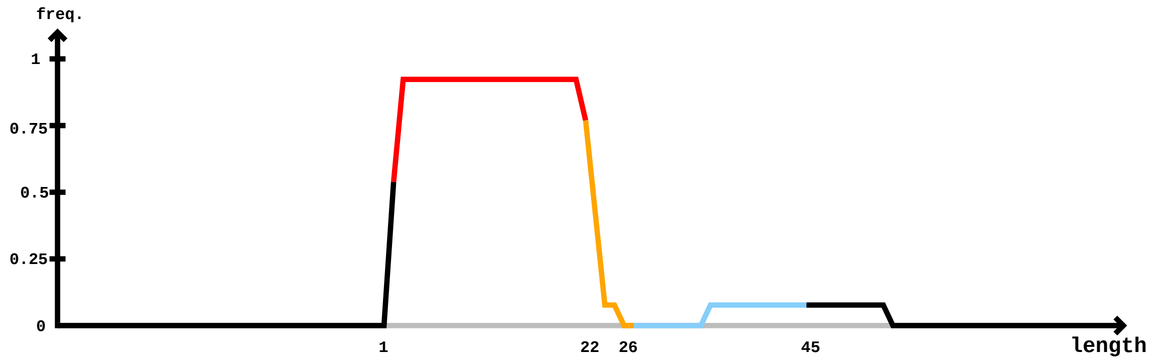

Mature Star

```
5'- uaccgacgaaucaauguucgucggaacauuccgacgaaccauaguccgucgguaguuuccgaggacuugguucgucgguauuuuucgaggacuugguucgucgguauau  -3'   exp
    (((((((((((((.((((.((((((.((.(((((((((((.(((((.((((......))))))))))))))))))))).)).)))))))))))))))))))))))...   reads    mm    sample
    .................................acgaaccaAaguccgucggu........................................................   1        1     S04
    .................................acgaaccauaguccgucggua.......................................................   6        0     S04
    ..................................cgaaccauaguccgucggC........................................................   1        1     S04
    ..................................cgaaccauaguccgucggua.......................................................   1        0     S04
    ..................................cgaaccauaguccgucgguag......................................................   1        0     S04
    ..................................cgaaccauaguccgucgguaA......................................................   1        1     S04
    ..................................cgaaccauaguccgucgguaCuu....................................................   1        1     S04
    ...................................................................uugguucgucggAauuuuu.......................   1        1     S04
```

Provisional ID	:	chrA05_430842
Score total	:	0
Score for star read(s)	:	3.9
Score for read counts	:	-4.4
Score for mfe	:	0.5
Score for randfold	:	0
Score for cons. seed	:	
Total read count	:	3
Mature read count	:	2
Loop read count	:	0
Star read count	:	1

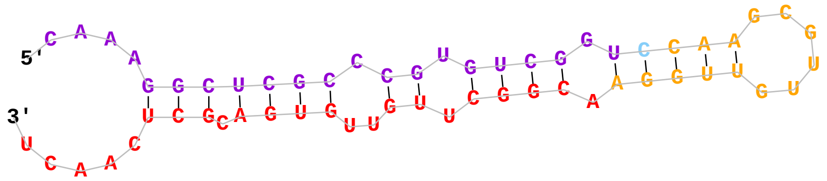

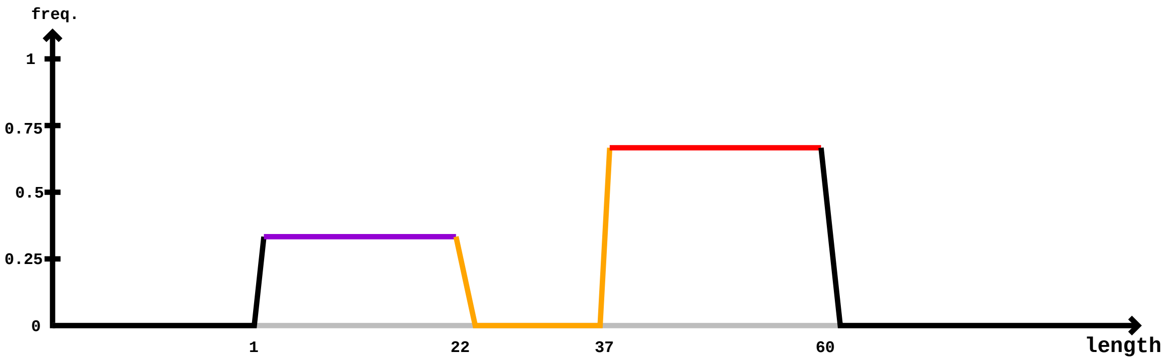

Star **Mature**

```
5'- aguagaggagauucuauuaccaaaggcucgcccgugucgguccaagcguuguuggaacggcuuguugugacgcucaacugguucucuuguuucucuggcuuuuggcugagg   -3'    obs
    aguagaggagauucuauuaccaaaggcucgcccgugucgguccaagcguuguuggaacggcuuguugugacgcucaacugguucucuuguuucucuggcuuuuggcugagg          exp
    .....(((((.......((((..(((((((.((.((((.(((((......))))).)))).))..)))).)))....)))))))))....(((.(((.....)))))).   reads    mm    sample
    ....................caaaggcucgcccgugucggu......................................................................   1        0     S03

    ........................................................acggcuuguugugacgcucaacC................................   1        1     S04
    ........................................................acggcuuguugugacgcGcaacu................................   1        1     S04
```

Provisional ID	:	chrA05_439469
Score total	:	1757.8
Score for star read(s)	:	3.9
Score for read counts	:	1751.9
Score for mfe	:	2
Score for randfold	:	0
Score for cons. seed	:	
Total read count	:	3448
Mature read count	:	3436
Loop read count	:	0
Star read count	:	12

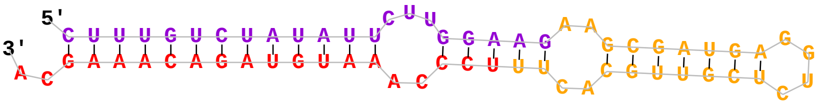

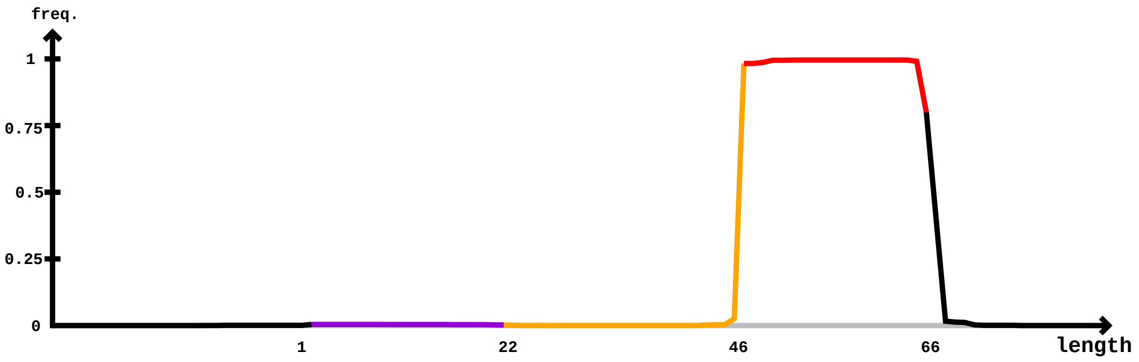

Star Mature

```
5'- aaaauaacacgucuucucgguauuucuuugucuauauucuuggaagaagcgaugaggucucguugcacuuucccaaauguagacaaagcaaaaccgugaugacgucaa  -3'  obs
    aaaauaacacgucuucucgguauuucuuugucuauauucuuggaagaagcgaugaggucucguugcacuuucccaaauguagacaaagcaaaaccgugaugacgucaa       exp
    ........(((((.((.((((.((.(((((((((((((...(((((..(((((((....)))))))..)))))..))))))))))))).)).)))).)).)))))...  reads  mm  sample
    ..............ucucgguauuucuuugucu...........................................................................  1  0  S03
    ................ucgguauuucuuugucuauauucu....................................................................  1  0  S03
    .........................cuuugucuauauucuuggaaC..............................................................  1  1  S03
    .................................................................cacuuucccaaauguagacaaagc...................  1  0  S03
    ..................................................................acuuucccaaauguagacaaagca..................  1  0  S03
    ...................................................................cuuucccaaauguagacaaag....................  2  0  S03
    .....................................................................uucccaaauguagacaaagc...................  1  0  S03
    .....................................................................uucccaaauguagacaaagU...................  2  1  S03
    .....................................................................Cucccaaauguagacaaagc...................  1  1  S03
    .....................................................................uucccGaauguagacaaagc...................  1  1  S03
    .....................................................................uuGccaaauguagacaaagca..................  3  1  S03
    .....................................................................Cucccaaauguagacaaagca..................  1  1  S03
    .....................................................................uucccaaauguagacaaagca..................  15  0  S03
    .....................................................................uGcccaaauguagacaaagca..................  1  1  S03
    .....................................................................Gucccaaauguagacaaagca..................  1  1  S03
    .....................................................................uucccaaauguagacaaaAca..................  1  1  S03
    .....................................................................uucccaaauguagacaaaCca..................  1  1  S03
    .....................................................................uucccaaauguagacaaagcaU.................  1  1  S03
    ......................................................................ucccaaauguagacaaaA....................  1  1  S03
    ......................................................................ucccaaaugAagacaaag....................  1  1  S03
    ......................................................................ucccaaauguagacaaag....................  5  0  S03
    ......................................................................ucccaaauguagGcaaagc...................  1  1  S03
    ......................................................................ucccaaaugGagacaaagc...................  1  1  S03
    ......................................................................ucccaaauguagacaaagU...................  54  1  S03
    ......................................................................ucccaaauguagacaaagc...................  175  0  S03
    ......................................................................ucccaaaCguagacaaagc...................  1  1  S03
    ......................................................................ucccaaauguagacaaagG...................  2  1  S03
    ......................................................................ucccaaauguagaUaaagc...................  1  1  S03
    ......................................................................Ccccaaauguagacaaagc...................  1  1  S03
    ......................................................................ucccaGauguagacaaagc...................  1  1  S03
    ......................................................................ucccaaauguagacaaagA...................  7  1  S03
    ......................................................................Gcccaaauguagacaaagc...................  3  1  S03
    ......................................................................uccAaaauguagacaaagca..................  1  1  S03
```

```
                         Star                                           Mature
aaaauaacacgucuucucgguauuucuuugucuauauucuuggaagaagcgaugaggucucguugcacuuucccaaauguagacaaagcaaaaccgugaugacgucaa
......................................................................ucccaaauguagacaaagca..................  733  0  S03
......................................................................ucccaaauguagacaaagcU..................  40  1  S03
......................................................................ucccaGauguagacaaagca..................  1  1  S03
......................................................................ucccaaauguagacaaGgca..................  2  1  S03
......................................................................ucccaaauguagacGaagca..................  3  1  S03
......................................................................ucccaaaCguagacaaagca..................  1  1  S03
......................................................................ucccaaauguagacaaagAa..................  1  1  S03
......................................................................ucccaaauguagacaCagca..................  1  1  S03
......................................................................ucccaaauguagacaaaCca..................  2  1  S03
......................................................................ucccaaauguaAacaaagca..................  2  1  S03
......................................................................ucccaaauguagacaaaUca..................  3  1  S03
......................................................................Ccccaaauguagacaaagca..................  2  1  S03
......................................................................Gcccaaauguagacaaagca..................  2  1  S03
......................................................................ucccaaaugGagacaaagca..................  3  1  S03
......................................................................ucccaaauguagGcaaagca..................  4  1  S03
......................................................................uGccaaauguagacaaagca..................  1  1  S03
......................................................................ucccaaauguagaGaaagca..................  1  1  S03
......................................................................ucccaCauguagacaaagca..................  1  1  S03
......................................................................ucccaaauguaCacaaagca..................  1  1  S03
......................................................................ucccaaauguagacaaagUa..................  1  1  S03
......................................................................ucccaaauguagacaaaAca..................  1  1  S03
......................................................................Ncccaaauguagacaaagca..................  1  1  S03
......................................................................ucccaaauguUgacaaagca..................  2  1  S03
......................................................................ucccGaauguagacaaagca..................  3  1  S03
......................................................................ucccaaauguagacaaagcG..................  34  1  S03
......................................................................ucccaaGuguagacaaagca..................  2  1  S03
......................................................................ucccaaauguGgacaaagca..................  2  1  S03
......................................................................ucccaaauguagacaUagca..................  1  1  S03
......................................................................ucccaaauguagCcaaagca..................  1  1  S03
......................................................................ucccaaaugCagacaaagca..................  4  1  S03
......................................................................ucccCaauguagacaaagca..................  1  1  S03
......................................................................ucccaaauguagacaaagcC..................  9  1  S03
......................................................................ucccaaaugAagacaaagca..................  6  1  S03
......................................................................ucccaaauguagacaGagca..................  4  1  S03
......................................................................ucccaaauguagacaaagGa..................  1  1  S03
......................................................................ucccaaauguagacaaagcUa.................  1  1  S03
......................................................................ucccaaauguagacaaagcaU.................  25  1  S03
......................................................................ucccaaauguagacaaagcaC.................  6  1  S03
......................................................................ucccaaauguagacaaagcaa.................  2  0  S03
........................................................................ccaaauguagacaaagcaaa................  1  0  S03
........................................................................ccaaauguagacaaagcaaU................  1  1  S03
........................................................................ccaaauguagacaaagcaaC................  1  1  S03
........................................................................ccaaauguagacaaagcaaaaU..............  1  1  S03
........................................................................ccaaauguagacaaagcaaaac..............  2  0  S03
........................................................................ccaaauguagacaaagcaaaacU.............  2  1  S03
.........................................................................caaauguagacaaagcaaaa...............  2  0  S03
.........................................................................caaauguagacaaagcaaaac..............  8  0  S03
.........................................................................caaauguagacaaagcaaaaU..............  2  1  S03
...........................................................................aauguagacaaagcaaaaccguga.........  1  0  S03

................ucgguauuucuuugucuauauucC....................................................................  1  1  S04
.........................cuuugucuauauucuugga................................................................  3  0  S04
.........................cuuugucuauauucuuggaa...............................................................  1  0  S04
.........................cuuugucuauauucuuggaag..............................................................  1  0  S04
.........................cuuugucuauauucuuggaaU..............................................................  2  1  S04
.........................cuuugucuauauucuuggaagU.............................................................  2  1  S04
.........................cuuugucuauauucuuggaagC.............................................................  1  1  S04
.........................cuuugucuauauucuuggaagaaUc..........................................................  1  1  S04
..................................................................acuuucccaaauguagacaaagc...................  1  0  S04
..................................................................acuuucccaaauguagacaGagca..................  1  1  S04
..................................................................acuuAcccaaauguagacaaagca..................  1  1  S04
..................................................................acuuucccaaauguagacaaagca..................  1  0  S04
..................................................................acuuucccaaauguagacaaagcaU.................  1  1  S04
...................................................................cuuucccaaauguagacaaagca..................  1  0  S04
....................................................................uuucccaaauguagacaaagA...................  1  1  S04
.....................................................................uucccaaauguagacaaagc...................  6  0  S04
.....................................................................uuGccaaauguagacaaagc...................  2  1  S04
.....................................................................uucccaaauguagacaaagA...................  1  1  S04
.....................................................................Aucccaaauguagacaaagc...................  1  1  S04
.....................................................................uuGccaaauguagacaaagca..................  4  1  S04
```

Star **Mature**

```
aaaauaacacgucuucucgguauuucuuugucuauauucuuggaagaagcgaugaggucucguugcacuuucccaaauguagacaaagcaaaaccgugaugacgucaa
.....................................................................uucccaaauguagacaaagcU..................	1	1	S04
.....................................................................uucccaaauguagacaaaAca..................	1	1	S04
.....................................................................uucccaaauguagacaaagcG..................	3	1	S04
.....................................................................uucccCaauguagacaaagca..................	1	1	S04
.....................................................................uucccaaauguagacaaagca..................	25	0	S04
.....................................................................uucccGaauguagacaaagca..................	1	1	S04
.....................................................................Cucccaaauguagacaaagca..................	1	1	S04
.....................................................................uucccaaauguagacaaagcaU.................	1	1	S04
......................................................................ucccaaauguagacaaag....................	6	0	S04
......................................................................ucccaaauguagacaaaU....................	1	1	S04
......................................................................ucccaaauguagacaaCgc...................	1	1	S04
......................................................................ucccaaauguGgacaaagc...................	2	1	S04
......................................................................ucccaaauguagGcaaagc...................	1	1	S04
......................................................................Gcccaaauguagacaaagc...................	1	1	S04
......................................................................ucccaaauguagacaaagA...................	11	1	S04
......................................................................ucccaGauguagacaaagc...................	2	1	S04
......................................................................ucccaaauguagacaaaUc...................	1	1	S04
......................................................................ucccaaauguagacaaagc...................	289	0	S04
......................................................................ucccaaauguCgacaaagc...................	1	1	S04
......................................................................ucccaaauguagacGaagc...................	1	1	S04
......................................................................ucccaaauguagacaaagU...................	78	1	S04
......................................................................ucccaaauguagaGaaagc...................	1	1	S04
......................................................................ucccaaauguagacaaagG...................	1	1	S04
......................................................................ucccaaaugAagacaaagc...................	2	1	S04
......................................................................ucccaaauguagacaaGgc...................	1	1	S04
......................................................................Ncccaaauguagacaaagc...................	1	1	S04
......................................................................ucccaaauguagacaaaAc...................	3	1	S04
......................................................................ucccaaauguaCacaaagca..................	1	1	S04
......................................................................ucccaaauguagacaaagca..................	1312	0	S04
......................................................................ucccaaaAguagacaaagca..................	1	1	S04
......................................................................ucccaaauguagacaaagAa..................	2	1	S04
......................................................................ucccaaauguagacCaagca..................	1	1	S04
......................................................................ucUcaaauguagacaaagca..................	1	1	S04
......................................................................uccGaaauguagacaaagca..................	1	1	S04
......................................................................Gcccaaauguagacaaagca..................	4	1	S04
......................................................................ucccaaauguagacaaagcG..................	87	1	S04
......................................................................ucccaaauguagCcaaagca..................	1	1	S04
......................................................................ucccaaauguagGcaaagca..................	5	1	S04
......................................................................ucccaaauguagacaaUgca..................	3	1	S04
......................................................................ucccaaauguagacaGagca..................	2	1	S04
......................................................................ucccaaaugCagacaaagca..................	6	1	S04
......................................................................ucccaaauguagacaaagcC..................	9	1	S04
......................................................................ucccaaaCguagacaaagca..................	2	1	S04
......................................................................ucGcaaauguagacaaagca..................	1	1	S04
......................................................................ucccaaauguUgacaaagca..................	2	1	S04
......................................................................ucccGaauguagacaaagca..................	4	1	S04
......................................................................ucccaaauguagacaaGgca..................	2	1	S04
......................................................................ucccCaauguagacaaagca..................	1	1	S04
......................................................................ucccaaauguagacaaagUa..................	1	1	S04
......................................................................ucccaGauguagacaaagca..................	3	1	S04
......................................................................ucccaaauguagacaaaAca..................	13	1	S04
......................................................................ucccaaaugAagacaaagca..................	6	1	S04
......................................................................Ncccaaauguagacaaagca..................	3	1	S04
......................................................................Ccccaaauguagacaaagca..................	7	1	S04
......................................................................ucccaaauUuagacaaagca..................	1	1	S04
......................................................................ucccaaaugGagacaaagca..................	9	1	S04
......................................................................ucccaaUuguagacaaagca..................	1	1	S04
......................................................................uGccaaauguagacaaagca..................	2	1	S04
......................................................................ucccaaauguaUacaaagca..................	3	1	S04
......................................................................ucccaaauguagacGaagca..................	3	1	S04
......................................................................Acccaaauguagacaaagca..................	2	1	S04
......................................................................ucccaaGuguagacaaagca..................	1	1	S04
......................................................................uccAaaauguagacaaagca..................	1	1	S04
......................................................................ucccaaauguagacaaagcU..................	113	1	S04
......................................................................ucccaaauguGgacaaagca..................	4	1	S04
......................................................................uccUaaauguagacaaagca..................	2	1	S04
......................................................................ucccaaauguagacaaaCca..................	2	1	S04
......................................................................ucccaaauguagacaaaUca..................	3	1	S04
......................................................................ucccaaauguagacaaagcaa.................	6	0	S04
......................................................................ucccaaauguagacaaagcaC.................	13	1	S04
```

Star Mature

```
aaaauaacacgucuucucgguauuucuuugucuauauucuuggaagaagcgaugaggucucguugcacuuucccaaauguagacaaagcaaaaccgugaugacgucaa
......................................................................ucccaaauguagacaaagcUa.................    2     1     S04
......................................................................ucccaaauguagacaaagcaU.................    85    1     S04
......................................................................ucccaaauguagacaaagcaG.................    1     1     S04
......................................................................ucccaaauguagacaaagcaaU................    6     1     S04
......................................................................ucccaaauguagacaaagcaUa................    1     1     S04
......................................................................ucccaaauguagacaaagcaaC................    1     1     S04
......................................................................ucccaaauguagacaaagcaUaa...............    1     1     S04
......................................................................ucccaaauguagacaaagcaaUa...............    1     1     S04
......................................................................ucccaaauguagacaaagcaaaUc..............    1     1     S04
........................................................................ccaaauguagacaaagcaaaac..............    3     0     S04
........................................................................ccaaauguagacaaagcaaaaU..............    2     1     S04
........................................................................ccaaauguagacaaagcaaaacU.............    1     1     S04
.........................................................................caaUuguagacaaagcaaaac..............    1     1     S04
.........................................................................Naaauguagacaaagcaaaac..............    1     1     S04
.........................................................................caaauguagacaaagcaaaUc..............    1     1     S04
.........................................................................caaauguagacaaagcaaaac..............    10    0     S04
.........................................................................caaauguagacaaagcaaaacU.............    3     1     S04
...........................................................................aauguagacaaagcaaaaccguga.........    1     0     S04
............................................................................auguagacaaagcaaaaccguga.........    1     0     S04
```

Provisional ID	:	chrA05_469150
Score total	:	14.6
Score for star read(s)	:	3.9
Score for read counts	:	9.3
Score for mfe	:	1.4
Score for randfold	:	0
Score for cons. seed	:	
Total read count	:	30
Mature read count	:	27
Loop read count	:	0
Star read count	:	3

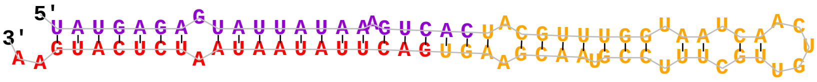

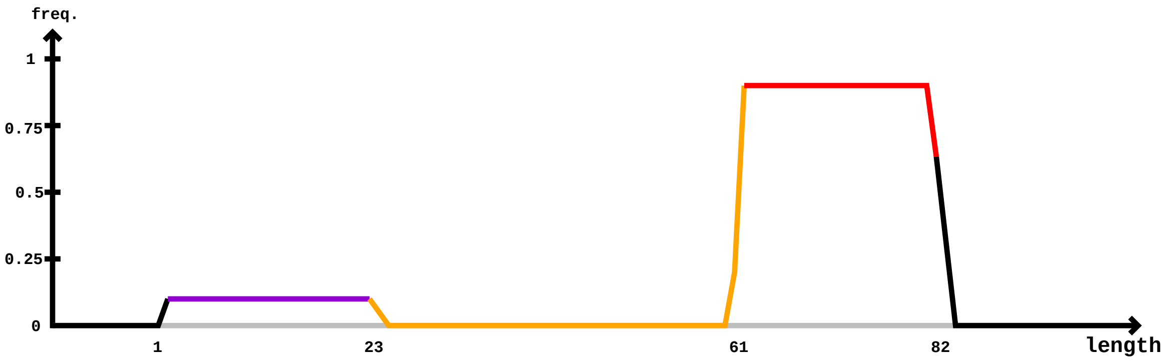


```
          Star                                                        Mature
5'- cucaacuguuuaugagaguauuauaaagucacuacguuugguaaucaacuguugcuuuccguaacgaagugacuuauaauaaucucaugaauggaucuauguuaacuguug   -3'    obs
    cucaacuguuuaugagaguauuauaaagucacuacguuugguaaucaacuguugcuuuccguaacgaagugacuuauaauaaucucaugaauggaucuauguuaacuguug          exp
    .....((((((((((((.((((((((.((((((.(((((((.((.((.....)).)).))).)))).))))))))))))))).)))))))))))).................    reads    mm    sample
    ..........uaugagaguauuauaaagucac...............................................................................    1        0     S03
    ..........uaugagaguauuauaaGgucacu..............................................................................    1        1     S03
    .....................................................................ugacuuauaauaaucucaugaa....................    1        0     S03
    ......................................................................gacuuauaauaaucucauga.....................    3        0     S03
    ......................................................................gacuuauaauaaucucaugaa....................    10       0     S03

    ..........uaugagaguauuauaaagucac...............................................................................    1        0     S04
    .....................................................................ugacuuauaauaaucucaugU.....................    1        1     S04
    .....................................................................ugacuuauaauaaucucauga.....................    3        0     S04
    .....................................................................ugacuuauaauaaucucaugaa....................    1        0     S04
    ......................................................................gacuuauaauaaucucauga.....................    1        0     S04
    ......................................................................gacuuauaauaaucucaugaG....................    1        1     S04
    ......................................................................Nacuuauaauaaucucaugaa....................    1        1     S04
    ......................................................................gacuuauaauaaucucaugaa....................    5        0     S04
```

Provisional ID	:	chrA05_random_2717832
Score total	:	0.8
Score for star read(s)	:	-1.3
Score for read counts	:	0
Score for mfe	:	2.1
Score for randfold	:	0
Score for cons. seed	:	
Total read count	:	136
Mature read count	:	129
Loop read count	:	2
Star read count	:	5

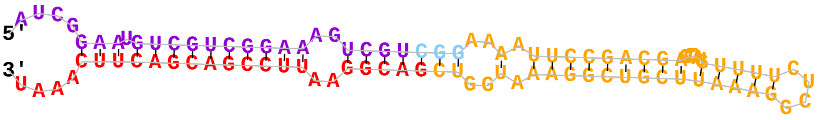

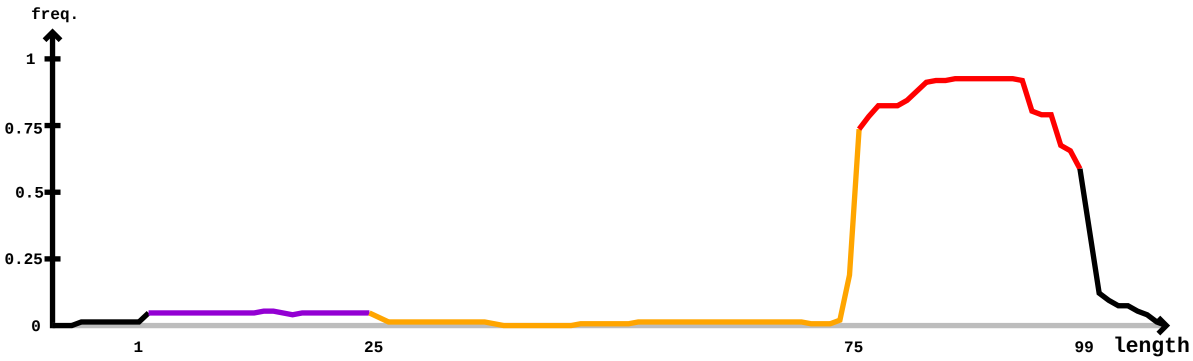

Star **Mature**

```
5'- uauuuuuuaucggaaugucgucggaaagucgucggaaaauuccgacgagccauguuuucucggaaauucgucggaaauggucgacggaauuccgacgacuucaaauuuuuuguu  -3'  obs
    uauuuuuuaucggaaugucgucggaaagucgucggaaaauuccgacgagccauguuuucucggaaauucgucggaaauggucgacggaauuccgacgacuucaaauuuuuuguu        exp
    ............(((.((((((((((..(((((((..(.(((((((((.....(((((....))))))))))))))).)..)))))))..)))))))))))))............  reads  mm  sample
    .auuuuuuaucggaaugucguc............................................................................................  1  0  S03
    ........aucggaaugucgucAgaaagucgu..................................................................................  1  1  S03
    ...........................................................ucggaaUuucgucggaaaugguc................................  1  1  S03
    ................................................................................Ncgacggaauuccgacgacuucaa..........  1  1  S03
    .................................................................................cgacggaauuccgacgCcu..............  2  1  S03
    .................................................................................cgacggaauuccgacgacuuca...........  2  0  S03
    .................................................................................cgacggaauuccgacgacuucaaa.........  5  0  S03
    ..................................................................................gacggaauuccgacgacu..............  3  0  S03
    ..................................................................................gacggaauuccgacgCcu..............  2  1  S03
    ..................................................................................gacggaauuccgacgacuuca...........  2  0  S03
    ..................................................................................gacggaauuccgacgacuucaaa.........  1  0  S03
    ..................................................................................gacggaauuccgaUgacuucaaau........  1  1  S03
    ..................................................................................gacggaauuccgacgacuucaaaC........  1  1  S03
    ..................................................................................gacggaauuccgacgacuucaaau........  22  0  S03
    ..................................................................................gacggGauuccgacgacuucaaau........  1  1  S03
    ..................................................................................gacggaauuccgacgacuucaaaG........  1  1  S03
    ..................................................................................gacggaauuccAacgacuucaaau........  1  1  S03
    ...................................................................................acggaauuccgacgacuuca...........  1  0  S03
    ...................................................................................acggaauuccgacgacuucaaau........  1  0  S03
    ....................................................................................cggaauuccgacgacuuca...........  1  0  S03
    ....................................................................................cggaauuccgacgacuucaaauu.......  1  0  S03
    ....................................................................................cggaauuccgacgacuucaaauuu......  2  0  S03
    .......................................................................................aauuccgacgacuucaaauuu......  1  0  S03
    .......................................................................................aauuccgacgacuucaaauuuu.....  1  0  S03
    .......................................................................................aauuccgacgacuucaaauuuuuC...  1  1  S03
    ........................................................................................auuccgacgacuucaaauuuu.....  2  0  S03
    ........................................................................................auuccgacgUcuucaaauuuuuug..  1  1  S03
    .........................................................................................uuccgacgacuucaaauuuuuugu.  2  0  S03
    .........................................................................................uuccgacgacuucaaauuuuuugA.  1  1  S03

    .auuuuuuaucggaaugucgucg...........................................................................................  1  0  S04
    ........aucggaaugucgucggaaGgucgu..................................................................................  1  1  S04
    ........aucggaaugucgucAgaaagucgu..................................................................................  1  1  S04
```

Star **Mature**

```
uauuuuuuaucggaaugucgucggaaagucgucggaaaauuccgacgagccauguuuucucggaaauucgucggaaauggucgacggaauuccgacgacuucaaauuuuuuguu
........aucggaaugucgucggaaagucgu..................................................................................  2  0  S04
....................ucggaaagucgucggaaaauuccg......................................................................  1  0  S04
........................aaagucgucggaaaauuccga.....................................................................  1  0  S04
.....................................................guuuucucggaaauucgucggaaa.....................................  1  0  S04
................................................................................ucgacggaauuccgacgCcu..............  1  1  S04
.................................................................................Agacggaauuccgacgac...............  1  1  S04
.................................................................................cgacggaauuccgacgCcu..............  3  1  S04
.................................................................................cgacggaauuccgacgacu..............  1  0  S04
.................................................................................cgacggaauuccgacgacuu.............  1  0  S04
.................................................................................cgacggaauuccgacgacuucG...........  1  1  S04
.................................................................................cgacggaauuccgacgacuuca...........  5  0  S04
.................................................................................cgacggaauuccgacgacuGca...........  1  1  S04
.................................................................................cgacggaauuccgacgacuucaaa.........  2  0  S04
.................................................................................cgacggaauuccgacgacuucaaauuu......  1  0  S04
..................................................................................gacggaauuccgacgacu..............  5  0  S04
..................................................................................gacggaauuccgaAgacuu.............  1  1  S04
..................................................................................gacggaauuccgacgacuucU...........  1  1  S04
..................................................................................gacggaauuccgacgacuuca...........  2  0  S04
..................................................................................gacggaauuccgacgacuucaa..........  2  0  S04
..................................................................................gacggaauuccgacgacuucaaa.........  2  0  S04
..................................................................................gacgAaauuccgacgacuucaaau........  2  1  S04
..................................................................................gacggaauuccgacgacuucaaaG........  1  1  S04
..................................................................................gacggaauuccgacgacuucaaau........  29  0  S04
..................................................................................gacggaauuccgacgacuucaaauu.......  2  0  S04
...................................................................................acggaauuccgacgacuucaaauu.......  5  0  S04
....................................................................................cggaauuccgacgacuuca...........  1  0  S04
....................................................................................cggaauuccgacgacuucaaau........  1  0  S04
........................................................................................auuccgacgacuucaaauuuuuA...  1  1  S04
........................................................................................auuccgacgacuucaaauuuuuug..  1  0  S04
.........................................................................................uuccgacgacuucaaauuuuuA...  1  1  S04
.........................................................................................uuccgacgacuucaaauuuuuugu.  1  0  S04
..........................................................................................uccgacgacuucaaauuuuuuguu  1  0  S04
............................................................................................cgacgacuucaaauuuuuuguu  1  0  S04
```

Provisional ID	:	chrA05_random_2727006
Score total	:	0.7
Score for star read(s)	:	-1.3
Score for read counts	:	0
Score for mfe	:	2
Score for randfold	:	0
Score for cons. seed	:	
Total read count	:	37
Mature read count	:	33
Loop read count	:	0
Star read count	:	4

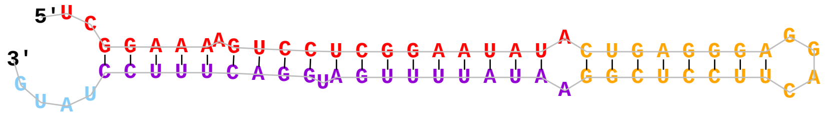

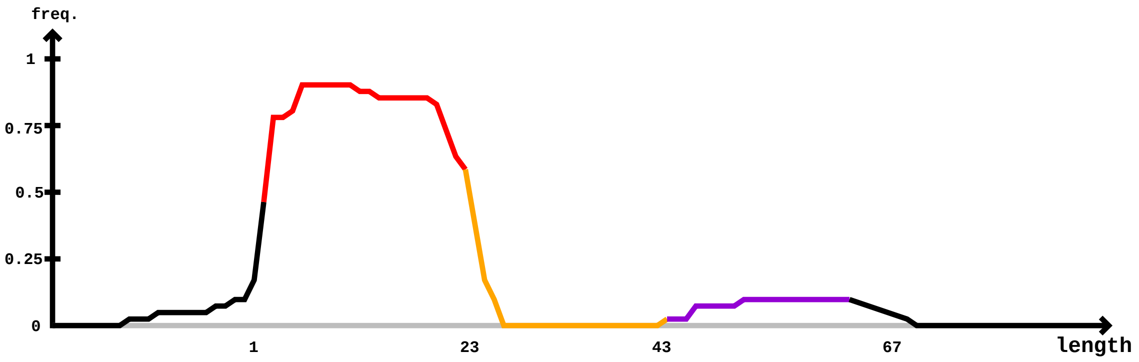


```
                         Mature                                        Star
5'- cuccgaggaccaccaguucgucggaaaaguccucggaauauacugagggaggacuuccucggaauauuuugauggacuuuccuaugguccaauccuuggaaguuccga  -3'   obs
    cuccgaggaccaccaguucgucggaaaaguccucggaauauacugagggaggacuuccucggaauauuuugauggacuuuccuaugguccaauccuuggaaguuccga        exp
    .((((((((..((((.......(((((.(((((((((((((.((((((((....)))))))).))))))))).)))))))))..))))....))))))))........   reads   mm   sample
    ......ggaccaccaguucgucggaaaCgu..............................................................................   1   1   S03
    ...................gucggaaaaguccucggaauaua..................................................................   1   0   S03
    ....................ucggaaaaguccucggaauaua..................................................................   2   0   S03
    .....................cggaaaaguccucggaau.....................................................................   1   0   S03
    .....................cggaaaaguccucggaauU....................................................................   2   1   S03
    .....................cggaaaaguccucggaauauG..................................................................   1   1   S03
    .....................cggaaaaguccucggaauaua..................................................................   1   0   S03
    .....................cggaaaagAccucggaauauac.................................................................   1   1   S03
    .........................aaaguccucggaauauacuU...............................................................   2   1   S03
    .................................................................auuuugauggacuuucGu.........................   1   1   S03
    .................................................................auuuugauggacuuucGua........................   1   1   S03

    .........ccaccaguucgucggaaaagucc............................................................................   1   0   S04
    ...............guucgucggaaaaguccucggaau.....................................................................   1   0   S04
    .................ucgucggaaaaguccucggaa......................................................................   1   0   S04
    ...................gucggaaaaguccucggaauauG..................................................................   1   1   S04
    ...................gucggaaaaguccucggaauauac.................................................................   1   0   S04
    ....................ucggaaaaguccucggaaua....................................................................   1   0   S04
    ....................ucggaaaaguccucggaauau...................................................................   1   0   S04
    ....................ucggaaaaguccucggaauaC...................................................................   1   1   S04
    ....................ucggaaaaguccucggaauaua..................................................................   4   0   S04
    ....................ucggaaaaguccucggaauauU..................................................................   1   1   S04
    ....................ucggaaaaguccucggaauauacA................................................................   1   1   S04
    ....................ucggaaaaguccucggaauauacu................................................................   1   0   S04
    .....................cggaaaaguccucggaUu.....................................................................   1   1   S04
    .....................cggaaaaguccucggaau.....................................................................   1   0   S04
    .....................cggaaaaguccucggaaua....................................................................   1   0   S04
    .....................cggaaaaguccucggaauaua..................................................................   1   0   S04
    .....................cggaaaaguccucggaauauac.................................................................   2   0   S04
    .....................cggaaaaguccucggaauauacA................................................................   1   1   S04
    .......................gaaaaguccucggaaCauac.................................................................   1   1   S04
    .........................aaaguccucggaauauacuU...............................................................   2   1   S04
    ..............................................................aauauuuugauggacuuucA..........................   1   1   S04
```

Mature Star

cuccgaggaccaccaguucgucggaaaaguccucggaauauacugagggaggacuuccucggaauauuuugauggacuuuccuaugguccaauccuuggaaguuccga

....gauggacuuuccuauggu.................... 1 0 S04

Provisional ID	:	chrA06_495712
Score total	:	6.8
Score for star read(s)	:	3.9
Score for read counts	:	1.7
Score for mfe	:	1.3
Score for randfold	:	0
Score for cons. seed	:	
Total read count	:	15
Mature read count	:	4
Loop read count	:	9
Star read count	:	2

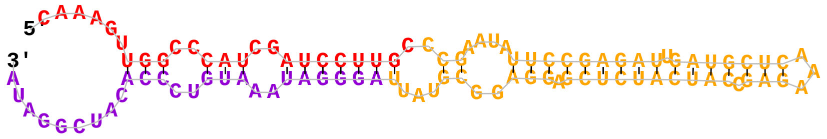

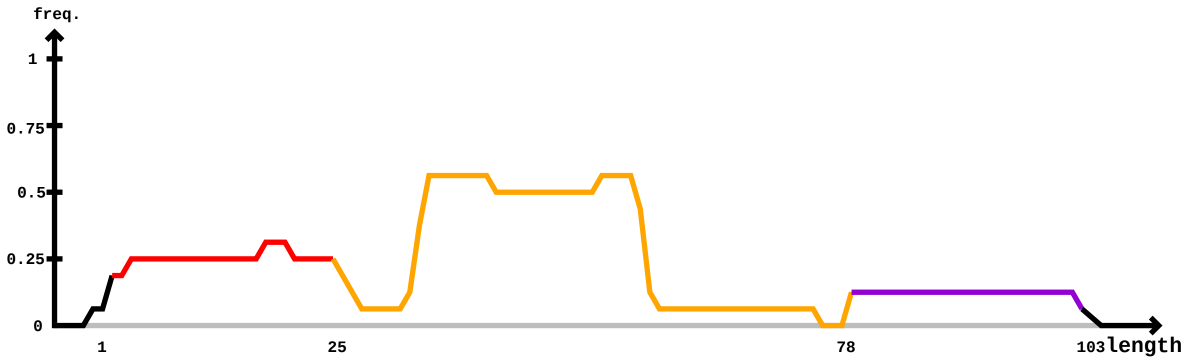


```
        Mature                                                                     Star
5'- aguccaaaguuggcccaucgauccuugcccgaauauuccgagauugaugcucaaagagccaucaucucgaggaggcguauuagggauaaaugucccacaucggauauugaaaa   -3'    obs
    aguccaaaguuggcccaucgauccuugcccgaauauuccgagauugaugcucaaagagccaucaucucgaggaggcguauuagggauaaaugucccacaucggauauugaaaa          exp
    .((((...((.((..(((..(((((((..((....(((((((((.((((((...))).))))))))).)))..))...)))))))..)))..))))...))))..........    reads    mm    sample
    ..uccaaaguuggcccaucgauc..........................................................................................    1        0     S03
    ....Aaaaguuggcccaucgauccuugc.....................................................................................    2        1     S03
    ....................................uccgagauugaugcucaaagagcA.....................................................    1        1     S03
    .....................................ccgagauugaugcucaaagagc......................................................    1        0     S03
    .................................................................................agggauaaaugucccacaucggaua.......    1        0     S03

    ......aaguuggcccaucgauccuugccc...................................................................................    1        0     S04
    ....................auccuugcccgaauauuccgagaC.....................................................................    1        1     S04
    ...................................uuccgagauugaugcucaaagagA......................................................    1        1     S04
    ....................................uccgagauugaugcucaaagagcA.....................................................    1        1     S04
    ....................................uccgagauugaugcucaaagagcc.....................................................    2        0     S04
    .....................................ccgagauugaugcucaaagaAcc.....................................................    1        1     S04
    .....................................ccgagauugaugcucaaagagcca....................................................    1        0     S04
    .......................................................gagccaucaucucgaggaggAgu...................................    1        1     S04
    .................................................................................agggauaaaugucccacaucggau........    1        0     S04
```

Provisional ID : chrA06_504777
Score total : 0.8
Score for star read(s) : -1.3
Score for read counts : 0
Score for mfe : 2.1
Score for randfold : 0
Score for cons. seed :
Total read count : 183
Mature read count : 175
Loop read count : 7
Star read count : 1

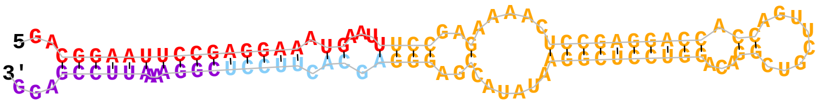

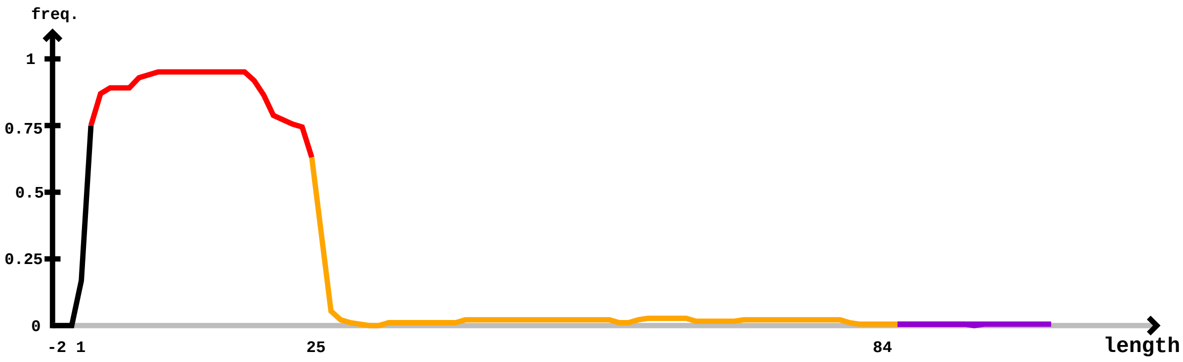

Mature **Star**

5'- ucgacggaauuccgaggaaaugaauuuccgagaaaacuccgaggaccaccaguucgucggacagguccucggaauauaccgagggagcacuuccucggaaaauuccgaggaag -3'	obs			
ucgacggaauuccgaggaaaugaauuuccgagaaaacuccgaggaccaccaguucgucggacagguccucggaauauaccgagggagcacuuccucggaaaauuccgaggaag	exp			
....(((((((((((((((.((...((((..(.....((((((((((.((........))...))))))))))......)..)))).)).)))))))))..))))))......		reads	mm	sample
.cgacggaauuccgaggaa..		3	0	S03
.cgacggaauuccgaggaaa...		1	0	S03
.cgacggaauuccgaggaaau..		1	0	S03
.cgacggaauuccgaggaaaugG..		1	1	S03
.cgacggaauuccgaggaaaugaau..		3	0	S03
.cgacggaauuccgaggaaaugaaC..		2	1	S03
.cgacggaauuccgaggaaaugaauu...		2	0	S03
.cgGcggaauuccgaggaaaugaauuu..		1	1	S03
..gacggaauuccgaggaaa...		2	0	S03
..gacggaauuccgaggaaau..		3	0	S03
..gacggaauuccgaggaaaugaa...		1	0	S03
..gacggaauuccgaggaaaugaau..		2	0	S03
..gacggaauuccgaggaaaugaaA..		1	1	S03
..gacggaauuccgaggaaaugaauA...		1	1	S03
..gacggaauuccgaggaaaugaauG...		1	1	S03
..gacggaauuccgaggaaaugaauu...		26	0	S03
..gacggaauuccAaggaaaugaauu...		1	1	S03
..gacggaGuuccgaggaaaugaauu...		1	1	S03
..gacggaauuccgaggaaaugaauC...		4	1	S03
...acggaauuccgaggaaaugaau..		1	0	S03
...Ccggaauuccgaggaaaugaauuu..		1	1	S03
...acggaauuccgaggaaaugaauuG..		1	1	S03
...acggaauuccgaggaaaugaauuu..		6	0	S03
...acggaauuccgaggaaaugaauuC..		1	1	S03
....cggaauuccgaggaaaugaauu...		2	0	S03
......aauuccgaggaaaugaauuuc..		4	0	S03
......aauuccgaggaaaugaauuuccg..		1	0	S03
........uuccgaggaaaugaauuC...		1	1	S03
........uuccgaggaaaugaauuuc..		1	0	S03
....aggaccaccaguucgucggacagg..		1	0	S03
....aGagguccucggaauauaccga...............................		1	1	S03
....cggaaaauuccgaggaag		1	0	S03
.cgacggaauuccgaggaa..		3	0	S04

```
Mature                                                                                  Star
ucgacggaauuccgaggaaaugaauuuccgagaaaacuccgaggaccaccaguucgucggacagguccucggaauauaccgagggagcacuucccucggaaaauuccgaggaag
.cgacggaauuccgaggaaa..............................................................................................  2   0   S04
.cgacggaauuccgaggaaau.............................................................................................  4   0   S04
.cgacggaauuccgaggaaaug............................................................................................  2   0   S04
.cgacggaauuccgaggaaaugaau.........................................................................................  6   0   S04
..gacggaauuccgaggaaa..............................................................................................  5   0   S04
..gacggaauuccgaggaaau.............................................................................................  6   0   S04
..gacggaauuccgaggaaaug............................................................................................  1   0   S04
..gacggaauuccgaggaaauga...........................................................................................  1   0   S04
..gacggaauuccAaggaaauga...........................................................................................  1   1   S04
..gacggaauuccgaggaaaugaa..........................................................................................  1   0   S04
..gacggaauuccgaggaaaugaau.........................................................................................  3   0   S04
..gacggaauuccgaggaaaugaaC.........................................................................................  2   1   S04
..gacggaauuccgaggaaaugaauC........................................................................................  2   1   S04
..Aacggaauuccgaggaaaugaauu........................................................................................  1   1   S04
..gacggaauuccgaggaaaugaauu........................................................................................  39  0   S04
..gacggGauuccgaggaaaugaauu........................................................................................  1   1   S04
..gaUggaauuccgaggaaaugaauu........................................................................................  1   1   S04
...acggaauuccgaggaaaugaau.........................................................................................  1   0   S04
...acggaauuccgaggaaaugaauu........................................................................................  4   0   S04
...acggaauuccgaggaaaugaauuC.......................................................................................  1   1   S04
...acggaauuccUaggaaaugaauuu.......................................................................................  1   1   S04
...acggaauuccgaggaaaugaauuG.......................................................................................  1   1   S04
...acggaauuccgaggaaaugaauuu.......................................................................................  4   0   S04
....cggaauuccgaggaaaugaauuu.......................................................................................  2   0   S04
.......aauuccgaggaaaugaauuuc......................................................................................  1   0   S04
.......aauuccgaggaaaugaauuuccga...................................................................................  1   0   S04
........auuccgaggaaaugaauuucc.....................................................................................  2   0   S04
.................................aaacuccgaggaccaccaguucgu.........................................................  2   0   S04
.........................................aggaccaccaguucgucggacagA.................................................  1   1   S04
...........................................................gaGagguccucggaauauaccg.................................  1   1   S04
...........................................................gaUagguccucggaauauaccg.................................  1   1   S04
......................................................................ggaauauaccgagggagcacuucU....................  1   1   S04
```

Provisional ID	:	chrA06_523926
Score total	:	0.1
Score for star read(s)	:	3.9
Score for read counts	:	-4.9
Score for mfe	:	1.2
Score for randfold	:	0
Score for cons. seed	:	
Total read count	:	2
Mature read count	:	1
Loop read count	:	0
Star read count	:	1

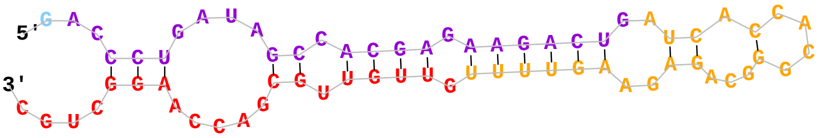

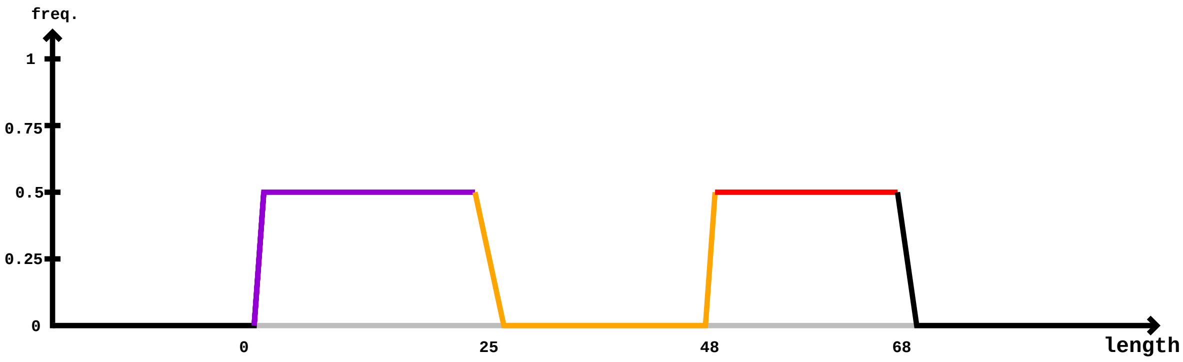

Star Mature

```
5'- acagcgcaaauuuggcagcgacccugauagccacgagaagacugaucaccacgggcagagaaguuuuguuguugcgaccaaggcugcuguugguuacgauuguuguuguu   -3'    obs
    acagcgcaaauuuggcagcgacccugauagccacgagaagacugaucaccacgggcagagaaguuuuguuguugcgaccaaggcugcuguugguuacgauuguuguuguu          exp
    ((((((((..(..((((((.((.((...((.((((.((((((..((.(....)...))..)))))).)))).))...)).)).))))))..)......))).))))))....   reads      mm         sample
    ....................acccugauagccacgagaagacuA..................................................................   1          1          S04
    ...................................................................guuguugcgaccaaggcuUc.......................   1          1          S04
```

Provisional ID	:	chrA06_527292
Score total	:	2.4
Score for star read(s)	:	3.9
Score for read counts	:	-3.9
Score for mfe	:	2.5
Score for randfold	:	0
Score for cons. seed	:	
Total read count	:	4
Mature read count	:	3
Loop read count	:	0
Star read count	:	1

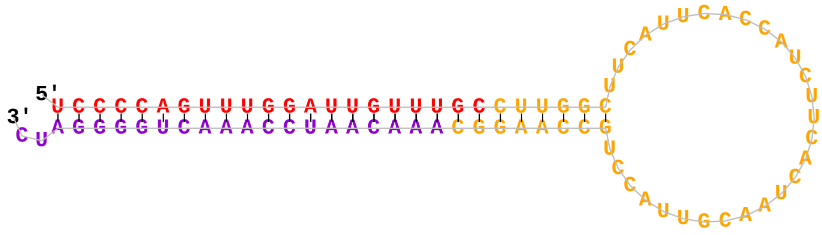

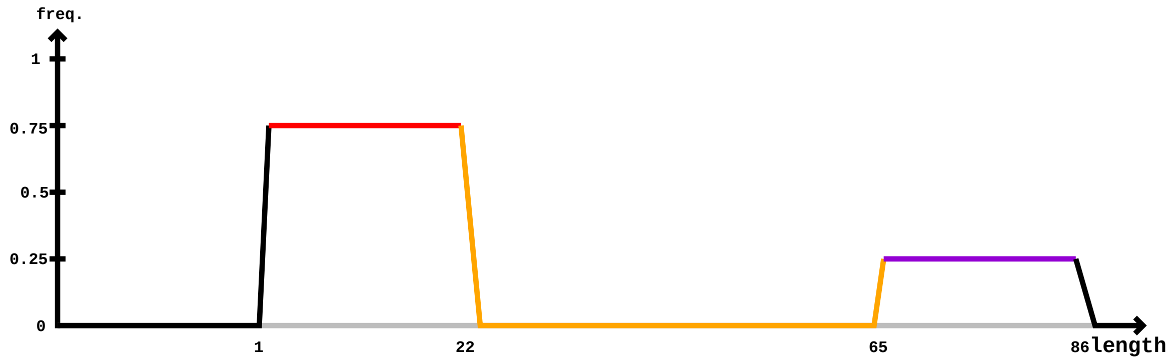


```
                           Mature                                                                Star
5'- uuauaucucucucucuaugauccccaguuuggauuguuugccuuggcuucauucaccaucuucacuaacguuaccugccaaggcaaacaauccaaacuggggaucuuagaa   -3'   obs
    uuauaucucucucucuaugauccccaguuuggauuguuugccuuggcuucauucaccaucuucacuaacguuaccugccaaggcaaacaauccaaacuggggaucuuagaa         exp
    ............((((.(((((((((((((((((((((((((((((..............................))))))))))))))))))))))))))))).)))).   reads   mm   sample
    ....................uccccaguuuggauuguuugc......................................................................   1       0    S03
    ....................................................................................aaacaauccaaacuggggauc......   1       0    S03

    ....................uccccaguuuggauuguuugc......................................................................   2       0    S04
```

Provisional ID	:	chrA06_528202
Score total	:	70.4
Score for star read(s)	:	3.9
Score for read counts	:	64.9
Score for mfe	:	1.6
Score for randfold	:	0
Score for cons. seed	:	
Total read count	:	139
Mature read count	:	138
Loop read count	:	0
Star read count	:	1

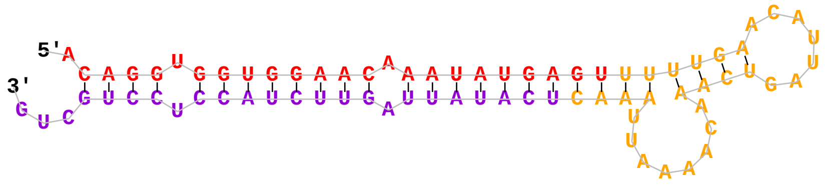

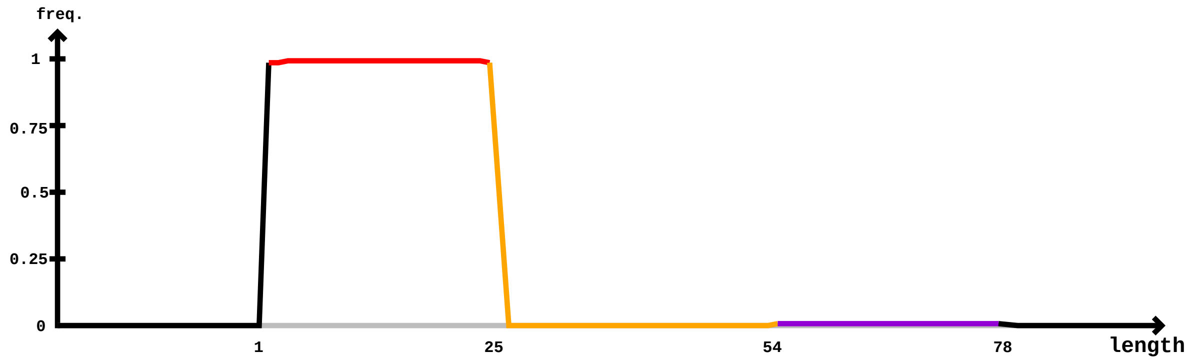


```
                    Mature                                               Star
5'- uuaagcuuaaguguguauugacaggugguggaacaaauaugaguuuuugaacauuagucaaacaaaauuaaacucauauuaguucuaccuccugcugaagcccacauuaaucu  -3'   obs
    uuaagcuuaaguguguauugacaggugguggaacaaauaugaguuuuugaacauuagucaaacaaaauuaaacucauauuaguucuaccuccugcugaagcccacauuaaucu         exp
    ...((.(((((((.((.((..((((.((((((((.(((((((((((((((.......))))........))))))))))).)))))))).))))..)).)).))).)))).))   reads   mm   sample
    ....................acaggugguggaacaaauaugagu.....................................................................   4       0    S03

    ....................acaggugguggaacaaauaugag......................................................................   1       0    S04
    ....................acagguggCggaacaaauaugagu.....................................................................   1       1    S04
    ....................acaggugguggaacaaauaugagA.....................................................................   3       1    S04
    ....................aUaggugguggaacaaauaugagu.....................................................................   1       1    S04
    ....................Ucaggugguggaacaaauaugagu.....................................................................   1       1    S04
    ....................acaggugguggaacaaauaugagu.....................................................................   116     0    S04
    ....................acaggugguggaaAaaauaugagu.....................................................................   1       1    S04
    ....................acaggugguggaacaaauaugagC.....................................................................   6       1    S04
    ....................acaggugguggaacaaauaugagG.....................................................................   3       1    S04
    ......................aggugguggaacaaauaGgagu.....................................................................   1       1    S04
    .........................................................................ucauauuaguucuaccuccugcuU................   1       1    S04
```

Provisional ID	:	chrA06_546717
Score total	:	155.2
Score for star read(s)	:	3.9
Score for read counts	:	151
Score for mfe	:	0.3
Score for randfold	:	0
Score for cons. seed	:	
Total read count	:	308
Mature read count	:	290
Loop read count	:	0
Star read count	:	18

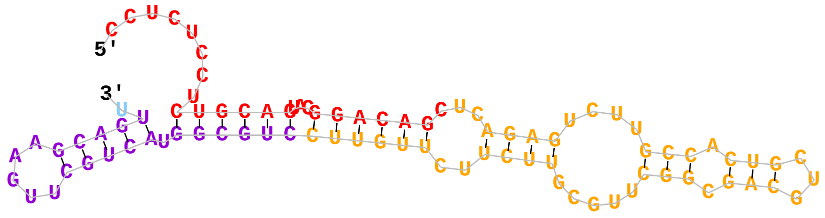

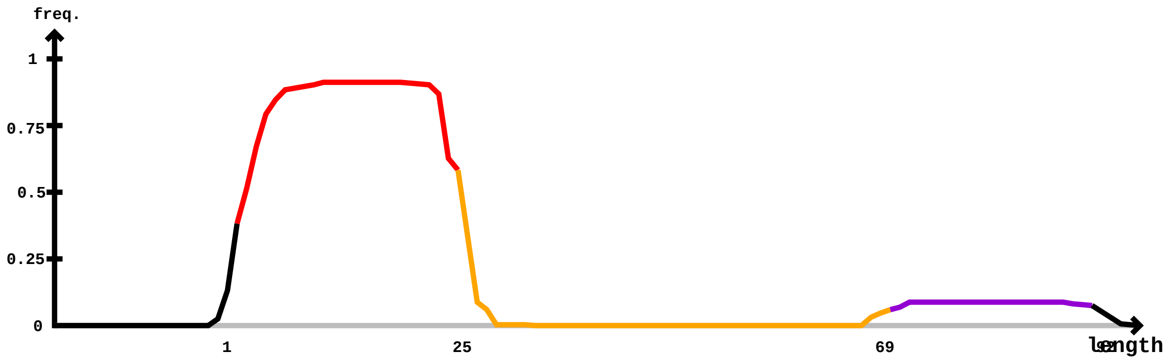

Mature **Star**

```
5'- cuucaucgucccgucaaccucuccucugcaguacggacagcucagagucuugccacugcugcagcggcuugcguucuucuuguuccugcgguacugcuugaagcaguugga   -3'    obs
    cuucaucgucccgucaaccucuccucugcaguacggacagcucagagucuugccacugcugcagcggcuugcguucuucuuguuccugcgguacugcuugaagcaguugga          exp
    .............(((((..((.(...(((((((.(.(((..(((((...(((..((((....))))...)))...))).))...)))).)))))))..).))..))))).   reads   mm   sample
    ...............aaccucuccucugcaguacggaca........................................................................   2       0    S03
    ................accucuccucugcaguacggac.........................................................................   1       0    S03
    ................accucuccucugcaguacggaU.........................................................................   1       1    S03
    ................accucuccucugcaguacggaca........................................................................   5       0    S03
    ................accucuccucugcaguacggacG........................................................................   2       1    S03
    ................accucuccucugcaguacggacagU......................................................................   1       1    S03
    .................ccucuccucugcaguacggaca........................................................................   7       0    S03
    .................ccucuccucugcaguacggacaA.......................................................................   1       1    S03
    .................ccucuccucugcaguacggacagU.....................................................................   13      1    S03
    .................ccucuccucugcaguacggacagUu....................................................................   1       1    S03
    ..................cucuccucugcaguacgg...........................................................................   1       0    S03
    ..................cucuccucugcaguacggaca........................................................................   4       0    S03
    ..................cucuccucugcaguacggacag.......................................................................   1       0    S03
    ..................cucuccucugcaguacggacagU......................................................................   3       1    S03
    ..................cucuccucugcaguacggacagUu.....................................................................   4       1    S03
    ...................ucuccucugcaguacggaca........................................................................   3       0    S03
    ...................ucuccucugcaguacggacag.......................................................................   3       0    S03
    ...................ucuccucugcaguacggacagc......................................................................   2       0    S03
    ...................ucuccucugcaguacggacagU......................................................................   8       1    S03
    ...................ucuccucugcaguaAggacagc......................................................................   1       1    S03
    ...................ucuccucugcaguacggacagUu.....................................................................   2       1    S03
    ....................cuccucugcaguacggUca........................................................................   1       1    S03
    ....................cuccucugcaguacggaca........................................................................   1       0    S03
    ....................cuccucugcaguacggacagc......................................................................   1       0    S03
    ....................cuccucugcaguacggacagG......................................................................   1       1    S03
    ....................cuccucugcaguaAggacagc......................................................................   1       1    S03
    ....................cuccucugcaguacggacagU......................................................................   3       1    S03
    ....................cuccucugcaguacggacagUu.....................................................................   6       1    S03
    .....................uccucugcaguacggacagUu.....................................................................   1       1    S03
    .....................uccucugcaguacggacagUuc....................................................................   1       1    S03
    .....................uccucugcaguacggacagUuca...................................................................   2       1    S03
    ......................ccucugcaguacggacagU......................................................................   1       1    S03
    ......................ccucugcaguacggacagA......................................................................   1       1    S03
```

```
                 Mature                                                Star
cuucaucgucccgucaaccucuccucugcaguacggacagcucagagucuugccacugcugcagcggcuugcguucuucuuguuccugcgguacugcuugaagcaguugga
......................ccucugcaguacggacagUuca...................................................................	1	1	S03
........................ucugcaguacggacagcucagagG...............................................................	1	1	S03
..........................ugcaguacggacagUuca...................................................................	2	1	S03
...................................................................................uccugcgguacugcuugaaAc.......	1	1	S03
...................................................................................uccugcgguacugcuugaaAcagu....	2	1	S03
...................................................................................ucUugcgguacugcuugaagcagu....	2	1	S03
....................................................................................ccugcgguacugcuugaaAcagu....	1	1	S03
....................................................................................ccugcgguacugcuugaaAcaguu...	1	1	S03
.....................................................................................Uugcgguacugcuugaagcagu....	2	1	S03
.....................................................................................cugcgguacugcuugaaAcagu....	1	1	S03
......................................................................................ugcgguacugcuugaagcaguug..	1	0	S03
.......................................................................................gcgguacugcuugaaUcaguu...	1	1	S03
.......................................................................................gcgguacugcuugaagcaguug..	1	0	S03
.......................................................................................gcgguacugcuugaagcaguugg.	1	0	S03

...............aaccucuccucugcaguacgga..........................................................................	1	0	S04
...............aaccucuccucugcaguacggac.........................................................................	2	0	S04
...............aaccucuccucugcaguacggacG........................................................................	1	1	S04
...............aaccucuccucugcaguacggaca........................................................................	2	0	S04
................accucuccucugcaguacggac.........................................................................	5	0	S04
................accucuccucugcaguacggacG........................................................................	1	1	S04
................accucuccucugcaguacggaca........................................................................	12	0	S04
................accucuccucugcaguacggacU........................................................................	1	1	S04
................accucuccucugcaguacggacag.......................................................................	4	0	S04
................Nccucuccucugcaguacggacag.......................................................................	1	1	S04
.................ccucuccucugcaguacg............................................................................	1	0	S04
.................ccucuccucugcaguacggac.........................................................................	1	0	S04
.................ccucuccucugcaguacggaca........................................................................	14	0	S04
.................ccucuccucugcaguacggacG........................................................................	1	1	S04
.................ccucuccucugcaguacggacag.......................................................................	1	0	S04
.................ccucuccucugcaguacggacagG......................................................................	3	1	S04
.................ccucuccucugcaguacggacagU......................................................................	32	1	S04
.................ccucuccucugcaguacggacagc......................................................................	5	0	S04
..................cucuccucugcaguacggac.........................................................................	1	0	S04
..................cucuccucugcaguacggaca........................................................................	8	0	S04
..................cucuccucugcaguacggacag.......................................................................	1	0	S04
..................cucuccucugcaguacggacagG......................................................................	2	1	S04
..................cucuccucugcaguacggacagc......................................................................	2	0	S04
..................cucuccucugcaguacggacagU......................................................................	9	1	S04
..................cucuccucugcaguacggacaAc......................................................................	1	1	S04
..................cucuccucugcaguacggacagUu.....................................................................	5	1	S04
...................ucuccucugcaguacggaca........................................................................	3	0	S04
...................ucuccucugcaguacggacag.......................................................................	1	0	S04
...................ucuccucugcaguacggacagU......................................................................	18	1	S04
...................ucuccucugcaguacggacagG......................................................................	2	1	S04
...................Ccuccucugcaguacggacagc......................................................................	1	1	S04
...................ucuccucugcaguacggacagUu.....................................................................	5	1	S04
...................ucuccucugcaguaAggacagcuc....................................................................	1	1	S04
....................cuccucugcaguacggaca........................................................................	7	0	S04
....................cuccucugcaAuacggaca........................................................................	1	1	S04
....................cuccucugcaguacggacag.......................................................................	1	0	S04
....................cuccucugcaguacggacagc......................................................................	1	0	S04
....................cuccucuAcaguacggacagc......................................................................	1	1	S04
....................cuccucugcaguacggacagG......................................................................	1	1	S04
....................cuccucugcaguacggacagU......................................................................	7	1	S04
....................cuccucugcaguacggacagUu.....................................................................	4	1	S04
....................cuccucugcaguacggacagUuca...................................................................	2	1	S04
.....................uccucugcaguacggaca........................................................................	1	0	S04
.....................uccucugcaguacggacagU......................................................................	1	1	S04
.....................uccucugcaguacggacagUu.....................................................................	4	1	S04
.....................uccucugcaguacggacagUuc....................................................................	6	1	S04
.....................uccucugcaguacggacagUuca...................................................................	1	1	S04
......................ccucugcaguacggacagU......................................................................	2	1	S04
......................ccucugcaguacggacagUu.....................................................................	1	1	S04
......................ccucugcaguacggacagUuca...................................................................	6	1	S04
.......................cucugcaguacggacagUuc....................................................................	1	1	S04
.......................cucugcaguacggacagUuca...................................................................	1	1	S04
........................ucugcaguacggacagUu.....................................................................	1	1	S04
.........................cugcaguacggacagUuca...................................................................	2	1	S04
..........................ugcaguacggacagUuca...................................................................	1	1	S04
```

Mature Star

```
cuucaucgucccgucaaccucuccucugcaguacggacagcucagagucuugccacugcugcagcggcuugcguucuucuuguuccugcgguacugcuugaagcaguugga
...................................................................................uccugcgguacugcuugaaAc.......	1	1	S04
...................................................................................ucUugcgguacugcuugaagcagu....	1	1	S04
...................................................................................uccugcgguacugcuugaaAcagu....	3	1	S04
....................................................................................cUugcgguacugcuugaagca......	1	1	S04
....................................................................................ccugcgguacugcuugaaAcag.....	1	1	S04
....................................................................................ccugcgguacugcuugaaAcagu....	1	1	S04
.....................................................................................cugcgguacugcuugaaAcagu....	1	1	S04
......................................................................................ugcggCacugcuugaagcagu....	1	1	S04
......................................................................................ugcgguacugcuugaagcagu....	1	0	S04
.......................................................................................gcgguacugcuugaagcagu....	1	0	S04
.......................................................................................gcgguacugcuugaagcaguu...	1	0	S04
.......................................................................................gcgguacugcuugaagcaguugga	1	0	S04
```

Provisional ID	:	chrA06_563043
Score total	:	1.3
Score for star read(s)	:	-1.3
Score for read counts	:	0
Score for mfe	:	2.6
Score for randfold	:	0
Score for cons. seed	:	
Total read count	:	41
Mature read count	:	41
Loop read count	:	0
Star read count	:	0

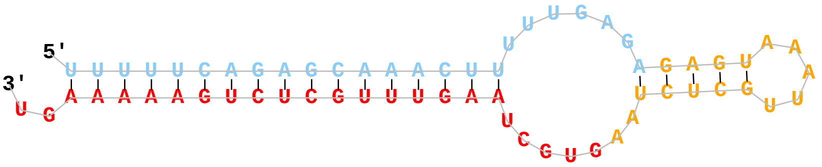

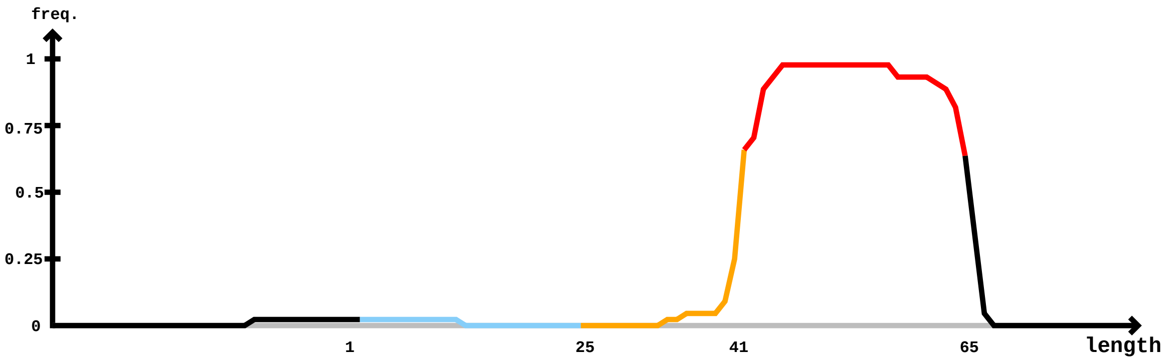

Star Mature

```
5'- ggaguccuaggcgagaggcaugagagagacuuuuucagagcaaacuuuuugagagaguaaaauugcucuaagugcuaaguuugcucugaaaaaguaucucucaugccucucg   -3'    exp
    ............((((((((((((((((.(((((((((((((((((((......(((((.....))))).......))))))))))))))))))).))))))))))))))))   reads   mm   sample
    ...................augagagagacuuuuucagagU.......................................................................   1       1    S03
    .................................................................cucuaagugcuaaguuugcucuA........................   1       1    S03
    .....................................................................aagugcuaaguGugcucugaaaa....................   1       1    S03
    .....................................................................aagugcuaaguuugcucugaaaaag..................   1       0    S03
    ......................................................................agugcuaaguuugcucugaaaaa...................   1       0    S03
    ......................................................................agugcuaaguGugcucugaaaaa...................   1       1    S03
    ......................................................................agugcuaaguuugcucugaaaaag..................   3       0    S03
    .......................................................................gugcuaaguuugcucugaaaaag..................   1       0    S03
    .......................................................................gugcuaaguuugcucugaaaaagu.................   10      0    S03
    .......................................................................gugcuaaguuugcucugaGaaagu.................   1       1    S03
    ........................................................................ugcuaaguuugcucugaaaaagu.................   2       0    S03
    .........................................................................gcuaaguuugcucugaaG.....................   1       1    S03
    .........................................................................gcuaaguuugcucugaaaaa...................   1       0    S03
    .........................................................................gcuaaguuugcucugaaaaag..................   1       0    S03
    .........................................................................gcuaaguuugcucugaaaaagu.................   3       0    S03
    .........................................................................gcuaaguuugcucugaaaaaguUu...............   1       1    S03
    ..........................................................................cuaaguuugcucugaaaaagu.................   1       0    S03
    ...........................................................................uaaguuugcucugaaaaagu.................   1       0    S03
    ...........................................................................uaaguuugcucugaaaaaguUu...............   1       1    S03

    ...............................................................ugcucuaagugcuaaguuugcucuA........................   1       1    S04
    ......................................................................agugcuaaguuugcucugaaaaag..................   2       0    S04
    .......................................................................gugcuaaguuugcucugaGaaagu.................   1       1    S04
    .......................................................................gugAuaaguuugcucugaaaaagu.................   1       1    S04
    .......................................................................gugcuaaguuugcucugaaaaagG.................   2       1    S04
    .......................................................................gugcuaaguuugcucugaaaaagu.................   2       0    S04
    .........................................................................gcuaaguuugcucugaaaaagu.................   1       0    S04
    ..........................................................................cuaaguuugcucugaaaaagA.................   1       1    S04
```

Provisional ID : chrA07_600694
Score total : 0.2
Score for star read(s) : -1.3
Score for read counts : 0
Score for mfe : 1.5
Score for randfold : 0
Score for cons. seed :
Total read count : 69
Mature read count : 69
Loop read count : 0
Star read count : 0

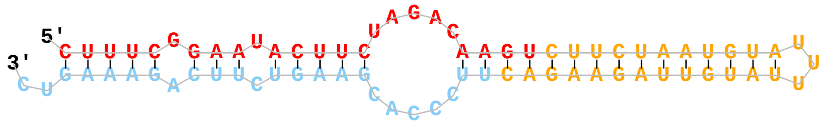

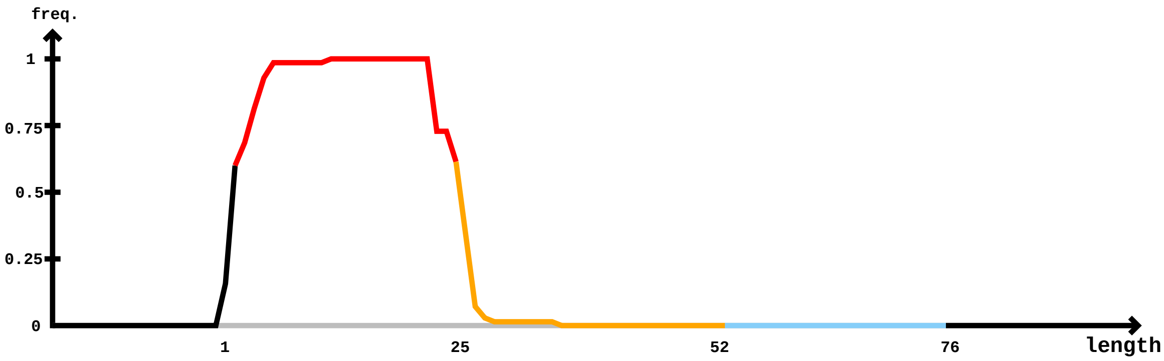

Mature Star

```
5'- aaagacuucucagaauacuuucggaauacuucuagacaagucuucuaauguauuuuauguuagaagacuucccacgaagucuucagaaagucuucugccuaaagugguaca   -3'   exp
    ....((((..(((((.((((((.(((.(((((.....(((((((((((((((...))))))))))))))).....))))).))).)))))).)))))....))))......   reads   mm   sample
    ................acuCucggaauacuucuagaca.........................................................................   3       1    S03
    ................acuCucggaauacuucuagacaag.......................................................................   7       1    S03
    ................acuCucggaauacuucuagacaagu......................................................................   1       1    S03
    .................cuCucggaauacuucuagaca.........................................................................   10      1    S03
    .................cuCucggaauacuucuagacaag.......................................................................   1       1    S03
    .................cuCucggaauacuucuagacaagu......................................................................   18      1    S03
    ..................uCucggaauacuucuagaca.........................................................................   3       1    S03
    ..................uCucggaauacuucuagacaagu......................................................................   2       1    S03
    ..................uCucggaauacuucuagacaaguc.....................................................................   1       1    S03
    ...................Cucggaauacuucuagaca.........................................................................   2       1    S03
    ...................Cucggaauacuucuagacaagu......................................................................   6       1    S03
    ...................Cucggaauacuucuagacaagucu....................................................................   1       1    S03
    ....................ucggaauacuucuagaca.........................................................................   1       0    S03
    ....................ucggaauacuucuagacaagu......................................................................   4       0    S03
    ....................ucggaauacuucuagacaaguc.....................................................................   1       0    S03
    ....................ucggaauacuucuagacaagucu....................................................................   1       0    S03
    ....................ucggaauacuucuagacaagucuC...................................................................   1       1    S03
    .....................cggaauacuucuagacaagu......................................................................   1       0    S03
    .....................cggaauacuucuagacaaguc.....................................................................   2       0    S03
    .....................cggaauacuucuagacaagucu....................................................................   1       0    S03
    ...........................acuucuagacaagucCucuaaugu............................................................   1       1    S03

    .................cuCucggaauacuucuagacaagu......................................................................   2       1    S04
```

Provisional ID	:	chrA07_603214
Score total	:	3652.7
Score for star read(s)	:	3.9
Score for read counts	:	3661.2
Score for mfe	:	-12.3
Score for randfold	:	0
Score for cons. seed	:	
Total read count	:	7193
Mature read count	:	7192
Loop read count	:	0
Star read count	:	1

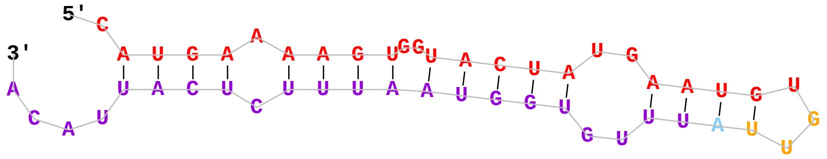

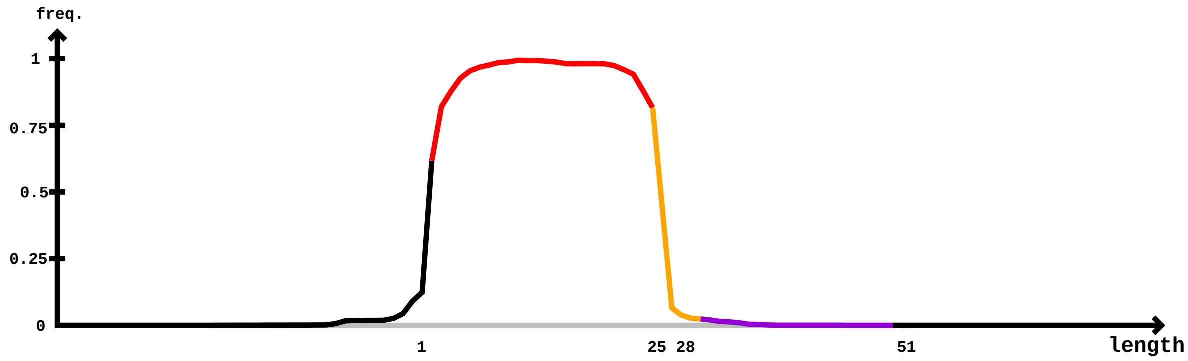

Mature **Star**

```
5'- ccuucauuaaaaauuaggguaaaaauauuaguguaaacaugaaaagugguacuaugaauguguuauuugugguaauuucucauuacauaggggguuguuucuuuuuaucacaa   -3'   obs
    ccuucauuaaaaauuaggguaaaaauauuaguguaaacaugaaaagugguacuaugaauguguuauuugugguaauuucucauuacauaggggguuguuucuuuuuaucacaa         exp
    .................(((((((......(((((...((((.((((..(((((..((((...))))..))))))))).)))))))))..((((....)))))))))))....   reads   mm   sample
    .........................uauuaguguaaacaugaaaagugg................................................................   1       0    S03
    ..........................auuaguguaaacaugaaaaguggu...............................................................   1       0    S03
    ...........................uuaguguaaacaugaaaagu..................................................................   1       0    S03
    ...........................uuaguguaaacaugaaaagug.................................................................   1       0    S03
    ...........................uuaguguaaacaugaaaagugg................................................................   12      0    S03
    ...........................uuaguguaaacaugaaaaguggu...............................................................   5       0    S03
    ...........................uuaguguaaacaugaaaaguggua..............................................................   4       0    S03
    ............................uaguguaaacaugaaaagu..................................................................   6       0    S03
    ............................uaguguaaacaugaaaagug.................................................................   2       0    S03
    ............................uaguguaaacaugaaaagugg................................................................   1       0    S03
    ............................uaguguaaacaugaaaaguggG...............................................................   1       1    S03
    ............................uaguguaaacaugaaaaguggu...............................................................   7       0    S03
    ............................uaguguaaacaugaaaaguggA...............................................................   1       1    S03
    ............................uaguguaaacaugaaaaguggua..............................................................   21      0    S03
    ............................uaguguaaacaugaaaagugguac.............................................................   2       0    S03
    .............................aguguaaacaugaaaagu..................................................................   2       0    S03
    .............................aguguaaacaugaaaagug.................................................................   2       0    S03
    .............................aguguaaacaugaaaaguggu...............................................................   3       0    S03
    .............................aguguaaacaugaaaaguggua..............................................................   1       0    S03
    ..............................guguaaacaugaaaagug.................................................................   1       0    S03
    ..............................guguaaaAaugaaaagugguacu............................................................   1       1    S03
    ................................guaaacaugaaaagugguacuaug.........................................................   4       0    S03
    .................................uaaacaugaaaagugguacua...........................................................   1       0    S03
    .................................uaaacaugaaaagugguacuaug.........................................................   6       0    S03
    .................................uaaacaugaaaagugguacuauga........................................................   9       0    S03
    .................................Aaaacaugaaaagugguacuauga........................................................   5       1    S03
    .................................uCaacaugaaaagugguacuauga........................................................   1       1    S03
    .................................uaaacaugaaaagugguacuaugaa.......................................................   1       0    S03
    .................................Aaaacaugaaaagugguacuaugaa.......................................................   1       1    S03
    .................................uaaacaugaaaagugguacuaugaau......................................................   3       0    S03
    ..................................aaacaugaaaagugguacuaug.........................................................   9       0    S03
    ..................................aaacaugaaaagugguacuauga........................................................   10      0    S03
    ..................................aaacaugaaaagugguacuaugaG.......................................................   2       1    S03
```

Mature **Star**

```
ccucauuaaaaauuaggguaaaaauauuaguguaaacaugaaaagugguacuaugaauguguuauuugugguaauuucucauuacauagggguuguuucuuuuuaucacaa
.................................aaacaugaaaagugguacuaugaa......................................................  32     0    S03
.................................aaacaugCaaagugguacuaugaa......................................................  2      1    S03
.................................aaacaugaaaagugguacuaugaC......................................................  2      1    S03
.................................aCacaugaaaagugguacuaugaau.....................................................  1      1    S03
.................................aaacaugaaaagugguacuaugaau.....................................................  6      0    S03
.................................aaacaugaaaagugguacuaugaGu.....................................................  1      1    S03
.................................aaacaugaaaagugguacuaugaaA.....................................................  1      1    S03
.................................aaacaugaaaagugguacuaugaauC....................................................  1      1    S03
..................................aacaugaaaagugguacuaug........................................................  1      0    S03
..................................aacaugaaaagugguacuauga.......................................................  11     0    S03
..................................aacaugaaaagugguacuaugaa......................................................  11     0    S03
..................................aacaugaaaagugguacuaugaG......................................................  1      1    S03
..................................aacaugCaaagugguacuaugaa......................................................  3      1    S03
..................................aacaugaaaagugguacuaugaau.....................................................  106    0    S03
..................................aacaugCaaagugguacuaugaau.....................................................  1      1    S03
..................................aacaugaaaagugguacuaugaGu.....................................................  3      1    S03
..................................aaAaugaaaagugguacuaugaau.....................................................  1      1    S03
..................................aacaugaaaagugguacuaugaaG.....................................................  3      1    S03
..................................aacaugaaaagugguacuaugaaC.....................................................  10     1    S03
..................................aacaugaaaagugguacuaugaaA.....................................................  4      1    S03
..................................aacaugaGaagugguacuaugaau.....................................................  1      1    S03
..................................aaAaugaaaagugguacuaugaaug....................................................  1      1    S03
..................................aacaugaaaagugguacuaugaaug....................................................  3      0    S03
...................................acaugaaaagugguacuau.........................................................  1      0    S03
...................................acaugaaaagugguacuaug........................................................  2      0    S03
...................................acaugaaaagugguacuauga.......................................................  1      0    S03
...................................acaugaaaagugguacuaugaa......................................................  2      0    S03
...................................acaugaaaagugguacuaugaG......................................................  1      1    S03
...................................acaugaaaagugguacuaugaau.....................................................  5      0    S03
...................................acaugaaaagugguGcuaugaau.....................................................  1      1    S03
...................................acaCgaaaagugguacuaugaaug....................................................  2      1    S03
...................................acaugaaaagGgguacuaugaaug....................................................  1      1    S03
...................................aAaugaaaagugguacuaugaaug....................................................  1      1    S03
...................................acaugaaaagugguacuaugaUug....................................................  1      1    S03
...................................acaugaaaagugguacuaugaaug....................................................  107    0    S03
...................................acaugaaaGgugguacuaugaaug....................................................  2      1    S03
....................................caugaaaagugguacuaug........................................................  2      0    S03
....................................caugaaaagugguacuauga.......................................................  9      0    S03
....................................caugaaaagugguacuaugaa......................................................  3      0    S03
....................................caugaaaagugguacuaugaaG.....................................................  1      1    S03
....................................caugaaaagugguacuaugaau.....................................................  30     0    S03
....................................caugaaaagugguUcuaugaau.....................................................  1      1    S03
....................................caugaaaagugguacuaugaGu.....................................................  1      1    S03
....................................caugaaaagugguacuaugaaC.....................................................  5      1    S03
....................................caugaaaagugguacuaugaauC....................................................  3      1    S03
....................................caugaaaagugguGcuaugaaug....................................................  1      1    S03
....................................caugaaaGgugguacuaugaaug....................................................  1      1    S03
....................................caugaaaagugguacuaugaaug....................................................  79     0    S03
....................................caugaaaagugguacuaugaaugA...................................................  55     1    S03
....................................caugaaaagGgguacuaugaaugu...................................................  7      1    S03
....................................Aaugaaaagugguacuaugaaugu...................................................  5      1    S03
....................................caCgaaaagugguacuaugaaugu...................................................  8      1    S03
....................................caugaUaagugguacuaugaaugu...................................................  1      1    S03
....................................caugaaaagugguacuaugaGugu...................................................  3      1    S03
....................................caGgaaaagugguacuaugaaugu...................................................  1      1    S03
....................................Naugaaaagugguacuaugaaugu...................................................  4      1    S03
....................................caugaaaagugguacuaugaaugG...................................................  77     1    S03
....................................caugaaaagugguacuaugaaugu...................................................  1423   0    S03
....................................caugaaaagugguacuaugaCugu...................................................  1      1    S03
....................................caugCaaagugguacuaugaaugu...................................................  15     1    S03
....................................caugaaaagugguacuaugaauCu...................................................  1      1    S03
....................................caugaaaGgugguacuaugaaugu...................................................  6      1    S03
....................................caugaaaagugguGcuaugaaugu...................................................  2      1    S03
....................................caugaGaagugguacuaugaaugu...................................................  6      1    S03
....................................caugaaaagugguCcuaugaaugu...................................................  2      1    S03
....................................caugaaaagugguacuaugaauguU..................................................  3      1    S03
....................................caugaaaagugguacuaugaauguC..................................................  1      1    S03
....................................caugaaaGgugguacuaugaaugug..................................................  1      1    S03
....................................caugaaaagugguacuaugaaugug..................................................  95     0    S03
....................................caugaaaagugguacuaugGaugugu.................................................  1      1    S03
```

```
                                     Mature                  Star
ccuucauuaaaaauuaggguaaaaauauuaguguaaacaugaaaagugguacuaugaauguguuauuugugguaauuucucauuacauagggguuguuucuuuuuaucacaa
.....................................cUugaaaagugguacuaugaaugugu.................................................  2    1  S03
.....................................Gaugaaaagugguacuaugaaugugu.................................................  1    1  S03
.....................................caugaaaagugguacuaugaaugugu.................................................  2    0  S03
.....................................caugaaaagugguacuaugaauguguu................................................  1    0  S03
......................................augaaaagugguacuaug........................................................  1    0  S03
......................................augaaaagugguacuauga.......................................................  2    0  S03
......................................augaaaagugguacuaugaCu.....................................................  1    1  S03
......................................augaaaagugguacuaugaau.....................................................  9    0  S03
......................................augaaaagugguacuaugaauC....................................................  2    1  S03
......................................augaaaagugguacuaugaaug....................................................  37   0  S03
......................................augaaaagugguacuaugaaugA...................................................  3    1  S03
......................................augaaaGgugguacuaugaaugu...................................................  1    1  S03
......................................augaaaagugguacuaugaaugu...................................................  60   0  S03
......................................augaaaagugguacuaugaaugG...................................................  2    1  S03
......................................augaaaagugguacuaugaaugGg..................................................  2    1  S03
......................................augaaaGgugguacuaugaaugug..................................................  5    1  S03
......................................augCaaagugguacuaugaaugug..................................................  14   1  S03
......................................augaaaagugguacuaugaauguU..................................................  9    1  S03
......................................augaaaagugguacuaGgaaugug..................................................  1    1  S03
......................................Nugaaaagugguacuaugaaugug..................................................  3    1  S03
......................................augaaaagugguacuaugaaugug..................................................  573  0  S03
......................................augaaaagugguacuaugUaugug..................................................  1    1  S03
......................................augaGaagugguacuaugaaugug..................................................  1    1  S03
......................................augaaaaguggGacuaugaaugug..................................................  2    1  S03
......................................augaUaagugguacuaugaaugug..................................................  1    1  S03
......................................augaaaagugguacuaugaGugug..................................................  1    1  S03
......................................augaaaagugguacuaugaaugAg..................................................  1    1  S03
......................................augaaaagugguacuaugaaugugu.................................................  4    0  S03
.......................................ugaaaagugguacuaugaau.....................................................  2    0  S03
.......................................ugaaaagugguacuaugaaug....................................................  14   0  S03
.......................................Ngaaaagugguacuaugaaugu...................................................  1    1  S03
.......................................ugaaaagugguacuaugaaugG...................................................  1    1  S03
.......................................ugaaaGgugguacuaugaaugu...................................................  2    1  S03
.......................................ugaaaagugguacuaugaaugA...................................................  1    1  S03
.......................................ugaaaagugguacuaugaGugu...................................................  1    1  S03
.......................................ugCaaagugguacuaugaaugu...................................................  3    1  S03
.......................................ugaaaagugguacuaugaaugu...................................................  71   0  S03
.......................................ugaaaagugguacuaugaauguU..................................................  4    1  S03
.......................................ugaGaagugguacuaugaaugug..................................................  2    1  S03
.......................................ugaaaagugguacuaugaaugug..................................................  91   0  S03
.......................................ugaaaagCgguacuaugaaugugu.................................................  1    1  S03
.......................................ugaaaagugguacCaugaaugugu.................................................  1    1  S03
.......................................ugaaaagugguacuaugaaugugu.................................................  28   0  S03
.......................................ugaaaagugguacuaugaauguAu.................................................  1    1  S03
.......................................ugaaaagugguacuaugaaugGgu.................................................  1    1  S03
.......................................ugaaaagugguacuaugaaugAgu.................................................  1    1  S03
........................................gaaaagugguacuaugaa......................................................  2    0  S03
........................................gaaaagugguacuaugaau.....................................................  9    0  S03
........................................gaaaagugguacuaugaaug....................................................  18   0  S03
........................................gaaaagugguacuaugaUug....................................................  1    1  S03
........................................gaaaagugguacuaugaaGgu...................................................  1    1  S03
........................................gaaaagugguacuaugaaugG...................................................  2    1  S03
........................................gaaaagugguacuaugaaugA...................................................  2    1  S03
........................................gaaaagugguacuaugaaugu...................................................  45   0  S03
........................................gaaaGgugguacuaugaaugu...................................................  1    1  S03
........................................gaaaagugguacuaugaauguU..................................................  4    1  S03
........................................gCaaagugguacuaugaaugug..................................................  3    1  S03
........................................gaaaagugguacuaugaaugug..................................................  59   0  S03
........................................gaaaagugguacuaugaaugugu.................................................  20   0  S03
........................................Aaaaagugguacuaugaaugugu.................................................  1    1  S03
........................................gaaaagugguacuaugaGugugu.................................................  1    1  S03
........................................gaaaagugguacuaugaaugAgu.................................................  1    1  S03
........................................Naaaagugguacuaugaaugugu.................................................  1    1  S03
........................................gaaaagugguacCaugaaugugu.................................................  1    1  S03
........................................gaaaagugguacuaugaaugCgu.................................................  1    1  S03
........................................Aaaaagugguacuaugaauguguu................................................  2    1  S03
........................................gaaaagAgguacuaugaauguguu................................................  1    1  S03
........................................gaaaGgugguacuaugaauguguu................................................  1    1  S03
........................................gaaaagugguacuaugGauguguu................................................  2    1  S03
........................................gaaaagugguacCaugaauguguu................................................  1    1  S03
```

Mature Star

ccuucauuaaaaauuagguaaaaauauuaguguaaacaugaaaagugguacuaugaauguguuauuugugguaauuucucauuacauaggggguuguuucuuuuuaucacaa

....gaaaagugguacuaugaauguguG...	2	1	S03
....gaaaagugguacuaugaauguguC...	1	1	S03
....gUaaagugguacuaugaauguguu...	1	1	S03
....gaaaagugguacuaugaauguguu...	26	0	S03
....gaaaagugguacuaugaauguguua..	1	0	S03
....aaaagugguacuaugaau..	1	0	S03
....aaaagugguacuaugaaug...	1	0	S03
....aaaagugguacuaugaaugA..	1	1	S03
....aaaagugguacuaugaaugu..	18	0	S03
....aaaagugguacuaugaaugG..	1	1	S03
....aaaagugguacuaugaaugug...	50	0	S03
....aGaagugguacuaugaaugug...	1	1	S03
....Caaagugguacuaugaaugug...	2	1	S03
....aaaagugguacuaugaauguU...	6	1	S03
....aaaagugguacuaugaauguUu..	2	1	S03
....aaaagugguacuaugaaugugu..	25	0	S03
....aaaagugguaAuaugaaugugu..	1	1	S03
....aaaagugguacCaugaaugugu..	1	1	S03
....Caaagugguacuaugaaugugu..	1	1	S03
....aaaaguggAacuaugaaugugu..	1	1	S03
....aaaagugguacuaugaauguguu...	7	0	S03
....aaaagugguacuaugaauguguC...	2	1	S03
....aaaagugguacuaugaauguguA...	1	1	S03
....aaaagugguacCaugaauguguu...	1	1	S03
....aaaagugguacuaugaauguguua..	3	0	S03
....aaagugguacuaugaaugu..	8	0	S03
....aaagugguacuaugaaugug...	16	0	S03
....aaagugguacuaugaaugugu..	11	0	S03
....aaagugguacuaugaauguguu...	7	0	S03
....aaagugguacuaugaauguguC...	2	1	S03
....aaagugguacuaugaauguguuG..	2	1	S03
....aaagugguacuauAaauguguua..	1	1	S03
....aaagugguacuaugaauguguuU..	3	1	S03
....aaagugguacuaugaauguguua..	7	0	S03
....aaagugguacuaugaauguguuau...	9	0	S03
....aaagugguacuaugaauguguuaC...	4	1	S03
....aaagugguacuaugaauguguuauA..	2	1	S03
....aagugguacuaugaaugu..	4	0	S03
....aagugguacuaugaaugA..	1	1	S03
....aagugguacuaugaaugug...	5	0	S03
....aagugguacuaugaaugugu..	5	0	S03
....aagugguacuaugaauguAu..	1	1	S03
....aagugguacuaugGaugugu..	1	1	S03
....aagugguacuaugaauguguC...	1	1	S03
....aagugguaAuaugaauguguu...	1	1	S03
....aagugguacuaugaauguguu...	5	0	S03
....aagugguacuaugaauguguua..	1	0	S03
....aagugguaAuaugaauguguua..	1	1	S03
....aagugguacuaugaauguguuaA...	1	1	S03
....aagugguacuaugaauguguuau...	4	0	S03
....aagugguacuaugaauguguuauA..	11	1	S03
....aagugguacuaugaauguguuauu..	5	0	S03
....aagugguaAuaugaauguguuauu..	1	1	S03
....agugguacuaugaaugug...	6	0	S03
....agugguacuaugaaugugu..	10	0	S03
....agugguacuaugaauguguu...	7	0	S03
....agugguacuaugaauguguG...	1	1	S03
....agugguacuaugGauguguuau...	1	1	S03
....agugguacuauAaauguguuau...	1	1	S03
....agugguacuaugaauguguuau...	6	0	S03
....agugguacuaugaauguguuaC...	1	1	S03
....agugguacuaugaauguguuauA..	11	1	S03
....agugguacuaugaauguguuUuu..	1	1	S03
....agugguacuaugaauguguuauG..	2	1	S03
....agugguacuaugaauguguuauAu...	13	1	S03
....agugguacuaugaauguguuauuu...	1	0	S03
....agugguacuaugaauguguuauAug..	1	1	S03
....gugguacuaugaaugugu..	2	0	S03
....gugguacuaugaauguguu...	1	0	S03
....gugguacuaugaauguguuauu..	1	0	S03

```
                                          Mature              Star
ccuucauuaaaaauuaggguaaaaauauuaguguaaacaugaaaagugguacuaugaauguguuauuugugguaauuucucauuacauaggggguuguuucuuuuuaucacaa
.............................................gugguacuaugaauguguuauAug............................................  11  1  S03
..............................................ugguacuaugaauguguu.................................................  1   0  S03
..............................................ugguacuaugaauguguuauA..............................................  1   1  S03
..............................................ugguacuaugaauguguuauCug............................................  3   1  S03
..............................................ugguacuaugaauguguuauAug............................................  6   1  S03
..............................................ugguacuaugaauguguuauCugu...........................................  26  1  S03
..............................................ugguacuaugaauguguuauAugu...........................................  9   1  S03
................................................guacuaugaauguguuauCug............................................  2   1  S03
................................................guacuaugaauguguuauCugu...........................................  3   1  S03
................................................guacuaugaauguguuauCugug..........................................  3   1  S03
.................................................uacuaugaauguguuauCug............................................  1   1  S03
.................................................uacuaugaauguguuauCugug..........................................  2   1  S03
.................................................uacuaugaauguguuauCugugg.........................................  2   1  S03
.................................................uacuaugaauguguuauAugugg.........................................  1   1  S03
.................................................uacuaugaauguguuauCuguggu........................................  2   1  S03
..................................................acuaugaauguguuauCugugg.........................................  8   1  S03
...................................................cuaugaauguguuauCugugg.........................................  1   1  S03
....................................................uaugaauguguuaAuuguggu........................................  3   1  S03
....................................................uaugaauguguuauCuguggu........................................  1   1  S03
.....................................................augaauguguuauCugug..........................................  1   1  S03
.....................................................augaauguguuauCugugg.........................................  1   1  S03
.....................................................augaauguguuaAuuguggu........................................  1   1  S03
......................................................ugaauguguuaAuuguggu........................................  1   1  S03
.........................................................augugCuauuugugguaauuucu.................................  1   1  S03

..............Gaggguaaaaauauuaguguaaac...........................................................................  1   1  S04
...............aggguaaaaauauuagugua..............................................................................  1   0  S04
.................gguaaaaauauuaguguaaacaug........................................................................  1   0  S04
...................Aaaaaauauuaguguaaacaugaa......................................................................  1   1  S04
....................aaaaauauuaguguaaacaugaaG.....................................................................  2   1  S04
......................aaauauuaguguaaacauga.......................................................................  1   0  S04
.........................uauuaguguaaacaugaaaaUugg................................................................  1   1  S04
..........................auuaguguaaacaugaaaaguggu...............................................................  1   0  S04
...........................uuaguguaaacaugaaaagug.................................................................  2   0  S04
...........................uuaguguaaacaugaaaagugg................................................................  9   0  S04
...........................uuaguguaaacaugaaaaguggu...............................................................  3   0  S04
...........................uuaguguaaacaugaaaaguggua..............................................................  2   0  S04
............................uaguguaaacaugaaaagu..................................................................  2   0  S04
............................uaguguaaacaugaaaagugg................................................................  3   0  S04
............................uaguguaaacaugaaaaguggu...............................................................  9   0  S04
............................uaguguaaacaugaaaaguggG...............................................................  1   1  S04
............................uaguguaaacaugaaaaguggua..............................................................  20  0  S04
............................uaguguaaacaugaaaagugguC..............................................................  1   1  S04
............................uaguguaaacaugaaaagugguac.............................................................  3   0  S04
.............................aguguaaacaugaaaagug.................................................................  1   0  S04
..............................guguaaacaugaaaaguggu...............................................................  1   0  S04
.................................uaaacaugaaaagugguacuau..........................................................  1   0  S04
.................................uaaacaugaaaagugguacuaug.........................................................  5   0  S04
.................................uaaacaugCaaagugguacuaug.........................................................  2   1  S04
.................................uaaacaugaGaagugguacuaug.........................................................  1   1  S04
.................................uaaacaugCaaagugguacuauga........................................................  2   1  S04
.................................uaaacaugaaaagugguacuauga........................................................  10  0  S04
.................................Aaaacaugaaaagugguacuauga........................................................  3   1  S04
.................................uaaacaugaGaagugguacuauga........................................................  1   1  S04
..................................aaacaugaaaagGgguacuau..........................................................  1   1  S04
..................................aaacaugaaaagugguacuau..........................................................  1   0  S04
..................................aaacaugaaaagugguacuaug.........................................................  5   0  S04
..................................aaacaugCaaagugguacuaug.........................................................  2   1  S04
..................................aaacaugaaaagugguacuauga........................................................  13  0  S04
..................................aaacaugCaaagugguacuauga........................................................  1   1  S04
..................................aaacaugCaaagugguacuaugaa.......................................................  4   1  S04
..................................aaacaugaaaagugguacuaugaU.......................................................  2   1  S04
..................................aaacaugaaaagugguacuaugaa.......................................................  38  0  S04
..................................aaacaugaaaagugguacuaugaau......................................................  1   0  S04
..................................aaacaugaaaagugguacuaugaaA......................................................  1   1  S04
..................................aaacaugCaaagugguacuaugaau......................................................  2   1  S04
..................................aaacaugaaaagugguacuaugaauCu....................................................  1   1  S04
...................................aacaugCaaagugguacuaug.........................................................  1   1  S04
...................................aacaugaaaagugguacuaug.........................................................  3   0  S04
...................................aacaugaaaagugguacuauga........................................................  20  0  S04
```

Mature Star

```
ccuucauuaaaaauuaggguaaaaauauuaguguaaacaugaaaagugguacuaugaauguguuauuugugguaauuucucauuacauaggggguuguuucuuuuuaucacaa
...................................aacaugCaaagugguacuauga........................................................ 2 1 S04
...................................aacaugaaaagugguacuaugaa....................................................... 10 0 S04
...................................aacaugaaaagugguacuaugaaG...................................................... 3 1 S04
...................................aacaugaaaagugguacuaugaaC...................................................... 12 1 S04
...................................aacaugaaaagGgguacuaugaau...................................................... 1 1 S04
...................................aacaugaaaagugguacuaugaaA...................................................... 2 1 S04
...................................aacaugaaaagugguacuaugaGu...................................................... 1 1 S04
...................................Nacaugaaaagugguacuaugaau...................................................... 1 1 S04
...................................aacaugaaaagugguacuaugaau...................................................... 133 0 S04
...................................aUcaugaaaagugguacuaugaau...................................................... 1 1 S04
...................................aacaugaaaGgugguacuaugaau...................................................... 1 1 S04
...................................aacaugCaaagugguacuaugaau...................................................... 10 1 S04
...................................Cacaugaaaagugguacuaugaaug..................................................... 1 1 S04
...................................aacaugaaaagugguacuaugaaug..................................................... 1 0 S04
....................................acaugCaaagugguacuaug......................................................... 2 1 S04
....................................acaugaaaagugguacuauga........................................................ 4 0 S04
....................................acaugaaaagGgguacuauga........................................................ 1 1 S04
....................................acaugCaaagugguacuauga........................................................ 2 1 S04
....................................acaugCaaagugguacuaugaa....................................................... 1 1 S04
....................................acaugaaaagugguacuaugaa....................................................... 3 0 S04
....................................acaugaaaagugguacuaugaau...................................................... 9 0 S04
....................................acaugCaaagugguacuaugaau...................................................... 1 1 S04
....................................aAaugaaaagugguacuaugaaug..................................................... 2 1 S04
....................................acaugCaaagugguacuaugaaug..................................................... 4 1 S04
....................................acaugaaaagugguacuaugaaug..................................................... 94 0 S04
....................................acaugaaaagugguacuaugaUug..................................................... 3 1 S04
....................................acaugaaaagGgguacuaugaaug..................................................... 1 1 S04
.....................................caugaaaagugguacuaug......................................................... 3 0 S04
.....................................caugCaaagugguacuaug......................................................... 2 1 S04
.....................................caugCaaagugguacuauga........................................................ 1 1 S04
.....................................caugaaaagugguacuauga........................................................ 5 0 S04
.....................................caugCaaagugguacuaugaa....................................................... 1 1 S04
.....................................caugaaaagugguacuaugaG....................................................... 1 1 S04
.....................................caugaaaagugguacuaugaa....................................................... 7 0 S04
.....................................caugCaaagugguacuaugaau...................................................... 9 1 S04
.....................................caugaaaagugguacuaugaaC...................................................... 6 1 S04
.....................................caCgaaaagugguacuaugaau...................................................... 1 1 S04
.....................................caugaaaagugguacuaugaaG...................................................... 3 1 S04
.....................................caugaaaagugguacuaugaau...................................................... 42 0 S04
.....................................caCgaaaagugguacuaugaaug..................................................... 1 1 S04
.....................................caugCaaagugguacuaugaaug..................................................... 7 1 S04
.....................................caugaaaagugguacuaugaaug..................................................... 50 0 S04
.....................................caugaGaagugguacuaugaaugu.................................................... 2 1 S04
.....................................caugaaaagugguacuaGgaaugu.................................................... 1 1 S04
.....................................caugaaaagugguacuaugaaugA.................................................... 44 1 S04
.....................................caugaaaagugguacuaugaaCgu.................................................... 1 1 S04
.....................................caugaaaagugguacuaugaaugG.................................................... 71 1 S04
.....................................caugaaaagugguGcuaugaaugu.................................................... 3 1 S04
.....................................caugaaaagugguacuaugaauCu.................................................... 2 1 S04
.....................................caugaaaagGgguacuaugaaugu.................................................... 4 1 S04
.....................................caCgaaaagugguacuaugaaugu.................................................... 5 1 S04
.....................................caugaaCagugguacuaugaaugu.................................................... 2 1 S04
.....................................caugaaaagugguacuaugaUugu.................................................... 1 1 S04
.....................................caugaaaagugguacuaugaGugu.................................................... 4 1 S04
.....................................caugaaaagugguacuaugaaugu.................................................... 1508 0 S04
.....................................caugCaaagugguacuaugaaugu.................................................... 78 1 S04
.....................................caugaaaagugguacuaugUaugu.................................................... 1 1 S04
.....................................caugaaaGgugguacuaugaaugu.................................................... 7 1 S04
.....................................Naugaaaagugguacuaugaaugu.................................................... 4 1 S04
.....................................caugaaaGgugguacuaugaaugug................................................... 1 1 S04
.....................................caugaaaagugguacuaugaauguU................................................... 4 1 S04
.....................................caugaaaagugguacuaugaaugug................................................... 45 0 S04
.....................................caugaaaagugguacCaugaaugugu.................................................. 1 1 S04
.....................................caugaaaagugguacuaugaaugugu.................................................. 2 0 S04
.....................................caugaaaagugguacuaugaauguUu.................................................. 1 1 S04
......................................augaaaagugguacuaug......................................................... 1 0 S04
......................................augaaaagugguacuauga........................................................ 4 0 S04
......................................augaaaagugguacuaugaa....................................................... 1 0 S04
......................................augCaaagugguacuaugaau...................................................... 2 1 S04
......................................augaaaagugguacuaugaau...................................................... 14 0 S04
```

```
                                     Mature                     Star
ccuucauuaaaaauuaggguaaaaauauuaguguaaacaugaaaagugguacuaugaaugguuauuugugguaauuucucauuacauagggggguuguuucuuuuuaucacaa
......................................augCaaagugguacuaugaaug.....................................................    4     1    S04
......................................augaaaagugguacuaugaaug.....................................................    17    0    S04
......................................augaaaagugguacuaugaauC.....................................................    1     1    S04
......................................augaaaagugguacuaugaaugA....................................................    1     1    S04
......................................augaaaagugguacuaugaaugu....................................................    57    0    S04
......................................augCaaagugguacuaugaaugu....................................................    9     1    S04
......................................augaaaagugguacuaugaaugG....................................................    3     1    S04
......................................augaaaGgugguacuaugaaugug...................................................    4     1    S04
......................................augaaaaguggGacuaugaaugug...................................................    1     1    S04
......................................augaGaagugguacuaugaaugug...................................................    3     1    S04
......................................augaaaagugguacuaugaaugug...................................................    597   0    S04
......................................augaaaagugguacuaugaaugAg...................................................    1     1    S04
......................................aGgaaaagugguacuaugaaugug...................................................    1     1    S04
......................................augaaaagugguacuaAgaaugug...................................................    1     1    S04
......................................augaaaagugguacuaugaGugug...................................................    1     1    S04
......................................augaaaagugguacuaugaaugGg...................................................    1     1    S04
......................................augaaaagugguacuaugaauguU...................................................    7     1    S04
......................................augCaaagugguacuaugaaugug...................................................    79    1    S04
......................................augaaaagugguGcuaugaaugug...................................................    1     1    S04
......................................augaaaagugguacuaugaaugugu..................................................    3     0    S04
......................................augaaaagugguacuaCgaaugugu..................................................    1     1    S04
......................................auAaaaagugguacuaugaaugugu..................................................    1     1    S04
......................................augCaaagugguacuaugaaugugu..................................................    2     1    S04
......................................augaaaagugguacuaugaauguguu.................................................    1     0    S04
.......................................ugCaaagugguacuaugaau......................................................    1     1    S04
.......................................ugaaaagugguacuaugaaG......................................................    1     1    S04
.......................................ugaaaagugguacuaugaau......................................................    3     0    S04
.......................................ugaGaagugguacuaugaaug.....................................................    1     1    S04
.......................................ugaaaagugguacuaugaaug.....................................................    14    0    S04
.......................................ugaaaagugguacuaugaaugu....................................................    75    0    S04
.......................................ugaaaagugguacuaugaaugG....................................................    1     1    S04
.......................................ugaGaagugguacuaugaaugu....................................................    1     1    S04
.......................................ugaaaagugguacuaugaaugA....................................................    9     1    S04
.......................................ugCaaagugguacuaugaaugu....................................................    4     1    S04
.......................................ugaaaagugguacuaugaauguU...................................................    4     1    S04
.......................................ugaaaaguggGacuaugaaugug...................................................    1     1    S04
.......................................ugaaaGgugguacuaugaaugug...................................................    1     1    S04
.......................................ugCaaagugguacuaugaaugug...................................................    9     1    S04
.......................................ugaaaagugguacuaugaaugug...................................................    70    0    S04
.......................................ugaaaagugguacuaugaGugug...................................................    2     1    S04
.......................................ugaaaagugguacuaugaaugGg...................................................    1     1    S04
.......................................ugCaaagugguacuaugaaugugu..................................................    5     1    S04
.......................................ugaaaGgugguacuaugaaugugu..................................................    1     1    S04
.......................................ugaaaagugguacuaugaauguAu..................................................    1     1    S04
.......................................ugaaaaCugguacuaugaaugugu..................................................    1     1    S04
.......................................ugaaaagugguacuaugaaugugu..................................................    13    0    S04
........................................gaaaagugguacuaugaa.......................................................    2     0    S04
........................................gaaaagugguacuaugaau......................................................    2     0    S04
........................................gaaaagugguacuaugaaG......................................................    1     1    S04
........................................gaaaagugguacuaugaaug.....................................................    13    0    S04
........................................gaaaagugguacuaugaaugG....................................................    3     1    S04
........................................gCaaagugguacuaugaaugu....................................................    3     1    S04
........................................gaaaagugguacuaugaaugu....................................................    46    0    S04
........................................gaaaagugguacuaugaaugA....................................................    3     1    S04
........................................gaaaagugguacuaugaauguU...................................................    3     1    S04
........................................gCaaagugguacuaugaaugug...................................................    8     1    S04
........................................gaaaagugguacuaugaGugug...................................................    1     1    S04
........................................gaaaagugguacuaugaaugug...................................................    72    0    S04
........................................gaaaagugguacuaugaaugugu..................................................    9     0    S04
........................................gaaaagugguacuaugaauguUu..................................................    1     1    S04
........................................gGaaagugguacuaugaaugugu..................................................    1     1    S04
........................................gCaaagugguacuaugaaugugu..................................................    1     1    S04
........................................gaaaagugguacuaugaauguguu.................................................    1     0    S04
.........................................aaaagugguacuaugaau......................................................    2     0    S04
.........................................aaaagugguacuaugaaug.....................................................    1     0    S04
.........................................aaaagugguacuaugaaugG....................................................    3     1    S04
.........................................aaaagugguacuaugaaugu....................................................    12    0    S04
.........................................aaaagugguacuaugaauguU...................................................    3     1    S04
.........................................Naaagugguacuaugaaugug...................................................    1     1    S04
.........................................Caaagugguacuaugaaugug...................................................    4     1    S04
```

```
                                    Mature            Star
ccuucauuaaaaauuaggguaaaaauauuaguguaaacaugaaaagugguacuaugaauguguuauuugugguaauuucucauuacauaggggguuguuucuuuuuaucacaa
.........................................aaaagugguacuaugaaugug...................................................    39    0    S04
.........................................aaaagugguacuaugaaugugu..................................................    8     0    S04
.........................................Caaagugguacuaugaaugugu..................................................    4     1    S04
.........................................aaaagugguaAuaugaaugugu..................................................    2     1    S04
.........................................Gaaagugguacuaugaauguguu.................................................    1     1    S04
.........................................aaaagugguaAuaugaauguguu.................................................    2     1    S04
.........................................aaaagugguacuaugaauguguu.................................................    2     0    S04
.........................................aaaagugguaAuaugaauguguua................................................    1     1    S04
..........................................aaagugguacuaugaaug.....................................................    1     0    S04
..........................................aaagugguacuaugaaugu....................................................    7     0    S04
..........................................aaagugguacuaugaauguU...................................................    1     1    S04
..........................................aaagugguacuaugaaugug...................................................    5     0    S04
..........................................aaagugguacCaugaaugugu..................................................    1     1    S04
..........................................aaagugguaAuaugaaugugu..................................................    2     1    S04
..........................................aaagugguacuaugaaugugu..................................................    6     0    S04
..........................................aaagugguacuaugaauguAu..................................................    2     1    S04
..........................................aaagugguacuaugaauguguA.................................................    1     1    S04
..........................................aaagugguacuaugaauguguu.................................................    1     0    S04
..........................................aaagugguacuaugaauguguuU................................................    1     1    S04
..........................................aaagugguacuaugaauguguuG................................................    2     1    S04
..........................................aaagugguacuaugaauguguuau...............................................    2     0    S04
..........................................aaagugguacuaugaauguguuGu...............................................    1     1    S04
...........................................aagugguacuaugaaugu....................................................    3     0    S04
...........................................aagugguacuaugaaugA....................................................    1     1    S04
...........................................aagugguacuaugaaugug...................................................    1     0    S04
...........................................aagugguacuaugaaugugu..................................................    1     0    S04
...........................................aagugguaAuaugaaugugu..................................................    2     1    S04
...........................................aagugguacuaugaauguguA.................................................    1     1    S04
...........................................aagugguaAuaugaauguguu.................................................    1     1    S04
...........................................aagugguacuaugaauguguu.................................................    1     0    S04
...........................................aagugguacuCugaauguguuauu..............................................    2     1    S04
...........................................aagugguacuaugaauguguuauu..............................................    1     0    S04
............................................agugguacuaugaaugug...................................................    4     0    S04
............................................agugguaAuaugaaugugu..................................................    1     1    S04
............................................agugguacuaugaaugugu..................................................    4     0    S04
............................................agugguacuaugaauguguuauA..............................................    2     1    S04
............................................agugguacuaugaauguguuauuu.............................................    1     0    S04
.............................................gugguacuaugaaugugu..................................................    1     0    S04
.............................................gugguacuCugaauguguuauuug............................................    1     1    S04
..............................................ugguacuaugaauguguuauCugu...........................................    1     1    S04
..............................................ugguacuaugaauguguuauuugu...........................................    1     0    S04
...............................................gguacuCugaauguguuauuugu...........................................    1     1    S04
................................................guaAuaugaauguguuauuugugg.........................................    1     1    S04
.................................................uacuaugaauguguuauCugugg.........................................    1     1    S04
........................................................aauguguuauuuguggCaauuucu.................................    1     1    S04
.................................................................uuuguggCaauuucucauuaca..........................    1     1    S04
```

Provisional ID	:	chrA07_618899
Score total	:	0
Score for star read(s)	:	-1.3
Score for read counts	:	-0.3
Score for mfe	:	1.7
Score for randfold	:	0
Score for cons. seed	:	
Total read count	:	11
Mature read count	:	11
Loop read count	:	0
Star read count	:	0

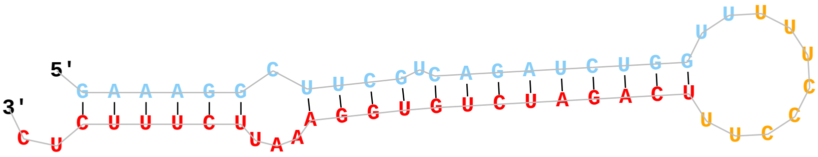

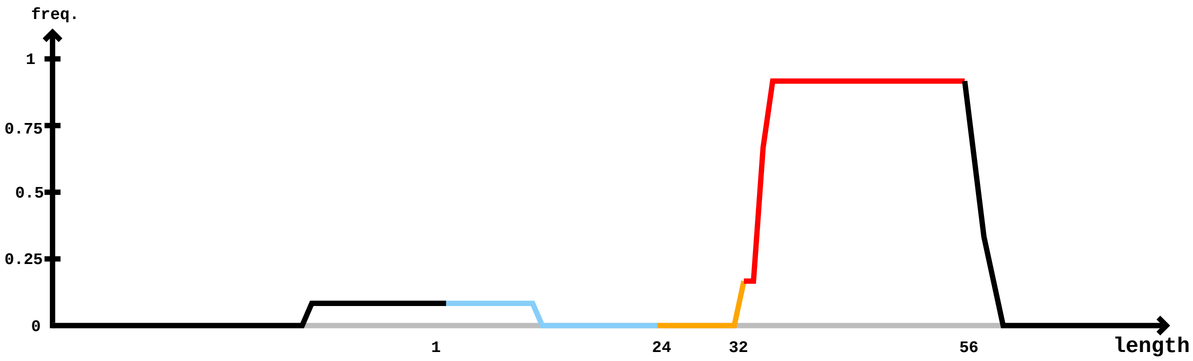


```
                                                    Star                          Mature
5'- acggcgugacgacagugaggagggucggcguuuaggacugaaaggcuucgucagaucugguuuuucccuuucagaucuguggaaauucuuucucggcgaccgcucgaaucacgu   -3'    exp
    .((......))...((((.((((((((.((.........((((((.((((.(((((((((..........)))))))))))))...)))))).)).))))).)))...))))..     reads      mm        sample
    .........................cggcguuuaggacugaaaggcuuA.................................................................     1          1         S03
    ........................................................................agaucuguggaaauuUuuucuc....................     1          1         S03
    ........................................................................agaucuguggaaauucuuucuU....................     1          1         S03
    ........................................................................agaucuguggaaauucuuucucgg..................     1          0         S03

    ......................................................................ucagaucuguggaaauucuuucuc....................     2          0         S04
    ........................................................................agaucuguggaaauucuuucuc....................     2          0         S04
    ........................................................................agGucuguggaaauucuuucucgg..................     1          1         S04
    .........................................................................gaucuguggaaauucuuucuc....................     1          0         S04
    .........................................................................gaucuguggaaauucuuucucggc.................     2          0         S04
```

Provisional ID : chrA07_620955
Score total : 111.6
Score for star read(s) : 3.9
Score for read counts : 107.2
Score for mfe : 0.5
Score for randfold : 0
Score for cons. seed :
Total read count : 222
Mature read count : 205
Loop read count : 4
Star read count : 13

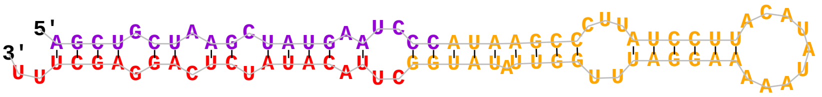

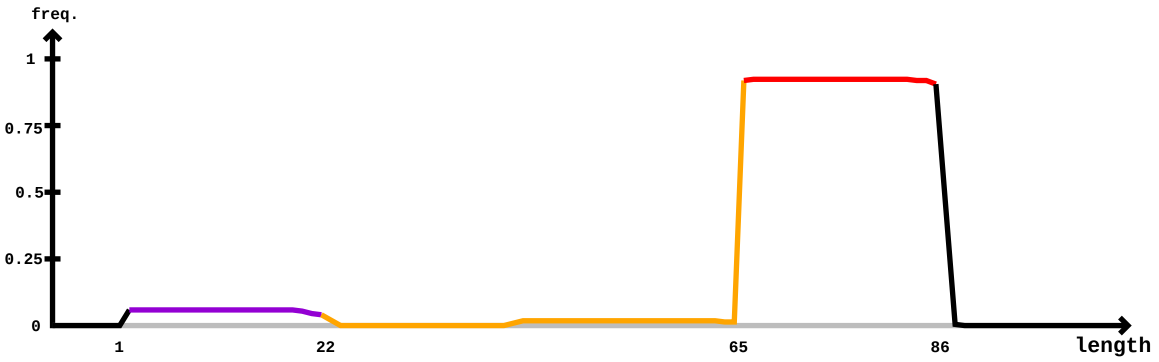

Star Mature

```
5'- aagaaaagcugcuaagcuaugaaucccauaagcccuuauccuuacauauaaaaaggauuugguuauauggcuuacauaucucaggagcuuuuacuugcccuuuaauugcu  -3'  obs
    aagaaaagcugcuaagcuaugaaucccauaagcccuuauccuuacauauaaaaaggauuugguuauauggcuuacauaucucaggagcuuuuacuugcccuuuaauugcu        exp
    ((((((((((.((.((.((((.(..(((((((((...((((((.........))))))..)))).)))))..).)))).)).)).))))))).)))..............   reads   mm   sample
    ......agcugcuaagcuaugaau......................................................................................   1       0    S03
    ......agcugcuaagcuaugaaucc....................................................................................   1       0    S03
    ......agcugcuaagcuaugaauccU...................................................................................   1       1    S03
    ......agcugcuaagcuaugaauccc...................................................................................   5       0    S03
    ..............................................uauaaaaaggauuugguuauaugA........................................   1       1    S03
    ...............................................auaaaaaggauuugguuauau..........................................   1       0    S03
    ...............................................auaaaaaggauuugguuauaugg........................................   1       0    S03
    ......................................................................cuuacauaucucaggagA......................   1       1    S03
    ......................................................................cuuacauaucucaggagcuuA...................   1       1    S03
    ......................................................................cuuacauaucucaggagcuuG...................   1       1    S03
    ......................................................................cuuacauaucucaggagcuuC...................   3       1    S03
    ......................................................................cuuacauaucucaggagcuuu...................   54      0    S03
    ......................................................................cuuacauaucucaggagcuuuuU.................   1       1    S03

    ......agcCgcuaagcuaugaauc.....................................................................................   1       1    S04
    ......agcugcuaagcuaugaauc.....................................................................................   1       0    S04
    ......agcugcuaagcuaugaauccU...................................................................................   1       1    S04
    ......agcugcuaagcuaugaauccA...................................................................................   1       1    S04
    ......agcugcuaagcuaugaauccca..................................................................................   1       0    S04
    ..............................................uauaaaaaggauuugguuauaugg........................................   1       0    S04
    ......................................................................cuuacauaucucaggagcuu....................   3       0    S04
    ......................................................................cuuCcauaucucaggagcuuu...................   1       1    S04
    ......................................................................cuuacauaucucaggagcuuu...................   108     0    S04
    ......................................................................cuuacauaucuUaggagcuuu...................   4       1    S04
    ......................................................................cuuacCuaucucaggagcuuu...................   1       1    S04
    ......................................................................cuuacauaucucaggagcuuG...................   4       1    S04
    ......................................................................cuuacGuaucucaggagcuuu...................   1       1    S04
    ......................................................................cuuacauaucucaggagcuCu...................   1       1    S04
    ......................................................................cuuacauaucucaggagcuuC...................   11      1    S04
    ......................................................................cuuacauaucucaggagUuuu...................   2       1    S04
    ......................................................................cuuUcauaucucaggagcuuu...................   1       1    S04
    ......................................................................cuuacauauUucaggagcuuu...................   1       1    S04
    ......................................................................cuuacauaucCcaggagcuuu...................   1       1    S04
```

```
      Star                                                          Mature
aagaaaagcugcuaagcuaugaaucccauaagcccuuauccuuacauauaaaaaggauuugguuauauggcuuacauaucucaggagcuuuuacuugcccuuuaauugcu
......................................................................cuuacauaucucaggagcuuuu..................     4     0     S04
.......................................................................uuacauaucucaggagcuuu...................     1     0     S04
```

Provisional ID	:	chrA07_622710
Score total	:	1.6
Score for star read(s)	:	3.9
Score for read counts	:	-3.9
Score for mfe	:	1.7
Score for randfold	:	0
Score for cons. seed	:	
Total read count	:	4
Mature read count	:	3
Loop read count	:	0
Star read count	:	1

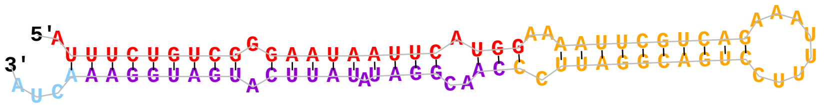

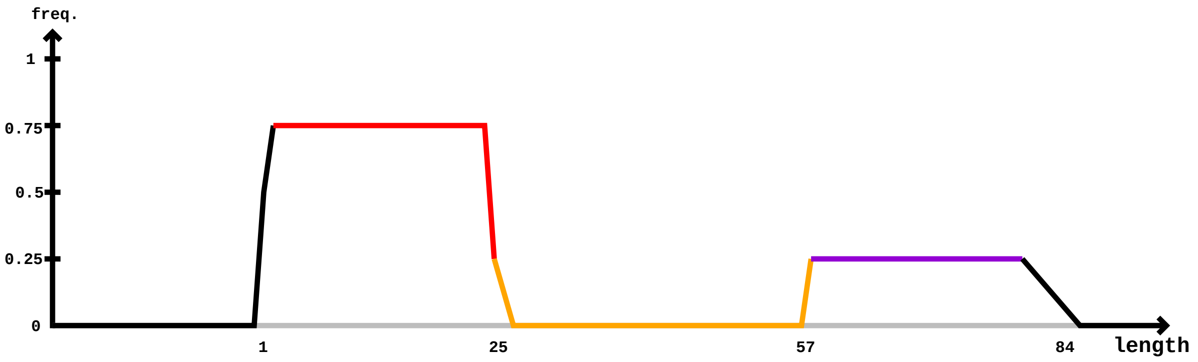


```
                     Mature                                                    Star
5'- ccgacaaauucccaacauauaauuucugucgggaauaauucauggaaaauucgucagaaauuuuccugacggauucccaacggauauauucaugauggaaacuaucugucggga   -3'    obs
    ccgacaaauucccaacauauaauuucugucgggaauaauucauggaaaauucgucagaaauuuuccugacggauucccaacggauauauucaugauggaaacuaucugucggga          exp
    .........((((.(((.(((.((((((((.((((((((.(((..(((((((((........))))))))).)))..)))).))))).)))))))).))).))).))))   reads     mm     sample
    ...................aauuucugucgggaauaauucaug.......................................................................   1         0      S03
    .............................................................................caacggauauauucaugauggaa..............   1         0      S03

    ...................aauuucugucgggaauaauucaug.......................................................................   1         0      S04
    ....................auuucugucgggaauaauucaugg......................................................................   1         0      S04
```

Provisional ID	:	chrA07_642024
Score total	:	112.7
Score for star read(s)	:	3.9
Score for read counts	:	107.7
Score for mfe	:	1.1
Score for randfold	:	0
Score for cons. seed	:	
Total read count	:	223
Mature read count	:	130
Loop read count	:	92
Star read count	:	1

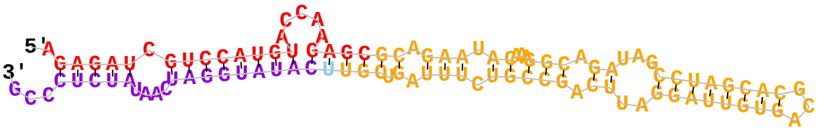

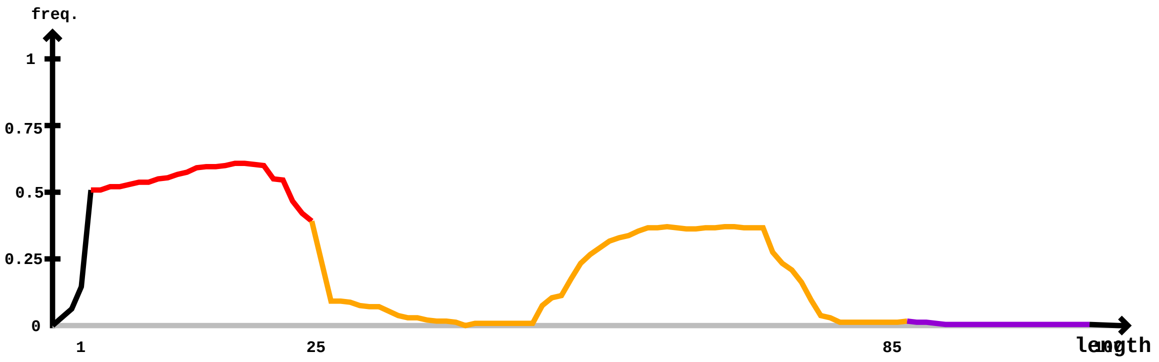

Mature **Star**

```
5'- ucagagaucguccauguaccaagagcgcagaauacaaaggcagauagccuagcacgcaguguuaggauucagccgucuuuagucguucauauggaucaauaucucccggu -3'   obs
    ucagagaucguccauguaccaagagcgcagaauacaaaggcagauagccuagcacgcaguguuaggauucagccgucuuuagucguucauauggaucaauaucucccggu        exp
    ...(((((.((((((((.....((((((.(((.((...(((.((...((((((((...))))))))..)).))))).))).).)))))))))))))....))))).....
```

sequence	reads	mm	sample
ucagCgaucguccauguac	1	1	S03
ucagagaucguccauguacc	1	0	S03
ucagagaucguccauguacca	1	0	S03
ucaCagaucguccauguaccaa	1	1	S03
ucagagaucguccaugGaccaag	1	1	S03
ucagagaucguccauguaccaaga	5	0	S03
ucagagaucguccauguaccaagG	1	1	S03
.cagagaucguccauguaccU	1	1	S03
.cagagaucguccGuguacca	1	1	S03
.cagagaucguccauguacca	6	0	S03
.cagagaucguccauguaccaUg	1	1	S03
.cagagaucguccauguaccaag	6	0	S03
.Gagagaucguccauguaccaagag	1	1	S03
.cagagaucgCccauguaccaagag	1	1	S03
.cagagaucguccauguaccaagag	3	0	S03
..agagaucguccauguacca	3	0	S03
..agagaucguccauguaccaag	10	0	S03
..agagaCcguccauguaccaag	1	1	S03
..agagaucguccauguaccaaga	1	0	S03
..agagaucguccauguaccaagag	1	0	S03
..agagaucguccauguacUaagag	1	1	S03
..agagaucguccauguaccaagagU	15	1	S03
..agagaucguccauguaccaagaUc	1	1	S03
..agagaucguccauguaccaagagG	1	1	S03
..agagaucguccauguaccaagagc	39	0	S03
..agagaucguccauguaccaagagA	3	1	S03
..Ggagaucguccauguaccaagagc	3	1	S03
..agagauUguccauguaccaagagc	1	1	S03
..agGgaucguccauguaccaagagc	1	1	S03
....agaucguccauguaccaagagc	3	0	S03
......aucguccauguaccaagagcgca	1	0	S03
......aucguccauguaccaagagcgcag	1	0	S03
.......ucguccauguaccaagagcgcag	1	0	S03

Mature Star

ucagagaucguccauguaccaagagcgcagaauacaaaggcagauagccuagcacgcaguguuaggauucagccgucuuuagucguucauauggaucaauaucucccgggu			
.......ucguccauguaccaagagcgcaga..	1	0	S03
.........guccauguaccaagagcgcagaau..	2	0	S03
.........guccauguaccaagagcgcagaaC..	1	1	S03
..........Cccauguaccaagagcgcagaaua...	1	1	S03
...........ccauguaccaagagcgcagaauG...	1	1	S03
...........ccauguaccaagagcgcagaaua...	1	0	S03
...........ccauguaccaagagcgcagaauac..	1	0	S03
............cauguaccaagagcgcag...	1	0	S03
............cauguaccaagagcgcagaauC...	1	1	S03
.............auguaccaagagcgcagaau..	1	0	S03
.............auguaccaagagcgcagaauaU..	1	1	S03
.............auguaccaagagcgcagaauacaa..	2	0	S03
..............uguaccaagagcgcagaauacaaa...	1	0	S03
................uaccaagagcgcagaauacaaagA...	1	1	S03
.................accaagagcgcagaauacaaaggU..	2	1	S03
..................ccaagagAgcagaauacaaaggc..	1	1	S03
....gauagccuagcacgcaguguu..	1	0	S03
....gauagccuagcacgcaguguua...	1	0	S03
....uagcacgcaguguuaggauucagc......................................	10	0	S03
....uagcacgcaguguuaggauucagA......................................	1	1	S03
....uagUacgcaguguuaggauucagc......................................	1	1	S03
....agcacgcaguguuaggauuc...	1	0	S03
....agcacgcaguguuaggauucagcU.....................................	1	1	S03
....agcacgcaguguuaggauucagcc.....................................	4	0	S03
....gcacgcaguguuaggauucagccg....................................	1	0	S03
....gcacgcaguguuaggauucagccA....................................	1	1	S03
....cacgcaguguuaggauucagc......................................	2	0	S03
....cacgcaguguuaggauucagcA.....................................	1	1	S03
....cacgcaguguuaggauucagccA....................................	1	1	S03
....cacgcaguguuaggauucagccg....................................	1	0	S03
....cacgcaguguuaggauucagccgG...................................	1	1	S03
....cacgcaguguuaggauucagccgu...................................	5	0	S03
....cacgcaguguuaggauucagccgC...................................	1	1	S03
....cacgcaguguuaggauucagccguA..................................	1	1	S03
....acgcaguguuaggauucagc......................................	1	0	S03
....acgcaguguuaggauucagcU.....................................	1	1	S03
....acgcaguguuaggauucagcc.....................................	2	0	S03
....acgcaguguuaggauucagccg....................................	1	0	S03
....acgcaguguuaggauucagccguU..................................	1	1	S03
....acgcagAguuaggauucagccguc..................................	1	1	S03
....acgcaguguuaggauucagccguc..................................	6	0	S03
....acgcaguguuaggauucagccgucC.................................	1	1	S03
....cgcaguguuaggauucagc......................................	1	0	S03
....cgcaguguuaggauucagccg....................................	1	0	S03
....cgcaguguuaggauucagccgC...................................	1	1	S03
....cgcaguguuaggauucagccgucu.................................	4	0	S03
....cgcaguguuaggauucagccgucC.................................	1	1	S03
....gcaguguuaggauucagc......................................	2	0	S03
....gcaguguuaggauucagccgu...................................	1	0	S03
....gcaguguuaggauucagccguc..................................	1	0	S03
....gcaguguuaggauucagccguU..................................	1	1	S03
....gcaguguuaggauucagccgucuu................................	1	0	S03
....caguguuaggauucagccgucu.................................	2	0	S03
....caguguuaggauucagccgucG.................................	1	1	S03
....caguguuaggauucagccgucuu................................	1	0	S03
....caguguuaggauucagccgucuuu...............................	2	0	S03
....aguguuaggauucagccgucu.................................	1	0	S03
....aguguuaggGuucagccgucuuu...............................	1	1	S03
....guguuaggauucagccguc..................................	1	0	S03
....uguuaggauucagccguc..................................	2	0	S03
....uguuaggauucagccgucC.................................	1	1	S03
....guuaggauucagccgucu.................................	2	0	S03
....uaggauucagccgucuuu...............................	1	0	S03
....auucagUcgucuuuagucguuc.......................	1	1	S03
....ucagUcgucuuuagucguucaua....................	1	1	S03
....agccgucuuuagucguucau.....................	1	0	S03
ucagagaucguccaugua...	1	0	S04
ucagagaucguccauguaccaaga...	3	0	S04
..agagaucguccauguaccaaga...	1	0	S04

Mature **Star**

```
ucagagaucguccauguaccaagagcgcagaauacaaaggcagauagccuagcacgcaguguuaggauucagccgucuuuagucguucauauggaucaauaucucccggu
..agagaucguccauguacAaagagc....................................................................................    1    1    S04
..agagaucguccauguaccaagagU....................................................................................    1    1    S04
..agagaucguccauguaccaagagc....................................................................................    3    0    S04
.................................................uagcacgcaguguuaggauuc........................................    1    0    S04
.................................................uagcacgcaguguuaggauucagc.....................................    2    0    S04
.................................................Gagcacgcaguguuaggauucagc.....................................    1    1    S04
..................................................agcacgcaguguuaggauucagcc....................................    1    0    S04
....................................................cacgcaguguuaggauuUagc.....................................    1    1    S04
....................................................cacgcaguguuaggCuucagccgu..................................    1    1    S04
.........................................................aguguuaggauucagccguc.................................    1    0    S04
..........................................................guguuaggauucagccgu..................................    1    0    S04
...........................................................uguuaggauucagccguc.................................    1    0    S04
............................................................guuaggauucagccgucu................................    1    0    S04
.......................................................................................cauauggaucaGuaucucccg..    1    1    S04
```

Provisional ID	:	chrA07_648408
Score total	:	0.6
Score for star read(s)	:	-1.3
Score for read counts	:	0
Score for mfe	:	1.9
Score for randfold	:	0
Score for cons. seed	:	
Total read count	:	35
Mature read count	:	35
Loop read count	:	0
Star read count	:	0

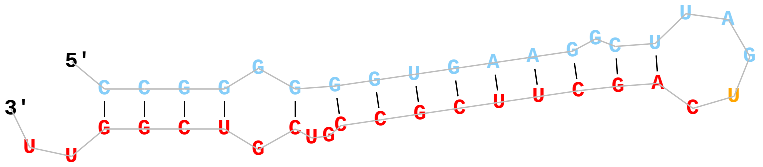

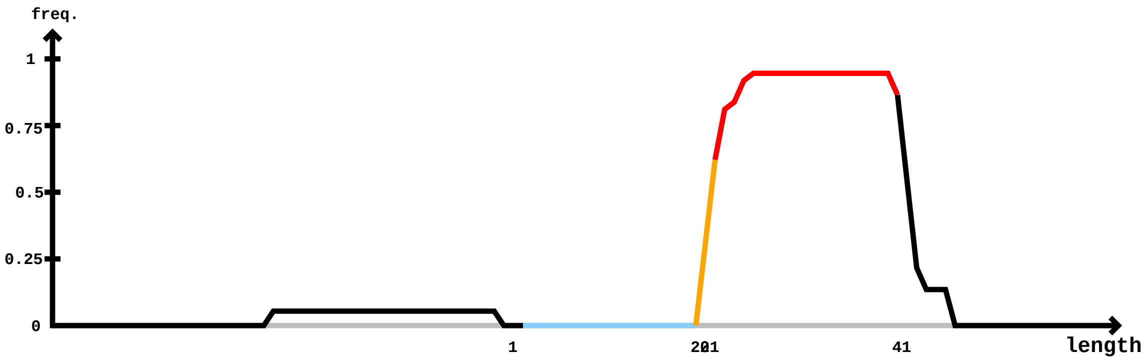

Star Mature

```
5'- ccgggagguggaggcuacuacagcuccaucgccgccggcucgugucuccggggggugaaggcuuagucagcuucgccgucgucgguucuguauccggggugcgaagggu   -3'    exp
    ((.((((.((.((....)).)).)))).(((((.((((.....(...((((.((((((((.((.....)))))))))..).))))..).....)))).).)))).))..    reads     mm     sample
    .....................agcuccaucgccgccggcucgugu................................................................    1         0      S03
    ..................................................................ucagcuAcgccgucgucggu.......................    1         1      S03
    ..................................................................ucagcuAcgccgucgucgguu......................    5         1      S03
    ..................................................................ucagcuAcgccgucgucgguuc.....................    3         1      S03
    ...................................................................cagcuAcgccgucgucggu.......................    1         1      S03
    ...................................................................cagcuAcgccgucgucgguu......................    7         1      S03
    ....................................................................agcuAcgccgucgucgguu......................    3         1      S03
    ....................................................................agcuucAccgucgucgguucugua.................    1         1      S03

    .....................agcuccaucgccgccggcucgugC................................................................    1         1      S04
    ..................................................................ucagcuucgccgucgucgguuU.....................    1         1      S04
    ...................................................................cagcuucgccgucgucggu.......................    1         0      S04
    ...................................................................cagcuAcgccgucgucgguu......................    1         1      S04
    ...................................................................cagcuucgccgucgucgguu......................    1         0      S04
    ...................................................................cagcuucgccgucgucgguuU.....................    1         1      S04
    ...................................................................cagcuAcgccgucgucgguucu....................    1         1      S04
    ....................................................................agcuucgccgucgucgguu......................    2         0      S04
    ....................................................................agcuucgccgucgucgguucGgua.................    1         1      S04
    .....................................................................gcuucgccgucgucgguucA....................    1         1      S04
    ......................................................................cuucgccgucgucgguucu....................    1         0      S04
    ......................................................................cuucgccgucgucgguucGgua.................    2         1      S04
    .......................................................................uucgccgucgucgguucGgua.................    1         1      S04
```

Provisional ID	:	chrA07_653380
Score total	:	0
Score for star read(s)	:	-1.3
Score for read counts	:	0
Score for mfe	:	1.3
Score for randfold	:	0
Score for cons. seed	:	
Total read count	:	495
Mature read count	:	490
Loop read count	:	5
Star read count	:	0

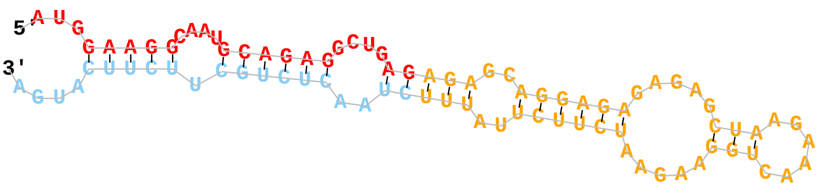

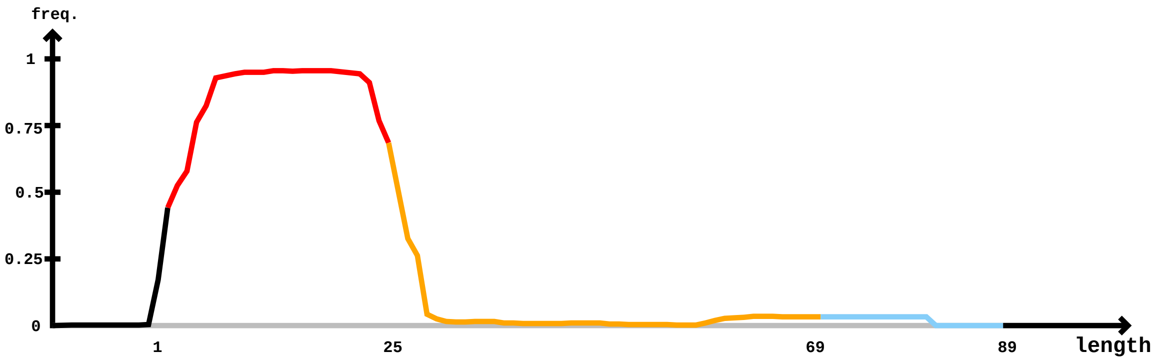

Mature **Star**

```
5'- gaguuaugccauggaaggcaaugcagaggcugagagagcaggagagagagcuaagaaacuggaagaaucuucuuauuucuaacucugcuucuucaugauaaauaacucuu  -3'   exp
    ((((((..((((.((((....((((((....(((((..((((((.....(((......))).....))))))..)))))..)))))).))))))))....))))))..   reads   mm   sample
    ........Ncauggaaggcaaugcagaggcug..............................................................................   1   1   S03
    .........cauggaagAcaaugcagag..................................................................................   1   1   S03
    .........cauggaaggcaaCgcagaggc................................................................................   1   1   S03
    .........cauggaaggcaaugcagaggcA...............................................................................   1   1   S03
    .........cauggaaggcaaugcagaggcu...............................................................................   4   0   S03
    .........cauggaaggcaaugcagaggcug..............................................................................   8   0   S03
    .........cauggaaggcaaugcagaggcuA..............................................................................   5   1   S03
    .........cauggaaggcaaugcagaggcuU..............................................................................   3   1   S03
    .........cauggaaggcaaugcagagAcug..............................................................................   1   1   S03
    .........cauggaaggcaaugcagaggcAga.............................................................................   1   1   S03
    .........cauggaaggcaaugcagaggcuga.............................................................................   7   0   S03
    .........cauggaaggcaaugcagaggcugU.............................................................................   1   1   S03
    .........cauggaaggcaaugcagaggcugG.............................................................................   1   1   S03
    ..........auggaaggcaaugcagagg.................................................................................   1   0   S03
    ..........auggaaggcaaugcagaggcA...............................................................................   1   1   S03
    ..........auggaaggcaaugcagaggcu...............................................................................   1   0   S03
    ..........auggaaggcaaugcagaggcuA..............................................................................   1   1   S03
    ..........auggaaggcaaugcagaggcug..............................................................................   4   0   S03
    ..........auggaaggcaGugcagaggcuga.............................................................................   1   1   S03
    ..........auggaaggcaaugcagaggcuga.............................................................................   3   0   S03
    ..........auggaaggcaaugcagaggcugaA............................................................................   6   1   S03
    ..........auggaaggcaaugcagaggcugag............................................................................   39  0   S03
    ..........auggaaggcaaugcagaAgcugag............................................................................   1   1   S03
    ..........auggaaggcaaugcagaggcugGg............................................................................   1   1   S03
    ..........auggaaggcaaugcagaggcugagG...........................................................................   1   1   S03
    ..........auggaaggcaaugcagaggcugaga...........................................................................   2   0   S03
    ...........uggaaggcaaugcagaggcu...............................................................................   1   0   S03
    ...........uggaaggcaaugcagaggcug..............................................................................   1   0   S03
    ...........Aggaaggcaaugcagaggcug..............................................................................   1   1   S03
    ...........uggaaggcGaugcagaggcug..............................................................................   1   1   S03
    ...........uggaaggcaaugUagaggcug..............................................................................   1   1   S03
    ...........uggaaggcaaugcagaggcugag............................................................................   1   0   S03
    ...........uggaaggcaGugcagaggcugag............................................................................   1   1   S03
    ...........uggaaggcaaugcagaggcugaga...........................................................................   9   0   S03
```

Mature Star

```
gaguuaugccauggaaggcaaugcagaggcugagagagcaggagagagagcuaagaaacuggaagaaucuucuuauuucuaacucugcuucuucaugauaaauaacucuu
...........Aggaaggcaaugcagaggcugaga...........................................................................  1  1  S03
............ggaaggcaaugcagaggcuC..............................................................................  1  1  S03
............ggaaggcaaugcagaggcugag............................................................................  3  0  S03
............ggaaggcaaugcagaggcugGga...........................................................................  1  1  S03
............ggaaggcaaugcagaggcugaga...........................................................................  2  0  S03
............ggaaggcaaugcagaggcugagag..........................................................................  6  0  S03
............ggaaggcaaugUagaggcugagag..........................................................................  1  1  S03
............ggaaggcaaugcagaggcugagaga.........................................................................  1  0  S03
.............gaaggcaaugcagaggcugag............................................................................  1  0  S03
.............gaaggcaaugcGgaggcugag............................................................................  1  1  S03
.............gaaggcaaugcagaggcugaga...........................................................................  2  0  S03
.............gaaggcaaugcagaggcugagag..........................................................................  4  0  S03
.............gaaggcaaugcagaggcugagagC.........................................................................  14  1  S03
.............gaaggcaaugcagaggcugagaga.........................................................................  7  0  S03
.............gaaggcaaugcagaggcugagagG.........................................................................  4  1  S03
..............aaggcaaugcagaggcug..............................................................................  1  0  S03
..............aaggcaaugcagaggcuAa.............................................................................  1  1  S03
..............aaggcaaugcagaggcugaga...........................................................................  1  0  S03
..............aaggcaaugcagaggcugagaC..........................................................................  1  1  S03
..............aaggcaaugcagaggcugagag..........................................................................  1  0  S03
..............aaggcaaugcagaggcugagaga.........................................................................  2  0  S03
..............aaggcaaugcagaggcugagagC.........................................................................  1  1  S03
..............aaggcaaugcagaggcugagagGg........................................................................  1  1  S03
..............aaggcaaugcagaggcugagagag........................................................................  2  0  S03
..............aaggcaaugcagaggcugagagagU.......................................................................  1  1  S03
...............aggcaaugcagaggcugU.............................................................................  1  1  S03
...............aggcaaugcagaggcugag............................................................................  3  0  S03
...............aggcaaugcagaggcugagU...........................................................................  1  1  S03
...............aggcaaugcagaggcugaga...........................................................................  2  0  S03
...............aggcaaugcagaggcugagag..........................................................................  2  0  S03
...............aggcaaugcagaggcugagaga.........................................................................  2  0  S03
...............aggcaaugcagaggcugagagC.........................................................................  3  1  S03
...............aggcaaugcagaggcugagagag........................................................................  1  0  S03
...............aggcaaugcagaggcugagagCg........................................................................  1  1  S03
...............aggcaaugcagaggcugagagCgc.......................................................................  1  1  S03
................ggcaaugcagaggcugagagC.........................................................................  1  1  S03
.................gcaaugcagaggcugagU...........................................................................  1  1  S03
..................caaugcagaggcugagagG.........................................................................  1  1  S03
.....................ugcagaggcugagagCgcaggaga.................................................................  1  1  S03
..................................agUgcaggagagagagcuaagaaa....................................................  1  1  S03
.....................................gcaggagagagagcuaaga......................................................  2  0  S03
..................................................................aucuucuuauuuUuaacucugcuu....................  2  1  S03
...................................................................ucuucuuauuucuaacucugcuu....................  1  0  S03
...................................................................ucuucuuauuuUuaacucugcuu....................  1  1  S03
....................................................................cuucuuauuuUuaacucugcuu....................  1  1  S03
.....................................................................uucuuauuucuaacucugcuA....................  1  1  S03
......................................................................ucuuauuuUuaacucugcuu....................  1  1  S03
.......................................................................cuuauuuUuaacucugcuu....................  2  1  S03

gaguuGugccauggaaggcaaug.......................................................................................  1  1  S04
.........cauggaaggcaaugcagag..................................................................................  1  0  S04
.........cauggaaggcaaugcagaggc................................................................................  1  0  S04
.........cauggaaggcaaugcagaggcA...............................................................................  1  1  S04
.........cauggaaggcaaugcagaggcG...............................................................................  3  1  S04
.........cauggaaggcaaugcagaggcC...............................................................................  1  1  S04
.........cauggaaggcaaugcagaggcu...............................................................................  4  0  S04
.........cauggaaggcaaugcagaggcuU..............................................................................  4  1  S04
.........cauggaaggcaaugcagaggcuA..............................................................................  4  1  S04
.........cauggaaggcaaCgcagaggcug..............................................................................  1  1  S04
.........cauggaaggcaaugcagaggcug..............................................................................  15  0  S04
.........cauggaaggcaaugcGgaggcuga.............................................................................  1  1  S04
.........cauggaagAcaaugcagaggcuga.............................................................................  2  1  S04
.........cauggaaggcaaugcagaggcugU.............................................................................  1  1  S04
.........cauggGaggcaaugcagaggcuga.............................................................................  1  1  S04
.........cauggaaggcaaugcagaggcuga.............................................................................  13  0  S04
..........auggaaggcaaugcagagg.................................................................................  1  0  S04
..........auggaaggcaaugcagaggcug..............................................................................  17  0  S04
..........augAaaggcaaugcagaggcug..............................................................................  1  1  S04
..........auggaaggcaaugcagagAcug..............................................................................  1  1  S04
..........auggaaggcaaugcagaggcuA..............................................................................  1  1  S04
```

Mature **Star**

```
gaguuaugccauggaaggcaaugcagaggcugagagagcaggagagagagcuaagaaacuggaagaaucuucuuauuucuaacucugcuucuucaugauaaauaacucuu
..........auggaaggcaaugcagaggcuga.............................................................................  2   0  S04
..........auggaaggcaaugcagaggcugaA............................................................................  4   1  S04
..........auggaaggcaaugcagaggcugag............................................................................  44  0  S04
..........Guggaaggcaaugcagaggcugag............................................................................  1   1  S04
..........auggaaggcaaugcagagUcugag............................................................................  1   1  S04
..........auggaagAcaaugcagaggcugag............................................................................  1   1  S04
..........auggaaggcaaugcagaggcugaga...........................................................................  1   0  S04
..........auggaaggcaaugcagaggcugagU...........................................................................  1   1  S04
..........auggaaggcaaugcagaggcugagG...........................................................................  1   1  S04
..........auggaaggcaaugcagaggcugagaA..........................................................................  1   1  S04
...........uggaaggcaaugcagaggcug..............................................................................  1   0  S04
...........uggaaggcaaugcagaggcugG.............................................................................  1   1  S04
...........uggaaggcaaugcagaggcuga.............................................................................  4   0  S04
...........uggaaggcaaugcagaggcugag............................................................................  2   0  S04
...........uggaaggcaaCgcagaggcugaga...........................................................................  1   1  S04
...........Gggaaggcaaugcagaggcugaga...........................................................................  1   1  S04
...........uggaaggcaaugcagaggcugaga...........................................................................  15  0  S04
...........uggaaggcaaugcagaggcugagU...........................................................................  1   1  S04
............ggaaggcaaugcagaggcugag............................................................................  1   0  S04
............ggaaggcaaugcagaggcugaga...........................................................................  3   0  S04
............ggaaggcaaugcagaggcugagag..........................................................................  7   0  S04
............ggaaggcaaugcagaggcugagaA..........................................................................  2   1  S04
.............gaaggcaaugcagaggcugag............................................................................  6   0  S04
.............gaaggcaaugcagaggcugaga...........................................................................  3   0  S04
.............gaaggcaaugcagaggcugagag..........................................................................  2   0  S04
.............gaaggcaaugcagaggcugagaA..........................................................................  1   1  S04
.............gaaggcaaugcaUaggcugagaga.........................................................................  1   1  S04
.............gaaggcaaugcagaCgcugagaga.........................................................................  1   1  S04
.............gaaggcaaugcagaggcugagagG.........................................................................  14  1  S04
.............gaaggcaaugcagaggcugagagC.........................................................................  25  1  S04
.............gaaggcaaugcagaggcugagaga.........................................................................  9   0  S04
..............aaggcaaugcagaggcugag............................................................................  3   0  S04
..............aaggcaaugcagaggcugaga...........................................................................  4   0  S04
..............aaggcaaugcagaggcugagag..........................................................................  1   0  S04
..............aaggcaaugcagaggcugagagG.........................................................................  4   1  S04
..............aaggcaaugcagaggcugagagC.........................................................................  3   1  S04
..............aaggcaaugcagaggcugagaga.........................................................................  2   0  S04
..............Gaggcaaugcagaggcugagaga.........................................................................  1   1  S04
..............aaggcaaugcagaggcugagagag........................................................................  1   0  S04
..............aaggcaaugcaAaggcugagagag........................................................................  1   1  S04
...............aggcaaugcagaggcuga.............................................................................  2   0  S04
...............aggcaaugcagaggcugag............................................................................  1   0  S04
...............aggcaaugcagaggcugaga...........................................................................  10  0  S04
...............aggcaaugcagaggcugagag..........................................................................  4   0  S04
...............aggcaaugcagaggcugagagC.........................................................................  9   1  S04
...............aggcaaugcagaggcugagagG.........................................................................  4   1  S04
...............aggcaaugcagaggcugagaga.........................................................................  3   0  S04
...............aggcaaugcagaggcugagagCg........................................................................  2   1  S04
...............aggcaaugcagaggcugagagagc.......................................................................  1   0  S04
...............aggcaaugcagaggcugagagagU.......................................................................  1   1  S04
................ggcaaugcagaggcugaga...........................................................................  1   0  S04
................ggcaaugcagaggcugagagG.........................................................................  1   1  S04
................ggcaaugcagaggcugagagC.........................................................................  1   1  S04
.................gcaaugcagaggcugaga...........................................................................  1   0  S04
.................gcaaugcagaggcugagagagc.......................................................................  1   0  S04
.................gcaaugcagaggcugagagagUa......................................................................  1   1  S04
..................caaugcagaggcugagagC.........................................................................  2   1  S04
.....................ugcagaggcugagagagcaggaga.................................................................  1   0  S04
.....................ugcagaggcugagagagcaggaAa.................................................................  1   1  S04
........................agaggcugagagCgcaggagaga...............................................................  1   1  S04
..........................................agagagagcuaagaaacugga...............................................  1   0  S04
....................................................aagaaacuggaagaaucuucuu....................................  1   0  S04
..................................................................aucuucuuauuuUuaacucugcuu....................  2   1  S04
...................................................................ucuucuuauuuUuaacucugcuu....................  3   1  S04
....................................................................cuucuuauuuUuaacucugcuu....................  3   1  S04
```

Provisional ID	:	chrA07_672834
Score total	:	1.1
Score for star read(s)	:	-1.3
Score for read counts	:	0
Score for mfe	:	2.4
Score for randfold	:	0
Score for cons. seed	:	
Total read count	:	33
Mature read count	:	30
Loop read count	:	0
Star read count	:	3

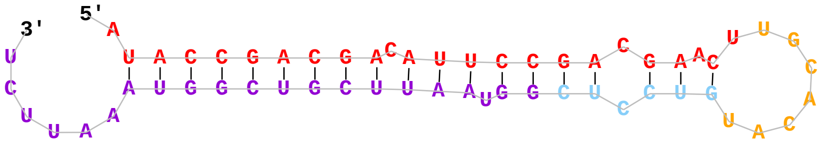

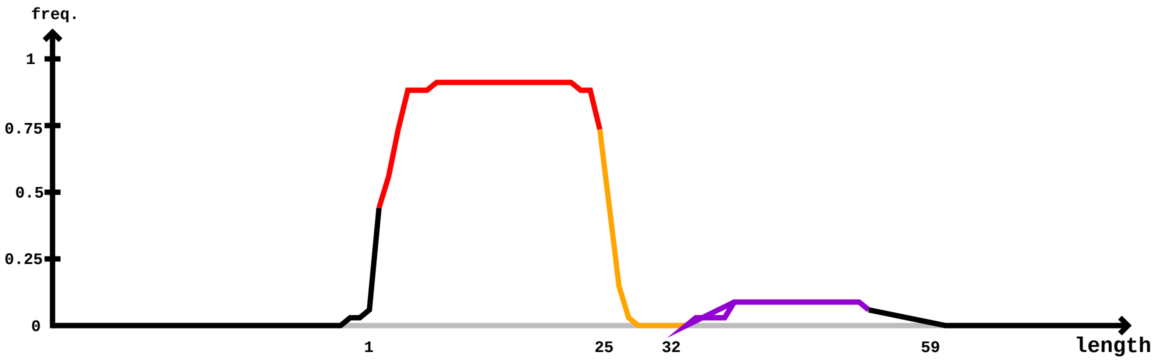


```
                                   Mature                               Star
5'- agaaacacauguccucgguaauucaucggaauauaccgacgacauuccgacgaacuugcacauguccucgguaauucgucgguaaauucugacgaauuaccgaggacauu   -3'    obs
    agaaacacauguccucgguaauucaucggaauauaccgacgacauuccgacgaacuugcacauguccucgguaauucgucgguaaauucugacgaauuaccgaggacauu          exp
    ........((((((((((((((((.(((((((.(((((((((.(((((((.((.(........))).)))).)))))))))))).))))))).)))))))))))))))).    reads    mm    sample
    ................................Guaccgacgacauuccgacgaac.......................................................    1        1     S03
    ................................auaGcgacgacauuccgacgaacu......................................................    1        1     S03
    ...................................ccgacgacauuccgacgaacu......................................................    1        0     S03
    ...................................ccgaGgacauuccgacgaacuugc...................................................    1        1     S03

    .............................aauauaccgacgacauuccgacga.........................................................    1        0     S04
    ...............................uauaccgacgacauuccgacgaac.......................................................    1        0     S04
    ................................auaccgacgacauuccgacgaacG......................................................    2        1     S04
    ................................auaccgacgacauuccgacgaacC......................................................    3        1     S04
    ................................auaccgacgacauuccgacgaacu......................................................    6        0     S04
    .................................uaccgacgacauuccgacgaac.......................................................    1        0     S04
    .................................uaccgacgacauuccgacgaacu......................................................    3        0     S04
    ..................................accgacgacauuccgacgaac.......................................................    2        0     S04
    ..................................accgacgacauuccgacgaacuug....................................................    2        0     S04
    ..................................accgacgacauuccgGcgaacuug....................................................    1        1     S04
    ..................................accgacgacauuccgacgaacuAg....................................................    1        1     S04
    ...................................ccgacgacauuccgacgaacu......................................................    2        0     S04
    ...................................ccgacgacauuccgacgaacuu.....................................................    1        0     S04
    ......................................acgacauuccgacgaacuu.....................................................    1        0     S04
    .................................................................ccucgguaauucUucggu...........................    1        1     S04
    .....................................................................gguaauucgucgguaaauucG....................    1        1     S04
    .....................................................................gguaauCcgucgguaaauucu....................    1        1     S04
```

Provisional ID	:	chrA07_678676
Score total	:	19.3
Score for star read(s)	:	3.9
Score for read counts	:	21
Score for mfe	:	-5.5
Score for randfold	:	0
Score for cons. seed	:	
Total read count	:	53
Mature read count	:	40
Loop read count	:	1
Star read count	:	12

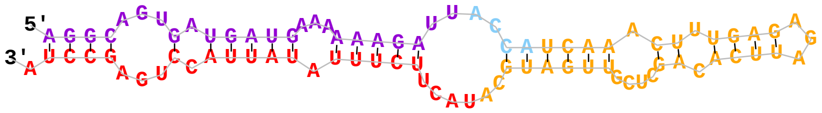

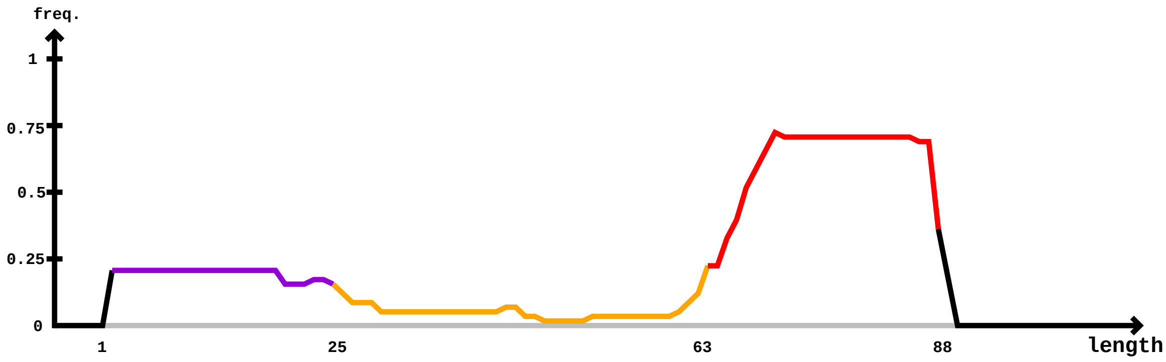

Star Mature

```
5'- uacuaggcagugaugaugaaaaaagauuaccaucaaacuuugagagauucacagcucguugaugcauacuucuuuauauuaccugagccuaguuauguuuuuguucugcuu   -3'   obs
    uacuaggcagugaugaugaaaaaagauuaccaucaaacuuugagagauucacagcucguugaugcauacuucuuuauauuaccugagccuaguuauguuuuuguucugcuu         exp
    .(((((((...(.(((((...(((((....((((((.((.((((...)))).))....))))))......))))).))))).)...))))))).................. reads  mm  sample
    ....aggcagugaugaugaaaa.........................................................................................  2     0   S03
    ....aggcagugaugaugaaaaaagauu...................................................................................  2     0   S03
    ....aggcagugaugaugaaaaaagauuacca...............................................................................  2     0   S03
    .........................auuaccaucaaacuuugagaga................................................................  1     0   S03
    ......................................................cucguugaugcauacuucuu.....................................  1     0   S03
    ...............................................................gcauacuucuuuauauuaccugagU.......................  1     1   S03
    .................................................................auacuucuuuauauuaccugagccua....................  1     0   S03
    ..................................................................uacuucuuuauauuaccugagccu.....................  1     0   S03
    ....................................................................cuucuuuauauuaccugagccu.....................  2     0   S03
    .....................................................................uucuuuauauuaccugagccG.....................  1     1   S03
    ......................................................................ucuuuauauuaccugagccu.....................  1     0   S03
    ......................................................................ucuuuauauuaccugagccC.....................  1     1   S03
    ......................................................................Ccuuuauauuaccugagccu.....................  1     1   S03
    ......................................................................ucuuuauauuaccugagccua....................  3     0   S03
    .......................................................................cuuuauauuaccugagccG.....................  1     1   S03
    .......................................................................cuuuauauuaccugagccu.....................  1     0   S03
    ........................................................................uuuauauuaccugagccA.....................  1     1   S03
    ........................................................................uuuauauuaccugagccu.....................  1     0   S03

    ....aggcagugaugaugaaaa.........................................................................................  1     0   S04
    ....aggcagugaugaugaaaaaa.......................................................................................  1     0   S04
    ....aggcagugaugaugaaaaaag......................................................................................  1     0   S04
    ....aggcagugaugaugaaaaaagau....................................................................................  1     0   S04
    ....aggcagugaugaugaaaaaagauA...................................................................................  1     1   S04
    ....aggcagugaugaugaaaaaagauu...................................................................................  1     0   S04
    ........................gauCaccaucaaacuuugagagauu..............................................................  1     1   S04
    .........................auuaccaucaaacuuugagaga................................................................  1     0   S04
    .............................................gauucacagcucguugaugcauacu.........................................  1     0   S04
    ................................................................cauacuucuuuauauuaccugagccu.....................  1     0   S04
    ................................................................cauacuucuuuauauuaccugagccua....................  1     0   S04
    .................................................................auacuucuuuauauuaccugagccua....................  1     0   S04
    ..................................................................uacuucuuuauauuaccugagccu.....................  1     0   S04
    ..................................................................uacuucuuuauauuaccugagccua....................  3     0   S04
```

Star Mature

```
uacuaggcagugaugaugaaaaaagauuaccaucaaacuuugagagauucacagcucguugaugcauacuucuuuauauuaccugagccuaguuauguuuuguucugcuu
..................................................................uacuucuuuauauuaccugagccuU...................   1   1   S04
....................................................................cuucuuuauauuaccugagccu....................   2   0   S04
....................................................................cuucuuuauauuaccugagccua...................   2   0   S04
.....................................................................uucuuuauauuaccugagccu....................   2   0   S04
.....................................................................uucuuuauauuaccugagccua...................   1   0   S04
......................................................................ucuuuauauuaccugagccua...................   1   0   S04
......................................................................ucuuuauauuaccugagccuU...................   1   1   S04
.......................................................................cuuuauauuaccugagccA....................   1   1   S04
.......................................................................cuuuauauuaccugagccCa...................   1   1   S04
........................................................................uuuauauuaccugagccu....................   1   0   S04
........................................................................uuuauauuaccugagccua...................   1   0   S04
.........................................................................uuauauuaccugagccAa...................   1   1   S04
.........................................................................uuauauuaccugagccua...................   3   0   S04
```

Provisional ID	:	chrA07_679018
Score total	:	299
Score for star read(s)	:	3.9
Score for read counts	:	295.3
Score for mfe	:	-0.2
Score for randfold	:	0
Score for cons. seed	:	
Total read count	:	591
Mature read count	:	538
Loop read count	:	0
Star read count	:	53

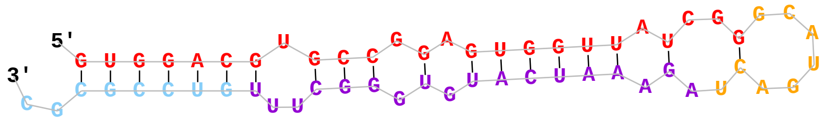

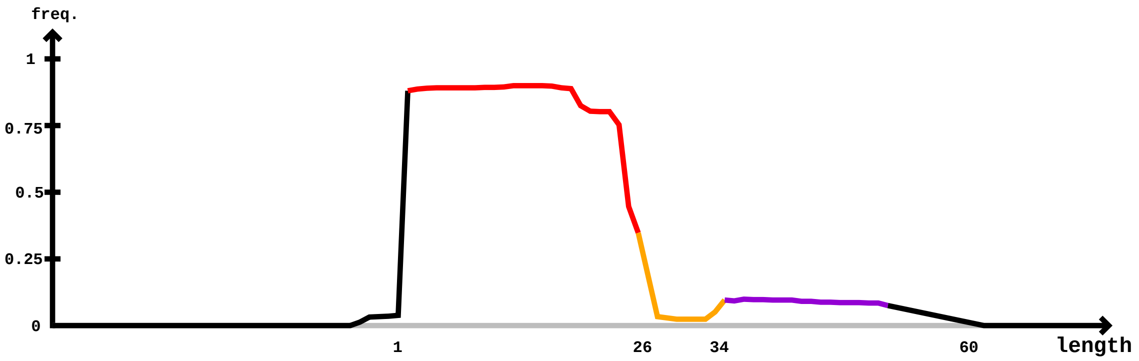

Mature Star

```
5'- uaaucaaauauauuuuauuuuagcuucaacaaaaguggacgugccggagugguuaucgggcaugacuagaaaucaugugggcuuuguccgcgcugguucgaauccug  -3'   obs
    uaaucaaauauauuuuauuuuagcuucaacaaaaguggacgugccggagugguuaucgggcaugacuagaaaucaugugggcuuuguccgcgcugguucgaauccug         exp
    ....................(((..((((((....(((((((.(((.(.((((((.(..(......)..).)))))).).)))..)))))))....))).)))..)))   reads   mm   sample
    .............................Aaaaaguggacgugccggag...........................................................   1       1    S03
    .............................Aaaaaguggacgugccggagugguuaucgg.................................................   1       1    S03
    ..............................aaaaguggacgugccggagu..........................................................   1       0    S03
    ..............................aaaaguggacgugccggagug.........................................................   1       0    S03
    ..............................aaaaguggacgugccggagugguuaucggg................................................   3       0    S03
    ...............................aaaguggacgugccggagu..........................................................   1       0    S03
    ................................aaguggacgugccggagug.........................................................   1       0    S03
    .................................Cguggacgugccggagugguuauc...................................................   1       1    S03
    .................................aAuggacgugccggagugguuauc...................................................   1       1    S03
    ..................................guggacgugccgAagugg........................................................   2       1    S03
    ..................................guggacgugccggGgugg........................................................   1       1    S03
    ..................................gCggacgugccggagugg........................................................   1       1    S03
    ..................................guggacgugccggagugA........................................................   15      1    S03
    ..................................gGggacgugccggagugg........................................................   1       1    S03
    ..................................guggacgugccggaguggC.......................................................   5       1    S03
    ..................................guggacgugccggaguggG.......................................................   2       1    S03
    ..................................guggacgGgccggaguggu.......................................................   1       1    S03
    ..................................guggacgugccggagugguC......................................................   1       1    S03
    ..................................guggacgugccggagAgguuau....................................................   1       1    S03
    ..................................guggacgugccggagugguuaG....................................................   1       1    S03
    ..................................guggacgugccggagugguuaA....................................................   2       1    S03
    ..................................Nuggacgugccggagugguuau....................................................   1       1    S03
    ..................................guggacgugccggagugguuaC....................................................   7       1    S03
    ..................................guggacgugccggagCgguuau....................................................   2       1    S03
    ..................................guggacgugccggagugguuGuc...................................................   1       1    S03
    ..................................Auggacgugccggagugguuauc...................................................   1       1    S03
    ..................................guggacgugccggagugguuauG...................................................   2       1    S03
    ..................................guggacgugccCgagugguuauc...................................................   1       1    S03
    ..................................guggacgugccggagCgguuauc...................................................   7       1    S03
    ..................................gAggacgugccggagugguuauc...................................................   3       1    S03
    ..................................guggacUugccggagugguuauc...................................................   1       1    S03
    ..................................guggacgugccggagugguuauA...................................................   12      1    S03
    ..................................Nuggacgugccggagugguuauc...................................................   1       1    S03
```

```
                                  Mature                          Star
uaaucaaauauauuuuauuuuagcuucaacaaaaguggacgugccggagugguuaucgggcaugacuagaaaucaugugggcuuuguccgcgcugguucgaauccug
..................................guggacgugccggGgugguuauc..................................................  3   1   S03
..................................guggacgugccggaguggGuauc..................................................  2   1   S03
..................................guggacguAccggagugguuauc..................................................  2   1   S03
..................................guggacgugccggagugguuaCc..................................................  3   1   S03
..................................guggacgugccggagugguuaGc..................................................  2   1   S03
..................................gCggacgugccggagugguuauc..................................................  6   1   S03
..................................guggacgugccggagugguuauU..................................................  26  1   S03
..................................guggacgugccggaguggAuauc..................................................  1   1   S03
..................................guggacgugccggagAgguuauc..................................................  3   1   S03
..................................gGggacgugccggagugguuauc..................................................  1   1   S03
..................................guggacgGgccggagugguuauc..................................................  1   1   S03
..................................guggacguUccggagugguuauc..................................................  1   1   S03
..................................guggacgugccggaguggCuauc..................................................  2   1   S03
..................................guggacgugccggagugguuaAc..................................................  1   1   S03
..................................guggacguAccggagugguuaucg.................................................  1   1   S03
..................................guggacgugccggagugguuaucC.................................................  1   1   S03
..................................guggacgugccggagugguuaucA.................................................  22  1   S03
..................................gugAacgugccggagugguuaucg.................................................  1   1   S03
..................................guggacgugccggagugAuuaucgg................................................  1   1   S03
..................................guggacgugccggagugguuaucgU................................................  7   1   S03
..................................guggacgugccggGgugguuaucgg................................................  1   1   S03
..................................gGggacgugccggagugguuaucgg................................................  1   1   S03
..................................gCggacgugccggagugguuaucgg................................................  3   1   S03
..................................guggacgugccggagugguuaucgC................................................  4   1   S03
..................................guggacgugccgAagugguuaucgg................................................  1   1   S03
..................................guggacgAgccggagugguuaucgg................................................  1   1   S03
..................................guggacgugccggagugguuaucAg................................................  1   1   S03
..................................guggacgugccggagugguuGucgg................................................  1   1   S03
..................................guggacgugccggaAugguuaucgg................................................  1   1   S03
..................................Auggacgugccggagugguuaucgg................................................  1   1   S03
..................................guggacgugccggagugguuauGgg................................................  1   1   S03
..................................guggacgugccggagAgguuaucgg................................................  1   1   S03
..................................guggacgugccggagGgguuaucgg................................................  1   1   S03
..................................guggacgugccggagCgguuaucgg................................................  1   1   S03
..................................guggacgugccggagugguuaucgA................................................  38  1   S03
..................................guggacgugccggagugguuaucgggU..............................................  1   1   S03
...................................Gggacgugccggagugguuauc..................................................  1   1   S03
...................................uggacgugccggagugguuaucgA................................................  3   1   S03
....................................ggacgugccggagugguuaucgA................................................  1   1   S03
.....................................gacgugccggagugguuaucA.................................................  1   1   S03
.............................................ggagugguuaucggAcaugacua.......................................  1   1   S03
.............................................ggagugguuaucgggcaugacuG.......................................  1   1   S03
.............................................ggagugguuaucgggcaugGcua.......................................  1   1   S03
..................................................gguuaucgggcaugacuU.......................................  1   1   S03
..................................................gguuGucgggcaugacuaga.....................................  1   1   S03
....................................................uuaucgggcaugacuagaaaucU................................  1   1   S03
....................................................uuaucgggUaugacuagaaauca................................  1   1   S03
..................................................................uGgaaaucaugugggcuu.......................  2   1   S03
..................................................................uGgaaaucaugugggcuuu......................  5   1   S03
...................................................................Ugaaaucaugugggcuuu......................  1   1   S03
...................................................................Ggaaaucaugugggcuuu......................  13  1   S03
....................................................................gaaaucaCgugggcuuug.....................  1   1   S03
....................................................................gaaaucaugugggcuuugu....................  1   0   S03
.....................................................................aaaucaugugggcuCugu....................  1   1   S03

.............................Aaaaaguggacgugccggagu.........................................................  1   1   S04
.............................Aaaaaguggacgugccggaguggu......................................................  2   1   S04
.............................Aaaaaguggacgugccggagugguu.....................................................  2   1   S04
.............................Aaaaaguggacgugccggagugguuaucgg................................................  1   1   S04
..............................aaaaguggacgugccggagu.........................................................  3   0   S04
..............................aaaaguggacgugccggagug........................................................  1   0   S04
..............................aaaaguggacgugccggagugguuaucg.................................................  1   0   S04
..............................aaaaguggacgugccggagugguuaucggg...............................................  2   0   S04
..................................guggacgugccggagugA.......................................................  22  1   S04
..................................guggacgugccggGgugg.......................................................  1   1   S04
..................................guggacgugccggaguggA......................................................  2   1   S04
..................................guggacgugccggaguggC......................................................  1   1   S04
..................................guggacgugccggaguggG......................................................  1   1   S04
..................................guggacgugccggagCgguuau...................................................  2   1   S04
..................................guggacgCgccggagugguuau...................................................  1   1   S04
```

```
                                  Mature                          Star
uaaucaaauauauuuuauuuuagcuucaacaaaaguggacgugccggagugguuaucgggcaugacuagaaaucaugugggcuuuguccgcgcugguucgaauccug
..................................guggacgugccggagugguuaA...................................................	1	1	S04
..................................guggacgugccggagugguuaC...................................................	7	1	S04
..................................guggacgugccggagugguuUu...................................................	1	1	S04
..................................guggacgugccggagGgguuau...................................................	1	1	S04
..................................guggacgugccggagugguuaG...................................................	3	1	S04
..................................guggacgugccCgagugguuau...................................................	1	1	S04
..................................guAgacgugccggagugguuauc..................................................	2	1	S04
..................................guggacgugccggagugguCauc..................................................	1	1	S04
..................................guggacgugccgUagugguuauc..................................................	1	1	S04
..................................guggacgCgccggagugguuauc..................................................	2	1	S04
..................................guggacgugccggagCgguuauc..................................................	8	1	S04
..................................guggacgugccUgagugguuauc..................................................	1	1	S04
..................................guggacgugccggaAugguuauc..................................................	2	1	S04
..................................guggacgugccggagugguuauG..................................................	1	1	S04
..................................guCgacgugccggagugguuauc..................................................	1	1	S04
uaaucaaauauauuuuauuuuagcuucaacaaaaguggacgugccggGgugguuauc..................................................	3	1	S04
..................................guggacgugccggagugguuGuc..................................................	1	1	S04
..................................guggacgugUcggagugguuauc..................................................	1	1	S04
..................................guggacgugccggagugguuCuc..................................................	1	1	S04
..................................gCggacgugccggagugguuauc..................................................	3	1	S04
..................................guggacgugccggaguggCuauc..................................................	2	1	S04
..................................guggacgugccggagugguuaGc..................................................	1	1	S04
..................................gGggacgugccggagugguuauc..................................................	2	1	S04
..................................Auggacgugccggagugguuauc..................................................	2	1	S04
..................................guggacguAccggagugguuauc..................................................	2	1	S04
..................................guggacgugccggagugguuauU..................................................	47	1	S04
..................................guggacgugccggagugguuaCc..................................................	5	1	S04
..................................guggacgugccggagugguuauA..................................................	14	1	S04
..................................guggacgugccggaguggAuauc..................................................	3	1	S04
..................................gugAacgugccggagugguuaucg.................................................	1	1	S04
..................................Auggacgugccggagugguuaucg.................................................	1	1	S04
..................................guggacgugccggagugguuaucA.................................................	25	1	S04
..................................guggacgugccggCgugguuaucg.................................................	1	1	S04
..................................guggacgugccggagugguuauUg.................................................	4	1	S04
..................................guggacgugccggagAgguuaucg.................................................	1	1	S04
..................................guggacgugccggagugguuaucC.................................................	1	1	S04
..................................guggacgugAcggagugguuaucg.................................................	1	1	S04
..................................guggacgugccggagugguuaGcgg................................................	1	1	S04
..................................guggacgugccgUagugguuaucgg................................................	1	1	S04
..................................guggacgugccggagugguuGucgg................................................	1	1	S04
..................................guggacgugccggagGgguuaucgg................................................	1	1	S04
..................................gGggacgugccggagugguuaucgg................................................	2	1	S04
..................................guggacgugccggagCgguuaucgg................................................	7	1	S04
..................................gCggacgugccggagugguuaucgg................................................	3	1	S04
..................................guggacgugccggagugguuaucAg................................................	2	1	S04
..................................guggacgugccggagugguCaucgg................................................	1	1	S04
..................................guggacguAccggagugguuaucgg................................................	1	1	S04
..................................guggacgugccggaguggAuaucgg................................................	1	1	S04
..................................guggacgugccggagugguuaucgU................................................	7	1	S04
..................................gAggacgugccggagugguuaucgg................................................	2	1	S04
..................................guggacgugccggagAgguuaucgg................................................	3	1	S04
..................................guggacgugccggagugguuaucgA................................................	86	1	S04
..................................guggacgugccggagugguuaucgC................................................	1	1	S04
..................................guggacgugccggagugguuaucggAc..............................................	1	1	S04
..................................guggacgugccggagugguuaucgggU..............................................	1	1	S04
..................................guggacgugccggagugguuaucggUc..............................................	1	1	S04
..................................guggacgugccggagugguuaucggUca.............................................	2	1	S04
..................................guggacgugccggagugguuaucgggcG.............................................	1	1	S04
....................................ggacgugccggagugguuaucggA...............................................	1	1	S04
..........................................Accggagugguuaucgggcaugac.........................................	1	1	S04
............................................cggagugguuaucgggcaugacC........................................	1	1	S04
...................................................guuaucgggcaugacuagaaaucC................................	1	1	S04
....................................................uuaucgggcaugacuagaaaucauA..............................	1	1	S04
.....................................................uaucgggcaugacuGgaaaucaug..............................	1	1	S04
......................................................Gucgggcaugacuagaaa...................................	1	1	S04
......................................................aucgggcauUacuagaaaucaugug............................	1	1	S04
.............................................................augacuGgaaaucaugugggc.........................	1	1	S04
..................................................................uGgaaaucaugugggcuu.......................	4	1	S04
..................................................................uUgaaaucaugugggcuuu......................	1	1	S04
..................................................................uGgaaaucaugugggcuuu......................	6	1	S04
```

	Mature	Star			
uaaucaaauauauuuuauuuuagcuuucaacaaaaguggacgugccggagugguuaucgggcaugacuagaaaucaugugggcuuuguccgcgcugguucgaauccug					
....Ggaaaucaugugggcuuu......................			15	1	S04
....aaaucaugugggcuuugu....................			1	0	S04
....aaaucaugugggcuCugu....................			1	1	S04
....aaaucaugugggcuuuUu....................			1	1	S04

Provisional ID	:	chrA07_random_2743826
Score total	:	0.4
Score for star read(s)	:	-1.3
Score for read counts	:	0
Score for mfe	:	1.7
Score for randfold	:	0
Score for cons. seed	:	
Total read count	:	33
Mature read count	:	33
Loop read count	:	0
Star read count	:	0

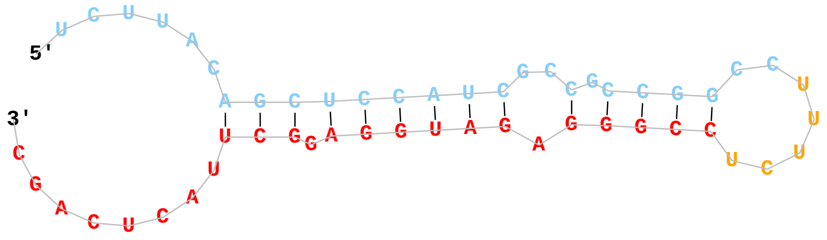

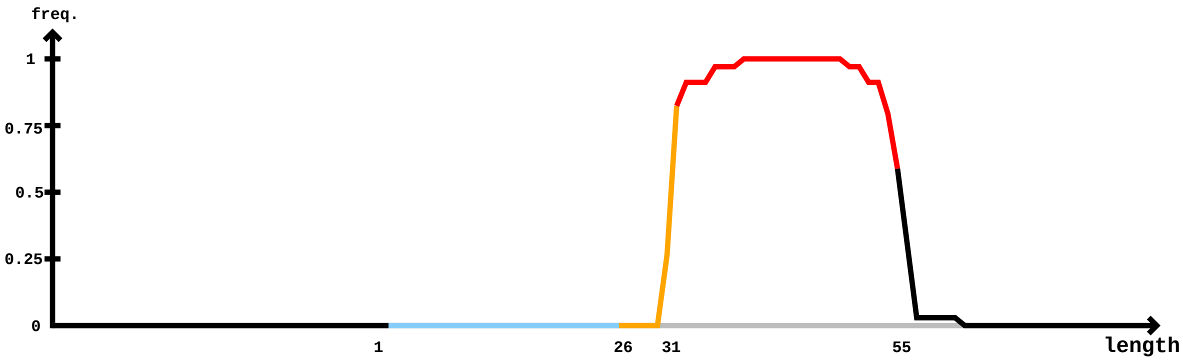


```
                            Star                          Mature
5'- ucagaccaagguucgaauccgggagguggaggcucuuacagcuccaucgccgccggccuuucuccgggagauggaggcuuacucagcucugucgcugccgguuuuguugcugg   -3'    exp
    .........(((.((((.((((..((((((((((.....(((((((((..(.((((.......))))).)))))).))).....))).)).))))).)))).))))..)))..   reads    mm    sample
    ..............................................................uccgggagauggaggcuuaUu..............................   1        1     S03
    ..............................................................uccgggagauggaggcuuaUuca............................   1        1     S03
    ..............................................................uccgggagauggaggcuuaUucag...........................   5        1     S03
    ...............................................................ccgggagauggaggcuuaUu..............................   1        1     S03
    ...............................................................ccgggagauggaggcuuaUuca............................   2        1     S03
    ...............................................................ccgggagauggaggcuuaUucagc..........................   6        1     S03
    ................................................................cgggagauggaggcuuaUucag...........................   1        1     S03
    ...................................................................gagauggaggcuuaUucagc..........................   1        1     S03

    ..............................................................uccgggagauggaggcuCa................................   1        1     S04
    ..............................................................uccgggagauggaggcuuaUuca............................   1        1     S04
    ...............................................................ccgggagauggaggcuuaUucagc..........................   10       1     S04
    ................................................................cgggagauggaggcuuaUucag...........................   1        1     S04
    ................................................................cgggagauggaggcuuaUucagcu.........................   1        1     S04
    ...................................................................gagauggaggcuuaUucagc..........................   1        1     S04
    ......................................................................auggaggcuuacucagcucuguc....................   1        0     S04
```

Provisional ID	: chrA07_random_2746710
Score total	: 1784.3
Score for star read(s)	: 3.9
Score for read counts	: 1779.4
Score for mfe	: 1
Score for randfold	: 0
Score for cons. seed	:
Total read count	: 3502
Mature read count	: 3380
Loop read count	: 2
Star read count	: 120

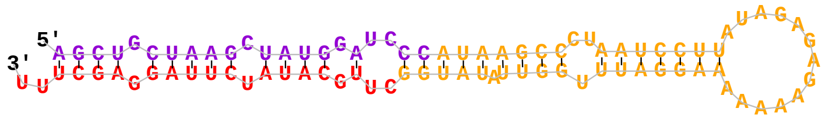

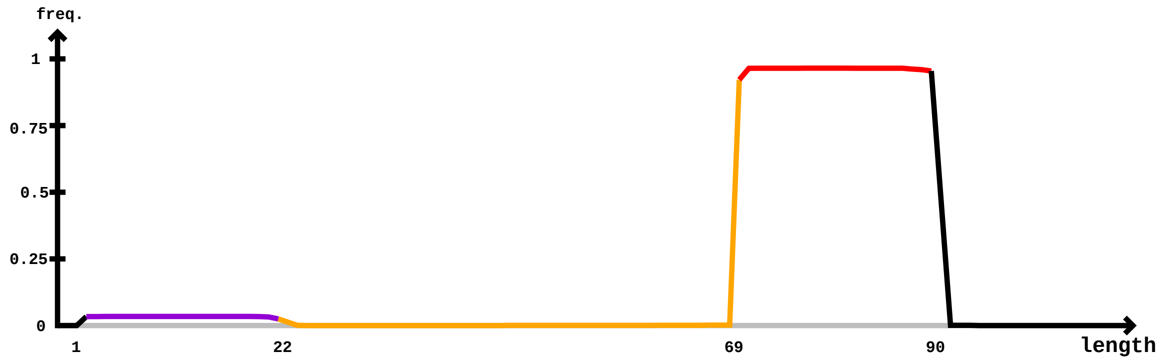

Star **Mature**

```
5'- aagcugcuaagcuauggaucccauaaagcccuaauccuuauagagagaaaaaaaggauuugguuauauggcuugcauaucuuaggagcuuuaacuugcccuuuaauugcuu  -3'   obs
    aagcugcuaagcuauggaucccauaaagcccuaauccuuauagagagaaaaaaaggauuugguuauauggcuugcauaucuuaggagcuuuaacuugcccuuuaauugcuu        exp
    (((((.(((((.((((.(..(((((((((..(((((((...............))))))).)))).)))))..).)))).))))).))))).....................   reads   mm   sample
    .agcugcuaagcuauggau............................................................................................   2   0   S03
    .agcugcuaagcuauggauc...........................................................................................   2   0   S03
    .agcugcuaagcuauggaucc..........................................................................................   8   0   S03
    .agcugcuaagcuauggaucU..........................................................................................   2   1   S03
    .agcugcuaagcuauggauccA.........................................................................................   1   1   S03
    .agcugcuaagcuauggaCccc.........................................................................................   1   1   S03
    .agcugcuaagcuauggauccc.........................................................................................   22  0   S03
    .agcugcuaagcuauggauccU.........................................................................................   9   1   S03
    .agUugcuaagcuauggauccc.........................................................................................   1   1   S03
    .agcugcuaagcuauggauccca........................................................................................   2   0   S03
    .agcugcuaagcuauggaucccU........................................................................................   1   1   S03
    ...................................................................uggcuugcauaucuuaggagU.......................   1   1   S03
    ......................................................................cuugcauaucuuaggagc.......................   2   0   S03
    ......................................................................cuugcauaucuuaggagcC......................   1   1   S03
    ......................................................................cuugcauaucuuaggagcu......................   1   0   S03
    ......................................................................cuugcauaucuuaggagcuu.....................   7   0   S03
    ......................................................................cuugcauaucuuaggagcuC.....................   1   1   S03
    ......................................................................cuugcauaucuuagAagcuuu....................   3   1   S03
    ......................................................................cuugcauaucuuaggagUuuu....................   1   1   S03
    ......................................................................cuugcauauUuuaggagcuuu....................   1   1   S03
    ......................................................................cuugcauaucuuaUgagcuuu....................   1   1   S03
    ......................................................................cuugcauGucuuaggagcuuu....................   1   1   S03
    ......................................................................Guugcauaucuuaggagcuuu....................   2   1   S03
    ......................................................................cuugcauaucuuaggagcuuA....................   16  1   S03
    ......................................................................cCugcauaucuuaggagcuuu....................   7   1   S03
    ......................................................................cuugcauaucuuaggaAcuuu....................   1   1   S03
    ......................................................................cuugcauaucuuagUagcuuu....................   2   1   S03
    ......................................................................cuugcauauAuuaggagcuuu....................   1   1   S03
    ......................................................................Nuugcauaucuuaggagcuuu....................   1   1   S03
    ......................................................................cuugcaCaucuuaggagcuuu....................   4   1   S03
    ......................................................................cuuUcauaucuuaggagcuuu....................   7   1   S03
    ......................................................................cuGgcauaucuuaggagcuuu....................   1   1   S03
    ......................................................................cuugcauaCcuuaggagcuuu....................   5   1   S03
```

```
Star                                                                   Mature
aagcugcuaagcuauggaucccauaaagcccuaauccuuauagagagaaaaaaaggauuugguuauauggcuugcauaucuuaggagcuuuaacuugcccuuuaauugcuu
......................................................................cuAgcauaucuuaggagcuuu....................   1     1   S03
......................................................................cuugcauaucuuaggaUcuuu....................   2     1   S03
......................................................................cuugcUuaucuuaggagcuuu....................   1     1   S03
......................................................................cuugcauaucuuaggagcCuu....................   5     1   S03
......................................................................cuugcauaucuuaggagcuuC....................   82    1   S03
......................................................................cuugcauaucuuaggaCcuuu....................   1     1   S03
......................................................................cuCgcauaucuuaggagcuuu....................   3     1   S03
......................................................................Auugcauaucuuaggagcuuu....................   1     1   S03
......................................................................cuugcauaucuuaggagcuuu....................   1064  0   S03
......................................................................cuugcauaucuuaggCgcuuu....................   2     1   S03
......................................................................cuugcauaucuuaggGgcuuu....................   4     1   S03
......................................................................cuugcauaucuuGggagcuuu....................   2     1   S03
......................................................................cuugcauaucuuaggagcuGu....................   2     1   S03
......................................................................cuugcauaucuuaggagcuCu....................   2     1   S03
......................................................................cuugcauaucuuaggagcuuG....................   29    1   S03
......................................................................cGugcauaucuuaggagcuuu....................   2     1   S03
......................................................................cuugcauaucAuaggagcuuu....................   1     1   S03
......................................................................cuugcauaucuuUggagcuuu....................   1     1   S03
......................................................................cuugcauaucCuaggagcuuu....................   4     1   S03
......................................................................cuugcCuaucuuaggagcuuu....................   1     1   S03
......................................................................cuugcauaucuuaggagcuuuG...................   1     1   S03
......................................................................cuugcauaucuuaggagcuuuU...................   1     1   S03
.......................................................................uugcauaucuuaggagcuu.....................   1     0   S03
.......................................................................uugcauaucuuaggagGuuu....................   1     1   S03
.......................................................................uugcauaucuuaggGgcuuu....................   1     1   S03
.......................................................................uugcauaucuuaggagcuuC....................   3     1   S03
.......................................................................uugcauauUuuaggagcuuu....................   1     1   S03
.......................................................................uCgcauaucuuaggagcuuu....................   2     1   S03
.......................................................................uugcauaucuuaggaAcuuu....................   1     1   S03
.......................................................................uugcauaucuuaggagcuuA....................   1     1   S03
.......................................................................uugcGuaucuuaggagcuuu....................   1     1   S03
.......................................................................uugcauaucuuaggagcuuu....................   57    0   S03
.......................................................................uugcauaucuuaggagcuuuU...................   1     1   S03

.agcugcuaagcuauggauc...........................................................................................   3     0   S04
.agcugcuaagcuauggaucA..........................................................................................   1     1   S04
.agcugcuaagcuauggaucc..........................................................................................   13    0   S04
.agcugcuaagcuauggaCcc..........................................................................................   1     1   S04
.agcugcuaagcuauggaucU..........................................................................................   1     1   S04
.agcugcuaagcuauggauccU.........................................................................................   16    1   S04
.agcugcuaagcuauggauccc.........................................................................................   25    0   S04
.agcugcuaagcuauggauccA.........................................................................................   7     1   S04
...cugcuaagcuauggaucccau.......................................................................................   2     0   S04
..............................................Aaaaaaaaggauuugguuauaugg.........................................   2     1   S04
..............................................................uuauauggcuugcauaucuu.............................   1     0   S04
...................................................................uggcuugcauaucuuaggagA.......................   1     1   S04
...................................................................uggcuugcauaucuuaggagc.......................   1     0   S04
......................................................................cuugcauaucuuaggagc.......................   5     0   S04
......................................................................cuugcauaucuuaggagA.......................   1     1   S04
......................................................................cuugcauaucuuaggagcu......................   6     0   S04
......................................................................cuugcauaucuuaggagcuu.....................   7     0   S04
......................................................................cuugcauaucuuaggagcuC.....................   1     1   S04
......................................................................cuugcauaCcuuaggagcuuu....................   4     1   S04
......................................................................cuugcauaucuuaggagcuuA....................   23    1   S04
......................................................................cuugcauauGuuaggagcuuu....................   1     1   S04
......................................................................cuugcauaucuuaggaCcuuu....................   2     1   S04
......................................................................cuugcauaucuuagAagcuuu....................   1     1   S04
......................................................................cuugcUuaucuuaggagcuuu....................   2     1   S04
......................................................................cuCgcauaucuuaggagcuuu....................   6     1   S04
......................................................................cGugcauaucuuaggagcuuu....................   5     1   S04
......................................................................cuugcaCaucuuaggagcuuu....................   5     1   S04
......................................................................cAugcauaucuuaggagcuuu....................   3     1   S04
......................................................................cuugcauauUuuaggagcuuu....................   1     1   S04
......................................................................cuugcCuaucuuaggagcuuu....................   1     1   S04
......................................................................cuugcauaucuuaggagcCuu....................   2     1   S04
......................................................................cuugcauaucuuaggaUcuuu....................   1     1   S04
......................................................................cuugcauaucuAaggagcuuu....................   1     1   S04
......................................................................cuuAcauaucuuaggagcuuu....................   4     1   S04
......................................................................cuugcauUucuuaggagcuuu....................   1     1   S04
......................................................................cuuUcauaucuuaggagcuuu....................   3     1   S04
```

Star **Mature**

```
aagcugcuaagcuauggaucccauaagcccuaauccuuauagagagaaaaaaaggauuugguuauauggcuugcauaucuuaggagcuuuaacuugcccuuuaauugcuu
.....................................................................cuuCcauaucuuaggagcuuu....................	1	1	S04
.....................................................................Uuugcauaucuuaggagcuuu....................	2	1	S04
.....................................................................cuugcauaucuuaggagcuuC....................	152	1	S04
.....................................................................cuugcauaucuuUggagcuuu....................	2	1	S04
.....................................................................Nuugcauaucuuaggagcuuu....................	2	1	S04
.....................................................................cuugcauaucGuaggagcuuu....................	1	1	S04
.....................................................................cuugcauaucuuaggagcuuG....................	55	1	S04
.....................................................................cCugcauaucuuaggagcuuu....................	2	1	S04
.....................................................................cuugcauaucuuaggagcGuu....................	1	1	S04
.....................................................................cuugcauaucuuaggaAcuuu....................	1	1	S04
.....................................................................cuugcauaucAuaggagcuuu....................	1	1	S04
.....................................................................cuugcauaAcuuaggagcuuu....................	1	1	S04
.....................................................................cuugUauaucuuaggagcuuu....................	5	1	S04
.....................................................................cuugcauaucuuaggagcuuu....................	1585	0	S04
.....................................................................cuugcauaucuuaAgagcuuu....................	3	1	S04
.....................................................................cuugcauaucuuaUgagcuuu....................	1	1	S04
.....................................................................cuugcauaucuuaggGgcuuu....................	6	1	S04
.....................................................................cuugcauaucuuaggagcuCu....................	5	1	S04
.....................................................................cuugcauaucuuaggagUuuu....................	5	1	S04
.....................................................................cuugcauaucuuaggCgcuuu....................	1	1	S04
.....................................................................cuugAauaucuuaggagcuuu....................	1	1	S04
.....................................................................cuugcauaucuuaggagcAuu....................	1	1	S04
.....................................................................cuugcGuaucuuaggagcuuu....................	8	1	S04
.....................................................................cuugcauGucuuaggagcuuu....................	4	1	S04
.....................................................................cuugcauaucuuGggagcuuu....................	1	1	S04
.....................................................................cuugcauaucuuaggUgcuuu....................	3	1	S04
.....................................................................cuugcauaucCuaggagcuuu....................	4	1	S04
.....................................................................cuugcauaucuuaggGgcuuua...................	1	1	S04
.....................................................................cuugcauaucuuaggagcuuuC...................	2	1	S04
.....................................................................cuugcauaucuuaggagcuuuU...................	3	1	S04
.....................................................................cuugcauaucuuaggagcuuua...................	1	0	S04
.....................................................................cuugcauaucuuaggagcuuuG...................	1	1	S04
.....................................................................cuugcauaucuuaggagcuuuaacA................	2	1	S04
......................................................................uugcauaucuuaggagcuuu....................	61	0	S04
......................................................................Cugcauaucuuaggagcuuu....................	1	1	S04
......................................................................uugcauaucuuaggagcuuC....................	10	1	S04
......................................................................Gugcauaucuuaggagcuuu....................	2	1	S04
......................................................................uugcauaucuuaggaAcuuu....................	1	1	S04
......................................................................uCgcauaucuuaggagcuuu....................	1	1	S04
......................................................................uugcaCaucuuaggagcuuu....................	1	1	S04
......................................................................uugcGuaucuuaggagcuuu....................	1	1	S04
......................................................................uugcauaucuuaggagcuuG....................	2	1	S04
.......................................................................ugcauaucuuaggagcuuua...................	1	0	S04
............................................................................aucuuaggagcuAuaacu................	1	1	S04
```

Provisional ID	:	chrA08_694262
Score total	:	0.4
Score for star read(s)	:	-1.3
Score for read counts	:	0
Score for mfe	:	1.7
Score for randfold	:	0
Score for cons. seed	:	
Total read count	:	12
Mature read count	:	12
Loop read count	:	0
Star read count	:	0

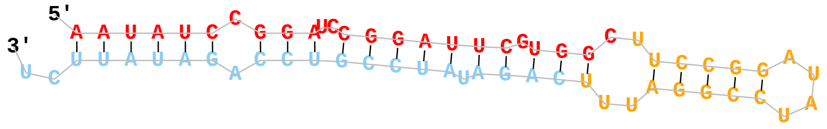

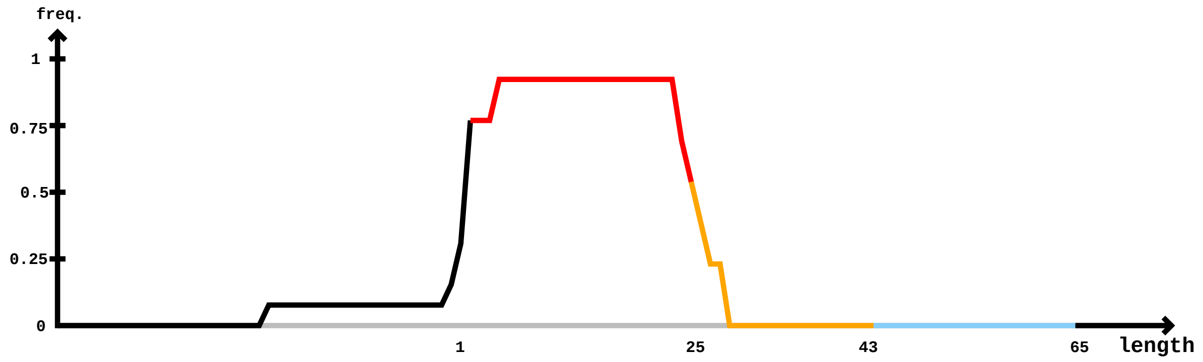

Mature Star

```
5'- ccagauauacggauauucaggauaccuggauccgugaauuuaauauccggauccggauucguggcuuccggauauccggauuucagauauccguccagauauucuauuacccgc   -3'    exp
    ..((((.((((((..((((((...)))))))))))).))))((((((.(((..(((((((.(((..(((((....)))))..))))).)))))))).))))))..........    reads      mm         sample
    .......................................uuaauauccggauccggauucgug...................................................    1          0          S03
    ........................................uaauauccggauccggauucgugg..................................................    1          0          S03
    .........................................aauauccggauccggauucgugg..................................................    1          0          S03
    .........................................aauauccggauccggauucguggc.................................................    1          0          S03
    .........................................aauauccggauccggauucguggG.................................................    1          1          S03
    .........................................aauauccggauccggauucguggA.................................................    1          1          S03
    ............................................auccggaucUggauucguggcuuc..............................................    1          1          S03

    ....................gauaccuggauccgugaauuuaau......................................................................    1          0          S04
    ........................................uaauauccggauccggauucgug...................................................    1          0          S04
    .........................................aauauccggauccggauucgug...................................................    1          0          S04
    .........................................aauauccggauccggauucguggG.................................................    1          1          S04
    ............................................auccggaucUggauucguggcuuc..............................................    1          1          S04
    ............................................auccggauccggauucguAgcuuc..............................................    1          1          S04
```

Provisional ID	:	chrA08_708988
Score total	:	0
Score for star read(s)	:	-1.3
Score for read counts	:	0
Score for mfe	:	1.3
Score for randfold	:	0
Score for cons. seed	:	
Total read count	:	183
Mature read count	:	178
Loop read count	:	0
Star read count	:	5

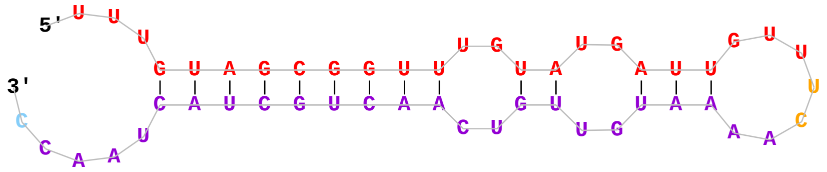

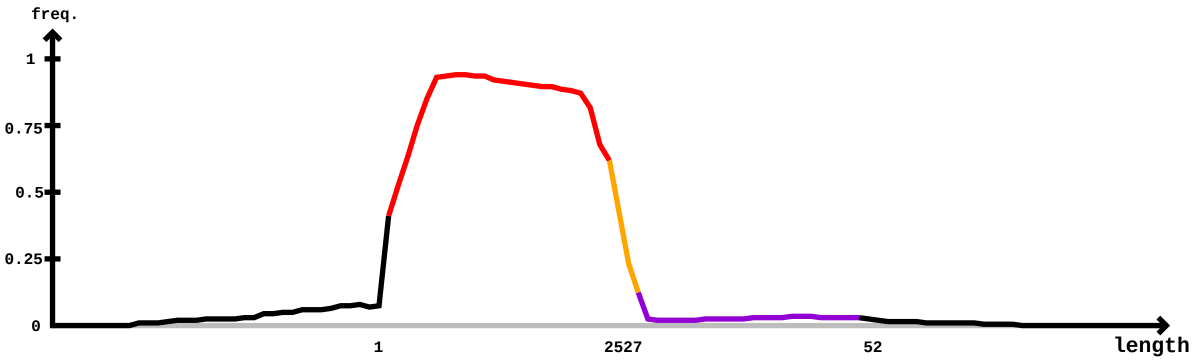

Mature Star

```
5'- aaccagcuuuugauuuuaagagguucagagcgguuuguagcgguuuguaugauuguuucaaaauguugucaacugcuacuaaccgcaaaagcugcguuugcggaucguaccggg  -3'    obs
    aaccagcuuuugauuuuaagagguucagagcgguuuguagcgguuuguaugauuguuucaaaauguugucaacugcuacuaaccgcaaaagcugcguuugcggaucguaccggg          exp
    (((((((((((((...........)))..((((((.(((((((((..((..(((.......)))..))..))))))))).)))))).))))))).)))..(((......))).. reads     mm     sample
    ...........gauuuuaagagguucagagcggu................................................................................ 1         0      S03
    ..............................cgguuuguagcgguuuguaug............................................................... 1         0      S03
    ...............................gguuuguagcgguuuguaugauu............................................................ 1         0      S03
    ................................Uuuuguagcgguuuguaugauugu.......................................................... 1         1      S03
    .................................uuuguagcgguuuguauga.............................................................. 1         0      S03
    .................................uuuguagcgguuuguaugau............................................................. 2         0      S03
    .................................uuuguagcgguuuguaugauu............................................................ 4         0      S03
    .................................uuuguagcgguuuguauCauug........................................................... 1         1      S03
    .................................uuuguagcgguuuguaugauug........................................................... 16        0      S03
    .................................uuuguagcgguuuguaugauuC........................................................... 1         1      S03
    .................................uuuguagcgguuuguaugauuguG......................................................... 3         1      S03
    .................................uuuguagcgguuuguaugauuguu......................................................... 19        0      S03
    .................................uuuguagcgguuuguaugauuguC......................................................... 1         1      S03
    ..................................uuguagcgguuuguaugauu............................................................ 2         0      S03
    ..................................uuguagcgguuuguaugauugu.......................................................... 2         0      S03
    ..................................uuguagcgguuuguaugauugA.......................................................... 2         1      S03
    ..................................uuguagcgguuuguaugauuguu......................................................... 4         0      S03
    ..................................uuguagcgguuuguaugauuguG......................................................... 2         1      S03
    ..................................uuguagcgguuuguaugauuguC......................................................... 2         1      S03
    ..................................uuguagcgguuuguaugauuguuu........................................................ 7         0      S03
    ...................................uguagcgguuuguaugauugC.......................................................... 1         1      S03
    ...................................uguagcgguuuguaugauugu.......................................................... 1         0      S03
    ...................................uguagcgguuuguaugauuguuA........................................................ 3         1      S03
    ...................................uguagcgguuuguaugauuguuu........................................................ 4         0      S03
    ...................................uguagcgguuuguaugauuguuuG....................................................... 1         1      S03
    ...................................uguagcgguuuguaugauuguuuA....................................................... 1         1      S03
    ...................................uguagcgguuuguaugauuguuuc....................................................... 3         0      S03
    ....................................guagcgguuuguaugauu............................................................ 3         0      S03
    ....................................guagcgguuuguaugauug........................................................... 1         0      S03
    ....................................guagcgguuuguaugauugA.......................................................... 1         1      S03
    ....................................Nuagcgguuuguaugauuguu......................................................... 1         1      S03
    ....................................guagcgguuuguaugauuguu......................................................... 6         0      S03
    ....................................guagcgguuuguaugauuguuA........................................................ 1         1      S03
```

```
                                 Mature        Star
aaccagcuuuugauuuuaagagguucagagcggUUUGUAGCGGUUUGUAUGAUUGUUucAAAAUGUUGUCAACUGCUACUAACcgcaaaagcugcguuugcggaucguaccggg
```

```
aaccagcuuuugauuuuaagagguucagagcgguuuguagcgguuuguaugauuguuucaaaauguugucaacugcuacuaaccgcaaaagcugcguuugcggaucguaccggg
....................................guagcgguuuguaugauuguuu........................................................  1  0  S03
....................................guagcgguuuguaugauuguuuG.......................................................  2  1  S03
....................................guagcgguuCguaugauuguuuc.......................................................  1  1  S03
....................................guagcgguuuguaugauuguuuA.......................................................  1  1  S03
.....................................uagcgguuuguaugauugC..........................................................  1  1  S03
.....................................uagcgguuuguaugauuguu.........................................................  1  0  S03
.....................................uagcgguGuguaugauuguuuca......................................................  1  1  S03
.....................................uagcgguuuguaugauuguuucU......................................................  1  1  S03
.....................................uagcgguuuguaugauuguuuca......................................................  4  0  S03
......................................agcgguuuguaugauugu..........................................................  1  0  S03
......................................agcgguuuguaugauuguu.........................................................  1  0  S03
......................................agcgguuuguaugauuguuu........................................................  1  0  S03
......................................agcgguuuguaugauuguuuG.......................................................  2  1  S03
......................................agcgguuuguaugauuguuuA.......................................................  1  1  S03
......................................agcgguuuguaugauuguuuc.......................................................  2  0  S03
......................................agcgguuCguaugauuguuuca......................................................  1  1  S03
......................................agcgguuuguaugauuguuuca......................................................  1  0  S03
..........................................................caaaauguugucaacugcua....................................  1  0  S03
...........................................................aaaauguugucaacugcuacuaac...............................  1  0  S03
................................................................guugucaacugcuacuaacc..............................  1  0  S03

.......uuuugauuuuaagagguucagagc...................................................................................  2  0  S04
..........ugauuuuaagagguucagagc...................................................................................  1  0  S04
..............uuuaagagguucagagcgguuugu............................................................................  1  0  S04
..................agagguucaAagcgguuuguagcg........................................................................  1  1  S04
....................agguucagagcgguuuguagcggu......................................................................  3  0  S04
......................guucagagcgguuuguagcggCu.....................................................................  1  1  S04
........................ucagagcgguuuguagcgguuug...................................................................  1  0  S04
........................ucagagcgguuuguagcgguuugu..................................................................  1  0  S04
...........................gagcgguuuguagcgguuugua.................................................................  1  0  S04
............................agcgguuuguagcgguCu....................................................................  1  1  S04
............................agcgguCuguagcgguuuguaug...............................................................  1  1  S04
.................................uuuguagcgguuuguaugauug...........................................................  4  0  S04
.................................uuuguagcgguuuguaugauuguG.........................................................  2  1  S04
.................................Guuguagcgguuuguaugauuguu.........................................................  1  1  S04
.................................uuuguagcgguuuguaugauuguC.........................................................  1  1  S04
.................................uuuguagcgguuuguaugauuguu.........................................................  12  0  S04
..................................uuguagcgguuuguaugauugu..........................................................  1  0  S04
..................................uuguagcgguuuguaugauuguu.........................................................  2  0  S04
...................................uguagcgguuuguaugauug...........................................................  2  0  S04
...................................uguagcgguuuguaugauuguu.........................................................  1  0  S04
...................................uguagcgguuuguaugauuguuu........................................................  1  0  S04
...................................uguagcgguuuguaugauuguuuc.......................................................  2  0  S04
...................................uguagcgguuuguaugauuguuuA.......................................................  2  1  S04
....................................guagcgguuuguaugauA............................................................  1  1  S04
....................................guagcgguuuguaugauug...........................................................  3  0  S04
....................................guagcgguuuguaugauuguu.........................................................  1  0  S04
....................................guagcgguuuguaugauuguuuA.......................................................  1  1  S04
.....................................uagcgguuuguaugauuguuuc.......................................................  1  0  S04
.....................................uagcgguuuguaugauuguuuca......................................................  11  0  S04
......................................agcgguuuguaugauugu..........................................................  1  0  S04
......................................agcgguuuguaugauuguC.........................................................  1  1  S04
......................................agcgguuuguaugauuguuuc.......................................................  3  0  S04
......................................agcgguuuguaugGuuguuuca......................................................  1  1  S04
......................................agcgguuuguaugauuguuucaa.....................................................  1  0  S04
.......................................gcgguuuguaugauuguuuc.......................................................  1  0  S04
........................................cgAuuuguaugauuguuucaaaau..................................................  1  1  S04
...........................................................aaaauguugucaacugcuacuaac...............................  1  0  S04
..................................................................ugucaacugcuacuaaccgcaaa.........................  1  0  S04
.......................................................................acugcuacuaaccgcaaaagcugc...................  1  0  S04
...........................................................................cuacuaaccgcaaaagcugcguuu...............  1  0  S04
```

Provisional ID	:	chrA08_709000
Score total	:	0
Score for star read(s)	:	-1.3
Score for read counts	:	0
Score for mfe	:	1.3
Score for randfold	:	0
Score for cons. seed	:	
Total read count	:	183
Mature read count	:	178
Loop read count	:	0
Star read count	:	5

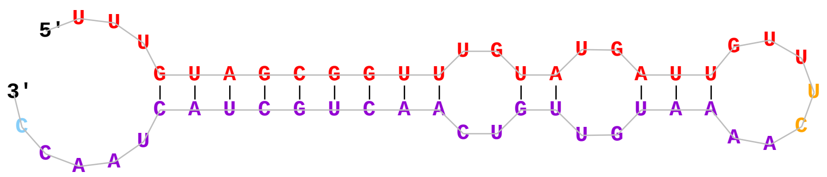

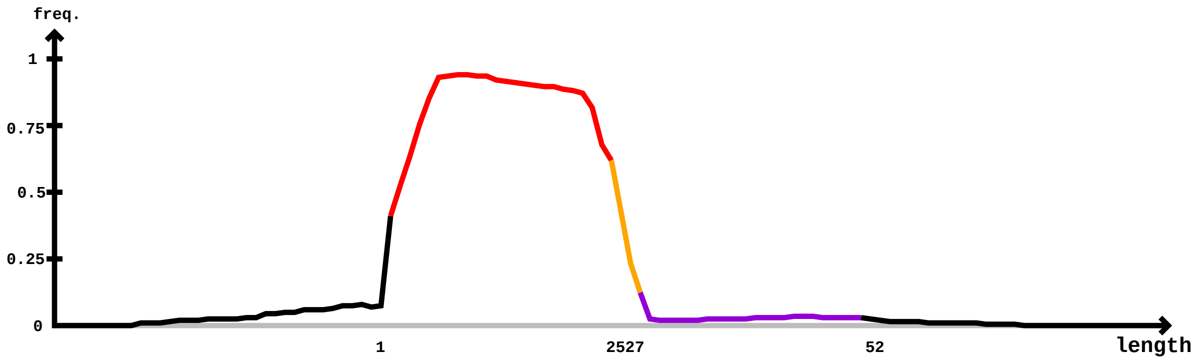

Mature Star

5'-	aaccagcuuuugauuuuaagagguucagagcgguuuguagcgguuuguaugauuguuucaaaauguugucaacugcuacuaaccgcaaaagcugcguuugcggaucguaccggg	-3'	obs	
	aaccagcuuuugauuuuaagagguucagagcgguuuguagcgguuuguaugauuguuucaaaauguugucaacugcuacuaaccgcaaaagcugcguuugcggaucguaccggg		exp	
	(((((((((((((...........)))..((((((.(((((((((..((..(((.......)))..))..))))))))).)))))).))))))).)))..(((......)))..	reads	mm	sample
	...........gauuuuaagagguucagagcggu..	1	0	S03
	..............................cgguuuguagcgguuuguaug...	1	0	S03
	...............................gguuuguagcgguuuguaugauu..	1	0	S03
	................................Uuuuguagcgguuuguaugauugu..	1	1	S03
	.................................uuuguagcgguuuguauga..	1	0	S03
	.................................uuuguagcgguuuguaugau...	2	0	S03
	.................................uuuguagcgguuuguaugauu..	4	0	S03
	.................................uuuguagcgguuuguauCauug...	1	1	S03
	.................................uuuguagcgguuuguaugauug...	16	0	S03
	.................................uuuguagcgguuuguaugauuC...	1	1	S03
	.................................uuuguagcgguuuguaugauuguG...	3	1	S03
	.................................uuuguagcgguuuguaugauuguu...	19	0	S03
	.................................uuuguagcgguuuguaugauuguC...	1	1	S03
	..................................uuguagcgguuuguaugauu..	2	0	S03
	..................................uuguagcgguuuguaugauugu..	2	0	S03
	..................................uuguagcgguuuguaugauugA..	2	1	S03
	..................................uuguagcgguuuguaugauuguu...	4	0	S03
	..................................uuguagcgguuuguaugauuguG...	2	1	S03
	..................................uuguagcgguuuguaugauuguC...	2	1	S03
	..................................uuguagcgguuuguaugauuguuu..	7	0	S03
	...................................uguagcgguuuguaugauugC..	1	1	S03
	...................................uguagcgguuuguaugauugu..	1	0	S03
	...................................uguagcgguuuguaugauuguuA..	3	1	S03
	...................................uguagcgguuuguaugauuguuu..	4	0	S03
	...................................uguagcgguuuguaugauuguuuG...	1	1	S03
	...................................uguagcgguuuguaugauuguuuA...	1	1	S03
	...................................uguagcgguuuguaugauuguuuc...	3	0	S03
	....................................guagcgguuuguaugauu..	3	0	S03
	....................................guagcgguuuguaugauug...	1	0	S03
	....................................guagcgguuuguaugauugA..	1	1	S03
	....................................Nuagcgguuuguaugauuguu...	1	1	S03
	....................................guagcgguuuguaugauuguu...	6	0	S03
	....................................guagcgguuuguaugauuguuA..	1	1	S03

Mature Star

aaccagcuuuugauuuuaagagguucagagcgguuuguagcgguuuguaugauuguuucaaaauguugucaacugcuacuaaccgcaaaagcugcguuugcggaucguaccggg

....................................guagcgguuuguaugauuguuu..	1	0	S03
....................................guagcgguuuguaugauuguuuG...	2	1	S03
....................................guagcgguuCguaugauuguuuc...	1	1	S03
....................................guagcgguuuguaugauuguuuA...	1	1	S03
.....................................uagcgguuuguaugauugC..	1	1	S03
.....................................uagcgguuuguaugauuguu...	1	0	S03
.....................................uagcgguGuguaugauuguuuca..	1	1	S03
.....................................uagcgguuuguaugauuguuucU..	1	1	S03
.....................................uagcgguuuguaugauuguuuca..	4	0	S03
......................................agcgguuuguaugauugu..	1	0	S03
......................................agcgguuuguaugauuguu...	1	0	S03
......................................agcgguuuguaugauuguuu..	1	0	S03
......................................agcgguuuguaugauuguuuG...	2	1	S03
......................................agcgguuuguaugauuguuuA...	1	1	S03
......................................agcgguuuguaugauuguuuc...	2	0	S03
......................................agcgguuCguaugauuguuuca..	1	1	S03
......................................agcgguuuguaugauuguuuca..	1	0	S03
....caaaauguugucaacugcua....................................	1	0	S03
....aaaauguugucaacugcuacuaac...............................	1	0	S03
....guugucaacugcuacuaacc..............................	1	0	S03
.......uuuugauuuuaagagguucagagc...	2	0	S04
..........ugauuuuaagagguucagagc...	1	0	S04
..............uuuaagagguucagagcgguuugu..	1	0	S04
..................agagguucaAagcgguuuguagcg..	1	1	S04
....................agguucagagcgguuuguagcggu..	3	0	S04
......................guucagagcgguuuguagcggCu...	1	1	S04
........................ucagagcgguuuguagcgguuug...	1	0	S04
........................ucagagcgguuuguagcgguuugu..	1	0	S04
...........................gagcgguuuguagcgguuugua...	1	0	S04
............................agcgguuuguagcgguCu..	1	1	S04
............................agcgguCuguagcgguuuguaug...	1	1	S04
.................................uuuguagcgguuuguaugauug...	4	0	S04
.................................uuuguagcgguuuguaugauuguG...	2	1	S04
.................................Guuguagcgguuuguaugauuguu...	1	1	S04
.................................uuuguagcgguuuguaugauuguC...	1	1	S04
.................................uuuguagcgguuuguaugauuguu...	12	0	S04
..................................uuguagcgguuuguaugauugu..	1	0	S04
..................................uuguagcgguuuguaugauuguu...	2	0	S04
...................................uguagcgguuuguaugauug...	2	0	S04
...................................uguagcgguuuguaugauuguu...	1	0	S04
...................................uguagcgguuuguaugauuguuu..	1	0	S04
...................................uguagcgguuuguaugauuguuuc...	2	0	S04
...................................uguagcgguuuguaugauuguuuA...	2	1	S04
....................................guagcgguuuguaugauA..	1	1	S04
....................................guagcgguuuguaugauug...	3	0	S04
....................................guagcgguuuguaugauuguu...	1	0	S04
....................................guagcgguuuguaugauuguuuA...	1	1	S04
.....................................uagcgguuuguaugauuguuuc...	1	0	S04
.....................................uagcgguuuguaugauuguuuca..	11	0	S04
......................................agcgguuuguaugauugu..	1	0	S04
......................................agcgguuuguaugauuguC...	1	1	S04
......................................agcgguuuguaugauuguuuc...	3	0	S04
......................................agcgguuuguaugGuuguuuca..	1	1	S04
......................................agcgguuuguaugauuguuucaa...	1	0	S04
....gcgguuuguaugauuguuuc...	1	0	S04
....cgAuuuguaugauuguuucaaaau..	1	1	S04
....aaaauguugucaacugcuacuaac...............................	1	0	S04
....ugucaacugcuacuaaccgcaaa.........................	1	0	S04
....acugcuacuaaccgcaaaagcugc...................	1	0	S04
....cuacuaaccgcaaaagcugcguuu...............	1	0	S04

Provisional ID	:	chrA08_711459
Score total	:	0.1
Score for star read(s)	:	3.9
Score for read counts	:	-3.9
Score for mfe	:	0.2
Score for randfold	:	0
Score for cons. seed	:	
Total read count	:	4
Mature read count	:	3
Loop read count	:	0
Star read count	:	1

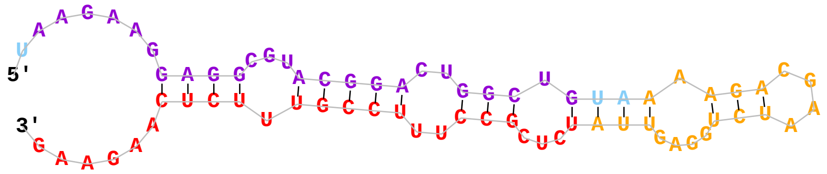

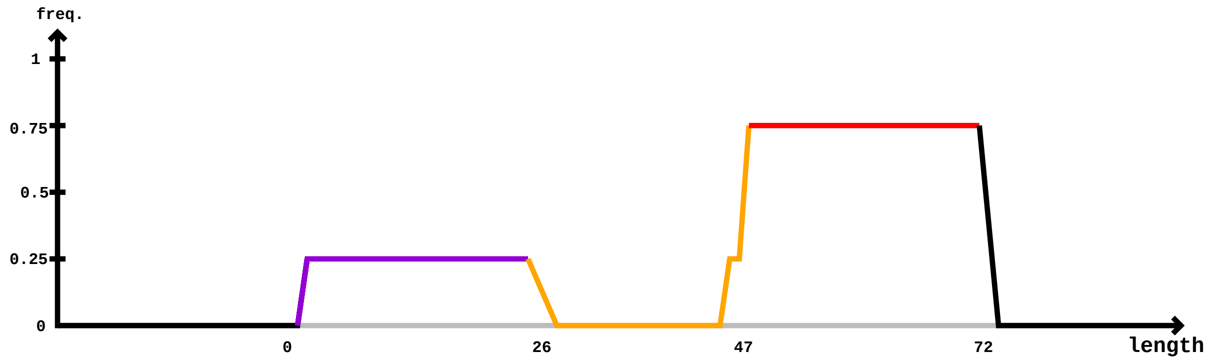

Star Mature

```
5'- uuauccauaaugggagagguccuuaagaaggaggcguacggacuggcuguaaaagacgaaucuggaguuaucucgccuuuccguuucucaagaagguuccaguuuccuuuuaacg   -3'   obs
    uuauccauaaugggagagguccuuaagaaggaggcguacggacuggcuguaaaagacgaaucuggaguuaucucgccuuuccguuucucaagaagguuccaguuuccuuuuaacg         exp
    .......(((.((((((((.((((......((((...(((((..(((.((((.(((....)))....))))...)))..))))).))))...))))..))..)))))).)))...   reads   mm   sample
    ....................................................................uaucucgccuuuccguuucucaagaag....................   1       0    S03

    ........................aagaaggaggcguacggacuggcug..................................................................   1       0    S04
    ......................................................................ucucgccuuuccguuucucaagaag....................   2       0    S04
```

Provisional ID	:	chrA08_714585
Score total	:	0.4
Score for star read(s)	:	-1.3
Score for read counts	:	0
Score for mfe	:	1.7
Score for randfold	:	0
Score for cons. seed	:	
Total read count	:	45
Mature read count	:	45
Loop read count	:	0
Star read count	:	0

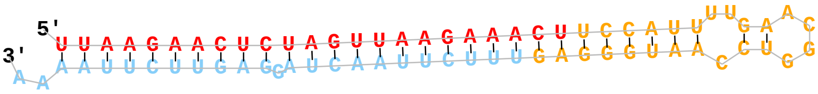

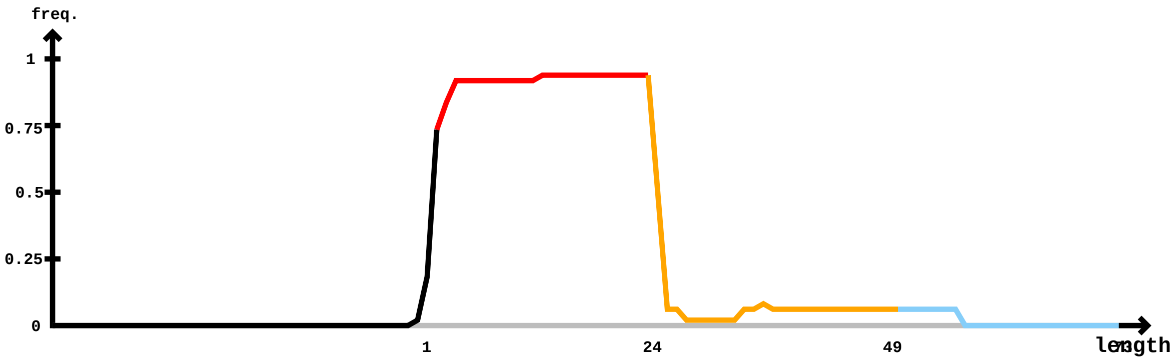

Mature **Star**

```
5'- uaauaauuauuuaauauuuuaaauguacuuaaguggggguuaagaacucuaguuaagaaacuuccauuuugaacgguccaaugggaguuucuuaacuaggaguucuuaaaaaa  -3'    exp
    ......((((((((((.......))).))))))))..((((((((((((((((((((((((((((((..((....)).))))))))))))))))))))).))))))))))....   reads      mm         sample
    .....................................gCuaagaacucuaguuaagaaacu...................................................   1          1          S03
    .....................................guuaagaacucuaguuaagaaacG...................................................   1          1          S03
    .....................................guuaagaacucuaguuaagaaacA...................................................   1          1          S03
    .....................................guuaagaacucuaguuaagaaacu...................................................   1          0          S03
    ......................................uuaagaacucuaguuaagaaacu...................................................   7          0          S03
    ......................................uuaagaacucuaguuaagaaacC...................................................   2          1          S03
    ......................................uuaagaacucuaguuaagaaacG...................................................   1          1          S03
    .......................................uaagaacucuaguuaagaaacu...................................................   2          0          S03
    ........................................aagaacucuaguuaagaaacu...................................................   1          0          S03
    ........................................aagaacucuaguuaagaaacuucc................................................   2          0          S03
    .................................................aguuaagaaacuuccauuuugaac.......................................   1          0          S03
    ......................................................................aacgguccaaugggaguuucuuG...................   1          1          S03
    ........................................................................cgguccaaugggaguuucuua...................   1          0          S03

    ....................................gguuaagaacucuaguuaagaaacu...................................................   1          0          S04
    .....................................guuaagaacucuaguuaagaaacu...................................................   2          0          S04
    .....................................guuaagaacucuaguuaagaaacC...................................................   1          1          S04
    .....................................guuaagaacucuaguuaagaaacA...................................................   1          1          S04
    ......................................uuaGgaacucuaguuaagaaacu...................................................   1          1          S04
    ......................................uuaagaacucuaguuaagaaacu...................................................   16         0          S04
    .......................................uaagaacucuaguuaagaaacu...................................................   2          0          S04
    .......................................uaagaacucuaguuaagaaacuu..................................................   1          0          S04
    ........................................Uagaacucuaguuaagaaacu...................................................   1          1          S04
    ......................................................................aacgguccaaugggaguuucuuG...................   1          1          S04
```

Provisional ID	:	chrA08_731031
Score total	:	311.5
Score for star read(s)	:	3.9
Score for read counts	:	305.5
Score for mfe	:	2.1
Score for randfold	:	0
Score for cons. seed	:	
Total read count	:	611
Mature read count	:	610
Loop read count	:	0
Star read count	:	1

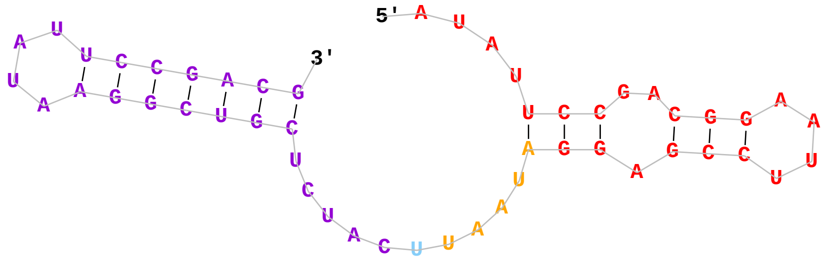

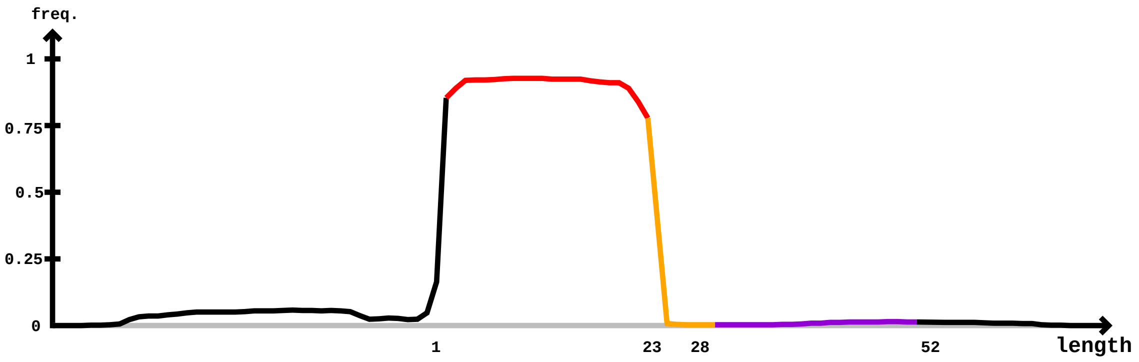

Mature **Star**

```
5'- aggaaacacaaaauuuggcuuccucggaauuuccuuggaauauuccgacggaauuccgaggauaauucaucucgucggaauauuccgacggaauaccgaggaaaauag  -3'   obs
    aggaaacacaaaauuuggcuuccucggaauuuccuuggaauauuccgacggaauuccgaggauaauucaucucgucggaauauuccgacggaauaccgaggaaaauag         exp
    .(....)............((((((((.(((((.((((((((((((((((..((...(((.....)))))..))))))))))))))))).))))).))))))))..... reads   mm   sample
    ......cacaaaauuuggcuuccucggaau............................................................................ 1   0   S03
    .......acaaaauuGggcuuccucggaauu............................................................................. 1   1   S03
    .......acaaaauuuggcuuccucggaauu............................................................................. 2   0   S03
    ..........aaauuuggcuuccucggaauu............................................................................. 1   0   S03
    ...........aauuuggcuuccucggaauuuccu......................................................................... 1   0   S03
    ............auuuggcuuccucggaauuucc.......................................................................... 1   0   S03
    ...................uuccucggaauuuccuug....................................................................... 2   0   S03
    .......................ucAgaauuuccuuggaauauu................................................................ 1   1   S03
    ...........................aauuCccuuggaauauuccgacg.......................................................... 1   1   S03
    ................................ccuuggaauauucUgacggaau...................................................... 1   1   S03
    ................................ccuuggaauauucUgacggaauuc.................................................... 1   1   S03
    .................................cuuggaauauucUgacggaau...................................................... 2   1   S03
    .................................cuuggaauauucUgacggaauu..................................................... 1   1   S03
    ..................................uuggaauauucUgacggaau...................................................... 1   1   S03
    ..................................uuggaauauucUgacggaauu..................................................... 1   1   S03
    ..................................uuggaauauucUgacggaauuc.................................................... 1   1   S03
    ....................................ggaauauucGgacggaauu..................................................... 1   1   S03
    .....................................gaauauuccgacggaauuccga................................................. 2   0   S03
    .....................................gaauauuccgacggaauuccgag................................................ 3   0   S03
    .....................................gaauauuccgacggaauuccgagg............................................... 1   0   S03
    .....................................gaauauuccgacggaauuccgagC............................................... 1   1   S03
    .....................................gaauauuccgacggaauuccgagU............................................... 3   1   S03
    ......................................aauauuccgacggaauuccC.................................................. 1   1   S03
    ......................................aauauuccgacggaauuccg.................................................. 5   0   S03
    ......................................aauauuccgacggaauuccga................................................. 5   0   S03
    ......................................aauauuccgacggaauuccgag................................................ 16  0   S03
    ......................................aauauuccgacggaauuccgagU............................................... 15  1   S03
    ......................................aauauuccgacggaauuccgagg............................................... 2   0   S03
    ......................................aauauuAcgacggaauuccgagga.............................................. 1   1   S03
    ......................................aauauuccgacggaauuccgagga.............................................. 1   0   S03
    .......................................auGuuccgacggaauuccg.................................................. 1   1   S03
    .......................................auauuccgacggaauuccg.................................................. 4   0   S03
    .......................................auauuccgacggGauuccga................................................. 1   1   S03
```

Mature Star

aggaaacacaaaauuuggcuuccucggaauuuccuuggaauauuccgacggaauuccgaggauaauucaucucgucggaauauuccgacggaauaccgaggaaaauag			
....auauuccgacggaauuccga...	2	0	S03
....auauuccgacggaauuccgag..	5	0	S03
....auauuccgacggaauuccgagC...	27	1	S03
....auauuccgGcggaauuccgagg...	1	1	S03
....auauucAgacggaauuccgagg...	1	1	S03
....auauuccgacggaauuccgagU...	273	1	S03
....auauuccgacggaauuccgagg...	26	0	S03
....uauuccgacggaauuccUa...	1	1	S03
....uauuccgacggaauuccgag..	3	0	S03
....uauuccgacggaauuccgagg...	2	0	S03
....uauuccgacggaauuccgagU...	10	1	S03
....uauuccgacggaauuccgagC...	1	1	S03
....uauuccgacggaauuccgagga..	1	0	S03
....auuccgacggaauuccga...	8	0	S03
....auuccgacggaauuccgag..	2	0	S03
....auuccgacggaauuccgagg...	1	0	S03
....auuccgacggaauuccgagU...	4	1	S03
....ccgacggaauuccgagga..	1	0	S03
....cgacggaauuccgaggau...	1	0	S03
....gacggaauucUgaggaua..	1	1	S03
....caucucgucggaauauuccgacA..................	1	1	S03
....gaauauAccgacggaauaccga.........	1	1	S03
....auauuccgacggaauaccgagU.......	1	1	S03
....auuccgacggaauaccgCgg.......	1	1	S03
..gaaacacaaaauuuggcuuccu..	1	0	S04
....aacacaaaauuuggcuuccucgga..	1	0	S04
.....acacaaaauuuggcuuccucggaa...	2	0	S04
......cacaaaauuuggcuuccucg..	1	0	S04
......cacaaaauuuggcuuccucggG..	1	1	S04
......cacaaaauuuggcuuccucggaaC..	3	1	S04
......cacaaaauuuggcuuccucggaau..	4	0	S04
......cacaaaauuGggcuuccucggaau..	1	1	S04
.......acaaaauuuggcuuccucggaauG...	1	1	S04
.......acaaaauuuggcuuccucggaauu...	3	0	S04
........caaaauuuggcuuccucggaau..	1	0	S04
........caaaauuuggcuuccucggaauu...	1	0	S04
..........aaauuuggcuuccucggaauuu..	1	0	S04
..........aaauuuggcuuccucggaauuucc..	1	0	S04
...........aauuuggcuuccucggaauuuc...	1	0	S04
............auuuggcuuccucggaauuucc..	2	0	S04
.............uuuggcuuccucggaauuuccu...	2	0	S04
..................cuuccucggaauuuccuuU...	1	1	S04
......................cucggaauuuccuuggaau...	1	0	S04
............................auuCccuuggaauauuccgacg..	1	1	S04
.....................................gaauauuccgacggaauuccga...	2	0	S04
.....................................gaauauuccgacggaauuccgagg...	1	0	S04
.....................................gaauauuccgacggaauuccgagU...	6	1	S04
......................................aauauuccgacggaauuccg..	1	0	S04
......................................NauauuccgacggaauuccG..	1	1	S04
......................................aauGuuccgacggaauuccga...	1	1	S04
......................................aauauuccgacggaauuccgC...	1	1	S04
......................................aauauuccgacggaauuccga...	4	0	S04
......................................aauauuccgacggaauuccgag..	8	0	S04
......................................aauauuccgacggaauuccgagg...	2	0	S04
......................................aauauuccgacggaauuccgagU...	13	1	S04
......................................aauauuccgacggaauuccgagga..	1	0	S04
....auauuccgacggaauuccga...	2	0	S04
....auauuccgacggaauuccgag..	3	0	S04
....auauuccgacggaauuccgagg...	9	0	S04
....auauuccgacggaauuccgagC...	11	1	S04
....auauuccgacggaauuccgagU...	96	1	S04
....auauuccgacggaauuccgaCgau...	1	1	S04
....uGuuccgacggaauuccg..	1	1	S04
....uauuccgacggaauuccga...	3	0	S04
....uauuccgacggaauuccgagU...	2	1	S04
....auuccgacggaauuccga...	2	0	S04
....auuccgacggaauuccgag..	1	0	S04
....auuccgacggaauuccgagU...	3	1	S04
....uuccgacggaauuccgaCgau...	1	1	S04

```
                                       Mature                    Star
aggaaacacaaaauuuggcuuccucggaauuuccuuggaauauuccgacggaauuccgaggauaauucaucucgucggaauauuccgacggaauaccgaggaaaauag
............................................ccgacggaauuccgagga..............................................	1	0	S04
.............................................cgacggaauuccgaggauaUuu.........................................	1	1	S04
...............................................................aauucaucucgucggaauauuccg.....................	1	0	S04
..........................................................................ucggaauauAccgacggaaua.............	1	1	S04
............................................................................ggaauauuccgacggaCuac............	1	1	S04
.............................................................................gaauauuccgacggaauaccgaggC......	1	1	S04
...............................................................................auauuccgacggaauaccgagU.......	1	1	S04
.....................................................................................cgacggaauaccgaggaaa....	1	0	S04
```

Provisional ID	:	chrA08_733963
Score total	:	0
Score for star read(s)	:	-1.3
Score for read counts	:	-1.3
Score for mfe	:	2.6
Score for randfold	:	0
Score for cons. seed	:	
Total read count	:	9
Mature read count	:	9
Loop read count	:	0
Star read count	:	0

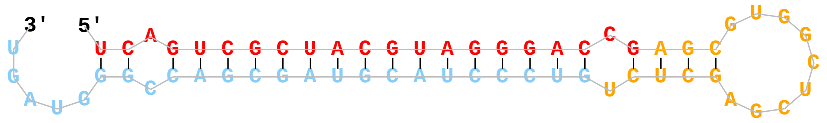

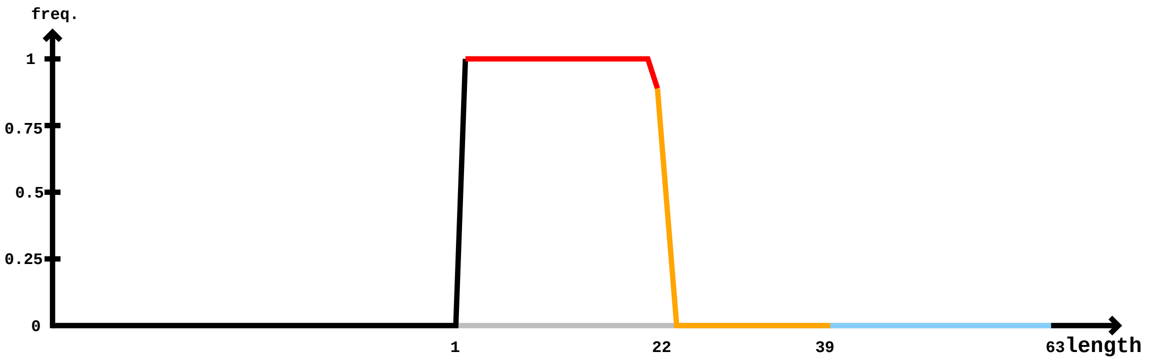

Mature Star

```
5'- gugcgcucggucacuacguagcgaucgagcgggacgugcgcucagucgcuacguagggaccgagcguggcucgagcucugucccuacguagcgaccggguagugugcau   -3'    exp
    ...(((((((((.((....)).)))))))))....(..(((((.((((((((((((((((.((((.........)))).))))))))))))))))...).))))..)..      reads      mm         sample
    .........................................ucagucgcuacguagAgacc...............................................      1          1          S03
    .........................................ucagucgcuacguagAgaccg..............................................      8          1          S03
```

Provisional ID : chrA08_744962
Score total : 0.1
Score for star read(s) : -1.3
Score for read counts : 0
Score for mfe : 1.4
Score for randfold : 0
Score for cons. seed :
Total read count : 27
Mature read count : 27
Loop read count : 0
Star read count : 0

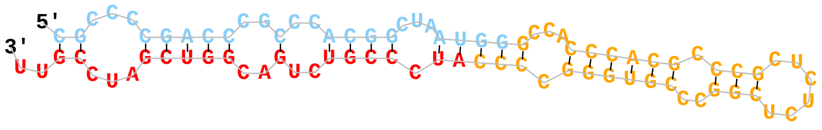

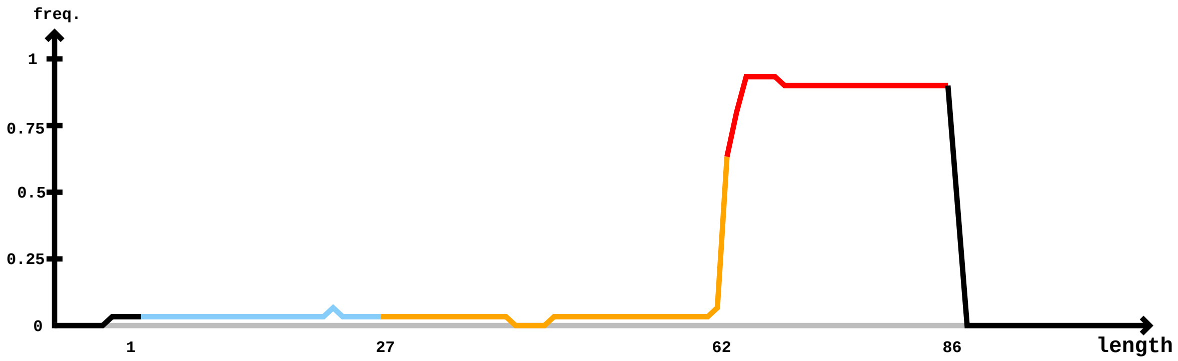

Star Mature

```
5'- cuuauaacgccccgacccgcccacggcuaaugggccacccacgcccgcucucucggcccgugggccccaucccgucugacggucgauccguuaauuuucuaaaggcucgaaa   -3'    exp
    ....(((((...(((((..(..((((...(((((...((((((.(((......)))..)))))).))))).)))).)..)))))...)))))...................   reads    mm    sample
    ....................................................................aucccgucugacggucgauccguu....................   8        0     S03
    ....................................................................aucccgucugacggucgauccguC....................   1        1     S03
    ....................................................................aucccgucugacggucgauccguG....................   1        1     S03
    .....................................................................ucccgucugacggucgauccguu....................   1        0     S03
    .....................................................................ucccgucugacggucgauccguua...................   1        0     S03
    ......................................................................cccgucugacggucgauccguua...................   1        0     S03

    ....uaacgccccgacccgcccacggcC....................................................................................   1        1     S04
    ...........................uaaugggccacccacgccG..................................................................   1        1     S04
    ..................................................ucucggccUgugggccccaucccg......................................   1        1     S04
    ...................................................................caucccgucugacggucgauccguu....................   1        0     S04
    ....................................................................aucccgucugacggucgauccguC....................   3        1     S04
    ....................................................................aucccgucugacggucgauccguu....................   4        0     S04
    .....................................................................ucccgucugacggucgauccguu....................   3        0     S04
    ......................................................................cccgucugacggucgauccguu....................   1        0     S04
    ......................................................................cccgucugacggucgauccguuU...................   1        1     S04
    ......................................................................cccgucugacggucgauccguua...................   1        0     S04
```

Provisional ID	:	chrA08_756509
Score total	:	0.2
Score for star read(s)	:	-1.3
Score for read counts	:	0
Score for mfe	:	1.5
Score for randfold	:	0
Score for cons. seed	:	
Total read count	:	714
Mature read count	:	713
Loop read count	:	1
Star read count	:	0

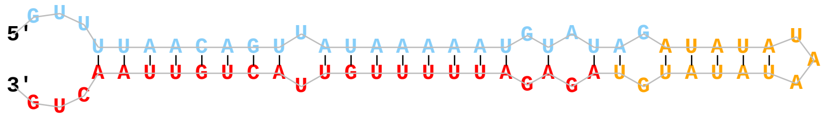

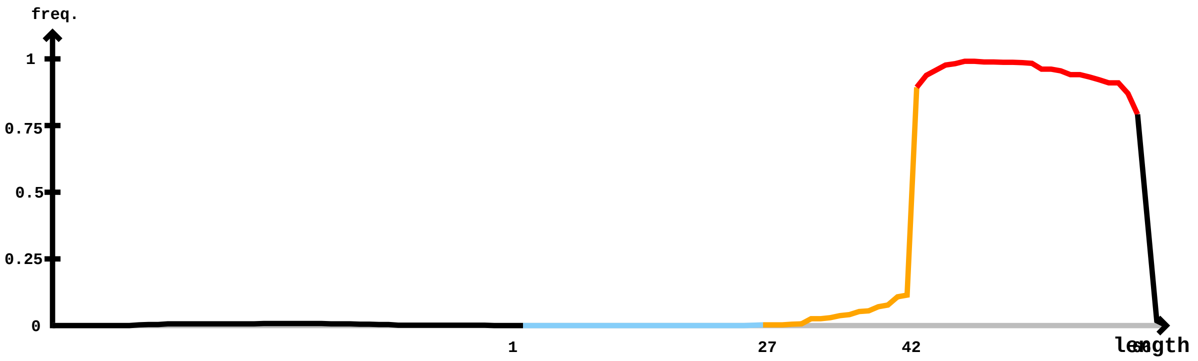

Star Mature

```
5'- ugacugcgguuuaagcggugcggaacaagcgauucaacugcagugcaguuuuaacaguuauaaaaauguauagauauauaauauauguagagauuuuuguuacuguuaacugca  -3'   exp
    ..(((((((((.((....(((.......))).)).)))))))))(((((..(((((((.((((((((.(.((.(((((...))))).)).).)))))))).))))))))))))).   reads   mm   sample
    .......gguuuaagcggugcggaacaagc....................................................................................   1   0   S03
    ....................cggaacaagcgauucaacugcagu......................................................................   1   0   S03
    .......................................................................agauauauaauauauguagagauu...................   1   0   S03
    .............................................................................auaauauauguagagauuuuugu..............   6   0   S03
    ................................................................................auauauguagagauuuuugu..............   1   0   S03
    ................................................................................aAauauguagagauuuuuguuac...........   1   1   S03
    .................................................................................uauauguagagauuuuuguuacu..........   1   0   S03
    ..................................................................................auauguagagauuuuuguuacu..........   3   0   S03
    ..................................................................................auauguagagauuuuuguuacugu........   1   0   S03
    ..................................................................................auauguagagauuuuuguuaAugu........   1   1   S03
    ...................................................................................uauguagagauuuuuguuacuguu.......   1   0   S03
    ....................................................................................auguagagauuuuuguuacu..........   2   0   S03
    ....................................................................................auguagagauuuuuguuacuguua......   2   0   S03
    ....................................................................................auguagagauuuuuguuacugGua......   1   1   S03
    .....................................................................................uguagagauuuuuguuacuguua......   3   0   S03
    ......................................................................................guagagauuuuuguuacuguuaac....   9   0   S03
    .......................................................................................uagagauuuuuguuacuguuaacu...   2   0   S03
    .......................................................................................Gagagauuuuuguuacuguuaacu...   1   1   S03
    ........................................................................................agagauuuuuguuacugu........   1   0   S03
    ........................................................................................agagauuuuuguuacuguuaac....   5   0   S03
    ........................................................................................agagauuuuuguuacuguuaacu...   21   0   S03
    ........................................................................................agagauuuAuguuacuguuaacug..   1   1   S03
    ........................................................................................agagauuuuuguuacuguuaacuC..   1   1   S03
    ........................................................................................agagauCuuuguuacuguuaacug..   5   1   S03
    ........................................................................................agagauuuuuUuuacuguuaacug..   1   1   S03
    ........................................................................................Ggagauuuuuguuacuguuaacug..   2   1   S03
    ........................................................................................agagauuuuGguuacuguuaacug..   1   1   S03
    ........................................................................................agagauGuuuguuacuguuaacug..   1   1   S03
    ........................................................................................agagauuuuuguuacugCuaacug..   1   1   S03
    ........................................................................................agagauuuuuguuacuguuaacug..   173   0   S03
    ........................................................................................agagauuuuuguuacuguuaacugc.   1   0   S03
    .........................................................................................gagauuuuuguuacuguuaacu...   5   0   S03
    .........................................................................................gagauuuuuguuacuguuaacug..   2   0   S03
    .........................................................................................gagauuuuuguuacuguuaacugc.   6   0   S03
```

```
                                               Star                                     Mature
ugacugcgguuuaagcggugcggaacaagcgauucaacugcagugcaguuuuaacaguuauaaaaauguauagauauauaauauauguagagauuuuuguuacuguuaacugca
..........................................................................................agauGuuuguuacuguuaacug..   1     1   S03
..........................................................................................agauuuuuguuacuguuaacuC..   1     1   S03
..........................................................................................agauuuuuguuacuguuaacugc.   1     0   S03
..........................................................................................agauuuuuguuacuguuaacugca   4     0   S03
...........................................................................................gauuuuuguuacuguuaacug..   2     0   S03
...........................................................................................gauuuuuguuacuguuaacugc.   1     0   S03
...........................................................................................gauuuuuguuacuguuaacugca   2     0   S03
...........................................................................................gauuuuuguuacuguuaacuUca   1     1   S03
............................................................................................auuuuuguuacuguuaacugc.   1     0   S03
.............................................................................................uuuuuguuacuguuaacugc.   1     0   S03
.............................................................................................uuuuuguuacuguuaacugGa   1     1   S03
.............................................................................................uuuuuguuacuguuaacugca   1     0   S03

.......gguuuaagcggugcggaaca.......................................................................................   1     0   S04
........guuuaagcggugcAgaacaagcga..................................................................................   1     1   S04
..........uuaagcggugcggGacaagcgauu................................................................................   2     1   S04
........................................................................gauauauaauauauguagaga.....................   1     0   S04
...........................................................................auauaauauauguagagauuuu.................   1     0   S04
...........................................................................auauaauauauguagagauuuuug...............   1     0   S04
............................................................................uauaauaCauguagagauuuuugu..............   1     1   S04
.............................................................................auaauauauguagagauu...................   1     0   S04
.............................................................................auaauauauguagagauuuuuguC.............   1     1   S04
.............................................................................auaauauauguagagauuuuuguG.............   1     1   S04
.............................................................................auaauauauguagagauuuuuguu.............   6     0   S04
...............................................................................aauauauguagagauuuuuguuac...........   1     0   S04
...............................................................................aauauauguagagauuuuuguuaU...........   2     1   S04
................................................................................auauauguagagauuuuuguu.............   2     0   S04
................................................................................auauauguagagauuuuuguuacu..........   2     0   S04
.................................................................................uauauguagagauuuuuguuac...........   1     0   S04
.................................................................................uauauguagagauuuuuguuacu..........   1     0   S04
..................................................................................auauguagagauuuuugG..............   1     1   S04
..................................................................................auauguagagauuuuuguuacu..........   1     0   S04
..................................................................................auauguagagauuuuuguuacugu........   1     0   S04
..................................................................................auauguagagauuuuAguuacugu........   1     1   S04
...................................................................................uauguagagauuuuuguuacuguu.......   1     0   S04
....................................................................................auguagagauuuuuguuacu..........   1     0   S04
....................................................................................auguagagauuuuuguuacuguu.......   2     0   S04
....................................................................................auguagagauuuuuguuacuguC.......   2     1   S04
....................................................................................auguagagauuuuuguuacuguua......   1     0   S04
....................................................................................auguagGgauuuuuguuacuguua......   1     1   S04
.....................................................................................uguagagauuuuuguuacuguu.......   1     0   S04
.....................................................................................uguagagauuuuuguuacuguua......   1     0   S04
......................................................................................guagagauuuuuguuacugu........   2     0   S04
......................................................................................guagagauuuuuguuacuguuaaA....   1     1   S04
......................................................................................guagagauuuuuguuUcuguuaac....   1     1   S04
......................................................................................guagagauuuuuguuacuguuaac....   11    0   S04
.......................................................................................uagagauuuuuguuacuguuaacu...   2     0   S04
........................................................................................agagauuuuuguuacuguu.......   1     0   S04
........................................................................................Ggagauuuuuguuacuguuaac....   1     1   S04
........................................................................................agagauuuuuguuacuguuaac....   3     0   S04
........................................................................................agagauuuuuguuacuguuaacu...   21    0   S04
........................................................................................agGgauuuuuguuacuguuaacug..   1     1   S04
........................................................................................Cgagauuuuuguuacuguuaacug..   1     1   S04
........................................................................................Ggagauuuuuguuacuguuaacug..   8     1   S04
........................................................................................agagauuuuCguuacuguuaacug..   2     1   S04
........................................................................................agagauuuuuguuacuguCaacug..   1     1   S04
........................................................................................agagauuuuuguuacuguuaacug..   341   0   S04
........................................................................................agagauuuuuguuNcuguuaacug..   1     1   S04
........................................................................................agagauuuuuguuacuguuaacuC..   3     1   S04
........................................................................................Ngagauuuuuguuacuguuaacug..   2     1   S04
........................................................................................agagauuuuuguuacugCuaacug..   2     1   S04
........................................................................................agagauuCuuguuacuguuaacug..   2     1   S04
........................................................................................agagauuuuuguuacuguuaacugc.   3     0   S04
.........................................................................................gagauuuuuguuacuguuaacu...   7     0   S04
.........................................................................................gagauuuuuguuacuguuaacug..   4     0   S04
.........................................................................................gagauuuuuguuacuguuaacugc.   11    0   S04
..........................................................................................agauuuuuguuacuguuaacu...   1     0   S04
..........................................................................................agauuuuuguuacuguuaacug..   2     0   S04
..........................................................................................agauuuuuguuacuguuaacugc.   2     0   S04
..........................................................................................agauGuuuguuacuguuaacugca   1     1   S04
```

```
                                               Star                                     Mature
ugacugcgguuuaagcggugcggaacaagcgauucaacugcagugcaguuuuaacaguuauaaaaauguauagauauauaauauauguagagauuuuuguuacuguuaacugca
..........................................................................................agauuuuuguuacuguuaacugca	2	0	S04
...........................................................................................gauuuuuguuacuguuaacug..	6	0	S04
...........................................................................................gauuuuuguuacuguuaacugc.	2	0	S04
...........................................................................................gauuuuuguuacuguuaacugca	1	0	S04
............................................................................................auuuuuguuacuguuaacug..	2	0	S04
............................................................................................auuuuuguuacuguuaacugc.	1	0	S04
.............................................................................................uuuuuguuacuguuaacu...	1	0	S04
.............................................................................................uuuuuguuacuguuaacugc.	3	0	S04
.............................................................................................uuuuuguuacuguuaacugca	1	0	S04
```

Provisional ID	:	chrA08_759096
Score total	:	0.6
Score for star read(s)	:	-1.3
Score for read counts	:	0
Score for mfe	:	1.9
Score for randfold	:	0
Score for cons. seed	:	
Total read count	:	14
Mature read count	:	14
Loop read count	:	0
Star read count	:	0

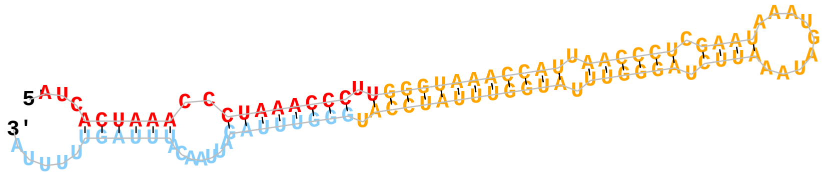

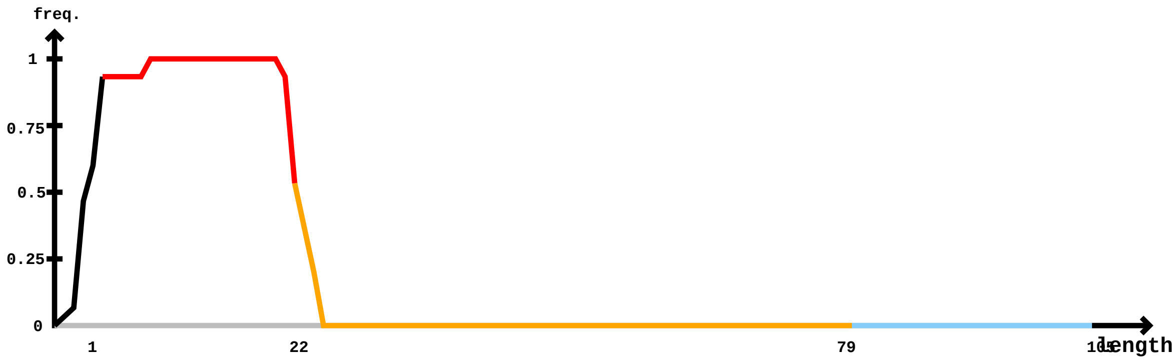

Mature **Star**

```
5'- cuaaucacuaaacccuaaacccuuggguaaaccauuaacccucgaauaaaugauaaauucuaggguuuaugguuuauccaugggguuuagauuaacauuuaguuuuuaauauu -3'   exp
    .(((..((((((..((((((((.((((((((((((.((((((.((((.........)))).)))))).)))))))))))).))))))))).......))))))..))).....   reads   mm   sample
    cuaaucaGuaaacccuaaacccu.........................................................................................   1       1    S04
    .uaaucacuaaacccuaaaccc..........................................................................................   1       0    S04
    .uaaucaGuaaacccuaaacccu.........................................................................................   2       1    S04
    .uaaucacuaaacccuaaacccu.........................................................................................   2       0    S04
    .uaaucacuaaacccuaaacccuug.......................................................................................   1       0    S04
    ..aaucacuaaacccuaaacccu.........................................................................................   1       0    S04
    ..aaucacuaaacccuaaacccuugg......................................................................................   1       0    S04
    ...aucacuaaacccuaaacccuu........................................................................................   3       0    S04
    ...aucacuaaacccuaaacccuug.......................................................................................   1       0    S04
    ...aucacuaaacccuaaacccuugg......................................................................................   1       0    S04
    ........uaaacccuaaacccuugg......................................................................................   1       0    S04
```

Provisional ID	:	chrA08_random_2753265
Score total	:	0.2
Score for star read(s)	:	-1.3
Score for read counts	:	0
Score for mfe	:	1.5
Score for randfold	:	0
Score for cons. seed	:	
Total read count	:	60
Mature read count	:	53
Loop read count	:	7
Star read count	:	0

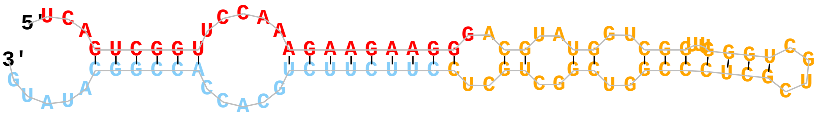

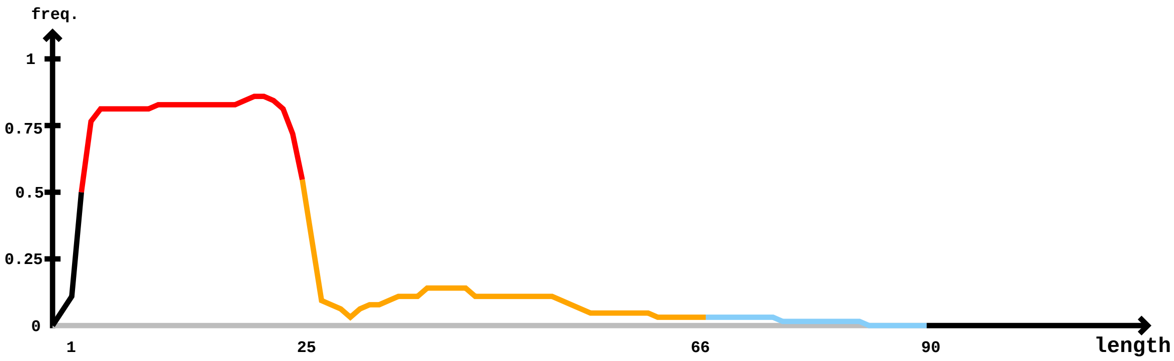

Mature Star

```
5'- uucagucgguuccaaagaagaagggacguauggucgguuugggucgucgcucccggucggcugcuccuucuucugcaccaccggcauauguugauccagaaguucuuacggc   -3'    exp
    ....((((((.....(((((((((..((..((..(((...((((....)))))))..))..))..))))))))).....))))))...........................   reads      mm        sample
    uucagucgguuccaaagaagaa..........................................................................................   1          0         S03
    .ucagucgguuccaaagaagG...........................................................................................   1          1         S03
    .ucagucgguuccaaagaagaag.........................................................................................   4          0         S03
    .ucagucgguuccaaagaagaagg........................................................................................   1          0         S03
    .ucagucgguuccaaagaagaaggA.......................................................................................   1          1         S03
    .ucagucgguuccaaagaagaaggg.......................................................................................   5          0         S03
    ..cagucgguuccaaagaagaag.........................................................................................   1          0         S03
    ..cagucgguuccaaagaagaaggga......................................................................................   4          0         S03
    ..Gagucgguuccaaagaagaaggga......................................................................................   1          1         S03
    ..cagucgguuccaaagaagaagggacA....................................................................................   1          1         S03
    ...agucgguuccaaagaagaaggga......................................................................................   1          0         S03
    ...agucgguuccaaagaagaagggac.....................................................................................   1          0         S03
    ...................gaagggacguauggucgguuugA......................................................................   1          1         S03
    ..............................uggucgguuugggucgucgcuc............................................................   1          0         S03
    ...............................gUucgguuugggucgucgcu.............................................................   1          1         S03
    .................................ucgguuugggucgucgcucU...........................................................   1          1         S03
    .............................................................ugcuccuucuucugcaccaccA.............................   1          1         S03

    uucagucgguuccaaagaagaagg........................................................................................   4          0         S04
    uucagucgguuccaaGgaagaagg........................................................................................   1          1         S04
    uucagucgguuccaaagaagaagggGcgu...................................................................................   1          1         S04
    .ucagucgguuccaaagaagaag.........................................................................................   1          0         S04
    .ucagucgguuccaaagaagaagg........................................................................................   1          0         S04
    .ucagucgguuccaaagaagaaggg.......................................................................................   11         0         S04
    ..cagucgguuccaaagaagaa..........................................................................................   1          0         S04
    ..cagucgguuccaaagaagaagg........................................................................................   3          0         S04
    ..cagucgguuccaaGgaagaaggg.......................................................................................   1          1         S04
    ..cagucgguuccaaagaagaaUgga......................................................................................   1          1         S04
    ..cagucgguuccaaagaagaaggga......................................................................................   4          0         S04
    ...agucgguuccaaagaagaagg........................................................................................   1          0         S04
    .........uuccaaagaagaagggacgu...................................................................................   1          0         S04
    ..................agaaggAacguauggucgguuugg......................................................................   1          1         S04
    ..............................uggucgguuugggucgucgcuccc..........................................................   1          0         S04
    ..................................cgguuugggucgucgcucA...........................................................   1          1         S04
```

Mature **Star**

```
uucagucgguuccaaagaagaagggacguauggucgguuugggucgucgcucccggucggcugcuccuucuucugcaccaccggcauauguugauccagaaguucuuacggc
.....................................uuugggucgucgcucccggucggc...................................................	2	0	S04
.....................................................cggucggcugUuccuucuucu......................................	1	1	S04
```

Provisional ID	:	chrA09_774684
Score total	:	293.7
Score for star read(s)	:	3.9
Score for read counts	:	287.7
Score for mfe	:	2.1
Score for randfold	:	0
Score for cons. seed	:	
Total read count	:	576
Mature read count	:	475
Loop read count	:	0
Star read count	:	101

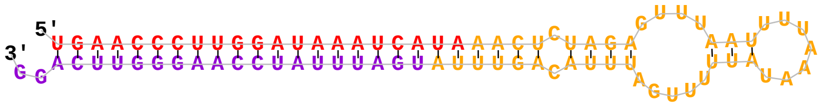

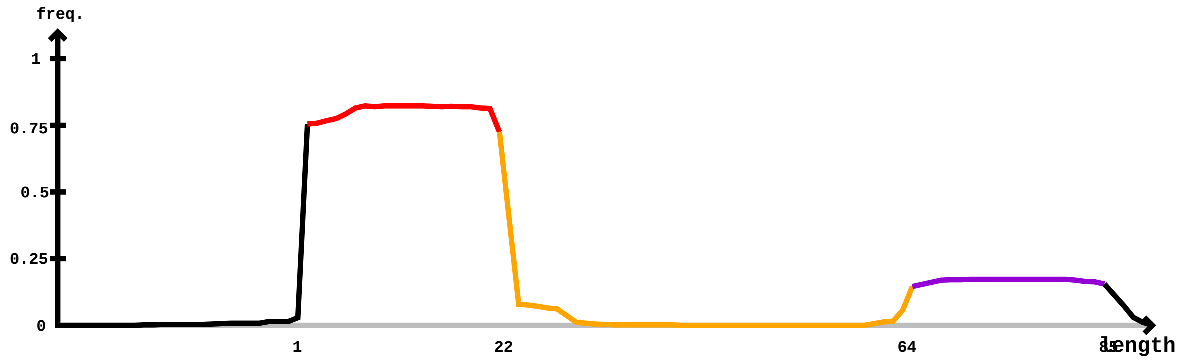

Mature Star

```
5'- aacccuaaacccuuggguaaacacugaacccuuggauaaaucauaaacucuagaguuuaauuuuaaauauuuuugauuuacaguuuaugauuuauccaaggguucaggguuu  -3'   obs
    aacccuaaacccuuggguaaacacugaacccuuggauaaaucauaaacucuagaguuuaauuuuaaauauuuuugauuuacaguuuaugauuuauccaaggguucaggguuu        exp
    .((((.........))))((((.((((((((((((((((((((((((.((((....(((.......))).....)))).)))))))))))))))))))))))).))))   reads   mm   sample
    .......aacccuuggguaaacacugaaccG.................................................................................   1   1   S03
    ................guaaacacugaacccuuggauaaa........................................................................   1   0   S03
    .......................cugaacccuuggauaaaucau....................................................................   1   0   S03
    .......................cugaacccuuggauaaaucauaaa.................................................................   1   0   S03
    ........................ugaacccuuggauaaaucau....................................................................   1   0   S03
    ........................ugaacccuugAauaaaucau....................................................................   22   1   S03
    ........................ugaacccuuggauaaaucaua...................................................................   3   0   S03
    ........................ugaacccuugAauaaaucaua...................................................................   136   1   S03
    ........................ugaacccuugUauaaaucaua...................................................................   1   1   S03
    ........................ugaacccuuggauaaaucauaaac................................................................   1   0   S03
    ..........................aacccuuggauaaaucauaaacu...............................................................   1   0   S03
    ..........................aGcccuuggauaaaucauaaacu...............................................................   1   1   S03
    ..........................aacccuuggGuaaaucauaaacuc..............................................................   1   1   S03
    ..........................aacccuuggauaaaucauaaacucu.............................................................   1   0   S03
    ............................cccuuggauaaaucauaaacucC.............................................................   1   1   S03
    ............................cccuuggauaaaucauaaacucua............................................................   2   0   S03
    .............................ccuuggauaaaucauaaacucu.............................................................   2   0   S03
    .............................ccuuggauaaaucauaaacucua............................................................   5   0   S03
    .............................ccuuggauaaaucauaaacucuaU...........................................................   1   1   S03
    ..............................cuuggauaaaucauaaacucuaga..........................................................   2   0   S03
    ......................................................................................augauuuauccaaggguucag.....   2   0   S03
    ......................................................................................augauuuauccaaAgguucagggu..   1   1   S03
    ......................................................................................augGuuuauccaaggguucagggu..   1   1   S03
    ......................................................................................augauuuauccaaggguucagggu..   8   0   S03
    .......................................................................................ugauuuauccaaggguucag.....   1   0   S03
    .......................................................................................ugCuuuauccaaggguucagg....   1   1   S03
    .......................................................................................ugauuuauccaaggguucagg....   14   0   S03
    .......................................................................................ugauuuauccaaggguucaggguG.   1   1   S03
    .........................................................................................auuuauccaaggguucagggu..   1   0   S03
    .........................................................................................auuuauccaaggguucaggguC.   1   1   S03
    ..........................................................................................uuuauccaaggguucaggguu.   1   0   S03
    ..........................................................................................uuuauccaaggguucaggguuu   1   0   S03

    .........cccuuggguaaacacugaaccc.................................................................................   1   0   S04
```

```
                       Mature                                                      Star
aacccuaaacccuuggguaaacacugaacccuuggauaaaucauaaacucuagaguuuaauuuuaaauauuuuugauuuacaguuuaugauuuauccaaggguucaggguuu
..............ggguaaacacugaacccuuggaua..........................................................................  1    0    S04
...............gguaaacacugaacccCuggau...........................................................................  1    1    S04
....................acacugaacccuuggauaaauc......................................................................  1    0    S04
....................acacugaacccuuggauaaaucau....................................................................  3    0    S04
.......................cugaacccuuggauaaaucaua...................................................................  3    0    S04
.......................cugaacccuuggauaaaucauCa..................................................................  1    1    S04
.......................cugaacccuuggauaaaucauaa..................................................................  1    0    S04
.......................cugaacccuuggauaaaucauaU..................................................................  1    1    S04
.......................cugaacccuuggauaaaucauaaa.................................................................  1    0    S04
........................ugaacccuugAauaaauc......................................................................  2    1    S04
........................ugaacccuugAauaaauca.....................................................................  1    1    S04
........................ugaacccuugAauaaaucau....................................................................  29   1    S04
........................ugaacccuugAauaaaucaua...................................................................  249  1    S04
........................ugaacccuuggauaaaucaua...................................................................  12   0    S04
........................ugaacccuuggauaaaucauaaac................................................................  2    0    S04
.........................gaacccuuggauaaaucauaaacu...............................................................  2    0    S04
..........................aacccuuggauaaaucaua...................................................................  1    0    S04
..........................aacccuuggauaaaucauaaacucu.............................................................  1    0    S04
...........................acccuuggauaaaucauaaacucC.............................................................  1    1    S04
...........................acccuuggauaaaucauaaacucu.............................................................  4    0    S04
............................cccuuggauaaaucauaaacucu.............................................................  3    0    S04
............................cccuuggauaaaucauaaacucua............................................................  4    0    S04
............................cccuuggauaaaucauaaacucuC............................................................  1    1    S04
.............................ccuuggauaaaucauaaacuc..............................................................  1    0    S04
.............................ccuuggauaaaucauaaacucC.............................................................  1    1    S04
.............................ccuuggauaaaucauaaacucua............................................................  4    0    S04
..............................cuuggauaaaucauaaacucu.............................................................  1    0    S04
..............................cuuggauaaaucauaaacucA.............................................................  1    1    S04
..............................cuuggauaaaucauaaacucuaAag.........................................................  1    1    S04
................................uggGuaaaucauaaacucuag...........................................................  1    1    S04
................................uggauaaaucauaaacucuagagu........................................................  1    0    S04
.......................................aucauaaacucuagagCuuaauuu.................................................  1    1    S04
...................................................................................uuuaugauuuauccaaggguu........  2    0    S04
...................................................................................uuuaugauuuauccaaggguuc.......  1    0    S04
...................................................................................uuuaugauuuauccaaggguucag.....  1    0    S04
....................................................................................uuaugauuuauccaaggguuc.......  2    0    S04
....................................................................................uuaugauuuauccaaggguucagg....  2    0    S04
.....................................................................................uaugauuuauccaaggguucaggU...  1    1    S04
.....................................................................................uaugauuuauccaaggguucaggg...  1    0    S04
......................................................................................augauuuauccaaggguucag.....  1    0    S04
......................................................................................augauuuauccaaggguucagggu..  8    0    S04
......................................................................................augauuuauccaaggguucagggC..  1    1    S04
......................................................................................augauuuauccaaggguucagggA..  1    1    S04
......................................................................................augauuuauccaGggguucagggu..  1    1    S04
......................................................................................augauuuauccaaggguCcagggu..  1    1    S04
......................................................................................augauuuauccaaggguucaggguu.  1    0    S04
.......................................................................................ugauuuauccaaggguucagg....  31   0    S04
.......................................................................................ugauuuauccaaggguucGgg....  1    1    S04
.......................................................................................ugauuuauccaaggguucagggC..  1    1    S04
.......................................................................................ugauuuauccaaggguucagggu..  3    0    S04
.......................................................................................ugauuuauccaaggguucaggguu.  2    0    S04
.......................................................................................ugauuuauccaaggguucaggguG.  1    1    S04
........................................................................................gauuuauccaaggguucG......  1    1    S04
........................................................................................gauuuauccaaggguucaggguuC  1    1    S04
........................................................................................gauuuauccaaggguucaggguuu  2    0    S04
........................................................................................gauuuauccaGggguucaggguuu  1    1    S04
.........................................................................................auuuauccaaggguucagggu..  1    0    S04
.........................................................................................auuuaucGaaggguucaggguu.  2    1    S04
..........................................................................................uuuauccaaggguucaggguu.  1    0    S04
..........................................................................................uuuauccaaggguucaggguC.  1    1    S04
..........................................................................................uuuaucGaaggguucaggguuu  1    1    S04
...........................................................................................uuauccaaggguucaggguuu  1    0    S04
.............................................................................................auccaaggguucaggguC.  1    1    S04
```

Provisional ID	:	chrA09_780201
Score total	:	0.6
Score for star read(s)	:	3.9
Score for read counts	:	-4.9
Score for mfe	:	1.6
Score for randfold	:	0
Score for cons. seed	:	
Total read count	:	2
Mature read count	:	1
Loop read count	:	0
Star read count	:	1

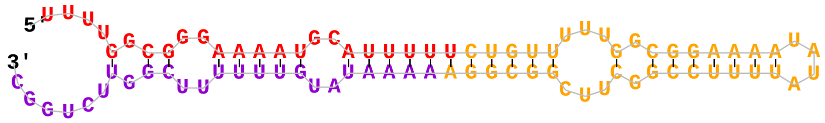

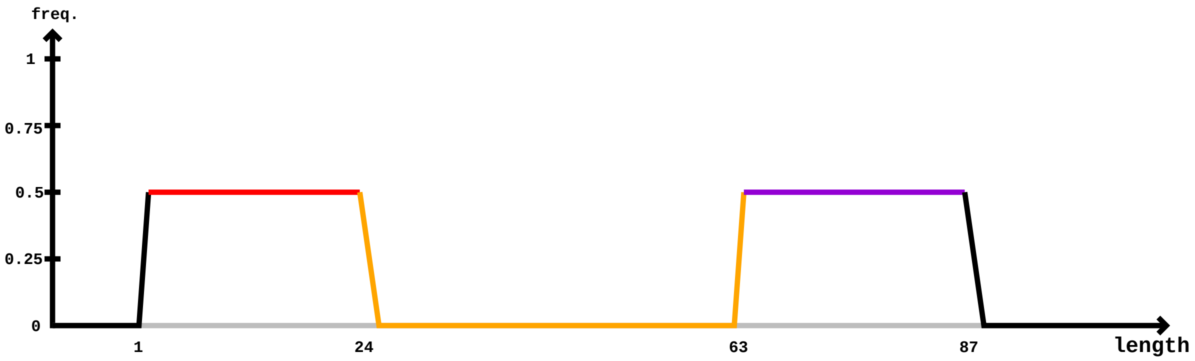


```
             Mature                                                     Star
5'- uuuugcguuuuuggcgggaaaaugcauuuuucuguuuuuggcggaaaauauauuuuccggcuucggcggaaaaauauguuuuuucgguucuggcgggaaaauacguuuuuuccg   -3'    obs
    uuuugcguuuuuggcgggaaaaugcauuuuucuguuuuuggcggaaaauauauuuuccggcuucggcggaaaaauauguuuuuucgguucuggcgggaaaauacguuuuuuccg          exp
    .....((((...(.((..(((((..(((((((((((...(.(((((((....))))))).)...)))))))))))..)))))..)).)...))))((((((......)))))).   reads      mm         sample
    ......................................................................aaaauauguuuuuucgguucuggc....................   1          0          S03

    ........uuuuggcgggaaaaugcauuuuu...................................................................................   1          0          S04
```

Provisional ID	:	chrA09_788111
Score total	:	0.2
Score for star read(s)	:	-1.3
Score for read counts	:	0
Score for mfe	:	1.5
Score for randfold	:	0
Score for cons. seed	:	
Total read count	:	154
Mature read count	:	154
Loop read count	:	0
Star read count	:	0

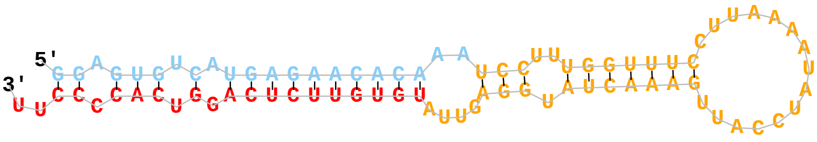

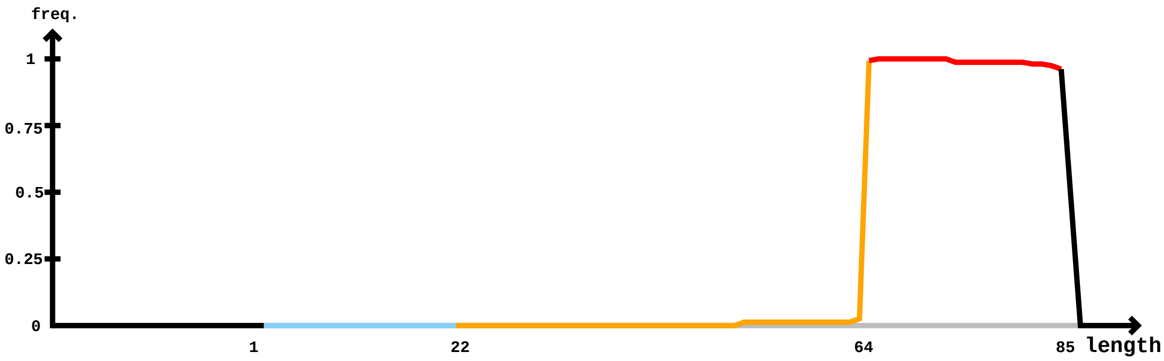

Star Mature

```
5'- acaaguggugaaaucucaaaggagugucaugagaacacaaauccuuugguuuccuuaaaauauccauugaaacuauggaguuauguguucucaggucaccccuucgaguuc  -3'   exp
    .............(((.((((.(((.(.((((((((((..(((..(((((((................))))))).)))....))))))))))).).))).)))).)))...   reads   mm   sample
    ......................................................................aacuauggaguuauguguucuc...................   1       0    S03
    ......................................................................aacuauggaguuauguguucuU...................   1       1    S03
    ..................................................................................auguguucucagguUacc...........   1       1    S03
    ..................................................................................auguguucucaggucaccccuG.......   1       1    S03
    ...................................................................................uguguucucaggucacccA.........   1       1    S03
    ...................................................................................uguguucucaggucaccccuG.......   62      1    S03
    ...................................................................................uguguucucaggucaccccuC.......   2       1    S03
    ...................................................................................uguguucucaggucaccccuu.......   3       0    S03
    ...................................................................................uguguucucaggucaccccuA.......   5       1    S03

    ...................................................................................uguguucucaggucaccccu........   1       0    S04
    ...................................................................................uguguucucaggucaccccuC.......   2       1    S04
    ...................................................................................uguguucucaggucaccccuG.......   63      1    S04
    ...................................................................................uguguucucaggucaccccuA.......   4       1    S04
    ...................................................................................uguguucucaggucaccccuu.......   7       0    S04
    ...................................................................................uguguucucaggucaccccCu.......   1       1    S04
    ....................................................................................guguucucaggucaccccu........   1       0    S04
```

Provisional ID	:	chrA09_793296
Score total	:	28.5
Score for star read(s)	:	3.9
Score for read counts	:	23.6
Score for mfe	:	1.1
Score for randfold	:	0
Score for cons. seed	:	
Total read count	:	58
Mature read count	:	49
Loop read count	:	0
Star read count	:	9

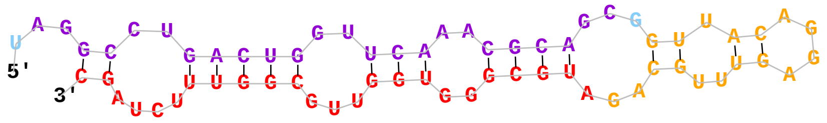

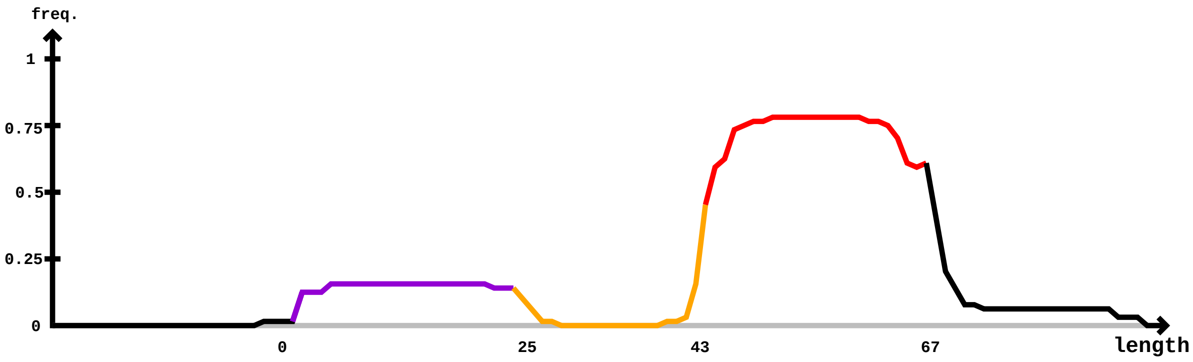

Star Mature

```
5'- aguaaaugcaauuuugugauauauaggccugacugguucaaacgcagcgguuacaggaguuugcagaugcgggugguugcgguuucuagcgguuagaaauuggugcguuugcgg -3'   obs
    aguaaaugcaauuuugugauauauaggccugacugguucaaacgcagcgguuacaggaguuugcagaugcgggugguugcgguuucuagcgguuagaaauuggugcguuugcgg        exp
    .(((((((((..(((.((((...((((...(((((..(((..((((...((.((....))..))...))))..)))...)))))))))...)))).)))....)))))))))..   reads   mm   sample
    ....................auauaggccugacugguucaaacg......................................................................   1       0    S03
    ........................aggccugacugUuucaaacgcagc..................................................................   1       1    S03
    ........................aggccugacugguucaaacgcagc..................................................................   1       0    S03
    ...........................ccugacugguucaaacgcaC...................................................................   1       1    S03
    ................................................................agaugcgggugguugcgguuuc............................   1       0    S03
    .................................................................gaugcgggugguugcgguuu.............................   1       0    S03
    .................................................................gaugcgggugguugcgguuuc............................   2       0    S03
    .................................................................gaugcgggugguugcgguuucu...........................   1       0    S03
    .................................................................gaugcgggugguugcgguuucua..........................   1       0    S03
    ..................................................................augcgggugguugcgguuucuagc........................   6       0    S03
    ...................................................................ugcgggugguugcgguuucu...........................   1       0    S03
    ...................................................................ugcgggugguugcgguuucuagcgC......................   1       1    S03
    .....................................................................cgggugguugcgguuucuagc........................   2       0    S03
    .....................................................................cgggugguugcgguuucuagcgg......................   1       0    S03
    .....................................................................cgggugguugcgguuucuagcggu.....................   1       0    S03
    ......................................................................gggugguugcgguuucuagcgUu.....................   1       1    S03
    .........................................................................ugguugcgguuucuagcggu.....................   1       0    S03
    ........................................................................................gcgguuagaaauuggugcguuugc..   1       0    S03

    ........................aggAcugacugguucaaacgcagc..................................................................   3       1    S04
    ........................aggccugacugguucaaacgcagc..................................................................   2       0    S04
    ...........................ccugacugguucGaacgcagcggu...............................................................   1       1    S04
    ..............................................................gcagaugcgggugguugcggu...............................   1       0    S04
    .................................................................gaugcgggugguugcgguuucu...........................   1       0    S04
    .................................................................gaugcgggugguugcgguuucua..........................   1       0    S04
    .................................................................gaugcgggugguugcgguuucuag.........................   1       0    S04
    ..................................................................augcgggugguugcgguuucu...........................   2       0    S04
    ..................................................................augcgggugguugcgguuucuagc........................   9       0    S04
    ..................................................................aGgcgggugguugcgguuucuagc........................   1       1    S04
    ..................................................................augcgggugguugcgguuucuagA........................   1       1    S04
    ...................................................................ugcgggugguugcgguuucGa..........................   1       1    S04
    ...................................................................ugcgggugguugcgguuucuagc........................   4       0    S04
    ...................................................................ugcgggugguugcgguuucuagcg.......................   2       0    S04
```

```
                        Star                                      Mature
aguaaaugcaauuuugugauauauaggccugacugguucaaacgcagcgguuacaggaguuugcagaugcggguggguugcgguuucuagcgguuagaaauuggugcguuugcgg
....................................................................gcgggugguugcgguuucC...........................   1   1   S04
....................................................................gcgggugguugcgguuucuagc........................   1   0   S04
.....................................................................cgggugguugcgguuucuagcgg......................   2   0   S04
.....................................................................cgggugguugcgguuucuagcggu.....................   1   0   S04
.......................................................................gAugguugcgguuucuagcgguua...................   1   1   S04
........................................................................................gcgguuagaaauuggugcguu.....   1   0   S04
.........................................................................................cgguuagaaauuggugcgGu.....   1   1   S04
.........................................................................................cgguuagaaauuggugcguuugc..   1   0   S04
```

Provisional ID	:	chrA09_799695
Score total	:	5.2
Score for star read(s)	:	3.9
Score for read counts	:	1.1
Score for mfe	:	0.1
Score for randfold	:	0
Score for cons. seed	:	
Total read count	:	14
Mature read count	:	13
Loop read count	:	0
Star read count	:	1

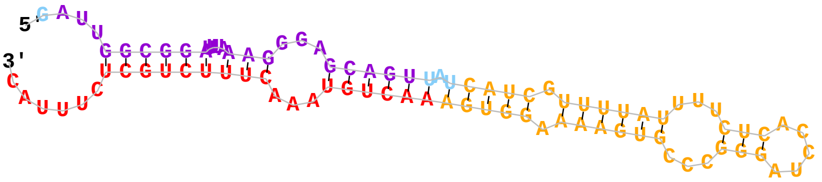

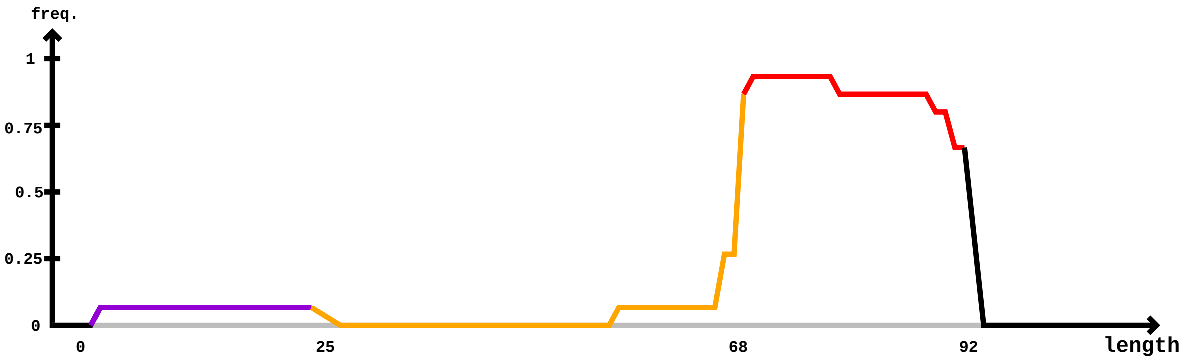

Star **Mature**

```
5'- gugauuggcggauuuaaagggagcaguuaucaucguuuuauuuucucaccuagggcccgugaaaaggugaaacuguaaacuuucugcucuuuacgguuucuaccucucgaucu   -3'    obs
    guga uuggcggauuuaaagggagcaguuaucaucguuuuauuuucucaccuagggcccgugaaaaggugaaacuguaaacuuucugcucuuuacgguuucuaccucucgaucu          exp
    ((((..((((((....(((...((((((.(((((.((((((...(((.....)))...)))))).)))))))))))...)))))))))..))))(((....))).........   reads    mm    sample
    ....................................................................gaaacuguaaacuuucugcucuuu.....................   1        0     S03
    ......................................................................aacuguaaacuuucugcucuuuac...................   1        0     S03

    ...auuggcggauuuaaagggagcagu......................................................................................   1        0     S04
    .........................................................cgugaaaaggugaaacuguaaac.................................   1        0     S04
    ....................................................................gaaacuguaaacuuucugcucu.......................   1        0     S04
    ....................................................................gaaacuguaaacuuucugcucuuu.....................   1        0     S04
    ......................................................................aacuguaaacuuucugcucuuuaU...................   1        1     S04
    ......................................................................aacuguaaacuuucugcucuuuac...................   7        0     S04
    .......................................................................acuguaaacuuucugcucuuuaU...................   1        1     S04
```

Provisional ID	:	chrA09_806149
Score total	:	2.6
Score for star read(s)	:	3.9
Score for read counts	:	1.1
Score for mfe	:	-2.4
Score for randfold	:	0
Score for cons. seed	:	
Total read count	:	14
Mature read count	:	13
Loop read count	:	0
Star read count	:	1

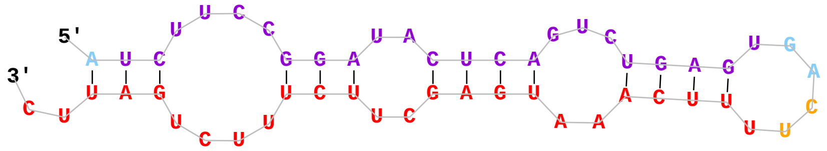

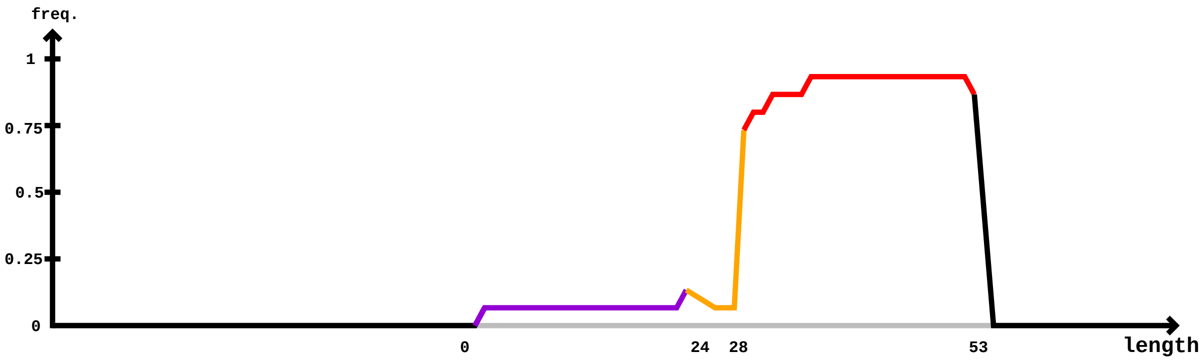

Star Mature

```
5'- gaauguuucuuggugcagcagcuuuuaacucugcuaugccgaaucuuccggauacucagucugagugacuuuucaaaugagcuucuuucugauucagucaucuucuacuucccuu  -3'  obs
    gaauguuucuuggugcagcagcuuuuaacucugcuaugccgaaucuuccggauacucagucugagugacuuuucaaaugagcuucuuucugauucagucaucuucuacuucccuu       exp
    (((........(((..(((((.........)))))..)))(((((....(((..((((...((((......))))..))))..)))....))))).......)))..........  reads  mm  sample
    ...........................................ucuuccggauacucagucugagu.................................................  1      0   S03
    ......................................................................uuucaaaugagcuucuuucugauuc....................  6      0   S03
    .......................................................................uucaaaugagcuucuuucugauuc....................  1      0   S03

    ................................................................gugacuuuucaaaugagcuucuuucugauu.....................  1      0   S04
    ......................................................................uuGcaaaugagcuucuuucugauuc....................  1      1   S04
    ......................................................................uuucaaaugagcuucuuucugauuc....................  3      0   S04
    .........................................................................caaaugagcuucuuucugauuc....................  1      0   S04
    .............................................................................ugagcuucuuucugauuc....................  1      0   S04
```

Provisional ID	:	chrA09_812406
Score total	:	0.7
Score for star read(s)	:	-1.3
Score for read counts	:	0
Score for mfe	:	2
Score for randfold	:	0
Score for cons. seed	:	
Total read count	:	47
Mature read count	:	40
Loop read count	:	7
Star read count	:	0

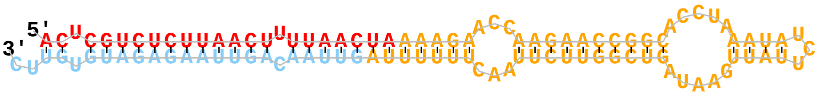

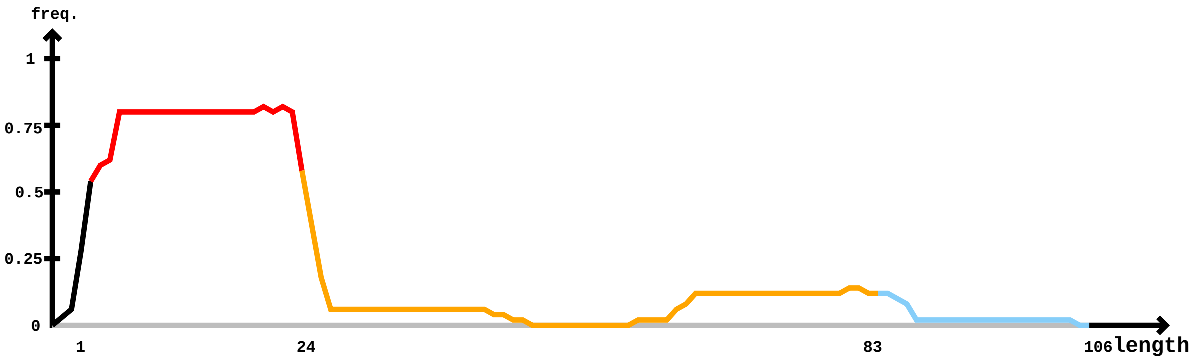

Mature   Star

```
5'- aaacucgucucuuaacuuuuaacuaaaagaaccaagaaccggcaccuaaauaucuuauugaauagucgguucuuaacuuuuuuaguuaacaguuaagagauguguucuuauauu   -3'    exp
    .(((.((((((((((((.((((((((((((...((((((((((.....((((...)))).....))))))))))...)))))))))))).)))))))))))).))).........   reads     mm      sample
    aaacucgucucuuaacuuuuaacu..........................................................................................   1         0       S03
    .aacucgucucuuaacuuuuaacu..........................................................................................   2         0       S03
    .aacucgucucuuaacuuuuaacua.........................................................................................   1         0       S03
    .aacucgucucuuaacuuuuaacuU.........................................................................................   1         1       S03
    ..acucgucucuuaacuuuuaac...........................................................................................   1         0       S03
    ..acucgucucuuaacuuuuaacuG.........................................................................................   1         1       S03
    ..acucgucucuuaacuuuuaacua.........................................................................................   3         0       S03
    ..acucguGucuuaacuuuuaacua.........................................................................................   1         1       S03
    ..acucgucucuuaacuuuuaacuaa........................................................................................   1         0       S03
    ...cucgucucuuaacuuuua.............................................................................................   1         0       S03
    ....ucgucucuuaacuuuuaacA..........................................................................................   1         1       S03
    .....cgucucuuaacuuuuaacua.........................................................................................   1         0       S03
    .....cgucucuuaacuuuuaacuaa........................................................................................   1         0       S03
    .....cgucGcuuaacuuuuaacuaa........................................................................................   1         1       S03
    .....cgucucuuaacuuuuaacuaaa.......................................................................................   2         0       S03
    ....................aacuaaaagaaccaagaaccggca......................................................................   1         0       S03
    ......................cuaaaagaaccaagaaccggcacc....................................................................   1         0       S03
    ...........................................................gaauagucgguucuuaacuuuuuu...............................   1         0       S03
    ...............................................................agucgguucuuaacuuuuuuagu............................   1         0       S03
    .................................................................ucgguucuuaacuuuuuuaguua..........................   1         0       S03

    aaacucguGucuuaacuuuuaacu..........................................................................................   1         1       S04
    aaacucgucucuuaacuuuuaacu..........................................................................................   1         0       S04
    .aacucgucucuuaacuuuuaacA..........................................................................................   1         1       S04
    .aacucgucuGuuaacuuuuaacu..........................................................................................   1         1       S04
    .aacucgucucuuaacuuuuaacu..........................................................................................   3         0       S04
    .aacucgucucuuaacuuuuaacua.........................................................................................   1         0       S04
    .aacucguGucuuaacuuuuaacua.........................................................................................   1         1       S04
    ..acucguGucuuaacuuuuaacu..........................................................................................   1         1       S04
    ..acucguGucuuaacuuuuaacua.........................................................................................   3         1       S04
    ..acucgucucuuaacuuuuaacuaa........................................................................................   2         0       S04
    ...cucgucucuuaacuuuuaacuaaa.......................................................................................   2         0       S04
    .....cgucucuuaacuuuuaacuaa........................................................................................   2         0       S04
    .....cgucucuuaacuuuuaacuaaa.......................................................................................   2         0       S04
```

Mature Star

```
aaacucgucucuuaacuuuuaacuaaaagaaccaagaaccggcaccuaaauaucuuauugaauagucgguucuuaacuuuuuuaguuaacaguuaagagauguguucuuauauu
........................aaaagaaccaagaaccggcaccua..................................................................	1	0	S04
...............................................................agucgguucuuaacuuuuuuaguC...........................	1	1	S04
................................................................gucgguucuuaacuuuuuuaguua..........................	1	0	S04
.................................................................ucgguucuuaacuuuuuuaguua..........................	1	0	S04
.................................................................................uuaguuaaAaguuaagagaugugu.........	1	1	S04
```

Provisional ID	:	chrA09_812936
Score total	:	7.3
Score for star read(s)	:	3.9
Score for read counts	:	10.8
Score for mfe	:	-7.3
Score for randfold	:	0
Score for cons. seed	:	
Total read count	:	33
Mature read count	:	32
Loop read count	:	0
Star read count	:	1

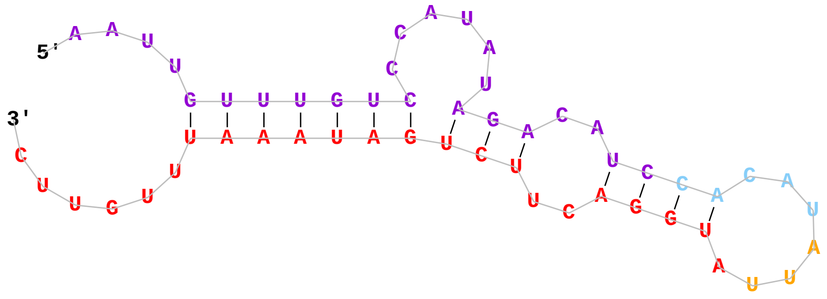

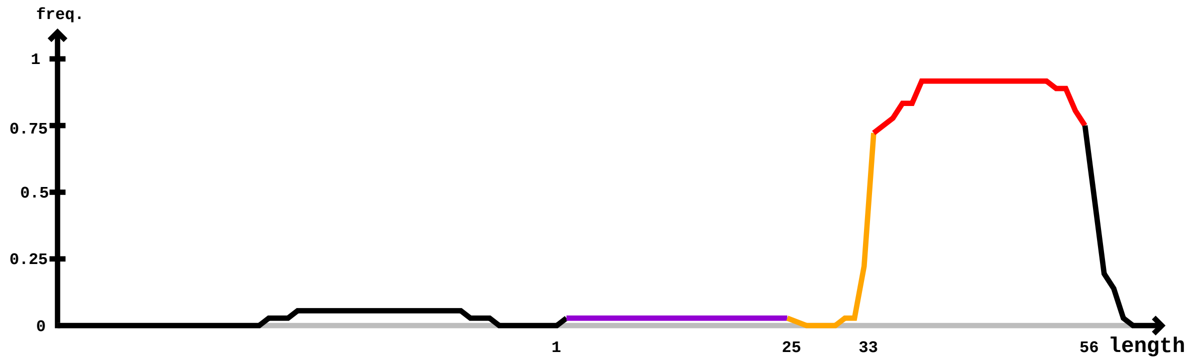

Star **Mature**

```
5'- uauauauuuuaggauugguuuuugugacuauauucuauaauugucuuaaaaaauuguuugucccauauagacauccacauauuauggacuucugauaaauuuguucauugaga  -3'   obs
    uauauauuuuaggauugguuuuugugacuauauucuauaauugucuuaaaaaauuguuugucccauauagacauccacauauuauggacuucugauaaauuuguucauugaga        exp
    .........(((((((((((.....))))).))))))......((((((..(((.(((((((......(((..((((.......))))..))))))))))..)))..))))))  reads   mm   sample
    ....................uuugugacuauauucuauaauugC.....................................................................  1       1    S03
    .......................gugacuauauucuauaau........................................................................  1       0    S03
    ..................................................................................uauggacuucugauaaauuuguuc.......  2       0    S03
    ...................................................................................auggacuucugauaaauuuguuc.......  2       0    S03
    ...................................................................................auggGcuucugauaaauuuguuc.......  1       1    S03
    ...................................................................................auggacuucugauaaauuuguuca......  2       0    S03
    .....................................................................................ggacuucugauaaauuuguucauu....  1       0    S03
    ......................................................................................gacuucugauaaauuuguucauu....  1       0    S03
    ........................................................................................cuucugauaaauuuguucau.....  1       0    S03
    ........................................................................................cGucugauaaauuuguucauu....  1       1    S03
    ........................................................................................cuucugauaaauuuguucauu....  1       0    S03

    ...................................................aauuguuugucccauGuagacauc......................................  1       1    S04
    ................................................................................auuauggacuucugauaaauuugu.........  1       0    S04
    ..................................................................................uauggacuucugauaaauuuguuc.......  4       0    S04
    ..................................................................................uauggacuucugauaaauuuguuU.......  1       1    S04
    ...................................................................................auggacuucugauaaauCu...........  1       1    S04
    ...................................................................................auggacuucugauaaauuugu.........  2       0    S04
    ...................................................................................auggacuucugauaaauuuguG........  2       1    S04
    ...................................................................................auggacuucugauaaauuuguuc.......  5       0    S04
    ...................................................................................auCgacuucugauaaauuuguuc.......  1       1    S04
    ...................................................................................auggGcuucugauaaauuuguuc.......  2       1    S04
    ....................................................................................uggGcuucugauaaauuuguucau.....  1       1    S04
    ......................................................................................gacuucugauaaauuuguucauuA...  1       1    S04
```

Provisional ID	:	chrA09_813614
Score total	:	186.8
Score for star read(s)	:	3.9
Score for read counts	:	180.1
Score for mfe	:	2.8
Score for randfold	:	0
Score for cons. seed	:	
Total read count	:	365
Mature read count	:	179
Loop read count	:	185
Star read count	:	1

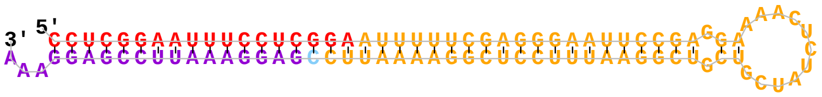

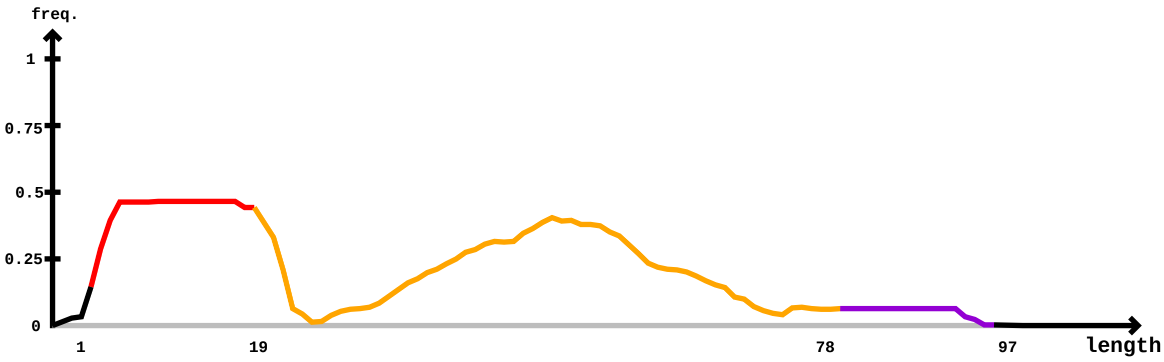

Mature **Star**

```
5'- uuccucggaauuuccucggaauuuuucgagggaauuccgaggaaaacucuaucgucgucggaauuuccucggaaaauuccgaggaaauuccgaggaaaaggaauguauaau  -3'  obs
    uuccucggaauuuccucggaauuuuucgagggaauuccgaggaaaacucuaucgucgucggaauuuccucggaaaauuccgaggaaauuccgaggaaaaggaauguauaau        exp
    ((((((((((((((((((((((((((((((((((((((((.((...........)).))))))))))))))))))))))))))))))))))))))))..............  reads  mm  sample
```

read	reads	mm	sample
uuccucggaauuuccucg	5	0	S03
uuccucggaauuuccucggaauu	1	0	S03
.uccucggaauuuccucggaa	1	0	S03
..ccucggaauuuccucgga	7	0	S03
..ccucggaauuuccucggaa	1	0	S03
..ccucggaauuuccucggaau	4	0	S03
..ccucggaauuuccucggaauu	1	0	S03
..ccucggaauuuccucggaauuu	1	0	S03
..ccucggaauuuccucggaauuuG	1	1	S03
..ccucggaauuuccucggaauuuuu	1	0	S03
...cucggaauuuccucggaa	7	0	S03
...cucggaauuuccucggaaC	1	1	S03
...cucggaauuuccucggaau	8	0	S03
...cucggaauGuccucggaauu	1	1	S03
...cucggUauuuccucggaauu	3	1	S03
...cucggaauuuccucggaauu	4	0	S03
...cucggaauuuccucggaauuu	1	0	S03
...cucggaauuuccucggaauuuu	3	0	S03
....ucggaauuuccucggaau	11	0	S03
....ucggaauuuccucggaauu	5	0	S03
....ucggaauuuccucggaauuu	1	0	S03
....ucggaauuuccucggaauuuu	2	0	S03
.....cggaauuuccucggaauu	11	0	S03
.....cggaauuuccucggaauuuuGc	1	1	S03
..........................cgagggaauuccgaggaaU	2	1	S03
..........................cgagggaauuccgaUgaaa	1	1	S03
...........................gagggaauuccgaggaCa	1	1	S03
...........................gagggaauuccgaggaaU	1	1	S03
...........................gagggaauuccgaggaaCa	3	1	S03
...........................gaggAaauuccgaggaaaacucua	2	1	S03
............................agggaauuccgaggaaCa	2	1	S03
............................agggaauuccgaggaaCac	1	1	S03
............................aggAaauuccgaggaaaacucua	2	1	S03

Mature **Star**

```
uuccucggaauuuccucggaauuuuucgagggaauuccgaggaaaacucuaucgucgucggaauuuccucggaaaauuccgaggaaauuccgaggaaaaggaauguauaau
.............................ggAaauuccgaggaaaacucua............................................................   2   1   S03
..............................ggaauuccgaUgaaaacucuauc..........................................................   1   1   S03
...............................gaauuccgaUgaaaacucuauc..........................................................   1   1   S03
................................aauuccgaUgaaaacucuauc..........................................................   1   1   S03
................................aauuccgaggaaaacucuauc..........................................................   1   0   S03
................................aauuccgaggaaaacucuaucguc.......................................................   1   0   S03
.................................auuccgaggaaaacucuau...........................................................   1   0   S03
.................................auuccgaUgaaaacucuaucgu........................................................   2   1   S03
.................................auuccgaggaaaacucuaucguc.......................................................   2   0   S03
..................................uuccgaggaaaacucuaucgGcg......................................................   1   1   S03
..................................uuccgaggaaaacucuaucgucgG.....................................................   1   1   S03
..................................uAccgaggaaaacucuaucgucgu.....................................................   1   1   S03
...................................uccgaggaaaacucuaucgucgu.....................................................   2   0   S03
...................................uccgaggaaaacucuaucgucguc....................................................   1   0   S03
....................................ccgaggaaaacucuaucgucguc....................................................   2   0   S03
.....................................cgaggaaaacucuaucgucguU....................................................   1   1   S03
.....................................cgaggaaaacucuaucgucgucA...................................................   4   1   S03
......................................gaggaaaacucuaucgucgu.....................................................   1   0   S03
......................................gaggaaaacucuaucgucgucgg..................................................   1   0   S03
......................................gaggaaaacucuaucgucgucgga.................................................   1   0   S03
.......................................aggaaaacucuaucgucgucgA..................................................   1   1   S03
........................................Ugaaaacucuaucgucguc....................................................   1   1   S03
........................................ggaaaacucuaucgucguU....................................................   1   1   S03
........................................ggaaaacucuaucgucguc....................................................   1   0   S03
........................................Ugaaaacucuaucgucgucggaau...............................................   1   1   S03
.........................................gaaaacucuaucgucgucggaauu..............................................   2   0   S03
.........................................Aaaaacucuaucgucgucggaauu..............................................   1   1   S03
..........................................aaaacucuaucgucgucggaauuG.............................................   1   1   S03
...........................................aaacucuaucgucgucggaauu..............................................   1   0   S03
...........................................aaacucuaucgucgucggaauuuc............................................   3   0   S03
............................................aacucuaucgucgGcggaauuu.............................................   1   1   S03
............................................aacucuaucgucgucggaauuucG...........................................   1   1   S03
.............................................acucuaucgucgucggaauuuccu..........................................   2   0   S03
..............................................cucuaucgucgucggaauu..............................................   1   0   S03
..............................................cucuaucgucgucggaauuuccu..........................................   1   0   S03
..............................................cucuaucgucgAcggaauuuccu..........................................   1   1   S03
..............................................cucuaucgucgucggaauuuccuU.........................................   1   1   S03
..............................................cucuaucgucgucggaauuuccuc.........................................   1   0   S03
...............................................ucuaucgucgucggaauuuccu..........................................   2   0   S03
...............................................ucuaucgucgucggaauuuccuU.........................................   1   1   S03
...............................................ucuaucgucgucggaauuuccuc.........................................   1   0   S03
...............................................ucuaucgucgucggaauuuccucg........................................   2   0   S03
................................................cuaucgucgucggaauuuccu..........................................   1   0   S03
................................................cuaucgucgucggaauuuccucgA.......................................   1   1   S03
................................................cuaucgucgucggaauuuccucgg.......................................   3   0   S03
.................................................uaucgucgucggaauuuccucA........................................   1   1   S03
.................................................uaucgucgucggaauuuccucg........................................   1   0   S03
.................................................uaucgucgucggaauuuccucgga......................................   2   0   S03
..................................................aucgucgucggaauuuccucgg.......................................   1   0   S03
..................................................aucgucgucggaauuuccucggaa.....................................   1   0   S03
...................................................ucgucgucggCauuuccucgg.......................................   1   1   S03
...................................................ucgucgucggaauuuccucgga......................................   1   0   S03
....................................................cgucgucggaauuuccucAg.......................................   1   1   S03
.....................................................................cggaaaauuccgaggaaauucUga..................   1   1   S03
......................................................................ggaaaauuccgaggaaauucUgag.................   2   1   S03
........................................................................aaaauuccgaggaaauuccga..................   3   0   S03
..........................................................................aauuccgaggaaauuccga..................   1   0   S03
...........................................................................auuccgaggaaauuccga..................   2   0   S03
...........................................................................auuccgaggaaauuccgagU................   1   1   S03
...........................................................................auuccgaggaaauuccgagg................   2   0   S03
...........................................................................Guuccgaggaaauuccgagg................   1   1   S03
............................................................................uuccgaggaaauuccgag.................   1   0   S03

uuccucggaauuuccucg.............................................................................................   4   0   S04
uuccucggaauuuccucgga...........................................................................................   1   0   S04
.uccucggUauuuccucggaauu........................................................................................   1   1   S04
..ccGcggaauuuccucgga...........................................................................................   1   1   S04
..ccucggaauuuccucgga...........................................................................................   13  0   S04
..ccucggaauuuccucggaa..........................................................................................   6   0   S04
..ccucggaauuuccucggaaC.........................................................................................   1   1   S04
```

Mature **Star**

uuccucggaauuuccucggaauuuuucgagggaauuccgaggaaaacucuaucgucgucggaauuuccucggaaaauuccgaggaaauuccgaggaaaaggaauguauaau			
..ccucggaauuuccucggaau...	3	0	S04
..ccucggaauuuccucggaauG..	1	1	S04
..ccucggaauuuccucggaauu..	1	0	S04
..ccucggaauuuccucggaauuu...	1	0	S04
..ccucggaauuuccucggaauuuu..	1	0	S04
...cucggaauuuccucggaa...	8	0	S04
...cucggaauuuccucggaau..	9	0	S04
...cucggaauuuccucggaauu...	6	0	S04
...cucggaauuuccucggCauu...	1	1	S04
...cucggaauuuccucggaauG...	1	1	S04
...cucggaauuuccucggaauuu..	1	0	S04
...cucggaauuuccucggaauuuu...	2	0	S04
....ucggaauuuccucggaau...	11	0	S04
....ucggaauuuccucggaauu..	8	0	S04
....ucggaauuuccucggaauuu...	1	0	S04
....ucggaauuuccucggaauuuu..	2	0	S04
....ucggaauuuccucggaauuuuu...	1	0	S04
.....cggaauuuccucggaauu..	12	0	S04
.....cggaauuuccucggaauuu...	2	0	S04
.....cggaauuuccucggaauuuu..	1	0	S04
.........auuuccucggaauuuuGc..	1	1	S04
....................auuuuucgagggaauuccgagU...	1	1	S04
...........................gagggaauuccgaggaaU..	1	1	S04
...........................gagggaauuccgaggaaa..	1	0	S04
...........................gagggaauuccgaggaaCa...	2	1	S04
............................aggAaauuccgaggaaaacucua..	1	1	S04
.............................ggAaauuccgaggaaaacucua..	1	1	S04
...............................gaauuccgaggaaaacucu...	1	0	S04
................................aauuccgaggaaaacucuaucguc...	2	0	S04
................................aauuccgaggaaGacucuaucguc...	1	1	S04
.................................auuccgaggaaaacucuaucgu..	1	0	S04
.................................auuccgaggaaaacucuaucguc...	1	0	S04
.................................auuccgaggaaaacucuaucguU...	1	1	S04
.................................auuccgaggaaaacucuaucgucg..	1	0	S04
.................................auuccgaggaaaacucuaucgucA..	1	1	S04
..................................uuccgaggaaaacucuGuc..	2	1	S04
..................................uuccgaggaaaacucuaucguc...	1	0	S04
..................................uuccgaggaaaacucuaucgucg..	1	0	S04
..................................uuccgaggaaaacucuaucgucgu...	1	0	S04
..................................uuccgaggaaaacucuaucgucgC...	2	1	S04
...................................uccgaggaaaacucuaucguG...	1	1	S04
...................................uccgaggaaaacucuaucgucg..	1	0	S04
...................................uccgaggaaaacucuaucgucgu...	2	0	S04
...................................uccgaggaaaacucuaucgucguc..	3	0	S04
....................................ccgaggaaaacucuaucgucA..	1	1	S04
....................................ccgaggaaaacucuaucgucg..	2	0	S04
....................................ccgaggaaaacucuaucgucgu...	1	0	S04
.....................................cgaggaaaacucuaucgucgu...	1	0	S04
.....................................cgaggaaaacucuaucgucguU..	1	1	S04
.....................................cgaggaaaacucuaucgucgucU...	1	1	S04
.....................................cgaggaaaacucuaucgucgucAg..	1	1	S04
......................................gaggaaaacucuaucgucgucgg..	1	0	S04
......................................gaggaaaacucuaucgucgucggU...	1	1	S04
....aggaaaacucuaucgucgu...	1	0	S04
....aggaaaacucuaucgucgucg...	1	0	S04
....aggaaaacucuaucgucgucA...	3	1	S04
....aggaaaacucuaucgucgucgA..	1	1	S04
....aggaaaacucuaucgucgucgg..	1	0	S04
....ggaaaacucuaucgucguc..	1	0	S04
....ggaaaacucuaucgucgucA...	1	1	S04
....ggaaaacucuaucgucgucU...	1	1	S04
....gaaaacucuaucgucguU..	1	1	S04
....gaaaacucuaucgucgucC...	1	1	S04
....gaaaacucuaucgucgucU...	1	1	S04
....gaaaacucuaucgucgucggU...	1	1	S04
....gaaaacucuaucgucgucggaa..	1	0	S04
....gaaaacucuaucgucgucggaUu...	1	1	S04
....gaaaacucuaucgCcgucggaauu..	1	1	S04
....aaaacucuaucgucgucg...	1	0	S04
....aaaacucuaucgucgucggaau...	1	0	S04

Mature **Star**

```
uuccucggaauuuccucggaauuuuucgagggaauuccgaggaaaacucuaucgucgucggaauuuccucggaaaauuccgaggaaauuccgaggaaaaggaauguauaau
..........................................aaaacucuaucgucgucAgaauuu.............................................   1   1   S04
..........................................aaaacucuaucgucgucggaauuC.............................................   1   1   S04
...........................................aaacucuaucgucgucAgaauuu.............................................   1   1   S04
...........................................aaacucuaucgucgucggaauuu.............................................   1   0   S04
...........................................aaacucuaucgucgucggaauuuc............................................   2   0   S04
............................................aacucuaucgucgucggaauuu.............................................   1   0   S04
............................................aacucuaucgucgucggaauuucc...........................................   1   0   S04
.............................................acucuaucgucgucggaauuuc............................................   1   0   S04
.............................................acucuaucgucgucggaauuuccA..........................................   1   1   S04
.............................................acucuaucgucgucggaauuuccu..........................................   1   0   S04
.............................................acucuaucgCcgucggaauuuccu..........................................   1   1   S04
..............................................cucuaucgucgucggaauuucU...........................................   1   1   S04
..............................................cucuaucgucgucggaauuuccu..........................................   1   0   S04
..............................................cucuaucgucgucggaauuuccuA.........................................   1   1   S04
...............................................ucuaucgucgucggaauuucA...........................................   1   1   S04
...............................................ucuaucgucgucggaauuuccu..........................................   1   0   S04
...............................................ucuaucgucgucggaauuuccG..........................................   1   1   S04
...............................................ucuaucgucgucggaauuuccuc.........................................   1   0   S04
...............................................ucuaucgucgucggaauuuccucg........................................   3   0   S04
................................................cuaucgucgucggaauuuccG..........................................   1   1   S04
................................................cuaucgucgucggaauuuccucgg.......................................   1   0   S04
.................................................uaucgucgucggaauuuccu..........................................   1   0   S04
.................................................uaucgucgucggaauuuccucA........................................   1   1   S04
.................................................uaucgucgucggaauuuccucg........................................   1   0   S04
.................................................uaucgucgucggaauuuccucU........................................   1   1   S04
.................................................uaucgucgucggaauuuccucgg.......................................   1   0   S04
..................................................aucgucgucggaauuuccuU.........................................   1   1   S04
..................................................aucgucgucggaauuuccucgg.......................................   2   0   S04
..................................................aucgucgucggaauuuccucgA.......................................   1   1   S04
..................................................aucgucgucggaauuuccucggaa.....................................   2   0   S04
...................................................ucgucgucggaauuuccucA........................................   1   1   S04
....................................................cgucgucggaauuuccucg........................................   1   0   S04
.......................................................cgucggaauuuccucgga......................................   1   0   S04
........................................................Uucggaauuuccucggaaaau..................................   1   1   S04
.........................................................ucggaauuuccucggaaaau..................................   1   0   S04
.........................................................ucggaauGuccucggaaaauu.................................   1   1   S04
......................................................................ggaaaauuccgaggaaauucUga..................   1   1   S04
......................................................................ggaaaauuccgaggaaauucUgag.................   1   1   S04
.......................................................................gaaaCuuccgaggaaauuccga..................   1   1   S04
........................................................................aaaauuccgaggaaauuccga..................   3   0   S04
...........................................................................auuccgaggaaauuccgagg................   4   0   S04
................................................................................gaggaaauuccgaggaaa.............   1   0   S04
```

Provisional ID	:	chrA09_825457
Score total	:	5.2
Score for star read(s)	:	3.9
Score for read counts	:	-0.3
Score for mfe	:	1.7
Score for randfold	:	0
Score for cons. seed	:	
Total read count	:	11
Mature read count	:	9
Loop read count	:	1
Star read count	:	1

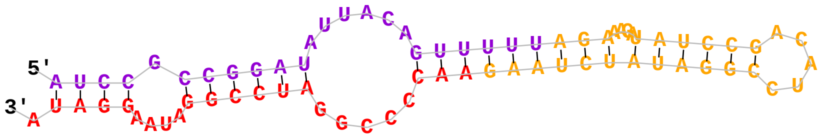

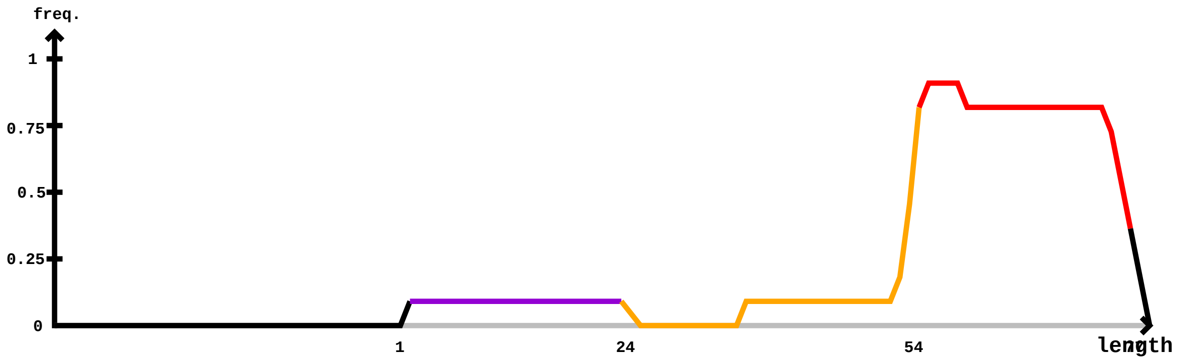


```
                                               Star                                                   Mature
5'- uuauuuauuuuaaggauucaaaucuggauccgaauauccgccggauauuacaguuuuuagaaagauauccgacauccggauaucuaagaaccccggauccggauaaggauag  -3'   obs
    uuauuuauuuuaaggauucaaaucuggauccgaauauccgccggauauuacaguuuuuagaaagauauccgacauccggauaucuaagaaccccggauccggauaaggauag         exp
    .............(((((((....)))))))...(((((.((((((......(((((((((....((((((.....))))))))))))))).....))))))....))))).   reads   mm   sample
    ...................................auccgccggauauCacaguuuuu......................................................   1       1    S03
    .......................................................................................gaaccccggauccggauaagg....   1       0    S03
    .......................................................................................gaaccccggauccggauaagga...   1       0    S03
    ........................................................................................aaccccggauccggauaaggau..   1       0    S03
    .........................................................................................accccggauccggauaaggau..   1       0    S03

    ......................................................................gacauccggauaucuaagaaccU...................   1       1    S04
    ......................................................................................agaaccccggauccggauaagga...   1       0    S04
    .......................................................................................gaaccccggauccggauaaggaGa.   1       1    S04
    ........................................................................................aaccccggauccggauaaggaua.   3       0    S04
```

Provisional ID	:	chrA09_833195
Score total	:	1.6
Score for star read(s)	:	3.9
Score for read counts	:	-3.3
Score for mfe	:	1.1
Score for randfold	:	0
Score for cons. seed	:	
Total read count	:	5
Mature read count	:	4
Loop read count	:	0
Star read count	:	1

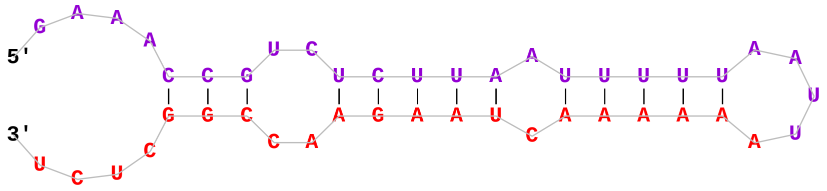

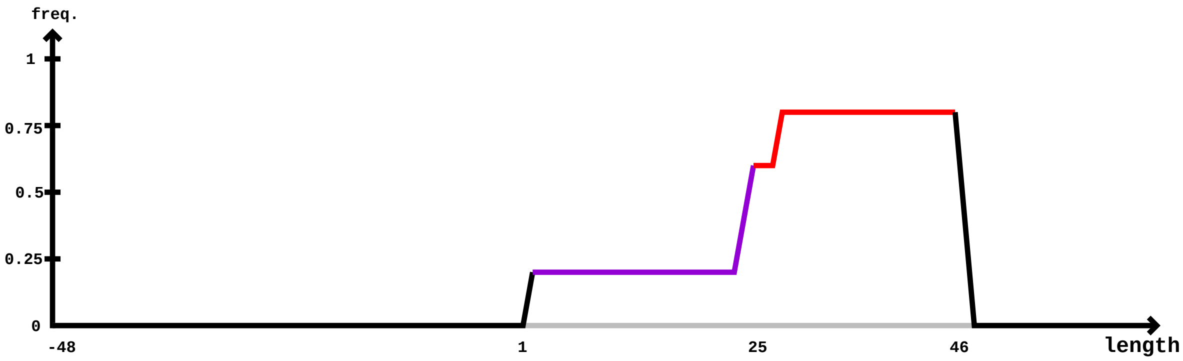

Star Mature

```
5'- agcaugauuaauuggggguuguuaagguagaguucuuaacgaaauauaagaaaccgucucuuaauuuuuaauuaaaaaacuaagaaccggcucucuccauuaaucauguucuaa   -3'    obs
    agcaugauuaauuggggguuguuaagguagaguucuuaacgaaauauaagaaaccgucucuuaauuuuuaauuaaaaaacuaagaaccggcucucuccauuaaucauguucuaa          exp
    ((((((((((((.((((((((((((((......))))))))))......(((..(((..(((((.(((((.....))))).)))))..))).))))))))))))))))))....    reads      mm      sample
    .......................................................................uuaaaaaacuaagaaccggcucuG...................    1          1       S03
    ...........................................................................aaaacuaagaaccgUcucuc...................    1          1       S03

    .................................................gaaaccAucucuuaauuuuuaauu.........................................    1          1       S04
    ........................................................................uaaaaaacuaagaaccgAcucuc...................    1          1       S04
    .........................................................................aaaaaacuaagaaccggcucu....................    1          0       S04
```

Provisional ID	: chrA09_848786
Score total	: 1.3
Score for star read(s)	: 3.9
Score for read counts	: -3.3
Score for mfe	: 0.9
Score for randfold	: 0
Score for cons. seed	:
Total read count	: 5
Mature read count	: 4
Loop read count	: 0
Star read count	: 1

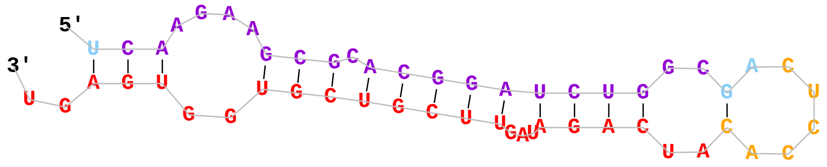

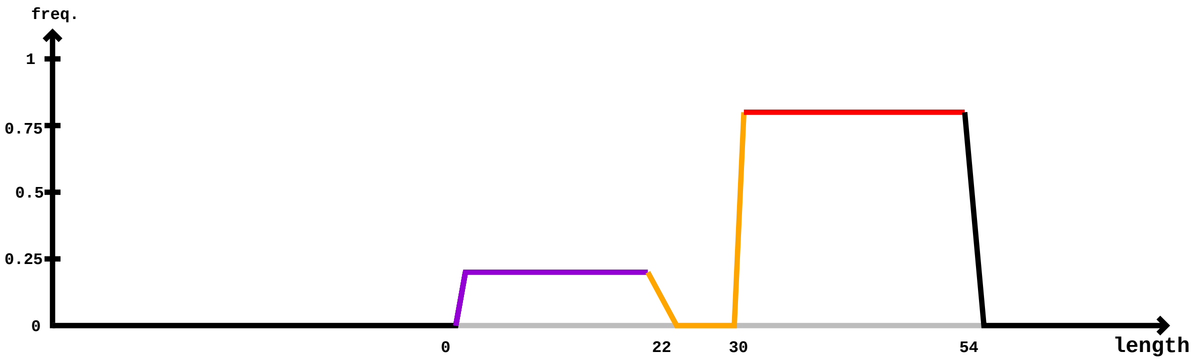

Star Mature

```
5'- gguguuguuaucaucaauaaagugaugaaguucaugacccucaagaagcgcacggaucuggcgacuccacaucagauaguucgucguggugaguuuggucacgcucgacauguu   -3'   obs
    gguguuguuaucaucaauaaagugaugaaguucaugacccucaagaagcgcacggaucuggcgacuccacaucagauaguucgucguggugaguuuggucacgcucgacauguu         exp
    .((((((...((((((......))))))......(((((((((....(((.(((((((((..(......)..))))...))))))))..))))...)))))....))))))...    reads   mm   sample
    ......................................................................aucagauaguucgucguggugagu....................    1       0    S03

    .........................................caagaagcgcacggaucuggc....................................................    1       0    S04
    ......................................................................aucagauaguucgucguggugagu....................    3       0    S04
```

Provisional ID	: chrA09_874033
Score total	: 0
Score for star read(s)	: -1.3
Score for read counts	: 0
Score for mfe	: 1.3
Score for randfold	: 0
Score for cons. seed	:
Total read count	: 168
Mature read count	: 168
Loop read count	: 0
Star read count	: 0

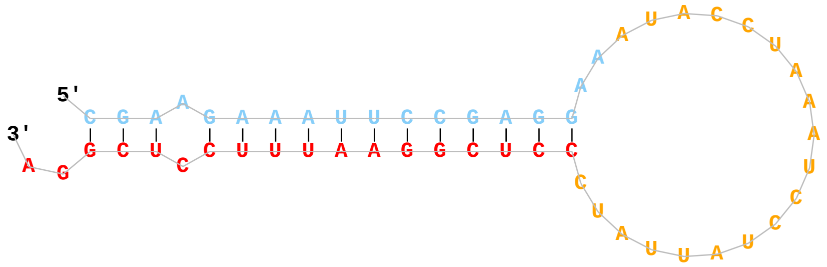

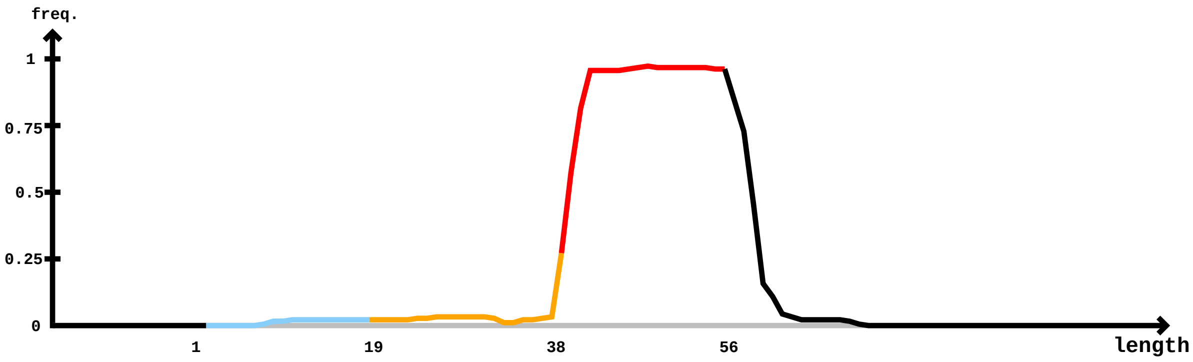

Star Mature

```
5'- gucucggacuauuccgaagaaauuccgaggaaauaccuaaaauccuauuaucccucggaauuuccucggaauuuuuaaaauccccuaagggcucuguagcagcuauaauauaucc  -3'   exp
    ((..((((..(((((((.((((((((((((......................)))))))))))).)))))))........(((....))).))))..))................   reads     mm     sample
    .......................................aaauccuauuaucccucggaauu.....................................................   1         0      S03
    ................................................uauccAucggaauuuccucggaau...........................................   2         1      S03
    ...................................................cccucggaauuuccucggaauuA.........................................   1         1      S03
    ....................................................ccucggaauuuccucgga.............................................   7         0      S03
    ....................................................ccucggaauuuccucggaa............................................   1         0      S03
    ....................................................ccucggaauuuccucggaau...........................................   4         0      S03
    ....................................................ccucggaauuuccucggaauu..........................................   1         0      S03
    ....................................................ccucggaauuuccucggaauuu.........................................   1         0      S03
    ....................................................ccucggaauuuccucggaauuuG........................................   1         1      S03
    ....................................................ccucggaauuuccucggaauuuuu.......................................   1         0      S03
    .....................................................cucggaauuuccucggaa............................................   7         0      S03
    .....................................................cucggaauuuccucggaaC...........................................   1         1      S03
    .....................................................cucggaauuuccucggaau...........................................   8         0      S03
    .....................................................cucggaauGuccucggaauu..........................................   1         1      S03
    .....................................................cucggaauuuccucggaauu..........................................   4         0      S03
    .....................................................cucggUauuuccucggaauu..........................................   3         1      S03
    .....................................................cucggaauuuccucggaauuu.........................................   1         0      S03
    .....................................................cucggaauuuccucggaauuuu........................................   3         0      S03
    ......................................................ucggaauuuccucggaau...........................................   11        0      S03
    ......................................................ucggaauuuccucggaauu..........................................   5         0      S03
    ......................................................ucggaauuuccucggaauuu.........................................   1         0      S03
    ......................................................ucggaauuuccucggaauuuu........................................   2         0      S03
    ......................................................ucggaauuuccucggaauuuuua......................................   1         0      S03
    .......................................................cggaauuuccucggaauu..........................................   11        0      S03
    .............................................................uuccucggaauuuuuaaaauc.................................   1         0      S03
    .............................................................uuccucggaauuuuuaaaauccc...............................   1         0      S03

    ....................aauuccgaggaaauaccuaaaaucc......................................................................   1         0      S04
    .....................auuccgaggaaauaccuaaaauccG.....................................................................   2         1      S04
    .......................uccgaggaaauaccuaaaauccG.....................................................................   1         1      S04
    ....................................cuaaaauccuauuaucccucggaau......................................................   1         0      S04
    ..................................................ucccucggaauuuccucg...............................................   1         0      S04
    ....................................................ccGcggaauuuccucgga.............................................   1         1      S04
    ....................................................ccucggaauuuccucgga.............................................   13        0      S04
```

```
               Star                                Mature
gucucggacuauuccgaagaaauuccgaggaaauaccuaaaauccuauuaucccucggaauuuccucggaauuuuuaaaauccccuaagggcucuguagcagcuauaauauaucc
....................................................ccucggaauuuccucggaa............................................   6    0   S04
....................................................ccucggaauuuccucggaau...........................................   3    0   S04
....................................................ccucggaauuuccucggaaC...........................................   1    1   S04
....................................................ccucggaauuuccucggaauu..........................................   1    0   S04
....................................................ccucggaauuuccucggaauG..........................................   1    1   S04
....................................................ccucggaauuuccucggaauuu.........................................   1    0   S04
....................................................ccucggaauuuccucggaauuuu........................................   1    0   S04
.....................................................cucggaauuuccucggaa............................................   8    0   S04
.....................................................cucggaauuuccucggaau...........................................   9    0   S04
.....................................................cucggaauuuccucggaauu..........................................   6    0   S04
.....................................................cucggaauuuccucggaauG..........................................   1    1   S04
.....................................................cucggaauuuccucggCauu..........................................   1    1   S04
.....................................................cucggaauuuccucggaauuu.........................................   1    0   S04
.....................................................cucggaauuuccucggaauuuu........................................   2    0   S04
......................................................ucggaauuuccucggaau...........................................  11    0   S04
......................................................ucggaauuuccucggaauu..........................................   8    0   S04
......................................................ucggaauuuccucggaauuu.........................................   1    0   S04
......................................................ucggaauuuccucggaauuuu........................................   2    0   S04
......................................................ucggaauuuccucggaauuuuu.......................................   1    0   S04
......................................................ucggaauuuccucggaauuuuua......................................   1    0   S04
.......................................................cggaauuuccucggaauu..........................................  12    0   S04
.......................................................cggaauuuccucggaauuu.........................................   2    0   S04
.......................................................cggaauuuccucggaauuuu........................................   1    0   S04
...........................................................auuuccucggaauuuuuaaaaucA................................   1    1   S04
............................................................uuuccucggaauuuuuaaaaucA................................   1    1   S04
```

Provisional ID	:	chrA09_880769
Score total	:	0.8
Score for star read(s)	:	3.9
Score for read counts	:	-4.9
Score for mfe	:	1.9
Score for randfold	:	0
Score for cons. seed	:	
Total read count	:	2
Mature read count	:	1
Loop read count	:	0
Star read count	:	1

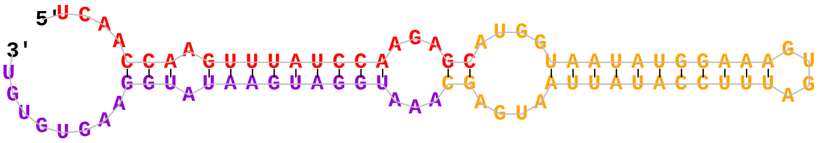

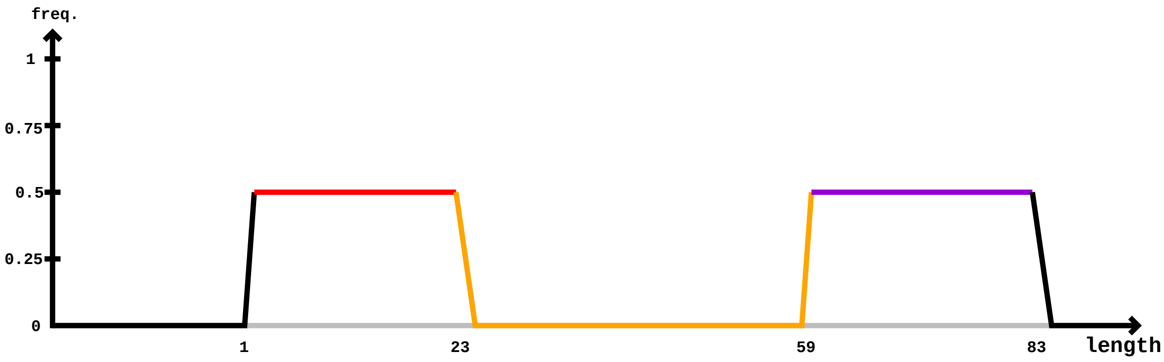


```
                      Mature                                                Star
5'- aacaaucaugaccccucacucaaccaaguuuauccaagagcaugguaauauggaaagugauuuccauauuaaugagcaaauggaugaauauggaagugugucaagaggggg   -3'    obs
    aacaaucaugaccccucacucaaccaaguuuauccaagagcaugguaauauggaaagugauuuccauauuaaugagcaaauggaugaauauggaagugugucaagaggggg          exp
    ...........((((((((.((.(((.(((((((((...((....(((((((((((....)))))))))))....))...))))))))).)))...)).))...)))))).   reads      mm         sample
    ...................ucaaccaaguuuauccaagagU......................................................................   1          1          S03

    ............................................................................aaauggaugaauauggaagugugu..........   1          0          S04
```

Provisional ID	:	chrA09_882837
Score total	:	0
Score for star read(s)	:	3.9
Score for read counts	:	-4.9
Score for mfe	:	1
Score for randfold	:	0
Score for cons. seed	:	
Total read count	:	2
Mature read count	:	1
Loop read count	:	0
Star read count	:	1

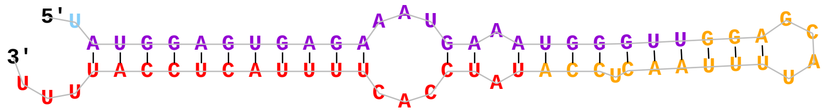

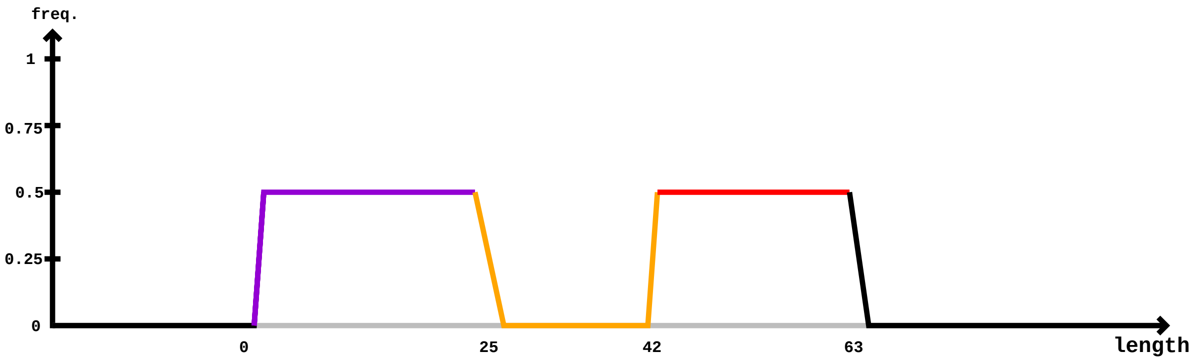

Star **Mature**

```
5'- uguuuguuuauuacuccauuauggagugagaaaugaaauggguuggagcauuuuaacuccauauccacuuuuacuccauuuuggaagaaaaaauagaguuuaacauuggagaug   -3'    obs
    uguuuguuuauuacuccauuauggagugagaaaugaaauggguuggagcauuuuaacuccauauccacuuuuacuccauuuuggaagaaaaaauagaguuuaacauuggagaug          exp
    ..(((((((.((..((((..(((((((((((...((.((((((((((....)))))).)))).))...)))))))))))..))))..)).))))))).................   reads   mm   sample
    ....................auggagugagaaaugaaauggguu......................................................................   1       0    S04
    .............................................................uauccacuuuuacuccauuuu................................   1       0    S04
```

Provisional ID	: chrA09_884500
Score total	: 0
Score for star read(s)	: -1.3
Score for read counts	: -0.3
Score for mfe	: 1.7
Score for randfold	: 0
Score for cons. seed	:
Total read count	: 11
Mature read count	: 6
Loop read count	: 5
Star read count	: 0

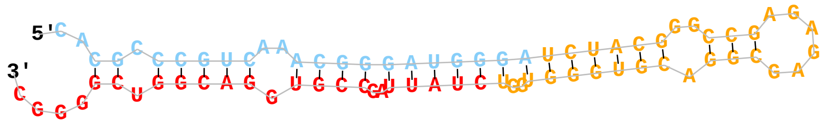

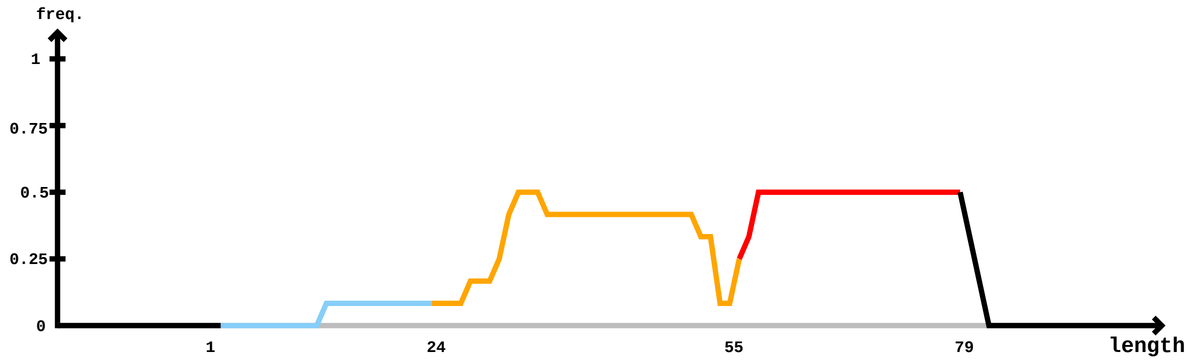

Star Mature

5'- uuggaaaaauuaacgcacgcccgucaaacgggaugggaucuacgggccgagagagcggacgugggguggucuauuagccguggacggucggggcguuacaagaagacacaagua -3'	exp		
..........((((((.((.(((((..(((((((((((((((((..(((.......))).)))))))..))))))..)))).))))).))..))))))................	reads	mm	sample
..........................aacgggaugggaucCacgggccg...	1	1	S03
....acggAccgagagagcggacguggg...	1	1	S03
....ggcUgagagagcggacgugggug...	1	1	S03
....gcUgagagagcggacgugggug...	1	1	S04
....gccgaAagagcggacguggguggu...	1	1	S04
....ccgagagagcggacgugggug...	1	0	S04
....cuauuagccguggacggucggggc....................	2	0	S04
....cuauuagccguggacggucggggU....................	1	1	S04
....Cauuagccguggacggucggggc....................	1	1	S04
....auuagccguggacggucggggcgu..................	1	0	S04
....auuagccguggacggucgggCcgu..................	1	1	S04

Provisional ID	:	chrA09_885030
Score total	:	158.1
Score for star read(s)	:	3.9
Score for read counts	:	154.6
Score for mfe	:	-0.3
Score for randfold	:	0
Score for cons. seed	:	
Total read count	:	315
Mature read count	:	312
Loop read count	:	1
Star read count	:	2

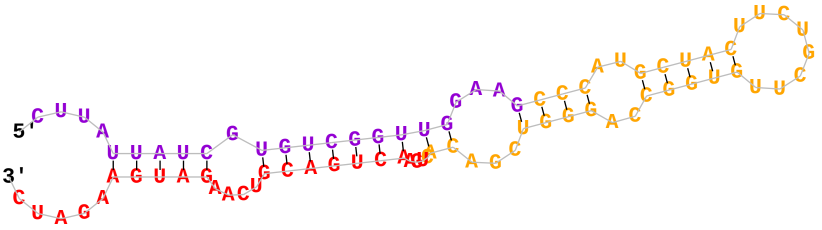

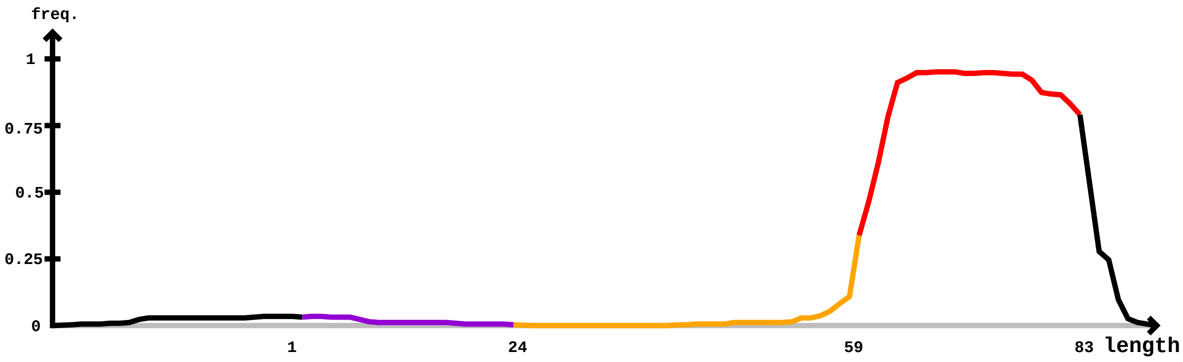

Star **Mature**

```
5'- uauaaauauguggccgcgaaaauacuuauuaucgugucgguuggaagcccaugcuacuucugcuuguggccagggucgacacugaacugacgucaagaugaagaucgugaagu  -3'   obs
    uauaaauauguggccgcgaaaauacuuauuaucgugucgguuggaagcccaugcuacuucugcuuguggccagggucgacacugaacugacgucaagaugaagaucgugaagu        exp
    ..............(((((......((.(((((.(((((((((...((((..(((((........)))))..))))...))....)))))))....))))).)))))))....   reads   mm   sample
    uauaaauaCguggccgcgaaaaua.........................................................................................   1       1    S03
    .auaaauauguggccgcgaaaaua.........................................................................................   1       0    S03
    .......auguCgccgcgaaaauacuuauua..................................................................................   1       1    S03
    .....................................................................ccagggucgacacugaacugacgu....................   1       0    S03
    ............................................................................cgacacugaacugacgucaaga...............   1       0    S03
    ..............................................................................acacugaacugacgucaagaug.............   1       0    S03
    ...............................................................................cacugaacugacgucaagauU.............   1       1    S03
    ...............................................................................cacugaacugacgucaagaug.............   1       0    S03
    ................................................................................acugaacugacgucaagaugaaga.........   1       0    S03
    ................................................................................Ccugaacugacgucaagaugaaga.........   1       1    S03
    .................................................................................cugaacugacgucaagaugaagaG........   1       1    S03
    .................................................................................cugaacugacgucaagaugaagau........   3       0    S03
    ..................................................................................ugaacugacgucaagauga............   4       0    S03
    ..................................................................................ugaacugacgucaagGugaagauc.......   1       1    S03
    ..................................................................................ugaacugacgucaagaugaagauc.......   23      0    S03
    ..................................................................................ugaacugacgucaagaugaagauA.......   1       1    S03
    ..................................................................................Ggaacugacgucaagaugaagauc.......   1       1    S03
    ...................................................................................gaacugacgucaagauga............   2       0    S03
    ...................................................................................gaacugacgucaagaugGagauc.......   1       1    S03
    ...................................................................................gaacugacgucaagaugaagauc.......   3       0    S03
    ...................................................................................gaacugacgucaagaugaagaucg......   7       0    S03
    ...................................................................................gaacugacgucaagaugaagaucU......   1       1    S03
    ....................................................................................aacugacgucaagaugaagau........   1       0    S03
    ....................................................................................aacugacgucaagaugaagauc.......   10      0    S03
    ....................................................................................aacAgacgucaagaugaagauc.......   1       1    S03
    ....................................................................................Nacugacgucaagaugaagaucg......   1       1    S03
    ....................................................................................aacugacgucaagaugaagaucg......   7       0    S03
    ....................................................................................aacugacgucaagaugaagaucUu.....   1       1    S03
    ....................................................................................aacugacgucaagaugaagaucgG.....   1       1    S03
    ....................................................................................aacugacgucaagaugaagaucgu.....   2       0    S03
    .....................................................................................acugacgucaagaugaaga.........   1       0    S03
    .....................................................................................acugacgucaagaugaagauc.......   3       0    S03
    .....................................................................................acugacgucaagaugaagaucg......   5       0    S03
```

```
                              Star                                                    Mature
uauaaauauguggccgcgaaaauacuuauuaucgugucgguuggaagcccaugcuacuucugcuuguggccagggucgacacugaacugacgucaagaugaagaucgugaagu
.....................................................................................acugacgucaagaugaagaucgG.....   1   1   S03
.....................................................................................acugacgucaagaugaagaucUu.....   1   1   S03
.....................................................................................acugacgucaagaugaagaucgug....   9   0   S03
.....................................................................................acugacgucaagaugaaCaucgug....   1   1   S03
......................................................................................cugacgucaagaugaagauc.......   2   0   S03
......................................................................................cugacgucaagaugaagaucU......   1   1   S03
......................................................................................cugacgucaagaugaagaucgug....   7   0   S03
......................................................................................cugacgucaagaugaagaucgAg....   1   1   S03
......................................................................................cugacgucaagaugaagaucguU....   1   1   S03
......................................................................................cugacgucaagaugaagaucguga...   5   0   S03
.......................................................................................ugacgucaagaugaGgauc.......   1   1   S03
.......................................................................................ugacgucaagaugaagaucguga...   1   0   S03
........................................................................................gacgucaagaugaagaucgugaag.   2   0   S03
...............................................................................................agaugGagaucgugaagu   1   1   S03

....aauauguggccgcgaaaauacuu......................................................................................   1   0   S04
......uauguggccgcgaaaauacuuauu...................................................................................   1   0   S04
.......auguggccgcgaaaauacuuauu...................................................................................   1   0   S04
.......auguggccgcgaaaauacuuauua..................................................................................   2   0   S04
........uguggccgcgaaaauacuuauu...................................................................................   1   0   S04
........uguggccgcgaaaauacuuauuau.................................................................................   1   0   S04
...................aaauacuuauuaucgugucggu........................................................................   1   0   S04
....................aauacuuauuaucgugucgg.........................................................................   1   0   S04
........................cuuauuaucguguUgguuggaag..................................................................   1   1   S04
.........................uuauuaucguguUgguuggaa...................................................................   1   1   S04
...............................................................uuguggccagggucgacacuga............................   1   0   S04
.................................................................guggccaUggucgacacugaacu.........................   1   1   S04
.....................................................................ccagggucgacacugaacugacgA....................   1   1   S04
...........................................................................ucgacacugaacugacgucaag................   1   0   S04
............................................................................cgacacugaacugacgucaagaug.............   4   0   S04
..............................................................................acacugaacugacgucaagaugaa...........   2   0   S04
...............................................................................cacugaacugacgucaagaug.............   1   0   S04
...............................................................................cacugaacugacgucaagauga............   2   0   S04
...............................................................................cacugaacugacgucaagaugU............   1   1   S04
................................................................................acugaacugacgucaagaugU............   1   1   S04
................................................................................acuUaacugacgucaagaugaaga.........   1   1   S04
................................................................................acugaacugacgucaagaugaaga.........   6   0   S04
.................................................................................cuUaacugacgucaagauga............   1   1   S04
.................................................................................cugaacugacgucaagaugaaga.........   1   0   S04
.................................................................................cugaacugacgucaagaugaagau........   3   0   S04
..................................................................................ugaacugacgucaagauga............   3   0   S04
..................................................................................ugaacugacgucaagaugaaga.........   1   0   S04
..................................................................................ugaacugacgucaagaugaagau........   3   0   S04
..................................................................................ugaacCgacgucaagaugaagauc.......   1   1   S04
..................................................................................ugaacugacgucaagaugaagauc.......   39  0   S04
..................................................................................ugCacugacgucaagaugaagauc.......   1   1   S04
..................................................................................ugaacugacgucaagaugaagauA.......   1   1   S04
..................................................................................ugGacugacgucaagaugaagauc.......   1   1   S04
...................................................................................gaacugacgucaagauga............   2   0   S04
...................................................................................gaacugacgucaagaugaag..........   1   0   S04
...................................................................................gaacugacgucaagaugaagauc.......   8   0   S04
...................................................................................gaacugacgucaagaugaagaucg......   18  0   S04
...................................................................................gaaAugacgucaagaugaagaucg......   1   1   S04
....................................................................................aacugacgucaagaugaagau........   2   0   S04
....................................................................................aacugacgucaagaugaagauc.......   14  0   S04
....................................................................................aacugacgucaagaugaagaucg......   6   0   S04
....................................................................................aacugacgucaagaugaagaucgu.....   3   0   S04
....................................................................................aaAugacgucaagaugaagaucgu.....   1   1   S04
....................................................................................aacugacgucaagaugaagaucguU....   1   1   S04
.....................................................................................acugacgucaagaugaagau........   1   0   S04
.....................................................................................acugacgucaagaugaagauc.......   6   0   S04
.....................................................................................acugacgucaagaugaagauA.......   1   1   S04
.....................................................................................acugacgucaagaugaagaucg......   10  0   S04
.....................................................................................acugacgucaagaugaagaucU......   1   1   S04
.....................................................................................acugacgucaagaugGagaucgug....   1   1   S04
.....................................................................................acugacgGcaagaugaagaucgug....   1   1   S04
.....................................................................................acugacgucaagaugaagaucgug....   19  0   S04
......................................................................................cugacgucaagaugaagaucU......   2   1   S04
......................................................................................cugacgucaagaugaagaucgA.....   1   1   S04
......................................................................................cugacgucaagaugaagaucUug....   1   1   S04
```

Star **Mature**

```
uauaaauauguggccgcgaaaauacuuauuaucgugucgguuggaagcccaugcuacuucugcuuguggccagggucgacacugaacugacgucaagaugaagaucgugaagu
......................................................................................cugacgucaagaugaagaucgug....   7    0    S04
......................................................................................cugacgucaagaugaagaucgGga...   1    1    S04
......................................................................................cugacgucaagaugaagaucguga...   13   0    S04
......................................................................................cugacgucaagaugaagaucgugU...   2    1    S04
......................................................................................cugacgucaagaugaagaucgugaa..   1    0    S04
.......................................................................................ugacgucaagaugaagaucgug....   1    0    S04
.......................................................................................Cgacgucaagaugaagaucguga...   1    1    S04
.......................................................................................ugacgucaagaugaagaucgugaa..   2    0    S04
........................................................................................gacgucaagaugaagaucgug....   2    0    S04
........................................................................................gacgucaagaugaagaucguga...   2    0    S04
........................................................................................gacgucaagaugaagaucgugaa..   2    0    S04
..........................................................................................cgucaagGugaagaucgugaagu   1    1    S04
```

Provisional ID	:	chrA09_896765
Score total	:	0.2
Score for star read(s)	:	-1.3
Score for read counts	:	0
Score for mfe	:	1.5
Score for randfold	:	0
Score for cons. seed	:	
Total read count	:	821
Mature read count	:	820
Loop read count	:	1
Star read count	:	0

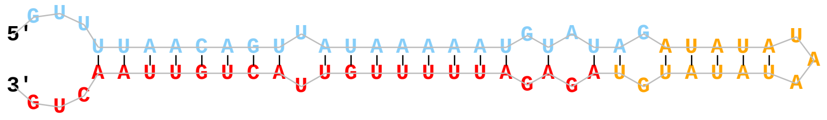

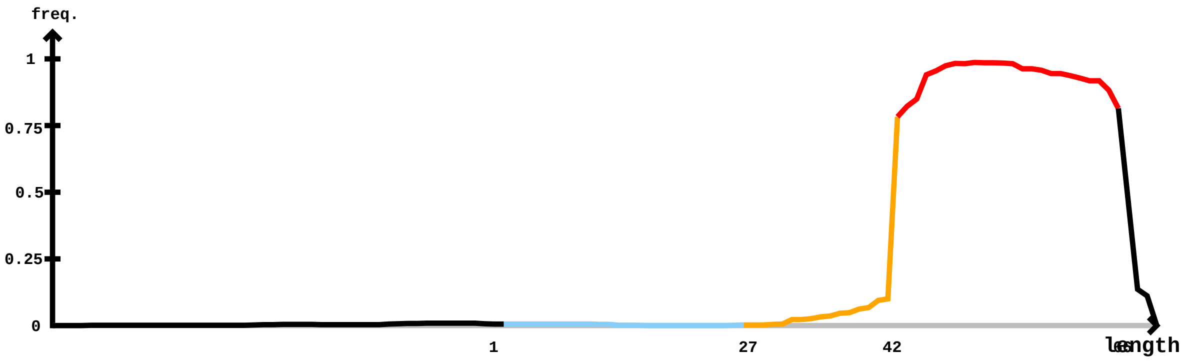

Star Mature

```
5'- acugcgguuugagcggugcauaacaagcgguucaauugcggugcgguuuuaacaguuauaaaaauguauagauauauaauauaugaagagauuuuuguuacuguuaacugcgg  -3'   exp
    (((((((((.((((.((.........)).)))))))))))))(((((..(((((((.((((((((.(.((.(((((...))))).)).).)))))))).))))))))))))..   reads   mm   sample
    .................................aauAgcggugcgguuuuaacaguu........................................................   2   1   S03
    ..................................auugcgguAcgguuuuaacaguu........................................................   1   1   S03
    .....................................gcggugcgguuuuaacaguuaua.....................................................   1   0   S03
    .....................................................................agauauauaauauauguagagauu....................   1   0   S03
    ...........................................................................auaauauauguagagauuuuuguu..............   6   0   S03
    ..............................................................................auauauguagagauuuuuguu..............   1   0   S03
    ..............................................................................aAauauguagagauuuuuguuac............   1   1   S03
    ...............................................................................uauauguagagauuuuuguuacu...........   1   0   S03
    ................................................................................auauguagagauuuuuguuacu...........   3   0   S03
    ................................................................................auauguagagauuuuuguuacugu.........   1   0   S03
    ................................................................................auauguagagauuuuuguuaAugu.........   1   1   S03
    .................................................................................uauguagagauuuuuguuacuguu........   1   0   S03
    ..................................................................................auguagagauuuuuguuacu...........   2   0   S03
    ..................................................................................auguagagauuuuuguuacuguua.......   2   0   S03
    ..................................................................................auguagagauuuuuguuacugGua.......   1   1   S03
    ...................................................................................uguagagauuuuuguuacuguua.......   3   0   S03
    ....................................................................................guagagauuuuuguuacuguuaac.....   9   0   S03
    .....................................................................................Gagagauuuuuguuacuguuaacu....   1   1   S03
    .....................................................................................uagagauuuuuguuacuguuaacu....   2   0   S03
    ......................................................................................agagauuuuuguuacugu.........   1   0   S03
    ......................................................................................agagauuuuuguuacuguuaac.....   5   0   S03
    ......................................................................................agagauuuuuguuacuguuaacu....   21   0   S03
    ......................................................................................Ggagauuuuuguuacuguuaacug...   2   1   S03
    ......................................................................................agagauuuuuguuacuguuaacuC...   1   1   S03
    ......................................................................................agagauuuuuUuuacuguuaacug...   1   1   S03
    ......................................................................................agagauuuAuguuacuguuaacug...   1   1   S03
    ......................................................................................agagauuuuuguuacuguuaacug...   173   0   S03
    ......................................................................................agagauGuuuguuacuguuaacug...   1   1   S03
    ......................................................................................agagauuuuuguuacugCuaacug...   1   1   S03
    ......................................................................................agagauCuuuguuacuguuaacug...   5   1   S03
    ......................................................................................agagauuuuGguuacuguuaacug...   1   1   S03
    ......................................................................................agagauuuuuguuacuguuaacugc..   1   0   S03
    .......................................................................................gagauuuuuguuacuguuaacu....   5   0   S03
    .......................................................................................gagauuuuuguuacuguuaacug...   2   0   S03
```

```
                                             Star                                        Mature
acugcgguuugagcggugcauaacaagcgguucaauugcggugcgguuuuaacaguuauaaaaauguauagauauauaauauauguagagauuuuuguuacuguuaacugcgg
.......................................................................................gagauuuuuguuacuguuaacugc..   6    0   S03
........................................................................................agauuuuuguuacuguuaacuC...   1    1   S03
........................................................................................agauGuuuguuacuguuaacug...   1    1   S03
........................................................................................agauuuuuguuacuguuaacugc..   1    0   S03
........................................................................................agauuuuuguuacuguuaacugcg.   6    0   S03
.........................................................................................gauuuuuguuacuguuaacug...   2    0   S03
.........................................................................................gauuuuuguuacuguuaacugc..   1    0   S03
.........................................................................................gauuuuuguuacuguuaacugcgg   15   0   S03
..........................................................................................auuuuuguuacuguuaacugc..   1    0   S03
..........................................................................................auuuuuguuacuguuaacugcgg   1    0   S03
...........................................................................................uuuuuguuacuguuaacugc..   1    0   S03
...........................................................................................uuuuuguuacuguuaacugcg.   2    0   S03
...........................................................................................uuuuuguuacuguuaacugcgg   1    0   S03
...........................................................................................uuuuGguuacuguuaacugcgg   1    1   S03
............................................................................................Guuuguuacuguuaacugcgg   1    1   S03
............................................................................................uuuuguuacuguuaacugcgg   2    0   S03
.............................................................................................uuuguuacuguuaacugcgg   1    0   S03

..Cgcgguuugagcggugcauaacaa.......................................................................................   1    1   S04
...................aGaacaagcgguucaauugcggug......................................................................   1    1   S04
....................uaacaagcgguucaauugcggug......................................................................   1    0   S04
......................acaagcgguucaauugcggugc.....................................................................   1    0   S04
...................................uugcggugcgguuuuaacaU..........................................................   1    1   S04
......................................................................gauauauaauauauguagaga......................   1    0   S04
.........................................................................auauaauauauguagagauuuu..................   1    0   S04
.........................................................................auauaauauauguagagauuuuug................   1    0   S04
..........................................................................uauaauaCauguagagauuuuugu...............   1    1   S04
...........................................................................auaauauauguagagauu....................   1    0   S04
...........................................................................auaauauauguagagauuuuuguC..............   1    1   S04
...........................................................................auaauauauguagagauuuuuguu..............   6    0   S04
...........................................................................auaauauauguagagauuuuuguG..............   1    1   S04
.............................................................................aauauauguagagauuuuuguuaU............   2    1   S04
.............................................................................aauauauguagagauuuuuguuac............   1    0   S04
..............................................................................auauauguagagauuuuuguu..............   2    0   S04
..............................................................................auauauguagagauuuuuguuacu...........   2    0   S04
...............................................................................uauauguagagauuuuuguuac............   1    0   S04
...............................................................................uauauguagagauuuuuguuacu...........   1    0   S04
................................................................................auauguagagauuuuugG...............   1    1   S04
................................................................................auauguagagauuuuuguuacu...........   1    0   S04
................................................................................auauguagagauuuuuguuacugu.........   1    0   S04
................................................................................auauguagagauuuuAguuacugu.........   1    1   S04
.................................................................................uauguagagauuuuuguuacuguu........   1    0   S04
..................................................................................auguagagauuuuuguuacu...........   1    0   S04
..................................................................................auguagagauuuuuguuacuguC........   2    1   S04
..................................................................................auguagagauuuuuguuacuguu........   2    0   S04
..................................................................................auguagagauuuuuguuacuguua.......   1    0   S04
..................................................................................auguagGgauuuuuguuacuguua.......   1    1   S04
...................................................................................uguagagauuuuuguuacuguu........   1    0   S04
...................................................................................uguagagauuuuuguuacuguua.......   1    0   S04
....................................................................................guagagauuuuuguuacugu.........   2    0   S04
....................................................................................guagagauuuuuguuacuguuaaA.....   1    1   S04
....................................................................................guagagauuuuuguuacuguuaac.....   11   0   S04
....................................................................................guagagauuuuuguuUcuguuaac.....   1    1   S04
.....................................................................................uagagauuuuuguuacuguuaacu....   2    0   S04
......................................................................................agagauuuuuguuacuguu........   1    0   S04
......................................................................................Ggagauuuuuguuacuguuaac.....   1    1   S04
......................................................................................agagauuuuuguuacuguuaac.....   3    0   S04
......................................................................................agagauuuuuguuacuguuaacu....   21   0   S04
......................................................................................agagauuuuuguuacugCuaacug...   2    1   S04
......................................................................................Ggagauuuuuguuacuguuaacug...   8    1   S04
......................................................................................Cgagauuuuuguuacuguuaacug...   1    1   S04
......................................................................................agagauuuuuguuacuguCaacug...   1    1   S04
......................................................................................agagauuuuuguuacuguuaacuC...   3    1   S04
......................................................................................Ngagauuuuuguuacuguuaacug...   2    1   S04
......................................................................................agagauuCuuguuacuguuaacug...   2    1   S04
......................................................................................agagauuuuuguuacuguuaacug...   341  0   S04
......................................................................................agGgauuuuuguuacuguuaacug...   1    1   S04
......................................................................................agagauuuuCguuacuguuaacug...   2    1   S04
......................................................................................agagauuuuuguuNcuguuaacug...   1    1   S04
......................................................................................agagauuuuuguuacuguuaacugc..   3    0   S04
```

```
                                            Star                                     Mature
acugcgguuugagcggugcauaacaagcgguucaauugcggugcgguuuuaacaguuauaaaaauguauagauauauaauauauguagagauuuuuguuacuguuaacugcgg
.......................................................................................gagauuuuuguuacuguuaacu....    7    0    S04
.......................................................................................gagauuuuuguuacuguuaacug...    4    0    S04
.......................................................................................gagauuuuuguuacuguuaacugc..    11   0    S04
........................................................................................agauuuuuguuacuguuaacu....    1    0    S04
........................................................................................agauuuuuguuacuguuaacug...    2    0    S04
........................................................................................agauuuuuguuacuguuaacugc..    2    0    S04
........................................................................................agauuuuuguuacuguuaacugcg.    10   0    S04
.........................................................................................gauuuuuguuacuguuaacug...    6    0    S04
.........................................................................................gauuuuuguuacuguuaacugc..    2    0    S04
.........................................................................................gauuuuuguuacuguuaacugcgg    51   0    S04
.........................................................................................gauuuuuguuacugCuaacugcgg    1    1    S04
.........................................................................................gauGuuuguuacuguuaacugcgg    2    1    S04
.........................................................................................gauuuuuguuacuguuaacugcgC    1    1    S04
..........................................................................................auuuuuguuacuguuaacug...    2    0    S04
..........................................................................................auuuuuguuacuguuaacugc..    1    0    S04
..........................................................................................auuuuuguuacuguuaacugcg.    2    0    S04
..........................................................................................auuuuuguuacuguuaacugcgg    6    0    S04
...........................................................................................uuuuuguuacuguuaacu....    1    0    S04
...........................................................................................uuuuuguuacuguuaacugc..    3    0    S04
...........................................................................................uuuuuguuacuguuaacugcg.    2    0    S04
...........................................................................................uuuuuguuacuguuaacugcgg    7    0    S04
............................................................................................uuuuguuacuguuaacugcgg    5    0    S04
..............................................................................................uuguuacuguuaacugcgg    3    0    S04
..............................................................................................uuguuacuguuaacugcgC    1    1    S04
```

Provisional ID	:	chrA09_random_2758710
Score total	:	664.5
Score for star read(s)	:	3.9
Score for read counts	:	668.5
Score for mfe	:	-7.8
Score for randfold	:	0
Score for cons. seed	:	
Total read count	:	1323
Mature read count	:	1322
Loop read count	:	0
Star read count	:	1

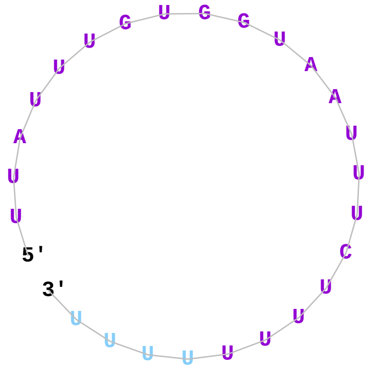

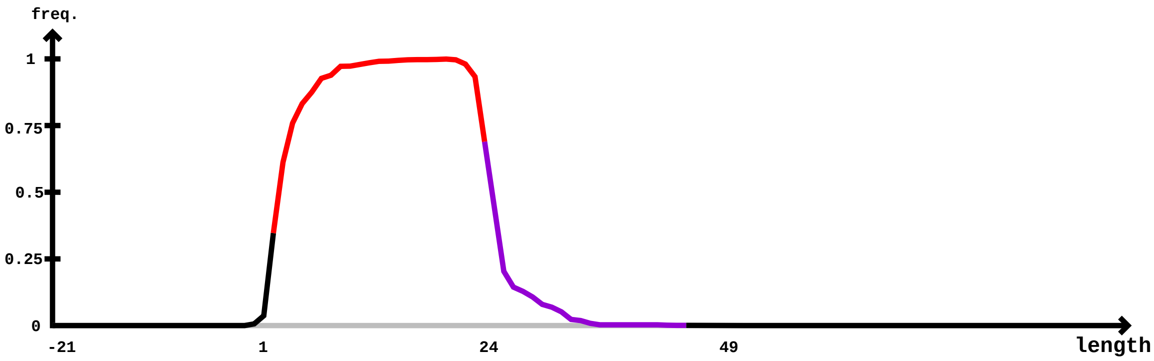


```
                      Mature       Star
5'- aaccgggaaauaaguuuucauugaaaagugguacuaugaauguguuauuugugguaaauuucuuuuuuuuuuaauccuuaaacaaacauaguuguguucaaucgaagacau  -3'   obs
    aaccgggaaauaaguuuucauugaaaagugguacuaugaauguguuauuugugguaaauuucuuuuuuuuuuaauccuuaaacaaacauaguuguguucaaucgaagacau        exp
    .............((((((...((((((...(((((..((((...))))..)))))....))))))..................(((((....))))).....))))))..   reads   mm   sample
    ...................aAugaaaagugguacuaugaaugu....................................................................   5       1    S03
    ...................Nuugaaaagugguacuaugaaugu....................................................................   2       1    S03
    ...................Cuugaaaagugguacuaugaaugugu..................................................................   2       1    S03
    ....................uugaaaagugguacuaugaaC......................................................................   1       1    S03
    ....................uugaaaagugguacuaugaauA.....................................................................   2       1    S03
    ....................uugaaaagugguacuauAaaug.....................................................................   1       1    S03
    ....................uugaaaagugguacuaugaaug.....................................................................   1       0    S03
    ....................uugaaaagugguacuaugaaugu....................................................................   6       0    S03
    ....................uugaaaagugguacuaugaaugA....................................................................   1       1    S03
    ....................Nugaaaagugguacuaugaaugug...................................................................   3       1    S03
    ....................uugaaaagugguacuaugaauguU...................................................................   1       1    S03
    ....................uugaaaagugguacuaugaaugug...................................................................   22      0    S03
    ....................uugaaaagugguacuaugaauguA...................................................................   4       1    S03
    .....................ugaaaagugguacuaugaau......................................................................   2       0    S03
    .....................ugaaaagugguacuaugaaug.....................................................................   14      0    S03
    .....................ugaaaagugguacuaugaaugu....................................................................   71      0    S03
    .....................ugaaaagugguacuaugaaugA....................................................................   1       1    S03
    .....................ugCaaagugguacuaugaaugu....................................................................   3       1    S03
    .....................ugaaaagugguacuaugaGugu....................................................................   1       1    S03
    .....................ugaaaagugguacuaugaaugG....................................................................   1       1    S03
    .....................Ngaaaagugguacuaugaaugu....................................................................   1       1    S03
    .....................ugaaaGgugguacuaugaaugu....................................................................   2       1    S03
    .....................ugaaaagugguacuaugaaugug...................................................................   91      0    S03
    .....................ugaaaagugguacuaugaauguU...................................................................   4       1    S03
    .....................ugaGaagugguacuaugaaugug...................................................................   2       1    S03
    .....................ugaaaagCgguacuaugaaugugu..................................................................   1       1    S03
    .....................ugaaaagugguacuaugaaugGgu..................................................................   1       1    S03
    .....................ugaaaagugguacuaugaauguAu..................................................................   1       1    S03
    .....................ugaaaagugguacuaugaaugugu..................................................................   28      0    S03
    .....................ugaaaagugguacuaugaaugAgu..................................................................   1       1    S03
    .....................ugaaaagugguacCaugaaugugu..................................................................   1       1    S03
    ......................gaaaagugguacuaugaa.......................................................................   2       0    S03
    ......................gaaaagugguacuaugaau......................................................................   9       0    S03
```

Mature Star

```
aaccgggaaauaaguuuucauugaaaagugguacuaugaauguguuauuugugguaauuucuuuuuuuuuuaauccuuaaacaaacauaguuguguucaaucgaagacau
......................gaaaagugguacuaugaUug....................................................................  1   1  S03
......................gaaaagugguacuaugaaug....................................................................  18  0  S03
......................gaaaGgugguacuaugaaugu...................................................................  1   1  S03
......................gaaaagugguacuaugaaugA...................................................................  2   1  S03
......................gaaaagugguacuaugaaugu...................................................................  45  0  S03
......................gaaaagugguacuaugaaugG...................................................................  2   1  S03
......................gaaaagugguacuaugaaGgu...................................................................  1   1  S03
......................gCaaagugguacuaugaaugug..................................................................  3   1  S03
......................gaaaagugguacuaugaaugug..................................................................  59  0  S03
......................gaaaagugguacuaugaauguU..................................................................  4   1  S03
......................gaaaagugguacuaugaaugAgu.................................................................  1   1  S03
......................gaaaagugguacCaugaaugugu.................................................................  1   1  S03
......................gaaaagugguacuaugaaugCgu.................................................................  1   1  S03
......................Aaaaagugguacuaugaaugugu.................................................................  1   1  S03
......................Naaaagugguacuaugaaugugu.................................................................  1   1  S03
......................gaaaagugguacuaugaaugugu.................................................................  20  0  S03
......................gaaaagugguacuaugaGugugu.................................................................  1   1  S03
......................gUaaagugguacuaugaauguguu................................................................  1   1  S03
......................gaaaGgugguacuaugaauguguu................................................................  1   1  S03
......................Aaaaagugguacuaugaauguguu................................................................  2   1  S03
......................gaaaagugguacuaugaauguguG................................................................  2   1  S03
......................gaaaagAgguacuaugaauguguu................................................................  1   1  S03
......................gaaaagugguacCaugaauguguu................................................................  1   1  S03
......................gaaaagugguacuaugGauguguu................................................................  2   1  S03
......................gaaaagugguacuaugaauguguC................................................................  1   1  S03
......................gaaaagugguacuaugaauguguu................................................................  26  0  S03
......................gaaaagugguacuaugaauguguua...............................................................  1   0  S03
.......................aaaagugguacuaugaau.....................................................................  1   0  S03
.......................aaaagugguacuaugaaug....................................................................  1   0  S03
.......................aaaagugguacuaugaaugG...................................................................  1   1  S03
.......................aaaagugguacuaugaaugu...................................................................  18  0  S03
.......................aaaagugguacuaugaaugA...................................................................  1   1  S03
.......................aGaagugguacuaugaaugug..................................................................  1   1  S03
.......................aaaagugguacuaugaaugug..................................................................  50  0  S03
.......................Caaagugguacuaugaaugug..................................................................  2   1  S03
.......................aaaagugguacuaugaauguU..................................................................  6   1  S03
.......................aaaagugguacCaugaaugugu.................................................................  1   1  S03
.......................aaaaguggAacuaugaaugugu.................................................................  1   1  S03
.......................aaaagugguaAuaugaaugugu.................................................................  1   1  S03
.......................aaaagugguacuaugaauguUu.................................................................  2   1  S03
.......................Caaagugguacuaugaaugugu.................................................................  1   1  S03
.......................aaaagugguacuaugaaugugu.................................................................  25  0  S03
.......................aaaagugguacuaugaauguguu................................................................  7   0  S03
.......................aaaagugguacuaugaauguguC................................................................  2   1  S03
.......................aaaagugguacCaugaauguguu................................................................  1   1  S03
.......................aaaagugguacuaugaauguguA................................................................  1   1  S03
.......................aaaagugguacuaugaauguguua...............................................................  3   0  S03
........................aaagugguacuaugaaugu...................................................................  8   0  S03
........................aaagugguacuaugaaugug..................................................................  16  0  S03
........................aaagugguacuaugaaugugu.................................................................  11  0  S03
........................aaagugguacuaugaauguguC................................................................  2   1  S03
........................aaagugguacuaugaauguguu................................................................  7   0  S03
........................aaagugguacuauAaauguguua...............................................................  1   1  S03
........................aaagugguacuaugaauguguuU...............................................................  3   1  S03
........................aaagugguacuaugaauguguua...............................................................  7   0  S03
........................aaagugguacuaugaauguguuG...............................................................  2   1  S03
........................aaagugguacuaugaauguguuau..............................................................  9   0  S03
........................aaagugguacuaugaauguguuaC..............................................................  4   1  S03
........................aaagugguacuaugaauguguuauA.............................................................  2   1  S03
.........................aagugguacuaugaaugA...................................................................  1   1  S03
.........................aagugguacuaugaaugu...................................................................  4   0  S03
.........................aagugguacuaugaaugug..................................................................  5   0  S03
.........................aagugguacuaugaaugugu.................................................................  5   0  S03
.........................aagugguacuaugaauguAu.................................................................  1   1  S03
.........................aagugguacuaugGaugugu.................................................................  1   1  S03
.........................aagugguacuaugaauguguC................................................................  1   1  S03
.........................aagugguacuaugaauguguu................................................................  5   0  S03
.........................aagugguaAuaugaauguguu................................................................  1   1  S03
.........................aagugguaAuaugaauguguua...............................................................  1   1  S03
.........................aagugguacuaugaauguguua...............................................................  1   0  S03
```

```
                    Mature      Star
aaccgggaaauaaguuuucauugaaaagugguacuaugaauguguuauuugugguaauuucuuuuuuuuuaauccuuaaacaaacauaguuguguucaaucgaagacau
.........................aagugguacuaugaauguguuau.............................................................   4    0    S03
.........................aagugguacuaugaauguguuaA.............................................................   1    1    S03
.........................aagugguaAuaugaauguguuauu............................................................   1    1    S03
.........................aagugguacuaugaauguguuauu............................................................   5    0    S03
.........................aagugguacuaugaauguguuauA............................................................   11   1    S03
..........................agugguacuaugaaugug.................................................................   6    0    S03
..........................agugguacuaugaaugugu................................................................   10   0    S03
..........................agugguacuaugaauguguG...............................................................   1    1    S03
..........................agugguacuaugaauguguu...............................................................   7    0    S03
..........................agugguacuauAaauguguuau.............................................................   1    1    S03
..........................agugguacuaugaauguguuaC.............................................................   1    1    S03
..........................agugguacuaugaauguguuau.............................................................   6    0    S03
..........................agugguacuaugGauguguuau.............................................................   1    1    S03
..........................agugguacuaugaauguguuauA............................................................   11   1    S03
..........................agugguacuaugaauguguuUuu............................................................   1    1    S03
..........................agugguacuaugaauguguuauG............................................................   2    1    S03
..........................agugguacuaugaauguguuauAu...........................................................   13   1    S03
..........................agugguacuaugaauguguuauuu...........................................................   1    0    S03
..........................agugguacuaugaauguguuauAug..........................................................   1    1    S03
...........................gugguacuaugaaugugu................................................................   2    0    S03
...........................gugguacuaugaauguguu...............................................................   1    0    S03
...........................gugguacuaugaauguguuauu............................................................   1    0    S03
...........................gugguacuaugaauguguuauAug..........................................................   11   1    S03
............................ugguacuaugaauguguu...............................................................   1    0    S03
............................ugguacuaugaauguguuauA............................................................   1    1    S03
............................ugguacuaugaauguguuauCug..........................................................   3    1    S03
............................ugguacuaugaauguguuauAug..........................................................   6    1    S03
............................ugguacuaugaauguguuauCugu.........................................................   26   1    S03
............................ugguacuaugaauguguuauAugu.........................................................   9    1    S03
..............................guacuaugaauguguuauCug..........................................................   2    1    S03
..............................guacuaugaauguguuauCugu.........................................................   3    1    S03
..............................guacuaugaauguguuauCugug........................................................   3    1    S03
...............................uacuaugaauguguuauCug..........................................................   1    1    S03
...............................uacuaugaauguguuauCugug........................................................   2    1    S03
...............................uacuaugaauguguuauAugugg.......................................................   1    1    S03
...............................uacuaugaauguguuauCugugg.......................................................   2    1    S03
...............................uacuaugaauguguuauCuguggu......................................................   2    1    S03
................................acuaugaauguguuauCugugg.......................................................   8    1    S03
.................................cuaugaauguguuauCugugg.......................................................   1    1    S03
..................................uaugaauguguuaAuuguggu......................................................   3    1    S03
..................................uaugaauguguuauCuguggu......................................................   1    1    S03
...................................augaauguguuauCugug........................................................   1    1    S03
...................................augaauguguuauCugugg.......................................................   1    1    S03
...................................augaauguguuaAuuguggu......................................................   1    1    S03
....................................ugaauguguuaAuuguggu......................................................   1    1    S03
.......................................augugCuauuugugguaauuucu...............................................   1    1    S03
............................................Cuauuugugguaauuucuuuu............................................   1    1    S03

....................uugaaaagugguacuaugaaugu..................................................................   1    0    S04
....................uugCaaagugguacuaugaaugug.................................................................   1    1    S04
.....................ugaaaagugguacuaugaaG....................................................................   1    1    S04
.....................ugCaaagugguacuaugaau....................................................................   1    1    S04
.....................ugaaaagugguacuaugaau....................................................................   3    0    S04
.....................ugaGaagugguacuaugaaug...................................................................   1    1    S04
.....................ugaaaagugguacuaugaaug...................................................................   14   0    S04
.....................ugaaaagugguacuaugaaugG..................................................................   1    1    S04
.....................ugaGaagugguacuaugaaugu..................................................................   1    1    S04
.....................ugaaaagugguacuaugaaugA..................................................................   9    1    S04
.....................ugaaaagugguacuaugaaugu..................................................................   75   0    S04
.....................ugCaaagugguacuaugaaugu..................................................................   4    1    S04
.....................ugaaaagugguacuaugaauguU.................................................................   4    1    S04
.....................ugaaaaguggGacuaugaaugug.................................................................   1    1    S04
.....................ugaaaagugguacuaugaaugug.................................................................   70   0    S04
.....................ugCaaagugguacuaugaaugug.................................................................   9    1    S04
.....................ugaaaagugguacuaugaGugug.................................................................   2    1    S04
.....................ugaaaGgugguacuaugaaugug.................................................................   1    1    S04
.....................ugaaaagugguacuaugaaugGg.................................................................   1    1    S04
.....................ugCaaagugguacuaugaaugugu................................................................   5    1    S04
.....................ugaaaagugguacuaugaauguAu................................................................   1    1    S04
.....................ugaaaaCugguacuaugaaugugu................................................................   1    1    S04
```

```
                    Mature       Star
aaccgggaaauaaguuuucauugaaaagugguacuaugaauguguuauuugugguaauuucuuuuuuuuuaauccuuaaacaaacauaguuguguucaaucgaagacau
.....................ugaaaagugguacuaugaaugugu................................................................   13   0   S04
.....................ugaaaGgugguacuaugaaugugu................................................................   1    1   S04
......................gaaaagugguacuaugaa.....................................................................   2    0   S04
......................gaaaagugguacuaugaau....................................................................   2    0   S04
......................gaaaagugguacuaugaaG....................................................................   1    1   S04
......................gaaaagugguacuaugaaug...................................................................   13   0   S04
......................gaaaagugguacuaugaaugA..................................................................   3    1   S04
......................gaaaagugguacuaugaaugG..................................................................   3    1   S04
......................gaaaagugguacuaugaaugu..................................................................   46   0   S04
......................gCaaagugguacuaugaaugu..................................................................   3    1   S04
......................gaaaagugguacuaugaGugug.................................................................   1    1   S04
......................gaaaagugguacuaugaaugug.................................................................   72   0   S04
......................gaaaagugguacuaugaauguU.................................................................   3    1   S04
......................gCaaagugguacuaugaaugug.................................................................   8    1   S04
......................gaaaagugguacuaugaaugugu................................................................   9    0   S04
......................gGaaagugguacuaugaaugugu................................................................   1    1   S04
......................gaaaagugguacuaugaauguUu................................................................   1    1   S04
......................gCaaagugguacuaugaaugugu................................................................   1    1   S04
......................gaaaagugguacuaugaauguguu...............................................................   1    0   S04
.......................aaaagugguacuaugaau....................................................................   2    0   S04
.......................aaaagugguacuaugaaug...................................................................   1    0   S04
.......................aaaagugguacuaugaaugu..................................................................   12   0   S04
.......................aaaagugguacuaugaaugG..................................................................   3    1   S04
.......................Caaagugguacuaugaaugug.................................................................   4    1   S04
.......................aaaagugguacuaugaaugug.................................................................   39   0   S04
.......................aaaagugguacuaugaauguU.................................................................   3    1   S04
.......................Naaagugguacuaugaaugug.................................................................   1    1   S04
.......................Caaagugguacuaugaaugugu................................................................   4    1   S04
.......................aaaagugguacuaugaaugugu................................................................   8    0   S04
.......................aaaagugguaAuaugaaugugu................................................................   2    1   S04
.......................aaaagugguaAuaugaauguguu...............................................................   2    1   S04
.......................Gaaagugguacuaugaauguguu...............................................................   1    1   S04
.......................aaaagugguacuaugaauguguu...............................................................   2    0   S04
.......................aaaagugguaAuaugaauguguua..............................................................   1    1   S04
........................aaagugguacuaugaaug...................................................................   1    0   S04
........................aaagugguacuaugaaugu..................................................................   7    0   S04
........................aaagugguacuaugaaugug.................................................................   5    0   S04
........................aaagugguacuaugaauguU.................................................................   1    1   S04
........................aaagugguaAuaugaaugugu................................................................   2    1   S04
........................aaagugguacuaugaaugugu................................................................   6    0   S04
........................aaagugguacCaugaaugugu................................................................   1    1   S04
........................aaagugguacuaugaauguAu................................................................   2    1   S04
........................aaagugguacuaugaauguguu...............................................................   1    0   S04
........................aaagugguacuaugaauguguA...............................................................   1    1   S04
........................aaagugguacuaugaauguguuU..............................................................   1    1   S04
........................aaagugguacuaugaauguguuG..............................................................   2    1   S04
........................aaagugguacuaugaauguguuGu.............................................................   1    1   S04
........................aaagugguacuaugaauguguuau.............................................................   2    0   S04
.........................aagugguacuaugaaugA..................................................................   1    1   S04
.........................aagugguacuaugaaugu..................................................................   3    0   S04
.........................aagugguacuaugaaugug.................................................................   1    0   S04
.........................aagugguacuaugaaugugu................................................................   1    0   S04
.........................aagugguaAuaugaaugugu................................................................   2    1   S04
.........................aagugguacuaugaauguguA...............................................................   1    1   S04
.........................aagugguacuaugaauguguu...............................................................   1    0   S04
.........................aagugguaAuaugaauguguu...............................................................   1    1   S04
.........................aagugguacuaugaauguguuauu............................................................   1    0   S04
.........................aagugguacuCugaauguguuauu............................................................   2    1   S04
..........................agugguacuaugaaugug.................................................................   4    0   S04
..........................agugguaAuaugaaugugu................................................................   1    1   S04
..........................agugguacuaugaaugugu................................................................   4    0   S04
..........................agugguacuaugaauguguuauA............................................................   2    1   S04
..........................agugguacuaugaauguguuauuu...........................................................   1    0   S04
...........................gugguacuaugaaugugu................................................................   1    0   S04
...........................gugguacuCugaauguguuauuug..........................................................   1    1   S04
............................ugguacuaugaauguguuauCugu.........................................................   1    1   S04
............................ugguacuaugaauguguuauuugu.........................................................   1    0   S04
.............................gguacuCugaauguguuauuugu.........................................................   1    1   S04
..............................guaAuaugaauguguuauuugugg.......................................................   1    1   S04
...............................uacuaugaauguguuauCugugg.......................................................   1    1   S04
```

Mature **Star**

```
aaccgggaaauaaguuuucauugaaaagugguacuaugaauguguuauuuugugguaauuucuuuuuuuuuuaauccuuaaacaaacauaguuguguucaaucgaagacau
......................................aauguguuauuuguggCaauuucu.................................................	1	1	S04
.......................................augugGuauuugugguaauuucuu................................................	1	1	S04
```

Provisional ID	:	chrA09_random_2761059
Score total	:	0.4
Score for star read(s)	:	-1.3
Score for read counts	:	0
Score for mfe	:	1.7
Score for randfold	:	0
Score for cons. seed	:	
Total read count	:	470
Mature read count	:	470
Loop read count	:	0
Star read count	:	0

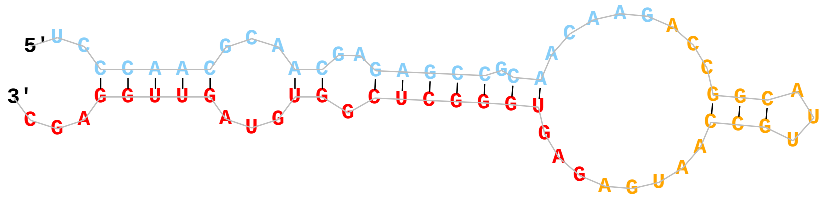

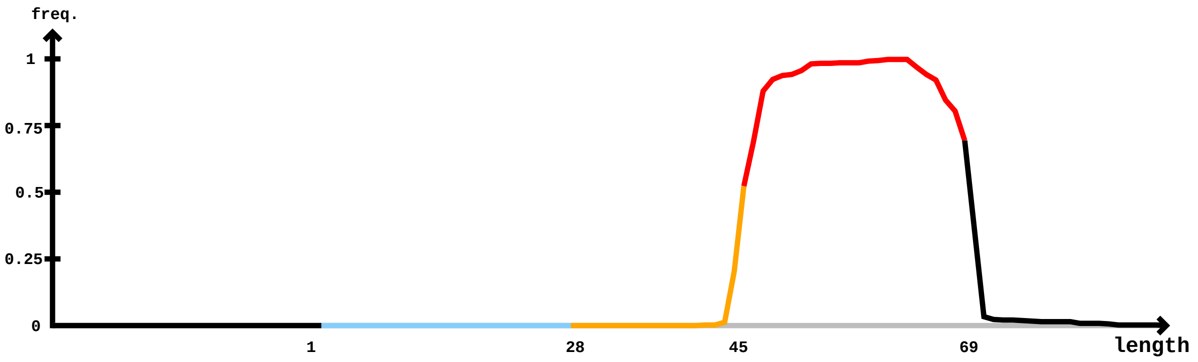

Star Mature

5'- ccguucuuguccaagguuccuccuccucccaacgcaacgagagccgcaacaagaccggcauugccaaugagagugggcucgguguaguuggagcugguccggacaucuuggaag -3'	exp		
.........((((((((((.((..((((.(((...((..(((((.((........(((...)))........))))))).))...)))))))..))...))).))))))))..	reads	mm	sample
....aCgagagugggcucgguguaguu.........................	1	1	S03
....gagagugggcucgAuguaguug........................	1	1	S03
....agagugggcucgguguagC..........................	1	1	S03
....agagugggcucgguguagu..........................	5	0	S03
....agagugggcucggCguagu..........................	1	1	S03
....agagugggcucgguguaguG.........................	1	1	S03
....agagugggcucgguguaguu.........................	4	0	S03
....agagugggcucgguguaguC.........................	2	1	S03
....agagugggcucgguguaguug........................	2	0	S03
....agagugggcucgUuguaguug........................	1	1	S03
....Ngagugggcucgguguaguugg.......................	1	1	S03
....agagugggcucgguguaguugA.......................	1	1	S03
....agagugggcucgguguaguugg.......................	5	0	S03
....agaUugggcucgguguaguugg.......................	1	1	S03
....agagugggcucgguguaguuggG......................	1	1	S03
....agagugggcucgguguaguugga......................	7	0	S03
....agagugggcucgguguaguuggaA.....................	1	1	S03
....agagugggcucgguguaguuggag.....................	14	0	S03
....agagugggcucgguguaguuggagU....................	2	1	S03
....agagugggcucgguguaguuggagc....................	4	0	S03
....agagugggcucgguguUguuggagc....................	1	1	S03
....gagugggcucgguguagu..........................	2	0	S03
....gUgugggcucgguguaguu.........................	1	1	S03
....gagugggcucgguguaguugg.......................	3	0	S03
....gagugggcucgguguaguugga......................	2	0	S03
....gGgugggcucgguguaguuggag.....................	1	1	S03
....gagugggcucgguguaguuggag.....................	2	0	S03
....gaAugggcucgguguaguuggag.....................	1	1	S03
....gagugggcucgguguaguuggagA....................	5	1	S03
....gUgugggcucgguguaguuggagc....................	16	1	S03
....gagugggcucgguguaguuggagU....................	8	1	S03
....gagugggcucgguguaguuggagc....................	44	0	S03
....agugggcucgguguaguu.........................	1	0	S03
....agugggcucgguguaguug........................	1	0	S03

Star Mature

ccguucuuguccaagguuccuccuccucccaacgcaacgagagccgcaacaagaccggcauugccaaugagagugggcucgguguaguuggagcugguccggacaucuuggaag

....agugggcucgguguaguugA.......................	1	1	S03
....agugggcucgguguaguugg.......................	3	0	S03
....agugggcucgguguaguugga......................	1	0	S03
....agugggcucgguguaguuggaA.....................	1	1	S03
....agugggcucgguguaguuggag.....................	8	0	S03
....agugggcucgguguaguuggagA....................	1	1	S03
....agugggcucggCguaguuggagc....................	1	1	S03
....agugggcucgguguaguuggagU....................	3	1	S03
....agugggcucgguguaguuggagc....................	2	0	S03
....agugggcucgguguaguuggagcu...................	22	0	S03
....agugggcucgguguaguuggagcC...................	2	1	S03
....Ggugggcucgguguaguuggagcu...................	1	1	S03
....agugggcucgguguaguuggagcG...................	2	1	S03
....gugggcucgguguaguuU........................	1	1	S03
....gugggcucgguguaguugg.......................	3	0	S03
....gugggcucgguguaguugga......................	2	0	S03
....gugggcucgguguaguuggaA.....................	2	1	S03
....gugggcucgguguaguuggaC.....................	1	1	S03
....gugggcucgguguaguuggagA....................	4	1	S03
....gugggcucgguguaguuggagG....................	1	1	S03
....gugggcucgguguaguuggagU....................	6	1	S03
....gGgggcucgguguaguuggagc....................	1	1	S03
....gugggcucgguguaguuggagc....................	21	0	S03
....gugggcucgguguaguuggagcA...................	1	1	S03
....gugggcucgguguaguuggagcu...................	8	0	S03
....gugggcucgguguaguuggagcC...................	2	1	S03
....gCgggcucgguguaguuggagcu...................	1	1	S03
....gugggcucgguguaguuggGgcu...................	1	1	S03
....gugggcucgguguaguuggagcuC..................	1	1	S03
....ugggcucgguguaguugga......................	2	0	S03
....ugggcucgguguaguuggag.....................	1	0	S03
....ugggcucgguguaguuggagU....................	1	1	S03
....ugggcucgguguaguuggagA....................	1	1	S03
....ugggcucgguguaguuggagc....................	2	0	S03
....ugggcucgguguaguuggaAc....................	1	1	S03
....ugggcucgguguaguuggagcA...................	1	1	S03
....ugggcucgguguaguuggagcu...................	2	0	S03
....ugggcucgguguaguuggagcuA..................	1	1	S03
....gggcucgguguaguuggag.....................	1	0	S03
....gggcucgguguaguuggagc....................	3	0	S03
....gggcucgguguaguuggagcu...................	1	0	S03
....ggcucgguguaguuggagc....................	1	0	S03
....ggcucgguguaguuggagA....................	1	1	S03
....gcucgguguaguuggagcu...................	2	0	S03
....gcucgguguaguuggagcA...................	1	1	S03
....gcucgguguaguuggagcuCg.................	1	1	S03
....cucgguguaguuggagcA...................	1	1	S03
....cucgguguaguuggagcG...................	1	1	S03
....cucgguguaguuggagcu...................	1	0	S03
....cucgguguaguuggagcC...................	1	1	S03
....cucgAuguaguuggagcug..................	1	1	S03
....cucgguguaguuggagcuCguccg.............	1	1	S03
....ucgguguaguuggagcuA..................	1	1	S03
....gguguaguuggagcuAgucc..............	1	1	S03
....aguuggagcuCguccggacaucuu.....	2	1	S03
....gagagugggcucgAuguagu..........................	1	1	S04
....gagagugggcucggGguaguu.........................	1	1	S04
....gagUgugggcucgguguaguu.........................	1	1	S04
....gagagugggcucgAuguaguug........................	1	1	S04
....agagugggcucgguguaUu..........................	1	1	S04
....agagugggcucgguguagu..........................	2	0	S04
....agagugggcucgguguaguug........................	2	0	S04
....agagugggcucgguguaguugg.......................	5	0	S04
....agagugggcucgguguaguugA.......................	1	1	S04
....Ggagugggcucgguguaguugg.......................	1	1	S04
....agagugUgcucgguguaguugg.......................	1	1	S04
....agagugggcucgguAuaguugg.......................	1	1	S04
....agagugggcucgguguaguugga......................	2	0	S04
....agagugggcucgguguaguuggaA.....................	3	1	S04

Star Mature

ccguucuuguccaagguuccuccuccucccaacgcaacgagagccgcaacaagaccggcauugccaaugagagugggcucgguguaguuggagcugguccggacaucuuggaag			
....agagugggcucggugCaguuggag.....................	1	1	S04
....agagugggcucgguguaguuggag.....................	8	0	S04
....agagugggcucgguguagCuggag.....................	1	1	S04
....agagugggcucgguguaguuggagU....................	5	1	S04
....agagugggcucgguguaguuggagc....................	2	0	S04
....agagugggcucgguguaguuggaUcu...................	1	1	S04
....gagugggcucgguguagu..........................	1	0	S04
....gagugggcucgguguaguu.........................	1	0	S04
....gagugggcucgguguaguugg.......................	5	0	S04
....gUgugggcucgguguaguugg.......................	1	1	S04
....gagugggcucgguguaguuggag.....................	2	0	S04
....gUgugggcucgguguaguuggag.....................	1	1	S04
....gagugggGucgguguaguuggag.....................	1	1	S04
....gagugggcucgguguGguuggagc....................	1	1	S04
....gagugggcucgguguaguuggagc....................	31	0	S04
....gagugggcucgCuguaguuggagc....................	1	1	S04
....Uagugggcucgguguaguuggagc....................	1	1	S04
....gagAgggcucgguguaguuggagc....................	1	1	S04
....gagugggcucgguguaguuggagU....................	9	1	S04
....gUgugggcucgguguaguuggagc....................	4	1	S04
....gagugggcucgguguaguuggagA....................	3	1	S04
....Aagugggcucgguguaguuggagc....................	1	1	S04
....gagugggcucgguguaguuggagcu...................	2	0	S04
....gUgugggcucgguguaguuggagcu...................	2	1	S04
....agugggcucgguguaguug........................	1	0	S04
....agugggcucgguguaguugg.......................	1	0	S04
....agugggcucgguguaguugga......................	2	0	S04
....agugggcucgguguaguuggag.....................	2	0	S04
....agugggcucgguguaguuggagA....................	2	1	S04
....agugggcucgguguaguuggagc....................	3	0	S04
....agugggcucgguguaguuggagU....................	1	1	S04
....agugggcucggGguaguuggagc....................	1	1	S04
....agugggcucgguguaguuggagcu...................	10	0	S04
....agugggcucgguguUguuggagcu...................	1	1	S04
....agugggcucgguguaguuggagcC...................	1	1	S04
....Ugugggcucgguguaguuggagcu...................	1	1	S04
....agugggcucgguguaguuggagcA...................	1	1	S04
....agugggcucggugAaguuggagcu...................	1	1	S04
....agugggcucgguguaguuggagcG...................	2	1	S04
....gugggcucgguguaguugg.......................	1	0	S04
....gugggcucgguguaguugU.......................	1	1	S04
....gugggcucgguguaguugga......................	2	0	S04
....gugggcucgguguaguuggag.....................	1	0	S04
....gugggcucgguguaguuggagc....................	11	0	S04
....gGgggcucgguguaguuggagc....................	1	1	S04
....gugggcucgguguaguuggagU....................	6	1	S04
....gugggcucgguguaguuggagcA...................	1	1	S04
....guggAcucgguguaguuggagcu...................	1	1	S04
....gugggcucgguguaguuggagcu...................	8	0	S04
....gugggcucgguguaguuggagcG...................	1	1	S04
....gugggcucgguguaguuggagcug..................	1	0	S04
....ugggcucgguguaguuggagc....................	4	0	S04
....ugggcCcgguguaguuggagc....................	1	1	S04
....ugggcucgguguaguuggagUu...................	1	1	S04
....Ngggcucgguguaguuggagcu...................	1	1	S04
....ugggcucgguguaguuggagcu...................	2	0	S04
....gggcucgguguaguuggagc....................	1	0	S04
....gggcucgguguaguuggagcC...................	1	1	S04
....gcucgguguaguuggagcu...................	3	0	S04
....cucgguguaguuggagcu...................	4	0	S04
....cucgguAuaguuggagcu...................	1	1	S04
....cucgguguaguuggagcugguc...............	1	0	S04
....guaguuggagcuCguccggaca.........	3	1	S04
....uaguuggagcuCguccggacaucu......	1	1	S04
....gcuUguccggacaucuuggaag	1	1	S04

Provisional ID	:	chrA10_903655
Score total	:	0.3
Score for star read(s)	:	3.9
Score for read counts	:	-4.4
Score for mfe	:	0.9
Score for randfold	:	0
Score for cons. seed	:	
Total read count	:	3
Mature read count	:	1
Loop read count	:	1
Star read count	:	1

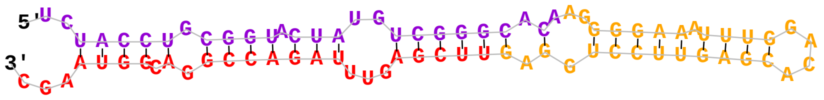

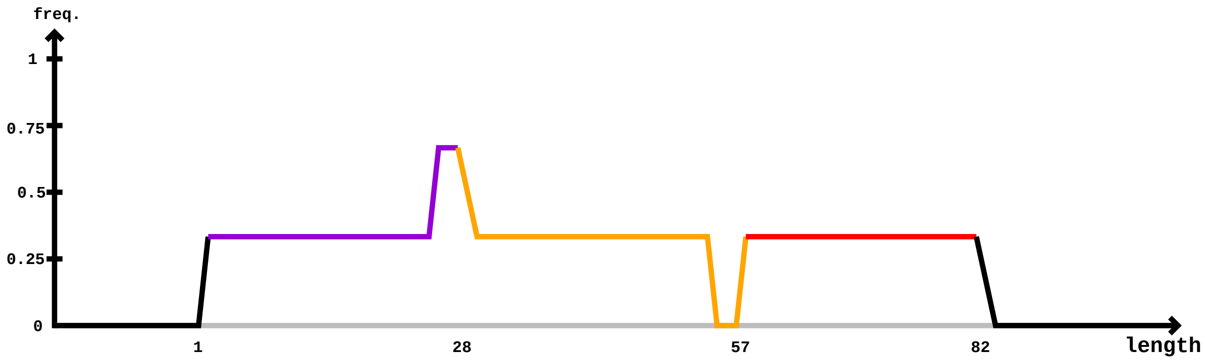


```
                 Star                                                    Mature
5'- agaagaaaacgaguucuaccugcgguacuaugucgggcacaaggggaaauuuggacacgaguuccuggaguucgaguuuagaccggacgguaagcuccgauacgccaauaacuca   -3'    obs
    agaagaaaacgaguucuaccugcgguacuaugucgggcacaaggggaaauuuggacacgaguuccuggaguucgaguuuagaccggacgguaagcuccgauacgccaauaacuca          exp
    ..........(((((.(((((.((((.(((..((((((.(...(((((.((((....))))))))).).))))))...))))))).).)))))))))..................    reads     mm      sample
    ......................................acaaggggaaauuuggacacgaguuccug................................................    1         0       S03

    ..............ucuaccugcgguacuaugucgggcaca..........................................................................    1         0       S04
    ......................................................................uucgaguuuagaccggacgguaagc....................    1         0       S04
```

Provisional ID	:	chrA10_905376
Score total	:	20.9
Score for star read(s)	:	3.9
Score for read counts	:	15.9
Score for mfe	:	1.1
Score for randfold	:	0
Score for cons. seed	:	
Total read count	:	43
Mature read count	:	29
Loop read count	:	11
Star read count	:	3

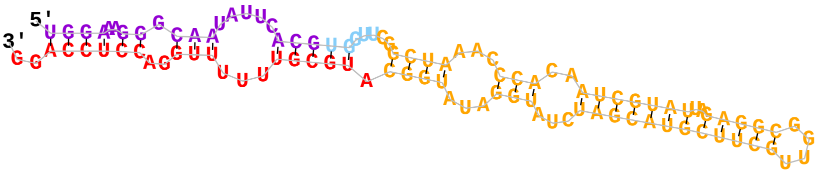

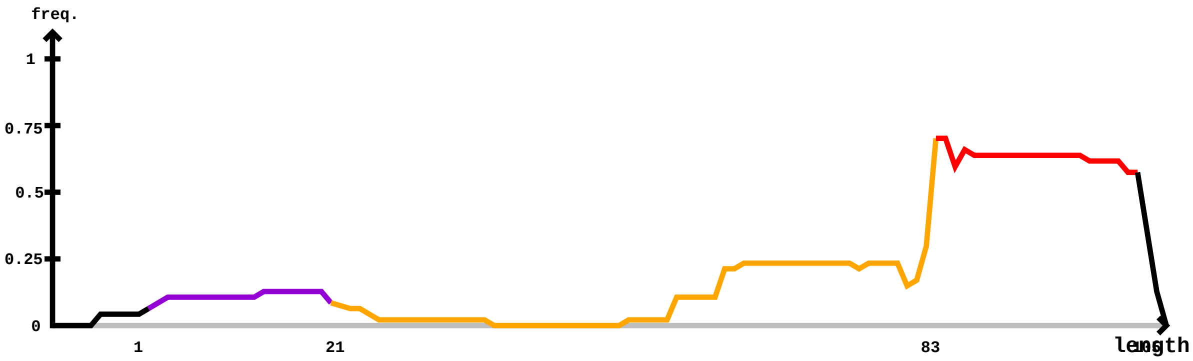

Star **Mature**

```
5'- ccaccgcuuggaaagggcaauauucacgugguucggcuaaacccacaaucguauuagaggcgguugcuucguacgaucuauggauauggcaugcguuuuuuggaccuccaggua  -3'   obs
    ccaccgcuuggaaagggcaauauucacgugguucggcuaaacccacaaucguauuagaggcgguugcuucguacgaucuauggauauggcaugcguuuuuuggaccuccaggua         exp
    .....(((((((..((.(((.....(((((.....((((...(((..(((((((..(((((....))))))))))))...)))...)))).)))))...)))..))))))))).   reads   mm   sample
    ....................uauucacgugguucggcuaaaccc......................................................................   1       0    S03
    ...............................................................uugcuucguacgaucuauggauau...........................   2       0    S03
    ....................................................................ucguacgaucuauggauauggcau......................   1       0    S03
    ..........................................................................................augcguuuuuuggaccuccaggCa   1       1    S03

    ...ccgcuuggaaagggcaauauucac.......................................................................................   2       0    S04
    ........uggaaagggcaauauucacA......................................................................................   1       1    S04
    .........ggaaagggcaauauucacguggu..................................................................................   1       0    S04
    ..........gaaagggcaauauucacgugg...................................................................................   1       0    S04
    ..........................................................ggcgguugcuucguacgaucuaug................................   1       0    S04
    ...............................................................uugcuucguacgaucuauggauau...........................   2       0    S04
    ....................................................................ucguacgaucuauggauaugAcau......................   2       1    S04
    ....................................................................ucguacgaucuauggauauggcau......................   2       0    S04
    ......................................................................guacgaucuauggauauggcaugU....................   1       1    S04
    ...................................................................................auauggcaugcguuuuuuggacA........   1       1    S04
    ........................................................................................gcaugcguuuuuuggaccucGa....   1       1    S04
    .........................................................................................caugcguuuuuuggaccuccG....   1       1    S04
    .........................................................................................caugcguuuuuuggaccuccagA..   2       1    S04
    .........................................................................................caugcguuuuuuggaccuccagUu.   1       1    S04
    .........................................................................................caugcguuuuuuggaccuccagAu.   2       1    S04
    ..........................................................................................augcguuuuuuggaccuccagU..   1       1    S04
    ..........................................................................................augcguuuuuuggaccuccagA..   13      1    S04
    ..........................................................................................augcguuuuuuggaccuccagAu.   1       1    S04
    ..........................................................................................augcguuuuuuggaccuccagAua   3       1    S04
    .............................................................................................cguuuuuuggaccucUaggu.   1       1    S04
    .............................................................................................cguuuuuuggaccuccagAua   2       1    S04
```

Provisional ID	:	chrA10_925211
Score total	:	11.7
Score for star read(s)	:	3.9
Score for read counts	:	5.7
Score for mfe	:	2.1
Score for randfold	:	0
Score for cons. seed	:	
Total read count	:	23
Mature read count	:	16
Loop read count	:	1
Star read count	:	6

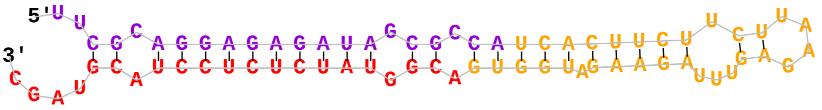

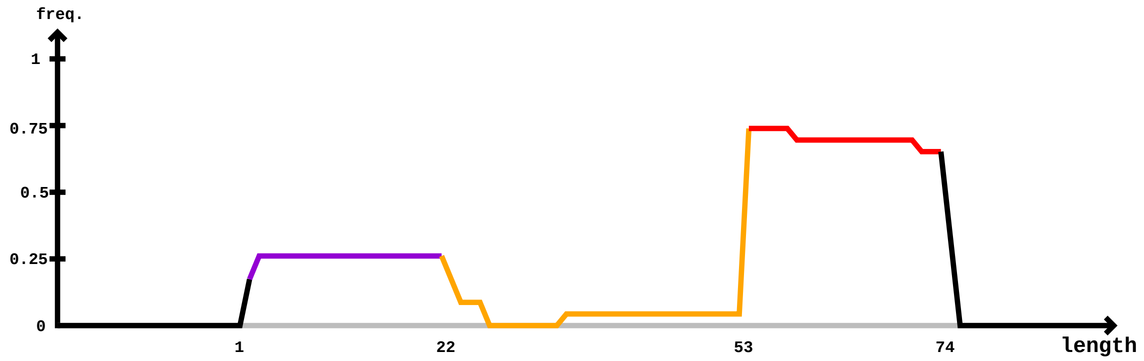

Star　　　　　　　　　　　　　　　　Mature

```
5'- cugcaaauaaagguucgcuucgcaggagagagauagcgccaucacuucuucuuaagaguuuagaagauggugacgguaucucuccuacguagcaauccuuauauaugcau   -3'   obs
    cugcaaauaaagguucgcuucgcaggagagagauagcgccaucacuucuucuuaagaguuuagaagauggugacgguaucucuccuacguagcaauccuuauauaugcau         exp
    .((((.(((((((...(((.((.((((((((((.((.((((((((((.((....))...))))).))))).)).))))))))))).)).)))...)))).))).)))).    reads    mm    sample
    ..................uucgcaggagagagauagcgcca.....................................................................    1        0     S03
    ...................ucgcaggagagagauagcgccaucac.................................................................    1        0     S03
    ........................................................................acgguaucucuccuacgu....................    1        0     S03
    ........................................................................Gcgguaucucuccuacguagc.................    1        1     S03
    ........................................................................acgguaucucuccuacguagU.................    1        1     S03
    ........................................................................acgguaucucuccuacguagc.................    5        0     S03

    ..................uucgcaggagagagauagcgccG.....................................................................    1        1     S04
    ..................uucgcaggagagagauagcgccU.....................................................................    1        1     S04
    ..................uucgcaggagagagauagcgcca.....................................................................    1        0     S04
    ...................ucgcaggagagagauagcgccaucaA.................................................................    1        1     S04
    .....................................................aagaguuuagaagauggugacggA.................................    1        1     S04
    ........................................................................acgguaucucuccuacguagA.................    1        1     S04
    ........................................................................acgguaucucuccuacguagU.................    1        1     S04
    ........................................................................acgguaucucuccuacguagc.................    6        0     S04
```

Provisional ID	:	chrA10_943534
Score total	:	0.3
Score for star read(s)	:	-1.3
Score for read counts	:	0
Score for mfe	:	1.6
Score for randfold	:	0
Score for cons. seed	:	
Total read count	:	16
Mature read count	:	16
Loop read count	:	0
Star read count	:	0

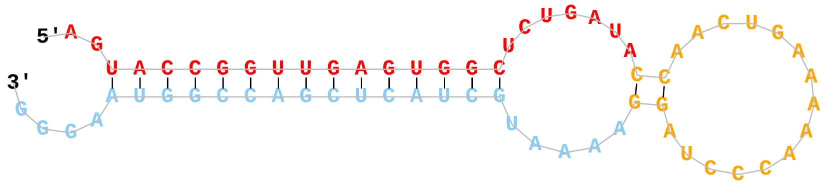

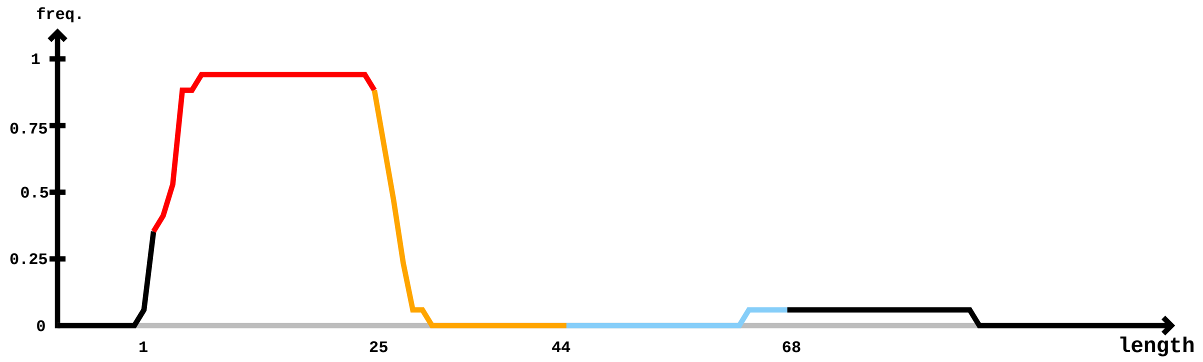

Mature Star

```
5'- aacagacaaguaccgguugaguggcucugauaccaacugaaaaacccuaggaaaaugcuacucgaccgguaagggggauauggacucagauccgguaggauugagaacuggugg   -3'    exp
    ..(((.(...(((((((((((((((.......((...............)).....)))))))))))))))...).........(((((.(((....))))))))..)))....   reads      mm      sample
    ........aguaccgguugaguggcucugaua..................................................................................   3          0       S03
    ...........accgguugaguggcucugauacc................................................................................   2          0       S03
    ...........accgguugaguggcucugauacca...............................................................................   1          0       S03
    ......................................................................aagggggauauggacucagauccg....................   1          0       S03

    .......aaguaccgguugaguggcucugau...................................................................................   1          0       S04
    ........aguaccgguugaguggcucugaua..................................................................................   2          0       S04
    .........guaccgguugaguggcucugauaccU...............................................................................   1          1       S04
    ..........uaccgguugaguggcucugauacA................................................................................   1          1       S04
    ..........uaccgguugaguggcucugauaccU...............................................................................   1          1       S04
    ...........accgguugaguggcCcugaua..................................................................................   1          1       S04
    ...........accgguugaguggcucugauac.................................................................................   1          0       S04
    ...........accgguugaguggcucugauacA................................................................................   1          1       S04
    .............cgguugaguggcucugauaccaaG.............................................................................   1          1       S04
```

Provisional ID	:	chrA10_956355
Score total	:	0
Score for star read(s)	:	-1.3
Score for read counts	:	0
Score for mfe	:	1.2
Score for randfold	:	0
Score for cons. seed	:	
Total read count	:	54
Mature read count	:	54
Loop read count	:	0
Star read count	:	0

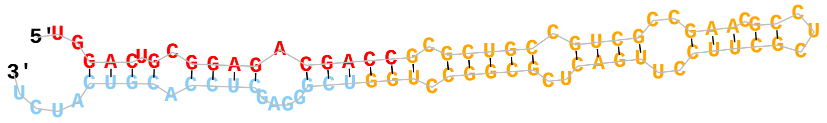

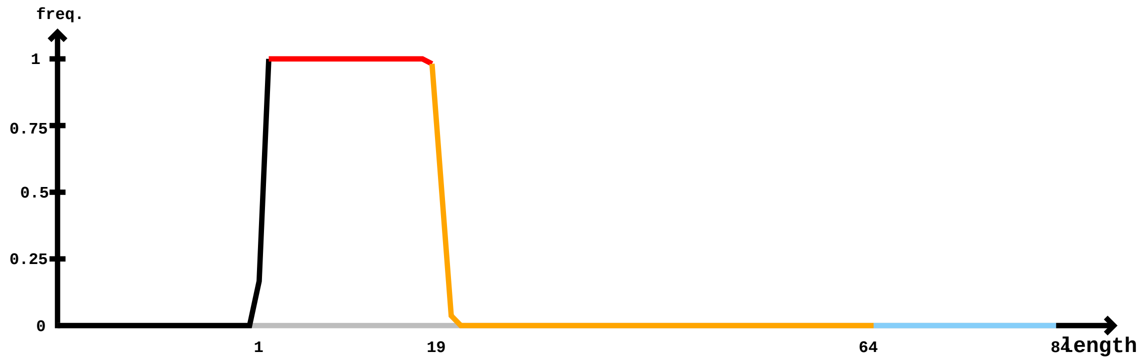

Mature Star

```
5'- aucuuguaaauuguaacgcauggacugcggagacgaccgcgcugccgucgccgaacgccucgcuuccuugacucgcggccuggucgggagcuccacgucaucuucuuu   -3'    exp
    ......................(((.(.((((.((((((.(((((.((((..(((.((...)))))..))))..))))).))))))....)))).)))).........   reads   mm   sample
    ...................Guggacugcggagacgaccg.....................................................................   4       1    S03
    ...................auggacugcggagacgaccgU....................................................................   1       1    S03
    ...................Guggacugcggagacgaccgc....................................................................   1       1    S03
    ....................uggacugcggagacgacA......................................................................   3       1    S03
    ....................uggacugcggagUcgacc......................................................................   1       1    S03
    ....................uggacugcggagacgacc......................................................................   16      0    S03
    ....................uggacugcggagacgacU......................................................................   4       1    S03
    ....................uggacugcggagGcgacc......................................................................   1       1    S03

    ...................Uuggacugcggagacgac.......................................................................   1       1    S04
    ...................Guggacugcggagacgaccg.....................................................................   2       1    S04
    ....................uggacugcggagacgacU......................................................................   3       1    S04
    ....................uggacugcggagacgacc......................................................................   13      0    S04
    ....................uggacugcggagacgacA......................................................................   2       1    S04
    ....................ugUacugcggagacgacc......................................................................   1       1    S04
    ....................uggacugcggagacgacUg.....................................................................   1       1    S04
```

Provisional ID	:	chrA10_957408
Score total	:	148.9
Score for star read(s)	:	3.9
Score for read counts	:	164.3
Score for mfe	:	-19.2
Score for randfold	:	0
Score for cons. seed	:	
Total read count	:	334
Mature read count	:	310
Loop read count	:	17
Star read count	:	7

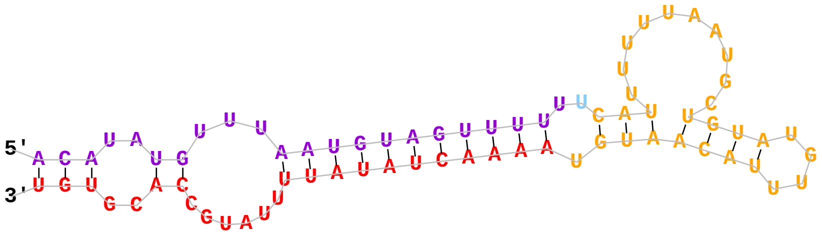

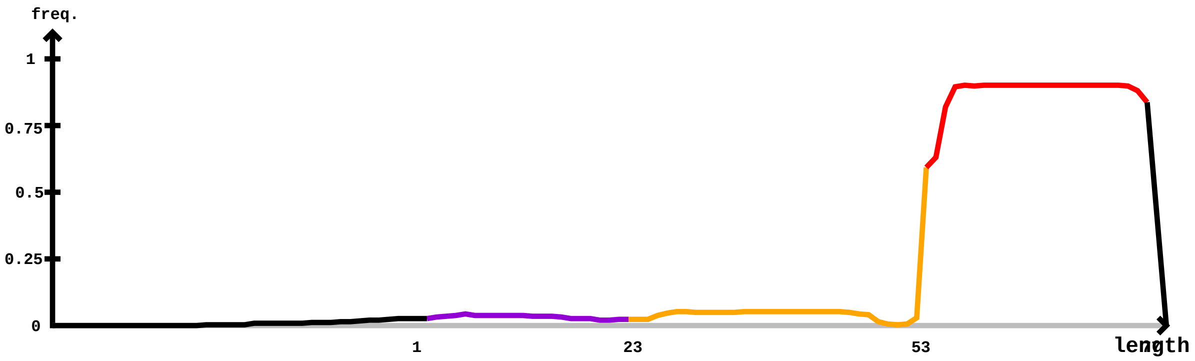

Star **Mature**

```
5'- aagccauuuuuuuuuuuaagugaaaaauaguaaucuuacauauguuuaauguaguuuuuucauuuuuuaaugcuguauguuuacaauguaaaacuauauuuuaugccacguguc  -3'   obs
    aagccauuuuuuuuuuuaagugaaaaauaguaaucuuacauauguuuaauguaguuuuuucauuuuuuaaugcuguauguuuacaauguaaaacuauauuuuaugccacguguc         exp
    ..((.(((((((.........))))))).))......(((..((...((((((((((..(((..........((((....))))))).))))))))))......))..))).    reads   mm   sample
    ..............uuuaagugaaaaauaguaaucuu.............................................................................    1       0    S03
    ...................gugaaaaauaguaaucuuacaua........................................................................    2       0    S03
    .........................Gauaguaaucuuacauauguuua..................................................................    1       1    S03
    ............................aguaaucuuacauauguuuaaugC..............................................................    1       1    S03
    ..............................uaaucuuacauauguuuaaugu..............................................................    1       0    S03
    .................................ucuuacaCauguuuaauguagu...........................................................    1       1    S03
    ..................................cuuacauauguuuaaugCagu...........................................................    1       1    S03
    ......................................cauauguuuaauguaguuuuu.......................................................    1       0    S03
    .......................................auauguuuaauguaguAuuuuca....................................................    1       1    S03
    .........................................................uuucauuuuuuaaugcuguauguu.................................    1       0    S03
    ............................................................cauuuuuuaaugcuguauguuu................................    1       0    S03
    ............................................................cauuuuuuaaugcuguauguuuac..............................    1       0    S03
    .............................................................auuuuuuaaugcuguauguuuac..............................    2       0    S03
    .............................................................auuuuuuaaugcuguauguuuaca.............................    2       0    S03
    ..............................................................uuuuuuaaugcuguauguuuac..............................    5       0    S03
    ...............................................................uuuuuaaugcuguauguuua...............................    1       0    S03
    ......................................................................ugcuguauguuuacaauguaaaac....................    1       0    S03
    .......................................................................................guaaaacuauauuuuaugccacgu...    1       0    S03
    ........................................................................................uaaaacuauauuuuaugccacgu...    1       0    S03
    ........................................................................................uaaaacuauauuuuaugccacgug..    5       0    S03
    .........................................................................................aaaacuauauuuuaugccacg....    1       0    S03
    .........................................................................................aaaacuauauuuuaugccacgu...    2       0    S03
    .........................................................................................aaaacuauauuuuaugccacgug..    1       0    S03
    .........................................................................................aaaacuauauuuuaCgccacgug..    1       1    S03
    .........................................................................................aaaacuauauuuuaugccacguA..    2       1    S03
    .........................................................................................aaaacuauauuuuaugccacgugG.    2       1    S03
    .........................................................................................aaaacuauauuuuaugccacgGgu.    1       1    S03
    .........................................................................................aaaacuauauuuuaugccacgugC.    7       1    S03
    .........................................................................................aaaUcuauauuuuaugccacgugu.    1       1    S03
    .........................................................................................aaaacuauauuuuaugccacgugA.    4       1    S03
    .........................................................................................aaaacuauauuuuaCgccacgugu.    1       1    S03
    .........................................................................................aaaacuauauuuuaugccacgugu.    72      0    S03
    .........................................................................................aaaacuauauuuuaugccacguCu.    1       1    S03
```

Star Mature

aagccauuuuuuuuuuuaagugaaaaauaguaaucuuacauauguuuaauguaguuuuuucauuuuuuaaugcuguauguuuacaauguaaaacuauauuuuaugccacguguc			
....aaacuauauuuuaugccacgu...	1	0	S03
....aaacuauauuuuaugccacgugC.	1	1	S03
....aaacuauaCuuuaugccacgugu.	1	1	S03
....aaacuauauuuuaugccacgugu.	4	0	S03
....aaacuauauuuuaugccacguguc	1	0	S03
....aaacuauauuCuaugccacguguc	1	1	S03
....aacuauauuuuaugccacgug..	1	0	S03
....aacuauauuuuaugccacgugG.	3	1	S03
....Nacuauauuuuaugccacgugu.	1	1	S03
....aacuauauuuuaugccacgugC.	7	1	S03
....aacuauauuuuaugccacgugA.	1	1	S03
....aacuauauuuuaugccacgugu.	29	0	S03
....aacuauauuuuaugccacguguc	2	0	S03
....acuauauuuuaugccacgug..	1	0	S03
....acuauauuuuaugccacgugG.	1	1	S03
....acuauauuuuaugccacgugu.	10	0	S03
....acuauauuuuaugccacguguA	1	1	S03
....acuauauuuuaugccacguguc	3	0	S03
....auauuuuaugccacgugu.	1	0	S03
...............................aaucuuacauauguuuaaug...	1	0	S04
.....................................acauauguuuaauguaguuuuu...	1	0	S04
......................................cauauguuuaauguaguuuuCu..	1	1	S04
....uauguuuaauguaguuuuuucU..	1	1	S04
....auguuuUauguaguuuuuucauu..	1	1	S04
....auguCuaauguaguuuuuucauuu...	1	1	S04
....cauuuuuuaaugcuguauguuu................................	1	0	S04
....auuuuuuaaugcuguauguuuac..............................	1	0	S04
....uuuuuCaugcuguauguuuaca.............................	1	1	S04
....uuuuaaugcuguauguuuacaC............................	1	1	S04
....uaaaacuauauuuuaugccacguA..	1	1	S04
....uaaaacuauauuuuaugccacgug..	1	0	S04
....aaaacuauauuuuaugccacgug..	2	0	S04
....aaaacuauauuuuaugccacgugC.	8	1	S04
....aaaacuauauuuuaugccacgugG.	6	1	S04
....aaaacuauauuuuaCgccacgugu.	2	1	S04
....aaaacuauauuuuaugccacgCgu.	1	1	S04
....aaaacuauauuuuaugccacgugu.	73	0	S04
....aaaGcuauauuuuaugccacgugu.	1	1	S04
....aaaacuauauuuuaugccacgugA.	5	1	S04
....aaacuauauuuuaugccacgugu.	2	0	S04
....aaacuauauuuuaugccacguguc	2	0	S04
....aacuauauuuuaugccacgu...	1	0	S04
....aacuauauuuuGugccacgugu.	1	1	S04
....aacuauauuuuaugccacgugA.	2	1	S04
....aacuauauuuuaugccacgugG.	2	1	S04
....aacuauauuuuaugccacgugu.	12	0	S04
....aacuauauuuuaugccacgugC.	1	1	S04
....aacuauauuuuaugccacguguc	2	0	S04
....acuauauuuuaugccacgugA.	2	1	S04
....acuauauuuuaugccacgugu.	6	0	S04
....acuauauuuuaugccacguguA	1	1	S04
....acuauauuuuaugccacguguc	1	0	S04
....cuauauuuuaugccacgugu.	2	0	S04

Provisional ID	:	chrA10_961933
Score total	:	0.1
Score for star read(s)	:	-1.3
Score for read counts	:	0
Score for mfe	:	1.4
Score for randfold	:	0
Score for cons. seed	:	
Total read count	:	798
Mature read count	:	798
Loop read count	:	0
Star read count	:	0

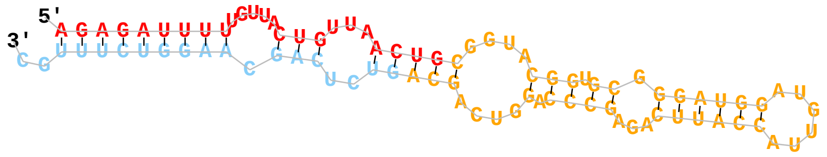

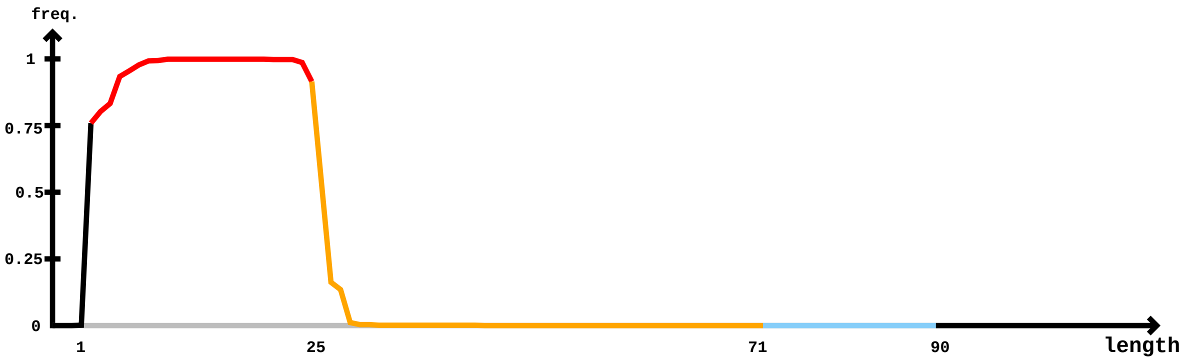

Mature **Star**

```
5'- gcagagauuuuuguuacuguuaacugcgguacggugcgggauggauguuaccauucagagcccaggucagcagucucagcaaggucuuugcuuuccaaggcauugccaauuuu   -3'    exp
    ((((((((((.....(((...(((((....(((.((.((((((......))))))...)))).)....)))))..))).)))))))))).......(((...)))......    reads       mm          sample
    .Gagagauuuuuguuacuguuaacu.......................................................................................    1           1           S03
    ..agagauuuuuguuacugu............................................................................................    1           0           S03
    ..agagauuuuuguuacuguuaac........................................................................................    5           0           S03
    ..agagauuuuuguuacuguuaacu.......................................................................................    21          0           S03
    ..agagauuuuuguuacuguuaacug......................................................................................    173         0           S03
    ..agagauGuuuguuacuguuaacug......................................................................................    1           1           S03
    ..Ggagauuuuuguuacuguuaacug......................................................................................    2           1           S03
    ..agagauuuAuguuacuguuaacug......................................................................................    1           1           S03
    ..agagauuuuuguuacugCuaacug......................................................................................    1           1           S03
    ..agagauuuuuguuacuguuaacuC......................................................................................    1           1           S03
    ..agagauuuuuUuuacuguuaacug......................................................................................    1           1           S03
    ..agagauCuuuguuacuguuaacug......................................................................................    5           1           S03
    ..agagauuuuGguuacuguuaacug......................................................................................    1           1           S03
    ..agagauuuuuguuacuguuaacugc.....................................................................................    1           0           S03
    ...gagauuuuuguuacuguuaacu.......................................................................................    5           0           S03
    ...gagauuuuuguuacuguuaacug......................................................................................    2           0           S03
    ...gagauuuuuguuacuguuaacugc.....................................................................................    6           0           S03
    ....agauuuuuguuacuguuaacuC......................................................................................    1           1           S03
    ....agauGuuuguuacuguuaacug......................................................................................    1           1           S03
    ....agauuuuuguuacuguuaacugc.....................................................................................    1           0           S03
    ....agauuuuuguuacuguuaacugcg....................................................................................    6           0           S03
    .....gauuuuuguuacuguuaacug......................................................................................    2           0           S03
    .....gauuuuuguuacuguuaacugc.....................................................................................    1           0           S03
    .....gauuuuuguuacuguuaacugcgg...................................................................................    15          0           S03
    ......auuuuuguuacuguuaacugc.....................................................................................    1           0           S03
    ......auuuuuguuacuguuaacugcgg...................................................................................    1           0           S03
    ......auuuuuguuacuguuaacugcggu..................................................................................    1           0           S03
    .......uuuuuguuacuguuaacugc.....................................................................................    1           0           S03
    .......uuuuuguuacuguuaacugcg....................................................................................    2           0           S03
    .......uuuuGguuacuguuaacugcgg...................................................................................    1           1           S03
    .......uuuuuguuacuguuaacugcgg...................................................................................    1           0           S03
    ........Guuuguuacuguuaacugcgg...................................................................................    1           1           S03
    ........uuuuguuacuguuaacugcgg...................................................................................    2           0           S03
    ........uuuuguuacuguuaacugcggu..................................................................................    1           0           S03
```

Mature   Star

```
gcagagauuuuuguuacuguuaacugcgguacggugcgggauggauguuaccauucagagcccaggucagcagucucagcaaggucuuugcuuuccaaggcauugccaauuuu
.........uuuguuacuguuaacugcgg....................................................................................    1      0      S03
....................uaacugcgguacggugcgggaug......................................................................    1      0      S03

..agagauuuuuguuacuguu............................................................................................    1      0      S04
..Ggagauuuuuguuacuguuaac.........................................................................................    1      1      S04
..agagauuuuuguuacuguuaac.........................................................................................    3      0      S04
..agagauuuuuguuacuguuaacu........................................................................................    21     0      S04
..Cgagauuuuuguuacuguuaacug.......................................................................................    1      1      S04
..Ggagauuuuuguuacuguuaacug.......................................................................................    8      1      S04
..agagauuuuuguuacuguuaacug.......................................................................................    341    0      S04
..agagauuuuCguuacuguuaacug.......................................................................................    2      1      S04
..Ngagauuuuuguuacuguuaacug.......................................................................................    2      1      S04
..agagauuuuuguuNcuguuaacug.......................................................................................    1      1      S04
..agagauuuuuguuacuguCaacug.......................................................................................    1      1      S04
..agagauuCuuguuacuguuaacug.......................................................................................    2      1      S04
..agGgauuuuuguuacuguuaacug.......................................................................................    1      1      S04
..agagauuuuuguuacugCuaacug.......................................................................................    2      1      S04
..agagauuuuuguuacuguuaacuC.......................................................................................    3      1      S04
..agagauuuuuguuacuguuaacugc......................................................................................    3      0      S04
...gagauuuuuguuacuguuaacu........................................................................................    7      0      S04
...gagauuuuuguuacuguuaacug.......................................................................................    4      0      S04
...gagauuuuuguuacuguuaacugc......................................................................................    11     0      S04
....agauuuuuguuacuguuaacu........................................................................................    1      0      S04
....agauuuuuguuacuguuaacug.......................................................................................    2      0      S04
....agauuuuuguuacuguuaacugc......................................................................................    2      0      S04
....agauuuuuguuacuguuaacugcg.....................................................................................    10     0      S04
.....gauuuuuguuacuguuaacug.......................................................................................    6      0      S04
.....gauuuuuguuacuguuaacugc......................................................................................    2      0      S04
.....gauuuuuguuacuguuaacugcgg....................................................................................    51     0      S04
.....gauuuuuguuacuguuaacugcgC....................................................................................    1      1      S04
.....gauuuuuguuacugCuaacugcgg....................................................................................    1      1      S04
.....gauGuuuguuacuguuaacugcgg....................................................................................    2      1      S04
......auuuuuguuacuguuaacug.......................................................................................    2      0      S04
......auuuuuguuacuguuaacugc......................................................................................    1      0      S04
......auuuuuguuacuguuaacugcg.....................................................................................    2      0      S04
......auuuuuguuacuguuaacugcgg....................................................................................    6      0      S04
......auuuuuguuacuguuaacugcggu...................................................................................    3      0      S04
.......uuuuuguuacuguuaacu........................................................................................    1      0      S04
.......uuuuuguuacuguuaacugc......................................................................................    3      0      S04
.......uuuuuguuacuguuaacugcg.....................................................................................    2      0      S04
.......uuuuuguuacuguuaacugcgg....................................................................................    7      0      S04
........uuuuguuacuguuaacugcgg....................................................................................    5      0      S04
........uuuuguuacuguuaacugcggu...................................................................................    1      0      S04
........uuuuguuaAuguuaacugcgguac.................................................................................    1      1      S04
........uuuuguuacuguuaacugcgUuac.................................................................................    1      1      S04
..........uuguuacuguuaacugcgC....................................................................................    1      1      S04
..........uuguuacuguuaacugcgg....................................................................................    3      0      S04
```

Provisional ID	:	chrAnn_random_2964015
Score total	:	71
Score for star read(s)	:	3.9
Score for read counts	:	68.4
Score for mfe	:	-1.2
Score for randfold	:	0
Score for cons. seed	:	
Total read count	:	146
Mature read count	:	144
Loop read count	:	0
Star read count	:	2

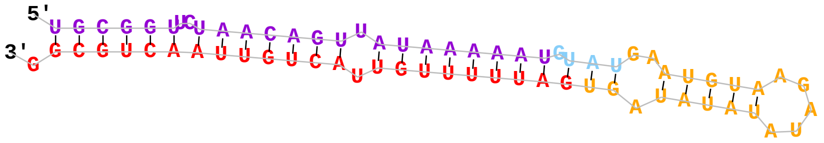

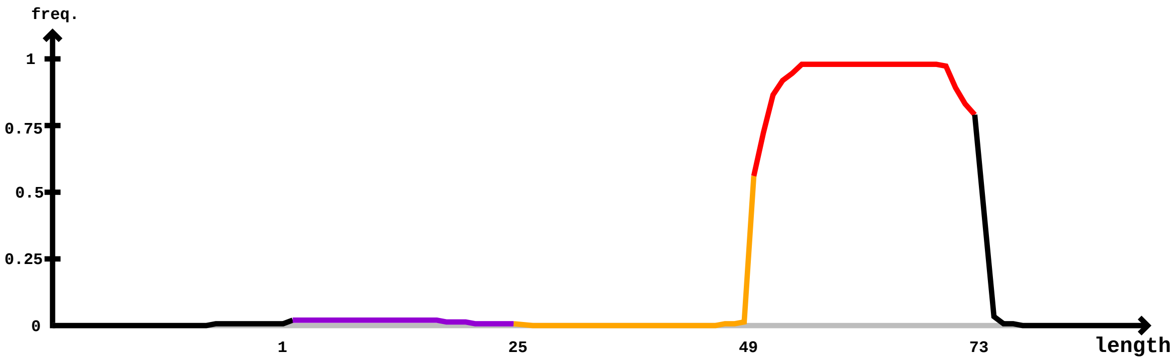

Star Mature

```
5'- gcggaaaaacgguuuaauuguggugcgguucuaacaguuauaaaaauguaugaauguaagauauauauagugauuuuuguuacuguuaacugcgggacgaaacggaaaacau -3'  obs
    gcggaaaaacgguuuaauuguggugcgguucuaacaguuauaaaaauguaugaauguaagauauauauagugauuuuuguuacuguuaacugcgggacgaaacggaaaacau       exp
    .........(.(((...((((..((((((..(((((((.((((((((.(((..(((((.....))))).))))))))))).)))))))))))))..))))))).).......  reads  mm  sample
    .......................ugcgguucuaacaguuaua......................................................................  1  0  S03
    .......................................................................gauuuuuguuacuguuaacug....................  2  0  S03
    .......................................................................gauuuuuguuacuguuaacugc...................  1  0  S03
    .......................................................................gauuuuuguuacuguuaacugcgg.................  15  0  S03
    ........................................................................auuuuuguuacuguuaacugc...................  1  0  S03
    ........................................................................auuuuuguuacuguuaacugcgg.................  1  0  S03
    ........................................................................auuuuuguuacuguuaacugcggg................  3  0  S03
    .........................................................................uuuuuguuacuguuaacugc...................  1  0  S03
    .........................................................................uuuuuguuacuguuaacugcg..................  2  0  S03
    .........................................................................uuuuuguuacuguuaacugcgg.................  1  0  S03
    .........................................................................uuuuGguuacuguuaacugcgg.................  1  1  S03
    ..........................................................................Guuuguuacuguuaacugcgg.................  1  1  S03
    ..........................................................................uuuuguuacuguuaacugcgg.................  2  0  S03
    ...........................................................................uuuguuacuguuaacugcgg.................  1  0  S03
    ...........................................................................uuuguuacuguuaacugcggg................  1  0  S03
    ...........................................................................uuuguuacuAuuaacugcgggacg.............  1  1  S03

    ...............aauugAggugcgguucuaacaguu.........................................................................  1  1  S04
    .......................ugcgguucuaacaguuauaaaaau.................................................................  1  0  S04
    ....................................................................agGgauuuuuguuacuguuaacug....................  1  1  S04
    ......................................................................ugauuuuuguuacuguuaacug....................  1  0  S04
    .......................................................................gauuuuuguuacuguuaacug....................  6  0  S04
    .......................................................................gauuuuuguuacuguuaacugc...................  2  0  S04
    .......................................................................gauGuuuguuacuguuaacugcgg.................  2  1  S04
    .......................................................................gauuuuuguuacuguuaacugcgC................  1  1  S04
    .......................................................................gauuuuuguuacugCuaacugcgg.................  1  1  S04
    .......................................................................gauuuuuguuacuguuaacugcgg.................  51  0  S04
    ........................................................................auuuuuguuacuguuaacug....................  2  0  S04
    ........................................................................auuuuuguuacuguuaacugc...................  1  0  S04
    ........................................................................auuuuuguuacuguuaacugcg..................  2  0  S04
    ........................................................................auuuuuguuacuguuaacugcgg.................  6  0  S04
    ........................................................................auuuuuguuacuguuaacugcggg................  5  0  S04
    ........................................................................auuuuCguuacuguuaacugcggg................  1  1  S04
```

```
                       Star                                               Mature
gcggaaaaacgguuuaauuguggugcgguucuaacaguuauaaaaauguaugaauguaagauauauauagugauuuuuguuacuguuaacugcgggacgaaacggaaaacau
........................................................................Uuuuuuguuacuguuaacugcggg................	1	1	S04
........................................................................aAuuuuguuacuguuaacugcggg................	1	1	S04
.........................................................................uuuuuguuacuguuaacu.....................	1	0	S04
.........................................................................uuuuuguuacuguuaacugc...................	3	0	S04
.........................................................................uuuuuguuacuguuaacugcg..................	2	0	S04
.........................................................................uuuuuguuacuguuaacugcgg.................	7	0	S04
.........................................................................uuuuuguuacuguuaacugcggg................	1	0	S04
.........................................................................uuuuuguuacuguuaacugcggga...............	1	0	S04
.........................................................................uuuuuguuacuguuaacugcgUga...............	1	1	S04
..........................................................................uuuuguuacuguuaacugcgg.................	5	0	S04
...........................................................................uuuguuacuguuaacugcggga...............	1	0	S04
............................................................................uuguuacuguuaacugcgg.................	3	0	S04
............................................................................uuguuacuguuaacugcgC.................	1	1	S04
............................................................................uuguuacuguuaacugcggga...............	1	0	S04
```

Provisional ID	:	chrAnn_random_2965608
Score total	:	34.7
Score for star read(s)	:	3.9
Score for read counts	:	28.7
Score for mfe	:	2.1
Score for randfold	:	0
Score for cons. seed	:	
Total read count	:	68
Mature read count	:	66
Loop read count	:	0
Star read count	:	2

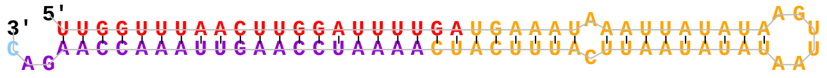

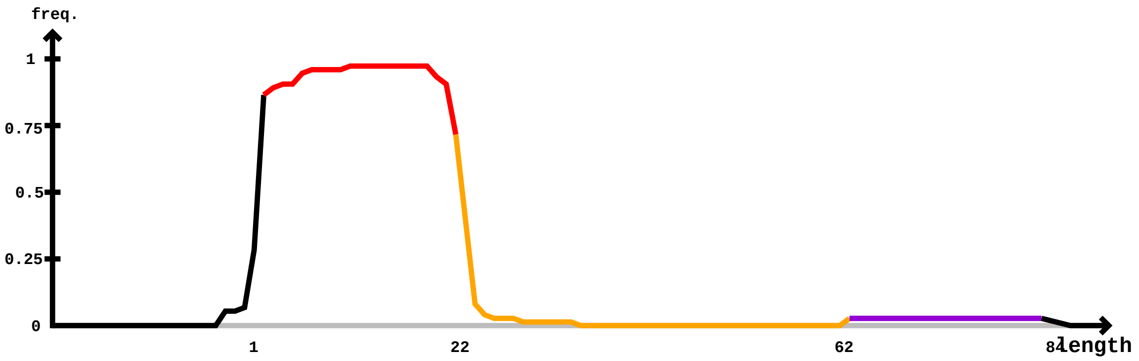

Mature Star

```
5'- ucugguuuggugaauuuuguuugguuuaacuuggauuuugaugaaauaaauuauauaaguuaauauauaauucauuucaucaaaauccaaguuaaaccaagacaacau  -3'  obs
    ucugguuuggugaauuuuguuugguuuaacuuggauuuugaugaaauaaauuauauaaguuaauauauaauucauuucaucaaaauccaaguuaaaccaagacaacau       exp
    ................(((((((((((((((((((((((((((((((.(((((((((......))))))))).)))))))))))))))))))))))))).))))))...  reads  mm  sample
    ...................uuugguuuaacuuggauuuuga...................................................................  1      0   S03
    ............................cuuggauuuugaugaaauaaauua.......................................................  1      0   S03

    ................uuguuugguuuaacuuggauuuuU....................................................................  1      1   S04
    ................uuguuugguuuaacuuggauuuug....................................................................  3      0   S04
    ..................guuugguuuaacuuggauuuugU...................................................................  1      1   S04
    ...................uuugguuuaacuuggauuu......................................................................  1      0   S04
    ...................uuugguuuaacuuggauuuC.....................................................................  1      1   S04
    ...................uuugguuuaacuuggauuuu.....................................................................  1      0   S04
    ...................uuugguuuaacuuggGuuuug....................................................................  1      1   S04
    ...................uuugguuuaacuuggauuuug....................................................................  8      0   S04
    ...................uuugguuuaacuuggauuuugU...................................................................  1      1   S04
    ...................uuugguuuaacuuggauuuuga...................................................................  2      0   S04
    ....................uugguuuaacuuggauuu......................................................................  2      0   S04
    ....................uGgguuuaacuuggauuuug....................................................................  1      1   S04
    ....................uugguuuaacuuggauuuugG...................................................................  2      1   S04
    ....................uugguuuaacuuggauuuuga...................................................................  32     0   S04
    ....................uugguuuUacuuggauuuuga...................................................................  1      1   S04
    ....................uugguuuaacuuggauuuugC...................................................................  1      1   S04
    ....................uuggAuuaacuuggauuuuga...................................................................  1      1   S04
    ....................uCgguuuaacuuggauuuuga...................................................................  1      1   S04
    ....................uuggCuuaacuuggauuuuga...................................................................  1      1   S04
    ....................uugguuuaacuuggauuuugau..................................................................  1      0   S04
    .....................ugguuuaacuuggauuuugau..................................................................  1      0   S04
    .....................ugguuuaacuuggauuuugaC..................................................................  1      1   S04
    ......................gguuuaacuuggauuuugaug.................................................................  1      0   S04
    ........................uuCaacuuggauuuugaug.................................................................  1      1   S04
    ........................uuuaacuuggauuuugauA.................................................................  1      1   S04
    ........................uuuaacuuggauuuugaugU................................................................  1      1   S04
    .........................uuaacuuggauuuugaugaaau.............................................................  1      0   S04
    .................................................................................aaaauccaaguuaaaccaaga......  2      0   S04
```

Provisional ID	:	chrAnn_random_2969865
Score total	:	3.8
Score for star read(s)	:	3.9
Score for read counts	:	1.1
Score for mfe	:	-1.1
Score for randfold	:	0
Score for cons. seed	:	
Total read count	:	14
Mature read count	:	4
Loop read count	:	9
Star read count	:	1

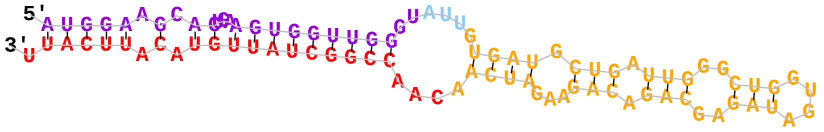

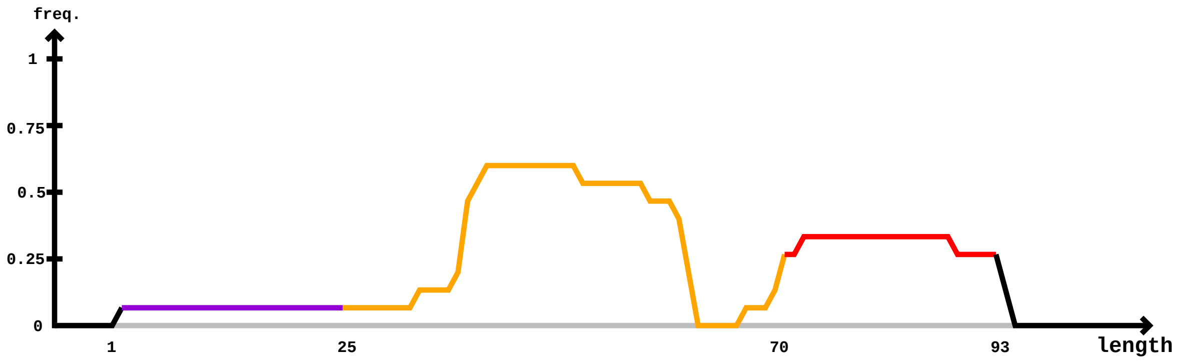

Star **Mature**

```
5'- uuguaauggaagcacugaagugguugggguauugugaugcugauugggcuggugauagagcagacagaagaucaacaaccggcuauuguacauucauuggaggcaacuugguu  -3'   obs
    uuguaauggaagcacugaagugguuggguauugugaugcugauugggcuggugauagagcagacagaagaucaacaaccggcuauuguacauucauuggaggcaacuugguu        exp
    ...(((((((.(.((...(((((((((......((((.(((.(((..(((....)))..))).)))...))))....))))))))))).).)))))))(((....)))....   reads   mm   sample
    ..............................uugugaugcugauugggcugguA...........................................................   1       1    S03
    ....................................ugcAgauugggcuggugauagagc....................................................   1       1    S03
    .........................................auugggcuggugauagaAcagac................................................   1       1    S03
    .........................................auugggcuggugauagagcagacG...............................................   1       1    S03
    .........................................auugggcuggugauagagcagaca...............................................   1       0    S03
    ..........................................uugggcuggugauagagcUga.................................................   1       1    S03
    .........................................................................acaaccggcuauugCacauucauu...............   1       1    S03
    ..........................................................................caaccggcuauugCacauucauu...............   2       1    S03

    .....auggaagcacugaagugguugAgu...................................................................................   1       1    S04
    ........................................gauugggcuggugauagagUagac................................................   1       1    S04
    .........................................auugggcuggugauagagcagaca...............................................   1       0    S04
    ...........................................ugggcuggugauagagcagac................................................   1       0    S04
    ......................................................................ucaacaaccggcuauuguacau....................   1       0    S04
    ............................................................................accggcuauuguacauucauG...............   1       1    S04
```

Provisional ID : chrAnn_random_2981340
Score total : 0.1
Score for star read(s) : -1.3
Score for read counts : 0
Score for mfe : 1.4
Score for randfold : 0
Score for cons. seed :
Total read count : 363
Mature read count : 360
Loop read count : 3
Star read count : 0

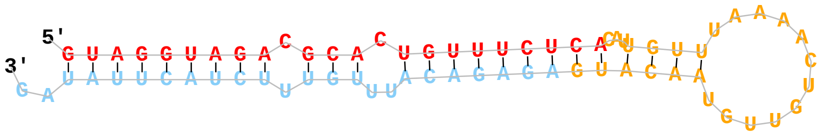

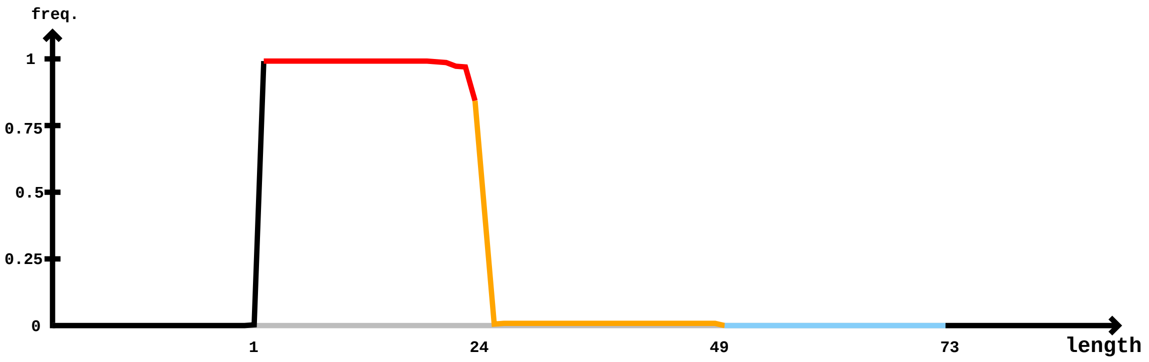

Mature Star

```
5'- ucgcuccuuuguguuuccugguagguagacgcacuguuucucacauuguuuaaaacuguuguaacaugagagacauuuguuucuacuuauaggggagcagauucagauc   -3'    exp
    .........(.((((((((.(((((((((.(((.((((((((((...((((............)))))))))))))..))).))))))))).))))))))).)........    reads    mm    sample
    ...................Cguagguagacgcacuguuucuca..................................................................    1    1    S03
    ....................guagguagacgcacuguu.......................................................................    1    0    S03
    ....................guagguagacgcacuguuu......................................................................    1    0    S03
    ....................guagguagacgcacuguuuc.....................................................................    1    0    S03
    ....................guagguagacgcacuguuuU.....................................................................    2    1    S03
    ....................guagguagacgcacuguuucu....................................................................    1    0    S03
    ....................guagguUgacgcacuguuucuc...................................................................    1    1    S03
    ....................guagguagacgcacuguuucuc...................................................................    13   0    S03
    ....................Uuagguagacgcacuguuucuc...................................................................    1    1    S03
    ....................guagguagacgcacuguuucuA...................................................................    2    1    S03
    ....................guagguagacgcacuguuucuca..................................................................    91   0    S03
    ....................guagguagacgcacCguuucuca..................................................................    2    1    S03
    ....................guagguagacgcacuguuucucG..................................................................    5    1    S03
    ....................Uuagguagacgcacuguuucuca..................................................................    2    1    S03
    ....................Nuagguagacgcacuguuucuca..................................................................    1    1    S03
    ....................guagguagacgcacuguuucCca..................................................................    1    1    S03
    ....................guagguagGcgcacuguuucuca..................................................................    1    1    S03

    ....................guagguagacgcacuguuuc.....................................................................    2    0    S04
    ....................guagguGgacgcacuguuucuc...................................................................    1    1    S04
    ....................guGgguagacgcacuguuucuc...................................................................    1    1    S04
    ....................guagguagacgcacuguuucuA...................................................................    1    1    S04
    ....................guagguagacgcacuguuucuc...................................................................    24   0    S04
    ....................guagguagacgcacuguuucuU...................................................................    2    1    S04
    ....................guagguagacgcacuguuucucU..................................................................    3    1    S04
    ....................guaggAagacgcacuguuucuca..................................................................    1    1    S04
    ....................guagguagacgcUcuguuucuca..................................................................    1    1    S04
    ....................guagguagacgcacuguuucAca..................................................................    2    1    S04
    ....................Uuagguagacgcacuguuucuca..................................................................    3    1    S04
    ....................guagguagacgcacuguuucuca..................................................................    167  0    S04
    ....................guaggCagacgcacuguuucuca..................................................................    2    1    S04
    ....................guagguagGcgcacuguuucuca..................................................................    1    1    S04
    ....................gCagguagacgcacuguuucuca..................................................................    2    1    S04
    ....................guagguagacgcacuguuucucG..................................................................    15   1    S04
```

```
                    Mature                                              Star
ucgcuccuuuguguuuccugguagguagacgcacuguuucucacauuguuuaaaacuguuguaacaugagagacauuuguuucuacuuauaggggagcagauucagauc
....................guagguagacgcacuguuucCca..................................................................    1    1    S04
....................gGagguagacgcacuguuucuca..................................................................    2    1    S04
....................guagguagacgcacuguuCcuca..................................................................    2    1    S04
...........................................cauuguuuaaaacuguuguaacaug.........................................    2    0    S04
.............................................uuguuuaaaacuguuguaacaug.........................................    1    0    S04
```

Provisional ID	:	chrAnn_random_2985149
Score total	:	0.2
Score for star read(s)	:	3.9
Score for read counts	:	-4.9
Score for mfe	:	1.3
Score for randfold	:	0
Score for cons. seed	:	
Total read count	:	2
Mature read count	:	1
Loop read count	:	0
Star read count	:	1

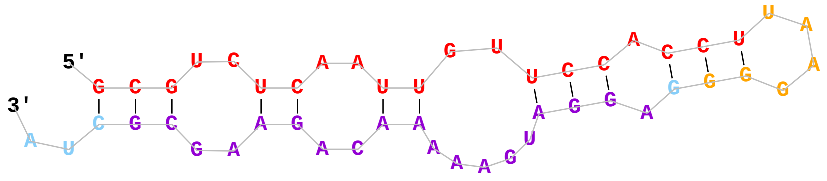

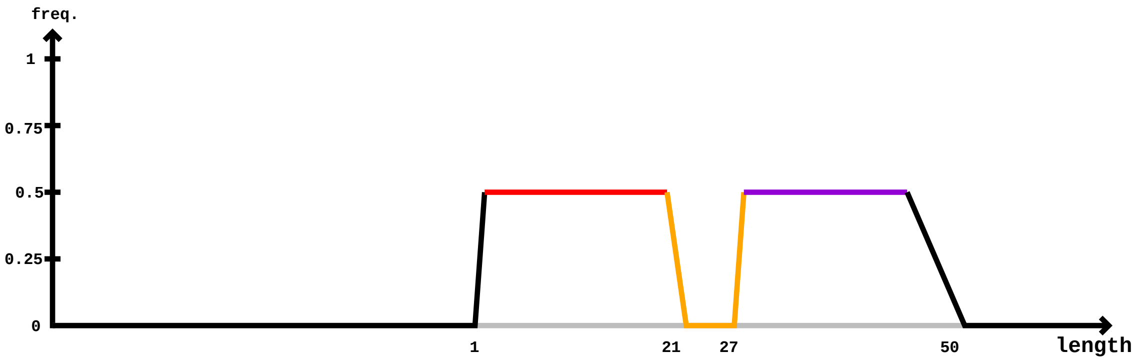

Mature Star

```
5'- ccuuucuuuuugagcucagagcugaguuuuguuggugcaggug gcgucucaauuguuccaccu uaagggg aggaugaaaaacagaagcg cuacuaaggcugaaaggga   -3'   obs
    ccuuucuuuuugagcucagagcugaguuuuguuggugcaggugggcgucucaauuguuccaccuuaaggggaggaugaaaaacagaagcgcuacuaaggcugaaaggga          exp
    ((((((.....(((((((....))))))).......((.(((((((..((..((..(((.(((....))).))).....))..))..)))))))...)).)))))).. reads   mm   sample
    ...........................................gcgucucaauuguuccaccu.............................................   1     0    S04
    ......................................................................aggaugaaaaacagaagcA...................   1     1    S04
```

Provisional ID	:	chrAnn_random_2999963
Score total	:	13.4
Score for star read(s)	:	3.9
Score for read counts	:	7.3
Score for mfe	:	2.3
Score for randfold	:	0
Score for cons. seed	:	
Total read count	:	26
Mature read count	:	11
Loop read count	:	0
Star read count	:	15

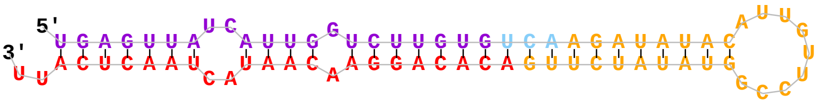

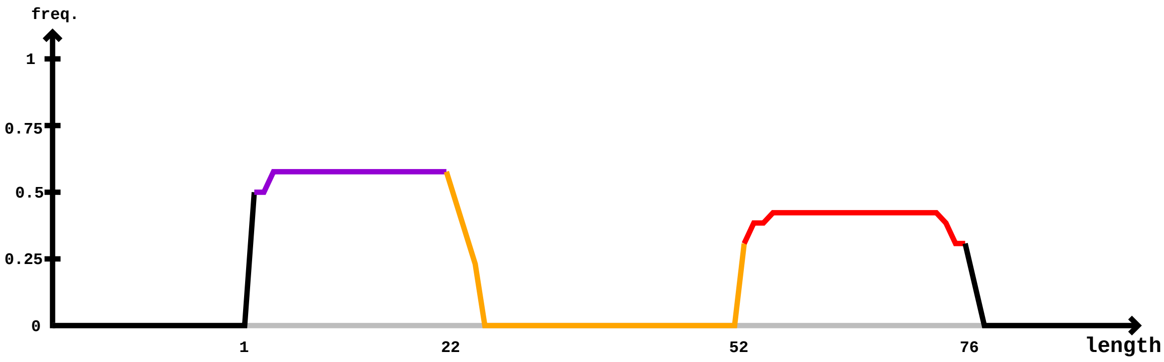

Star **Mature**

```
5'- auauauugguuuugauaagugaguuaucauuggucuugugucaagauauacauuguuccgguauaucuugacacaggaacaauacuaacucauuuaucaauaaaaauguau  -3'   obs
    auauauugguuuugauaagugaguuaucauuggucuuguguca agauauacauuguuccgguauaucuugacacaggaacaauacuaacucauuuaucaauaaaaauguau        exp
    ((((((....((((((((((((((..((((.(((((((((((((((((.........)))))))))))))))))).)))).)))))))))))))))....)))))))  reads   mm   sample
    ...................ugaguuaucauuggucuuguguca....................................................................  1   0   S03
    .....................aguuaucauuggucuuguguA.....................................................................  1   1   S03
    ......................................................................acacaggaacaauacuaacuc....................  1   0   S03

    ...................ugaguuaucauuggucuugug.......................................................................  5   0   S04
    ...................ugaguuaucauuggucAugug.......................................................................  1   1   S04
    ...................ugaguuaucauuggucuuguguU.....................................................................  1   1   S04
    ...................ugaguuaucauuggucuuguguc.....................................................................  1   0   S04
    ...................ugaguuaucauuggucuuguguca....................................................................  4   0   S04
    .....................aguuaucauuggucuuguguca....................................................................  1   0   S04
    ......................................................................acacaggaacaauacuaacucauu.................  7   0   S04
    ........................................................................cacaggaacaauacuaUcuca..................  1   1   S04
    ........................................................................cacaggaacaauacuaacuca..................  1   0   S04
    ..........................................................................caggaacaauacuaacucauu................  1   0   S04
```

Provisional ID	:	chrAnn_random_3000011
Score total	:	13.4
Score for star read(s)	:	3.9
Score for read counts	:	7.3
Score for mfe	:	2.3
Score for randfold	:	0
Score for cons. seed	:	
Total read count	:	26
Mature read count	:	11
Loop read count	:	0
Star read count	:	15

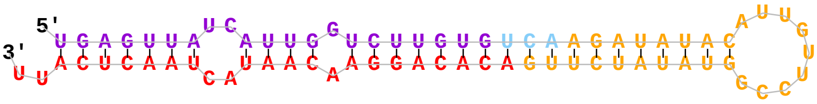

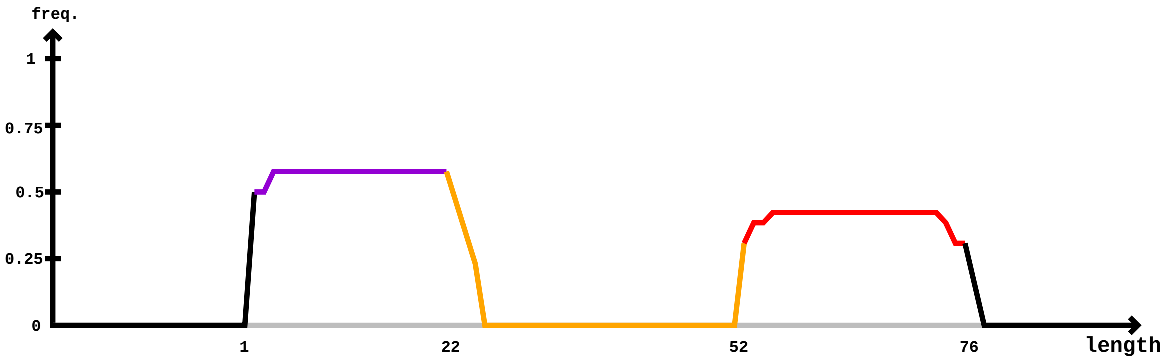


```
                     Star                                     Mature
5'- auauauugguuuugauaagugaguuaucauuggucuugugucaagauauacauuguuccgguauaucuugacacaggaacaauacuaacucauuuaucaauaaaaauguau   -3'   obs
    auauauugguuuugauaagugaguuaucauuggucuuguguca agauauacauuguuccgguauaucuugacacaggaacaauacuaacucauuuaucaauaaaaauguau          exp
    (((((((....(((((((((((((((..((((.((((((((((((((((((.........)))))))))))))))))).)))).)))))))))))))))....)))))))  reads   mm   sample
    ...................ugaguuaucauuggucuuguguca....................................................................  1       0    S03
    .....................aguuaucauuggucuuguguA.....................................................................  1       1    S03
    ......................................................................acacaggaacaauacuaacuc....................  1       0    S03

    ...................ugaguuaucauuggucuugug.......................................................................  5       0    S04
    ...................ugaguuaucauuggucAugug.......................................................................  1       1    S04
    ...................ugaguuaucauuggucuuguguU.....................................................................  1       1    S04
    ...................ugaguuaucauuggucuuguguc.....................................................................  1       0    S04
    ...................ugaguuaucauuggucuuguguca....................................................................  4       0    S04
    .....................aguuaucauuggucuuguguca....................................................................  1       0    S04
    ......................................................................acacaggaacaauacuaacucauu.................  7       0    S04
    .......................................................................cacaggaacaauacuaUcuca...................  1       1    S04
    .......................................................................cacaggaacaauacuaacuca...................  1       0    S04
    .........................................................................caggaacaauacuaacucauu.................  1       0    S04
```

Provisional ID	:	chrAnn_random_3000316
Score total	:	0
Score for star read(s)	:	-1.3
Score for read counts	:	0
Score for mfe	:	1.3
Score for randfold	:	0
Score for cons. seed	:	
Total read count	:	304
Mature read count	:	301
Loop read count	:	0
Star read count	:	3

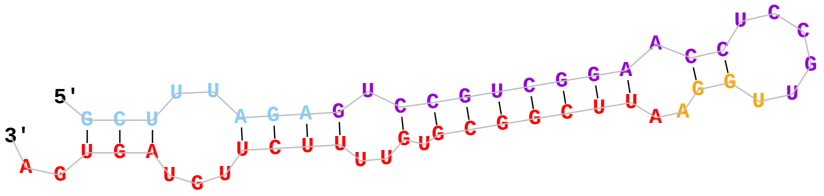

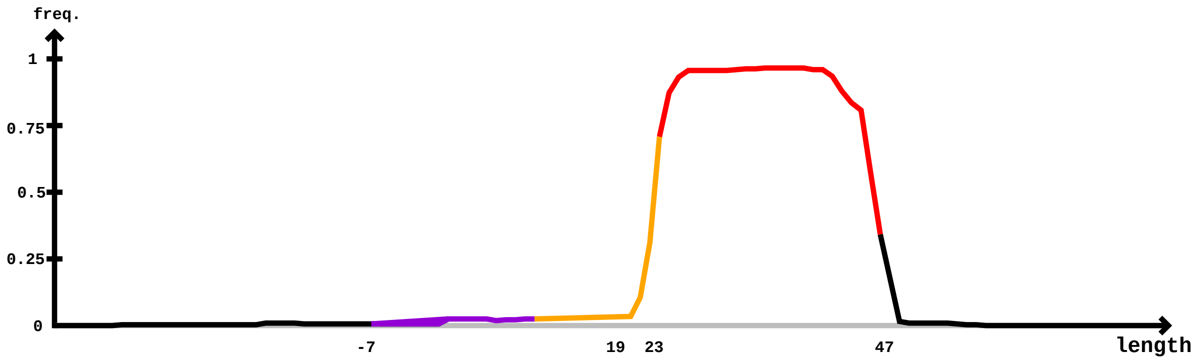

Star **Mature**

5'- uucgucagaaauccgucacaaacguccgauggcuuuagaguccgucggaaccuccguuggaauucggcguguuuucuuguagugauugguuuguggaagguuugcgacuugaga -3'	obs		
uucgucagaaauccgucacaaacguccgauggcuuuagaguccgucggaaccuccguuggaauucggcguguuuucuuguagugauugguuuguggaagguuugcgacuugaga	exp		
...(((..(((((..(((((((...(((((.(((..((((.((((((((.((......))..))))))).)..))))...))).))))))))))))..)))))..)))......	reads	mm	sample
.....cagaaauccgucacaaacA..	1	1	S03
....guccgucggaaccuccguuC...	1	1	S03
....cggaaccuccguuggaauucggcU...	1	1	S03
....ggaauucggcguguuuucuug...................................	1	0	S03
....ggaauucggcguguuuucuugua.................................	1	0	S03
....gaauucggcguguuuucuug...................................	1	0	S03
....gaauucggcguguuuucuGg...................................	1	1	S03
....gaauucggcguguuuucuugua.................................	1	0	S03
....gaauucggcguguuuucuuguagC...............................	2	1	S03
....gaauucggcguguuuucuuguagu...............................	6	0	S03
....aauucggcguguuuucuugu..................................	1	0	S03
....aauucggcguguuuucuuguag................................	2	0	S03
....aauucggcguguuuucuuguagu...............................	6	0	S03
....aauucggcguguCuucuuguagug..............................	1	1	S03
....aauucggcgugCuuucuuguagug..............................	1	1	S03
....aauucggAguguuuucuuguagug..............................	1	1	S03
....aauucggcguguuuucuuguagug..............................	4	0	S03
....auucggcguguuuucuug...................................	1	0	S03
....auucggcguguuuucuugu..................................	2	0	S03
....auucggcguguuuucuugG..................................	1	1	S03
....auucggcguguuuucuuguagu...............................	13	0	S03
....auucggcguguuuucuuguagC...............................	1	1	S03
....auucggcguguCuucuuguagu...............................	1	1	S03
....auucggcguguuuucuuguagug..............................	9	0	S03
....Uuucggcguguuuucuuguaguga.............................	1	1	S03
....auucggcguguuuucuuguaguga.............................	19	0	S03
....uucggcguguuuucuugu..................................	2	0	S03
....uucggcguguuuucuuguag................................	1	0	S03
....uucggcguguuuucuuguagu...............................	1	0	S03
....uucggcguguuuucuuguagC...............................	1	1	S03
....uucggcguguuuucuuguaguU..............................	2	1	S03
....uucggcguguuuucuuguagug..............................	4	0	S03
....uucggcguguuuucuuguaguga.............................	12	0	S03

Star **Mature**

```
uucgucagaaauccgucacaaacguccgauggcuuuagaguccgucggaaccuccguuggaauucggcguguuuucuuguagugauugguuuguggaagguuugcgacuugaga
...............................................................ucggcguguuuucuuguagug..............................  1   0   S03
...............................................................ucggcguguuuucuuguaguga.............................  3   0   S03
................................................................cggcguguuuucuuguagu...............................  1   0   S03
................................................................cggcguguuuucuuguaguga.............................  1   0   S03
......................................................................guuuucuuguagugauugUuuug.....................  1   1   S03

....................aacguccgauggcuuuagaguccg......................................................................  2   0   S04
.......................................guccgucggaaccuccgA.........................................................  2   1   S04
.......................................guccguCgaaccuccgu..........................................................  1   1   S04
.......................................guccgucggaaccuccguu........................................................  1   0   S04
.......................................guccgucggaaccuccguuU.......................................................  1   1   S04
...............................................gGaccuccguuggaauucggcgug...........................................  1   1   S04
..........................................................ggaauucggcguguuuucuug...................................  1   0   S04
..........................................................ggaauucggcguguuuucuugu..................................  1   0   S04
..........................................................ggaauucggcguguuuucuuguG.................................  1   1   S04
..........................................................ggaauucggcguguuuucuugua.................................  2   0   S04
...........................................................gaauucggcguguuuucu.....................................  2   0   S04
...........................................................gaauucggcguguuuucuug...................................  1   0   S04
...........................................................gaauucggcguguuuucuugua.................................  1   0   S04
...........................................................gaauucggcgugGuuucuugua.................................  1   1   S04
...........................................................gaauucggcguguuuucuuguagC...............................  1   1   S04
...........................................................gaauucggcguguuuucuCguagu...............................  1   1   S04
...........................................................gaauucggcguguuuucuuguagu...............................  7   0   S04
............................................................aauucggcguguuuucuug...................................  1   0   S04
............................................................aauucggcguguuuucuugu..................................  5   0   S04
............................................................aauucggcguguuuucuugC..................................  1   1   S04
............................................................aauucggcguguuuucuuUu..................................  1   1   S04
............................................................aauucggcguguuuucuugua.................................  2   0   S04
............................................................aauucggcguguuuucuuguG.................................  1   1   S04
............................................................aauucggcguguuuucuuguag................................  2   0   S04
............................................................aUuucggcguguuuucuuguagu...............................  1   1   S04
............................................................aauucggcguguuuucuuguagC...............................  1   1   S04
............................................................aauucggcguguuuucuuguagu...............................  9   0   S04
............................................................aauucggcguguuuucuuguagCg..............................  1   1   S04
............................................................aauucggcguguuuucuuguagug.............................. 25   0   S04
.............................................................auucggcguguuuucuug...................................  1   0   S04
.............................................................auucggcguguuuucuugu..................................  4   0   S04
.............................................................auucggcguguuuucuugua.................................  2   0   S04
.............................................................auucggcguguuuucuuguUg................................  1   1   S04
.............................................................auucggcguguuuucuuguaA................................  1   1   S04
.............................................................auucggcguguuuucuuguag................................  2   0   S04
.............................................................auucggcguguuuucuuguagC...............................  3   1   S04
.............................................................auucggcguguuuucuuguagu............................... 10   0   S04
.............................................................auucggcguguuuucuugAagu...............................  1   1   S04
.............................................................auucggcguguuuucuuguGgu...............................  1   1   S04
.............................................................auucggcguguuuucuuguagug.............................. 12   0   S04
.............................................................auucggcguguuuucuuguaguC..............................  2   1   S04
.............................................................auucggcguguuuucuuguaguU..............................  1   1   S04
.............................................................auucggcguguuuucuCguaguga.............................  1   1   S04
.............................................................auucggcguguuuucuuguaguga............................. 36   0   S04
.............................................................auucggcguguuuucuuguagCga.............................  1   1   S04
.............................................................Uuucggcguguuuucuuguaguga.............................  1   1   S04
..............................................................uucggcguguuuucuugua.................................  1   0   S04
..............................................................uucggcguguuuucuuguU.................................  1   1   S04
..............................................................uucggcguguuuucuuguagu...............................  7   0   S04
..............................................................uucggcguguuuucuuguagug..............................  4   0   S04
..............................................................uucggcguguuuucuuguaguU..............................  2   1   S04
..............................................................uucggcguguuuucuuguaguga............................. 14   0   S04
..............................................................uucggcguguuuucuuguagugau............................  1   0   S04
...............................................................ucggcguguuuucuuguagug..............................  3   0   S04
...............................................................ucggcguguuuucuuguaguUa.............................  1   1   S04
...............................................................ucggcguguuuucuuguaguga............................. 11   0   S04
................................................................cggcguguuuucuuguagu...............................  2   0   S04
................................................................cggcguguuuucuuguagug..............................  1   0   S04
................................................................cggcguguuuucuuguaguga.............................  1   0   S04
................................................................cggcguguuuucuuguagugau............................  2   0   S04
.....................................................................uguuuucuuguagugauu...........................  2   0   S04
.......................................................................uuuucuuguagugauAgguuu......................  1   1   S04
.........................................................................uucuuguagugauugguuugug...................  1   0   S04
```

Provisional ID	:	chrAnn_random_3000733
Score total	:	490.4
Score for star read(s)	:	3.9
Score for read counts	:	484.5
Score for mfe	:	2.1
Score for randfold	:	0
Score for cons. seed	:	
Total read count	:	962
Mature read count	:	338
Loop read count	:	598
Star read count	:	26

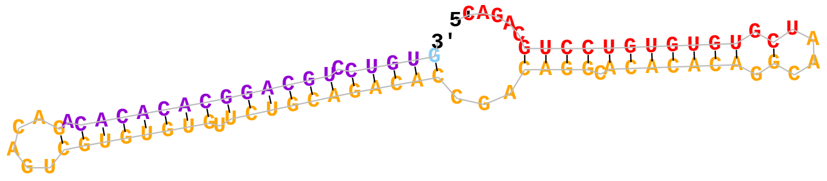

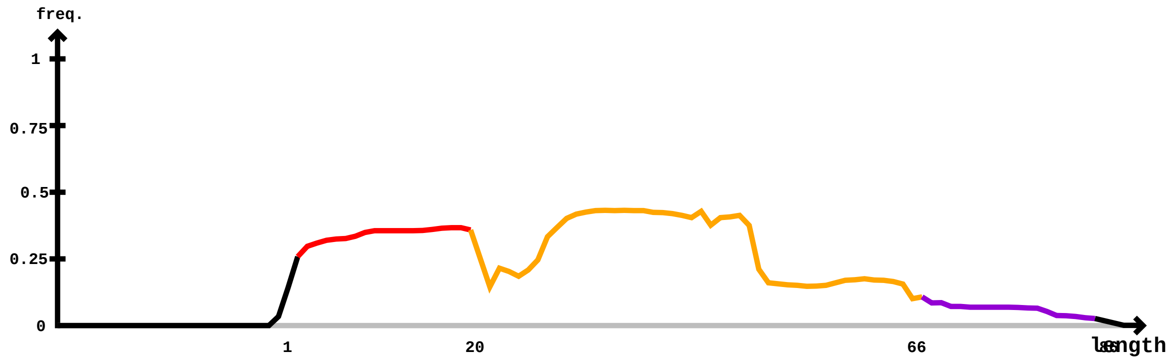

Mature Star

```
5'- nnnnnnnnnnnnnnnnnnnnncacagacguccugugugugcuaacggacacacacggacagccacagacgucuugugugugcugacagacacacacggacguccugugugu   -3'   obs
    nnnnnnnnnnnnnnnnnnnnncacagacguccugugugugcuaacggacacacacggacagccacagacgucuugugugugcugacagacacacacggacguccugugugu         exp
    .....................(((((((((((.(((((((((..(((.(((((((((((..........))).)))))))))))..)).))))))))))))).)))))...   reads   mm   sample
    .....................cacagacguccugugugugcu......................................................................   2   0   S03
    .....................cacagacguccugugugugcuC.....................................................................   1   1   S03
    ......................acagacguccugugugugc.......................................................................   1   0   S03
    ......................acagacguccugugugAgcu......................................................................   2   1   S03
    ......................acagacguccugugugugcu......................................................................   9   0   S03
    ......................acagacguccugugugugcG......................................................................   1   1   S03
    ......................Ucagacguccugugugugcu......................................................................   1   1   S03
    ......................acagacguAcugugugugcua.....................................................................   1   1   S03
    ......................aUagacguccugugugugcua.....................................................................   1   1   S03
    ......................acagacguccugugugugcuaacg..................................................................   1   0   S03
    .......................cagacguccugugugugc.......................................................................   1   0   S03
    .......................cagacguccugugugugcG......................................................................   1   1   S03
    .......................cagacguccugugugugcu......................................................................   14   0   S03
    .......................cagacguccugugugugcuC.....................................................................   1   1   S03
    .......................cagacguccugugugugcCa.....................................................................   1   1   S03
    .......................cagacguccugugugugcuaacg..................................................................   1   0   S03
    ........................agacguccugugugugcu......................................................................   3   0   S03
    ........................agacguccugugugugcG......................................................................   1   1   S03
    ........................agacguccugugugugcuaac...................................................................   1   0   S03
    ........................agacguccugugugugcuaacg..................................................................   1   0   S03
    .........................gacguccugCgugugcuaacgg.................................................................   1   1   S03
    .........................gacguccugugugugcuaacgg.................................................................   1   0   S03
    ..........................acguccugugugugcuaacg..................................................................   2   0   S03
    ..........................acguccugugugugcuaacggC................................................................   1   1   S03
    .............................Cccugugugugcuaacggaca..............................................................   1   1   S03
    .............................uccugugugugcuaacggacaU.............................................................   1   1   S03
    ..............................ccugugugugcuaacggaca..............................................................   2   0   S03
    ..............................ccugugugugcuaacggaAac.............................................................   1   1   S03
    ...............................cugugugugcuaacggCca..............................................................   1   1   S03
    ...............................cugugugugcuaacggacUca............................................................   2   1   S03
    .......................................gcuaacggacacacacggaca....................................................   1   0   S03
    ..........................................aacggacacacacggacaCc..................................................   1   1   S03
    ...........................................acggacacacacggacagc..................................................   1   0   S03
```

Mature **Star**

nnnnnnnnnnnnnnnnnnnnncacagacguccugugugugcuaacggacacacacggacagccacagacgucuugugugugcugacagacacacacggacguccugugugu			
....acggacacacacggacagccU...	1	1	S03
....acggacacacacggacagcca...	2	0	S03
....acggacacacacggacagccac..	2	0	S03
....acggacacacacggacagccaca...	1	0	S03
....cggacacacacggacagcc..	1	0	S03
....cggacacacacggacagccac..	1	0	S03
....cggacaAacacggacagccac..	1	1	S03
....cggacacacGcggacagccac..	1	1	S03
....cggacacacacggacagccaca...	11	0	S03
....acacacacggacagccaca...	1	0	S03
....acacacGcggacagccacagacgu..	1	1	S03
....acacacacggacagccacagacgu..	5	0	S03
....cacacacggacagccacagGcg...	1	1	S03
....cacUcacggacagccacagacgu..	1	1	S03
....cacacacggacagccacagacgG..	1	1	S03
....cacacacggacagccacagacgu..	1	0	S03
....Aacacacggacagccacagacgu..	1	1	S03
....acacacggacagccacagacgu..	6	0	S03
....acacacggacagAcacagacgu..	2	1	S03
....acacacggacagccacagacguc.......................................	1	0	S03
....acacacggacagccacagacUucu......................................	1	1	S03
....cacGcggacagccacagacgu..	1	1	S03
....cUcacggacagccacagacgu..	1	1	S03
....cacacggacagccacagacguc.......................................	3	0	S03
....cacacggacagccacagacguA.......................................	1	1	S03
....acaGggacagccacagacgu..	1	1	S03
....acacggacagccacagacgu..	1	0	S03
....acacggacagccacagacguc.......................................	2	0	S03
....cacggacagccacagacgu..	2	0	S03
....cacggacaCccacagacguc.......................................	1	1	S03
....cacggacagccacagacguc.......................................	1	0	S03
....cacagacgucuuguguAugcugac.........................	1	1	S03
....aGagacgucuugugugugcuga..........................	1	1	S03
....cagacgucGugugugugcug...........................	1	1	S03
....cagacgucuugugugugcugacaga......................	1	0	S03
....agGcgucuugugugugcu............................	1	1	S03
....agacgucuugugugugcugaca........................	1	0	S03
....agacgucuugugugugcugacaga......................	1	0	S03
....agacgucuuguUugugcugacaga......................	1	1	S03
....acgucuuguguCugcugaca........................	1	1	S03
....acAucuugugugugcugaca........................	1	1	S03
....acAucuugugugugcugacag.......................	1	1	S03
....acgucuugugugugcugacaga......................	1	0	S03
....acgucuugugugugcugacagaca....................	1	0	S03
....cgucuugugugugcugaca........................	1	0	S03
....gucuugugugugcugaca........................	1	0	S03
....gucuGgugugugcugacaga......................	1	1	S03
....Aucuugugugugcugacagaca....................	1	1	S03
....gucuugugugugcugaGagaca....................	1	1	S03
....ucuugugugugcugacagaca....................	1	0	S03
....gugcugacagacacacacggacgA.........	1	1	S03
....ugcugacagacacacacggacgu.........	1	0	S03
....gcuAacagacacacacggacg..........	1	1	S03
....gcugacagacacacacgAacg..........	1	1	S03
....gcugacagacacacacgAacgucc.......	1	1	S03
....Cacacacacggacguccugu....	1	1	S03
....acacacacggacguccugu....	1	0	S03
....cacacacggacguccugAgu..	1	1	S03
.....................caGagacguccugugugugc..	1	1	S04
.....................caGagacguccugugugugcu...	1	1	S04
.....................cacagacguccugugugugcG...	3	1	S04
.....................cacagacguccugugugugcu...	11	0	S04
.....................cacagacgGccugugugugcu...	2	1	S04
.....................Nacagacguccugugugugcu...	1	1	S04
.....................Gacagacguccugugugugcua..	1	1	S04
.....................caAagacguccugugugugcua..	1	1	S04
.....................cacagacguccuAugugugcua..	1	1	S04
.....................cacagacguccugugugugcuC..	6	1	S04
.....................cacUgacguccugugugugcua..	2	1	S04

Mature Star

nnnnnnnnnnnnnnnnnnnnncacagacguccugugugugcuaacggacacacacggacagccacagacgucuugugugugcugacagacacacacggacguccugugugu			
.....................cCcagacguccugugugugcua..	1	1	S04
.....................cacagacguccugugugugcuCa...	1	1	S04
.....................Aacagacguccugugugugcuaacg...	1	1	S04
......................acagacguccugugugug...	1	0	S04
......................acagacguccugugugugc..	5	0	S04
......................acagacgucGugugugugcu...	2	1	S04
......................acagacguccugugugugcG...	4	1	S04
......................acagacguccugugugugcu...	67	0	S04
......................acagacgGccugugugugcu...	1	1	S04
......................acagacguccugAgugugcu...	1	1	S04
......................acagacguccugugugugcua..	2	0	S04
......................acUgacguccugugugugcua..	1	1	S04
......................acagacguccugugugugcuC..	2	1	S04
......................acaAacguccugugugugcua..	1	1	S04
......................aAagacguccugugugugcua..	1	1	S04
......................acagacguccugCgugugcua..	1	1	S04
......................acagacguccuAugugugcuaa...	1	1	S04
......................acagacguccugugugugcuCa...	1	1	S04
......................aUagacguccugugugugcuaac..	1	1	S04
......................acagacguccugugugugcuaacg...	4	0	S04
......................acagacgucAugugugugcuaacg...	1	1	S04
......................aUagacguccugugugugcuaacg...	1	1	S04
......................acagacAuccugugugugcuaacg...	1	1	S04
.......................cagacguccugugugugc..	6	0	S04
.......................cagacguccugugugugcu...	69	0	S04
.......................cagacguccugugugugcG...	5	1	S04
.......................cagacguUcugugugugcua..	1	1	S04
.......................cagacguccugugAgugcua..	1	1	S04
.......................cagaGguccugugugugcua..	1	1	S04
.......................cagacguccugugugugcuC..	4	1	S04
.......................cagacguccugugugugcua..	2	0	S04
.......................cUgacguccugugugugcua..	1	1	S04
.......................cagacguccugugugugcAa..	1	1	S04
.......................cagacguccugugugugcuaa...	1	0	S04
.......................cagacguUcugugugugcuaa...	1	1	S04
.......................caAacguccugugugugcuaac..	1	1	S04
.......................cagacguccuguguAugcuaac..	1	1	S04
.......................cagacguccugugugugcuCac..	1	1	S04
.......................cagacguccuguUugugcuaac..	1	1	S04
.......................cagacguccugugugugcuaac..	2	0	S04
.......................cagacguccugugugugcuaacg...	1	0	S04
.......................Aagacguccugugugugcuaacgg..	1	1	S04
.......................cagacguccugugugugcuaacgg..	2	0	S04
.......................cagacguccugugugugcuaacgU..	1	1	S04
........................agacguccugugugugcG...	3	1	S04
........................agacguccugugugugcu...	14	0	S04
........................Ngacguccugugugugcu...	2	1	S04
........................agacguccugugugAgcu...	1	1	S04
........................agacguccugugugugcua..	3	0	S04
........................agacguccugugugugcCa..	1	1	S04
........................agaAguccugugugugcua..	1	1	S04
........................agacguccugugugugcuaaU..	1	1	S04
........................agacguccugugugugcuaac..	1	0	S04
........................Ugacguccugugugugcuaacg...	1	1	S04
........................agacguccugugugugcuaacg...	2	0	S04
........................agacguccugugugugcuCacg...	1	1	S04
........................agacguccugugugugcuaGcg...	1	1	S04
........................agacguAcugugugugcuaacg...	1	1	S04
........................Cgacguccugugugugcuaacg...	1	1	S04
........................agacguccugugugugcuaacgA..	1	1	S04
.........................gacguccugugugugcGa..	1	1	S04
.........................gacguccugugugugcua..	4	0	S04
.........................gacguccugugugugcuaa...	1	0	S04
.........................gacguccugugugugcuaac..	1	0	S04
.........................gacguccugugugugcuaaA..	1	1	S04
.........................gacguccugugugugcuaacgg..	2	0	S04
.........................Aacguccugugugugcuaacgg..	1	1	S04
..........................acguccGgugugugcuaac..	1	1	S04
..........................acguccugugugugcuaacgg..	5	0	S04
..........................acguccugugugugcuaacgga...	1	0	S04

Mature Star

```
nnnnnnnnnnnnnnnnnnnnncacagacguccugugugugcuaacggacacacacggacagccacagacgucuugugugugcugacagacacacacggacguccugugugu
..........................acguccugugugugcuaaAgga...............................................................  1  1  S04
...........................cguccugugugugcuaacgg................................................................  2  0  S04
...........................cguccuguAugugcuaacgga...............................................................  1  1  S04
...........................cguAcugugugugcuaacggac..............................................................  1  1  S04
...........................cgucAugugugugcuaacggac..............................................................  1  1  S04
............................gCccugugugugcuaacg.................................................................  1  1  S04
............................guccugugugugcAaacggaca.............................................................  1  1  S04
.............................uccugugugugcuaUcggac..............................................................  1  1  S04
.............................uccugugugugcuaacggaca.............................................................  4  0  S04
.............................uccugugGgugcuaacggaca.............................................................  1  1  S04
.............................uccugugugugcuaacggacacU...........................................................  1  1  S04
..............................ccugugugugcuaacgga...............................................................  1  0  S04
..............................ccugugugugcuaacggac..............................................................  1  0  S04
..............................ccugugugugcuaacggCca.............................................................  1  1  S04
..............................ccugugugugcuaacggaca.............................................................  5  0  S04
..............................ccugugugugcuaacUgaca.............................................................  1  1  S04
..............................ccugugugugcuaacggacU.............................................................  1  1  S04
..............................ccugugugugcuaacggaAaca...........................................................  1  1  S04
..............................ccugugugugcuaacggacaca...........................................................  1  0  S04
...............................cugugugugcuaacggac..............................................................  2  0  S04
...............................cugugugugcuaacggacU.............................................................  1  1  S04
...............................Gugugugugcuaacggaca.............................................................  1  1  S04
....................................ugugcuaacggacacaGacgga.....................................................  1  1  S04
.....................................gugcuaacggacacaGacg.......................................................  1  1  S04
.....................................gugcuaacggacacacacggacC...................................................  1  1  S04
.....................................gugcuaacggacacacacggaca...................................................  1  0  S04
.....................................gugcuaacggacacacacggacag..................................................  1  0  S04
......................................ugcuaacggacacacacAgaca...................................................  1  1  S04
......................................ugcuaacggacacacacggaca...................................................  2  0  S04
......................................ugcuaacggacacacacggacagc.................................................  1  0  S04
......................................ugcuaacggacacacacggacaCc.................................................  1  1  S04
.......................................gcuaacggacacacacggaca...................................................  1  0  S04
........................................cuaacggacacacaUggacag..................................................  1  1  S04
.........................................uaacggacacacacggacagU.................................................  1  1  S04
.........................................uaacggacacacacggacagcA................................................  1  1  S04
.........................................uaacggacacacacggacagcU................................................  1  1  S04
.........................................uaacggacacacacAgacagcc................................................  1  1  S04
.........................................uaaAggacacacacggacagcc................................................  1  1  S04
..........................................aacggacacacacggacagcc................................................  1  0  S04
...........................................acggacGcacacggacagc.................................................  1  1  S04
...........................................acggacacacacggacagcc................................................  2  0  S04
...........................................acggUcacacacggacagcc................................................  1  1  S04
...........................................acggacacacacggacagcca...............................................  6  0  S04
...........................................acggacacacUcggacagccac..............................................  1  1  S04
...........................................acggacacacGcggacagccac..............................................  1  1  S04
...........................................acggacacacacggacagccUc..............................................  1  1  S04
...........................................acggacacacacggacagccac..............................................  8  0  S04
...........................................acggacacacacgCacagccaca.............................................  1  1  S04
...........................................acggacacacacggacagccaca.............................................  9  0  S04
............................................cggacacacacggacagcc................................................  7  0  S04
............................................cggacacCcacggacagcca...............................................  1  1  S04
............................................cggGcacacacggacagcca...............................................  1  1  S04
............................................cggacacacacggacagccU...............................................  1  1  S04
............................................cggacacacacggacagcca...............................................  8  0  S04
............................................cggacacacacCgacagcca...............................................  1  1  S04
............................................cggacacacacggacagccG...............................................  1  1  S04
............................................cggacacacacggacagccac..............................................  3  0  S04
............................................cggUcacacacggacagccac..............................................  1  1  S04
............................................cggacacacacggacagcGac..............................................  1  1  S04
............................................cggacacacacggacagccaca............................................. 29  0  S04
............................................cgUacacacacggacagccaca.............................................  1  1  S04
............................................cggacacacacggacagUcaca.............................................  2  1  S04
............................................cggacacacacggacaUccaca.............................................  1  1  S04
............................................cggacacacacggacagccaAa.............................................  1  1  S04
............................................cggacacacacggacCgccaca.............................................  1  1  S04
............................................cggacacacGcggacagccaca.............................................  1  1  S04
............................................Nggacacacacggacagccaca.............................................  1  1  S04
............................................cggacacacacggGcagccaca.............................................  1  1  S04
............................................cggacacacacggacagccCca.............................................  1  1  S04
............................................cggacacacacggacagccacaC............................................  1  1  S04
```

```
                      Mature                                                            Star
nnnnnnnnnnnnnnnnnnnnncacagacguccugugugugcuaacggacacacacggacagccacagacgucuugugugugcugacagacacacacggacguccugugugu
..............................................gacacacacggacagccG...............................................   1    1    S04
..............................................gacacacacggacagccaca.............................................   1    0    S04
..............................................gacacacacggacagccaAa.............................................   1    1    S04
...............................................acacacacggacagccaca.............................................   1    0    S04
...............................................acacCcacggacagccaca.............................................   1    1    S04
...............................................acacacacggacagccacag............................................   1    0    S04
...............................................acacacacggacagccacaga...........................................   4    0    S04
...............................................acacacacggacagccacagG...........................................   1    1    S04
...............................................acacacacggacagccacagac..........................................   2    0    S04
...............................................acacacacggacagccaGagacg.........................................   1    1    S04
...............................................acacacacggacagcAacagacg.........................................   1    1    S04
...............................................Ncacacacggacagccacagacg.........................................   1    1    S04
...............................................acacacacggacagccacagacg.........................................   4    0    S04
...............................................acacacUcggacagccacagacgu........................................   1    1    S04
...............................................acacacacggacagAcacagacgu........................................   1    1    S04
...............................................acacacacggacagcAacagacgu........................................   1    1    S04
...............................................acGcacacggacagccacagacgu........................................   1    1    S04
...............................................acacacacggacCgccacagacgu........................................   2    1    S04
...............................................acacacacggacagccacagacgu........................................   12   0    S04
................................................cacacacggacagccaca.............................................   2    0    S04
................................................cacacacggacagAcacaga...........................................   1    1    S04
................................................cacacacggacagcGacaga...........................................   1    1    S04
................................................cacacacggacagccacaga...........................................   2    0    S04
................................................cacacacggacagccacagac..........................................   1    0    S04
................................................cacacacggacagccacagaA..........................................   1    1    S04
................................................cacacacggacagAcacagacg.........................................   2    1    S04
................................................cacacacggacagccacagacg.........................................   10   0    S04
................................................cacacacggacagcGacagacg.........................................   1    1    S04
................................................cacacacggaGagccacagacg.........................................   1    1    S04
................................................cacacacggacagAcacagacgu........................................   1    1    S04
................................................cacacacggacCgccacagacgu........................................   1    1    S04
................................................cacacacggacagccacagacgu........................................   9    0    S04
................................................cacGcacggacagccacagacgu........................................   1    1    S04
................................................cacacacggacagccacagacgG........................................   1    1    S04
................................................cacacGcggacagccacagacgu........................................   1    1    S04
................................................cacacacggacagccacagacguc.......................................   1    0    S04
................................................cacacacggacagccacagacguA.......................................   1    1    S04
................................................Aacacacggacagccacagacguc.......................................   1    1    S04
................................................cacacacggacagcAacagacguc.......................................   1    1    S04
.................................................Gcacacggacagccacagac..........................................   1    1    S04
.................................................acacacggacagccacagacg.........................................   6    0    S04
.................................................acCcacggacagccacagacg.........................................   1    1    S04
.................................................acacacggacagAcacagacgu........................................   4    1    S04
.................................................acacacggacagccCcagacgu........................................   1    1    S04
.................................................Gcacacggacagccacagacgu........................................   1    1    S04
.................................................acacacggacagcAacagacgu........................................   1    1    S04
.................................................acacacggacCgccacagacgu........................................   1    1    S04
.................................................acacacggacagccacagacgu........................................   58   0    S04
.................................................acacUcggacagccacagacgu........................................   3    1    S04
.................................................acacacggacagGcacagacgu........................................   1    1    S04
.................................................acacacggacagccacagacgG........................................   4    1    S04
.................................................acCcacggacagccacagacgu........................................   2    1    S04
.................................................acacacggacagccacagacguA.......................................   1    1    S04
.................................................acacacggacagccacagacguc.......................................   2    0    S04
.................................................acacacggacagccacagacgucuu.....................................   1    0    S04
..................................................cacacggacagccUcagacg.........................................   1    1    S04
..................................................cacacggacagccacagacg.........................................   5    0    S04
..................................................cacacggacagccacagacgu........................................   24   0    S04
..................................................cCcacggacagccacagacguc.......................................   1    1    S04
..................................................cacacggacagccCcagacguc.......................................   1    1    S04
..................................................cacacggacagccacagacguc.......................................   16   0    S04
..................................................cacacggacagccacCgacguc.......................................   1    1    S04
..................................................cGcacggacagccacagacguc.......................................   1    1    S04
..................................................cacacggacagccacagacguUu......................................   1    1    S04
...................................................acacggacagccacagac..........................................   1    0    S04
...................................................acacggacagccacagacg.........................................   5    0    S04
...................................................acacggacagccGcagacgu........................................   1    1    S04
...................................................acGcggacagccacagacgu........................................   1    1    S04
...................................................acUcggacagccacagacgu........................................   1    1    S04
...................................................acacggacagccacagacgu........................................   13   0    S04
```

Mature Star

```
nnnnnnnnnnnnnnnnnnnnncacagacguccugugugugcuaacggacacacacggacagccacagacgucuugugugugcugacagacacacacggacguccugugugu
...................................................acacggacagccacagacguc.......................................   6   0   S04
...................................................acacggCcagccacagacguc.......................................   1   1   S04
...................................................acacggacagccacagacguA.......................................   2   1   S04
...................................................acacggacCgccacagacguc.......................................   1   1   S04
...................................................acacggacagccacagacgucu......................................   2   0   S04
....................................................cacggacagccacagacg.........................................   3   0   S04
....................................................cacggacagccacagacgu........................................   6   0   S04
....................................................cacggacagccacagacguc.......................................   6   0   S04
....................................................cGcggacagccacagacgucuu.....................................   2   1   S04
....................................................cacggacagccacagacgucGu.....................................   1   1   S04
.....................................................acggacagccCcagacgu........................................   1   1   S04
.....................................................acggacagccacagacgu........................................   2   0   S04
.....................................................acggacagccacagacguc.......................................   2   0   S04
.....................................................acggacagccacagacgucuuU....................................   1   1   S04
.....................................................acggacagccacagacgucuug....................................   1   0   S04
.....................................................acggacagccacagacgucGug....................................   1   1   S04
......................................................cggacagccacagacgucuugu...................................   2   0   S04
......................................................cggacagccaGagacgucuugu...................................   1   1   S04
......................................................cggacagccacagacgucuGgugu.................................   1   1   S04
......................................................cggacagccacagacgucuugugu.................................   2   0   S04
.......................................................ggacagccacagacgucGugu...................................   1   1   S04
.........................................................acagccGcagacgucuugugugug..............................   1   1   S04
.............................................................ccaAagacgucuugugugugcu............................   1   1   S04
..............................................................cacagacgucuugugugugcug...........................   1   0   S04
...............................................................acagacgucGugugugugcu............................   2   1   S04
...............................................................acagacgucuugugugugcG............................   1   1   S04
...............................................................acagacgucGugugugugcug...........................   1   1   S04
...............................................................aAagacgucuugugugugcuga..........................   1   1   S04
...............................................................acagGcgucuugugugugcuga..........................   1   1   S04
...............................................................acagacgucuugugugugcugaca........................   1   0   S04
...............................................................acagacgucGugugugugcugaca........................   2   1   S04
................................................................cagacgucuugugugugc.............................   1   0   S04
................................................................cagacgucuugugugugcA............................   1   1   S04
................................................................cagacgucuugugugugcu............................   1   0   S04
................................................................cagacgucuugugugugcugac.........................   1   0   S04
................................................................cUgacgucuugugugugcugac.........................   1   1   S04
................................................................cagacgucGugugugugcugac.........................   3   1   S04
................................................................cagaAgucuugugugugcugaca........................   1   1   S04
................................................................cagacgucuugugugugcugacU........................   1   1   S04
................................................................Uagacgucuugugugugcugaca........................   2   1   S04
.................................................................agacgucuugugugugcuC...........................   1   1   S04
.................................................................agacgucuugugugugcugC..........................   2   1   S04
.................................................................agacgucuugugugugcuga..........................   1   0   S04
.................................................................agacgucuugugugugcugac.........................   3   0   S04
.................................................................agacgucuuguguCugcugac.........................   1   1   S04
.................................................................agacgucuugugugugcugacC........................   4   1   S04
.................................................................aUacgucuugugugugcugaca........................   1   1   S04
.................................................................agacgucuugugugugcugaUa........................   1   1   S04
.................................................................Ugacgucuugugugugcugaca........................   1   1   S04
.................................................................agacgucuuguCugugcugaca........................   1   1   S04
.................................................................agacgucuugugugugcuUaca........................   1   1   S04
.................................................................agacgucGugugugugcugaca........................   1   1   S04
.................................................................agacgAcuugugugugcugaca........................   1   1   S04
.................................................................agacgucuugugugugcugaca........................   15  0   S04
.................................................................agacgucuuCugugugcugaca........................   1   1   S04
.................................................................agaAgucuugugugugcugaca........................   1   1   S04
.................................................................agacgucuugugugugcugacU........................   1   1   S04
.................................................................agacAucuugugugugcugaca........................   1   1   S04
.................................................................agacgucuugugugugcugGca........................   1   1   S04
.................................................................agacgucuuguCugugcugacaga......................   1   1   S04
.................................................................agacgucuugugugugcugacaga......................   2   0   S04
..................................................................gacgucuugugugugcuC...........................   1   1   S04
..................................................................gacgucuugugugugcuga..........................   1   0   S04
..................................................................gacgucuugugugugcugac.........................   1   0   S04
..................................................................gacgucuugugugugcugaca........................   5   0   S04
..................................................................gacgucuugCgugugcugaca........................   1   1   S04
..................................................................gacgucuugugugugcugacU........................   1   1   S04
..................................................................gacgucuugugugugcugacaC.......................   1   1   S04
..................................................................gacgucuugugugugcugacaga......................   2   0   S04
...................................................................acgucuugugugugcugU..........................   1   1   S04
```

Mature / Star

nnnnnnnnnnnnnnnnnnnnnca**cagacguccugugugugcu**aacggacacacacggacagccacagacgucuugugugugcugacag**acacacacggacguccugu**gugu			
....acgucuugugugugcugaca........................	3	0	S04
....acgucuugugugugcugCca........................	1	1	S04
....acgucuugugugugcugacU........................	1	1	S04
....acgucuuguguguAcugaca........................	1	1	S04
....acgucuugugugugcugaGag.......................	1	1	S04
....acgucuuCugugugcugacaga......................	1	1	S04
....acgucuugugugugcugGcaga......................	1	1	S04
....acgucuugugugugcugacagG......................	1	1	S04
....acguAuugugugugcugacaga......................	1	1	S04
....acgucuugugugugcugacaCa......................	3	1	S04
....acgucuugugugugcugaGaga......................	2	1	S04
....acgucuugugugugcugCcaga......................	1	1	S04
....acgGcuugugugugcugacaga......................	1	1	S04
....acgucuugugugugcugacagU......................	1	1	S04
....acgucuugugugugcugacaga......................	5	0	S04
....acgCcuugugugugcugacaga......................	1	1	S04
....acgucuugugugugcugacagaca....................	1	0	S04
....cgucuugugugugcugac.........................	2	0	S04
....cgucuugugugugcugacC........................	1	1	S04
....cAucuugugugugcugaca........................	1	1	S04
....cgucuugugugugcugaca........................	1	0	S04
....cgucuugugugugcugCca........................	1	1	S04
....cAucuugugugugcugacaga......................	1	1	S04
....cgucuugugugugcugacagaca....................	4	0	S04
....gucuugugugugcugaca........................	4	0	S04
....gucuuguCugugcugaca........................	1	1	S04
....gucuugugugugcugGcaga......................	1	1	S04
....Aucuugugugugcugacaga......................	1	1	S04
....Aucuugugugugcugacagaca....................	1	1	S04
....ucuugugugugcugUcagaca....................	1	1	S04
....ucuugugugugcugacagaca....................	1	0	S04
....cuugugugugcugacagacC....................	1	1	S04
....cuugugugugcugaUagaca....................	1	1	S04
....cuugugugugcugaGagaca....................	1	1	S04
....cuugugugugcugacagacacC..................	1	1	S04
....cuugugugugcugacagacaGa..................	1	1	S04
....cuugugugGgcugacagacaca..................	1	1	S04
....cuugugugugcugacagaGaca..................	1	1	S04
....ugugugcugacagacacacacggacU..........	1	1	S04
....ugugcGgacagacacacacgga............	1	1	S04
....gugcugaUagacacacacgg.............	1	1	S04
....gugcugacagacacacacUgac...........	1	1	S04
....gugcugacagacacacacgAacg..........	1	1	S04
....gugcugacagacacacacggacg..........	1	0	S04
....gugcugacagacacacacggacgu.........	1	0	S04
....ugcugacagacacacaAgga............	1	1	S04
....ugcugacagacacacacgAacg..........	2	1	S04
....ugcugacagacacacacggacU..........	1	1	S04
....ugcugacagacacaAacggacg..........	1	1	S04
....ugcugacagacacacacggacgG.........	2	1	S04
....ugcugacagaUacacacggacgu.........	1	1	S04
....ugcugacagacacacacgAacgu.........	1	1	S04
....gcugacagacacacacggacg..........	1	0	S04
....gcugacagacacUcacggacgu.........	1	1	S04
....gcugacagacaUacacggacgu.........	1	1	S04
....gcugacaAacacacacggacgu.........	1	1	S04
....gcugacagacacacacggacgu.........	3	0	S04
....cugacagacacaGacggacg..........	1	1	S04
....cugacagacacacacggacg..........	2	0	S04
....ugacagacacacacggacgu.........	1	0	S04
....ugacagacacaAacggacgu.........	1	1	S04
....ugacagacacacacggacguU........	1	1	S04
....ugacagacacUcacggacgucc.......	1	1	S04
....ugacaAacacacacggacguccu......	1	1	S04
....gacagacacacacggacgu.........	1	0	S04
....acagacacacacggacgucc.......	1	0	S04
....acagacacacGcggacguccu......	2	1	S04
....acagacacacacggacguccugug...	1	0	S04
....cagacacacacggacguccug.....	1	0	S04
....cagacacacacggacguccugu....	2	0	S04

```
                       Mature                                                        Star
nnnnnnnnnnnnnnnnnnnnncacagacguccugugugugcuaacggacacacacggacagccacagacgucuugugugugcugacagacacacacggacguccugugugu
......................................................................................agacacacacggacguUcu......    1    1    S04
......................................................................................agacacacacggacguccugug...    1    0    S04
......................................................................................aCacacacacggacguccugug...    1    1    S04
.......................................................................................gacacacacggacguccu......    1    0    S04
.......................................................................................gaAacacacggacguccugu....    1    1    S04
.......................................................................................gacacacacggacguccAgu....    1    1    S04
.......................................................................................gacacacacggacguccugu....    1    0    S04
.......................................................................................gacacacacggacguccuguC...    1    1    S04
.......................................................................................gacacacGcggacguccugug...    1    1    S04
........................................................................................acacacacggacguccug.....    2    0    S04
........................................................................................acacacacggacguccugu....    7    0    S04
.........................................................................................cacacacggacguccugC....    1    1    S04
.........................................................................................cacacacggacguccugu....    3    0    S04
.........................................................................................cacacacggacguccugCg...    1    1    S04
.........................................................................................cacacacggacguccugCgu..    1    1    S04
..........................................................................................acaAacggacguccugugu..    1    1    S04
.............................................................................................cacggacgAccugugugu    1    1    S04
```

Provisional ID	:	chrAnn_random_3010500
Score total	:	0
Score for star read(s)	:	-1.3
Score for read counts	:	0
Score for mfe	:	1.3
Score for randfold	:	0
Score for cons. seed	:	
Total read count	:	243
Mature read count	:	232
Loop read count	:	0
Star read count	:	11

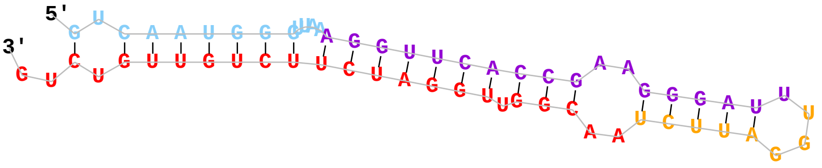

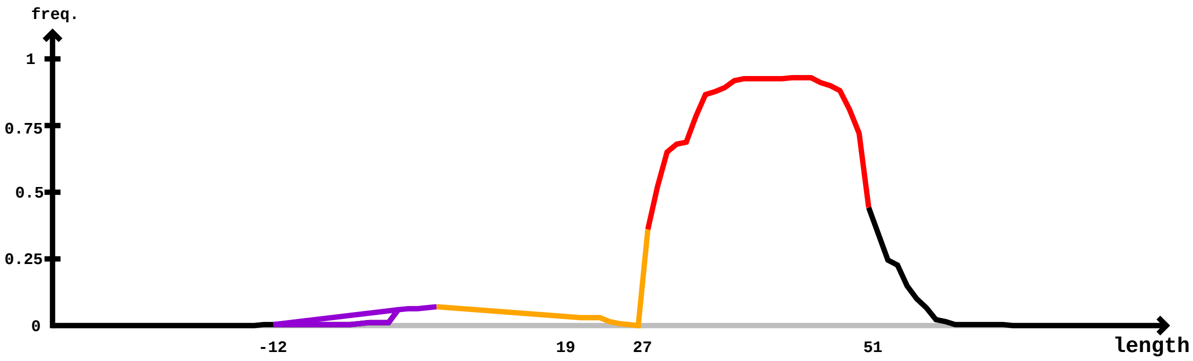


```
                                   Star                     Mature
5'- auuuugugggaagcuucgucagucaauggguuaaagguucaccgaagggauuuggauucuaacgguuggaucuucuguugucugggguuuucgugaagcuggucgucacaguucu   -3'   obs
    auuuugugggaagcuucgucagucaauggguuaaagguucaccgaagggauuuggauucuaacgguuggaucuucuguugucugggguuuucgugaagcuggucgucacaguucu         exp
    ...(((((((((((((((((((.(((((((....((((((((((..(((((....)))))..))).)))))))))))))).)))).......)))))))..)).))))))....   reads   mm   sample
    ..............................uuaaagguucaccgaagggauuu.............................................................   1       0    S03
    ...............................uaCagguucaccgaagggauuu.............................................................   1       1    S03
    ..................................agguucaccgaagggauu..............................................................   1       0    S03
    ..................................agguucaccgaagggauuugga..........................................................   3       0    S03
    ..................................agguucaccgaagggauuuggau.........................................................   2       0    S03
    ...................................gguucaccgaagggauuu.............................................................   1       0    S03
    ......................................ucaccgaagggauuuggauuc.......................................................   1       0    S03
    ............................................................aacgguuggaucuucugu....................................   2       0    S03
    ............................................................aacgguuggaucuucuguug..................................   2       0    S03
    ............................................................aacgguuggaucuucuguugu.................................   5       0    S03
    ............................................................aacgguuggaucuucuguuguc................................   7       0    S03
    ............................................................aacgguuggaucuucuguugucu...............................   11      0    S03
    ............................................................aacgguuggaucuucuguugucC...............................   2       1    S03
    ............................................................aacgguuAgaucuucuguugucug..............................   1       1    S03
    ............................................................Gacgguuggaucuucuguugucug..............................   1       1    S03
    ............................................................aacgguuggaucuucuguugucug..............................   14      0    S03
    ............................................................aacgguuggaucuucuguuguAug..............................   1       1    S03
    .............................................................acgguuggaucuucuguugu.................................   2       0    S03
    .............................................................acgguuggaucuucuguuguc................................   5       0    S03
    .............................................................acgguuggaucuucuguugucu...............................   6       0    S03
    .............................................................acgguuggaucuucuguugucC...............................   1       1    S03
    .............................................................acgguuggGucuucuguugucu...............................   1       1    S03
    .............................................................acgguuggaucuucuguugucug..............................   1       0    S03
    .............................................................acgguuggaucuucuguugucugg.............................   3       0    S03
    ..............................................................cgguuggaucuucuguuguc................................   1       0    S03
    ..............................................................cgguuggaucuucuguugucA...............................   1       1    S03
    ..............................................................cggGuggaucuucuguugucu...............................   1       1    S03
    ..............................................................cgguuggaucuucuguugucu...............................   6       0    S03
    ..............................................................cgguuggaucuucuguugucug..............................   2       0    S03
    ..............................................................cgguuggaucuucuguugucugg.............................   2       0    S03
    ..............................................................cgguuggaucuucuguugucuggg............................   2       0    S03
    ...............................................................gguuggaucuucuguugu.................................   1       0    S03
    ...............................................................gguuggaucuucuguugucu...............................   2       0    S03
```

Star Mature

```
auuuugugggaagcuucgucagucaauggguuaaagguucaccgaagggauuuggauucuaacgguuggaucuucuguugucugggguuuucgugaagcuggucgucacaguucu
...............................................................gguuggaucuucuguugucugg.............................  1  0  S03
...............................................................gguuggaucuucuguugucugggu...........................  1  0  S03
................................................................guugAaucuucuguugucug..............................  1  1  S03
................................................................guuggaucuucuguugucuggguu..........................  1  0  S03
.................................................................uuggaucuucuguugucugggu...........................  5  0  S03
.................................................................uugAaucuucuguugucugggu...........................  1  1  S03
.................................................................uuggaucuucuguugucuggguu..........................  2  0  S03
.................................................................uuggaucuucuguugucuggguuu.........................  4  0  S03
..................................................................uggaucuucuguugucugg.............................  1  0  S03
..................................................................uggaucuucuguugucuggg............................  2  0  S03
..................................................................uggaucuucuguugucuggguC..........................  1  1  S03
..................................................................uggaucuucuguugucuggguu..........................  1  0  S03
..................................................................uggaucuucuguugucuggguuuu........................  5  0  S03
...................................................................ggaucuucuguugucuggg............................  1  0  S03
...................................................................ggaucuucuguugucuggguC..........................  1  1  S03
...................................................................ggaucuucuguugucuggguu..........................  1  0  S03
....................................................................gaucuucuguugucugggA...........................  1  1  S03
.....................................................................aucuucuguugucugggu...........................  2  0  S03
.....................................................................aucuucuguugucuggguuuuc.......................  1  0  S03
......................................................................ucuucuguugucuggguuuucC......................  1  1  S03
...........................................................................uguugucuggguuuucCugaagc................  1  1  S03

....................agucaauggguuaaagguucaccg......................................................................  1  0  S04
..................................agguucaccgaagggauu..............................................................  6  0  S04
..................................agguucaccgaagggauuuggauA........................................................  1  1  S04
.....................................uucaccgaagggauuugga..........................................................  1  0  S04
............................................................aacgguuggaucuucugu....................................  2  0  S04
............................................................aacgguuggaucuucugC....................................  1  1  S04
............................................................aacgguuggaucuucuguC...................................  1  1  S04
............................................................aacgguuggaucuucuguug..................................  1  0  S04
............................................................aacgguuggaucuucuguugC.................................  1  1  S04
............................................................aacgguuggaucuucuguugu.................................  8  0  S04
............................................................aacgguugCaucuucuguugu.................................  1  1  S04
............................................................aacgguuggaucuucuguugCc................................  2  1  S04
............................................................aacgguuggaucuucuguuguc................................  4  0  S04
............................................................aacgguuggaucuucuguugucu...............................  13  0  S04
............................................................aacgguuggaucuucuguugucC...............................  1  1  S04
............................................................aacgguuggaucuucuguugucA...............................  3  1  S04
............................................................aacgguuggaucuucuguuguAug..............................  1  1  S04
............................................................aacgguuggaucuucuguugucug..............................  11  0  S04
............................................................aacgguuggaucuucuguugucugg.............................  1  0  S04
.............................................................acgguuggaucuucuguu...................................  2  0  S04
.............................................................acgguuggaucuucuguug..................................  1  0  S04
.............................................................acgguuggaucuucuguuguc................................  2  0  S04
.............................................................acgguuggaucuucuguugucA...............................  1  1  S04
.............................................................acgguuggaucuucuguugucu...............................  12  0  S04
.............................................................acgguuggaucuucuguugucug..............................  1  0  S04
.............................................................acgguuggaucuucuguugucugg.............................  5  0  S04
..............................................................cgguuggaucuucuguug..................................  1  0  S04
..............................................................cgguuggaucuucuguuguc................................  2  0  S04
..............................................................cggCuggaucuucuguugucu...............................  1  1  S04
..............................................................cgguuggaucuucuguugucA...............................  1  1  S04
..............................................................cgguuggaucuucuguugucC...............................  2  1  S04
..............................................................cgguuggaucuucuguugucu...............................  10  0  S04
..............................................................cgguuggaucuucuguugucug..............................  3  0  S04
...............................................................gguuggaucuucuguugC.................................  1  1  S04
...............................................................gguuggaucuucuguuguc................................  1  0  S04
...............................................................gguuggaucuucuguugucugggu...........................  1  0  S04
.................................................................uuggaucuucuguugucugg.............................  2  0  S04
.................................................................uuggaucuucuguugucugggA...........................  1  1  S04
.................................................................uuggaucuucuguugucugggu...........................  5  0  S04
.................................................................uuggaucuucuguugucugggAu..........................  1  1  S04
.................................................................uuggaucuucuguugucuggAuu..........................  3  1  S04
.................................................................uuggaucuucuguugucuggguuu.........................  1  0  S04
.................................................................uuggaucuucuguugucuggguuC.........................  1  1  S04
..................................................................uggaucuucuguugucugg.............................  1  0  S04
..................................................................uggaucuucuguugucugggu...........................  3  0  S04
..................................................................uggaucuucuguugucuggguu..........................  2  0  S04
..................................................................uggaucuucuguugucuggAuuu.........................  1  1  S04
..................................................................uggaucuucuguugucuggguuu.........................  1  0  S04
```

Star **Mature**

```
auuuugugggaagcuucgucagucaauggguuaaagguucaccgaagggauuuggauucuaacgguuggaucuucuguugucuggguuuucgugaagcuggucgucacaguucu
..................................................................uggaucuucuguuNucuggguuuu........................   1   1   S04
..................................................................uggaucuucuguugucuggguuuu........................   3   0   S04
....................................................................gaucuucuguugucuggguuu.........................   1   0   S04
....................................................................gaucuucuguugucuggguuuu........................   1   0   S04
....................................................................Uaucuucuguugucuggguuuucg......................   1   1   S04
.....................................................................aucuucuguugucugggu...........................   1   0   S04
.....................................................................aucuucuguugucuggguuuu........................   2   0   S04
.....................................................................aucuucuguugucuggguuuuc.......................   1   0   S04
......................................................................ucuucuguugucuggguuuucC......................   1   1   S04
```

Provisional ID	:	chrAnn_random_3025499
Score total	:	1.4
Score for star read(s)	:	-1.3
Score for read counts	:	0
Score for mfe	:	2.7
Score for randfold	:	0
Score for cons. seed	:	
Total read count	:	55
Mature read count	:	24
Loop read count	:	31
Star read count	:	0

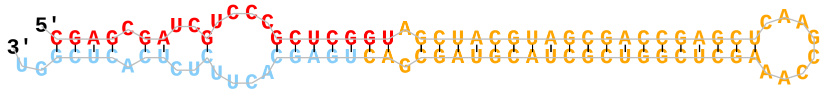

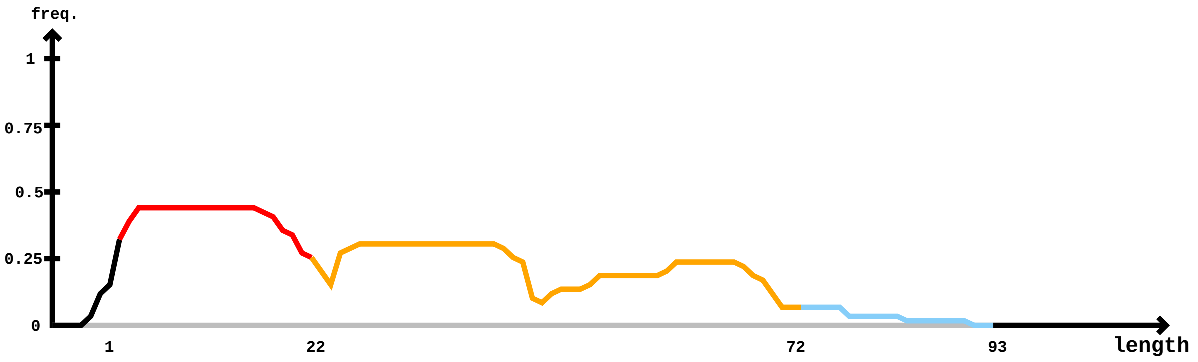

Mature Star

```
5'- gugaccgagcgaucgucccgcucgguagcuacguagcgaccgagcucaagccaaagcucggucgcuacguagcgacugagcacuucucucacucggucgcuacgcaacgaccgg  -3'    exp
    (((((((((.((..(....(((((((.(((((((((((((((((((........))))))))))))))))))).))))))).....)..)).))))))))).............. reads   mm   sample
    ..gaccgagcgaucgucccg.............................................................................................. 1   0   S03
    ..gaccgagcgaucgucccgc............................................................................................. 1   0   S03
    ...accgaAcgaucgucccgcu............................................................................................ 2   1   S03
    ...accgaAcgaucgucccgcucg.......................................................................................... 2   1   S03
    ....ccgagcgaucgucccgcCc........................................................................................... 1   1   S03
    .....cgaAcgaucgucccgcucg.......................................................................................... 1   1   S03
    .....cgaAcgaucgucccgcucggu........................................................................................ 9   1   S03
    ......gaAcgaucgucccgcucgg......................................................................................... 1   1   S03
    ......gaAcgaucgucccgcucggu........................................................................................ 3   1   S03
    .......agcgaucgucccgcucggu........................................................................................ 2   0   S03
    .......agcgaucgucccgcucgguU....................................................................................... 1   1   S03
    ..........................Ggcuacguagcgaccgagcuc................................................................... 1   1   S03
    ..........................agcuacguagcgaccgagcuca.................................................................. 1   0   S03
    ...........................gcuacguagcgaccgagc..................................................................... 1   0   S03
    ...........................gcuacguagcgaccgagcuc................................................................... 1   0   S03
    ...........................gcuacguagcgaccgagcuca.................................................................. 3   0   S03
    ............................cuacguagcgaccgagcu.................................................................... 2   0   S03
    ............................cuacguagcgaccgagcuca.................................................................. 5   0   S03
    ..............................acguagcgaccgagcucaa................................................................. 1   0   S03
    ................................................agccaaaAcucggucgcuacgu............................................ 1   1   S03
    ................................................agccaaagcucggucgcuacguag.......................................... 1   0   S03
    ..................................................ccaaagcucggucgcuacguagcg........................................ 2   0   S03
    ...................................................caaagcucggucgcuacgu............................................ 1   0   S03
    ...................................................caaagcucggucgcuacguagc......................................... 1   0   S03
    ......................................................agcucggucgcuacguagc......................................... 1   0   S03
    .......................................................gcucggucgcuacguagc......................................... 1   0   S03
    ...............................................................gcuacguagcgacugagc................................. 1   0   S03
    ......................................................................agcgacugagcacuucucucacuc.................... 1   0   S03

    ...accgaAcgaucgucccgcucg.......................................................................................... 1   1   S04
    ....ccgagcgaucgucccgcu............................................................................................ 1   0   S04
    ...........................gcuacguagcgaccgagcuca.................................................................. 1   0   S04
    ...........................gcuacguagcgaccgaAcucaagc............................................................... 1   1   S04
    .............................uacguagcgaccgagcuc................................................................... 1   0   S04
```

```
   Mature                                                        Star
gugaccgagcgaucgucccgcucgguagcuacguagcgaccgagcucaagccaaagcucggucgcuacguagcgacugagcacuucucucacucggucgcuacgcaacgaccgg
...............................................aagccaaagcucggucgcuGcgua...........................................	1	1	S04
...............................................aagccaaGgcucggucgcuacgua...........................................	1	1	S04
.......................................................gcucggucgcuacguagcg........................................	1	0	S04
..............................................................cgcuacguagcgacugagc.................................	1	0	S04
...............................................................gcuacguagcgacugagcUcuucu...........................	1	1	S04
```

Provisional ID	: chrAnn_random_3038174	
Score total	:	0.4
Score for star read(s)	:	-1.3
Score for read counts	:	0
Score for mfe	:	1.7
Score for randfold	:	0
Score for cons. seed	:	
Total read count	:	76
Mature read count	:	76
Loop read count	:	0
Star read count	:	0

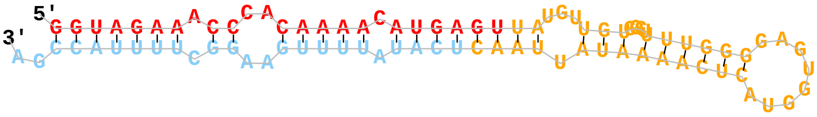

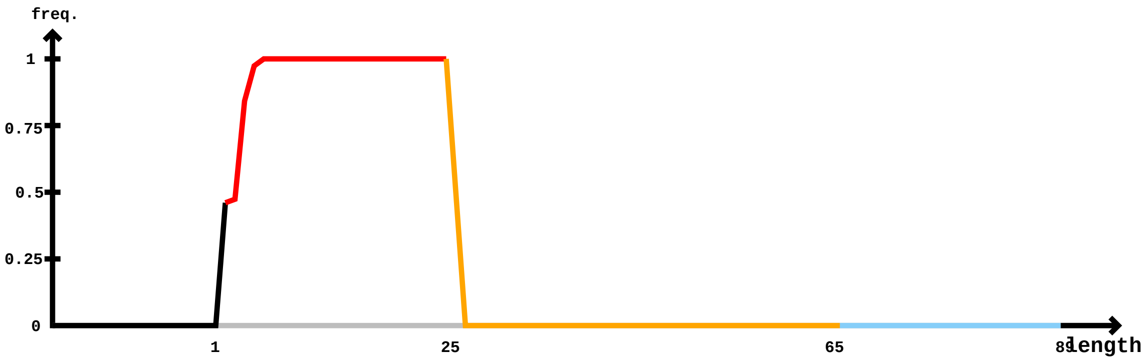

Mature Star

```
5'- gauuuccuccauacucgguagaaacccacaaaacaugaguuauguuguggcuuuuggggagugguacucaaaauauuaacucauauuuugaaggcuuuuaccgaguaug   -3'    exp
    .........(((((((((((((.((..(((((.((((((((...(((....((((((........))))))))).)))))))).)))))..)).)))))))))))))   reads      mm          sample
    ................gguCgaaacccacaaaacaugagu.....................................................................   24         1           S03
    .................guCgaaacccacaaaacaugagu.....................................................................   1          1           S03
    ..................uCgaaacccacaaaacaugagu.....................................................................   12         1           S03
    ...................Cgaaacccacaaaacaugagu.....................................................................   6          1           S03
    ....................gaaacccacaaaacaugagA.....................................................................   1          1           S03
    ....................gaaacccacaaaacaugagu.....................................................................   1          0           S03

    ................gguCgaaacccacaaaacaugagu.....................................................................   11         1           S04
    ..................uCgaaacccacaaaacaugagu.....................................................................   16         1           S04
    ...................agaaacccacaaaacaugagu.....................................................................   1          0           S04
    ...................Cgaaacccacaaaacaugagu.....................................................................   3          1           S04
```

Provisional ID	:	chrAnn_random_3040064
Score total	:	266.7
Score for star read(s)	:	3.9
Score for read counts	:	260.6
Score for mfe	:	2.2
Score for randfold	:	0
Score for cons. seed	:	
Total read count	:	523
Mature read count	:	521
Loop read count	:	0
Star read count	:	2

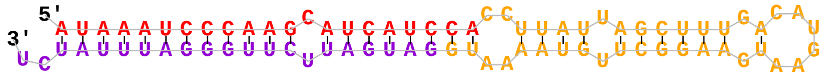

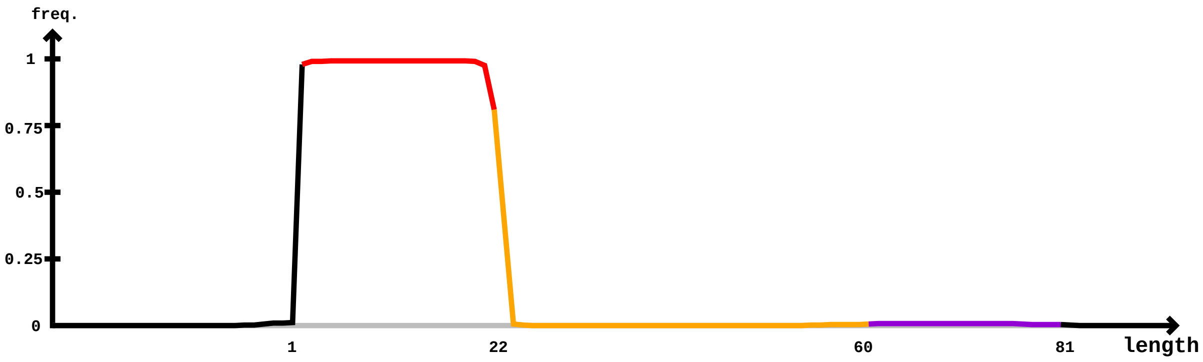


```
                              Mature                                                     Star
5'- ucuuuuuguuugggugauaacaagauaaaucccaagcaucauccaccuuauuagcuuugacaugaaugaaggcuuguaaaauggaugauucuugggauuuaucuuuuuguagccu  -3'   obs
    ucuuuuuguuugggugauaacaagauaaaucccaagcaucauccaccuuauuagcuuugacaugaaugaaggcuuguaaaauggaugauucuugggauuuaucuuuuuguagccu        exp
    ..........((((.((((.((((((((((((((.((((((((..((((.((((((.(......).)))))).))))..)))))))).))))))))))))))).)))).))))  reads   mm   sample
    .......................gauaaaucccaagcaucaucA.......................................................................  1       1    S03
    ........................auaaaucccaagcaucauc........................................................................  3       0    S03
    ........................auaaaucccaagcaucaucc.......................................................................  18      0    S03
    ........................auaaaucccaagcaucaucG.......................................................................  1       1    S03
    ........................auaaaucccaagcaucaucU.......................................................................  5       1    S03
    ........................auaaaucccaagcaucaucA.......................................................................  1       1    S03
    ........................Nuaaaucccaagcaucaucc.......................................................................  1       1    S03
    ........................auaaaucccGagcaucaucca......................................................................  1       1    S03
    ........................auaaaucccaagcaucauccG......................................................................  7       1    S03
    ........................auaaaucccaagGaucaucca......................................................................  1       1    S03
    ........................auaUaucccaagcaucaucca......................................................................  1       1    S03
    ........................auaaaucccaagcaucauccC......................................................................  5       1    S03
    ........................auaaaucccaagcaucauccU......................................................................  2       1    S03
    ........................auaaaucccaagcaCcaucca......................................................................  1       1    S03
    ........................auaaaucccaagcaucaucca......................................................................  123     0    S03
    ........................auGaaucccaagcaucaucca......................................................................  1       1    S03
    ........................auaaaucccaagcaucGucca......................................................................  1       1    S03
    ........................auaaaucccaagcaucauccaA.....................................................................  1       1    S03
    .........................uaaaucccaagcaucaucc.......................................................................  1       0    S03
    .........................uaaaucccaagcaucaucGa......................................................................  1       1    S03
    ...............................................................................aauggaugauucuugggauuG...............  1       1    S03
    ....................................................................................augauucuugggauuuaucu...........  1       0    S03

    ..................aacaagauaaaucccaagcaucaucca......................................................................  1       0    S04
    ....................caagauaaaucccaagcaucaucca......................................................................  2       0    S04
    .....................aagauaaaucccaagcaucauccG......................................................................  1       1    S04
    .....................aagauaaaucccaagcaucauccaU.....................................................................  1       1    S04
    ........................auaaaucccaagcaucaG.........................................................................  1       1    S04
    ........................auaaaucccaagcaucauc........................................................................  5       0    S04
    ........................aCaaaucccaagcaucaucc.......................................................................  1       1    S04
    ........................auaaaucccaagcaucaucA.......................................................................  3       1    S04
    ........................auaaaucccaagcaucUucc.......................................................................  1       1    S04
    ........................auaaaucccaagcaucaucc.......................................................................  39      0    S04
```

```
                        Mature                                                     Star
ucuuuuuguuugggugauaacaagauaaaucccaagcaucauccaccuuauuagcuuugacaugaaugaaggcuuguaaaauggaugauucuugggauuuaucuuuuuguagccu
........................auaaaucccaagcaucaucU.......................................................................    16    1    S04
........................auaaaucccaGgcaucaucca......................................................................    1     1    S04
........................auaaaucccaagcaucauccG......................................................................    18    1    S04
........................aGaaaucccaagcaucaucca......................................................................    1     1    S04
........................auaaaucccCagcaucaucca......................................................................    1     1    S04
........................auaaaucccaagcaucauccU......................................................................    6     1    S04
........................auaaaucccaagcaucauccC......................................................................    8     1    S04
........................auaaaucccaagcUucaucca......................................................................    1     1    S04
........................auaaaucccaagcaucaGcca......................................................................    1     1    S04
........................auaaaucccaagcaucaucGa......................................................................    1     1    S04
........................auaaGucccaagcaucaucca......................................................................    1     1    S04
........................auaaaucccaagcaucaucca......................................................................    228   0    S04
........................auaaauccUaagcaucaucca......................................................................    1     1    S04
........................Nuaaaucccaagcaucaucca......................................................................    1     1    S04
........................auaaaucccaagcaucaCcca......................................................................    2     1    S04
........................auaaaucccaagcaucauccaU.....................................................................    1     1    S04
........................auaaaucccaagcaucauccacA....................................................................    1     1    S04
........................auaaaucccaagcaucauccacc....................................................................    1     0    S04
.........................uaaaucccaagcaucauccG......................................................................    1     1    S04
.........................uaaaucccaagcaucaucca......................................................................    3     0    S04
...........................aaucccaagcaucauccaccu...................................................................    1     0    S04
.............................................................................aaaauggaugauucuugggauA................    1     1    S04
...................................................................................gaugauucuugggauuuaucu...........    1     0    S04
```

Provisional ID	:	chrAnn_random_3042427
Score total	:	0
Score for star read(s)	:	-1.3
Score for read counts	:	0
Score for mfe	:	1.2
Score for randfold	:	0
Score for cons. seed	:	
Total read count	:	37
Mature read count	:	37
Loop read count	:	0
Star read count	:	0

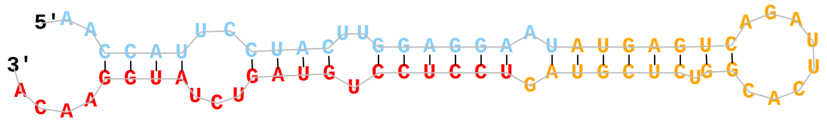

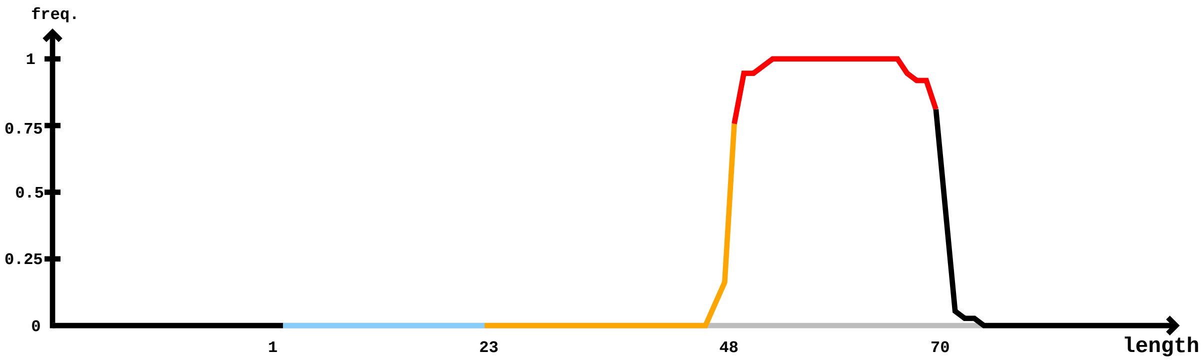


```
                   Star                                    Mature
5'- gguucuugcuuugaauccguucaaccauuccuacuuggaggaauaugagucagauucacggucucguaguccuccuguagucuauggaacaauucaauuuucgacgcgaaugcug  -3'   exp
    (((..((((.(((((.........((((..((((..((((((.((((((((........)).)))))).)))))).))))...))))...........))))).))))..))).  reads   mm   sample
    ...................................................................aguccuccuguagucuCuggaaca........................  2       1    S03
    ....................................................................guccuccuguagucuCuggaaca........................  2       1    S03
    .....................................................................uccuccuguagucuCuggaac.........................  2       1    S03
    .....................................................................uccuccuguagucuCuggaaca........................  9       1    S03
    .....................................................................uccuccuguagucuUuggaaca........................  1       1    S03
    .....................................................................uccuccuguagucuCuggaacaa.......................  1       1    S03
    ......................................................................ccuccuguagucuCuggaac.........................  1       1    S03
    ......................................................................ccuccuguagucuCuggaaca........................  3       1    S03
    ......................................................................ccuccuguagucuCuggaacaa.......................  1       1    S03
    ......................................................................ccuccuguagucuauggaacaauuc....................  1       0    S03
    ........................................................................uccuguagucuCuggaacaau......................  1       1    S03

    ...................................................................aguccuccuguagucuCuggaaca........................  1       1    S04
    ....................................................................guccuccuguagucuCuggaac.........................  1       1    S04
    .....................................................................uccuccuguagucuCugg............................  2       1    S04
    .....................................................................uccuccuguagucuCugga...........................  1       1    S04
    .....................................................................uccuccuguagucuCuggaaca........................  5       1    S04
    .....................................................................uccuccuguagucuCuggaacaa.......................  1       1    S04
    ......................................................................ccuccuguagucuCuggaaca........................  1       1    S04
    .........................................................................ccuguagucuCuggaacaa.......................  1       1    S04
```

Provisional ID	:	chrAnn_random_3061750
Score total	:	0.5
Score for star read(s)	:	-1.3
Score for read counts	:	0
Score for mfe	:	1.8
Score for randfold	:	0
Score for cons. seed	:	
Total read count	:	28
Mature read count	:	21
Loop read count	:	2
Star read count	:	5

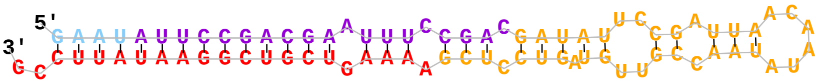

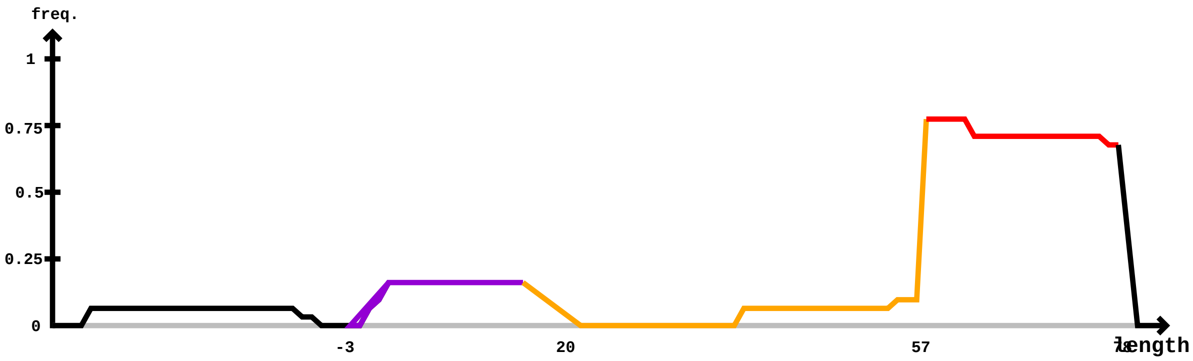


```
                                 Star                                                  Mature
5'- uaaccguuguagucgucgaaaaguugucggaauauuccgacgaauuuccgacgauauuccgauuaacaauauaaccguuguaguccucgaaaagucgucggaauauuccgacga -3'   obs
    uaaccguuguagucgucgaaaaguugucggaauauuccgacgaauuuccgacgauauuccgauuaacaauauaaccguuguaguccucgaaaagucgucggaauauuccgacga        exp
    .......................((((((((((((((((((((.(((.(((.(((((..((.(((......))).))..)).))).))).))).)))))))))))))))))))))   reads   mm   sample
    ..accguuguagucgucgaaaagu..........................................................................................   1       0    S03
    ......................................................................auaaccguuguaguccucgaaaag....................   2       0    S03
    .........................................................................................aaaagucgucggaauauuc......   1       0    S03
    .........................................................................................aaaagucgucggaauauuccC....   1       1    S03
    .........................................................................................aaaGgucgucggaauauuccg....   1       1    S03
    .........................................................................................aaaagucgucggaauauuccg....   9       0    S03
    .........................................................................................aaaagucgucggaauauuccA....   2       1    S03

    ..accguuguagucgucgaaaaguug........................................................................................   1       0    S04
    ...............................auauuccgacgaauuuccgac..............................................................   1       0    S04
    ...............................auauuccgacgaauuuccgacA.............................................................   1       1    S04
    ................................uauuccgacgaauuuccgac..............................................................   1       0    S04
    .................................auuccgacgaauuuccgac..............................................................   2       0    S04
    ......................................................................................ucgaaaagucgucggaauauuccA....   1       1    S04
    .........................................................................................aaaGgucgucggaauauuccg....   1       1    S04
    .........................................................................................aaaagucgucggaauauuccA....   1       1    S04
    .........................................................................................aaaagucgucggaauauuccg....   5       0    S04
```

Provisional ID	:	chrAnn_random_3077354
Score total	:	1.7
Score for star read(s)	:	3.9
Score for read counts	:	-3.3
Score for mfe	:	1.3
Score for randfold	:	0
Score for cons. seed	:	
Total read count	:	5
Mature read count	:	1
Loop read count	:	0
Star read count	:	4

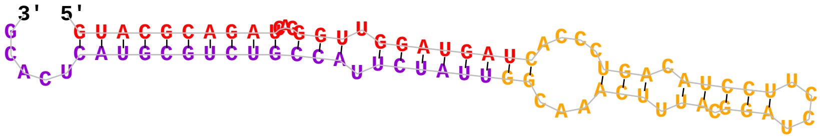

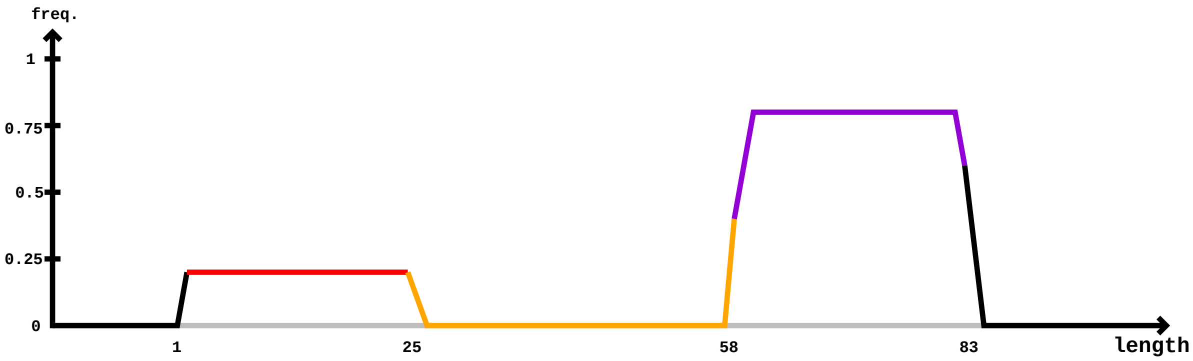


```
                Mature                                              Star
5'- uaucaccgcaagguacgcagaucaggguuggaugaucacccugacauccuucuaggcauuucaaacgguuaucuuaccgucugcguacucacgcaaacaccaaagagaacuua   -3'   obs
    uaucaccgcaagguacgcagaucaggguuggaugaucacccugacauccuucuaggcauuucaaacgguuaucuuaccgucugcguacucacgcaaacaccaaagagaacuua         exp
    .......((..(((((((((((...(((.((((((((....(((.(((((....))).)).)))...)))))))).))))))))))))))...))..................   reads   mm   sample
    ............guacgcagaucaggguuggaugau.............................................................................   1       0    S03
    .....................................................................uaucuuaccgucugcguacucacg....................   1       0    S03

    ....................................................................uuaucuuaccgucugcguacucac.....................   1       0    S04
    ....................................................................uuaucuuaccgucugcguacucacA....................   1       1    S04
    ......................................................................aucuuaccgucugcguacucacgU...................   1       1    S04
```

Provisional ID	:	chrAnn_random_3078550
Score total	:	1890.6
Score for star read(s)	:	3.9
Score for read counts	:	1885.5
Score for mfe	:	1.2
Score for randfold	:	0
Score for cons. seed	:	
Total read count	:	3710
Mature read count	:	3706
Loop read count	:	1
Star read count	:	3

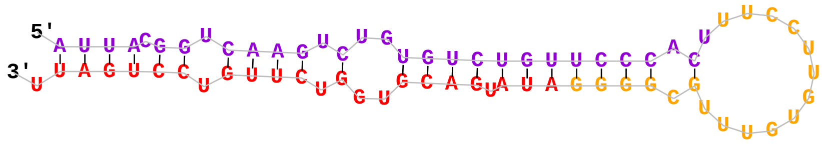

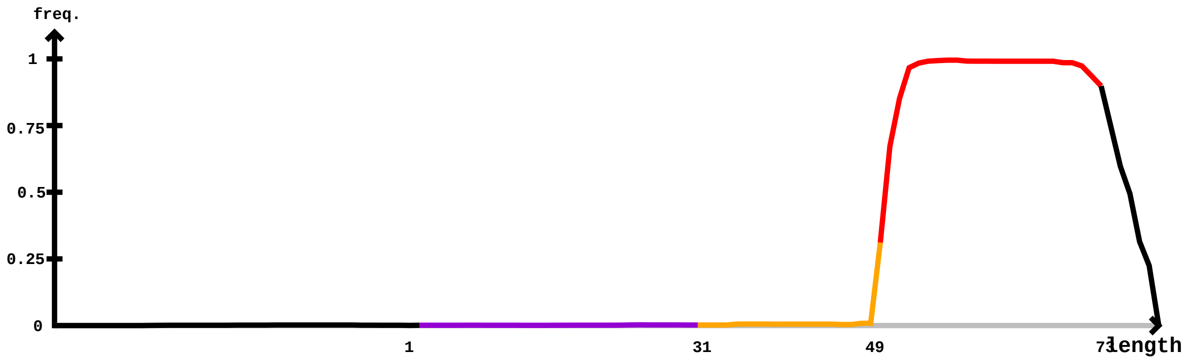

Star **Mature**

```
5'- ucuguccugcuaagcacguuugucuccggacaaaagauuacggucaagucugugucuguucccacuuuccuuguguuugcggggauaugacguggucuuguccugauuuauga  -3'   obs
    ucuguccugcuaagcacguuugucuccggacaaaagauuacggucaagucugugucuguucccacuuuccuuguguuugcggggauaugacguggucuuguccugauuuauga         exp
    ..(((((.(.((((....)))).)...)))))..((((((.((.((((.(..(((((((((((.(.............).))))))).))))..).)))).))))))))....   reads   mm   sample
    ........gcuaagcacguuugucuccgga...................................................................................   2   0   S03
    .................guuugucuccggacaaaG..............................................................................   1   1   S03
    .....................gucuccggacaaaagauuacgg......................................................................   1   0   S03
    .....................gucuAcggacaaaagauuacggucaa..................................................................   1   1   S03
    ....................................auGacggucaagucugugucuguucccacu...............................................   1   1   S03
    ....................................................Cgucuguucccacuuuccuug........................................   1   1   S03
    ..........................................................uucccacuuuccuuguguuugU.................................   1   1   S03
    ..................................................................uuccuuAuguuugcggggauaugacgu....................   1   1   S03
    .....................................................................cuuguguuugcggggauaugacgu....................   2   0   S03
    ..................................................................................ggauaugacguggucuuguccugauu.....   2   0   S03
    ..................................................................................gCauaugacguggucuuguccugauu.....   1   1   S03
    ..................................................................................ggauaugacguggucuuguccugauC.....   1   1   S03
    ..................................................................................ggauaugacguggucuuguccugauuua...   2   0   S03
    ..................................................................................ggauaugacguggucuuguccugauuuau..   1   0   S03
    ...................................................................................gauaugacguggucuuguccug........   1   0   S03
    ....................................................................................auaugacguggucuugucU..........   1   1   S03
    ....................................................................................auaugacguggucuugucc..........   1   0   S03
    ....................................................................................auauUacguggucuuguccug........   1   1   S03
    ....................................................................................auaugacguggucuuguccuA........   2   1   S03
    ....................................................................................auaugacguggucuuguccug........   3   0   S03
    ....................................................................................auaugacguggucuuguccugG.......   1   1   S03
    ....................................................................................auaugacguggucuuguccuga.......   9   0   S03
    ....................................................................................auaugacgCggucuuguccuga.......   1   1   S03
    ....................................................................................auaugacguggucuuguccugau......   10  0   S03
    ....................................................................................auaugacguggucuuguccugaA......   4   1   S03
    ....................................................................................auaCgacguggucuuguccugauu.....   1   1   S03
    ....................................................................................auGugacguggucuuguccugauu.....   1   1   S03
    ....................................................................................auaugacgCggucuuguccugauu.....   2   1   S03
    ....................................................................................aCaugacguggucuuguccugauu.....   1   1   S03
    ....................................................................................auaugacguggucuuguccugauG.....   6   1   S03
    ....................................................................................auaugacguggucuuguccugauu.....   274 0   S03
    ....................................................................................auaugacguggucuuguccugaAu.....   1   1   S03
    ....................................................................................auaugacguggucuuguccugGuu.....   1   1   S03
```

```
                                    Star                                              Mature
ucuguccugcuaagcacguuugucuccggacaaaagauuacggucaagucugugucuguucccacuuuccuuguguuugcggggauaugacguggucuuguccugauuuauga
....................................................................................auaGgacguggucuuguccugauu.....	1	1	S03
....................................................................................auaugacguggucuuUuccugauu.....	1	1	S03
....................................................................................aAaugacguggucuuguccugauu.....	1	1	S03
....................................................................................auaugacguggucuuguUcugauu.....	1	1	S03
....................................................................................auaugacguggucuuguccCgauu.....	1	1	S03
....................................................................................auaugacguggucuugAccugauu.....	2	1	S03
....................................................................................auaugacguggucuuguccugauC.....	22	1	S03
....................................................................................auaugacguggucuCguccugauu.....	2	1	S03
....................................................................................auaugacguggucuuguccugauA.....	6	1	S03
....................................................................................auaugacguggucuAguccugauu.....	1	1	S03
....................................................................................auaugacguggucuuguccugauuA....	1	1	S03
....................................................................................auaugacguggucuuguccugauuua...	12	0	S03
....................................................................................auaugacguggucuuguccugauuuG...	1	1	S03
....................................................................................auaugacguggucuuUuccugauuua...	1	1	S03
....................................................................................auaugacguggucuuguccugauuuU...	1	1	S03
....................................................................................auaugacguggucuuguccugauuuau..	3	0	S03
....................................................................................auaugacguggucuuguccugauuAau..	1	1	S03
....................................................................................auaugacguggucuuguccugauuuaA..	2	1	S03
....................................................................................auaugacguggucuuguccugauuuaug.	7	0	S03
....................................................................................auaugacguggucuuguccugauuuauA.	1	1	S03
....................................................................................auaugacgGggucuuguccugauuuaug.	1	1	S03
....................................................................................auaugacguggucuuguccugauuuauC.	1	1	S03
....................................................................................auaugacguggucuuguccugauuuaAg.	1	1	S03
....................................................................................auaugacguggucuuguccugauuuauU.	2	1	S03
....................................................................................auaugacguggucuuguccugauuuaugG	3	1	S03
....................................................................................auaugacguggucuuguccugauuuaugU	2	1	S03
....................................................................................auaugacguggucuuguccugauuuauga	25	0	S03
.....................................................................................uaugacguggucuugucc..........	3	0	S03
.....................................................................................uaugacguggucuugucU..........	4	1	S03
.....................................................................................uaugacguggucuuguccug........	5	0	S03
.....................................................................................uaugacguggucuuguccugG.......	2	1	S03
.....................................................................................uaugGcguggucuuguccuga.......	1	1	S03
.....................................................................................uaugacguggucuuguccuga.......	30	0	S03
.....................................................................................uaugacguggucuuguccugaA......	2	1	S03
.....................................................................................Caugacguggucuuguccugau......	1	1	S03
.....................................................................................uaugaUguggucuuguccugau......	1	1	S03
.....................................................................................uaugacguggucuuguccugau......	17	0	S03
.....................................................................................uaugacguggucuuguccugauA.....	10	1	S03
.....................................................................................uaugacguggucuuguccugauG.....	2	1	S03
.....................................................................................uGugacguggucuuguccugauu.....	1	1	S03
.....................................................................................uaugacguggucuugucUugauu.....	1	1	S03
.....................................................................................uaugacguggucuuguccugauC.....	2	1	S03
.....................................................................................uaugacguggucuuguccugauu.....	15	0	S03
.....................................................................................uaugacguggucuugAccugauuu....	1	1	S03
.....................................................................................uaugacguggucuuguccugauCu....	1	1	S03
.....................................................................................uaugacguggucuuguccugauuG....	1	1	S03
.....................................................................................uaugacguggucuuguccugauuu....	3	0	S03
.....................................................................................uaugacguggucuuguccugauuua...	73	0	S03
.....................................................................................uaugacguggucuuguccugauuuU...	2	1	S03
.....................................................................................uaugacguUgucuuguccugauuua...	1	1	S03
.....................................................................................uaugacguggucuuguccugauuuG...	3	1	S03
.....................................................................................uaugGcguggucuuguccugauuua...	2	1	S03
.....................................................................................Caugacguggucuuguccugauuua...	1	1	S03
.....................................................................................uaugacgCggucuuguccugauuua...	1	1	S03
.....................................................................................uaugacguggucuCguccugauuuau..	1	1	S03
.....................................................................................uaGgacguggucuuguccugauuuau..	1	1	S03
.....................................................................................uaugacguggucuuguccugGuuuau..	1	1	S03
.....................................................................................uaugacUuggucuuguccugauuuau..	1	1	S03
.....................................................................................uaugacguggucuuguccCgauuuau..	1	1	S03
.....................................................................................uaugacguggucuuguccugauuuau..	103	0	S03
.....................................................................................uaugacguggucuuguccugauuuaA..	7	1	S03
.....................................................................................uaugacguggucuuguccugauuuGu..	1	1	S03
.....................................................................................uauCacguggucuuguccugauuuau..	1	1	S03
.....................................................................................uaugacguggucuuguccugauuuUu..	1	1	S03
.....................................................................................uaugacguggucuuguccugauuuaC..	14	1	S03
.....................................................................................uaugGcguggucuuguccugauuuau..	2	1	S03
.....................................................................................uaugacguggucCuguccugauuuau..	1	1	S03
.....................................................................................uaugacguggucuuguccugauuuaug.	41	0	S03
.....................................................................................uaugacgCggucuuguccugauuuaug.	1	1	S03
.....................................................................................uaugacguggucuuguccugauuuauA.	2	1	S03
```

```
                                    Star                                          Mature
ucuguccugcuaagcacguuugucuccggacaaaagauuacggucaagucugugucuguucccacuuuccuuguguuugcggggauaugacguggucuuguccugauuuauga
.....................................................................................uaugacguggucuuguccugauuuauU.	1	1	S03
.....................................................................................uaugacguggucuuguccugauuuauC.	1	1	S03
.....................................................................................uaugacguggucuuguccugauuuGug.	2	1	S03
.....................................................................................uaugacguggucuuguccCgauuuaug.	1	1	S03
.....................................................................................uaugGcguggucuuguccugauuuaug.	1	1	S03
.....................................................................................uaugacguggucuuguccugGuuuaug.	1	1	S03
.....................................................................................uaugGcguggucuuguccugauuuauga	1	1	S03
.....................................................................................uUugacguggucuuguccugauuuauga	1	1	S03
.....................................................................................uaugacguggucuuguccugauCuauga	1	1	S03
.....................................................................................uaugacguggucuuguGcugauuuauga	1	1	S03
.....................................................................................uGugacguggucuuguccugauuuauga	1	1	S03
.....................................................................................uaugacguggucuuguccugauuuauga	138	0	S03
.....................................................................................uaugacguggAcuuguccugauuuauga	1	1	S03
.....................................................................................uaugacguggucuuguccugauuuaugU	5	1	S03
.....................................................................................uaugacguggucuuguccugauuuaugG	5	1	S03
.....................................................................................uaugacguggucuuguccugauuuauUa	1	1	S03
......................................................................................augacguggucuuguccug........	4	0	S03
......................................................................................augacguggucuuguccuga.......	5	0	S03
......................................................................................augacguggucuuguccugau......	8	0	S03
......................................................................................augacguggucuuguccugauu.....	10	0	S03
......................................................................................augacguggucuuguccugauA.....	1	1	S03
......................................................................................augacguggucuuguccugauC.....	1	1	S03
......................................................................................augacguggucuuguccugauuu....	1	0	S03
......................................................................................augacguggucuuguccugauuA....	1	1	S03
......................................................................................augacguggucuuguccugauuAa...	1	1	S03
......................................................................................augacguggucuuguccugauuuU...	1	1	S03
......................................................................................augacguggucuuguccugGuuua...	1	1	S03
......................................................................................augacguggucuuguccugauuua...	15	0	S03
......................................................................................augacguggucuuguccugauuuaC..	1	1	S03
......................................................................................augacguggucuuguccugauuuau..	59	0	S03
......................................................................................aCgacguggucuuguccugauuuau..	1	1	S03
......................................................................................aGgacguggucuuguccugauuuau..	1	1	S03
......................................................................................augacgugguUuuguccugauuuau..	1	1	S03
......................................................................................augacguggucuuguccugauuuaA..	2	1	S03
......................................................................................auAacguggucuuguccugauuuau..	1	1	S03
......................................................................................augacguggucuuguccugauuuaG..	1	1	S03
......................................................................................augacguggucuuguccugauuuauU.	6	1	S03
......................................................................................augacguggucuuguccugauuCaug.	1	1	S03
......................................................................................augacguggucuuguccugauuuaug.	32	0	S03
......................................................................................augacguggucuuguccugauuuaCg.	1	1	S03
......................................................................................augacguggucuuguccugauuuauA.	11	1	S03
......................................................................................augacguggucuuguccugauuuauga	63	0	S03
......................................................................................augacgCggucuuguccugauuuauga	1	1	S03
......................................................................................augacguggucuuguccugauuuaugG	3	1	S03
......................................................................................augacguggucuuguccugauuuaugU	2	1	S03
......................................................................................augacguggucuuguccugauuuaugC	1	1	S03
......................................................................................augacguggucuuguccugauuuaCga	2	1	S03
.......................................................................................ugacguggucuuguccug........	2	0	S03
.......................................................................................ugacguggucuuguccAga.......	1	1	S03
.......................................................................................ugacguggucuuguccuga.......	5	0	S03
.......................................................................................ugacguggucuuguccugau......	2	0	S03
.......................................................................................ugacguggucuuguccugauC.....	5	1	S03
.......................................................................................uUacguggucuuguccugauu.....	1	1	S03
.......................................................................................ugacguggucuuguccugauA.....	3	1	S03
.......................................................................................ugacguggucuuguccugauG.....	1	1	S03
.......................................................................................ugacguggucuuguccugauu.....	28	0	S03
.......................................................................................ugacguggucuuguccugauuu....	1	0	S03
.......................................................................................ugacguggucuuguccCgauuua...	1	1	S03
.......................................................................................ugacguggucuCguccugauuua...	1	1	S03
.......................................................................................ugacguggucuuguccugauuua...	18	0	S03
.......................................................................................Cgacguggucuuguccugauuua...	1	1	S03
.......................................................................................Ggacguggucuuguccugauuua...	1	1	S03
.......................................................................................ugacguggucuuguccugauuuU...	2	1	S03
.......................................................................................ugacguggucuuguccugauuuG...	1	1	S03
.......................................................................................ugacguggucuuguccugauuuau..	38	0	S03
.......................................................................................ugacguggucuuguccugauuuaC..	5	1	S03
.......................................................................................ugacguggucuuguccuUauuuau..	1	1	S03
.......................................................................................ugacguggucuuguccugauuuaG..	2	1	S03
.......................................................................................ugacAuggucuuguccugauuuau..	2	1	S03
.......................................................................................ugacguggucuuguccugauuuaA..	2	1	S03
```

```
                                    Star                                              Mature
ucuguccugcuaagcacguuugucuccggacaaaagauuacggucaagucugugucuguucccacuuucccuuguguuugcggggauaugacguggucuuguccugauuuauga
........................................................................................ugacguggucuuguccugauuuauA.    2     1     S03
........................................................................................ugacguggucuuguccugauuuauU.    2     1     S03
........................................................................................ugacguggucuuguccugauuuaug.    10    0     S03
........................................................................................ugacguggucuuguccugauuuaugU    1     1     S03
........................................................................................ugacguggucuuguccugauuuaugG    1     1     S03
........................................................................................ugacguggucuuguccuAauuuauga    1     1     S03
........................................................................................ugacguggucuuguccugauuuGuga    1     1     S03
........................................................................................ugacguggucuuguccugauuuauga    42    0     S03
.........................................................................................gacguggucuuguccuga.......    1     0     S03
.........................................................................................gacguggucuuguccugau......    1     0     S03
.........................................................................................gacguggucuuguccugauu.....    1     0     S03
.........................................................................................gacguggucuuguccugauC.....    1     1     S03
.........................................................................................gacguggucuuguccugauuua...    2     0     S03
.........................................................................................gacgCggucuuguccugauuua...    1     1     S03
.........................................................................................gacguggucuuguccugauuuaC..    1     1     S03
.........................................................................................gacguggucuuguccugauuuau..    3     0     S03
.........................................................................................gacguggucuuguccugauuuaA..    1     1     S03
.........................................................................................gacguggucuuguccCgauuuau..    1     1     S03
.........................................................................................gacguggucuuguccugauuuaug.    1     0     S03
.........................................................................................gacguggucuugGccugauuuaug.    1     1     S03
.........................................................................................gacguggucuuguccuAauuuauga    1     1     S03
.........................................................................................gacguggucuuguccugauuuauga    4     0     S03
..........................................................................................acguggucuuguccugauu.....    1     0     S03
..........................................................................................acguggucuuguccugauuua...    1     0     S03
..........................................................................................acguggucuuguccugauuuau..    1     0     S03
..........................................................................................acUuggucuuguccugauuuau..    1     1     S03
..........................................................................................acguggucuuguccugauuuaug.    2     0     S03
..........................................................................................acguggucuuguccugauuuauga    6     0     S03
...........................................................................................cguggucuuguccugauuuauU.    1     1     S03
...........................................................................................cguggucuuguccugauuuaug.    2     0     S03
............................................................................................guggucuuguccugauuuauga    2     0     S03

.........cuaagcacguuugucuccgga....................................................................................    1     0     S04
..........uaagcacguuugucuccggaca..................................................................................    1     0     S04
.................guuugucuccggacaaaa...............................................................................    1     0     S04
....................................auuacggucaagucugugucuguu......................................................    1     0     S04
.........................................ggucaagucugugucuguuccca..................................................    1     0     S04
..................................................ugCgucuguucccacuuucccuug........................................    1     1     S04
.........................................................guucccacuuucccuuguguuugc.................................    1     0     S04
..........................................................uucccacuuucccuuguguuugc.................................    2     0     S04
...........................................................ucccacuuucccuuguguuugU.................................    1     1     S04
......................................................................cuuguguuugGggggauaugacgu....................    1     1     S04
......................................................................cuuguguuugcggggauaugacgu....................    5     0     S04
......................................................................cuuguguuugcgggCauaugacgu....................    1     1     S04
......................................................................cuuguguuugcgggUauaugacgu....................    1     1     S04
......................................................................cuuguguuugcggggauUugacgu....................    1     1     S04
......................................................................cuuguguuugcggggauaugacgA....................    1     1     S04
.......................................................................uuguguuGgcggggauaugacgug...................    1     1     S04
...........................................................................guuugcggggauaugacguggu.................    1     0     S04
...................................................................................gCauaugacguggucuuguccuga.......    1     1     S04
...................................................................................gUauaugacguggucuuguccuga.......    1     1     S04
...................................................................................ggauaugacguggucuuguccugaA......    1     1     S04
...................................................................................ggauaugacguggucuuguccugauu.....    4     0     S04
...................................................................................ggauaugacguggucuuguccugauuG....    1     1     S04
...................................................................................ggauaugacguggucuuguccugauuGa...    1     1     S04
.....................................................................................auaugacguggucuugucc..........    3     0     S04
.....................................................................................auaugacguggucuuguccuA........    4     1     S04
.....................................................................................auGugacguggucuuguccug........    1     1     S04
.....................................................................................auaugacguggucuuguccug........    8     0     S04
.....................................................................................aCaugacguggucuuguccug........    1     1     S04
.....................................................................................auaugacguggucuuguccugG.......    1     1     S04
.....................................................................................auaugUcguggucuuguccuga.......    1     1     S04
.....................................................................................auaugacguggGcuuguccuga.......    1     1     S04
.....................................................................................auaugacguggucuuguccuga.......    23    0     S04
.....................................................................................auaugacguggucuuguccugau......    21    0     S04
.....................................................................................auaugacguggucuuguccugaA......    4     1     S04
.....................................................................................auaugacguggucuuguccugaG......    1     1     S04
.....................................................................................auaugacguggucuuguccugUu......    1     1     S04
.....................................................................................auaugacguggucuuguccugaC......    3     1     S04
.....................................................................................auaugacgGggucuuguccugau......    1     1     S04
```

Star Mature

```
ucuguccugcuaagcacguuugucuccggacaaaagauuacggucaagucugugucuguucccacuuuccuuguguuugcggggauaugacguggucuuguccugauuuauga
....................................................................................auaugacguggucuuguccugaCu.....	2	1	S04
....................................................................................auaugacguggucuuguUcugauu.....	1	1	S04
....................................................................................auaugaAguggucuuguccugauu.....	1	1	S04
....................................................................................auauAacguggucuuguccugauu.....	1	1	S04
....................................................................................Nuaugacguggucuuguccugauu.....	2	1	S04
....................................................................................auaugacgCggucuuguccugauu.....	2	1	S04
....................................................................................auaugaGguggucuuguccugauu.....	1	1	S04
....................................................................................auaugacguggucuuguccugauA.....	20	1	S04
....................................................................................auaugacguggucuuguccugaGu.....	2	1	S04
....................................................................................auaugacguggucuuguccugauu.....	422	0	S04
....................................................................................auaugUcguggucuuguccugauu.....	2	1	S04
....................................................................................auaCgacguggucuuguccugauu.....	1	1	S04
....................................................................................auaugacguggucuuguccugGuu.....	2	1	S04
....................................................................................auaugacguggucuugAccugauu.....	1	1	S04
....................................................................................aCaugacguggucuuguccugauu.....	6	1	S04
....................................................................................auaugGcguggucuuguccugauu.....	1	1	S04
....................................................................................aAaugacguggucuuguccugauu.....	2	1	S04
....................................................................................auaugacguggucuCguccugauu.....	1	1	S04
....................................................................................Uuaugacguggucuuguccugauu.....	3	1	S04
....................................................................................Guaugacguggucuuguccugauu.....	2	1	S04
....................................................................................auaugacguggucuugucUugauu.....	1	1	S04
....................................................................................auaugacguggucCuguccugauu.....	3	1	S04
....................................................................................auaugacAuggucuuguccugauu.....	1	1	S04
....................................................................................auGugacguggucuuguccugauu.....	3	1	S04
....................................................................................auaugacguggucuuguccugauG.....	7	1	S04
....................................................................................auaugacguggucuuguccugauC.....	29	1	S04
....................................................................................auUugacguggucuuguccugauu.....	1	1	S04
....................................................................................auaugacguggucuuguccugauuC....	1	1	S04
....................................................................................auaugacguggucuuguccugauuu....	5	0	S04
....................................................................................auaugacguggucuuguccugauuA....	3	1	S04
....................................................................................auaugacguggucuCguccugauuu....	1	1	S04
....................................................................................auaugacguggucuuguccugGuua...	2	1	S04
....................................................................................auauAacguggucuuguccugauuua...	1	1	S04
....................................................................................auaugacguggucuuguccugauuAa...	1	1	S04
....................................................................................auaugacguggucuuguccugauuua...	19	0	S04
....................................................................................auaugacguggucuuguccugauuuau..	10	0	S04
....................................................................................auaugacguggucuuguccugauuuaC..	1	1	S04
....................................................................................auaugacguggucuuguccugauuuaA..	1	1	S04
....................................................................................auaugacguggucuuguccugauuuUu..	1	1	S04
....................................................................................auaugacguggucuuguccugauuuauA.	5	1	S04
....................................................................................auaugacguggucuuguccugauuuaug.	18	0	S04
....................................................................................auaugacguggucuuguccugauuuauU.	1	1	S04
....................................................................................auGugacguggucuuguccugauuuauga	1	1	S04
....................................................................................auaugacguggucuuguccugauuuaugC	1	1	S04
....................................................................................auaugacguggucuuguccugauuuauga	40	0	S04
....................................................................................auaugacguggucuuAuccugauuuauga	1	1	S04
....................................................................................auaugacguggucuuguccugauuuaugU	1	1	S04
....................................................................................auaugacguggucuuguccugauuuaugG	1	1	S04
.....................................................................................uaugacguggucuugucA..........	2	1	S04
.....................................................................................uaugacguggucuugucc..........	4	0	S04
.....................................................................................uaugacguggucuugucU..........	2	1	S04
.....................................................................................uaugacguggucuuguccC.........	1	1	S04
.....................................................................................uaugacguggucuuguccug........	7	0	S04
.....................................................................................uaugacguggucuuAuccuga.......	1	1	S04
.....................................................................................Caugacguggucuuguccuga.......	1	1	S04
.....................................................................................uaugacguggucuuguAcuga.......	1	1	S04
.....................................................................................uaugacguggucuuguccuga.......	34	0	S04
.....................................................................................uaugacguggucuuguccugG.......	3	1	S04
.....................................................................................uaugacguggucuuguccugaA......	1	1	S04
.....................................................................................Gaugacguggucuuguccugau......	1	1	S04
.....................................................................................Naugacguggucuuguccugau......	1	1	S04
.....................................................................................uaugGcguggucuuguccugau......	2	1	S04
.....................................................................................uaugacguggucuuguccCgau......	1	1	S04
.....................................................................................uaugacguggucuuguccugau......	36	0	S04
.....................................................................................uaugacguggucuuguccugaC......	1	1	S04
.....................................................................................uGugacguggucuuguccugauu.....	1	1	S04
.....................................................................................uaugacguggucuuguccugauu.....	40	0	S04
.....................................................................................uaugacguggucAuguccugauu.....	1	1	S04
.....................................................................................uaugacguggucuuguccugauG.....	2	1	S04
.....................................................................................uaugacguggucuuguccugauC.....	2	1	S04
```

Star Mature

ucuguccugcuaagcacguuugucuccggacaaaagauuacggucaagucugugucuguucccacuuuccuuguguuugcggggauaugacguggucuuguccugauuuauga			
....uaugacguggucuuguccugauA.....	11	1	S04
....uaugacguggucuuguccCgauu.....	1	1	S04
....uaugacguggucuugAccugauuu....	4	1	S04
....uaugacguggucuuguccugauuu....	14	0	S04
....uaugacguggucuuguccugauuG....	1	1	S04
....Caugacguggucuuguccugauuua...	2	1	S04
....uaugacguggucuuguccugauuCa...	1	1	S04
....Gaugacguggucuuguccugauuua...	1	1	S04
....uaugacguggucuuguccugauuuU...	2	1	S04
....uaugacguggucuuguccugauuua...	119	0	S04
....uaugacguggucuuguccugauuGa...	2	1	S04
....uaugacguggAcuuguccugauuua...	1	1	S04
....uauUacguggucuuguccugauuuau..	1	1	S04
....Caugacguggucuuguccugauuuau..	1	1	S04
....Aaugacguggucuuguccugauuuau..	1	1	S04
....uaugacguggucuuAuccugauuuau..	1	1	S04
....uaugacguggucuuguccugauuuGu..	1	1	S04
....uaugacguggucuuguccugauuuaA..	17	1	S04
....uaugacguggucuuguccugauuuau..	150	0	S04
....uaugacguggucCuguccugauuuau..	1	1	S04
....uaugacgugguUuuguccugauuuau..	1	1	S04
....uaugacguggucuuguccugauuuaG..	4	1	S04
....uaugacguggucuCguccugauuuau..	1	1	S04
....uaugacguggucuuguccugauuuaC..	12	1	S04
....uaugacguggucuuguccugauuuauU.	4	1	S04
....uaugacguggucuuguccugauuuaug.	64	0	S04
....uaugacguggucuuguccugauCuaug.	1	1	S04
....uaugacguggucuuguccugauuuaAg.	1	1	S04
....uaugacguggucuuguccugauuuauA.	7	1	S04
....uaCgacguggucuuguccugauuuaug.	1	1	S04
....Gaugacguggucuuguccugauuuaug.	1	1	S04
....uaugacgCggucuuguccugauuuauga	1	1	S04
....uaugacguggucuuguAcugauuuauga	1	1	S04
....uaugacguggucuuguccugauuuaugC	1	1	S04
....uaugacguggucuuguccugGuuuauga	1	1	S04
....uaugacguggucuuguccugauGuauga	1	1	S04
....uaGgacguggucuuguccugauuuauga	1	1	S04
....uaugacguggucuuguccugauuuauAa	1	1	S04
....uGugacguggucuuguccugauuuauga	1	1	S04
....uaugacguggucuuguccugCuuuauga	1	1	S04
....uaugacguggucuuguccugauuuaugG	6	1	S04
....uaugacguggucuuguccugauuuaCga	1	1	S04
....uaugacguUgucuuguccugauuuauga	1	1	S04
....uaugacguggucuuguccCgauuuauga	1	1	S04
....Caugacguggucuuguccugauuuauga	1	1	S04
....uaugaUguggucuuguccugauuuauga	1	1	S04
....Gaugacguggucuuguccugauuuauga	1	1	S04
....uaugacguggucuuguccugauuuauga	216	0	S04
....Naugacguggucuuguccugauuuauga	1	1	S04
....uaugacguggucuuguccugauuuaugU	9	1	S04
....uaugacguggucuuguUcugauuuauga	1	1	S04
....uaugGcguggucuuguccugauuuauga	2	1	S04
....augacguggucuuguccug........	3	0	S04
....augacguggucuuguccuga.......	7	0	S04
....augacguggucuuguccugaC......	1	1	S04
....augacguggucuuguccugaA......	1	1	S04
....augacgugAucuuguccugau......	1	1	S04
....augacguggucuuguccugau......	7	0	S04
....augacguggucuuguccugauu.....	27	0	S04
....augacguggucAuguccugauu.....	1	1	S04
....augacguggucuuguccugauC.....	4	1	S04
....augacgCggucuuguccugauu.....	1	1	S04
....augacguggucuuguccugauuu....	3	0	S04
....augacguggucuugGccugauuu....	1	1	S04
....augacguggucuuguccugauAu....	1	1	S04
....augacgugguUuuguccugauuua...	1	1	S04
....augacguggucuuguccugauuua...	54	0	S04
....augacguggucuuguccugauuuaA..	7	1	S04
....aCgacguggucuuguccugauuuau..	1	1	S04
....Nugacguggucuuguccugauuuau..	1	1	S04

```
                                    Star                                          Mature
ucuguccugcuaagcacguuugucuccggacaaaagauuacggucaagucugugucuguucccacuuuccuuguguuugcggggauaugacguggucuuguccugauuuauga
......................................................................................augacguggucuuguccugauuuaG..  1    1    S04
......................................................................................augGcguggucuuguccugauuuau..  1    1    S04
......................................................................................Gugacguggucuuguccugauuuau..  2    1    S04
......................................................................................augacguggucuuguccugGuuuau..  1    1    S04
......................................................................................augacguggucuuguccugauuuaC..  9    1    S04
......................................................................................augacguggucuuguccugauuuau..  90   0    S04
......................................................................................augacguggucuuguccugauuuauC.  1    1    S04
......................................................................................augacAuggucuuguccugauuuaug.  1    1    S04
......................................................................................augacguggucuuguccugauuuauU.  1    1    S04
......................................................................................augacguggucuuguccugauuuauA.  8    1    S04
......................................................................................augacCuggucuuguccugauuuaug.  1    1    S04
......................................................................................augacguggucuGguccugauuuaug.  1    1    S04
......................................................................................aGgacguggucuuguccugauuuaug.  1    1    S04
......................................................................................augacguggucuuguccugauuuaug.  51   0    S04
......................................................................................augacguggucuuguccugauuuaugG  3    1    S04
......................................................................................aGgacguggucuuguccugauuuauga  1    1    S04
......................................................................................augacgCggucuuguccugauuuauga  2    1    S04
......................................................................................augacguggucuuguccugauuuaugC  1    1    S04
......................................................................................augacguggucuuguccugauuuauga  114  0    S04
......................................................................................aCgacguggucuuguccugauuuauga  1    1    S04
......................................................................................augacguggucuuguccCgauuuauga  2    1    S04
......................................................................................augacguggucuuguccugauuuauUa  1    1    S04
......................................................................................augacguggucuuguccugauuuauAa  1    1    S04
......................................................................................augacguggGcuuguccugauuuauga  1    1    S04
......................................................................................Nugacguggucuuguccugauuuauga  1    1    S04
......................................................................................augacguggucuuguccugauuuaugU  6    1    S04
......................................................................................augGcguggucuuguccugauuuauga  1    1    S04
......................................................................................augacguggucuCguccugauuuauga  1    1    S04
......................................................................................augacguggucuuguccugauuuaCga  1    1    S04
......................................................................................augacAuggucuuguccugauuuauga  1    1    S04
.......................................................................................ugacguggucuuguccug........  4    0    S04
.......................................................................................ugacguggucuuguccuga.......  6    0    S04
.......................................................................................ugacguggucuuguccugG.......  1    1    S04
.......................................................................................ugacguggucuuguccugaC......  1    1    S04
.......................................................................................ugacguggucuuguccugau......  3    0    S04
.......................................................................................ugacguggucuuguccugaG......  1    1    S04
.......................................................................................ugacguggucuuguccugaA......  1    1    S04
.......................................................................................ugacguggAcuuguccugauu.....  1    1    S04
.......................................................................................ugacguggucuuguccugauu.....  33   0    S04
.......................................................................................ugacguggucuuguccugauA.....  2    1    S04
.......................................................................................ugaUguggucuuguccugauu.....  1    1    S04
.......................................................................................ugacguggucuuguccugauC.....  3    1    S04
.......................................................................................ugacguggucuuguccugauuu....  4    0    S04
.......................................................................................ugacguggucuuguccugauuA....  1    1    S04
.......................................................................................ugacguggucuuguccugauuCa...  1    1    S04
.......................................................................................ugacgCggucuuguccugauuua...  1    1    S04
.......................................................................................ugacgugguUuuguccugauuua...  1    1    S04
.......................................................................................ugacguggucuuguccugauuuU...  1    1    S04
.......................................................................................ugacguggucuuguccugauuua...  18   0    S04
.......................................................................................ugacguggucuuguccugauuuaA..  7    1    S04
.......................................................................................ugacguggAcuuguccugauuuau..  1    1    S04
.......................................................................................ugacguggucuuguccugauuuau..  60   0    S04
.......................................................................................ugacguggucuuguccugauuuaC..  5    1    S04
.......................................................................................ugacguggucuuguccugauuuGu..  1    1    S04
.......................................................................................ugacguggucuuguGcugauuuau..  1    1    S04
.......................................................................................ugacguggucuuguccugauuuaG..  1    1    S04
.......................................................................................ugacguggucuuguccugauuuaAg.  1    1    S04
.......................................................................................ugacguggucuuguccugauuuaug.  18   0    S04
.......................................................................................ugacguggucuuguccugauuuauUa  1    1    S04
.......................................................................................ugacguggAcuuguccugauuuauga  1    1    S04
.......................................................................................ugacguggucuuguccugauuuaCga  2    1    S04
.......................................................................................ugacguggucuuguccugauuuaugG  3    1    S04
.......................................................................................ugacgugguUuuguccugauuuauga  1    1    S04
.......................................................................................Ggacguggucuuguccugauuuauga  1    1    S04
.......................................................................................ugacguggucuCguccugauuuauga  1    1    S04
.......................................................................................ugacguggucuuguccugauuuGuga  1    1    S04
.......................................................................................ugacguggucuuguccugauCuauga  1    1    S04
.......................................................................................ugacguggucuuguccugauuuauga  56   0    S04
.......................................................................................ugacguggucuuguccugUuuuauga  1    1    S04
........................................................................................gacguggucuuguccuga........  1    0    S04
```

Star Mature

```
ucuguccugcuaagcacguuugucuccggacaaaagauuacggucaagucugugucuguucccacuuuccuuguguuugcggggauaugacguggucuuguccugauuuauga
........................................................................................gacguggucuuguccugau......   4   0   S04
........................................................................................gacguggucuuguccugauu.....   3   0   S04
........................................................................................gacguggucuuguccCgauuu....   1   1   S04
........................................................................................Uacguggucuuguccugauuua...   1   1   S04
........................................................................................gacguggucuuguccugauuua...   7   0   S04
........................................................................................gacguggucuuguccugauuAa...   1   1   S04
........................................................................................gacgugAucuuguccugauuua...   1   1   S04
........................................................................................gacguggucuuguccugauuuaA..   3   1   S04
........................................................................................gacguggucuuguccugauuuau..   2   0   S04
........................................................................................gacguggucuugGccugauuuaug.   2   1   S04
........................................................................................gacguggucuuguccugauuuauU.   1   1   S04
........................................................................................gacguggucuuguccugauuuauA.   2   1   S04
........................................................................................Uacguggucuuguccugauuuaug.   1   1   S04
........................................................................................gacguggucuuguccugauuuaug.   2   0   S04
........................................................................................gGcguggucuuguccugauuuauga   1   1   S04
........................................................................................gacguggucuuguccugauuuauga   11  0   S04
........................................................................................gacguggucuuUuccugauuuauga   1   1   S04
.........................................................................................acguggucuuguccugau......   1   0   S04
.........................................................................................acguggucuuguccugauC.....   1   1   S04
.........................................................................................acguggucuuguccugauuu....   1   0   S04
.........................................................................................acguggucuuguccugauuuau..   1   0   S04
.........................................................................................acguggucuugGccugauuuaug.   1   1   S04
.........................................................................................acguggucuuguccugauuuaug.   3   0   S04
.........................................................................................acguggucuuguccCgauuuauga   1   1   S04
.........................................................................................acguggucuugGccugauuuauga   1   1   S04
.........................................................................................acguggucuuguccugauuuauga   3   0   S04
.........................................................................................acguggucuuguccugauuuaugG   1   1   S04
.........................................................................................acguggucuuguccugauuuaCga   1   1   S04
..........................................................................................cguggucuuguccugauu.....   1   0   S04
..........................................................................................cguggucuuguccugauuuau..   1   0   S04
..........................................................................................cguggucuuguccugauuuaug.   3   0   S04
...........................................................................................guggucuuguccugauuuaC..   1   1   S04
...........................................................................................guggucuuguccugauuuaugG   1   1   S04
...........................................................................................guggucuuguccugauuuauga   2   0   S04
```

Provisional ID	:	chrAnn_random_3081079
Score total	:	1.6
Score for star read(s)	:	-1.3
Score for read counts	:	0
Score for mfe	:	2.9
Score for randfold	:	0
Score for cons. seed	:	
Total read count	:	131
Mature read count	:	131
Loop read count	:	0
Star read count	:	0

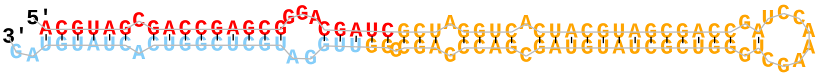

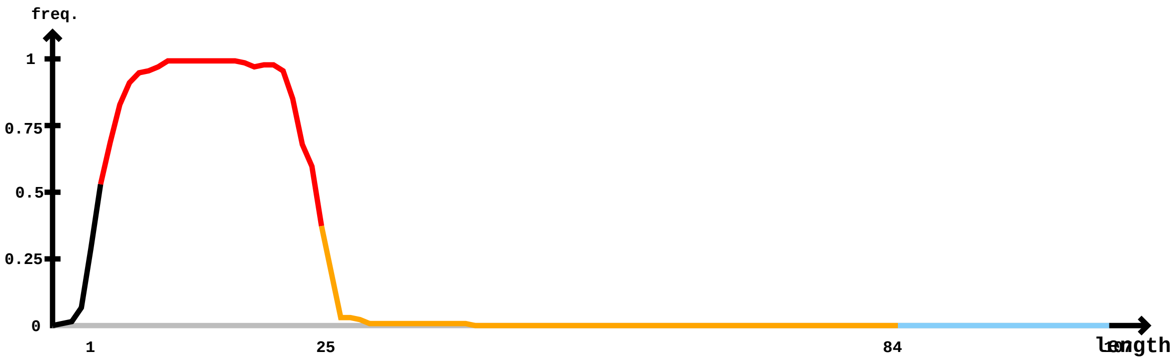

Mature Star

```
5'- gcuacguagcgaccgagcgggacgaucgcuaggucacuacguagcgaccgauccaaagcugggucgcuauguagcgaccgagcggguuggaugcucggucacuauguagcga -3'    exp
    ((((((((.((((((((...(((((((.((((.(((((((((((((.(........).))))))))))))).)))).))).)))))..)))))))).))))))))..    reads   mm   sample
    gcuacguagcgaccgagc..............................................................................................    1   0   S03
    gcuacUuagcgaccgagcg.............................................................................................    1   1   S03
    .cuacguagcgaccgagcg.............................................................................................    1   0   S03
    .cuacguagcgaccgagcgggac.........................................................................................    3   0   S03
    .cuacguagcgaccgagcgggacg........................................................................................    2   0   S03
    ..uacguagcgaccgagcgggC..........................................................................................    1   1   S03
    ..uacguagcgaccgagcgggac.........................................................................................    3   0   S03
    ..uacguagcgaccgagcgggacg........................................................................................    8   0   S03
    ..uacguagcgaccgUgcgggacg........................................................................................    1   1   S03
    ..uacguagcgaccgagcgggacgaC......................................................................................    2   1   S03
    ..uaUguagcgaccgagcgggacgau......................................................................................    2   1   S03
    ..uacguagcgaccgagcgggacgau......................................................................................    9   0   S03
    ..uacUuagcgaccgagcgggacgau......................................................................................    1   1   S03
    ...acguagcgaccgagcggga..........................................................................................    2   0   S03
    ...acguagcgaccgagcgggac.........................................................................................    2   0   S03
    ...acguagcgaccgagcgggacg........................................................................................    2   0   S03
    ...acguagcgaccgagcgggacga.......................................................................................    4   0   S03
    ...acguagcgaccgagcgggacgC.......................................................................................    1   1   S03
    ...acguagcgaccgagcgggacgau......................................................................................    1   0   S03
    ...acguagcgaccgagcgggacgauc.....................................................................................    10  0   S03
    ...acUuagcgaccgagcgggacgauc.....................................................................................    3   1   S03
    ...acguagcgaccgagcgggacgauG.....................................................................................    1   1   S03
    ....cguagcgaccgagcgggac.........................................................................................    3   0   S03
    ....cguagcgaccgagcgggacg........................................................................................    2   0   S03
    ....cguagcgaccgagcgggacC........................................................................................    1   1   S03
    ....cguagcgaccgagcgggacga.......................................................................................    2   0   S03
    ....cguagcgaccgagcgggacgau......................................................................................    5   0   S03
    ....cUuagcgaccgagcgggacgau......................................................................................    1   1   S03
    ....cguagcgaccgagcgggacgaC......................................................................................    1   1   S03
    ....cguagcgaccgagcgggacgauA.....................................................................................    1   1   S03
    ....cguagcgaccgagcgggacgauc.....................................................................................    1   0   S03
    ....cUuagcgaccgagcgggacgaucg....................................................................................    1   1   S03
    .....guagcgaccgagcgggac.........................................................................................    1   0   S03
    .....guagcgaccgagcgggacg........................................................................................    2   0   S03
```

```
   Mature                                                                                 Star
gcuacguagcgaccgagcgggacgaucgcuaggucacuacguagcgaccgauccaaagcugggucgcuauguagcgaccgagcggguuggaugcucggucacuauguagcga
.....Nuagcgaccgagcgggacg........................................................................................    1    1    S03
.....guagcgaccgagcgggacgau......................................................................................    2    0    S03
.....guagcgaccgagcgggacCau......................................................................................    1    1    S03
.....guagcgacUgagcgggacgauc.....................................................................................    1    1    S03
.....guagcgaccgagcgggacgauc.....................................................................................    4    0    S03
.....Uuagcgaccgagcgggacgauc.....................................................................................    4    1    S03
.....Uuagcgaccgagcgggacgaucg....................................................................................    2    1    S03
.....guagcgaccgagcgggacgaucg....................................................................................    1    0    S03
......uagcgaccgagcgggacg........................................................................................    2    0    S03
......uagcgaccgagcgggacga.......................................................................................    1    0    S03
......uagcgaccgagcgggacgC.......................................................................................    1    1    S03
......uagcgaccgagcgggacgauc.....................................................................................    4    0    S03
......uagcgaccgagcgggacgauG.....................................................................................    1    1    S03
......uagcgaccgagcgggacgaucg....................................................................................    2    0    S03
.......agcgaccgagcgggacgau......................................................................................    2    0    S03
........gcgaccgagcgggacgauc.....................................................................................    1    0    S03
.........cgaccgagcgggacgaucgcu..................................................................................    1    0    S03
.........cgaccgagcgggacgaucgcuG.................................................................................    1    1    S03
..........gaccgagcgggacgaucg....................................................................................    2    0    S03
..........gaccgagcgggacgaucAcua.................................................................................    1    1    S03

.cuacguagcgaccgagcgggacg........................................................................................    1    0    S04
..uacguagcgaccgagcgggacg........................................................................................    1    0    S04
..uacUuagcgaccgagcgggacga.......................................................................................    1    1    S04
..uaUguagcgaccgagcgggacgau......................................................................................    1    1    S04
...acguagcgaccgagcgggac.........................................................................................    1    0    S04
...acguagcgaccgagAgggac.........................................................................................    1    1    S04
...acguagcgaccgagcgggacgauc.....................................................................................    2    0    S04
...aUguagcgaccgagcgggacgauc.....................................................................................    1    1    S04
...acUuagcgaccgagcgggacgauc.....................................................................................    1    1    S04
....cguagcgaccgagcgggacga.......................................................................................    1    0    S04
....cguagcgaccgagcgggacgau......................................................................................    1    0    S04
....cUuagcgaccgagcgggacgau......................................................................................    1    1    S04
.......agcgaccgagcgggacgauc.....................................................................................    2    0    S04
.......agcgaccgagcgggacgaucU....................................................................................    1    1    S04
....................gacgaucgcuaggucacuacgu......................................................................    1    0    S04
```

Provisional ID	:	chrAnn_random_3083516
Score total	:	0.2
Score for star read(s)	:	-1.3
Score for read counts	:	0
Score for mfe	:	1.5
Score for randfold	:	0
Score for cons. seed	:	
Total read count	:	57
Mature read count	:	53
Loop read count	:	4
Star read count	:	0

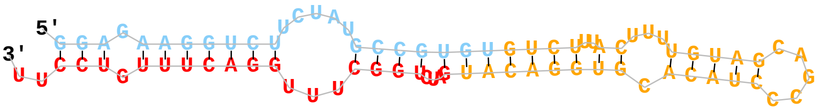

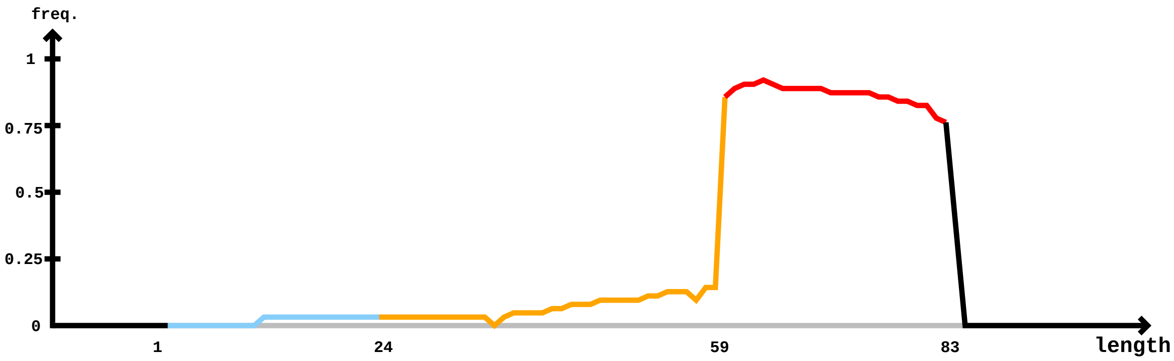

Star Mature

```
5'- gguggaggaaggagaaggucuucuaugccgugugucuuuacuuuuguagcagcccuacacguggacaugaucuggcuuuggacuuuguccuuuucuccuuuuggguuucuaa  -3'   exp
    ...((((((((((.(((((((.....(((((((((((..((...(((((.....))))).)))))))))...))))...))))))).))).)))))))..............   reads   mm   sample
    ..............................................uagcagcccuacacguggacau............................................   1       0    S03
    ....................................................cccuacacguggacaugaucG.......................................   1       1    S03
    ............................................................guggacaugaucuggcuuuggacG............................   1       1    S03
    ..................................................................augaucuggcuuuggacuuugucU......................   1       1    S03
    ..................................................................augaucuggcuuuggacuuugucc......................   1       0    S03
    ....................................................................gaucuggcuuuggacuuuguccu.....................   1       0    S03
    ....................................................................gaucuggcuuuggacuuuguccuC....................   1       1    S03
    ....................................................................gaucCggcuuuggacuuuguccuu....................   1       1    S03
    ....................................................................gaucuggcuuuggacuuuguccuu....................   22      0    S03
    .....................................................................aucuggcuuuggacuuuguccuu....................   1       0    S03

    ....................uucuaugccgugugucuuuacuuu....................................................................   2       0    S04
    .............................................guagcagcccuacacgugga...............................................   2       0    S04
    ..................................................agcccuacacguggacaugaucug......................................   1       0    S04
    .......................................................uacacguggacaugaucuggcuuu.................................   1       0    S04
    ..............................................................ggacaugaucuggcuuuggacuuu..........................   1       0    S04
    ..................................................................augaucuggcuuuggacuuugucA......................   1       1    S04
    ....................................................................gaucuggcuuuggacuuugu........................   1       0    S04
    ....................................................................gaucuggcuuuggacuuuguccuC....................   2       1    S04
    ....................................................................gaucuggcuuuggacuuuguccuu....................   15      0    S04
    ....................................................................gaucuggcuuuggacuuCguccuu....................   1       1    S04
    ....................................................................gaucuggcuuuggacuuuguccCu....................   1       1    S04
    ....................................................................gaucuggcuuuggacuuuguccAuu...................   1       1    S04
    .....................................................................aucuggcuuuggacuuuguccuC....................   1       1    S04
    ......................................................................ucuggcuuuggacuuuguccuu....................   1       0    S04
    ........................................................................uggcuuuggacuuugCccuu....................   1       1    S04
```

Provisional ID	:	chrAnn_random_3091761
Score total	:	0.6
Score for star read(s)	:	-1.3
Score for read counts	:	0
Score for mfe	:	1.9
Score for randfold	:	0
Score for cons. seed	:	
Total read count	:	63
Mature read count	:	63
Loop read count	:	0
Star read count	:	0

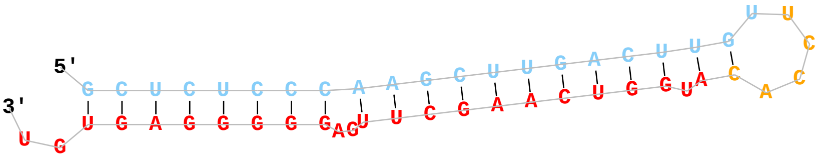

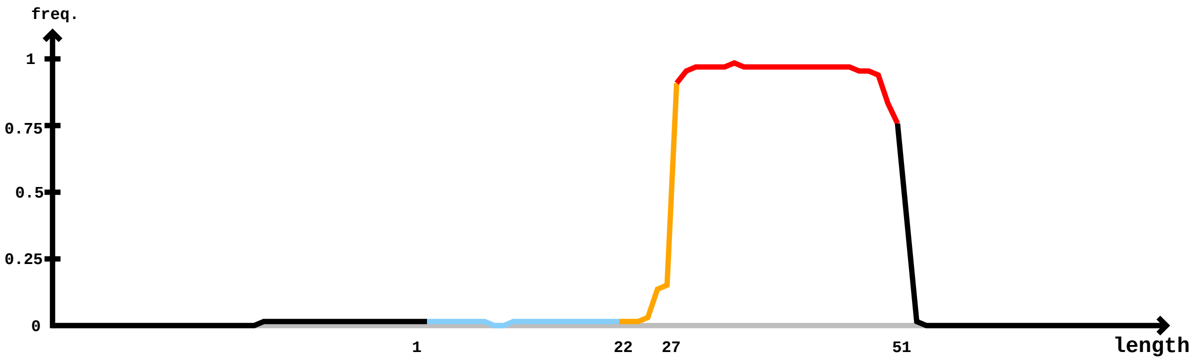

Star Mature

```
5'- aaaugcugcaagggucugauggugugaucaugaagcugcucucccaagcuugacuuguuccacauggucaagcuugagggggaguguugagaagacuucaaaugaucacaaagg   -3'   exp
    ......................(((((((((((((((((((((((((((((((((((.....)).))))))))))..))))))))).......)).)))))..))))))))....    reads   mm   sample
    ....................ggugugaucaugaagcugcucucc......................................................................    1       0    S03
    ..............................................................cauggucaagcuugagggggagug............................    1       0    S03
    ...............................................................auggucaagcuugagggggaguA............................    1       1    S03
    ...............................................................auggucaagcuugagggggagug............................    1       0    S03
    ...............................................................auggucaagcuugagggggagugC...........................    1       1    S03
    ...............................................................auggucaagcuugGgggggagugu...........................    2       1    S03
    ...............................................................auggucaagcuugagggggagugu...........................    11      0    S03
    ................................................................uggucaagcuugaggggg................................    1       0    S03

    ..............................................agcuugacuuguuccacaugguca............................................    1       0    S04
    ............................................................cacauggucaagcuugaggggag..............................    1       0    S04
    .............................................................acauUgucaagcuugagggggagu.............................    1       1    S04
    .............................................................acauggucaagcuugagggggagu.............................    6       0    S04
    ...............................................................auggucaagcuugagggggagug............................    2       0    S04
    ...............................................................auggucaagcuugagggggagugA...........................    1       1    S04
    ...............................................................auggucaagcuugUgggggagugu...........................    2       1    S04
    ...............................................................auggucaagcuugagggggagugG...........................    3       1    S04
    ...............................................................auggucaagcuugagggggagugC...........................    1       1    S04
    ...............................................................auggucaagcuugagggggagugu...........................    25      0    S04
    ................................................................uggucaagcuugagggggagugA...........................    1       1    S04
    ................................................................uggucaagcuugagggggagugu...........................    1       0    S04
    .................................................................ggucaagcCugagggggaguguug.........................    1       1    S04
    .....................................................................aagcuugagggggagugu...........................    1       0    S04
```

Provisional ID	: chrAnn_random_3097010
Score total	: 0.7
Score for star read(s)	: -1.3
Score for read counts	: 0
Score for mfe	: 2
Score for randfold	: 0
Score for cons. seed	:
Total read count	: 12
Mature read count	: 12
Loop read count	: 0
Star read count	: 0

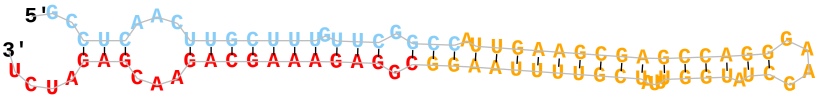

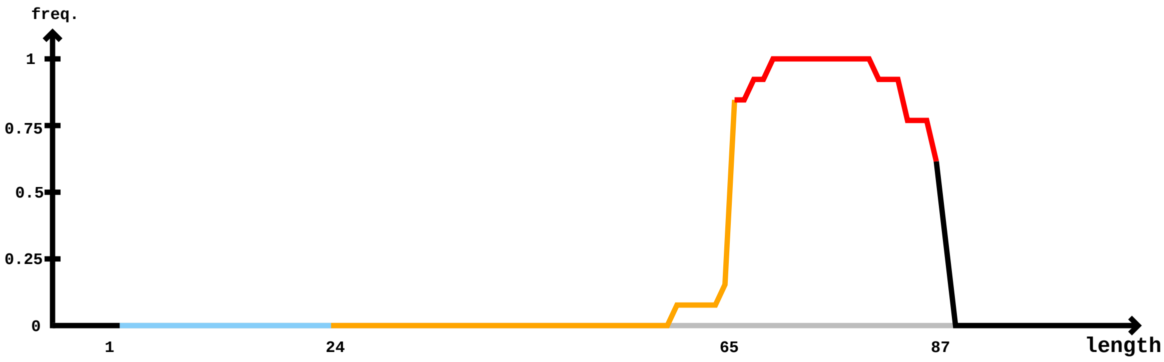

Star Mature

```
5'- gcagggccucaacuugcuuuguucggccauugaagcgagccagggaagcuaugguuuaucguuuuaaggcggagaaagcagaacgagaucuugcucgcgugcaaggcgaga  -3'   exp
    (((((..(((...(((((((.(((.(((.(((((((((((((((....)).))))...)))))))))))).))))))))))...)))..)))))((((.......))))..   reads   mm   sample
    ................................................................uuaagUcggagaaagcagaac..........................   1       1    S03
    ....................................................................gcggagaaagcagaacgag........................   1       0    S03
    .....................................................................cggagaaagcagaacgag........................   1       0    S03
    .....................................................................cggagaaagcagaacgagauc.....................   1       0    S03
    .....................................................................cggagaaagcagaacgagaucu....................   2       0    S03

    .....................................................................cggagaaagcagaacgagauc.....................   1       0    S04
    .....................................................................cggagaaagcagaacgaUaucu....................   1       1    S04
    .....................................................................cggagaaagcagaacgagaucG....................   1       1    S04
    .....................................................................cggagaaagcagaacgagaucC....................   1       1    S04
    .....................................................................cggagaaagcagaacgagaucu....................   1       0    S04
    .......................................................................gagaaagcagaacgagaucuu...................   1       0    S04
    .........................................................................gaaagcagaacgagaucu....................   1       0    S04
```

Provisional ID	:	chrAnn_random_3102718
Score total	:	0.2
Score for star read(s)	:	-1.3
Score for read counts	:	0
Score for mfe	:	1.5
Score for randfold	:	0
Score for cons. seed	:	
Total read count	:	27
Mature read count	:	27
Loop read count	:	0
Star read count	:	0

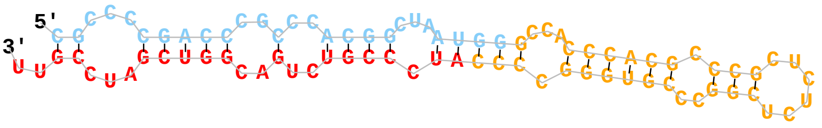

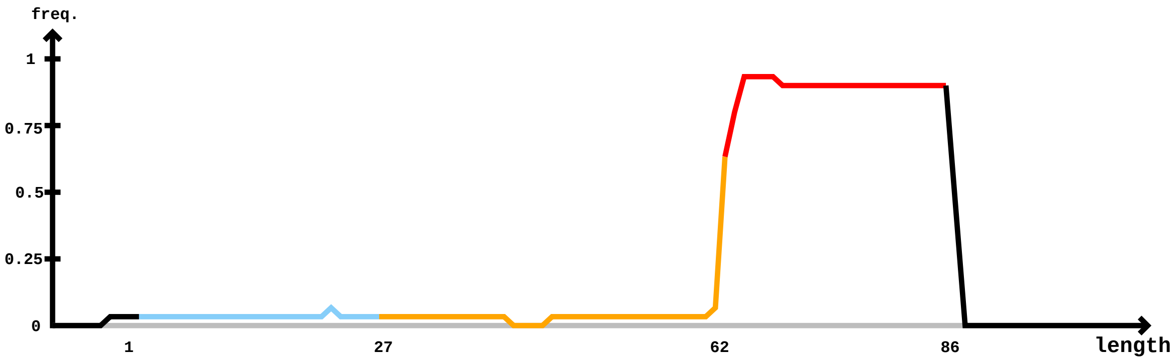

Star Mature

```
5'- ccuguaacgccccgacccgcccacggcuaaugggccacccacgcccgcucucucggcccgugggccccaucccgucugacggucgauccguuaauuuuccaaaggcucgaaa  -3'   exp
    (((.(((((...(((((..(..((((...(((((...((((((.(((......)))..)))))).))))).))))..)..)))))...))))).........)))....... reads   mm   sample
    ....................................................................aucccgucugacggucgauccguu.................... 8       0    S03
    ....................................................................aucccgucugacggucgauccguC.................... 1       1    S03
    ....................................................................aucccgucugacggucgauccguG.................... 1       1    S03
    .....................................................................ucccgucugacggucgauccguu.................... 1       0    S03
    .....................................................................ucccgucugacggucgauccguua................... 1       0    S03
    ......................................................................cccgucugacggucgauccguua................... 1       0    S03

    ....uaacgccccgacccgcccacggcC.................................................................................... 1       1    S04
    ...........................uaaugggccacccacgccG.................................................................. 1       1    S04
    ..................................................ucucggccUgugggccccaucccg...................................... 1       1    S04
    ...................................................................caucccgucugacggucgauccguu.................... 1       0    S04
    ....................................................................aucccgucugacggucgauccguC.................... 3       1    S04
    ....................................................................aucccgucugacggucgauccguu.................... 4       0    S04
    .....................................................................ucccgucugacggucgauccguu.................... 3       0    S04
    ......................................................................cccgucugacggucgauccguu.................... 1       0    S04
    ......................................................................cccgucugacggucgauccguuU................... 1       1    S04
    ......................................................................cccgucugacggucgauccguua................... 1       0    S04
```

Provisional ID	:	chrAnn_random_3107930
Score total	:	1.1
Score for star read(s)	:	-1.3
Score for read counts	:	0
Score for mfe	:	2.4
Score for randfold	:	0
Score for cons. seed	:	
Total read count	:	172
Mature read count	:	164
Loop read count	:	0
Star read count	:	8

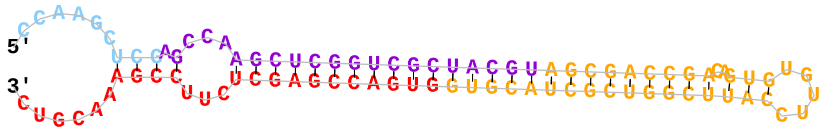

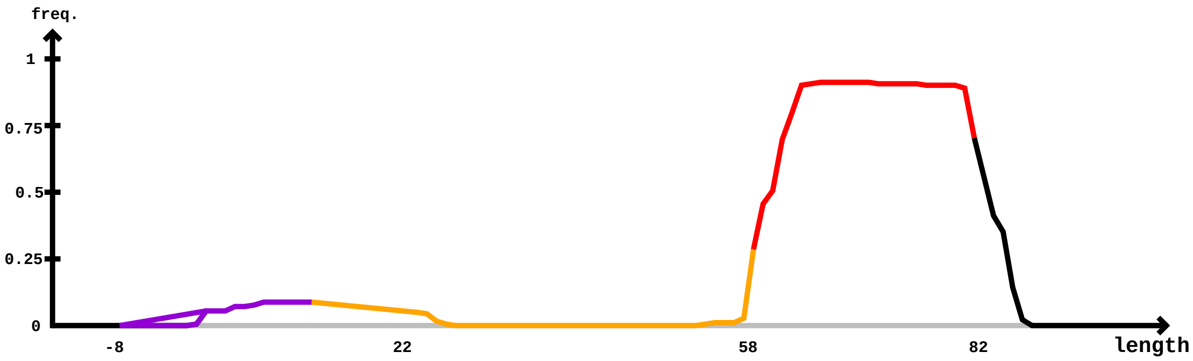

Star **Mature**

```
5'- agcgaccaagcucgagccaagcucggucgcuacguagcgaccgacaguguguuccauucggucgcuacguggugaccgagcucuuccgaaacgucgauaugacacgaauccaug  -3'   obs
    agcgaccaagcucgagccaagcucggucgcuacguagcgaccgacaguguguuccauucggucgcuacguggugaccgagcucuuccgaaacgucgauaugacacgaauccaug         exp
    ..((((.....(((.(...(((((((((((((((((((((((((..(((.....))))))))))))))))))))))))))))...))))...)))).................. reads     mm     sample
    .............gagccaagcucggucgcuacgua..............................................................................  1         0      S03
    ..............agccaagcucggucgcuacg................................................................................  1         0      S03
    ..............agccaagcucggucgcuacgu...............................................................................  2         0      S03
    ..............agccaagcCcggucgcuacgu...............................................................................  1         1      S03
    ..............agccaagcucggucgcuacgua..............................................................................  1         0      S03
    ..............agccaagcucggucgcuacguagG............................................................................  1         1      S03
    ..............agccaagcucggucgcuacguagc............................................................................  1         0      S03
    ..............agccaagcucggucgcuacguagcgU..........................................................................  1         1      S03
    .................caagcucggucgcuacgG...............................................................................  1         1      S03
    .................caagcucggucgcuacguag.............................................................................  1         0      S03
    .................caagcucggucgcuacguagcg...........................................................................  1         0      S03
    ...................agcucggucgcuacguagc............................................................................  1         0      S03
    ....................gcucggucgcuacguagc............................................................................  1         0      S03
    ...................................................................cguggugaccgagcucuuccga.........................  1         0      S03
    ......................................................................Ugugaccgagcucuuccgaaacgu....................  1         1      S03
    ......................................................................ggCgaccgagcucuuccgaaacgu....................  1         1      S03
    ......................................................................ggugaccgagcucuuccgaaacgu....................  1         0      S03
    .......................................................................gugaccgagcucuuccgaaacgC....................  3         1      S03
    .......................................................................gugaccgagcucuuccgaaacgu....................  10        0      S03
    .......................................................................gugaccgaAcucuuccgaaacguc...................  1         1      S03
    .......................................................................gugaccgagcucuuccgaaacguc...................  30        0      S03
    .......................................................................gugaccgagcAcuuccgaaacguc...................  1         1      S03
    .......................................................................gugaccgagcucuuccgaaacguA...................  2         1      S03
    ........................................................................ugaccgagcuUuuccgaaacgu....................  1         1      S03
    ........................................................................ugaccgagcucuuccgaaacgu....................  11        0      S03
    ........................................................................ugaccgagcucCuccgaaacgu....................  2         1      S03
    ........................................................................ugaccgagcucuuccgaaacgC....................  3         1      S03
    ........................................................................ugaccgCgcucuuccgaaacgu....................  1         1      S03
    ........................................................................ugaccgagcucuuccgaaacguA...................  2         1      S03
    ........................................................................ugaccgagcucuuccgaaacguc...................  1         0      S03
    ........................................................................ugaccgagcucuuccgaaacguU...................  1         1      S03
    ........................................................................ugaccCagcucuuccgaaacguc...................  1         1      S03
    ........................................................................ugaccgagcucuuccgaaacguUg..................  1         1      S03
```

```
              Star                                                         Mature
agcgaccaagcucgagccaagcucggucgcuacguagcgaccgacaguguguuccauucggucgcuacguggugaccgagcucuuccgaaacgucgauaugacacgaauccaug
........................................................................ugaccUagcucuuccgaaacgucg..................   1    1    S03
........................................................................ugaccgagcucuuccgaaacgucg..................   6    0    S03
.........................................................................gaccgaAcucuuccgaaacgucga.................   1    1    S03
.........................................................................gaccgagcucuuccgaaacgucgC.................   1    1    S03
.........................................................................gaccgagcucuuccgaaacgucga.................   4    0    S03
..........................................................................accgagcucuuccgaaacgucg..................   2    0    S03
..........................................................................accgagcCcuuccgaaacgucgau................   1    1    S03
..........................................................................accgaCcucuuccgaaacgucgau................   1    1    S03
..........................................................................accgagcucuuccgaaacgucgaA................   1    1    S03
..........................................................................accgagcucuuccgaaacgucgaC................   2    1    S03
..........................................................................accgagcucuuccgaaacgucgau................   19   0    S03
..........................................................................accgagcucuuccgaaacgucgaua...............   1    0    S03
...........................................................................ccgagcucuuccgaaacg.....................   1    0    S03
...........................................................................ccgagcucuuccgaaacgucga.................   2    0    S03
...........................................................................ccgagcucuuccgaaacgucgaC................   1    1    S03
...........................................................................ccgagcucuuccgGaacgucgau................   1    1    S03
...........................................................................ccgagcucuuccgaaacgucgau................   2    0    S03
...........................................................................ccgagcucuuccgaaacgucgaua...............   9    0    S03
............................................................................cgagcucuuccgaaacgucga.................   1    0    S03
............................................................................cgagcucuuccgaaacgucgau................   4    0    S03
............................................................................cgagcucuuccgaaacgucgaA................   1    1    S03
............................................................................cgagcucuuccgaaacgucgaua...............   7    0    S03
............................................................................cgagcucuuccgaaacgucgauau..............   4    0    S03
.............................................................................gagcucuuccgaaacgucgaua...............   1    0    S03
..............................................................................agcucuuccgaaacgucgaua...............   1    0    S03

..............agccaagcucggucgcuacguagc............................................................................   1    0    S04
....................gcucggucgcuacguagcg...........................................................................   1    0    S04
..................................................................acguggCgaccgagcucu..............................   1    1    S04
.........................................................................gaccgagcucuuccgaaacgucg..................   1    0    S04
.........................................................................gaccgagcucuuccgaaacgucga.................   2    0    S04
..........................................................................accgagcucuuccgaaacg.....................   1    0    S04
..........................................................................accgaAcucuuccgaaacguc...................   1    1    S04
..........................................................................accgagcucuuccgaaacguc...................   2    0    S04
..........................................................................accgagcucuuccgaaacgucgau................   4    0    S04
...........................................................................ccgagcucuuccgaaacgucgau................   1    0    S04
...........................................................................ccgagcucuuccgaaacgucgauU...............   1    1    S04
............................................................................cgagcucuuccgaaacgucgaua...............   2    0    S04
```

Provisional ID	:	chrAnn_random_3108883
Score total	:	1.4
Score for star read(s)	:	3.9
Score for read counts	:	-3.3
Score for mfe	:	1
Score for randfold	:	0
Score for cons. seed	:	
Total read count	:	5
Mature read count	:	4
Loop read count	:	0
Star read count	:	1

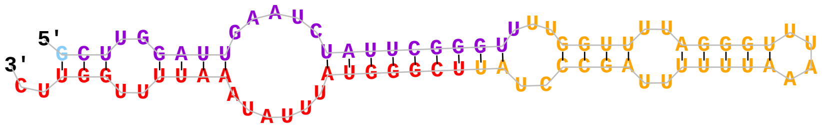

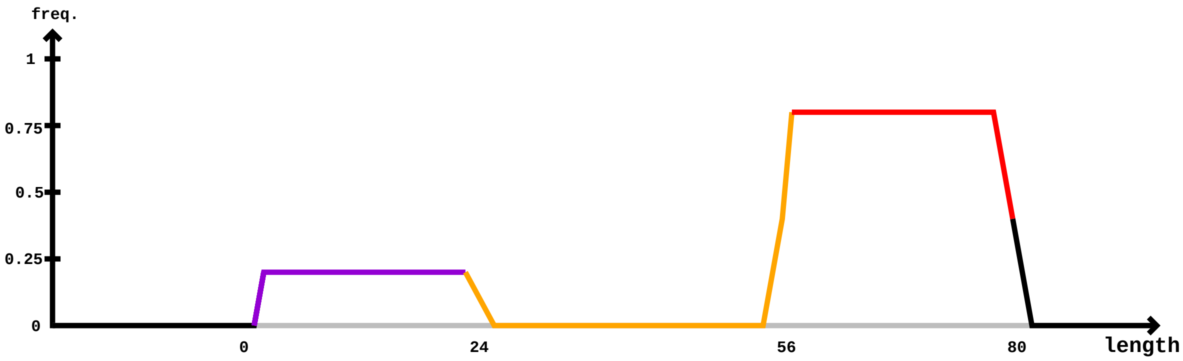

Star Mature

```
5'- ggauacccauuuggguuuggcuuggauugaaucuauucggguuuugguuuuaggguuuaaauuuuuuagcccuauucggguauuuauaaauuuugguucagaucuuugugggu   -3'   obs
    ggauacccauuuggguuuggcuuggauugaaucuauucggguuuugguuuuaggguuuaaauuuuuuagcccuauucggguauuuauaaauuuugguucagaucuuugugggu         exp
    ....((((((..((((((((((..((((.....(((((((((...((((..(((((....)))))..))))..)))))))))......))))..)).))))))))..))))))   reads   mm   sample
    ...........................................................................ucggguauuuauaaauuuugguuc..............   2       0    S03

    ....................cuuggauugaaucuauucggguu......................................................................   1       0    S04
    .........................................................................auucggguauuuauaaauuuuggu................   1       0    S04
    ..........................................................................uucggguauuuauaaauuuugguu...............   1       0    S04
```

Provisional ID	:	chrAnn_random_3109904
Score total	:	0
Score for star read(s)	:	3.9
Score for read counts	:	-4.9
Score for mfe	:	1
Score for randfold	:	0
Score for cons. seed	:	
Total read count	:	2
Mature read count	:	1
Loop read count	:	0
Star read count	:	1

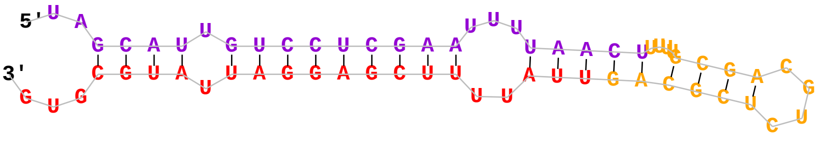

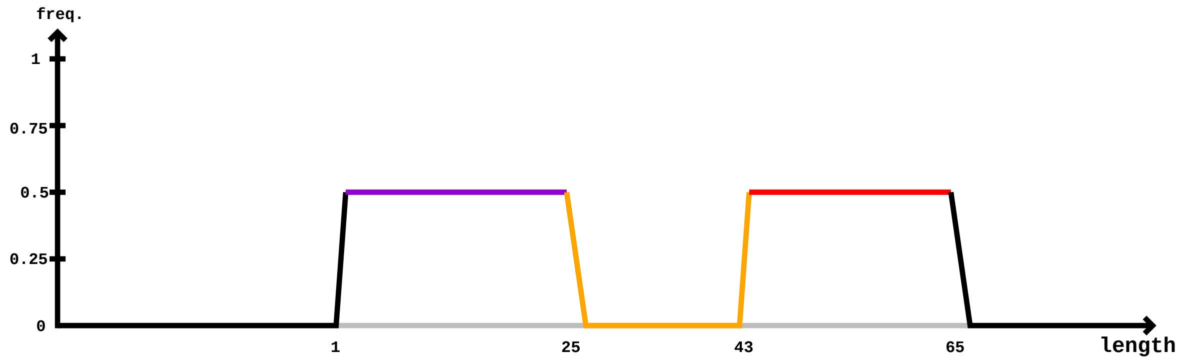


```
                                 Star                                       Mature
5'- nnnnnnnnnnnnguucggacaauuuguuuagcauuguccucgaauuuuaacuuuuugcgacgucucgcaguuauuuucgaggauuaugcguguuugaaaggugauaaucuuu   -3'    obs
    nnnnnnnnnnnnguucggacaauuuguuuagcauuguccucgaauuuuaacuuuuugcgacgucucgcaguuauuuucgaggauuaugcguguuugaaaggugauaaucuuu          exp
    .............((((((((.........((((.(((((((((...(((((....((((....)))))))))..))))))))).)))).))))))))..............    reads   mm    sample
    ............................uagcauuguccucgaauCuuaacu............................................................    1       1     S04
    ......................................................................uuauuuucgaggauuaugcgug....................    1       0     S04
```

Provisional ID	: chrAnn_random_3117276
Score total	: 1.8
Score for star read(s)	: -1.3
Score for read counts	: 0
Score for mfe	: 3.1
Score for randfold	: 0
Score for cons. seed	:
Total read count	: 49
Mature read count	: 49
Loop read count	: 0
Star read count	: 0

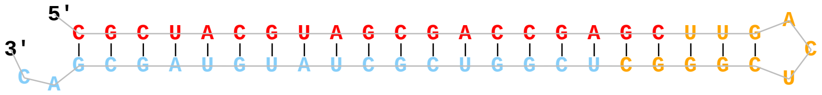

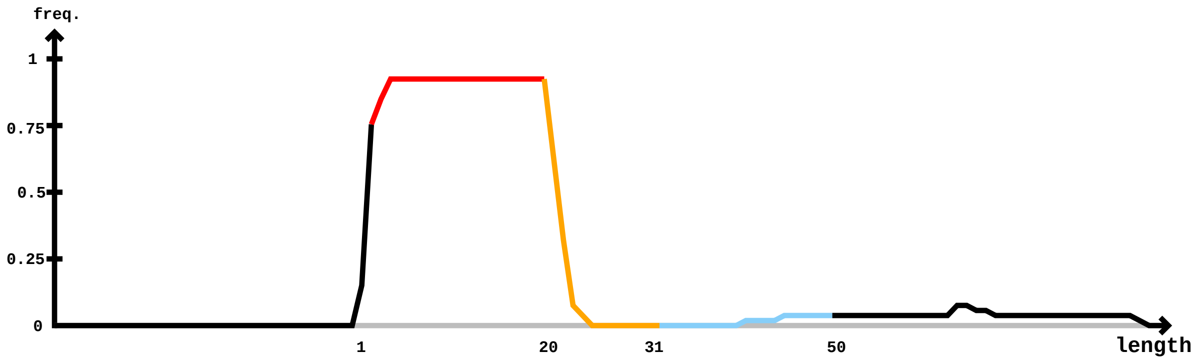

Mature Star

```
5'- gcguagcgaccuagcgggacguacgcucgaucgcuacguagcgaccgagcuugacucgggcucggucgcuauguagcgaccgagcggacggacgcucggucgcuaaguagcgac   -3'    exp
    ((.((((((((.((((...(((.((((((.(((((((((((((((((((((((...))))))))))))))))))))))).)))))).))).)))).))))))))....))...   reads      mm         sample
    ..............................ucgcuacguagcgaccgagc................................................................   3          0          S03
    ..............................ucgcuacguagcgaccgagcu...............................................................   2          0          S03
    ..............................Ccgcuacguagcgaccgagcuu..............................................................   1          1          S03
    ...............................cgcuacguagcgaccgagc................................................................   17         0          S03
    ...............................cgcuacguagcgaccgagcu...............................................................   2          0          S03
    ...............................cgcuacguagcgCccgagcu...............................................................   1          1          S03
    ...............................cgcuacguagcgaccgagcuu..............................................................   7          0          S03
    ...............................cgcuacguagcgaccgagcuug.............................................................   2          0          S03
    ................................gcuacguagcgaccgagc................................................................   1          0          S03
    ................................gcuacguagcgaccgagcuu..............................................................   3          0          S03
    .................................cuacguagcgaccgagcu...............................................................   2          0          S03
    .................................cuacguagUgaccgagcuuga............................................................   2          1          S03
    ............................................................................................cgcucggucgcuaaguagc...   1          0          S03
    ............................................................................................cgcucggucgcuaaguagcA..   1          1          S03

    ..............................ucgcuacguagcgaccgagc................................................................   1          0          S04
    ..............................ucgcuacguagcgaccgagcu...............................................................   1          0          S04
    ...............................cgcuacguagcgaccgagc................................................................   1          0          S04
    ...............................cgcuacguagcgaccgagcu...............................................................   1          0          S04
    ...............................cgcuacguagcgaccgagcuu..............................................................   1          0          S04
    ................................gcuacguagcgaccgagcuu..............................................................   1          0          S04
    ......................................................................auguagcgaccgagcggacggacg....................   1          0          S04
    ..........................................................................agcgaccgagcggacgAacgcu..................   1          1          S04
```

Provisional ID	:	chrAnn_random_3117543
Score total	:	0.5
Score for star read(s)	:	-1.3
Score for read counts	:	0
Score for mfe	:	1.8
Score for randfold	:	0
Score for cons. seed	:	
Total read count	:	104
Mature read count	:	104
Loop read count	:	0
Star read count	:	0

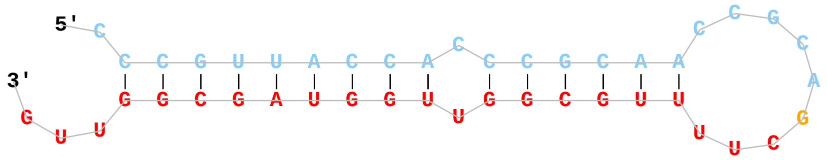

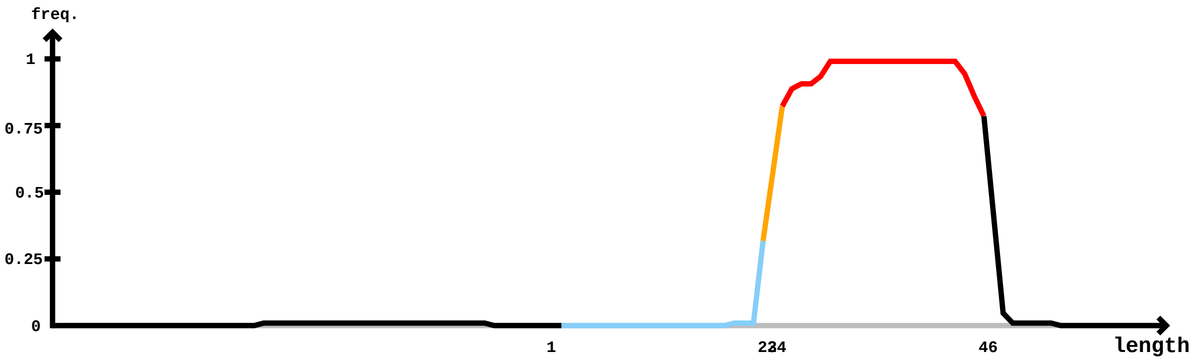

Star **Mature**

```
5'- uuuuauaaauagcuaccaaagcaauuugugacuaaggaguugauugguuuucccguuaccacccgcaaccgcagcuuuugcgguugguagcgguugauggccuuucaaaacaau   -3'   exp
    ..((((((((.(((.....))).))))))))....(...((((..(((..((((((((((.((((((.........)))))).))))))))))..))..)))..))))..)...   reads  mm  sample
    ....................gcaauuugugacuaaggaguugau......................................................................   1      0   S03
    ........................................................................agcuuuugcgguugguagcggu....................   3      0   S03
    ........................................................................agcuuuugcgguugguagcgguug..................   9      0   S03
    ........................................................................agcuuuugcgguugguagcgUuug..................   1      1   S03
    .........................................................................gcuuuugcgguugguagcgg.....................   1      0   S03
    .........................................................................gcuuuugcgguugguagcggu....................   1      0   S03
    .........................................................................gcuuuugcgguugguagcgguug..................   4      0   S03
    .........................................................................gcuuuugcgguuUguagcgguuga.................   1      1   S03
    .........................................................................gcuuuugcgguugguagcgguuga.................   11     0   S03
    ..........................................................................cuuuugcgguugguagcggu....................   1      0   S03
    ..........................................................................cuuuugcgguugguagcggC....................   1      1   S03
    ..........................................................................cuuuugcgguugguagcgguu...................   2      0   S03
    ..........................................................................cuuuugcgguugguagcgguug..................   14     0   S03
    ..........................................................................cuuuugcgguugguagcgguugau................   1      0   S03
    ...........................................................................uuuugcgguugguagcgg.....................   2      0   S03
    ...........................................................................uuuugcgguugguagcggu....................   1      0   S03
    ...........................................................................uuuugcgguugguagcgguu...................   1      0   S03
    ...........................................................................uuuugcgguugguagcgguug..................   1      0   S03
    ............................................................................uuugcgguugguagcgguug..................   1      0   S03
    ..............................................................................ugcgguugguagcgguug..................   1      0   S03
    ..............................................................................ugcgguugguagcgguugau................   1      0   S03
    ...............................................................................gcgguugguagcgguuga.................   2      0   S03
    ...............................................................................gcgguugguagcgguugCuggccu...........   1      1   S03

    .....................................................................cgcagcuuuugcgguugguagcgg.....................   1      0   S04
    ........................................................................agcuuuugcgguugguagcggu....................   1      0   S04
    ........................................................................agcuuuugcgguugguagcgguu...................   5      0   S04
    ........................................................................agcuuuugcgguugguagcgguug..................   13     0   S04
    ........................................................................agcuuuugcgguugguagcgguuga.................   1      0   S04
    .........................................................................gcuuuugcgguugguagcgguug..................   1      0   S04
    .........................................................................gcuuuugcgguugguagcgguuga.................   12     0   S04
    .........................................................................gcuuuugcgguugguagcggGuga.................   1      1   S04
    ..........................................................................cuuuugcgguugguagcgguug..................   2      0   S04
    ..........................................................................cuuuAgcgguugguagcgguugau................   1      1   S04
```

Star Mature

```
uuuuauaaaauagcuaccaaagcaauuugugacuaaggaguugauugguuuucccguuaccacccgcaaccgcagcuuuugcgguugguagcgguugauggccuuucaaaacaau
............................................................................uuuugcgguugguagcgg.....................     1     0     S04
............................................................................uuuugcgguugguagcgguug..................     1     0     S04
.............................................................................uuugcgguugguagcggu....................     1     0     S04
...............................................................................ugcgguugguagcgguug..................     1     0     S04
................................................................................gcgguugguagcgguuga.................     2     0     S04
................................................................................gcgguugguagcgguugau................     1     0     S04
```

Provisional ID	:	chrAnn_random_3127320
Score total	:	27.8
Score for star read(s)	:	3.9
Score for read counts	:	25.1
Score for mfe	:	-1.1
Score for randfold	:	0
Score for cons. seed	:	
Total read count	:	61
Mature read count	:	59
Loop read count	:	0
Star read count	:	2

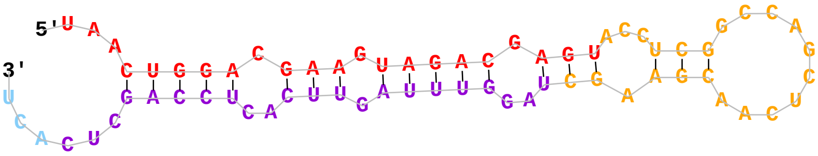

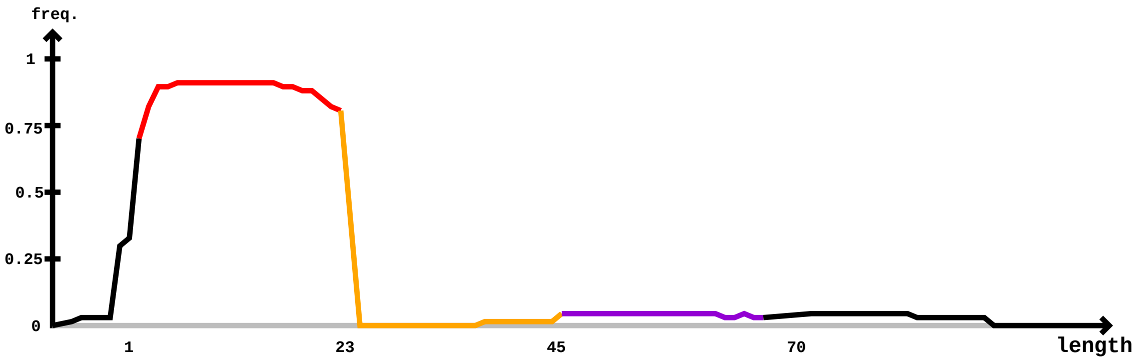

Mature **Star**

```
5'- aagacgguaacuggacgaaguagacgaguaccucggccagcucaacgaagcuagguuuaguucacuccagcucacucccuacuaagucagcuccacuagcuggacuac  -3'   obs
    aagacgguaacuggacgaaguagacgaguaccucggccagcucaacgaagcuagguuuaguucacuccagcucacucccuacuaagucagcuccacuagcuggacuac         exp
    .((..(((..(((((.(((.(((((.(((...(((..........))).)))..))))).)))..)))))...)))..))....((((((((.....)))).))))..   reads    mm    sample
    .....gguaacuggacgaaguagacgagu...............................................................................   4        0     S03
    .......uaacuggacgaaguagacgagG...............................................................................   1        1     S03
    .......uaacuggacgaaguagacgagu...............................................................................   4        0     S03
    ........aacuggacgaaguagacgGgu...............................................................................   1        1     S03
    ........aacuggacgaaguagacgagu...............................................................................   2        0     S03
    .........acuggacgaaguagacgagC...............................................................................   1        1     S03
    ...........uggacgaaguagacgagu...............................................................................   1        0     S03
    .............................................................................ccuacuaagucagcuccaA............   1        1     S03

    aagacgguaacuggacgaagua......................................................................................   1        0     S04
    .agacgguaacuggacgaaguaga....................................................................................   1        0     S04
    .....gguaacuggacgaaguagacg..................................................................................   1        0     S04
    .....gguaacuggacgaaguagacga.................................................................................   1        0     S04
    .....gguaacuggacgaaguagacgagu...............................................................................   11       0     S04
    .....gguaacuggacgaaguagacgagA...............................................................................   1        1     S04
    ......guaacuggacgaaguagacgagu...............................................................................   2        0     S04
    .......uaacuggacgaaguagacgU.................................................................................   1        1     S04
    .......uaacuggacgaaguagacgag................................................................................   1        0     S04
    .......uaacuggacgaaguagGcgagu...............................................................................   1        1     S04
    .......uaacuggacgaaguagacgagG...............................................................................   1        1     S04
    .......uaacuggacgaaguagacgagC...............................................................................   2        1     S04
    .......uaacuggacgaaguagacgagu...............................................................................   13       0     S04
    .......uaacugCacgaaguagacgagu...............................................................................   1        1     S04
    ........aacuggacgaaguagacg..................................................................................   1        0     S04
    ........aacuggacgaUguagacgagu...............................................................................   1        1     S04
    ........aacuggacgaaguagacgagu...............................................................................   3        0     S04
    .........acuggacgaaguagacgagu...............................................................................   2        0     S04
    .........acugAacgaaguagacgagu...............................................................................   1        1     S04
    .........acuggacgaaguagacgagC...............................................................................   1        1     S04
    ...........................................aacgaagUuagguuuaguucacucc........................................   1        1     S04
    ...................................................uagguuuaguucacuccagG.....................................   1        1     S04
    ...................................................uagguuuaguucacuccagcuc...................................   1        0     S04
    ......................................................................cucacucccuacuaaguc....................   1        0     S04
```

Mature Star

aagacgguaacuggacgaaguagacgaguaccucggccagcucaacgaagcuagguuuaguucacuccagcucacucccuacuaagucagcuccacuagcuggacuac

....ucccuacuaagucagcuccac............ 1 0 S04

Provisional ID	:	chrAnn_random_3130007
Score total	:	200.8
Score for star read(s)	:	3.9
Score for read counts	:	204.1
Score for mfe	:	-7.1
Score for randfold	:	0
Score for cons. seed	:	
Total read count	:	412
Mature read count	:	410
Loop read count	:	0
Star read count	:	2

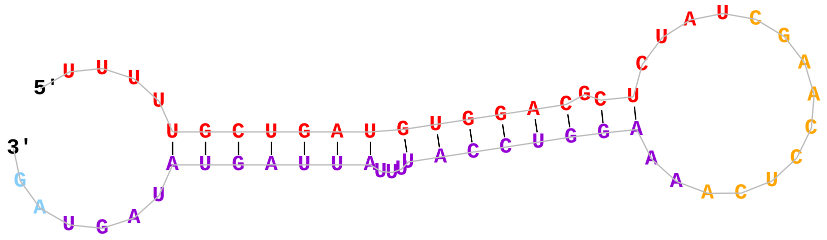

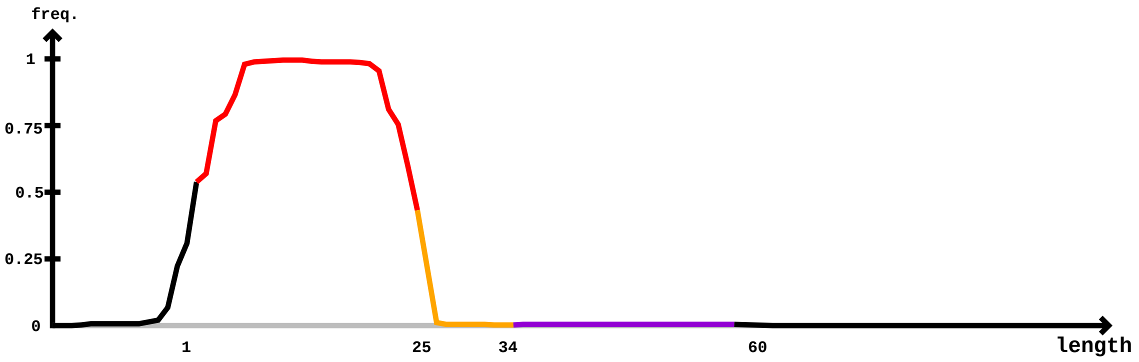

Mature **Star**

```
5'- ucgggagaaauuuuuuugcugauguggacgcucuaucgaaccucaaaagguccauuuuauuaguauaguaguaaucuuacauauuuuuaauguaguauuuccauuuu   -3'   obs
    ucgggagaaauuuuuuugcugauguggacgcucuaucgaaccucaaaagguccauuuuauuaguauaguaguaaucuuacauauuuuuaauguaguauuuccauuuu         exp
    ...(((((.........(((((((((((((.((...............))))))))...)))))))...........((((((.......))))))..)))))..... reads  mm  sample
    ..gAgagaaauuuuuuuugcugaug...................................................................................  1     1   S03
    ........aauuuuuuuugcugauguggacgc............................................................................  1     0   S03
    ........aauuuuuuCugcugauguggacgc............................................................................  1     1   S03
    .........auuuuuuuugcugauguggacgcA...........................................................................  1     1   S03
    .........auuuuuuCugcugauguggacgcu...........................................................................  1     1   S03
    ..........uuuuuuuugcuAauguggacgc............................................................................  1     1   S03
    ..........uuuuuuuugcugauguggacgc............................................................................  4     0   S03
    ..........uuuuuuuugcugaCguggacgcu...........................................................................  1     1   S03
    ..........uuuuuuuugcugauguggacgcu...........................................................................  2     0   S03
    ..........uuuuuuuugcugauguggacgcuc..........................................................................  1     0   S03
    ...........uuuuuuugcugauguggac..............................................................................  1     0   S03
    ...........uuuuuuugcugauguggacgc............................................................................  2     0   S03
    ...........uuuuuuugcugauguggacgcu...........................................................................  21    0   S03
    ...........uuuuuuugcugauguggacgcG...........................................................................  2     1   S03
    ...........uuuuuuugcuAauguggacgcu...........................................................................  4     1   S03
    ...........uuuuuuugcugauguggacgcuc..........................................................................  1     0   S03
    ...........uuuuuuugcugauguUgacgcucu.........................................................................  1     1   S03
    ...........uuuuuuugcugaCguggacgcucu.........................................................................  3     1   S03
    ...........uuuuuuugcugauguggacgcucu.........................................................................  7     0   S03
    ............uuuuuugcugauguggacgcu...........................................................................  4     0   S03
    ............uuuuuugcugauguggacgcuc..........................................................................  4     0   S03
    ............uuuGuugcugauguggacgcuc..........................................................................  1     1   S03
    ............uuuuuugcugauguggacgcucG.........................................................................  2     1   S03
    ............uuuuuugcugaugAggacgcucu.........................................................................  1     1   S03
    ............uuuuuugcugauguggacgcucu.........................................................................  3     0   S03
    ............uuuuuugcugauguggacgcucua........................................................................  1     0   S03
    ............uuuuuugcugauguggacgcucGa........................................................................  1     1   S03
    .............uuuuugcuAauguggacgcu...........................................................................  2     1   S03
    .............uuuuugcugauguggacgcu...........................................................................  2     0   S03
    .............uuuuugcugauguggacgcG...........................................................................  1     1   S03
    .............uuuuugcugauguggacgcuc..........................................................................  3     0   S03
    .............uuuuugcugauguggacgcuA..........................................................................  1     1   S03
    .............uuuuugcugauguggacgcucu.........................................................................  5     0   S03
```

```
             Mature                          Star
ucgggagaaauuuuuuuugcugauguggacgcucuaucgaaccucaaaagguccauuuuauuaguauaguaguaaucuuacauauuuuaaguaguauuuccauuuu
.............uuuuugcugauguggacgcucG.......................................................................   1   1   S03
.............uuuuugcugauguggacgcucGa......................................................................   1   1   S03
.............uuuuugcugauguggacgcucua......................................................................   11  0   S03
.............uuuuugcugauguggacgcucuU......................................................................   1   1   S03
.............uuuuugcugauCuggacgcucuau.....................................................................   1   1   S03
.............uuuCugcugauguggacgcucuau.....................................................................   1   1   S03
.............uuuuugcugauguggacgcucuau.....................................................................   26  0   S03
.............uuuuugcugauguggacUcucuau.....................................................................   1   1   S03
.............uuuuugcugauguggacgcucuaA.....................................................................   1   1   S03
.............uuuuCgcugauguggacgcucuauc....................................................................   1   1   S03
.............uuuuugcugauguggacgcucuauc....................................................................   2   0   S03
.............uuuuugcugauguggacgcucuauU....................................................................   2   1   S03
..............uuuugcugauguggacgcu.........................................................................   2   0   S03
..............uuuugcugauguggacgcucua......................................................................   3   0   S03
..............uuuugcugauguggacgcucuaA.....................................................................   1   1   S03
..............uuuugcugauguggacgcucuau.....................................................................   1   0   S03
..............uuuugcugauguggacgcucuauA....................................................................   3   1   S03
..............uuuugcugauguggacgcucuauc....................................................................   1   0   S03
...............uuugcugauguggacgcuA........................................................................   1   1   S03
...............uuugcugGuguggacgcucu.......................................................................   1   1   S03
...............uuugcugauguggacgcucu.......................................................................   3   0   S03
...............uuugcugauguggacgcucua......................................................................   2   0   S03
...............uCugcugauguggacgcucuau.....................................................................   1   1   S03
...............uuGgcugauguggacgcucuau.....................................................................   1   1   S03
...............uuugcugauguggacgcucuau.....................................................................   11  0   S03
...............uuugcugUuguggacgcucuau.....................................................................   1   1   S03
...............uuugcugauguggacgUucuauc....................................................................   1   1   S03
...............uuugcugauguggacgcucuauU....................................................................   4   1   S03
...............uuugcugauguUgacgcucuauc....................................................................   1   1   S03
...............uuugcugauguggacgcucuauA....................................................................   21  1   S03
................uugcugauguggacgcucu.......................................................................   1   0   S03
................uugcugauguggacgcucua......................................................................   1   0   S03
................uugcugauguggacgcucuauA....................................................................   4   1   S03
.................ugcugauguggacgcucu.......................................................................   7   0   S03
.................ugcugauguggacgcucG.......................................................................   2   1   S03
.................ugcugauguggacgcucua......................................................................   4   0   S03
.................ugcugauguggacgcucuau.....................................................................   1   0   S03
.................ugcugauguggacgcucuaG.....................................................................   2   1   S03
.................ugcugauguggacgcucuauc....................................................................   1   0   S03
.................ugcugauguggacgcucuauA....................................................................   2   1   S03
.................ugcugauguggacgcucuauU....................................................................   2   1   S03
..................gcugauguggacgcucua......................................................................   21  0   S03
..................gcugauguggacgcucuau.....................................................................   13  0   S03
..................gcugauguggacgcucuaG.....................................................................   1   1   S03
..................gcugauguggacgcucuauc....................................................................   3   0   S03
..................gcugauguAgacgcucuauc....................................................................   1   1   S03
..................gcugauguggacgcucuaCc....................................................................   1   1   S03
...................cugauguggacgcucuau.....................................................................   3   0   S03
....................ugauguggacgcucuauc....................................................................   1   0   S03
.............................................aaagguccauuuuauuaguauagC.....................................   1   1   S03
..............................................aaggucUauuuuauuaguauagu.....................................   1   1   S03

.cgggagaaauuuGuuuugcugaug.................................................................................   1   1   S04
..gAgagaaauuuuuuuugcugaugu................................................................................   1   1   S04
........aauuuuuuCugcugauguggacgc..........................................................................   1   1   S04
.........auuuuuuuugcugauguggacgcC.........................................................................   1   1   S04
..........uuuuuuuugcuAauguggacg...........................................................................   1   1   S04
..........uuuuuuuugcugauguggacgc..........................................................................   1   0   S04
..........uuuuuuuugcuAauguggacgcu.........................................................................   1   1   S04
..........uuuuuuuugcugauguggacgcG.........................................................................   1   1   S04
..........uuuuuuuugcugauguggacgcu.........................................................................   2   0   S04
..........uuuuuuuugcugaCguggacgcuc........................................................................   1   1   S04
..........uuuuuuuugcugauguggacgcuc........................................................................   3   0   S04
..........uuuuuuuugcugauguggacUcucu.......................................................................   1   1   S04
..........uuuuuuuugcugauguggacgcucu.......................................................................   1   0   S04
...........uuuuuuugcugauguggacgc..........................................................................   1   0   S04
...........uuuuuuugcugauguggacgcu.........................................................................   4   0   S04
...........uuuuuuugcuAauguggacgcu.........................................................................   3   1   S04
...........uuuuuuugcugauguggacgcG.........................................................................   1   1   S04
...........uuuuuuugcugaCguggacgcuc........................................................................   1   1   S04
```

```
           Mature                          Star
ucgggagaaauuuuuuuugcugauguggacgcucuaucgaaccucaaaagguccauuuuauuaguauaguaguaaucuuacauauuuuuaauguaguauuuccauuuu
...........uuuuuuugcugauguggacgcuc..........................................................................   1    0    S04
...........uuuuuuugcugaCguggacgcucu.........................................................................   8    1    S04
...........uuuuuuugcugauguggacgcucu.........................................................................   7    0    S04
...........uuuuuuugcugauguggacgcucG.........................................................................   1    1    S04
............uuuuuugcugauguggacg.............................................................................   1    0    S04
............uuuuuugcugauguggacgcu...........................................................................   2    0    S04
............uuuuuugcugauguggacgcuc..........................................................................   4    0    S04
............uuuuuugcugauguggacgcuA..........................................................................   1    1    S04
............uuuuuugcugauguggacgcucu.........................................................................   2    0    S04
............uuuuuugcugauguggacgcucG.........................................................................   4    1    S04
............uuuuuugcugauguggacgcucua........................................................................   7    0    S04
.............uuuuugcugauguggacgcu...........................................................................   5    0    S04
.............uuuuugcugauguggacgcuc..........................................................................   2    0    S04
.............uuuuugcugauguggacgcucu.........................................................................   3    0    S04
.............uuuuugcugauguggacgcucua........................................................................   8    0    S04
.............uuuuugcugGuguggacgcucua........................................................................   1    1    S04
.............uuuuugcugauguggacUcucuau.......................................................................   2    1    S04
.............uuuuugcugauguggacgcucuaG.......................................................................   1    1    S04
.............uuuuAgcugauguggacgcucuau.......................................................................   1    1    S04
.............uuuuugcugauguggacgcucuau.......................................................................   13   0    S04
.............uuuuugcugauguggacgcuAuau.......................................................................   1    1    S04
.............uCuuugcugauguggacgcucuau.......................................................................   1    1    S04
.............uuuuugcugauguggacgcucuauU......................................................................   1    1    S04
..............uuuugcugauguggacgcucu.........................................................................   2    0    S04
..............uuuugcugauguggacgcucuauA......................................................................   1    1    S04
...............uuugcugauguggacgcu...........................................................................   1    0    S04
...............uuugcugauguggacgcucu.........................................................................   1    0    S04
...............uuugcugauguggacgcucua........................................................................   2    0    S04
...............uuugcugauguggacgcucuGu.......................................................................   1    1    S04
...............uuugcugauguggacgcucuaA.......................................................................   2    1    S04
...............uuugcugauguggacgcucuau.......................................................................   2    0    S04
...............uuugcugauguggacgcucuauU......................................................................   1    1    S04
...............uuCgcugauguggacgcucuauc......................................................................   4    1    S04
...............uuugcugauguggacgcucuauA......................................................................   22   1    S04
...............uuugcugauguggacgcucuauc......................................................................   1    0    S04
...............uuugcugaCguggacgcucuauc......................................................................   1    1    S04
...............uuugcugauguggacgcucuauUg.....................................................................   1    1    S04
...............uuugcugauguggacgcucuauAg.....................................................................   1    1    S04
................uugcugauguggacgcucuauU......................................................................   1    1    S04
................uugcugauguggacgcucuauc......................................................................   1    0    S04
................uugcugauguggacgcuUuauc......................................................................   1    1    S04
................uCgcugauguggacgcucuauc......................................................................   1    1    S04
................uugcugauguggacgcucuauA......................................................................   1    1    S04
.................ugcugauguggacgcucu.........................................................................   2    0    S04
.................ugcugauguggacgcucua........................................................................   4    0    S04
.................Ggcugauguggacgcucuau.......................................................................   1    1    S04
.................ugcugauguggacgcucuau.......................................................................   2    0    S04
.................ugcugauguggacgcucuauA......................................................................   1    1    S04
.................ugcugauguggacgcucuaucg.....................................................................   1    0    S04
..................gcugauguggacgcucua........................................................................   6    0    S04
..................gcugauguggacgcucuau.......................................................................   3    0    S04
..................gcugauguggacgcucuauc......................................................................   2    0    S04
...................cugauguggacgcucuauc......................................................................   1    0    S04
.....................gauguggacgcucuauAgaaccu................................................................   1    1    S04
......................auguggacgcucuauAgaaccuca..............................................................   1    1    S04
```

Provisional ID	:	chrAnn_random_3133343
Score total	:	0
Score for star read(s)	:	-1.3
Score for read counts	:	0
Score for mfe	:	1.2
Score for randfold	:	0
Score for cons. seed	:	
Total read count	:	27
Mature read count	:	25
Loop read count	:	2
Star read count	:	0

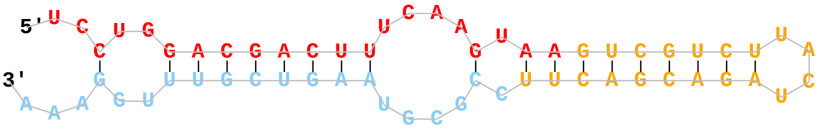

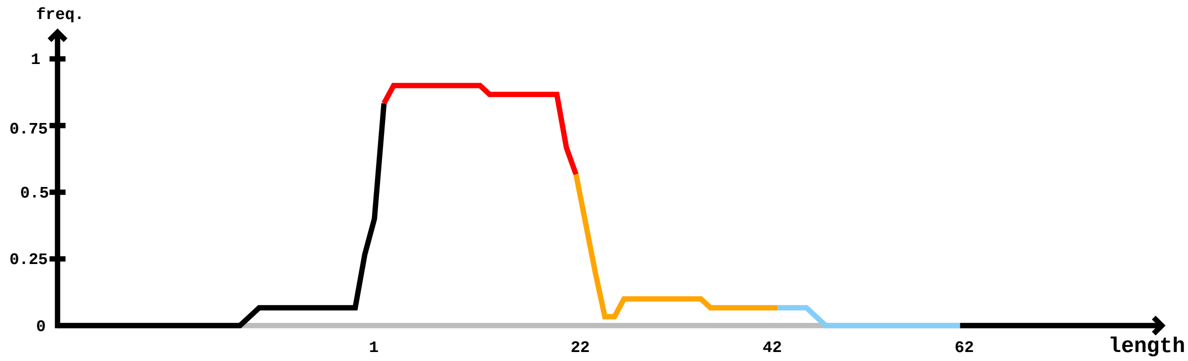

Mature Star

```
5'- aucuaggaaauaaaucuagacgacuuuguuuauccuggacgacuuucaaguaagucgucuuacuagacgacuuccgcguaagucguuuggaaaaaguuaaauauuuaaguuuu   -3'    exp
    .((((((.......)))))).((((((.....(((.(((((((((....(.(((((((((....))))))))).)....))))))))))))).))))))...............    reads     mm     sample
    ..............................uauccuggacgacuuucaagG..............................................................    1         1      S03
    ..............................uauccuggacgacuuucaagA..............................................................    1         1      S03
    ..............................uauccuggacgacuuucaagu..............................................................    3         0      S03
    ...............................auccuggacgacuuucaagua.............................................................    1         0      S03
    ...............................aucAuggacgacuuucaaguaagu..........................................................    1         1      S03
    ...............................auccuggacgacuuucaaguaagu..........................................................    1         0      S03
    ................................uccuggacgacuuucaagua.............................................................    1         0      S03
    ................................uccuggacgacuuucaaguaa............................................................    6         0      S03
    ................................uccuggacgacuuucaaguaag...........................................................    2         0      S03
    ................................uccuggacgacuuucaaguaUg...........................................................    1         1      S03
    ................................uccuggacgacuuucaaguaaU...........................................................    1         1      S03
    ................................uccuggacgacuuucaaguaagA..........................................................    1         1      S03
    .................................ccuggacgacuuucaaguaagu..........................................................    1         0      S03
    .........................................................ucuuacuGgacgacuuccgcg...................................    1         1      S03

    ..................gacgacuuuguuuauccuggacga.......................................................................    1         0      S04
    ...................acgacuuuguuuauccuggacgaU......................................................................    1         1      S04
    ..............................uauccuggacgacuuucaagu..............................................................    1         0      S04
    ...............................auccuggacgacuuucaagua.............................................................    1         0      S04
    ................................uccuggacgacuuucaaguaa............................................................    1         0      S04
    .................................ccuggacgacuuucaaCuaagu..........................................................    1         1      S04
    ..........................................cuuucaaguaagucgucuuacuGg...............................................    1         1      S04
    .........................................................ucuuacuGgacgacuuccgc....................................    1         1      S04
```

Provisional ID	:	chrAnn_random_3133344
Score total	:	0.7
Score for star read(s)	:	-1.3
Score for read counts	:	0
Score for mfe	:	2
Score for randfold	:	0
Score for cons. seed	:	
Total read count	:	25
Mature read count	:	25
Loop read count	:	0
Star read count	:	0

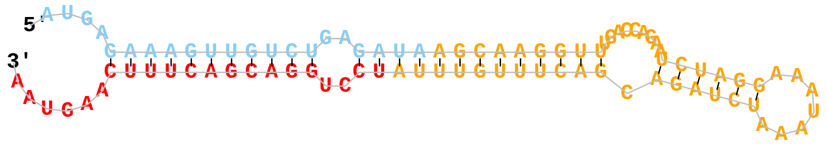

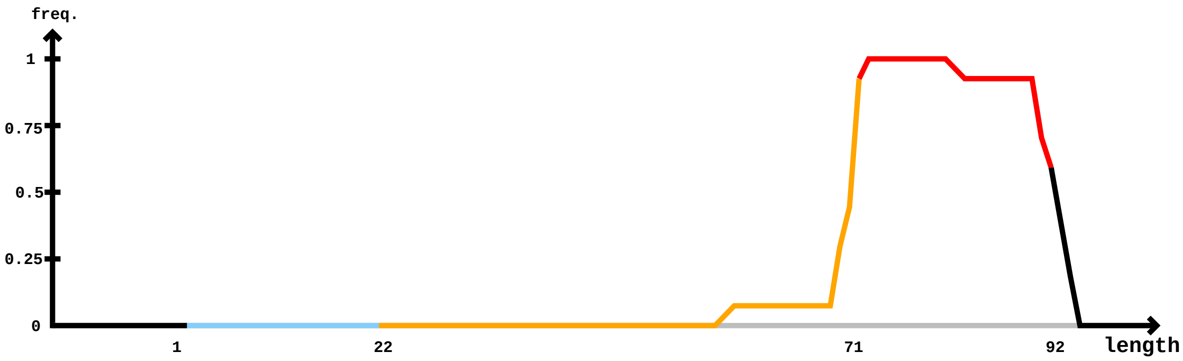


```
                 Star                                                     Mature
5'- gguagacgauguaugagaaaguugucugagauaagcaagguuugaccagaaucuaggaaauaaaucuagacgacuuuguuuauccuggacgacuuucaaguaagucgucuuac   -3'    exp
    ...(((((((.(((..(((((((((((..(((((((((((((.........((((((.......)))))).)))))))))))))..)))))))))))..))).)))))))...    reads       mm          sample
    ................................................................................uauccuggacgacuuucaagu............    3           0           S03
    ................................................................................uauccuggacgacuuucaagA............    1           1           S03
    ................................................................................uauccuggacgacuuucaagG............    1           1           S03
    .................................................................................auccuggacgacuuucaagua...........    1           0           S03
    .................................................................................auccuggacgacuuucaaguaagu........    1           0           S03
    .................................................................................aucAuggacgacuuucaaguaagu........    1           1           S03
    ..................................................................................uccuggacgacuuucaagua...........    1           0           S03
    ..................................................................................uccuggacgacuuucaaguaa..........    6           0           S03
    ..................................................................................uccuggacgacuuucaaguaag.........    2           0           S03
    ..................................................................................uccuggacgacuuucaaguaUg.........    1           1           S03
    ..................................................................................uccuggacgacuuucaaguaaU.........    1           1           S03
    ..................................................................................uccuggacgacuuucaaguaagA........    1           1           S03
    ...................................................................................ccuggacgacuuucaaguaagu........    1           0           S03

    ....................................................................gacgacuuuguuuauccuggacga.....................    1           0           S04
    .....................................................................acgacuuuguuuauccuggacgaU....................    1           1           S04
    ................................................................................uauccuggacgacuuucaagu............    1           0           S04
    .................................................................................auccuggacgacuuucaagua...........    1           0           S04
    ..................................................................................uccuggacgacuuucaaguaa..........    1           0           S04
    ...................................................................................ccuggacgacuuucaaCuaagu........    1           1           S04
```

Provisional ID	:	chrAnn_random_3148615
Score total	:	0.7
Score for star read(s)	:	-1.3
Score for read counts	:	0
Score for mfe	:	2
Score for randfold	:	0
Score for cons. seed	:	
Total read count	:	1250
Mature read count	:	936
Loop read count	:	10
Star read count	:	304

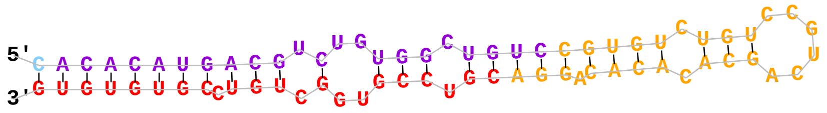

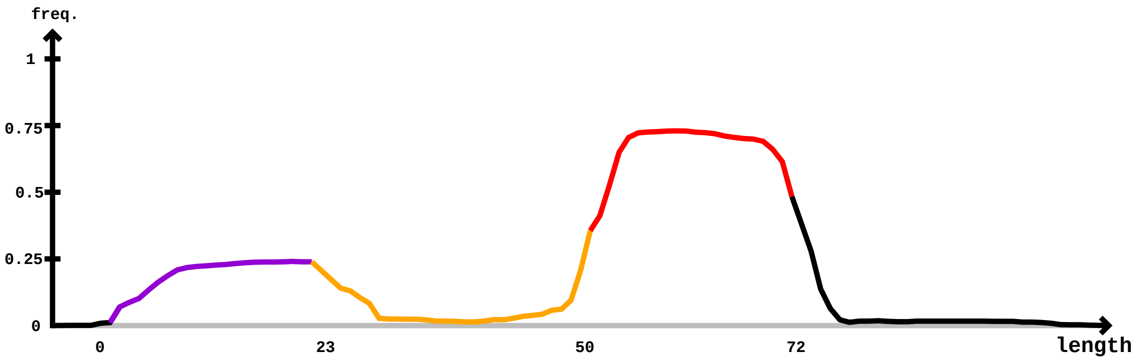

Star   Mature

```
5'- agcacacacaugacgucuguggcugucccgugucuguccgucagcacacacaggacguccguggcuguccgugugugucc gcgugugucc gugugugucc guccgcaca   -3'   obs
    agcacacacaugacgucuguggcugucccgugucuguccgucagcacacacaggacguccguggcuguccgugugugucc gcgugugucc gugugugucc guccgcaca         exp
    .((((((((..((((.(..(((.((((((((.(((......))).)))).))))).)))..).)))).))))))))....(((((..((........))..))))).   reads   mm   sample
    ...Gcacacaugacgucuguggcugu..................................................................................   1   1   S03
    ...acacacaugacgucuguggcugGc.................................................................................   1   1   S03
    ....cacacaugacgucuguggcuguc.................................................................................   1   0   S03
    .....acacaugacgucuguggcuguc.................................................................................   4   0   S03
    .....acacaugacgucuguggcugCc.................................................................................   1   1   S03
    .....acacaCgacgucuguggcuguc.................................................................................   1   1   S03
    ......cacaugacgucuguUgcugucc................................................................................   1   1   S03
    .......acaugacgucuguggcugu..................................................................................   1   0   S03
    .......acaugacgucuguggcuguc.................................................................................   2   0   S03
    .......acaugacgucuguggcugucA................................................................................   1   1   S03
    .......acaugacgucuguggcuguccgu..............................................................................   2   0   S03
    ........caugacgucuguggcuguc.................................................................................   1   0   S03
    ........caugacgucuguggcuguccg...............................................................................   1   0   S03
    ........caugacgucugGggcuguccgugu............................................................................   1   1   S03
    ........caugacgucuguggcuguccgugC............................................................................   2   1   S03
    ........caugacgucuguggcuguccgugu............................................................................   1   0   S03
    .........augacgucuguggcuguccgu..............................................................................   1   0   S03
    .........augacgucuguggcuguGcgug.............................................................................   1   1   S03
    .........augacgucuguggcuguccgug.............................................................................   4   0   S03
    ..........Cgacgucuguggcuguccg...............................................................................   1   1   S03
    ..........uUacgucuguggcuguccgu..............................................................................   1   1   S03
    ..........ugacgucuguggcuguAcgugu............................................................................   1   1   S03
    ..........ugacgucuguggcuguccgugu............................................................................   1   0   S03
    ...........gacgucuguggcuguccgugu............................................................................   2   0   S03
    ............acgucuguggcuguccgugu............................................................................   1   0   S03
    ...................uAgcuguccgugucuguccguc...................................................................   1   1   S03
    ...................uggcuguccgugucuguAcguc...................................................................   1   1   S03
    ......................cuguccUugucuguccgucagc................................................................   1   1   S03
    .........................................agcacacacaggUcguccguggc............................................   1   1   S03
    ...........................................cacacacaggUcguccguggc............................................   1   1   S03
    ...........................................cacacacaggacguccguggcugG.........................................   1   1   S03
    ............................................acacacaggacguccguggcugG.........................................   1   1   S03
    ...............................................cacaggacguccguggcugG.........................................   1   1   S03
```

Star **Mature**

agcacacacaugacgucuguggcugucCgugucuguccgucagcacacacaggacguccguggcuguccguguguguccgcguguguccguguguguccguccgcaca			
....cacaggacguccguggcuguGcgu.....................................	1	1	S03
....acaggacguccguggcuguccg......................................	1	0	S03
....caggacguccguggcuguccgugu...................................	1	0	S03
....aggacguccguggcuguccgugug..................................	6	0	S03
....aggacguccguggcuguccgugugu.................................	1	0	S03
....gacguccguggcuguccg.......................................	1	0	S03
....gacguccguggcuguccgu......................................	1	0	S03
....Uacguccguggcuguccgugug...................................	1	1	S03
....gacguccguggcuguccgugug...................................	2	0	S03
....gacguccguggcuguccgugugu..................................	1	0	S03
....gacguccguggcuguccgugugug.................................	4	0	S03
....acguccguggcuguccgugu....................................	1	0	S03
....acguccguggcuguccgugug...................................	7	0	S03
....acguccguggcuguccgugGgu..................................	1	1	S03
....acguccguggcuguccgugugu..................................	13	0	S03
....acguccguggcuguccgugugC..................................	3	1	S03
....acgCccguggcuguccgugugu..................................	1	1	S03
....acguccguggcuguccgugugA..................................	2	1	S03
....acguccguggcuguccgugugug.................................	2	0	S03
....acguccguggcuguccgugugugu................................	6	0	S03
....aGguccguggcuguccgugugugu................................	1	1	S03
....cguccguggcuguccgugu...................................	1	0	S03
....cguccguggcuguccgugug..................................	1	0	S03
....cguccguggcuguccgugugC.................................	1	1	S03
....cguccguggcuguccgugugu.................................	4	0	S03
....cguccguggcuguccgugugug................................	31	0	S03
....cguccgAggcuguccgugugug................................	1	1	S03
....cguccguggcuguccgugugugu...............................	1	0	S03
....cguccguggcuguccguguguguc..............................	7	0	S03
....guccguggcuguccgugug..................................	2	0	S03
....guccguggcuguccgugugug................................	5	0	S03
....guccguggcuguccguguguA...............................	1	1	S03
....guccguggcuguccgugugugu...............................	3	0	S03
....uccguggcuguccgugugu.................................	1	0	S03
....uccguggcuguccgugugG.................................	1	1	S03
....uccguggcuguccgugugug................................	1	0	S03
....uccguggcuguccgugugugu...............................	3	0	S03
....uccguggcuguccguguguguc..............................	10	0	S03
....uccgCggcuguccguguguguc..............................	1	1	S03
....ucGguggcuguccguguguguc..............................	1	1	S03
....uccguggcuguccgugugugucccg............................	4	0	S03
....ccguggcuguccgugugu.................................	3	0	S03
....ccguggcuguccgugugug................................	1	0	S03
....ccguggcuguccgugugugu...............................	4	0	S03
....ccguggcuguccgugugCgu...............................	1	1	S03
....ccguggcuguccgugugugA...............................	1	1	S03
....ccguggcuguccguCuguguc..............................	1	1	S03
....ccguggcuguccguguguguc..............................	17	0	S03
....ccguggcugAccguguguguc..............................	1	1	S03
....ccguggcuguccguguguguAc.............................	1	1	S03
....ccguggcuguccguCugugucc.............................	1	1	S03
....ccguggcuguccgugugugucc.............................	5	0	S03
....ccguggcuguccguguguguccg............................	2	0	S03
....cguggcuguccguguguguc..............................	3	0	S03
....cguggcuguccguCugugucc.............................	2	1	S03
....cguggcuguccgugugugucc.............................	1	0	S03
....cguggcuguccguguguguccg............................	7	0	S03
....cguggcuguccgugAguguccg............................	1	1	S03
....cguggcuguccguguguguccgG...........................	1	1	S03
....guggcuguccguguguguccg............................	5	0	S03
....guggcuguccguguguguccgc...........................	1	0	S03
....ggcuguccguguguguccg............................	1	0	S03
....gcuguccguguguguccg............................	1	0	S03
....ugucUgcguguguccgugugug............	1	1	S03
....uguguccguguguguccgu.......	1	0	S03
....uguguccguguguguccguc......	2	0	S03
....uguccAuguguguccgucc.....	1	1	S03
....cguguguguccgucGgcac.	1	1	S03
agGacacacaugacgucug...	1	1	S04

Star Mature

```
agcacacacaugacgucuguggcugucgugucuguccgucagcacacacaggacguccguggcuguccgugugugu ccgcguguguccgugugugucc guccgcaca
...acacacaugacgucuCuggcugu..................................................................................  1  1  S04
...acacacaugacgucuguggcugu..................................................................................  1  0  S04
...acacacaugacgucuguggcuguc.................................................................................  5  0  S04
...acacacaugacgucuguggcuguU.................................................................................  1  1  S04
...acacacaugacgucugAggcuguc.................................................................................  1  1  S04
....cacacaugacgucugugUcu....................................................................................  1  1  S04
....cacacaugacgucugCggcugu..................................................................................  1  1  S04
....cacacaugacgucuguggcuguc.................................................................................  1  0  S04
.....acacaugacgucuguggc.....................................................................................  1  0  S04
.....acacaugacgucuguggcugu..................................................................................  4  0  S04
.....Gcacaugacgucuguggcugu..................................................................................  1  1  S04
.....acacaCgacgucuguggcuguc.................................................................................  4  1  S04
.....acacaugacgucuguggcCguc.................................................................................  1  1  S04
.....acacaugacgucuguggcugCc.................................................................................  2  1  S04
.....acacaugacgucuguggcuguA.................................................................................  2  1  S04
.....acacUugacgucuguggcuguc.................................................................................  1  1  S04
.....acacaugacgucuguggcuCuc.................................................................................  1  1  S04
.....acacaugacgucuguggcuguU.................................................................................  7  1  S04
.....acacaugacgucAguggcuguc.................................................................................  1  1  S04
.....acaUaugacgucuguggcuguc.................................................................................  1  1  S04
.....acacaugacgucuguggcuguc.................................................................................  41  0  S04
.....acacaCgacgucuguggcugucc................................................................................  1  1  S04
.....acacaugacgucuguggcugucA................................................................................  1  1  S04
.....acacaugacgucuguggcugucc................................................................................  3  0  S04
.....acacaugacguGuguggcuguccg...............................................................................  1  1  S04
.....acacaugacgucuguggcuguccg...............................................................................  2  0  S04
......cacaugacgucuguggcu....................................................................................  1  0  S04
......cacaugacgucugCggcuguc.................................................................................  1  1  S04
......cacaugacgucuguggcugAc.................................................................................  1  1  S04
......cacaugacgucuguggcuguU.................................................................................  1  1  S04
......cacaugacgucuguggcuguc.................................................................................  3  0  S04
......cacaugacgucugugAcugucc................................................................................  1  1  S04
......cacaugacgucuguggcugucc................................................................................  6  0  S04
......cacaugacgucuguggcugucA................................................................................  3  1  S04
......cacaCgacgucuguggcugucc................................................................................  3  1  S04
......cacaugacgucuguggcugucG................................................................................  1  1  S04
......cacaugacgucugugCcugucc................................................................................  1  1  S04
......cacaugacgucuguggcuguUcg...............................................................................  1  1  S04
.......acaugacgucugugCcuguc.................................................................................  1  1  S04
.......acaugacgucuguggcuguG.................................................................................  1  1  S04
.......acaugacguGuguggcuguc.................................................................................  1  1  S04
.......acaugacgucuguggcuguc.................................................................................  1  0  S04
.......acaugacguGuguggcugucc................................................................................  1  1  S04
.......acaugacgucuguggcugucc................................................................................  4  0  S04
.......acaugacgucAguggcuguccg...............................................................................  1  1  S04
.......acaugacgucuguggcuguccg...............................................................................  2  0  S04
.......acaugacgucuguggcuCuccgu..............................................................................  1  1  S04
.......acaugacgucuguggcuguccgu..............................................................................  1  0  S04
........caugacguGuguggcugu..................................................................................  1  1  S04
........caugacgucuguggcuguc.................................................................................  2  0  S04
........caugacgucuguggcuCucc................................................................................  1  1  S04
........caugacgucuguggcugucc................................................................................  3  0  S04
........caugacgucuguggcuguccg...............................................................................  2  0  S04
........caugacgucugugCcuguccg...............................................................................  1  1  S04
........caugacgucuguggcuguccgG..............................................................................  1  1  S04
........caugacgucuguggcugucAgu..............................................................................  1  1  S04
........caugacgucugAggcuguccgu..............................................................................  1  1  S04
........caugacgucuguggcuguGcgu..............................................................................  1  1  S04
........caugacgucAguggcuguccgu..............................................................................  1  1  S04
........caugacgucuguggcuCuccgu..............................................................................  1  1  S04
........caugacgucugugUcuguccgu..............................................................................  1  1  S04
........caugacgucuguggcuguccgu..............................................................................  8  0  S04
........caCgacgucuguggcuguccgu..............................................................................  1  1  S04
........caugacgucuguggcuguccgA..............................................................................  1  1  S04
........Aaugacgucuguggcuguccgug.............................................................................  1  1  S04
........caugacgucugugUcuguccgug.............................................................................  1  1  S04
........caugacgucuguggcuguccgugu............................................................................  5  0  S04
........caugacgucuguggcuguccgugC............................................................................  2  1  S04
........caugacgucugCggcuguccgugu............................................................................  1  1  S04
........caugacgucugGggcuguccgugu............................................................................  1  1  S04
```

Star **Mature**

agcacacacaugacgucuguggcugucgugucuguccgucagcacacacaggacguccguggcuguccgugugugucgcguguguccgugugugucc guccgcaca

.........augacgucuguggcuguc...	1	0	S04
.........Gugacgucuguggcuguc...	1	1	S04
.........augacgucuguggcugucA..	1	1	S04
.........augacgucuguggcugucc..	1	0	S04
.........augacgucuguggcuguccg...	2	0	S04
.........augacgucuUggcuguccg..	1	1	S04
.........augacgucuguggcuguccgG..	1	1	S04
.........augacgAcuguggcuguccgu..	2	1	S04
.........augacgucuguggcuguccgu..	2	0	S04
.........augacgucuguAgcuguccgug...	2	1	S04
.........augacgucuAuggcuguccgug...	1	1	S04
.........augacgucuguggcuguccgCg...	1	1	S04
.........augGcgucuguggcuguccgug...	1	1	S04
.........augacgucuguggcuguccgug...	9	0	S04
.........augacgucuguggcuguccgugu..	6	0	S04
.........augacgucugGggcuguccgugu..	1	1	S04
.........augacgucuguggcuguccguCu..	1	1	S04
.........augacgucuguggcuguccguguA...	1	1	S04
..........ugacgucuguggcuguccgA..	1	1	S04
..........ugacgucuguggcuguccgG..	1	1	S04
..........Cgacgucuguggcuguccgu..	1	1	S04
..........ugacgucuguggcuguccgu..	2	0	S04
..........ugacgucuguggcuguccgug...	1	0	S04
..........ugacgucugugUcuguccgug...	1	1	S04
..........ugacgucuguggcuguUcgugu..	1	1	S04
..........ugacgucuguggcuguGcgugu..	1	1	S04
..........ugacgucuguggcuAuccgugu..	1	1	S04
..........uUacgucuguggcuguccgugu..	1	1	S04
..........ugacgucuguggcuguccgugA..	2	1	S04
..........ugacgucuguggcugucAgugu..	1	1	S04
..........ugacgucuguggcuguccgugC..	1	1	S04
..........ugacgucuguggcuguccgugu..	15	0	S04
...........gacgucuguggcuguccgu..	1	0	S04
...........gacgucugGggcuguccgug...	1	1	S04
...........gacgucuguggcuguccgug...	5	0	S04
...........gacgucuguggcuguccgugu..	16	0	S04
...........gacgucuguggcuguccgugC..	2	1	S04
...........gacgucuguggcuguccguguU...	3	1	S04
............acgucuguggcuguccgA..	1	1	S04
............acgucugGggcuguccgugu..	1	1	S04
............acgucuguggcuguccgugu..	8	0	S04
............acgucuguggcuguccguguU...	1	1	S04
.............cgucuCuggcuguccgug...	1	1	S04
.............cgucugGggcuguccgug...	2	1	S04
.............cgucuguggcuguccgugu..	2	0	S04
.............cgucuguggcuCuccgugu..	1	1	S04
..............gucuguggcuguccgugu..	1	0	S04
..............gucugGggcuguccgugu..	1	1	S04
..............gucuguggcuAuccgugucug...	1	1	S04
...............ucuguggcuguccguAucug...	1	1	S04
...............ucuguggcuguccgugucuguc...	1	0	S04
...............ucuguggcuguAcgugucuguc...	1	1	S04
...............ucuguggcuguccAugucuguc...	1	1	S04
................cuguggcuguccgugucugucU..	2	1	S04
................cuguggcuguccgugucuAucc..	1	1	S04
.................Cguggcuguccgugucuguc...	1	1	S04
.................uguggcuguccgugucuguc...	1	0	S04
.................uguggcuguccgugucugAcc..	1	1	S04
.................uguggcuguccgugucuguccgu..	1	0	S04
.................uguggcuguccgugucuguccguA...	1	1	S04
..................guggcuguccgugucugucU..	1	1	S04
..................guggcuguccgugucuguccgu..	1	0	S04
..................guggcuguccgugucuguUcguc...	1	1	S04
..................guggcuguccgugucugucUguc...	1	1	S04
...................uggcuguccgugucuguA...	1	1	S04
...................uggcugucUgugucuguccgucag...	1	1	S04
....................Agcuguccgugucuguccg...	1	1	S04
.......................uguccgugucuguccgucagc..	1	0	S04
.......................uguccgugucuguccgucagAac..	2	1	S04
...........................cgugucuguccguAagca...	1	1	S04

Star **Mature**

```
agcacacacaugacgucuguggcuguccgugucuguccgucagcacacacaggacguccguggcuguccguguguguccgcguguguccguguguguccguccgcaca
.............................ugucuguccgucagcacacaca.........................................................  1  0  S04
...............................ucuguccgucaUcacacaca.........................................................  1  1  S04
...............................ucuguccgucagcacacacaggaU.....................................................  1  1  S04
................................cuguccgucagcacacacagga......................................................  2  0  S04
................................cugucGgucagcacacacagga......................................................  1  1  S04
.................................ugAccgucagcacacacaggacgu...................................................  1  1  S04
...................................uccgucagcacacacaggacgA...................................................  1  1  S04
.....................................Ngucagcacacacaggacgu...................................................  1  1  S04
.......................................ucagcacacacaggacguccgugC.............................................  1  1  S04
........................................cagcacacacaggUcguccgu...............................................  1  1  S04
.........................................agcaAacacaggacguccgugg.............................................  1  1  S04
...........................................cacUcacaggacguccgu...............................................  1  1  S04
...........................................cacacacaggUcguccguggc............................................  1  1  S04
...........................................cacacacaggUcguccguggcu...........................................  1  1  S04
...........................................caAacacaggacguccguggcu...........................................  1  1  S04
............................................acUcacaggacguccguggcu...........................................  1  1  S04
............................................acacacaggUcguccguggcu...........................................  2  1  S04
............................................acacacaggacguccgAggcug..........................................  1  1  S04
............................................acacacaggUcguccguggcuguc........................................  1  1  S04
............................................acacacaggacguccguggcuguG........................................  1  1  S04
............................................aAacacaggacguccguggcuguc........................................  2  1  S04
..............................................acacaggUcguccguggcug..........................................  1  1  S04
..............................................acacaggacguccguggcugG.........................................  3  1  S04
..............................................acacaggacguccguggcuguG........................................  1  1  S04
..............................................acacaggUcguccguggcuguc........................................  2  1  S04
..............................................acacaggacguccgAggcuguc........................................  2  1  S04
..............................................acacaggacguccguggcuguccg......................................  1  0  S04
...............................................cacaggacguccgAggcu...........................................  1  1  S04
...............................................Nacaggacguccguggcuguc........................................  1  1  S04
...............................................cacaggacguccguggcuguG........................................  1  1  S04
...............................................cacaggacguccguggcugGcc.......................................  1  1  S04
...............................................cacaggacguccgAggcugucc.......................................  1  1  S04
...............................................cacaggUcguccguggcugucc.......................................  1  1  S04
...............................................cacaggacguccguggcugucG.......................................  2  1  S04
................................................acaggacguccguggcuguG........................................  1  1  S04
................................................acaggacguccguggcuguccg......................................  2  0  S04
................................................acagUacguccguggcuguccgugu...................................  1  1  S04
.................................................caggacguccguggcuguGc.......................................  1  1  S04
.................................................caggaGguccguggcugucc.......................................  1  1  S04
.................................................caggacguccguggcuguccgu.....................................  1  0  S04
.................................................cagUacguccguggcuguccgugu...................................  1  1  S04
.................................................caggacguccguggcuguccgugu...................................  1  0  S04
..................................................aggacguccguggcuguccgug....................................  2  0  S04
..................................................aggacguccguggcugAccgugu...................................  3  1  S04
..................................................aggacguccguggcuguccgugug..................................  5  0  S04
..................................................aggGcguccguggcuguccgugug..................................  1  1  S04
..................................................agUacguccguggcuguccgugug..................................  1  1  S04
..................................................aggacguccguggcuguccgugugu.................................  1  0  S04
..................................................aggacguccguggcuguccgugugA.................................  1  1  S04
...................................................ggacguccguggcugucGg......................................  1  1  S04
...................................................ggacguccguggcuguccgug....................................  1  0  S04
...................................................gUacguccguggcuguccgug....................................  2  1  S04
...................................................ggacguccguggcuguccgugu...................................  1  0  S04
...................................................ggacguccguggcuguccgugugA.................................  1  1  S04
...................................................ggacguccguggcuguccgugugu.................................  1  0  S04
....................................................gacguccguggcuguccgug....................................  3  0  S04
....................................................gacguccguggcuguccgugu...................................  9  0  S04
....................................................gacguccguggcugAccgugu...................................  1  1  S04
....................................................gacguccguggcuguccgugA...................................  1  1  S04
....................................................gacguccguggcuguccgGgu...................................  1  1  S04
....................................................gacguccguggcuguccgugug..................................  12  0  S04
....................................................Cacguccguggcuguccgugug..................................  1  1  S04
....................................................gacguccguggcuguccguguUu.................................  1  1  S04
....................................................gacguccguggcuguccgugugG.................................  1  1  S04
....................................................gacguccguggcuguccgugugu.................................  3  0  S04
....................................................gacguccguggcuguccgugugug................................  2  0  S04
....................................................gacguccguggcuguccguguguguccgA...........................  2  1  S04
.....................................................acguccguggcuguccgugu...................................  13  0  S04
.....................................................Ucguccguggcuguccgugu...................................  1  1  S04
.....................................................acgCccguggcuguccgugu...................................  1  1  S04
```

```
     Star                                           Mature
agcacacacaugacgucuguggcugucccgugucuguccgucagcacacacaggacguccguggcuguccgugugugucccgcguguguccguguguguccguccgcaca
.....................................................acguccguggcuguccgugug..................................  14  0  S04
.....................................................acguccguggcuguccgugugG.................................  3  1  S04
.....................................................acguccguggcuguccgugGgu.................................  1  1  S04
.....................................................acguccguggcuguccgugugu.................................  54  0  S04
.....................................................acguccguggcuguccguguUu.................................  1  1  S04
.....................................................Ncguccguggcuguccgugugu.................................  1  1  S04
.....................................................Gcguccguggcuguccgugugu.................................  1  1  S04
.....................................................acguccgCggcuguccgugugu.................................  1  1  S04
.....................................................acguAcguggcuguccgugugu.................................  1  1  S04
.....................................................acguccguggcuguccgugugA.................................  5  1  S04
.....................................................acguccguggcuguccgugugC.................................  8  1  S04
.....................................................acguccguggcuguccgugugug................................  2  0  S04
.....................................................acguccguggcuguccgugugugu...............................  11  0  S04
.....................................................acguccguggcuguccgugugugucc.............................  1  0  S04
......................................................cguccguggcuguccgug....................................  1  0  S04
......................................................cguccguggcugAccgug....................................  1  1  S04
......................................................cguccgAggcuguccgug....................................  1  1  S04
......................................................cguccguggcuguccgugu...................................  3  0  S04
......................................................cguccguggcuguccgugA...................................  1  1  S04
......................................................cguccguggcuguccgugug..................................  4  0  S04
......................................................cguccgCggcuguccgugug..................................  1  1  S04
......................................................cguccguggcuguccgugugu.................................  30  0  S04
......................................................cguccguggcuguccgugugC.................................  4  1  S04
......................................................cguccguggcuguccgugugA.................................  3  1  S04
......................................................cguccguggcugAccgugugug................................  1  1  S04
......................................................Gguccguggcuguccgugugug................................  1  1  S04
......................................................cguccguggcuguccgugugGg................................  1  1  S04
......................................................cguccguggcuguccgCgugug................................  1  1  S04
......................................................cguccguggcuguccgugugug................................  87  0  S04
......................................................cgCccguggcuguccgugugug................................  1  1  S04
......................................................cguccgAggcuguccgugugug................................  1  1  S04
......................................................cguccguggcuguccgugugugu...............................  6  0  S04
......................................................cguccguggcuguccgugugugG...............................  1  1  S04
......................................................cguAcguggcuguccguguguguc..............................  1  1  S04
......................................................cguccguggcuguccguguguguc..............................  11  0  S04
......................................................cguccguggcuguccguguguguA..............................  1  1  S04
.......................................................guccguggcuguccgugu...................................  1  0  S04
.......................................................guccguggcuguccgugug..................................  5  0  S04
.......................................................guccguggcuguccgugugu.................................  5  0  S04
.......................................................guccguggcuguccgugugC.................................  2  1  S04
.......................................................guccguggcuguccgugugug................................  14  0  S04
.......................................................guccguggcuguccguCugugu...............................  2  1  S04
.......................................................guccguggcuguccgugugugu...............................  22  0  S04
.......................................................guccguggcuguccgugugGgu...............................  1  1  S04
.......................................................guccguggcuguccgugugugA...............................  3  1  S04
.......................................................guccgAggcuguccguguguguc..............................  1  1  S04
.......................................................guccguggcuguccguguguguc..............................  5  0  S04
.......................................................guccguggcuguccguCuguguc..............................  1  1  S04
.......................................................guccguggcuguccgugugugucc.............................  5  0  S04
.......................................................guccguggcuguccgugugugucG.............................  1  1  S04
........................................................uccguggcuguccgugug..................................  2  0  S04
........................................................uccguggcuguccgugugC.................................  2  1  S04
........................................................uccguggcuguccgugugu.................................  4  0  S04
........................................................uccguggcuguccgugugug................................  11  0  S04
........................................................uccguggcuguccgugugCg................................  1  1  S04
........................................................uccguggcuguccgugugugu...............................  14  0  S04
........................................................uccguggcuguccgugugAgu...............................  1  1  S04
........................................................uccguggcuguccguCugugu...............................  1  1  S04
........................................................uccguggcuguccgugugugA...............................  1  1  S04
........................................................uccguggcuguccgGguguguc..............................  1  1  S04
........................................................uccguggcuguccgugugGguc..............................  3  1  S04
........................................................uAcguggcuguccguguguguc..............................  3  1  S04
........................................................uccguggcuguccguguguguc..............................  70  0  S04
........................................................uccguggcugucGguguguguc..............................  1  1  S04
........................................................uccguggcuguccguCuguguc..............................  2  1  S04
........................................................uccguggcuguccgugugugucc.............................  7  0  S04
........................................................uccguggcuguccgugugugucG.............................  1  1  S04
........................................................uccguggcugAccgugugugucc.............................  1  1  S04
........................................................uccguggcuguccgCguguguccg............................  1  1  S04
........................................................uccguggcuguccguguguguccg............................  10  0  S04
```

```
    Star                                            Mature
agcacacacaugacgucuguggcugucCgugucuguccgucagcacacacaggacguccguggcuguccgugugugUccgcguguguccguguguguccguccgcaca
.........................................................ccguggcugAccgugugu.................................  1  1  S04
.........................................................ccguggcuguccgugugu.................................  10  0  S04
.........................................................Acguggcuguccgugugu.................................  1  1  S04
.........................................................ccguggcuguccgugugG.................................  1  1  S04
.........................................................ccguggcuguccgugugC.................................  2  1  S04
.........................................................ccguggcuguccguCugug................................  1  1  S04
.........................................................ccguggcuguccgugugug................................  9  0  S04
.........................................................ccgCggcuguccgugugugu...............................  1  1  S04
.........................................................ccguggcuguccgugugugu...............................  13  0  S04
.........................................................ccguggcuguccgugugGgu...............................  1  1  S04
.........................................................ccguggcuguccgugugGguc..............................  1  1  S04
.........................................................ccguggcuguccgugugugAc..............................  1  1  S04
.........................................................ccguggcuguccguguguguc..............................  30  0  S04
.........................................................ccguggcuguccgugugGgucc.............................  1  1  S04
.........................................................ccgCggcuguccgugugugucc.............................  1  1  S04
.........................................................ccguggcugAccgugugugucc.............................  1  1  S04
.........................................................ccguggcuguccguCugugucc.............................  4  1  S04
.........................................................ccguggcuguccgugugugucG.............................  1  1  S04
.........................................................ccguggcuguccguguguguAc.............................  2  1  S04
.........................................................ccguggcuguccgugugugucc.............................  45  0  S04
.........................................................Ncguggcuguccgugugugucc.............................  1  1  S04
.........................................................ccguggcuguccguguguguccg............................  2  0  S04
.........................................................ccgugCcuguccguguguguccg............................  1  1  S04
.........................................................ccguggcuguccguguguguccgc...........................  2  0  S04
.........................................................ccguggcuguccguguguguccgG...........................  2  1  S04
.........................................................ccguggcuguccgugugugucUgc...........................  1  1  S04
..........................................................cguggcuguccgugugug................................  1  0  S04
..........................................................cguggcuguccgugugugu...............................  5  0  S04
..........................................................cguggcuguccguguUuguc..............................  2  1  S04
..........................................................cguggcuguccguguCuguc..............................  1  1  S04
..........................................................cguggcuguccguguguguc..............................  17  0  S04
..........................................................cguggcuguccguCuguguc..............................  1  1  S04
..........................................................cguggcuguccgCguguguc..............................  1  1  S04
..........................................................cguggcuguccgugugugucc.............................  14  0  S04
..........................................................cguggcuguccgugugCgucc.............................  2  1  S04
..........................................................cguggcuguccguCuguguccg............................  1  1  S04
..........................................................cguggcuguccguguguguccg............................  15  0  S04
..........................................................cguggcuguccgugugCguccg............................  1  1  S04
..........................................................cgugAcuguccguguguguccgc...........................  1  1  S04
..........................................................cguggcuguccguguguguccgG...........................  1  1  S04
...........................................................guggcuguccgugugugu...............................  1  0  S04
...........................................................guggcuguccguguguguc..............................  3  0  S04
...........................................................guggcuguccgugugugucc.............................  1  0  S04
...........................................................guggcuguccguguguguccg............................  6  0  S04
...........................................................guggcuguAcguguguguccgc...........................  1  1  S04
...........................................................guggcuguccguguguguccgc...........................  3  0  S04
...........................................................guggcuguccgugugugucUgc...........................  3  1  S04
............................................................uggcuguccguguguguc..............................  1  0  S04
............................................................uggcuguccgugugugucc.............................  1  0  S04
............................................................uggcuguccguguguguccgc...........................  1  0  S04
............................................................uggcuguccguguguguccgcA..........................  1  1  S04
.............................................................ggcuguccgugugugucc.............................  1  0  S04
.............................................................ggcuguccguguguguccgc...........................  1  0  S04
.............................................................ggcuguccguCuguguccgcgugu.......................  1  1  S04
..............................................................gcuguccguguguguccg............................  2  0  S04
...............................................................cuguccguguguguccCc...........................  1  1  S04
...............................................................cuguccgugugugucUgcgugu.......................  2  1  S04
................................................................uguccgugugugucUgcgugug......................  1  1  S04
................................................................uguccguguguguccgcCugug......................  1  1  S04
..............................................................................cgcguguguccgugugugucU.........  1  1  S04
................................................................................cgugugucUgugugugucc.........  1  1  S04
.................................................................................guguguccgugugugucc.........  1  0  S04
.................................................................................guguguccgugugugucA.........  1  1  S04
.................................................................................guguguccguguguguccgu.......  1  0  S04
.................................................................................guguguccgugCguguccguc......  1  1  S04
.................................................................................guguguccguCuguguccgucc.....  2  1  S04
.................................................................................gugugucUguguguguccgucc.....  1  1  S04
.................................................................................guguUuccguguguguccgucc.....  1  1  S04
..................................................................................uguguccguguguguccguc......  1  0  S04
..................................................................................ugugucUguguguguccgucc.....  1  1  S04
```

```
    Star                                            Mature
agcacacacaugacgucuguggcugucgugucuguccgucagcacacacaggacguccguggcuguccgugugugucgcguguguccgugugugucc guccgcaca
..................................................................................uguguccgugugugucc gucG.....   1   1   S04
..................................................................................uguguccgugugugucc guccg....   1   0   S04
....................................................................................uguccgugugugucc guccgUa..   1   1   S04
........................................................................................cgugugugucc gucGgca..   1   1   S04
........................................................................................cgugugugucc guccgAaca   1   1   S04
```

Provisional ID	:	chrAnn_random_3153785
Score total	:	0
Score for star read(s)	:	-1.3
Score for read counts	:	0
Score for mfe	:	1.2
Score for randfold	:	0
Score for cons. seed	:	
Total read count	:	23
Mature read count	:	23
Loop read count	:	0
Star read count	:	0

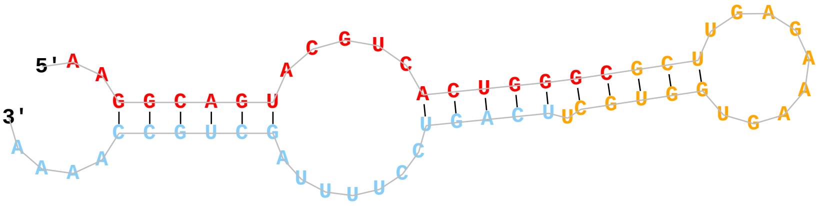

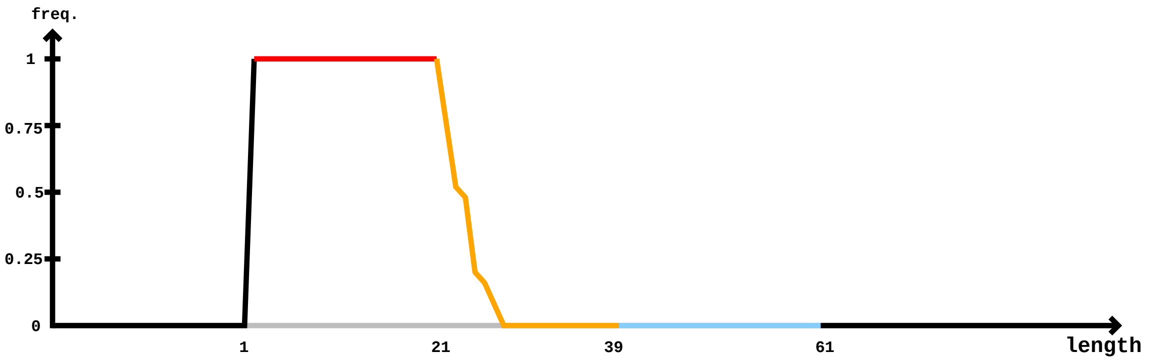

Mature   Star

```
5'- uauuuuaucgaguugauacaaggcaguacgucacugggcgcuugagaaaguggugcuucaguccuuuuagcugccaaaauagacgggagcacuaaugcuucguuugcc   -3'    exp
    .....((((.....)))...(((((.....((((((((((.........))))).)))))).......))))))....((((((..((((....))))..))))))..    reads   mm   sample
    ...................aaggcaguacgucacugggc.....................................................................    2       0    S03
    ...................aaggcaguacgucacugggcgcu..................................................................    3       0    S03

    ...................aaggcaguacgucacugggU.....................................................................    1       1    S04
    ...................aaggcaguacgucacugggc.....................................................................    9       0    S04
    ...................aaggcaguacgucacugggcgc...................................................................    1       0    S04
    ...................aGggcaguacgucacugggcgcu..................................................................    1       1    S04
    ...................aaggcaguacgucacugggcgcu..................................................................    2       0    S04
    ...................aaggcaguacgucacugggcgcG..................................................................    1       1    S04
    ...................aaggcaguacgCcacugggcgcuu.................................................................    1       1    S04
    ...................aaggcaguacgucacugggcgcuuA................................................................    1       1    S04
    ...................aaggcaguacgucacugggcgcuug................................................................    1       0    S04
    ...................aaggcaguacgucacugggcgcuuga...............................................................    2       0    S04
```

Provisional ID	:	chrAnn_random_3157487
Score total	:	0.3
Score for star read(s)	:	-1.3
Score for read counts	:	0
Score for mfe	:	1.6
Score for randfold	:	0
Score for cons. seed	:	
Total read count	:	12
Mature read count	:	11
Loop read count	:	1
Star read count	:	0

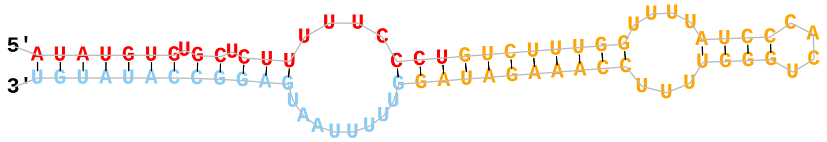

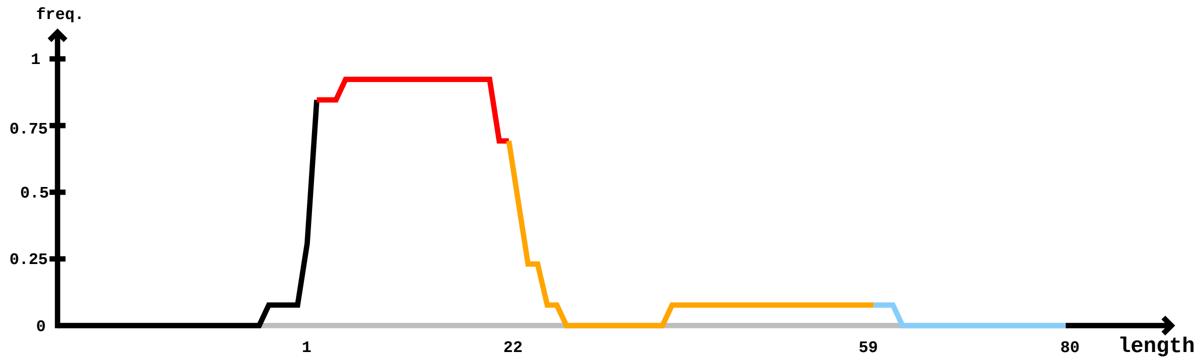

Mature Star

```
5'- naugaucaucaaaugagcaaguagcauaugugugcucuuuuucccugucuuugguuuuaucccacuggguuuuccaaagauagguuuuuaaugaggccauauguuacuuucaca   -3'    exp
    .............(((..((((((((((((.((.(((......(((((((((((....((((....))))...)))))))))))........))))))))))))))))))))..   reads   mm   sample
    ....................guagcauaugugugcucuuuuucc......................................................................   1       0    S03
    ........................cauaugugugcucuuuuucccu....................................................................   1       0    S03
    ........................cauaugugugcucuuuuucccC....................................................................   1       1    S03
    ..............................................................cacuggguuuuccaaagauagguC............................   1       1    S03

    ........................cauaugugugcucuuuuuUccu....................................................................   1       1    S04
    .........................auaugugugcucuuuuucc......................................................................   2       0    S04
    .........................auaugugugcucuuuuucccu....................................................................   2       0    S04
    .........................auaugugugcucuuuuucccA....................................................................   1       1    S04
    .........................auaugugugcucuuuuucccuguU.................................................................   1       1    S04
    .........................auaugugugcucuuuuucccuguc.................................................................   1       0    S04
    ............................ugugugcucuuuuucccugucuu...............................................................   1       0    S04
```

Provisional ID	:	chrC01_974221
Score total	:	1.8
Score for star read(s)	:	3.9
Score for read counts	:	-3.9
Score for mfe	:	1.8
Score for randfold	:	0
Score for cons. seed	:	
Total read count	:	4
Mature read count	:	3
Loop read count	:	0
Star read count	:	1

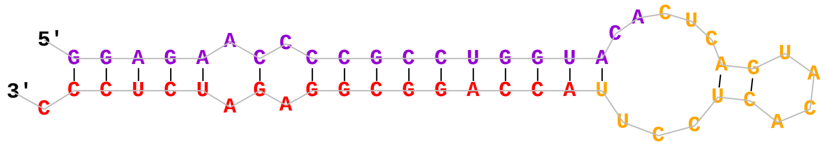

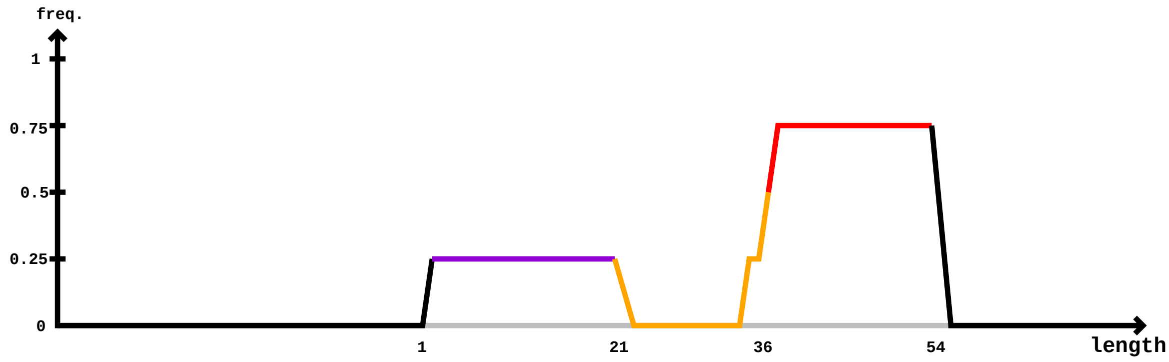

Star Mature

```
5'- acacacacguccccaccgugaucauccgcaacauccuggagaaccccgccugguacacucaguacacuccuuaccaggcggagaucucccaggggaggcucgagucucucc   -3'   obs
    acacacacguccccaccgugaucauccgcaacauccuggagaaccccgccugguacacucaguacacuccuuaccaggcggagaucucccaggggaggcucgagucucucc         exp
    .....((((.......)))).................(((((.(.((((((((((.....((....))...)))))))))).).)))))..((((((((....))))))))   reads   mm   sample
    ........................................................................accaggcggagaucuccc.....................   1       0    S03
    .........................................................................ccaggcggagaucuccca....................   1       0    S03

    .....................................ggagaaccccgccugguaca......................................................   1       0    S04
    ......................................................................uuaccaggcggagaucuccca....................   1       0    S04
```

Provisional ID : chrC01_975061
Score total : 0.4
Score for star read(s) : -1.3
Score for read counts : 0
Score for mfe : 1.7
Score for randfold : 0
Score for cons. seed :
Total read count : 76
Mature read count : 76
Loop read count : 0
Star read count : 0

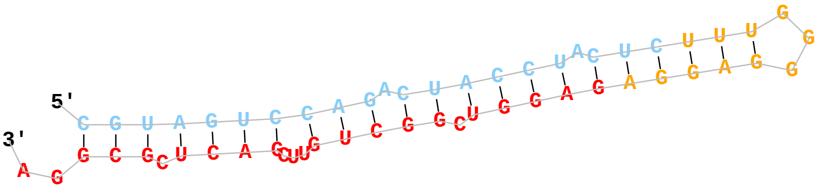

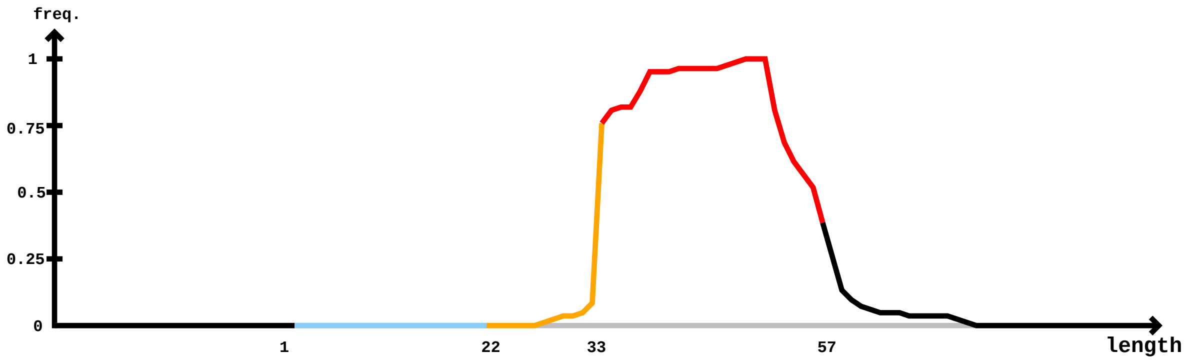

Star Mature

```
5'- uauuaugcaugcacccaggaucucguaguccagacuaccuacucuuuggggaggagaggucggcuguucgacucgcggagagggggaggcgugcccaggacccuaaccgguac   -3'   exp
    ......((((((.(((....(((((((((((((.((((((.((((((...)))))))))).)))))...)))).)).)))).)))...)))))).....(((......)))..   reads   mm   sample
    .................................................ggaggagaggucggcuguucgacu........................................   1   0   S03
    ..................................................gaggagaggucggcuguucgacuU.......................................   1   1   S03
    ......................................................Ugaggucggcuguucgacucgcgg...................................   1   1   S03
    .......................................................gaggucggcuguucgGcu........................................   1   1   S03
    .......................................................gaggucggcuguucgacA........................................   5   1   S03
    .......................................................gaggucggcuguucgacG........................................   1   1   S03
    .......................................................gaggucggcuguucgacGc.......................................   1   1   S03
    .......................................................gaggucggcuguucgacuU.......................................   5   1   S03
    .......................................................gaggucggcuguucgacucA......................................   3   1   S03
    .......................................................gaggucggcuguucgacucU......................................   1   1   S03
    .......................................................gaggucggcuguucgacucgU.....................................   1   1   S03
    .......................................................Uaggucggcuguucgacucgcg....................................   1   1   S03
    .......................................................gaggucggcuguucgGcucgcgg...................................   1   1   S03
    .......................................................gaggucggcuguucgacucgcgC...................................   1   1   S03
    .......................................................gaggucUgcuguucgacucgcgg...................................   1   1   S03
    .......................................................gaggucggcuguucgacucgcggU..................................   2   1   S03
    .......................................................gaggCcggcuguucgacucgcgga..................................   1   1   S03
    .......................................................gaggucggcuguucgacucgcggC..................................   2   1   S03
    .......................................................gaggucggcuguucgacucgcgga..................................   3   0   S03
    ........................................................aggucggcuguucgacuUgcgg...................................   1   1   S03
    ........................................................aggGcggcuguucgacucgcgg...................................   1   1   S03
    .........................................................Ugucggcuguucgacucgcgg...................................   1   1   S03
    ...........................................................ucggcuguucgacucgcU....................................   1   1   S03
    ...........................................................ucggcuguucgacuUgcgg...................................   1   1   S03
    ...........................................................ucggcuguucgacucgcggaga................................   1   0   S03
    ............................................................cggcuguucgacucgcggC..................................   1   1   S03
    ............................................................cggcuguucgacucgcggagaA...............................   1   1   S03
    ...............................................................cuguucgacucgcggagaggggga..........................   1   0   S03

    ...................................................aggagaggucggcuguucgacucg......................................   1   0   S04
    .....................................................gagaggucggcuguucgacu........................................   1   0   S04
    ......................................................agaggucggcuguucgacucgU.....................................   1   1   S04
    ......................................................agaggucggcuguucgacucgcgg...................................   1   0   S04
    .......................................................gaggucggcuguucgacG........................................   1   1   S04
```

Star Mature

```
uauuaugcaugcacccaggaucucguaguccagacuaccuacucuuuggggaggagaggucggcuguucgacucgcggagagggggaggcgugcccaggacccuaaccgguac
.......................................................gaggucggcuguucgaAu........................................	1	1	S04
.......................................................gaggucggcuguucgacA........................................	4	1	S04
.......................................................gaggAcggcuguucgacu........................................	1	1	S04
.......................................................gaggucggcuguucgacuU.......................................	3	1	S04
.......................................................gaggucggcuguucgacucA......................................	1	1	S04
.......................................................gaggucggcuguucgacucgU.....................................	1	1	S04
.......................................................gaggucggcuguucgacucAc.....................................	1	1	S04
.......................................................gaggucggcuguucgacucgcU....................................	2	1	S04
.......................................................gaggucggcuguucgacucgGgg...................................	1	1	S04
.......................................................gaggucggcuguucgacucgcggC..................................	1	1	S04
.......................................................gaggucggcuguucgacucgcgga..................................	6	0	S04
.......................................................gaggucggcuguucgacucgcggU..................................	3	1	S04
........................................................aggucggcuguucgacucgcgC...................................	1	1	S04
........................................................aggucggcuguucgacucgcgga..................................	1	0	S04
...........................................................ucggcuguucgacucgcggaga................................	1	0	S04
...........................................................ucggcuguucgacucgcggagagg..............................	1	0	S04
............................................................cggcuguucgacucgcggaA.................................	1	1	S04
............................................................cgAcuguucgacucgcggaga................................	1	1	S04
............................................................cggcuguucgacucgcggagag...............................	1	0	S04
............................................................cggcuguucgacucgcggagaAgg.............................	1	1	S04
....................................................................cgacucgcggagagggggaggcgu.....................	1	0	S04
.....................................................................gacucgcggagagggggaggcgug....................	1	0	S04
......................................................................acucgcggagagggggaggcgugA...................	1	1	S04
```

Provisional ID	:	chrC01_979632
Score total	:	1.2
Score for star read(s)	:	-1.3
Score for read counts	:	0
Score for mfe	:	2.5
Score for randfold	:	0
Score for cons. seed	:	
Total read count	:	1397
Mature read count	:	932
Loop read count	:	465
Star read count	:	0

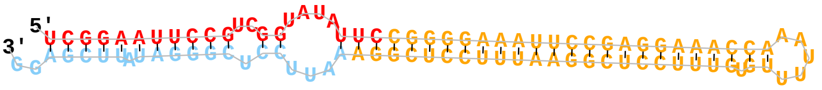

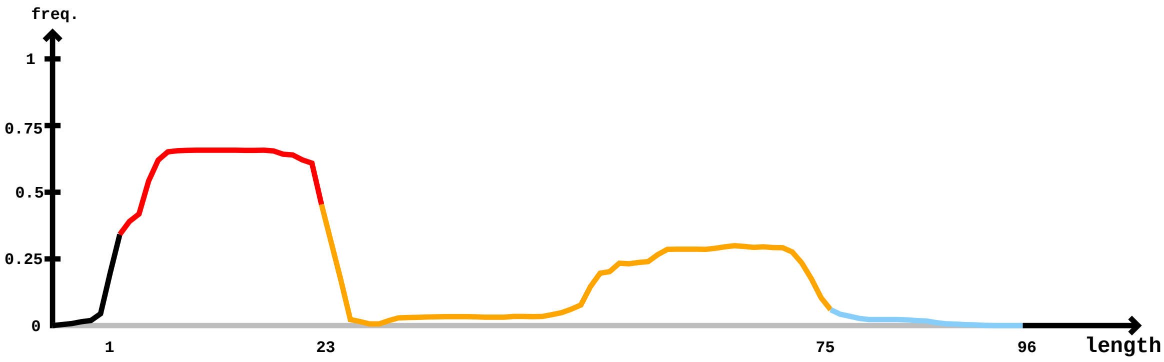

Mature **Star**

```
5'- uuuccucggaauuccgucgguauauuccggggaaauuccgaggaaaccaaauuuuguguuuccucggaauuuccucggaaauuccucgggauauucgaggauuucauuuua   -3'   exp
    ..(((((((((((((((..((....((((((((((((((((((((((((.....)).)))))))))))))))))))))))...)).)))))).))))))))..........   reads   mm   sample
    uuuccucggaauuccgucggu..........................................................................................   1   0   S03
    uuuccucggaauuccgucgguC.........................................................................................   1   1   S03
    uuuccucggaauuccgucggua.........................................................................................   1   0   S03
    uuuccucggaauuccgucgguaua.......................................................................................   1   0   S03
    .uuccucggaauuccgucgguauG.......................................................................................   1   1   S03
    .uuccucggaauuccgucgguauau......................................................................................   1   0   S03
    .uuccucggaauucGgucgguauau......................................................................................   1   1   S03
    ...ccucggaauuccgucggua.........................................................................................   1   0   S03
    ...ccucggaauuccgucgguauauu.....................................................................................   2   0   S03
    ...ccucggaauuccgucgguauauuc....................................................................................   12   0   S03
    ....cucggaauuccgucggua.........................................................................................   7   0   S03
    ....cucggaauuccgucgguau........................................................................................   1   0   S03
    ....cucggaauuccgucgguaua.......................................................................................   1   0   S03
    ....cucggaauuccgucgguauG.......................................................................................   1   1   S03
    ....cucggaauuccgucgguauau......................................................................................   2   0   S03
    ....cucggaauuccgucgguauGuu.....................................................................................   1   1   S03
    ....cucggUauuccgucgguauauu.....................................................................................   2   1   S03
    ....cucggaauuccgucgguauauu.....................................................................................   50   0   S03
    ....cucggaauuccgucgguauauuc....................................................................................   20   0   S03
    ....cucggaauuccgucgguauauucc...................................................................................   14   0   S03
    .....ucggaauuccgucgguau........................................................................................   1   0   S03
    .....ucggaauuccgucgguaua.......................................................................................   7   0   S03
    .....ucggaauuccgucgguauG.......................................................................................   1   1   S03
    .....ucggaauuccgucgguauau......................................................................................   2   0   S03
    .....ucggaauuccgucgguauauu.....................................................................................   20   0   S03
    .....ucggaauuccgucgguauauuc....................................................................................   53   0   S03
    .....ucggaauuccgucgguauauucc...................................................................................   1   0   S03
    ......cggaauuccgucgguaua.......................................................................................   1   0   S03
    ......cggaauuccgucgguauau......................................................................................   4   0   S03
    ......cggaauuccgucgguauauu.....................................................................................   9   0   S03
    ......cggaauuccgucgguauauuc....................................................................................   15   0   S03
    ......cggaauuccguGgguauauuc....................................................................................   1   1   S03
    ......cggaauuccgucgguauauucc...................................................................................   2   0   S03
    .......ggaauuccgucgguauauu.....................................................................................   16   0   S03
```

Mature **Star**

```
uuuccucggaauuccgucgguauauuccggggaaauuccgaggaaaccaaauuuuguguuuccucggaauuuccucggaaauuccucgggauauucgaggauuucauuuua
.......ggaauuccgucgguauauuc....................................................................................	4	0	S03
........gaauuccgucgguauauu.....................................................................................	6	0	S03
........gaauuccgucgguauauuc....................................................................................	2	0	S03
........gaauuccgucgguauauucc...................................................................................	10	0	S03
........gaauuccgucgguauauuccg..................................................................................	61	0	S03
........gaauuccgucgguauauuccUg.................................................................................	1	1	S03
........gaauuccgucggAauauuccgg.................................................................................	1	1	S03
.........aauuccgucgguauauuc....................................................................................	10	0	S03
.........aauuccgucgguauauucc...................................................................................	20	0	S03
.........aauuccgucgguauauuccC..................................................................................	2	1	S03
.........aauuccgucgguauauuccg..................................................................................	30	0	S03
.........aauuccgucgguauauuccAg.................................................................................	1	1	S03
.........aauuccgucggAauauuccggg................................................................................	8	1	S03
..........auuccgucgguauauucc...................................................................................	8	0	S03
..........auuccgucgguauauuccg..................................................................................	9	0	S03
..........auuccgucggAauauuccggg................................................................................	2	1	S03
...........uuccgucgguauauuccg..................................................................................	1	0	S03
...........uuccgucggAauauuccggg................................................................................	3	1	S03
.....................auauuccggggGaauuccgagg....................................................................	2	1	S03
...............................gaaauuccgaggaaaccaaauu..........................................................	1	0	S03
................................aaauuccgaggaaaccaaauuuug.......................................................	1	0	S03
.................................aauuccgaggaaaccaaauu..........................................................	1	0	S03
.................................aauuccgaggaaaccaaauuuug.......................................................	1	0	S03
.................................aauuccgaggaaaccaaauuuugu......................................................	6	0	S03
.................................aauuccgaggaaaccaaauuuugC......................................................	1	1	S03
..................................auuccgaggaaaccaaauuuug.......................................................	1	0	S03
..................................auuccgaggaaaccaaauuuugug.....................................................	3	0	S03
..................................auuccgaggaaaccaaauuuuguU....................................................	1	1	S03
...................................uuccgaggaaaccaaauuuugugu....................................................	1	0	S03
.....................................ccgaggaaaccaaauuuugu......................................................	1	0	S03
.......................................gaggaaaccaaauuuugug.....................................................	1	0	S03
..............................................ccaaauuuuguguuuccucggaau.........................................	2	0	S03
...............................................caaauuuuguguuuccucU.............................................	1	1	S03
.................................................aauuuuguguuuccucggaauu........................................	1	0	S03
..................................................auuuuguguuuccucggaauuucc.....................................	3	0	S03
...................................................uuuuguguuuccucggaauuuccu....................................	2	0	S03
...................................................uuuuguguuuccucggaauuuccC....................................	1	1	S03
....................................................uuuguguuuccucggaauC........................................	1	1	S03
....................................................uuuguguuuccucggaauuuc......................................	1	0	S03
....................................................Guuguguuuccucggaauuuccuc...................................	1	1	S03
....................................................uuuguguuuccucggaauuuccuc...................................	6	0	S03
.....................................................uuguguuuccucggaauu........................................	1	0	S03
.....................................................uuguguuuccucggaauuuccucg..................................	9	0	S03
.....................................................uuguguuuccucggaauuuccucC..................................	1	1	S03
.....................................................Cuguguuuccucggaauuuccucg..................................	1	1	S03
......................................................uguguuuccucggaauuuccu....................................	4	0	S03
......................................................uguguuuccucggaauuuccuc...................................	8	0	S03
......................................................uguguuuccucggaauuuccucg..................................	4	0	S03
......................................................uguguuuccucggaauuuccAcg..................................	1	1	S03
......................................................uguguuuccucggaauuuccucU..................................	1	1	S03
......................................................uguguuuccCcggaauuuccucgg.................................	1	1	S03
......................................................uguguuuccucggaauuuccucgg.................................	22	0	S03
......................................................uguguuuccucggaaCuuccucgg.................................	1	1	S03
......................................................uguguuuccucggaauCuccucgg.................................	1	1	S03
......................................................uguguuuccucggaauuuccucgC.................................	1	1	S03
.......................................................guguuuccucggaauuuccuc...................................	2	0	S03
.......................................................guguuuccucggaauuuccucg..................................	4	0	S03
.......................................................guguuuccucggaauuuccucgU.................................	1	1	S03
.......................................................guguuuccucggaauuuccucgg.................................	5	0	S03
.......................................................guguuuccucggaauuuccucgga................................	11	0	S03
.......................................................guguuuccucggaauuuccucggU................................	1	1	S03
.......................................................guguuuccGcggaauuuccucgga................................	1	1	S03
.......................................................guguuuccucggaauuuccucggaa...............................	1	0	S03
........................................................uguuuccucggaauuucc.....................................	1	0	S03
........................................................uguuuccucggaauuuccu....................................	1	0	S03
........................................................uguuuccucggaauuuccucg..................................	3	0	S03
.........................................................guuuccucggaauuuccu....................................	4	0	S03
.........................................................guuuccucggaauuuccuA...................................	1	1	S03
.........................................................guuuccucggaauuuccuc...................................	2	0	S03
.........................................................guuuccGcggaauuuccuc...................................	1	1	S03
```

Mature **Star**

uuuccucggaauuccgucgguauauuccggggaaauuccgaggaaaccaaauuuuguguuuccucggaauuuccucggaaauuccucgggauauucgaggauuucauuuua			
....guuuccucggaauuuccucg..................................	10	0	S03
....guuuccucggGauuuccucg..................................	1	1	S03
....guuuccucggaauuuccucgg.................................	8	0	S03
....guuuccucggaauuuccucggC................................	2	1	S03
....guuuccucggaauuuccucgga................................	3	0	S03
....guuuccucggaauuuccucggaa...............................	1	0	S03
....uuuccucggaauuuccuc...................................	2	0	S03
....uuuccucggaauuuccucg..................................	3	0	S03
....uuccucggaauuuccucg..................................	5	0	S03
....uccucggaauuuccucggaa...............................	1	0	S03
....uccucggaauuuccucggaaauu............................	1	0	S03
....ccucggaauuuccucgga................................	7	0	S03
....ccucggaauuuccucggaa...............................	1	0	S03
....ccucggaauuuccucggaaa..............................	1	0	S03
....ccucggaauuuccucggaaau.............................	1	0	S03
....cucggaauuuccucggaa...............................	7	0	S03
....cucggaauuuccucggaaa..............................	3	0	S03
....cucggaauuuccucggaaC..............................	1	1	S03
....cucggaauuuccucggaaau.............................	2	0	S03
....aaAuuccucggaaauuccucg.......................	1	1	S03
....aauuuccucggaaUuuccucggga....................	1	1	S03
....aauuuccucggaaauuccucggga....................	2	0	S03
....aauuuccucggaaauuccucgggG....................	1	1	S03
....auuuccucggaaauuccucgg......................	1	0	S03
....auuuccucggaaauuccucggg.....................	2	0	S03
....aAuuccucggaaauuccucggg.....................	1	1	S03
....uuuccucggaaauuccuc........................	1	0	S03
....uuuccucggaaauuccucg.......................	1	0	S03
....uuuccucggaaauuccucggg.....................	1	0	S03
....uuccucggaaauuccucggg.....................	1	0	S03
....uuccucggaaauuccucgggau...................	1	0	S03
....ccucggaaauuccucggg.....................	1	0	S03
....ccucggaaauuccucgggau...................	1	0	S03
uuuccucggaauuccguc...	1	0	S04
uuuccucggaauuccgucggu..	4	0	S04
uuuccucggaauuccgucgguaua...	2	0	S04
.uuccucggaauuccgucggua...	1	0	S04
.uuccucggaauuccgucgguauG...	4	1	S04
.uuccucggaauuccgucgguaua...	1	0	S04
.uuccucggaauuccgucgguauau..	1	0	S04
..uccucggaauuccgucggu..	1	0	S04
..uccucggaauuccgucggua...	1	0	S04
..uccucggaauuccgucgguauauu...	4	0	S04
..ucAucggaauuccgucgguauauu...	1	1	S04
...ccucggaauuccgucgguauauu...	3	0	S04
...ccucggaauuccgucgguauauuc..	19	0	S04
....cucggaauuccgucggua...	6	0	S04
....cucggaauuccgucgguaua...	1	0	S04
....cucggaauuccgucgguauau..	3	0	S04
....cucggGauuccgucgguauauu...	1	1	S04
....cucggaauuccgucgguauauu...	60	0	S04
....cucggaauuccgucgguauauuc..	23	0	S04
....cucggaauuccgucgguauauuG..	1	1	S04
....Nucggaauuccgucgguauauucc...	1	1	S04
....cucggaauuccgucgguauauucc...	29	0	S04
.....ucggaauuccgucgguau..	2	0	S04
.....ucggaauuccgucgguaua...	4	0	S04
.....ucggaauuccgucgguaGa...	1	1	S04
.....ucggaauuccgucgguauau..	2	0	S04
.....ucggaauuccgucgguauauu...	30	0	S04
.....ucggaauuccgucgguauauuc..	85	0	S04
.....Ncggaauuccgucgguauauuc..	1	1	S04
.....ucggaauuccgGcgguauauuc..	1	1	S04
......cggaauuccgucgguaua...	1	0	S04
......cggaauuccgucgguauau..	1	0	S04
......cggaauuccgucgguauauu...	8	0	S04
......cggaauuccgucgguauauuc..	23	0	S04
......cggaauuccgucgguauauuG..	1	1	S04
......cggaauuccgucgguauauucc...	3	0	S04

Mature / Star

```
uuuccucggaauuccgucgguauauuccggggaaauuccgaggaaaccaaauuuuguguuuccucggaauuuccucggaaauuccucgggauauucgaggauuucauuuua
......cggaauuccgucgguauauuccC..................................................................................	1	1	S04
......cggaauuccgucgguauauuccg..................................................................................	1	0	S04
.......ggaauuccgucgguauau......................................................................................	1	0	S04
.......ggaauuccgucgguauauu.....................................................................................	13	0	S04
.......ggaauuccgucgguauauuc....................................................................................	5	0	S04
.......ggaauuccgucgguauauuccg..................................................................................	1	0	S04
.......ggaauuccgucgguauauuccggg................................................................................	1	0	S04
........gaauuccgucgguauauu.....................................................................................	1	0	S04
........gaauuccgucgguauauuc....................................................................................	9	0	S04
........gaauuccgucgguauauucc...................................................................................	13	0	S04
........gaauuccgucgguauauuccg..................................................................................	74	0	S04
........gUauuccgucgguauauuccg..................................................................................	1	1	S04
........gaauuccgucgguauauuccgg.................................................................................	2	0	S04
.........aauuccgucgguauauuc....................................................................................	2	0	S04
.........aauuccgucgguauauucc...................................................................................	16	0	S04
.........Nauuccgucgguauauuccg..................................................................................	1	1	S04
.........aauuccgGcgguauauuccg..................................................................................	1	1	S04
.........aauuccgucgguauauuccC..................................................................................	1	1	S04
.........aauuccgucgguauauuccg..................................................................................	21	0	S04
.........aauuccgucgguauauuccAg.................................................................................	1	1	S04
.........aauuccgucgguauauuccgg.................................................................................	1	0	S04
..........auucGgucgguauauucc...................................................................................	1	1	S04
..........auuccgucgguauauucc...................................................................................	9	0	S04
..........auuccgucgguauauuccg..................................................................................	12	0	S04
..........auuccgucgguauauuUcgg.................................................................................	1	1	S04
..........auuccgucgguauauuccgC.................................................................................	1	1	S04
..........auuccgucgguauauuccAg.................................................................................	1	1	S04
..........auuccgucgguauauuccgggA...............................................................................	1	1	S04
...........uuccgucgguauauuccg..................................................................................	2	0	S04
............uccgucgguauauuUcgg.................................................................................	1	1	S04
............uccgucgguauauuccgggUa..............................................................................	1	1	S04
.............ccgucgguauauuccgggUa..............................................................................	1	1	S04
....................uauauuccggggaaauuccgaC.....................................................................	1	1	S04
............................ggggaaauuccgaggaaCc................................................................	1	1	S04
............................ggggaaauuccgaggaaacA...............................................................	1	1	S04
.................................aauuccgaggaaaccaaauuuugu......................................................	9	0	S04
.................................aauuccgaggaaaccaaauuuugC......................................................	2	1	S04
..................................auuccgaggaaaccaaauuuugCg.....................................................	1	1	S04
..................................auuccgaggaaaccaaauuuugug.....................................................	9	0	S04
...................................uuccgaggaaaccaaauuuuguU.....................................................	1	1	S04
....................................uccgaggaaaccaaauuuug.......................................................	1	0	S04
.....................................ccgaggaaaccaaauuuugugu....................................................	1	0	S04
......................................cgaggaaaccaaauuuugugu....................................................	1	0	S04
..............................................ccaaauuuuguguuuccucggaaG.........................................	1	1	S04
..............................................ccaaauuuuguguuuccucggaau.........................................	1	0	S04
..................................................auuuuguguuuccucggaau.........................................	1	0	S04
..................................................auuuuguguuuccucggaauu........................................	3	0	S04
..................................................auuuuguguuuccucggaauuu.......................................	1	0	S04
..................................................auuuuguguuuccucggaauuuc......................................	1	0	S04
..................................................Uuuuuguguuuccucggaauuucc.....................................	1	1	S04
...................................................uuuuguguuuccucggaau.........................................	1	0	S04
...................................................uuuuguguuuccucggaauuuc......................................	3	0	S04
...................................................uuuuguguuuccucggaauuuccC....................................	1	1	S04
...................................................uuuuguguuuccucggaauuuccu....................................	3	0	S04
....................................................uuuguguuuccucggaauuuccu....................................	1	0	S04
....................................................uuuguguuuccucggaauuuccuA...................................	1	1	S04
....................................................uuuguguuuccucggaauuuccuc...................................	8	0	S04
.....................................................uuguguuuccucggaauuuccu....................................	1	0	S04
.....................................................uuguguuuccucggaauuuccuc...................................	6	0	S04
.....................................................uuguguuuccucggaauuuccucg..................................	2	0	S04
.....................................................uugCguuuccucggaauuuccucg..................................	1	1	S04
.....................................................Guguguuuccucggaauuuccucg..................................	2	1	S04
......................................................uguguuuccucggaauuuccu....................................	1	0	S04
......................................................uguguuuccucggaauuuccuc...................................	15	0	S04
......................................................Aguguuuccucggaauuuccucg..................................	1	1	S04
......................................................uguguuuccucggaauuuccucg..................................	4	0	S04
......................................................uguguuuccucggaauuuccucU..................................	1	1	S04
......................................................uguguuuccucggaauuuccucgg.................................	30	0	S04
......................................................ugugGuuccucggaauuuccucgg.................................	1	1	S04
......................................................ugCguuuccucggaauuuccucgg.................................	2	1	S04
```

Mature **Star**

uuuccucggaauuccgucgguauauuccggggaaauuccgaggaaaccaaauuuuguguuuccucggaauuuccucggaaauuccucgggauauucgaggauuucauuuua			
....uguguuuccucggaauuuccucgU.................................	3	1	S04
....guguuuccucggaauuuccu....................................	1	0	S04
....guguuuccucggaCuuuccuc...................................	1	1	S04
....guguuuccucggaauuuccuc...................................	2	0	S04
....guguuuccucggaauuuccucg..................................	9	0	S04
....guguuuccucggaauuuccucC..................................	1	1	S04
....guguuuccucggaauuuccucgg.................................	14	0	S04
....guguuuccucggaauCuccucgg.................................	1	1	S04
....guguuuccucggGauuuccucgga................................	1	1	S04
....guguuuccucggaauuuccucgga................................	17	0	S04
....uguuuccucggaauuuccuc...................................	1	0	S04
....uguuuccucggaauuuccucg..................................	3	0	S04
....uguuuccucggaauuuccucU..................................	1	1	S04
....uguuuccucggaauuuccucgC.................................	1	1	S04
....uguuuccucggaauuuccucgU.................................	1	1	S04
....uguuuccucggaauuuccucgg.................................	1	0	S04
....guuuccucggaauuuccu....................................	4	0	S04
....guuuccucggaauuuccuc...................................	4	0	S04
....guuuccucggaauuuccucg..................................	10	0	S04
....guuuccucggaauuuccucgg.................................	9	0	S04
....guuuccucggaauuuccucgga................................	3	0	S04
....guuuccucggaauuuccucggC................................	2	1	S04
....uuuccucggaauuuccucg..................................	5	0	S04
....uuuccucggaauuuccucgga................................	3	0	S04
....uuccucggaauuuccucg..................................	4	0	S04
....uuccucggaauuuccucgga................................	1	0	S04
....uccucggaauuuccucggaaa..............................	1	0	S04
....uAcucggaauuuccucggaaau.............................	1	1	S04
....uccucggaauuuccucggaaauu............................	1	0	S04
....ccucggaauuuccucgga................................	13	0	S04
....ccGcggaauuuccucgga................................	1	1	S04
....ccucggaauuuccucggaa...............................	6	0	S04
....ccucggaauuuccucggaaa..............................	1	0	S04
....ccucggaauuuccucggaaC..............................	1	1	S04
....ccucggaauuuccucggaCa..............................	1	1	S04
....ccucggaauuuccucggaaau.............................	4	0	S04
....ccucggaauuuccucggaaauu............................	1	0	S04
....cucggaauuuccucggaa...............................	8	0	S04
....cucggaauuuccucggaaa..............................	2	0	S04
....cucggaauuuccucggaaau.............................	3	0	S04
....cucggaauuuccucggaaauC............................	1	1	S04
....cucggaauuuccucggaaauu............................	2	0	S04
....ucggaauuuccucggaaau.............................	1	0	S04
....aauuuccucggaaauuccucggg.....................	1	0	S04
....auuuccucggaaCuuccuc........................	1	1	S04
....auuuccucggaaauuccucg.......................	1	0	S04
....auuuccucggaaUuuccucggg.....................	1	1	S04
....auuuccucggaaauuccucgggauG..................	1	1	S04
....uuuccucggaaauuccucg.......................	1	0	S04
....uuuccucggaaauuccucgg......................	1	0	S04
....uuuccucggaaauuccucgggC....................	1	1	S04
....uccucggaaauuccucggg.....................	1	0	S04
....ccucggaaauuccucgggaua..................	2	0	S04
....cucggaaauuccucggga....................	1	0	S04
....ucggaaauuccucgggauau.................	1	0	S04
....ucggaaauuccucgggauauu................	2	0	S04
....ucggaaauuccucgggauauCc...............	1	1	S04
....cggaaauuccucgggauauu................	1	0	S04

Provisional ID	:	chrC01_988803
Score total	:	1.4
Score for star read(s)	:	-1.3
Score for read counts	:	0
Score for mfe	:	2.7
Score for randfold	:	0
Score for cons. seed	:	
Total read count	:	15
Mature read count	:	6
Loop read count	:	9
Star read count	:	0

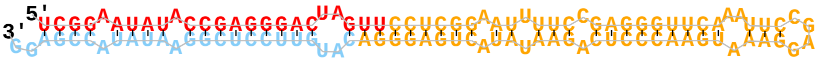

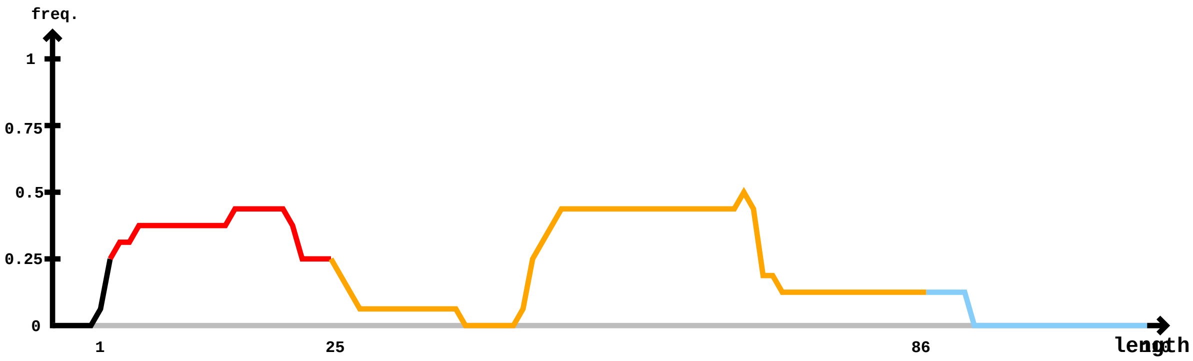

Mature **Star**

```
5'- acccucggaauauaccgagggacuaguuccucggaauuuuccgaggguucaaauuccgaggaaaugaacccucagaauauacugagggacauguuccucggaauauaccgagggg   -3'    exp
    .((((((.((((.(((((((((..((((((((.((.(((.((((((((..(((....))).)))))))))).))).)).)))))))))..))))))))).)))).)))))))   reads   mm   sample
    ................................................ucaaauuccgaggaaaugaacccA..........................................   1       1    S03
    ................................................uAaaauuccgaggaaaugaacccu..........................................   1       1    S03
    ......................................................................cucagaauauacugagggacaugu....................   1       0    S03

    ...Uucggaauauaccgagggacu..........................................................................................   1       1    S04
    ....ucggaauauaccgagggacu..........................................................................................   1       0    S04
    ....ucggaauauaccgagggacuaguu......................................................................................   2       0    S04
    .....cggaauauCccgagggac...........................................................................................   1       1    S04
    .......gaauauaGcgagggacuaguucc....................................................................................   1       1    S04
    .................agggacuaguuccucggaauuuuc.........................................................................   1       0    S04
    ...............................................uucaaauuccgaggaaaugaaccc...........................................   1       0    S04
    ................................................ucaaauuccgaggaaaugaacccu..........................................   1       0    S04
    .................................................cGaauuccgaggaaaugaacc............................................   1       1    S04
    ..................................................aaauuccgaggaaaugaacccA..........................................   1       1    S04
    ...................................................aauuccAaggaaaugaacccuca........................................   1       1    S04
    ......................................................................cucGgaauauacugagggacaugu....................   1       1    S04
```

Provisional ID	:	chrC01_990042
Score total	:	1.5
Score for star read(s)	:	-1.3
Score for read counts	:	0
Score for mfe	:	2.8
Score for randfold	:	0
Score for cons. seed	:	
Total read count	:	174
Mature read count	:	131
Loop read count	:	7
Star read count	:	36

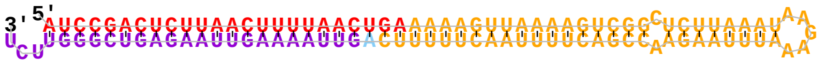

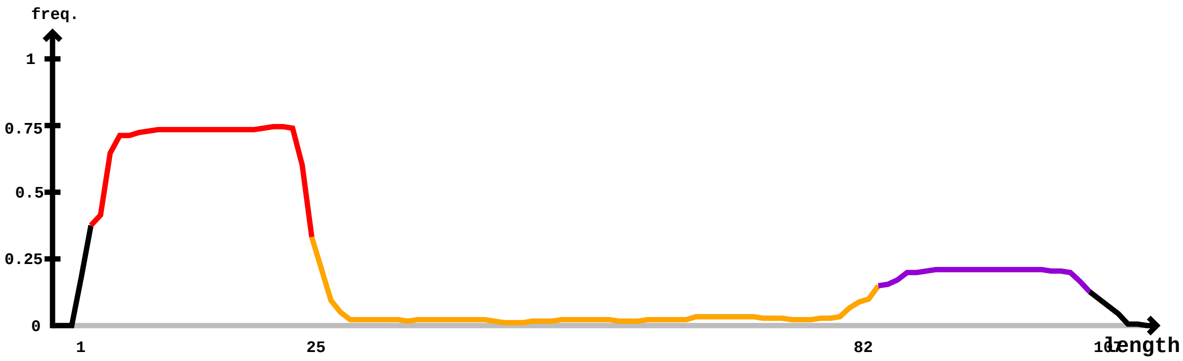

Mature **Star**

```
5'- aaauccgacucuuaacuuuuaacugaaaaaguuaaaagucggcucuuaaauaagaaauuuaagaaccgacuuuuaacuuuuucaguuaaaaguuaagagucggguucuuauau -3'   obs
    aaauccgacucuuaacuuuuaacugaaaaaguuaaaagucggcucuuaaauaagaaauuuaagaaccgacuuuuaacuuuuucaguuaaaaguuaagagucggguucuuauau        exp
    .(((((((((((((((((((((((((((((((((((((((((.((((((((.....)))))))).)))))))))))))))))))))))))))))))))))))))))).......   reads   mm   sample
    .aauccgacucuuaacuuuuaacu.........................................................................................   6   0   S03
    .aauccgacucuuaacuuuuaacA.........................................................................................   1   1   S03
    .aauccgacucuuaacuuuuaacug........................................................................................   2   0   S03
    ..auccgacucuuaacuuuuaacu.........................................................................................   2   0   S03
    ..auccgacucuuaacuuuuaacug........................................................................................   1   0   S03
    ..auccgacucuuaacuuuuaacugG.......................................................................................   2   1   S03
    ..auccgacucuuaacuuuuaacuga.......................................................................................   2   0   S03
    ...uccgacucuuaacuuuuaacug........................................................................................   1   0   S03
    ...uccgacucuuaacuuuuaacuga.......................................................................................   1   0   S03
    ....ccgacucuuaacuuuuaacu.........................................................................................   2   0   S03
    ....ccgacucuuaacuuuuaacug........................................................................................   8   0   S03
    ....ccgacucuuaacuuuuaacugaG......................................................................................   1   1   S03
    ....ccgacucuuaacuuuuaacugaa......................................................................................   3   0   S03
    .....cgacucuuaacuuuuaacC.........................................................................................   1   1   S03
    .....cgacucuuaacuuuuaacuga.......................................................................................   2   0   S03
    .....cgacucuuaacuuuuaacugaa......................................................................................   2   0   S03
    .....cgacucuuaacuuuuaacugaaaa....................................................................................   1   0   S03
    .......acucuuaacuuuuaacugaaaa....................................................................................   1   0   S03
    ........Nucuuaacuuuuaacugaaaa....................................................................................   1   1   S03
    .........ucuuaacuuuuaacugaaaaaguu................................................................................   1   0   S03
    .....................acugaaaaaguuaaaagucggcuU....................................................................   1   1   S03
    ...................................................aagaGauuuaagaaccgacuuuua......................................   1   1   S03
    .................................................................................ucaguuaaaaguuaagagucgggu........   2   0   S03
    ..................................................................................caguuaaaaguuaagagucgggGu.......   1   1   S03
    ..................................................................................caguuaaaaguuaagagucggguu.......   3   0   S03
    ...................................................................................aguuaaaaguuaagagucggguu.......   1   0   S03
    ...................................................................................aguuaaaaguuaagagucgggu A.......   1   1   S03
    ....................................................................................guuaaaaguuaagagucggguucG.....   1   1   S03
    ....................................................................................guuaaaaguuaagagucggguucu.....   3   0   S03
    ......................................................................................uaaaaguuaagagucggguucuua...   1   0   S03
    .......................................................................................aaaaguuaagagucggguucG.....   1   1   S03
    .......................................................................................aaaaguuaagagucggguucuua...   1   0   S03
    .......................................................................................aaaaguuaagagucggguuAuua...   1   1   S03
```

Mature **Star**

```
aaauccgacucuuaacuuuuaacugaaaaguuaaaaagucggcucuuaaauaagaaauuuaagaaccgacuuuuaacuuuuucaguuaaaaguuaagagucggguucuuauau
..........................................................................................aaguuaagagucggguucuua...   1   0   S03
..........................................................................................aaguuaagagucggguucuuaua.   1   0   S03
...........................................................................................aguuaagagucggguucC.....   1   1   S03

.aauccgacucuuaacuuuuaac...........................................................................................   1   0   S04
.aauccgacucuuaacuuuuaacu..........................................................................................   6   0   S04
.aauccgacucuuaacuuuuaacA..........................................................................................   1   1   S04
.aauccgacucuuaacuuuuaacG..........................................................................................   1   1   S04
.aauccgacucCuaacuuuuaacu..........................................................................................   1   1   S04
.aaucUgacucuuaacuuuuaacug.........................................................................................   1   1   S04
.aauccgacucuuaacuuuuaacug.........................................................................................   12  0   S04
.aauccgacucuuaacuuuuaacuga........................................................................................   1   0   S04
..auccgacucuuaacuuuuaacG..........................................................................................   1   1   S04
..auccgacucuuaacuuuuaacuU.........................................................................................   1   1   S04
..auccgacucuuaacuuuuaacug.........................................................................................   3   0   S04
..auccgGcucuuaacuuuuaacug.........................................................................................   1   1   S04
..auccgacucuCaacuuuuaacug.........................................................................................   1   1   S04
..auccgacucuuaacuuuuaacugG........................................................................................   2   1   S04
..auccgacucuuaacuuuuaacuga........................................................................................   19  0   S04
...uccgacucuuaacuuuuaacu..........................................................................................   2   0   S04
...uccgacucuuaacuuuuaacuga........................................................................................   1   0   S04
...uccgacucuuaacuuuuaacugaa.......................................................................................   2   0   S04
....ccgacucuuaacuuuuaacu..........................................................................................   1   0   S04
....ccgacucCuaacuuuuaacug.........................................................................................   1   1   S04
....ccgacucuuaacuuuuaacug.........................................................................................   14  0   S04
....ccgacucuuUacuuuuaacug.........................................................................................   1   1   S04
....ccgacucuuaacuuCuaacug.........................................................................................   1   1   S04
....ccgacucuuaacuuuuaacuga........................................................................................   2   0   S04
....cUgacucuuaacuuuuaacugaaa......................................................................................   1   1   S04
....ccgacucuuaacuuuuaacugaaa......................................................................................   6   0   S04
....ccgacucuuaacuuuuaacugaaaa.....................................................................................   1   0   S04
.....cgacucuuaacuuuuaacug.........................................................................................   1   0   S04
.....cgacucuuaacuuuuaacuga........................................................................................   3   0   S04
.....cgacucuuaacuuuuaacugaaaa.....................................................................................   1   0   S04
.....cgacucuuaacuuuuaacugaaaaaguuaa...............................................................................   1   0   S04
.......acucuuaacuuuuaacugaaa......................................................................................   1   0   S04
....................aacugaaaaaguuaaaaagucggcC.....................................................................   1   1   S04
..................................aaaagucggcucuuaaauaagaaa........................................................   1   0   S04
.....................................agucggcucuuaaauaagaaauuu.....................................................   1   0   S04
.................................................aauaagaGauuuaagaaccgacuu.........................................   1   1   S04
.............................................................aagaaccgacuuuuaacuuuuuc..............................   1   0   S04
.............................................................aagaaccgacuuuuaacuuuuuca.............................   1   0   S04
..................................................................ccgacuuuuaacuuuuucaC............................   1   1   S04
..................................................................ccgacuuuuaacuuuuucaguuaa........................   1   0   S04
...............................................................................uuuucaguuaaaaguuaagagucg...........   1   0   S04
.................................................................................uucaguuaaaaguuaagagucggg.........   1   0   S04
..................................................................................ucaguuaaaaguuaagagucgggu........   3   0   S04
..................................................................................ucaguuaaaaguuaagagucggguG.......   1   1   S04
....................................................................................aguuaaaaguuaagagucggguuc......   1   0   S04
.....................................................................................guuaaaaguuaagagucgggu........   1   0   S04
.....................................................................................guuaaaaguuaagagucggguuc......   1   0   S04
.....................................................................................guuaaaaguuaagagucggguucA.....   1   1   S04
.....................................................................................guuaaaaguuaagagucggguucu.....   3   0   S04
......................................................................................uuaaaaguuaagagucggguucA.....   2   1   S04
.......................................................................................uaaaaguuaagagucggguucuua...   1   0   S04
.......................................................................................Gaaaaguuaagagucggguucuua...   1   1   S04
........................................................................................aaaaguuaagagucggguuc......   1   0   S04
........................................................................................aaaaguuaagagucggguucuuG...   1   1   S04
```

Provisional ID	:	chrC01_1000651
Score total	:	0.8
Score for star read(s)	:	-1.3
Score for read counts	:	0
Score for mfe	:	2.1
Score for randfold	:	0
Score for cons. seed	:	
Total read count	:	9475
Mature read count	:	9475
Loop read count	:	0
Star read count	:	0

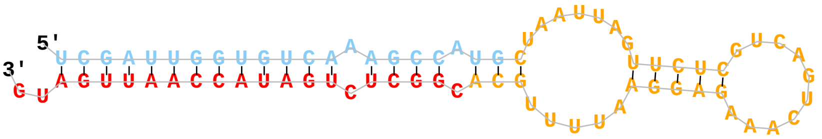

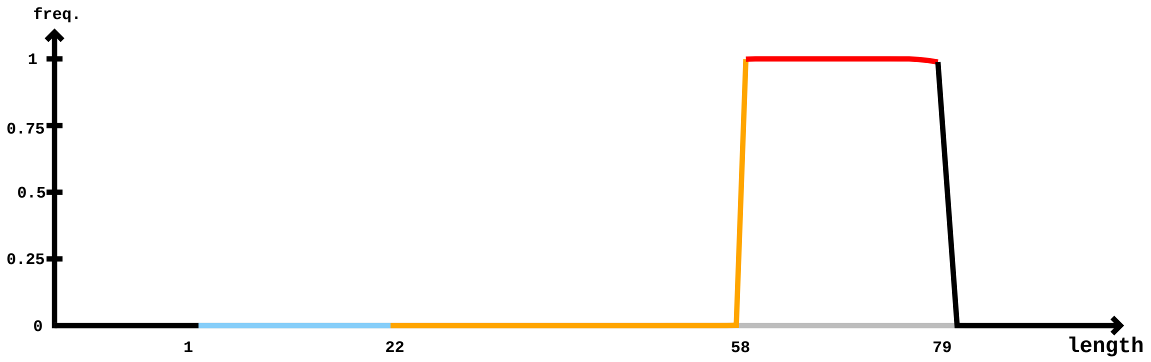

Star Mature

```
5'- cguuuucaucacgucgauuggugucaaagccaugcuaauuaguucucgucagucaaagaggaauuuugcacggcucugauaccaauugaugcaauggaagcauccauuu -3'   exp
    .((((((((..((((((((((((((.((((.(((.......(((((..........))))).....))).)))).))))))))))))))..))))))))..........   reads   mm   sample
    .....................................................................Ccggcucugauaccaauugaug..................   1   1   S03
    .....................................................................acggcucugauaccaauugaug..................   1   0   S03
    .....................................................................acggcucugauaccaauugauA..................   1   1   S03
    ......................................................................cggcucugauaccaauuA.....................   1   1   S03
    ......................................................................cggcucugauaccaauug.....................   8   0   S03
    ......................................................................cggcucugauaccaauuga....................   16   0   S03
    ......................................................................cggcucugauaccaauugau...................   18   0   S03
    ......................................................................cggcucugauaccaauugaC...................   3   1   S03
    ......................................................................cggcucugauaccaauugaA...................   1   1   S03
    ......................................................................cggcucugauaccaauugGug..................   11   1   S03
    ......................................................................cggcucuAauaccaauugaug..................   6   1   S03
    ......................................................................cCgcucugauaccaauugaug..................   6   1   S03
    ......................................................................cggcucugauaccaaGugaug..................   1   1   S03
    ......................................................................cggcucugauaccaauuUaug..................   3   1   S03
    ......................................................................cggcucugauaccaUuugaug..................   3   1   S03
    ......................................................................cggcucugauaccaauCgaug..................   7   1   S03
    ......................................................................cggcucugaGaccaauugaug..................   3   1   S03
    ......................................................................Gggcucugauaccaauugaug..................   5   1   S03
    ......................................................................cggcucugauaccGauugaug..................   5   1   S03
    ......................................................................cggcucugauaccaauugCug..................   2   1   S03
    ......................................................................cggcucuUauaccaauugaug..................   1   1   S03
    ......................................................................cggcucCgauaccaauugaug..................   3   1   S03
    ......................................................................cggcucugauaccUauugaug..................   2   1   S03
    ......................................................................cgAcucugauaccaauugaug..................   4   1   S03
    ......................................................................cggcucugauaccaauugaCg..................   19   1   S03
    ......................................................................Uggcucugauaccaauugaug..................   5   1   S03
    ......................................................................cgUcucugauaccaauugaug..................   7   1   S03
    ......................................................................cggcucugauaccaauugaAg..................   2   1   S03
    ......................................................................Nggcucugauaccaauugaug..................   8   1   S03
    ......................................................................cggcucugauaccaauGgaug..................   4   1   S03
    ......................................................................cggcucugauCccaauugaug..................   1   1   S03
    ......................................................................cgCcucugauaccaauugaug..................   2   1   S03
    ......................................................................cggcucugauGccaauugaug..................   8   1   S03
    ......................................................................cUgcucugauaccaauugaug..................   12   1   S03
```

```
              Star                                                       Mature
cguuuucaucacgucgauuggugucaaagccaugcuaauuaguucucgucagucaaagaggaauuuugcacggcucugauaccaauugaugcaauggaagcauccauuu
......................................................................cggcCcugauaccaauugaug..................   12     1   S03
......................................................................cggcucugauaccaauuCaug..................   1      1   S03
......................................................................cggcucugauaccaauugaug..................   2808   0   S03
......................................................................cggGucugauaccaauugaug..................   2      1   S03
......................................................................cggcucugauaUcaauugaug..................   2      1   S03
......................................................................cggcucugaCaccaauugaug..................   7      1   S03
......................................................................cggcucugUuaccaauugaug..................   5      1   S03
......................................................................cggcucGgauaccaauugaug..................   1      1   S03
......................................................................cggcucugauaccaauuAaug..................   2      1   S03
......................................................................cggcucugGuaccaauugaug..................   7      1   S03
......................................................................cggcucugauaccaauAgaug..................   2      1   S03
......................................................................cggcucugauaccaauugauU..................   17     1   S03
......................................................................cggcucugauacUaauugaug..................   5      1   S03
......................................................................cggcucugauaccaGuugaug..................   5      1   S03
......................................................................cggcucugauaccCauugaug..................   2      1   S03
......................................................................cggcGcugauaccaauugaug..................   1      1   S03
......................................................................cggcucAgauaccaauugaug..................   4      1   S03
......................................................................cggcucugauUccaauugaug..................   4      1   S03
......................................................................cggUucugauaccaauugaug..................   1      1   S03
......................................................................cggcucugauaccaauugaGg..................   4      1   S03
......................................................................cggcucugauaccaauugauC..................   2      1   S03
......................................................................cggcucugauaccaauugauA..................   270    1   S03
......................................................................cggcuGugauaccaauugaug..................   1      1   S03
......................................................................Aggcucugauaccaauugaug..................   1      1   S03
......................................................................cggcAcugauaccaauugaug..................   4      1   S03
......................................................................cggcucugauaccaauugaugU.................   4      1   S03
......................................................................cggcucugauaccaauugaugA.................   1      1   S03
.......................................................................ggcucugauaccaauugaug..................   4      0   S03
.......................................................................ggcucugauaccaauuUaug..................   1      1   S03

..............................................................auuuugcacggcucugauaccaau.......................   1      0   S04
....................................................................cacggcucugauaccaauugaugc.................   1      0   S04
.....................................................................acggcucugauGccaauugaug..................   1      1   S04
.....................................................................Ccggcucugauaccaauugaug..................   1      1   S04
.....................................................................acggcucugauaccaauugaug..................   2      0   S04
......................................................................cggcucCgauaccaauug.....................   1      1   S04
......................................................................cggcAcugauaccaauug.....................   1      1   S04
......................................................................cggcucugauaccaauuA.....................   1      1   S04
......................................................................cggcucugauaccaauug.....................   10     0   S04
......................................................................cggcucugauaccaauuga....................   17     0   S04
......................................................................cggcucugaCaccaauuga....................   1      1   S04
......................................................................cggcucugauaccaauugG....................   1      1   S04
......................................................................cggcucugauaccaauugaA...................   1      1   S04
......................................................................cggcucugauaccaauugaC...................   3      1   S04
......................................................................cggcucugauaccaauugau...................   26     0   S04
......................................................................cgCcucugauaccaauugau...................   1      1   S04
......................................................................cggcucugauaccaauugaG...................   1      1   S04
......................................................................cggcucugauGccaauugaug..................   18     1   S04
......................................................................cggcGcugauaccaauugaug..................   4      1   S04
......................................................................cggcucugauaccaauuAaug..................   6      1   S04
......................................................................cggcuUugauaccaauugaug..................   1      1   S04
......................................................................cgCcucugauaccaauugaug..................   1      1   S04
......................................................................cggcucugauCccaauugaug..................   2      1   S04
......................................................................cggcucAgauaccaauugaug..................   4      1   S04
......................................................................Nggcucugauaccaauugaug..................   18     1   S04
......................................................................cggcucugauaccaauugauA..................   567    1   S04
......................................................................cggcucugauaccUauugaug..................   4      1   S04
......................................................................cggcucugauaccaauugCug..................   3      1   S04
......................................................................cggcucuCauaccaauugaug..................   1      1   S04
......................................................................cggcucugauaccaauugauC..................   4      1   S04
......................................................................cggcCcugauaccaauugaug..................   23     1   S04
......................................................................cggcucugauaccaCuugaug..................   3      1   S04
......................................................................cggcucugUuaccaauugaug..................   6      1   S04
......................................................................cggcucugCuaccaauugaug..................   3      1   S04
......................................................................cggcucugauUccaauugaug..................   3      1   S04
......................................................................cggcucugauaccCauugaug..................   4      1   S04
......................................................................cggcucuAauaccaauugaug..................   2      1   S04
......................................................................cggcucCgauaccaauugaug..................   18     1   S04
......................................................................cggcucugauaccaauugGug..................   30     1   S04
......................................................................cggcAcugauaccaauugaug..................   8      1   S04
```

```
             Star                                                 Mature
cguuuucaucacgucgauuggugucaaagccaugcuaauuaguucucgucagucaaagaggaauuuugcacggcucugauaccaauugaugcaauggaagcauccauuu
......................................................................cggcucugauaccaauugaCg..................   13     1     S04
......................................................................cggcuAugauaccaauugaug..................   2      1     S04
......................................................................cggcucugGuaccaauugaug..................   20     1     S04
......................................................................cgUcucugauaccaauugaug..................   7      1     S04
......................................................................cggcucugauaccaauuCaug..................   1      1     S04
......................................................................cUgcucugauaccaauugaug..................   12     1     S04
......................................................................cggGucugauaccaauugaug..................   4      1     S04
......................................................................cggcucugauaccaGuugaug..................   15     1     S04
......................................................................cggcucugauaccaauugaug..................   5050   0     S04
......................................................................cggcucuUauaccaauugaug..................   6      1     S04
......................................................................cggcucugaAaccaauugaug..................   6      1     S04
......................................................................cggcucugauaccaauugauU..................   34     1     S04
......................................................................cggcucugauaGcaauugaug..................   1      1     S04
......................................................................cggAucugauaccaauugaug..................   2      1     S04
......................................................................cggcucugauacAaauugaug..................   2      1     S04
......................................................................cggcucugauaccaaGugaug..................   1      1     S04
......................................................................cgAcucugauaccaauugaug..................   10     1     S04
......................................................................cggcucugauaccaauugaGg..................   9      1     S04
......................................................................cggcucugaCaccaauugaug..................   10     1     S04
......................................................................cggUucugauaccaauugaug..................   6      1     S04
......................................................................cggcucugauaUcaauugaug..................   6      1     S04
......................................................................cggcucugauaccaauCgaug..................   12     1     S04
......................................................................cggcucugauaccaauGgaug..................   4      1     S04
......................................................................cggcucugauacUaauugaug..................   9      1     S04
......................................................................Uggcucugauaccaauugaug..................   11     1     S04
......................................................................cggcucugauaccGauugaug..................   15     1     S04
......................................................................Aggcucugauaccaauugaug..................   2      1     S04
......................................................................cggcucGgauaccaauugaug..................   2      1     S04
......................................................................cCgcucugauaccaauugaug..................   5      1     S04
......................................................................Gggcucugauaccaauugaug..................   5      1     S04
......................................................................cggcucugauaccaauuUaug..................   4      1     S04
......................................................................cggcucugauaccaauAgaug..................   1      1     S04
......................................................................cggcucugauaccaUuugaug..................   3      1     S04
......................................................................cggcucugauaccaauugaAg..................   4      1     S04
......................................................................cggcucugauaccaauugaugc.................   6      0     S04
......................................................................cggcucugauaccaauugaugU.................   2      1     S04
......................................................................cggcucugauaccaauugaugA.................   3      1     S04
......................................................................cggcucugauaccaauugaugG.................   2      1     S04
......................................................................cggcucugauaccaauugaugUa................   1      1     S04
.......................................................................ggcucugauaccaauugaug..................   5      0     S04
```

Provisional ID	:	chrC01_1001948
Score total	:	1.1
Score for star read(s)	:	3.9
Score for read counts	:	-3.9
Score for mfe	:	1.2
Score for randfold	:	0
Score for cons. seed	:	
Total read count	:	4
Mature read count	:	3
Loop read count	:	0
Star read count	:	1

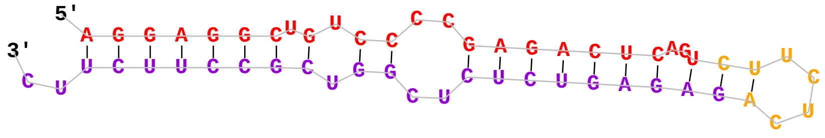

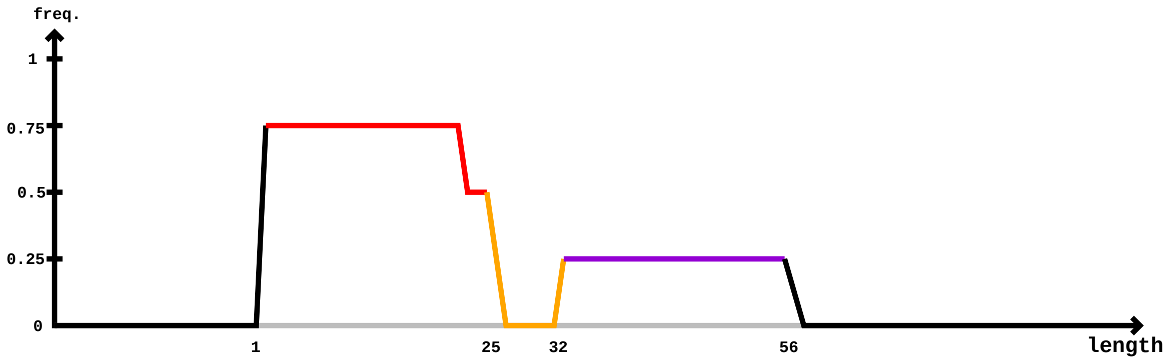

Mature Star

```
5'- agcucccgguucuuuggguc aggaggcuguccccgagacucagu cuucuca gagagucucucggucgccuucuuc uagugcacuaucgguucgucaugugccuccuccgau  -3'   obs
    agcucccgguucuuugggucaggaggcuguccccgagacucagucuucucagagagucucucggucgccuucuucuagugcacuaucgguucgucaugugccuccuccgau         exp
    .((.(((((....)))))..((((((((.(.((..(((((((..(((....))))))))))..)).))))))))......))...(((((................)))))   reads   mm   sample
    ....................aggaggcuguccccgagacuc......................................................................   1       0    S03
    ....................aggaggcuguccccgagacucagu...................................................................   2       0    S03

    ...................................................gagagucucucggucgccuucuuc....................................   1       0    S04
```

Provisional ID	:	chrC01_1005977
Score total	:	0.3
Score for star read(s)	:	-1.3
Score for read counts	:	0
Score for mfe	:	1.6
Score for randfold	:	0
Score for cons. seed	:	
Total read count	:	36
Mature read count	:	36
Loop read count	:	0
Star read count	:	0

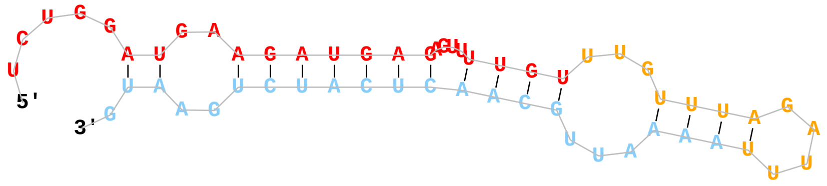

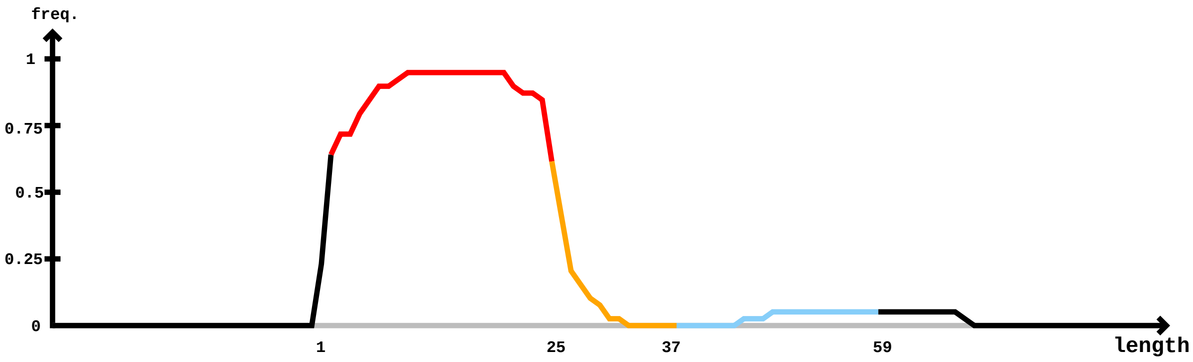

Mature Star

```
5'- uguuuguuucacuauccagaugaguuaucuggaugaagaugagaguuuuguuuguuuagauuuaaaauugcaacucaucugaaugacucaucuggaugaguuuuagaaauuuau   -3'    exp
    .((..((((((((((((((((((((((..((((((.(.((..(((((((..(......)..))))))).)).)))))))..))))))))))))))).)))....)))))..))   reads    mm    sample
    ..........................aucuggaugaagaugagaguC...................................................................   1        1     S03
    ..........................aucuggaugaagaugagaguuuug................................................................   4        0     S03
    ...........................ucuggaugaagaugagagu....................................................................   1        0     S03
    ...........................ucuggaugaagaugagaguuuug................................................................   1        0     S03
    ...........................ucuggaugaagaugagaguuuugu...............................................................   4        0     S03
    ............................cuggaugaagaugagaguuuugC...............................................................   1        1     S03
    ............................cuggaugaagaugagaguuuuguu..............................................................   1        0     S03
    ..............................ggaugaagaugagaguuuuguu..............................................................   1        0     S03
    ..............................Ugaugaagaugagaguuuuguu..............................................................   1        1     S03
    ...............................gaugaagaugagaguuuuguuugu...........................................................   1        0     S03
    ................................augaagaugagaguuuuguuuguu..........................................................   1        0     S03
    ...................................aagUugagaguuuuguuuguuua........................................................   1        1     S03
    ......................................................................caacucaucugaaugacucaucug....................   1        0     S03

    ..........................aucuggaugaagaugagaguuuu.................................................................   1        0     S04
    ..........................aucuggaugaagaugagaguuuug................................................................   3        0     S04
    ...........................ucuggaugaagaugagagu....................................................................   1        0     S04
    ...........................ucuggaugaagaugagaguuuug................................................................   1        0     S04
    ...........................ucuggaugaagaugagaguuuugC...............................................................   2        1     S04
    ...........................ucuggaugaagaugaAaguuuugu...............................................................   1        1     S04
    ...........................ucuggaugaagaugagaguuuugu...............................................................   4        0     S04
    ...........................ucuggaugaagaugagaguuuuguuu.............................................................   1        0     S04
    ............................cuggaugaagaugagaguuuugu...............................................................   1        0     S04
    ..............................ggaugaagaugagaguuuuguuu.............................................................   1        0     S04
    ...............................gaugaagaugagaguuuuguuug............................................................   1        0     S04
    ................................augaagaugagaguuuuguuuguu..........................................................   1        0     S04
    ..................................gaagaugagaguuuuguuuU............................................................   1        1     S04
    .........................................................................cucaucugaaugacucaucu.....................   1        0     S04
```

Provisional ID	:	chrC01_1006921
Score total	:	10.1
Score for star read(s)	:	3.9
Score for read counts	:	6.2
Score for mfe	:	0
Score for randfold	:	0
Score for cons. seed	:	
Total read count	:	24
Mature read count	:	22
Loop read count	:	0
Star read count	:	2

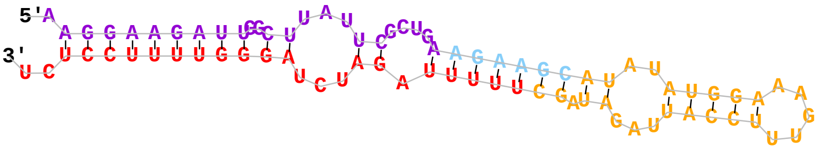

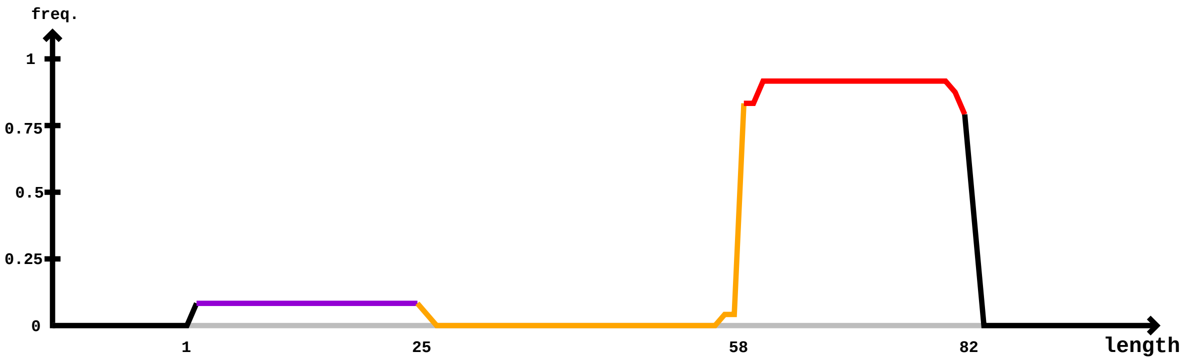

Star **Mature**

```
5'- ccaguuuauagauaaggaagauuggcuuauucgcugaagaagcauauauggaaaguuuccauuagauagcuuuuuagaucuaggguuuuccucuaaaaauauaaaguggaguc   -3'   obs
    ccaguuuauagauaaggaagauuggcuuauucgcugaagaagcauauauggaaaguuuccauuagauagcuuuuuagaucuaggguuuuccucuaaaaauauaaaguggaguc          exp
    (((.((((((....(((((((((..((...((....(((((((((..(((((.....)))))...)).))))))).))...))))))))))).......)))))).)))....   reads   mm   sample
    .............aaggaagauuggcuuauucgcuga............................................................................   1       0    S03
    ....................................................................gcuuuuuagaucuaggguuuuccC.....................   1       1    S03
    ......................................................................uuuuuagaucuaggguuuuccuc....................   1       0    S03
    ......................................................................Cuuuuagaucuaggguuuuccucu...................   1       1    S03
    ......................................................................uuuuuagaucuaggguuuuccucC...................   1       1    S03
    ......................................................................uuuuuagaucuaggguuuuccucu...................   3       0    S03
    ......................................................................uuuuuagaucuaggguuCuccucu...................   1       1    S03
    ........................................................................uuuagaucuaggguuuuccucu...................   1       0    S03

    .............aaggaagauuggcuuauucgcuga............................................................................   1       0    S04
    ......................................................................uuuuuagaucuaggguuuuccuc....................   1       0    S04
    ......................................................................uuuuuagaucuaggguuuuccucC...................   1       1    S04
    ......................................................................uuuuuagaucuaggguuuuccucu...................   9       0    S04
    ......................................................................uuuuuagaucuagggCuuuccucu...................   1       1    S04
    ........................................................................uuuagaucuaggguuuuccucua..................   1       0    S04
```

Provisional ID : chrC01_1010244
Score total : 357.2
Score for star read(s) : 3.9
Score for read counts : 353.4
Score for mfe : -0.1
Score for randfold : 0
Score for cons. seed :
Total read count : 705
Mature read count : 499
Loop read count : 0
Star read count : 206

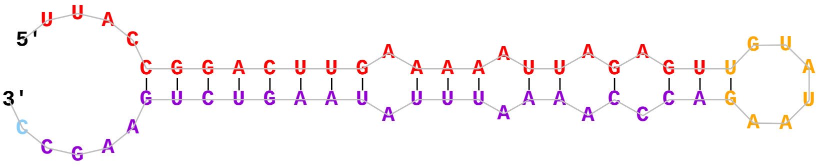

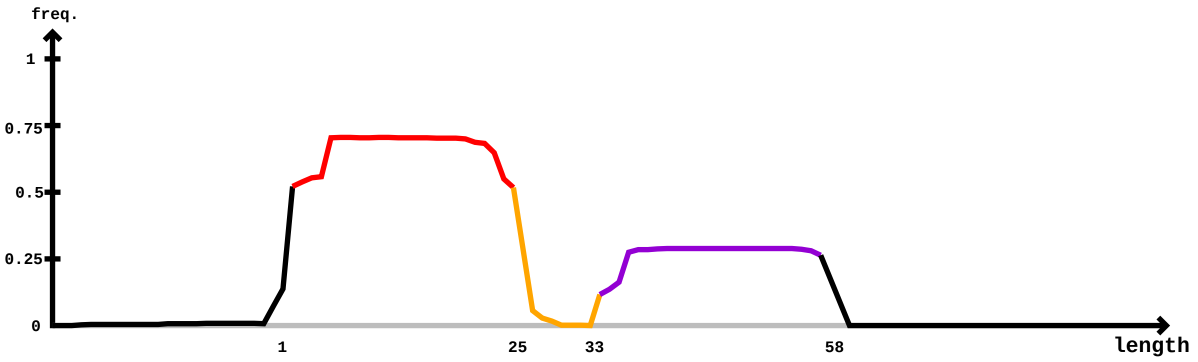

Mature Star

```
5'- gguucagagguggacuuuguagguuaccggacuugaaaaauuagaguuguauaagacccaaaauuuauaagucugaagccaaaauaaaguuguaacggacacgugucgacucug -3'  obs
    gguucagagguggacuuuguagguuaccggacuugaaaaauuagaguuguauaagacccaaaauuuauaagucugaagccaaaauaaaguuguaacggacacgugucgacucug       exp
    ....(((((...((((((((.((((..((((((((.(((.((.(.(((......))).).)).))).)))))))).))))...))))))))......(((....)))..)))))   reads  mm  sample
    .guucagGgguggacuuugu..............................................................................................   1      1   S03
    .guucagaggAggacuuuguagguu.........................................................................................   1      1   S03
    ..uucagGgguggacuuugu..............................................................................................   1      1   S03
    .....................gguuaUcggacuugaaaaauuag......................................................................   3      1   S03
    .....................gguuaUcggacuugaaaaauuaga.....................................................................   16     1   S03
    .....................gguuaUcggacuugaaaaauuagagu...................................................................   1      1   S03
    ......................guuaUcggacuugaaaaau.........................................................................   1      1   S03
    ......................guuaUcggacuugaaaaauu........................................................................   1      1   S03
    ......................guuaUcggacuugaaaaauua.......................................................................   1      1   S03
    ......................guuaUcggacuugaaaaauuag......................................................................   3      1   S03
    ......................guuaUcggacuugaaaaauuaga.....................................................................   5      1   S03
    ......................guuaUcggacuugaaaaauuagag....................................................................   5      1   S03
    ......................guuaUcggacuugaaaaauuagagu...................................................................   3      1   S03
    ......................guuaUcggacuugaaaaauuagaguu..................................................................   1      1   S03
    .......................uuaUcggacuugaaaaau.........................................................................   1      1   S03
    .......................uuaUcggacuugaaaaauu........................................................................   1      1   S03
    .......................uuaUcggacuugaaaaauuag......................................................................   7      1   S03
    .......................uuaUcggacuugaaaaauuaga.....................................................................   8      1   S03
    .......................uuaUcggacuugaaaaauuagag....................................................................   3      1   S03
    .......................uuaUcggacuugaaaaauuagagu...................................................................   120    1   S03
    .......................uuaccggacuugaaaaauuagagu...................................................................   3      0   S03
    .......................uuaAcggacuugaaaaauuagagu...................................................................   1      1   S03
    .......................uuaUcggacuugaaaaauuagaguu..................................................................   2      1   S03
    ........................uaccggacuugaaaaauuagaguu..................................................................   3      0   S03
    .........................aGcggacuugaaaaauuagagu...................................................................   1      1   S03
    .........................accggacuugaaaaauuagaguu..................................................................   2      0   S03
    .........................accggacuugaaaaauuagaguug.................................................................   2      0   S03
    ..........................ccggacuugaaaaauuagaguu..................................................................   1      0   S03
    ...........................cggacuugaaaaauuagC.....................................................................   1      1   S03
    ...........................cggacuugaaaaauuaga.....................................................................   1      0   S03
    ...........................cggacuugaaaaauuagagu...................................................................   31     0   S03
    ...........................cggacuugaaaaauuagagA...................................................................   2      1   S03
    ...........................cggaUuugaaaaauuagagu...................................................................   1      1   S03
```

Mature **Star**

gguucagagguggacuuuguagguuaccggacuugaaaaauuagaguuguauaagacccaaaauuuauaagucugaagccaaaauaaaguuguaacggacacgugucgacucug			
...........................cggacuugaaGaauuagagu...	1	1	S03
...........................cggacuugaaaaauuagagG...	1	1	S03
...........................cggacuugaaaaauuagaguC..	2	1	S03
...........................cggacuugaaaaauuagaguu..	10	0	S03
...........................cggacuugaaaaauuagaguuA...	1	1	S03
...........................cggacuugaaaaauuagaguug...	7	0	S03
...........................cggacuugaaaaauuagaguugu..	2	0	S03
...........................cggacuugaaaaauuagaguugG..	1	1	S03
...........................cggacuugaaaaauuagaguuguU...	2	1	S03
...........................cggacuugaaGaauuagaguugua...	1	1	S03
...........................cggacuugaaaaauuagaguugua...	2	0	S03
....acccaaaauuuauaagucugaC.....................................	1	1	S03
....acccaaaauuuauaagucugaag....................................	1	0	S03
....acccaaaauuuauaagucugaagc...................................	28	0	S03
....acccaaaauuuauaagucugaagA...................................	4	1	S03
....acccaaaauuuauaagAcugaagc...................................	1	1	S03
....acccaaaauuuauaagucugaagU...................................	6	1	S03
....cccaaaauuuauaagucugaa.....................................	1	0	S03
....cccaaaauuuauaagucugaag....................................	4	0	S03
....cccaaaauuuauaagucugaagc...................................	1	0	S03
....cccaaaauuuauaagucugaagcc..................................	1	0	S03
....cccaaaauuuauaagucugaGgcc..................................	1	1	S03
....ccaaaauuuauaagucugaag....................................	1	0	S03
....ccaaaauuuauaagucugaagc...................................	7	0	S03
....ccaaaauuuauaagucugaGgc...................................	1	1	S03
....Aaaaauuuauaagucugaagc...................................	2	1	S03
....caaaauuuauaagucugaagc...................................	20	0	S03
....caaaauuuauaagucugaagcc..................................	2	0	S03
....aaaauuuauaagucugaa.....................................	1	0	S03
....aaaauuuauaagucugaagc...................................	1	0	S03
....aaaauuuauaagucugaagcca.................................	1	0	S03
....aauuuauaagucugaagcc..................................	1	0	S03
....auuuGuaagucugaagcc..................................	1	1	S03
..........uggacuuuguagguuaUcgg..	1	1	S04
..........uggacuuuguagguuaUcggacuu..	1	1	S04
..............cuuuguagguuaUcggacuugaaa..	1	1	S04
....................agguuaccggacuugaaaaauuag..	1	0	S04
.....................gguuaUcggacuugaaaaauu..	3	1	S04
.....................gguuaUcggacuugaaaaauua...	2	1	S04
.....................gguuaUcggacuugaaaaauuaga...	22	1	S04
......................guuaUcggacuugaaaaauu..	1	1	S04
......................guuaUcggacuugaaaaauuag..	4	1	S04
......................guuaccggacuugaaaaauuaga...	1	0	S04
......................guuaUcggacuugaaaaauuaga...	5	1	S04
......................guuaUcggacuugaaaaauuagag..	12	1	S04
......................guuaUcggacuugaaaaauuagagu...	2	1	S04
......................guuaUcggacuugaaaaauuagaguu..	1	1	S04
.......................uuaUcggacuugaaaaauu..	3	1	S04
.......................uuaUcggacuugaaaaauuag..	7	1	S04
.......................uuaUcggacuugaaaaauuaga...	11	1	S04
.......................uuaUcggacuugaaaaauuagag..	3	1	S04
.......................uuaUcggacuugaaaaauuagagu...	95	1	S04
.......................uuaccggacuugaaaaauuagagC...	1	1	S04
.......................uuaccggacuugaaaaauuagagu...	6	0	S04
.......................uuaUcggacuugaaaaauuagaguu..	2	1	S04
........................uaccggacuugaaaaauuagagC...	1	1	S04
........................uaccggacuugaaaaauuagagu...	1	0	S04
........................uaccggGcuugaaaaauuagaguu..	1	1	S04
........................uGccggacuugaaaaauuagaguu..	1	1	S04
........................uaccggacuugaaaaauuagaguu..	4	0	S04
........................uaccggaUuugaaaaauuagaguu..	1	1	S04
.........................accggacuugaaaaauuagagu...	1	0	S04
.........................accggaUuugaaaaauuagaguu..	1	1	S04
.........................accggacuugaaaaauuagaguu..	2	0	S04
.........................accggaGuugaaaaauuagaguu..	1	1	S04
.........................aAcggacuugaaaaauuagaguug...	1	1	S04
.........................accggacuugaaaaauuagaguug...	1	0	S04
..........................ccggacuugaaaaauuagaguuA...	1	1	S04
..........................ccggacuugaaaaauuagaguAgu..	1	1	S04

Mature **Star**

```
gguucagagguggacuuuguagguuaccggacuugaaaaauuagaguuguauaagacccaaaauuuauaagucugaagccaaaauaaaguuguaacggacacgugucgacucug
...........................cggacCugaaaaauuagagu...................................................................	1	1	S04
...........................cggacuugaaaaauuagagG...................................................................	1	1	S04
...........................cggacuugaaaaauuagagu...................................................................	12	0	S04
...........................cggacuugaaaaauuagagC...................................................................	3	1	S04
...........................cggacuugaaaaauuagaguG..................................................................	1	1	S04
...........................cggacuugaaaaauuagaguu..................................................................	4	0	S04
...........................cggacuugaaaaauuagaguug.................................................................	4	0	S04
...........................cggacuugaaaaauuagaguuA.................................................................	1	1	S04
...........................cggacuugaaaaaAuagaguug.................................................................	1	1	S04
...........................cggacuugaaaaauuagaguugA................................................................	1	1	S04
...........................cggacuugGaaaauuagaguugu................................................................	1	1	S04
...........................cggacuugaaaaauuagaguugu................................................................	2	0	S04
...........................cggacuugaaaaauuagaguuguU...............................................................	1	1	S04
...........................cggacuugaaaaauuagaguugCa...............................................................	1	1	S04
...........................cggacuugaaaaauuagagAugua...............................................................	1	1	S04
...........................cggaUuugaaaaauuagaguugua...............................................................	1	1	S04
...........................cggacuugaaaaauuagaguugua...............................................................	2	0	S04
............................ggacuugaaaaauuagaguug.................................................................	1	0	S04
................................uugaaaaauuagaguuguauaa............................................................	1	0	S04
.......................................................acccaaaauuuauaagucuga......................................	2	0	S04
.......................................................acccaaaauuuauaagCcugaag....................................	1	1	S04
.......................................................acccaaaauuuauaagucugaagG...................................	1	1	S04
.......................................................acccaaaauuuauaagucugaagc...................................	29	0	S04
.......................................................acccaaaauuuauaagucCgaagc...................................	1	1	S04
.......................................................acccaaaauuuauaagucugaCgc...................................	1	1	S04
.......................................................acccaaaauuuauaagucugaagA...................................	4	1	S04
.......................................................acccaaaauuuauaagucugaagU...................................	3	1	S04
........................................................cccaaaauuuauaagucugaa.....................................	1	0	S04
........................................................cccaaaauuuauaagucugaag....................................	2	0	S04
........................................................cccaaaauuuauaagucugaagc...................................	1	0	S04
........................................................cccaaaauuuauaagucugaagcc..................................	2	0	S04
.........................................................ccaaaauuuauaagucugaag....................................	1	0	S04
.........................................................ccaaaauuuauaagucugaagc...................................	6	0	S04
.........................................................ccaaaauuuauaagucugaagA...................................	1	1	S04
.........................................................ccaaaauuuauaagucugaGgc...................................	1	1	S04
.........................................................ccaaaauuuauaagucugaagG...................................	1	1	S04
..........................................................caaaauuuauaagucugaag....................................	2	0	S04
..........................................................Aaaaauuuauaagucugaagc...................................	2	1	S04
..........................................................caaaauuuauaagucugaagc...................................	47	0	S04
..........................................................caaaauCuauaagucugaagc...................................	1	1	S04
..........................................................caaaauuuauaagucugaagcc..................................	3	0	S04
..........................................................caaaauuuauaagucugaagcA..................................	1	1	S04
...........................................................aaaauuuauaagucugaagc...................................	1	0	S04
...........................................................aaaauuuauaagucugaagA...................................	2	1	S04
...........................................................aaaauuuauaagucugaagcc..................................	1	0	S04
.............................................................aauuuauaagucugaagcc..................................	1	0	S04
```

Provisional ID	:	chrC01_1014173
Score total	:	0.4
Score for star read(s)	:	-1.3
Score for read counts	:	0
Score for mfe	:	1.7
Score for randfold	:	0
Score for cons. seed	:	
Total read count	:	12
Mature read count	:	11
Loop read count	:	0
Star read count	:	1

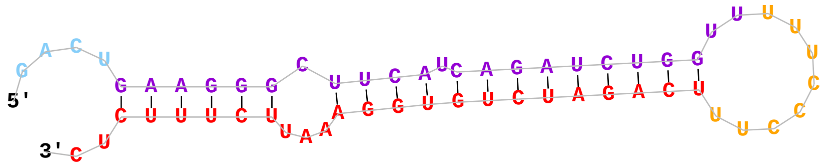

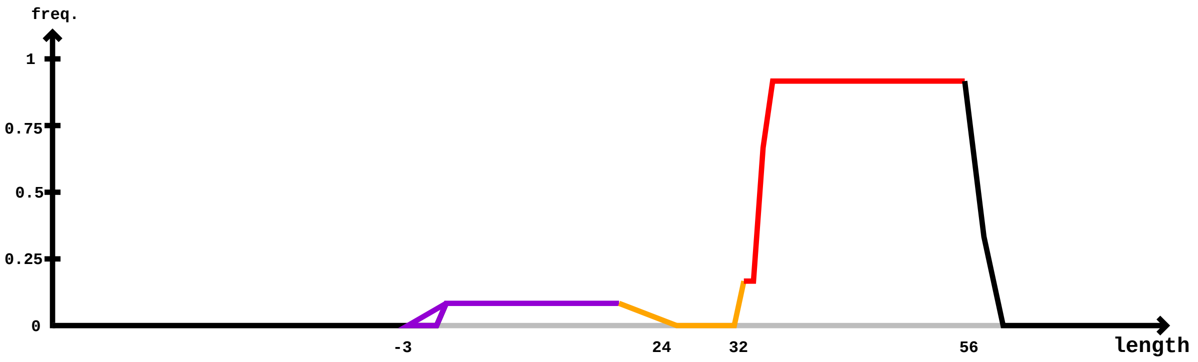


```
                              Star                          Mature
5'- acggcgugacgacggugaagagggucggcguuuaggacugaagggcuucaucagaucugguuuuucccuuucagaucuguggaaauucuuucucggcgaccgcucgaaucacgu  -3'  obs
    acggcgugacgacggugaagagggucggcguuuaggacugaagggcuucaucagaucugguuuuucccuuucagaucuguggaaauucuuucucggcgaccgcucgaaucacgu        exp
    .((......))...((((.((((((((.((....(((..(((....((((.(((((((((..........)))))))))))))..)))..))))).))))).)))...))))..   reads    mm    sample
    ........................................................................agaucuguggaaauucuuucuU....................   1        1     S03
    ........................................................................agaucuguggaaauuUuuucuc....................   1        1     S03
    ........................................................................agaucuguggaaauucuuucucgg..................   1        0     S03

    .......................................gaagggcuucaucagaucugguC....................................................   1        1     S04
    ......................................................................ucagaucuguggaaauucuuucuc....................   2        0     S04
    ........................................................................agaucuguggaaauucuuucuc....................   2        0     S04
    ........................................................................agGucuguggaaauucuuucucgg..................   1        1     S04
    .........................................................................gaucuguggaaauucuuucuc....................   1        0     S04
    .........................................................................gaucuguggaaauucuuucucggc.................   2        0     S04
```

Provisional ID	:	chrC01_1020227
Score total	:	33.5
Score for star read(s)	:	3.9
Score for read counts	:	28.2
Score for mfe	:	1.5
Score for randfold	:	0
Score for cons. seed	:	
Total read count	:	67
Mature read count	:	59
Loop read count	:	0
Star read count	:	8

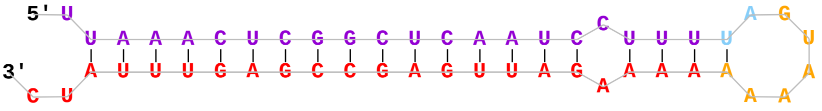

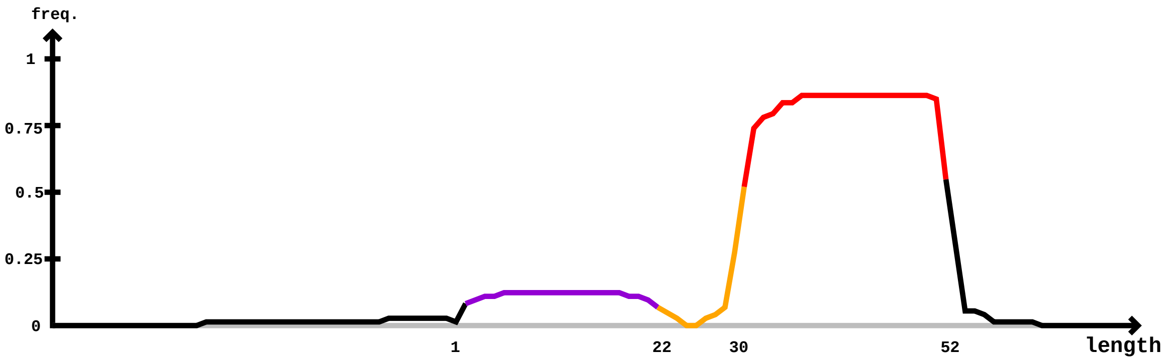

Star Mature

```
5'- uagauuaguaauucacguccguuuuauuaguuuaauugcaauuaaacucggcucaauccuuuuaguaaaaaaaagauugagccgaguuuaucuaguguauauacuguuuuu  -3'   obs
    uagauuaguaauucacguccguuuuauuaguuuaauugcaauuaaacucggcucaauccuuuuaguaaaaaaaagauugagccgaguuuaucuaguguauauacuguuuuu         exp
    ((((((((((((..(......)..)))))))))))..((((..((((((((((((((((.((((......)))).)))))))))))))))).....))))............   reads   mm   sample
    .................................aauCgcaauuaaacucggcucaauc.....................................................   1   1   S03
    .........................................uuaaacucggcucaauccuuu.................................................   3   0   S03
    .........................................uuaaacucggcucaauccuuuua...............................................   1   0   S03
    ...........................................aaacucggcucaauccuu..................................................   1   0   S03
    .............................................acucggcucaauccuuuua...............................................   1   0   S03
    ..................................................................aaaaaaaagauugagccgaguuuaC....................   1   1   S03
    ..................................................................aaaaaaaagauugagccgaguuuauc...................   1   0   S03
    ...................................................................aaaaaaagauugagccgaguuuaA....................   1   1   S03
    ....................................................................aaaaaagauugagccgaguuuauA...................   1   1   S03
    .....................................................................aaaaagauugagccgaguuuau....................   1   0   S03
    .....................................................................aaaaagauugagccgaguuuaA....................   2   1   S03
    .....................................................................aaaaagauugagccgaguuuauA...................   2   1   S03
    ......................................................................aaaagauugagccgaguuuau....................   3   0   S03
    ......................................................................aaaagauugagccgaguuuauc...................   1   0   S03
    ......................................................................aaaagauugagccgaguuuauA...................   1   1   S03
    ......................................................................aaaagauugagccgaguuuaucuagu...............   1   0   S03
    .......................................................................aaagauugagccgaguuuauA...................   4   1   S03
    .........................................................................agauugagccgaguuuauA...................   1   1   S03
    ............................................................................uugagccgaguuuaucuagu...............   1   0   S03

    ..............acguccguuuuauuaguuuaauugca.......................................................................   1   0   S04
    .........................................uuaaacucggcucaaucUu...................................................   1   1   S04
    ..........................................uaaacucggcucaauccuu..................................................   1   0   S04
    ...................................................................aaaaaaagauugagccgaguuua.....................   1   0   S04
    .....................................................................aaaaGgauugagccgaguuuau....................   6   1   S04
    .....................................................................aaaaGgauugagccgaguuuauc...................   4   1   S04
    ......................................................................aaaGgauugagccgaguuuau....................   4   1   S04
    ......................................................................aaaGgauugagccgaguuuauc...................   7   1   S04
    ......................................................................aaaGgauugagccgaguuuaucu..................   1   1   S04
    .......................................................................aaGgauugagccgaguuuau....................   2   1   S04
    .......................................................................aaGgauugagccgaguuuauc...................   6   1   S04
    .......................................................................aaGgauugagccgaguuuaucu..................   3   1   S04
    .......................................................................aaGgauugagccgaguuuaucuag................   1   1   S04
```

Star Mature

uagauuaguaauucacguccguuuuauuaguuuaauugcaauuaaacucggcucaauccuuuuaguaaaaaaaagauugagccgaguuuaucuaguguauauacuguuuuu			
....aGgauugagccgaguuuau....................	2	1	S04
....aGgauugagccgaguuuauc...................	1	1	S04
....gauugagccgaguuuauA...................	3	1	S04
....uugagccgaguuuaucuaguguaua..........	1	0	S04

Provisional ID	:	chrC01_1023865
Score total	:	6.4
Score for star read(s)	:	3.9
Score for read counts	:	0.1
Score for mfe	:	2.4
Score for randfold	:	0
Score for cons. seed	:	
Total read count	:	12
Mature read count	:	7
Loop read count	:	3
Star read count	:	2

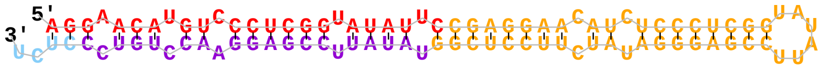

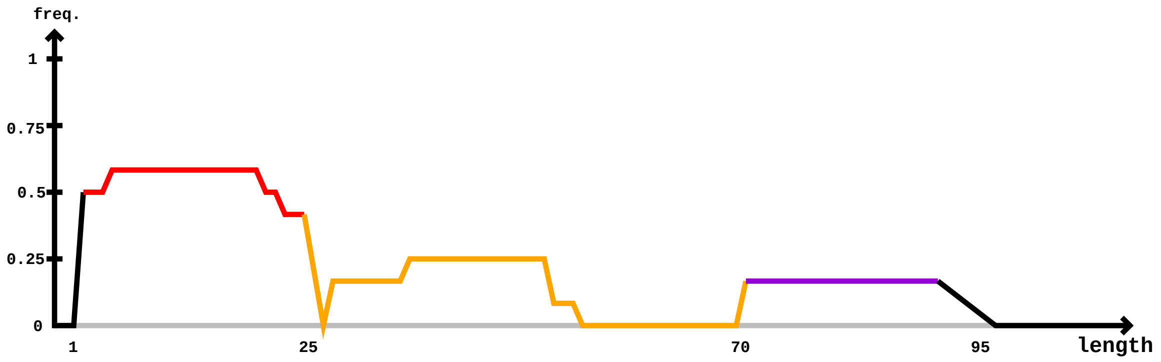

Mature **Star**

```
5'- gaggaacaugucccucgguauauuccgaggaacaucucccucgguauauuccgagggauaucuuccucgguauauuccgaggaaccugucccucuguauagcccgaacgu  -3'   obs
    gaggaacaugucccucgguauauuccgaggaacaucucccucgguauauuccgagggauaucuuccucgguauauuccgaggaaccugucccucuguauagcccgaacgu        exp
    ((((.(((.((.((((((.((((.((((((((.((.((((((((......)))))))).)).)))))))).)))).)))))).)).))).))))................   reads    mm     sample
    .aggaacaugucccucggua..........................................................................................   1        0      S03
    .aggaacaugucccucgguaua........................................................................................   1        0      S03
    ...................................cucccucgguauauuccg.........................................................   1        0      S03
    ......................................................................uauauuccgaggaaccugucc...................   1        0      S03

    .aggaacGugucccucgguauauuc.....................................................................................   1        1      S04
    .aggaacaugucccucgguauauuc.....................................................................................   3        0      S04
    ....aacaugucccucgguauauuc.....................................................................................   1        0      S04
    ...........................aggaacaucucccucgguauauu............................................................   2        0      S04
    ......................................................................uauauuccgaggaaccugucU...................   1        1      S04
```

Provisional ID	:	chrC01_1039577
Score total	:	0.3
Score for star read(s)	:	-1.3
Score for read counts	:	0
Score for mfe	:	1.6
Score for randfold	:	0
Score for cons. seed	:	
Total read count	:	237
Mature read count	:	116
Loop read count	:	28
Star read count	:	93

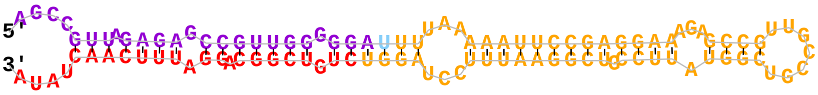

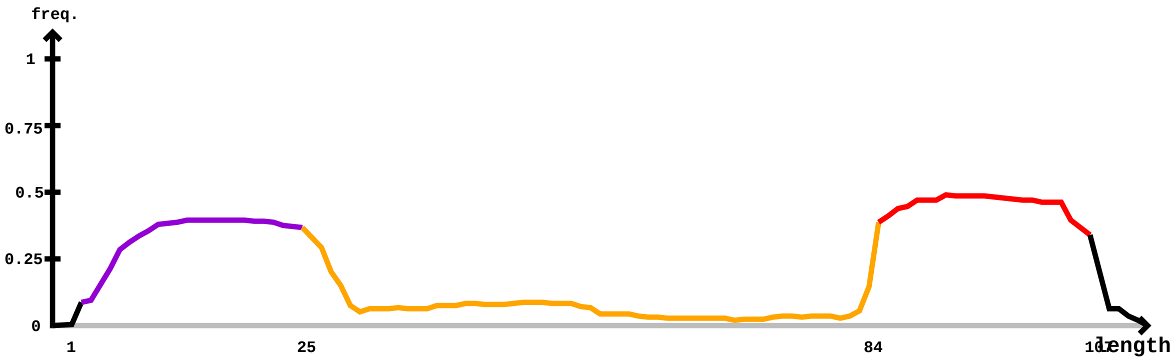

Star **Mature**

```
5'- uagccguuagagagccguugggggauuuuaaaaauuccgaggaaagagccguugccgucgguauuccgucggaauuuccuaggucugucggcaggauuucaacuauaaauac -3'   obs
    uagccguuagagagccguugggggauuuuuaaaaauuccgaggaaagagccguugccgucgguauuccgucggaauuuccuaggucugucggcaggauuucaacuauaaauac        exp
    .....(((.((((.(((((((.((((((...((((((((((((...((((.......)))).)))).)))))))))...)))))).))))).)).)))))))..........   reads   mm   sample
    .agccguuagagagccguu.............................................................................................   1       0    S03
    .agccguuagagagccguugg...........................................................................................   1       0    S03
    .agccguuagagagccguuggg..........................................................................................   1       0    S03
    .agccguuagagagccguuggggga.......................................................................................   4       0    S03
    .agccguuagagagccguCggggga.......................................................................................   5       1    S03
    ..gccguuagagagccguuggggga.......................................................................................   1       0    S03
    ...ccguuagagagccguCgggggauu.....................................................................................   1       1    S03
    ...ccguuagagagccguugggggauA.....................................................................................   1       1    S03
    ...ccguuagagagccguugggggauu.....................................................................................   7       0    S03
    ....cguuagagagccguugggggauu.....................................................................................   3       0    S03
    ....cguuagagagccguCgggggauu.....................................................................................   1       1    S03
    ....cguuagagagccguCgggggauuu....................................................................................   4       1    S03
    ....cguuagagagccguugggggauuu....................................................................................   2       0    S03
    .....guuagagagccguCgggggauu.....................................................................................   1       1    S03
    .....guuagagagccguCgggggauuu....................................................................................   3       1    S03
    .....guuagagagccguugggggauuuu...................................................................................   4       0    S03
    .....guuagagagccguCgggggauuuu...................................................................................   5       1    S03
    ......uuagagagccguCgggggauuu....................................................................................   1       1    S03
    ......uuagagagccguCgggggauuuu...................................................................................   3       1    S03
    ......uuagagagccguCgggggauuuua..................................................................................   1       1    S03
    .......uagagagccguCgggggauuuu...................................................................................   1       1    S03
    .......uagagagccguCgggggauuuua..................................................................................   1       1    S03
    ........agagagccguCgggggau......................................................................................   2       1    S03
    ........agagagccguCgggggauuuuaa.................................................................................   1       1    S03
    ........agagagccguCgggggauuuuaaa................................................................................   2       1    S03
    .........gagagccguugggggauu.....................................................................................   1       0    S03
    .........gagagccguugggggauuuu...................................................................................   2       0    S03
    .........gagagccguCgggggauuuua..................................................................................   1       1    S03
    ...........gagccguCgggggauuuuaaaaau.............................................................................   1       1    S03
    ............agccguugggggauuuuaaaUauu............................................................................   1       1    S03
    .............................aaaaauuccgaggaaagagccguu...........................................................   1       0    S03
    ...............................aaauuccgaggaaagagccguug..........................................................   1       0    S03
    ................................aauuccgaggaaagagccguu...........................................................   1       0    S03
```

```
 Star                                                                             Mature
uagccguuagagagccguugggggauuuuaaaaauuccgaggaaagagccguugccgucgguauuccgucggaauuuccuaggucugucggcaggauuucaacuauaaauac
......................................gaggaaagagccguugccgucggu..................................................   1    0    S03
.........................................gaaagagccguugccguc.....................................................   1    0    S03
.........................................gaaagagccguugccgucggua.................................................   1    0    S03
..............................................................auuccgucggaauuuccu................................   2    0    S03
...............................................................Guccgucggaauuuccua...............................   1    1    S03
.........................................................................auuuccuaggucugucggcagga................   1    0    S03
.........................................................................auuuccuaggucugucggcaggaC...............   1    1    S03
.................................................................................ggucugucggcaggauuucaacu........   2    0    S03
...................................................................................ucugucggcaggauuucaacu........   2    0    S03
...................................................................................ucugucggcaggauuucaacuauG.....   1    1    S03
...................................................................................ucugucggcaggauuucaacuaua.....   7    0    S03
....................................................................................cugucggcaggauuucaacuaua.....   22   0    S03
....................................................................................cugucggcaggauuucaacuauG.....   5    1    S03
....................................................................................cugucggcaggauuucaacuaCa.....   1    1    S03
....................................................................................cugucggcaggauuucaacuauaa....   1    0    S03
.....................................................................................ugucggcaggauuucaacuaua.....   1    0    S03
......................................................................................gucggcaggauuucaacu........   1    0    S03
......................................................................................gucggcaggauuucaacuau......   1    0    S03
......................................................................................gucggcaggauuucaacuauaaau..   2    0    S03
.......................................................................................ucggcaggauuucaacuauaaaua.   1    0    S03
........................................................................................cggcaggauuucaacuauaaau..   1    0    S03
........................................................................................cggcaggauuucaacuauaaauac   1    0    S03

uagccguuagagagccguuggggC........................................................................................   1    1    S04
.agccguuagagagccguuggg..........................................................................................   2    0    S04
.agccguuagagagccguugggg.........................................................................................   1    0    S04
.agccguuagagagccguCggggga.......................................................................................   1    1    S04
.agccguuagagagccguugggggU.......................................................................................   1    1    S04
.agccguuagagagccguuggggga.......................................................................................   4    0    S04
..gccguuagagagccguugggggau......................................................................................   1    0    S04
...ccguuagagagccguugggggau......................................................................................   2    0    S04
...ccguuagagagccguugggggauu.....................................................................................   4    0    S04
....cguuagagagccguugggggauu.....................................................................................   1    0    S04
....cguuagagagccguugggggauG.....................................................................................   1    1    S04
....cguuagagagccguugggggauuu....................................................................................   3    0    S04
.....guuagagagccguugggggaC......................................................................................   1    1    S04
.....guuagagagccguugggggauuuu...................................................................................   3    0    S04
.....guuaAagagccguugggggauuuu...................................................................................   1    1    S04
......uuagagagccguugggggauu.....................................................................................   1    0    S04
......uuagagagccguugggggauuuu...................................................................................   1    0    S04
.......uagagagccguugggggauuuua..................................................................................   3    0    S04
.......uagagagccguugggggauuuuaa.................................................................................   1    0    S04
.........gagagccguugggggauu.....................................................................................   1    0    S04
.........gagagccguugggggauuuuaaaa...............................................................................   1    0    S04
..........agagccguugggggauuuuaa.................................................................................   1    0    S04
............agccguugggggauuuuaaaaau.............................................................................   1    0    S04
.........................uuuuaaaaauuccgagga.....................................................................   1    0    S04
..........................uuuaaaaauuccgaggaaagagcA..............................................................   1    1    S04
..........................uuuaaaaauuccgaggaaagagcc..............................................................   1    0    S04
...............................aGauuccgaggaaagagccguugc.........................................................   1    1    S04
...............................aaauuccgaggaaagagccguugc.........................................................   3    0    S04
...............................aaauuccgaggaaagagccguugU.........................................................   1    1    S04
................................aauuccgaggaaagagccguu...........................................................   1    0    S04
.................................auuccgaggaaagagccguugc.........................................................   1    0    S04
..................................uuccgaggaaagagccguugccgG......................................................   1    1    S04
...................................uccgaggaaagagccguugccguc.....................................................   1    0    S04
....................................ccgaggaaagagccguugccgucg....................................................   1    0    S04
......................................gaggaaagagccguugccgucggu..................................................   2    0    S04
..............................................agccguugccgucgguauuccguc..........................................   1    0    S04
...............................................gccguugccgucgguauuccgA...........................................   1    1    S04
..................................................guugccgucgguauuccgu...........................................   1    0    S04
..........................................................cgguauuccgucggaauC....................................   1    1    S04
......................................................................ggaauuuccuaggucugucggc....................   2    0    S04
..........................................................................uuuccuaggucugucggcaggauu..............   1    0    S04
.............................................................................ccuaggucugucggcaggauuu.............   1    0    S04
.................................................................................ggucugucggcaggauuucaacA........   1    1    S04
..................................................................................gucugucggcaggauuucaacua.......   2    0    S04
..................................................................................gucugucggcaggauuucaacuaC......   1    1    S04
..................................................................................gucugucggcaggauuucaacuau......   1    0    S04
..................................................................................gucugucggcaggauuucaacuauG.....   1    1    S04
```

Star **Mature**

```
uagccguuagagagccguugggggauuuuaaaaauuccgaggaaagagccguugccgucgguauuccgucggaauuuccuaggucugucggcaggauuucaacuauaaauac
...................................................................................ucugucggcaggauuuca...........   1   0   S04
...................................................................................ucugucggcaggauuucU...........   1   1   S04
...................................................................................ucugucggcaggauuucaacA........   1   1   S04
...................................................................................ucugucggcaggauuucaacu........   1   0   S04
...................................................................................ucugucggcaggUuuucaacu........   1   1   S04
...................................................................................ucugucggcaggauuucaacua.......   2   0   S04
...................................................................................ucugucggcaggauuucaacuaua.....   6   0   S04
....................................................................................cugucggcaggauuucaacG........   1   1   S04
....................................................................................cugucggcaggauuucaacC........   1   1   S04
....................................................................................cugucggcaggauuucaacu........   5   0   S04
....................................................................................cugucggcaggauuucaacuau......   3   0   S04
....................................................................................cugucggcaggauuucaacuaG......   1   1   S04
....................................................................................cGgucggcaggauuucaacuaua.....   1   1   S04
....................................................................................cugucggcaggauuucaacuaua.....  14   0   S04
....................................................................................cugucggcaggauuucaacuauG.....   5   1   S04
....................................................................................cugucggcaggauuucaacuauaa....   1   0   S04
.....................................................................................ugucggcaggauuucaacu........   1   0   S04
.....................................................................................ugucggcaggauuucaacua.......   1   0   S04
.....................................................................................ugucggcaggauuucaacuaua.....   2   0   S04
.....................................................................................ugucggcaggauuucaacuauG.....   1   1   S04
......................................................................................gucggcaggauuucaacua.......   1   0   S04
......................................................................................gucggcaggauuucaacuaua.....   1   0   S04
......................................................................................gucggcaggauuucaacuauGaau..   1   1   S04
.......................................................................................ucggcaggauuucaacuC.......   1   1   S04
........................................................................................cggcaggauuucaacuauaaaua.   2   0   S04
........................................................................................cggcaggauuucaacuauaaauac   2   0   S04
...........................................................................................caggauuucaacuauaaau..   2   0   S04
...........................................................................................caggauuucaacuauaaaua.   1   0   S04
...........................................................................................caggauuucaacuauaaauac   2   0   S04
............................................................................................aggauuucaacuauaaau..   1   0   S04
```

Provisional ID	:	chrC01_1040543
Score total	:	20
Score for star read(s)	:	3.9
Score for read counts	:	13.4
Score for mfe	:	2.7
Score for randfold	:	0
Score for cons. seed	:	
Total read count	:	38
Mature read count	:	37
Loop read count	:	0
Star read count	:	1

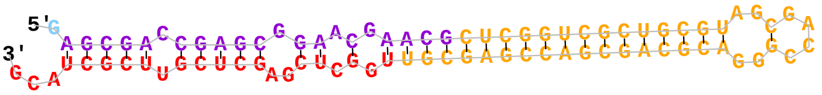

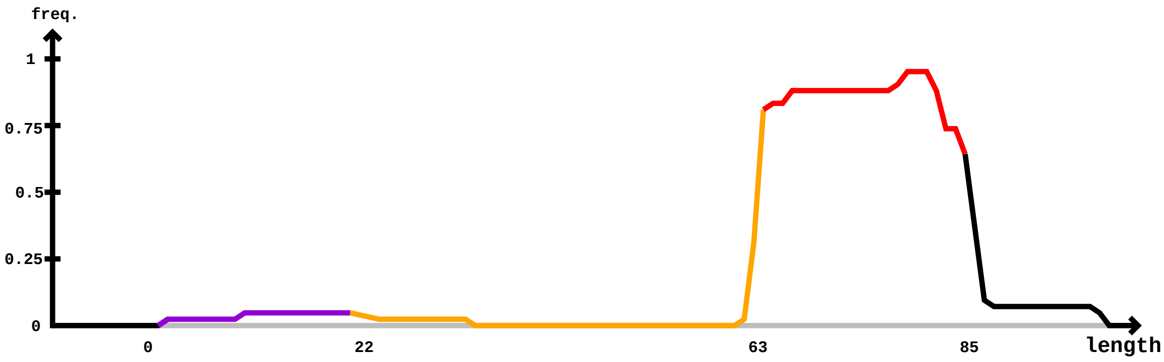

Star **Mature**

```
5'- gucgauacagagcgaccgagcggaacgaacgcucggucgcugcguagcgaccgggacgcagcgaccgagcguuggcucgagcucguucgcuacguaacgaccgagcuucgg   -3'   obs
    gucgauacagagcgaccgagcggaacgaacgcucggucgcugcguagcgaccgggacgcagcgaccgagcguuggcucgagcucguucgcuacguaacgaccgagcuucgg         exp
    ((((.(((..(((((.(((((.((.(.((((((((((((((((((..(....)..)))))))))))))))))).).))..))))).)))))..))).))))..........   reads   mm   sample
    ..........agcgaccgagcggaacgaacA................................................................................   1       1    S03
    ......................................................................guuggcucgagcucguucgcu....................   1       0    S03
    .......................................................................uuggcucgagcucguucgc.....................   2       0    S03
    .......................................................................uuggcucgagcucguucgU.....................   1       1    S03
    .......................................................................uuggcucgagcucguucgcu....................   1       0    S03
    .......................................................................uuggcucgagcucguucgcuaA..................   1       1    S03
    .......................................................................uuggcucgagcucguucgcuac..................   3       0    S03
    .......................................................................uuggcucgagcucguucgcuacg.................   2       0    S03
    .......................................................................uuggcucgagcucguucgcuacgu................   1       0    S03
    .......................................................................uuggcucgagcucguucgcuacgC................   1       1    S03
    ........................................................................uggcucgagcucguucgcu....................   4       0    S03
    ........................................................................uggcucgagcucguucgcuacA.................   6       1    S03
    ........................................................................uggcucgagcucguucgcuacg.................   5       0    S03
    ........................................................................uggcucgagcucguucgcuacgu................   1       0    S03
    ........................................................................Gggcucgagcucguucgcuacgu................   1       1    S03
    ........................................................................uggcucgagcucguucgcuacgC................   1       1    S03
    ........................................................................uggcucgagcucguucgcuacgua...............   1       0    S03
    .........................................................................ggcucgagcucguucgcuacgu................   1       0    S03
    ...........................................................................cucgagcucguucgcuacgu................   1       0    S03
    ......................................................................................ucgcuacguaacgaccgagcuu...   1       0    S03
    .......................................................................................cgcuacguaacgaccgagcu....   1       0    S03

    ..................aUcggaacgaacgcucggucgcug.....................................................................   1       1    S04
    ........................................................................uggcucgagcucguucgcuacg.................   2       0    S04
    ...........................................................................cucgagcucguucgcuacgu................   1       0    S04
    .......................................................................................cgcuacguaacgaccgagcuu...   1       0    S04
```

Provisional ID	:	chrC01_1057249
Score total	:	0.1
Score for star read(s)	:	-1.3
Score for read counts	:	0
Score for mfe	:	1.4
Score for randfold	:	0
Score for cons. seed	:	
Total read count	:	23
Mature read count	:	23
Loop read count	:	0
Star read count	:	0

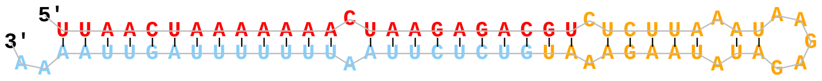

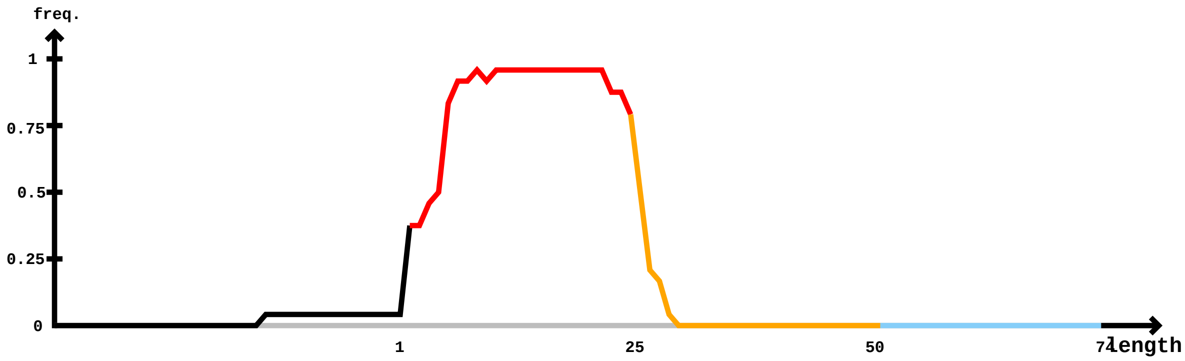

Mature **Star**

```
5'- uuuuuaacguaauauaagauacggauuuuuaguuuuuaacuaaaaaaacuaagagacgucucuuaaauaagagauauaagaaaugucucuuaauuuuuuuaguuaaaaaguaa   -3'    exp
    .......((((........)))).....(((.((((((((((((((((.((((((((((.(((((.((.....)).))))).)))))))))).))))))))))))))))).)))   reads   mm   sample
    ....................acggauuuuuaguuuuuaacuaa......................................................................   1       0    S03
    ...................................uuaacuaaaaaaacuaagaga.........................................................   1       0    S03
    ...................................uuaacuaaaaaaacuaagagU.........................................................   1       1    S03
    ...................................uuaacuaaaaaaacuaagagacg.......................................................   1       0    S03
    ...................................uuaacuaaaaaaacuaagagacgu......................................................   4       0    S03
    ......................................acuaaaaaaacuaagagacgucuA...................................................   1       1    S03
    .......................................cuaaaaaaacuaagagacgu......................................................   3       0    S03
    ............................................aaaacuaagagacgucuc...................................................   1       0    S03

    ...................................uuaacuaaaaaaacuaagagacgu......................................................   1       0    S04
    .....................................aacuaaaaaaacuaagagacg.......................................................   1       0    S04
    .....................................aacuaUaaaaacuaagagacgucu....................................................   1       1    S04
    .......................................cuaaaaaaacuaagagacgu......................................................   4       0    S04
    .......................................cuaaaaaaacuaagagacgucuA...................................................   1       1    S04
    ........................................uaaaaaaacuaagagacgu......................................................   2       0    S04
    ..........................................aaaaaacuaagagacgucucu..................................................   1       0    S04
```

Provisional ID	:	chrC01_1057622
Score total	:	334.1
Score for star read(s)	:	3.9
Score for read counts	:	327.9
Score for mfe	:	2.3
Score for randfold	:	0
Score for cons. seed	:	
Total read count	:	655
Mature read count	:	578
Loop read count	:	74
Star read count	:	3

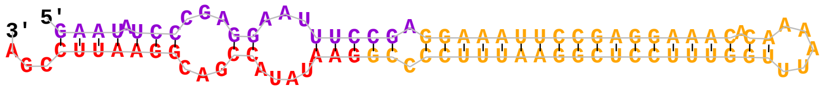

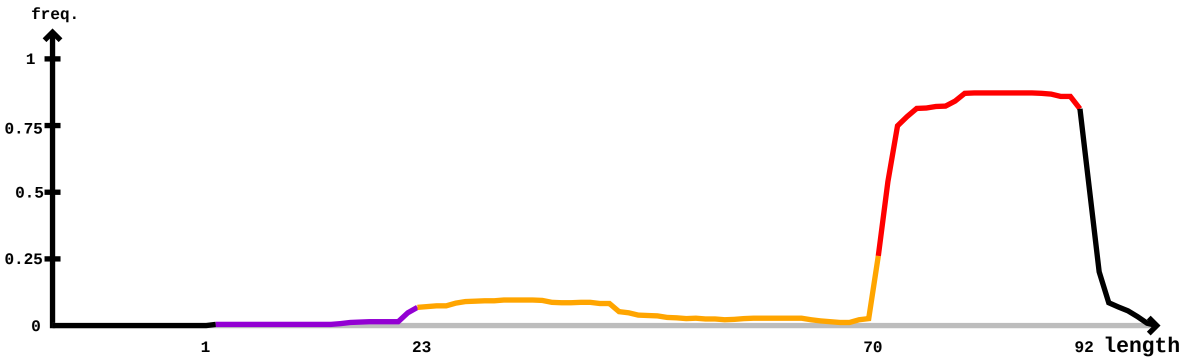

Star Mature

```
5'- uaaaaugaaauccucgaauaucccgaggaauuuccgaggaaauuccgaggaaacacaaaauuugguuuccucggaauuuccccggaauauaccgacggaauuccgaggaaaua   -3'   obs
    uaaaaugaaauccucgaauaucccgaggaauuuccgaggaaauuccgaggaaacacaaaauuugguuuccucggaauuuccccggaauauaccgacggaauuccgaggaaaua         exp
    ..........(((((((((.(((...((...(((((.(((((((((((((((((.((.....))))))))))))))))))).))))).....))...)))))).))))))....    reads   mm   sample
    ............................aauuuccgaggaaauuccgagg...............................................................    1       0    S03
    .............................auuuccgaggaaauuccgagg...............................................................    1       0    S03
    ..............................Guuccgaggaaauuccgagg...............................................................    1       1    S03
    ...............................uuccgaggaaauuccgag................................................................    1       0    S03
    ...................................gaggaaauuccgagCaaac...........................................................    1       1    S03
    ...................................gaggaaauuccgaggaaCcac.........................................................    1       1    S03
    ...................................gaggaaauuccgaggaaacaca........................................................    4       0    S03
    ...................................gaggaaauuccgaggaaacacaaa......................................................    3       0    S03
    ....................................aggaaauuccgaggaaacaca........................................................    2       0    S03
    ....................................aggaaauuccgaggaaacacaCa......................................................    1       1    S03
    ....................................aggaaauuccgaggaaacacaaaa.....................................................    1       0    S03
    .....................................ggaaauuccgaggaaacaca........................................................    1       0    S03
    .....................................ggaaauuccgaggaaacacaaaau....................................................    1       0    S03
    ......................................gaaauuccgaggaaacaca........................................................    2       0    S03
    ......................................gaaauuccgaggaaacaGa........................................................    1       1    S03
    ........................................aauuccgaggaaacacaaaauu...................................................    1       0    S03
    .........................................auuccgaggaaacacaaaauu...................................................    1       0    S03
    .........................................auuccgaggaaacacaaaauuugg................................................    1       0    S03
    ...........................................uccgaggaaacacaaaauu...................................................    1       0    S03
    .............................................cgaggaaacacaaaauuugguuC.............................................    1       1    S03
    ......................................................acaaaauuugguuuccucggaauu...................................    1       0    S03
    .......................................................caaaauuugguuuccucggaauu...................................    1       0    S03
    ...........................................................auuugguuuccucggaau....................................    1       0    S03
    ............................................................uuugguuuccucggaauuu..................................    1       0    S03
    ......................................................................ucggaauuuccccggaauauUc.....................    1       1    S03
    ......................................................................ucggaauuuccccggaauauacc....................    1       0    S03
    .......................................................................cggaauuuccccggaauauU......................    1       1    S03
    ..................................................................................cggaauauaccgacggaau............    1       0    S03
    ..................................................................................cggaauauaccgacggaauuc..........    2       0    S03
    ...................................................................................ggaauauaccgacggaauu...........    1       0    S03
    ...................................................................................ggaauauaccgacggaauuccgag......    1       0    S03
    ...................................................................................ggaauauaccgacggaauuccgagC.....    1       1    S03
    ....................................................................................gaauauaccgacggaauuccg........    6       0    S03
```

```
               Star                                                                  Mature
uaaaaugaaauccucgaauaucccgaggaauuuccgaggaaauuccgaggaaacacaaaauuugguuuccucggaauuuccccggaauauaccgacggaauuccgaggaaaua
....................................................................................gaaCauaccgacggaauuccga.......   1   1   S03
....................................................................................gaauauaccgacggaauAccga.......   1   1   S03
....................................................................................gaauauaccgacggaauuccga.......   41  0   S03
....................................................................................gaauauaccgacggaauuccgG.......   6   1   S03
....................................................................................gaauauaccgacggaauuccgag......   14  0   S03
....................................................................................gaauauaccgacggaauuccgagC.....   1   1   S03
....................................................................................gaauauaccgacggaauuccgagg.....   4   0   S03
.....................................................................................aauauaccgacggaauuccg........   5   0   S03
.....................................................................................aauauaccgCcggaauuccg........   1   1   S03
.....................................................................................aauauaccgacggaauuccga.......   32  0   S03
.....................................................................................aauauaccgacggaauuccgag......   30  0   S03
.....................................................................................aauauaccgacggaauCccgag......   1   1   S03
.....................................................................................aauauaccgacggaauuccgagg.....   4   0   S03
.....................................................................................aauauaccgacggaauuccgagC.....   2   1   S03
.....................................................................................aauauaccgacggaauuccgagga....   3   0   S03
......................................................................................auauaccgacggaauuccg........   5   0   S03
......................................................................................auauaccgacggaauuccUa.......   1   1   S03
......................................................................................auauaccgacggaauuccga.......   28  0   S03
......................................................................................auauaccgacggaauuccgag......   15  0   S03
......................................................................................auauaccgacggaauuccgagC.....   20  1   S03
......................................................................................auauaccgacggaauuccgagg.....   8   0   S03
......................................................................................auauaccgacggaauuccgaggaa...   1   0   S03
.......................................................................................uauaccgacggaauuccga.......   5   0   S03
.......................................................................................uauaccgacggaauuccgag......   3   0   S03
.......................................................................................uauaccgacggaauuccgaggaa...   1   0   S03
.......................................................................................Cauaccgacggaauuccgaggaa...   1   1   S03
.......................................................................................uauaccgacggaauuccgaggaaa..   3   0   S03
........................................................................................auaccgacggaauuccga.......   3   0   S03
........................................................................................auaccgacggaauuccgag......   1   0   S03
........................................................................................auaccgacggaauuccgagg.....   1   0   S03
........................................................................................auaccgacggaauuccgaggaaa..   1   0   S03
........................................................................................auaccgacggaauuccgaggaaau.   1   0   S03
..........................................................................................acAgacggaauuccgaggaa...   1   1   S03
...........................................................................................ccgacggaauuccgagga....   1   0   S03
...........................................................................................ccgacggaauuccgaggaaau.   1   0   S03
............................................................................................cgacggaauuccgaggaa...   3   0   S03
............................................................................................cgacggaauuccgaggaaa..   1   0   S03
............................................................................................cgacggaauuccgaggaaau.   1   0   S03
.............................................................................................gacggaauuccgaggaaa..   2   0   S03
.............................................................................................gacggaauuccgaggaaau.   3   0   S03
.............................................................................................gacggaauuccgaggaaaua   1   0   S03

...............gaauaucccgaggaauuuccga............................................................................   3   0   S04
............................aauuuccgaggaaauuccgagga..............................................................   1   0   S04
.............................auuuccgaggaaauuccgagg...............................................................   2   0   S04
...................................gaggaaauuccgaggaaa............................................................   1   0   S04
...................................gaggaaauCccgaggaaaca..........................................................   1   1   S04
...................................gaggaaauuccgaggaaaca..........................................................   1   0   S04
...................................gGggaaauuccgaggaaaca..........................................................   1   1   S04
...................................gaggaaauuccgagCaaaca..........................................................   1   1   S04
...................................gaggaaauuccgaggaaacaca........................................................   7   0   S04
...................................gaggaaauuccgaggaaacGcaaa......................................................   1   1   S04
...................................gaggaaauuccgaggaaacacaaa......................................................   1   0   S04
....................................aggaaauuccgaggaaac...........................................................   1   0   S04
....................................aggaaauuccgaggaaaca..........................................................   1   0   S04
....................................aggaaauuccgaggaaacac.........................................................   1   0   S04
....................................aggaaauuccgaggaaacaca........................................................   3   0   S04
....................................aggaaauuccgaggaaacacaU.......................................................   2   1   S04
....................................aggaaauuccgaggaaacacaa.......................................................   1   0   S04
....................................aggaaauuccgaggaaacacaaa......................................................   1   0   S04
.....................................ggaaauuccgaggaaacaca........................................................   2   0   S04
........................................aauuccgaggaaacacaaaauu...................................................   1   0   S04
........................................aauuccgaggaaacacaaaauuu..................................................   1   0   S04
........................................aauuccgaggGaacacaaaauuug.................................................   1   1   S04
........................................aauuccgaggaaacacaaaauuug.................................................   3   0   S04
.........................................auuccgaggaaacacaaaauuuggu...............................................   2   0   S04
..........................................uuccgaggaaacacaaaa.....................................................   1   0   S04
.............................................cgaggaaacacaaaauuugguuu.............................................   1   0   S04
.....................................................cacaaaauuugguuuccucggaau....................................   2   0   S04
......................................................acaaaauuugguuuccucggaau....................................   1   0   S04
```

Star **Mature**

```
uaaaaugaaauccucgaauaucccgaggaauuuccgaggaaauuccgaggaaacacaaaauuugguuuccucggaauuuccccggaauauaccgacggaauuccgaggaaaua
.......................................................caaaauuugguuuccucggaauuu..................................  1   0   S04
........................................................aaaauuugguuuccucggaauuuc.................................  2   0   S04
.........................................................aaauuugguuuccucggaauu...................................  1   0   S04
................................................................guuuccucggaauuuccc...............................  1   0   S04
................................................................guuuccucggaauuucccU..............................  1   1   S04
.................................................................uuuccucggaauuucccA..............................  1   1   S04
.................................................................uuuccucggaauuuccccA.............................  1   1   S04
.....................................................................cucggaauuuccccggaauUua......................  1   1   S04
..................................................................................cggaauauaccgacggaauu...........  1   0   S04
..................................................................................cggaauauaccgacggaauuc..........  4   0   S04
...................................................................................ggaauauaccgacggaauuccga.......  1   0   S04
...................................................................................ggaauauaccgacggaauuccgag......  1   0   S04
....................................................................................gaauauaccgacggaauuccg........  5   0   S04
....................................................................................gaauauaccgacggaauuccgU.......  2   1   S04
....................................................................................gaauauaccgacggaauuccUa.......  1   1   S04
....................................................................................gaauauaccgacggaauuccga....... 56   0   S04
....................................................................................gaauauaccgacggaauuccgag...... 16   0   S04
....................................................................................gaauauaccgacggaauuccgGg......  1   1   S04
....................................................................................gaauauaccgacggaauuccgagC.....  2   1   S04
....................................................................................gaauauaccgacggaauuccgagg.....  5   0   S04
....................................................................................gaauauacAgacggaauuccgagg.....  1   1   S04
.....................................................................................aauauaccgacggaauuccg........  5   0   S04
.....................................................................................aauauaccgacggaauuccgC.......  2   1   S04
.....................................................................................aauauaccgacggaauuccga....... 44   0   S04
.....................................................................................aaCauaccgacggaauuccga.......  1   1   S04
.....................................................................................aauauaccgacggaauuccUa.......  1   1   S04
.....................................................................................aauauaccgacggaauuccgG.......  3   1   S04
.....................................................................................aauauaccgacggaauuccgag...... 49   0   S04
.....................................................................................aauauaccgacggaauuccgUg......  1   1   S04
.....................................................................................aauauaccgacggaauuccgagg.....  2   0   S04
.....................................................................................aauauaccgacggaauuccgagC.....  1   1   S04
.....................................................................................aauauaccgacggaauuccgagga....  5   0   S04
......................................................................................auauaccgacggaauuccC........  1   1   S04
......................................................................................auauaccgacggaauuccg........  3   0   S04
......................................................................................auauaccgacggaauuccgG.......  1   1   S04
......................................................................................auauaccgacggaauuccga....... 24   0   S04
......................................................................................auauaccgacggaauuccgag...... 14   0   S04
......................................................................................auauaccgacggaauuccgagC..... 12   1   S04
......................................................................................auauaccgacggaauuccgagg..... 10   0   S04
......................................................................................auauacAgacggaauuccgagga....  1   1   S04
.......................................................................................uauaccgacggaauuccg........  1   0   S04
.......................................................................................uauaccgacggaauuccga.......  7   0   S04
.......................................................................................uauaccgacggaauuccgag......  1   0   S04
.......................................................................................uauaccgacggaauuccgagg.....  2   0   S04
........................................................................................auaccgacggaauuccga.......  5   0   S04
........................................................................................auaccgacggaauuccgag......  6   0   S04
........................................................................................auaccgacggaauuccgagg.....  3   0   S04
.........................................................................................uaccgacggaauuccgag......  1   0   S04
..........................................................................................accgacggaauuccgagg.....  1   0   S04
..........................................................................................accgacggaauuccgaggaaa..  1   0   S04
..........................................................................................accgacggaauuccCaggaaau.  1   1   S04
...........................................................................................ccgacggaauuccgagga....  1   0   S04
............................................................................................cgacggaauuccgaggaa...  3   0   S04
............................................................................................cgacggaauuccgaggaaa..  2   0   S04
............................................................................................cgacggaauuccgaggaaau.  4   0   S04
.............................................................................................gacggaauuccgaggaaa..  5   0   S04
.............................................................................................gacggaauuccgaggaaau.  6   0   S04
.............................................................................................gacggaauuccgaggaaaua  4   0   S04
..............................................................................................acggaauuccgaggaaaua  1   0   S04
```

Provisional ID	:	chrC01_1059518
Score total	:	140.9
Score for star read(s)	:	3.9
Score for read counts	:	135.2
Score for mfe	:	1.8
Score for randfold	:	0
Score for cons. seed	:	
Total read count	:	277
Mature read count	:	265
Loop read count	:	0
Star read count	:	12

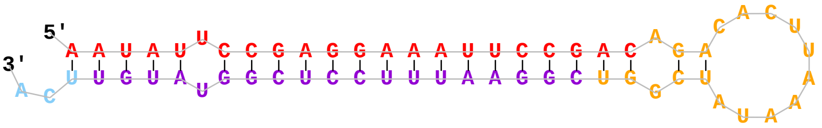

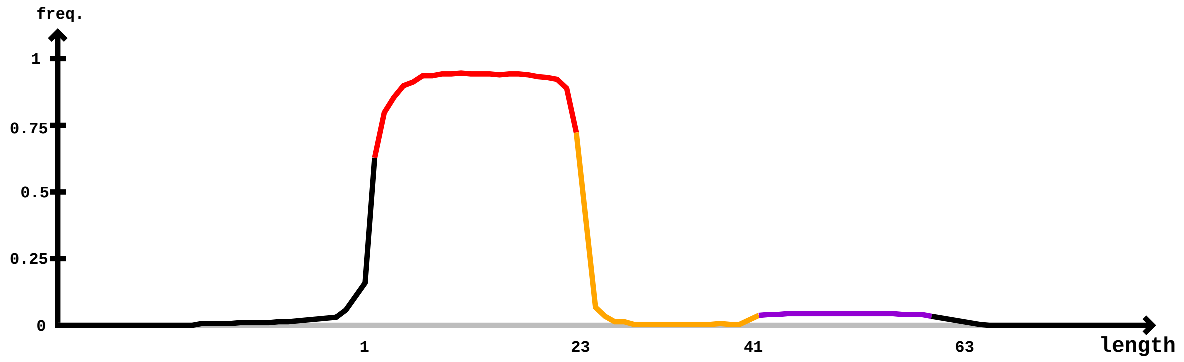

Mature Star

```
5'- auuuggcggcugguuugcccgguuaaaugaaaauauuccgaggaaauuccgacagacacuuaaaauaucggucggaauuuccucgguauguucauuaacucgaggaaaaagaa  -3'  obs
    auuuggcggcugguuugcccgguuaaaugaaaauauuccgaggaaauuccgacagacacuuaaaauaucggucggaauuuccucgguauguucauuaacucgaggaaaaagaa        exp
    .......(((......)))(((((((.(((..((((.((((((((((((((((.((..........)).)))))))))))))))).))))))))))))).))...........  reads  mm  sample
    .................cccgguuaaaugaaaauauuccUa........................................................................  1  1  S03
    ........................aaaugaaaauauuccgaggaaauu.................................................................  1  0  S03
    ............................gaaaauauuccgaggaaauuccga.............................................................  1  0  S03
    ............................gaaaauauCccgaggaaauuccga.............................................................  1  1  S03
    .............................aaaauauuccgaggaaauuccga.............................................................  2  0  S03
    .............................aaaauauuccgaggaaauuccgac............................................................  4  0  S03
    .............................aaaauauuccgaggaaauuccgaca...........................................................  1  0  S03
    ..............................aaauauuccgaggaaauuccg..............................................................  1  0  S03
    ..............................aaauauuccgaggaaauuccga.............................................................  2  0  S03
    ..............................aaauauuccgaggaaauuccgac............................................................  3  0  S03
    ..............................aaauauuccgaggaaauuccgaca...........................................................  2  0  S03
    ...............................aauauuccgaggaaauuc................................................................  1  0  S03
    ...............................aauauuccgaggaaauuccg..............................................................  3  0  S03
    ...............................aauauuccgaggaaauuccgU.............................................................  1  1  S03
    ...............................aauauuccgaggaaauuccga.............................................................  14  0  S03
    ...............................aauauuccgaggaaauuccgac............................................................  21  0  S03
    ...............................aauauuccgGggaaauuccgac............................................................  1  1  S03
    ...............................aauauuccgCggaaauuccgac............................................................  1  1  S03
    ...............................aauauuccgaggaaauuccAac............................................................  2  1  S03
    ...............................aauauuccgaggaaauucUgaca...........................................................  1  1  S03
    ...............................aauauuccgaggaaauuccgaUa...........................................................  1  1  S03
    ...............................aauauuccgaggaaauuccgaca...........................................................  17  0  S03
    ...............................aauauuccgaggaaGuuccgaca...........................................................  1  1  S03
    ...............................aauauuccgaggaaauuccAaca...........................................................  4  1  S03
    ...............................aauauuccgaggaaauuccgacC...........................................................  1  1  S03
    ...............................aauauuccgaggaaauuccgacag..........................................................  6  0  S03
    ...............................aauauuccgaggaaauuccgacaU..........................................................  1  1  S03
    ................................auauuccgaggaaauuccg..............................................................  2  0  S03
    ................................auauuccgaggaaauuccga.............................................................  2  0  S03
    ................................auauuccgaggaaauuccAac............................................................  1  1  S03
    ................................auauuccgaggaaauuccgac............................................................  7  0  S03
    ................................Nuauuccgaggaaauuccgaca...........................................................  1  1  S03
    ................................auauuccgaggaaauuccgacC...........................................................  1  1  S03
```

Mature Star

```
auuuggcggcugguuugcccgguuaaaugaaaauauuccgaggaaauuccgacagacacuuaaaauaucggucggaauuuccucgguaugucauuaacucgaggaaaaagaa
................................auauuccgaggaaauuccAaca...........................................................    1    1    S03
................................auauuccgaggaaauuccgaca...........................................................    6    0    S03
................................auauuccgaggaaauuccgacaga.........................................................    1    0    S03
.................................uauuccgaggaaauuccg..............................................................    1    0    S03
.................................uauuccgaggaaauuccgac............................................................    3    0    S03
.................................uauuccgaggaaauuccgaca...........................................................    4    0    S03
..................................auuccgaggaaauuccga.............................................................    2    0    S03
..................................auuccgaggaaauuccgac............................................................    2    0    S03
..................................auuccgaggaaUuuccgaca...........................................................    2    1    S03
..................................auuccgaggaaauuccgaca...........................................................    5    0    S03
...................................uuccgaggaaauucUgacagaca.......................................................    1    1    S03
....................................uccgaggaaauuccgaca...........................................................    1    0    S03
.............................................auuccgacagacacuuaaaauauc............................................    1    0    S03
.......................................................................ucggaauuuccucgguaugu......................    2    0    S03
.......................................................................ucggaauuuccucgguauguu.....................    1    0    S03
........................................................................cggaauuuccucgguaugC......................    1    1    S03
........................................................................cggaauuuccucgguaugG......................    1    1    S03
........................................................................cggaauuuccucgguaugu......................    1    0    S03

.............uuugcccgguuaaaugaaaaC...............................................................................    1    1    S04
.............uuugcccgguuaaaugaaaau...............................................................................    1    0    S04
.....................guuGaaugaaaauauuccgagga.....................................................................    1    1    S04
.......................uaaaugaaaauauuccgaggaaau..................................................................    1    0    S04
.........................aaugaaaauauuccgaggaaCuu.................................................................    1    1    S04
..........................augaaaauauuccgaggaaauucc...............................................................    1    0    S04
...........................ugaaaauauuccgaggaaauuccg..............................................................    1    0    S04
............................gaaaauauuccgaggaaauuccCa.............................................................    1    1    S04
............................gaaaauauuccgaggaaauuccga.............................................................    5    0    S04
.............................aaaauauuccgaggaaauuccgac............................................................    7    0    S04
.............................aaaauauuccgGggaaauuccgac............................................................    1    1    S04
..............................aaauauuccgaggaaauuccga.............................................................    3    0    S04
..............................aaauauuccgaggaaauuccgac............................................................    3    0    S04
..............................aaauaCuccgaggaaauuccgaca...........................................................    1    1    S04
...............................aauauuccgaggaaauucc...............................................................    1    0    S04
...............................aauauuccgaggaaauuccgU.............................................................    1    1    S04
...............................aauauuccgaggaaauuccga.............................................................    7    0    S04
...............................aauauuccgUggaaauuccga.............................................................    1    1    S04
...............................aauauuccgaggaaauuccAac............................................................    7    1    S04
...............................aauauuccgaggaaauuccgac............................................................    24   0    S04
...............................aauauuccgaggaaauuccAaca...........................................................    3    1    S04
...............................aUuauuccgaggaaauuccgaca...........................................................    1    1    S04
...............................aauauuccgaCgaaauuccgaca...........................................................    1    1    S04
...............................aauauuccgaggaaauucUgaca...........................................................    1    1    S04
...............................aauauuccgaggaaauuccgaca...........................................................    14   0    S04
...............................aauauuccgagUaaauuccgacag..........................................................    1    1    S04
...............................aauauuccgaggaaauuccgacaC..........................................................    1    1    S04
................................auauuccgaggaaauuccg..............................................................    2    0    S04
................................auauuccgaggaaauuccga.............................................................    5    0    S04
................................auauuccgaggaaauuccgac............................................................    5    0    S04
................................auauuccgaggaaauuccgaca...........................................................    11   0    S04
................................aCauuccgaggaaauuccgaca...........................................................    1    1    S04
................................auauuUcgaggaaauuccgaca...........................................................    1    1    S04
................................Uuauuccgaggaaauuccgaca...........................................................    1    1    S04
................................auauuccgaggaaauuccgacaga.........................................................    1    0    S04
................................auauuccgaggaaauuccgacagU.........................................................    1    1    S04
.................................uauuccgaggaaauuccga.............................................................    1    0    S04
.................................uauuccgaggaaauuccgac............................................................    1    0    S04
.................................uauuccgaggaaauuccgacC...........................................................    1    1    S04
.................................uauuccgaggaaauuccgaca...........................................................    3    0    S04
.................................uauuccgaggaaauuccgacaU..........................................................    1    1    S04
.................................uauuccgaggaaauuccgacaUa.........................................................    1    1    S04
.................................uauuccgaggaaauucUgacaga.........................................................    1    1    S04
..................................auuccgaggaaauuccgac............................................................    1    0    S04
..................................auuccgaggaCauuccgaca...........................................................    1    1    S04
..................................auuccgaggaaauuccgaca...........................................................    2    0    S04
...................................uuccgaggaaauuccgac............................................................    1    0    S04
...................................uuccgaggaaauuccgaca...........................................................    2    0    S04
....................................uccgaggaaauuccgaca...........................................................    6    0    S04
......................................cgaggaaauuccgacaga.........................................................    1    0    S04
......................................cgaggGaauuccgacagaca.......................................................    1    1    S04
```

```
                               Mature                                 Star
auuuggcggcugguuugcccgguuaaaugaaaauauuccgaggaaauuccgacagacacuuaaauaucggucggaauuuccucgguauguucauuaacucgaggaaaaagaa
.......................................aggaaauuccgacagaca.......................................................    1    0    S04
...................................................................cggucggaauuuccucggu..........................    1    0    S04
......................................................................ucggaaAuuccucgguaug.......................    1    1    S04
......................................................................ucggaauuuccucgguauguu.....................    1    0    S04
.......................................................................cggaauuuccucgguaug.......................    1    0    S04
.......................................................................cggaauuuccucgguaugu......................    1    0    S04
........................................................................ggaauuuccucgguaugu......................    1    0    S04
..........................................................................aauuuccucgguauguucauu.................    1    0    S04
```

Provisional ID	:	chrC01_1078374
Score total	:	49.9
Score for star read(s)	:	3.9
Score for read counts	:	89.9
Score for mfe	:	-43.8
Score for randfold	:	0
Score for cons. seed	:	
Total read count	:	188
Mature read count	:	187
Loop read count	:	0
Star read count	:	1

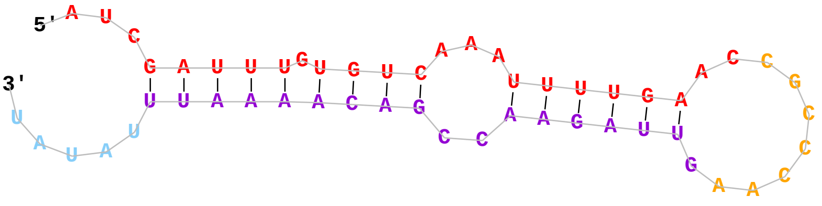

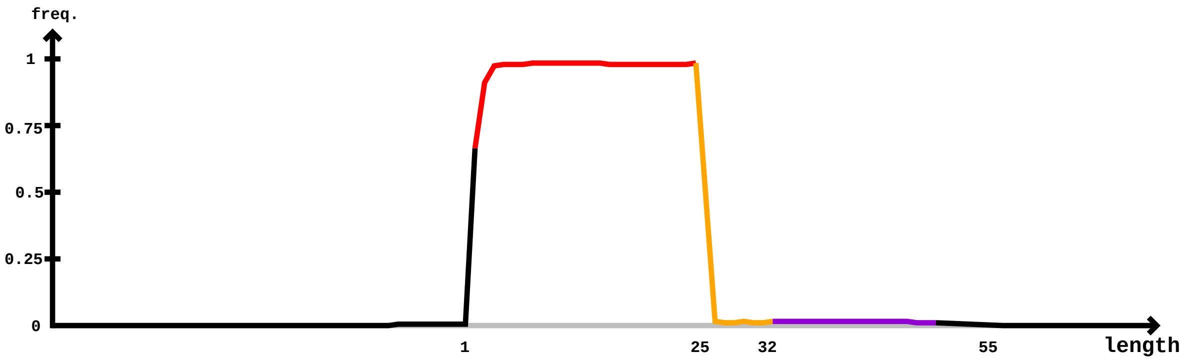


```
                                           Mature                    Star
5'- auauacauagaauuuucuaaaaauuuauuaucuaauuauuuuaucgauuugugucaaauuuugaaccgcccaaguuagaaccgacaaaauuuauauuuauagagauuauuaau  -3'  obs
    auauacauagaauuuucuaaaaauuuauuaucuaauuauuuuaucgauuugugucaaauuuugaaccgcccaaguuagaaccgacaaaauuuauauuuauagagauuauuaau       exp
    .......((((....)))).....(((..((((...((...(((.(((((.((((...((((((..........))))))..))))))))).)))..))...))))...))).   reads   mm   sample
    ..................................auuauuuuaucgauuuguguca.........................................................   1       0    S03
    ..........................................aucgauuugugucaaauuuugCac...............................................   1       1    S03
    ..........................................aCcgauuugugucaaauuuugaac...............................................   2       1    S03
    ..........................................aucgauuugugucaaauuuugaac...............................................   39      0    S03
    ..........................................aucgauuugugucaaauuuugUac...............................................   1       1    S03
    ..........................................aucgauuugugucaaauuuugaacc..............................................   3       0    S03
    ...........................................ucgauuugugucaaauuuugaacA..............................................   1       1    S03
    ...........................................ucgauuugugucaaauuuugaacc..............................................   13      0    S03
    ...........................................ucgauuugugucaaaCuuugaacc..............................................   1       1    S03
    ............................................cgauuugugucaaauuuugaac...............................................   1       0    S03
    ............................................cgauuugugucaaauuuugaacc..............................................   2       0    S03
    ......................................................................caaguuagaaccgacaaaauuua....................   1       0    S03

    ..........................................aucgauuugugucaaauuuugaGc...............................................   3       1    S04
    ..........................................aucgauuugugucaaauuuugaac...............................................   70      0    S04
    ..........................................aucgauuugugucaaauuuugaacc..............................................   7       0    S04
    ...........................................ucgauuugugucaaauuuugaac...............................................   1       0    S04
    ...........................................ucgauuugugucaaauuuugaGcc..............................................   1       1    S04
    ...........................................ucgauuugugucaaauuuugaacc..............................................   27      0    S04
    ...........................................ucgauuugugucaaaCuuugaacc..............................................   1       1    S04
    ...........................................ucgauuugugucaaauuuugaacA..............................................   2       1    S04
    ............................................cgauuugugucaaauuuugaac...............................................   2       0    S04
    ............................................cgauuugugucaaauuuugaacA..............................................   1       1    S04
    ............................................cgauuugugucaaauuuugaacc..............................................   5       0    S04
    ............................................cgauuugugucaaauuuugaaccg.............................................   1       0    S04
    .............................................gauuugugucaaauuuugaacc..............................................   1       0    S04
    ................................................uugugucaaauuuugaaccgccG..........................................   1       1    S04
    .................................................................ccgcccaaguuagaaccgacaaU.........................   1       1    S04
    .........................................................................guuagaaccgGcaaaauu......................   1       1    S04
```

Provisional ID	: chrC01_1094641
Score total	: 0.9
Score for star read(s)	: -1.3
Score for read counts	: 0
Score for mfe	: 2.2
Score for randfold	: 0
Score for cons. seed	:
Total read count	: 27
Mature read count	: 26
Loop read count	: 0
Star read count	: 1

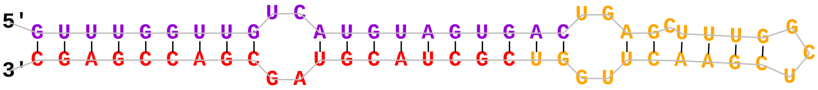

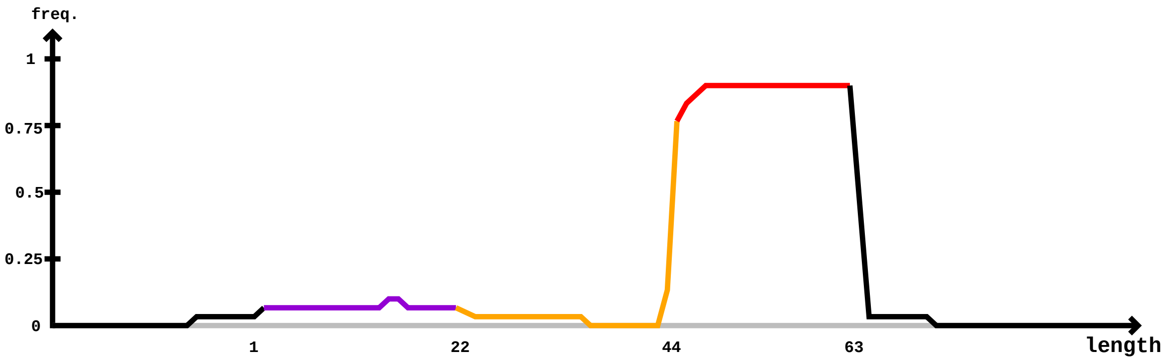


```
                    Star                                         Mature
5'- agcgaccgagcggaaugggcguuugguugucauguagugacugagcuuuggcucgaacuuggucgcuacguagcgaccgagcggagcacacguuuguucgcugcguaacga  -3'   obs
    agcgaccgagcggaaugggcguuugguugucauguagugacugagcuuuggcucgaacuuggucgcuacguagcgaccgagcggagcacacguuuguucgcugcguaacga         exp
    (((((.((((((.......((((((((((..((((((((((..((.((((...))))))..))))))))))..)))))))))).......)))))).)))))......... reads    mm    sample
    ....................guuugguugucauguagugac...................................................................... 1        0     S03
    ..............................................................ucgcuacguagcgaccgagc............................. 3        0     S03
    ...............................................................cgcuacguagcgaccgagc............................. 17       0     S03
    ................................................................gcuacguagcgaccgagc............................. 1        0     S03
    ................................................................gcuacUuagcgaccgagcg............................ 1        1     S03
    .................................................................cuacguagcgaccgagcg............................ 1        0     S03

    .............aaugggcguuugguuguUaugu............................................................................ 1        1     S04
    .................................guagugacugagcuuuggcuc......................................................... 1        0     S04
    ..............................................................ucgcuacguagcgaccgagc............................. 1        0     S04
    ...............................................................cgcuacguagcgaccgagc............................. 1        0     S04
    ...............................................................cgcuacguagcgaccgagcg............................ 1        0     S04
    ..................................................................uacguagcgaccgagcggaAcaca..................... 1        1     S04
```

Provisional ID	:	chrC01_1105894
Score total	:	0.1
Score for star read(s)	:	3.9
Score for read counts	:	10.3
Score for mfe	:	-14
Score for randfold	:	0
Score for cons. seed	:	
Total read count	:	32
Mature read count	:	18
Loop read count	:	12
Star read count	:	2

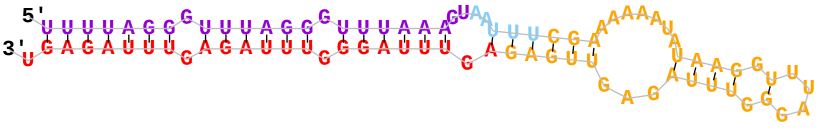

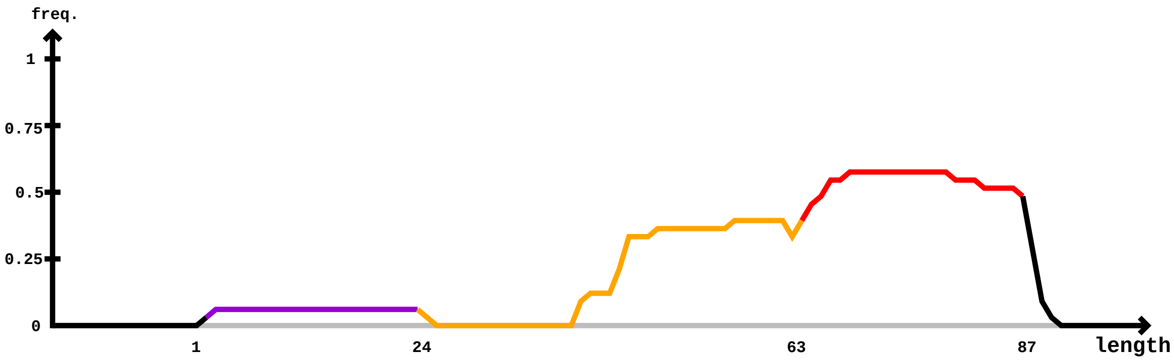


```
                    Star                                                          Mature
5'- auugucuuuuaugguuuuaggguuuaggguuuaaaguaauuucgaaaaaauauaagguuuaggguuuagaguugagaguuuaggguuuagaguuuagaguugaagguuuggg  -3'   obs
    auugucuuuuaugguuuuaggguuuaggguuuaaaguaauuucgaaaaaauauaagguuuaggguuuagaguugagaguuuaggguuuagaguuuagaguugaagguuuggg         exp
    .....((((((...(((((((.((((((.((((((....((((((.......((((.(....).))))...)))))).)))))).)))))).))))))).))))))......   reads   mm   sample
    ...............uuuaggguuuaggguuuaaagG...........................................................................   1       1    S03
    .....................................................aagguuuaggguuuagaguugaga...................................   1       0    S03
    ......................................................agguuuaggguuuagaguug......................................   1       0    S03
    .........................................................uuuaggguuuagaguuga.....................................   1       0    S03
    .........................................................uuuaggguuuagaguugag....................................   1       0    S03
    ..........................................................uuaggguuuagaguugag....................................   2       0    S03
    ..........................................................uuaggguuuagaguugaga...................................   1       0    S03
    ............................................................................aguuuaggguuuagaguuuagag.............   1       0    S03
    ............................................................................aguuuaggguuuagaguuuagagu............   3       0    S03
    .............................................................................guuuaggguuuagaguuuagaguu...........   2       0    S03
    ...............................................................................uuaggguuuagaguuuagagu............   1       0    S03
    .................................................................................aggguuuagaguuuagaguu...........   1       0    S03
    .................................................................................aggAuuuagaguuuagaguug..........   1       1    S03

    ..............uuuuUggguuuaggguuuaaagu...........................................................................   1       1    S04
    .....................................................aagguuuaggguuuagagAugaga...................................   1       1    S04
    .....................................................aagUuuuaggguuuagaguugaga...................................   1       1    S04
    .........................................................uuuaggguuuagaguuga.....................................   1       0    S04
    ..........................................................uuaggguuuagaguugag....................................   1       0    S04
    .............................................................ggguuuagaguugagagGuu...............................   1       1    S04
    .....................................................................aguugagaguuuaggguuuagaA....................   1       1    S04
    ..........................................................................agaguuuaggguuuagaguu..................   1       0    S04
    ............................................................................aguuuaggguuuagaguuuagagG............   1       1    S04
    ............................................................................aguuuaggguuuagaguuuagagu............   1       0    S04
    .............................................................................guuuaggguuuagaguuuagagu............   3       0    S04
    .............................................................................Uuuuaggguuuagaguuuagaguug..........   1       1    S04
    ..............................................................................uuuaggguuuagaguuuagagu............   1       0    S04
    ...............................................................................uuaggguuuagaguuuagaguugU.........   1       1    S04
```

Provisional ID	:	chrC01_1113776
Score total	:	0.3
Score for star read(s)	:	3.9
Score for read counts	:	-4.9
Score for mfe	:	1.4
Score for randfold	:	0
Score for cons. seed	:	
Total read count	:	2
Mature read count	:	1
Loop read count	:	0
Star read count	:	1

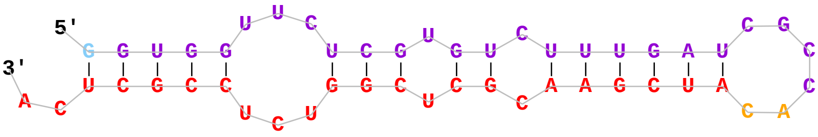

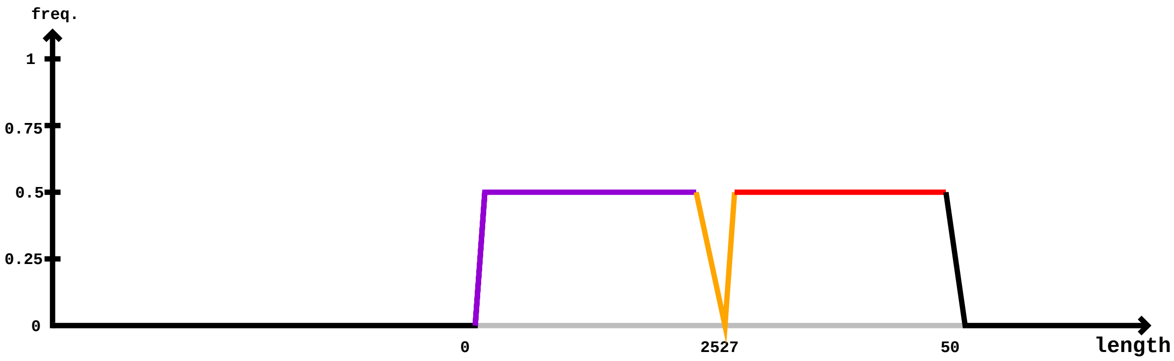

Star Mature

```
5'- cgccaauuucgguuugucgguucucgacggcuacgggcaugugguggguucucgugucuuugaucgccacaucgaacgcucggucuccgcucacaucccagcaccucguaggu   -3'   obs
    cgccaauuucgguuugucgguucucgacggcuacgggcaugugguggguucucgugucuuugaucgccacaucgaacgcucggucuccgcucacaucccagcaccucguaggu         exp
    .(((...........((((.....)))).(((..(((.(((((((((...(((.((.((((((......)))))).)).)))...)))).)))))))))))........)))   reads   mm   sample
    .....................................................................aucgaacgcucggucuccgcucG....................   1       1    S03

    ...........................................gugguucucgugucuuugaucgcA.............................................   1       1    S04
```

Provisional ID	: chrC01_random_2784817	
Score total	:	0
Score for star read(s)	:	-1.3
Score for read counts	:	-1.3
Score for mfe	:	2.7
Score for randfold	:	0
Score for cons. seed	:	
Total read count	:	9
Mature read count	:	3
Loop read count	:	6
Star read count	:	0

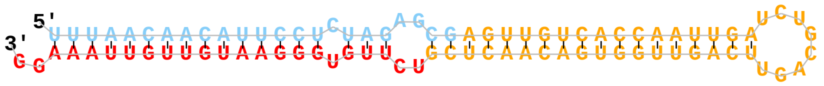

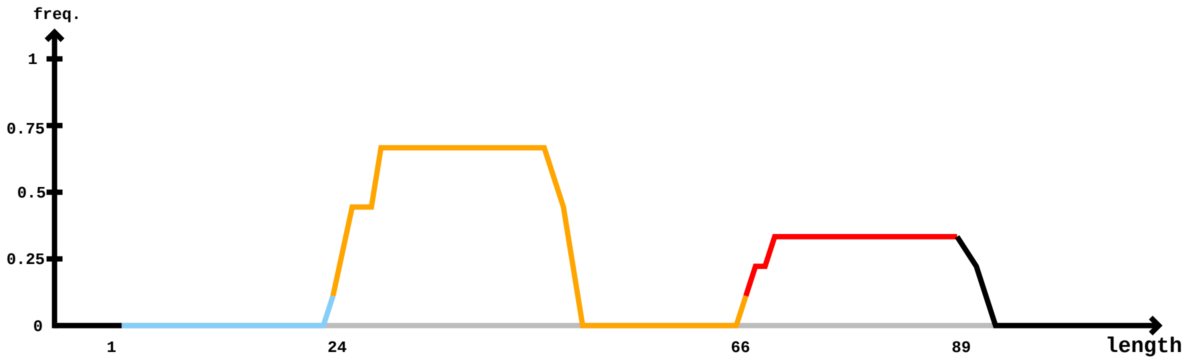

Star Mature

```
5'- cacccuuuaacaacauuccucuagagcgaguugucaccaauugaucugcaguucaguuggugacaacucgucuugugggaauguuguuaaagggugagguauuagauucgaau   -3'    exp
    ((((((((((((((((((((.(((..((((((((((((((((((........))))))))))))))))))..))).))))))))))))))))))))).................    reads      mm         sample
    ............................aguugucaccaauugaAcugcagu.............................................................    1          1          S03
    ............................aguugucaccaauugaucugcagu.............................................................    1          0          S03

    ...........................gaguugucaccaauugaucugca...............................................................    1          0          S04
    ..............................guugucaccaauugaAcugcaguu...........................................................    1          1          S04
    ................................gucaccaauugaucugcUg..............................................................    1          1          S04
    ................................gucaccaauugaucugcaCuu............................................................    1          1          S04
    ......................................................................ucuugugggaauguuguuaaagg....................    1          0          S04
    .......................................................................cuugugggaauguuguuaaagggu..................    1          0          S04
    .........................................................................ugugggaauguuguuaaaggguA.................    1          1          S04
```

Provisional ID	:	chrC01_random_2798728
Score total	:	0.8
Score for star read(s)	:	3.9
Score for read counts	:	-4.4
Score for mfe	:	1.3
Score for randfold	:	0
Score for cons. seed	:	
Total read count	:	3
Mature read count	:	2
Loop read count	:	0
Star read count	:	1

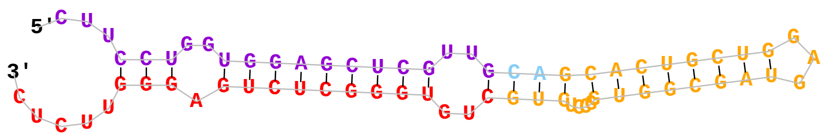

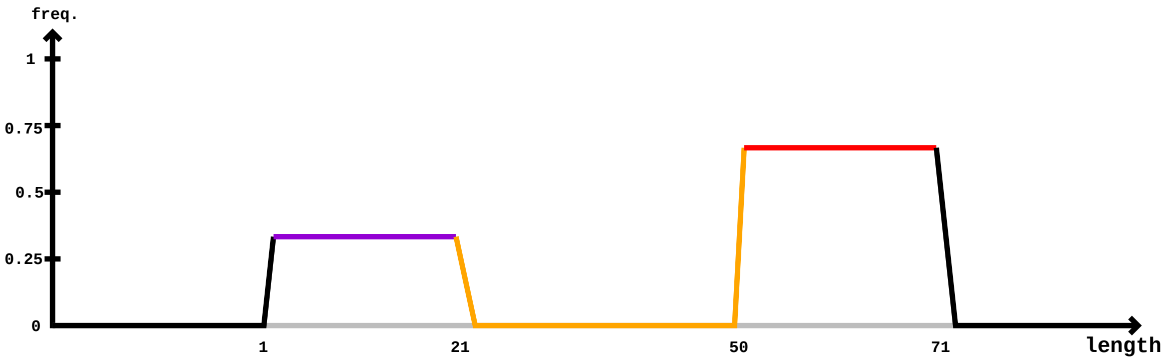

Star Mature

```
5'- uugggcaaacacaaaguggcucuuccugguggagcucguugcagcacugcuggaguagcgguggccucugcugugggcucugagguucuccaugucgucuucaaaaucaa   -3'    obs
    uugggcaaacacaaaguggcucuuccugguggagcucguugcagcacugcuggaguagcgguggccucugcugugggcucugagguucuccaugucgucuucaaaaucaa          exp
    ..((((..(((.....(((.....(((..(((((((((..((((((((((((...))))))))....))))..))))))))).)))....)))))).)))).........    reads     mm        sample
    .....................cuuccugguggagcucguug.....................................................................    1         0         S04
    ......................................................................cugugggcucugagguucuc....................    2         0         S04
```

Provisional ID	:	chrC02_1135776
Score total	:	1.1
Score for star read(s)	:	-1.3
Score for read counts	:	0
Score for mfe	:	2.4
Score for randfold	:	0
Score for cons. seed	:	
Total read count	:	409
Mature read count	:	407
Loop read count	:	0
Star read count	:	2

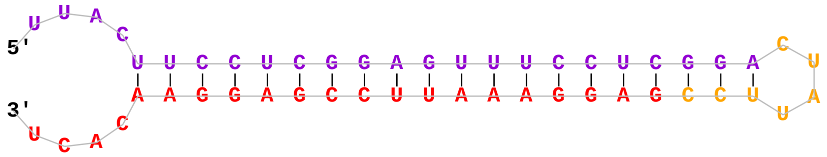

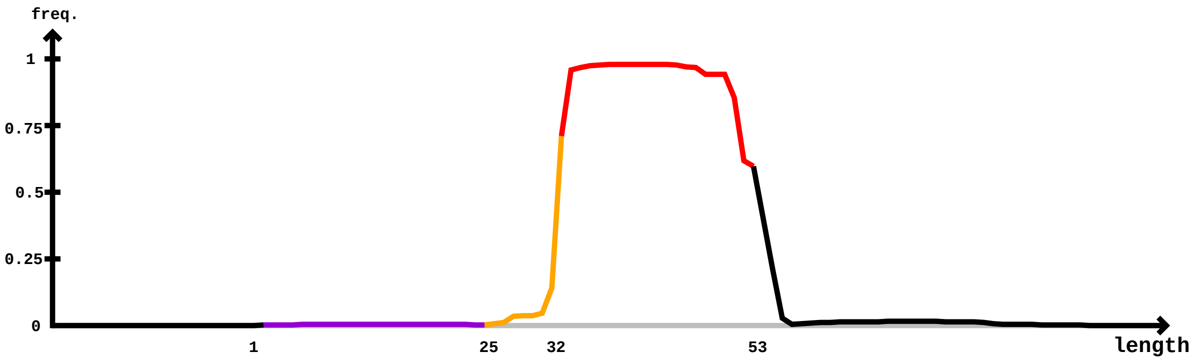

Star **Mature**

```
5'- uuguuuucgguuuaaaaaccuuacuuccucggaguuuccucggacuauuccgaggaaauuccgaggaacacuuggauuuccucggaaauucuucggaauauuccgaggcuuuuc  -3'  obs
    uuguuuucgguuuaaaaaccuuacuuccucggaguuuccucggacuauuccgaggaaauuccgaggaacacuuggauuuccucggaaauucuucggaauauuccgaggcuuuuc        exp
    ........((((....))))....((((((((((((((((((((....)))))))))))))))))))).....(((...((((((((((((....)))).))))))))...)))   reads  mm  sample
    ....................uuacuuccucggaguuuccucgga......................................................................   1      0   S03
    ........................uuccucggaguuuccucU........................................................................   1      1   S03
    .............................................uauuccgaggaaauuccg...................................................   1      0   S03
    .............................................uauuccgaggaaauuccgagU................................................   1      1   S03
    .............................................uauuccgaggaaauuccgagg................................................   2      0   S03
    ..............................................auuccgaggaaauuccga..................................................   2      0   S03
    ..............................................Guuccgaggaaauuccgagg................................................   1      1   S03
    ..............................................auuccgaggaaauuccgagg................................................   2      0   S03
    ..............................................auuccgaggaaauuccgagU................................................   1      1   S03
    ...............................................uuccgaggaaauuccgag.................................................   1      0   S03
    .................................................ccgaUgaaauuccgaggaaca............................................   1      1   S03
    ..................................................cgaUgaaauuccgaggaac.............................................   1      1   S03
    ..................................................cgaggaaauuccgaggaac.............................................   1      0   S03
    ..................................................cgaUgaaauuccgaggaaca............................................   1      1   S03
    ..................................................cgaggaaauuccgaggaaca............................................   1      0   S03
    ..................................................cgaggaaauuccgaggaacacG..........................................   1      1   S03
    ..................................................cgaggaaauuccgaggaacacu..........................................   4      0   S03
    ..................................................cgaggaaauuccgaggaacacuu.........................................   1      0   S03
    ..................................................cgaggaaauuccgaggaacacuug........................................   2      0   S03
    ..................................................Agaggaaauuccgaggaacacuug........................................   1      1   S03
    ..................................................cgaggaaauuccgaggaacacuugC.......................................   1      1   S03
    ...................................................gaggaaauuccgaggaac.............................................   10     0   S03
    ...................................................gaggGaauuccgaggaaca............................................   3      1   S03
    ...................................................gaggaaauuccgaggaaca............................................   11     0   S03
    ...................................................gaggaaauuccgaUgaaca............................................   1      1   S03
    ...................................................gaggaaauuccgaggCacac...........................................   1      1   S03
    ...................................................gaggaaauuccgaggaacac...........................................   3      0   S03
    ...................................................gaggaaauuccgaggaacacu..........................................   40     0   S03
    ...................................................gaggaaauuccgaggaacacG..........................................   2      1   S03
    ...................................................gaggaaauuccgaggaaUacu..........................................   1      1   S03
    ...................................................gaggaaauuccgaggaacacuu.........................................   2      0   S03
    ...................................................gaggaaauuccgaggaacUcuu.........................................   1      1   S03
    ...................................................gaggaaauuccgaggaacacuug........................................   3      0   S03
```

Star Mature

```
uuguuuucgguuuaaaaaccuuacuuccucggaguuuccucggacuauuccgaggaaauuccgaggaacacuuggauuuccucggaaauucuucggaauauuccgaggcuuuuc
...................................................gaggaaauuccgaggaacacuugg.......................................	3	0	S03
....................................................aggGaauuccgaggaaca............................................	2	1	S03
....................................................aggaaauuccgaggaaca............................................	7	0	S03
....................................................aggaaauuccgaggaacac...........................................	1	0	S03
....................................................aggGaauuccgaggaacac...........................................	1	1	S03
....................................................aggaaauuccgaggaacacu..........................................	4	0	S03
....................................................aggaaauuccgaggaacacuu.........................................	6	0	S03
....................................................aggaaauuccgaggaacUcuu.........................................	1	1	S03
....................................................aggaaauuccgaggaGcacuu.........................................	1	1	S03
....................................................aUgaaauuccgaggaacacuug........................................	1	1	S03
....................................................aggaaauuccgaggaacacuuC........................................	1	1	S03
....................................................aggaaauuccgaggaacacuug........................................	15	0	S03
....................................................aggaaauuccgaggaacacuugg.......................................	1	0	S03
.....................................................ggaaauuccgaggaacacuG.........................................	1	1	S03
.....................................................ggaaauuccgaggaacacuug........................................	1	0	S03
......................................................gaaauuccgaggaacacu..........................................	2	0	S03
........................................................aaGuccgaggaacacuug........................................	1	1	S03
...........................................................................auuuccucggaaaAucuucg...................	1	1	S03
................................................................................cucggaaCuucuucggaauau.............	1	1	S03

.............................................uauuccgaggaaauuccga..................................................	1	0	S04
..............................................auuccgaggaaauuccgagg................................................	4	0	S04
.................................................ccgaUgaaauuccgaggaac.............................................	1	1	S04
.................................................ccgaUgaaauuccgaggaaca............................................	2	1	S04
..................................................cgaUgaaauuccgaggaac.............................................	2	1	S04
..................................................cgaggaaauuccgaggaaca............................................	2	0	S04
..................................................cgaUgaaauuccgaggaaca............................................	1	1	S04
..................................................cgaggaaauuccgaUgaaca............................................	1	1	S04
..................................................cgaggaaauuccgaggaacacu..........................................	5	0	S04
..................................................cgaggaaauuccgaUgaacacu..........................................	1	1	S04
..................................................cgaggaaauuccgaggaacacuG.........................................	2	1	S04
..................................................Agaggaaauuccgaggaacacuug........................................	2	1	S04
..................................................cgaggaaauuccgaggaacacuug........................................	10	0	S04
..................................................cgaggaaauuccgaggaacacuGg.......................................	1	1	S04
...................................................gaggaaauuccgaggaac.............................................	23	0	S04
...................................................gaggaaauuAcgaggaaca............................................	1	1	S04
...................................................gaggaaauuccgaggaaca............................................	40	0	S04
...................................................gaggGaauuccgaggaaca............................................	2	1	S04
...................................................gaUgaaauuccgaggaaca............................................	2	1	S04
...................................................gaggaaauuccgaggaacac...........................................	2	0	S04
...................................................gaggaaauuccgaggaacacG..........................................	7	1	S04
...................................................gaggaaauuccgaggaacacu..........................................	53	0	S04
...................................................gaggaGauuccgaggaacacu..........................................	2	1	S04
...................................................gaggaaauuccgGggaacacu..........................................	1	1	S04
...................................................gaggaaauuccgCggaacacu..........................................	1	1	S04
...................................................gaggaaauuccgUggaacacu..........................................	1	1	S04
...................................................gaggaaauuccgaggaacacuC.........................................	1	1	S04
...................................................gaggaaauuccgCggaacacuu.........................................	1	1	S04
...................................................gaggaaauuccgaggaacacuu.........................................	7	0	S04
...................................................gaggaaauuccgaggaacacuug........................................	13	0	S04
...................................................gaggaaauuccgaggaacaGuug........................................	1	1	S04
...................................................gaggaaauuccgaggaacaAuug........................................	1	1	S04
...................................................gaggaaauuccgaggaacacuugg.......................................	4	0	S04
...................................................gaggaaauuccgaggGacacuugg.......................................	1	1	S04
...................................................gaggaaauuccgaggaacacuugC.......................................	1	1	S04
....................................................aggaaauuccgaggaaca............................................	24	0	S04
....................................................aggaaauuccgaggaacac...........................................	1	0	S04
....................................................agUaaauuccgaggaacac...........................................	1	1	S04
....................................................aggaaauuccgaggaacacu..........................................	10	0	S04
....................................................aggaaauuccgaggaacacuu.........................................	5	0	S04
....................................................aggaaauuccgaggaacacuuU........................................	1	1	S04
....................................................aggaaauuccgaggaacacuug........................................	24	0	S04
.....................................................ggaaauuccgaggaacacu..........................................	1	0	S04
.....................................................ggaaGuuccgaggaacacuu.........................................	1	1	S04
......................................................gaaauuccgaggaacacuu.........................................	1	0	S04
.......................................................aaauuccgaggaacacuug........................................	1	0	S04
.......................................................................uuggauuucGucggaaauuc.......................	1	1	S04
............................................................................uuuccucggaaauucuucggC.................	1	1	S04
.............................................................................uuccucggaaauucuucgg..................	1	0	S04
..............................................................................uccucggaaauucuucgg..................	1	0	S04
```

Star Mature

```
uuguuuucgguuuaaaaaccuuacuuccucggaguuuccucggacuauuccgaggaaauuccgaggaacacuuggauuuccucggaaauucuucggaauauuccgaggcuuuuc
.....................................................................................aaauucuucggaauauuccga........    1    0    S04
```

Provisional ID	:	chrC02_1148397
Score total	:	59.1
Score for star read(s)	:	3.9
Score for read counts	:	58.2
Score for mfe	:	-2.9
Score for randfold	:	0
Score for cons. seed	:	
Total read count	:	126
Mature read count	:	113
Loop read count	:	0
Star read count	:	13

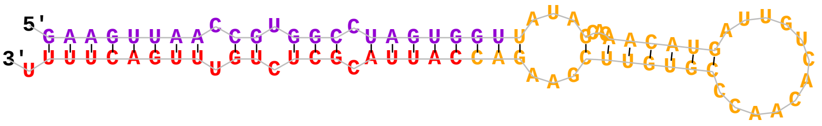

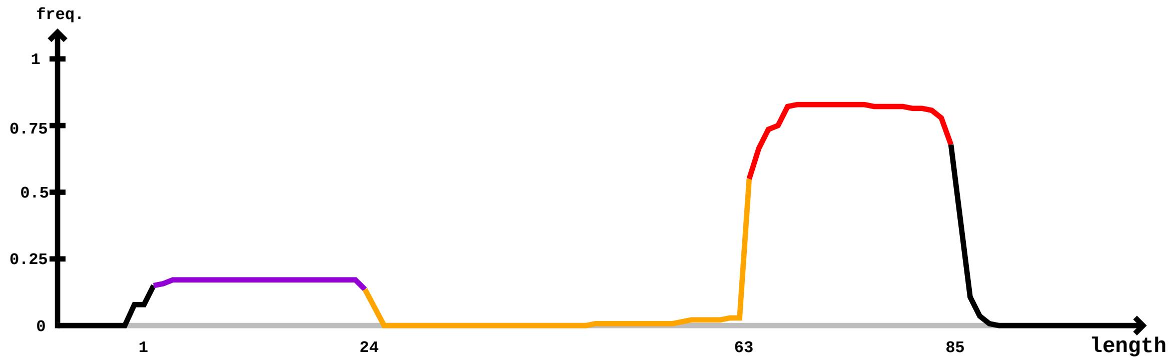

Star **Mature**

```
5'- ggaacugugaaguuaaccguggccuaguggguauagcacaacaugauugucacaacccguguucgaagaccauuacgcucuguuugacuuuuuuuuguguuuuuucauua   -3'    obs
    ggaacugugaaguuaaccguggccuaguggguauagcacaacaugauugucacaacccguguucgaagaccauuacgcucuguuugacuuuuuuuuguguuuuuucauua          exp
    (((((...((((((((.((.(((.((((((((...(...((((((...........)))))))...)))))))).))).)).))))))))........))))).......   reads    mm    sample
    ......gugaaguuaaccguggccuagugg................................................................................   3        0     S03
    ........gaaguuaaccguggccuaguggu...............................................................................   4        0     S03
    ........gaaguuaaccguggccuaguggC...............................................................................   1        1     S03
    ........gaaguuaaccguggccuagGggu...............................................................................   1        1     S03
    .........aaguuaaccguggccuaguggu...............................................................................   1        0     S03
    ......................................................................cauuacgcucugCuugacuu....................   1        1     S03
    ......................................................................cauuacgcucuguuugacuC....................   1        1     S03
    ......................................................................cauuacgcucuguuugacuuu...................   6        0     S03
    ......................................................................cauuacgcucuguuugacuuG...................   1        1     S03
    ......................................................................cauuacCcucuguuugacuuu...................   1        1     S03
    ......................................................................cauuacgcucuguuugacuuuu..................   12       0     S03
    ......................................................................cauuacgcucuguuugacuuuG..................   1        1     S03
    ......................................................................cUuuacgcucuguuugacuuuu..................   1        1     S03
    ......................................................................cauuacgcucuguuugacuuuA..................   1        1     S03
    ......................................................................cauuacgcucugCuugacuuuu..................   1        1     S03
    ......................................................................cauuacgcucuguuugacuuuuA.................   2        1     S03
    ......................................................................cauuacgcucuguuugacuuuuC.................   2        1     S03
    ......................................................................cauuacgcucuguuugacuuuuu.................   3        0     S03
    .......................................................................auuacgcucuguuugacuuuC..................   1        1     S03
    .......................................................................auuacgcucuguuugacuuuuA.................   1        1     S03
    .......................................................................auuacgcucuguuugacuuuuu.................   1        0     S03
    .......................................................................auuacgcucuguuugacuuuuuA................   1        1     S03
    .......................................................................auuacgcucuguuugacuuuuuuu...............   1        0     S03
    ........................................................................uuacgcucuguuugacuuuu..................   1        0     S03
    ........................................................................uuacgcucuguuugacuuuuu.................   1        0     S03
    ........................................................................uuacgcucuguuugacuuuuAuuu..............   1        1     S03
    ..........................................................................acgcucuguuugacuuuu..................   2        0     S03
    ..........................................................................acgcucuguuugacuuuuu.................   2        0     S03
    ..........................................................................acgcucuguuugacuuuuuA................   1        1     S03
    ..........................................................................acgcucuguuugacuuuuuuu...............   1        0     S03
    ...........................................................................cgcucuguuugacuuuuuuu...............   1        0     S03

    ......gugaaguuaaccguggccuagugg................................................................................   2        0     S04
```

```
        Star                                                    Mature
ggaacugugaaguuaaccguggccuagugguuauagcacaacaugauugucacaacccguguucgaagaccauuacgcucuguuugacuuuuuuuuguguuuuuucauua
......gugaaguuaaccguggccuaguggA...............................................................................   1    1   S04
......gugaaguuaaccguggccuaguggG...............................................................................   1    1   S04
......gugaaguuaaccguggccuaguggu...............................................................................   3    0   S04
......gugaaguuaaccguggccuagugguA..............................................................................   1    1   S04
........gaaguuaaccguggccuaguggA...............................................................................   1    1   S04
........gaaguuaacUguggccuaguggu...............................................................................   1    1   S04
........gaaguuaaccguggccuaguggu...............................................................................   2    0   S04
..........aguuaaccguggccuaguggA...............................................................................   1    1   S04
..........aguuaaccguggccuaguggu...............................................................................   1    0   S04
......................................................acccguguucgaagaccauuacgcucugu...........................   1    0   S04
...............................................................cgaagaccauuacgcucuguuuga.......................   1    0   S04
................................................................gaagaccauuacgcucuguuugacuuuu..................   1    0   S04
....................................................................accauuacgcucuguuugacuuu...................   1    0   S04
......................................................................cauuacgcucuguuugacC.....................   1    1   S04
......................................................................cauuacgcucuguuugacuu....................   2    0   S04
......................................................................cGuuacgcucuguuugacuuu...................   1    1   S04
......................................................................cauuacgcucuguuugacuuu...................   2    0   S04
......................................................................cauuacgcucuguuugacuuC...................   1    1   S04
......................................................................cauuacgcucuguuugacuuuu..................  14    0   S04
......................................................................cauuacgcucuguuugacuuuA..................   1    1   S04
......................................................................cauuacgcAcuguuugacuuuu..................   1    1   S04
......................................................................cauuacgcucuguuugacCuuu..................   1    1   S04
......................................................................cauuacgcucuguuugacuuuG..................   1    1   S04
......................................................................cauuacgcucuguuugacuuuGu.................   1    1   S04
......................................................................cauuacgcucuguuugacuuuuA.................   3    1   S04
......................................................................cauuacgcucuguuugacuuuuu.................   8    0   S04
......................................................................cauuacgcucuguuugacuuuuC.................   2    1   S04
......................................................................cauuacgcucuguuCgacuuuuu.................   1    1   S04
.......................................................................auuacgcucuguuugacuuuu..................   4    0   S04
.......................................................................auuacgcucuguuugacuuuuA.................   1    1   S04
.......................................................................auuacgcucuguuugacuuuuu.................   1    0   S04
.......................................................................auuacgcucuguuugacuuuuuG................   1    1   S04
.......................................................................auuacgcucuguuugacuuuuuu................   3    0   S04
.......................................................................auuacgcucuguuugacuuuuuA................   1    1   S04
........................................................................uuacgcucuguuugacuuu...................   1    0   S04
........................................................................uuacgcucuguuugacuuuu..................   2    0   S04
........................................................................uuacgcucuguuugacuuuA..................   2    1   S04
........................................................................uuacgcucuguuugacuuuGu.................   1    1   S04
........................................................................uuacgcucuguuugacuuuuuC................   1    1   S04
.........................................................................uacgcucuguuugacuuuuuu................   2    0   S04
..........................................................................acgcucuguuugacuuuu..................   1    0   S04
..........................................................................acgcucuguuugacuuuuu.................   2    0   S04
..........................................................................acgcucuguuugacuuuuuuC...............   1    1   S04
```

Provisional ID	:	chrC02_1150845
Score total	:	0.1
Score for star read(s)	:	-1.3
Score for read counts	:	0
Score for mfe	:	1.4
Score for randfold	:	0
Score for cons. seed	:	
Total read count	:	18
Mature read count	:	18
Loop read count	:	0
Star read count	:	0

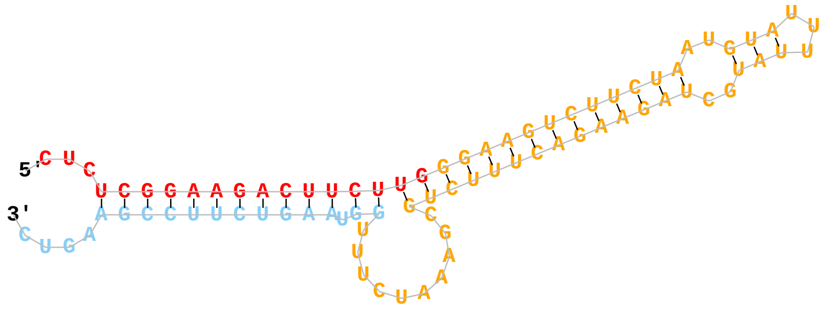

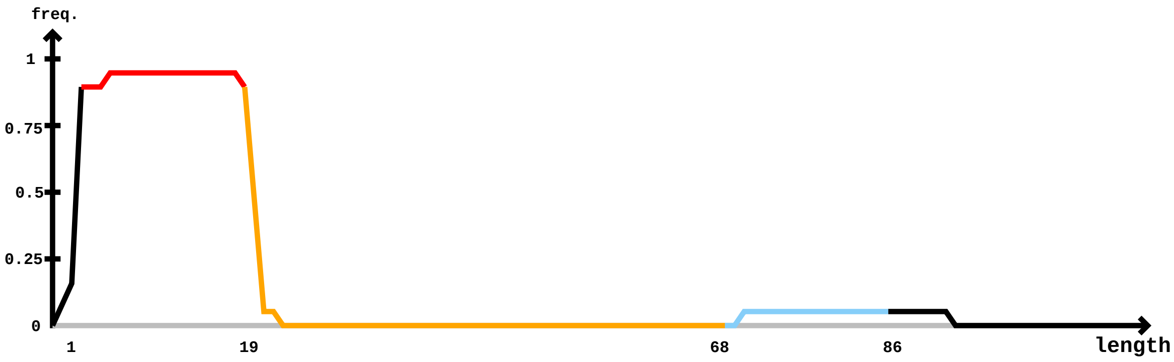

Mature **Star**

```
5'- acucucggaagacuucuugggaagucuucuaauguauuuuaugcuagaagacuuucugcgaaaucuuugguaagucuuccgaagucuucugcccaaggugguacaaacgaau   -3'    exp
    (((.(((((((((((((((((((((((((..(((...)))..))))))))))))))))..........)).)))))))))))))))...(..((...))..)...........   reads      mm         sample
    acucucggaagacuucuu..............................................................................................   1          0          S03
    acucucggaagacuucuug.............................................................................................   1          0          S03
    .cucucggaagacuucuug.............................................................................................   7          0          S03
    ....ucggaGgacuucuuggga..........................................................................................   1          1          S03

    acucucggaagacuucuuCg............................................................................................   1          1          S04
    .cucucggaagacuucuug.............................................................................................   7          0          S04
    ......................................................................uaagucuuccgaagucuucugc....................   1          0          S04
```

Provisional ID : chrC02_1153123
Score total : 0.4
Score for star read(s) : -1.3
Score for read counts : 0
Score for mfe : 1.7
Score for randfold : 0
Score for cons. seed :
Total read count : 169
Mature read count : 168
Loop read count : 1
Star read count : 0

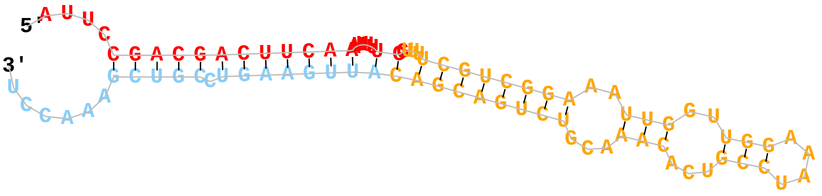

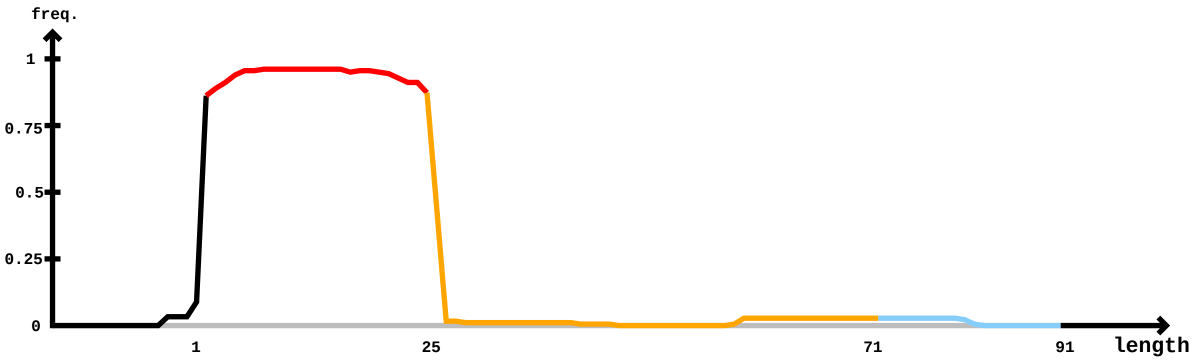

Mature Star

```
5'- aaauggccggcggaauuccgacgacuucaaauuuuugguuuucgucggaaauugguuggaaauccgucacaaacgucugacgacauugaaguccgucgaaaccuccgucgaaau   -3'   exp
    .......(((((((.((.((((((((((((.....((....((((((((..(((..(((....)))...)))...))))))))))))))))).))))).))..)))))))....   reads   mm   sample
    ..........cggaauuccgacgacuuca.....................................................................................   1   0   S03
    ..........cggaauuccgacgacuucaaauu.................................................................................   1   0   S03
    ..........cggaauuccgacgacuucaaauuu................................................................................   2   0   S03
    .............aauuccgacgacuucaaauuu................................................................................   1   0   S03
    .............aauuccgacgacuucaaauuuu...............................................................................   1   0   S03
    .............aauuccgacgacuucaaauuuuuC.............................................................................   1   1   S03
    .............aauuccgacgacuucaaauuuuug.............................................................................   1   0   S03
    ..............auuccgacgacuucaaauuuu...............................................................................   2   0   S03
    ..............auuccgacgacuucaaauuuuGg.............................................................................   1   1   S03
    ..............Guuccgacgacuucaaauuuuugg............................................................................   1   1   S03
    ..............auuccgacgacuucaaauuuCugg............................................................................   1   1   S03
    ..............auuccgacgacuucaaauuuuugA............................................................................   6   1   S03
    ..............auuccgacgCcuucaaauuuuugg............................................................................   1   1   S03
    ..............aCuccgacgacuucaaauuuuugg............................................................................   1   1   S03
    ..............auuccgacgaUuucaaauuuuugg............................................................................   1   1   S03
    ..............auuccgacgacuucaaauuuuugg............................................................................   51   0   S03
    ...............uuccgacgacuucaaauuuuugg............................................................................   1   0   S03
    .................ccgacgacuucaaauuuuugg............................................................................   1   0   S03
    ..............................auuuuugguuuucgucggaaauu.............................................................   1   0   S03
    .....................................guuuucgucggaaauCgguu.........................................................   1   1   S03
    ......................................................................aaacgucugacgacauugaagucc....................   2   0   S03

    ..........cggaauuccgacgacuuca.....................................................................................   1   0   S04
    ..........cggaauuccgacgacuucaaau..................................................................................   1   0   S04
    .............aauuccgacgacuucaaauuuuug.............................................................................   3   0   S04
    .............aauuccgacgacuucaaauCuuugg............................................................................   1   1   S04
    .............aauuccgacgacuucaaauuuuugg............................................................................   2   0   S04
    ..............auuccgacgacuucaaauuuuuA.............................................................................   1   1   S04
    ..............auGccgacgacuucaaauuuuugg............................................................................   1   1   S04
    ..............auuccgacgacuucaaauCuuugg............................................................................   1   1   S04
    ..............auuccgacgacuucaaauuuuugU............................................................................   1   1   S04
    ..............auuccgacgacuucaaauuuuugg............................................................................   57   0   S04
    ..............auuccgacgacuucaaauuuuugA............................................................................   10   1   S04
    ..............auuccgacgGcuucaaauuuuugg............................................................................   1   1   S04
```

```
              Mature                                                        Star
aaauggccggcggaauuccgacgacuucaaauuuuugguuuucgucggaaaauugguuggaaauccgucacaaacgucugacgacauugaaguccgucgaaaccuccgucgaaau
..............auuccgacgacuucaaGuuuuugg.............................................................................     1     1     S04
..............auuccgacgUcuucaaauuuuugg.............................................................................     1     1     S04
..............auCccgacgacuucaaauuuuugg.............................................................................     1     1     S04
...............uuccgacgacuucaaauuuuuA..............................................................................     1     1     S04
...............uuccgacgacuucaaauuuuugg.............................................................................     2     0     S04
...............uuccgacgacuucaaauuuuugA.............................................................................     1     1     S04
................uccgacgacuucaaauuuuugg.............................................................................     3     0     S04
................uccgacgacuucaaauuuuugA.............................................................................     1     1     S04
.................ccgacgacuucaaauuuuugg.............................................................................     3     0     S04
.................ccgacgacuucaaauuuuuggGuu..........................................................................     1     1     S04
..................cgacgacuucaaauuuuugg.............................................................................     3     0     S04
....................acgacuucaaauuuuugg.............................................................................     1     0     S04
......................................................................caaacgucugacgacauugaaguc.....................     1     0     S04
.......................................................................aaacgucugacgacauugaagucc....................     1     0     S04
.......................................................................aaacgucugacgacauugaaguccU...................     1     1     S04
```

Provisional ID : chrC02_1155231
Score total : 31.7
Score for star read(s) : 3.9
Score for read counts : 29.7
Score for mfe : -1.8
Score for randfold : 0
Score for cons. seed :
Total read count : 70
Mature read count : 69
Loop read count : 0
Star read count : 1

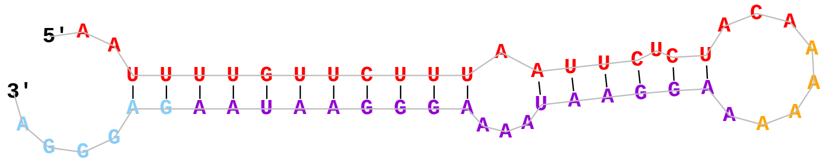

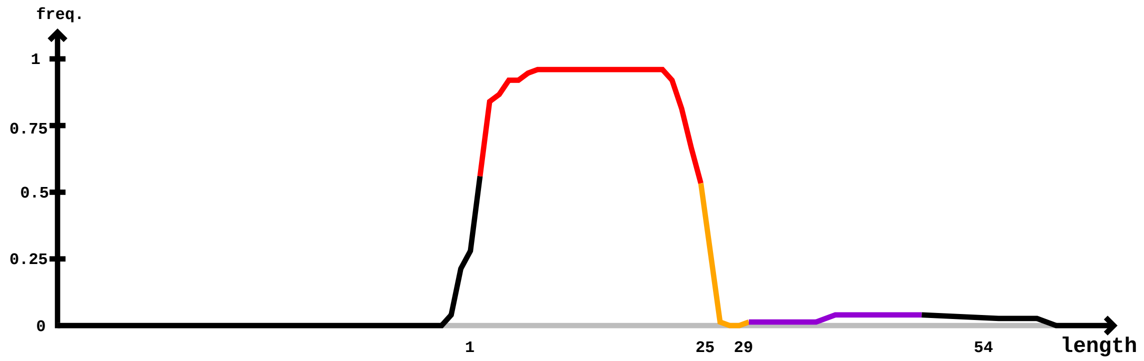


```
                                     Mature                    Star
5'- augaaaaggaaugguuguuccuuaucaaauuuaauaagaaauaauuuuguucuuuaauucucuacaaaaaaaggaauaaaagggaauaagagggaauuauuauuccuu  -3'   obs
    augaaaaggaaugguuguuccuuaucaaauuuaauaagaaauaauuuuguucuuuaauucucuacaaaaaaggaauaaaagggaauaagagggaauuauuauuccuu          exp
    .....(((((((((.(((((.......((((......))))..((((((((((.((((.((........))))))...)))))))))).))))).)))))))))  reads  mm  sample
    .......................................aauaauGuuguucuuuaauucucu.............................................  3  1  S03
    ........................................auaauGuuguucuuuaauucuc..............................................  3  1  S03
    ........................................auaauGuuguucuuuaauucucu.............................................  2  1  S03
    ........................................auaauGuuguucuuuaauucucua............................................  7  1  S03
    .........................................uaauGuuguucuuuaauucucua............................................  1  1  S03
    .........................................uaauGuuguucuuuaauucucuac...........................................  4  1  S03
    ..........................................aauGuuguucuuuaauucucu.............................................  1  1  S03
    ..........................................aauGuuguucuuuaauucucua............................................  1  1  S03
    ..........................................aauGuuguucuuuaauucucuac...........................................  1  1  S03
    ..........................................aauGuuguucuuuaauucucuaca..........................................  16  1  S03
    ...........................................auGuuguucuuuaauucucua............................................  1  1  S03
    ...........................................auGuuguucuuuaauucucuac...........................................  3  1  S03
    ...........................................auGuuguucuuuaauucucuaca..........................................  14  1  S03
    ...........................................auuuuguucuuuaauucucuaca..........................................  1  0  S03
    ............................................uGuuguucuuuaauucucu.............................................  1  1  S03
    ............................................uuuuguucuuuaauucucuaca..........................................  1  0  S03
    .............................................Guuguucuuuaauucucu.............................................  1  1  S03
    .............................................Guuguucuuuaauucucuac...........................................  1  1  S03
    .............................................Guuguucuuuaauucucuaca..........................................  2  1  S03
    ................................................guucuuuaauucucuacaaa........................................  1  0  S03
    ..............................................................................aaagggaauaagagggaauuau........  1  0  S03
    ...............................................................................aagggaauaagagggaauuauua......  1  0  S03

    ........................................auaauGuuguucuuuaauucucua............................................  1  1  S04
    ..........................................aauuuuguucuuuaauucucuaca..........................................  2  0  S04
    ...........................................auuuuguucuuuaauucucuac...........................................  1  0  S04
    ...........................................auGuuguucuuuaauucucuaca..........................................  1  1  S04
    ...............................................uguucuuuaauucucuaca..........................................  2  0  S04
    ......................................................................aaggaauaaaagggaauaG...................  1  1  S04
```

Provisional ID	:	chrC02_1156554
Score total	:	0.3
Score for star read(s)	:	-1.3
Score for read counts	:	0
Score for mfe	:	1.6
Score for randfold	:	0
Score for cons. seed	:	
Total read count	:	146
Mature read count	:	145
Loop read count	:	0
Star read count	:	1

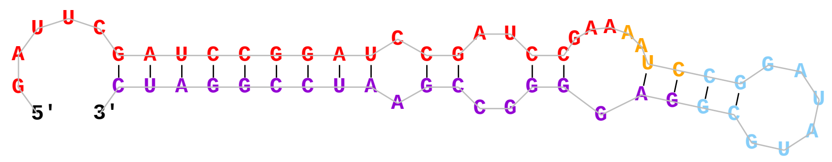

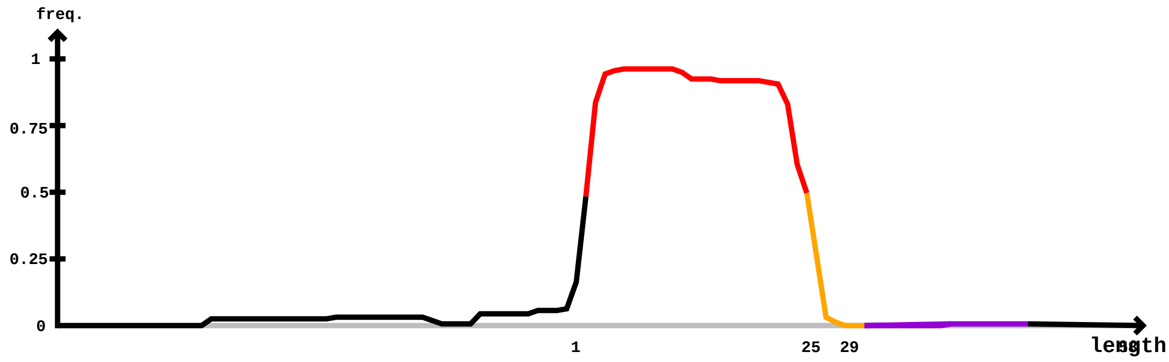

Mature **Star**

```
5'- aaaauuaaguagcaaggccugaaacuuuuauccgagaucuggauucgauccgagauucgauccggauccgauccgaaaauccggauaugcggaggggccgaauccggaucc -3'   obs
    aaaauuaaguagcaaggccugaaacuuuuauccgagaucuggauucgauccgagauucgauccggauccgauccgaaaauccggauaugcggaggggccgaauccggaucc        exp
    ......((((..((.....))..))))..(((((.....)))))..((((((.((((((..((...(((((((((......)))))...)))).))..)))))))))))).   reads   mm   sample
    ..............aggccugaaacuuuuauccgagau..........................................................................   1       0    S03
    ..............aggccugaaacuuAuauccgagau..........................................................................   1       1    S03
    ..........................................auucgauccgagauucgaucc.................................................   1       0    S03
    ..........................................auucgauccgagauucgauccg................................................   2       0    S03
    ................................................uccgagauucgauccggauccgauc.......................................   1       0    S03
    ...................................................gagauucgauccggauccgaucc......................................   1       0    S03
    ...................................................gagauucgauccggauccgauccg.....................................   1       0    S03
    ....................................................agauucgauccggauccgaucc......................................   1       0    S03
    ....................................................agauucgauccggauccgauccg.....................................   7       0    S03
    ....................................................agauucgaAccggauccgauccg.....................................   1       1    S03
    ....................................................agauucgauccggauccgauccgG....................................   1       1    S03
    ....................................................agauucgauccggauccgauccga....................................   3       0    S03
    ....................................................agauucgauccggauccgauccgaa...................................   1       0    S03
    .....................................................gauucgauccggauccgaucc......................................   4       0    S03
    .....................................................gauucgauccggauccgauccg.....................................   4       0    S03
    .....................................................gauucgauccggauccgauccC.....................................   1       1    S03
    .....................................................gauucgauccggauccgauccga....................................   5       0    S03
    .....................................................gauucgauccggauccgauccgaa...................................   30      0    S03
    .....................................................gGuucgauccggauccgauccgaa...................................   1       1    S03
    .....................................................gauucgauccggauccgauccgaaa..................................   1       0    S03
    ......................................................auucgauccggauccgau........................................   1       0    S03
    ......................................................auucgauccggauccgaucc......................................   4       0    S03
    ......................................................auucgauccggauccgauccg.....................................   14      0    S03
    ......................................................auucgauccggauccgauccgU....................................   1       1    S03
    ......................................................auucgauccggauccgauccga....................................   2       0    S03
    ......................................................auucgauccggauccgauccgaa...................................   4       0    S03
    ......................................................auucgauccggauccgauccgaaa..................................   20      0    S03
    ......................................................auucgauccggauccgauccgaaC..................................   1       1    S03
    ......................................................auucgauccggauccgGuccgaaa..................................   1       1    S03
    ......................................................auucgauccggauccgauccgaGa..................................   1       1    S03
    .......................................................uucgauccggauccgaucc......................................   2       0    S03
    .......................................................uucgauccggauccgauccC.....................................   1       1    S03
    .......................................................uucgauccggauccgauccg.....................................   2       0    S03
```

```
                                                     Mature                                Star
aaaauuaaguagcaaggccugaaacuuuuauccgagaucuggauucgauccgagauucgauccggauccgauccgaaaauccggauaugcggaggggccgaauccggaucc
.......................................................uucgauccggaCccgauccg....................................   1   1   S03
.......................................................uucgauccggauccgauccgC...................................   1   1   S03
.......................................................uucgauccggauccgauccga...................................   1   0   S03
.......................................................uucgauccggauccgauccgaa..................................   2   0   S03
.......................................................uucgauccggauccgauccgaaa.................................   2   0   S03
.......................................................uucgauccggauccgauccgaaaa................................   3   0   S03
........................................................ucgauccggauccgauccgaaaau...............................   2   0   S03

..............aggccugaaacuuuuauccgaga..........................................................................   2   0   S04
...........................uuauccgagaucuggauucgaucc............................................................   1   0   S04
..........................................auucgauccgagauucgaucc................................................   1   0   S04
..........................................auucgauccgagauucgauccg...............................................   2   0   S04
................................................uccgagauucgauccgAau............................................   1   1   S04
....................................................agauucgauccggauccgauccg....................................   1   0   S04
....................................................agauucgauccggauccgauccga...................................   1   0   S04
.....................................................gauucgauccggauccgauccgC...................................   1   1   S04
.....................................................gauucgauccggauccgauccgaa..................................   4   0   S04
......................................................auucgauccggauccgauccg....................................   1   0   S04
......................................................auucgauccggauccgauccga...................................   1   0   S04
......................................................auucgauccggauccgauccgaaa.................................   5   0   S04
.......................................................uucgauccggauccgauccg....................................   2   0   S04
.........................................................cgauccggauccgauccgaaC.................................   1   1   S04
...........................................................................................gaggggccgaauccggauc.   1   0   S04
```

Provisional ID : chrC02_1179245
Score total : 0.2
Score for star read(s) : -1.3
Score for read counts : 0
Score for mfe : 1.5
Score for randfold : 0
Score for cons. seed :
Total read count : 21
Mature read count : 21
Loop read count : 0
Star read count : 0

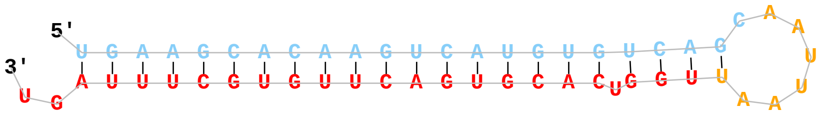

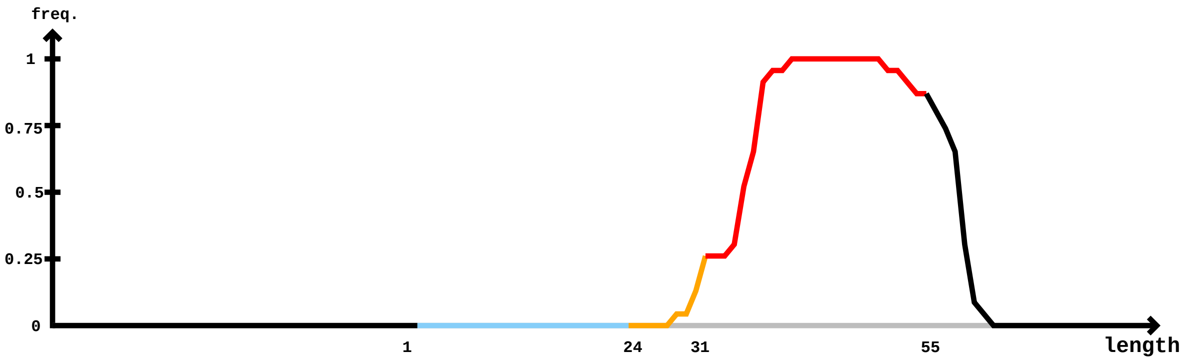

Star Mature

```
5'- uaaaaauacauuuuaauuuuguuuuuaaaaauauaaugaagcacaagucaugugucagcaauuaauuggucacgugacuugugcuuuaguauagaagagacuacaaaauagau -3'   exp
    ...............((((((..........((((.((((((((((((((((((((((.......)))).)))))))))))))))))).)))).........))))))....   reads   mm   sample
    .....................................................................ucacgugacuugugcuuuaguaua....................   1       0    S03
    ......................................................................cacgugacuugugcuuuaguaua....................   1       0    S03
    ......................................................................cacgugacuuguAcuuuaguaua....................   1       1    S03
    ......................................................................cacgugacuugugcuuuaguauU....................   1       1    S03
    .......................................................................acgugacuugugcuuuaguauaAaa.................   1       1    S03
    ........................................................................Agugacuugugcuuuaguauag...................   1       1    S03
    .........................................................................gugacuuguAcuuuaguauaga..................   1       1    S03

    ...............................................................aauuggucacgugacuugugcuuu..........................   1       0    S04
    .................................................................uuggucacgugacuugugcA............................   1       1    S04
    .................................................................uuggucacgugacuugugcuuua.........................   1       0    S04
    ..................................................................uggucacgugacuugugcuuuagu.......................   2       0    S04
    ..................................................................uggucacgugacuCgugcuuuagu.......................   1       1    S04
    ......................................................................cacgugacuugugcuuuaguaua....................   2       0    S04
    .......................................................................acgugacuugugcuuuaguaua....................   1       0    S04
    .......................................................................aAgugacuugugcuuuaguauag...................   1       1    S04
    ........................................................................cgugacuugugcCuuaguau.....................   1       1    S04
    ........................................................................cgugacuugugcuuuaguau.....................   1       0    S04
    ........................................................................Agugacuugugcuuuaguauag...................   2       1    S04
    ........................................................................Ugugacuugugcuuuaguauag...................   1       1    S04
    ...........................................................................gacuugugcuuuaguaua....................   1       0    S04
```

Provisional ID	:	chrC02_1190245
Score total	:	3.6
Score for star read(s)	:	3.9
Score for read counts	:	8.8
Score for mfe	:	-9
Score for randfold	:	0
Score for cons. seed	:	
Total read count	:	29
Mature read count	:	21
Loop read count	:	0
Star read count	:	8

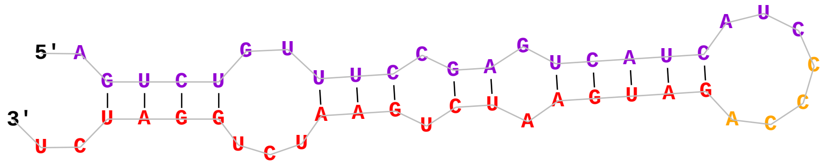

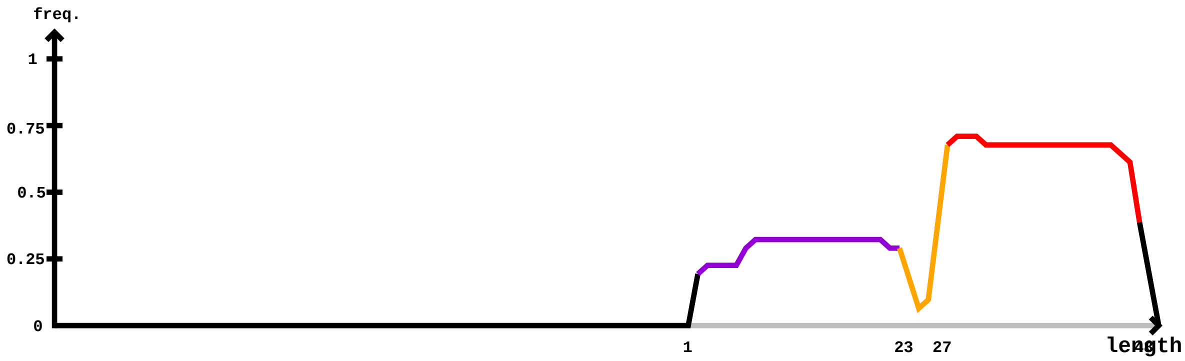


```
                                                                      Star                  Mature
5'- aaaaauaguuuuaaugauaauuaaaauaguuaaauaauauaguaauuauauuauauauauauauaagucuguuuccgagucaucaucccccagaugaaucugaaucuggaucuu  -3'   obs
    aaaaauaguuuuaaugauaauuaaaauaguuaaauaauauaguaauuauauuauauauauauauaagucuguuuccgagucaucaucccccagaugaaucugaaucuggaucuu        exp
    ...(((.((((((((....)))))))).))).....((((((((....))))))))..........((((..(((.((.(((((.......))))).)).)))...))))...   reads    mm    sample
    .................................................................agucuguuuccgagucaucauU..........................   2        1     S03
    ......................................................................guuuccgaguUaucaucc.........................   1        1     S03
    .........................................................................................cagaGgaaucugaaucuggaucuu   1        1     S03
    ..........................................................................................agaugaaucugaaucugga....   1        0     S03
    ..........................................................................................agaugaaucugaaucuggauc..   1        0     S03
    ..........................................................................................agaugaaucugaaucuggaucA.   1        1     S03
    ..........................................................................................agaugaaucugaaucuggaucu.   2        0     S03
    ..........................................................................................agaugaaucugaaucuggaucuA   1        1     S03
    ...........................................................................................gaugaaucugaaucuggauc..   2        0     S03
    ...........................................................................................gaugaaucugaaucuggaucu.   2        0     S03

    .................................................................agucuguuuccgagucaucauc..........................   4        0     S04
    ..................................................................gucuguuuccgagucauca............................   1        0     S04
    ......................................................................guuuccgagucaucauccccaga....................   1        0     S04
    .......................................................................uuuccgagucaucaucUccagaug..................   1        1     S04
    ..........................................................................................agaugaaucugGaucuggau...   1        1     S04
    ..........................................................................................agaugaaucugaaucuggauc..   1        0     S04
    ..........................................................................................agaugaaucugaaucuggaucu.   1        0     S04
    ...........................................................................................gaugaaucugaaucuggauc..   1        0     S04
    ...........................................................................................gaugaaucugaaucuggaucu.   4        0     S04
    ............................................................................................augaaucugaaucuggauc..   1        0     S04
    .............................................................................................ugaaucugaaucuggauc..   1        0     S04
```

Provisional ID : chrC02_1199259
Score total : 1.3
Score for star read(s) : -1.3
Score for read counts : 0
Score for mfe : 2.6
Score for randfold : 0
Score for cons. seed :
Total read count : 516
Mature read count : 425
Loop read count : 87
Star read count : 4

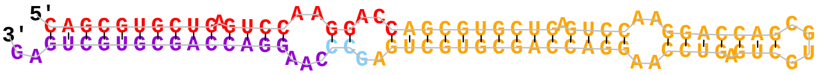

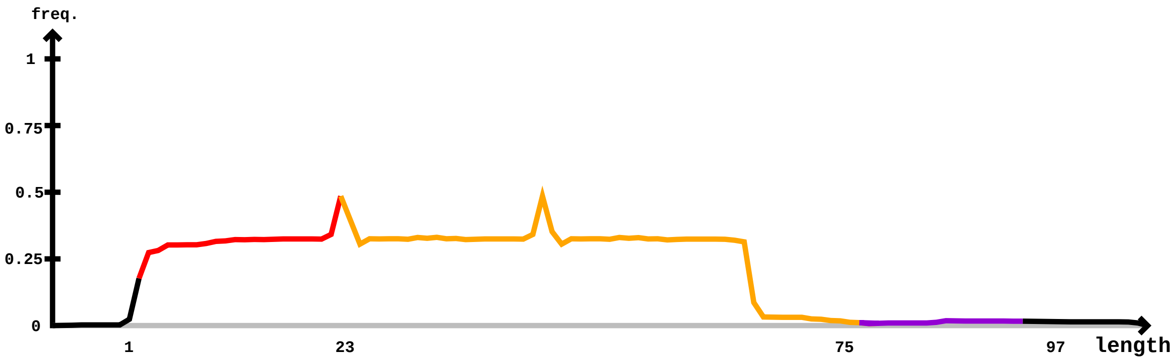

Mature Star

```
5'- caaggaccagcgugcugaguccaaggaccagcgugcugaguccaaggaccagcgugcugaguccaaggaccagcgugcugagcccaaggaccagcgugcugaguccaaggac  -3'   obs
    caaggaccagcgugcugaguccaaggaccagcgugcugaguccaaggaccagcgugcugaguccaaggaccagcgugcugagcccaaggaccagcgugcugaguccaaggac        exp
    ...((((((((((((((.((((..((..((((((((((.((((..(((((((....))).))))..))))))))))))))..))...))))))))))))))).))))......   reads   mm   sample
    .aaggaccagcgugcugag.............................................................................................   5       0    S03
    ......ccagcgugcugaguccaaggC.....................................................................................   4       1    S03
    ......ccagcgugcugaguccaaggacc...................................................................................   3       0    S03
    ......ccagcgugcugaguccaaggacca..................................................................................   3       0    S03
    ......ccagcgugcugaguccaGggacca..................................................................................   3       1    S03
    .......cagcgugcugaguccaaggac....................................................................................   8       0    S03
    .......cUgcgugcugaguccaaggacc...................................................................................   3       1    S03
    .......cagcgugcugaguccaaggacc...................................................................................   102     0    S03
    ........agcgugcugaguccaaggac....................................................................................   4       0    S03
    ........agcgugcugaguccaaggacc...................................................................................   42      0    S03
    ........agcgugcugagucNaaggacc...................................................................................   3       1    S03
    ........agcgugcugaguccaaggaccU..................................................................................   3       1    S03
    ........agcgugcugaguccaaggacca..................................................................................   21      0    S03
    .........gcgugcugaguccaaggacc...................................................................................   6       0    S03
    .........gcgugcugaguccaaggacca..................................................................................   3       0    S03
    ..........cgugcugaguccaaggacc...................................................................................   9       0    S03
    ..........cgugcugaguccaaggacca..................................................................................   6       0    S03
    ..............cugaguccaaggaccagcgug.............................................................................   3       0    S03
    ..............cugaUuccaaggaccagcgug.............................................................................   3       1    S03
    ..............cugaguccaaggaccUgcgugc............................................................................   3       1    S03
    ...............ugaguccaaggaccagcgug.............................................................................   9       0    S03
    ......................aaggaccagcgugcugag........................................................................   5       0    S03
    ...........................ccagcgugcugaguccaaggC................................................................   4       1    S03
    ...........................ccagcgugcugaguccaaggacc..............................................................   3       0    S03
    ...........................ccagcgugcugaguccaGggacca.............................................................   3       1    S03
    ...........................ccagcgugcugaguccaaggacca.............................................................   3       0    S03
    ............................cagcgugcugaguccaaggac...............................................................   8       0    S03
    ............................cagcgugcugaguccaaggacc..............................................................   102     0    S03
    ............................cUgcgugcugaguccaaggacc..............................................................   3       1    S03
    .............................agcgugcugaguccaaggac...............................................................   4       0    S03
    .............................agcgugcugagucNaaggacc..............................................................   3       1    S03
    .............................agcgugcugaguccaaggacc..............................................................   42      0    S03
    .............................agcgugcugaguccaaggacca.............................................................   21      0    S03
```

```
Mature                                                                      Star
caaggaccagcgugcugaguccaaggaccagcgugcugaguccaaggaccagcgugcugaguccaaggaccagcgugcugagcccaaggaccagcgugcugaguccaaggac
.............................agcgugcugaguccaaggaccU.............................................................  3    1  S03
..............................gcgugcugaguccaaggacc..............................................................  6    0  S03
..............................gcgugcugaguccaaggacca.............................................................  3    0  S03
...............................cgugcugaguccaaggacc..............................................................  9    0  S03
...............................cgugcugaguccaaggacca.............................................................  6    0  S03
...................................cugaguccaaggaccagcgug........................................................  3    0  S03
...................................cugaUuccaaggaccagcgug........................................................  3    1  S03
...................................cugaguccaaggaccUgcgugc.......................................................  3    1  S03
....................................ugaguccaaggaccagcgug........................................................  9    0  S03
...........................................aaggaccagcgugcugag...................................................  5    0  S03
................................................ccagcgugcugaguccaaggC...........................................  4    1  S03
................................................ccagcgugcugaguccaaggacc.........................................  3    0  S03
................................................ccagcgugcugaguccaGggacca........................................  3    1  S03
................................................ccagcgugcugaguccaaggacca........................................  3    0  S03
.................................................cagcgugcugaguccaaggac..........................................  8    0  S03
.................................................cagcgugcugaguccaaggacc.........................................  102  0  S03
.................................................cUgcgugcugaguccaaggacc.........................................  3    1  S03
..................................................agcgugcugaguccaaggac..........................................  4    0  S03
..................................................agcgugcugagucNaaggacc.........................................  3    1  S03
..................................................agcgugcugaguccaaggacc.........................................  42   0  S03
..................................................agcgugcugaguccaaggacca........................................  21   0  S03
..................................................agcgugcugaguccaaggaccU........................................  3    1  S03
...................................................gcgugcugaguccaaggacc.........................................  6    0  S03
...................................................gcgugcugaguccaaggacca........................................  3    0  S03
....................................................cgugcugaguccaaggacc.........................................  9    0  S03
....................................................cgugcugaguccaaggacca........................................  6    0  S03
........................................................cugaUuccaaggaccagcgug...................................  3    1  S03
........................................................cugaguccaaggaccagcgug...................................  3    0  S03
........................................................cugaguccaaggaccUgcgugc..................................  3    1  S03
.........................................................ugaguccaaggaccagcgug...................................  9    0  S03
................................................................aaggaccagcgugcugag..............................  5    0  S03
.....................................................................................aaggaccagcgugcugag.........  5    0  S03
..........................................................................................ccagcgugcugaguccaaggC.  4    1  S03
...........................................................................................cagcgugcugaguccaaggac  8    0  S03
............................................................................................agcgugcugaguccaaggac  4    0  S03

caaggaccagcgugcugU..............................................................................................  5    1  S04
caaggaccagUgugcugag.............................................................................................  5    1  S04
.aaggaccagcgugcugagA............................................................................................  5    1  S04
......ccagcgugcugaguccaagga.....................................................................................  4    0  S04
......ccagcgugcuAaguccaaggac....................................................................................  4    1  S04
......ccagcgugcugaguccaaggac....................................................................................  4    0  S04
......ccagcgugcugaguccaaggacG...................................................................................  3    1  S04
......ccagcgugcugaguccaaggacA...................................................................................  6    1  S04
......ccagcgugcugaguccaaggacc...................................................................................  51   0  S04
......ccagcgugcugaguccaaggaccC..................................................................................  6    1  S04
......ccagcgugcugaguccaaggaccU..................................................................................  6    1  S04
......ccagcgugcugaguccaaggacca..................................................................................  12   0  S04
.......cagcgugcugaguccaagga.....................................................................................  12   0  S04
.......cagcgugcugaguccaaggac....................................................................................  20   0  S04
.......cagcgugcugaguGcaaggacc...................................................................................  3    1  S04
.......cUgcgugcugaguccaaggacc...................................................................................  6    1  S04
.......cagcgugcugaguccaaggacc...................................................................................  546  0  S04
.......cagcgugcugagAccaaggacc...................................................................................  4    1  S04
.......cagcgugcugaguccaaggacG...................................................................................  9    1  S04
.......cagcgugcugaguccaaggacca..................................................................................  36   0  S04
.......cagcgugcugaguccaaggaccU..................................................................................  18   1  S04
........agcgugcugaguccaagg......................................................................................  4    0  S04
........agcgugcugaguccaagga.....................................................................................  4    0  S04
........agcgugcugaguccaaggac....................................................................................  16   0  S04
........agcgugcugaguccaaggacG...................................................................................  3    1  S04
........agcgugcugaguccaaggacc...................................................................................  255  0  S04
........agcgugcugaguccaaggaccU..................................................................................  24   1  S04
........agcgugcugaguccaaggacca..................................................................................  105  0  S04
.........gcgugcugaguccaaggac....................................................................................  4    0  S04
.........gcgugcugaguccaaggacc...................................................................................  24   0  S04
.........gcgugcugaguccaaggacca..................................................................................  3    0  S04
..........cgugcugaguccaaggac....................................................................................  4    0  S04
..........cgugcugaguccaaggacc...................................................................................  54   0  S04
..........cgugcugaguccaaggacG...................................................................................  3    1  S04
```

Mature Star

```
caaggaccagcgugcugaguccaaggaccagcgugcugaguccaaggaccagcgugcugaguccaaggaccagcgugcugagcccaaggaccagcgugcugaguccaaggac
..........cgugcugaguccaaggaccU..................................................................................  6    1  S04
..........cgugcugaguccaaggacca..................................................................................  15   0  S04
..........cgugcugaUuccaaggaccag.................................................................................  3    1  S04
..........cgugcugaguccaaggaccagc................................................................................  3    0  S04
............ugGugaguccaaggaccagcgug.............................................................................  3    1  S04
..............cugaguccaaggaccagcgug.............................................................................  6    0  S04
..............cugaguccaagUaccagcgug.............................................................................  3    1  S04
..............cuAaguccaaggaccagcgugc............................................................................  3    1  S04
..............cugaguccaaggaccagcgugcu...........................................................................  3    0  S04
...............ugaguccaaggaccagcgug.............................................................................  3    0  S04
...............ugaguccaaggaccagcgugcC...........................................................................  3    1  S04
...............ugaguccaaggaccagcgugcu...........................................................................  12   0  S04
...............ugaguccaaggaccagcgugcuC..........................................................................  3    1  S04
...............ugaguccaaggaccagcgugcuga.........................................................................  9    0  S04
................gaguccaaggaccagcgug.............................................................................  3    0  S04
................gaguccaaggaccagcgugcG...........................................................................  3    1  S04
................gaguccaaggaccagcgugcu...........................................................................  3    0  S04
.................aguccaaggaccagcgugcug..........................................................................  3    0  S04
.................aguccaaggaccagcgugcugU.........................................................................  3    1  S04
.................aguccaaggaccagcgugcuga.........................................................................  12   0  S04
.................aguccaaggacUagcgugcugagu.......................................................................  2    1  S04
.................aguccaaggaccagcgugcugagu.......................................................................  2    0  S04
...................uccaaggaccagcgugcugagu.......................................................................  8    0  S04
.....................caaggaccagcgugcugU.........................................................................  5    1  S04
.....................caaggaccagUgugcugag........................................................................  5    1  S04
......................aaggaccagcgugcugagA.......................................................................  5    1  S04
...........................ccagcgugcugaguccaagga................................................................  4    0  S04
...........................ccagcgugcugaguccaaggac...............................................................  4    0  S04
...........................ccagcgugcuAaguccaaggac...............................................................  4    1  S04
...........................ccagcgugcugaguccaaggacG..............................................................  3    1  S04
...........................ccagcgugcugaguccaaggacA..............................................................  6    1  S04
...........................ccagcgugcugaguccaaggacc..............................................................  51   0  S04
...........................ccagcgugcugaguccaaggaccC.............................................................  6    1  S04
...........................ccagcgugcugaguccaaggacca.............................................................  12   0  S04
...........................ccagcgugcugaguccaaggaccU.............................................................  6    1  S04
............................cagcgugcugaguccaagga................................................................  12   0  S04
............................cagcgugcugaguccaaggac...............................................................  20   0  S04
............................cagcgugcugaguGcaaggacc..............................................................  3    1  S04
............................cagcgugcugaguccaaggacc..............................................................  546  0  S04
............................cagcgugcugagAccaaggacc..............................................................  4    1  S04
............................cUgcgugcugaguccaaggacc..............................................................  6    1  S04
............................cagcgugcugaguccaaggacG..............................................................  9    1  S04
............................cagcgugcugaguccaaggacca.............................................................  36   0  S04
............................cagcgugcugaguccaaggaccU.............................................................  18   1  S04
.............................agcgugcugaguccaagg.................................................................  4    0  S04
.............................agcgugcugaguccaagga................................................................  4    0  S04
.............................agcgugcugaguccaaggac...............................................................  16   0  S04
.............................agcgugcugaguccaaggacc..............................................................  255  0  S04
.............................agcgugcugaguccaaggacG..............................................................  3    1  S04
.............................agcgugcugaguccaaggacca.............................................................  105  0  S04
.............................agcgugcugaguccaaggaccU.............................................................  24   1  S04
..............................gcgugcugaguccaaggac...............................................................  4    0  S04
..............................gcgugcugaguccaaggacc..............................................................  24   0  S04
..............................gcgugcugaguccaaggacca.............................................................  3    0  S04
...............................cgugcugaguccaaggac...............................................................  4    0  S04
...............................cgugcugaguccaaggacc..............................................................  54   0  S04
...............................cgugcugaguccaaggacG..............................................................  3    1  S04
...............................cgugcugaguccaaggacca.............................................................  15   0  S04
...............................cgugcugaguccaaggaccU.............................................................  6    1  S04
...............................cgugcugaUuccaaggaccag............................................................  3    1  S04
...............................cgugcugaguccaaggaccagc...........................................................  3    0  S04
.................................ugGugaguccaaggaccagcgug........................................................  3    1  S04
...................................cugaguccaagUaccagcgug........................................................  3    1  S04
...................................cugaguccaaggaccagcgug........................................................  6    0  S04
...................................cuAaguccaaggaccagcgugc.......................................................  3    1  S04
...................................cugaguccaaggaccagcgugcu......................................................  3    0  S04
....................................ugaguccaaggaccagcgug........................................................  3    0  S04
....................................ugaguccaaggaccagcgugcC......................................................  3    1  S04
....................................ugaguccaaggaccagcgugcu......................................................  12   0  S04
....................................ugaguccaaggaccagcgugcuC.....................................................  3    1  S04
```

Mature **Star**

```
caaggaccagcgugcugaguccaaggaccagcgugcugaguccaaggaccagcgugcugaguccaaggaccagcgugcugagcccaaggaccagcgugcugaguccaaggac
....................................ugaguccaaggaccagcgugcuga....................................................	9	0	S04
.....................................gaguccaaggaccagcgug........................................................	3	0	S04
.....................................gaguccaaggaccagcgugcG......................................................	3	1	S04
.....................................gaguccaaggaccagcgugcu......................................................	3	0	S04
......................................aguccaaggaccagcgugcug.....................................................	3	0	S04
......................................aguccaaggaccagcgugcuga....................................................	12	0	S04
......................................aguccaaggaccagcgugcugU....................................................	3	1	S04
......................................aguccaaggacUagcgugcugagu..................................................	2	1	S04
......................................aguccaaggaccagcgugcugagu..................................................	2	0	S04
........................................uccaaggaccagcgugcugagu..................................................	8	0	S04
..........................................caaggaccagcgugcugU....................................................	5	1	S04
..........................................caaggaccagUgugcugag...................................................	5	1	S04
...........................................aaggaccagcgugcugagA..................................................	5	1	S04
................................................ccagcgugcugaguccaagga...........................................	4	0	S04
................................................ccagcgugcugaguccaaggac..........................................	4	0	S04
................................................ccagcgugcuAaguccaaggac..........................................	4	1	S04
................................................ccagcgugcugaguccaaggacA.........................................	6	1	S04
................................................ccagcgugcugaguccaaggacc.........................................	51	0	S04
................................................ccagcgugcugaguccaaggacG.........................................	3	1	S04
................................................ccagcgugcugaguccaaggaccC........................................	6	1	S04
................................................ccagcgugcugaguccaaggacca........................................	12	0	S04
................................................ccagcgugcugaguccaaggaccU........................................	6	1	S04
.................................................cagcgugcugaguccaagga...........................................	12	0	S04
.................................................cagcgugcugaguccaaggac..........................................	20	0	S04
.................................................cagcgugcugaguccaaggacG.........................................	9	1	S04
.................................................cUgcgugcugaguccaaggacc.........................................	6	1	S04
.................................................cagcgugcugaguGcaaggacc.........................................	3	1	S04
.................................................cagcgugcugagAccaaggacc.........................................	4	1	S04
.................................................cagcgugcugaguccaaggacc.........................................	546	0	S04
.................................................cagcgugcugaguccaaggacca........................................	36	0	S04
.................................................cagcgugcugaguccaaggaccU........................................	18	1	S04
..................................................agcgugcugaguccaagg............................................	4	0	S04
..................................................agcgugcugaguccaagga...........................................	4	0	S04
..................................................agcgugcugaguccaaggac..........................................	16	0	S04
..................................................agcgugcugaguccaaggacG.........................................	3	1	S04
..................................................agcgugcugaguccaaggacc.........................................	255	0	S04
..................................................agcgugcugaguccaaggacca........................................	105	0	S04
..................................................agcgugcugaguccaaggaccU........................................	24	1	S04
...................................................gcgugcugaguccaaggac..........................................	4	0	S04
...................................................gcgugcugaguccaaggacc.........................................	24	0	S04
...................................................gcgugcugaguccaaggacca........................................	3	0	S04
....................................................cgugcugaguccaaggac..........................................	4	0	S04
....................................................cgugcugaguccaaggacc.........................................	54	0	S04
....................................................cgugcugaguccaaggacG.........................................	3	1	S04
....................................................cgugcugaguccaaggacca........................................	15	0	S04
....................................................cgugcugaguccaaggaccU........................................	6	1	S04
....................................................cgugcugaUuccaaggaccag.......................................	3	1	S04
....................................................cgugcugaguccaaggaccagc......................................	3	0	S04
......................................................ugGugaguccaaggaccagcgug...................................	3	1	S04
........................................................cugaguccaagUaccagcgug...................................	3	1	S04
........................................................cugaguccaaggaccagcgug...................................	6	0	S04
........................................................cuAaguccaaggaccagcgugc..................................	3	1	S04
........................................................cugaguccaaggaccagcgugcu.................................	3	0	S04
.........................................................ugaguccaaggaccagcgug...................................	3	0	S04
.........................................................ugaguccaaggaccagcgugcu.................................	12	0	S04
.........................................................ugaguccaaggaccagcgugcC.................................	3	1	S04
.........................................................ugaguccaaggaccagcgugcuC................................	3	1	S04
.........................................................ugaguccaaggaccagcgugcuga...............................	9	0	S04
..........................................................gaguccaaggaccagcgug...................................	3	0	S04
..........................................................gaguccaaggaccagcgugcG.................................	3	1	S04
..........................................................gaguccaaggaccagcgugcu.................................	3	0	S04
...........................................................aguccaaggaccagcgugcug................................	3	0	S04
...........................................................aguccaaggaccagcgugcuga...............................	12	0	S04
...........................................................aguccaaggaccagcgugcugU...............................	3	1	S04
............................................................guccaaggaccagcgugcugaUc.............................	1	1	S04
.............................................................uccaaggaccagcgugcugaUc.............................	3	1	S04
...............................................................caaggaccagcgugcugU...............................	5	1	S04
...............................................................caaggaccagUgugcugag..............................	5	1	S04
...............................................................caaggaccagcgugcugaUc.............................	1	1	S04
................................................................aaggaccagcgugcugagA.............................	5	1	S04
```

Mature Star

caaggaccagcgugcugaguccaaggaccagcgugcugaguccaaggaccagcgugcugaguccaaggaccagcgugcugagccaaggaccagcgugcugaguccaaggac			
....cagcgugcugagAccaaggacc....................	4	1	S04
....cagcgugcugagcccaaggacc....................	2	0	S04
....caAcgugcugagcccaaggacc....................	1	1	S04
....cagUgugcugagcccaaggacc....................	1	1	S04
....cagcgugcugagcccaaggaccaA..................	1	1	S04
....agcgugUugagcccaaggacc....................	1	1	S04
....agcgugcugagcccaaggacA....................	1	1	S04
....agcgugcugagcccaaggacca...................	2	0	S04
....gUgugcugagcccaaggacc....................	1	1	S04
....cugagcccaaggaccaAcgug..............	1	1	S04
....caaggaccagcgugcugU..........	5	1	S04
....caaggaccagUgugcugag.........	5	1	S04
....aaggaccagcgugcugagA........	5	1	S04
....ccagcgugcugaguccaagga.	4	0	S04
....ccagcgugcuAaguccaaggac	4	1	S04
....ccagcgugcugaguccaaggac	4	0	S04
....cagcgugcugaguccaagga.	12	0	S04
....cagcgugcugaguccaaggac	20	0	S04
....agcgugcugaguccaagg..	4	0	S04
....agcgugcugaguccaagga.	4	0	S04
....agcgugcugaguccaaggac	16	0	S04
....gcgugcugaguccaaggac	4	0	S04
....cgugcugaguccaaggac	4	0	S04

Provisional ID	:	chrC02_1208830
Score total	:	0.5
Score for star read(s)	:	-1.3
Score for read counts	:	0
Score for mfe	:	1.8
Score for randfold	:	0
Score for cons. seed	:	
Total read count	:	156
Mature read count	:	148
Loop read count	:	8
Star read count	:	0

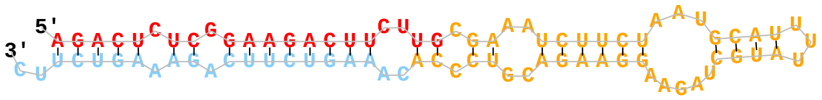

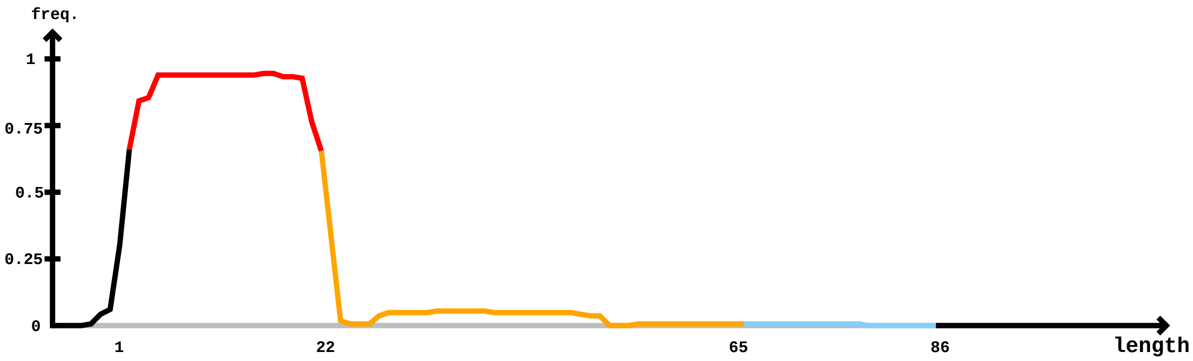

Mature **Star**

```
5'- uuuggaagacucucggaagacuucuugcgaaaucuucuaaugcauuuuaugcuagaaggaagacgucccacaaagucuucagaaagucuuccgaaguuuucucucccaaguggu   -3'    exp
    (((((((((((.((.((((((((..((.((..((((((...((((...)))).....))))))..)).))..)))))))).)).)))))))))))..........((....)).      reads   mm   sample
    ..uggaagacucucggaagacu............................................................................................      1       0    S03
    ...ggGagacucucggaagacuuc..........................................................................................      1       1    S03
    ...ggaagacucucggaagacuucuug.......................................................................................      1       0    S03
    ...gAaagacucucggaagacuucuugc......................................................................................      1       1    S03
    ....gaagacucucggaagacuucuug.......................................................................................      1       0    S03
    ....Aaagacucucggaagacuucuugc......................................................................................      1       1    S03
    .....aagacucucggaagacuucu.........................................................................................      2       0    S03
    .....aagacucucggaagacuucuu........................................................................................      2       0    S03
    .....aagacucGcggaagacuucuug.......................................................................................      1       1    S03
    .....aagacucucggaagacuCcuug.......................................................................................      1       1    S03
    .....aagacucAcggaagacuucuug.......................................................................................      1       1    S03
    .....aagacucucggaagacuucuug.......................................................................................      18      0    S03
    ......agacucucggaagacuucu.........................................................................................      3       0    S03
    ......agacucucggaagacuucuu........................................................................................      4       0    S03
    ......agacucucggaagacuucuug.......................................................................................      30      0    S03
    ......agacucucggaagacuucGug.......................................................................................      1       1    S03
    ......agacucucggaagacuucuugUg.....................................................................................      1       1    S03
    .......gacucucggaagacuucu.........................................................................................      8       0    S03
    .......gacucucggaagacuucuG........................................................................................      1       1    S03
    .......gacucucggaagacuucuu........................................................................................      3       0    S03
    .......gacucucggaagacuucuug.......................................................................................      1       0    S03
    .......gacucucggGagacuucuug.......................................................................................      1       1    S03
    ........acucucggaagacuucuu........................................................................................      1       0    S03
    ........acucucggaagacuucuug.......................................................................................      1       0    S03
    .........cucucggaagacuucuug.......................................................................................      7       0    S03
    ................................ucuucuaaugcauuuuaugcuaga..........................................................      2       0    S03
    .................................cuucuaaugcauuuuaugcuC............................................................      1       1    S03
    ...........................................................aagacgucccacaaagCcuucaga...............................      1       1    S03

    ...ggaagacucucggaagacu............................................................................................      1       0    S04
    ...ggaagacucucggaagacuucuug.......................................................................................      1       0    S04
    ...gAaagacucucggaagacuucuugc......................................................................................      1       1    S04
    ....Aaagacucucggaagacuucuugc......................................................................................      1       1    S04
    .....aagacucucggaagacuucu.........................................................................................      4       0    S04
```

```
      Mature                                                        Star
uuuggaagacucucggaagacuucuugcgaaaucuucuaaugcauuuuaugcuagaaggaagacgucccacaaagucuucagaaagucuuccgaaguuuucucucccaaguggu
.....aagacucucggaagacuucuu.........................................................................................    1    0    S04
.....aagacucucggaagacuucuug........................................................................................    10   0    S04
......agacucucggaagacuucuu.........................................................................................    3    0    S04
......agacucucggaagacuucuug........................................................................................    15   0    S04
......Ggacucucggaagacuucuug........................................................................................    1    1    S04
......agacucucggaagacuucuugcg......................................................................................    1    0    S04
.......gacucucggaagacuucu..........................................................................................    10   0    S04
.......gacucucggaagacuucuu.........................................................................................    3    0    S04
.......gacucucggaagacuucuug........................................................................................    3    0    S04
.........cucucggaagacuucuug........................................................................................    7    0    S04
....................cuucuugcgaaaucuucuaaugca.......................................................................    1    0    S04
................................ucuucuaaugcauuuuaugcuaga...........................................................    3    0    S04
.................................cuucuaaugcauuuuaugcu..............................................................    1    0    S04
......................................aaugcauuuuaugcuaga...........................................................    1    0    S04
```

Provisional ID	:	chrC02_1209721
Score total	:	0.7
Score for star read(s)	:	-1.3
Score for read counts	:	0
Score for mfe	:	2
Score for randfold	:	0
Score for cons. seed	:	
Total read count	:	466
Mature read count	:	443
Loop read count	:	0
Star read count	:	23

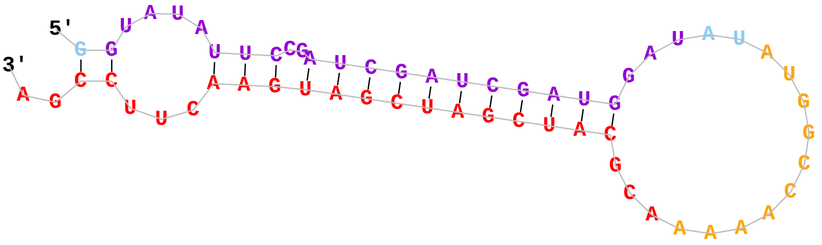

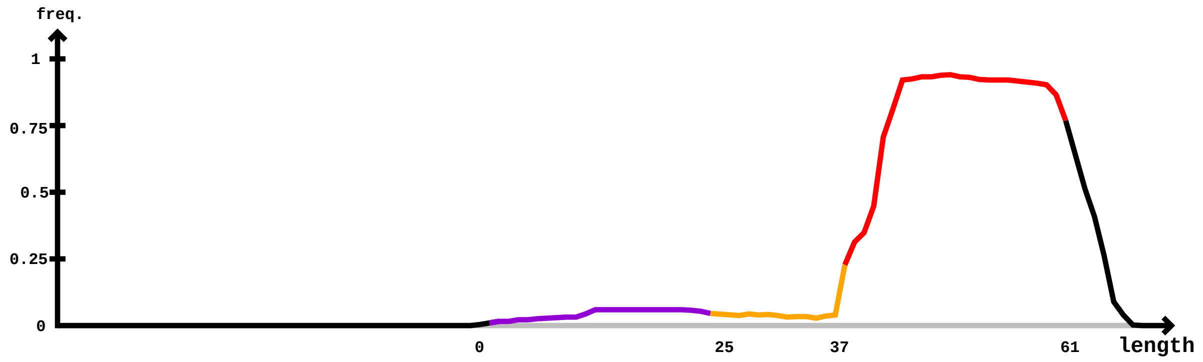

Star Mature

```
5'- ugcucggaauauuccgaggaacauuccgagcaacaugccccucgguauauuccgaucgaucgauggauauauggccaaaaacgcaucgaucgaugaacuuccgaggaaauauuc   -3'   obs
    ugcucggaauauuccgaggaacauuccgagcaacaugccccucgguauauuccgaucgaucgauggauauauggccaaaaacgcaucgaucgaugaacuuccgaggaaauauuc         exp
    ((((((((((.(((....))).)))))))))).......((((((....(((..(((((((((((.................))))))))))))))...)))))).........   reads   mm   sample
    ..............................................auauuccgaucgaucgauggaua.............................................   1       0    S03
    ................................................auuccgaucgaucgaugC................................................   1       1    S03
    ..................................................uccgaucgaucgauggauau............................................   1       0    S03
    .....................................................gaucgaucgauggauauaug.........................................   2       0    S03
    ......................................................aucgaucgauggauauaugAc.......................................   1       1    S03
    ......................................................aucgaucgauggauUuauggcca.....................................   2       1    S03
    ......................................................aucgaucgauggGuauauggcca.....................................   1       1    S03
    .....................................................................uaugAccaaaaacgcaucgaucg......................   1       1    S03
    ......................................................................augAccaaaaacgcaucgaucgau....................   1       1    S03
    ......................................................................auggccaaaaacgcaucgaucgau....................   1       0    S03
    ........................................................................ggAcaaaaacgcaucgaucg......................   1       1    S03
    ...........................................................................caaaaacgcaucgaucgU.....................   1       1    S03
    ...........................................................................caaaaacgcaucgaucgau....................   1       0    S03
    ..............................................................................aaacgcaucgaucgaCgaacuuc.............   1       1    S03
    ..............................................................................aaacgcaucgaucgaugaacuuccga..........   1       0    S03
    ...............................................................................aacgcaucgaucgaugaacA...............   1       1    S03
    ...............................................................................aacgcaucgaucgaugaacuuccA...........   1       1    S03
    ................................................................................acgcaucgaucgaugaacuu..............   1       0    S03
    ................................................................................acgcaucgaucgaugaacuuc.............   1       0    S03
    ................................................................................acgcaucgaucgaugaacuucc............   5       0    S03
    ................................................................................acgcaucgaucgaugaacuuccA...........   1       1    S03
    ................................................................................acgcaucgaucgaugaacuuccg...........   2       0    S03
    ................................................................................acgcaucgaucgaugaacAuccg...........   1       1    S03
    ................................................................................acgcaucgaucgaugaacuuccgG..........   1       1    S03
    ................................................................................acgcaucgaucgaugaacuuccga..........   3       0    S03
    .................................................................................cgcaucgaucgaugaacuu..............   1       0    S03
    .................................................................................cgcaucgaucgaugaacuucA............   1       1    S03
    .................................................................................cgcaucgaucgaugaacuucc............   1       0    S03
    .................................................................................cgcaucgaAcgaugaacuuccg...........   1       1    S03
    .................................................................................cgcaucgaucgaugaacuuccA...........   1       1    S03
    .................................................................................cgcaucgaucgaugaacuuccg...........   1       0    S03
    .................................................................................cgcaucgaucgaugaacuuccga..........   3       0    S03
    .................................................................................cgcaucgaucgaugaacuuccgag.........   4       0    S03
```

Star **Mature**

```
ugcucggaauauuccgaggaacauuccgagcaacaugccccucgguauauuccgaucgaucgauggauauauggccaaaaacgcaucgaucgaugaacuuccgaggaaauauuc
..................................................................................gcaucgaucgaugaacuuccga..........    1    0    S03
...................................................................................caucgaucgaugaacuuccA...........    1    1    S03
...................................................................................caucgaucgaugaacuuccga..........    4    0    S03
...................................................................................caucgaucgaugaacuuccgag.........    5    0    S03
...................................................................................caucgaucgaugaacuuccgagg........    3    0    S03
....................................................................................aucgaucgaugaacuuccA...........    1    1    S03
....................................................................................aucgaucgaugaacuuccCa..........    1    1    S03
....................................................................................aucgaucgaugaacuuccgag.........    3    0    S03
....................................................................................Uucgaucgaugaacuuccgag.........    2    1    S03
....................................................................................aucgaucgaugaacuuccgagg........    8    0    S03
....................................................................................aucgaucgaugaacuuccgagU.......    7    1    S03
....................................................................................aucgaucgaugaacuuccgagga.......    10   0    S03
....................................................................................aucgaucgaugaacuuccgaggaa......    11   0    S03
....................................................................................aGcgaucgaugaacuuccgaggaa......    1    1    S03
....................................................................................aucgaucgaugaacuuccgaggGa......    1    1    S03
.....................................................................................ucgaucgaugaacuuccA...........    1    1    S03
.....................................................................................ucgaucgaugaacuuccgag.........    1    0    S03
.....................................................................................ucgaucgaugaacuuccgagg........    3    0    S03
.....................................................................................ucgaucgaugaacuuccgagga.......    6    0    S03
.....................................................................................ucgaucgaugaacuuccgaggaa......    1    0    S03
.....................................................................................ucgaucgaugaacuuccgaggaaC.....    4    1    S03
.....................................................................................ucgaucgaugaacuuccgaggaaa.....    1    0    S03
......................................................................................cgaucgaugaacuuccgag.........    1    0    S03
......................................................................................cgaucgaugGacuuccgag.........    1    1    S03
......................................................................................cgaucgaugaacuuccgaggU.......    1    1    S03
......................................................................................cgaucgaugaacuuccgaggaa......    2    0    S03
......................................................................................cgaucgaugaacuuccgaggaaau....    3    0    S03
.......................................................................................gaucgaugaacuuccgaggaaau....    1    0    S03
........................................................................................aucgaugaacuuccgaggaa......    3    0    S03
..........................................................................................cgaugaacuuccgaggaa......    1    0    S03
..........................................................................................cgaugaacuuccgaggaaC.....    1    1    S03
...........................................................................................gaugaacuuccgaggaaaua...    1    0    S03

..........................................cgguauauuccgaucgaucgauA.................................................    1    1    S04
..........................................cgguauauuccgaucgaucgaugg................................................    1    0    S04
...........................................gguauauuccgaucgaucgaC..................................................    1    1    S04
...........................................gguauauuccgaucgaucgaug.................................................    1    0    S04
...........................................gguauauuccgaucgaucgaugg................................................    1    0    S04
............................................guauauuccgaucgaucgaugg................................................    1    0    S04
............................................guauauuccgaucgaucgaugAau..............................................    2    1    S04
..............................................auauuccgaucgaucgauggau..............................................    1    0    S04
..............................................auauuccgaucgaucgauggauC.............................................    1    1    S04
................................................auuccgaucgaucgauggauauau..........................................    1    0    S04
.................................................uuccgaucgaucgauggauaua...........................................    1    0    S04
...................................................cGgaucgaucgauggauauaugg........................................    1    1    S04
.....................................................gaucgaucgauggauaua...........................................    1    0    S04
.....................................................gaucgaucgauggGuauaugg........................................    2    1    S04
.....................................................gaucgaucgauggauUuauggcca.....................................    1    1    S04
......................................................aucgaucgaugUauauaugg........................................    1    1    S04
......................................................aucgaucgauggauUuauggcca.....................................    1    1    S04
......................................................aucgaucgauggauUuauggccaa....................................    2    1    S04
......................................................................augAccaaaaacgcaucgaucgau....................    1    1    S04
........................................................................ggAcaaaaacgcaucgaucg......................    1    1    S04
........................................................................ggAcaaaaacgcaucgaucgaug...................    1    1    S04
..........................................................................ccaaaaacgcaucgaucg......................    1    0    S04
.............................................................................aaaacgcUucgaucgaugaac................    1    1    S04
.............................................................................aaaacgcaucgaucgaugaacuuc.............    1    0    S04
..............................................................................aaacgcaucgaucgaugGac................    1    1    S04
..............................................................................aaacgcaucgaucgaugaacuucc............    1    0    S04
..............................................................................aaacgcaucgaucgaCgaacuucc............    1    1    S04
..............................................................................aGacgcaucgaucgaugaacuucc............    1    1    S04
................................................................................acgcaucgaucgauUaacuucc............    1    1    S04
................................................................................acgcaCcgaucgaugaacuucc............    1    1    S04
................................................................................acgcaucgaucgaugaacuucc............    6    0    S04
................................................................................acgcaucgaucgaugaacuucA............    1    1    S04
................................................................................acgcaucgaucgaugaacuuccg...........    14   0    S04
................................................................................acgcaucgaucgGugaacuuccg...........    1    1    S04
................................................................................acgcaucgaucgaugaacuuccA...........    10   1    S04
................................................................................acgcaucgaucgaCgaacuuccg...........    1    1    S04
................................................................................acgcaucgaucgaugaacuuccgU..........    1    1    S04
```

Star Mature

```
ugcucggaauauuccgaggaacauuccgagcaacaugccccucgguauauuccgaucgaucgauggauauauggccaaaaacgcaucgaucgaugaacuuccgaggaaauauuc
................................................................................acgcaucgaucgaugaacuuccAa..........	1	1	S04
................................................................................acgcaucgaucgaugaacuuccga..........	40	0	S04
................................................................................acgcaAcgaucgaugaacuuccga..........	1	1	S04
................................................................................acUcaucgaucgaugaacuuccga..........	1	1	S04
................................................................................acgcaucgaucgaugaacuuccCa..........	1	1	S04
.................................................................................cgcaucgaucgaugaacu...............	1	0	S04
.................................................................................cgcaucgaucgaugaacuuccg...........	5	0	S04
.................................................................................cgcaucgaucgaugaacuuccA...........	2	1	S04
.................................................................................cgcaucgaucgaugGacuuccga..........	1	1	S04
.................................................................................cgcaucgaucgaugaacuuccga..........	3	0	S04
.................................................................................cgcaucgaucgaugaacuuccgag.........	18	0	S04
..................................................................................gcaucgaucgaugaacuuccA...........	2	1	S04
..................................................................................gcaucgaucgaugaacuuccga..........	4	0	S04
..................................................................................gcaucgaucgaugaacuuccgU..........	1	1	S04
..................................................................................gcaucgaAcgaugaacuuccga..........	1	1	S04
..................................................................................gcaucgaucgaugaacuuccgag.........	3	0	S04
..................................................................................gcaucgaucgaugaacuuccgagg........	6	0	S04
...................................................................................caucgaucgaugaacuuccg...........	1	0	S04
...................................................................................caucgaucgaugaacuuccgU..........	1	1	S04
...................................................................................caucgaucgaugaacuuccga..........	1	0	S04
...................................................................................caucgaucgaugaacuuccgC..........	1	1	S04
...................................................................................caucgaucgaugaacuuccgag.........	7	0	S04
...................................................................................caucgaucgaugaacuuccgagg........	4	0	S04
...................................................................................caucgaucgaugaacuuccgagU........	1	1	S04
...................................................................................caucgaucgaugaacuuccgaggU.......	1	1	S04
...................................................................................caucgaucgaugaacuuccgaggG.......	2	1	S04
...................................................................................caucgaucgaugaacuuccgagga.......	17	0	S04
...................................................................................caucgaucUaugaacuuccgagga.......	1	1	S04
....................................................................................aucgaucgaugaacuuccA...........	1	1	S04
....................................................................................aucgaucgaugGacuuccg...........	1	1	S04
....................................................................................aucgaucgaugaacuuccgG..........	1	1	S04
....................................................................................aucgaucgaugaacuuccgU..........	2	1	S04
....................................................................................aucgaucgGugaacuuccga..........	1	1	S04
....................................................................................aucgaucgaugaacuuccgag.........	5	0	S04
....................................................................................aucgaucgaugaacuuccgagU........	2	1	S04
....................................................................................aucgaucgaugaacuuccgagg........	10	0	S04
....................................................................................aucgaucgGugaacuuccgagg........	1	1	S04
....................................................................................aucgaucgaugaacuuccgagga.......	11	0	S04
....................................................................................aCcgaucgaugaacuuccgagga.......	1	1	S04
....................................................................................aGcgaucgaugaacuuccgagga.......	1	1	S04
....................................................................................aucgaucgaugaacuuccgaggGa......	2	1	S04
....................................................................................aGcgaucgaugaacuuccgaggaa......	1	1	S04
....................................................................................aucgaucgaugaacuuccgaggaa......	39	0	S04
....................................................................................aucgaCcgaugaacuuccgaggaa......	1	1	S04
....................................................................................Nucgaucgaugaacuuccgaggaa......	1	1	S04
....................................................................................aucgaucgaugaacuuccgaggaG......	2	1	S04
....................................................................................aucgaucgaugaacuuccgaggaaCu....	3	1	S04
.....................................................................................ucgaucgaugaacuuccgag.........	1	0	S04
.....................................................................................ucgaucgaugaacuuccgagg........	5	0	S04
.....................................................................................ucgaucgaugaacuuccgagga.......	7	0	S04
.....................................................................................ucgaucgaugaacuuccgaggC.......	1	1	S04
.....................................................................................Ccgaucgaugaacuuccgagga.......	1	1	S04
.....................................................................................ucgaucgaugaacuuccgaggG.......	1	1	S04
.....................................................................................ucgaucgaugaacuuccgaggaa......	4	0	S04
.....................................................................................ucgaucgaugaacuuccgaggGa......	1	1	S04
.....................................................................................ucgaucgaugaacuuccgaggaaC.....	8	1	S04
.....................................................................................ucgaucgaugaacuuccgaggaaa.....	3	0	S04
.....................................................................................ucgaucgaugaacuuccgaggaaG.....	4	1	S04
......................................................................................cgaucgaugaacuuccgG..........	1	1	S04
......................................................................................cgaucgaugaacuuccgC..........	1	1	S04
......................................................................................cgaucgaugaacuuccgagg........	1	0	S04
......................................................................................cgaucgaugaacuuccgagU........	1	1	S04
......................................................................................cgaucgaugaacuuccgagC........	1	1	S04
......................................................................................cgaucgaugaacuuccgaggU.......	1	1	S04
......................................................................................cgaucgaugaacuuccgaggG.......	2	1	S04
......................................................................................cgaucgaugaacuuccgagga.......	10	0	S04
......................................................................................cgaucgaugaacuCccgaggaa......	1	1	S04
......................................................................................cgaucgaugaacuuccgaggaC......	1	1	S04
......................................................................................cgaucgaugaacuuccgaggaa......	13	0	S04
......................................................................................cgaucgaugaacuuccgaggaaC.....	2	1	S04
```

Star Mature

```
ugcucggaauauuccgaggaacauuccgagcaacaugccccucgguauauuccgaucgaucgauggauauauggccaaaaacgcaucgaucgaugaacuuccgaggaaauauuc
.....................................................................................cgaucgaugaacuuccgaggaaau....   8   0   S04
.....................................................................................cgaucgaugaacuuccgaggaaaC....   1   1   S04
.....................................................................................cgaucgaugaacuuccgaggaaCu....   1   1   S04
.....................................................................................Agaucgaugaacuuccgaggaaau....   1   1   S04
.....................................................................................cgaucgaugaacuuccgaggaaGu....   1   1   S04
......................................................................................gaucgaugaacuuccgaggaaC.....   1   1   S04
.......................................................................................aucgaugaacuuccgaggaaa.....   1   0   S04
.........................................................................................cgaugaacuuccgaggaa......   1   0   S04
```

Provisional ID	:	chrC02_1214158
Score total	:	0.2
Score for star read(s)	:	-1.3
Score for read counts	:	0
Score for mfe	:	1.5
Score for randfold	:	0
Score for cons. seed	:	
Total read count	:	1143
Mature read count	:	1143
Loop read count	:	0
Star read count	:	0

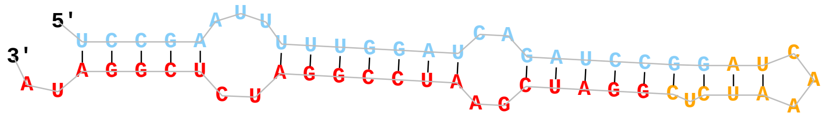

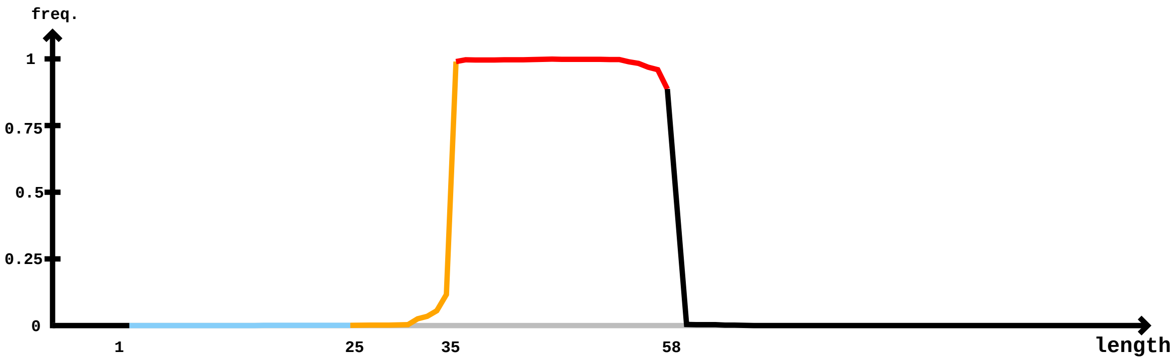


```
                Star                                Mature
5'- cgaauauccgaauuuuuggaucagauccggaucaaaucucggaucgaauccggaucucggauaaaaguuucaugacaaccagaucagcugccuuaucuaugagacguggguc   -3'    exp
    ....(((((((...(((((((..(((((((((...))).))))))..)))))))..)))))))...(((((((((....(((.....))).....)).)))))))).......     reads      mm         sample
    ...............................ucaaaucucggaucgaaucc.............................................................     1          0          S03
    ...................................aucucggaucgaauccggaucuG......................................................     1          1          S03
    ....................................ucucggaucgaauccggaucuc......................................................     2          0          S03
    ....................................ucucggaucgaauccggaucucg.....................................................     3          0          S03
    ....................................ucucggaucgaauccggaucucgg....................................................     4          0          S03
    ....................................ucucggaucgaauccggaucucgC....................................................     1          1          S03
    .....................................cucggaucgaauccggauc........................................................     1          0          S03
    .....................................cucggaucgaauccggaucuc......................................................     1          0          S03
    .....................................cucggaucgaauccggaucucg.....................................................     1          0          S03
    .....................................cucggaucgaauccggaucucgga...................................................     3          0          S03
    .....................................cucggaucgaauccggaucucggau..................................................     1          0          S03
    ......................................ucggaucgaauccggaucucggau..................................................     1          0          S03
    ......................................ucggaucgaauccggaucucggaA..................................................     1          1          S03
    ......................................ucggaucgaauccggaucucggaua.................................................     4          0          S03
    ......................................ucggaucgaauccggaucucggauU.................................................     8          1          S03
    .......................................cggaucgaauccggaucucgg....................................................     1          0          S03
    .......................................cggaucgaauccggaucucgga...................................................     1          0          S03
    .......................................cggaucgaauccggaucucggau..................................................     11         0          S03
    .......................................cggaucgaauccggaucucggaua.................................................     21         0          S03
    .......................................cggaucgaauccggUucucggaua.................................................     1          1          S03
    .......................................cggaucgaauccggaucucggauG.................................................     2          1          S03
    ........................................ggaucgaauccggaucucggaA..................................................     1          1          S03
    ........................................ggaucgaauccggaucucggau..................................................     23         0          S03
    ........................................ggaucgaauccggaucucggauG.................................................     119        1          S03
    ........................................ggaCcgaauccggaucucggaua.................................................     1          1          S03
    ........................................ggaucgaauccggaucucggauU.................................................     3          1          S03
    ........................................ggaucgaauccggaucucggaGa.................................................     3          1          S03
    ........................................Ngaucgaauccggaucucggaua.................................................     2          1          S03
    ........................................ggaucgaauccggaucucggCua.................................................     1          1          S03
    ........................................ggaucgaauccggUucucggaua.................................................     1          1          S03
    ........................................ggaucgaauccggaucucggaua.................................................     324        0          S03
    ........................................ggGucgaauccggaucucggaua.................................................     2          1          S03
    ........................................ggaucgaauccggaucucggauUa................................................     1          1          S03
    ........................................ggaucgaauccggaucucggauaa................................................     4          0          S03
```

```
              Star                                   Mature
cgaauauccgaauuuuuggaucagauccggaucaaaucucggaucgaauccggaucucggauaaaaguuucaugacaaccagaucagcugccuuaucuaugagacguggguc
.........................................gGucgaauccggaucucggaua.................................................   1   1   S03
.............................................gaauccggaucucggauaaaagu............................................   1   0   S03
.................................................ccggaucucggauaaaagu............................................   1   0   S03
..................................................cggaucucggauaaaaguuuU.........................................   1   1   S03

....................ucagauccggaucaaaucucgg......................................................................   1   0   S04
..................................aaucucggaucgaauccggaucuc......................................................   1   0   S04
....................................ucucggaucgaauccggaucuc......................................................   4   0   S04
....................................ucucggaucgaauccggaucucg.....................................................   3   0   S04
....................................ucucggaucgaauccggaucucgg....................................................   9   0   S04
.....................................cucggaucgaauccggaucuc......................................................   1   0   S04
.....................................cucggaucgaauccggaucucggU...................................................   1   1   S04
.....................................cucggaucgaauccggaucucgga...................................................   1   0   S04
.....................................cucggaucgaauccggaucucggau..................................................   1   0   S04
......................................ucggaucgaauccggaucucggauU.................................................   11  1   S04
.......................................cggaucgaauccggaucucggau..................................................   6   0   S04
.......................................cggaucgaauccggGucucggau..................................................   1   1   S04
.......................................cggaucgaauccggaucucggaua.................................................   25  0   S04
.......................................cggaucgaauccggaucucggauU.................................................   2   1   S04
.......................................cggaucgaauccggaucucggauG.................................................   1   1   S04
........................................ggaucgaauccggaucucgg....................................................   2   0   S04
........................................ggaucgaauccggaucucgga...................................................   4   0   S04
........................................ggaucgaauccggGucucggau..................................................   1   1   S04
........................................ggaucgaauccggaucucggau..................................................   39  0   S04
........................................ggaucgaauccggaucucggauG.................................................   108 1   S04
........................................ggaucgaauccggGucucggaua.................................................   2   1   S04
........................................ggaucgaauccggaGcucggaua.................................................   1   1   S04
........................................ggaucgaauccggaucucggauU.................................................   4   1   S04
........................................gCaucgaauccggaucucggaua.................................................   1   1   S04
........................................ggaucgaauccggaAcucggaua.................................................   1   1   S04
........................................ggaucgaauccggaucucggaGa.................................................   4   1   S04
........................................ggaCcgaauccggaucucggaua.................................................   2   1   S04
........................................ggGucgaauccggaucucggaua.................................................   1   1   S04
........................................ggaucgaauccggaucucggaAa.................................................   1   1   S04
........................................ggaucgaauccgCaucucggaua.................................................   1   1   S04
........................................ggaucgaauccggaucucggaua.................................................   365 0   S04
........................................ggaucgaauccggaucucggauaU................................................   1   1   S04
........................................ggaucgaauccggaucucggauaG................................................   1   1   S04
........................................ggaucgaauccggaucucggauaa................................................   11  0   S04
........................................ggaucgaauccggaucucggauaaa...............................................   1   0   S04
.........................................gaucgaauccggaucucgga...................................................   1   0   S04
.........................................gaucgaauccggaucucggauG.................................................   2   1   S04
.........................................gaucgaauccggaucucggaua.................................................   4   0   S04
................................................uccggaucucggauaaaaguuu..........................................   1   0   S04
```

Provisional ID	: chrC02_1226545
Score total	: 1.7
Score for star read(s)	: -1.3
Score for read counts	: 0
Score for mfe	: 3
Score for randfold	: 0
Score for cons. seed	:
Total read count	: 15
Mature read count	: 11
Loop read count	: 2
Star read count	: 2

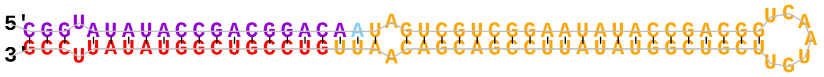

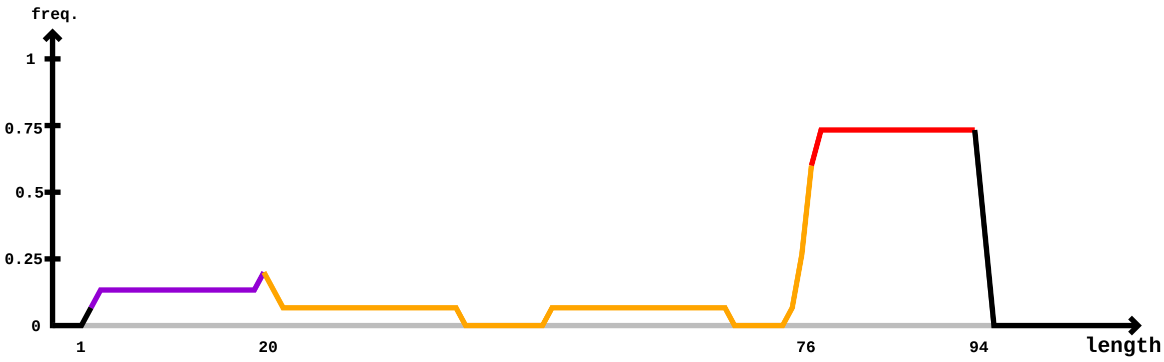

Star **Mature**

```
5'- gucgguauauaccgacggacaauagucgucggaauauaccgacggucaauguucgucgguauauuccgacgacaauuguccgucgguauauuccgacgacnnnnnnnnnnn  -3'   obs
    gucgguauauaccgacggacaauagucgucggaauauaccgacggucaauguucgucgguauauuccgacgacaauuguccgucgguauauuccgacgacnnnnnnnnnnn         exp
    (((((.((((((((((((((((.((((((((((((((((((((.......)))))))))))))))))))).)))))))))))))))).))))).................. reads    mm    sample
    ...........................................................................uuguccgucgguauauuccg................ 1        0     S03
    .............................................................................guccgucgguauauuccg................ 1        0     S03
    ...............................................................................uccgucgguauauuccga.............. 1        0     S03

    ..cggGauauaccgacggaca.......................................................................................... 1        1     S04
    ...gguauauaccgacggaca.......................................................................................... 1        0     S04
    ....................aauagucgucggaauauaccg...................................................................... 1        0     S04
    ..................................................guucgucgguauauuccgU.......................................... 1        1     S04
    ............................................................................uguccgucgguauauuccg................ 2        0     S04
    ............................................................................uguccgucgguauauuccga............... 1        0     S04
    .............................................................................guccgucgguauaCuccg................ 1        1     S04
    .............................................................................guccgucgguauauuccg................ 3        0     S04
    ...............................................................................uccgucgguauauuccga.............. 1        0     S04
```

Provisional ID : chrC02_1229494
Score total : 1.1
Score for star read(s) : -1.3
Score for read counts : 0
Score for mfe : 2.4
Score for randfold : 0
Score for cons. seed :
Total read count : 37
Mature read count : 36
Loop read count : 1
Star read count : 0

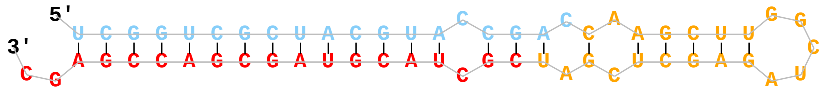

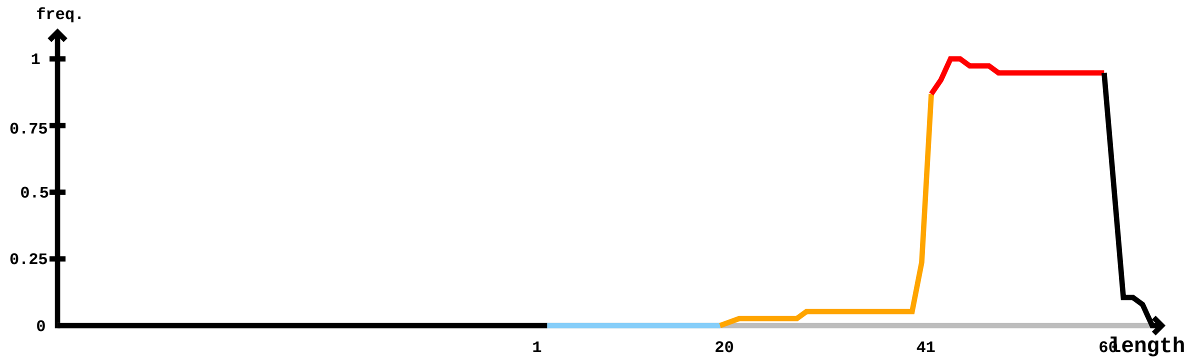

Star Mature

```
5'- gauucaaacgagaacgcaaaggaggaccuuucugaggccuugugcgugcucggucgcuacguaccgaccaagcuuggcuagagcucgaucgcuacguagcgaccgagcugugu  -3'   exp
    .............(((((...((((.((((...)))))))).)))))((((((((((((((((.(((.(.(((((.....))))).).))).)))))))))))))))).....   reads     mm      sample
    ............................................................................gcuagagcucUaucgcuacg.................   1         1       S03
    ........................................................................................ucgcuacguagcgaccgagc.....   3         0       S03
    ........................................................................................ucgcuacguagcgaccgagcu....   2         0       S03
    .........................................................................................cgcuacguagcgaccgagc.....   17        0       S03
    .........................................................................................cgcuacguagcgCccgagcu....   1         1       S03
    .........................................................................................cgcuacguagcgaccgagcu....   2         0       S03
    ..........................................................................................gcuacguagcgaccgagc.....   1         0       S03
    ..........................................................................................gcuaAguagcgaccgagcugu..   1         1       S03
    ...........................................................................................cuacguagcgaccgagcu....   2         0       S03
    ...........................................................................................cuacguagcgaccgagcugug.   1         0       S03

    .....................................................................aagcuuggcuagagcucgaucgcG....................   1         1       S04
    ........................................................................................ucgcuacguagcgaccgagc.....   1         0       S04
    ........................................................................................ucgcuacguagcgaccgagcu....   1         0       S04
    .........................................................................................cgcuacguagcgaccgagc.....   1         0       S04
    .........................................................................................cgcuacguagcgaccgagcu....   1         0       S04
    .........................................................................................cgcuacguagcgaccgagcugug.   1         0       S04
    .........................................................................................cgcuacguagcgaccgagcuCug.   1         1       S04
```

Provisional ID	:	chrC02_1246413
Score total	:	1.2
Score for star read(s)	:	-1.3
Score for read counts	:	0
Score for mfe	:	2.5
Score for randfold	:	0
Score for cons. seed	:	
Total read count	:	50
Mature read count	:	46
Loop read count	:	2
Star read count	:	2

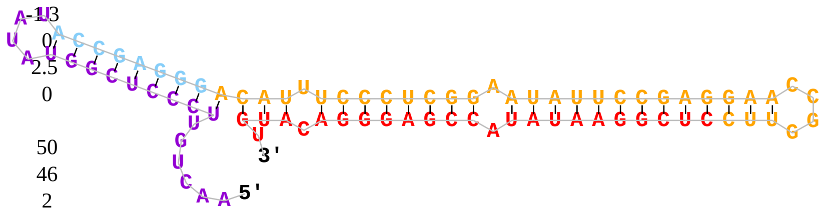

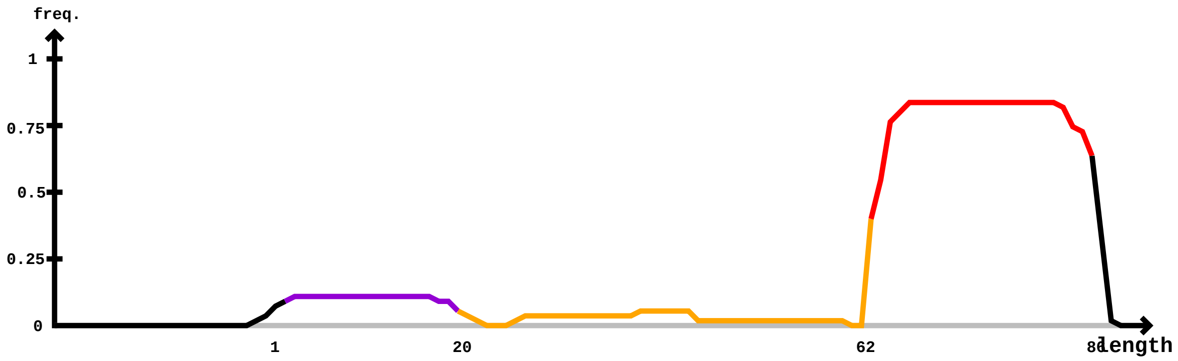


```
                        Star                                                            Mature
5'- ccuuacugacuagagaaaugggaacuguucccucgguauauaccgagggacauuucccucggaauauuccgaggaaccgguuccucggaauauaccgagggacauguuccuc  -3'   obs
    ccuuacugacuagagaaaugggaacuguucccucgguauauaccgagggacauuucccucggaauauuccgaggaaccgguuccucggaauauaccgagggacauguuccuc         exp
    ...................(((((((((.((((((((((((.(((((((((.....(((((((....))))))).....))))))))).))))))))))))))).)))))).   reads   mm   sample
    ...................gggaacuguucccucgguauauU......................................................................   1       1    S03
    .....................gaacuguucccucgguaua........................................................................   1       0    S03
    ...........................................................cggaauauuccgaggaacGggu...............................   1       1    S03
    ...................................................................................cuUggaauauaccgagggacaugu.....   1       1    S03
    ...................................................................................cucggaauauaccgagggacaugu.....   7       0    S03
    ...................................................................................cucggaauauaccgaAggacaugu.....   1       1    S03
    ....................................................................................ucggaauauaccgagggaca........   1       0    S03
    ....................................................................................ucggaauauaccgagggacaug......   2       0    S03
    .....................................................................................cggaauauacAgagggacaugu.....   1       1    S03
    .....................................................................................cggaauauaccgagggacaugu.....   1       0    S03
    .......................................................................................gaauauaccgagggacau.......   1       0    S03

    ....................ggaacuguucccucggua..........................................................................   1       0    S04
    .....................gaacuguucccucgguaua........................................................................   1       0    S04
    ......................aacuguucccucgguauau.......................................................................   1       0    S04
    .......................acuguucccucgguauauCc.....................................................................   1       1    S04
    ..............................................gggacauAucccucggaau...............................................   1       1    S04
    ...............................................ggacauuucccucggaUu...............................................   1       1    S04
    ...................................................................................cucggaauauaccgagggacaugu.....   11      0    S04
    ...................................................................................cucggaauauacUgagggacaugu.....   1       1    S04
    ...................................................................................cucggaauauaccgagggacaugC.....   1       1    S04
    ....................................................................................ucggaauauaccgagggaca........   1       0    S04
    ....................................................................................ucggaauauaccgagggacaug......   2       0    S04
    ....................................................................................ucggaauauaccgagggacaugCu....   1       1    S04
    ....................................................................................ucggaauauaccgagggacauguu....   1       0    S04
    .....................................................................................cggaauauCccgagggac.........   1       1    S04
    .....................................................................................cggaauauaccgagggaca........   2       0    S04
    .....................................................................................cggaauauaccgagggacaugG.....   1       1    S04
    .....................................................................................cggaauauaccgagggacaugu.....   3       0    S04
    .....................................................................................cggaauauaccgagggacaugC.....   1       1    S04
    .....................................................................................cggaauauaccgagggacauguu....   1       0    S04
    .....................................................................................Aggaauauaccgagggacauguuc...   1       1    S04
    ......................................................................................ggaauauaccgagggacaug......   1       0    S04
```

```
                      Star                                                            Mature
ccuuacugacuagagaaaugggaacuguucccucgguauauaccgagggacauuucccucggaauauuccgaggaaccgguuccucggaauauaccgagggacauguuccuc
......................................................................................ggaauauaccgagggacaugu.....    1    0    S04
.......................................................................................gaauauaccgagggacaugu.....    1    0    S04
```

Provisional ID	:	chrC02_1246936
Score total	:	114.4
Score for star read(s)	:	3.9
Score for read counts	:	110.2
Score for mfe	:	0.2
Score for randfold	:	0
Score for cons. seed	:	
Total read count	:	228
Mature read count	:	104
Loop read count	:	4
Star read count	:	120

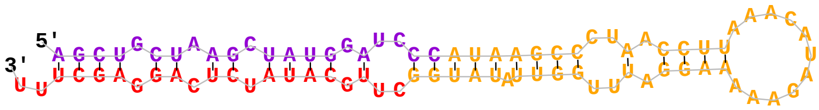

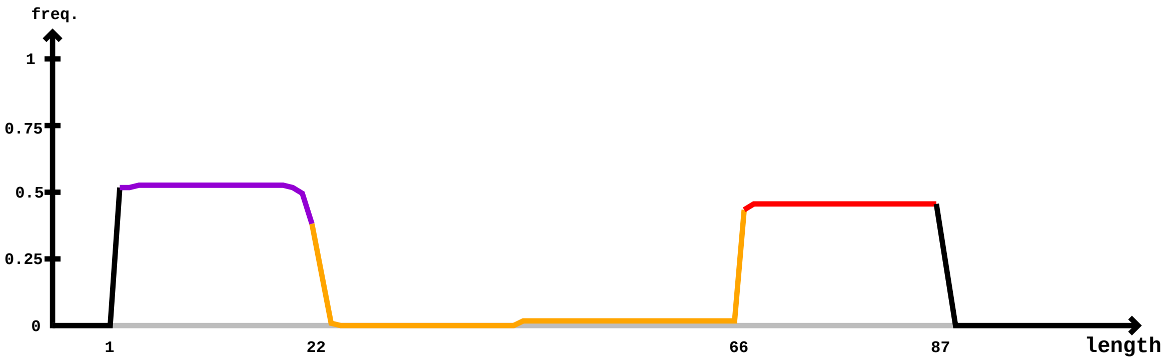

Star Mature

```
5'- agaaaagcugcuaagcuauggaucccauaagcccuaaccuuaaacauagaaaaaggauuugguuauauggcuugcauaucucaggagcuuuaacuugcccuuuaauggcuu  -3'  obs
    agaaaagcugcuaagcuauggaucccauaagcccuaaccuuaaacauagaaaaaggauuugguuauauggcuugcauaucucaggagcuuuaacuugcccuuuaauggcuu        exp
    ...((((((.((.((.((((.(..(((((((((..(.((((..........)))).)..)))).)))))..).)))).)).)).))))))).....(((.......)))..  reads  mm  sample
    .....agcugcuaagcuauggau........................................................................................  2  0  S03
    .....agcugcuaagcuauggauc.......................................................................................  2  0  S03
    .....agcugcuaagcuauggaucc......................................................................................  8  0  S03
    .....agcugcuaagcuauggaucU......................................................................................  2  1  S03
    .....agcugcuaagcuauggauccU.....................................................................................  9  1  S03
    .....agUugcuaagcuauggauccc.....................................................................................  1  1  S03
    .....agcugcuaagcuauggaCccc.....................................................................................  1  1  S03
    .....agcugcuaagcuauggauccc.....................................................................................  22  0  S03
    .....agcugcuaagcuauggauccA.....................................................................................  1  1  S03
    .....agcugcuaagcuauggauccca....................................................................................  2  0  S03
    .....agcugcuaagcuauggaucccU....................................................................................  1  1  S03
    ......................................................................cuugcauaucucGggagcuuu....................  1  1  S03
    ......................................................................cuugcauaucucaggagcuuG....................  1  1  S03
    ......................................................................cuugcauaucucaggagcuCu....................  1  1  S03
    ......................................................................cuugcauaucucaggagcuuA....................  1  1  S03
    ......................................................................cuugcauaucucaggagcuuC....................  7  1  S03
    ......................................................................cuugcauaucucaggagcuuu....................  35  0  S03
    .......................................................................uugcauaucucaggagcuuu....................  1  0  S03
    .......................................................................uugcauaucucaggagcCuu....................  1  1  S03
    .......................................................................uugcauaucucaAgagcuuu....................  1  1  S03

    .....agcugcuaagcuauggauc.......................................................................................  3  0  S04
    .....agcugcuaagcuauggaucA......................................................................................  1  1  S04
    .....agcugcuaagcuauggaucc......................................................................................  13  0  S04
    .....agcugcuaagcuauggaCcc......................................................................................  1  1  S04
    .....agcugcuaagcuauggaucU......................................................................................  1  1  S04
    .....agcugcuaagcuauggauccc.....................................................................................  25  0  S04
    .....agcugcuaagcuauggauccA.....................................................................................  7  1  S04
    .....agcugcuaagcuauggauccU.....................................................................................  16  1  S04
    .......cugcuaagcuauggaucccau...................................................................................  2  0  S04
    ...............................................agaaaaaggauuugguuauaugg.........................................  3  0  S04
    ...............................................Ggaaaaaggauuugguuauaugg.........................................  1  1  S04
    ......................................................................cuugcauaucucaggagcuuG....................  1  1  S04
```

```
     Star                                                 Mature
agaaaagcugcuaagcuauggaucccauaaagcccuaaccuuaaacauagaaaaaggauuugguuauauggcuugcauaucucaggagcuuuaacuugcccuuuaauggcuu
.......................................................................cuugcauaucucaggagcuuu....................  43   0   S04
.......................................................................cuuCcauaucucaggagcuuu....................  1    1   S04
.......................................................................cuuUcauaucucaggagcuuu....................  1    1   S04
.......................................................................cuugcauaucucaggagcuuA....................  1    1   S04
.......................................................................cuugcauaucucaggagcuCu....................  1    1   S04
.......................................................................cuugcauaucuAaggagcuuu....................  1    1   S04
.......................................................................cuugcauaucucaggagcuuC....................  3    1   S04
.......................................................................cuugcauaucucaggagcuuuG...................  1    1   S04
........................................................................uugcauaucucaggagcuuu....................  2    0   S04
```

Provisional ID : chrC02_1280968
Score total : 1075.1
Score for star read(s) : 3.9
Score for read counts : 1082.5
Score for mfe : -11.2
Score for randfold : 0
Score for cons. seed :
Total read count : 2135
Mature read count : 2134
Loop read count : 0
Star read count : 1

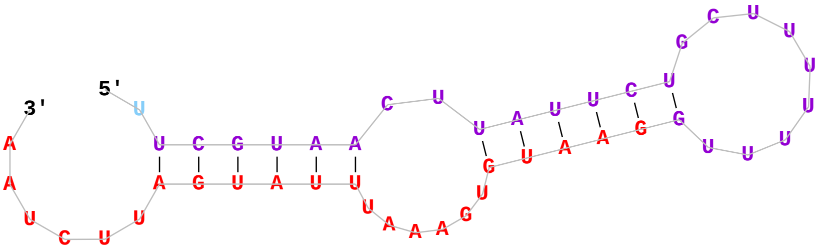

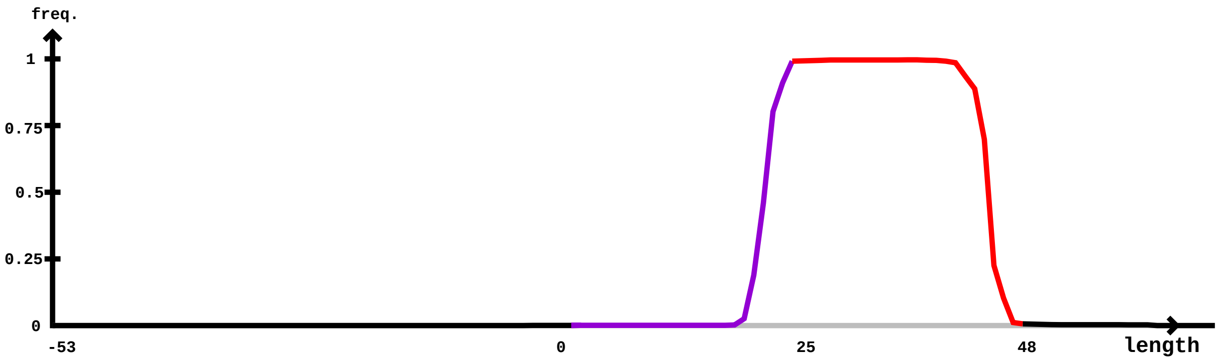

Star Mature

```
5'- auuugauggguaauauguaaauaaaaaucauuguuauuuguauuuaucagaguucguaacuuauucugcuuuuuuuggaaugugaaauuuaugauucuaauaaauauauaaaauaa   -3'   obs
    auuugauggguaauauguaaauaaaaaucauuguuauuuguauuuaucagaguucguaacuuauucugcuuuuuuuggaaugugaaauuuaugauucuaauaaauauauaaaauaa         exp
    ....(((((....(((....)))....)))))......(((((((((.(((((.(((((..((((((.........))))))......)))))))))).)))))))))........   reads   mm   sample
    ................................................agaguucguaacuuauucugcuu.............................................   1   0   S03
    .....................................................ucguaacuuaCucugcuuuuuuug.......................................   1   1   S03
    .....................................................................uuuuuuuggaaugugaaauuuaugaC.....................   1   1   S03
    .....................................................................uuuuuuuggaaugugaGauuuaugau.....................   1   1   S03
    ......................................................................uuuuuuggaaugugaaauuuGu........................   1   1   S03
    ......................................................................uuuuuuggaaugugaCauuuaug.......................   1   1   S03
    ......................................................................uuuuuuggaaugugaaauuuauA.......................   2   1   S03
    ......................................................................uuuuuuggaaugugaaauuuaug.......................   8   0   S03
    ......................................................................uuuuuuggaaugugaaauuuauga......................   3   0   S03
    ......................................................................uuuuuuggaaugugaaauuuaugau.....................   2   0   S03
    .......................................................................uuuuuggaaugugaaauu...........................   3   0   S03
    .......................................................................uuuuuggaaugugaaauuuau........................   2   0   S03
    .......................................................................uuuuuggaaugugaaauuuaug.......................   13   0   S03
    .......................................................................uuuuuggaaugugaaGuuuaug.......................   1   1   S03
    .......................................................................uuuuuggaauguUaaauuuaug.......................   2   1   S03
    .......................................................................uuuuuggaaugugUaauuuaug.......................   1   1   S03
    .......................................................................uuuuuggaaugugaaauuuauga......................   16   0   S03
    .......................................................................uuuuuggaaugugaaauuuaugG......................   1   1   S03
    .......................................................................uuuuuggaaugugaaauuuauUau.....................   1   1   S03
    .......................................................................uuuuuggaaugugaaauuuaugaG.....................   1   1   S03
    .......................................................................uuuuuggaauguUaaauuuaugau.....................   5   1   S03
    .......................................................................uuuuuggaaugugaaauuuaugGu.....................   1   1   S03
    .......................................................................uuuuuggaGugugaaauuuaugau.....................   1   1   S03
    .......................................................................uuuuuggaaugugaGauuuaugau.....................   1   1   S03
    .......................................................................uuuuuggaaugugaaauuuaugau.....................   95   0   S03
    .......................................................................uuuuuggaaugCgaaauuuaugau.....................   1   1   S03
    .......................................................................uuuuuggaaugugaaauuuaugaA.....................   2   1   S03
    .......................................................................uuuuuggaaugugaaauuuaugaC.....................   11   1   S03
    .......................................................................uuuuuggaaGgugaaauuuaugau.....................   1   1   S03
    .......................................................................uuuuuUgaaugugaaauuuaugau.....................   1   1   S03
    .......................................................................uuuuuggaaugugaaauuuaugauu....................   5   0   S03
    .......................................................................uuuuuggaaugugaaauuuaugauG....................   1   1   S03
    .......................................................................uuuuuggaaugugaaauuuaugauuc...................   1   0   S03
```

Star Mature

```
auuugauggguaauauguaaauaaaaaucauuguuauuuguauuuaucagaguucguaacuuauucugcuuuuuuuggaaugugaaauuuaugauucuaauaaauauauaaauaa
........................................................................Cuuuggaaugugaaauuu.........................   1     1    S03
........................................................................uuuuggaaugugaaauuu.........................   2     0    S03
........................................................................uuuuggaaugugaaauuua........................   1     0    S03
........................................................................uuuuggaaugugaaauuuau.......................   1     0    S03
........................................................................uuuuggaaugugaaauuuaug......................   12    0    S03
........................................................................uuuuggaaugugaaauuuaugG.....................   1     1    S03
........................................................................uuuuggaaugugaaauuuauga.....................   3     0    S03
........................................................................Cuuuggaaugugaaauuuaugau....................   1     1    S03
........................................................................uuuuggaaugugaaauuuaugaA....................   1     1    S03
........................................................................uuuuggaaugugUaauuuaugau....................   1     1    S03
........................................................................uuuuggaaugugaaauuuaugau....................   29    0    S03
........................................................................uuuuggaaugugaGauuuaugauu...................   2     1    S03
........................................................................uuuuggaaugugaaauuuaugauC...................   19    1    S03
........................................................................uuuuggaaugugaaauuuaugauu...................   170   0    S03
........................................................................uuuuggaaugAgaaauuuaugauu...................   1     1    S03
........................................................................uuuuggaaugugaaauuuaugaCu...................   2     1    S03
........................................................................uuuuggaaugugaaauuuaugauG...................   3     1    S03
........................................................................uuuuggaauguUaaauuuaugauu...................   9     1    S03
........................................................................uuuuggaaugGgaaauuuaugauu...................   2     1    S03
........................................................................Cuuuggaaugugaaauuuaugauu...................   1     1    S03
........................................................................Auuuggaaugugaaauuuaugauu...................   1     1    S03
........................................................................uuuuggaaugugaaauuuaugauA...................   1     1    S03
........................................................................uuuuggaaugugUaauuuaugauu...................   1     1    S03
.........................................................................uuuggaaugugaaauuuaug......................   5     0    S03
.........................................................................uuuggaaugugaaauuuauga.....................   2     0    S03
.........................................................................uuuggaaugugaaauuuaugUu....................   1     1    S03
.........................................................................uuuggaaugugaaauuuaugaC....................   1     1    S03
.........................................................................uuuggaaugugaaauuuaugau....................   13    0    S03
.........................................................................uuuggaaugugaaauuuaugauC...................   19    1    S03
.........................................................................uuAggaaugugaaauuuaugauu...................   1     1    S03
.........................................................................uuuUgaaugugaaauuuaugauu...................   1     1    S03
.........................................................................uuuggaaugugaaauuuaugUuu...................   1     1    S03
.........................................................................uuuggaaugCgaaauuuaugauu...................   1     1    S03
.........................................................................Guuggaaugugaaauuuaugauu...................   3     1    S03
.........................................................................uuuggaaugugaaauuuaugauG...................   10    1    S03
.........................................................................uuuggaaugugaaauuuaugauA...................   5     1    S03
.........................................................................uuuggaaugugaaauuuaugauu...................   143   0    S03
.........................................................................uuuggaaugugaaauuuaugaCu...................   2     1    S03
.........................................................................uuuggaauguUaaauuuaugauu...................   5     1    S03
.........................................................................uuuggaaugugaaauuuaugGuu...................   1     1    S03
.........................................................................uuuggaaugugaaauuuaugauuc..................   80    0    S03
.........................................................................uuuggaaugCgaaauuuaugauuc..................   1     1    S03
.........................................................................uuuggaauguUaaauuuaugauuc..................   3     1    S03
.........................................................................uuugUaaugugaaauuuaugauuc..................   1     1    S03
.........................................................................uuuggaaugugaaauuuaugauuG..................   2     1    S03
.........................................................................uuuggaaugugaaauuuaugauucA.................   2     1    S03
..........................................................................uuggaaugugaaauuuaug......................   1     0    S03
..........................................................................uuggaaugGgaaauuuauga.....................   1     1    S03
..........................................................................uuggaaugugaaauuuaugUu....................   1     1    S03
..........................................................................uuggaaugugaaauuuaugau....................   4     0    S03
..........................................................................uuggaaugugaaauuuaugauC...................   3     1    S03
..........................................................................uuggaauguUaaauuuaugauu...................   1     1    S03
..........................................................................uuggaaugugaaauuuaugauu...................   25    0    S03
..........................................................................uuggaauguUaaauuuaugauuc..................   1     1    S03
..........................................................................uuggaaugugaaauuuaugaCuc..................   1     1    S03
..........................................................................uuggaaugugaaauuuaugauuc..................   14    0    S03
..........................................................................uuggaaugGgaaauuuaugauucu.................   1     1    S03
..........................................................................uuggaaugugaaauuuaugauucu.................   37    0    S03
..........................................................................Nuggaaugugaaauuuaugauucu.................   1     1    S03
..........................................................................uuggaaugugaaauuuaugauucA.................   4     1    S03
..........................................................................uuggaaugugaaauuuaugauucC.................   9     1    S03
..........................................................................uuggaaugugaaauuuaugauucuU................   1     1    S03
...........................................................................uggaaugugaaauuuaug......................   3     0    S03
...........................................................................uggaauguUaaauuuauga.....................   1     1    S03
...........................................................................uggaaugugaaauuuaugau....................   3     0    S03
...........................................................................uggaaugugaaauuuaugaC....................   1     1    S03
...........................................................................uggaaugugaaauuuaugauu...................   11    0    S03
...........................................................................uggaauguUaaauuuaugauu...................   1     1    S03
...........................................................................uggaaugugaaauuuaugauC...................   1     1    S03
...........................................................................ugUaaugugaaauuuaugauuc..................   1     1    S03
```

Star Mature

```
auuugauggguaauauguaaauaaaaaucauuguuauuuguauuuaucagaguucguaacuuauucugcuuuuuuuggaaugugaaauuuaugauucuaauaaauauauaaauaa
...........................................................................uggaaugugaaauuuaugGuuc..................   1    1    S03
...........................................................................uggaaugugaaauuuaugauuc..................   9    0    S03
...........................................................................uggaaugugaaauuuaugauucA.................   36   1    S03
...........................................................................uggaaugugaaauuuaugauucu.................   4    0    S03
...........................................................................uggaaugugaaauuuaugauucuU................   1    1    S03
...........................................................................uggaaugugaaauuuaugauucAa................   1    1    S03
............................................................................ggaaugugaaauuuaugauu...................   1    0    S03
............................................................................gUaaugugaaauuuaugauucua................   1    1    S03
.............................................................................Uaaugugaaauuuaugauucuaau..............   1    1    S03
.............................................................................gaaugugaaauuuaugauucuaau..............   1    0    S03
..............................................................................aaugugaaauuuaugauucuG................   1    1    S03
..............................................................................aaugugaaauuuaugauucuaau..............   1    0    S03
...............................................................................augugaaauuuaugauucuaaua.............   1    0    S03
...............................................................................augugaaauuuaugauucuaauaa............   1    0    S03
.........................................................................................uaugauucuaauaaauauauaaau..   1    0    S03
..........................................................................................augauucuaauaaauauauaaau..   1    0    S03

.....................................................................uuuuuuuggaaugugaaauuua........................   1    0    S04
......................................................................uuuuuuggaaugugaaauuuU........................   1    1    S04
......................................................................Nuuuuuggaaugugaaauuuau.......................   1    1    S04
......................................................................uuuuuuggaaugugaaauuuauA......................   3    1    S04
......................................................................uuuuuuggaaugugaaauuuaug......................   9    0    S04
......................................................................uuuuuuggaaugugaaauuuauga.....................   13   0    S04
......................................................................uuuuuuggaauguUaaauuuauga.....................   1    1    S04
......................................................................uuuuuuggaaugugaaauuuaugaG....................   1    1    S04
......................................................................uuuuuuggaaugAgaaauuuaugau....................   1    1    S04
......................................................................uuuuuuggaaugugaaauuuaugau....................   5    0    S04
.......................................................................uuuuuggaaugugaaauu..........................   1    0    S04
.......................................................................uuuuuggaaugugaaauuu.........................   2    0    S04
.......................................................................uuuuuggaaugugaaauuua........................   1    0    S04
.......................................................................uuuuuggaaugugaaauuuau.......................   1    0    S04
.......................................................................uuuuuggaaugugaaauuuauC......................   1    1    S04
.......................................................................uuuuuggaaugugaaauuuauU......................   3    1    S04
.......................................................................uuuuuggaaugugaaauuuaug......................   25   0    S04
.......................................................................uuuuuggaaugugaaauuuauga.....................   19   0    S04
.......................................................................uuuuuggaaugugaaauuuaugau....................   116  0    S04
.......................................................................uuuuuggaaugugaaauuuaugGu....................   2    1    S04
.......................................................................uuuuuggaaugugaaauuuauUau....................   2    1    S04
.......................................................................uuuuuggaaugugaaauuuaugaC....................   6    1    S04
.......................................................................uuuuuggaauguUaaauuuaugau....................   5    1    S04
.......................................................................uuuuuggaaugugaaauuuaugaG....................   3    1    S04
.......................................................................uuuuuggaaugugaaauuuaugauu...................   4    0    S04
.......................................................................uuuuuggaaugugaGauuuaugauu...................   1    1    S04
.......................................................................uuuuuggUaugugaaauuuaugauu...................   1    1    S04
........................................................................uuuuggaaugugaaauuu.........................   1    0    S04
........................................................................uuuuggaaugugaaauuua........................   1    0    S04
........................................................................uuuuggaaugugaaauuuau.......................   3    0    S04
........................................................................uuuuggaauguUaaauuuau.......................   1    1    S04
........................................................................uuuuggaaugugaaauuuaug......................   6    0    S04
........................................................................uuuuggaaugugaaauuuauga.....................   28   0    S04
........................................................................uuuuggaaugugaaauuuaugG.....................   2    1    S04
........................................................................uuuuggaaugugaaauuuaugC.....................   1    1    S04
........................................................................Cuuuggaaugugaaauuuaugau....................   1    1    S04
........................................................................uuuuggaaugugaaauuuaugau....................   47   0    S04
........................................................................uuuuggaauguUaaauuuaugau....................   2    1    S04
........................................................................uuuuggaaugugaaauuuaugaG....................   1    1    S04
........................................................................uuuuggaaugAgaaauuuaugau....................   1    1    S04
........................................................................uGuuggaaugugaaauuuaugau....................   1    1    S04
........................................................................uuuuggaaugugaaauuuaugaC....................   5    1    S04
........................................................................Guuuggaaugugaaauuuaugauu...................   1    1    S04
........................................................................uuuGggaaugugaaauuuaugauu...................   1    1    S04
........................................................................uuuuUgaaugugaaauuuaugauu...................   1    1    S04
........................................................................uuuuggaaugCgaaauuuaugauu...................   1    1    S04
........................................................................uuuuggaaugugaaauuuaugauA...................   1    1    S04
........................................................................uuuuggaaugugUaauuuaugauu...................   1    1    S04
........................................................................uuuuggaaGgugaaauuuaugauu...................   1    1    S04
........................................................................uuuuggaaugugaaauuuaugUuu...................   1    1    S04
........................................................................uuuuggaaugugaaauuuaugauC...................   19   1    S04
........................................................................uuuuggaaugugaaauuuaugaCu...................   2    1    S04
........................................................................uuuuggaaugugaaauuuaugauu...................   186  0    S04
```

Star Mature

```
auuugauggguaauauguaaauaaaaaucauuguuauuuguauuuaucagaguucguaacuuauucugcuuuuuuuggaaugugaaauuuaugauucuaauaaauauauaaauaa
........................................................................Cuuuggaaugugaaauuuaugauu...................   2     1    S04
........................................................................uuuuggaaugugaGauuuaugauu...................   2     1    S04
........................................................................uuuuggaauguUaaauuuaugauu...................   9     1    S04
........................................................................uuuuggaaugugaaauuuaugauG...................   2     1    S04
.........................................................................uuuggaaugugaaauuua........................   2     0    S04
.........................................................................uAuggaaugugaaauuuau.......................   1     1    S04
.........................................................................uuuggaaugugaaauuuau.......................   1     0    S04
.........................................................................uuuggaaugugaaauuuaug......................   10    0    S04
.........................................................................uuuggaauguUaaauuuauga.....................   1     1    S04
.........................................................................uuuggaaugugaaauuuaugG.....................   1     1    S04
.........................................................................uuuggaaugugaaauuuauga.....................   5     0    S04
.........................................................................uuuggaaugugaaUuuuaugau....................   1     1    S04
.........................................................................uuuggaGugugaaauuuaugau....................   2     1    S04
.........................................................................uuuggaaugugaaauuuaugau....................   17    0    S04
.........................................................................uuuggaaugAgaaauuuaugau....................   1     1    S04
.........................................................................uuuggaaugugaaauuuaugaA....................   1     1    S04
.........................................................................uuuggaaugugaaauuuaugaC....................   2     1    S04
.........................................................................uuuggaauguUaaauuuaugau....................   1     1    S04
.........................................................................uuuggaaugugaaUuuuaugauu...................   1     1    S04
.........................................................................uuuggaaugugUaauuuaugauu...................   1     1    S04
.........................................................................uuuggaaugCgaaauuuaugauu...................   1     1    S04
.........................................................................uuuggaaugugaaauuuaugauA...................   6     1    S04
.........................................................................uuuggaaugugaCauuuaugauu...................   1     1    S04
.........................................................................uuuggaaugugaaauuuaugauG...................   9     1    S04
.........................................................................uuuggaaugAgaaauuuaugauu...................   2     1    S04
.........................................................................uuuggaaugugaaauuuaugauu...................   240   0    S04
.........................................................................uuuggaauguUaaauuuaugauu...................   17    1    S04
.........................................................................uuuUgaaugugaaauuuaugauu...................   1     1    S04
.........................................................................uuuggaaugugaaauuuaugauC...................   24    1    S04
.........................................................................uuuggaaugugaaauuuaugaCu...................   1     1    S04
.........................................................................uuuggaauguCaaauuuaugauu...................   1     1    S04
.........................................................................uuuUgaaugugaaauuuaugauuc..................   1     1    S04
.........................................................................uuuggaauguUaaauuuaugauuc..................   1     1    S04
.........................................................................uuuggaGugugaaauuuaugauuc..................   2     1    S04
.........................................................................uuuggaaugugaaauuuaugauGc..................   1     1    S04
.........................................................................uuuggaaugugaaauuuaugauuG..................   1     1    S04
.........................................................................uuuggaaugugaaauuuaugauuc..................   93    0    S04
.........................................................................uuuggaaugugaaauuuaugauucA.................   1     1    S04
..........................................................................uuggaaugugaaauuuau.......................   1     0    S04
..........................................................................uuggaaugugaaauuuaug......................   2     0    S04
..........................................................................uAggaaugugaaauuuauga.....................   1     1    S04
..........................................................................uuggaaugugaaauuuauga.....................   3     0    S04
..........................................................................uuggaaugugaaauuuaugau....................   5     0    S04
..........................................................................uuggaaugugaaauuuaugUuu...................   1     1    S04
..........................................................................uuggaaugugaaauuuaugauC...................   3     1    S04
..........................................................................uuggaaugugaaauuuaugauG...................   1     1    S04
..........................................................................uuggaaugugaaauuuaugauu...................   26    0    S04
..........................................................................uAggaaugugaaauuuaugauuc..................   1     1    S04
..........................................................................uuggaaugugaaauuuaugGuuc..................   1     1    S04
..........................................................................uuggaaugugaaauuuaugaCuc..................   1     1    S04
..........................................................................uuggaaugugaaauuuaugauuG..................   2     1    S04
..........................................................................uuggaaugugaaauuuaugauuc..................   26    0    S04
..........................................................................uuggaaugugaaauuuaugauucu.................   41    0    S04
..........................................................................uuggaaugugaaauuuaugauucA.................   7     1    S04
..........................................................................Cuggaaugugaaauuuaugauucu.................   1     1    S04
..........................................................................uuggaaugugaaauuuaugauCcu.................   1     1    S04
..........................................................................uuggaaugugaaauuuaugauucC.................   6     1    S04
..........................................................................uuggaaugugaaauuuaugauucuU................   1     1    S04
..........................................................................uuggaaugugaaauuuaugauucuC................   1     1    S04
...........................................................................uggaaugugaaauuuaug......................   1     0    S04
...........................................................................uggaaugCgaaauuuauga.....................   1     1    S04
...........................................................................uggaaugugaaauuuauga.....................   2     0    S04
...........................................................................uggaaugugaaauuuaugau....................   7     0    S04
...........................................................................uggaaugugaaauuuaugaCu...................   1     1    S04
...........................................................................uggaaugugaaauuuaugauu...................   13    0    S04
...........................................................................uggaaugugaaauuuaugauC...................   1     1    S04
...........................................................................uggaauguUaaauuuaugauu...................   1     1    S04
...........................................................................uggaaugugaaauuuaugauuc..................   24    0    S04
...........................................................................uggaaugugaaauuuaugauucu.................   4     0    S04
...........................................................................uggaaugugaaauuuaugauucA.................   47    1    S04
```

Star Mature

```
auuugauggguaauauguaaauaaaaaucauuguuauuuguauuuaucagaguucguaacuuauucugcuuuuuuuggaaugugaaauuuaugauucuaauaaauauauaaauaa
...........................................................................uggaaugugaaauuuaugauucua................	3	0	S04
..............................................................................aaugugaaauuuaugauucuG................	1	1	S04
..............................................................................aaugugaaauuuaugauucuGa...............	1	1	S04
...............................................................................augugaaauuuaugauucuaaua.............	1	0	S04
.......................................................................................uuuaugauucuaauaaauauaua.....	1	0	S04
..........................................................................................augauucuaauaaUuauauaaau..	1	1	S04
..........................................................................................augauucuaauaaauauauaaau..	3	0	S04
```

Provisional ID	:	chrC02_1292332
Score total	:	3.2
Score for star read(s)	:	3.9
Score for read counts	:	-1.8
Score for mfe	:	1.2
Score for randfold	:	0
Score for cons. seed	:	
Total read count	:	8
Mature read count	:	7
Loop read count	:	0
Star read count	:	1

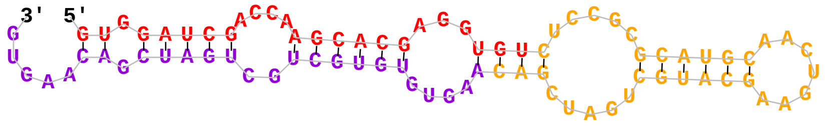

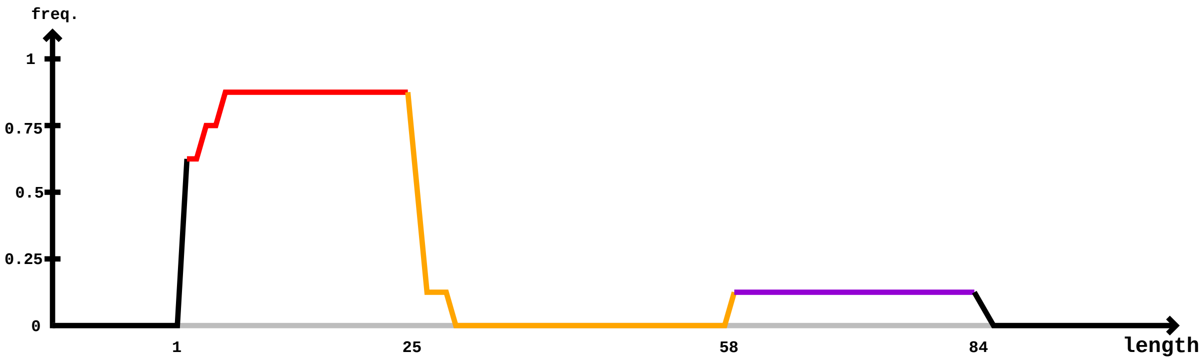


```
                Mature                                                Star
5'- cagaagcacaagguggaucgaccaagcacgaggugucuccgcgcaugcaacugaagcaugcugaucgacaagugugugcugcugaucgacaagugaguuccagucaugaagccug   -3'    obs
    cagaagcacaagguggaucgaccaagcacgaggugucuccgcgcaugcaacugaagcaugcugaucgacaagugugugcugcugaucgacaagugaguuccagucaugaagccug          exp
    ..(((.(((...((.(((((....((((((...((((.....((((((.......)))))).....))))....))))))..))))).))..)))..)))...............   reads     mm     sample
    ...........Uuggaucgaccaagcacgaggugu................................................................................   1         1      S04
    ............guggaucgaccaagcacgaggugu...............................................................................   3         0      S04
    ............guggaucgaccaagcacgaggugC...............................................................................   1         1      S04
    ..............ggaucgaccaagcacgaggugu...............................................................................   1         0      S04
    ................aucgaccaagcacgaggugucucc...........................................................................   1         0      S04
    .....................................................................aagugugugcugcugaucgacaaguA....................   1         1      S04
```

Provisional ID	:	chrC02_random_2810655
Score total	:	0.8
Score for star read(s)	:	-1.3
Score for read counts	:	0
Score for mfe	:	2.1
Score for randfold	:	0
Score for cons. seed	:	
Total read count	:	63
Mature read count	:	63
Loop read count	:	0
Star read count	:	0

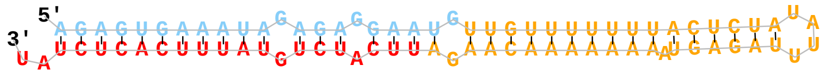

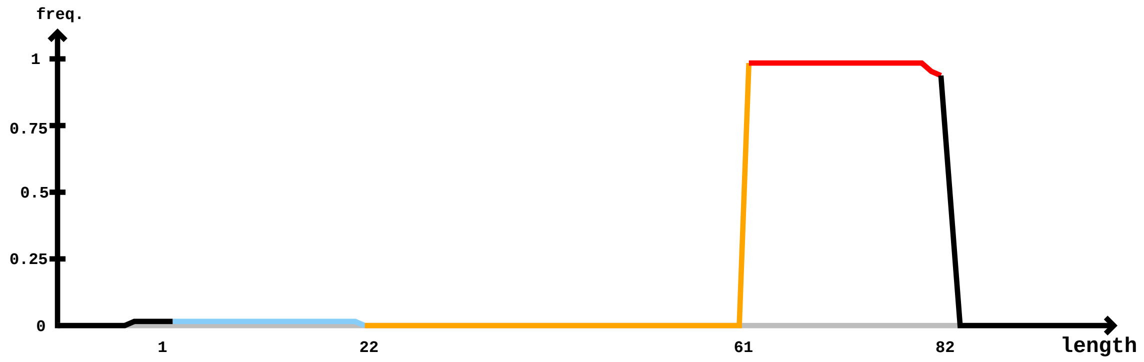

Star **Mature**

```
5'- uauauauaauagagugaaauagagaggaauguuguuuuuuuacucuauauuuagaguaaaaaaaaacaagauucaucuguauuucacucuauuauagaguagaccauua  -3'    exp
    ....((((((((((((((((.(((.((((.(((((((((((((((....)))))).))))))))).)))).))).)))))))))))))))))............  reads    mm    sample
    .......................................................................uucaucuguauuucacucG...................  1    1    S03
    .......................................................................uucaucuguauuucacucu...................  1    0    S03
    .......................................................................uCcaucuguauuucacucuau.................  1    1    S03
    .......................................................................uucaucuguauuucacucuau.................  18   0    S03
    .......................................................................uucGucuguauuucacucuau.................  1    1    S03
    .......................................................................Cucaucuguauuucacucuau.................  1    1    S03
    .......................................................................uuGaucuguauuucacucuau.................  1    1    S03
    .......................................................................uucaucuguauuucacCcuau.................  2    1    S03
    .......................................................................uucaucuguauuucacucuaC.................  5    1    S03

    ......uaauagagugaaauagagaggaau...............................................................................  1    0    S04
    .......................................................................uucaucuguauuucacucua..................  1    0    S04
    .......................................................................uucaucuguauuucacucuaG.................  1    1    S04
    .......................................................................uucaucuguauuucacucGau.................  1    1    S04
    .......................................................................uucaucuguauuucacucuaC.................  1    1    S04
    .......................................................................uucaucuguauuucacucuau.................  28   0    S04
```

Provisional ID	:	chrC02_random_2820048
Score total	:	0.4
Score for star read(s)	:	-1.3
Score for read counts	:	0
Score for mfe	:	1.7
Score for randfold	:	0
Score for cons. seed	:	
Total read count	:	104
Mature read count	:	104
Loop read count	:	0
Star read count	:	0

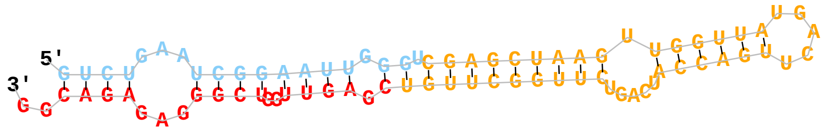

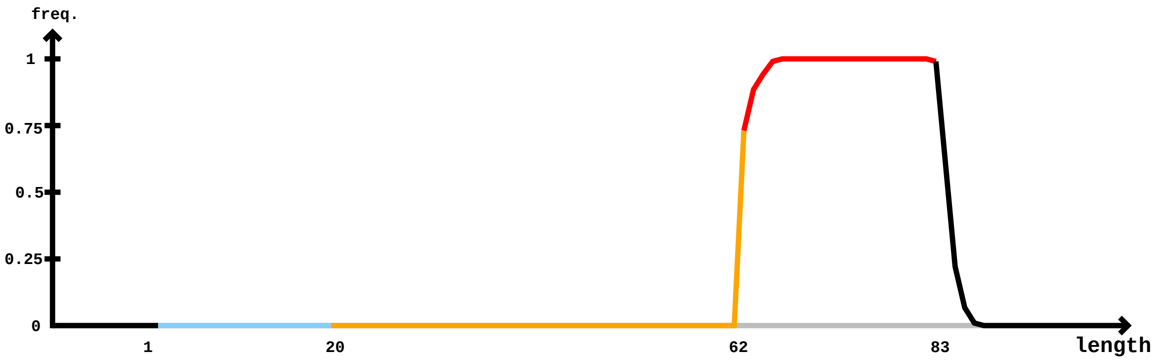

Star **Mature**

```
5'- gaagccauggucugaaucggaauugggucgagcuaaguugguuaugacuugaccaucagucuuggcuugucgaguuggucgggagagacgggcuucaguucggccaguuc   -3'    exp
    ((((((...((((...((((((((.((.(((((((((.((((((.....)))))).....))))))))))).))))..))))...)))).)))))).............   reads      mm        sample
    ......................................................................cgaguuggucgggaAagacgg...................   11         1         S03
    ......................................................................cgaguuggucgggaAagacggg..................   12         1         S03
    ......................................................................cgaguuggucgggaAagacgggc.................   1          1         S03
    ......................................................................cgaguuggucgggaAagacgggcu................   1          1         S03
    .......................................................................gaguuggucgggaAagacgg...................   2          1         S03
    .......................................................................gaguuggucgggaAagacggg..................   2          1         S03
    .......................................................................gaguuggucgggaAagacgggc.................   2          1         S03
    .........................................................................guuggucgggaAagacgg...................   1          1         S03
    .........................................................................guuggucgggaAagacgggcu................   1          1         S03

    ......................................................................cgaguuggucgggagagacg....................   1          0         S04
    ......................................................................cgaguuggucgggaAagacgg...................   31         1         S04
    ......................................................................cgaguuggucgggaAagacggg..................   17         1         S04
    ......................................................................cgaguuggucgggaAagacgggc.................   1          1         S04
    ......................................................................cgaguuggucgggaAagacgggcu................   1          1         S04
    .......................................................................gaguuggucgggaAagacggg..................   2          1         S04
    .......................................................................gaguuggucgggaAagacgggc.................   8          1         S04
    ........................................................................aguuggucgggaAagacggg..................   2          1         S04
    ........................................................................aguuggucgggaAagacgggc.................   4          1         S04
    .........................................................................guuggucgggaAagacgggcu................   3          1         S04
    ..........................................................................uuggucgggaAagacgggcuu...............   1          1         S04
```

Provisional ID	:	chrC03_1316826
Score total	:	0.8
Score for star read(s)	:	3.9
Score for read counts	:	-4.9
Score for mfe	:	1.9
Score for randfold	:	0
Score for cons. seed	:	
Total read count	:	2
Mature read count	:	1
Loop read count	:	0
Star read count	:	1

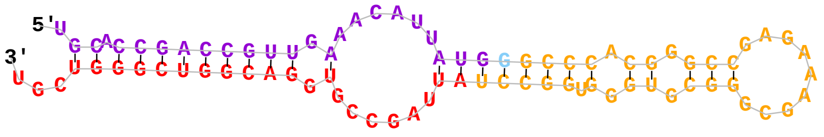

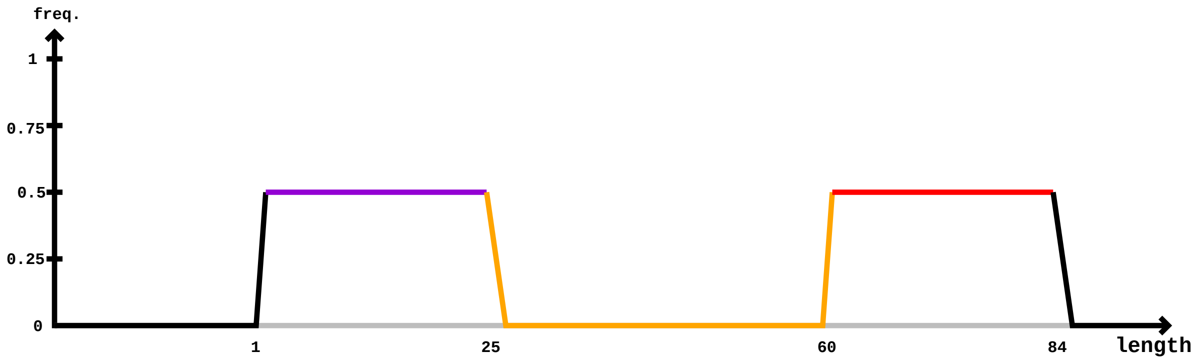


```
                       Star                                                       Mature
5'- uucgggccuugaaaaauuaaugcaccgaccguugaaacauuaugggcccacgggccgagaaagcgggcgugggguggccuauuagccguggacggucgggucguauauuuuccca   -3'    obs
    uucgggccuugaaaaauuaaugcaccgaccguugaaacauuaugggcccacgggccgagaaagcgggcgugggguggccuauuagccguggacggucgggucguauauuuuccca          exp
    ...(((......(((((..(((..(((((((((.(......((((((((.((.(((.........))).)).).))))))))......).)))))))))..)))..)))))))).   reads      mm          sample
    ....................ugcaccgaccguugaaacauuaug.......................................................................   1          0           S03

    ................................................................................auuagccguggacggucgggCcgu...........   1          1           S04
```

Provisional ID	:	chrC03_1328701
Score total	:	9.9
Score for star read(s)	:	3.9
Score for read counts	:	5.2
Score for mfe	:	0.8
Score for randfold	:	0
Score for cons. seed	:	
Total read count	:	22
Mature read count	:	13
Loop read count	:	0
Star read count	:	9

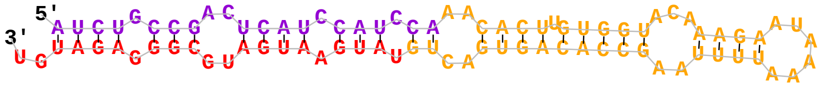

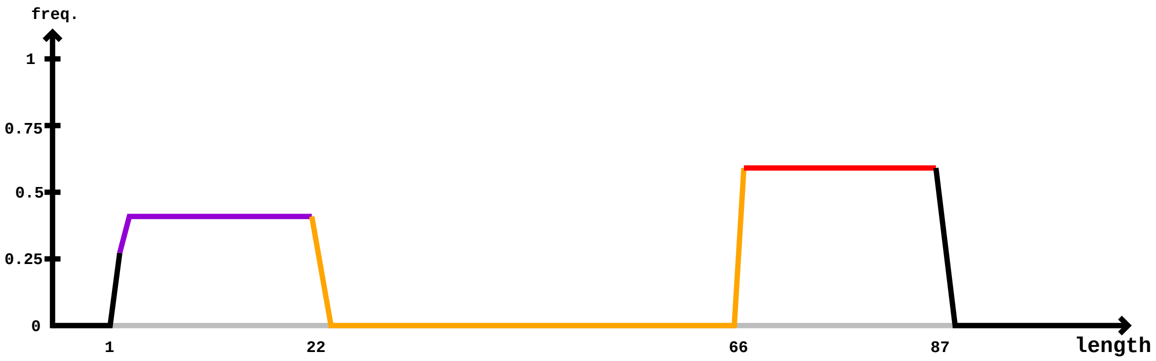


```
        Star                                                           Mature
5'- gaaauaucugccgacucauccauccaaacacuugugguacaaagaauaaaauuuuaagccacagugacuguaugaaugaugcgggagauguuuuuaccucuuuaucugug   -3'    obs
    gaaauaucugccgacucauccauccaaacacuugugguacaaagaauaaaauuuuaagccacagugacuguaugaaugaugcgggagauguuuuuaccucuuuaucugug          exp
    ((((((((.(((..((((.(((.((..((((.(((((...((((......))))..)))))))))..)).))).)))).))).)))))))))................  reads    mm      sample
    .....aucugccgacucauccauccG....................................................................................  1        1       S03
    .....aucugccgacucauccauccC....................................................................................  1        1       S03
    .....aucugccgacucauccaucca....................................................................................  1        0       S03

    .....aucugccgacucauccaucca....................................................................................  3        0       S04
    ......ucugccgacucauccaucca....................................................................................  1        0       S04
    ......ucugccgacucauccauccaU...................................................................................  1        1       S04
    ......ucugccgacucauccauccaC...................................................................................  1        1       S04
    .................................................................uaugaaugaugcgggagaugC........................  1        1       S04
    .................................................................uaugaaugaugcgggagaugu........................  12       0       S04
```

Provisional ID : chrC03_1343668
Score total : 104
Score for star read(s) : 3.9
Score for read counts : 105.2
Score for mfe : -4.9
Score for randfold : 0
Score for cons. seed :
Total read count : 218
Mature read count : 217
Loop read count : 0
Star read count : 1

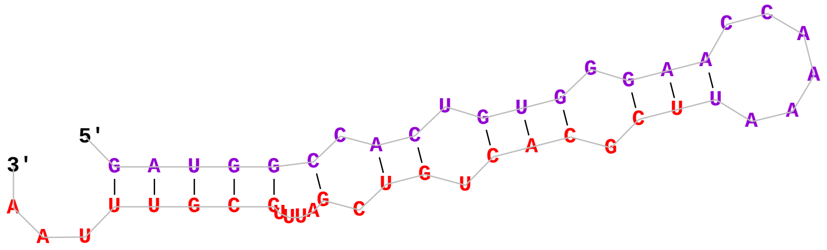

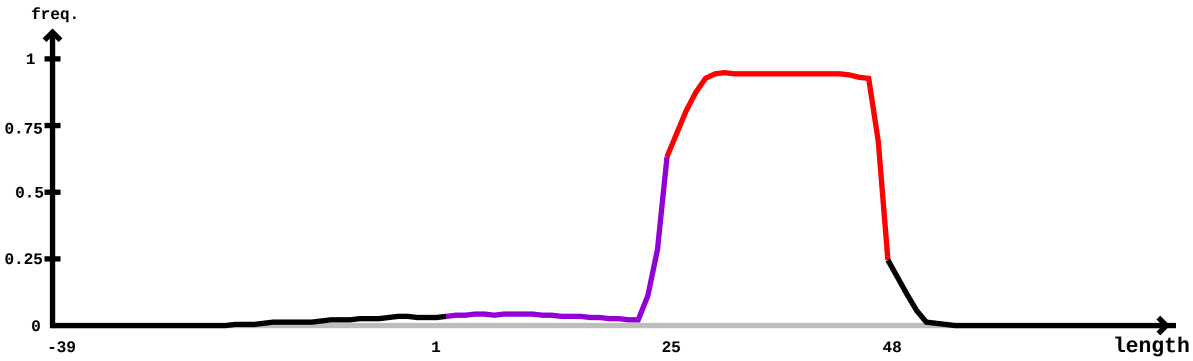

Star **Mature**

```
5'- aggugaaucuuucuccggagagagaauaugaaaaaauucugauggccacugugggaaccaaaauucgcacugucgauuuccguuuaaauaaagaaacuaggaaaacccuaauuuu   -3'   obs
    aggugaaucuuucuccggagagagaauaugaaaaaauucugauggccacugugggaaccaaaauucgcacugucgauuuccguuuaaauaaagaaacuaggaaaacccuaauuuu         exp
    .......(((((((....))))))).........(((..((((((.((.(((.(((......))).))).)).)....)))))..)))...((((.((((.....)))).))))   reads   mm   sample
    .....................gagaauaugaaaaaauucugauggc.....................................................................   1       0    S03
    ...............................aaaaauucugauggccacuguggg............................................................   1       0    S03
    ..................................aauucugauggccacugugggaa..........................................................   1       0    S03
    ........................................gauggccacugugggaaccaaaau...................................................   1       0    S03
    .........................................auggccacugugggaaccaaaauu..................................................   1       0    S03
    ..............................................cacugugggaaccaaaauucgc...............................................   1       0    S03
    .............................................................aauucgcacugucgauuuccguuu..............................   11      0    S03
    .............................................................aauucgcacugucgauuuccguuC..............................   1       1    S03
    .............................................................aauucgcacugucgauuuccguuua.............................   1       0    S03
    ..............................................................auucgcacugCcgauuuccguu...............................   1       1    S03
    ..............................................................auucgcacugCcgauuuccguuu..............................   1       1    S03
    ..............................................................auucgcacugucgauuuccguuu..............................   7       0    S03
    ..............................................................auucgcacugucgauuuccguuuG.............................   1       1    S03
    ..............................................................auucgcacugucgauuuccguuuU.............................   2       1    S03
    ..............................................................auucgcacugucgauuuccguuua.............................   8       0    S03
    ..............................................................auucgcacugucgauuuccguuuaa............................   1       0    S03
    ..............................................................auucgcacugucgauuuccguuuUa............................   2       1    S03
    ...............................................................uucgcacugucgauuuccguuC..............................   1       1    S03
    ...............................................................uucgcacugucgauuuccguuu..............................   10      0    S03
    ...............................................................uucgcacugucgauuuccguuuC.............................   1       1    S03
    ...............................................................uucgcacugucgauuuccguuua.............................   36      0    S03
    ...............................................................uucgcacugCcgauuuccguuua.............................   1       1    S03
    ...............................................................uucgcacugucgauuuccguuuG.............................   10      1    S03
    ...............................................................uucgcacugucgauuuccguuuaa............................   1       0    S03
    ...............................................................uucgcacugucgauuuccguuuUa............................   1       1    S03
    ................................................................ucgcacugucgauuuccC..................................   1       1    S03
    ................................................................ucgcacugucgauuuccguuC..............................   1       1    S03
    ................................................................ucgcacugucgauuuccguuu..............................   4       0    S03
    ................................................................ucgcacugucgauuuccguuua.............................   2       0    S03
    ................................................................ucgcacugucgauuuccguuuU.............................   2       1    S03
    ................................................................ucgcacugucgauuuccguuuUa............................   3       1    S03
    ................................................................ucgcacugucgauuuccguuuaa............................   3       0    S03
    ................................................................ucgcacugucgauuuccguuuaaa...........................   2       0    S03
```

Star **Mature**

```
aggugaaucuuucuccggagagagaauaugaaaaauucugauggccacugugggaaccaaaauucgcacugucgauuuccguuuaaauaaagaaacuaggaaaacccuaauuuu
................................................................cgcacugucgauuuccgC................................  2  1  S03
................................................................cgcacugucgauuuccguuu..............................  6  0  S03
................................................................cgcacugucgauuuccguuua.............................  3  0  S03
................................................................cgcacugucgauuuccguuuaaa...........................  1  0  S03
................................................................cgcacugucgauuuccguuuaaau..........................  2  0  S03
................................................................cgcacugucgauuuccguuuaaUu..........................  1  1  S03
.................................................................gcacugucgauuuccguuu..............................  2  0  S03
.................................................................gcacugucgauuuccguuua.............................  2  0  S03
.................................................................gcacugucgauuuccguuuaC............................  1  1  S03
.................................................................gcacugucgauuuccguuuaa............................  1  0  S03
.................................................................gcacugucgauuuccguuuaaa...........................  1  0  S03
.................................................................gcacugucgauuuccguuuaaau..........................  4  0  S03
.................................................................gcacugucgauuuccguuuaaaua.........................  2  0  S03
..................................................................cacugucgauuuccguuuaaa...........................  3  0  S03
..................................................................cacugucgauuuccguuuaaau..........................  3  0  S03
..................................................................cacugucgauuuccguuuaaaua.........................  4  0  S03
..................................................................cacugucgauuuccguuuaaauaa........................  1  0  S03
...................................................................acugucgauuuccguuuaaau..........................  1  0  S03
...................................................................acugucgauuuccguuuaaaua.........................  1  0  S03
....................................................................cugucgauuucUguuuaaauaaag......................  1  1  S03

.................gagagagaauaugaaaaGu..............................................................................  1  1  S04
....................agagaauaugaaaaauucugaugg......................................................................  1  0  S04
..........................uaugaaaaauucugauggccacu.................................................................  1  0  S04
...........................augaaaaauucugauggccacugu...............................................................  1  0  S04
..................................auucugauggccacugugggaacc........................................................  1  0  S04
..........................................ggccacugugggaaccaaaauucg................................................  1  0  S04
.............................................cacugugggaaccaaaauucgcaU.............................................  1  1  S04
............................................................aauucgcacugucgauuuccguuu..............................  6  0  S04
............................................................aauucgcacugucgauuuccguuuG.............................  1  1  S04
............................................................aauucgcacugucgauuuccguuuaa............................  1  0  S04
.............................................................auucgcacugucgauuuccguuu..............................  3  0  S04
.............................................................auucgcacugucgauuuccguuC..............................  1  1  S04
.............................................................auucgcacugucgauuuccguuua.............................  11  0  S04
.............................................................auucgcacugucgauuuccguuuU.............................  2  1  S04
..............................................................uucgcacugucgauuuccCuuua.............................  1  1  S04
..............................................................uucgcacugucgauuuccguuuC.............................  1  1  S04
..............................................................uucgcacugucgauuuccguuua.............................  11  0  S04
..............................................................uucgcacugucgauuuccguuuG.............................  5  1  S04
..............................................................uucgcacugucgauuuccguuuaa............................  2  0  S04
...............................................................ucgcacugucgauuuccguuuU.............................  2  1  S04
...............................................................ucgcacugucgauuuccguuuUa............................  2  1  S04
................................................................cgcacugucgauuuccguuu..............................  1  0  S04
................................................................cgcacugucgauuuccguuuaa............................  1  0  S04
................................................................cgcacugucgauuuccguuuaU............................  1  1  S04
................................................................cgcacugucgauuuccguuuaaa...........................  1  0  S04
................................................................cgcacugucgauuuccguuuaaG...........................  1  1  S04
.................................................................gcacugucgauuuccguuuaaau..........................  1  0  S04
.................................................................gcacugucgauuuccguuuaGau..........................  1  1  S04
.................................................................gcacugucgauuuccguuuaaaua.........................  1  0  S04
..................................................................cacugucgauuuccguuuaa............................  1  0  S04
..................................................................cacugucgauuuccguuuaaau..........................  1  0  S04
...................................................................acugucgauuuccguuuaaaua.........................  2  0  S04
...................................................................acugucgauuuccguuuaaauaUa.......................  1  1  S04
```

Provisional ID	:	chrC03_1344684
Score total	:	0.3
Score for star read(s)	:	3.9
Score for read counts	:	-4.4
Score for mfe	:	0.8
Score for randfold	:	0
Score for cons. seed	:	
Total read count	:	3
Mature read count	:	1
Loop read count	:	1
Star read count	:	1

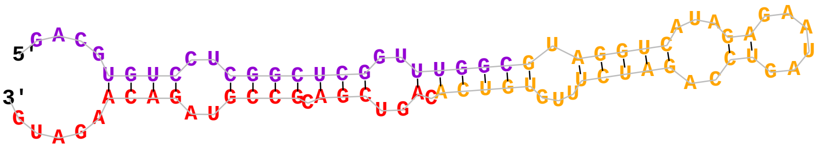

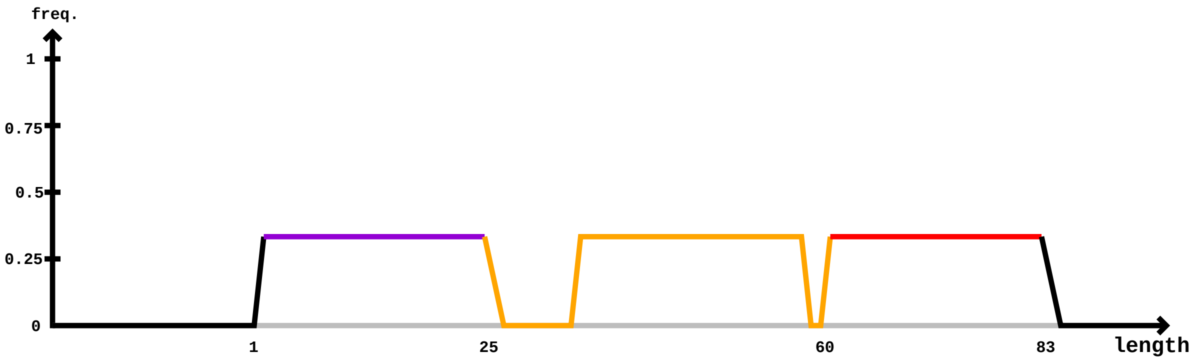

Star   Mature

```
5'- agcuguugacgaagcugcuugacguguccucggcucgguuuggcguaggucauagagaauaguccagaucuuugugucacagucgacgccguagacaagauggguugaacggua   -3'    obs
    agcuguugacgaagcugcuugacguguccucggcucgguuuggcguaggucauagagaauaguccagaucuuugugucacagucgacgccguagacaagauggguugaacggua          exp
    .((((((.(.......(((((.(.((((..(((((((..((((((.(((((...((......))..)))))...))))).)..))).))))..)))).).)))))).)))))).   reads    mm    sample
    ....................gacguguccucggcucgguuuggc......................................................................   1        0     S04
    .......................................................agagaauaguccagaucuuugugu...................................   1        0     S04
    ...............................................................................cagucgacgccguagacaagaug............   1        0     S04
```

Provisional ID	:	chrC03_1354444
Score total	:	716.9
Score for star read(s)	:	3.9
Score for read counts	:	714.4
Score for mfe	:	-1.3
Score for randfold	:	0
Score for cons. seed	:	
Total read count	:	1413
Mature read count	:	1406
Loop read count	:	0
Star read count	:	7

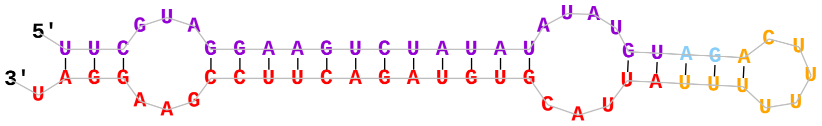

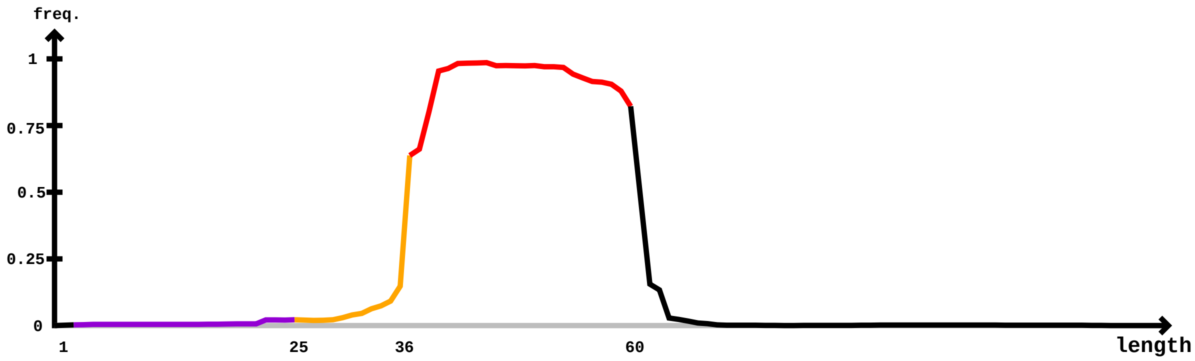

Star **Mature**

```
5'- uucguaggaagucuauauauauguagacuuuuuuuauuacguguagacuuccgaaggauaaaaacguaaaaauauauuucuguuuuuuuuguuuggucauaaagguuaaauagua   -3'    obs
    uucguaggaagucuauauauauguagacuuuuuuuauuacguguagacuuccgaaggauaaaaacguaaaaauauauuucuguuuuuuuuguuuggucauaaagguuaaauagua          exp
    (((...((((((((((((....(((((.....)))))...))))))))))))...))).(((((((.(((.....))).))))))).(((((((..(.....)..))))))).. reads  mm  sample
    .ucguaggaagucuauauauaugua.......................................................................................... 1      0   S03
    ..cguaggaagucuauauauaugu........................................................................................... 1      0   S03
    ....................auguagacuuuuuuuauuacgugu....................................................................... 3      0   S03
    ....................auguagacuuuuuuuauuaAgugu....................................................................... 1      1   S03
    ....................auguagacuuuuuuuauuaUgugu....................................................................... 2      1   S03
    .......................uagacuuuuuuuauuacguguaga.................................................................... 1      0   S03
    .........................gacuuuuuuuauuacguguagacu.................................................................. 1      0   S03
    ...........................cuuuuuuuauuacguguagacuuA................................................................ 1      1   S03
    ............................uuuuuuuauuacguguagacuucc............................................................... 1      0   S03
    ............................uuuuuuuauuacguguagacuucU............................................................... 1      1   S03
    .............................uuuuuuauuacguguagacu.................................................................. 1      0   S03
    .............................uuuuuuauuacguguagacuucc............................................................... 2      0   S03
    .............................uuuuuuauuacguguagacuuccg.............................................................. 1      0   S03
    .............................Auuuuuauuacguguagacuuccg.............................................................. 2      1   S03
    ..............................uuuuuauuacguguagacuuccg.............................................................. 1      0   S03
    ..............................Nuuuuauuacguguagacuuccg.............................................................. 1      1   S03
    ..............................uuuuuauuacguguagacuuccga............................................................. 1      0   S03
    ..............................uuuuuauuacguguagacuuccgU............................................................. 1      1   S03
    ...............................uuuuauuacguguagacu.................................................................. 3      0   S03
    ...............................uuuuauuacguguagacuucc............................................................... 6      0   S03
    ...............................uuuuauuacguguagacuucA............................................................... 1      1   S03
    ...............................uuuGauuacguguagacuucc............................................................... 1      1   S03
    ...............................uuuuauuacguguagacuuccgU............................................................. 1      1   S03
    ................................uuuauuacguguagacuucc............................................................... 2      0   S03
    ................................uuuauuacguguagacuuccg.............................................................. 1      0   S03
    ................................uuuauuacguguagacuuccgC............................................................. 1      1   S03
    ................................uuuauuacguguagacuuccga............................................................. 1      0   S03
    ................................uuuaCuacguguagacuuccgaag........................................................... 1      1   S03
    .................................uuauuacguguagacuuccg.............................................................. 2      0   S03
    .................................uuauuacguguagacuuccgaagg.......................................................... 4      0   S03
    ..................................uauuacguguagacCuccg.............................................................. 1      1   S03
    ..................................uauuacguguagacuuccgaagg.......................................................... 2      0   S03
    ..................................uauuacguguagacuuccgaagga......................................................... 10     0   S03
```

Star **Mature**

```
uucguaggaagucuauauauauguagacuuuuuuauuacguguagacuuccgaaggauaaaaacguaaaauauauuucuguuuuuuuuguuuggucauaaagguuaaauagua
..................................auuacguguagacuuccga...........................................................  1    0  S03
..................................auuacguguagacuuccgU...........................................................  1    1  S03
..................................auuacguguagacuuccgaagg........................................................  1    0  S03
..................................auuacguguagacCuccgaagg........................................................  1    1  S03
..................................auuacguguagacuuccgaagga.......................................................  1    0  S03
..................................auuacguguagacuuccgaGggau......................................................  1    1  S03
..................................auuacAuguagacuuccgaaggau......................................................  1    1  S03
..................................auuacguguagacuuccgaaggau......................................................  164  0  S03
..................................auuacguguagacuuccgGaggau......................................................  2    1  S03
..................................auuacguguagacuuccgaaggaA......................................................  5    1  S03
..................................auuacguguaUacuuccgaaggau......................................................  1    1  S03
..................................auuacguguagacuCccgaaggau......................................................  1    1  S03
..................................auuacguguagacuuccgaaggaG......................................................  13   1  S03
..................................auuacguguGgacuuccgaaggau......................................................  1    1  S03
..................................auuacgGguagacuuccgaaggau......................................................  1    1  S03
..................................auuacguguagacuuccUaaggau......................................................  1    1  S03
..................................auuacguguagacuuccgaaggaC......................................................  24   1  S03
..................................Nuuacguguagacuuccgaaggau......................................................  1    1  S03
..................................auCacguguagacuuccgaaggau......................................................  1    1  S03
..................................Cuuacguguagacuuccgaaggau......................................................  1    1  S03
..................................auuacguguagacuuccgaaggaua.....................................................  2    0  S03
..................................auuacguguagacuuccgaaggauU.....................................................  1    1  S03
..................................auuacguguagacuuccgaaggauG.....................................................  1    1  S03
...................................uuacguguagacuuccgaaggGu......................................................  1    1  S03
...................................uuacguguagacuuccgaaggau......................................................  4    0  S03
...................................uuacguguagacuuccgaaggaCa.....................................................  1    1  S03
...................................uuacguguagacuuccgaaggauU.....................................................  1    1  S03
...................................uuacguguagacuuccgaaggauG.....................................................  1    1  S03
....................................uacguguagacuuccgaa..........................................................  1    0  S03
....................................uacguguagUcuuccgaaggau......................................................  1    1  S03
....................................uacguguagacuuccgaaggGu......................................................  1    1  S03
....................................uacguguagacuuccgaaggau......................................................  3    0  S03
....................................uacguguagGcuuccgaaggaua.....................................................  1    1  S03
....................................uacguguagacuuccgaaggauU.....................................................  1    1  S03
....................................uacguguagacuAccgaaggaua.....................................................  1    1  S03
....................................uacguguagacuuccgaGggaua.....................................................  1    1  S03
....................................uacguguagacCuccgaaggaua.....................................................  1    1  S03
....................................uacguguagacuuccgaaggaua.....................................................  40   0  S03
....................................uacguguagacuuccgaaggauG.....................................................  23   1  S03
....................................uacguguagacuuccgaaggGua.....................................................  1    1  S03
....................................uacguguagacuuccgaaggCua.....................................................  1    1  S03
....................................uacguguagacuuccgaaggauaa....................................................  3    0  S03
.....................................acguguagacuuccgaagga.......................................................  3    0  S03
.....................................acguguagaUuuccgaaggau......................................................  1    1  S03
.....................................acguguagacuuccgaaggUu......................................................  1    1  S03
.....................................acguguagacuuccgaaggau......................................................  10   0  S03
.....................................acguguagacuuccgaaggaua.....................................................  8    0  S03
.....................................acguguagacuuccgaaggauU.....................................................  1    1  S03
.....................................acgCguagacuuccgaaggaua.....................................................  1    1  S03
.....................................acguguagacuuccgaaggauaa....................................................  3    0  S03
.....................................acguguagacuuccgaaUgauaa....................................................  1    1  S03
.....................................acguguagacuuccgaagAauaaa...................................................  1    1  S03
.....................................acguguagacuuccgaaggauaaa...................................................  39   0  S03
.....................................acguguagacuuccgaaggauaUa...................................................  1    1  S03
.....................................acguguagUcuuccgaaggauaaa...................................................  1    1  S03
.....................................acguguagacuuccgaaggauaaU...................................................  1    1  S03
.....................................acguguagacuuccgaaggauaaaa..................................................  1    0  S03
......................................cguguagacuuccgaaggauG.....................................................  1    1  S03
......................................cguguagacuuccgaaggauaaa...................................................  1    0  S03
......................................cguguagacuuccgaagAauaaa...................................................  1    1  S03
......................................cguguagacuuccgaaggauaaaa..................................................  2    0  S03
.......................................guguagacuuccgaagga.......................................................  1    0  S03
.......................................guguagacuuccgaaggau......................................................  1    0  S03
.......................................guguagacuuccgaaggauaaaaa.................................................  2    0  S03
.......................................guguagacuuccgaaggauaaaaac................................................  2    0  S03
.......................................guguagacuuccgaaggauaaaaacA...............................................  1    1  S03
........................................uguagacuuccgaaggauaaaaac................................................  1    0  S03
..........................................uagacuuccgaagAauaaaaacgu..............................................  1    1  S03
...............................................uuccgaaggauaaaaacguaaaau.........................................  1    0  S03

uucguaggaagucuauauauau..........................................................................................  1    0  S04
```

Star   Mature

```
uucguaggaagucuauauauauguagacuuuuuuuauuacguguagacuuccgaaggauaaaaacguaaaauauauuucuguuuuuuuuguuuggucauaaagguuaaauagua
uucguaggaagucuauauauaugC.......................................................................................... 1 1 S04
uucguaggaagucuauauauaugu.......................................................................................... 2 0 S04
..cguaggaagucuauauauaugua......................................................................................... 1 0 S04
..............auauauGuguagacuuuuuu................................................................................ 1 1 S04
................auauauguagacuuuuuuuauuac.......................................................................... 1 0 S04
.................uauauguagacuuuuuuuauua........................................................................... 1 0 S04
....................auguagacuuuuuuuauuacgu........................................................................ 1 0 S04
....................auguagacuuuuuuuauuacgug....................................................................... 1 0 S04
....................auguagacuuuuuuuauuacgugG...................................................................... 2 1 S04
....................auguagacuuuuuuuauuacgugu...................................................................... 10 0 S04
....................auguagacuuGuuuuauuacgugu...................................................................... 3 1 S04
.......................uagacuuuuuuuauuacguguag.................................................................... 1 0 S04
.........................gacuuuuuuuauuacguguagacu................................................................. 1 0 S04
..........................acuuuuuuuauuacguguagacuu................................................................ 1 0 S04
...........................cuuuuuuuauuacguguagacuuc............................................................... 2 0 S04
............................uuuuuuuauuacguguagacuucA.............................................................. 1 1 S04
............................uuuuuuuauuacguguagaUuucc.............................................................. 1 1 S04
............................uuuuuuuauuacguguagacuucc.............................................................. 5 0 S04
............................uuuuuuuauuacguguagacuucU.............................................................. 1 1 S04
............................uuuuuuuauuacguguagGcuucc.............................................................. 1 1 S04
............................Guuuuuuauuacguguagacuuccg............................................................. 1 1 S04
.............................uuuuuuauuacguguagacuucc.............................................................. 4 0 S04
.............................uuuuuuUuuacguguagacuucc.............................................................. 1 1 S04
.............................uuuuuuauuacguguagacuucU.............................................................. 1 1 S04
.............................uGuuuuauuacguguagacuucc.............................................................. 1 1 S04
.............................Auuuuuauuacguguagacuuccg............................................................. 1 1 S04
.............................uuuuuuauuacguguagacuuccg............................................................. 2 0 S04
..............................uuuuuauuacguguagacuucc.............................................................. 1 0 S04
..............................uuuuuauuacguguagacuucU.............................................................. 1 1 S04
..............................Nuuuuauuacguguagacuuccg............................................................. 1 1 S04
..............................uuuuuauuacguguagacuuccgU............................................................ 1 1 S04
..............................Nuuuuauuacguguagacuuccga............................................................ 1 1 S04
...............................uuuuauuacguguagacu................................................................. 1 0 S04
...............................uuuuauuacguguagacuuc............................................................... 1 0 S04
...............................Auuuauuacguguagacuucc.............................................................. 1 1 S04
...............................uuuuauuacguguagacuucc.............................................................. 6 0 S04
...............................uuuuauuacguguagacuuccg............................................................. 2 0 S04
...............................uuuuauuacguguagacuuccgC............................................................ 1 1 S04
...............................uuuuauuacguguagacuuccga............................................................ 3 0 S04
................................uuuauuacguguagacuuccga............................................................ 1 0 S04
................................uuuauuacguguagacuuccgU............................................................ 1 1 S04
................................uuuauuacguguagacuuccgaa........................................................... 1 0 S04
................................uuuauuacguguagacuuccgaag.......................................................... 6 0 S04
................................Auuauuacguguagacuuccgaag.......................................................... 1 1 S04
................................uuuauuacAuguagacuuccgaagg......................................................... 1 1 S04
.................................uuauuacguguagacuuccg............................................................. 1 0 S04
.................................uuauuacguguagacuuccga............................................................ 3 0 S04
.................................Guauuacguguagacuuccga............................................................ 1 1 S04
.................................uuauuacguguagacuuccgaagg......................................................... 15 0 S04
.................................uuaCuacguguagacuuccgaagg......................................................... 1 1 S04
.................................uuauuacgCguagacuuccgaagg......................................................... 1 1 S04
..................................uauuacguguagacuuccU............................................................. 1 1 S04
..................................uauuacguguagacCuccg............................................................. 2 1 S04
..................................uauuacguguagGcuuccgaagg......................................................... 1 1 S04
..................................uauuacguguagacuuccgaagg......................................................... 4 0 S04
..................................uauuacguguagacuuccgaagga........................................................ 58 0 S04
..................................uauuacguguagacCuccgaagga........................................................ 1 1 S04
..................................Cauuacguguagacuuccgaagga........................................................ 1 1 S04
..................................uauuacguguagacuuccgaaggG........................................................ 6 1 S04
..................................uauCacguguagacuuccgaagga........................................................ 1 1 S04
...................................auuacguguagacuuccg............................................................. 2 0 S04
...................................auuacguguagacuuccgU............................................................ 2 1 S04
...................................auuacguguagacuuccgaa........................................................... 2 0 S04
...................................auuacguguagacuuccgaaC.......................................................... 1 1 S04
...................................auuacguguagacuuccgaag.......................................................... 2 0 S04
...................................auuacguguagacuuccgaagg......................................................... 8 0 S04
...................................auuacguguagacuuccgaagga........................................................ 6 0 S04
...................................auuacgCguagacuuccgaaggau....................................................... 1 1 S04
...................................auuacgugCagacuuccgaaggau....................................................... 1 1 S04
...................................auuacguguagacuuccgaaggaG....................................................... 15 1 S04
```

Star **Mature**

```
uucguaggaagucuauauauauguagacuuuuuuuauuacguguagacuuccgaaggauaaaaacguaaaauauauuucuguuuuuuuuguuuggucauaaagguuaaauagua
...................................auuacguguagacuuccgaaggGu.......................................................   5     1   S04
...................................auuacguguagacuuccgaaggaA.......................................................   4     1   S04
...................................auuacguguagacuuccgaaggau.......................................................   399   0   S04
...................................auuacAuguagacuuccgaaggau.......................................................   1     1   S04
...................................auuacguguagacuuccgaaggaC.......................................................   48    1   S04
...................................auCacguguagacuuccgaaggau.......................................................   2     1   S04
...................................auuacguguagGcuuccgaaggau.......................................................   2     1   S04
...................................aGuacguguagacuuccgaaggau.......................................................   1     1   S04
...................................Nuuacguguagacuuccgaaggau.......................................................   3     1   S04
...................................Cuuacguguagacuuccgaaggau.......................................................   1     1   S04
...................................auuacguguagacuCccgaaggau.......................................................   2     1   S04
...................................auuacguguagacuuccgaCggau.......................................................   1     1   S04
...................................Guuacguguagacuuccgaaggau.......................................................   2     1   S04
...................................auuacguguaUacuuccgaaggau.......................................................   1     1   S04
...................................auuacguguGgacuuccgaaggau.......................................................   2     1   S04
...................................auuacguguagacuuccgGaggau.......................................................   2     1   S04
...................................auuacguguaCacuuccgaaggau.......................................................   1     1   S04
...................................auuacguguagacuuccgaGggau.......................................................   1     1   S04
...................................auuacguguagacuuccgaaggaua......................................................   5     0   S04
...................................auuacguguagacuuccgUaggaua......................................................   1     1   S04
...................................auuacguguagacuuccgaaggauG......................................................   1     1   S04
...................................auuacguguagacuuccgaaggauU......................................................   9     1   S04
...................................auuacguguagacuuccgaaggaCa......................................................   1     1   S04
...................................auuacguguagacuuccgaaggauaU.....................................................   1     1   S04
....................................uuacguguagacuuccgaagg.........................................................   1     0   S04
....................................uuacguguagacuuccgaagga........................................................   1     0   S04
....................................uuacguguagacuuccgaaggaC.......................................................   1     1   S04
....................................uuacguguagacuuccgaaggau.......................................................   7     0   S04
....................................uuacguguagacuuccgaaggauG......................................................   2     1   S04
....................................uuacguguagacuuccgaaggauU......................................................   2     1   S04
....................................Cuacguguagacuuccgaaggaua......................................................   1     1   S04
....................................uuacguguagacuuccgaaggauC......................................................   1     1   S04
....................................uuacguguagacuuccgaaggGua......................................................   1     1   S04
....................................uuacguguagacuuccgaaggaua......................................................   12    0   S04
.....................................uacguguagacuuccgaaggaG.......................................................   1     1   S04
.....................................uacguguagacuuccgGaggau.......................................................   1     1   S04
.....................................uacguguagacuuccgaaggau.......................................................   13    0   S04
.....................................uacguguagacuuccgaaggaC.......................................................   2     1   S04
.....................................uacUuguagacuuccgaaggaua......................................................   1     1   S04
.....................................uacguguagacuuccgaaggauU......................................................   7     1   S04
.....................................uacguguagacuuccgaaggauG......................................................   24    1   S04
.....................................Gacguguagacuuccgaaggaua......................................................   2     1   S04
.....................................uacguguagacuuccgaaggaua......................................................   72    0   S04
.....................................uacguguagacuGccgaaggaua......................................................   1     1   S04
.....................................uacguguagacuuccgaaggauC......................................................   3     1   S04
.....................................uacguAuagacuuccgaaggaua......................................................   1     1   S04
.....................................uacguguagacuuccgaaggGua......................................................   1     1   S04
.....................................uacguguagGcuuccgaaggaua......................................................   1     1   S04
.....................................uacguguagacuuccgaaggauaa.....................................................   8     0   S04
.....................................uacguguagacuuccgaaggauUa.....................................................   1     1   S04
.....................................uacguguagacuuccgaaggauaU.....................................................   1     1   S04
......................................acguguagacuuccgaag..........................................................   1     0   S04
......................................acguguagacuuccgaaggau.......................................................   18    0   S04
......................................acguguagacuuccgaaggaC.......................................................   3     1   S04
......................................acguguagacuuccgaaggauC......................................................   1     1   S04
......................................acguguagacuuccgaaggauG......................................................   2     1   S04
......................................acguguagacuuccgaaggauU......................................................   1     1   S04
......................................acguguagacuuccgaaggaua......................................................   8     0   S04
......................................acguguagacuuccgaaggauaU.....................................................   1     1   S04
......................................acguguagacuuccgaaggGuaa.....................................................   1     1   S04
......................................acguguagacuuccgaaggauaa.....................................................   11    0   S04
......................................acguguagacuuccgaaggauGa.....................................................   1     1   S04
......................................acguguagacuuccgaaggauaaU....................................................   1     1   S04
......................................acguguagacuuccgaGggauaaa....................................................   2     1   S04
......................................acguguagacuuccgaaggaAaaa....................................................   1     1   S04
......................................acguguagacuuccgGaggauaaa....................................................   1     1   S04
......................................acguguagacuuccgaagAauaaa....................................................   1     1   S04
......................................acguguagacuuccgaaggGuaaa....................................................   1     1   S04
......................................acguguagacuuccgaaggUuaaa....................................................   1     1   S04
......................................acguguagacuuccgaaggauaaa....................................................   105   0   S04
```

Star **Mature**

uucguaggaagucuauauauauguagacuuuuuuuauuacguguagacuuccgaaggauaaaaacguaaaauauauuucuguuuuuuuuguuuggucauaaagguuaaauagua			
......................................acguguagacuuccgaaggauaaG..	1	1	S04
......................................acgCguagacuuccgaaggauaaa..	1	1	S04
......................................acguguagacuuccgaaggauaCa..	1	1	S04
....cguguagacuuccgaaggaC...	1	1	S04
....cguguagacuuccgaaggaua..	2	0	S04
....cguguagacuuccgaaggauaa...	1	0	S04
....cguguagacuuccgaaggauaaa..	3	0	S04
....cguguagacuuccgaaggauaaaa...	4	0	S04
....guguagacuuccgaaggauU..	1	1	S04
....guguagacuuccgaaggaua..	3	0	S04
....guguagacuuccgaaggauaa...	1	0	S04
....guguagacuuccgaaggauaaaaG..	2	1	S04
....guguagacuuccgaaggauUaaaa..	1	1	S04
....guguagacuuccgaaggauaaaaa..	5	0	S04
....guguagacuuccgaaggauaaaaac...	7	0	S04
....guguagacuuccgaaggauaaaaGc...	1	1	S04
....guguagacuuccgaaggauaaaaacA..	1	1	S04
....guguagacuuccgaaggauaaaaacgC...	1	1	S04
....uguagacuuccgaaggauaaaa...	1	0	S04
....guagacuuccgaaggauaaaaacg..	2	0	S04
....uagacuuccgaaggauaaaaacgu...	1	0	S04
....uagacuuccgaaggauaaaaacgG...	1	1	S04
....agacuuccgaaggauaaaaacgu...	2	0	S04
....agacuuccgaaggauaaaaacguG..	1	1	S04
....gacuuccgaaggauaaaaacguG..	1	1	S04
....uuccgaaggauaaaaacgu...	1	0	S04
....ccgaaggauaaaaacguaaaauau..	1	0	S04
....uucuguuuuuuuuguuugguc.................	1	0	S04
....uuuuuuuguuuggucauaaagguC........	1	1	S04
....uuuuuguuuggucauaaagguuaa......	1	0	S04

Provisional ID	:	chrC03_1357428
Score total	:	1
Score for star read(s)	:	-1.3
Score for read counts	:	0
Score for mfe	:	2.3
Score for randfold	:	0
Score for cons. seed	:	
Total read count	:	617
Mature read count	:	461
Loop read count	:	8
Star read count	:	148

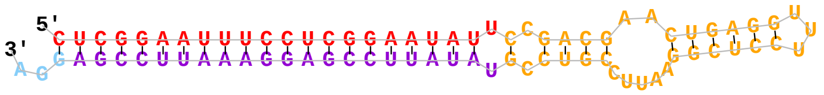

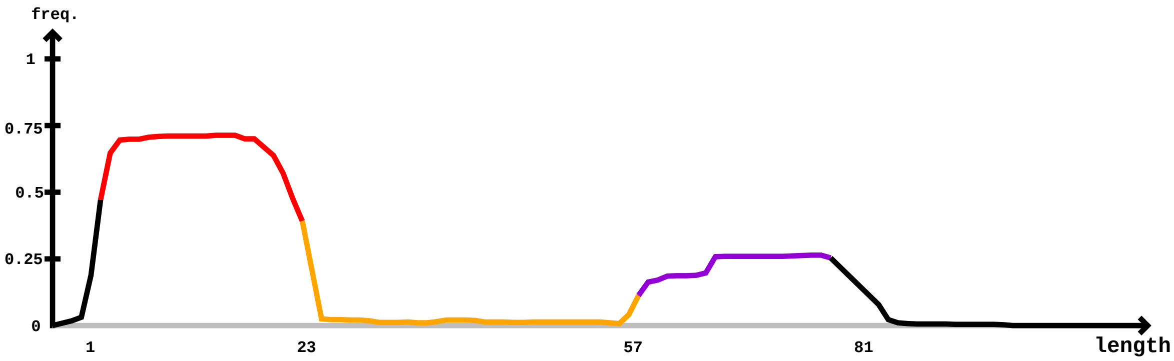

Mature **Star**

```
5'- uuccucggaauuuccucggaauauuccgacgaacugagguuuccucggaauuccguccguauauuccgaggaaauuccgaggaaccccaauuuuguguuaccucggaauuuc  -3'   obs
    uuccucggaauuuccucggaauauuccgacgaacugagguuuccucggaauuccguccguauauuccgaggaaauuccgaggaaccccaauuuuguguuaccucggaauuuc         exp
    ((((((((((((((((((((((((.(.((((..((((((...)))))).....)))).).))))))))))))))))))))))))....(((((((........)))))))..   reads    mm    sample
    uuccucggaauuuccucg..............................................................................................   5        0     S03
    .uccucggaauuuccucggaa...........................................................................................   1        0     S03
    .uccucggaauuuccucggaaua.........................................................................................   1        0     S03
    .uccucggaauuuccucggaauauC.......................................................................................   1        1     S03
    ..ccucggaauuuccucgga............................................................................................   7        0     S03
    ..ccucggaauuuccucggaa...........................................................................................   1        0     S03
    ..ccucggaauuuccucggaau..........................................................................................   4        0     S03
    ..ccucggaauuuccucggaaua.........................................................................................   4        0     S03
    ..ccucggaauuuccucggaauaA........................................................................................   1        1     S03
    ..ccucggaauuuccucggaauau........................................................................................   9        0     S03
    ..Acucggaauuuccucggaauau........................................................................................   1        1     S03
    ..ccucggaauuuccucggaauauu.......................................................................................   9        0     S03
    ..ccucggaauuuccucggaauauuc......................................................................................   12       0     S03
    ...cucggaauuuccucggaa...........................................................................................   7        0     S03
    ...cucggaauuuccucggaau..........................................................................................   8        0     S03
    ...cucggaauuuccucggaaC..........................................................................................   1        1     S03
    ...cucggaauuuccucggaaua.........................................................................................   13       0     S03
    ...cucggaauuuccucggaauau........................................................................................   8        0     S03
    ...cucggaauuuccucggaauauC.......................................................................................   4        1     S03
    ...cucggaauuuccucggaauauu.......................................................................................   39       0     S03
    ...cucggaauuuccucggaauauuc......................................................................................   5        0     S03
    ....ucggaauuuccucggaau..........................................................................................   11       0     S03
    ....ucggaauuuccucggaaua.........................................................................................   4        0     S03
    ....ucggaauuuccucggaauau........................................................................................   4        0     S03
    ....ucggaauuuccucggaauauC.......................................................................................   3        1     S03
    ....ucggaauuuccucggaauauu.......................................................................................   34       0     S03
    ....ucggaauuuccCcggaauauuc......................................................................................   1        1     S03
    .....cggaauuuccucggaaua.........................................................................................   4        0     S03
    .....cggaauuuccucggaauau........................................................................................   2        0     S03
    .....cggaauuuccCcggaauauu.......................................................................................   1        1     S03
    .....cggaauuuccucggaauauu.......................................................................................   12       0     S03
    .....cggaauuuccucggaauauC.......................................................................................   2        1     S03
    .....cggaauuuccucggaauauuc......................................................................................   1        0     S03
```

Mature **Star**

```
uuccucggaauuuccucggaauauuccgacgaacugagguuuccucggaauuccguccguauauuccgaggaaauuccgaggaaccccaauuuuguguuaccucggaauuuc
......ggaauuuccucggaauau........................................................................................   2   0   S03
........aauuuccucggaauauuccga...................................................................................   1   0   S03
........aauuuccucggaauauuccgacga................................................................................   2   0   S03
........aauuuccucggaauauucUgacga................................................................................   1   1   S03
.........auuuccucggaauauucc.....................................................................................   1   0   S03
.........auuuccucggaauauuccgacg.................................................................................   1   0   S03
..........uuuccucggaauauucUgacga................................................................................   1   1   S03
......................auuccgacgaacugagguuuc.....................................................................   1   0   S03
......................auuccgacgaacugagguuuccuc..................................................................   1   0   S03
......................................guuuccucggaauuccguc.......................................................   1   0   S03
.......................................Cuuccucggaauuccgucc......................................................   1   1   S03
..........................................................guauauuccgaggaaauuccCa................................   1   1   S03
..........................................................guauauuccgaggaaauuccga................................   9   0   S03
..........................................................guauauuccgaggaaauuccgaU...............................   1   1   S03
..........................................................guauauuccgaggaaauuccgag...............................   1   0   S03
..........................................................guauauuccgaggaaauuccgagU..............................   2   1   S03
...........................................................uauauuccgaggaaauuccga................................   16  0   S03
...........................................................uauauuccgaggaaauuccgag...............................   1   0   S03
...........................................................uauauuccgaggaaauuccgagU..............................   1   1   S03
............................................................auauuccgaggaaauuccg.................................   2   0   S03
............................................................auauuccgaggaaauuccga................................   2   0   S03
............................................................auauuccgaggaaauuccgag...............................   1   0   S03
............................................................auauuccgaggaaauuccgagU..............................   11  1   S03
............................................................auauuccgaggaaauuccgagg..............................   1   0   S03
.............................................................uauuccgaggaaauuccg.................................   1   0   S03
.............................................................uauuccgaggaaauuccgagU..............................   1   1   S03
.............................................................uauuccgaggaaauuccgagg..............................   2   0   S03
..............................................................auuccgaggaaauuccga................................   2   0   S03
..............................................................auuccgaggaaauuccgagU..............................   1   1   S03
..............................................................auuccgaggaaauuccgagg..............................   2   0   S03
..............................................................Guuccgaggaaauuccgagg..............................   1   1   S03
...............................................................uuccgaggaaauuccgag...............................   1   0   S03
..................................................................cgaUgaaauuccgaggaac...........................   1   1   S03
..................................................................cgaggaaauuccgaggaac...........................   1   0   S03
..................................................................cgaggGaauuccgaggaacc..........................   1   1   S03
...................................................................gaggaaauuccgaggaac...........................   10  0   S03
...................................................................gaggaaauuccgaggaacc..........................   2   0   S03
...................................................................gaggaaauuccgaggaaccU.........................   1   1   S03
...................................................................gaggaaauuccgaggaaccAc........................   1   1   S03
....................................................................aggaaauucUgaggaaccccaauu....................   1   1   S03
...........................................................................uccgaggaaccAcaauuuugug...............   1   1   S03

uuccucggaauuuccucg..............................................................................................   4   0   S04
uuccucggaauuuccucgga............................................................................................   1   0   S04
uuccucggaauuuccucggaaua.........................................................................................   1   0   S04
uuccucggaauuuccucggaauauu.......................................................................................   1   0   S04
.uGcucggaauuuccucggaauauu.......................................................................................   1   1   S04
.uccucggaauuuGcucggaauauu.......................................................................................   2   1   S04
.ucAucggaauuuccucggaauauu.......................................................................................   2   1   S04
.uAcucggaauuuccucggaauauu.......................................................................................   1   1   S04
..ccucggaauuuccucgga............................................................................................   13  0   S04
..ccGcggaauuuccucgga............................................................................................   1   1   S04
..ccucggaauuuccucggaa...........................................................................................   6   0   S04
..ccucggaauuuccucggaau..........................................................................................   3   0   S04
..ccucggaauuuccucggaaC..........................................................................................   1   1   S04
..ccucggaauuuccucggaaua.........................................................................................   4   0   S04
..ccucggaauuuccucggaauG.........................................................................................   1   1   S04
..ccucggaauuuccucggaauau........................................................................................   8   0   S04
..ccucggaauuuccucggaauauu.......................................................................................   5   0   S04
..ccucggaauuuccucggaauauC.......................................................................................   1   1   S04
..ccucggaauuuccucggaauauCc......................................................................................   1   1   S04
..ccucggaauuuccucggaauauuc......................................................................................   14  0   S04
...cucggaauuuccucggaa...........................................................................................   8   0   S04
...cucggaauuuccucggaau..........................................................................................   9   0   S04
...cucggaauuuccucggaaua.........................................................................................   22  0   S04
...cucggaauuuccucggaauG.........................................................................................   1   1   S04
...cucggaauuuccucggaauau........................................................................................   16  0   S04
...Nucggaauuuccucggaauauu.......................................................................................   1   1   S04
...cucggaauuuccucggaauauC.......................................................................................   4   1   S04
...cucggaauuuccucggaauauu.......................................................................................   40  0   S04
```

Mature **Star**

```
uuccucggaauuuccucggaauauuccgacgaacugagguuuccucggaauuccguccguauauuccgaggaaauuccgaggaaccccaauuuuguguuaccucggaauuuc
...cucggaauuuccucggaaCauuc......................................................................................   1    1    S04
...cucggaauuuccucggaauauuc......................................................................................   3    0    S04
...cucggaauuuccucggaauauucc.....................................................................................   1    0    S04
....ucggaauuuccucggaau..........................................................................................   11   0    S04
....ucggaauuuccucggaaua.........................................................................................   7    0    S04
....ucggaauuuccucggaauau........................................................................................   4    0    S04
....ucggaaCuuccucggaauauu.......................................................................................   1    1    S04
....Ncggaauuuccucggaauauu.......................................................................................   1    1    S04
....ucggaauCuccucggaauauu.......................................................................................   1    1    S04
....ucggaauuuccucggaauauC.......................................................................................   1    1    S04
....ucggaauuuccucggaauauu.......................................................................................   32   0    S04
....ucggaauuuccucggaauauuc......................................................................................   3    0    S04
.....cggaauuuccucggaaua.........................................................................................   2    0    S04
.....cggaauuuccucggaauau........................................................................................   2    0    S04
.....cggaauuuccucggaauauu.......................................................................................   6    0    S04
.....cggaauuuccucggaauauuc......................................................................................   1    0    S04
........aauuuccucggaauauuccgacg.................................................................................   1    0    S04
...............ucggaauauuccgacgaacug............................................................................   1    0    S04
...............ucggaaCauuccgacgaacug............................................................................   1    1    S04
....................auauuccgacgaacugagguuu......................................................................   1    0    S04
.....................uauuccgacgaacugagUuuuc.....................................................................   2    1    S04
......................auuccgacgaacugagUuuuc.....................................................................   1    1    S04
...................................gaUguuuccucggaauuccgucc......................................................   1    1    S04
......................................guuuccucggaauuccgu........................................................   2    0    S04
.......................................uuuccucggaauuccguc.......................................................   1    0    S04
.......................................uuuccucggaauuccgucc......................................................   1    0    S04
.......................................uuuccucggaauuccguccC.....................................................   1    1    S04
................................................aauuccguccgAauauuccg............................................   1    1    S04
..........................................................guauauuccgaggaaauuccg.................................   1    0    S04
..........................................................guauauuccgaggaaauuccga................................   11   0    S04
...........................................................uauGuuccgaggaaauuccg.................................   1    1    S04
...........................................................uauauuccgaggaaauuccCa................................   1    1    S04
...........................................................uauauuccgaggaaauuccga................................   18   0    S04
...........................................................uauauuccgaggaaauuccgag...............................   8    0    S04
...........................................................uauauuccgaggaaauuccgaU...............................   1    1    S04
...........................................................uauauuccgaggaaauuccgagU..............................   2    1    S04
............................................................auauuccgaggaaauuccg.................................   2    0    S04
............................................................auauuccgaggaaauuccga................................   5    0    S04
............................................................auauuccgaggaaauuccgag...............................   1    0    S04
............................................................auauuccgaggaaauuccgagU..............................   7    1    S04
............................................................auauuccgaggaaauuccgagg..............................   2    0    S04
.............................................................uauuccgaggaaauuccga................................   1    0    S04
..............................................................auuccgaggaaauuccgagg..............................   4    0    S04
.................................................................ccgaUgaaauuccgaggaac...........................   1    1    S04
..................................................................cgaUgaaauuccgaggaac...........................   2    1    S04
..................................................................cgaggaaauuccgaUgaacc..........................   1    1    S04
...................................................................gaggaaauuccgaggaac...........................   23   0    S04
...................................................................gaggaaauuccgaggaacc..........................   1    0    S04
...................................................................gaggGaauuccgaggaacc..........................   1    1    S04
...................................................................gGggaaauuccgaggaacc..........................   1    1    S04
...................................................................gaggaaauuccgaggCaccc.........................   1    1    S04
....................................................................aggaaauuccgaggaacc..........................   1    0    S04
............................................................................ccgaggaaccccaauuuugugC..............   1    1    S04
.............................................................................cgaggaaccccaauuuugGgu..............   1    1    S04
```

Provisional ID	:	chrC03_1359400
Score total	:	2
Score for star read(s)	:	3.9
Score for read counts	:	-3.9
Score for mfe	:	2
Score for randfold	:	0
Score for cons. seed	:	
Total read count	:	4
Mature read count	:	3
Loop read count	:	0
Star read count	:	1

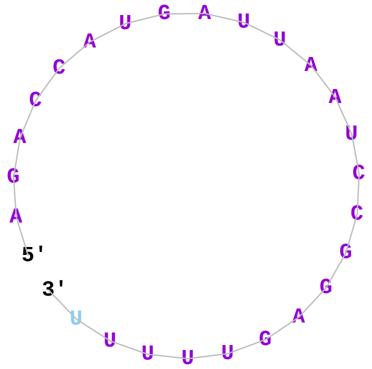

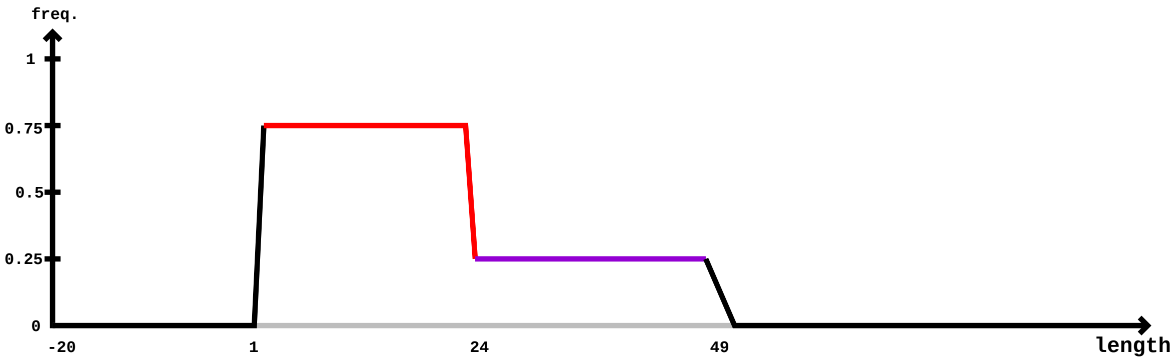

Mature Star

```
5'- cguacuaagaaccucguuaaggacuccggauaaacaugcucuaagaccaugauuaauccggaguuuuuaaagugggguucuuagcgagaguuaagaaauuguuuguuaacuu   -3'   obs
    cguacuaagaaccucguuaaggacuccggauaaacaugcucuaagaccaugauuaauccggaguuuuuaaagugggguucuuagcgagaguuaagaaauuguuuguuaacuu         exp
    ....(((((((((((.(((((((((((((((.((((((.((...)).)))).)).)))))))))))))))...)))))))))))...(((((((.((.....)).)))))))   reads   mm   sample
    ....................ggacuccggauaaacaugcucu......................................................................   1       0    S03
    ....................ggacuccggauaaacaugcucuG.....................................................................   1       1    S03

    ....................Ugacuccggauaaacaugcucu......................................................................   1       1    S04
    ...........................................agaccaugauuaauccggaguuGu.............................................   1       1    S04
```

Provisional ID	:	chrC03_1366790
Score total	:	1
Score for star read(s)	:	-1.3
Score for read counts	:	0
Score for mfe	:	2.3
Score for randfold	:	0
Score for cons. seed	:	
Total read count	:	617
Mature read count	:	461
Loop read count	:	8
Star read count	:	148

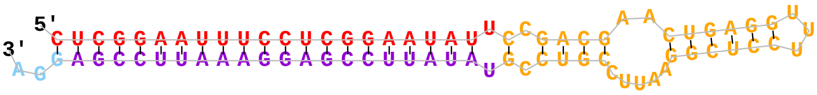

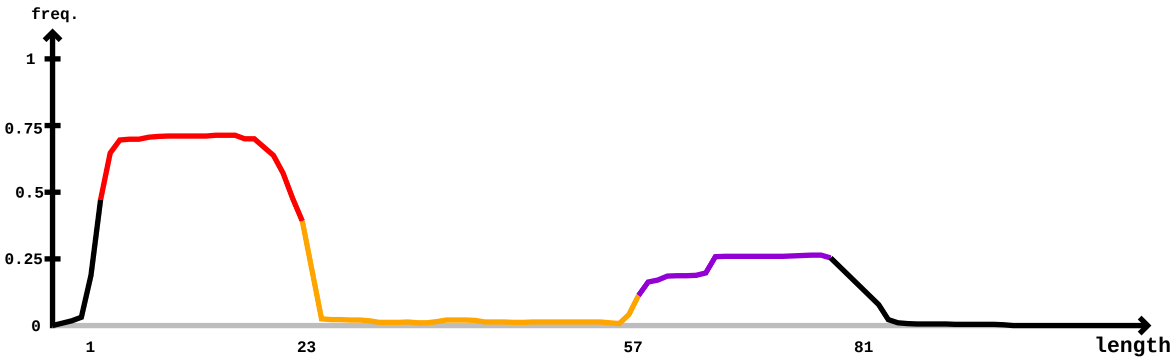

Mature **Star**

```
5'- uuccucggaauuuccucggaauauuccgacgaacugagguuuccucggaauuccguccguauauuccgaggaaauuccgaggaaccccaauuuuguguuaccucggaauuuc  -3'   obs
    uuccucggaauuuccucggaauauuccgacgaacugagguuuccucggaauuccguccguauauuccgaggaaauuccgaggaaccccaauuuuguguuaccucggaauuuc         exp
    ((((((((((((((((((((((.(.((((..((((((...))))))).....)))).).)))))))))))))))))))))))....(((((((........))))))).. reads   mm   sample
    uuccucggaauuuccucg..............................................................................................  5     0    S03
    .uccucggaauuuccucggaa...........................................................................................  1     0    S03
    .uccucggaauuuccucggaaua.........................................................................................  1     0    S03
    .uccucggaauuuccucggaauauC.......................................................................................  1     1    S03
    ..ccucggaauuuccucgga............................................................................................  7     0    S03
    ..ccucggaauuuccucggaa...........................................................................................  1     0    S03
    ..ccucggaauuuccucggaau..........................................................................................  4     0    S03
    ..ccucggaauuuccucggaaua.........................................................................................  4     0    S03
    ..ccucggaauuuccucggaauaA........................................................................................  1     1    S03
    ..ccucggaauuuccucggaauau........................................................................................  9     0    S03
    ..Acucggaauuuccucggaauau........................................................................................  1     1    S03
    ..ccucggaauuuccucggaauauu.......................................................................................  9     0    S03
    ..ccucggaauuuccucggaauauuc......................................................................................  12    0    S03
    ...cucggaauuuccucggaa...........................................................................................  7     0    S03
    ...cucggaauuuccucggaau..........................................................................................  8     0    S03
    ...cucggaauuuccucggaaC..........................................................................................  1     1    S03
    ...cucggaauuuccucggaaua.........................................................................................  13    0    S03
    ...cucggaauuuccucggaauau........................................................................................  8     0    S03
    ...cucggaauuuccucggaauauC.......................................................................................  4     1    S03
    ...cucggaauuuccucggaauauu.......................................................................................  39    0    S03
    ...cucggaauuuccucggaauauuc......................................................................................  5     0    S03
    ....ucggaauuuccucggaau..........................................................................................  11    0    S03
    ....ucggaauuuccucggaaua.........................................................................................  4     0    S03
    ....ucggaauuuccucggaauau........................................................................................  4     0    S03
    ....ucggaauuuccucggaauauC.......................................................................................  3     1    S03
    ....ucggaauuuccucggaauauu.......................................................................................  34    0    S03
    ....ucggaauuuccCcggaauauuc......................................................................................  1     1    S03
    .....cggaauuuccucggaaua.........................................................................................  4     0    S03
    .....cggaauuuccucggaauau........................................................................................  2     0    S03
    .....cggaauuuccCcggaauauu.......................................................................................  1     1    S03
    .....cggaauuuccucggaauauu.......................................................................................  12    0    S03
    .....cggaauuuccucggaauauC.......................................................................................  2     1    S03
    .....cggaauuuccucggaauauuc......................................................................................  1     0    S03
```

Mature	Star		
uuccucggaauuuccucggaauauuccgacgaacugagguuuccucggaauuccguccguauauuccgaggaaauuccgaggaaccccaauuuuguguuaccucggaauuuc			
......ggaauuuccucggaauau..	2	0	S03
........aauuuccucggaauauuccga...	1	0	S03
........aauuuccucggaauauuccgacga..	2	0	S03
........aauuuccucggaauauucUgacga..	1	1	S03
.........auuuccucggaauauucc...	1	0	S03
.........auuuccucggaauauuccgacg...	1	0	S03
..........uuuccucggaauauucUgacga..	1	1	S03
......................auuccgacgaacugagguuuc...	1	0	S03
......................auuccgacgaacugagguuuccuc..	1	0	S03
......................................guuuccucggaauuccguc...	1	0	S03
....Cuuccucggaauuccgucc..	1	1	S03
....guauauuccgaggaaauuccCa................................	1	1	S03
....guauauuccgaggaaauuccga................................	9	0	S03
....guauauuccgaggaaauuccgaU...............................	1	1	S03
....guauauuccgaggaaauuccgag...............................	1	0	S03
....guauauuccgaggaaauuccgagU..............................	2	1	S03
....uauauuccgaggaaauuccga................................	16	0	S03
....uauauuccgaggaaauuccgag...............................	1	0	S03
....uauauuccgaggaaauuccgagU..............................	1	1	S03
....auauuccgaggaaauuccg.................................	2	0	S03
....auauuccgaggaaauuccga................................	2	0	S03
....auauuccgaggaaauuccgag...............................	1	0	S03
....auauuccgaggaaauuccgagU..............................	11	1	S03
....auauuccgaggaaauuccgagg..............................	1	0	S03
....uauuccgaggaaauuccg.................................	1	0	S03
....uauuccgaggaaauuccgagU..............................	1	1	S03
....uauuccgaggaaauuccgagg..............................	2	0	S03
....auuccgaggaaauuccga................................	2	0	S03
....auuccgaggaaauuccgagU..............................	1	1	S03
....auuccgaggaaauuccgagg..............................	2	0	S03
....Guuccgaggaaauuccgagg..............................	1	1	S03
....uuccgaggaaauuccgag..............................	1	0	S03
....cgaUgaaauuccgaggaac...........................	1	1	S03
....cgaggaaauuccgaggaac...........................	1	0	S03
....cgaggGaauuccgaggaacc..........................	1	1	S03
....gaggaaauuccgaggaac...........................	10	0	S03
....gaggaaauuccgaggaacc..........................	2	0	S03
....gaggaaauuccgaggaaccU.........................	1	1	S03
....gaggaaauuccgaggaaccAc........................	1	1	S03
....aggaaauucUgaggaaccccaauu....................	1	1	S03
....uccgaggaaccAcaauuuugug...............	1	1	S03
uuccucggaauuuccucg..	4	0	S04
uuccucggaauuuccucgga..	1	0	S04
uuccucggaauuuccucggaaua...	1	0	S04
uuccucggaauuuccucggaauauu...	1	0	S04
.uGcucggaauuuccucggaauauu...	1	1	S04
.uccucggaauuuGcucggaauauu...	2	1	S04
.ucAucggaauuuccucggaauauu...	2	1	S04
.uAcucggaauuuccucggaauauu...	1	1	S04
..ccucggaauuuccucgga..	13	0	S04
..ccGcggaauuuccucgga..	1	1	S04
..ccucggaauuuccucggaa...	6	0	S04
..ccucggaauuuccucggaau..	3	0	S04
..ccucggaauuuccucggaaC..	1	1	S04
..ccucggaauuuccucggaaua...	4	0	S04
..ccucggaauuuccucggaauG...	1	1	S04
..ccucggaauuuccucggaauau..	8	0	S04
..ccucggaauuuccucggaauauu...	5	0	S04
..ccucggaauuuccucggaauauC...	1	1	S04
..ccucggaauuuccucggaauauCc..	1	1	S04
..ccucggaauuuccucggaauauuc..	14	0	S04
...cucggaauuuccucggaa...	8	0	S04
...cucggaauuuccucggaau..	9	0	S04
...cucggaauuuccucggaaua...	22	0	S04
...cucggaauuuccucggaauG...	1	1	S04
...cucggaauuuccucggaauau..	16	0	S04
...Nucggaauuuccucggaauauu...	1	1	S04
...cucggaauuuccucggaauauC...	4	1	S04
...cucggaauuuccucggaauauu...	40	0	S04

Mature **Star**

```
uuccucggaauuuccucggaauauuccgacgaacugagguuuccucggaauuccguccguauauuccgaggaaauuccgaggaaccccaauuuuguguuaccucggaauuuc
...cucggaauuuccucggaaCauuc......................................................................................    1    1    S04
...cucggaauuuccucggaauauuc......................................................................................    3    0    S04
...cucggaauuuccucggaauauucc.....................................................................................    1    0    S04
....ucggaauuuccucggaau..........................................................................................    11   0    S04
....ucggaauuuccucggaaua.........................................................................................    7    0    S04
....ucggaauuuccucggaauau........................................................................................    4    0    S04
....ucggaaCuuccucggaauauu.......................................................................................    1    1    S04
....Ncggaauuuccucggaauauu.......................................................................................    1    1    S04
....ucggaauCuccucggaauauu.......................................................................................    1    1    S04
....ucggaauuuccucggaauauC.......................................................................................    1    1    S04
....ucggaauuuccucggaauauu.......................................................................................    32   0    S04
....ucggaauuuccucggaauauuc......................................................................................    3    0    S04
.....cggaauuuccucggaaua.........................................................................................    2    0    S04
.....cggaauuuccucggaauau........................................................................................    2    0    S04
.....cggaauuuccucggaauauu.......................................................................................    6    0    S04
.....cggaauuuccucggaauauuc......................................................................................    1    0    S04
........aauuuccucggaauauuccgacg.................................................................................    1    0    S04
...............ucggaauauuccgacgaacug............................................................................    1    0    S04
...............ucggaaCauuccgacgaacug............................................................................    1    1    S04
....................auauuccgacgaacugagguuu......................................................................    1    0    S04
.....................uauuccgacgaacugagUuuc......................................................................    2    1    S04
......................auuccgacgaacugagUuuc......................................................................    1    1    S04
...................................gaUguuuccucggaauuccgucc......................................................    1    1    S04
......................................guuuccucggaauuccgu........................................................    2    0    S04
.......................................uuuccucggaauuccguc.......................................................    1    0    S04
.......................................uuuccucggaauuccgucc......................................................    1    0    S04
.......................................uuuccucggaauuccguccC.....................................................    1    1    S04
................................................aauuccguccgAauauuccg............................................    1    1    S04
..........................................................guauauuccgaggaaauuccg.................................    1    0    S04
..........................................................guauauuccgaggaaauuccga................................    11   0    S04
...........................................................uauGuuccgaggaaauuccg.................................    1    1    S04
...........................................................uauauuccgaggaaauuccCa................................    1    1    S04
...........................................................uauauuccgaggaaauuccga................................    18   0    S04
...........................................................uauauuccgaggaaauuccgag...............................    8    0    S04
...........................................................uauauuccgaggaaauuccgaU...............................    1    1    S04
...........................................................uauauuccgaggaaauuccgagU..............................    2    1    S04
............................................................auauuccgaggaaauuccg.................................    2    0    S04
............................................................auauuccgaggaaauuccga................................    5    0    S04
............................................................auauuccgaggaaauuccgag...............................    1    0    S04
............................................................auauuccgaggaaauuccgagU..............................    7    1    S04
............................................................auauuccgaggaaauuccgagg..............................    2    0    S04
.............................................................uauuccgaggaaauuccga................................    1    0    S04
..............................................................auuccgaggaaauuccgagg..............................    4    0    S04
.................................................................ccgaUgaaauuccgaggaac...........................    1    1    S04
..................................................................cgaUgaaauuccgaggaac...........................    2    1    S04
..................................................................cgaggaaauuccgaUgaacc..........................    1    1    S04
...................................................................gaggaaauuccgaggaac...........................    23   0    S04
...................................................................gaggaaauuccgaggaacc..........................    1    0    S04
...................................................................gaggGaauuccgaggaacc..........................    1    1    S04
...................................................................gGggaaauuccgaggaacc..........................    1    1    S04
...................................................................gaggaaauuccgaggCaccc.........................    1    1    S04
....................................................................aggaaauuccgaggaacc..........................    1    0    S04
............................................................................ccgaggaaccccaauuuugugC..............    1    1    S04
.............................................................................cgaggaaccccaauuuugGgu..............    1    1    S04
```

Provisional ID : chrC03_1369380
Score total : 1.1
Score for star read(s) : -1.3
Score for read counts : 0
Score for mfe : 2.4
Score for randfold : 0
Score for cons. seed :
Total read count : 8869
Mature read count : 7803
Loop read count : 992
Star read count : 74

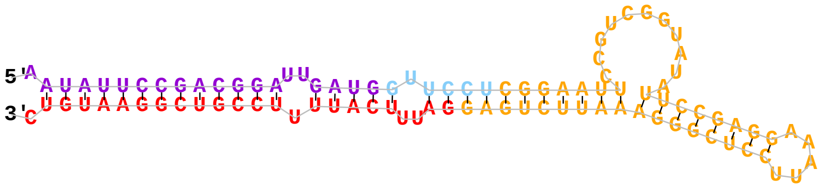

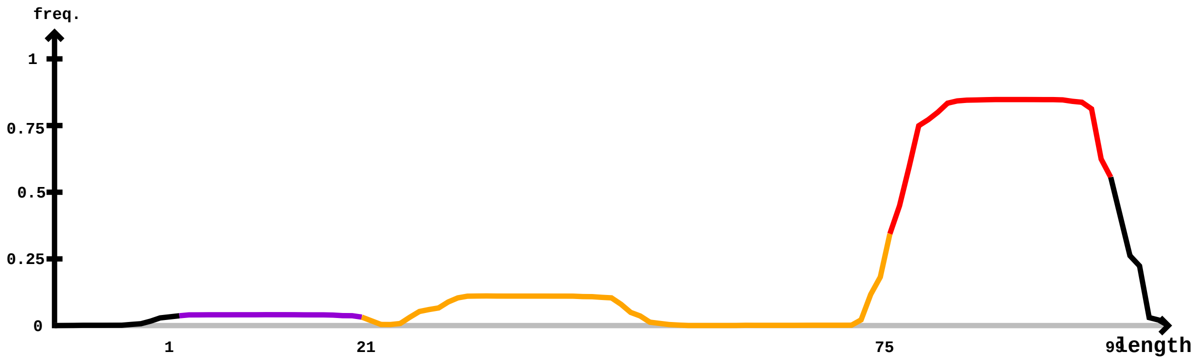


```
              Star                                                              Mature
5'- aauuuccucggaauauuccgacggauugaugguuccucggaauuccgucgguauauuccgaggaaauuccucgggaaauucugaggauuucauuuuccgucggaauguccguca   -3'   obs
    aauuuccucggaauauuccgacggauugaugguuccucggaauuccgucgguauauuccgaggaaauuccucgggaaauucugaggauuucauuuuccgucggaauguccguca         exp
    ........((((.((((((((((((..(((((.(((((((((((...........((((((((.....)))))))))))))))))))..))))).))))))))))))))))...  reads   mm   sample
    aauuuccucggaauauuccga.............................................................................................  1       0    S03
    aauuuccucggaauauuccgacgg..........................................................................................  1       0    S03
    .auuuccucggaauauucc...............................................................................................  1       0    S03
    .auuuccucggaauauuccgacg...........................................................................................  1       0    S03
    .auuuccucggaauauucGgacgga.........................................................................................  1       1    S03
    ....ucAucggaauauuccgacggauug......................................................................................  1       1    S03
    .....ccucggaauauuccgacggCuug......................................................................................  1       1    S03
    ......cucggaauauuccgacggauG.......................................................................................  1       1    S03
    ......cucggaauauuccgacggauug......................................................................................  3       0    S03
    ......cucggaauauuccgacggauugau....................................................................................  8       0    S03
    ......cucggaauauuccgacggauugauU...................................................................................  1       1    S03
    .......ucggaauauuccgacggauuga.....................................................................................  1       0    S03
    .......ucggaauauuccgacggauugaug...................................................................................  5       0    S03
    ........cggaauauuccgacggauu.......................................................................................  1       0    S03
    ........cggaauauuccgacggauug......................................................................................  3       0    S03
    ........cggaauauuccgacggauugau....................................................................................  5       0    S03
    ........cggaauauuccgacggauugauU...................................................................................  4       1    S03
    ........cggaauauuccgacggauugaug...................................................................................  32      0    S03
    ........cggaauauuccgacggauugauC...................................................................................  1       1    S03
    ........cggaauauuccgacggauugaugg..................................................................................  1       0    S03
    .........ggaauauuccgacggauu.......................................................................................  1       0    S03
    .........ggaauauuccCacggauu.......................................................................................  1       1    S03
    .........ggaauauuccgacggauug......................................................................................  4       0    S03
    .........ggaauauuccgacggauuga.....................................................................................  1       0    S03
    .........ggaauauuccgacggauugau....................................................................................  5       0    S03
    .........ggaauauuccgacggauugGug...................................................................................  1       1    S03
    .........ggaauauuccgacggauugauU...................................................................................  4       1    S03
    .........ggaauauuccgacggGuugaug...................................................................................  1       1    S03
    .........ggaauauuccgacggauugaug...................................................................................  21      0    S03
    .........ggaauauuccgacggauugauUg..................................................................................  2       1    S03
    .........ggaauauuccgacggauugaugC..................................................................................  2       1    S03
    .........ggaauauuccgacggauugaugCu.................................................................................  2       1    S03
    .........ggaauauuccgacggauugaugguu................................................................................  1       0    S03
```

Star   Mature

```
aauuuccucggaauauuccgacggauugaugguuccucggaauuccgucgguauauuccgaggaaauuccucgggaaauucugaggauuucauuuuccgucggaauguccguca
..........gaauauuccgacggauugau....................................................................................	1	0	S03
..........gaauauuccgacggauugauU...................................................................................	1	1	S03
..........gaauauuccgacggauugaug...................................................................................	7	0	S03
..........gaauauuccgacggauugaugg..................................................................................	2	0	S03
..........gaauauuccgacggauugaugC..................................................................................	3	1	S03
...........aauauuccgacggauugau....................................................................................	4	0	S03
...........aauauuccgacggauugauC...................................................................................	1	1	S03
...........aauauuccgacggauugaug...................................................................................	7	0	S03
...........aauauuccgacggauugaugg..................................................................................	2	0	S03
...........aauauuccgacggauugauggA.................................................................................	1	1	S03
...........aauauuccgacggauugaugguuc...............................................................................	1	0	S03
............auauuccgacggauugau....................................................................................	1	0	S03
............auauuccgacggauugauU...................................................................................	2	1	S03
............auauuccgacggauugauC...................................................................................	1	1	S03
............auauuccgacggauugaug...................................................................................	7	0	S03
............auauuccgacggauugaugguucc..............................................................................	2	0	S03
.............uauuccgacggGuugaugguuccu.............................................................................	1	1	S03
..............auuccgacggauugaugg..................................................................................	1	0	S03
..............auuccgacggauugauggu.................................................................................	1	0	S03
...................gacggauugaugguuccucgg..........................................................................	1	0	S03
....................acggauugaugguuccucggaauu......................................................................	1	0	S03
................................uuccucggaauuccgucgguauG...........................................................	1	1	S03
................................uuccucggaauucGgucgguauau..........................................................	1	1	S03
................................uuccucggaauuccgucgguauau..........................................................	1	0	S03
..................................ccucggaauuccgucggua.............................................................	1	0	S03
..................................ccucggaauuccgucgguauauu.........................................................	2	0	S03
..................................ccucggaauuccgucgguauauuc........................................................	12	0	S03
...................................cucggaauuccgucggua.............................................................	7	0	S03
...................................cucggaauuccgucgguau............................................................	1	0	S03
...................................cucggaauuccgucgguaua...........................................................	1	0	S03
...................................cucggaauuccgucgguauG...........................................................	1	1	S03
...................................cucggaauuccgucgguauau..........................................................	2	0	S03
...................................cucggUauuccgucgguauauu.........................................................	2	1	S03
...................................cucggaauuccgucgguauGuu.........................................................	1	1	S03
...................................cucggaauuccgucgguauauu.........................................................	50	0	S03
...................................cucggaauuccgucgguauauuc........................................................	20	0	S03
...................................cucggaauuccgucgguauauucc.......................................................	14	0	S03
....................................ucggaauuccgucgguau............................................................	1	0	S03
....................................ucggaauuccgucgguaua...........................................................	7	0	S03
....................................ucggaauuccgucgguauG...........................................................	1	1	S03
....................................ucggaauuccgucgguauau..........................................................	2	0	S03
....................................ucggaauuccgucgguauauu.........................................................	20	0	S03
....................................ucggaauuccgucgguauauuc........................................................	53	0	S03
....................................ucggaauuccgucgguauauucc.......................................................	1	0	S03
.....................................cggaauuccgucgguaua...........................................................	1	0	S03
.....................................cggaauuccgucgguauau..........................................................	4	0	S03
.....................................cggaauuccgucgguauauu.........................................................	9	0	S03
.....................................cggaauuccguGgguauauuc........................................................	1	1	S03
.....................................cggaauuccgucgguauauuc........................................................	15	0	S03
.....................................cggaauuccgucgguauauucc.......................................................	2	0	S03
......................................ggaauuccgucgguauauu.........................................................	16	0	S03
......................................ggaauuccgucgguauauuc........................................................	4	0	S03
......................................ggaauuccgucgguauauuccga.....................................................	3	0	S03
......................................ggaauuccgucgguauauuccgag....................................................	4	0	S03
.......................................gaauuccgucgguauauu.........................................................	6	0	S03
.......................................gaauuccgucgguauauuc........................................................	2	0	S03
.......................................gaauuccgucgguauauucc.......................................................	10	0	S03
.......................................gaauuccgucgguauauuccg......................................................	61	0	S03
.......................................gaauuccgucgguauauuccga.....................................................	13	0	S03
.......................................gaauuccgucgguauauuccgag....................................................	6	0	S03
........................................aauuccgucgguauauuc........................................................	10	0	S03
........................................aauuccgucgguauauucc.......................................................	20	0	S03
........................................aauuccgucgguauauuccC......................................................	2	1	S03
........................................aauuccgucgguauauuccg......................................................	30	0	S03
........................................aauuccgucgguauauuccga.....................................................	3	0	S03
........................................aauuccgucgguauauuccgag....................................................	9	0	S03
........................................aauuccgucgguauauuccgagg...................................................	1	0	S03
........................................aauuccgucgguauauuccgaggG..................................................	2	1	S03
........................................aauuccgucgguauauuccgagga..................................................	5	0	S03
.........................................auuccgucgguauauucc.......................................................	8	0	S03
```

```
                    Star                                                                           Mature
aauuuccucggaauauuccgacggauugaugguuccucggaauuccgucgguauauuccgaggaaauuccucgggaaauucugaggauuucauuuuccgucggaauguccguca
.........................................auuccgucgguauauuccg......................................................   9   0   S03
.........................................auuccgucgguauauuccga.....................................................   1   0   S03
.........................................auuccgucgguauauuccgag....................................................   1   0   S03
.........................................auuccgucgguauauuccgagg...................................................   5   0   S03
..........................................uuccgucgguauauuccg......................................................   1   0   S03
..........................................uuccgucgguauauuccgag....................................................   1   0   S03
...........................................uccgucgguauauuccga.....................................................   1   0   S03
.....................................................uauuccgaggGaauuccu...........................................   1   1   S03
.....................................................................cucgggaaauucugaggauuucau.....................   1   0   S03
......................................................................ucgggaaauucugaggaG..........................   1   1   S03
......................................................................ucgggaaauucugaggauuucauu....................   2   0   S03
......................................................................ucgAgaaauucugaggauuucauu....................   1   1   S03
.............................................................................auucugaggauuucauuuuccguc.............   1   0   S03
.................................................................................ugaggauuucauuuuccgucgga..........   1   0   S03
..................................................................................gaggauuucauuuuccgucgga..........   1   0   S03
..................................................................................gaggauuucauuCuccgucgga..........   1   1   S03
..................................................................................gaggauuucauuuuccgucggaa.........   3   0   S03
..................................................................................gaggauuucauuuuccgucggUa.........   1   1   S03
..................................................................................Naggauuucauuuuccgucggaau........   1   1   S03
..................................................................................gaggauuucauuCuccgucggaau........   1   1   S03
..................................................................................gaggauuucauuuuccgucggaau........   77  0   S03
..................................................................................gaggauuucauuuuccgucggaaG........   1   1   S03
..................................................................................gaggauuucauuuuccgucggaaA........   2   1   S03
..................................................................................gaggauuucauuuuccgucggaauC.......   1   1   S03
..................................................................................gaggauuucauuuuccgucggaaug.......   5   0   S03
..................................................................................gaggauuucauuuuccgucggaaugu......   2   0   S03
..................................................................................gaggauuucauuuuccgucggaaugA......   1   1   S03
..................................................................................gaggauuucauuuuccgucggaauguc.....   1   0   S03
...................................................................................aggauuucauuuuccgucgga..........   7   0   S03
...................................................................................aggauuucauuuuccgucggaa.........   5   0   S03
...................................................................................aggauuucauuuuccgucggaaG........   1   1   S03
...................................................................................aggauuucauuuuccgucggaau........   5   0   S03
...................................................................................aggauuucauuuuccgucggaaug.......   371 0   S03
...................................................................................aggauuucauuuuccgucggaauC.......   1   1   S03
...................................................................................aggauCucauuuuccgucggaaug.......   3   1   S03
...................................................................................aggauuucaCuuccgucggaaug.......   1   1   S03
...................................................................................aggauuucauuGuccgucggaaug.......   1   1   S03
...................................................................................aggauuucauuuuccgucggaaGg.......   2   1   S03
...................................................................................aggaAuucauuuuccgucggaaug.......   1   1   S03
...................................................................................aggauuucauuCuccgucggaaug.......   1   1   S03
...................................................................................aggauuucauuuuccgucggaaugu......   1   0   S03
...................................................................................aggauuucauuuuccgucggaauguc.....   1   0   S03
...................................................................................Nggauuucauuuuccgucggaaugucc....   1   1   S03
....................................................................................ggauuucauuuuccgucgg...........   2   0   S03
....................................................................................ggauuucauuuuccgucgga..........   1   0   S03
....................................................................................ggauuucauuuuccgucggaa.........   3   0   S03
....................................................................................ggauuucauuuuccgucggaau........   20  0   S03
....................................................................................ggauuucauuCuccgucggaau........   1   1   S03
....................................................................................ggauuucauCuuccgucggaaug.......   1   1   S03
....................................................................................ggauuucauuuuccgucggaaug.......   107 0   S03
....................................................................................ggauuucaCuuccgucggaaug.......   1   1   S03
....................................................................................ggauuucauuuuccgucggUaug.......   2   1   S03
....................................................................................ggauuucauuuuccgucggaaugG......   9   1   S03
....................................................................................ggauAucauuuuccgucggaaugu......   1   1   S03
....................................................................................Ngauuucauuuuccgucggaaugu......   2   1   S03
....................................................................................ggauuucauuuuccgucggaaugC......   16  1   S03
....................................................................................ggauuucauuCuccgucggaaugu......   1   1   S03
....................................................................................ggauuucauuuuccgucggaaugu......   168 0   S03
....................................................................................ggauuucauuuuccgucggaaugA......   8   1   S03
....................................................................................ggauCucauuuuccgucggaaugu......   1   1   S03
....................................................................................ggauuucauuuuccgucggaauguc.....   3   0   S03
.....................................................................................gauuucauuuuccgucgga..........   1   0   S03
.....................................................................................gauuucauuuuccgucggaaA........   1   1   S03
.....................................................................................gauuucauuuuccgucggaau........   19  0   S03
.....................................................................................gauuucaCuuccgucggaaug.......   1   1   S03
.....................................................................................Nauuucauuuuccgucggaaug.......   1   1   S03
.....................................................................................gauuucauuuuccgucggaaug.......   243 0   S03
.....................................................................................gauuucauuCuccgucggaaug.......   2   1   S03
.....................................................................................gauuucauuuuccCucggaaug.......   1   1   S03
.....................................................................................gauuucauuuAccgucggaaug.......   1   1   S03
```

Star **Mature**

aauuuccucggaauauuccgacggauugaugguuccucggaauuccgucgguauauuccgaggaaauuccucgggaaauucugaggauuucauuuuccgucggaauguccguca			
....gauuucauuuuccgucggaaugC......	1	1	S03
....gauuucauuuuccgucggaaugu......	8	0	S03
....gauuucauuuuccgucggaaugA......	3	1	S03
....gauuucaCuuuccgucggaauguc.....	1	1	S03
....gauCucauuuuccgucggaauguc.....	1	1	S03
....gauuucauuuuccgucggaaugGc.....	1	1	S03
....gaGuucauuuuccgucggaauguc.....	1	1	S03
....gauGucauuuuccgucggaauguc.....	1	1	S03
....gCuuucauuuuccgucggaauguc.....	1	1	S03
....gauuucauCuuccgucggaauguc.....	3	1	S03
....Nauuucauuuuccgucggaauguc.....	2	1	S03
....gauuAcauuuuccgucggaauguc.....	1	1	S03
....gauuucauuuuccgucggaauguG.....	7	1	S03
....gauuucauuCuccgucggaauguc.....	1	1	S03
....gauuucauuuuccgucggaaugCc.....	6	1	S03
....gauuucauuuuccgucggaauguc.....	426	0	S03
....gauuucauuuuccgucggaauguccguca	1	0	S03
....auuucauuuuccgucgga..........	3	0	S03
....auuucauuuuccgucggaa.........	2	0	S03
....auuucauuuuccgucggaau........	2	0	S03
....auuucaCuuuccgucggaaug.......	1	1	S03
....auuucauCuuccgucggaaug.......	2	1	S03
....auCucauuuuccgucggaaug.......	1	1	S03
....auuucauuuuccgucggaauC.......	1	1	S03
....auuucauuuuccgucggaaug.......	121	0	S03
....auuucauuuuccgucggaaugG......	4	1	S03
....auuucauuuuccgucggaaugC......	6	1	S03
....auuucauuuuccgucggUaugu......	1	1	S03
....auuucauuuuccgucggaaugA......	6	1	S03
....auuucauuuuccgucggaaugu......	77	0	S03
....auuucauuuuccgucggaauguG.....	2	1	S03
....auuGcauuuuccgucggaauguc.....	1	1	S03
....auuucauuuAccgucggaauguc.....	1	1	S03
....auCucauuuuccgucggaauguc.....	2	1	S03
....auuucauuuuccgucggaaugGc.....	1	1	S03
....auuucauuuuccgucggaaugAc.....	1	1	S03
....auuucauuuuccgucggaauguc.....	55	0	S03
....auuucauuuuccgucggaaugucc....	150	0	S03
....Nuuucauuuuccgucggaaugucc....	1	1	S03
....auuucaCuuuccgucggaaugucc....	1	1	S03
....Cuuucauuuuccgucggaaugucc....	1	1	S03
....auuucauuuuccgucggaaugucG....	3	1	S03
....auuucauCuuccgucggaaugucc....	1	1	S03
....auuucauuuuccgucggaauguccC...	1	1	S03
....auuucauuuuccgucggaauguccg...	1	0	S03
....uuucauuuuccgucggaa.........	1	0	S03
....Nuucauuuuccgucggaau........	1	1	S03
....uuucauuuuccgucggaau........	2	0	S03
....uuucauuuuccgucggaaug.......	13	0	S03
....uuucauuCuccgucggaaugu......	1	1	S03
....uuucauuuuccgucggaaugG......	1	1	S03
....uuucauuuuccgucggaaugu......	13	0	S03
....uuGcauuuuccgucggaauguc.....	1	1	S03
....uuucauuuuccgGcggaauguc.....	1	1	S03
....uuucauuuuccgucggaaGguc.....	1	1	S03
....uuucauuuuccgucggaaugGc.....	1	1	S03
....uGucauuuuccgucggaauguc.....	2	1	S03
....uuucauuuuccgucggaauguG.....	4	1	S03
....uuucauuuuccgucggUauguc.....	2	1	S03
....uCucauuuuccgucggaauguc.....	1	1	S03
....uuucauuuuccgucggaaugCc.....	2	1	S03
....uuucauuuuccgucggCauguc.....	1	1	S03
....uuucauuCuccgucggaauguc.....	2	1	S03
....uuucauuuuccgucggaauguc.....	411	0	S03
....Nuucauuuuccgucggaauguc.....	3	1	S03
....uuucaCuuuccgucggaauguc.....	1	1	S03
....uuucauuuuccgucggaaugAc.....	1	1	S03
....uuucauuuuccgucggaaugucc....	24	0	S03
....uuucauGuuccgucggaaugucc....	1	1	S03
....uuucauuuuccgucggaaugucG....	1	1	S03

Star **Mature**

aauuuccucggaauauuccgacggauugaugguuccucggaauuccgucgguauauuccgaggaaauuccucgggaaauucugaggauuucauuuuccgucggaauguccguca			
....Guucauuuuccgucggaaugucc....	1	1	S03
....uuucauuCuccgucggaaugucc....	1	1	S03
....Auucauuuuccgucggaaugucc....	1	1	S03
....uuucauCuuccgucggaauguccg...	1	1	S03
....uuucauuuuccgucggaauguccC...	1	1	S03
....uuucauuuuccgucggaauguccg...	71	0	S03
....uCucauuuuccgucggaauguccg...	1	1	S03
....uuucauuuuccgucggUauguccg...	1	1	S03
....uuucauuuuccgGcggaauguccgu..	1	1	S03
....uuucauuuuccgucggaauguccgA..	3	1	S03
....Auucauuuuccgucggaauguccgu..	1	1	S03
....uuucauuuuccgucggaaugGccgu..	1	1	S03
....uuucauuuuccgucggaauguccgG..	3	1	S03
....uuucauuuuccgucggaauguccgu..	69	0	S03
....uucauuuuccgucggaau........	2	0	S03
....uucauuuuccgucggaaug.......	4	0	S03
....Gucauuuuccgucggaaug.......	1	1	S03
....uucauuuuccgucggaaugG......	1	1	S03
....uucauuuuccgucggaaugu......	1	0	S03
....Gucauuuuccgucggaauguc.....	1	1	S03
....uucauuuuccgucggaauguc.....	63	0	S03
....uucauuuuccgucggaaugAcc....	1	1	S03
....uucauuuuccgucggaaugCcc....	1	1	S03
....uucauuuuccgucggaaugGcc....	1	1	S03
....uucauuuuccgucggaaugucG....	1	1	S03
....uucauuuuccgucggaaugucc....	22	0	S03
....uucauuuuccgucggaauguccg...	26	0	S03
....uucauuuuccgGcggaauguccg...	1	1	S03
....uucauuuuccgucggaaugAccgu..	1	1	S03
....uucauuuuccgucggaaGguccgu..	1	1	S03
....uucauCuuccgucggaauguccgu..	2	1	S03
....uucauuuuccgucggaauguccgG..	21	1	S03
....Nucauuuuccgucggaauguccgu..	1	1	S03
....uucauuuuccgucggaaAguccgu..	1	1	S03
....uucauuuuccgucggaauguccgu..	504	0	S03
....uucauuuuccgucggaaugCccgu..	1	1	S03
....uucauuuuccgucggaauguccCu..	3	1	S03
....uucauuCuccgucggaauguccgu..	4	1	S03
....uucauuuuccgGcggaauguccgu..	1	1	S03
....uGcauuuuccgucggaauguccgu..	1	1	S03
....Gucauuuuccgucggaauguccgu..	4	1	S03
....uucauuuuccgucggaauguccgA..	10	1	S03
....ucauuuuccgucggaaug.......	4	0	S03
....ucauuCuccgucggaaug.......	1	1	S03
....ucauuuuccgucggaaugC......	1	1	S03
....ucauuuuccgucggaauguc.....	16	0	S03
....ucauuuuccgucggaaugucc....	5	0	S03
....ucauuuuccgucggaauguccg...	14	0	S03
....ucauuuuccgucggaauguccgu..	15	0	S03
....ucauuuuccgucggaauguccguc.	23	0	S03
....cauuuuccgucggaauguc.....	3	0	S03
....cauuuuccgucggaaugucc....	2	0	S03
....cauuuuccgucggaaugCccg...	1	1	S03
....cauuuuccgucggaauguccg...	10	0	S03
....cauuuuccgucggaauguccgu..	74	0	S03
....caCuuccgucggaauguccgu..	1	1	S03
....cauuuuccgucggaauguccgG..	4	1	S03
....cauuuuccgucggaauguccgA..	3	1	S03
....Nauuuccgucggaauguccgu..	1	1	S03
....cauuuuccgucggaauguccguc.	2	0	S03
....cauuuuccgucggaauguccguca	16	0	S03
....auuuccgucggaauguc.....	1	0	S03
....auuuccgucggaaugucc....	3	0	S03
....auuuccgucggaauguccg...	4	0	S03
....auuuccgucggaauguccgG..	8	1	S03
....auuuccgucggaauguccgA..	4	1	S03
....auuuccgucggaauguccgu..	63	0	S03
....auuuccgucggaauguccguc.	6	0	S03
....auuuccgucggaauguccgAca	1	1	S03
....auuuccgucggaauguccguca	46	0	S03

```
         Star                                          Mature
aauuuccucggaauauuccgacggauugaugguuccucggaauuccgucgguauauuccgaggaaauuccucgggaaauucugaggauuucauuuuccgucggaaugucccguca
............................................................................................uuuuccgucggaaugucc....   2   0   S03
............................................................................................Cuuuccgucggaauguccg...   1   1   S03
............................................................................................uuuuccgucggaauguccg...   2   0   S03
............................................................................................uuuuccgucggaauguccgG..   1   1   S03
............................................................................................uuuuccgucggaauguccgA..   1   1   S03
............................................................................................uuuuccgucggaauguccgu..   18  0   S03
............................................................................................uuuuccgucggaauguccguc.   3   0   S03
............................................................................................uuuuccgucggaauguccguca   3   0   S03
.............................................................................................uuuccgucggaauguccgA..   1   1   S03
.............................................................................................uuuccgucggaauguccgu..   7   0   S03
.............................................................................................uuuccgucggaauguccguca   5   0   S03
.............................................................................................uuuccgucggaaugGccguca   1   1   S03
..............................................................................................uuccgucggaauguccgu..   1   0   S03
..............................................................................................uuccgucggaauguccguca   3   0   S03
...............................................................................................uccgucggaauguccguc.   1   0   S03
...............................................................................................uccgucggaauguccguca   1   0   S03
................................................................................................ccgucggaauguccguca   3   0   S03

aauuuccucggaauauuccgacg...........................................................................................   1   0   S04
aauuuccucggaauauuccgacgg..........................................................................................   2   0   S04
.auuuccucggaauGuuccgacgg..........................................................................................   1   1   S04
......cucggaauauuccgacggauug......................................................................................   4   0   S04
......cucggaauauuccgacggauugau....................................................................................   9   0   S04
......cucggaauauuccgacggauugauU...................................................................................   1   1   S04
......cucggaauauuccgacggauugauC...................................................................................   1   1   S04
......cucggaauauuUcgacggauugaugg..................................................................................   1   1   S04
.......ucggaauauuccgaAggauugaug...................................................................................   1   1   S04
.......ucggaauauuccgacggauugaug...................................................................................   17  0   S04
.......ucggaauauuccgGcggauugaug...................................................................................   1   1   S04
........cggaauauuccgacggauu.......................................................................................   1   0   S04
........Gggaauauuccgacggauug......................................................................................   1   1   S04
........cggaauauuccgacggauug......................................................................................   2   0   S04
........cggaauauuccgacggauugau....................................................................................   3   0   S04
........Aggaauauuccgacggauugau....................................................................................   1   1   S04
........cggaauauuccgacggauugaug...................................................................................   33  0   S04
........cggaauauuccgacggauugauU...................................................................................   1   1   S04
........cggaauauuccgacggauugaugg..................................................................................   1   0   S04
........cggaauauuccgacggaCugaugg..................................................................................   1   1   S04
.........ggaauauuccgacggauu.......................................................................................   2   0   S04
.........ggaauauuccgacggauug......................................................................................   1   0   S04
.........ggaauauuccgacggauuga.....................................................................................   1   0   S04
.........ggaauauuccgacggauugau....................................................................................   6   0   S04
.........ggaauauuccgacggaCugaug...................................................................................   1   1   S04
.........ggaauauCccgacggauugaug...................................................................................   1   1   S04
.........ggaauauuccgacggauugaug...................................................................................   38  0   S04
.........ggaauauuccgacggauugauU...................................................................................   5   1   S04
.........ggaauauuccgacggauugauC...................................................................................   2   1   S04
.........ggaauauuccgacggauugGug...................................................................................   1   1   S04
.........ggaauauuccgacggauugauUg..................................................................................   1   1   S04
.........ggaauauuccgacggauugaugC..................................................................................   4   1   S04
.........ggaauauuccgacggauugaugg..................................................................................   2   0   S04
.........ggaauauuccgacggauugauggu.................................................................................   1   0   S04
.........ggaauauuccgacggauugaugCu.................................................................................   3   1   S04
..........gaauauuccgacggauugau....................................................................................   2   0   S04
..........gaauauuccgacggauugaug...................................................................................   7   0   S04
..........gaauauuccgacggauugauC...................................................................................   1   1   S04
..........gaauauuccgacggauugauU...................................................................................   1   1   S04
..........gaauauuccgacggauugaCg...................................................................................   1   1   S04
..........gaauauuccgacggauugaugC..................................................................................   10  1   S04
..........gaauauuccgacggauugaugg..................................................................................   1   0   S04
..........gUauauuccgacggauugauggu.................................................................................   1   1   S04
...........aauauuccgacggauugau....................................................................................   4   0   S04
...........aauaCuccgacggauugaug...................................................................................   1   1   S04
...........aauauuccgacggauugaug...................................................................................   10  0   S04
...........aauauuccgacggauugauC...................................................................................   1   1   S04
...........aauauuccgacggauugauU...................................................................................   1   1   S04
...........aauauuccgacggauugaugg..................................................................................   3   0   S04
...........aauauuccgacggauugaugguuA...............................................................................   1   1   S04
............auauuccgacggauugau....................................................................................   1   0   S04
............auaCuccgacggauugaug...................................................................................   1   1   S04
```

Star **Mature**

```
aauuuccucggaauauuccgacggauugauguuccucggaauuccgucgguauauuccgaggaaauuccucgggaaauucugaggauuucauuuuccgucggaaugucccguca
............auauuccgacggauugauU...................................................................................  1   1   S04
............auauuccgacggauugaug...................................................................................  9   0   S04
............auauuccgacggauugaugguuA...............................................................................  1   1   S04
............auauuccgacggauugaugguucc..............................................................................  5   0   S04
..............auuccgacggauugaugguuccuU............................................................................  1   1   S04
.................ccgacggauugaugguuccu.............................................................................  1   0   S04
....................acggauugaugguuccucggaauu......................................................................  1   0   S04
....................acggauuAaugguuccucggaauu......................................................................  1   1   S04
.......................gauugaugguuccucggaauuc.....................................................................  1   0   S04
..............................gguuccucggaauuccgucA................................................................  1   1   S04
................................uuccucggaauuccgucggua.............................................................  1   0   S04
................................uuccucggaauuccgucgguaua...........................................................  1   0   S04
................................uuccucggaauuccgucgguauG...........................................................  4   1   S04
................................uuccucggaauuccgucgguauau..........................................................  1   0   S04
.................................uccucggaauuccgucggu..............................................................  1   0   S04
.................................uccucggaauuccgucggua.............................................................  1   0   S04
.................................ucAucggaauuccgucgguauauu.........................................................  1   1   S04
.................................uccucggaauuccgucgguauauu.........................................................  4   0   S04
..................................ccucggaauuccgucgguauauu.........................................................  3   0   S04
..................................ccucggaauuccgucgguauauuc........................................................  19  0   S04
...................................cucggaauuccgucggua.............................................................  6   0   S04
...................................cucggaauuccgucgguaua...........................................................  1   0   S04
...................................cucggaauuccgucgguauau..........................................................  3   0   S04
...................................cucggaauuccgucgguauauu.........................................................  60  0   S04
...................................cucggGauuccgucgguauauu.........................................................  1   1   S04
...................................cucggaauuccgucgguauauuc........................................................  23  0   S04
...................................cucggaauuccgucgguauauuG........................................................  1   1   S04
...................................Nucggaauuccgucgguauauucc.......................................................  1   1   S04
...................................cucggaauuccgucgguauauucc.......................................................  29  0   S04
....................................ucggaauuccgucgguau............................................................  2   0   S04
....................................ucggaauuccgucgguaGa...........................................................  1   1   S04
....................................ucggaauuccgucgguaua...........................................................  4   0   S04
....................................ucggaauuccgucgguauau..........................................................  2   0   S04
....................................ucggaauuccgucgguauauu.........................................................  30  0   S04
....................................ucggaauuccgucgguauauuc........................................................  85  0   S04
....................................Ncggaauuccgucgguauauuc........................................................  1   1   S04
....................................ucggaauuccgGcgguauauuc........................................................  1   1   S04
.....................................cggaauuccgucgguaua...........................................................  1   0   S04
.....................................cggaauuccgucgguauau..........................................................  1   0   S04
.....................................cggaauuccgucgguauauu.........................................................  8   0   S04
.....................................cggaauuccgucgguauauuG........................................................  1   1   S04
.....................................cggaauuccgucgguauauuc........................................................  23  0   S04
.....................................cggaauuccgucgguauauucc.......................................................  3   0   S04
.....................................cggaauuccgucgguauauuccC......................................................  1   1   S04
.....................................cggaauuccgucgguauauuccg......................................................  1   0   S04
......................................ggaauuccgucgguauau..........................................................  1   0   S04
......................................ggaauuccgucgguauauu.........................................................  13  0   S04
......................................ggaauuccgucgguauauuc........................................................  5   0   S04
......................................ggaauuccgucgguauauuccg......................................................  1   0   S04
......................................ggaauuccgucgguauauuccga.....................................................  2   0   S04
......................................ggaauuccgucgguauauuccgag....................................................  9   0   S04
.......................................gaauuccgucgguauauu.........................................................  1   0   S04
.......................................gaauuccgucgguauauuc........................................................  9   0   S04
.......................................gaauuccgucgguauauucc.......................................................  13  0   S04
.......................................gUauuccgucgguauauuccg......................................................  1   1   S04
.......................................gaauuccgucgguauauuccg......................................................  74  0   S04
.......................................gaauuccgucgguauauuccga.....................................................  10  0   S04
.......................................gaauuccgucgguauauuccgag....................................................  4   0   S04
.......................................gaauuccgucgguauauuccgagg...................................................  1   0   S04
........................................aauuccgucgguauauuc........................................................  2   0   S04
........................................aauuccgucgguauauucc.......................................................  16  0   S04
........................................Nauuccgucgguauauuccg......................................................  1   1   S04
........................................aauuccgucgguauauuccC......................................................  1   1   S04
........................................aauuccgucgguauauuccg......................................................  21  0   S04
........................................aauuccgGcgguauauuccg......................................................  1   1   S04
........................................aauuccgucgguauauuccga.....................................................  8   0   S04
........................................aauuccgucgguauauuccgag....................................................  3   0   S04
........................................aauuccgucgguauauuccgagg...................................................  2   0   S04
........................................aauuccgucgguauauuccgagga..................................................  8   0   S04
.........................................auuccgucgguauauucc.......................................................  9   0   S04
```

```
          Star                                                                    Mature
aauuuccucggaauauuccgacggauugaugguuccucggaauuccgucgguauauuccgaggaaauuccucgggaaauucugaggauuucauuuuccgucggaauguccguca
.........................................auucGgucgguauauucc.......................................................  1    1  S04
.........................................auuccgucgguauauuccg......................................................  12   0  S04
.........................................auuccgucgguauauuccgC.....................................................  1    1  S04
.........................................auuccgucgguauauuccgag....................................................  3    0  S04
.........................................auuccgucgguauauuccgagg...................................................  11   0  S04
..........................................uuccgucgguauauuccg......................................................  2    0  S04
..........................................uuccgucgguauauuccgag....................................................  1    0  S04
...........................................uccgucgguauauuccga.....................................................  1    0  S04
...............................................................aaauuccucgggaaauucuga..............................  1    0  S04
......................................................................ucgggaaauucugaggau..........................  1    0  S04
......................................................................ucgggaaauucugaggauuucauu....................  1    0  S04
............................................................................aauucugaggauuucauuuuccgu..............  1    0  S04
.............................................................................auucugaggauuucauuuuccguc.............  1    0  S04
..................................................................................gaggauuucauuuuccgucgg...........  2    0  S04
..................................................................................gaggauuucaCuuuccgucgga..........  1    1  S04
..................................................................................gaggauuucauuCuccgucgga..........  1    1  S04
..................................................................................gaggauuucauuuuccgucgga..........  6    0  S04
..................................................................................gaggauuucauuuuccgucggaa.........  4    0  S04
..................................................................................gaggauuucauuuuccgucggaaG........  1    1  S04
..................................................................................gaggauuucauuuGccgucggaau........  1    1  S04
..................................................................................gaggauuucauuuuccgucggaau........  66   0  S04
..................................................................................gaggauuucauuuuccgucggaaA........  1    1  S04
..................................................................................gaggauuucauuuuccgucggaaug.......  7    0  S04
..................................................................................gaggauuucauuuuccgucggaaugu......  1    0  S04
..................................................................................gaggauuucauuuuccgucggaauguc.....  1    0  S04
...................................................................................aggauuucauuuuccgucgg...........  1    0  S04
...................................................................................aggauuucauuuuccgucggC..........  1    1  S04
...................................................................................aggauuucauuuuccgucgga..........  11   0  S04
...................................................................................aggauuucauuuuccgucggaa.........  2    0  S04
...................................................................................aggauuucaCuuuccgucggaau........  1    1  S04
...................................................................................aggauuucauuuuccgucggaau........  3    0  S04
...................................................................................aggaAuucauuuuccgucggaau........  1    1  S04
...................................................................................aggauuucauuuuccgucggUaug.......  1    1  S04
...................................................................................Uggauuucauuuuccgucggaaug.......  1    1  S04
...................................................................................aggauuucaCuuuccgucggaaug.......  2    1  S04
...................................................................................aggauuucauCuuccgucggaaug.......  1    1  S04
...................................................................................aggauCucauuuuccgucggaaug.......  2    1  S04
...................................................................................aggauuucauuCuccgucggaaug.......  4    1  S04
...................................................................................aggauuucauuuuccgucggaaGg.......  2    1  S04
...................................................................................aggauuucauuuuccgucggaaug.......  463  0  S04
...................................................................................aggauuucauuuuccgucggaaugA......  1    1  S04
...................................................................................aggauuucauuuuccgucggaaugu......  1    0  S04
....................................................................................ggauuucauuuuccgucgg...........  3    0  S04
....................................................................................ggauuucauuuuccgucgga..........  6    0  S04
....................................................................................ggauuucauuuuccgucggaa.........  2    0  S04
....................................................................................ggauuucauuuuccgucggaau........  9    0  S04
....................................................................................ggauuucauuuuccgucggaaug.......  74   0  S04
....................................................................................Ngauuucauuuuccgucggaaug.......  1    1  S04
....................................................................................ggauuucauuCuccgucggaaug.......  1    1  S04
....................................................................................ggauuucauCuuccgucggaaugu......  1    1  S04
....................................................................................ggauuucauuuAccgucggaaugu......  1    1  S04
....................................................................................ggauuucauuuuccgucggaaugu......  134  0  S04
....................................................................................ggauuucauuCuccgucggaaugu......  1    1  S04
....................................................................................ggauuucauuuuccgucggaaugA......  8    1  S04
....................................................................................ggauuucauuuuccgucggaaugC......  19   1  S04
....................................................................................ggauuucauuuuccgucggaaugG......  7    1  S04
....................................................................................ggauuucauuuuccgucggaauguG.....  2    1  S04
.....................................................................................gauuucauuuuccgucgg...........  1    0  S04
.....................................................................................gauuucauuuuccgucgga..........  4    0  S04
.....................................................................................gauuucauuuuccgucggU..........  1    1  S04
.....................................................................................gauuucauuuuccgucggaC.........  1    1  S04
.....................................................................................gauuucauuuuccgucggaa.........  5    0  S04
.....................................................................................gauuucauuuuccgucggaau........  6    0  S04
.....................................................................................gauGucauuuuccgucggaaug.......  1    1  S04
.....................................................................................gauuucauuuuccgucggaauC.......  1    1  S04
.....................................................................................gauuucauuuuccgucggaaug.......  177  0  S04
.....................................................................................gCuuucauuuuccgucggaaug.......  1    1  S04
.....................................................................................gauuucauuuuccgucggaaugA......  8    1  S04
.....................................................................................gauuucauuuuccgucggaaugC......  2    1  S04
.....................................................................................gauuucauuuuccgucggaaugu......  6    0  S04
```

Star Mature

aauuuccucggaauauuccgacggauugaugguuccucggaauuccgucgguauauuccgaggaaauuccucgggaaauucugaggauuucauuuuccgucggaaugucaguca			
....gauuucauuuuccgucggaaugGc.....	2	1	S04
....gauuucauuuuccgucggUauguc.....	1	1	S04
....gauuucauuuuccgucggaauguc.....	563	0	S04
....gauCucauuuuccgucggaauguc.....	1	1	S04
....gauuucauuuuccgucggaauguG.....	5	1	S04
....gauuucauuuuccgucggaaugCc.....	3	1	S04
....gauuucaCuuuccgucggaauguc.....	3	1	S04
....gauuucauuCuccgucggaauguc.....	1	1	S04
....gauuucauuAuccgucggaauguc.....	1	1	S04
....gauuucauuuAccgucggaauguc.....	2	1	S04
....auuucauuuuccgucgga..........	4	0	S04
....auuucauuuuccgucggaa.........	2	0	S04
....auuucauuuuccgucggaau........	6	0	S04
....auuucauuuuccgucggaaug.......	114	0	S04
....auGucauuuuccgucggaaug.......	1	1	S04
....aGuucauuuuccgucggaaug.......	1	1	S04
....auuucauuuuccgucggaaugA......	10	1	S04
....auuucaCuuuccgucggaaugu......	1	1	S04
....Nuuucauuuuccgucggaaugu......	1	1	S04
....auuucauuuuccgucggaaugu......	54	0	S04
....auCucauuuuccgucggaaugu......	1	1	S04
....auuucauuuuccgucggaaugC......	13	1	S04
....auuucauuuuccgucggaaugG......	7	1	S04
....auuucauuuuccgucggaauguG.....	1	1	S04
....auuucauuuGccgucggaauguc.....	1	1	S04
....auuucauuuuccgucgCaauguc.....	1	1	S04
....auuucauuuuccgucggaauguc.....	68	0	S04
....auuucauuCuccgucggaauguc.....	2	1	S04
....auuucauCuuccgucggaaugucc....	1	1	S04
....auuucauuuuccgucggaaugucG....	1	1	S04
....auuGcauuuuccgucggaaugucc....	1	1	S04
....auuucauuuuccgucggaaugucc....	239	0	S04
....aAuucauuuuccgucggaaugucc....	1	1	S04
....auuucauuCuccgucggaaugucc....	1	1	S04
....auCucauuuuccgucggaaugucc....	2	1	S04
....auuucaCuuuccgucggaaugucc....	2	1	S04
....auuucUuuuuccgucggaaugucc....	1	1	S04
....auuucauuuuccgucggaauguccg...	2	0	S04
....auuucauuuuccgucggaauguccgu..	1	0	S04
....uuucauuuuccgucggaa.........	2	0	S04
....uuucauuuuccgucggaau........	2	0	S04
....uuucauuuuccgucggaaug.......	20	0	S04
....uuucauuuuccgucggaaugG......	3	1	S04
....uuucauuuuccgucggaaugC......	2	1	S04
....uuucauuuuccgucggaaugu......	14	0	S04
....uuucauuuuccgGcggaaugu......	1	1	S04
....uuucauuuuccgucggaaugA......	3	1	S04
....uGucauuuuccgucggaauguc.....	1	1	S04
....uuGcauuuuccgucggaauguc.....	2	1	S04
....uuucaCuuuccgucggaauguc.....	1	1	S04
....Auucauuuuccgucggaauguc.....	3	1	S04
....uuucauuuuccguNggaauguc.....	1	1	S04
....uuAcauuuuccgucggaauguc.....	1	1	S04
....uuucauuCuccgucggaauguc.....	3	1	S04
....uuucauuuuccgucggaauguG.....	11	1	S04
....uuucauCuuccgucggaauguc.....	2	1	S04
....uuucauuuAccgucggaauguc.....	1	1	S04
....uuucauuuuccgucggaauCuc.....	1	1	S04
....uCucauuuuccgucggaauguc.....	3	1	S04
....uuucauuuuccgucggaauguc.....	385	0	S04
....uuucauuuuccgucggaaugGc.....	1	1	S04
....uuucauuuuccgucggaaugCc.....	3	1	S04
....uuucauuuuccgucggUauguc.....	1	1	S04
....uuucauuuuccgucggaaugucc....	24	0	S04
....uuucauCuuccgucggaaugucc....	1	1	S04
....uuucauuuuccgucggaaugucG....	3	1	S04
....uuucauuuuccgucggaauguccg...	118	0	S04
....uuucauuuuccgucggaauguccC...	2	1	S04
....uuGcauuuuccgucggaauguccg...	1	1	S04
....uuucauuuuccgucggaaugCccg...	1	1	S04

```
          Star                                                                        Mature
aauuuccucggaauauuccgacggauugaugguuccucggaauuccgucgguauauuccgaggaaauuccucgggaaauucugaggauuucauuuuccgucggaauguccguca
.......................................................................................uuucauuuuccgucggUauguccgu..   1     1   S04
.......................................................................................uuucauuuuccgucggaauguccgu..   100   0   S04
.......................................................................................uuucauuuuccgucggaauguccgG..   7     1   S04
.......................................................................................uuucauuuuccgucggaauguccgA..   2     1   S04
.......................................................................................uuucauuuuccgucggaaugCccgu..   1     1   S04
........................................................................................uucauuuuccgucggaau........   2     0   S04
........................................................................................uucauuuuccgucggaaug.......   2     0   S04
........................................................................................uucauuuuccgucggaaugC......   1     1   S04
........................................................................................uucauuuuccgucggaaugu......   1     0   S04
........................................................................................uucauuuuccgucggaaAguc.....   1     1   S04
........................................................................................uucauuuuccgucggaauguc.....   63    0   S04
........................................................................................Nucauuuuccgucggaauguc.....   1     1   S04
........................................................................................uucauuuuccgucggaauguG.....   3     1   S04
........................................................................................uucauuuuccgucggaaugucc....   38    0   S04
........................................................................................uGcauuuuccgucggaaugucc....   1     1   S04
........................................................................................uucauuuuccgucggaauguccg...   31    0   S04
........................................................................................uucauuuuccgucggaauguccC...   2     1   S04
........................................................................................uucauuuuccgucggaaugucGgu..   1     1   S04
........................................................................................uucauuuuccgucggaaugAccgu..   1     1   S04
........................................................................................uucauuuuccgucggaauCuccgu..   1     1   S04
........................................................................................uGcauuuuccgucggaauguccgu..   2     1   S04
........................................................................................uucauuuuccgucggaaugCccgu..   1     1   S04
........................................................................................uucauuuuccgucggaaugGccgu..   1     1   S04
........................................................................................Gucauuuuccgucggaauguccgu..   1     1   S04
........................................................................................uucauuuuccgucggaauguccgG..   39    1   S04
........................................................................................uucauuuuccgucggaauguccgu..   543   0   S04
........................................................................................uucauuuuccgucggaauguccCu..   3     1   S04
........................................................................................Nucauuuuccgucggaauguccgu..   1     1   S04
........................................................................................uucauuCuccgucggaauguccgu..   3     1   S04
........................................................................................uucauuuuccgGcggaauguccgu..   4     1   S04
........................................................................................uucauCuuccgucggaauguccgu..   2     1   S04
........................................................................................uucauuuuccgucggaauguccgA..   28    1   S04
........................................................................................uucauuuuccgucggaauguccguc.   1     0   S04
.........................................................................................ucauuuuccgucggaaug.......   2     0   S04
.........................................................................................ucauuuuccgucggaaugu......   4     0   S04
.........................................................................................ucauuuuccgucggaauguG.....   1     1   S04
.........................................................................................ucauuuuccgucggaauguc.....   17    0   S04
.........................................................................................ucauuCuccgucggaauguc.....   1     1   S04
.........................................................................................ucauuuuccgucggaaugucG....   1     1   S04
.........................................................................................ucauuuuccgucggaaugucc....   13    0   S04
.........................................................................................ucauuuuccgucggaauguccg...   30    0   S04
.........................................................................................ucauuuuccgucggaAguccgu..   1     1   S04
.........................................................................................ucauuuuccgucggaauguccgA..   1     1   S04
.........................................................................................ucauuuuccgucggaauguccgG..   2     1   S04
.........................................................................................ucauuuuccgucggaauguccgu..   36    0   S04
.........................................................................................Ncauuuuccgucggaauguccguc.   1     1   S04
.........................................................................................ucauuuuccgucggaauguccguc.   26    0   S04
.........................................................................................ucauCuuccgucggaauguccguc.   1     1   S04
..........................................................................................cauuuuccgucggaaugu......   5     0   S04
..........................................................................................cauuuuccgucggaauguc.....   2     0   S04
..........................................................................................cauuuuccgucggaaugucc....   3     0   S04
..........................................................................................cauuuuccgucggaauguccg...   20    0   S04
..........................................................................................cauuuuccgucggaaugGccgu..   1     1   S04
..........................................................................................cauuuuccgucggaauguccgu..   87    0   S04
..........................................................................................caCuuuccgucggaauguccgu..   1     1   S04
..........................................................................................cauuuuccgucggaauguccgA..   6     1   S04
..........................................................................................cauuuuccgucggaauguccgG..   4     1   S04
..........................................................................................Gauuuuccgucggaauguccgu..   1     1   S04
..........................................................................................cauuuuccgucggaauguccguc.   1     0   S04
..........................................................................................cauuuuccgucggaauguccguca   17    0   S04
...........................................................................................auuuuccgucggaauguc.....   3     0   S04
...........................................................................................auuuuccgucggaaugucc....   6     0   S04
...........................................................................................auuuuccgucggaauguccg...   8     0   S04
...........................................................................................auuuuccgucggaauguccC...   1     1   S04
...........................................................................................auuuuccgucggaauguccgG..   1     1   S04
...........................................................................................auuuuccgucggaauguccgA..   3     1   S04
...........................................................................................auuuuccgucggaauguccgu..   71    0   S04
...........................................................................................auuuuccgucggaauguccguc.   15    0   S04
...........................................................................................Nuuuuccgucggaauguccguc.   1     1   S04
...........................................................................................auuuuccgucggaauguccguca   70    0   S04
```

```
            Star                                                                           Mature
aauuuccucggaauauuccgacggauugaugguuccucggaauuccgucgguauauuccgaggaaauuccucgggaaauucugaggauuucauuuuccgucggaauguccguca
...........................................................................................auGuuccgucggaauguccguca	1	1	S04
...........................................................................................auuCuccgucggaauguccguca	1	1	S04
............................................................................................uuuuccgucggaaugucc....	3	0	S04
............................................................................................uuuuccgucggaauguccg...	6	0	S04
............................................................................................uuuuccgucggaaugucC...	1	1	S04
............................................................................................uuuuccgucggaauguccgG..	1	1	S04
............................................................................................uuuuccgucggaauguccgu..	21	0	S04
............................................................................................uuuuccgucggaauguccgA..	1	1	S04
............................................................................................uuuuccgucggaauguccguc.	3	0	S04
............................................................................................uuuuccgucggaauguccguca	13	0	S04
.............................................................................................uuuccgucggaauguccg...	3	0	S04
.............................................................................................uuuccgucggaauguccgG..	1	1	S04
.............................................................................................uuuccgucggaauguccgu..	7	0	S04
.............................................................................................uuuccgucggaauguccguca	3	0	S04
..............................................................................................Nuccgucggaauguccgu..	1	1	S04
..............................................................................................uuccgucggaauguccgu..	3	0	S04
..............................................................................................uuccgucggaauguccguca	3	0	S04
...............................................................................................uccgucggaauguccguc.	3	0	S04
...............................................................................................uccgucggaauguccguca	3	0	S04
................................................................................................ccgucggaauguccguca	4	0	S04
```

Provisional ID	:	chrC03_1383226
Score total	:	43.1
Score for star read(s)	:	3.9
Score for read counts	:	39.4
Score for mfe	:	-0.1
Score for randfold	:	0
Score for cons. seed	:	
Total read count	:	89
Mature read count	:	83
Loop read count	:	0
Star read count	:	6

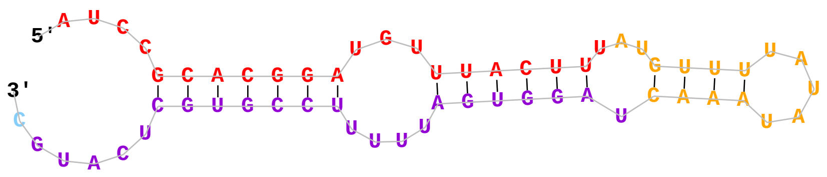

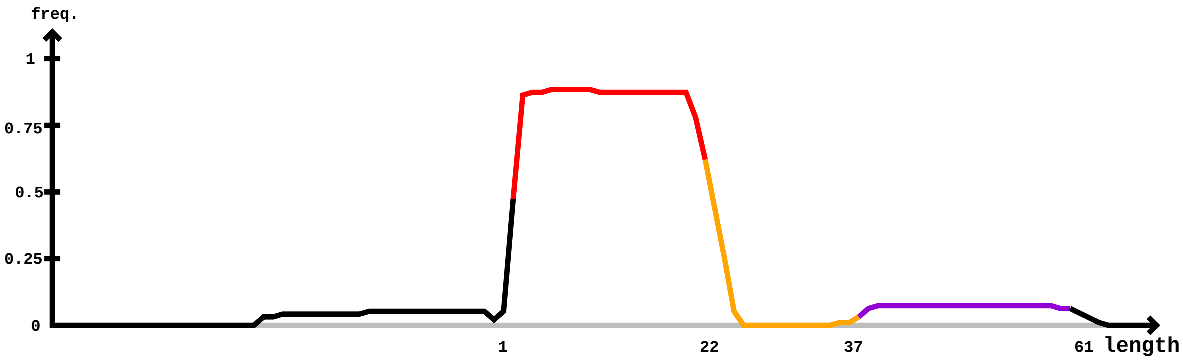


```
                                            Mature                            Star
5'- cacuuucgagcauuucuuuuaaugacaaguagauuuugaacccugcauccgcacggauguuuacuuuauguuuuauauaaacuaggugauuuuuccgugcucaugcacaugua  -3'   obs
    cacuuucgagcauuucuuuuaaugacaaguagauuuugaacccugcauccgcacggauguuuacuuuauguuuuauauaaacuaggugauuuuuccgugcucaugcacaugua         exp
    ....((((((.(((((((........))).))))))))))...(((((..(((((((...((((((...((((.....)))).))))))....)))))))..))))).......   reads   mm   sample
    ...............................gauuuugaacccugcauccgcacU..........................................................   1       1    S03
    .............................................cauccgcacggGuguuuacuu...............................................   1       1    S03
    .............................................cauccgcacggGuguuuacuuu..............................................   1       1    S03
    .............................................cauccgcacggGuguuuacuuua.............................................   1       1    S03
    ..............................................auccgcacggGuguuuacu................................................   1       1    S03
    ..............................................auccgcacggGuguuuacuu...............................................   5       1    S03
    ..............................................auccgcacggGuguuuacuuu..............................................   9       1    S03
    ..............................................auccgcacggGuguuuacuuua.............................................   4       1    S03
    ..............................................auccgcacggGuguuuacuuuau............................................   4       1    S03
    ..............................................auccgcacggauguuuacuuuauU...........................................   1       1    S03
    ...............................................uccgcacggGuguuuacu................................................   5       1    S03
    ...............................................uccgcacggGuguuuacuu...............................................   1       1    S03
    ...............................................uccgcacggGuguuuacuuu..............................................   3       1    S03
    ...............................................uccgcacggauguuuacuuua.............................................   1       0    S03
    ...............................................uccgcacggGuguuuacuuua.............................................   1       1    S03
    ...............................................uccgcacggGuguuuacuuuau............................................   4       1    S03
    ...............................................uccgcacggGuguuuacuuuaug...........................................   1       1    S03
    ................................................ccgcacggGuguuuacuuuaug...........................................   1       1    S03
    ..................................................................................uaggugauuuuuccgugcucaug........   1       0    S03
    ..................................................................................uaggugauuuuuccgugcucauU........   1       1    S03
    ...................................................................................aggugauuuuuccgugcucaugcaA.....   1       1    S03
    ....................................................................................ggugauuuuuccgugcucaugca......   1       0    S03

    ....................aaugacaaguagauuuugaacccu.....................................................................   2       0    S04
    ....................aaugacaaguagauuuugaacccA.....................................................................   1       1    S04
    ......................ugacaaguagauuuugaacccugc...................................................................   1       0    S04
    ..............................................auccgcacggGuguuuacuu...............................................   3       1    S04
    ..............................................auccgcacggGuguuuacuuu..............................................   7       1    S04
    ..............................................auccgcacggGuguuuacuuua.............................................   5       1    S04
    ..............................................auccgcacggGuguuuacuuuau............................................   2       1    S04
    ...............................................uccgcacggGuguuuacu................................................   3       1    S04
    ...............................................uccgcacggGuguuuacuu...............................................   5       1    S04
    ...............................................uccgcacggGuguuuacuuua.............................................   3       1    S04
```

```
                                              Mature                             Star
cacuuucgagcauuucuuuuaaugacaaguagauuuugaacccugcauccgcacggauguuuacuuuauguuuuauauaaacuaggugauuuuuccgugcucaugcacaugua
...............................................uccgcacggGuguuuacuuuau............................................     9     1     S04
...............................................uccgcacggauguuuacuuuaug...........................................     1     0     S04
..................................................gcacggGuguuuacuuuaug...........................................     1     1     S04
................................................................................acuaggugauuuuuccgugcucU..........     1     1     S04
...................................................................................aggugauuuuuccgugcucaug........     1     0     S04
...................................................................................aggugauuuuuAcgugcucaugc.......     1     1     S04
```

Provisional ID	:	chrC03_1387597
Score total	:	0.4
Score for star read(s)	:	3.9
Score for read counts	:	-3.3
Score for mfe	:	0
Score for randfold	:	0
Score for cons. seed	:	
Total read count	:	5
Mature read count	:	2
Loop read count	:	0
Star read count	:	3

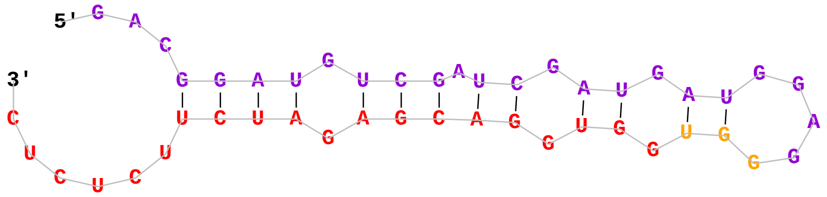

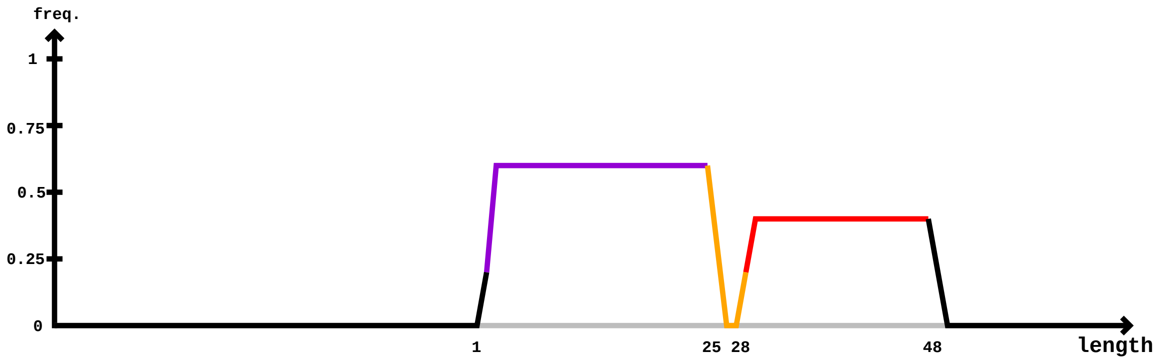


```
                                                    Star                            Mature
5'- uuaucauaauuaagguuuuugcuuacaguggaugaugguuucagacggaugucgaucgaugauggagggugguggacgagaucuucucucggggucaucauggguacuga   -3'    obs
    uuaucauaauuaagguuuuugcuuacaguggaugaugguuucagacggaugucgaucgaugauggagggugguggacgagaucuucucucggggucaucauggguacuga          exp
    ............((((....)))).(((((.(((((((..(((((.((((.(((.((.((.((.....)).)).))))).))))..))).))..)))))))...))))).   reads      mm        sample
    ............................................acggaugucgaucgaugauggag...........................................   1          0         S03
    ......................................................................gguggacgagaucuucucuc....................   1          0         S03

    ...........................................gacggaugucgaucgaugauggag...........................................   1          0         S04
    ............................................acggaugucgaucgaugauggagC..........................................   1          1         S04
    .......................................................................guggacgagaucuucucuc....................   1          0         S04
```

Provisional ID	:	chrC03_1394226
Score total	:	1
Score for star read(s)	:	-1.3
Score for read counts	:	0
Score for mfe	:	2.3
Score for randfold	:	0
Score for cons. seed	:	
Total read count	:	119
Mature read count	:	117
Loop read count	:	0
Star read count	:	2

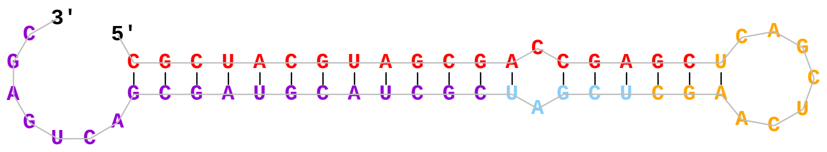

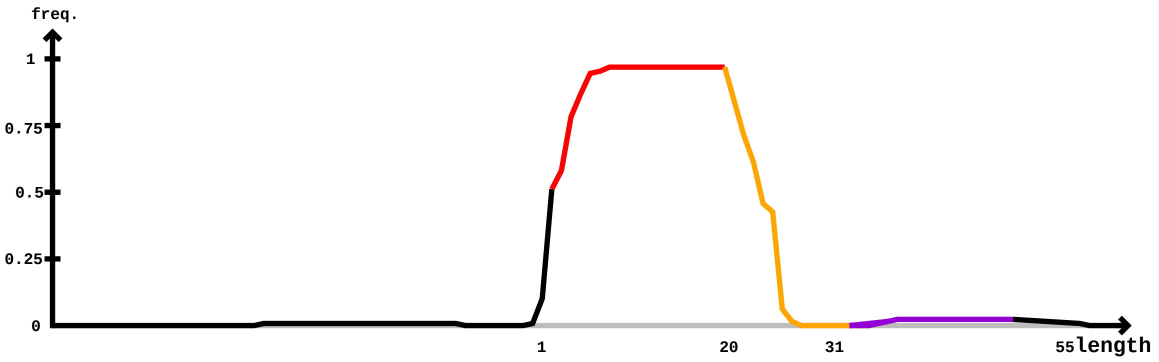

Mature **Star**

```
5'- uucgaguugcguuuuaggccucuuuggaccgagccgugugcgugcucguucgcuacguagcgaccgagcucagcucaagcucgaucgcuacguagcgacugagccaugua  -3'   obs
    uucgaguugcguuuuaggccucuuuggaccgagccgugugcgugcucguucgcuacguagcgaccgagcucagcucaagcucgaucgcuacguagcgacugagccaugua        exp
    ........(((.(((.((((.....)).))))).))).(((((((((.(((((((((((((.((((((.......)))))).))))))))))))).)))).))))))   reads   mm   sample
    ....................ucuuuggaccgagccgugugU.....................................................................   1       1    S03
    ................................................uucgcuacguagcgaccgagc.........................................   1       0    S03
    .................................................ucgcuacguagcgaccgagc.........................................   3       0    S03
    .................................................ucgcuacguagcgaccgagcu........................................   2       0    S03
    .................................................ucgcuacguagcgaccgagcuc.......................................   2       0    S03
    .................................................ucgcuacguagcgaccgagcucagU....................................   1       1    S03
    ..................................................cgcuacguagcgaccgagc.........................................   17      0    S03
    ..................................................cgcuacguagcgCccgagcu........................................   1       1    S03
    ..................................................cgcuacguagcgaccgagcu........................................   2       0    S03
    ..................................................Ggcuacguagcgaccgagcuc.......................................   1       1    S03
    ..................................................cgcuacguagcgaccgagcuc.......................................   7       0    S03
    ..................................................cgcuacguagcgaccgagcuAa......................................   1       1    S03
    ..................................................cgcuacguagUgaccgagcuca......................................   1       1    S03
    ..................................................cgcuacguagcgaccgagcuca......................................   6       0    S03
    ..................................................cgcuacguagcgaccgagcucag.....................................   1       0    S03
    ..................................................cgcuacguagcgaccgagcucagc....................................   7       0    S03
    ..................................................cgcuacguagcgaccgagcucagU....................................   2       1    S03
    ..................................................cgcuacguagcgaccgagcucagA....................................   1       1    S03
    ...................................................gcuacguagcgaccgagc.........................................   1       0    S03
    ...................................................gcuacguagcgaccgagcuc.......................................   1       0    S03
    ...................................................gcuacguagcgaccgagcuca......................................   3       0    S03
    ...................................................gcuacguagcgaccgagcucag.....................................   2       0    S03
    ...................................................gcuacguagcgaccgagcucagc....................................   1       0    S03
    ....................................................cuacguagcgaccgagcu........................................   2       0    S03
    ....................................................cuacguagcgaccgagcuca......................................   5       0    S03
    ....................................................cuacguagcgaccgagcucagc....................................   10      0    S03
    ....................................................cuacguagcgaccgagcucagU....................................   4       1    S03
    ....................................................cuacguagcgaccgagcucagcu...................................   1       0    S03
    .....................................................uacguagcgacUgagcucagc....................................   1       1    S03
    .....................................................uacguagcgaccgagcucagc....................................   4       0    S03
    .....................................................uacguagcgaccgagcucagU....................................   1       1    S03
    .....................................................uacguagcgaccgagcucagcu...................................   1       0    S03
    .....................................................Cacguagcgaccgagcucagcu...................................   1       1    S03
```

```
                                                   Mature                            Star
uucgaguugcguuuuaggccucuuuggaccgagccgugugcgugcucguucgcuacguagcgaccgagcucagcucaagcucgaucgcuacguagcgacugagccaugua
.....................................................uacguagcgaccgagcucagcuU..................................   1   1   S03
......................................................acguagcgaccgagAucagc....................................   1   1   S03
......................................................acguagcgaccgagcucagc....................................   5   0   S03
......................................................acguagcgaccgagcucagA....................................   1   1   S03
......................................................acguagcgaccgagcucagcu...................................   2   0   S03
.......................................................cguagcgaccgagcucagc....................................   1   0   S03
........................................................guagcgaccgagcucagA....................................   1   1   S03
........................................................guagcgaccgagcucagcuc..................................   1   0   S03
......................................................................................gcuacguagcgacugagc......   1   0   S03

.................................................ucgcuacguagcgaccgagc.........................................   1   0   S04
.................................................ucgcuacguagcgaccgagcu........................................   1   0   S04
.................................................ucgcuacguagcgaccgagcuca......................................   1   0   S04
.................................................ucgcuacguagUgaccgagcuca......................................   1   1   S04
..................................................cgcuacguagcgaccgagc.........................................   1   0   S04
..................................................cgcuacguagcgaccgagcu........................................   1   0   S04
..................................................cgcuacguagcgaccgagcuc.......................................   1   0   S04
..................................................cgcuGcguagcgaccgagcuca......................................   1   1   S04
..................................................cgcuacguagcgaccgagcucUg.....................................   1   1   S04
..................................................cgcuacguagcgaccgagcucagA....................................   1   1   S04
...................................................gcuacguagcgaccgagcuca......................................   1   0   S04
....................................................cuacguagcgaccgagcucagc....................................   3   0   S04
....................................................cuacguagcgaccgagcucagcu...................................   1   0   S04
.....................................................uacguagcgaccgagcuc.......................................   1   0   S04
.....................................................uacguagcgaccgagcucagc....................................   1   0   S04
......................................................acguagcgaccgagcucagc....................................   1   0   S04
....................................................................................ucgcuacguagcgacugagccG....   1   1   S04
.....................................................................................cgcuacguagcgacugagc......   1   0   S04
```

Provisional ID	:	chrC03_1394328
Score total	:	129.6
Score for star read(s)	:	3.9
Score for read counts	:	126.6
Score for mfe	:	-0.8
Score for randfold	:	0
Score for cons. seed	:	
Total read count	:	260
Mature read count	:	257
Loop read count	:	2
Star read count	:	1

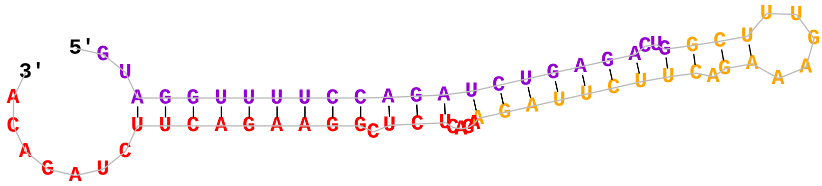

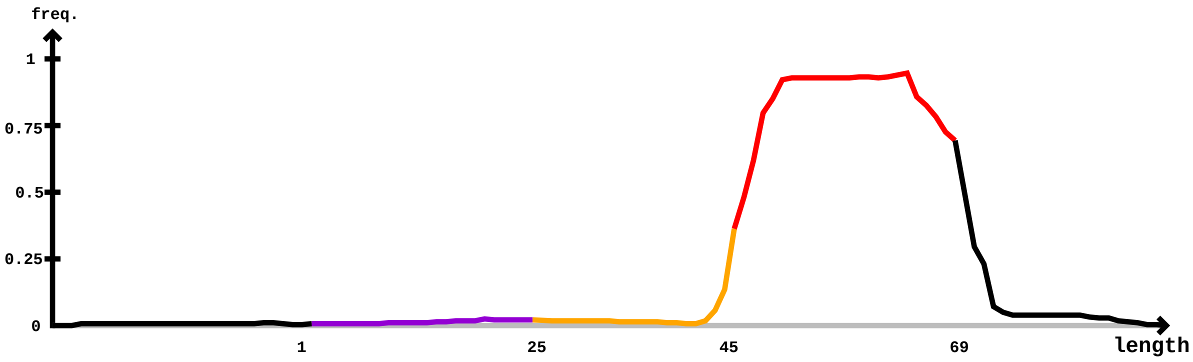

Star Mature

```
5'- aagccauuugaauguuuuugaauuuguagguuuuccagaucugagacuggcuuugaaagacuucuuagaagacucucggaagacuucuagacaagucuucuaaugcauuuuaug   -3'   obs
    aagccauuugaauguuuuugaauuuguagguuuuccagaucugagacuggcuuugaaagacuucuuagaagacucucggaagacuucuagacaagucuucuaaugcauuuuaug         exp
    ....(((..((((((....((((((((((((((((((((((((((((..((((.....)).)))))))))....))).)))))))))....))))))..))....)))))).)))  reads   mm   sample
    .................................uAcagaucugagacuggcuuugaa.........................................................  1       1    S03
    ......................................aucugagacuggcuuugaaagacu....................................................  1       0    S03
    ...........................................agacugAcuuugaaagacuucuu................................................  1       1    S03
    ...........................................agacugAcuuugaaagacuucuua...............................................  1       1    S03
    ..................................................................agaagacucucggaaUacuucuag........................  1       1    S03
    ...................................................................gaagacucucggaagacuucuag........................  1       0    S03
    ...................................................................gaagacucuUggaagacuucuag........................  1       1    S03
    ...................................................................gaagacucucggaaUacuucuag........................  1       1    S03
    ...................................................................gaagacucucggaaUacuucuaga.......................  5       1    S03
    ....................................................................aagacucucggaagacuucu..........................  2       0    S03
    ....................................................................aagacucucggaaUacuucua.........................  1       1    S03
    ....................................................................aagacucucggaaUacuucuag........................  4       1    S03
    ....................................................................aagacucucggaagacuucuaga.......................  1       0    S03
    ....................................................................aagacucucggaagacuucuUgac......................  4       1    S03
    .....................................................................agacucucggaagacuucu..........................  3       0    S03
    .....................................................................agacucucggaaUacuucuag........................  3       1    S03
    .....................................................................agacucucggaaUacuucuaga.......................  7       1    S03
    .....................................................................agacucucggaaUacuucuagaca.....................  11      1    S03
    .....................................................................agacucucggaagacuucuagaca.....................  1       0    S03
    ......................................................................gacucucggaagacuucu..........................  8       0    S03
    ......................................................................gacucucggaagacuucuG.........................  1       1    S03
    ......................................................................gacucucggaaUacuucuaga.......................  1       1    S03
    ......................................................................gacucucggaaUacuucuagaca.....................  7       1    S03
    ......................................................................gacucucggaagacuucuagacG.....................  1       1    S03
    .......................................................................acucucggaaUacuucuagaca.....................  3       1    S03
    .......................................................................acucucggaaUacuucuagacaag...................  7       1    S03
    .......................................................................acucucggaaUacuucuagacaagu..................  1       1    S03
    ........................................................................cucucggaaUacuucuagaca.....................  10      1    S03
    ........................................................................cucucggaaUacuucuagacaag...................  1       1    S03
    ........................................................................cucucggaagacuucuagacaagu..................  1       0    S03
    ........................................................................cucucggaaUacuucuagacaagu..................  18      1    S03
    .........................................................................ucucggaaUacuucuagaca.....................  3       1    S03
    .........................................................................ucucggaaUacuucuagacaagu..................  2       1    S03
```

```
                         Star                                           Mature
aagccauuugaauguuuuugaauuuguagguuuuccagaucugagacuggcuuugaaagacuucuuagaagacucucggaagacuucuagacaagucuucuaaugcauuuuaug
.........................................................................ucucggaaUacuucuagacaaguc.................   1   1   S03
..........................................................................cucggaaUacuucuagaca.....................   2   1   S03
..........................................................................cucggaGgacuucuagaca.....................   1   1   S03
..........................................................................cucggaaUacuucuagacaagu..................   6   1   S03
..........................................................................cucggaaUacuucuagacaagucu................   1   1   S03
..................................................................................acuucuagacaagucuucuaaugc........   1   0   S03
.....................................................................................ucuagacaagucuucuaaugc........   1   0   S03
.....................................................................................ucuagacaagucuucuaaugcauu.....   1   0   S03
.......................................................................................uagacaagucuucuaaugcauu.....   1   0   S03
........................................................................................agacaagucuucuaaugcauuuua..   1   0   S03

.agccauuugaaCguuuuugaa............................................................................................   1   1   S04
.agccauuugaaCguuuuugaau...........................................................................................   1   1   S04
....................aauuuguagguuuuccagaucuga......................................................................   1   0   S04
.........................guagguuuuUcagaucugagacug.................................................................   1   1   S04
........................................cugagacuggcuuugaaagacuuc..................................................   1   0   S04
..................................................................agaagacucucggaagac..............................   1   0   S04
..................................................................agaagacucucggaagacu.............................   1   0   S04
..................................................................agaagacucucggaagacuucuagaca.....................   1   0   S04
...................................................................gaagacucucggaaUacuucua.........................   1   1   S04
...................................................................gaagacucucggaaUacuucuag........................   1   1   S04
...................................................................gaagacucucggaaUacuucuaga.......................   1   1   S04
...................................................................gaagacucucggaagacuucuagaca.....................   1   0   S04
....................................................................aagacucucggaagacuucu..........................   4   0   S04
....................................................................aagacucucggaagacuucua.........................   2   0   S04
....................................................................aagacucucggaagacuucuagaG......................   1   1   S04
....................................................................aagacucucggaagacuucuagac......................   1   0   S04
....................................................................aagacucucggaaUacuucuagac......................   1   1   S04
....................................................................aagacucucggaagacuucuagaca.....................   1   0   S04
.....................................................................agacucucggaagacuucua.........................   1   0   S04
.....................................................................agacucucggaagacuucuag........................   1   0   S04
.....................................................................agacucucgUaagacuucuaga.......................   1   1   S04
.....................................................................agacucucggaagacuucuaga.......................   1   0   S04
.....................................................................agacucucggaagacuucuagac......................   1   0   S04
.....................................................................agacucucggaagacuucuUgac......................   1   1   S04
.....................................................................agacucucggGagacuucuagaca.....................   1   1   S04
.....................................................................agGcucucggaagacuucuagaca.....................   1   1   S04
.....................................................................aUacucucggaagacuucuagaca.....................   1   1   S04
.....................................................................agacucucggaagacuucuagacG.....................   1   1   S04
.....................................................................agacucucggaaUacuucuagaca.....................   3   1   S04
.....................................................................agacucucggaagacuucuagacU.....................   1   1   S04
.....................................................................agacucucAgaagacuucuagaca.....................   1   1   S04
.....................................................................agacucucggaagacuucuagaca.....................   23  0   S04
.....................................................................agacucucggaagacuucuaAaca.....................   1   1   S04
......................................................................gacucucggaagacuucu..........................   10  0   S04
......................................................................gacucucggaagacuucua.........................   1   0   S04
......................................................................gacucucggaagacuucuagaca.....................   3   0   S04
......................................................................gacucucggaagacuucCagacaa....................   1   1   S04
.......................................................................acucucggaagacuucua.........................   2   0   S04
.......................................................................acucucggaagacuucuagacG.....................   1   1   S04
.......................................................................acucucggaagacuucuagaca.....................   15  0   S04
.......................................................................acucucggaagacuucuagacU.....................   1   1   S04
.......................................................................acucucggaagacuucuagacaa....................   1   0   S04
.......................................................................acucucggaagacuucuagacaag...................   8   0   S04
........................................................................cucucggaagacuucuagaca.....................   9   0   S04
........................................................................cucucggaagacuucuagacG.....................   1   1   S04
........................................................................cucucggaagacuucuagacaag...................   2   0   S04
........................................................................cucucggaaUacuucuagacaagu..................   2   1   S04
........................................................................cucucggaagacuucuagacaagG..................   1   1   S04
........................................................................cucucggaagacuucuagacaagu..................   5   0   S04
.........................................................................ucucggaagacuucuagaca.....................   1   0   S04
.........................................................................ucucggaagacuucuagacU.....................   1   1   S04
.........................................................................ucucggaagacuucuagacaagu..................   1   0   S04
.........................................................................ucucggaagacuucuagacaaAu..................   1   1   S04
.........................................................................ucucggaagacuucuagacaaguc.................   4   0   S04
.........................................................................ucucggaagacuucuagacaagucA................   1   1   S04
..........................................................................cucggaagacuucuagaca.....................   3   0   S04
..........................................................................cucggaagacuucuagacaagu..................   5   0   S04
..........................................................................cucggaagacuucuagacaaguA.................   1   1   S04
..........................................................................cucggaagacuucuagacaagucu................   1   0   S04
```

Star Mature

```
aagccauuugaauguuuuugaauuuguagguuuuccagaucugagacuggcuuugaaagacuucuuagaagacucucggaagacuucuagacaagucuucuaaugcauuuuaug
...........................................................................ucggaagacuucuagacaagC..................	1	1	S04
...........................................................................ucggaagacuucuagacaagu..................	1	0	S04
......................................................................................cuagacaagucuucuaaugca.......	1	0	S04
......................................................................................cuagacaagucuucuaaugcauu.....	1	0	S04
.......................................................................................uagacaagucuucuaaugcauuuu...	1	0	S04
........................................................................................agacaagucuucuaaugcauuu....	1	0	S04
..........................................................................................acaagucuucuaaugcauuuuaug	1	0	S04
...........................................................................................caagucuucuaaugcauuuua..	1	0	S04
```

Provisional ID	:	chrC03_1412233
Score total	:	0.3
Score for star read(s)	:	3.9
Score for read counts	:	-4.9
Score for mfe	:	1.3
Score for randfold	:	0
Score for cons. seed	:	
Total read count	:	2
Mature read count	:	1
Loop read count	:	0
Star read count	:	1

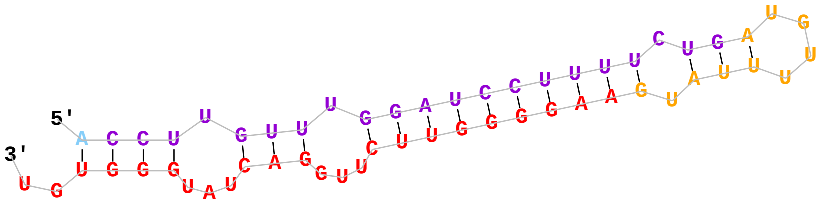

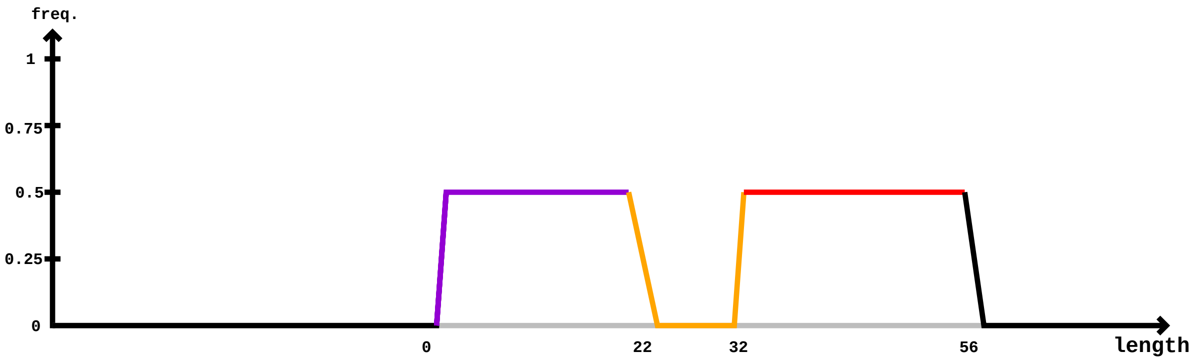

Star Mature

```
5'- agcgacuaguucguucgcggaccggucgcuacguggugaccuuguuuggauccuuuucugauguuuuaugaagggguucuuggacuaugggugu uuaguuucgaugaaucaacc   -3'   obs
    agcgacuaguucguucgcggaccggucgcuacguggugaccuuguuuggauccuuuucugauguuuuaugaagggguucuuggacuaugggugu uuaguuucgaugaaucaacc         exp
    ((((((..(((((....)))))..))))))........((((.(((.((((((((((.(((....))).))))))))))...)))...))))......................   reads   mm   sample
    ......................................................................aagggguucuuggacuaugggugu....................   1       0    S03

    .......................................ccuuguuuggauccuuuucuA......................................................   1       1    S04
```

Provisional ID	:	chrC03_1413212
Score total	:	0.2
Score for star read(s)	:	-1.3
Score for read counts	:	0
Score for mfe	:	1.5
Score for randfold	:	0
Score for cons. seed	:	
Total read count	:	12
Mature read count	:	12
Loop read count	:	0
Star read count	:	0

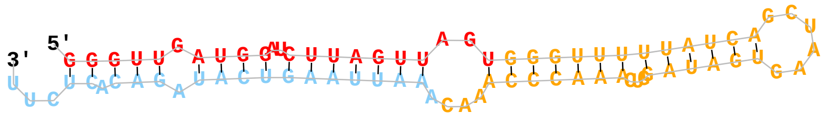

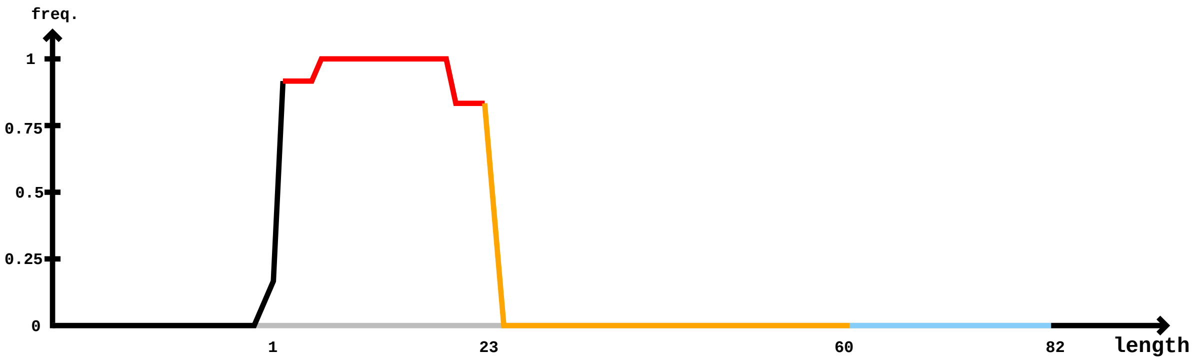

Mature Star

```
5'- gaggugagggcuuaggagauaaggguugauggaucuuaguuaguggguuuuuaucagcuaagugauaggucaaacccaaacaaauuaagucauagacacucuugugccaggccc   -3'    exp
    .......(((((.((..((((((((..((((..(((((((..((((((((((((((......))))))...)))))))....)))))))))))....)))))))).))))))))   reads    mm    sample
    .....................Uggguugauggaucuuagu...........................................................................   1        1     S03
    ......................gggAugauggaucuuaguuagu......................................................................   3        1     S03

    ....................aaggguugauggaucuuaguuagu......................................................................   1        0     S04
    ......................ggguugauggaucuuagu..........................................................................   1        0     S04
    ......................gggAugauggaucuuaguuagu......................................................................   5        1     S04
    ..........................ugauggaCcuuaguuagu......................................................................   1        1     S04
```

Provisional ID	:	chrC03_1419065
Score total	:	0
Score for star read(s)	:	3.9
Score for read counts	:	-4.9
Score for mfe	:	1
Score for randfold	:	0
Score for cons. seed	:	
Total read count	:	2
Mature read count	:	1
Loop read count	:	0
Star read count	:	1

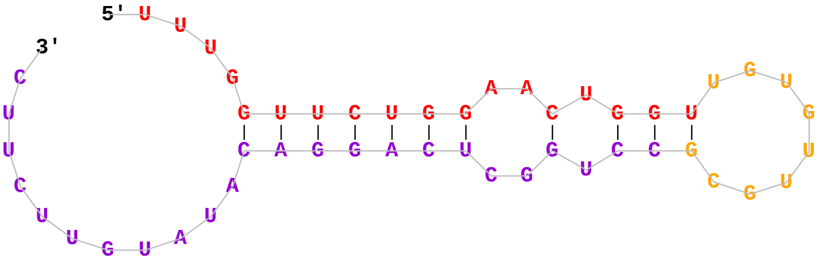

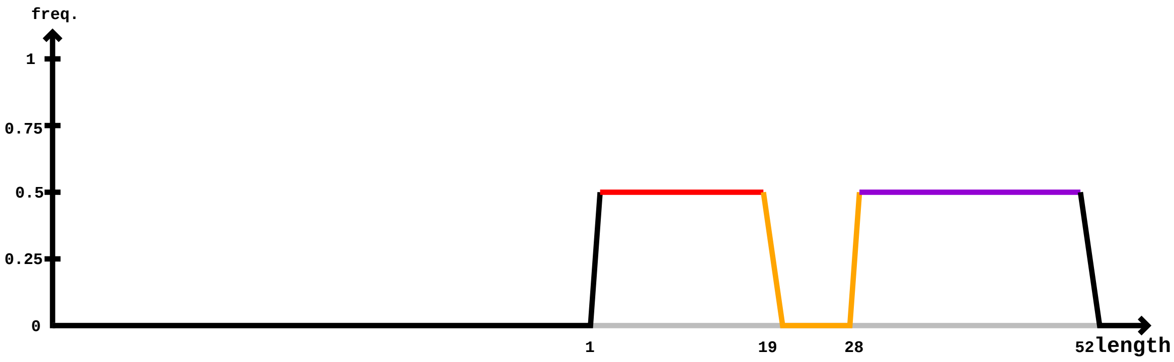

Mature Star

```
5'- ucgucuuggggagcaguuguuggcgaaggagcgguuucuucagcagucugcugcuuuugguucuggaacugguuguguugcgccuggcucaggacauauguucuuccuuggu  -3'  obs
    ucgucuuggggagcaguuguuggcgaaggagcgguuucuucagcagucugcugcuuuugguucuggaacugguuguguugcgccuggcucaggacauauguucuuccuuggu        exp
    .....(..(((((((((((((((.((((......)))).))))))).))))........(((((((..(.(((........))).)..))))))).........))))..).   reads    mm    sample
    .......................................................Guugguucuggaacuggu.......................................   1        1     S03

    ..................................................................................cUuggcucaggacauauguucuuc......   1        1     S04
```

Provisional ID	:	chrC03_1421586
Score total	:	16.9
Score for star read(s)	:	3.9
Score for read counts	:	13.9
Score for mfe	:	-0.8
Score for randfold	:	0
Score for cons. seed	:	
Total read count	:	39
Mature read count	:	36
Loop read count	:	0
Star read count	:	3

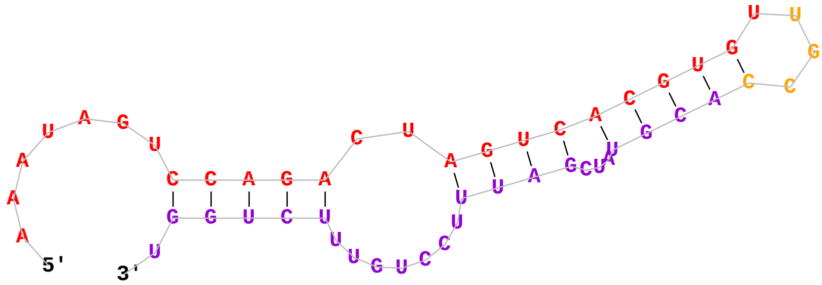

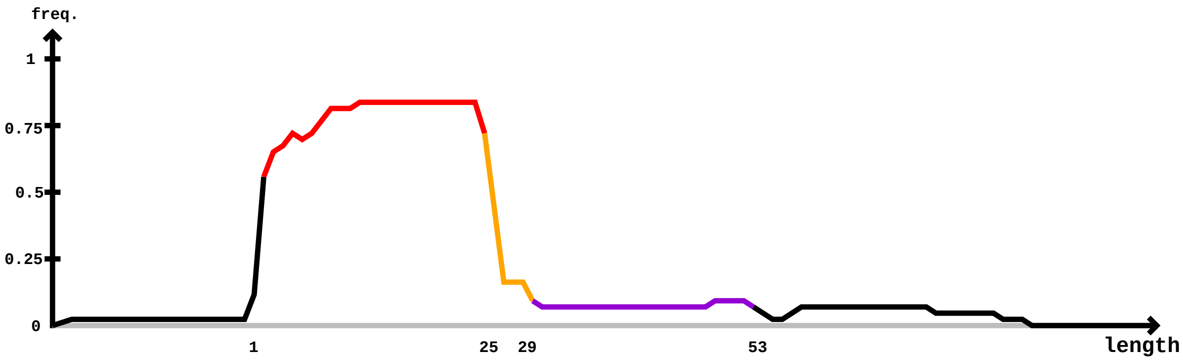

Mature Star

```
5'- aggauauucuggccccacugaaauaguccagacuagucacguguugccacguaucgauuuccuguuucuggugacgccgaaauguuaauucuccaguagccgagauucaaauc  -3'   obs
    aggauauucuggccccacugaaauaguccagacuagucacguguugccacguaucgauuuccuguuucuggugacgccgaaauguuaauucuccaguagccgagauucaaauc         exp
    ..((((((.((((..(((((((((((....((..(((((((((....)))))...)))))))))))).))))..))))).))))))...((((........))))........    reads    mm    sample
    ...................gaaauaguccagacuagucacgug......................................................................    1        0     S03
    ....................aaauaguccagacuagucacgugu.....................................................................    4        0     S03
    ....................Naauaguccagacuagucacgugu.....................................................................    1        1     S03
    ......................auaguccagacuagucacguguu....................................................................    1        0     S03
    .......................Aaguccagacuagucacguguugc..................................................................    1        1     S03
    ..........................uccagacuagucacguguugcA.................................................................    1        1     S03
    ...........................ccagacuagucacguguugcA.................................................................    1        1     S03
    ..............................gacuagucacguguugcA.................................................................    1        1     S03
    ................................................acguaucgauuuccuguuucuggC.........................................    1        1     S03
    ...................................................................cuggugacgccgaaauguuaauu.......................    1        0     S03
    ............................................................................ccgaaauguuaauucuccaguagc.............    1        0     S03

    aggauauucuggccccacugaaau.........................................................................................    1        0     S04
    ...................gaaauaguccGgacuagucacgug......................................................................    1        1     S04
    ...................gaaauaguccagacuagucacgug......................................................................    2        0     S04
    ....................aaauaguccagacuagucacgugA.....................................................................    4        1     S04
    ....................aUauaguccagacuagucacgugu.....................................................................    1        1     S04
    ....................aaauaguccagacuagucacgugC.....................................................................    2        1     S04
    ....................aaauaguccagacuagucacgugu.....................................................................    7        0     S04
    .....................aauaguccagacuagucacgug......................................................................    1        0     S04
    .....................aauaguccagacuagucacgugu.....................................................................    1        0     S04
    .....................aauaguccagacuagucacguguu....................................................................    1        0     S04
    .....................aauaguccagacuagucacguguG....................................................................    1        1     S04
    .......................uaguccagacuagucacguguu....................................................................    1        0     S04
    .........................guccagacuagucacguguugcA.................................................................    1        1     S04
    ..........................uccagacuagucacguguugccG................................................................    1        1     S04
    ...........................ccagacuagucUcguguugcc.................................................................    1        1     S04
    ...............................................cacgGaucgauuuccuguuucugg..........................................    1        1     S04
    ................................................acguaucgauuuccuguuucuggu.........................................    1        0     S04
    ...........................................................................gccgaaauguuaauucuccagu................    1        0     S04
```

Provisional ID	:	chrC03_1436279
Score total	:	24.7
Score for star read(s)	:	3.9
Score for read counts	:	23.1
Score for mfe	:	-2.2
Score for randfold	:	0
Score for cons. seed	:	
Total read count	:	57
Mature read count	:	48
Loop read count	:	8
Star read count	:	1

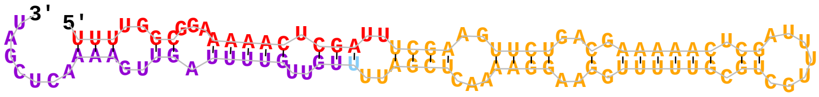

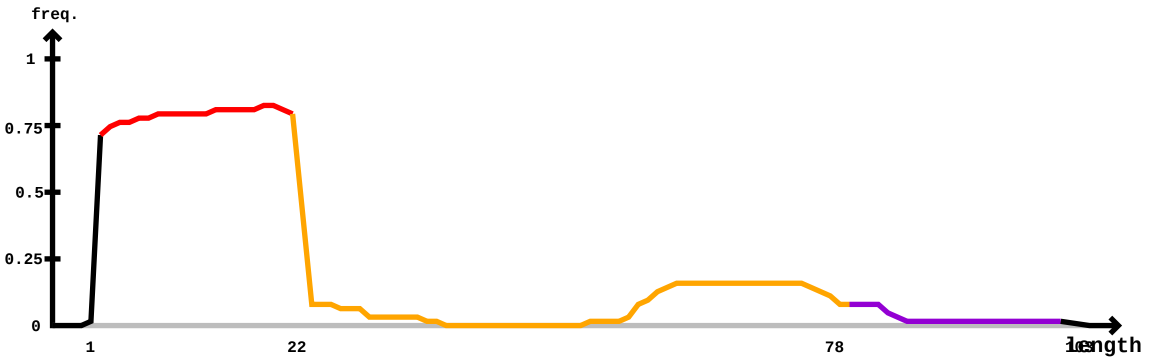

Mature **Star**

```
5'- auuuuuuggcggaaaaacucgauuucgaaguucugacgaaaaacucgauuuugcugcguuuuuggaaggaaaacucgauuuuguuguuuuaguugaaaacucgauuuug  -3'   obs
    auuuuuuggcggaaaaacucgauuucgaaguucugacgaaaaacucgauuuugcugcguuuuuggaaggaaaacucgauuuuguuguuuuaguugaaaacucgauuuug         exp
    ..((((..((...(((((.(((..((((..((((..(.((((((.((.......)).)))))).)..))))...))))..)))..))))).))..))))..........  reads     mm      sample
    ...uuuuggcggaaaaacucgau......................................................................................  1         0       S03
    ...uuuuggcggaaaaacucgauC.....................................................................................  3         1       S03
    ...uuuuggcggaaaaacucgauu.....................................................................................  15        0       S03
    ...uuuuggcggaaaaacucgauG.....................................................................................  1         1       S03
    ...uuuuggcggaaaaacucgauuA....................................................................................  2         1       S03
    .....uuggcggaaaaacucgauuuUga.................................................................................  1         1       S03
    .........cggaaaaacucgauuuUgaagu..............................................................................  1         1       S03
    ...............aacucgauuucgaaguucugac........................................................................  1         0       S03
    ...............................................................ggaaggaaaacucgauuuuguugu......................  1         0       S03
    .................................................................................uguuguuuuaguugaaaacucgau....  1         0       S03

    ..uuuuuggGggaaaaacucga.......................................................................................  1         1       S04
    ...uuuuggcggaaaaacucgauC.....................................................................................  2         1       S04
    ...uuuuggcggaaaaacucgauu.....................................................................................  17        0       S04
    ...uuuuggcggaaaaacucgauG.....................................................................................  1         1       S04
    ...uuuuggcggaaaaacucgauuu....................................................................................  2         0       S04
    ....uuuggcggaaaaacucgauu.....................................................................................  1         0       S04
    ....uuuggcggaaaaacucgauG.....................................................................................  1         1       S04
    .......ggcggaaaaacucgauuuUgaagu..............................................................................  1         1       S04
    ....................gauuucgaaguucugacga......................................................................  1         0       S04
    ......................................................ugcguuuuuggaaggaaaacucga...............................  1         0       S04
    ..........................................................uuuuuggaaggaaaacucgaC..............................  1         1       S04
    ...........................................................uuuuggaaggaaaacucg................................  1         0       S04
    ...........................................................uuuuggaaggaaaacucgauu.............................  2         0       S04
    ............................................................uuuggaaggaaaacucgauuC............................  1         1       S04
    .............................................................uuggaaggaaaacucgauuuuguu........................  1         0       S04
    .............................................................uuggaaggaaaacucgauuuuguC........................  1         1       S04
    ..............................................................uggaaggaaaacucgauuuuguug.......................  1         0       S04
```

Provisional ID	:	chrC03_1460011
Score total	:	0.1
Score for star read(s)	:	-1.3
Score for read counts	:	0
Score for mfe	:	1.4
Score for randfold	:	0
Score for cons. seed	:	
Total read count	:	15
Mature read count	:	15
Loop read count	:	0
Star read count	:	0

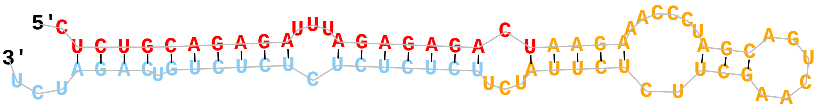

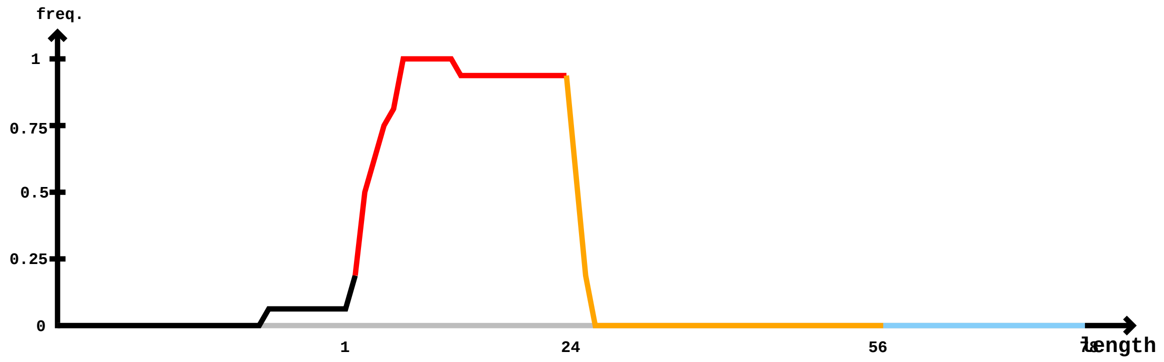

Mature Star

```
5'- uuucucauauucugagaucuaaaacggagcucugcagagauuuagagagacuaagaaacccuagcagucaagcuucucuuaucuucucucucucucugucagaucucuuu  -3'    exp
    ..(((((.....)))))........((((.((((((((((...(((((((.(((((......(((......)))..)))))...))))))).)))))).)))).))))..   reads      mm         sample
    ....................aaaacggagcucugcagaga......................................................................   1          0          S03
    ..............................ucugcagagauuuagagagacC..........................................................   1          1          S03
    ..............................ucugcagagauuuagagagacuG.........................................................   1          1          S03
    ...............................cugcagagauuuagagagacu..........................................................   1          0          S03
    ................................ugcagagauuuagagagacuaa........................................................   1          0          S03
    .................................gcagagauuuagagagacu..........................................................   1          0          S03
    ..................................cagagauuuagagagacu..........................................................   1          0          S03

    .............................cucugcagagauuuagagagacu..........................................................   2          0          S04
    ..............................ucugcagagauuuagagagacu..........................................................   1          0          S04
    ..............................ucugcagagauuuaAagagacua.........................................................   1          1          S04
    ..............................ucugcagagauuuGgagagacuaa........................................................   1          1          S04
    ...............................cugcagagauuuagagagacu..........................................................   1          0          S04
    ................................ugcagagauuuagagagacuaC........................................................   1          1          S04
    ..................................cagagauuuagagagacu..........................................................   2          0          S04
```

Provisional ID : chrC03_1461979
Score total : 0.6
Score for star read(s) : -1.3
Score for read counts : 0
Score for mfe : 1.9
Score for randfold : 0
Score for cons. seed :
Total read count : 3124
Mature read count : 3117
Loop read count : 6
Star read count : 1

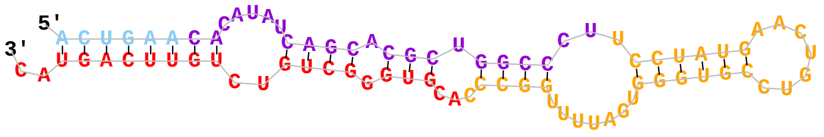

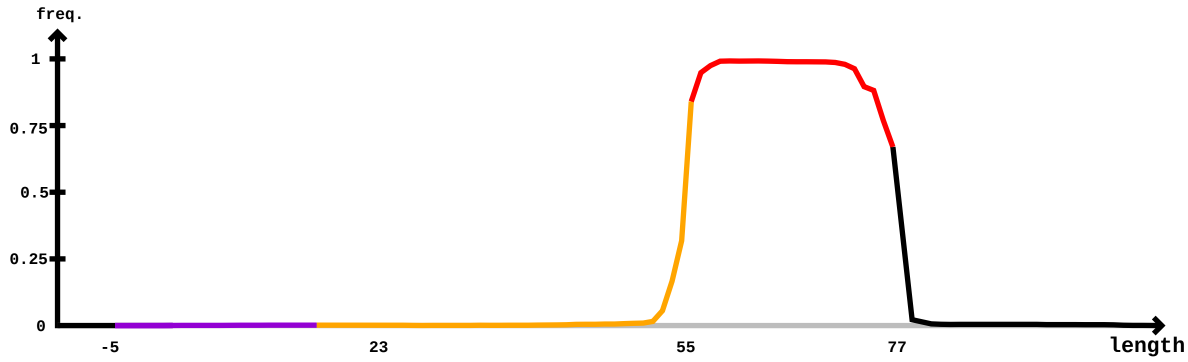

Star **Mature**

```
5'- guguacugaacacauaucagcacgcuggcccuuccuaugaacuguccgugggugauuuuggcccacgugggcugucuguucaguacacauaucagcaugcuggcccuucccgu   -3'   obs
    guguacugaacacauaucagcacgcuggcccuuccuaugaacuguccgugggugauuuuggcccacgugggcugucuguucaguacacauaucagcaugcuggcccuucccgu         exp
    ((((((((((((.....((((.(((.((((...((((((.......)))))).......))))...))).))))..))))))))))))...((((....))))..........   reads   mm   sample
    .....................................ugGacuguccguggguga..........................................................   1   1   S03
    ..........................................uguccgugggugauuuuggcc..................................................   1   0   S03
    .............................................ccgugggugauuuuggccc.................................................   1   0   S03
    ................................................ugggugauuuuggcccacgugggcugA......................................   1   1   S03
    ....................................................ugauuuuggcccacgugggcug.......................................   1   0   S03
    .....................................................gauuuuggcccacgugggcu........................................   1   0   S03
    .........................................................uuggcccacgugggcugucuguuc................................   1   0   S03
    ...........................................................ggcccacgugggcugucuguuc................................   1   0   S03
    ............................................................gcccacgugggcugucuguuc................................   4   0   S03
    ............................................................gcccacgugggcugucuguuca...............................   1   0   S03
    ............................................................gcccacgugggcugucuguucagu.............................   3   0   S03
    .............................................................cccacgugggcugucuguu.................................   2   0   S03
    .............................................................cccacgugggcugucuguuc................................   2   0   S03
    .............................................................cccacgugggcugucuguuca...............................   12   0   S03
    .............................................................cccacgugggcugucuguucC...............................   2   1   S03
    .............................................................cccacgugggcugucuguucaguaca..........................   1   0   S03
    ..............................................................ccacgugggcugucuguu.................................   1   0   S03
    ..............................................................ccacgugggcugucuguuc................................   3   0   S03
    ..............................................................ccacgugggcugucuguuca...............................   6   0   S03
    ..............................................................ccacgugggcuCucuguuca...............................   1   1   S03
    ..............................................................ccaGgugggcugucuguucagu.............................   1   1   S03
    ..............................................................ccacgugggcugucuguucagC.............................   1   1   S03
    ..............................................................ccacgugggcugucuguucagu.............................   19   0   S03
    ..............................................................ccacgugggcugucuguucaguC............................   1   1   S03
    ..............................................................ccacgugggcugucuguucagua............................   2   0   S03
    ..............................................................ccacgugggcuUucuguucaguac...........................   1   1   S03
    ..............................................................cUacgugggcugucuguucaguac...........................   3   1   S03
    ..............................................................ccacgAgggcugucuguucaguac...........................   1   1   S03
    ..............................................................ccacgugggcugucuguucGguac...........................   1   1   S03
    ..............................................................ccacgugggcugucuguucaguaG...........................   1   1   S03
    ..............................................................ccacgugggcugucuguucaguac...........................   20   0   S03
    ..............................................................ccacgugggcugCcuguucaguac...........................   1   1   S03
    ..............................................................ccacgugggcugucuguucaguaca..........................   1   0   S03
```

```
          Star                                         Mature
guguacugaacacauaucagcacgcuggcccuuccuaugaacuguccgugggugauuuuggcccacgugggcugucuguucaguacacauaucagcaugcuggcccuucccgu
..............................................................ccacgugggcugucuguucaguacU..........................   1   1   S03
...............................................................cacgugggcugucCguuca...............................   1   1   S03
...............................................................cacgugggcugucuguucG...............................   1   1   S03
...............................................................cacgugggcugucuguuca...............................   4   0   S03
...............................................................cacgugggcugucuguucag..............................   5   0   S03
...............................................................cacgugggcugucuguucagu.............................   21  0   S03
...............................................................cacgugggcugucuguucagC.............................   2   1   S03
...............................................................cacgugggcugucCguucagu.............................   1   1   S03
...............................................................cacgugggcugucuguucagCa............................   2   1   S03
...............................................................cacgugggcugucuguucagua............................   20  0   S03
...............................................................cacgugggcugucCguucagua............................   1   1   S03
...............................................................cacgugggcugucuguucaguac...........................   24  0   S03
...............................................................cacgugggcugucuguucaguaca..........................   3   0   S03
................................................................acgugggcugucuguuca...............................   2   0   S03
................................................................acgugggcugucuguucagu.............................   8   0   S03
................................................................acgugggcugucuguucagC.............................   1   1   S03
................................................................acgugggcugucuguucagua............................   19  0   S03
................................................................acgugggcugucCguucagua............................   1   1   S03
................................................................acgugggcuUucuguucagua............................   1   1   S03
................................................................acgugggcugucuguucCgua............................   1   1   S03
................................................................acgugCgcugucuguucaguac...........................   1   1   S03
................................................................Ncgugggcugucuguucaguac...........................   1   1   S03
................................................................acgugggcugucCguucaguac...........................   1   1   S03
................................................................acgugggcugucuguucaguCc...........................   1   1   S03
................................................................acgAgggcugucuguucaguac...........................   1   1   S03
................................................................acgugggcugucuguucGguac...........................   3   1   S03
................................................................acgugggcugucuguucaguaG...........................   1   1   S03
................................................................acgugggcugucuguucaguac...........................   185 0   S03
................................................................acgugggcuUucuguucaguac...........................   1   1   S03
................................................................acgugggcuCucuguucaguac...........................   3   1   S03
................................................................acgugggcugucuguucaguaca..........................   17  0   S03
................................................................acgugggcugucuguucaguacU..........................   2   1   S03
................................................................acgugggcugucuguucGguaca..........................   1   1   S03
................................................................acgugggcuCucuguucaguacac.........................   1   1   S03
.................................................................cgugggcugucuguucagu.............................   8   0   S03
.................................................................cgugggcugCcuguucagua............................   1   1   S03
.................................................................cgugggcugucuguucagua............................   3   0   S03
.................................................................cgugggcugucuguucaguac...........................   14  0   S03
.................................................................cgugggcuUucuguucaguaca..........................   1   1   S03
.................................................................cgugggcugucuguucaguaca..........................   11  0   S03
.................................................................cgugggcugucuguucCguacaca........................   1   1   S03
.................................................................cgugggcugucuguucaguacaca........................   1   0   S03
..................................................................gugggcugucuguucagu.............................   1   0   S03
..................................................................gugggcugucuguucagua............................   2   0   S03
..................................................................gugggcugucuguucaguac...........................   1   0   S03
..................................................................gugggcugucuguucaguacU..........................   1   1   S03
..................................................................gugggcuCucuguucaguaca..........................   1   1   S03
..................................................................gugggcugucuguucaguaca..........................   6   0   S03
..................................................................gugggcugucuguucaguacaG.........................   1   1   S03
..................................................................gugggcugucuguucCguacaca........................   1   1   S03
..................................................................gugggcuUucuguucaguacaca........................   1   1   S03
..................................................................gugggcugucuguucaAuacacau.......................   1   1   S03
...................................................................ugggcugucuguucaguac...........................   3   0   S03
...................................................................ugggcugucuguucaguaca..........................   2   0   S03
...................................................................ugggcugucuguucaguacaca........................   1   0   S03
...................................................................ugggcuCucuguucaguacaca........................   1   1   S03
....................................................................................acacauaucagcaugcuggcU........   1   1   S03
....................................................................................acacauaucagcaugcuggcccuu.....   1   0   S03
.....................................................................................cacauaucagcaugcuggcccuuc....   1   0   S03
.....................................................................................cacauaucagcaugcuggcccuuA....   1   1   S03
........................................................................................auaucagcaugcuggcccuucccg.   1   0   S03

..........cacauaucagcacgcuggcccu.................................................................................   1   0   S04
...........acauaucagcacAcuggcccuucc..............................................................................   1   1   S04
................ucagcacgcuggcccuucc..............................................................................   1   0   S04
.................cagcacgcuggccAuuccu.............................................................................   1   1   S04
....................cacgcuggcccuuccuaugaacu......................................................................   1   0   S04
..........................................uguccgugggugauuuuggc...................................................   1   0   S04
................................................uggCugauuuuggcccacgug............................................   1   1   S04
.................................................gggugauuuuggcccacgug............................................   1   0   S04
```

Star Mature

guguacugaacacauaucagcacgcuggcccuuccuaugaacuguccgugggugauuuuggcccacgugggcugucuguucaguacacauaucagcaugcuggcccuucccgu			
....ggugauuGuggcccacgugggc...	1	1	S04
....gugauuuuggcccacgugggcu..	1	0	S04
....gugauuuuggcccacguCggcug.......................................	1	1	S04
....ugauuuuggAccacgugggc...	1	1	S04
....ugauuuuggcccacgugggcu..	1	0	S04
....ugauuuuggcccacguCggcug.......................................	2	1	S04
....uuuuggAccacgugggcugucu....................................	1	1	S04
....uuuuggcccacgugggcugucug...................................	1	0	S04
....uuuuggcccacgugggcugucugu..................................	1	0	S04
....uuuuggAccacgugggcugucugu..................................	1	1	S04
....uuggcccacgugggcugucuguGc................................	1	1	S04
....uuggcccacgugggcugucuguuc................................	2	0	S04
....uuggcccacgugggcugucuguAc................................	1	1	S04
....uggcccacgugggcugucuguuc................................	3	0	S04
....uggcccacgugggcugucuguucC...............................	1	1	S04
....ggcccacgugggcugucuguuc................................	1	0	S04
....ggcccacgugCgcugucuguuc................................	1	1	S04
....gcccacgugggcugucugu..................................	1	0	S04
....gcccacgugggcugucuguu.................................	1	0	S04
....gcccacgugggcugucuguuc................................	3	0	S04
....gcccacgugggcugucuguuca...............................	2	0	S04
....gcccacgugggcugucuguucC...............................	2	1	S04
....gAccacgugggcugucuguucag..............................	1	1	S04
....gcccacgugggcugucuguucagu.............................	3	0	S04
....cccacgugggcuCucugu..................................	2	1	S04
....cccacgugggcugucugu..................................	3	0	S04
....cccacgugggcugucuguu.................................	10	0	S04
....cccacgugggcugucuguG.................................	1	1	S04
....cccacgugggcugucuguCc................................	2	1	S04
....cccacgugggcugucuguuc................................	15	0	S04
....cccacgugggcugucuguuca...............................	66	0	S04
....cccacgugggcuUucuguuca...............................	1	1	S04
....cccacgugCgcugucuguuca...............................	1	1	S04
....cccacgugggcugucuguucC...............................	3	1	S04
....cccacgugggcuCucuguuca...............................	1	1	S04
....cccacgugggcugucuguucG...............................	2	1	S04
....cccacgugggcuUucuguucag..............................	1	1	S04
....cccacgugggcugucuguucag..............................	1	0	S04
....cccacgugggcuCucuguucag..............................	1	1	S04
....cccacgugggcugucuguucagu.............................	1	0	S04
....cccacgugggcuUucuguucagu.............................	1	1	S04
....cccacgugggcugucuguucagua............................	1	0	S04
....cccacgugggcugucuguucaguac...........................	1	0	S04
....ccacgugggcugucuguu.................................	8	0	S04
....ccacgugggcugucuguG.................................	1	1	S04
....ccacgugggcugucuguGc................................	1	1	S04
....ccacgugggcugucuguuc................................	6	0	S04
....ccacgugggcugucuguAc................................	1	1	S04
....ccacgugggcuCucuguuc................................	1	1	S04
....ccacgugggcuUucuguuca...............................	1	1	S04
....ccacgugggcugucuguucC...............................	2	1	S04
....ccacgugggcugucuguAca...............................	1	1	S04
....ccacgugggcugCcuguuca...............................	1	1	S04
....ccacgugggcugucuguuca...............................	57	0	S04
....ccacgugggcugucuguucG...............................	4	1	S04
....ccacgugggcuCucuguuca...............................	1	1	S04
....ccacgugggcugucuguCca...............................	3	1	S04
....ccacgugggcugucuguucag..............................	10	0	S04
....ccacgugggcugucGguucagu.............................	1	1	S04
....ccacgugggcugucuguucagC.............................	9	1	S04
....ccacgugggcugucuguucagu.............................	83	0	S04
....ccacCugggcugucuguucagu.............................	1	1	S04
....ccacgugggcuUucuguucagu.............................	1	1	S04
....ccacgugggcuCucuguucagu.............................	5	1	S04
....ccacgugggcugucuguCcagu.............................	1	1	S04
....ccaGgugggcugucuguucagu.............................	1	1	S04
....ccacgugggcugucuguucCgu.............................	1	1	S04
....ccacgugggcugucuguucGgu.............................	1	1	S04
....ccacgugggcugucuguucagua............................	21	0	S04
....ccacgugggcuCucuguucagua............................	1	1	S04

Star **Mature**

```
guguacugaacacauaucagcacgcuggcccuuccuaugaacuguccgugggugauuuuggcccacgugggcugucuguucaguacacauaucagcaugcuggcccuucccgu
..............................................................ccacgugggcuUucuguucagua............................	1	1	S04
..............................................................ccacgugggcugucuguucagCa............................	1	1	S04
..............................................................ccacgugggcugucuguucaguC............................	3	1	S04
..............................................................ccaGgugggcugucuguucaguac...........................	1	1	S04
..............................................................ccacgugggcugucuguucCguac...........................	1	1	S04
..............................................................cUacgugggcugucuguucaguac...........................	3	1	S04
..............................................................ccacgAgggcugucuguucaguac...........................	1	1	S04
..............................................................ccacgugggcugucuguucaguac...........................	66	0	S04
..............................................................ccacgugggcuUucuguucaguac...........................	1	1	S04
..............................................................ccacgugggcugucuguucaguaca..........................	1	0	S04
..............................................................ccacgugggcugucuguucaguacU..........................	1	1	S04
...............................................................cacgugggcugucuguuc................................	4	0	S04
...............................................................cacgugggcugucCguuc................................	2	1	S04
...............................................................cacgugggcuCucuguuc................................	1	1	S04
...............................................................cacgugggcuCucuguuca...............................	3	1	S04
...............................................................cacgugggcugucuguucC...............................	1	1	S04
...............................................................cacgugggcugucuguucG...............................	2	1	S04
...............................................................cacgugggcugucuguuca...............................	25	0	S04
...............................................................cacgugggcuUucuguuca...............................	1	1	S04
...............................................................cacgugggcugucuguucGg..............................	1	1	S04
...............................................................cacgugggcugucuguucag..............................	19	0	S04
...............................................................cacgugggcuCucuguucag..............................	2	1	S04
...............................................................cacgugggcugucuguucagC.............................	11	1	S04
...............................................................cacgugggcugucCguucagu.............................	2	1	S04
...............................................................cacgugggcugCcuguucagu.............................	1	1	S04
...............................................................cacgugggcuCucuguucagu.............................	5	1	S04
...............................................................cacgugggcugucuguucGgu.............................	1	1	S04
...............................................................cacgugggcugucuguucagu.............................	75	0	S04
...............................................................cacgugggcugucuguGcagua............................	1	1	S04
...............................................................cacgugggcuCucuguucagua............................	1	1	S04
...............................................................cacgugggcuUucuguucagua............................	1	1	S04
...............................................................cacgAgggcugucuguucagua............................	1	1	S04
...............................................................cacgugCgcugucuguucagua............................	1	1	S04
...............................................................cacgugggcugucuguAcagua............................	1	1	S04
...............................................................cacgugggcugucuguucagCa............................	3	1	S04
...............................................................cacgugggcugucuguucaguC............................	4	1	S04
...............................................................cacgugggcugucuguucGgua............................	1	1	S04
...............................................................cacgugggcugucuguucagua............................	113	0	S04
...............................................................cacgugggcugCcuguucagua............................	1	1	S04
...............................................................Nacgugggcugucuguucagua............................	1	1	S04
...............................................................cacgugggcuCucuguucaguac...........................	2	1	S04
...............................................................cacgugggcugucuguucGguac...........................	1	1	S04
...............................................................Uacgugggcugucuguucaguac...........................	7	1	S04
...............................................................cacgugggcugucCguucaguac...........................	1	1	S04
...............................................................cacgugggcugucuguucCguac...........................	1	1	S04
...............................................................cacgugggcugucuguucaguac...........................	92	0	S04
...............................................................cacgugggcugucCguucaguaca..........................	1	1	S04
...............................................................cacgugggcugucuguucaguaca..........................	24	0	S04
...............................................................cacgugggcugCcuguucaguaca..........................	1	1	S04
...............................................................cacgugggcugucuguGcaguaca..........................	1	1	S04
...............................................................cacgugggcugucuguucagCaca..........................	2	1	S04
...............................................................cacgugggcuCucuguucaguaca..........................	1	1	S04
...............................................................cacgugggcugucuguucaguacU..........................	1	1	S04
................................................................acgAgggcugucuguuca...............................	1	1	S04
................................................................acgugggcugucuguucC...............................	1	1	S04
................................................................acgugggcugucuguucG...............................	1	1	S04
................................................................acgugggcugucuguuca...............................	9	0	S04
................................................................acgugggcugucCguucag..............................	1	1	S04
................................................................acgugggcugucuguucag..............................	3	0	S04
................................................................acgugggcugucuguucagu.............................	69	0	S04
................................................................acgugggcugucuguucagC.............................	5	1	S04
................................................................acgugggcugucuguucagCa............................	1	1	S04
................................................................acgugggcuUucuguucagua............................	2	1	S04
................................................................acgugggcugucuguucGgua............................	1	1	S04
................................................................acgugggcugucuguucagua............................	76	0	S04
................................................................acgugCgcugucuguucagua............................	1	1	S04
................................................................acgugggcugucuguucaguC............................	4	1	S04
................................................................acgugggcugucuguAcaguac...........................	1	1	S04
................................................................acgAgggcugucuguucaguac...........................	4	1	S04
................................................................acgugggcugucuguCcaguac...........................	1	1	S04
```

Star **Mature**

```
guguacugaacacauaucagcacgcuggcccuuccuaugaacuguccgugggugauuuuggcccacgugggcugucuguucaguacacauaucagcaugcuggcccuucccgu
................................................................acgugggcuCucuguucaguac...........................  8     1  S04
................................................................acgugggcugucuguucaguac...........................  1165  0  S04
................................................................acgugggcugucCguucaguac...........................  2     1  S04
................................................................acgugggcugucGguucaguac...........................  1     1  S04
................................................................acgugggcugCcuguucaguac...........................  6     1  S04
................................................................acgugggcugucuguucGguac...........................  7     1  S04
................................................................acgugggcuUucuguucaguac...........................  5     1  S04
................................................................acgugggcugucuguucaguaG...........................  3     1  S04
................................................................Ncgugggcugucuguucaguac...........................  4     1  S04
................................................................acgugggcugucuguucagCac...........................  4     1  S04
................................................................acgugggcugucuguucGguaca..........................  1     1  S04
................................................................acgugggcuCucuguucaguaca..........................  2     1  S04
................................................................acgugggcugucuguucaguaca..........................  75    0  S04
................................................................acgugggcugucuguucagCaca..........................  1     1  S04
................................................................acgugggcugucuguucaguacU..........................  9     1  S04
................................................................acgugggcuUucuguucaguacac.........................  2     1  S04
................................................................acgugggcugucuguucaguaGac.........................  4     1  S04
................................................................acgugggcuCucuguucaguacac.........................  1     1  S04
................................................................acgugggcugucuguucCguacac.........................  1     1  S04
................................................................acgugggcugucuguucGguacac.........................  1     1  S04
................................................................acgugggcugucuguucaguacUc.........................  1     1  S04
.................................................................cgugggcugucuguucag..............................  2     0  S04
.................................................................cgugggcuUucuguucagu.............................  1     1  S04
.................................................................cgugggcugucuguucagC.............................  3     1  S04
.................................................................cgugggcugucuguucagu.............................  20    0  S04
.................................................................cgugggcugucuguucagua............................  19    0  S04
.................................................................cgugggcugucuguucagCa............................  3     1  S04
.................................................................cgugggcuUucuguucagua............................  1     1  S04
.................................................................cgugggcugucGguucagua............................  1     1  S04
.................................................................cgugggcugucuguucaguC............................  2     1  S04
.................................................................cgugggcugucuguucaguac...........................  102   0  S04
.................................................................cgugggcugucuguAcaguac...........................  1     1  S04
.................................................................cgugggcuCucuguucaguac...........................  3     1  S04
.................................................................cgugggcuUucuguucaguac...........................  3     1  S04
.................................................................cgugggcugucuguucCguac...........................  1     1  S04
.................................................................cgugggcuUucuguucaguaca..........................  3     1  S04
.................................................................cgugggcugucuguucaguacU..........................  6     1  S04
.................................................................cgugggcugucuguucaguaca..........................  132   0  S04
.................................................................cgugggcugucuguucCguaca..........................  2     1  S04
.................................................................cgugggcuCucuguucaguaca..........................  8     1  S04
.................................................................cgugggcugucGguucaguaca..........................  2     1  S04
.................................................................cgugggcugucuguucagCaca..........................  1     1  S04
.................................................................cgugggcugucuguucaguacac.........................  1     0  S04
.................................................................cgugggcuCucuguucaguacac.........................  1     1  S04
.................................................................cgugggcuUucuguucaguacaca........................  1     1  S04
..................................................................gugggcugucuguucagu.............................  9     0  S04
..................................................................gugggcugucuguucagua............................  10    0  S04
..................................................................gugggcugucuguucGguac...........................  1     1  S04
..................................................................gugggcugucuguucaguac...........................  11    0  S04
..................................................................gugggcugucuguucGguaca..........................  1     1  S04
..................................................................gugggcugucuguucaguacU..........................  2     1  S04
..................................................................gugggcugucuguucaguaca..........................  32    0  S04
..................................................................gugggcuUucuguucaguaca..........................  1     1  S04
..................................................................gugggcuCucuguucaguacac.........................  2     1  S04
..................................................................gugggcugucuguucaguacac.........................  1     0  S04
..................................................................gugggcugucuguucCguacaca........................  2     1  S04
...................................................................ugggcugucuguucagua............................  2     0  S04
...................................................................ugggcugucuguucaguac...........................  6     0  S04
...................................................................ugggcugucuguucaguacU..........................  1     1  S04
...................................................................ugggcugucuguucGguaca..........................  2     1  S04
...................................................................ugggcugucuguucaguaca..........................  7     0  S04
...................................................................ugggcugucuguucaguacac.........................  10    0  S04
...................................................................ugggcugucuguucaguacaca........................  15    0  S04
...................................................................ugggcuguGuguucaguacacau.......................  1     1  S04
...................................................................ugggcugucuguucaguacacau.......................  2     0  S04
...................................................................ugggcugucuguucaguacacGu.......................  1     1  S04
...................................................................Agggcugucuguucaguacacaua......................  1     1  S04
....................................................................gggcugucuguucaguaca..........................  1     0  S04
....................................................................gggcugucuguucaguacac.........................  1     0  S04
....................................................................gggcugucuguucaguacaca........................  1     0  S04
```

Star **Mature**

```
guguacugaacacauaucagcacgcuggcccuuccuaugaacuguccgugggugauuuuggcccacgugggcugucuguucaguacacauaucagcaugcuggcccuucccgu
......................................................................gcugucuguucaguacacaua......................  1  0  S04
.......................................................................cugucuguucaguacaca........................  1  0  S04
...............................................................................ucaguacacauaucagcaAgcu............  1  1  S04
................................................................................caguacacauaucagcaugcu............  1  0  S04
.................................................................................aguacacauaucagcaugcu............  1  0  S04
....................................................................................acacauaucagcaugcuggcAcuu.....  1  1  S04
......................................................................................acauaucagcaugcuggcccuuc....  1  0  S04
......................................................................................acauaucagcaugcuggcccuucc...  1  0  S04
.......................................................................................cauaucagcaugcuggcccuuc....  1  0  S04
.......................................................................................Nauaucagcaugcuggcccuuc....  1  1  S04
........................................................................................auaucagcaugcuggcccuucc...  1  0  S04
............................................................................................cagcaugcuggcccuucccgG  1  1  S04
```

Provisional ID	:	chrC03_1465275
Score total	:	0
Score for star read(s)	:	-1.3
Score for read counts	:	0
Score for mfe	:	1.3
Score for randfold	:	0
Score for cons. seed	:	
Total read count	:	74
Mature read count	:	74
Loop read count	:	0
Star read count	:	0

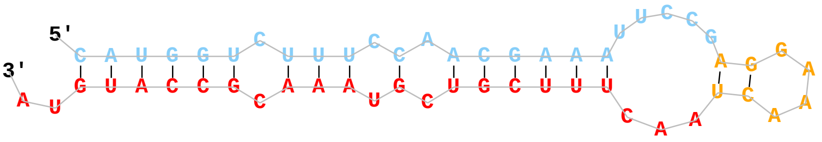

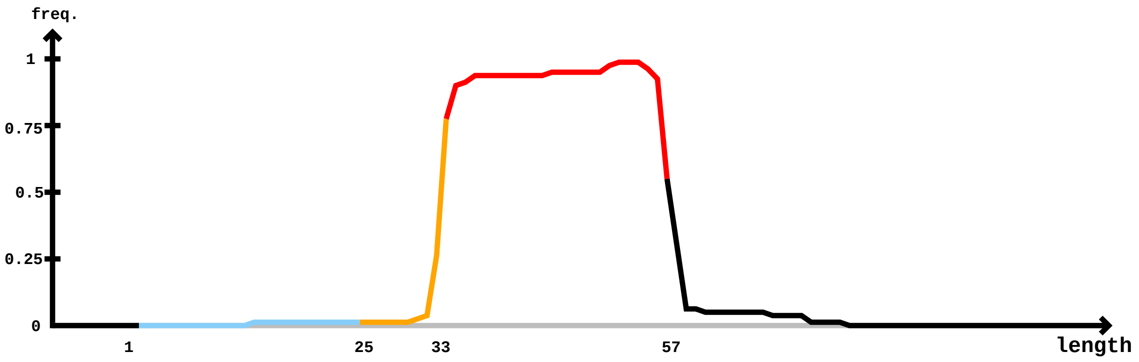

Star Mature

```
5'- aaauuuacauggucuuuccaacgaaauuccgaggaaacuaacuuucgucguaaacgccauguaaauuuacgacgaauaguuuucgucguaaacucuauguaaguuuac   -3'    exp
    .((((((((((((.(((.(.((((((.....((....))...)))))).).))).))))))))))))(((((((((.....))))))))))..................   reads   mm   sample
    .....................................cuaacuuucgucguaaacgccau................................................   1       0    S03
    ......................................uaacuuucgucguaaacgccaugu..............................................   6       0    S03
    .......................................aacuuucgucguaaacgccaugG..............................................   2       1    S03
    .......................................aacuuucgucguaaacgccaugu..............................................   3       0    S03
    .......................................aacuuucgucguaaacgccaGgua.............................................   1       1    S03
    .......................................aacuuucgucguaaCcgccaugua.............................................   1       1    S03
    .......................................aacuuucgucguaaacgccaugua.............................................   9       0    S03
    ........................................acuuucgucguaaacgccaugu..............................................   1       0    S03
    ........................................acCuucgucguaaacgccaugua.............................................   1       1    S03
    ........................................acuuucgucguaaacgccaugua.............................................   1       0    S03
    .........................................cuuucgucguaaacgccaugu..............................................   1       0    S03
    ........................................................ccauguaaauuuacgacgaau...............................   1       0    S03
    ........................................................cAauguaaauuuacgacgaau...............................   1       1    S03
    .........................................................cauguaaauuuacgacgaauagGu...........................   1       1    S03

    ...................aacgGaauuccgaggaaacu.....................................................................   1       1    S04
    ....................................acCaacuuucgucguaaacgccau................................................   1       1    S04
    ......................................uaacuuucgucguaaacgccauA...............................................   3       1    S04
    ......................................uaacuuucgucguaaacgccaugC..............................................   1       1    S04
    ......................................uaacuuucgucguaaacgccaugA..............................................   3       1    S04
    ......................................uaacuuucgucguaaacgccaugu..............................................   5       0    S04
    .......................................aacuuucgucguaaacgccaugC..............................................   1       1    S04
    .......................................aacuuucgucguaaacgccaugA..............................................   1       1    S04
    .......................................aacuuucgucguaaacgccaugu..............................................   3       0    S04
    .......................................aacuuucgucguaaacgccaGgu..............................................   2       1    S04
    .......................................aacuuucgucguaaacgccaugua.............................................   17      0    S04
    .......................................aacuuucgucguaaacgccauguG............................................   2       1    S04
    ........................................acuuucgucguaaacgccaugua.............................................   7       0    S04
    ..........................................uuucgucguaaacgccaugu..............................................   1       0    S04
    ..........................................uuucgucguaaacgccauguaaau..........................................   1       0    S04
    ..................................................uaaacgccauguaaauuuacgac...................................   1       0    S04
```

Provisional ID	:	chrC03_1471457
Score total	:	0.4
Score for star read(s)	:	-1.3
Score for read counts	:	0
Score for mfe	:	1.7
Score for randfold	:	0
Score for cons. seed	:	
Total read count	:	32
Mature read count	:	20
Loop read count	:	12
Star read count	:	0

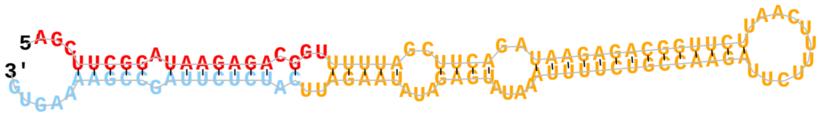

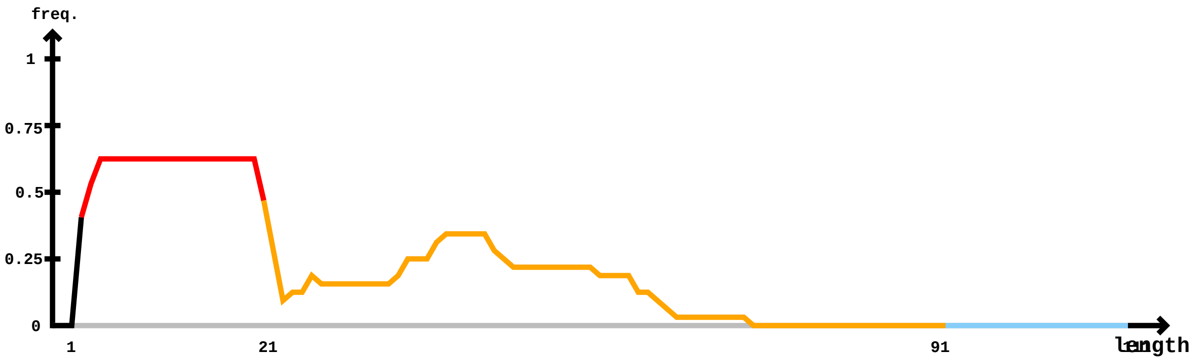

Mature **Star**

```
5'- uagcuucggauaagagacgguuuuuagcuucagauaagagacgguucuuaacuuuucuuagaaccgucuuuuaaauaugagauauaagauucaucucuuagccgaaaaguguaa   -3'    exp
    ....(((((.(((((((.(..(((((..((((..((((((((((((((..........))))))))))))))....))))...)))))..).))))))).)))))........      reads   mm   sample
    .agcuucggauaagagacgg..............................................................................................      1      0    S03
    .agcuucggauaagagacggu.............................................................................................      1      0    S03
    ..gcuucggauaagagacgU..............................................................................................      1      1    S03
    ..gcuucggauaagagacggC.............................................................................................      1      1    S03
    ...cuucggauaagagacgguuuuuU........................................................................................      1      1    S03
    ....................uuuuuagcuucagauaagagacgg......................................................................      2      0    S03
    .......................uuagcuucagauaagagacggCu....................................................................      1      1    S03
    .........................agcuucagauaagagacggu.....................................................................      1      0    S03
    .........................agcuucagauaagagacgguucuu.................................................................      1      0    S03
    ......................................agacggCucuuaacuuuucuuag.....................................................      1      1    S03
    ......................................agaAgguucuuaacuuuucuuaga....................................................      1      1    S03

    .agcuucggauaagagacgg..............................................................................................      3      0    S04
    .agcuucggauaagagacggC.............................................................................................      2      1    S04
    .agcuucggauaagagacggu.............................................................................................      6      0    S04
    ..gcuucggauaagagacgU..............................................................................................      1      1    S04
    ..gcuucggauaagagacgg..............................................................................................      1      0    S04
    ...cuucggauaagagacggC.............................................................................................      1      1    S04
    ...cuucggauaagagacgguu............................................................................................      1      0    S04
    ..................................uaagagacgguucuuaacuuG...........................................................      1      1    S04
    ...................................aagagUcgguucuuaacuuuucuu.......................................................      2      1    S04
    .......................................gacgguucuuaacuuuucuuagaC...................................................      1      1    S04
    .................................................aacuuuucuuagaaccgucuuu...........................................      1      0    S04
```

Provisional ID	:	chrC03_1480748
Score total	:	0
Score for star read(s)	:	-1.3
Score for read counts	:	0
Score for mfe	:	1.2
Score for randfold	:	0
Score for cons. seed	:	
Total read count	:	704
Mature read count	:	704
Loop read count	:	0
Star read count	:	0

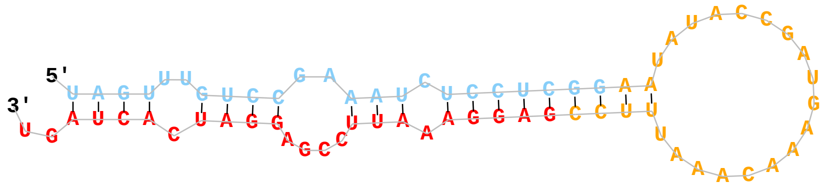

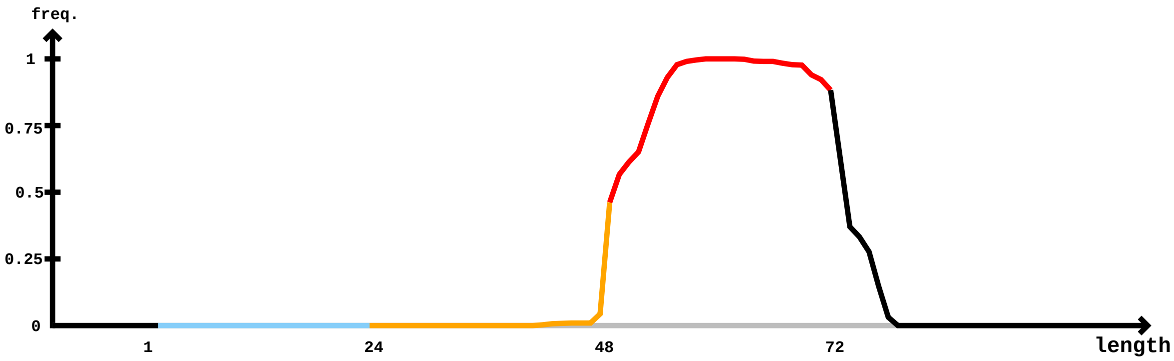

Star Mature

```
5'- gaggaucacuaguuuguccgaaaucuccucggaauauaccgaugaaacaaauuuccgaggaaauuccgaggaucacuaguuuguccgaaaucuccucggaauauaccgauga  -3'   exp
    ..((((.((((((..((((..(((.(((((((((..................))))))))).)))....)))).))))))..))))......((.((((......)))).))   reads   mm   sample
    .................................................aauuuccgaggaaauuccgagg.........................................   1       0    S03
    ..................................................auuuccgaggaaauuccgagg.........................................   1       0    S03
    ...................................................Guuccgaggaaauuccgagg.........................................   1       1    S03
    ....................................................uuccgaggaaauuccgag..........................................   1       0    S03
    .......................................................cgaggaaauuccgaggaucacu...................................   1       0    S03
    .......................................................cgaggaaauuccgaggaucacua..................................   2       0    S03
    .......................................................cgaggaaauuccgaggaucacuag.................................   5       0    S03
    ........................................................gaggaaauuccgaggauc......................................   1       0    S03
    ........................................................gaggaaauucUgaggauca.....................................   1       1    S03
    ........................................................gaggaaauucUgaggaucac....................................   1       1    S03
    ........................................................gaggaaauuccgaggaucacC...................................   1       1    S03
    ........................................................gaggaaauuccgaggaucacu...................................   8       0    S03
    ........................................................gaggaaauuccgaggaucacG...................................   1       1    S03
    ........................................................gaggaaauuccgaggaucacua..................................   1       0    S03
    ........................................................gaggaaauucUgaggaucacua..................................   1       1    S03
    ........................................................gaggaaauuccgaggaucacuag.................................   5       0    S03
    ........................................................gaggaaauuccgaggaucGcuagu................................   1       1    S03
    ........................................................gaggaaauuccgaggaucacuagA................................   2       1    S03
    ........................................................gaggaaauuccgaggaucacuagC................................   11      1    S03
    ........................................................Aaggaaauuccgaggaucacuagu................................   3       1    S03
    ........................................................gaggaaauuccCaggaucacuagu................................   1       1    S03
    ........................................................gaggaaauuccgaggaucacuagG................................   1       1    S03
    ........................................................gaggaaauuccgaggaucacuagu................................   106     0    S03
    .........................................................aggaaauuccgaggaucacA...................................   1       1    S03
    .........................................................aggaaauuccgaggaucacua..................................   1       0    S03
    .........................................................aggaaauuccgaggaucacuag.................................   1       0    S03
    .........................................................aggaaauuccgaggaucacuagu................................   5       0    S03
    .........................................................aggaaauuccgaggaucacuagC................................   1       1    S03
    .........................................................aggaaauuccgaggaucacuaguu...............................   27      0    S03
    .........................................................aggaaauucUgaggaucacuaguu...............................   1       1    S03
    .........................................................aggaaauuccgaggaucacuaguC...............................   1       1    S03
    .........................................................aggaaauuccgaggaucacuaguuA..............................   1       1    S03
    .........................................................aggaaauuccgaggaucacuaguuu..............................   1       0    S03
    ..........................................................ggaaauuccgaggaucacuagC................................   1       1    S03
```

Star Mature

gaggaucacuaguuuguccgaaaucuccucggaauauaccgaugaaacaaauuuccgaggaaauuccgaggaucacuaguuuguccgaaaucuccucggaauauaccgauga			
....ggaaauuccgaggaucacuagu................................	1	0	S03
....ggaaauuccgaggaucacuaguu...............................	5	0	S03
....Ugaaauuccgaggaucacuaguuu..............................	1	1	S03
....ggaaauuccgaggaucacuaguuu..............................	6	0	S03
....ggaaauuccgaggaucacuaguuC..............................	1	1	S03
....gaaauuccgaggaucacuagu................................	1	0	S03
....gaaauuccgaggaucacuaguC...............................	1	1	S03
....gaaauuccgaggaucacuaguu...............................	2	0	S03
....gaaauuccgaggaucacuaguuu..............................	3	0	S03
....gaaauuccgaggaucacuaguuC..............................	1	1	S03
....gaaauuccgaggaucacuaguuug.............................	5	0	S03
....aaauuccgaggaucacuaguu...............................	2	0	S03
....aaauuccgaggaucacuaguuu..............................	3	0	S03
....aaauuccgaggaucacuaguuug.............................	1	0	S03
....aaauuccgaggaucacuaguuugu............................	22	0	S03
....aaauuccgaggaucacuaguuugG............................	4	1	S03
....aaauuccgaggaucacuaguuugA............................	3	1	S03
....aaauuccgaggaucacuagGuuguc...........................	1	1	S03
....aauuccgaggaucacuagC................................	2	1	S03
....aauuccgaggaucacuagu................................	2	0	S03
....aauuccgaggaucacuaguuug.............................	8	0	S03
....aauuccgaggaucacuaguuugu............................	2	0	S03
....aauuccgaggaucacuaguuuguA...........................	2	1	S03
....aauuccgaggaucacuaguuuguc...........................	14	0	S03
....auuccgaggaucacuagu................................	1	0	S03
....auuccgaggaucacuaguuug.............................	1	0	S03
....auuccgaggaucacuaguuugu............................	10	0	S03
....auuccgaggaucacuaguuuguc...........................	3	0	S03
....auuccgaggaucacuaguuuguA...........................	1	1	S03
....auuccgaggaucacuaguuuguG...........................	1	1	S03
....Guuccgaggaucacuaguuuguc...........................	1	1	S03
....auuccgaggaucacuaguuugucU..........................	6	1	S03
....auuccgaggaucacuaguuugucc..........................	3	0	S03
....uuccgaggaucacuaguuug.............................	1	0	S03
....uuccgaggaucacuaguuugu............................	3	0	S03
....uuccgaggaucacuaguuuguc...........................	8	0	S03
....uuccgaggaucacuaguuuguA...........................	2	1	S03
....uuccgaggaucacuaguuugucU..........................	1	1	S03
....uccgaggaucacuaguuuguc...........................	2	0	S03
....uccgaggaucacuaguuugucU..........................	1	1	S03
....ccgaggaucacuaguuuguc...........................	1	0	S03
....cgaggaucacuaguuuguUc..........................	1	1	S03
....aauuuccgaggaaauuccgagga..	1	0	S04
....auuuccgaggaaauuccgagg...	2	0	S04
....cgaggaaauuccgaggaucacC...................................	1	1	S04
....cgaggaaauuccgaggaucacu...................................	4	0	S04
....cgaggaaauuccgaggaucacua..................................	1	0	S04
....cgaggaaauuccgaggaucacuG..................................	3	1	S04
....cgaggaaauuccgaggaucacuag.................................	7	0	S04
....cgaggaaauuccgaggaucacuaA.................................	1	1	S04
....gaggaaauuccgaggauG......................................	2	1	S04
....gaggaaauuccgaggauc......................................	2	0	S04
....gaggaaauuccgaggauca.....................................	1	0	S04
....gaggaaauucUgaggauca.....................................	2	1	S04
....gaggaaauuccgaggaucacC...................................	2	1	S04
....gaggaaauuccgaggaucacu...................................	5	0	S04
....gaggaaauuccgaggaucacua..................................	2	0	S04
....gaggaaauuccgaggaucacCa..................................	1	1	S04
....gaggaaauuccgaggaucacuag.................................	4	0	S04
....gaggaaGuuccgaggaucacuagu................................	2	1	S04
....Uaggaaauuccgaggaucacuagu................................	2	1	S04
....gaggaaauuccgaggaucacuagC................................	9	1	S04
....gaggaaauuccgaggaucacuagu................................	117	0	S04
....gaggaaauuccgUggaucacuagu................................	2	1	S04
....gaggaaauuccgaggGucacuagu................................	1	1	S04
....gaggaaauucUgaggaucacuagu................................	1	1	S04
....gaggaaauuccgaggaucacuagG................................	3	1	S04
....gaggGaauuccgaggaucacuagu................................	1	1	S04
....gaggaaauuccgaggaucacuaguu...............................	1	0	S04

Star Mature

gaggaucacuaguuuguccgaaaucuccucggaauauaccgaugaaacaaauuuccgaggaaauuccgaggaucacuaguuuguccgaaaucuccucggaauauaccgauga			
....gaggaaauuccgaggaucacuaguA...............................	1	1	S04
....Caggaaauuccgaggaucacuaguu...............................	1	1	S04
....aggaaauuccgaggaucacC...................................	1	1	S04
....aggaaauuccgaggaucacu...................................	1	0	S04
....aggaaaCuccgaggaucacuag.................................	1	1	S04
....aggaaauuccgaggaucacuag.................................	4	0	S04
....aggaaauuccgaggaucacuagu................................	3	0	S04
....aggaaauucUgaggaucacuaguu...............................	1	1	S04
....aggaaauuccgaggaucacuaguu...............................	23	0	S04
....aggaaauuccgaggaucacCaguu...............................	1	1	S04
....aggaaauuccgaggaucacuGguu...............................	1	1	S04
....aggaaauuccgaggaucacuaguuA..............................	1	1	S04
....ggaaauuccgaggaucacua..................................	1	0	S04
....ggaaauuccgaggaucacuag.................................	1	0	S04
....ggaaGuuccgaggaucacuagu................................	1	1	S04
....ggaaauuccgaggaucacuagu................................	5	0	S04
....ggaaauuccgaggaucacuagC................................	1	1	S04
....ggaaauuccgaggaucacuaguu...............................	4	0	S04
....ggaaauuccgaggaucacuaguuC..............................	1	1	S04
....ggaaaCuccgaggaucacuaguuu..............................	1	1	S04
....ggaaauuccgaggaucacuaguuu..............................	4	0	S04
....gaaauuccgaggaucacu...................................	1	0	S04
....gaaauuccgaggaucacuagu................................	4	0	S04
....gaaauuccgaggaucacuaguu...............................	2	0	S04
....gaaauuccgaggaucacuaguuu..............................	1	0	S04
....gaaauuccgaggaucacuaguuug.............................	7	0	S04
....aaauuccgaggaucacuagu................................	3	0	S04
....aaauuccgaggaucacuagC................................	1	1	S04
....aaauuccgaggaucacuagG................................	1	1	S04
....aaauuccgaggaucacuaguu...............................	2	0	S04
....aaauuccgaggaucacuaguuu..............................	1	0	S04
....aaauuccgaggaucacuaguuug.............................	1	0	S04
....aaauuccgaggaucacuagGuugu............................	1	1	S04
....aaauuccgaggaucacuaguuugG............................	1	1	S04
....Naauuccgaggaucacuaguuugu............................	2	1	S04
....aaauuccgaggaucacuaguuugA............................	1	1	S04
....aaauuccgaggaucacuaguuugu............................	28	0	S04
....aauuccgaggaucacuagA................................	1	1	S04
....aauuccgaggaucacuagu................................	1	0	S04
....aGuuccgaggaucacuaguuu..............................	1	1	S04
....aauuccgaggaucacuaguuuC.............................	1	1	S04
....aauuccgaggaucacuagGuug.............................	1	1	S04
....aauuccgaggaucacuaguuug.............................	9	0	S04
....aauuccgaggaucacuaguuugu............................	3	0	S04
....aauuccgaggaucaGuaguuugu............................	1	1	S04
....aauuccgaggaucacuaguuuUu............................	1	1	S04
....aauuccgaggaucacuaguuuguc...........................	23	0	S04
....aauuccgaggaucacuaguuuguA...........................	1	1	S04
....aauuccgaggaucacuaguuugCc...........................	1	1	S04
....aauuccgaggaucacuagCuuguc...........................	1	1	S04
....auuccgaggaucacuagu................................	1	0	S04
....auuccgaggaucacuaguuug.............................	4	0	S04
....auuccgaggaucacuaguuugu............................	9	0	S04
....auuccgaggaucacuaguuugA............................	2	1	S04
....auuccgaggaucacuaguuugCc...........................	1	1	S04
....auuccgaggaucacuaguuuguc...........................	1	0	S04
....auuccgaggaucacuaguuuguA...........................	3	1	S04
....auuccgaggaucacuaguuugucU..........................	4	1	S04
....uuccgaggaucacuaguu...............................	1	0	S04
....uuccgaggaucacuaguuu..............................	1	0	S04
....uuccgaggaucacuaguuugu............................	1	0	S04
....uuccgaggaucacuaguuuguc...........................	11	0	S04
....uuccgaggaucacuaguuugucc..........................	2	0	S04
....uuccgaggaucacuaguuugucU..........................	4	1	S04
....uccgaggaucacuaguuuU.............................	2	1	S04
....uccgaggaucacuaguuuguA...........................	1	1	S04
....uccgaggaucacuaguuuguc...........................	3	0	S04
....ccgaggaucacuaguuugu............................	1	0	S04
....ccgaggaucacuaguuuguc...........................	2	0	S04
....cgaggaucacuaguuuguc...........................	1	0	S04

Star Mature

```
gaggaucacuaguuuguccgaaaucuccucggaauauaccgaugaaacaaauuuccgaggaaauuccgaggaucacuaguuuguccgaaaucuccucggaauauaccgauga
..................................................................cgaggaucacuaguuugucU..........................      1      1      S04
```

Provisional ID	:	chrC03_1484919
Score total	:	1.9
Score for star read(s)	:	3.9
Score for read counts	:	-2.3
Score for mfe	:	0.4
Score for randfold	:	0
Score for cons. seed	:	
Total read count	:	7
Mature read count	:	6
Loop read count	:	0
Star read count	:	1

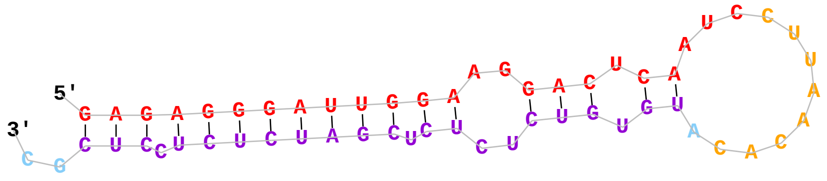

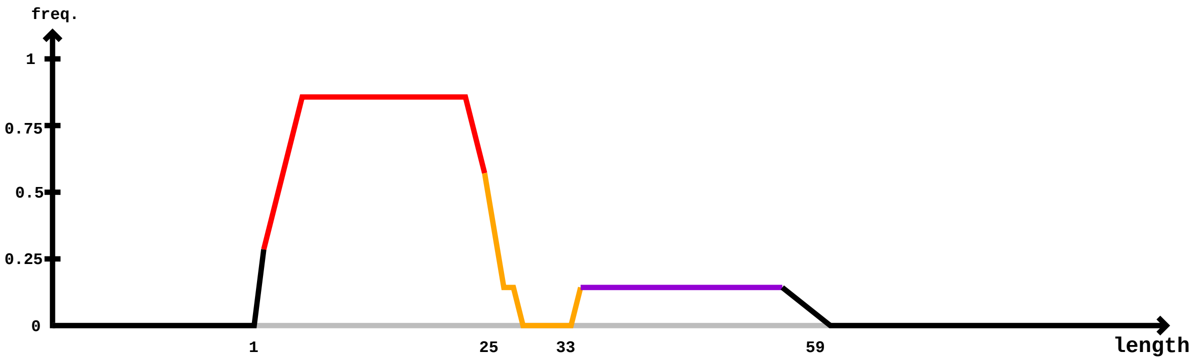


```
                       Mature               Star
5'- uagcuagggaucaauccuucgagagggauuggaaggacucaauccuuaacacaugugucucucucgaucucuccucgcuccaucaucucccaccauggcuccuucauaaucuuc   -3'    obs
    uagcuagggaucaauccuucgagagggauuggaaggacucaauccuuaacacaugugucucucucgaucucuccucgcuccaucaucucccaccauggcuccuucauaaucuuc          exp
    .(((((((((.........((((((((((((((..(((.((............)).)))..)).)))))))).))))..........))))....)))))..............  reads    mm    sample
    ....................gagagggauuggaaggacucaauc......................................................................  1        0     S03
    .......................agggauuggaaggacucaauc......................................................................  1        0     S03
    ........................gggauuggaaggacucaauccuu...................................................................  1        0     S03

    ....................gagagggauuggaaggacucaa........................................................................  1        0     S04
    .....................agagggauuggaaggacucaaucc.....................................................................  1        0     S04
    ......................gagggauuggaaggacucaau.......................................................................  1        0     S04
    .....................................................ugugucucucucgaucCcuccuc......................................  1        1     S04
```

Provisional ID	:	chrC03_1485517
Score total	:	2
Score for star read(s)	:	3.9
Score for read counts	:	-3.9
Score for mfe	:	2
Score for randfold	:	0
Score for cons. seed	:	
Total read count	:	4
Mature read count	:	3
Loop read count	:	0
Star read count	:	1

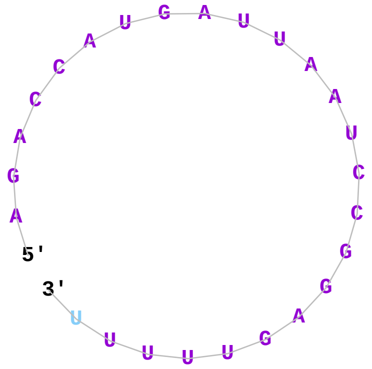

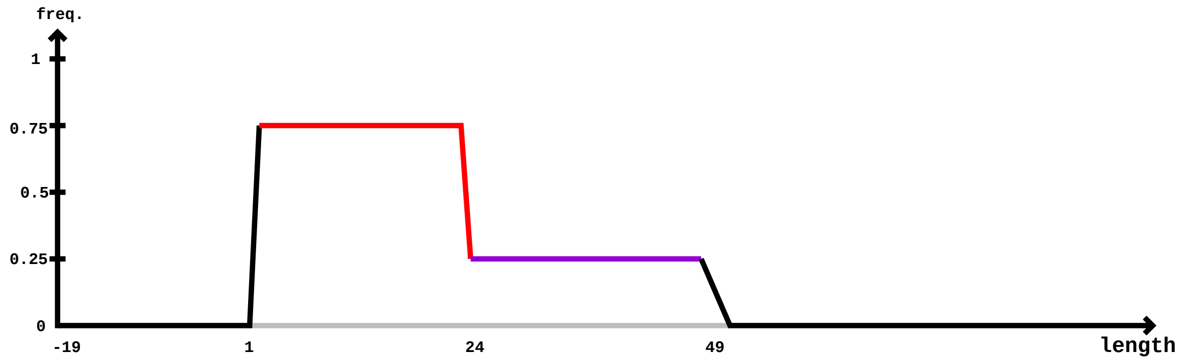


```
                         Mature          Star
5'- guacuaagaaccucguuaaggacuccggauaaacaugcucuaagaccaugauuaauccggaguuuuuaaagugggguucuuagcgagaguuaagaaauuguuuguuaacuuc   -3'    obs
    guacuaagaaccucguuaaggacuccggauaaacaugcucuaagaccaugauuaauccggaguuuuuaaagugggguucuuagcgagaguuaagaaauuguuuguuaacuuc          exp
    ...(((((((((((.(((((((((((((((.((((((.((...)).)))).)).)))))))))))))))...)))))))))))...(((((((.((.....)).))))))).   reads   mm   sample
    ...................ggacuccggauaaacaugcucu.......................................................................   1       0    S03
    ...................ggacuccggauaaacaugcucuG......................................................................   1       1    S03

    ...................Ugacuccggauaaacaugcucu.......................................................................   1       1    S04
    ..........................................agaccaugauuaauccggaguuGu..............................................   1       1    S04
```

Provisional ID : chrC03_1506159
Score total : 0
Score for star read(s) : -1.3
Score for read counts : 0
Score for mfe : 1.3
Score for randfold : 0
Score for cons. seed :
Total read count : 184
Mature read count : 184
Loop read count : 0
Star read count : 0

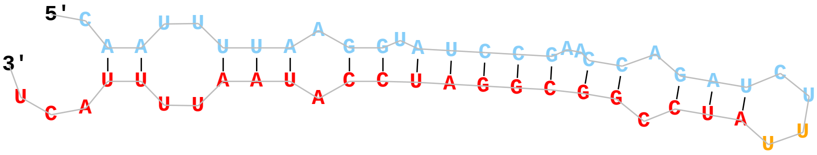

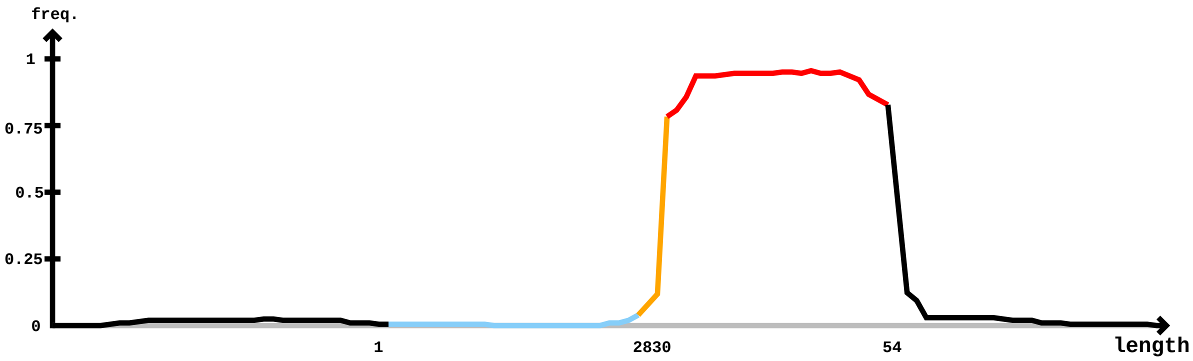

Star Mature

```
5'- gucgcuaggccugggacggaucggauauucgggcaauuuuaagguauccgaaccagaucuuuauccggcggauccauaauuuuacuauccuuauccggauccgagguuuucgga -3'   exp
    .(((..((((((....((((((((((....((.((..(((.((.(((((..((.(((....))).))))))))).)))..)).)).....))))).))))))))))).))).   reads   mm   sample
    ....cuaggccugggacggauc............................................................................................   1   0   S03
    .....uaggccugggacggaucggauauu.....................................................................................   1   0   S03
    ....................ucggauauucgggcaauuuuaagg......................................................................   1   0   S03
    ........................................................aucuuuauccggcggauc........................................   1   0   S03
    ........................................................aucuuuauccggcggauccauC....................................   1   1   S03
    ..........................................................cuuuauccggcggauccauaauuu................................   1   0   S03
    ...........................................................uCuauccggcggauccauaauuuu...............................   1   1   S03
    ...........................................................uuuauccggcggauccauaauuuu...............................   1   0   S03
    ............................................................uuauccggcggauccauaauuuu...............................   1   0   S03
    .............................................................uauccggcggauccauaauuuua..............................   1   0   S03
    .............................................................uauccggcggauccauaauuuuac.............................   1   0   S03
    .............................................................uauccggcggauccauaauuuuacu............................   1   0   S03
    ..............................................................auccggcggauccauaauu.................................   1   0   S03
    ..............................................................auccggcggauccauaauuuu...............................   1   0   S03
    ..............................................................auccggcggaAccauaauuuuacu............................   1   1   S03
    ..............................................................auccggcggauAcauaauuuuacu............................   1   1   S03
    ..............................................................auccggcggauccauGauuuuacu............................   1   1   S03
    ..............................................................auccggcggGuccauaauuuuacu............................   1   1   S03
    ..............................................................auccggcggauccauaauuuuacu............................   46  0   S03
    ..............................................................auccggcggauccauaauuuuacG............................   4   1   S03
    ..............................................................auccggcggauccauaauuuuacC............................   6   1   S03
    ...............................................................uccggcggauccauaauuuuacC............................   1   1   S03
    ................................................................ccggcggauccauaauuuuacuG...........................   2   1   S03
    ................................................................ccggcggauccauaauuuuacuau..........................   3   0   S03
    ................................................................ccggcggauccauaauuuuacuUu..........................   1   1   S03
    .................................................................cggcggauccauaauuuuacu............................   1   0   S03
    .................................................................cggcggauccauaauuuuacC............................   1   1   S03
    .................................................................cggcggauccauaauuuuacua...........................   1   0   S03
    .................................................................cggcggauccauaauuuuacuauc.........................   7   0   S03
    ....................................................................cggauccauaauuuuacu............................   1   0   S03
    ................................................................................uuuacuauccuuauccUgauccga..........   1   1   S03
    .........................................................................................Uuuauccggauccgagguuuucgg.   1   1   S03

    .......ggccugggacggaucggauauu.....................................................................................   1   0   S04
```

Star Mature

gucgcuaggccugggacggaucggauauucgggcaauuuuaagguauccgaaccagaucuuuauccggcggauccauaauuuuacuauccuuauccggauccgagguuuucgga

........gccugAgacggaucggauauucgg..	1	1	S04
....cuuuauccggcggaucca......................................	1	0	S04
....uuuauccggcggauccauaauuuu...............................	2	0	S04
....uuauccggcggauccaua....................................	1	0	S04
....uuauccggcggauccauaauu.................................	1	0	S04
....uuauccggcggauccauaauuu................................	1	0	S04
....uuauccggcggauccauaauuuu...............................	1	0	S04
....uuauccggcggauccauaauuuua..............................	2	0	S04
....uauccggcggauccauaauuu................................	1	0	S04
....uauccggcggauccauaauuuu...............................	1	0	S04
....uauccggcggauccauaauuuuac.............................	3	0	S04
....uauccggcggauccauaauuuuacu............................	1	0	S04
....auccggcggauccauaauu.................................	1	0	S04
....auccggcggauccauaauuuu...............................	2	0	S04
....Guccggcggauccauaauuuu...............................	1	1	S04
....auccggcggauccauaCuuuua..............................	1	1	S04
....auccggcggauccauaauuuuacC............................	6	1	S04
....auccggcggUuccauaauuuuacu............................	1	1	S04
....auccggcggauAcauaauuuuacu............................	1	1	S04
....Uuccggcggauccauaauuuuacu............................	1	1	S04
....auccggcggauccauaauuuuacu............................	51	0	S04
....auccggcggauccauaauuuuacG............................	6	1	S04
....auccggcggaAccauaauuuuacu............................	1	1	S04
....auccggcggauccauaauuuuacua...........................	1	0	S04
....uccggcggauccauaauuuuacu............................	3	0	S04
....uccggcggauccauaauuuuacua...........................	1	0	S04
....ccggcggauccauaauuuuacu............................	1	0	S04
....ccggcggauccauaauuuuacua...........................	1	0	S04
....ccggcggauccauaauuuuacuau..........................	2	0	S04
....cggcggauccauaauuuuacuauc.........................	4	0	S04
....cggcggauccauGauuuuacuauc.........................	1	1	S04
....cggcggauccauaauuuuacCauc.........................	1	1	S04
....ggauccauaCuuuuacuauc.........................	1	1	S04
....cauaauuuuacuauccuuauccg.................	1	0	S04
....cauaauuuuacuauccuuauccgg................	1	0	S04
....aauuuuacuauccuuauccggauc.............	2	0	S04

Provisional ID	: chrC03_1516134
Score total	: 31.3
Score for star read(s)	: 3.9
Score for read counts	: 28.7
Score for mfe	: -1.1
Score for randfold	: 0
Score for cons. seed	:
Total read count	: 68
Mature read count	: 67
Loop read count	: 0
Star read count	: 1

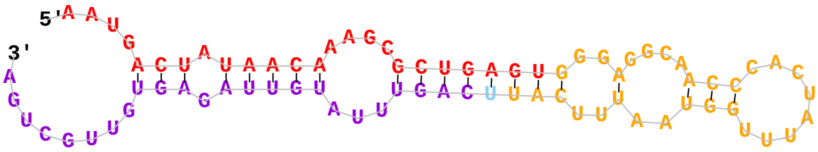

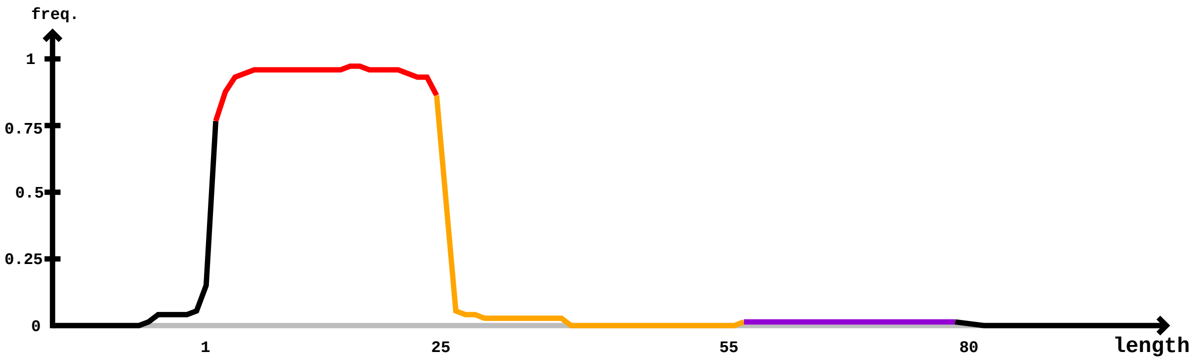

Mature **Star**

```
5'- uccaaagucaagcuaaaugacuauaacaaagcgcugagugggaggcaacccacuauuugguaauuucauucaguuuauguuagaguguugcugauuuuuguuuuuauuuuuaug  -3'   obs
    uccaaagucaagcuaaaugacuauaacaaagcgcugagugggaggcaacccacuauuugguaauuucauucaguuuauguuagaguguugcugauuuuuguuuuuauuuuuaug        exp
    ...(((((((.((.((...(((.(((((....((((((((..(....(((........)))..)..))))))))...))))).))).)))))))))))...............  reads  mm  sample
    ........cUagcuaaaugacuauaacaaag...................................................................................  1      1   S03
    .............uaaaugacuauaacaaagcgcug..............................................................................  1      0   S03
    ..............aaaugacuauaacaaagcgcugUg............................................................................  1      1   S03
    ..............aaaugacuauaacaaagcgcugag............................................................................  1      0   S03
    ...............aaugacuauaacaaagcgcugagu...........................................................................  11     0   S03
    ...............aUugacuauaacaaagcgcugagu...........................................................................  1      1   S03
    ...............aaAgacuauaacaaagcgcugagu...........................................................................  1      1   S03
    ...............aaugacAauaacaaagcgcugagu...........................................................................  1      1   S03
    ...............aaugacuauaacaaagcgcugagC...........................................................................  2      1   S03
    ...............aaugacuauaacaaaCcgcugagu...........................................................................  1      1   S03
    ................augacuauaacaaagcgcugagu...........................................................................  1      0   S03
    ................augacuauaacaaagcgcugagug..........................................................................  2      0   S03
    .................ugacuauaacaaagcgcugagCg..........................................................................  1      1   S03
    .................ugacuauaacaaagcgcugagug..........................................................................  1      0   S03
    ............................aagcgcugagugggaggcaacAca..............................................................  1      1   S03

    .........aagcuaaaugacuauaaca......................................................................................  1      0   S04
    .........aagcuaaaugacuauaacaaagcAcu...............................................................................  1      1   S04
    ..............aaaugacuauaacaaagcgAugag............................................................................  1      1   S04
    ..............aaaugacuauaacaaagcgcugag............................................................................  1      0   S04
    ..............aaaugacuauaacaaagcgcugagA...........................................................................  1      1   S04
    ..............aaaugacuauaacaaagcgcugagu...........................................................................  1      0   S04
    ..............aaaugacuauaacaaagGgcugagu...........................................................................  1      1   S04
    ...............aaugacuauaaUaaagcgcugag............................................................................  1      1   S04
    ...............aaugacuauaaUaaagcgcugagu...........................................................................  2      1   S04
    ...............aaugacuauaacaaagcgcugagu...........................................................................  20     0   S04
    ...............aaugacuaCaacaaagcgcugagu...........................................................................  1      1   S04
    ...............aaugacuauaacaaagcgcugagA...........................................................................  2      1   S04
    ...............aaugacuauaacaaagcgcugaCu...........................................................................  1      1   S04
    ...............aaugacuauaacaaagcAcugagu...........................................................................  1      1   S04
    ................augacuauaacaaagcgcugagu...........................................................................  1      0   S04
    ................augacuauaacaaagcgcugaCug..........................................................................  1      1   S04
    ................augacuauaacaaagcgcugagug..........................................................................  2      0   S04
```

```
                 Mature                                                 Star
uccaaagucaagcuaaaugacuauaacaaagcgcugagugggaggcaacccacuauuugguaauuucauucaguuuauguuagaguguugcugauuuuuguuuuuauuuuuaug
................augacuauaacaaagcgcugaAug..........................................................................     1     1     S04
.................ugacuauaacaaagcgcugagA...........................................................................     1     1     S04
.................ugacuauaacaaagcgcuAagugg.........................................................................     1     1     S04
..................gacuauaacaaagcgcugagu...........................................................................     1     0     S04
...................acuauaacaaagcgcugaguggga.......................................................................     1     0     S04
.............................agcgcugagugggaggcaacccG..............................................................     1     1     S04
......................................................................caguuuauguuagaguguugcuga....................     1     0     S04
```

Provisional ID	:	chrC03_1526870
Score total	:	0.1
Score for star read(s)	:	-1.3
Score for read counts	:	0
Score for mfe	:	1.4
Score for randfold	:	0
Score for cons. seed	:	
Total read count	:	23
Mature read count	:	8
Loop read count	:	12
Star read count	:	3

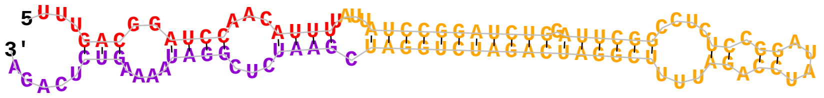

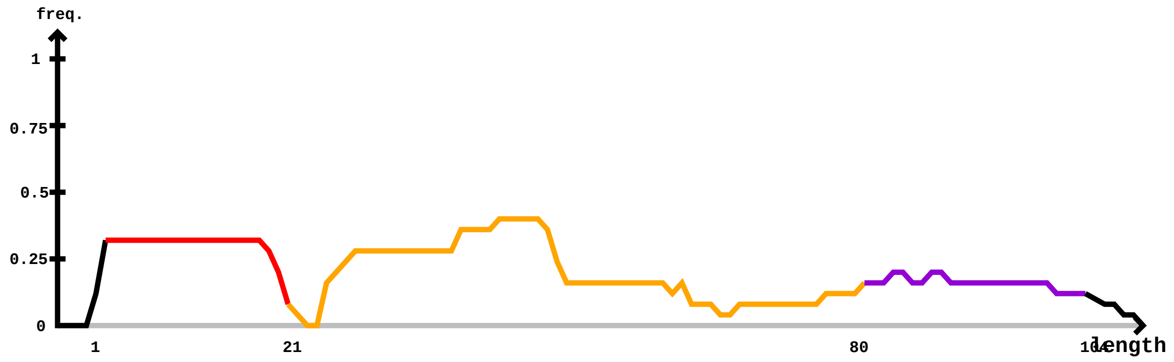

Mature **Star**

```
5'- ggcuuugacggauccaacauuuuauuauccggaucuggauucggccucuccggauauccagauuuucggaucagaucuggaucgaaucucggauaaaagucucagaccuac  -3'  obs
    ggcuuugacggauccaacauuuuauuauccggaucuggauucggccucuccggauauccagauuuucggaucagaucuggaucgaaucucggauaaaagucucagaccuac       exp
    ((((..(((..((((...((((....((((((((((.((((((....((.((....)).))...)))))))))))))))).))))...))))....)))..)).))...  reads  mm  sample
    ..cuuugacggauccaacau...........................................................................................  1  0  S03
    ...uuugacggauccaacauuC.........................................................................................  2  1  S03
    ............................ccggaucuggauucggccucucA............................................................  1  1  S03
    ...............................................................uuucggaucagaucuggaucgaau........................  1  0  S03
    ..............................................................................ggaucgaaucucggauaaaagucG.........  1  1  S03
    .....................................................................................aucucggauaaaagucucagaAcu..  1  1  S03

    ..cuuugacggauccaacauu..........................................................................................  1  0  S04
    ..cuuugacggauccaacauuu.........................................................................................  1  0  S04
    ...uuugacggauccaacauu..........................................................................................  1  0  S04
    ...uuugacggauccaacauuuu........................................................................................  2  0  S04
    ..........................auccggaucuggauucggccucG..............................................................  1  1  S04
    ..........................auccggaucuggauucgAccucuc.............................................................  1  1  S04
    ..........................auccggaucuggauucggccucuc.............................................................  2  0  S04
    ...........................uccggaucuggauucggccucucA............................................................  1  1  S04
    .............................cggaucuggauucggccu................................................................  1  0  S04
    ........................................ucggccucuccggauauccagauu...............................................  1  0  S04
    ........................................ucggccucucUggauauccagauu...............................................  1  1  S04
    ............................................ccucuccggauauccaUa.................................................  1  1  S04
    ...............................................cuccggauauccagauuuuc............................................  1  0  S04
    .....................................................................aucagaucuggaucgaaucucC....................  1  1  S04
    ..................................................................................cgaaucucggauaaaagucucaga.....  1  0  S04
    .........................................................................................cggauaaaaUucucagaccuac  1  1  S04
```

Provisional ID	:	chrC03_1539994
Score total	:	0.5
Score for star read(s)	:	3.9
Score for read counts	:	-4.9
Score for mfe	:	1.6
Score for randfold	:	0
Score for cons. seed	:	
Total read count	:	2
Mature read count	:	1
Loop read count	:	0
Star read count	:	1

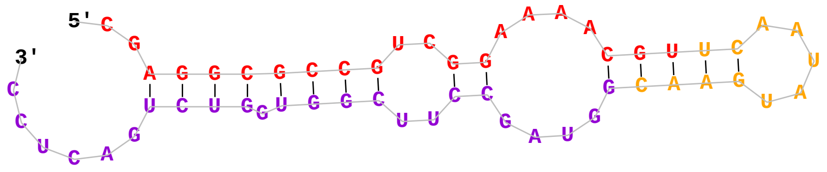

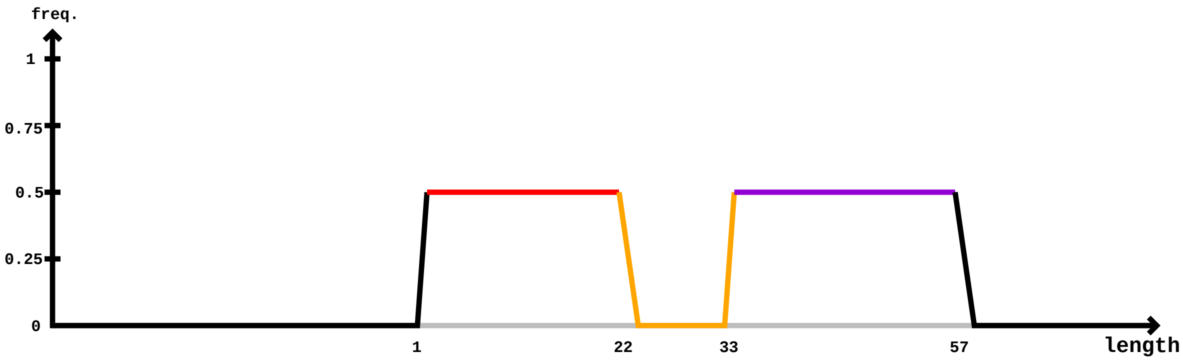


```
                                     Mature                        Star
5'- guucgcccuaauuucaaaaccucucaggcccagguuucgaggcgccgucggaaaacguucaauaugaacgguagccuucgguggucugacuccgaccugggcuccguuccaau   -3'    obs
    guucgcccuaauuucaaaaccucucaggcccagguuucgaggcgccgucggaaaacguucaauaugaacgguagccuucgguggucugacuccgaccugggcuccguuccaau          exp
    ..........................(((((((((((((.(.(((((..((....(((((.....)))))....))..))))).).)))....))))))))))..........   reads      mm         sample
    ....................................................................gguagccuucgguggucugacucU.....................   1          1          S03

    .....................................cgaggcgccgucggaaaacgu.......................................................   1          0          S04
```

Provisional ID	:	chrC03_1545569
Score total	:	0.4
Score for star read(s)	:	-1.3
Score for read counts	:	0
Score for mfe	:	1.7
Score for randfold	:	0
Score for cons. seed	:	
Total read count	:	18
Mature read count	:	2
Loop read count	:	0
Star read count	:	16

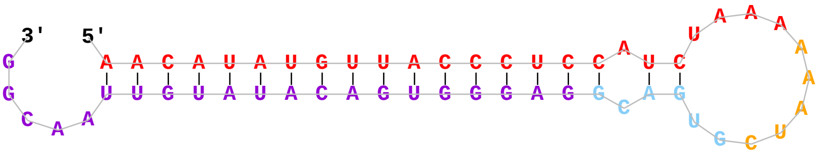

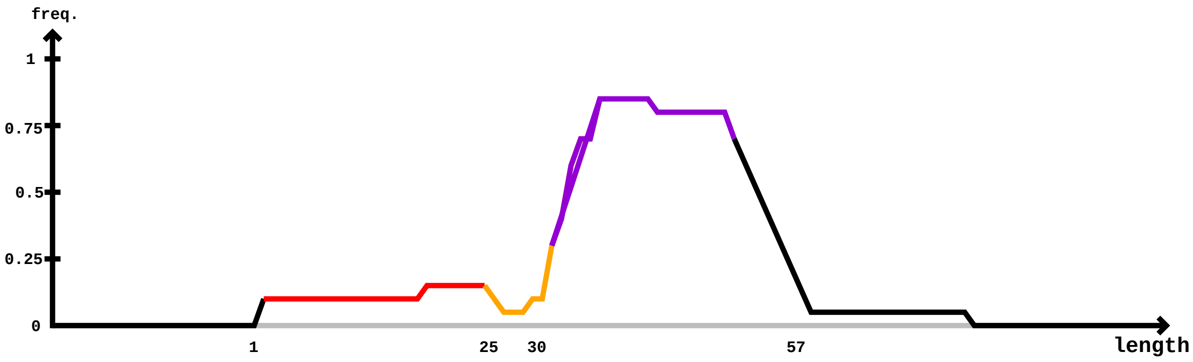

Mature **Star**

```
5'- acuucaauuuuaauuuuguuaacauauguuacccuccaucuaaaaaaucgugacggagggugacauauguuaacgguuuauaaaauucaggguuuacaauguugaaaacuuaguu   -3'   obs
    acuucaauuuuaauuuuguuaacauauguuacccuccaucuaaaaaaucgugacggagggugacauauguuaacgguuuauaaaauucaggguuuacaauguugaaaacuuaguu          exp
    .(((.(((((.....((((((((((((((((((((((.((...........)).))))))))))))))))))))))).....))))).))).........................   reads   mm   sample
    ................................................cgugacggagggugacauaug..............................................   1       0    S03
    ..................................................ugacggagggugacauauguu............................................   1       0    S03
    ...................................................gacggagggugacauauguuaacg........................................   1       0    S03
    ....................................................acggagggugacauauguuaacA........................................   1       1    S03
    .....................................................cggagggugacauauguuaacgg.......................................   1       0    S03
    .....................................................cggagggugacauauguuaacggG......................................   1       1    S03
    .......................................................gagggugGcauauguuaacgg.......................................   1       1    S03
    .......................................................................aacgguuuauaaaauucaggguuu....................   1       0    S03

    ....................aacauauguuacccuccaucuaaa.......................................................................   2       0    S04
    .....................................aucuaaaaaaucgugacggaAggu......................................................   1       1    S04
    ..................................................ugacggagggugacauauguu............................................   1       0    S04
    ..................................................ugacggagggugacauauguuaac.........................................   1       0    S04
    ..................................................ugacggagggugCcauauguuaac.........................................   1       1    S04
    ...................................................gacUgagggugacauaug..............................................   1       1    S04
    ....................................................acggagggugacauauguuaac.........................................   2       0    S04
    ....................................................acggagggugacauauguuaacg........................................   1       0    S04
    .......................................................gagggugacauauguuaacgg.......................................   2       0    S04
```

Provisional ID	:	chrC03_random_2825340
Score total	:	1
Score for star read(s)	:	3.9
Score for read counts	:	-4.4
Score for mfe	:	1.6
Score for randfold	:	0
Score for cons. seed	:	
Total read count	:	3
Mature read count	:	2
Loop read count	:	0
Star read count	:	1

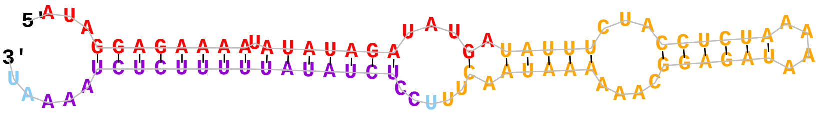

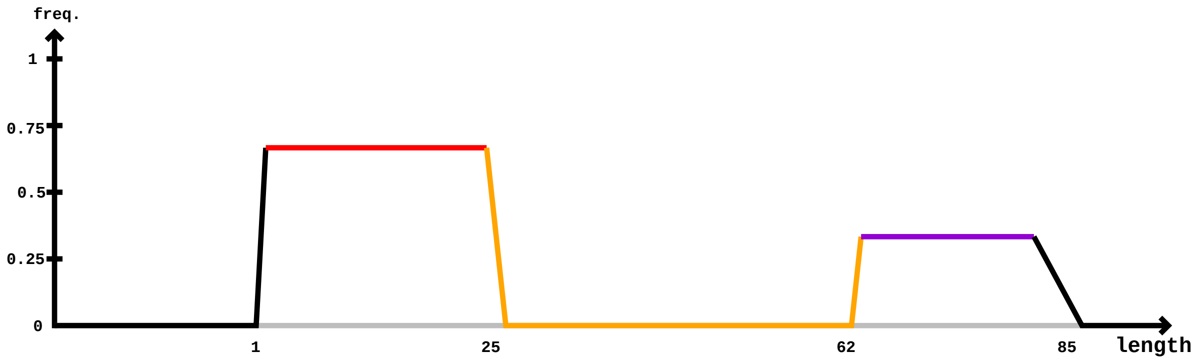

Mature Star

```
5'- auaauagaguuucucuauuuauaggagaaaauauauagauaugauauuucuaccucuaaaaauagaggcaaaaaaauaacuuuccucuauauuuuucucuaaaauagaggaacuc   -3'    obs
    auaauagaguuucucuauuuauaggagaaaauauauagauaugauauuucuaccucuaaaaauagaggcaaaaaaauaacuuuccucuauauuuuucucuaaaauagaggaacuc          exp
    ......((((((((((((((...((((((((.(((((((...(.(((((...((((((....))))))....))))).).....))))))))))))))).))))))))))))))   reads      mm         sample
    ....................auaggagaaaauauauagauauga.......................................................................   2          0          S03

    ..................................................................................ccucuauauuuuucucuaaa............   1          0          S04
```

Provisional ID	:	chrC03_random_2826462
Score total	:	225.8
Score for star read(s)	:	3.9
Score for read counts	:	224.4
Score for mfe	:	-2.4
Score for randfold	:	0
Score for cons. seed	:	
Total read count	:	452
Mature read count	:	440
Loop read count	:	0
Star read count	:	12

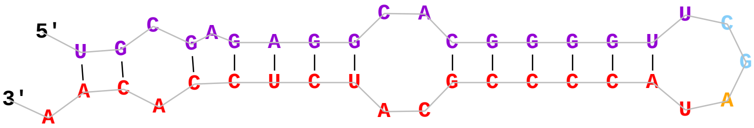

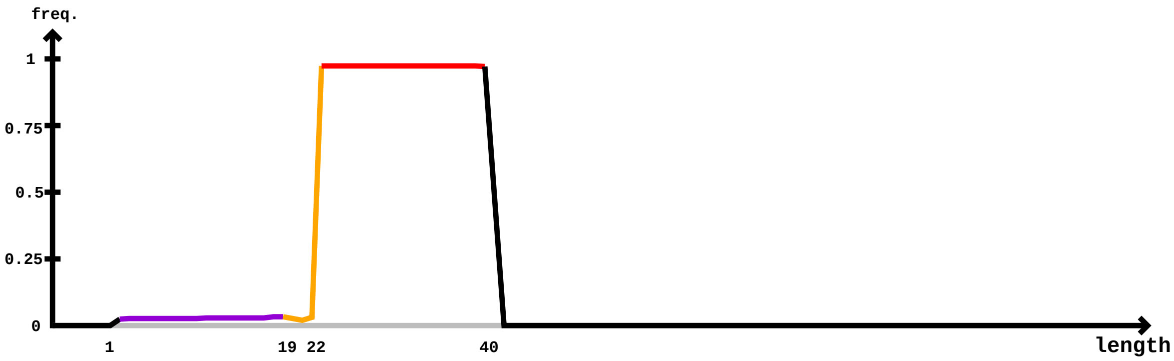

Star **Mature**

```
5'- uugcaugcgagaggcacgggguucgauaccccgcaucuccacaaauuuuaauuuuuucuugcuuuuaaauuacaagcccagacccaaucugcucaacgugcucaucuauuuu  -3'  obs
    uugcaugcgagaggcacgggguucgauaccccgcaucuccacaaauuuuaauuuuuucuugcuuuuaaauuacaagcccagacccaaucugcucaacgugcucaucuauuuu        exp
    ..(((((.(.((((..((((((.....))))))..))))).....................((((.........)))).((((.....)))).....))))).......... reads  mm  sample
    .....ugcgagaggcacgggguC......................................................................................... 4      1   S03
    .....ugcgagaggcacggggGu......................................................................................... 1      1   S03
    .....ugcgagaggcacgggguG......................................................................................... 1      1   S03
    .....Agcgagaggcacgggguu......................................................................................... 1      1   S03
    .....uUcgagaggcacgggguu......................................................................................... 1      1   S03
    ..............cacgggguucgauaccccgcaucuccacCa.................................................................... 1      1   S03
    .....................uucgauaccccgcaucuccacCa.................................................................... 2      1   S03
    ........................gauaccccgcaucuccacCa.................................................................... 2      1   S03
    .........................auaccccgcaucuccacCa.................................................................... 3      1   S03
    ..........................uaccccgcaucuccacCa.................................................................... 220    1   S03

    .....ugcgagaggcacgggguA......................................................................................... 2      1   S04
    .....Ggcgagaggcacgggguu......................................................................................... 1      1   S04
    ......gcgagaggcacgggguucC....................................................................................... 1      1   S04
    ........................gauaccccgcaucuccacC..................................................................... 1      1   S04
    ........................gauaccccgcaucuccacCa.................................................................... 2      1   S04
    .........................auaccccgcaucuccacCa.................................................................... 3      1   S04
    ..........................uCcccgcaucuccacaa..................................................................... 1      1   S04
    ..........................uaccccgcaucuccacCa.................................................................... 208    1   S04
```

Provisional ID	:	chrC03_random_2827735
Score total	:	40.4
Score for star read(s)	:	3.9
Score for read counts	:	37.3
Score for mfe	:	-0.7
Score for randfold	:	0
Score for cons. seed	:	
Total read count	:	85
Mature read count	:	84
Loop read count	:	0
Star read count	:	1

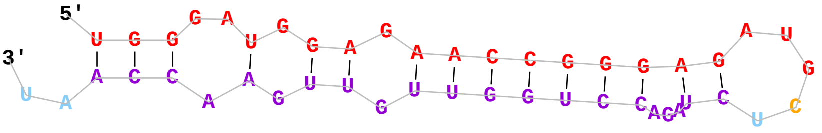

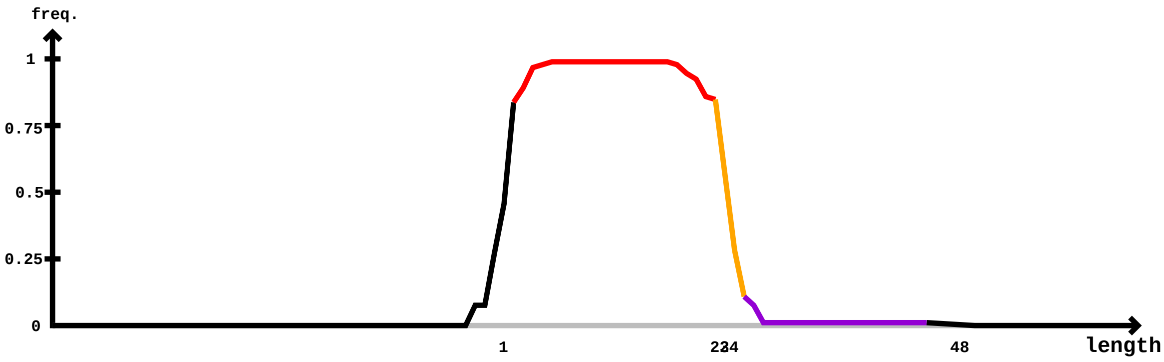


```
                                                                   Mature          Star
5'- aaauaaaauaaaaauacuaaccaaguuguaugucccggucauggguugggauggagaaccgggagaugcucuagaccugguuguugaaccaauuguugaacugacaugacc  -3'  obs
    aaauaaaauaaaaauacuaaccaaguuguaugucccggucauggguugggauggagaaccgggagaugcucuagaccugguuguugaaccaauuguugaacugacaugacc       exp
    ............................((((((....(((..((((((..(.((.(((((((((.....))...))))))).)).).))))))..)))...))))))...    reads    mm    sample
    ..........................................ggguugggauggagaaAcggga...............................................    2        1     S03
    ..........................................ggguugggauggagaaAcgggag..............................................    1        1     S03
    ..........................................ggguugggauggagaaAcgggaga.............................................    3        1     S03
    ............................................guugggauggagaaAcgggaga.............................................    3        1     S03
    ............................................guugggauggagaaAcgggagaug...........................................    13       1     S03
    .............................................uugggauggagaaAcggg................................................    1        1     S03
    .............................................uugggauggagaaAcgggag..............................................    1        1     S03
    .............................................uugggauggagaaAcgggagaug...........................................    7        1     S03
    .............................................uugggauggagaaccgggagaug...........................................    2        0     S03
    .............................................uugggauggagaaAcgggagaugc..........................................    3        1     S03
    ..............................................ugggauggagaaAcgggagaug...........................................    17       1     S03
    ..............................................ugggauggagaaAcgggagaugc..........................................    4        1     S03
    ..............................................ugggauggagaaccgggagaugcG.........................................    1        1     S03
    ..............................................ugggauggagaaAcgggagaugcu.........................................    9        1     S03
    ...............................................gggauggagaaAcgggagaugcu.........................................    1        1     S03
    ...............................................gggauggagaaAcgggagaugcuc........................................    1        1     S03
    ................................................ggauggagaaAcgggagau............................................    1        1     S03
    ................................................ggauggagaaAcgggagaugcucu.......................................    6        1     S03
    .................................................gauggagaaAcgggagaugcu.........................................    1        1     S03
    ..................................................auggagaaAcgggagaugcuc........................................    1        1     S03

    ..........................................ggguugggauggagaaAcgggagaugcu.........................................    1        1     S04
    ............................................guugggauggagaaccgggagaug...........................................    1        0     S04
    ............................................guugggauggagaaAcgggagaug...........................................    1        1     S04
    .............................................uugggauggagaaccgggagaugc..........................................    1        0     S04
    .............................................uugggauggagaaAcgggagaugc..........................................    2        1     S04
    ..............................................ugggauggagaaAcggga...............................................    1        1     S04
    ..............................................ugggauggagaaAcgggagaug...........................................    1        1     S04
    ..............................................ugggauggagaaAcgggagaugcu.........................................    2        1     S04
    ...............................................gggauggagaaAcgggagaugcu.........................................    1        1     S04
    ...............................................gggauggagaaccgggagaugcu.........................................    1        0     S04
    ...............................................gggauggagaaAcgggagaugcuc........................................    1        1     S04
    ......................................................................cuagaccugguuguugaacca....................    1        0     S04
```

Mature Star

aaauaaaauaaaaauacuaaccaaguuguaugucccggucaugggguugggauggagaaccgggagaugcucuagaccugguuguugaaccaauuguugaacugacaugacc

Provisional ID	:	chrC03_random_2830295
Score total	:	38.8
Score for star read(s)	:	3.9
Score for read counts	:	41.4
Score for mfe	:	-6.4
Score for randfold	:	0
Score for cons. seed	:	
Total read count	:	93
Mature read count	:	92
Loop read count	:	0
Star read count	:	1

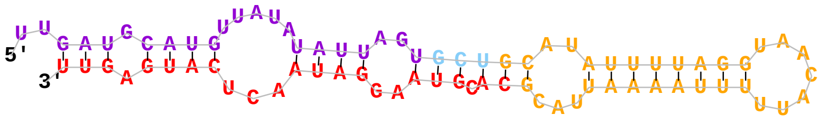

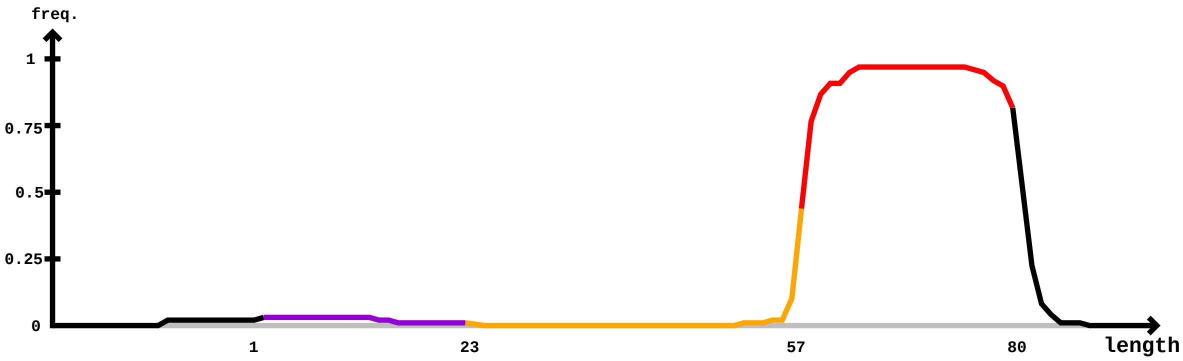


```
                    Star                                                      Mature
5'- ugagucaaauucuaguuuuauugaugcauguuauauauuagugcugcauauuuuagguaacauuuuuaaaauuacgcacguaaggauaacucaugaguuuuuugguugauuaa  -3'   obs
    ugagucaaauucuaguuuuauugaugcauguuauauauuagugcugcauauuuuagguaacauuuuuaaaauuacgcacguaaggauaacucaugaguuuuuugguugauuaa        exp
    ..((((((...((((....(((.(((...(((((...(((((((.....((((((((.......))))))))...)))).)))..))))).))).)))...))))))))))..    reads    mm    sample
    ....................uugaugcauguuauauauuagu.......................................................................    1        0     S03
    .........................................................................acgcacguaaggauaacucaugag................    1        0     S03
    ...........................................................................gcacguaaggauaacucauga.................    1        0     S03
    ...........................................................................gcacguaaggauaacucaugagu...............    1        0     S03
    ...........................................................................gcacguaaggauaacucaugaguC..............    1        1     S03
    ...........................................................................gcacguaaggauaacucaugaguu..............    1        0     S03
    ............................................................................cacguaaggauaacucauga.................    1        0     S03
    ............................................................................cacguaaggauaacucaugag................    1        0     S03
    ............................................................................cacguaaggauaacucaugaguu..............    9        0     S03
    ............................................................................cacguaaggauaacucaugaguuC.............    1        1     S03
    ............................................................................cacguaaggauaacucaugaguuu.............    2        0     S03
    ............................................................................cUcguaaggauaacucaugaguuu.............    1        1     S03
    .............................................................................acguaaggauaacucaugagu...............    1        0     S03
    .............................................................................acguaaggauaacucaugaguu..............    2        0     S03
    .............................................................................acguaaggauaacucaugaguuu.............    3        0     S03
    .............................................................................acguaaggauaacucaugaguuuC............    1        1     S03
    .............................................................................acguaaggauaacucaugaguuuu............    8        0     S03
    .............................................................................acguaaggauaacucaugaguuuuu...........    1        0     S03
    ..............................................................................cguaaggauaacucaugagu...............    1        0     S03
    ..............................................................................cguaaggauaacucaugaguu..............    2        0     S03
    ..............................................................................cguaaggauaacucaugaguuuuu...........    1        0     S03
    ...............................................................................guaaggauaacucaugaguu..............    1        0     S03
    ...............................................................................guaaggauaacucaugaguuu.............    1        0     S03
    ...............................................................................guaaggauaacucaugaguuG.............    1        1     S03
    .................................................................................aaggauaacucaugaguu..............    2        0     S03

    ..........ucuaguuuuauugaugUauguu.................................................................................    1        1     S04
    ..........ucuaguuuuauugaugcauguuau...............................................................................    1        0     S04
    ......................................................................auuacgcacguaaggauaacucaG...................    1        1     S04
    ...........................................................................gcacguaaggauaacucauga.................    1        0     S04
    ...........................................................................gcacguaaggauaacucaugagu...............    1        0     S04
    ...........................................................................gcacguaaggauaacucaugaguu..............    2        0     S04
    ............................................................................cacguaaggauaacucaug..................    1        0     S04
```

Star **Mature**

```
ugagucaaauucuaguuuuauugaugcauguuauauauuagugcugcauauuuuagguaacauuuuuaaaauuacgcacguaaggauaacucaugaguuuuuugguugauuaa
............................................................................cacguaaggauaacucaugagu...............	1	0	S04
............................................................................cacguaaggauaacucaugUgu...............	1	1	S04
............................................................................cacguaaggauaacucaugaguA..............	1	1	S04
............................................................................cacguaaggauaacucaugaguu..............	8	0	S04
............................................................................cacguaaggauaacucaugaguuu.............	5	0	S04
............................................................................caNguaaggauaacucaugaguuu.............	1	1	S04
.............................................................................acguaaggauaacucaugagu...............	1	0	S04
.............................................................................acguaaggauaacucaugaguu..............	2	0	S04
.............................................................................acguaaggauaacucaugaguuu.............	7	0	S04
.............................................................................acguaaggauaacucaugaguuC.............	1	1	S04
.............................................................................acguaaggauaacucaugaguuuC............	1	1	S04
.............................................................................acguaaggauaacucaugaguuuu............	4	0	S04
..............................................................................cguaaggauaacucaugagu...............	1	0	S04
..............................................................................cguaaggauaacucaugaguu..............	1	0	S04
..............................................................................cguaaggauaacucaugaguuu.............	2	0	S04
..............................................................................cguaaggauaacucaugaCuuuu............	1	1	S04
..............................................................................cguaaggauaacucaugaguuuuA...........	1	1	S04
...............................................................................Auaaggauaacucaugaguuuuuu..........	1	1	S04
.................................................................................aaggauaacucaugaguuC.............	1	1	S04
.................................................................................aaggauaacucaugaguuuuuu..........	1	0	S04
..................................................................................aggauaacucaugaguuuuuu..........	1	0	S04
..................................................................................aggauaacucaugaguuuuuuggu.......	1	0	S04
```

Provisional ID	:	chrC03_random_2838361
Score total	:	1
Score for star read(s)	:	-1.3
Score for read counts	:	0
Score for mfe	:	2.3
Score for randfold	:	0
Score for cons. seed	:	
Total read count	:	19
Mature read count	:	19
Loop read count	:	0
Star read count	:	0

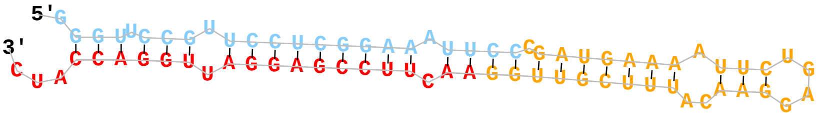

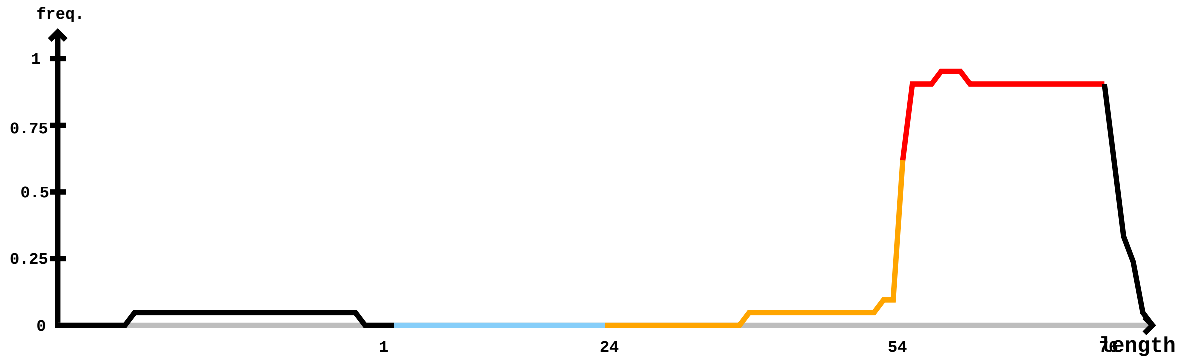

Star **Mature**

```
5'- auaccgagggacuccauccucggaauuuuccgaggguuccguuccucggaaauucccgaugaaaauucugaggaacauuucguuggaacuuccgaggauuggaccaucggaa  -3'   exp
    ...(((((((......)))))))....((((((.(((.(((.(((((((((.((((.(((((((.(((....)))..))))))))))).))))))))).)))))).))))))   reads    mm    sample
    ......................................................................aggaacauuucguuggaacuucA...................   1        1     S03
    ....................................................................................ggaacuuccgaggauuggaccaucg...   1        0     S03
    ......................................................................................aacuuccgaggauuggaccauc....   3        0     S03
    ......................................................................................aacuuccgaggauuggaccaucg...   1        0     S03
    .......................................................................................acuuccgaggauuggaccauUgga.   1        1     S03
    ..........................................................................................uccgaggauuggaccaucAgaa   1        1     S03

    ......agggacuccUuccucggaauuuuc....................................................................................   1        1     S04
    ......................................................................................aacuuccgaggauuggaccauc....   1        0     S04
    ......................................................................................aacuuccgaggauuggaccauU....   1        1     S04
    ......................................................................................aacuuccgaggauuggaccaucg...   1        0     S04
    ......................................................................................aacuuccgaggauuggaccaucU...   1        1     S04
    ......................................................................................aacuuccgaggauuggaccaucA...   2        1     S04
    ......................................................................................aacuuccgaggauuggaccaucgg..   1        0     S04
    .......................................................................................acuuccgaggauuggaccauc....   1        0     S04
    .......................................................................................acuuccgaggauuggaccaucgg..   1        0     S04
    .......................................................................................acuuccgaggauuggaccauAgga.   1        1     S04
    .......................................................................................acuuccgaggauuggaccaucgga.   2        0     S04
```

Provisional ID : chrC03_random_2840332
Score total : 0.7
Score for star read(s) : -1.3
Score for read counts : 0
Score for mfe : 2
Score for randfold : 0
Score for cons. seed :
Total read count : 40
Mature read count : 24
Loop read count : 16
Star read count : 0

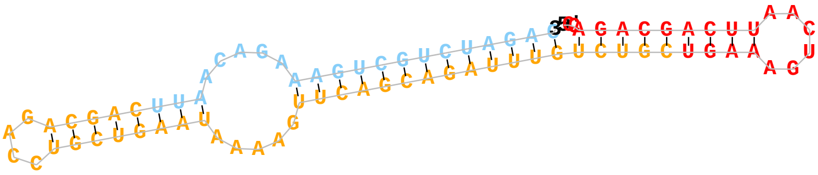

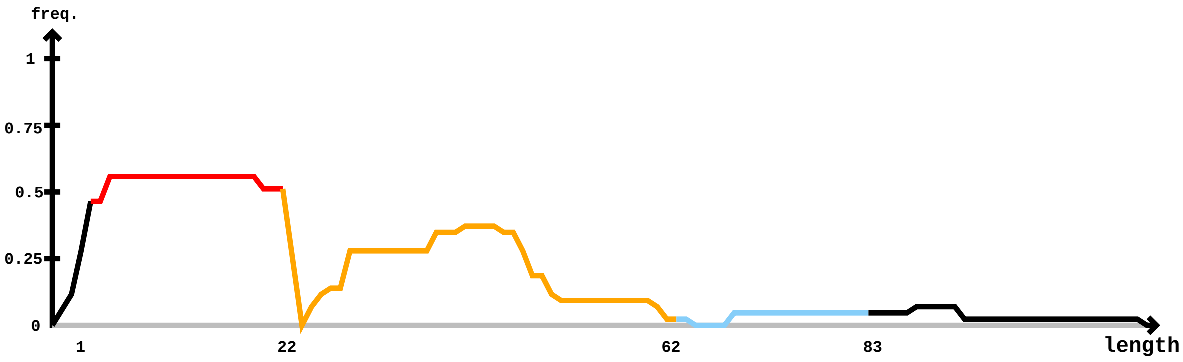

Mature **Star**

```
5'- guccagacgacuuaacugaaagucgucuguuuagacgacuugaaaauaagucguccagacgacuuaacagaaagucgucuagacaguuagucuuaaacauaauuuaaaaauuu   -3'    exp
    (((.(((((((((..(((.((((((((((....(((((((((....)))))))))))))))))))..))).))))))))).))).............................   reads   mm   sample
    gGccagacgacuuaacugaaaguc.........................................................................................   1       1    S03
    guccagacgacuuaacugaaaguA.........................................................................................   1       1    S03
    guccagacgacuuaCcugaaaguc.........................................................................................   2       1    S03
    .uccagacgacuuaacugaa.............................................................................................   1       0    S03
    .uccagacgacuuaacugaaagu..........................................................................................   2       0    S03
    ..ccagacgCcuuaacugaaagu..........................................................................................   1       1    S03
    ..ccagacgacuuaacugaaagu..........................................................................................   5       0    S03
    ....agacgacuuaacugaaagu..........................................................................................   1       0    S03
    ..........................cuguuuagacgacuugaaaaua.................................................................   1       0    S03
    ...........................uguuuagacgacuugaaaau..................................................................   1       0    S03
    .............................uuuagacgacuugaaaau..................................................................   1       0    S03
    .............................uuuagacgacuugaaaaua.................................................................   1       0    S03
    .............................uuuagacgacuugaaaauaaA...............................................................   1       1    S03
    .............................uuuagacgacuugaaaauaag...............................................................   2       0    S03
    .............................uuuagacgacuugaaaauaagu..............................................................   1       0    S03
    ......................................cuugaaaGuaagucguccagacg....................................................   1       1    S03
    ......................................cuugaaaauaagucguccagacgU...................................................   2       1    S03
    .....................................................................gaaagucgucuagacaguuagucu....................   1       0    S03

    guccagacgacuuaacugaaagu..........................................................................................   1       0    S04
    .uccagacgacuuaacugaa.............................................................................................   1       0    S04
    .uccagacgacuuaacugaaagu..........................................................................................   3       0    S04
    ..ccagacgacuuaacugaaagu..........................................................................................   2       0    S04
    ....agacgacuuaacugaaagu..........................................................................................   2       0    S04
    ....agacgacuuaacugaaaguU.........................................................................................   1       1    S04
    .........................ucuguuuagacgacuugaaa....................................................................   1       0    S04
    .........................ucuguuuagacgacuugaaaau..................................................................   1       0    S04
    .........................ucuguuuagacgacuugaaaaua.................................................................   1       0    S04
    ..........................cuguuuagacgacuugaaaauC.................................................................   1       1    S04
    .........................................gaaaauaagucguccagacgacuu................................................   1       0    S04
    .....................................................................gaaagucgucuagacaguuagucC....................   1       1    S04
    ........................................................................................agucuuaaacauaauuuaaaaauC.   1       1    S04
```

Provisional ID	:	chrC04_1557336
Score total	:	4.4
Score for star read(s)	:	3.9
Score for read counts	:	0.1
Score for mfe	:	0.4
Score for randfold	:	0
Score for cons. seed	:	
Total read count	:	12
Mature read count	:	8
Loop read count	:	0
Star read count	:	4

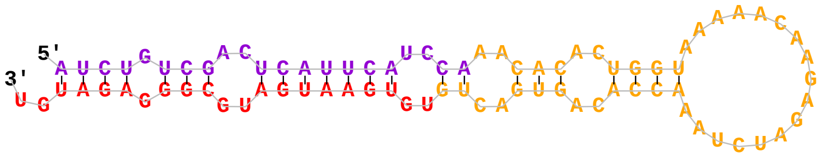

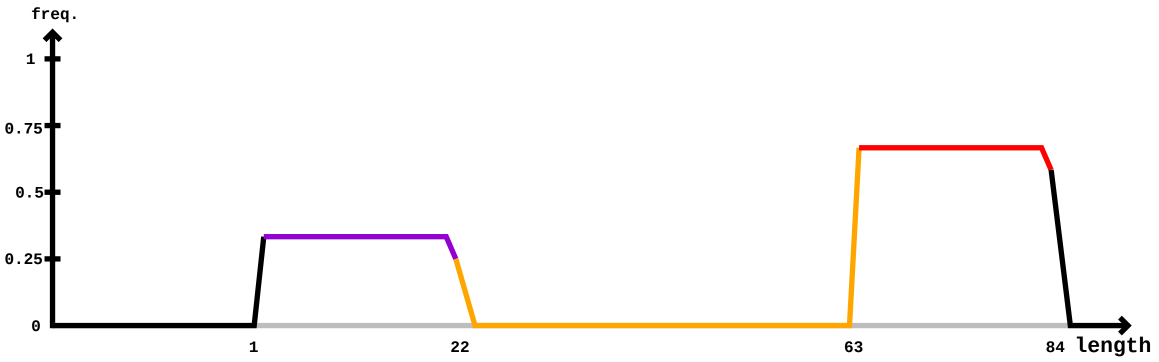


```
                    Star                                                        Mature
5'- uagaacaagaggguagaaauaucugucgacucauucauccaaacacacugguaaaaacaagagaucuaaaccacagugacugugugaaugaugcgggagauguuuuucgu   -3'    obs
    uagaacaagaggguagaaauaucugucgacucauucauccaaacacacugguaaaaacaagagaucuaaaccacagugacugugugaaugaugcgggagauguuuuucgu          exp
    ............(.((((((((((.(((..(((((((..((..(((..((((.................))))..)))..))..)))))))..))).))))))))))).. reads    mm    sample
    ....................aucugucgacucauucaucc......................................................................  1       0     S03
    ....................aucugucgacucauucaucca.....................................................................  2       0     S03
    ....................aucugucgacucauucauccaU....................................................................  1       1     S03
    ..................................................................................ugugaaugaugcgggagaugu.......  2       0     S03

    ..................................................................................ugugaaugaugcgggagauA........  1       1     S04
    ..................................................................................ugugaaugaugcgggagaugC.......  1       1     S04
    ..................................................................................ugugaaugaugcgggagaugu.......  2       0     S04
    ..................................................................................ugCgaaugaugcgggagaugu.......  1       1     S04
    ..................................................................................ugugaaugaugcgggagauguu......  1       0     S04
```

Provisional ID	:	chrC04_1566315
Score total	:	2.2
Score for star read(s)	:	3.9
Score for read counts	:	5.2
Score for mfe	:	-6.9
Score for randfold	:	0
Score for cons. seed	:	
Total read count	:	22
Mature read count	:	20
Loop read count	:	1
Star read count	:	1

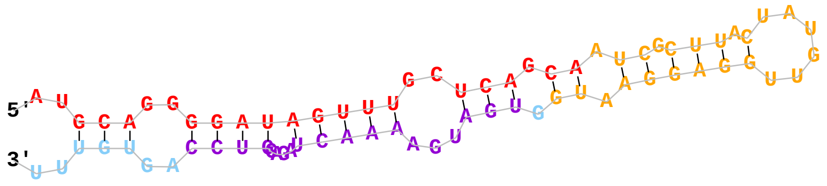

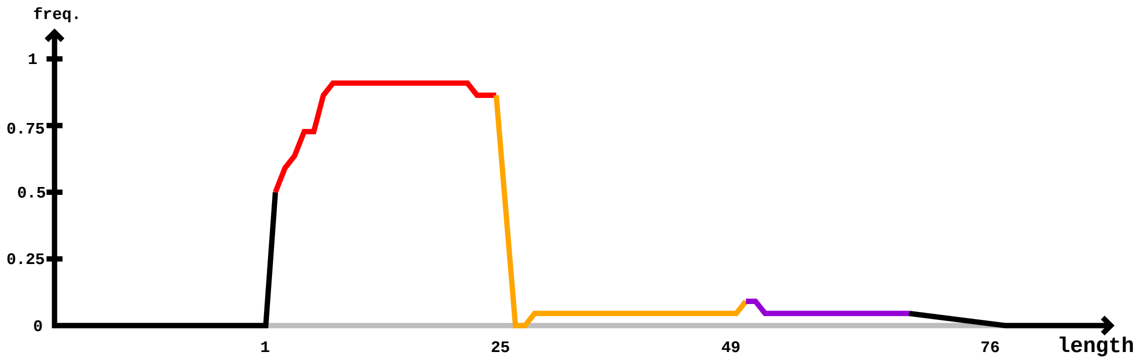


```
                                    Mature                                            Star
5'- uuaauuaugguaauaaaauggaugcaggggauaguuugcucagcaaucgcuuacuauguuggaggaauggugaugaaaacuagagguccaguguuuguaacagcuuuu  -3'  obs
    uuaauuaugguaauaaaauggaugcaggggauaguuugcucagcaaucgcuuacuauguuggaggaauggugaugaaaacuagagguccaguguuuguaacagcuuuu        exp
    .................(..(((((..(((.((((((..(((.((.((.(((.(......)))))).)).)))...))))))....))).)))))..)..........   reads  mm  sample
    .....................augcaggggauaguGugcucagca...............................................................   6      1   S03
    ......................ugcaggggauaguGugcucagca...............................................................   1      1   S03
    .......................gcaggggauaguGugcucagca...............................................................   1      1   S03
    ........................caggggauaguGugcucagca...............................................................   1      1   S03
    ........................caggggauaguGugcucagcaa..............................................................   1      1   S03
    ..........................ggggauaguGugcucagca...............................................................   1      1   S03
    ................................................Acuuacuauguuggaggaauggug....................................   1      1   S03
    ......................................................................ugaugaaaacuagaggucU...................   1      1   S03

    .....................augcaggggauaguGugcucagca...............................................................   5      1   S04
    ......................ugcaggggauaguAugcuca..................................................................   1      1   S04
    ..........................ggggauaguGugcucagca...............................................................   2      1   S04
    ...........................gggauaguGugcucagca...............................................................   1      1   S04
```

Provisional ID	:	chrC04_1569843
Score total	:	0.2
Score for star read(s)	:	3.9
Score for read counts	:	-4.9
Score for mfe	:	1.3
Score for randfold	:	0
Score for cons. seed	:	
Total read count	:	2
Mature read count	:	1
Loop read count	:	0
Star read count	:	1

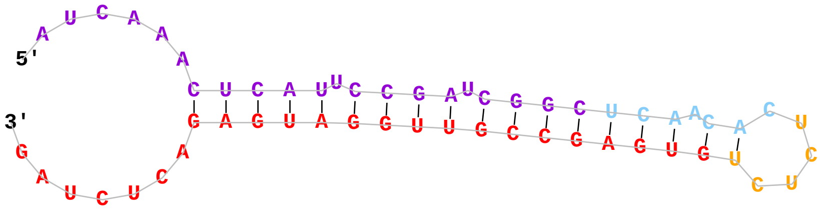

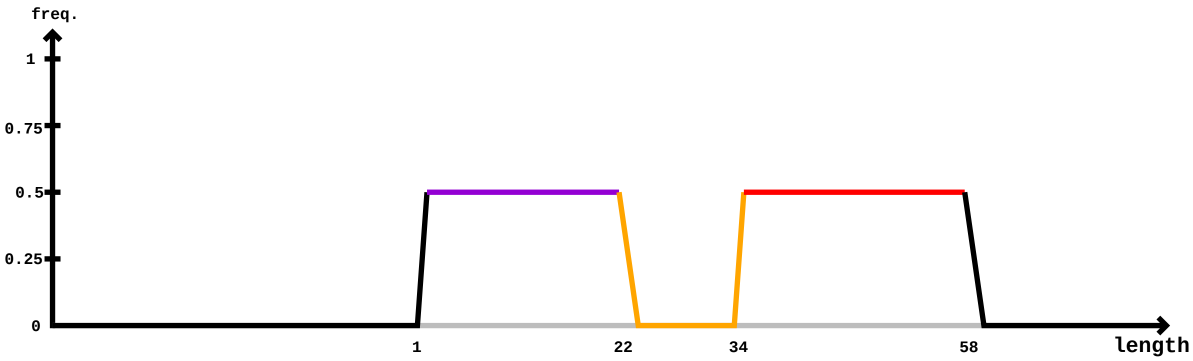

Star **Mature**

```
5'- cgccgccgccaacacuccccgacaucagcggcgcagaaucaaacucauuccgaucggcucaacacucucugugagccguuggaugagacucuaggccacucuuacugcuacguc   -3'    obs
    cgccgccgccaacacuccccgacaucagcggcgcagaaucaaacucauuccgaucggcucaacacucucugugagccguuggaugagacucuaggccacucuuacugcuacguc          exp
    .((((((((..................)))))...........(((((.((((.(((((((.((.....))))))))))))))))))......)))..................   reads      mm         sample
    .....................................aucaaacucauuccgaucgAc........................................................   1          1          S03
    ......................................................................gugagccguuggaugagacucuag....................   1          0          S03
```

Provisional ID	:	chrC04_1570307
Score total	:	3.9
Score for star read(s)	:	3.9
Score for read counts	:	18
Score for mfe	:	-17.8
Score for randfold	:	0
Score for cons. seed	:	
Total read count	:	47
Mature read count	:	46
Loop read count	:	0
Star read count	:	1

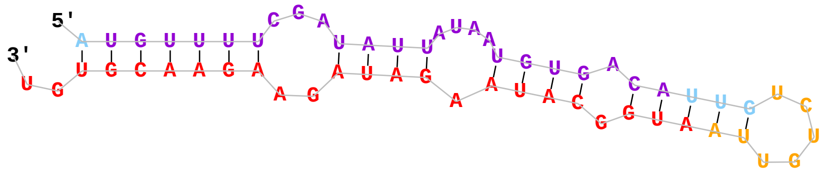

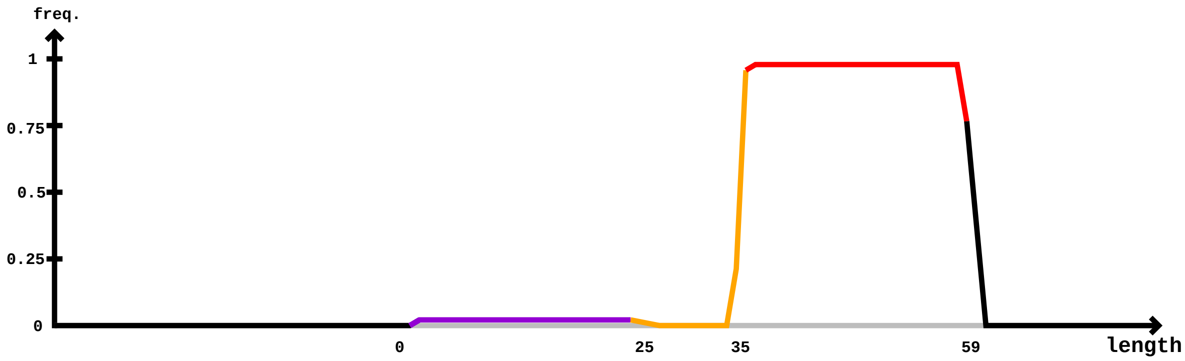

Star **Mature**

```
5'- uuuauuauaugguauauaucaguauauaugaauuuauguuuucgauauuauaaugugacauugucuguuaauggcauaagauagaagaacguguuaagauauauuuaguuuug   -3'    obs
    uuuauuauaugguauauaucaguauauaugaauuuauguuuucgauauuauaaugugacauugucuguuaauggcauaagauagaagaacguguuaagauauauuuaguuuug          exp
    .....(((((.(((((((....))))))).....((((((((...((((....((((.(((((.....))))).)))).))))..)))))))).....))))).........    reads     mm      sample
    .....................................................................aauggcauaagauagaagaacgug....................    2         0       S03
    ......................................................................auggcauaagauagaagaacgugA...................    1         1       S03
    ......................................................................auggcauaagauagaagaacgugu...................    7         0       S03

    ....................................uguuuucgauauuauaaugugacC.....................................................    1         1       S04
    .....................................................................aauggcauaagauagaagaacguA....................    4         1       S04
    .....................................................................aauggcauaagauagaagaacgug....................    4         0       S04
    ......................................................................auggcauaagauagGagaacgugu...................    1         1       S04
    ......................................................................auggcauaagauagaagaacgugG...................    2         1       S04
    ......................................................................auggcauaagauagaagaacgAgu...................    1         1       S04
    ......................................................................auggcauaagauagaagaacgugC...................    5         1       S04
    ......................................................................auggcauaagauagaagaacgugu...................    18        0       S04
    .......................................................................uggcauaagauagaagaacguguC..................    1         1       S04
```

Provisional ID	:	chrC04_1571022
Score total	:	0
Score for star read(s)	:	-1.3
Score for read counts	:	-0.3
Score for mfe	:	1.7
Score for randfold	:	0
Score for cons. seed	:	
Total read count	:	11
Mature read count	:	11
Loop read count	:	0
Star read count	:	0

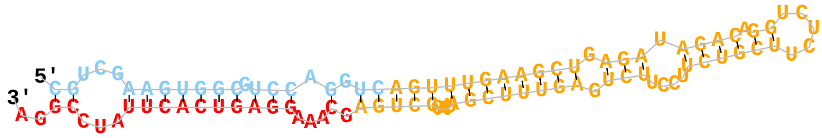

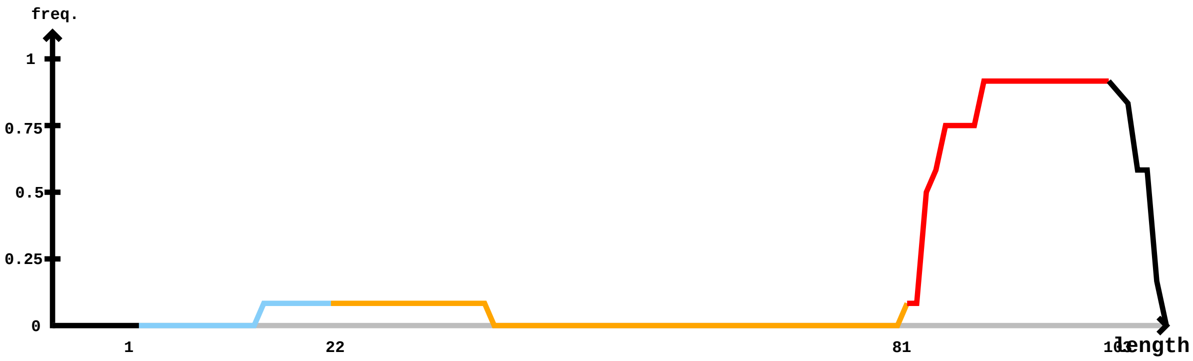

Star Mature

```
5'- ucaaagccgucgaaguggcguccaggucaguuugaagcugagauagacaggucucuucgucuuccuucugaguuucgaucaggcugagcaaaggagucacuuauccggagccca   -3'    exp
    ......(((...(((((((.(((.(.(((((((((((((.(((.((((.((.....))))))....))).))))))))....))))).)...))))))))))...)))......   reads   mm   sample
    .........................................................................................aaaggagucacuuauccggagccU.   1       1    S03
    ...............................................................................................gucacuuauccggagcccC   1       1    S03

    ....................uccaggucaguuugaagcugagau......................................................................   1       0    S04
    .......................................................................................gcaaaggagucacuuauccgga.....   1       0    S04
    .........................................................................................aaaggagucacuuauccggagc...   1       0    S04
    .........................................................................................Naaggagucacuuauccggagc...   1       1    S04
    .........................................................................................aaaggagucacuuauccggagU...   1       1    S04
    .........................................................................................aaaggagucacuuauccggagccc.   1       0    S04
    ..........................................................................................aaggagucacuuauccggagccc.   1       0    S04
    ...........................................................................................aggagucacuuauccggagccU.   1       1    S04
    ...........................................................................................aggagucacuuauccggagccc.   1       0    S04
    ...............................................................................................gucacuuauccggagccca   1       0    S04
```

Provisional ID	:	chrC04_1580937
Score total	:	0.1
Score for star read(s)	:	-1.3
Score for read counts	:	0
Score for mfe	:	1.4
Score for randfold	:	0
Score for cons. seed	:	
Total read count	:	12
Mature read count	:	12
Loop read count	:	0
Star read count	:	0

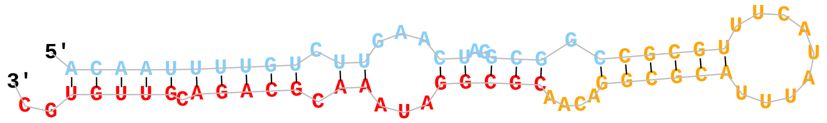

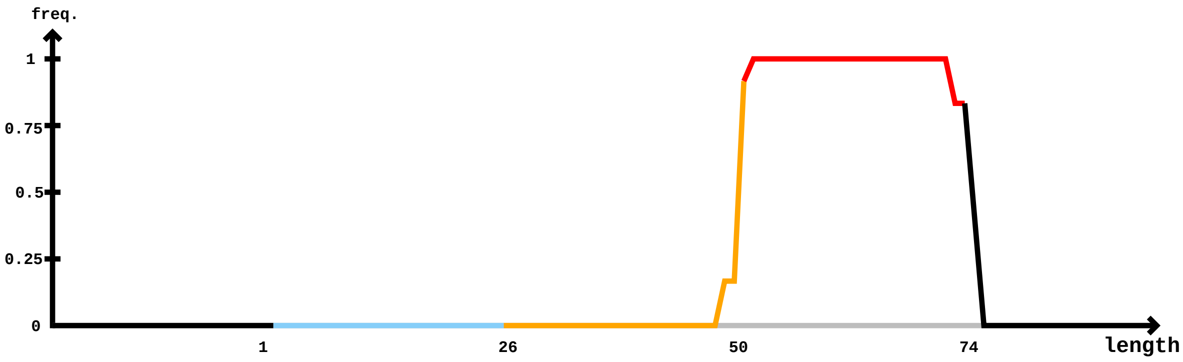


```
                              Star                                Mature
5'- gaacagagaacgguuagccccacaauuuugucuugaacuaggcggccgcguuucauauuuacgcggacaacgcggauaaacgcagacguugugcgguccaccgauuugaauca   -3'    exp
    ...((((...((((..(((.(((((((((((.((...((..(((.((((((.........))))))....)))))...)).))))).)))))).)))..)))).)))).....   reads      mm         sample
    ....................................................................aacgcggauaaacgcagacguugu.....................   1          0          S03
    ......................................................................cgcggauaaacgcagacguugugc...................   1          0          S03

    ....................................................................aacgcggauaaacgcagacguugu.....................   1          0          S04
    ......................................................................cgcggauaaacgcagacguugugU...................   3          1          S04
    ......................................................................cgcggauaaacgcagacguugugA...................   1          1          S04
    ......................................................................cgcggauaaacgcagacguugugc...................   4          0          S04
    .......................................................................gcggauaaacgcagacguugugcg..................   1          0          S04
```

Provisional ID : chrC04_1587116
Score total : 0.5
Score for star read(s) : -1.3
Score for read counts : 0
Score for mfe : 1.8
Score for randfold : 0
Score for cons. seed :
Total read count : 130
Mature read count : 126
Loop read count : 4
Star read count : 0

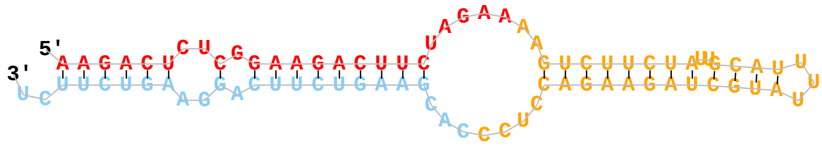

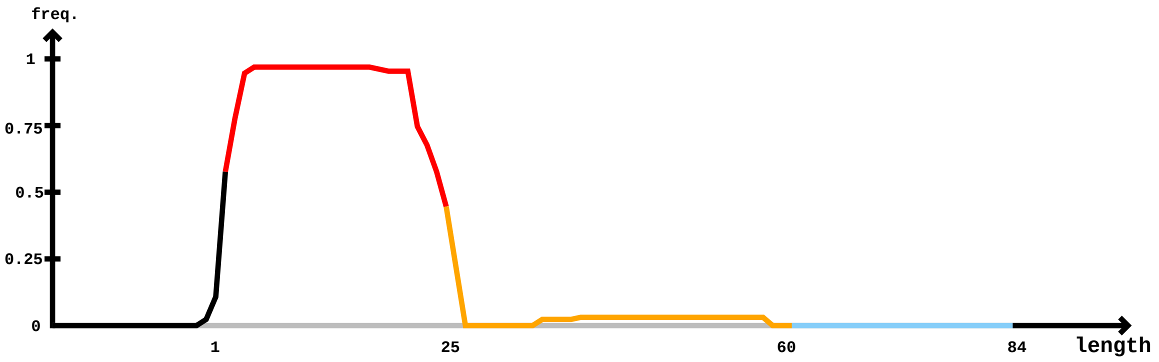

Mature Star

```
5'- uugaaagacuucuuagaagacucucggaagacuucuagaaaagucuucuauugcauuuuaugcuagaagaccucccacgaagucuucaggaagucuucuagucccaaagu   -3'   exp
    ......((((....((((((((..(.(((((((((.......((((((((..((((...)))))))))))).......))))))))).)..))))))))))))).......    reads   mm   sample
    ..............agaagacucucggaaUacuucuag........................................................................    1       1    S03
    ...............gaagacucucggaaUacuucuag........................................................................    1       1    S03
    ...............gaagacucuUggaagacuucuag........................................................................    1       1    S03
    ...............gaagacucucggaagacuucuag........................................................................    1       0    S03
    ...............gaagacucucggaaUacuucuaga.......................................................................    5       1    S03
    ................aagacucucggaagacuucu..........................................................................    2       0    S03
    ................aagacucucggaaUacuucua.........................................................................    1       1    S03
    ................aagacucucggaaUacuucuag........................................................................    4       1    S03
    ................aagacucucggaagacuucuaga.......................................................................    1       0    S03
    ................aagacucucggaagacuucuUgaa......................................................................    26      1    S03
    .................agacucucggaagacuucu..........................................................................    3       0    S03
    .................agacucucggaaUacuucuag........................................................................    3       1    S03
    .................agacucucggaaUacuucuaga.......................................................................    7       1    S03
    .................agacucucggaagacuucuUgaa......................................................................    6       1    S03
    ..................gacucucggaagacuucu..........................................................................    8       0    S03
    ..................gacucucggaagacuucuG.........................................................................    1       1    S03
    ..................gacucucggaaUacuucuaga.......................................................................    1       1    S03
    ...................acucucggaagacuucuUgaa......................................................................    1       1    S03
    .................................................aAugcauuuuaugcuagaagaccu.....................................    2       1    S03

    ..............agaagacucucggaagac..............................................................................    1       0    S04
    ..............agaagacucucggaagacu.............................................................................    1       0    S04
    ...............gaagacucucggaaUacuucua.........................................................................    1       1    S04
    ...............gaagacucucggaaUacuucuag........................................................................    1       1    S04
    ...............gaagacucucggaaUacuucuaga.......................................................................    1       1    S04
    ................aagacucucggaagacuucu..........................................................................    4       0    S04
    ................aagacucucggaagacuucua.........................................................................    2       0    S04
    ................aagacucucggaagacuucuUgaa......................................................................    20      1    S04
    ................aagacucucggaagacuucuagaG......................................................................    1       1    S04
    .................agacucucggaagacuucua.........................................................................    1       0    S04
    .................agacucucggaagacuucuag........................................................................    1       0    S04
    .................agacucucgUaagacuucuaga.......................................................................    1       1    S04
    .................agacucucggaagacuucuaga.......................................................................    1       0    S04
    .................agacucucggaagacuucuUgaa......................................................................    2       1    S04
```

Mature Star

```
uugaaagacuucuuagaagacucucggaagacuucuagaaaagucuucuauugcauuuuaugcuagaagaccucccacgaagucuucaggaagucuucuagucccaaagu
.................agacucucggaagacuucuUgaaa.....................................................................	1	1	S04
..................gacucucggaagacuucu..........................................................................	10	0	S04
..................gacucucggaagacuucua.........................................................................	1	0	S04
..................gacucucggaagacuucuUgaa......................................................................	1	1	S04
...................acucucggaagacuucua.........................................................................	2	0	S04
.................................................aAugcauuuuaugcuagaagaccu.....................................	1	1	S04
.....................................................cauuuuaugcuagaagaccu.....................................	1	0	S04
```

Provisional ID	:	chrC04_1599232
Score total	:	1.6
Score for star read(s)	:	-1.3
Score for read counts	:	0
Score for mfe	:	2.9
Score for randfold	:	0
Score for cons. seed	:	
Total read count	:	27
Mature read count	:	27
Loop read count	:	0
Star read count	:	0

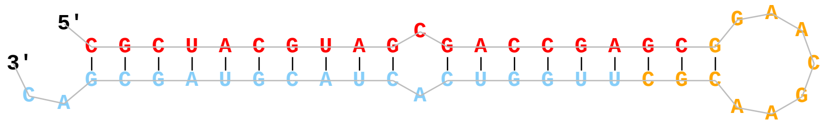

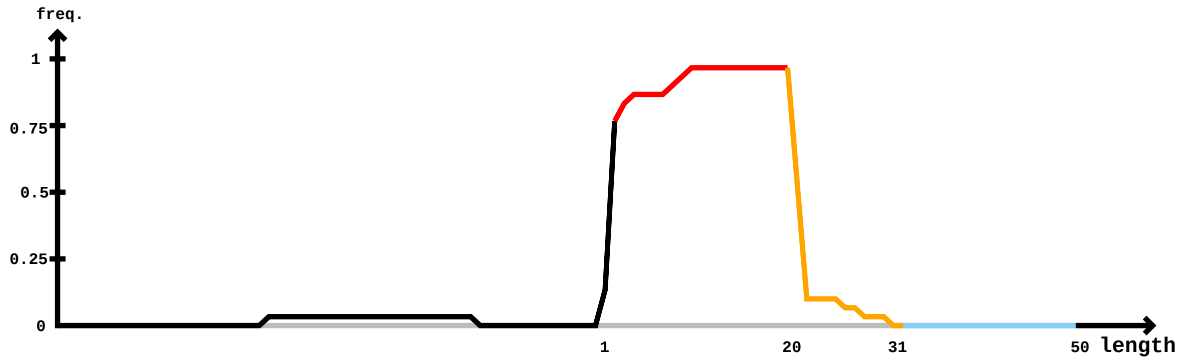

Mature Star

```
5'- agcuucggcucgagcuugguugcugcguagcgaccgagcuucggcuugagcuuggucgcuacguagcgaccgagcggaacgaacgcuuggucacuacguagcgaccgugcau   -3'    exp
    (((((.((((.(((((((((((((....))))))))))))).)))).))))).(((((((((((((.(((((((((.......))))))))).)))))))))))))......     reads      mm         sample
    ....................ugcugcguagcgaccgagcuuc......................................................................     1          0          S03
    .......................................................ucgcuacguagcgaccgagc.....................................     3          0          S03
    ........................................................cgcuacguagcgaccgagc.....................................     17         0          S03
    .........................................................gcuacguagcgaccgagc.....................................     1          0          S03
    .........................................................gcuacUuagcgaccgagcg....................................     1          1          S03
    ..........................................................cuacguagcgaccgagcg....................................     1          0          S03
    ..............................................................uagcgaUcgagcggaacga...............................     1          1          S03
    ...............................................................agcgaccgagcggaacgaacA............................     1          1          S03

    .......................................................ucgcuacguagcgaccgagc.....................................     1          0          S04
    ........................................................cgcuacguagcgaccgagc.....................................     1          0          S04
    ........................................................cgcuacguagcgaccgagcg....................................     1          0          S04
    .............................................................guagcgaccgagcggaac.................................     1          0          S04
```

Provisional ID	:	chrC04_1602818
Score total	:	0.8
Score for star read(s)	:	-1.3
Score for read counts	:	0
Score for mfe	:	2.1
Score for randfold	:	0
Score for cons. seed	:	
Total read count	:	27
Mature read count	:	25
Loop read count	:	1
Star read count	:	1

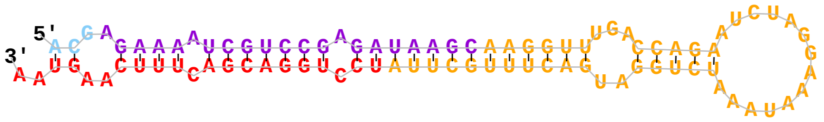

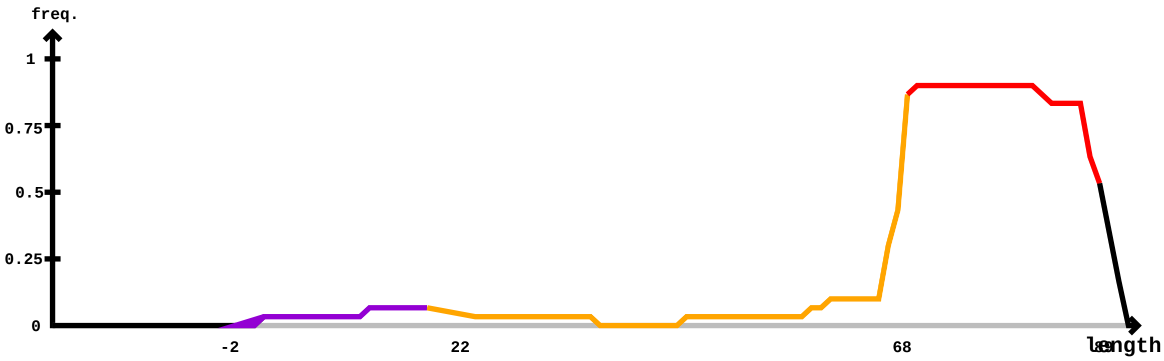

Star   Mature

```
5'- guccagguagacgacuuacgagaaaaucguccgagauaagcaagguuugaccagaaucuaggaaauaaaucuggaugacuuugcuuauccuggacgacuuucaaguaaguc  -3'  obs
    guccagguagacgacuuacgagaaaaucguccgagauaagcaagguuugaccagaaucuaggaaauaaaucuggaugacuuugcuuauccuggacgacuuucaaguaaguc       exp
    ...........(((((((..((((.(((((((.(((((((((((((...(((((...............)))))..))))))))))))).))))))).))))..)))))))  reads  mm  sample
    ....................agaaaaucguccgagauaagc......................................................................  1      0   S03
    .............................................................................acuuugcuuauccuggacgacuAu..........  1      1   S03
    .....................................................................................uauccuggacgacuuucaagA.....  1      1   S03
    .....................................................................................uauccuggacgacuuucaagG.....  1      1   S03
    .....................................................................................uauccuggacgacuuucaagu.....  3      0   S03
    ......................................................................................auccuggacgacuuucaagua....  1      0   S03
    ......................................................................................aucAuggacgacuuucaaguaagu.  1      1   S03
    ......................................................................................auccuggacgacuuucaaguaagu.  1      0   S03
    .......................................................................................uccuggacgacuuucaagua....  1      0   S03
    .......................................................................................uccuggacgacuuucaaguaa...  6      0   S03
    .......................................................................................uccuggacgacuuucaaguaUg..  1      1   S03
    .......................................................................................uccuggacgacuuucaaguaaU..  1      1   S03
    .......................................................................................uccuggacgacuuucaaguaag..  2      0   S03
    .......................................................................................uccuggacgacuuucaaguaagA.  1      1   S03
    ........................................................................................ccuggacgacuuucaaguaagu.  1      0   S03

    ...............................cgGgauaagcaagguuugaccaga........................................................  1      1   S04
    ................................................................auaaaucuggaugacuuugcuuau.......................  1      0   S04
    ...............................................................................uuugcuuauccuggacgacuuuU.........  1      1   S04
    .....................................................................................uauccuggacgacuuucaagu.....  1      0   S04
    ......................................................................................auccuggacgacuuucaagua....  1      0   S04
    .......................................................................................uccuggacgacuuucaaguaa...  1      0   S04
    ........................................................................................ccuggacgacuuucaaCuaagu.  1      1   S04
```

Provisional ID	:	chrC04_1606591
Score total	:	33.5
Score for star read(s)	:	3.9
Score for read counts	:	28.2
Score for mfe	:	1.4
Score for randfold	:	0
Score for cons. seed	:	
Total read count	:	67
Mature read count	:	66
Loop read count	:	0
Star read count	:	1

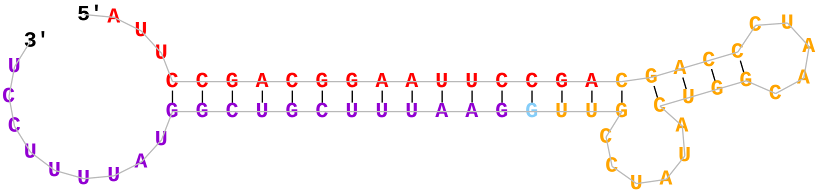

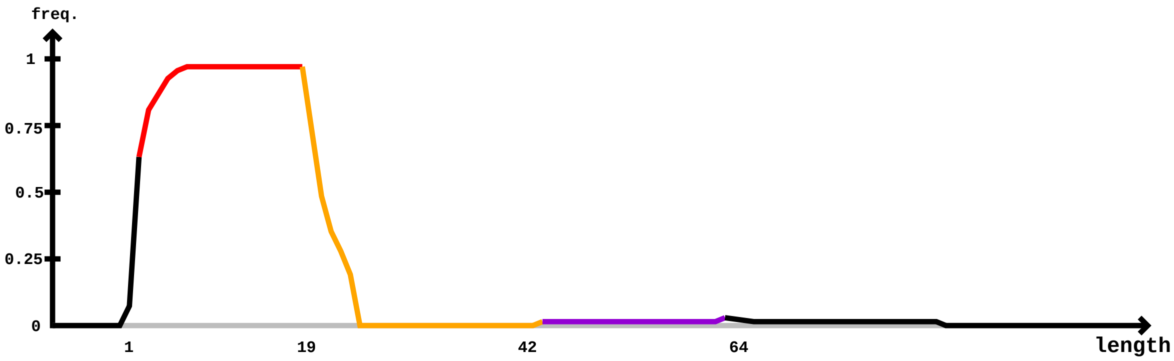

Mature Star

```
5'- cgauggcauuccgacggaauuccgacgacccuaacggucauauccguuggaauuucgucgguauuuuccuaucucaacggcuauaaaacgaucauauguauuugucggcaau   -3'   obs
    cgauggcauuccgacggaauuccgacgacccuaacggucauauccguuggaauuucgucgguauuuuccuaucucaacggcuauaaaacgaucauauguauuugucggcaau         exp
    .(((((....((((((((((((((((((((.....))))......))))))))))))))))).......)))))......(((((((((((......))).))))).)))...   reads   mm   sample
    ......cauuccgacggaauuccgac......................................................................................   1       0    S03
    ......cauuccgacggaauuccgGc......................................................................................   1       1    S03
    ......cauuccgacggaauuccgacgU....................................................................................   1       1    S03
    .......auuccgacggaauuccga.......................................................................................   8       0    S03
    .......auuccgacggaauuccgac......................................................................................   6       0    S03
    .......auuccgacggaauuccgacg.....................................................................................   1       0    S03
    .......auuccgacggaauuccgacUa....................................................................................   1       1    S03
    .......auuccgacgCaauuccgacgac...................................................................................   1       1    S03
    .......auuccgacggaauuccgacgCcc..................................................................................   2       1    S03
    ........uuccgacggaauuccgac......................................................................................   3       0    S03
    ........uuccgacggaauuccgacg.....................................................................................   1       0    S03
    ........uuccgacggaauuccgacgacc..................................................................................   2       0    S03
    .........uccgacggaauuccgacg.....................................................................................   1       0    S03
    .........uccgacggaauuccgaAgac...................................................................................   1       1    S03
    .........ucUgacggaauuccgacgacc..................................................................................   1       1    S03
    ..........ccgacggaauuccgacgacc..................................................................................   1       0    S03

    ......cauuccgacggaauuccgac......................................................................................   1       0    S04
    ......cauuccgacggaauuccgacgU....................................................................................   1       1    S04
    .......auuccgacggaauuccga.......................................................................................   2       0    S04
    .......auuccgacggaauuccgac......................................................................................   8       0    S04
    .......auuccgacggaauuccgacC.....................................................................................   4       1    S04
    .......auuccgacggaauuccgacga....................................................................................   1       0    S04
    .......auuccgacggaauuccgaUgac...................................................................................   2       1    S04
    .......auuccgacggaauuccgacgCcc..................................................................................   2       1    S04
    ........uuccgacggaauuccgac......................................................................................   3       0    S04
    ........uuccgacggaauuccgacg.....................................................................................   1       0    S04
    ........uuccgacggaauuccgacgaU...................................................................................   1       1    S04
    ........uuccgacggaauuccgacgacG..................................................................................   1       1    S04
    .........uccgacggaauuccgacg.....................................................................................   1       0    S04
    ..........ccgacggaauuccgacga....................................................................................   1       0    S04
    ..........ccgacggaauuccgacgacc..................................................................................   2       0    S04
    ...........Agacggaauuccgacgac...................................................................................   1       1    S04
```

```
      Mature                                  Star
cgauggcauuccgacggaauuccgacgacccuaacggucauauccguuggaauuucgucgguauuuuccuaucucaacggcuauaaaacgaucauauguauuugucggcaau
...........cgacggaauuccgacgacc..................................................................................   1   0   S04
............gaUggaauuccgacgacc..................................................................................   1   1   S04
.................................................gaauuucgucgguauAuuccu..........................................   1   1   S04
....................................................................cuaCcucaacggcuauaaaacga.....................   1   1   S04
```

Provisional ID : chrC04_1627871
Score total : 0.9
Score for star read(s) : -1.3
Score for read counts : 0
Score for mfe : 2.2
Score for randfold : 0
Score for cons. seed :
Total read count : 31
Mature read count : 25
Loop read count : 6
Star read count : 0

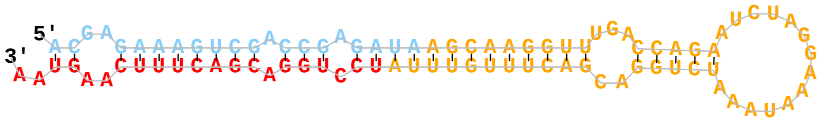

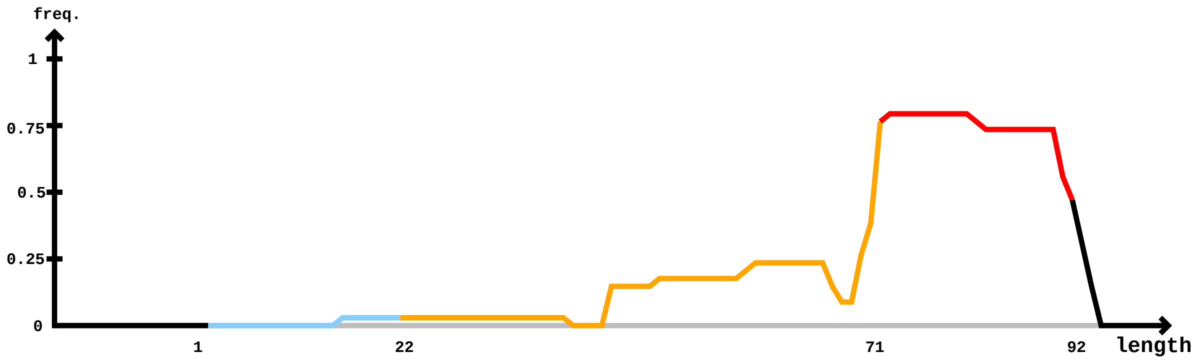

Star Mature

```
5'- cagguagacgacuuacgagaaagucgaccgagauaagcaagguuugaccagaaucuaggaaauaaaucuggacgacuuuguuuauccuggacgacuuucaaguaagucggacga   -3'    exp
    ...((...((((((((..((((((((.(((.(((((((((((((...(((((..............)))))..))))))))))))).))).))))))))..)))))))).))..   reads   mm   sample
    ........................................................aggaaauaaaucuggacgacuuuA..................................   1   1   S03
    ..................................................................................uauccuggacgacuuucaagu...........   3   0   S03
    ..................................................................................uauccuggacgacuuucaagA...........   1   1   S03
    ..................................................................................uauccuggacgacuuucaagG...........   1   1   S03
    ...................................................................................auccuggacgacuuucaagua..........   1   0   S03
    ...................................................................................aucAuggacgacuuucaaguaagu.......   1   1   S03
    ...................................................................................auccuggacgacuuucaaguaagu.......   1   0   S03
    ....................................................................................uccuggacgacuuucaagua..........   1   0   S03
    ....................................................................................uccuggacgacuuucaaguaa.........   6   0   S03
    ....................................................................................uccuggacgacuuucaaguaaU........   1   1   S03
    ....................................................................................uccuggacgacuuucaaguaUg........   1   1   S03
    ....................................................................................uccuggacgacuuucaaguaag........   2   0   S03
    ....................................................................................uccuggacgacuuucaaguaagA.......   1   1   S03
    .....................................................................................ccuggacgacuuucaaguaagu.......   1   0   S03

    ............................cgGgauaagcaagguuugaccaga..............................................................   1   1   S04
    ........................................................aggaaauaaaucuggGcgacuuu...................................   1   1   S04
    ........................................................aggaaauaaaucuggacgacuuG...................................   1   1   S04
    ........................................................aggaaauaaaucuggacgacuuu...................................   1   0   S04
    ........................................................aggaaauaaaucuggacgacuuug..................................   1   0   S04
    .............................................................auaaaucuggacgacuuuguuuau.............................   1   0   S04
    ......................................................................gacgacuuuguuuauccuggacga....................   1   0   S04
    .......................................................................acgacuuuguuuauccuggacgaU...................   1   1   S04
    ..................................................................................uauccuggacgacuuucaagu...........   1   0   S04
    ...................................................................................auccuggacgacuuucaagua..........   1   0   S04
    ....................................................................................uccuggacgacuuucaaguaa.........   1   0   S04
    .....................................................................................ccuggacgacuuucaaCuaagu.......   1   1   S04
```

Provisional ID	:	chrC04_1645394
Score total	:	2.1
Score for star read(s)	:	3.9
Score for read counts	:	-1.8
Score for mfe	:	0.1
Score for randfold	:	0
Score for cons. seed	:	
Total read count	:	8
Mature read count	:	7
Loop read count	:	0
Star read count	:	1

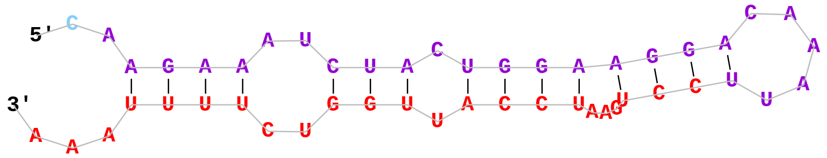

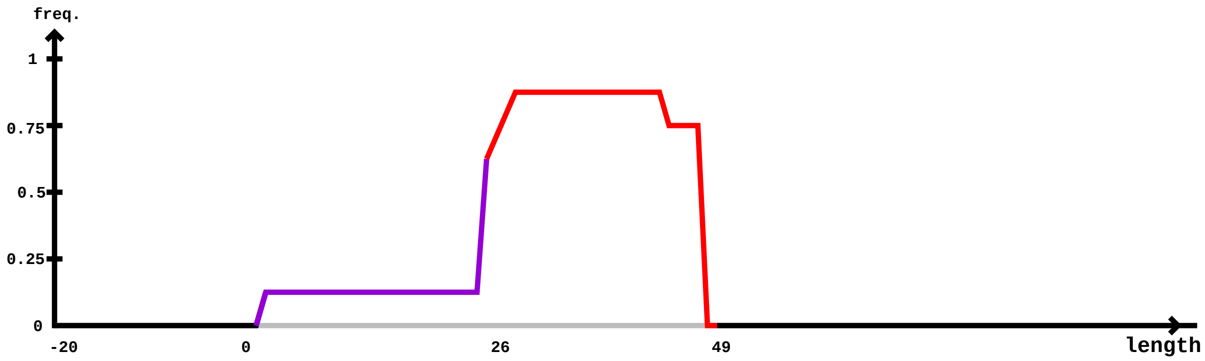

Star Mature

```
5'- uauugugcuauugacuuuucaagaaaucuacuggaaggacaaauuccugaauccauuggucuuuuaaagucgcugauugcacucaaucuaucgagcaacgccuucacaggccaua   -3'   obs
    uauugugcuauugacuuuucaagaaaucuacuggaaggacaaauuccugaauccauuggucuuuuaaagucgcugauugcacucaaucuaucgagcaacgccuucacaggccaua         exp
    .....((((...((((((..((((...(((.((((((((.....))))...)))).)))))))..))))))...(((((....))))).....))))..((((....))))....   reads   mm   sample
    ...........................................uuccugaauccauuggucuCuua.................................................   1       1    S03

    ....................aagaaaucuacuggaaggacaaauu......................................................................   1       0    S04
    ...........................................uuccugaauccauuggucuCuua.................................................   3       1    S04
    ............................................uccugaauccauuggucC.....................................................   1       1    S04
    ............................................uccugaauccauuggucuCuua.................................................   1       1    S04
    .............................................ccugaauccauuggucuCuua.................................................   1       1    S04
```

Provisional ID	:	chrC04_1645854
Score total	:	6
Score for star read(s)	:	3.9
Score for read counts	:	3.2
Score for mfe	:	-1
Score for randfold	:	0
Score for cons. seed	:	
Total read count	:	18
Mature read count	:	15
Loop read count	:	1
Star read count	:	2

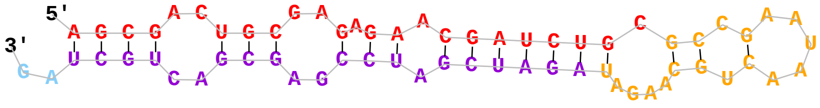

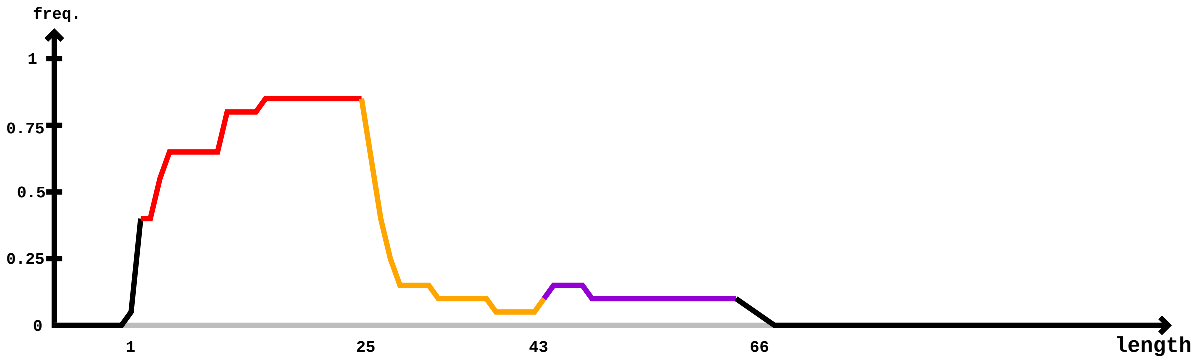

Mature **Star**

```
5'- ugaucugagcgacugcgagagaacgaucugcgccgaauaacugcaagauagaucgauccgagcgacugcuagagagcgaucaacgccgaguaacugcgagagagcgagacgaau   -3'   obs
    ugaucugagcgacugcgagagaacgaucugcgccgaauaacugcaagauagaucgauccgagcgacugcuagagagcgaucaacgccgaguaacugcgagagagcgagacgaau         exp
    ...(((.((((..(((..(.((.(((((((.((.(.....).))....))))))).)))..)))..)))))))..((..((..(((.(.....).))).))..)).........   reads   mm   sample
    .......agcgacugcgUgagaacgaucugc...................................................................................   1       1    S03
    .......agcgacugcgagagaacgaucugU...................................................................................   1       1    S03
    .........cgacugcgagagaacgaucugcgc.................................................................................   1       0    S03
    ..........gacugcgagagaacgaucugcg..................................................................................   1       0    S03
    ..............................cgccgaauaacugcaagauagauc............................................................   1       0    S03
    ..................................................gaucgauccgagcgacugcAa...........................................   1       1    S03

    ......gagcgacugcgagagaacgaucug....................................................................................   1       0    S04
    .......agcgacugcgagagaacgaucugc...................................................................................   2       0    S04
    .......agcgacugcgagagaacgaucugG...................................................................................   1       1    S04
    .......agcgacugcgagagaacgauAugc...................................................................................   1       1    S04
    .......agcgacugcgagagaacgaucugU...................................................................................   1       1    S04
    .........cgacugcgagagaacgaucugcgA.................................................................................   1       1    S04
    .........cgacugcgagagaacgaucugcgc.................................................................................   1       0    S04
    ..........gacugcgagagaacgaucugc...................................................................................   1       0    S04
    ................gagagaacgaucugcgcc................................................................................   2       0    S04
    ................gagagaaUgaucugcgccgaau............................................................................   1       1    S04
    ....................gaacgaucugcgccgaauaacugc......................................................................   1       0    S04
    .................................................agaucgauccgagcgacugcA............................................   1       1    S04
```

Provisional ID	:	chrC04_1651288
Score total	:	8.3
Score for star read(s)	:	3.9
Score for read counts	:	3.2
Score for mfe	:	1.2
Score for randfold	:	0
Score for cons. seed	:	
Total read count	:	18
Mature read count	:	10
Loop read count	:	0
Star read count	:	8

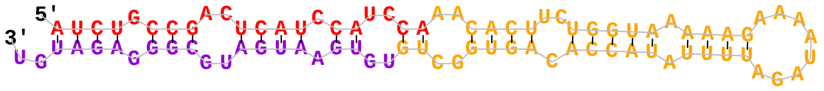

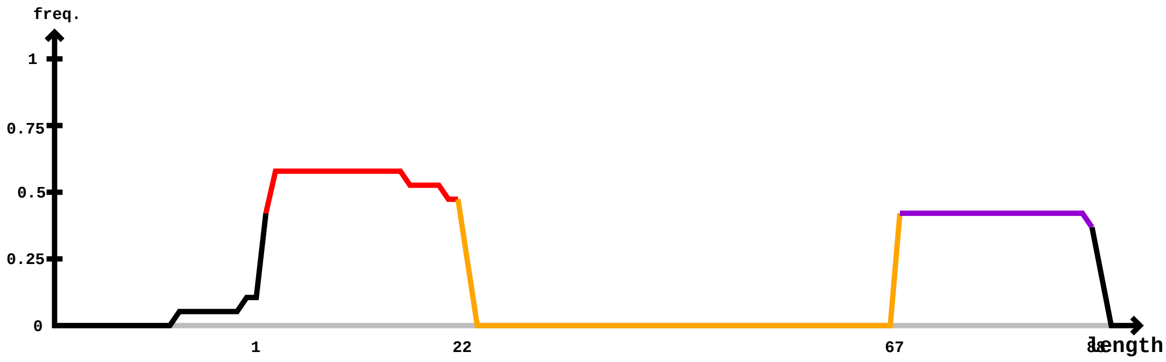

Mature **Star**

```
5'- aacaagaagaggguagaaacaucugccgacucauccauccaaacacuucugguaaaaagaaaauagauuuuauaccacaguggcugugugaaugaugcgggagauguuuuc   -3'    obs
    aacaagaagaggguagaaacaucugccgacucauccauccaaacacuucugguaaaaagaaaauagauuuuauaccacaguggcugugugaaugaugcgggagauguuuuc          exp
    ...............(((((((((.(((..((((.((..((..((((..(((((.((((........)))).))))).))))..))..)).))))..))).))))))))).    reads      mm       sample
    ..................acaucugccgacucauccauc........................................................................    1          0        S03
    ....................aucugccgacucauccauccG......................................................................    1          1        S03
    ....................aucugccgacucauccaucca......................................................................    1          0        S03
    ....................aucugccgacucauccauccC......................................................................    1          1        S03
    ......................................................................................ugugaaugaugcgggagaugu....    2          0        S03

    ...........gguagaaacaucugcAgacucauc............................................................................    1          1        S04
    ....................aucugccgacucauccaucca......................................................................    3          0        S04
    .....................ucugccgacucauccaucca......................................................................    1          0        S04
    .....................ucugccgacucauccauccaC.....................................................................    1          1        S04
    .....................ucugccgacucauccauccaU.....................................................................    1          1        S04
    ......................................................................................ugugaaugaugcgggagauA.....    1          1        S04
    ......................................................................................ugugaaugaugcgggagaugC....    1          1        S04
    ......................................................................................ugugaaugaugcgggagaugu....    2          0        S04
    ......................................................................................ugCgaaugaugcgggagaugu....    1          1        S04
    ......................................................................................ugugaaugaugcgggagauguu...    1          0        S04
```

Provisional ID : chrC04_1655638
Score total : 189.1
Score for star read(s) : 3.9
Score for read counts : 192.3
Score for mfe : -7
Score for randfold : 0
Score for cons. seed :
Total read count : 389
Mature read count : 382
Loop read count : 0
Star read count : 7

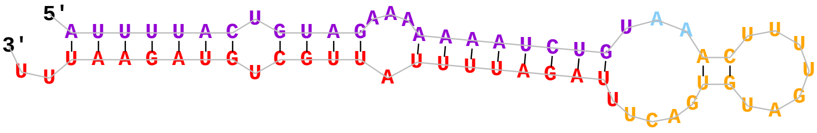

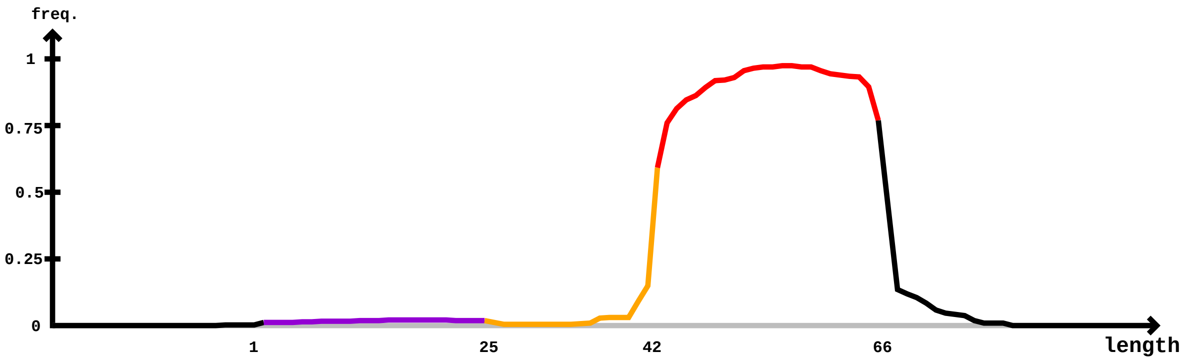


```
                        Star                    Mature
5'- uaauggcuguaguuuuaaaaauuuuacuguagaaaaaaaucuguaaacuuuugaugugacuuuagauuuuauugcuguagaauuuuauggaaagcacuaaaaaaauugcuuugg  -3'  obs
    uaauggcuguaguuuuaaaaauuuuacuguagaaaaaaaucuguaaacuuuugaugugacuuuagauuuuauugcuguagaauuuuauggaaagcacuaaaaaaauugcuuugg        exp
    ...(((.(((..(((((((((((((((.((((...((((((((...((.......)).....)))))))).)))).)))))))))).))))).))))))...............  reads  mm  sample
    ....................auuuuacuguagaaaaaaaucugG......................................................................  1      1   S03
    ....................auuuuacuguagaaaaaaaucugu......................................................................  1      0   S03
    ..........................cuguagaaaaCaaucugu......................................................................  1      1   S03
    ..............................agaaaaaaaucuguaaacuuuu..............................................................  1      0   S03
    .................................aaaaaaucuguaaacuuu...............................................................  1      0   S03
    ......................................................ugugacuuuagauuuuauugcugu....................................  1      0   S03
    .......................................................gugacuuuagauuuuauugcugu....................................  3      0   S03
    .......................................................gugUcuuuagauuuuauugcugua...................................  1      1   S03
    .......................................................gugacuuuagauuuuauugcugua...................................  1      0   S03
    ........................................................ugacuuuagauuuuauugcu......................................  1      0   S03
    ............................................................cuuuagauuuuauugcugu...................................  1      0   S03
    ............................................................cuuuagauuuuauugcugua..................................  1      0   S03
    ............................................................cuuuagauuuuauugcuguag.................................  1      0   S03
    ............................................................Nuuuagauuuuauugcuguag.................................  1      1   S03
    ............................................................cuuuagauuuuauugcuguaga................................  1      0   S03
    ............................................................cuuuagauuuuauugcuguagaau..............................  9      0   S03
    ............................................................cuuuagauuuuauugcuguagaaC..............................  2      1   S03
    ............................................................cuuuagauuuuauugcuguagaauu.............................  5      0   S03
    ............................................................cuuuagauuuuauugcuguagaauA.............................  1      1   S03
    ............................................................cuuuagauuuuauugcuguagaauuu............................  1      0   S03
    ............................................................cuuuGgauuuuauugcuguagaauuu............................  1      1   S03
    .............................................................uuuagauuuuauugcuguaga................................  1      0   S03
    .............................................................uuuagauuuuauugcuguagaauu.............................  16     0   S03
    .............................................................uuuagauuuuauugcuguagaauuu............................  3      0   S03
    ..............................................................uuagauuuuauugcuguagaau..............................  1      0   S03
    ..............................................................uuagauuuuauugcuguagaaC..............................  1      1   S03
    ..............................................................uuagauuuuauugcuguagaauA.............................  1      1   S03
    ..............................................................uuagauuuuauugcuguagaauu.............................  5      0   S03
    ..............................................................uuagauuuuauugcuguagaauC.............................  1      1   S03
    ..............................................................uuagauuuuauugcuguagaauuA............................  1      1   S03
    ..............................................................uuagauuuCauugcuguagaauuu............................  1      1   S03
    ..............................................................uuagGuuuuauugcuguagaauuu............................  2      1   S03
    ..............................................................uuagauuuuauugcuguagaauuu............................  132    0   S03
```

```
                    Star                                        Mature
uaauggcuguaguuuuaaaaauuuuacuguagaaaaaaaucuguaaacuuuugaugugacuuuagauuuuauugcuguagaauuuuauggaaagcacuaaaaaauugcuuugg
.............................................................uuagauuuuauugcugGagaauuu............................    1    1    S03
.............................................................uuagauuuuauugcuguagaauuG............................    1    1    S03
.............................................................uuagauuuuauugcuguagaauuC............................    11   1    S03
.............................................................Guagauuuuauugcuguagaauuu............................    1    1    S03
..............................................................uagauuuuauugcuguagaauuG............................    3    1    S03
..............................................................uagauuuuGuugcuguagaauuu............................    1    1    S03
..............................................................uagauuuuauugcuguagGauuu............................    1    1    S03
..............................................................uagauuuuauugcuguagaauuu............................    51   0    S03
..............................................................uagauuuuauugcuguagaauuC............................    2    1    S03
..............................................................uagauuuuauugcuguagaauuuu...........................    1    0    S03
...............................................................agauuuuauugcuguagaau..............................    1    0    S03
...............................................................agauuuuauugcuguagaauu.............................    8    0    S03
...............................................................agauuuuauugcuguagaGuuu............................    1    1    S03
...............................................................agauuuuauugcuguagaauuu............................    8    0    S03
...............................................................agauuuuauugcuguagaauuuu...........................    1    0    S03
...............................................................agauuuuauugcuguagaauuuua..........................    2    0    S03
................................................................gauuuuauugcuguagaa...............................    1    0    S03
................................................................gauuuuauugcuguagaaC..............................    1    1    S03
................................................................gauuuuauCgcuguagaauu.............................    1    1    S03
................................................................gauuuuauugcuguagaauu.............................    6    0    S03
................................................................gauuuuauugcuguagaauuuuau.........................    3    0    S03
.................................................................auuuuauugcuguagaau..............................    1    0    S03
.................................................................auuuuauugcuguagaauu.............................    2    0    S03
.................................................................auuuuUuugcuguagaauuuua..........................    1    1    S03
.................................................................auuuuauugcuguagaauuuuaug........................    2    0    S03
..................................................................uuuuauugcuguagaauuuu...........................    1    0    S03
..................................................................uuuuauugcuguagaauuuuaug........................    1    0    S03
..................................................................uuuuauugcuguagaauuuuaugg.......................    3    0    S03
..................................................................uuuuauugcuguagaauuuuaugga......................    1    0    S03
...................................................................uuuauugcuguagaauuu............................    1    0    S03
...................................................................uuuauugcuguagaauuuua..........................    1    0    S03
...................................................................uuuauugcuguagaauuuuau.........................    1    0    S03
...................................................................uuuauugcuguagaauuuuauC........................    1    1    S03
...................................................................uuuauugcuguagaauuuuaugg.......................    2    0    S03
...................................................................uuuauugcuguagaauuuuaugga......................    1    0    S03
...................................................................uuuauugcuguagaauuuuauCga......................    1    1    S03
....................................................................uuauugcuguagaauuuu...........................    1    0    S03
.....................................................................uauugcuguagaauuuuaug........................    1    0    S03
.....................................................................uauugcuguagaauuuuauggaaa....................    1    0    S03
......................................................................auugcuguagaauuuuau.........................    1    0    S03
......................................................................auugcuguagaauuuuaugga......................    1    0    S03
......................................................................auugcuguagaauuuuauggaaag...................    1    0    S03
.......................................................................uugcuguagaauuuuauggaaagc..................    1    0    S03
...........................................................................uguagaauuuuaCggaaagcacu...............    1    1    S03

................aaaaauuuuacuguagaaaaaaau.........................................................................    1    0    S04
....................auuuuacuguagaaaaaaaucugG.....................................................................    1    1    S04
....................auuuuacuguagaaaaaaaucugu.....................................................................    1    0    S04
........................uacuguagaaaaaaaucugu.....................................................................    1    0    S04
...................................................ugaugugacuuuagauuuuauugc......................................    1    0    S04
....................................................gaugugacuuuagauuuuauugcu.....................................    1    0    S04
.....................................................augugacuuuagauuuuauugc......................................    1    0    S04
.......................................................gugacuuuagauuuuauugcugu...................................    1    0    S04
.......................................................gugacuuuagauuuuauugcugua..................................    2    0    S04
...........................................................cuuuagauuuuauugcuguagaauu.............................    1    0    S04
...........................................................cuuuagauuuuauugcuguagaauuu............................    1    0    S04
............................................................uuuagauuuuauugcuguagaauA.............................    1    1    S04
............................................................uuuagauuuuauugcuguagaauu.............................    4    0    S04
.............................................................uuagauuuuauugcuguagaauu.............................    1    0    S04
.............................................................uuagauuuuauugcuguagaauuC............................    2    1    S04
.............................................................uuagauuuuauugcuguagaauuG............................    1    1    S04
.............................................................uuagauuuuGuugcuguagaauuu............................    1    1    S04
.............................................................uuagauuuuauugcuguagaauuA............................    1    1    S04
.............................................................uuagauuuuauugcuguagaauuu............................    25   0    S04
..............................................................uagauuuuauugcuguagaauCu............................    1    1    S04
..............................................................Cagauuuuauugcuguagaauuu............................    1    1    S04
..............................................................uagauuuuauugcuguagaauuu............................    11   0    S04
...............................................................agauuuuauugcuguagaauuu............................    1    0    S04
...............................................................agauuuuauugcuguagaauuuua..........................    1    0    S04
................................................................gauuuuauugcuguagaauu.............................    1    0    S04
```

Star **Mature**

```
uaauggcuguaguuuuaaaaauuuuacuguagaaaaaaaucuguaaacuuuugaugugacuuuagauuuuauugcuguagaauuuuauggaaagcacuaaaaaaauugcuuugg
................................................................gauuuuGuugcuguagaauuuua...........................	1	1	S04
.................................................................Uuuuuauugcuguagaauuuuaug.........................	1	1	S04
..................................................................uuuuauugcuguagaauuuuau..........................	1	0	S04
..................................................................uuuuauugcuguagaauuuuaug.........................	3	0	S04
..................................................................uuuuauugcuguagaauuuuaugg........................	3	0	S04
...................................................................uuuauugcuguagaauuuua...........................	1	0	S04
...................................................................uuuauugcuguagaauuuuaugg........................	1	0	S04
...................................................................uuuauugcuguagaauuuuauggG.......................	1	1	S04
.....................................................................uauugcuguagaauuuuauggaa......................	1	0	S04
.....................................................................uauugGuguagaauuuuauggaaa.....................	1	1	S04
......................................................................auugcuguagaauuuuUugg........................	1	1	S04
......................................................................auugcuguagaauuuuauggaaag....................	7	0	S04
.......................................................................uugcuguagaauuuuauggaa......................	1	0	S04
.......................................................................uugcuguagaauuuuGuggaaagc...................	1	1	S04
.......................................................................uugcuguagaauuuuauggaaagc...................	1	0	S04
........................................................................ugcuguagaauuuuaugg........................	1	0	S04
........................................................................ugcuguagaauuuuauggaaagc...................	1	0	S04
..........................................................................cuguagaauuuuauggaaagcacu................	2	0	S04
...........................................................................uguagaauuuuauggaaagcacu................	1	0	S04
```

Provisional ID	:	chrC04_1668371
Score total	:	0
Score for star read(s)	:	3.9
Score for read counts	:	-4.9
Score for mfe	:	1.1
Score for randfold	:	0
Score for cons. seed	:	
Total read count	:	2
Mature read count	:	1
Loop read count	:	0
Star read count	:	1

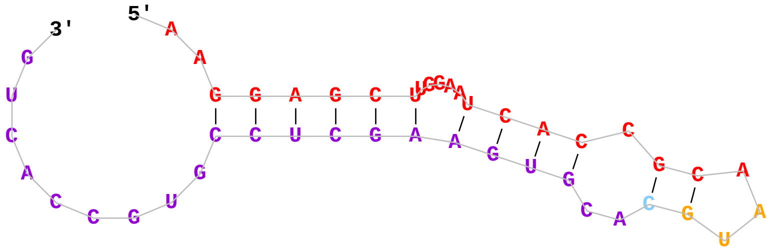

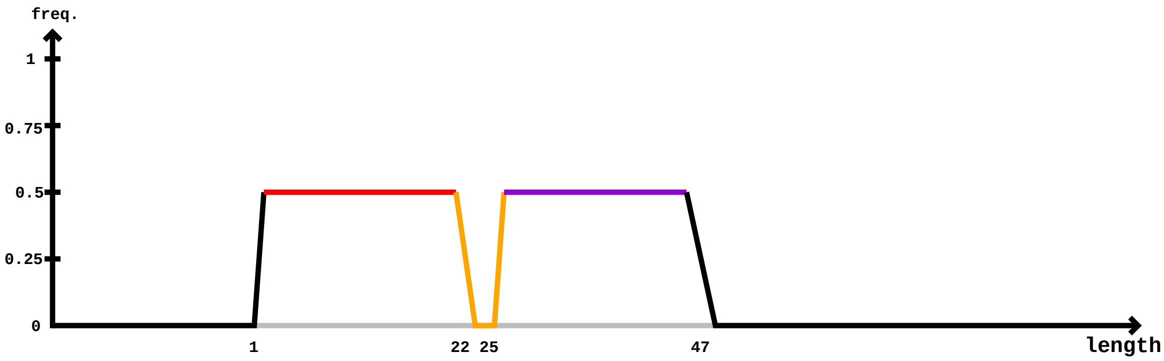

Mature Star

```
5'- aagauguugcucagcgaugcaaggagcuuggaaucaccgcaaugcacgugaagcuccgugccacugguggaaacaagaccaagacaccuggucccggugcucaguccgcuc   -3'    obs
    aagauguugcucagcgaugcaaggagcuuggaaucaccgcaaugcacgugaagcuccgugccacugguggaaacaagaccaagacaccuggucccggugcucaguccgcuc          exp
    ...........((((..(((.((((((.....((((.((...))..)))))))))).)))((((((.(....)..(((((.......))))))))))).......)))).    reads    mm    sample
    ...................aaggagcuuggaaucaccgca......................................................................    1        0     S03

    ..........................................acgugaagcuccgugccacug...............................................    1        0     S04
```

Provisional ID	:	chrC04_1672744
Score total	:	7.7
Score for star read(s)	:	3.9
Score for read counts	:	10.3
Score for mfe	:	-6.4
Score for randfold	:	0
Score for cons. seed	:	
Total read count	:	32
Mature read count	:	23
Loop read count	:	8
Star read count	:	1

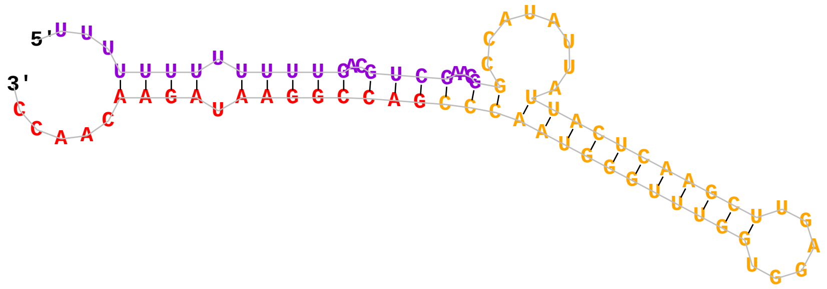

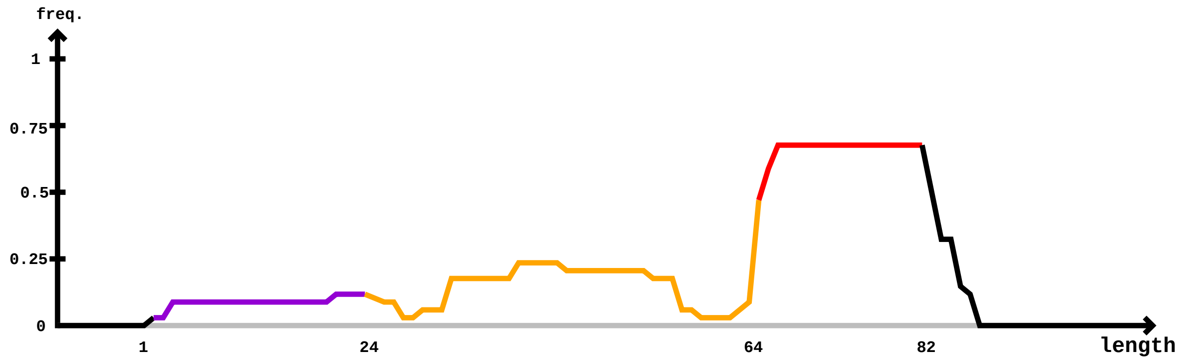

Star **Mature**

```
5'- auccauaguuuuuuuuuuuugacgucgaacggccauauuauuacucaagcuugaggugguuuggguaacccgaccggaauagaacaaccaaugauaaauaacucccuacgaa   -3'    obs
    auccauaguuuuuuuuuuuugacgucgaacggccauauuauuacucaagcuugaggugguuuggguaacccgaccggaauagaacaaccaaugauaaauaacucccuacgaa          exp
    ...(((.(((.((((.(((((..((((...((........((((((((((......))))))))))))))))))))).)))).)))..)))..................... reads    mm    sample
    .....................................................................ccgaccggaauagaacaacA....................... 1    1    S03
    ......................................................................Ngaccggaauagaacaaccaau.................... 1    1    S03
    .......................................................................gaccggaauagaacaacc....................... 1    0    S03
    .......................................................................gaccggaauagaacaacca...................... 1    0    S03
    .......................................................................gaccggaauagaacaaccaau.................... 1    0    S03
    .......................................................................gaccggaauagaacaaccaauga.................. 1    0    S03

    ........uuuuuuuuuuuugacgucgaacg................................................................................. 1    0    S04
    ..........uuuuuuuuuugacgucgaacggcG.............................................................................. 2    1    S04
    ...........................aacggccauUuuauuacucaagcu............................................................. 1    1    S04
    ....................................auuauuaUucaagcuugaggugggu.................................................... 1    1    S04
    .......................................auuacucaagcuugaggugguuug................................................. 1    0    S04
    .......................................auuacucaagcuugagguggAuug................................................. 2    1    S04
    .......................................auuacucaagcuugaggCgguuug................................................. 1    1    S04
    ..............................................aagcuugaggugguuuggg............................................... 1    0    S04
    ..............................................aagcuugagUugguuuggguaacc.......................................... 1    1    S04
    ......................................................................cgaccggaauagaacaaccaau.................... 1    0    S04
    .......................................................................gaccggaauagaacaacc....................... 5    0    S04
    .......................................................................gaccggaauagaacaacca...................... 1    0    S04
    .......................................................................gaccggaauagaacaaccaau.................... 1    0    S04
    .......................................................................gaccggaaCagaacaaccaauga.................. 1    1    S04
    .......................................................................gaccggaauagaacaaccaauga.................. 1    0    S04
    ........................................................................accggaauagaacaacca...................... 3    0    S04
    ........................................................................accggaauagaacaaccaauga.................. 1    0    S04
    .........................................................................ccggaauagaacaaccaau.................... 2    0    S04
    .........................................................................ccggaauagaacaaccaaug................... 1    0    S04
```

Provisional ID	:	chrC04_1697252
Score total	:	277.2
Score for star read(s)	:	3.9
Score for read counts	:	282.1
Score for mfe	:	-8.7
Score for randfold	:	0
Score for cons. seed	:	
Total read count	:	565
Mature read count	:	351
Loop read count	:	0
Star read count	:	214

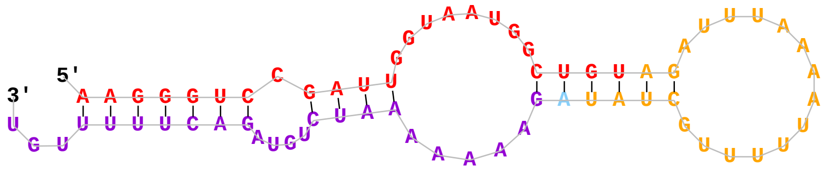

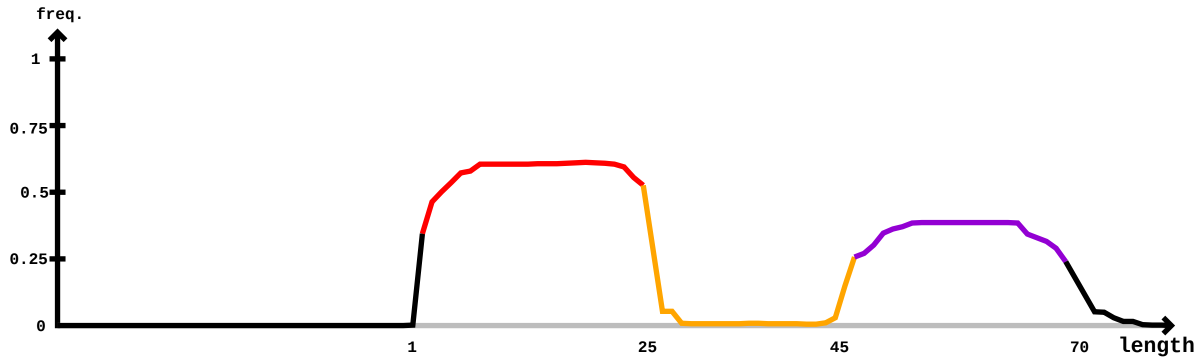

Mature Star

```
5'- uuaauuugaaaaugauaaaaauccaauaacauaaacaaaggguccgauugguaauggcuguagauuuaaaauuuuugcuauagaaaaaaaucuguagacuuuuuguugugacuuu -3'  obs
    uuaauuugaaaaugauaaaaauccaauaacauaaacaaaggguccgauugguaauggcuguagauuuaaaauuuuugcuauagaaaaaaaucuguagacuuuuuguugugacuuu      exp
    .............................(((((.(((((((((.((((........((((((..............)))))).....))))....))))))))))))))).....  reads  mm  sample
    ....................................aaGggguccgauugguaaugg..........................................................  1  1  S03
    .....................................aaggguccgauugguaau............................................................  1  0  S03
    .....................................aaggguccgauugguaaugg..........................................................  1  0  S03
    .....................................aaggguccgauugguaauggA.........................................................  1  1  S03
    .....................................aaggguccgauugguaauggc.........................................................  5  0  S03
    .....................................aaggguccgauugguaauggcC........................................................  1  1  S03
    .....................................aaggguccgauugguaauggcu........................................................  8  0  S03
    .....................................aaggguccgauugguaauggcA........................................................  1  1  S03
    .....................................aaggguccgauuAguaauggcug.......................................................  1  1  S03
    .....................................aaggguccgauugguaauggcug.......................................................  11  0  S03
    .....................................aaggguccgauuggAaauggcugu......................................................  1  1  S03
    .....................................Naggguccgauugguaauggcugu......................................................  1  1  S03
    .....................................aaggguccgauugguaauggcugG......................................................  7  1  S03
    .....................................aaggguccgCuugguaauggcugu......................................................  1  1  S03
    .....................................aaggguccgauugguaauggcugu......................................................  133  0  S03
    .....................................Gaggguccgauugguaauggcugu......................................................  1  1  S03
    .....................................aaggguccgauCgguaauggcugu......................................................  1  1  S03
    .....................................aaggguccgauugguaauggcugA......................................................  7  1  S03
    .....................................aaggguccgauugguaauggcuguU.....................................................  2  1  S03
    .....................................aaggguccgauugguaauggcugua.....................................................  1  0  S03
    ......................................aggguccgauugguaauC...........................................................  1  1  S03
    ......................................aggguccgauugguaauggcu........................................................  7  0  S03
    ......................................aggguccgauugguaauggcA........................................................  1  1  S03
    ......................................aggguccgauugguaauggcug.......................................................  2  0  S03
    ......................................aggguccgauugguaauggcugu......................................................  17  0  S03
    ......................................aggguccgauugguaauggcugG......................................................  3  1  S03
    ......................................aggguccgauuggCaauggcugua.....................................................  1  1  S03
    ......................................aggguccgauugguaGuggcugua.....................................................  1  1  S03
    ......................................aggguccgauugguaauggcuguG.....................................................  2  1  S03
    ......................................aggguccgauugguaauggcugua.....................................................  24  0  S03
    ......................................aggguccgaCugguaauggcugua.....................................................  1  1  S03
    ......................................aggguccgauugguaauggcuguU.....................................................  1  1  S03
    .......................................ggguccgauugguaauggcug.......................................................  1  0  S03
```

Mature Star

uuaauuugaaaaugauaaaauccaauaacauaaacaaaggguccgauugguaauggcuguagauuuaaaauuuuugcuauagaaaaaaaucuguagacuuuuuguugugacuuu			
......................................ggguccgauugguaauggcugu..	9	0	S03
......................................ggguccgGuugguaauggcugua...	1	1	S03
......................................ggguccgauugguaauggcuguU...	2	1	S03
......................................ggguccgauugguaauggcugua...	6	0	S03
......................................ggguccgauugguaauggcuguaga...	1	0	S03
....gguccgauugguaauggcugu..	1	0	S03
....gguccgauugguaauggcugA..	1	1	S03
....gguccgauugguaauggcugua...	3	0	S03
....gguccgauugguaauggcuguagU...	10	1	S03
....gguccgauugguaauggcuguaga...	2	0	S03
....guccgauugguaauggcu..	1	0	S03
....guccgauugguaauggcugu..	4	0	S03
....guccgauugguaauggcugua...	10	0	S03
....guccgauugguaauggcuguaga...	1	0	S03
....guccgauugguaauggcuguagU...	3	1	S03
....guccgauugguaauggcCguagau..	1	1	S03
....uccgauugguaauggcugu..	1	0	S03
....uccgauugguaauggcuguagU...	2	1	S03
....ccgauugguaauggcugu..	2	0	S03
....ccgauugguaauggcugGa...	1	1	S03
....ccgauugguaauggcugua...	2	0	S03
....Ncgauugguaauggcugua...	1	1	S03
....ccgauugguaauggcuguU...	2	1	S03
....ccgauugguaauggcuguagU...	4	1	S03
....ccgauugguaauggcuguaga...	2	0	S03
....gguaauggcuguagGuuuaaaauu..	1	1	S03
....aauggcCguagauuuaaaauuuuu.......................................	1	1	S03
....uggcuguagauuuaaaauuuuugcuU...................................	1	1	S03
....auagaaaaaaaucuguagacu...............	2	0	S03
....auagaaaaaaaucuguagacuuCu............	1	1	S03
....uagaaaaCaaucuguagac................	1	1	S03
....uagaaaaCaaucuguagacu...............	1	1	S03
....uagaaaaCaaucuguagacuu..............	3	1	S03
....uagaaaaCaaucuguagacuuuu............	1	1	S03
....uagaaaaCaaucuguagacuuuuu...........	5	1	S03
....agaaaaCaaucuguagacu...............	11	1	S03
....agaaaaCaaucuguagacuu..............	2	1	S03
....agaaaaaaaucuguagacuuuu............	1	0	S03
....agaaaaCaaucuguagacuuuu............	9	1	S03
....agaaaaaaaucuguagacuuuuu...........	1	0	S03
....agaaaaaaaucuguagacuuuuC...........	1	1	S03
....agaaaaCaaucuguagacuuuuu...........	15	1	S03
....agaaaaCaaucuguagacuuuuug..........	20	1	S03
....agaaaaaaaucuguagacuuuuug..........	3	0	S03
....gaaaaCaaucuguagacu...............	9	1	S03
....gaaaaCaaucuguagacuu..............	1	1	S03
....gaaaaCaaucuguagacuuu.............	4	1	S03
....gaaaaCaaucuguagacuuuu............	1	1	S03
....gaaaaCaaucuguagacuuuuu...........	5	1	S03
....gaaaaCaaucuguagacuuuuug..........	11	1	S03
....gaaaaCaaucuguagacuuuuugu.........	29	1	S03
....gaaaaUaaucuguagacuuuuugu.........	1	1	S03
....Aaaaaaaaucuguagacuuuuugu.........	1	1	S03
....gaaaaaaaucuguagacuuuuugu.........	1	0	S03
....aaaaCaaucuguagacuuu.............	1	1	S03
....aaaaCaaucuguagacuuuu............	2	1	S03
....aaaaCaaucuguagacuuuuu...........	1	1	S03
....aaaaCaaucuguagacuuuuugu.........	1	1	S03
....aaaCaaucuguagacuuu.............	1	1	S03
....aaaCaaucuguagacuuuuugu.........	16	1	S03
....aaaCaaucuguagacuuuuuguug.......	1	1	S03
....aaCaaucuguagacuuuuu...........	1	1	S03
....aaGaaucuguagacuuuuugu.........	1	1	S03
....aaCaaucuguagacuuuuugu.........	15	1	S03
....aaaaaucuguagacuuuuuguugu......	1	0	S03
....aaCaaucuguagacuuuuuguugu......	1	1	S03
....aCaaucuguagacuuuuugu.........	1	1	S03
....aaaaucuguagacuuuuugu.........	1	0	S03
[truncated: 2,076,357 more chars]
